# Supplementary material for: Rpd3/CoRest-mediated activity-dependent transcription regulates the flexibility in memory updating in Drosophila
Source: Nat Commun. 2021 Jan 27;12:628. doi: 10.1038/s41467-021-20898-x (PMC7840730; doi:10.1038/s41467-021-20898-x)
Supplement: Supplementary file 1 — Supplementary information [file 41467_2021_20898_MOESM1_ESM.pdf]

Rpd3/CoRest-mediated activity-dependent transcription regulates the flexibility in memory updating in *Drosophila*

Mai Takakura, Reiko Nakagawa, Takeshi Ota, Yoko Kimura, Man Yung NG, Abdalla G. Alia, Hiroyuki Okuno, Yukinori Hirano

Supplementary Fig. 1 Rpd3 and CoRest-F act as memory suppressors.

Supplementary Fig. 2 Optogenetic activation of MB neurons.

Supplementary Fig. 3 The recovery of the Rpd3/CoRest complex after neural activation.

Supplementary Fig. 4 The control experiments assessing *kay* mRNA expression.

Supplementary Fig. 5 Acetylated lysine residues of CoRest-C.

Supplementary Fig. 6 Acetylation of CoRest-F after neural activation, and CBP-dependent complex change in Rpd3/CoRest.

Supplementary Fig. 7 The biological process-associated GO terms

Supplementary Fig. 8 Nuclear RNA-seq analysis from the MB nuclei.

Supplementary Fig. 9 Expression of the target genes of CoRest-C, Rpd3, and CBP.

Supplementary Fig. 10 K318 of CoRest-C is important for activity-dependent transcription.

Supplementary Fig. 11 CoRest-C is not involved in memory which does not require gene expression.

Supplementary Fig. 12 CoRest-F/Rpd3 is required to limit behavioral flexibility.

Supplementary Fig. 13 Schematic diagram of MBsw2.

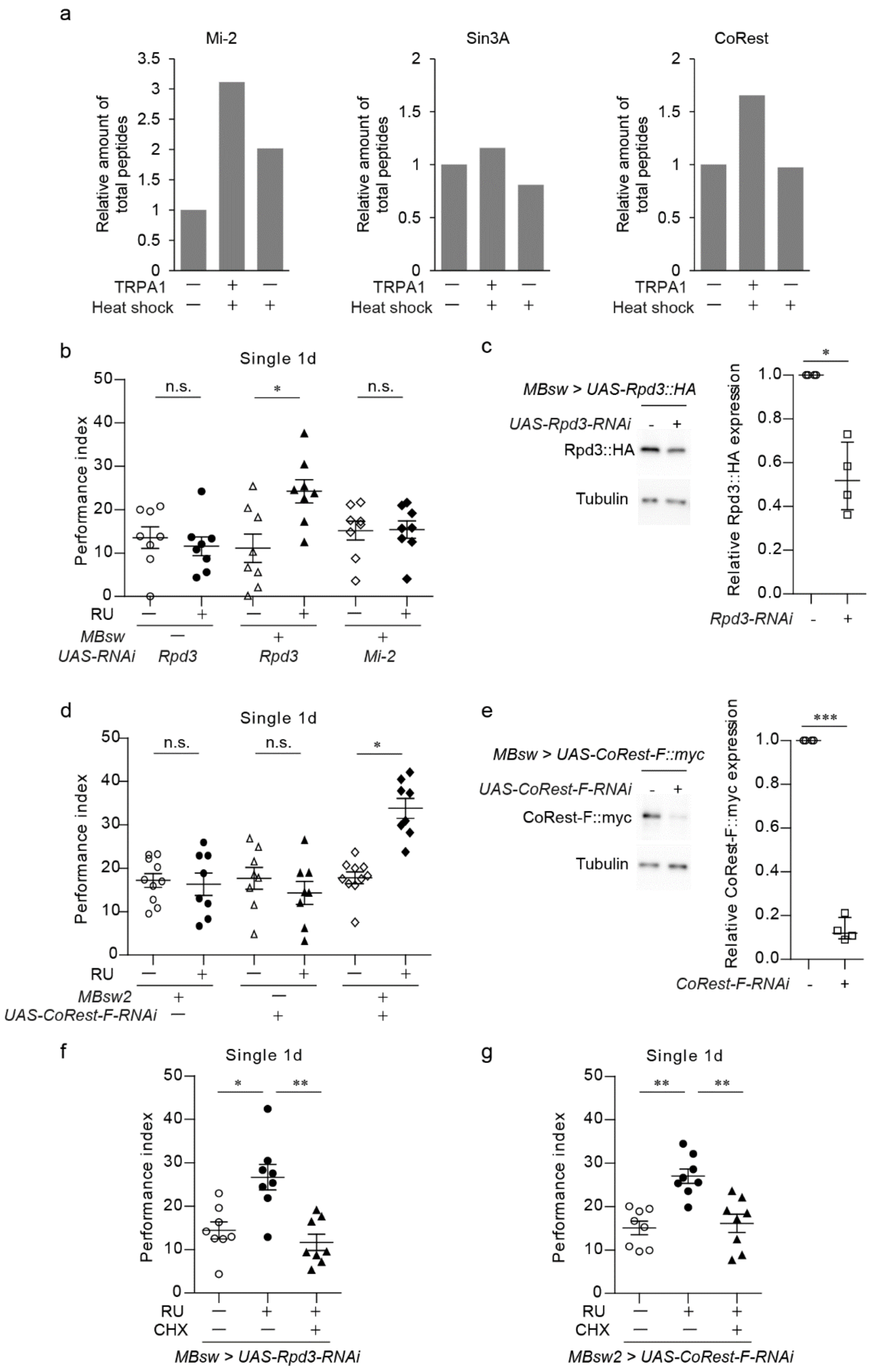

a
Mi-2
Sin3A
CoRest
Relative amount of total peptides
TRPA1
Heat shock
b
Single 1d
Performance index
n.s.
RU
MBsw
UAS-RNAi
Rpd3
Mi-2
c
MBsw > UAS-Rpd3::HA
UAS-Rpd3-RNAi
Rpd3::HA
Tubulin
Relative Rpd3::HA expression
Rpd3-RNAi
d
MBsw2
UAS-CoRest-F-RNAi
e
MBsw > UAS-CoRest-F::myc
CoRest-F::myc
Relative CoRest-F::myc expression
CoRest-F-RNAi
f
CHX
MBsw > UAS-Rpd3-RNAi
g
MBsw2 > UAS-CoRest-F-RNAi

Supplementary Fig. 1 Rpd3 and CoRest-F act as memory suppressors.

a, The relative amounts of the proteins in the Rpd3-immunocomplex (see more details in Supplementary Table 1) after thermogenetic activation. Rpd3::FLAG-HA was expressed by MBsw, together with dTRPA1. The flies were heat-shocked at 35℃ for 1 hour or rested at 25℃, and the heads were used for tandem-tag affinity purification. The amount of each peptide of Mi-2, Sin3A, and CoRest from the Rpd3-immunocomplex, which was determined through the mass spectrometry, was summed and normalized to that of Rpd3.

b,d, Knockdown of *Rpd3* or *CoRest-F,* but not *Mi-2*, enhanced 1-day memory formation. The flies with indicated transgenes were fed RU, and 1-day memory after a single training was assessed. Kruskal-Wallis tests (**b,** $P = 0.0263$; $n = 8$. **d,** $P = 0.0012$; $n = 10, 8, 8, 8, 10, 8$.) followed by Dunn's multiple comparison test (two-sided) were performed.

c,e, RNAi targeting *Rpd3 and CoRest-F* reduced expression of the ectopically induced tagged proteins. The flies carrying the indicated transgenes were fed RU for 3 days, and the head extracts were analyzed by a western blot analysis with anti-HA or anti-myc, and anti-tubulin antibodies. The amount of tubulin was used for normalization. **c,** Two-sided Mann-Whitney U-test, $P = 0.0100$; $n = 4$. **e,** Two-sided Mann-Whitney U-test, $P < 0.0001$; $n = 4$.

f,g, Cycloheximide (CHX) - feeding impaired memory enhancement by knockdown of *Rpd3* or *CoRest-F*. The flies with indicated transgenes were fed RU for 2 days, followed by CHX-feeding for 1 day, then 1-day memory after a single training was assessed. Kruskal-Wallis tests (**f,** $P = 0.0026$; $n = 8$. **g,** $P = 0.0011$; $n = 8$) followed by Dunn's multiple comparison test (two-sided) were performed.

Data are represented as mean ± s.e.m. n.s., not significant $P > 0.05$; *, $P < 0.05$; **, $P <$

0.01; ***, $P < 0.001$, which is based on experimental replicates using different pooled samples, and the number is indicated as n above. Source data are provided as a Source Data file.

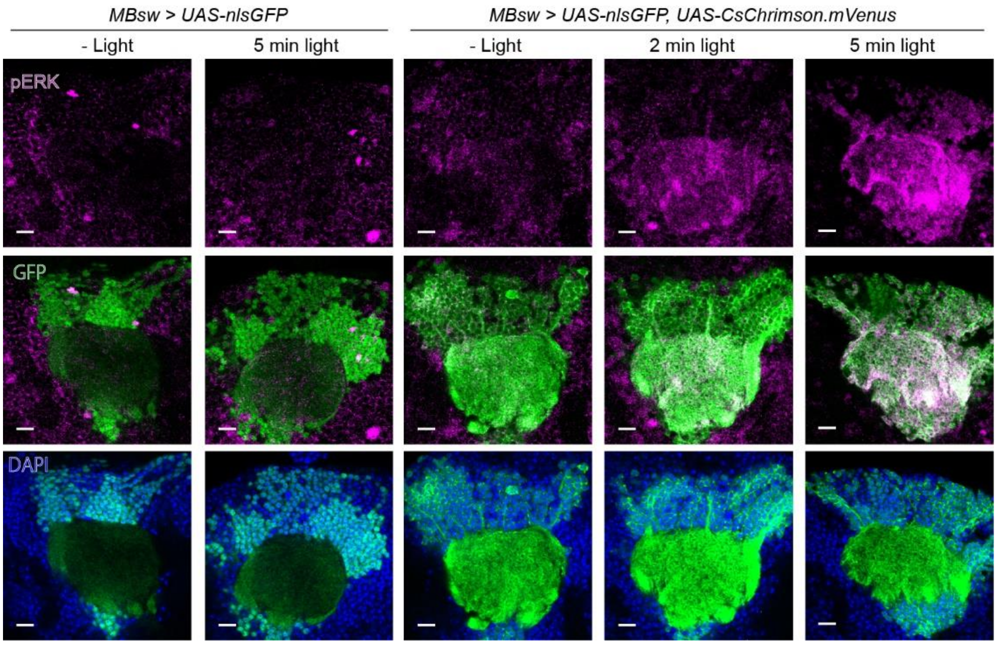

Supplementary Fig. 2 Optogenetic activation of MB neurons. CsChrimson and GFP fused to a nuclear localization signal (nlsGFP) were induced in MB neurons using MBsw. The brains were immunostained with anti-GFP (green) and anti-pERK (magenta) antibodies, and DAPI (blue). The images are representative of experimental replicates (n = 3, 4, 4, 4, 4). Scale bar: 10 μm.

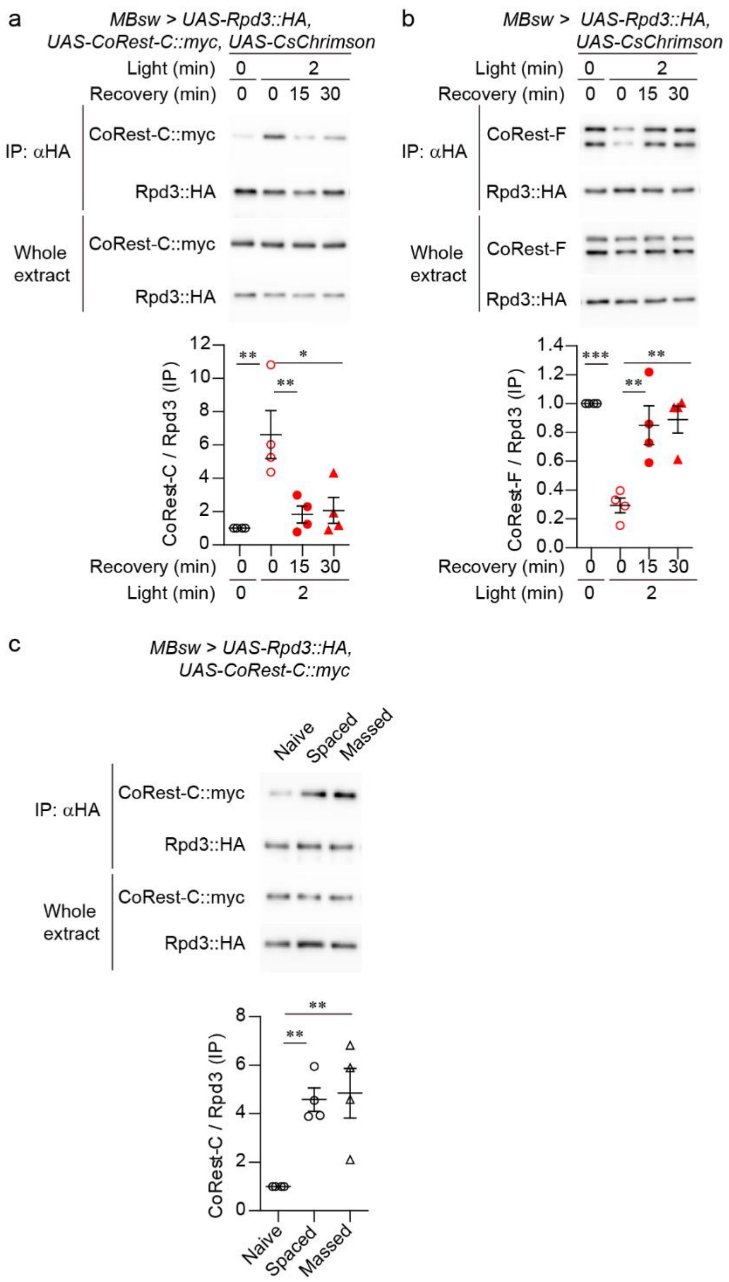

Supplementary Fig. 3 The recovery of the Rpd3/CoRest complex after neural activation.

a,b, The compositional change of the Rpd3/CoRest complex was recovered within 15 min. The flies with the indicated transgenes were fed RU, and illuminated with red light, and recovered for the indicated periods of time.

The head extracts from 100 flies were immunoprecipitated with the anti-HA antibody.

CoRest-F was detected with the anti-CoRest antibody (**b**), and other proteins were detected with the antibodies specific to the indicated epitope tags in a western blot analysis.

c, The compositional change of the Rpd3/CoRest complex was induced by olfactory aversive training. The flies with the indicated transgenes were fed RU, subjected to spaced or massed training, and collected immediately after the trainings. Rpd3 proteins were collected from 1,000 heads via immunoprecipitation with the anti-HA antibody, and analyzed as in (**a**).

One-way ANOVA (**a,** P = 0.0024; n = 4. **b,** P = 0.0004; n = 4. **c,** P = 0.004; n = 4.) followed by Tukey's multiple comparisons (two-sided) were performed.

Data are represented as mean ± s.e.m. n.s., not significant $P > 0.05$; *, $P < 0.05$; **, $P < 0.01$; ***, $P < 0.001$, which is based on experimental replicates using different pooled samples, and the number is indicated as n above. Source data are provided as a Source Data file.

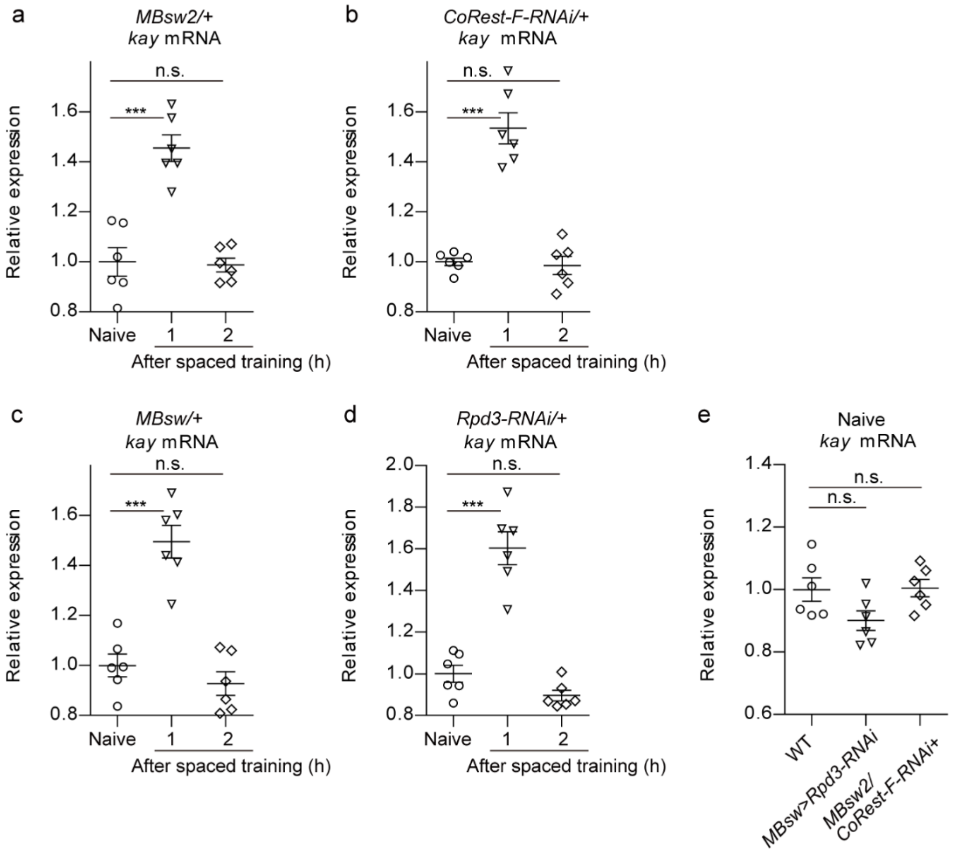

Supplementary Fig. 4 The control experiments assessing *kay* mRNA expression.

a-d, The flies carrying the indicated transgenes were fed RU, and subjected to spaced training. The head RNAs were collected at the indicated time after spaced training, and analyzed via RT-qPCR. A one-way ANOVA (P < 0.0001; n = 6 for all) followed by Tukey's multiple comparisons (two-sided) was performed.

e, Knockdown of *Rpd3* or *CoRest-F* did not affect basal expression of *kay* mRNA. The flies carrying the indicated transgenes were fed RU. The head RNAs were analyzed via RT-qPCR. A one-way ANOVA (P = 0.0658; n = 6) followed by Tukey's multiple comparisons (two-sided) was performed.

Data are represented as mean ± s.e.m. n.s., not significant P > 0.05; *, P < 0.05; **, P < 0.01; ***, P < 0.001, which is based on experimental replicates using different pooled samples, and the number is indicated as n above. Source data are provided as a Source

Data file.

a, K36

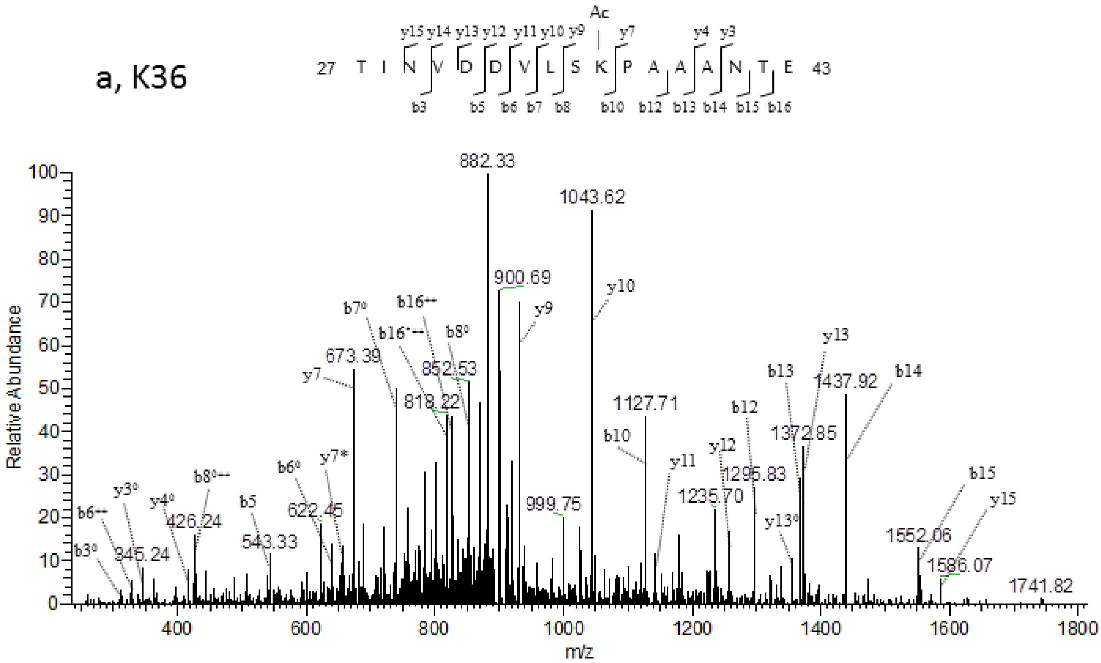

Experimental m/z value : 900.455040,2+
Experimental molecular mass : 1798.8955
Calculated Monoisotopic mass : 1798.8949
Difference (error) between the experimental and calculated masses : 0.38 ppm
Ions Score: 58
Expect: 2.6e-005

#	b	b^{++}	b*	b^{*++}	b^{0}	b^{0++}	Seq.	y	y^{++}	y*	y^{*++}	y^{0}	y^{0++}	#
1	102.05	51.53			84.04	42.53	T							17
2	215.14	108.07			197.13	99.07	I	1698.9	849.93	1681.8	841.42	1680.8	840.93	16
3	*329.18*	165.09	312.16	156.58	311.17	156.09	N	*1585.8*	793.39	1568.7	784.88	1567.8	784.38	15
4	428.25	214.63	411.22	206.12	410.24	205.62	V	*1471.7*	736.37	1454.7	727.85	1453.7	727.36	14
5	*543.28*	272.14	526.25	263.63	525.27	263.14	D	*1372.7*	686.83	1355.6	678.32	1354.7	677.83	13
6	658.3	329.66	641.28	321.14	640.29	320.65	D	*1257.6*	629.32	1240.6	620.81	1239.6	620.31	12
7	757.37	379.19	740.35	370.68	739.36	370.18	V	*1142.6*	571.81	1125.6	563.29	1124.6	562.8	11
8	870.46	435.73	853.43	427.22	852.45	426.73	L	*1043.5*	522.27	1026.5	513.76	1025.5	513.27	10
9	957.49	479.25	940.46	470.73	939.48	470.24	S	*930.45*	465.73	913.43	457.22	912.44	456.72	9
10	*1127.6*	564.3	1110.6	555.79	1109.6	555.3	K	843.42	422.21	826.39	413.7	825.41	413.21	8
11	1224.7	612.83	1207.6	604.31	1206.6	603.82	P	*673.32*	337.16	656.29	328.65	655.3	328.16	7
12	*1295.7*	648.35	1278.7	639.83	1277.7	639.34	A	576.26	288.63	559.24	280.12	558.25	279.63	6
13	*1366.7*	683.86	1349.7	675.35	1348.7	674.86	A	505.23	253.12	488.2	244.6	487.21	244.11	5
14	*1437.8*	719.38	1420.7	710.87	1419.8	710.38	A	434.19	217.6	417.16	209.08	416.18	208.59	4
15	*1551.8*	776.4	1534.8	767.89	1533.8	767.4	N	363.15	182.08	346.12	173.57	345.14	173.07	3
16	1652.9	826.93	1635.8	818.41	1634.8	817.92	T	249.11	125.06			231.1	116.05	2
17							E	148.06	74.53			130.05	65.53	1

b, K303

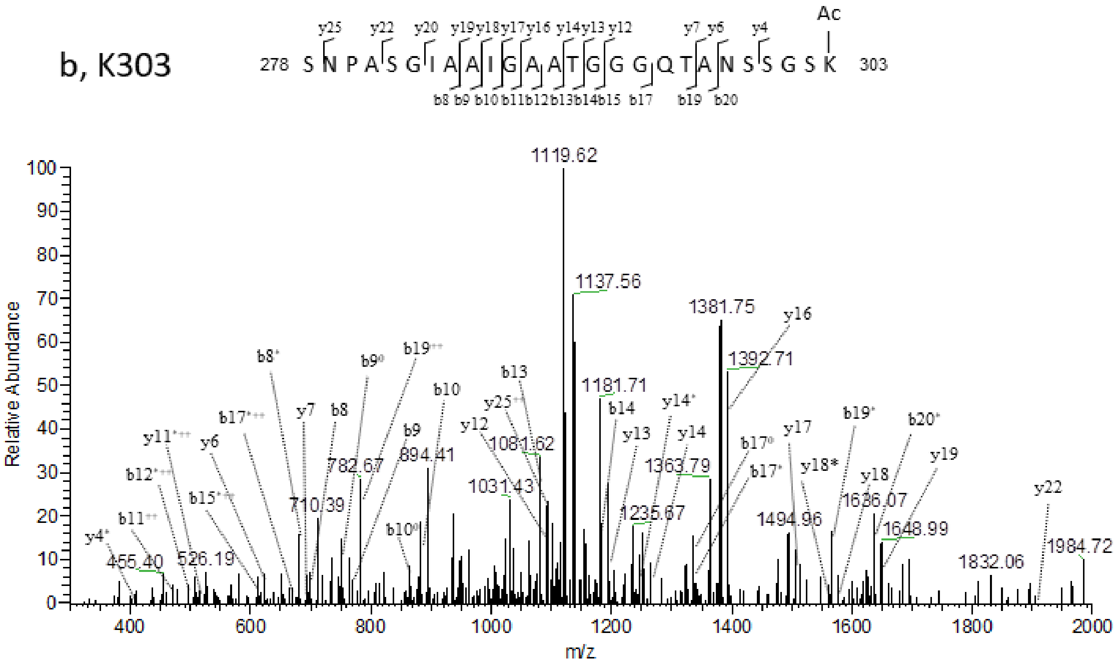

Experimental m/z value : 804.435020,2+
Experimental molecular mass : 1606.8555
Calculated Monoisotopic mass : 1606.8566
Difference (error) between the experimental and calculated masses : -0.70 ppm
Ions Score: 13
Expect: 0.37

#	b	b^{++}	b*	b*$^{++}$	b^{0}	b^{0++}	Seq.	y	y^{++}	y*	y*$^{++}$	y^{0}	y^{0++}	#
1	88.04	44.52			70.03	35.52	S							26
2	202.08	101.54	185.06	93.03	184.07	92.54	N	2187.1	1094	2170	1085.5	2169.1	1085	25
3	299.13	150.07	282.11	141.56	281.12	141.07	P	2073	1037	2056	1028.5	2055	1028	24
4	370.17	185.59	353.15	177.08	352.16	176.58	A	1976	988.49	1958.9	979.97	1958	979.48	23
5	457.2	229.11	440.18	220.59	439.19	220.1	S	1904.9	952.97	1887.9	944.46	1886.9	943.96	22
6	514.23	257.62	497.2	249.1	496.22	248.61	G	1817.9	909.45	1800.9	900.94	1799.9	900.45	21
7	627.31	314.16	610.28	305.65	609.3	305.15	I	1760.9	880.94	1743.9	872.43	1742.9	871.94	20
8	698.35	349.68	681.32	341.16	680.34	340.67	A	1647.8	824.4	1630.8	815.89	1629.8	815.39	19
9	769.38	385.2	752.36	376.68	751.37	376.19	A	1576.8	788.88	1559.7	780.37	1558.8	779.88	18
10	882.47	441.74	865.44	433.22	864.46	432.73	I	1505.7	753.36	1488.7	744.85	1487.7	744.36	17
11	939.49	470.25	922.46	461.74	921.48	461.24	G	1392.6	696.82	1375.6	688.31	1374.6	687.82	16
12	1010.5	505.77	993.5	497.25	992.52	496.76	A	1335.6	668.31	1318.6	659.8	1317.6	659.31	15
13	1081.6	541.29	1064.5	532.77	1063.6	532.28	A	1264.6	632.79	1247.6	624.28	1246.6	623.79	14
14	1182.6	591.81	1165.6	583.3	1164.6	582.8	T	1193.5	597.27	1176.5	588.76	1175.5	588.27	13
15	1239.6	620.32	1222.6	611.81	1221.6	611.31	G	1092.5	546.75	1075.5	538.24	1074.5	537.74	12
16	1296.7	648.83	1279.6	640.32	1278.6	639.83	G	1035.5	518.24	1018.4	509.73	1017.5	509.23	11
17	1353.7	677.34	1336.7	668.83	1335.7	668.34	G	978.45	489.73	961.42	481.21	960.44	480.72	10
18	1481.7	741.37	1464.7	732.86	1463.7	732.37	Q	921.43	461.22	904.4	452.7	903.42	452.21	9
19	1582.8	791.89	1565.8	783.38	1564.8	782.89	T	793.37	397.19	776.34	388.67	775.36	388.18	8
20	1653.8	827.41	1636.8	818.9	1635.8	818.41	A	692.32	346.66	675.29	338.15	674.31	337.66	7
21	1767.9	884.43	1750.8	875.92	1749.9	875.43	N	621.28	311.15	604.26	302.63	603.27	302.14	6
22	1854.9	927.95	1837.9	919.44	1836.9	918.95	S	507.24	254.12	490.21	245.61	489.23	245.12	5
23	1941.9	971.47	1924.9	962.95	1923.9	962.46	S	420.21	210.61	403.18	202.09	402.2	201.6	4
24	1999	999.98	1981.9	991.46	1980.9	990.97	G	333.18	167.09	316.15	158.58	315.17	158.09	3
25	2086	1043.5	2069	1035	2068	1034.5	S	276.16	138.58	259.13	130.07	258.14	129.58	2
26							K	189.12	95.07	172.1	86.55			1

c, K318

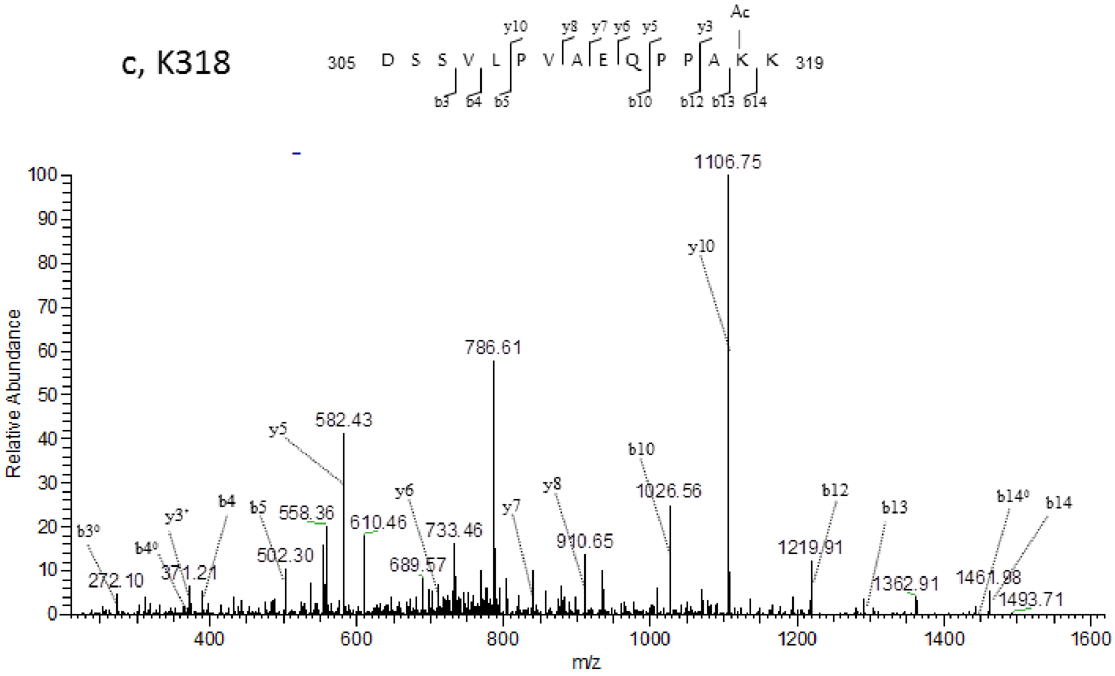

Experimental m/z value : 804.435020,2+
Experimental molecular mass : 1606.8555
Calculated Monoisotopic mass : 1606.8566
Difference (error) between the experimental and calculated masses : -0.70 ppm
Ions Score: 40
Expect: 0.0026

#	b	b^{++}	b*	b^{*++}	b^{o}	b^{o++}	Seq.	y	y^{++}	y*	y^{*++}	y^{o}	y^{o++}	#
1	116.03	58.52			98.02	49.52	D							15
2	203.07	102.04			185.06	93.03	S	1492.8	746.92	1475.8	738.41	1474.8	737.92	14
3	290.1	145.55			272.09	136.55	S	1405.8	703.41	1388.8	694.89	1387.8	694.4	13
4	***389.17***	195.09			371.16	186.08	V	1318.8	659.89	1301.8	651.38	1300.8	650.88	12
5	***502.25***	251.63			484.24	242.62	L	***1219.7***	610.36	1202.7	601.84	1201.7	601.35	11
6	599.3	300.16			581.29	291.15	P	***1106.6***	553.81	1089.6	545.3	1088.6	544.81	10
7	698.37	349.69			680.36	340.68	V	1009.6	505.29	992.54	496.77	991.56	496.28	9
8	769.41	385.21			751.4	376.2	A	***910.5***	455.75	893.47	447.24	892.49	446.75	8
9	898.45	449.73			880.44	440.72	E	***839.46***	420.23	822.44	411.72	821.45	411.23	7
10	***1026.5***	513.76	1009.5	505.25	1008.5	504.75	Q	***710.42***	355.71	693.39	347.2			6
11	1123.6	562.29	1106.5	553.77	1105.6	553.28	P	***582.36***	291.68	565.33	283.17			5
12	***1220.6***	610.81	1203.6	602.3	1202.6	601.81	P	485.31	243.16	468.28	234.64			4
13	***1291.7***	646.33	1274.6	637.82	1273.6	637.32	A	388.26	194.63	371.23	186.12			3
14	***1461.8***	731.38	1444.7	722.87	1443.8	722.38	K	317.22	159.11	300.19	150.6			2
15							K	147.11	74.06	130.09	65.55			1

Supplementary Fig. 5 Acetylated lysine residues of CoRest-C.

a, b, c, Bold italic red font represents the series contributing to the score. Bold red

indicates that the number of matches in the ion series is greater than that which would be expected by chance, indicating that the ion series is present. Non-bold red indicates that the number of matches in the ion series is no greater than that which would be expected by chance, such that the matches themselves may be by chance. *, -NH3 if fragment includes RKNQ; 0, -H2O if fragment includes STED (mass spectrum in each upper panel).

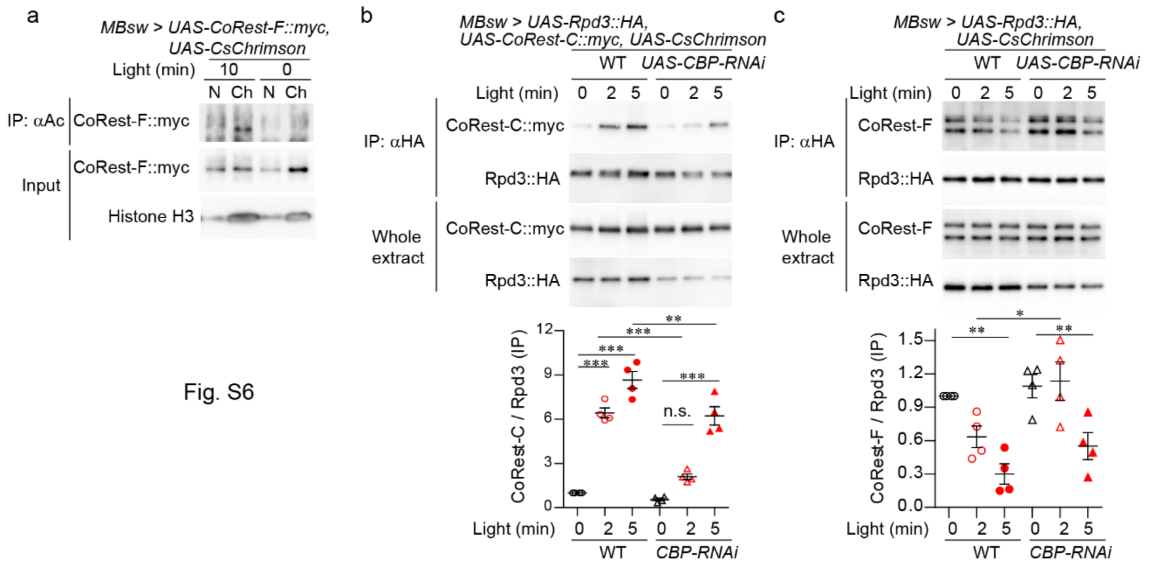

Supplementary Fig. 6 Acetylation of CoRest-F after neural activation, and CBP-dependent complex change in Rpd3/CoRest.

a, CoRest-F was acetylated after optogenetic activation. The flies carrying the indicated transgenes were fed RU, and illuminated with red light. Nuclear extracts prepared from the heads were separated into the nuclear proteins (N) and the chromatin-associating proteins (Ch) by centrifugation in the presence of Magnesium (Mg2+). Each fraction was immunoprecipitated with the anti-Acetyl lysine antibody (αAc). The antibodies for the indicated epitope tags or for histone H3 were used in a western blot analysis. The images are representative of three experimental replicates.

b, c, Knockdown of *CBP* attenuated CoRest-C-binding to (**b**) and CoRest-F dissociation from Rpd3 (**c**) after optogenetic activation. The flies with the indicated transgenes were fed RU, and illuminated with red light, and the head extracts were immunoprecipitated with the anti-HA antibody. CoRest-F was detected with the anti-CoRest antibody, and other proteins were detected with the antibodies specific to the indicated epitope tags in a western blot analysis. One-way ANOVA (**b,** $P < 0.0001$; $n = 4$. **c,** $P = 0.0002$; $n = 4$.) followed by Tukey's multiple comparisons (two-sided) were performed.

Data are represented as a mean ± s.e.m. n.s., not significant $P > 0.05$; **, $P < 0.01$; *** $P < 0.001$, which is based on experimental replicates using different pooled samples, and the number is indicated as n above.Source data are provided as a Source Data file.

a

b

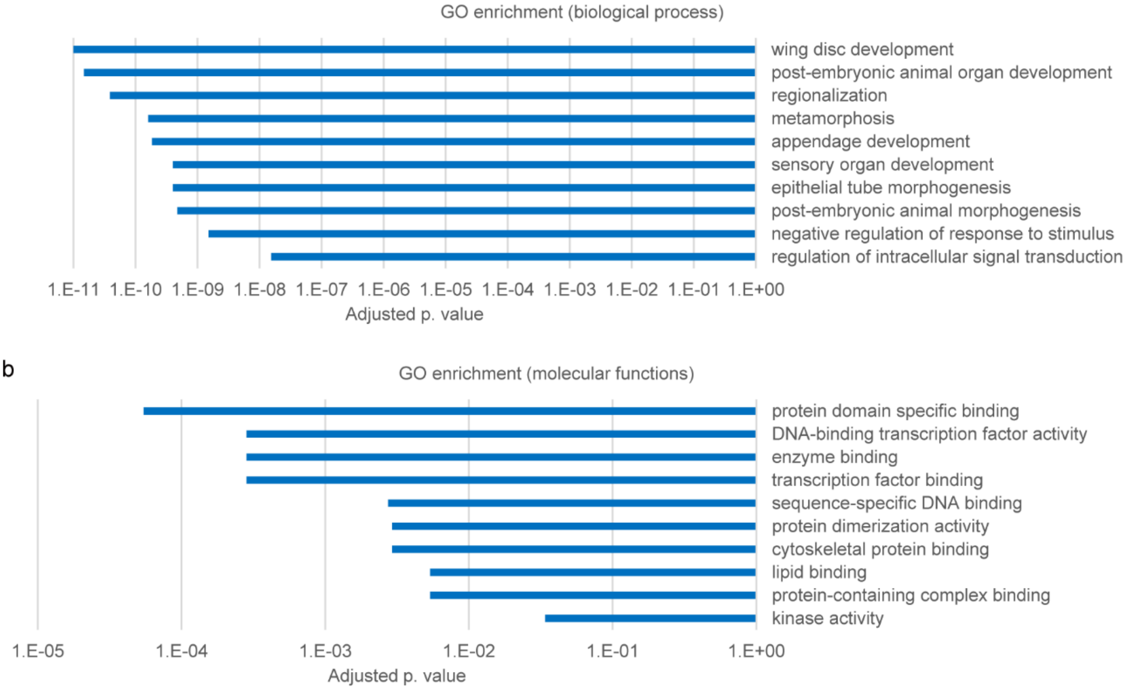

Supplementary Fig. 7 The biological process-associated GO terms (**a**) and the molecular function-associated GO terms (**b**) significantly enriched in the 704 genes bound by CoRest-C, CBP and Rpd3.

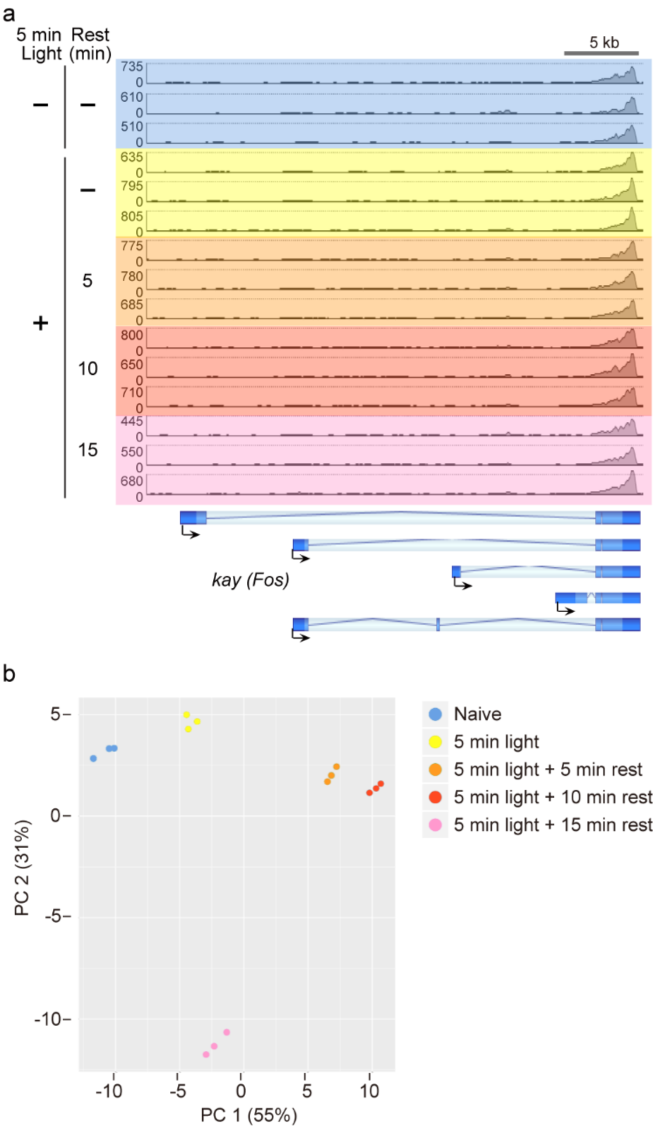

Supplementary Fig. 8 Nuclear RNA-seq analysis from the MB nuclei.

a, RNA-seq signals at the region near *kay*. The flies expressing FLAG-KASH and CsChrimson by MBpLexA were illuminated with red light, followed by the rest for the indicated period of time. MB nuclei prepared as in Fig. 6 were subjected to RNA-seq analysis (n=3). The y axes show the number of mapped reads, and the upper limits were adjusted to the number of total reads in each sample. The statistical analysis via DESeq2 indicated that *kay* mRNA expression is significantly increased after 10 min of rest following optogenetic activation (q value = 0.000098, log2 = 0.57).

b, Principal component analysis (PCA) of mRNA expression data from the nuclear RNA-seq analysis.

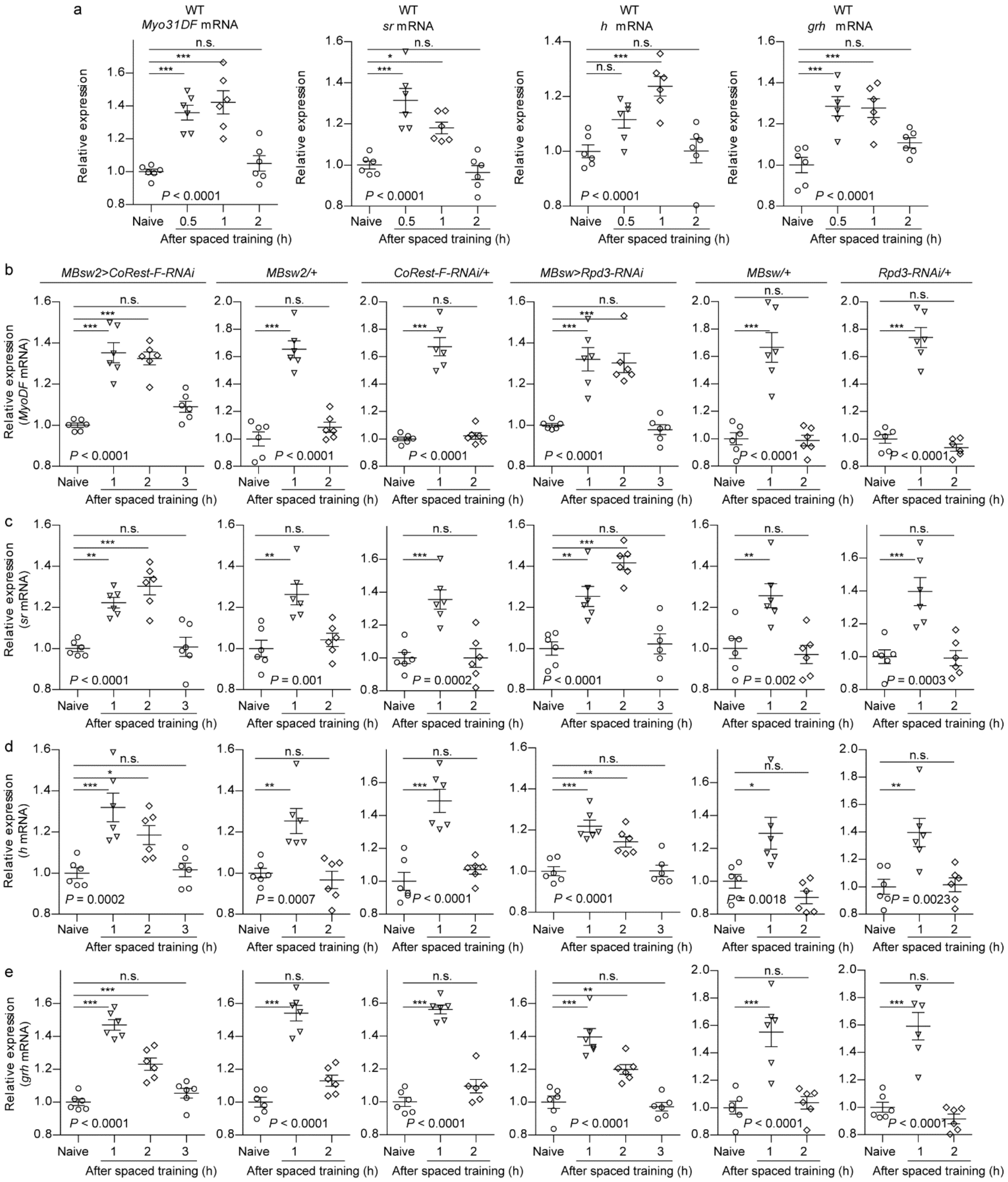

Supplementary Fig. 9 Expression of the target genes of CoRest-C, Rpd3, and CBP.

a, Expression of the indicated genes in WT flies after spaced training.

b-e, Expression of *Myo31DF* (**b**), *sr* (**c**), *h* (**d**), and *grh* (**e**) was increased up to 2 hours after spaced training by knockdown of *CoRest-F* or *Rpd3*.

The flies carrying the indicated transgenes were fed RU, and subjected to spaced training. The head RNAs were collected at the indicated time after spaced training, and analyzed via RT-qPCR. One-way ANOVA (P values are shown in each panel; n = 6 for all) followed by Tukey's multiple comparisons (two-sided) were performed.

Data are represented as mean ± s.e.m. n.s., not significant $P > 0.05$; *, $P < 0.05$; **, $P < 0.01$; ***, $P < 0.001$, which is based on experimental replicates using different pooled samples, and the number is indicated as n above. Source data are provided as a Source Data file.

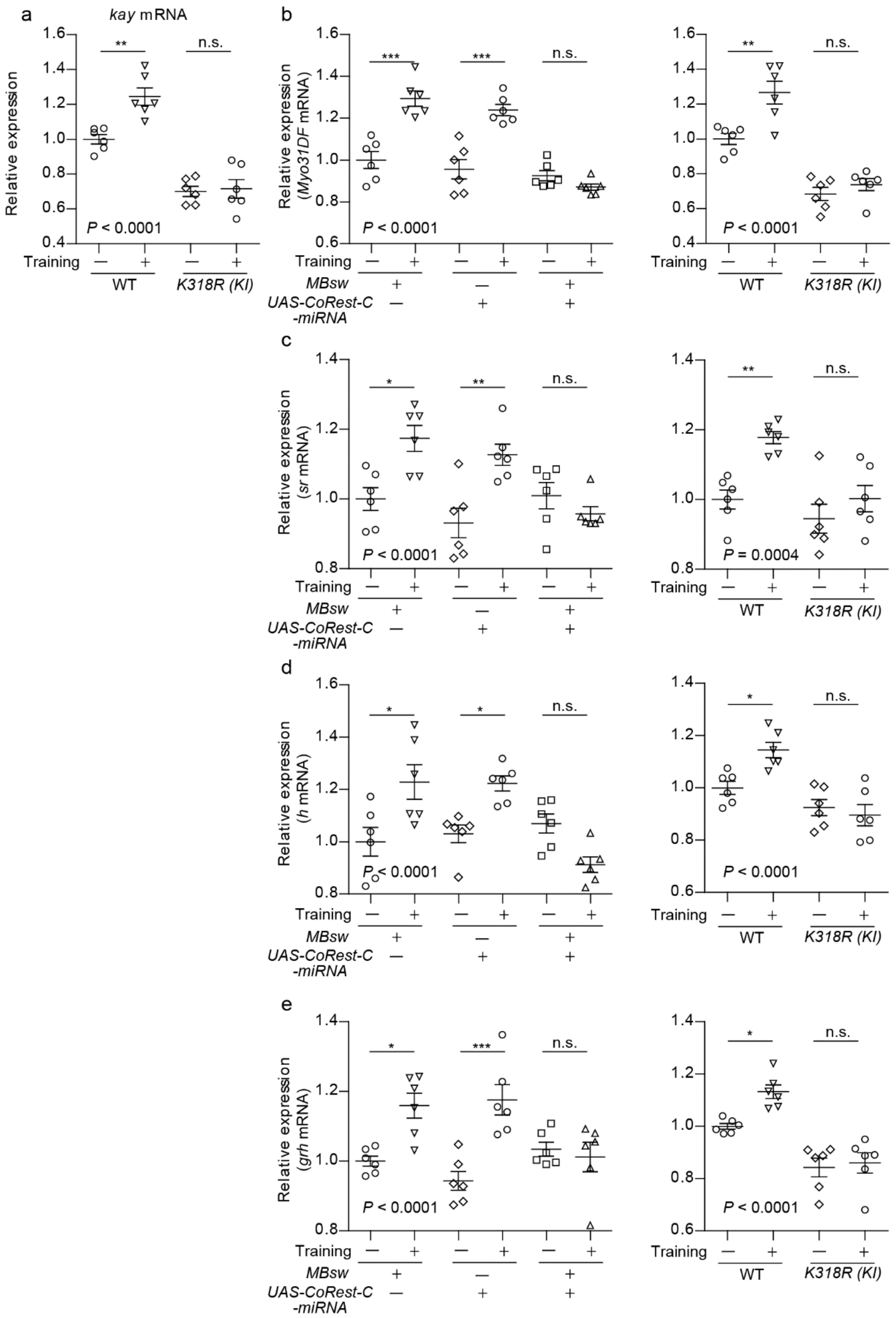

Supplementary Fig. 10 K318 of CoRest-C is important for activity-dependent transcription.

a, The *K318R* mutation in *CoRest*-C impaired the expression of *kay* mRNA after spaced training. *K318R (KI), CoRest-C-K318R* knock-in mutant.

b-e, Activity-dependent transcription requires CoRest-C and its K318. The flies carrying the indicated transgenes were fed normal food (right panels) or food containing RU (left panels), and subjected to spaced training. RNA extracted from the fly heads at 1-hour after spaced training was analyzed via RT-qPCR. One-way ANOVA (*P* values are shown in each panel. *n* = 6 for all) followed by Tukey's multiple comparisons (two-sided) were performed.

Data are represented as mean ± s.e.m. n.s., not significant $P > 0.05$; *, $P < 0.05$; **, $P < 0.01$; *** $P < 0.001$, which is based on experimental replicates using different pooled samples, and the number is indicated as n above. Source data are provided as a Source Data file.

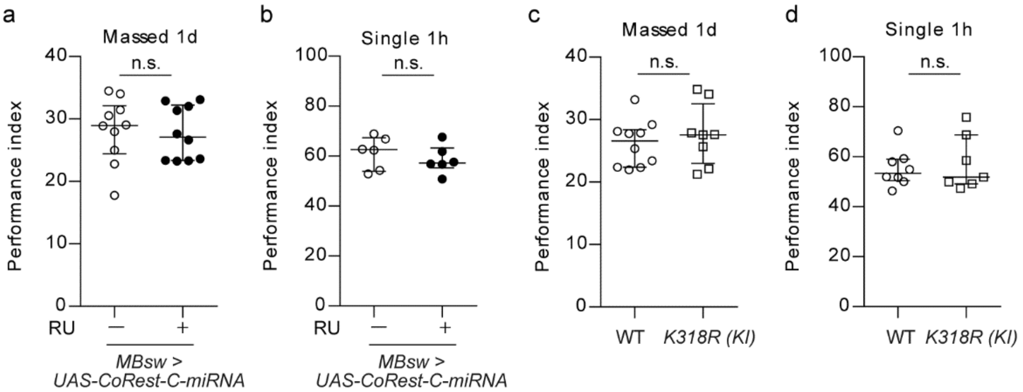

Supplementary Fig. 11 CoRest-C is not involved in memory which does not require gene expression.

The knockdown of *CoRest-C* or the knock-in of the *K318R* mutation did not affect 1-day memory after mass training or 1-hour memory after a single training. The flies carrying the indicated transgenes were fed RU (**a, b**), or normal food (**c, d**), and subjected to mass

training (**a, c**) or a single training (**b, d**). **a,** Two-sided Mann-Whitney U-test, $P = 0.7394$; $n = 10$. **b,** Two-sided Mann-Whitney U-test, $P = 0.6991$; $n = 6$. **c,** Two-sided Mann-Whitney U-test, $P = 0.8968$; $n = 10, 8$. **d,** Two-sided Mann-Whitney U-test, $P = 0.9551$; $n = 6$. *K318R (KI), CoRest-C-K318R* knock-in mutant.

Data are represented as mean ± s.e.m. n.s., not significant $P > 0.05$, which is based on experimental replicates using different pooled samples, and the number is indicated as n above. Source data are provided as a Source Data file.

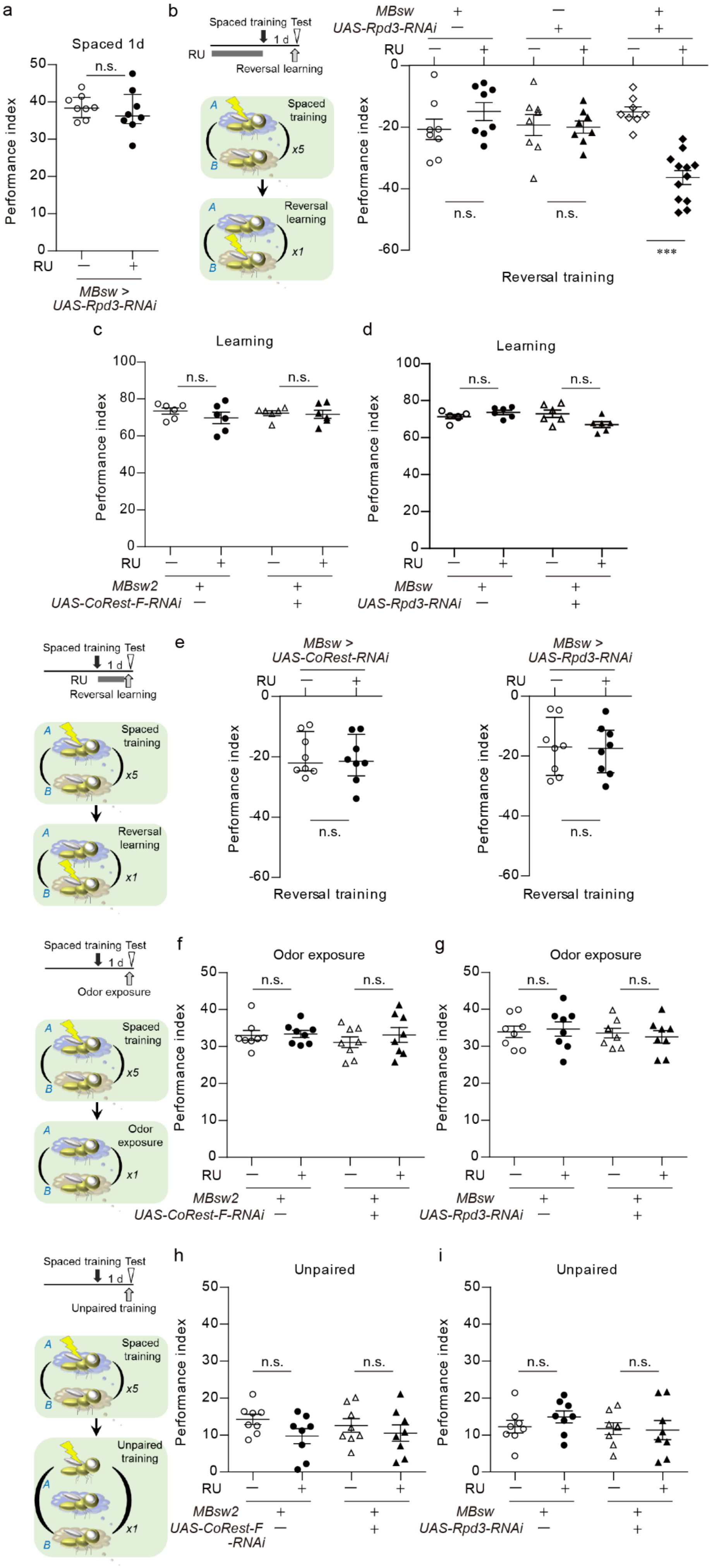
a
Spaced 1d
n.s.
Performance index
RU
MBsw > UAS-Rpd3-RNAi
b
Spaced training Test
1 d
RU
Reversal learning
Spaced training
x5
Reversal learning
x1
MBsw
UAS-Rpd3-RNAi
n.s.
***
Reversal training
c
Learning
MBsw2
UAS-CoRest-F-RNAi
d
Learning
MBsw
UAS-Rpd3-RNAi
e
MBsw > UAS-CoRest-RNAi
MBsw > UAS-Rpd3-RNAi
Reversal training
f
Odor exposure
g
Odor exposure
h
Unpaired
Unpaired training
i
Unpaired
UAS-CoRest-F -RNAi

Supplementary Fig. 12 CoRest-F/Rpd3 is required to limit behavioral flexibility.

a, Knockdown of *Rpd3* did not affect LTM formation. The flies with indicated transgenes were fed RU, and 1-day memory after spaced training was examined. Two-sided Mann-Whitney U-test, P = 0.5737; n = 10.

b, The flexibility in memory updating was increased by *Rpd3* knockdown. The flies with indicated transgenes were fed RU, and subjected to spaced training. On the following day, the flies were subjected to reversal learning, in which the association of the odors and shock was alternated, and memory was measured immediately. A Kruskal-Wallis test (P = 0.0001; n = 12) followed by Dunn's multiple comparison test (two-sided) was performed.

c, d, Learning was not affected by the knockdown of *CoRest-F* or *Rpd3*. The flies with indicated transgenes were fed RU, and subjected to a single training. Memory was measured immediately. Kruskal-Wallis tests (**c,** P = 0.7900; n = 6. **d,** P = 0.0586; n = 6) followed by Dunn's multiple comparison test (two-sided) were performed.

e, Acute knockdown of *CoRest-F* or *Rpd3* did not affect reversal learning. The flies with indicated transgenes were fed normal food, spaced-trained, and fed RU. On the following day, the flies were subjected to reversal learning. Memory was measured immediately. A Kruskal-Wallis test (P = 0.7984; n = 8) followed by Dunn's multiple comparison test (two-sided) was performed.

f,g, *CoRest-F* or *Rpd3* knockdown did not affect memory after exposure to odors. The flies with indicated transgenes were fed RU, and subjected to spaced training. On the following day, the conditioned and unconditioned odors were delivered to the flies, and memory was immediately measured. Kruskal-Wallis tests (**f,** P = 0.7649; n = 8. **g,** P = 0.9132; n = 8) followed by Dunn's multiple comparison test (two-sided) were performed.

h,i, The knockdown of *CoRest-F* or *Rpd3* did not affect memory following unpaired training. The flies with indicated transgenes were fed RU, and subjected to spaced training. On the following day, unpaired training, in which electric shocks precede the exposure to the odors, was carried out, and memory was measured immediately. Kruskal-Wallis tests (**h,** P = 0.3511; n = 8. **i,** P = 0.5069; n = 8) followed by Dunn's multiple comparison test (two-sided) were performed.

Data are represented as mean ± s.e.m. n.s., not significant $P > 0.05$; *** P < 0.001, which is based on experimental replicates using different pooled samples, and the number is indicated as n above. Source data are provided as a Source Data file.

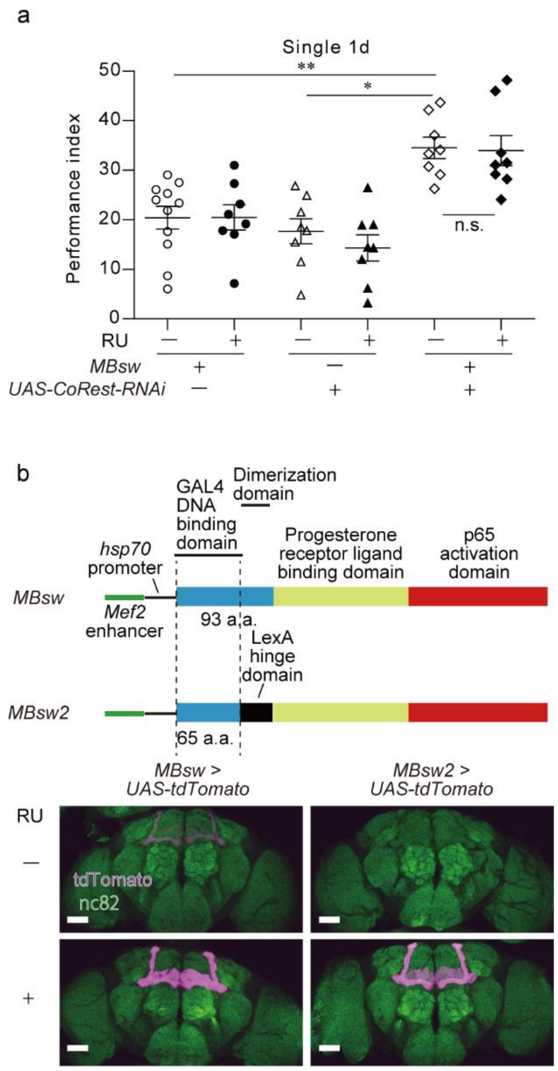

Supplementary Fig. 13 Schematic diagram of MBsw2.

a, Knockdown of *CoRest-F* by MBsw. The flies with indicated transgenes were fed RU486 (RU) for 3 days, and 1-day memory after a single training was assessed. A Kruskal-Wallis test ($P < 0.0001$; $n = 8, 8, 11, 8, 8, 8$) followed by Dunn's multiple comparison test (two-sided) was performed.

b, Schematic diagram of MBsw2.The partial dimerization domain of GAL4 was replaced with a LexA hinge domain. The flies with indicated transgenes were fed RU for 3 days, and the brain was immunostained with the anti-DsRed antibody (magenta). Anti-nc82 staining was shown in green, indicating the presynaptic sites. The images are

representative of experimental replicates (n = 3 for all). (upper) Scale bar: 50 μm. Data are represented as mean ± s.e.m. n.s., not significant $P > 0.05$; *, $P < 0.05$; **, $P < 0.01$; *** $P < 0.001$, which is based on experimental replicates using different pooled samples, and the number is indicated as n above. Source data are provided as a Source Data file.

Supplementary Table 1 Proteins identified in the coimmunoprecipitates with Rpd3
Rpd3-immunocomplex prepared from flies expressing TRPA1 without (A) or with heat shock (B), or flies without TRPA1 with heat shock (C) was analyzed by the shotgun LC-MS/MS analysis.
The peptide data was analyzed by Proteome discoverer 2.1., and the following scores were shown.
Sum PEP score, the sum of the scores of the individual peptides
Coverege, The percent coverage calculated by dividing the number of amino acids in all found peptides by the total number of amino acids in the entire protein sequence.
Peptides, The number of distinct peptide sequences in the protein group
PSMs, The total number of identified peptide sequences for the protein, including those redundantly identified.
Unique Peptides, The number of peptide sequences unique to a protein group.
AAs, the number of the amino acids in the entire protein sequuence
Fold enrichment in sample B / sample A ((B)/(A)) and sample B / sample C ((B)/(C)) were shown.

Accession	Gene name	Exp. q-value	Sum PEP Score	Coverage (%)	# Peptides	# PSMs	# Unique Peptides	# AAs	MW [kDa]	(A) Amount in the untreated sample (TRPA1)	(B) Amount in the hs-treated sample (TRPA1)	(C) Amount in the hs-treated sample (-TRPA1)	(B)/(A)	(B)/(C)	HDAC2 (Rpd3) complex
Q94517	Rpd3	0	402.859	74.6641	49	421	49	521	58.3	967810289.2	745019965.5	887410272.2	0.77	0.84	
Q9VNF7	MTA1-like	0	126.898	38.5071	34	130	1	844	92.7	11284715.78	6054035.875	3442014.781	0.54	1.76	NuRD
Q59E34	Mi-2	0	38.994	7.71558	11	20	11	1983	224.2	1989220.385	4769408.366	3672924.804	2.40	1.30	NuRD
A0A0B4K(	MBD-like	0	48.829	30.5882	10	41	10	340	36.3	18202976.06	23521349.79	18370316.48	1.29	1.28	NuRD
A0A0B4K7	Sin3A	0	120.961	26.3795	34	100	34	2066	220.9	19567021.46	17443854.59	14552056.95	0.89	1.20	Sin3, CoRest
C7LAG1	CoRest	0	21.402	9.8301	8	20	8	824	87.3	4820179.009	6136161.932	4313027.471	1.27	1.42	CoRest
P10676	ninaC	0	421.611	59.427	82	360	23	1501	174.2	85260150.47	38164521.28	34265331.45	0.45	1.11	
M9PCU0	ninaC	0	318.408	57.4449	60	288	1	1135	131.8	1520567.805	739563.0938	884278.5313	0.49	0.84	
P11147	Hsc70-4	0	217.88	65.4378	49	190	43	651	71.1	80230831.7	103015412.9	101641828.9	1.28	1.01	
Q9V8R9	cora	0	135.904	32.8033	42	99	42	1698	184.1	9946304.135	9056792.294	8963050.833	0.91	1.01	
Q9BIR7	Hsp70Bc	0	203.865	62.5585	40	136	5	641	70.2	0	24171643.45	53485415.78	N/A	0.45	
P82910	Hsp70Aa	0	209.523	62.4611	40	137	5	642	70.1	0	18003939.45	51288879.56	N/A	0.35	
Q9W392	CCT2	0	110.207	61.8692	26	74	26	535	58	20670075.76	16664617.96	21455224.44	0.81	0.78	
A0A0S0W	Mhc	0	88.798	20.0815	26	47	26	1962	224.4	1351046.833	4230360.313	1633411.262	3.13	2.59	
P02518	Hsp27	0	109.108	82.6291	23	83	23	213	23.6	0	244688175.3	196273797.2	N/A	1.25	
Q9VA91	RpS7	0	89.228	80.4124	23	79	23	194	22.2	79773508.27	72827556.15	70879388.29	0.91	1.03	
M9NFP8	CG43078	0	58.349	12.4409	22	43	22	2540	285.6	4463921.667	2892988.398	2378149.865	0.65	1.22	
Q24572	Caf1	0	101.977	56.9767	21	85	4	430	48.6	27824091.25	34779195.22	35502966.94	1.25	0.98	
E1JIL4	Caf1-55	0	82.961	56.8765	19	70	2	429	48.5	708632.3594	0	1771399.625	0.00	0.00	
Q9VNI3	CG1218	0	58.95	42.539	19	59	19	449	51.1	21781616.33	14883860.58	15351620.3	0.68	0.97	
M9NE68	Hsp23	0	117.564	95.1613	18	74	18	186	20.6	0	100647158.3	485136174.9	N/A	0.21	
P12613	T-cp1	0	88.289	43.8061	17	61	17	557	59.5	15126242.08	15644138.75	15003980.49	1.03	1.04	
A8DYJ2	hts	0	67.228	14.0207	17	56	17	1833	205.1	12432896.11	9718663.862	21563679.72	0.78	0.45	
O97125	Hsp68	0	66.649	32.2835	17	46	10	635	69.7	0	3692766.79	11122117.99	N/A	0.33	
C1C553	Mf	0	41.575	43.4505	16	45	2	313	36.3	702728.4922	0	730762.4531	0.00	0.00	
P39018	RpS19a	0	58.563	71.7949	16	61	16	156	17.3	22226729.11	18042287.72	14454801.32	0.81	1.25	
M9MSN3	rdgA	0	54.059	20.4163	16	43	16	1009	112.9	12581186.46	13348170.17	13567586.79	1.06	0.98	
Q9VFC7	Mf	0	49.163	47.9452	16	48	2	365	41.6	3553938.875	5091979.254	3323382.469	1.43	1.53	
Q24560	betaTub56D	0	60.371	53.6913	15	49	15	447	50.1	13037949.71	8508716.715	9979970.896	0.65	0.85	
P02515	Hsp22	0	58.027	75.8621	15	34	15	174	19.8	0	6048888.906	38364938.62	N/A	0.16	

P15372	Arr1	0	46.116	44.5055	15	36	15	364	40.7	4676472.893	4488139.813	4932498.294	0.96	0.91
G3M3A2	RpS3	0	34.337	56.9106	14	36	14	246	27.5	12572504.77	12982376.75	13077915.51	1.03	0.99
Q9XZ09	Sap130	0	42.158	20.1805	14	29	14	1219	123.5	4523420.254	5165159.242	2912575.516	1.14	1.77
E1JIK9	Mf	0	35.006	56.087	14	44	1	230	26.6	2400243.141	3353681.648	2917924.758	1.40	1.15
Q9VSK5	Gug	0	46.9	12.0907	14	29	14	1985	210.3	6374638.734	2651988.308	2770800.634	0.42	0.96
P13060	EF2	0	53.059	25	14	22	14	844	94.4	827154.8203	2222534.604	16317019.08	2.69	0.14
X2JGG6	Hsp26	0	94.115	79.8077	13	71	13	208	23	754062.8984	156869148.8	900475906.4	208.03	0.17
F3YDH0	Hsc70-3	0	56.428	22.561	13	45	8	656	72.2	2164753.762	2521602.792	2738083.487	1.16	0.92
P08985	His2Av	0	50.309	66.6667	11	39	9	141	15	64078072.95	71202002.85	54536393.7	1.11	1.31
Q9V597	RpL31	0	26.019	62.9032	11	27	11	124	14.5	17399235.43	14747982.44	16489288.38	0.85	0.89
Q9VN91	rtp	0	34.538	54.0404	11	34	11	198	22.7	36201033.13	9227083.968	16320103.88	0.25	0.57
P55830	RpS3A	0	33.701	45.1493	11	27	11	268	30.3	6544983.224	6077771.974	5562897.874	0.93	1.09
P35381	blw	0	26.856	21.7391	11	22	11	552	59.4	4528721.204	3560993.484	4830529.01	0.79	0.74
P15357	RpS27A	0	24.967	43.5897	10	27	10	156	17.9	54819419.09	50838096.86	53761889.78	0.93	0.95
A0A0B4LH	Act87E	0	33.671	35.1064	10	25	10	376	41.8	18623235.87	26628517.96	20485690.85	1.43	1.30
O62619	PyK	0	30.898	24.7655	10	26	10	533	57.4	7818848.849	6869361.313	8255422.944	0.88	0.83
A0A0B4K6	RpS8	0	39.262	54.8077	10	29	10	208	23.7	5432762.323	6388417.701	6120547.909	1.18	1.04
P06603	alphaTub84B	0	41.057	32	9	19	9	450	49.9	20878628.33	11743667.84	8618396.63	0.56	1.36
A0A0B4LG	RpL11	0	28.559	51.6304	9	27	9	184	21.1	10762258.28	10357152.62	4302627.546	0.96	2.41
X2JCX8	RpS14b	0	26.238	59.6026	9	26	9	151	16.3	6758056.406	6230443.953	5507445.607	0.92	1.13
O01666	ATPsyngamma	0	36.042	32.9966	9	20	9	297	32.9	6800136.982	6105486.701	6410791.607	0.90	0.95
Q24400	Mlp84B	0	19.841	30.9091	9	26	9	495	53.5	4841760.598	5871486.534	5498370.18	1.21	1.07
P41042	RpS4	0	25.228	40.9962	9	20	9	261	29.1	5136963.826	5123706.411	4820923.315	1.00	1.06
P19109	Rm62	0	27.167	15.9944	9	23	9	719	78.5	2420046.938	2505651.099	3005165.167	1.04	0.83
P48588	RpS25	0	19.066	50.4274	8	18	8	117	13.2	12209748.1	26579978.29	4844698.909	2.18	5.49
C6TP87	eEF1alpha1	0	26.184	25.27	8	23	8	463	50.3	15836738.99	15067204.71	17462189.26	0.95	0.86
M9PF79	simj	0	25.57	16.1153	8	23	8	937	98	8668242.982	10164848.41	7204113.102	1.17	1.41
O02649	Hsp60	0	20.62	23.5602	8	18	8	573	60.8	6243290.448	5576664.366	5291548.185	0.89	1.05
A1Z7S3	Rab32	0	27.528	19.242	8	15	8	686	74.7	3901962.927	4253446.401	5146282.361	1.09	0.83
Q9VWH4	l(1)G0156	0	26.84	29.443	8	25	8	377	40.8	4914854.568	3784066.652	3525324.628	0.77	1.07
A1Z6Z7	CG1620	0	51.854	21.6724	8	34	3	586	63.9	7134834.285	3756685.71	1910899.04	0.53	1.97
Q9VLC5	Aldh	0	20.252	21.5385	8	15	8	520	57	3345526.544	2276217.708	3016517.108	0.68	0.75
E1JIJ5	Vha55	0	17.956	22.449	8	11	8	490	54.5	1919443.926	1953904.344	2891898.887	1.02	0.68
P84051	His2A	0	51.41	60.4839	7	42	5	124	13.4	24631145.15	22062152.72	14533229.51	0.90	1.52
Q9VZF9	Cpr64Ad	0	30.279	57.4899	7	24	7	247	24.3	18357990.2	19296849.82	2623529.477	1.05	7.36
P17704	RpS17	0	25.817	62.5954	7	24	7	131	15.3	21613491.05	16117412.18	13596794.66	0.75	1.19
P84040	His4	0	17.967	43.6893	7	20	7	103	11.4	9536393.518	7485994.461	3020606.839	0.78	2.48
E1JJM9	RpS15Aa	0	18.115	61.5385	7	15	7	130	14.8	7735578.674	6806233.629	6438121.701	0.88	1.06
P22700	Ca-P60A	0	23.197	8.62745	7	15	7	1020	111.6	2043566.419	3152918.391	2853987.247	1.54	1.10
Q9VV75	UQCR-C2	0	23.142	31.1364	7	12	7	440	45.4	2376307.234	3118124.859	3507673.587	1.31	0.89
Q9VAC1	CG7920	0	18.628	24.3187	7	14	7	477	51.8	3243059.117	2948967.854	4006161.885	0.91	0.74
Q8IMV6	Saf-B	0	18.436	10.4526	7	9	7	928	102	1539987.761	2904389.438	3427030.059	1.89	0.85
M9NFC0	wupA	0	26.018	38.191	7	25	3	199	23.4	3140191.984	2748347.249	2503667.303	0.88	1.10
P13607	Atpalpha	0	13.94	8.16523	7	14	7	1041	115.5	2137956.919	1693054.919	1928072.578	0.79	0.88
X2JB48	sesB	0	18.497	20.0669	6	11	6	299	32.9	10493545.65	10347053.03	9637233.417	0.99	1.07

P02283	His2B	0	24.069	39.8374	6	21	6	123	13.7	13006865.48	7377205.182	4379997.945	0.57	1.68
P41094	RpS18	0	13.54	40.7895	6	18	6	152	17.6	7243862.518	7351199.094	5224847.078	1.01	1.41
Q7PLL3	eIF4B	0	22.177	22.0044	6	15	6	459	52.2	2964695.258	5538432.047	5332050.192	1.87	1.04
Q7Z020	TrpA1	0	12.848	6.59983	6	6	6	1197	135.1	0	4395985.737	0	N/A	N/A
P19107	Arr2	0	22.455	22.9426	6	18	6	401	45	3709281.26	2492894.708	4016643.378	0.67	0.62
C0HK95	fau	0	13.835	51.145	6	17	5	131	14.4	855638.5547	2131678.592	2954522.411	2.49	0.72
Q7JWQ7	Swim	0	13.571	13.2251	6	15	6	431	48.8	1563960.477	2094422.937	2018896.406	1.34	1.04
E1JGZ3	CG1620	0	36.414	23.6324	6	25	1	457	50.4	0	743683.4043	1055506.438	N/A	0.70
M9PHR2	up	0	14.43	13.8539	5	10	1	397	47.5	0	0	3001599.219	N/A	0.00
Q9VY42	CG1461	0	11.526	13.9721	5	5	5	501	55.9	0	0	3069784.445	N/A	0.00
Q7JZW2	RpS15	0	25.128	39.1892	5	17	5	148	17	12194926.13	16603063.26	16940452.95	1.36	0.98
Q9W237	RpS16	0	12.024	21.6216	5	15	5	148	16.8	16534770.15	14614283.96	12966200.76	0.88	1.13
Q9VJ19	RpL30	0	17.881	54.955	5	14	5	111	12.2	14661824.12	10723487.9	20496627.13	0.73	0.52
P36188	wupA	0	11.552	14.4981	5	16	1	269	30.1	14992814.91	10132858.84	0	0.68	N/A
P55828	RpS20	0	21.75	42.5	5	24	5	120	13.5	9803916.521	8414065.447	7815502.292	0.86	1.08
X2JDU0	RpL9	0	12.477	31.5789	5	15	5	190	21.4	8024036.341	6509929.404	10077546.82	0.81	0.65
Q8IM93	CG32017-RB	0	14.727	11.2033	5	11	5	482	55.3	2201052.842	3385017.237	4632138.727	1.54	0.73
P48159	RpL23	0	22.192	40.7143	5	14	5	140	14.9	4085154.12	3069857.771	2281161.434	0.75	1.35
Q9VNW6	CG7470	0	15.824	8.76289	5	8	5	776	84	3747523.671	2570529.859	3582915.688	0.69	0.72
M9NH07	up	0	14.076	13.7056	5	12	1	394	47.2	2738812.031	2344494.473	2387289.555	0.86	0.98
Q9W1H8	Thiolase	0	8.971	10.2345	5	7	5	469	50.6	2402524.152	525236.5078	1467340.453	0.22	0.36
Q9VKQ2	CG17127	0	13.356	75.5102	4	15	4	98	10.8	22036769.91	36526385.44	8573459.857	1.66	4.26
Q24154	RpL29	0	26.845	30.2632	4	29	4	76	8.9	7902226.427	10100368.72	8879488.707	1.28	1.14
O97102	smt3	0	9.046	38.8889	4	8	4	90	10.1	2350814.84	7044246.072	4079971.225	3.00	1.73
Q9W1B9	RpL12	0	18.966	28.4848	4	13	4	165	17.7	6951536.099	6523870.324	6844341.568	0.94	0.95
Q8SYJ2	ND-MLRQ	0	9.324	55.4217	4	9	4	83	9.4	2395253.505	5375343.106	2920055.115	2.24	1.84
Q9V3E7	Ref1	0	14.344	19.5489	4	9	4	266	27.8	4534058.036	4851266.182	5712961.069	1.07	0.85
A0A0B4LF	RpS23	0	20.783	34.965	4	18	4	143	16	3864632.122	4475390.645	4078021.935	1.16	1.10
B7YZQ3	prom	0	12.139	4.45344	4	8	4	1235	138.2	3599092.238	4146991.622	5175061.547	1.15	0.80
X2JKU5	RpS5a	0	18.359	23.2456	4	9	4	228	25.4	3158764.031	3714609.176	1894144.531	1.18	1.96
Q9W2J4	BEST:GH09393	0	13.488	10.1504	4	11	4	532	60.1	4413477.404	3675928.94	4472382.352	0.83	0.82
P55841	RpL14	0	16.53	20.4819	4	12	4	166	19.2	3811486.005	3304869.203	2756451.617	0.87	1.20
P41073	Pep	0	14.125	8.51955	4	8	4	716	78	1023023.069	3156347.309	2671022.121	3.09	1.18
P00334	Adh	0	19.485	28.125	4	7	4	256	27.7	0	2968401.112	6418530.867	N/A	0.46
P08928	Lam	0	14.963	9.6463	4	8	4	622	71.3	1637730.876	2783197.598	2241801.005	1.70	1.24
Q9VZ22	Lint-1	0	14.196	13.9535	4	14	4	602	67.9	2232850.809	2756971.492	3270213.543	1.23	0.84
Q8SYR3	Rsf1	0	10.751	23	4	9	4	200	21.4	1459546.891	2697379.949	2930132.914	1.85	0.92
A0A0B4LE	CG8635	0	8.272	10.1485	4	6	4	404	45.3	2426435.313	2130799.818	2839174.414	0.88	0.75
Q94920	porin	0	11.521	18.7943	4	6	4	282	30.5	0	1710676.177	1575810.916	N/A	1.09
Q7KKI0	CCT5	0	9.723	13.8376	4	8	4	542	59.2	0	1625098.684	1992681.978	N/A	0.82
A0A0B4K(	HtrA2	0	13.254	11.8483	4	6	4	422	46	2538365.438	1411625.047	1578824	0.56	0.89
Q9VWP5	htk	0	7.906	1.85036	3	4	3	2486	259.3	738610.7715	0	407652.9102	0.00	0.00
Q02748	eIF-4a	0	5.892	11.9107	3	3	3	403	45.8	0	0	2017314.992	N/A	0.00
B5X0J4	ple	0	8.03	6.73575	3	3	3	579	66	0	0	1851978.927	N/A	0.00
O97064	Ccp84Ag	0	8.053	27.2251	3	7	3	191	19.4	7795490.151	8584183.035	0	1.10	N/A

Q9VSU3	CG13315	0	8.966	40.5797	3	8	3	69	8	5699590.957	5602532.953	576649.8867	0.98	9.72
X2JC80	sta	0	7.918	17.7778	3	8	3	270	30.2	3013616.427	4552827.219	3848667.898	1.51	1.18
Q9VYI2	Brms1	0	5.614	12.3552	3	5	3	259	29.5	5295724.92	3978323.623	2490846	0.75	1.60
Q9VFJ0	Cyp313a1	0	6.533	6.70732	3	4	3	492	56.5	0	3769811.688	5489749.146	N/A	0.69
M9PB84	RpS2	0	4.812	12.3596	3	7	3	267	28.9	1941077.104	3441275.525	3976832.699	1.77	0.87
Q9VVU2	RpL26	0	6.949	12.0805	3	5	3	149	17.3	1673030.096	3431379.32	4255315.457	2.05	0.81
Q9V426	vig	0	12.097	9.18367	3	7	3	490	52.7	2148857.422	2868650.062	2866470.576	1.33	1.00
P54385	Gdh	0	7.224	6.9395	3	5	3	562	62.5	1742044.065	2551410.559	0	1.46	N/A
A0A0B4KE	14-3-3zeta	0	12.049	14.5161	3	7	3	248	28.2	1766273.725	2410791.77	1989242.294	1.36	1.21
L0MQ04	ATPsynbeta	0	8.218	10.1761	3	5	3	511	54.6	1898347.766	2263113.406	2190149.401	1.19	1.03
P02255	His1	0	6.505	12.8906	3	6	3	256	26.3	1731910.916	2227304.805	1900979.547	1.29	1.17
Q9VSW4	mRRF1	0	8.339	15.748	3	7	3	254	28.5	2912545.589	2146791.992	2676735.945	0.74	0.80
M9PEA2	CDK2AP1	0	14.023	16.9492	3	9	3	295	30.7	1660513.79	2058197.114	2157309.114	1.24	0.95
X2JDA5	Gs2	0	7.666	12.0643	3	7	3	373	41.7	2433561.984	1650556.363	5209830.156	0.68	0.32
C0HK94	CG45078	0	6.281	26.9939	3	5	2	163	18.8	498225.9375	1417738.742	0	2.85	N/A
Q9V397	Mtpalpha	0	7.283	7.02427	3	4	3	783	84	0	1309696.375	1562746.063	N/A	0.84
P35415	Prm	0	9.5	5.46075	3	4	3	879	102.3	0	1121654.863	638579.8613	N/A	1.76
Q9W4L1	mRpL33	0	8.233	60.9375	3	5	3	64	7.7	2308968.216	964333.8164	713888.5625	0.42	1.35
P13217	norpA	0	8.393	3.10502	3	6	3	1095	124.8	3471964.063	726763.3112	920395.5762	0.21	0.79
Q9VM14	muc	0	3.97	6.64063	3	3	3	512	54.2	0	712518.959	270563.0977	N/A	2.63
P22979	Hsp67Bc	0	7.171	27.1357	3	4	3	199	22.2	0	241342.2656	2305721.788	N/A	0.10
Q9VWG2	CG14220-RA	0.013	2.311	6.72783	2	2	2	327	38.3	847940.0742	0	3612195.492	0.00	0.00
P55935	RpS9	0	3.237	10.7692	2	2	2	195	22.6	1256635.926	0	0	0.00	N/A
M9PHM6	RpL18	0	5.502	14.3617	2	3	2	188	21.7	1716453.449	0	0	0.00	N/A
A0A0B4KE	RpL38	0	4.89	17.1429	2	2	2	70	8.2	12147231.69	0	3930533.32	0.00	0.00
A4V3G1	Ald	0.013	2.843	6.33609	2	3	2	363	39.5	0	0	652045.3809	N/A	0.00
Q9W503	CG3091	0	4.944	6.93069	2	3	2	303	34.6	0	0	2686329.219	N/A	0.00
P17276	Hn	0	6.705	7.52212	2	2	2	452	51.6	0	0	1887633.844	N/A	0.00
P02828	Hsp83	0	3.023	4.1841	2	3	2	717	81.8	0	0	1611730.67	N/A	0.00
A0A0B4K(	Stat92E	0	7.219	4.64548	2	3	2	818	92.5	0	0	1429757.047	N/A	0.00
A0A0B4K(	bel	0	7.139	3.99501	2	2	2	801	85.4	0	0	4561418.617	N/A	0.00
M9NFH8	CG9674	0	5.958	1.2766	2	2	2	2115	232.1	0	0	1917462.746	N/A	0.00
Q8SWS3	CG13049	0	8.187	28.7293	2	6	2	181	17.3	11957017.11	14282236.19	1993795.719	1.19	7.16
Q9VVZ4	CG14095	0	11.267	30.2469	2	8	2	162	15.9	13941723.83	13375445.48	1785546.563	0.96	7.49
Q9W1Q4	CG9812	0	17.219	18.6747	2	9	2	332	34.5	9110602.336	12980805.36	2381473.617	1.42	5.45
Q9VV18	CG13047	0	4.416	22.3529	2	6	2	170	16.6	10485386.32	12066830.45	0	1.15	N/A
E2QCP0	His3.3A	0	10.353	28.6765	2	5	2	136	15.3	6487947.938	7547454.625	4247874.797	1.16	1.78
Q9VUZ0	SsRbeta	0	10.317	19.4737	2	6	2	190	21.2	5251691.293	5401821.457	5788629.793	1.03	0.93
Q9W141	CG4692	0	6.544	23.3645	2	7	2	107	12.5	2660625.5	4303989.414	4944584.76	1.62	0.87
Q9VVH5	UQCR-Q	0	4.242	33.7079	2	4	2	89	10.1	2564173.867	4274689.313	5030356.066	1.67	0.85
Q9V3P0	Jafrac1	0	6.283	13.4021	2	4	2	194	21.7	3307798.945	3535806.125	3255330.82	1.07	1.09
Q9W229	RpS24	0	4.761	14.5038	2	5	2	131	15	3723454.59	3389926.966	5383087.531	0.91	0.63
Q9W078	Cpr62Bb	0	5.209	14.9485	2	5	2	194	21.2	1995377.773	2974720.93	0	1.49	N/A
Q9W2U8	Neb-cGP	0	3.356	62.5	2	6	2	48	5.1	3293587.684	2933632.219	2424710.184	0.89	1.21
M9MRF2	RpL37A	0	12.883	27.1739	2	9	2	92	10.3	2659197.667	2931897.1	1989811.006	1.10	1.47

Q4V619	mIF3	0	9.288	15.9483	2	3	2	232	25.8	1567461.992	2850617.328	2315311.094	1.82	1.23
Q53ZT0	DnaJ-1	0	4.46	15.8683	2	4	2	334	37	0	2574897.219	1981386.49	N/A	1.30
Q9W4N8	Vap33	0	5.375	11.8959	2	5	2	269	29.5	0	2438202.73	2652380.641	N/A	0.92
P07487	Gapdh2	0	5.7	8.73494	2	4	2	332	35.3	3934054.855	2151154.543	0	0.55	N/A
Q9VZG1	Cpr64Ab	0	3.614	20.8333	2	2	2	120	12.5	4013172.695	2068178.203	0	0.52	N/A
Q500Y7	UQCR-6.4	0	4.037	38.5965	2	5	2	57	6.3	3554335.672	2064087.641	433894.9688	0.58	4.76
Q9VBU9	RpS27	0	3.731	14.2857	2	5	2	84	9.4	2333544.418	1854069.578	2893267.672	0.79	0.64
Q94523	SdhA	0	9.235	3.78215	2	5	2	661	72.3	1797031.5	1824818.453	1896514.469	1.02	0.96
Q95RB2	Cpr49Ae	0	5.371	10.4478	2	4	2	134	14.3	1261056.301	1798706.602	3645149.063	1.43	0.49
A0A0B4KE	sbb	0	3.509	1.33047	2	4	2	2330	233.1	341227.0781	1746911.805	0	5.12	N/A
Q9W3L4	CG2233	0	4.076	9.90338	2	3	2	414	46.5	0	1741178.781	0	N/A	N/A
X2JEX8	Yp3	0	6.643	7.38095	2	2	2	420	46.1	1659393.742	1646337.063	0	0.99	N/A
M9PF20	tral	0	8.916	5.93607	2	4	2	657	69.8	0	1591842.934	1451189.684	N/A	1.10
Q9VQ35	mRpL48	0	4.067	16.0221	2	3	2	181	20.9	2104504.922	1573954.742	0	0.75	N/A
P50887	RpL22	0	7.18	8.02676	2	8	2	299	30.6	1940864.773	1521053.672	1279827.477	0.78	1.19
Q9V3Z9	BG:DS00180.3	0	4.42	11.5523	2	5	2	277	29.7	1334467.105	1499889.434	0	1.12	N/A
Q9W3Y0	CG3224	0	6.502	20.9877	2	7	2	162	18.9	1480837.559	1461780.557	1912675.385	0.99	0.76
E1JGL8	CG30122	0	14.673	2.67296	2	9	2	1272	140.5	1548688.844	1456514.355	1683468.703	0.94	0.87
A8DYI6	Phb2	0	5.637	8.28402	2	2	2	338	37.3	0	1391405.289	1096438.188	N/A	1.27
Q86B94	mfas	0	3.663	2.87293	2	4	2	905	100.2	958484.4688	1269863.363	0	1.32	N/A
Q9I7K0	Jupiter	0	4.403	12.0192	2	3	2	208	22.3	601285.75	1218124.715	0	2.03	N/A
P25007	Cyp1	0	7.228	10.5727	2	4	2	227	24.7	0	1168355.703	1956659.96	N/A	0.60
A0A0B4KH	chp	0	13.145	3.36323	2	11	2	1338	154.6	2112477.813	1124817.094	2585811.141	0.53	0.43
A0A0B4KF	LamC	0	5.8	5.3125	2	2	2	640	72.1	0	1123421.203	0	N/A	N/A
O62530	AP-2mu	0	3.294	3.89016	2	4	2	437	49.8	0	1120362.859	1040133.809	N/A	1.08
P46461	comt	0	4.361	3.75839	2	3	2	745	82.5	1059059.141	1109128.922	0	1.05	N/A
P54367	CkIalpha	0	3.551	7.12166	2	3	2	337	39.5	1109159.934	1045957.297	1166196.441	0.94	0.90
M9MSL3	Hsc70Cb	0	4.074	2.27273	2	3	2	836	91.8	2103046.676	1034598.742	853461.5391	0.49	1.21
Q8IGY1	Tm1	0	7.634	3.79747	2	4	2	711	80	637977.0156	1029770.578	1026158.195	1.61	1.00
O76521	Kap-alpha1	0	2.934	3.8674	2	3	2	543	60	0	983436.1172	1107750.668	N/A	0.89
Q9VB52	l(3)mbt	0	4.359	1.96344	2	4	2	1477	162.9	896680.1523	939410.7578	0	1.05	N/A
P21521	Syt1	0	4.33	5.06329	2	5	2	474	53.2	1347668.57	936913.4609	1295979.816	0.70	0.72
O02195	Trip1	0	5.574	7.36196	2	3	2	326	36.1	0	795631.707	1642018.867	N/A	0.48
M9PHL7	Flo2	0	4.551	5.35714	2	2	2	448	49.5	0	738828.3535	0	N/A	N/A
Q8IQ80	pst	0	2.903	3.39463	2	3	2	707	77.4	0	689472.8281	2361700.175	N/A	0.29
P48605	Cctgamma	0	5.922	4.22794	2	4	2	544	59.4	1736734.031	649559.25	650800.8594	0.37	1.00
A8JNK1	ens	0	5.967	1.85334	2	5	2	1241	135.7	1184188.758	580810.3967	23231.00781	0.49	25.00
B7Z001	FASN1	0	5.437	1.06299	2	2	2	2540	277.8	498967.0625	557598.8906	0	1.12	N/A
Q9VI10	SmD2	0	5.137	15.9664	2	3	2	119	13.5	835071.4512	509891.9746	0	0.61	N/A
Q0E8E8	Mpcp	0.018	1.227	2.52809	1	1	1	356	38.8	252260.6758	0	0	0.00	N/A
Q9VND7	CG2931	0.029	1.041	3.97351	1	1	1	302	33.6	338490.5625	0	0	0.00	N/A
Q9W3E2	PIP82	0.013	2.284	1.33891	1	1	1	1195	132.3	471306.7539	0	0	0.00	N/A
Q0E8Y1	PIP5K59B	0.013	1.99	2.04866	1	1	1	781	86	497778.1875	0	0	0.00	N/A
Q9V496	Rfabg	0.013	2.023	0.3581	1	2	1	3351	372.4	569208.2656	0	675307.4609	0.00	0.00
Q7JXC4	P32	0.027	1.055	3.42205	1	1	1	263	29	614611.7188	0	0	0.00	N/A

A1Z784	ACC	0.013	1.605	0.56406	1	2	1	2482	278.4	627321.8203	0	1727938.953	0.00	0.00
Q8MT18	CG5028	0.014	1.54	2.48756	1	1	1	402	44.4	630429.7344	0	0	0.00	N/A
Q9W1M9	Rrp4	0.013	2.775	4.02685	1	1	1	298	33.6	667385.9375	0	0	0.00	N/A
Q9VEX6	bor	0	3.944	2.31788	1	2	1	604	68.3	778728.1875	0	933052.5781	0.00	0.00
Q9VKM7	CG6089	0.013	2.129	1.62482	1	1	1	677	75.6	778774.9648	0	0	0.00	N/A
P23226	Map205	0.013	2.819	1.51899	1	3	1	1185	126.6	813109.1641	0	593726.6563	0.00	0.00
P21914	SdhB	0.013	2.236	3.367	1	1	1	297	33.7	959783.3477	0	0	0.00	N/A
M9PG47	RpS26	0.013	1.996	13.1579	1	1	1	114	13.3	1100433.813	0	0	0.00	N/A
Q95PE4	Mpp6	0.013	1.798	6.17284	1	1	1	162	18.5	1135067.734	0	0	0.00	N/A
M9PHQ0	shi	0	3.104	2.12054	1	1	1	896	99.8	1711311.484	0	0	0.00	N/A
Q9VHD1	CG16779	0.013	1.696	0.70707	1	1	1	1980	207.7	2054681.867	0	0	0.00	N/A
Q9VT33	CG6767	0	3.192	4.38144	1	3	1	388	42.7	2096243.047	0	2004849.59	0.00	0.00
M9NDP2	CG7394	0	6.393	16.1017	1	2	1	118	12.9	2634597.875	0	3083518	0.00	0.00
P05812	Hsp67Ba	0.016	1.408	7.56303	1	2	1	238	26.6	6504290.758	0	0	0.00	N/A
Q9VKG8	Dlg5	0.029	1.012	0.6263	1	1	1	1916	209.7	7033423.398	0	0	0.00	N/A
Q9VB86	TwdlT	0.029	1.028	2.7972	1	1	1	286	27	0	0	1372603.906	N/A	0.00
Q9VBN5	RpL27	0.013	2.478	7.40741	1	1	1	135	15.9	0	0	1327863.672	N/A	0.00
P04359	RpL32	0.026	1.089	6.71642	1	1	1	134	16	0	0	391358.9746	N/A	0.00
Q9VTA8	CG14153	0.029	1.021	6.75105	1	1	1	237	27.5	0	0	1380635.793	N/A	0.00
Q9W3W8	RpL17	0.013	2.213	5.91398	1	1	1	186	21.6	0	0	961493.7227	N/A	0.00
Q95083	Prosalpha5	0	4.067	4.91803	1	1	1	244	26.9	0	0	1297764.531	N/A	0.00
Q9VBL2	CG5890-RA	0.013	1.735	10.1942	1	1	1	206	24	0	0	0	N/A	N/A
Q8IQW5	CG14207-RB	0.013	2.738	13.0208	1	1	1	192	21.8	0	0	2637378.984	N/A	0.00
Q9VTP4	RpL10Ab	0	4.794	5.99078	1	1	1	217	24.3	0	0	3485973.094	N/A	0.00
Q9VMC9	KFase	0.013	2.59	3.66667	1	1	1	300	34.9	0	0	1383053.063	N/A	0.00
P22769	Prosalpha4	0	3.846	8.83534	1	2	1	249	28	0	0	2848129.656	N/A	0.00
Q94516	ATPsynB	0.024	1.098	3.7037	1	1	1	243	27.3	0	0	750190.4063	N/A	0.00
O76742	Rab7	0.014	1.521	4.83092	1	1	1	207	23.3	0	0	1020528.266	N/A	0.00
Q8IRH8	tfc	0.017	1.293	2.12766	1	1	1	376	42.9	0	0	1908787.672	N/A	0.00
Q9U9Q4	eIF-3p40	0.013	2.569	3.25444	1	1	1	338	38.4	0	0	524448.6797	N/A	0.00
P48809	Hrb27C	0	3.214	3.80048	1	1	1	421	44.7	0	0	1830851.938	N/A	0.00
Q9VIW5	CG10237	0.013	2.575	3.7037	1	1	1	324	37.6	0	0	1971984.516	N/A	0.00
Q9V3P3	REG	0	2.908	5.30612	1	1	1	245	28.1	0	0	1530383.125	N/A	0.00
P48596	Pu	0.014	1.491	3.7037	1	1	1	324	35.5	0	0	2895696.578	N/A	0.00
M9PDQ9	CG31974	0.018	1.186	8.65385	1	1	1	416	47.8	0	0	1165439.715	N/A	0.00
Q7K485	cathD	0.013	1.613	6.88776	1	1	1	392	42.4	0	0	0	N/A	N/A
Q9W5H1	EG:BACR37P7.9	0.013	2.42	5.15152	1	1	1	330	35.8	0	0	628973.8906	N/A	0.00
Q9VFV9	Droj2	0.013	2.156	3.22581	1	1	1	403	45.2	0	0	1133695.563	N/A	0.00
E1JJF3	Inx2	0	3.082	4.35967	1	1	1	367	42.5	0	0	0	N/A	N/A
M9PCC1	Rack1	0	3.195	3.77358	1	1	1	318	35.6	0	0	1715564.211	N/A	0.00
X2JFR6	eIF2alpha	0.013	2.844	3.51906	1	1	1	341	38.6	0	0	2500944.938	N/A	0.00
Q9VKJ0	hgo	0.013	2.208	2.2779	1	1	1	439	49.4	0	0	1197470.641	N/A	0.00
Q9VPF3	CG11796	0.016	1.299	4.47368	1	1	1	380	43.3	0	0	5436149.305	N/A	0.00
Q9VJI1	CG17928	0	5.029	3.1746	1	1	1	441	51.2	0	0	1593383.344	N/A	0.00
P14199	ref(2)P	0.013	1.787	1.66945	1	1	1	599	65.3	0	0	2750251.234	N/A	0.00

P54352	eas	0.026	1.078	4.05405	1	1	1	518	59.5	0	0	397316.6797	N/A	0.00
Q8IQB4	Zasp66	0.018	1.172	2.7907	1	1	1	430	47.6	0	0	1719786.188	N/A	0.00
Q9VFQ9	Dip-B	0	3.676	5.31496	1	1	1	508	55.5	0	0	2509038.813	N/A	0.00
Q9VAZ0	CG5003-RA	0	3.43	1.82328	1	1	1	713	80.4	0	0	1064672.531	N/A	0.00
Q9Y0Z0	mmy	0.013	1.939	2.11538	1	1	1	520	58.2	0	0	580262.1797	N/A	0.00
Q9V431	Aac11	0.013	2.249	2.05224	1	1	1	536	59.9	0	0	470999.4219	N/A	0.00
P21187	pAbp	0	3.367	2.68139	1	2	1	634	69.9	0	0	0	N/A	N/A
A0A0B4K6	alpha-Est10	0.013	1.706	3.61446	1	1	1	581	65.7	0	0	3984580.344	N/A	0.00
Q27597	Cpr	0.013	2.365	2.06186	1	1	1	679	76.3	0	0	1298412.688	N/A	0.00
Q9W261	Rtf1	0.013	2.645	2.19355	1	1	1	775	87.5	0	0	0	N/A	N/A
Q9W410	CG3815	0	2.892	2.3918	1	1	1	878	98.3	0	0	832702.0625	N/A	0.00
Q9VF51	AOX3	0	5.139	1.51515	1	1	1	1254	137.8	0	0	2376128.125	N/A	0.00
E1JGY6	l(2)01289	0.029	1.022	0.50392	1	1	1	1786	204.6	0	0	1647809.492	N/A	0.00
P06742	Mlc1	0	3.224	8.3871	1	3	1	155	17.5	4793547.188	8619149.063	5001028.234	1.80	1.72
Q9VSF9	CG13674	0	4.201	22.6277	1	5	1	137	13.3	7464044.195	8570083.531	1965625.75	1.15	4.36
P91941	Acp65Aa	0	6.668	24.7619	1	4	1	105	11.3	1363856.719	7148113.367	7958431.5	5.24	0.90
A0A0B4LC	Mlp60A	0	3.721	23.913	1	4	1	92	10	6472322.672	6738209.438	7249928.313	1.04	0.93
D1Z3A1	RpL28	0	4.461	8.33333	1	2	1	144	16	4653205.945	5607568.875	0	1.21	N/A
Q9VDH8	RpS30	0.013	1.659	8.33333	1	2	1	132	14.6	5934482.391	5247327.219	0	0.88	N/A
D1YSG8	CaMKII	0.013	2.57	2.44821	1	2	1	531	60	2379033.125	3348804.328	0	1.41	N/A
Q9V4B8	CG10323	0.013	2.144	1.09051	1	4	1	917	102	2780595.605	3311330.148	2604238.641	1.19	1.27
Q9VE04	mRpL55	0	4.061	15.8879	1	3	1	107	12.6	2908783.809	2828717.688	3155860.578	0.97	0.90
Q9VXN2	sun	0	4.996	24.5902	1	3	1	61	6.8	2777717.75	2714757.641	1844447.125	0.98	1.47
Q9VB15	tau	0.016	1.33	1.67364	1	2	1	717	77.9	0	2690846.156	2164158.875	N/A	1.24
Q9VEF5	CG7379	0	4.059	2.07852	1	5	1	433	45.7	2253603.188	2623673.953	2224414.305	1.16	1.18
A0A0B4LH	ATPsynD	0	3.098	7.30337	1	3	1	178	20.2	2672359.719	2615169.414	2297364	0.98	1.14
A1ZBW0	HnRNP-K	0	3.257	2.78884	1	1	1	502	51	0	2494804.938	0	N/A	N/A
Q9W1N3	levy	0	4.063	15.5963	1	3	1	109	12.5	0	2479924.109	2322013.023	N/A	1.07
A8JQV3	CG17816	0	3.97	2.26757	1	3	1	882	96.3	0	2173201.625	2775926.516	N/A	0.78
M9NDM4	wds	0	2.934	3.87812	1	1	1	361	39	0	2151409.906	0	N/A	N/A
Q8IP51	wb	0.031	1.004	0.41481	1	1	1	3375	374.5	0	2069401.148	0	N/A	N/A
O16797	RpL3	0	4.37	4.80769	1	3	1	416	46.9	0	2028442.625	1942195.594	N/A	1.04
Q9W164	Crtp	0.016	1.305	2.01342	1	1	1	745	86.1	0	1931744.141	0	N/A	N/A
A0A0C4DH	RpS29	0.018	1.163	23.2143	1	1	1	56	6.6	0	1928251.656	0	N/A	N/A
Q9W2X6	ATPsyndelta	0	6.943	9.55414	1	2	1	157	16.7	0	1697829.797	1456139.375	N/A	1.17
Q9VE73	CG12333	0.014	1.478	3.6961	1	2	1	487	53	0	1622983.188	0	N/A	N/A
P19334	trp	0.014	1.582	1.2549	1	1	1	1275	142.5	0	1620202.344	0	N/A	N/A
B5RIV0	bgm	0.013	1.962	3.003	1	1	1	666	73.6	0	1536955.828	0	N/A	N/A
Q95U38	skap	0	3.679	2.7833	1	3	1	503	54.9	2406199.504	1506430.563	1640346.023	0.63	0.92
Q9VGP7	mRpL40	0	3.214	4.59184	1	3	1	196	22.5	2300804.25	1501342.313	1301013.875	0.65	1.15
Q9VK69	CCT4	0.013	2.798	5.4409	1	3	1	533	57.1	4189494.852	1438966.313	0	0.34	N/A
Q9VWG3	RpS10b	0.013	1.892	8.125	1	2	1	160	17.9	1649193.719	1415037.5	0	0.86	N/A
Q9VV42	Pdh	0	4.01	5.39568	1	1	1	278	30.1	0	1349227.375	0	N/A	N/A
A0A0B4K7	mod	0.013	2.415	2.39852	1	2	1	542	60.3	0	1304791.664	704215.3711	N/A	1.85
Q9VCI3	Lsd-1	0	4.365	3.7123	1	2	1	431	48.8	1166754.609	1273400.781	0	1.09	N/A

P22058	D1	0.013	2.088	4.50704	1	2	1	355	37	0	1268329.828	1601835.223	N/A	0.79
P27398	sol	0.014	1.529	1.12923	1	1	1	1594	174.2	0	1212529.438	0	N/A	N/A
Q9W1G7	Nap1	0	4.267	4.86486	1	2	1	370	42.7	0	1175019.172	1527000.992	N/A	0.77
Q9W334	RpS28b	0.013	2.657	30.7692	1	1	1	65	7.5	0	1136351.359	0	N/A	N/A
Q9VE08	CG6013	0	3.321	6.10329	1	2	1	213	24.8	0	1125233.484	694050.2734	N/A	1.62
M9PF16	beta-Spec	0	4.587	0.82322	1	1	1	2308	267.5	0	1116245.973	0	N/A	N/A
Q9VV95	CG13026	0.013	1.835	11.0169	1	1	1	118	13.1	0	1104162.426	0	N/A	N/A
Q9VGF7	GC1	0	3.032	4.04984	1	2	1	321	34.5	1507563.633	1088181.984	0	0.72	N/A
Q9W4V3	CG14419	0.013	2.719	5.64103	1	1	1	195	21	0	1031873.813	0	N/A	N/A
A0A0B4KH	Syp	0.013	1.7	1.31406	1	1	1	761	83	0	1030648.781	0	N/A	N/A
O61491	Flo1	0	4.314	3.05164	1	2	1	426	47.1	0	1025742.336	1009637.234	N/A	1.02
M9PGH7	G9a	0	3.275	1.4484	1	2	1	1657	183.4	880593.6094	1023803.938	0	1.16	N/A
O97183	RpII33	0.013	2.157	5.09091	1	2	1	275	31.2	0	1007158.891	1463556.875	N/A	0.69
A0A0B4K6	aralar1	0.018	1.178	1.69731	1	1	1	707	78.3	0	938812.3164	0	N/A	N/A
Q9VZZ6	CG16985	0	3.04	9.39597	1	2	1	149	16.1	0	901808.6406	2668105.156	N/A	0.34
X2JEE5	CG2157	0	5.257	6.07287	1	1	1	247	24.2	0	885223.0313	0	N/A	N/A
Q0E9B6	RpS11	0.013	2.284	5.16129	1	3	1	155	18.1	887384.6563	876053.1992	631292.2031	0.99	1.39
Q9VMS1	cype	0.013	2.372	18.1818	1	2	1	77	8.3	662177.3047	843698.0391	0	1.27	N/A
A0A0B4KH	Tm2	0	3.326	4.92958	1	1	1	284	32.8	0	836704.6797	0	N/A	N/A
Q9VU46	CG11279	0.013	2.344	9.80392	1	3	1	102	11.1	1102136.715	805028.0352	0	0.73	N/A
P29327	RpS6	0.013	1.755	6.45161	1	3	1	248	28.4	888191.9063	804354.5156	1060000.406	0.91	0.76
Q8IPX7	Rrp40	0.013	2.019	4.31034	1	2	1	232	25	970316.3438	764314.457	0	0.79	N/A
Q9VZU0	CG12032	0.018	1.198	8.10811	1	3	1	111	12.1	1313739.914	761855.5938	658440.8555	0.58	1.16
Q9V405	Rpt3	0.013	2.192	3.38983	1	2	1	413	47	0	754169.457	771334.2539	N/A	0.98
O61231	RpL10	0.013	2.119	4.12844	1	2	1	218	25.5	0	713320.0938	0	N/A	N/A
Q9VP57	pzg	0.013	1.756	1.60643	1	1	1	996	105	0	685775.3711	0	N/A	N/A
Q9VM97	Tsp	0.013	2.321	0.84906	1	3	1	1060	118.2	1252495.555	612714.0957	1589860.938	0.49	0.39
Q9VD58	CG6439	0	3.123	3.51351	1	2	1	370	40.4	573764.6406	590435.3828	0	1.03	N/A
Q9VEJ3	P5cr-2	0	3.729	4.7619	1	2	1	273	28.1	806691.5781	519889.5938	0	0.64	N/A
Q9VGQ1	CG5214	0.013	2.001	2.99145	1	3	1	468	49.9	1115684.039	509621.7266	1173136.697	0.46	0.43
P06002	ninaE	0	5.934	4.55764	1	3	1	373	41.5	644597.5879	498174.8281	622486.7422	0.77	0.80
Q7KVX1	l(1)G0334	0.014	1.545	2.0316	1	3	1	443	48.8	416363.9453	386394.418	416600.0156	0.93	0.93
Q9XY35	ox	0.013	2.077	30.9091	1	1	1	55	6.3	0	333814.0625	0	N/A	N/A
Q9W327	LysRS	0.016	1.297	2.14168	1	1	1	607	68.5	0	307615.6563	0	N/A	N/A
Q9XYW6	STUB1	0.013	2.607	3.11419	1	3	1	289	33.8	371029.2891	305149.2656	765233.125	0.82	0.40
P32234	128up	0.013	2.396	4.07609	1	3	1	368	41.1	0	274242	669593.3672	N/A	0.41
A0A0B4KF	jbug	0.02	1.148	0.36789	1	1	1	2990	322.6	0	242410.0469	0	N/A	N/A

Supplementary Table 10 Primers used in this study

Primer name	Sequence of primers (5'→3')
Kay mRNA F	TGTTCAACATGCCGCTGTCG
Kay mRNA R	CCAGTGTGTCCTCGATGTTGC
Myo31DF mRNA F	TGGTGGGCATTGTGTGCAAG
Myo31DF mRNA R	ATAATGCGTGCCTTGCCGTC
sr mRNA F	CGCCAAGGTGCATCTGAAGC
sr mRNA R	TGCTGCTGCTGCTGTTGTTG
h mRNA F	CAAGCCATCGGTCATCCAGC
h mRNA R	TGCTCCTCCTCCTTGATCTGC
grh mRNA F	ATGAGACTGACTCGCCGGAC
grh mRNA R	GGTTGTGGAACTTGAGCGTCG

Supplementary Table 2 Peptides identified in the coimmunoprecipitates with Rpd3
The peptide amount in the control (untreated) or the TRPA1-activated samples were calculated using Skyline.
The relative amount of each peptide in the TRPA1-activated samples was determined by dividing the peptide amount in the TRPA1-activated samples by that in the untreated samples, which value was further normalized by the amount of Rpd3 determined using Proteome discoverer 2.1. (untreated:TRPA1-activated=1:0.77).

Peptide Sequence	Gene	Protein names	Precursor Mz	Precursor Charge	Retention Time (untreated)	Amount (untreated)	Retention Time (TRPA1-activated)	Amount (TRPA1-activated)	Relative amount	Number of amino acid in CoRest-F (824 a.a)
ALLVWSPTK	CoRest	CoRest, isoform G (CoRest, isoform H) (CoRest, isoform I) (IP20671p)	507.803	2	37.31	2318735	37.1	1637547	0.91741	120-128 a.a., exon 3
VLFEQAFQFHGK	CoRest	CoRest, isoform G (CoRest, isoform H) (CoRest, isoform I) (IP20671p)	484.254	3	38.03	957244	37.88	274801	0.37292	187-198 a.a., exon 4
SIASLVK	CoRest	CoRest, isoform G (CoRest, isoform H) (CoRest, isoform I) (IP20671p)	359.229	2	23.99	840931	23.99	310644	0.47987	211-217 a.a., exon 5
RPISGPEGNAPR	CoRest	CoRest, isoform G (CoRest, isoform H) (CoRest, isoform I) (IP20671p)	417.558	3	11.45	316853	11.49	105613	0.43299	433-444 a.a., exon 7
TQTSSEELSAQK	CoRest	CoRest, isoform G (CoRest, isoform H) (CoRest, isoform I) (IP20671p)	654.818	2	11.1	1476303	11.23	860048	0.75678	754-765 a.a., exon 9
DSSVLPVAEQPPAK	CoRest	CoRest, isoform G (CoRest, isoform H) (CoRest, isoform I) (IP20671p)	719.383	2	26.69	3357320	26.66	3402373	1.31647	793-806 a.a., exon 9-10
IALSTGGGSSVAEFLAN	CoRest	CoRest, isoform G (CoRest, isoform H) (CoRest, isoform I) (IP20671p)	797.41	2	44.77	1725679	44.51	2728085	2.05362	808-824 a.a., exon 10
TYSSRPVTTSYAVTR	CG45078	Uncharacterized protein CG45078	563.625	3	19.03	263179	19.02	412016	2.03369	
VPFVPRPSLISDPVTAFGVR	CG45078	Uncharacterized protein CG45078	718.741	3	48.47	199755	48.27	553533	3.59972	
LDLCTDRPGSHR	fau	Protein anoxia up-regulated	476.233	3	15.84	528306	15.79	874608	2.15055	
SGPGGSYNYSTER	fau	Protein anoxia up-regulated	687.8	2	14.6	687443	14.6	1415134	2.67413	
SSYDSSNPHSYRPER	fau	Protein anoxia up-regulated	594.599	3	11.24	242334	11.32	514451	2.75773	
TYSSRPVTTSYAVTTPR	fau	Protein anoxia up-regulated	629.658	3	21.82	429567	21.83	928750	2.8086	
IEDVTPIPSDSTR	RpS14a	40S ribosomal protein S14a	715.362	2	24.62	3641721	24.6	2484539	0.88626	
TLGITALHIK	RpS14a	40S ribosomal protein S14a	533.835	2	32.9	2242098	32.75	2243891	1.30008	
TLGITALHIK	RpS14a	40S ribosomal protein S14a	356.225	3	32.86	2809070	32.75	2994782	1.38492	
IEDVTPIPSDSTR	RpS14b	40S ribosomal protein S14b	715.362	2	24.62	3641721	24.6	2484539	0.88626	
TLGITALHIK	RpS14b	40S ribosomal protein S14b	533.835	2	32.9	2242098	32.75	2243891	1.30008	
TLGITALHIK	RpS14b	40S ribosomal protein S14b	356.225	3	32.86	2809070	32.75	2994782	1.38492	
ELIEIISGAAALT	ATPsynga	ATP synthase subunit gamma, mitochondrial (F-ATPase gamma subunit)	650.872	2	52.67	6635864	52.44	7721958	1.51165	
GELAQDEANTK	ATPsynga	ATP synthase subunit gamma, mitochondrial (F-ATPase gamma subunit)	588.281	2	10.74	294246	10.81	49515	0.2186	
GLCGAVHTGVAR	ATPsynga	ATP synthase subunit gamma, mitochondrial (F-ATPase gamma subunit)	599.311	2	16.19	595810	16.17	374856	0.8173	
GLCGAVHTGVAR	ATPsynga	ATP synthase subunit gamma, mitochondrial (F-ATPase gamma subunit)	399.877	3	16.19	1960498	16.14	986502	0.65366	
IANEVLQTGYDYTEGK	ATPsynga	ATP synthase subunit gamma, mitochondrial (F-ATPase gamma subunit)	900.936	2	31.83	2132738	31.71	1347957	0.82103	
LAVYDSLDSDVVK	ATPsynga	ATP synthase subunit gamma, mitochondrial (F-ATPase gamma subunit)	712.369	2	34.68	2559894	34.55	2322704	1.17868	
LLIAVTSDR	ATPsynga	ATP synthase subunit gamma, mitochondrial (F-ATPase gamma subunit)	494.295	2	29.56	1159992	29.57	707980	0.79285	
SVVSYQCSTLPIFSGSTVEK	ATPsynga	ATP synthase subunit gamma, mitochondrial (F-ATPase gamma subunit)	1095.04	2	42.41	556083	42.14	284362	0.66428	
FFHLIYEEEFAR	eIF3i	Eukaryotic translation initiation factor 3 subunit I (eIF3i) (Eukaryotic translatior	534.265	3	42.14	169782	42.06	535132	4.09441	
SYASGGEDGFVR	eIF3i	Eukaryotic translation initiation factor 3 subunit I (eIF3i) (Eukaryotic translatior	622.781	2	20.66	314052	20.64	486210	2.01115	
AAVEEGIVPGGGTALLR	Hsp60A	Heat shock protein 60A	805.449	2	37.57	3201747	37.41	3272869	1.3279	
FGPEVR	Hsp60A	Heat shock protein 60A	352.69	2	17.41	1545828	17.45	1317497	1.10716	
KPLVIIAEDIDGEALSTLVVNR	Hsp60A	Heat shock protein 60A	789.116	3	52.44	2091770	52.29	1657875	1.02958	
LVQDVANNTNEEAGDGTTTATVLAI	Hsp60A	Heat shock protein 60A	854.088	3	28.53	1313197	28.39	1332802	1.31843	
VEFQDALLLLSEK	Hsp60A	Heat shock protein 60A	752.917	2	52.37	697568	52.05	393900	0.73354	
VHDALNATR	Hsp60A	Heat shock protein 60A	498.765	2	10.63	556580	10.7	377032	0.87998	
DVLSVAFSADNR	Rack1	Guanine nucleotide-binding protein subunit beta-like protein (Receptor of activ	647.325	2	37.28	534801	37.1	522154	1.26832	
GAFGKPQGTVAR	RpL10	60S ribosomal protein L10 (QM protein homolog) (dQM)	396.888	3	13.7	378695	13.67	452705	1.55292	
IFDLGR	RpL10	60S ribosomal protein L10 (QM protein homolog) (dQM)	360.706	2	28.76	454688	28.68	577700	1.65048	
VAAEVAAPLSQAK	Flo1	Flotillin-1	627.856	2	23.88	83835	23.84	638161	9.88843	
IAAEVAAPLAK	Flo2	Flotillin-2	527.319	2	25.68	101116	25.6	592504	7.61191	
ASAIVVITTSGK	PyK	Pyruvate kinase (PK) (EC 2.7.1.40)	573.84	2	28.18	1401434	28.12	1081412	1.0024	
GAGTIDASHAAAIAAVEAATK	PyK	Pyruvate kinase (PK) (EC 2.7.1.40)	632.666	3	35.16	3178366	35.03	2437479	0.99623	
GDLGIEIPAEK	PyK	Pyruvate kinase (PK) (EC 2.7.1.40)	571.309	2	32.68	1575394	32.56	1255028	1.03487	
LQFDSPVPHVR	PyK	Pyruvate kinase (PK) (EC 2.7.1.40)	647.849	2	29.48	769777	29.09	16679	0.02815	

LQFDSPVPHVR	PyK	Pyruvate kinase (PK) (EC 2.7.1.40)	432.235	3	29.48	2084871	29.25	297903	0.18562
QGSGFTNTIR	PyK	Pyruvate kinase (PK) (EC 2.7.1.40)	540.775	2	18.98	564710	18.97	489131	1.12518
TGLIGGSGTAEIELK	PyK	Pyruvate kinase (PK) (EC 2.7.1.40)	723.396	2	34.9	2549848	34.74	1867825	0.95158
VFLAQK	PyK	Pyruvate kinase (PK) (EC 2.7.1.40)	353.218	2	18.01	683366	18.06	383744	0.72948
VFVDDGLISLIVR	PyK	Pyruvate kinase (PK) (EC 2.7.1.40)	723.422	2	56.88	4755565	56.58	4688422	1.2807
DAVITVPAYFNDSQR	Hsp68	Heat shock protein 68	848.42	2	39.29	42651	39	450593	13.7239
DNNVLGTFDLTGVPPAPR	Hsp68	Heat shock protein 68	941.987	2	45.67	0	45.84	849068	#DIV/0!
LSQADIDR	Hsp68	Heat shock protein 68	459.238	2	14.58	83445	14.79	271197	4.22189
NQLETYLFGVK	Hsp68	Heat shock protein 68	656.351	2	43.03	758445	42.8	2051711	3.5141
NTLEPVEK	Hsp68	Heat shock protein 68	465.251	2	14.95	24751	15.03	257980	13.5399
STAGDTHLGGEDFDNR	Hsp68	Heat shock protein 68	846.366	2	18.67	1657789	18.65	1831600	1.43524
STAGDTHLGGEDFDNR	Hsp68	Heat shock protein 68	564.58	3	18.67	10781481	18.68	14815577	1.7851
TFTTYADNQPAVTIQVFEGER	Hsp68	Heat shock protein 68	1194.08	2	43.53	110485	43.36	428462	5.03769
TFTTYADNQPAVTIQVFEGER	Hsp68	Heat shock protein 68	796.389	3	43.6	153324	43.44	1179773	9.99564
TLSSSTEASLEIDALYEGHDFYSK	Hsp68	Heat shock protein 68	888.417	3	43.54	56614	43.43	736672	16.9033
TTPSYVAFTDSER	Hsp68	Heat shock protein 68	737.346	2	28.48	159128	28.32	60403172	493.101
TTPSYVAFTDSER	Hsp68	Heat shock protein 68	491.9	3	28.44	0	28.4	323797	#DIV/0!
VEIIANDQGNR	Hsp68	Heat shock protein 68	614.818	2	18.24	20421124	18.27	30231408	1.9231
VQNLLQNFFGGK	Hsp68	Heat shock protein 68	682.87	2	49.08	23828	49.01	1232989	67.2193
ADNSAPAAQDDGSGAPVVR	Mi-2	Chromodomain-helicase-DNA-binding protein Mi-2 homolog (EC 3.6.4.12) (AT	899.422	2	16.91	470838	16.88	285548	0.78783
AVAAATSAATGATGK	Mi-2	Chromodomain-helicase-DNA-binding protein Mi-2 homolog (EC 3.6.4.12) (AT	624.333	2	13.11	1665996	13.11	983701	0.76703
HHYEQHQEDVGR	Mi-2	Chromodomain-helicase-DNA-binding protein Mi-2 homolog (EC 3.6.4.12) (AT	512.231	3	7.62	201593	7.78	286609	1.84687
HHYEQHQEDVGR	Mi-2	Chromodomain-helicase-DNA-binding protein Mi-2 homolog (EC 3.6.4.12) (AT	384.425	4	7.62	91019	7.78	138645	1.97877
IDGGITGTLR	Mi-2	Chromodomain-helicase-DNA-binding protein Mi-2 homolog (EC 3.6.4.12) (AT	501.783	2	24.03	1258518	24.04	897889	0.9268
QAIDYYQDLR	Mi-2	Chromodomain-helicase-DNA-binding protein Mi-2 homolog (EC 3.6.4.12) (AT	642.815	2	28.79	539792	28.68	906085	2.18054
SVTGDEPEEPR	Mi-2	Chromodomain-helicase-DNA-binding protein Mi-2 homolog (EC 3.6.4.12) (AT	608.278	2	13.23	438146	13.27	556388	1.64961
VGGNIEVLGFNAR	Mi-2	Chromodomain-helicase-DNA-binding protein Mi-2 homolog (EC 3.6.4.12) (AT	673.365	2	37.32	2342650	37.14	3356129	1.86103
LAPITGVTAYTVNPGITR	Adh	Alcohol dehydrogenase (EC 1.1.1.1)	922.517	2	39.24	126429	39	732773	7.52913
LAPITGVTAYTVNPGITR	Adh	Alcohol dehydrogenase (EC 1.1.1.1)	615.347	3	39.28	26716	39.04	408326	19.8545
NVIFVAGLGGIGLDTSK	Adh	Alcohol dehydrogenase (EC 1.1.1.1)	830.967	2	49.12	84006	48.78	717390	11.0935
VTVTFYPYDVTVPIAETTK	Adh	Alcohol dehydrogenase (EC 1.1.1.1)	1072.56	2	48.67	129647	48.42	771909	7.73439
AMSIMNSFVNDIFER	His2B;	Histone H2B	887.419	2	56.68	8868951	56.46	6505927	0.95293
AMSIMNSFVNDIFER	His2B;	Histone H2B	895.416	2	53.52	1479536	53.25	1206039	1.05891
EIQTAVR	His2B;	Histone H2B	408.732	2	12.88	14773905	12.91	10649531	0.93639
LLLPGELAK	His2B;	Histone H2B	477.305	2	35.91	102778656	35.74	124503536	1.57362
QVHPDTGISSK	His2B;	Histone H2B	584.801	2	10.81	2334514	10.96	693190	0.38573
QVHPDTGISSK	His2B;	Histone H2B	390.203	3	10.85	5805182	10.92	2011360	0.45009
STELLIR	His3;	Histone H3	416.25	2	25.89	5173880	25.9	6235095	1.56549
YRPGTVALR	His3;	Histone H3	516.801	2	17.48	3862821	17.48	3541424	1.19096
LSSPFHAFFHEPPVWSVALPR	Hsp22	Heat shock protein 22	807.755	3	53.04	54619	51.82	2099841	49.9419
LSSPFHAFFHEPPVWSVALPR	Hsp22	Heat shock protein 22	606.068	4	53	0	51.82	4645716	#DIV/0!
NWQQIAR	Hsp22	Heat shock protein 22	458.243	2	20.71	432610	20.82	4241642	12.7368
SEQQEAEQGGYSSR	Hsp22	Heat shock protein 22	778.335	2	9.79	28800	9.82	242324	10.9302
SLPMFWR	Hsp22	Heat shock protein 22	468.742	2	39.16	14794	42.1	979169	85.9794
VLDESVVLVEGK	Hsp22	Heat shock protein 22	643.864	2	32.87	228435	32.75	2015223	11.46
WQEQEFAPPATVNK	Hsp22	Heat shock protein 22	822.904	2	29.6	0	29.57	700123	#DIV/0!
DGFQVCMDVSHFKPSELVVK	Hsp23	Heat shock protein 23	774.713	3	43.1	204592	43.01	339238	2.15396
DGFQVCMDVSHFKPSELVVK	Hsp23	Heat shock protein 23	581.287	4	43.1	402779	42.97	317497	1.02399
DGFQVCMDVSHFKPSELVVK	Hsp23	Heat shock protein 23	780.045	3	38.76	29062	38.74	599164	26.782
DGFQVCMDVSHFKPSELVVK	Hsp23	Heat shock protein 23	585.286	4	38.73	18808	38.78	469777	32.4468
IVQIQQVGPAHLNVK	Hsp23	Heat shock protein 23	822.483	2	30.45	27968	30.12	3476212	161.461
IVQIQQVGPAHLNVK	Hsp23	Heat shock protein 23	548.658	3	30.33	1113	30.08	27440434	32027.1
MSMVPFYEPYYCQR	Hsp23	Heat shock protein 23	935.902	2	43.25	0	43.16	737059	#DIV/0!

MSMVPFYEPYYCQR	Hsp23	Heat shock protein 23	943.899	2	39.58	18216	39.45	774858	55.2575
MSMVPFYEPYYCQR	Hsp23	Heat shock protein 23	943.899	2	37.24	37268	37.1	726973	25.3399
MSMVPFYEPYYCQR	Hsp23	Heat shock protein 23	951.897	2	34.82	0	34.71	818198	#DIV/0!
NPYLALVGPMEQQLR	Hsp23	Heat shock protein 23	864.959	2	46.34	474657	45.99	3156756	8.6394
NPYLALVGPMEQQLR	Hsp23	Heat shock protein 23	576.975	3	46.1	37103	45.99	888801	31.1184
NPYLALVGPMEQQLR	Hsp23	Heat shock protein 23	872.956	2	38.21	28503	37.96	3708824	169.032
NPYLALVGPMEQQLR	Hsp23	Heat shock protein 23	582.307	3	38.46	368115	37.96	1423477	5.0233
QVGASSGSSGAVSK	Hsp23	Heat shock protein 23	611.307	2	13.12	130999	8.97	467450	4.63542
VASTLSSDGVLTIK	Hsp23	Heat shock protein 23	695.893	2	33.08	0	33.09	2054661	#DIV/0!
VQDNSVLVEGNHEER	Hsp23	Heat shock protein 23	575.611	3	16.47	0	16.8	50272	#DIV/0!
AEQVVSQLSSDGVLTVSIPKPQAVE	Hsp26	Heat shock protein 26	942.169	3	41.6	19465	41.36	1034803	69.0598
DGFQVCMDVAQFKPSELNVK	Hsp26	Heat shock protein 26	771.373	3	44.11	213799	44.1	1638807	9.95737
DGFQVCMDVAQFKPSELNVK	Hsp26	Heat shock protein 26	776.705	3	39.94	53212	39.69	2583608	63.0724
IIQIQQVGPAHLNVK	Hsp26	Heat shock protein 26	829.491	2	32.72	98730	32.53	9251943	121.732
IIQIQQVGPAHLNVK	Hsp26	Heat shock protein 26	553.33	3	32.72	645047	32.53	78215392	157.515
MSLSTLLSLVDELQEPR	Hsp26	Heat shock protein 26	644.343	3	65.46	0	65.23	11197	#DIV/0!
NDIHWPATAHVGK	Hsp26	Heat shock protein 26	482.581	3	20.47	18499	23.18	478607	33.6088
SPIYELGLGLHPHSR	Hsp26	Heat shock protein 26	838.449	2	33.39	144972	33.4	4457974	39.9462
SPIYELGLGLHPHSR	Hsp26	Heat shock protein 26	559.302	3	33.64	373200	33.4	49626936	172.742
SPIYELGLGLHPHSR	Hsp26	Heat shock protein 26	419.728	4	33.54	257619	33.4	45925152	231.577
VVDDSILVEGK	Hsp26	Heat shock protein 26	587.322	2	27.24	288145	27.53	4657387	20.9968
YVLPLGTQQR	Hsp26	Heat shock protein 26	587.833	2	30.91	472461	30.72	95333624	262.121
ASGGPNALLPAVGK	Hsp27	Heat shock protein 27	626.356	2	31.22	28381	31.28	8197708	375.221
DGFQVCMDVSQFKPNELTVK	Hsp27	Heat shock protein 27	781.377	3	43.41	309810	43.2	1758866	7.37496
DGFQVCMDVSQFKPNELTVK	Hsp27	Heat shock protein 27	786.708	3	39.05	29103	38.58	4310963	192.424
ELDHDYR	Hsp27	Heat shock protein 27	474.215	2	10.24	25905	10.22	309944	15.5425
GFDPNEVVSTVSSDGVLTLK	Hsp27	Heat shock protein 27	1032.53	2	49.31	578434	48.23	34145948	76.6845
GFDPNEVVSTVSSDGVLTLK	Hsp27	Heat shock protein 27	688.688	3	49.28	243916	48.23	17596958	93.7173
IVQIQQTGPAHLSVK	Hsp27	Heat shock protein 27	809.967	2	26.16	0	26.15	7420055	#DIV/0!
IVQIQQTGPAHLSVK	Hsp27	Heat shock protein 27	540.314	3	26.32	125560	26.23	62417060	645.765
LLLPNTLGLGR	Hsp27	Heat shock protein 27	583.866	2	42.95	0	42.98	26755810	#DIV/0!
MSIIPLLHLAR	Hsp27	Heat shock protein 27	653.389	2	60.18	0	59.42	2492957	#DIV/0!
VVDNTVVVEGK	Hsp27	Heat shock protein 27	579.822	2	18.32	82265	18.27	3377383	53.332
AGFAGDDAPR	Act42A	Actin-42A	488.728	2	15.06	5346544	15.03	6072874	1.47551
AVFPSIVGRPR	Act42A	Actin-42A	599.856	2	30.18	3527039	29.97	6233553	2.29587
DSYVGDEAQSK	Act42A	Actin-42A	599.765	2	11.94	533801	12.02	513658	1.25002
IWHHTFYNELR	Act42A	Actin-42A	505.921	3	25.48	1669489	25.49	2792816	2.17311
SYELPDGQVITIGNER	Act42A	Actin-42A	895.95	2	39.17	5791673	39	8517636	1.91046
SYELPDGQVITIGNER	Act42A	Actin-42A	597.635	3	39.17	427938	38.97	800279	2.42931
TTGIVLDSGDGVSHTVPIYEGYALPI	Act42A	Actin-42A	1051.22	3	45.78	1749429	45.58	1016220	0.75459
VAPEEHPVLLTEAPLNPK	Act42A	Actin-42A	977.536	2	34.25	620752	34.11	748708	1.56681
VAPEEHPVLLTEAPLNPK	Act42A	Actin-42A	652.026	3	34.21	12031327	34.11	16999678	1.83548
AGFAGDDAPR	Act79B	Actin, larval muscle (Actin-79B)	488.728	2	15.06	5346544	15.03	6072874	1.47551
AVFPSIVGRPR	Act79B	Actin, larval muscle (Actin-79B)	599.856	2	30.18	3527039	29.97	6233553	2.29587
SYELPDGQVITIGNER	Act79B	Actin, larval muscle (Actin-79B)	895.95	2	39.17	5791673	39	8517636	1.91046
SYELPDGQVITIGNER	Act79B	Actin, larval muscle (Actin-79B)	597.635	3	39.17	427938	38.97	800279	2.42931
TTGIVLDSGDGVSHTVPIYEGYALPI	Act79B	Actin, larval muscle (Actin-79B)	1051.22	3	45.78	1749429	45.58	1016220	0.75459
VAPEEHPVLLTEAPLNPK	Act79B	Actin, larval muscle (Actin-79B)	977.536	2	34.25	620752	34.11	748708	1.56681
VAPEEHPVLLTEAPLNPK	Act79B	Actin, larval muscle (Actin-79B)	652.026	3	34.21	12031327	34.11	16999678	1.83548
DAGHIAGLNVLR	Hsp70Ab	Major heat shock 70 kDa protein Ab (Heat shock protein 70Ab) (HSP70-87A7	618.346	2	31.67	40614	31.54	13040718	417.108
DAGHIAGLNVLR	Hsp70Ab	Major heat shock 70 kDa protein Ab (Heat shock protein 70Ab) (HSP70-87A7	412.567	3	31.59	26572	31.54	33980956	1661.24
DNNALGTFDLSGIPPAPR	Hsp70Ab	Major heat shock 70 kDa protein Ab (Heat shock protein 70Ab) (HSP70-87A7	927.971	2	45.07	166587	43.39	30976200	241.551
DNNALGTFDLSGIPPAPR	Hsp70Ab	Major heat shock 70 kDa protein Ab (Heat shock protein 70Ab) (HSP70-87A7	618.983	3	45.21	24051	43.65	6711729	362.513

ETAEAYLGESITDAVITVPAYFNDSQ	Hsp70Ab	Major heat shock 70 kDa protein Ab (Heat shock protein 70Ab) (HSP70-87A7	987.477	3	58.6	304104	58.36	1308713	5.59042	
FAPEEISSMVLTK	Hsp70Ab	Major heat shock 70 kDa protein Ab (Heat shock protein 70Ab) (HSP70-87A7	726.376	2	41.77	22231	41.79	11132469	650.511	
FAPEEISSMVLTK	Hsp70Ab	Major heat shock 70 kDa protein Ab (Heat shock protein 70Ab) (HSP70-87A7	484.587	3	41.77	0	41.79	276280	#DIV/0!	
FAPEEISSMVLTK	Hsp70Ab	Major heat shock 70 kDa protein Ab (Heat shock protein 70Ab) (HSP70-87A7	734.374	2	35.12	297494	34.86	2047640	8.94124	
FEELCADLFR	Hsp70Ab	Major heat shock 70 kDa protein Ab (Heat shock protein 70Ab) (HSP70-87A7	650.306	2	41.55	32123	41.36	4667637	188.757	
GQIHDIVLVGGSTR	Hsp70Ab	Major heat shock 70 kDa protein Ab (Heat shock protein 70Ab) (HSP70-87A7	726.402	2	27.66	141880	27.58	2013950	18.4395	
GQIHDIVLVGGSTR	Hsp70Ab	Major heat shock 70 kDa protein Ab (Heat shock protein 70Ab) (HSP70-87A7	484.604	3	27.43	42940	27.54	4848927	146.692	
HCSPIMTK	Hsp70Ab	Major heat shock 70 kDa protein Ab (Heat shock protein 70Ab) (HSP70-87A7	487.233	2	11.2	35165	11.25	2786296	102.929	
HCSPIMTK	Hsp70Ab	Major heat shock 70 kDa protein Ab (Heat shock protein 70Ab) (HSP70-87A7	495.231	2	9.26	11777	9.24	218205	24.0687	
IQDVLLVDVAPLSLGIETAGGVMTK	Hsp70Ab	Major heat shock 70 kDa protein Ab (Heat shock protein 70Ab) (HSP70-87A7	1270.21	2	61.32	0	61.17	571901	#DIV/0!	
IQDVLLVDVAPLSLGIETAGGVMTK	Hsp70Ab	Major heat shock 70 kDa protein Ab (Heat shock protein 70Ab) (HSP70-87A7	847.14	3	61.32	519	61.21	3335041	8347.49	
IQDVLLVDVAPLSLGIETAGGVMTK	Hsp70Ab	Major heat shock 70 kDa protein Ab (Heat shock protein 70Ab) (HSP70-87A7	852.472	3	57.66	0	57.38	359486	#DIV/0!	
LSQAEIDR	Hsp70Ab	Major heat shock 70 kDa protein Ab (Heat shock protein 70Ab) (HSP70-87A7	466.246	2	14.26	24538	14.28	4887219	258.729	
NALESYVFNVK	Hsp70Ab	Major heat shock 70 kDa protein Ab (Heat shock protein 70Ab) (HSP70-87A7	642.335	2	40.6	35451	38.81	48323708	1770.74	
NLNLSINPDEAVAYGAAVQAAILSGI	Hsp70Ab	Major heat shock 70 kDa protein Ab (Heat shock protein 70Ab) (HSP70-87A7	1493.76	2	58.87	44314	57.77	661805	19.4004	
NLNLSINPDEAVAYGAAVQAAILSGI	Hsp70Ab	Major heat shock 70 kDa protein Ab (Heat shock protein 70Ab) (HSP70-87A7	996.175	3	58.34	121646	57.81	3315510	35.4058	
NQVAMNPR	Hsp70Ab	Major heat shock 70 kDa protein Ab (Heat shock protein 70Ab) (HSP70-87A7	465.235	2	11.74	204889	11.84	365105	2.31484	
STAGDTHLGGEDFDNR	Hsp70Ab	Major heat shock 70 kDa protein Ab (Heat shock protein 70Ab) (HSP70-87A7	846.366	2	18.67	1657789	18.65	1831600	1.43524	
STAGDTHLGGEDFDNR	Hsp70Ab	Major heat shock 70 kDa protein Ab (Heat shock protein 70Ab) (HSP70-87A7	564.58	3	18.67	10781481	18.68	14815577	1.7851	
TFSTYADNQPGVSIQVYEGER	Hsp70Ab	Major heat shock 70 kDa protein Ab (Heat shock protein 70Ab) (HSP70-87A7	787.705	3	34.9	43097	34.9	7443709	224.37	
TLSSSTEATIEIDALFEGQDFYTK	Hsp70Ab	Major heat shock 70 kDa protein Ab (Heat shock protein 70Ab) (HSP70-87A7	1333.64	2	55.81	30514	54.36	4199024	178.76	
TLSSSTEATIEIDALFEGQDFYTK	Hsp70Ab	Major heat shock 70 kDa protein Ab (Heat shock protein 70Ab) (HSP70-87A7	889.429	3	55.66	365012	54.36	14991373	53.3527	
TTPSYVAFTDSER	Hsp70Ab	Major heat shock 70 kDa protein Ab (Heat shock protein 70Ab) (HSP70-87A7	737.346	2	28.48	159128	28.32	60403172	493.101	
TTPSYVAFTDSER	Hsp70Ab	Major heat shock 70 kDa protein Ab (Heat shock protein 70Ab) (HSP70-87A7	491.9	3	28.44	0	28.4	323797	#DIV/0!	
VEIIANDQGNR	Hsp70Ab	Major heat shock 70 kDa protein Ab (Heat shock protein 70Ab) (HSP70-87A7	614.818	2	18.24	20421124	18.27	30231408	1.9231	
VQSLLQDFFHGK	Hsp70Ab	Major heat shock 70 kDa protein Ab (Heat shock protein 70Ab) (HSP70-87A7	709.875	2	45.25	30856	45.28	4190765	176.431	
VQSLLQDFFHGK	Hsp70Ab	Major heat shock 70 kDa protein Ab (Heat shock protein 70Ab) (HSP70-87A7	473.586	3	45.21	0	45.24	14899044	#DIV/0!	
ALLFIPR	Hsp83	Heat shock protein 83 (HSP 82)	415.268	2	39.76	1322147	39.6	1321862	1.29876	
FHTSASGDDFCSLADYVSR	Hsp83	Heat shock protein 83 (HSP 82)	712.309	3	37.38	903559	37.17	836957	1.20329	
HFSVEGQLEFR	Hsp83	Heat shock protein 83 (HSP 82)	674.836	2	31.64	477696	31.52	426943	1.16102	
HFSVEGQLEFR	Hsp83	Heat shock protein 83 (HSP 82)	450.226	3	31.72	303941	31.52	380731	1.62724	
IGGIGTVPVGR	eEF1alph	Elongation factor 1-alpha 2 (EF-1-alpha-2)	513.309	2	26.78	8471779	26.71	7294916	1.11858	
IHINIVVIGHVDSGK	eEF1alph	Elongation factor 1-alpha 2 (EF-1-alpha-2)	534.311	3	34.63	1881392	34.42	1040176	0.71821	
LPLQDVYK	eEF1alph	Elongation factor 1-alpha 2 (EF-1-alpha-2)	488.279	2	28.46	1429878	28.37	739398	0.67174	
STTTGHLIYK	eEF1alph	Elongation factor 1-alpha 2 (EF-1-alpha-2)	560.803	2	15.84	2355586	15.79	1523568	0.8402	
ALDSMQASLEAEAK	Mhc	Myosin heavy chain, muscle	732.356	2	29.64	321135	29.49	790332	3.19701	
ALDSMQASLEAEAK	Mhc	Myosin heavy chain, muscle	740.353	2	21.04	48359	20.88	260236	6.99057	
DLEEANIQHESTLANLR	Mhc	Myosin heavy chain, muscle	651.661	3	31.08	667254	31	1249882	2.43333	
GALQDYQER	Mhc	Myosin heavy chain, muscle	540.259	2	15.57	503772	15.55	1324414	3.41517	
IEELEEEVEAER	Mhc	Myosin heavy chain, muscle	737.849	2	27.66	871072	27.57	2436877	3.63414	
LAEAEETIESLNQK	Mhc	Myosin heavy chain, muscle	787.899	2	29.52	307761	29.44	1119309	4.72453	
LAGADIETYLLEK	Mhc	Myosin heavy chain, muscle	718.388	2	42.99	649408	42.76	1212276	2.42497	
LTQEAVADLER	Mhc	Myosin heavy chain, muscle	622.828	2	25.2	753282	25.18	1645891	2.83835	
NYSTELFR	Mhc	Myosin heavy chain, muscle	515.254	2	28.61	584482	28.59	1201453	2.67029	
QIEEAEEIAALNLAK	Mhc	Myosin heavy chain, muscle	821.438	2	39.84	244676	39.61	641209	3.40432	
QLEEAESQVSQLSK	Mhc	Myosin heavy chain, muscle	788.397	2	24.66	238416	24.6	763584	4.16048	
TALLDSLSGEK	Mhc	Myosin heavy chain, muscle	567.306	2	31.71	635833	31.55	1551318	3.16942	
TLLEQADR	Mhc	Myosin heavy chain, muscle	473.254	2	18.74	809820	18.73	1724785	2.76674	
VIAYFATVGASK	Mhc	Myosin heavy chain, muscle	613.843	2	33.65	620379	33.48	1435185	3.0052	
AVCMLSNTTAIAEAWAR	alphaTub	Tubulin alpha-1 chain	940.953	2	39.84	709606	39.65	489135	0.89543	
AVFVDLEPTVVDEVR	alphaTub	Tubulin alpha-1 chain	844.449	2	44.89	13338792	44.71	10363053	1.00924	
AVFVDLEPTVVDEVR	alphaTub	Tubulin alpha-1 chain	563.302	3	44.92	3137291	44.67	2222947	0.92044	
DVNAAIATIK	alphaTub	Tubulin alpha-1 chain	508.293	2	27.73	3407066	27.65	2606071	0.99364	

FDGALNVDLTEFQTNLVPYPR	alphaTub	Tubulin alpha-1 chain	1205.11	2	53.41	332260	53.09	0	0
IHFPLVTYAPVISAEK	alphaTub	Tubulin alpha-1 chain	595.67	3	44.88	6502554	44.74	4535771	0.90613
LIGQIVSSITASLR	alphaTub	Tubulin alpha-1 chain	729.438	2	53.27	1768446	53.14	157727	0.11586
TIQFVDWCPTGFK	alphaTub	Tubulin alpha-1 chain	799.887	2	45.66	2155301	45.43	964521	0.58133
TVGGGDDSFNTFFSETGAGK	alphaTub	Tubulin alpha-1 chain	997.442	2	40.1	757733	39.88	250306	0.42912
VGINYQPPTVVPGGDLAK	alphaTub	Tubulin alpha-1 chain	912.996	2	36.97	4567866	36.76	2064425	0.58709
AVCMLSNTTAIAEAWAR	alphaTub	Tubulin alpha-2 chain	940.953	2	39.84	709606	39.65	489135	0.89543
AVFVDLEPTVVDEVR	alphaTub	Tubulin alpha-2 chain	844.449	2	44.89	13338792	44.71	10363053	1.00924
AVFVDLEPTVVDEVR	alphaTub	Tubulin alpha-2 chain	563.302	3	44.92	3137291	44.67	2222947	0.92044
DVNAAIATIK	alphaTub	Tubulin alpha-2 chain	508.293	2	27.73	3407066	27.65	2606071	0.99364
FDGALNVDLTEFQTNLVPYPR	alphaTub	Tubulin alpha-2 chain	1205.11	2	53.41	332260	53.09	0	0
LIGQIVSSITASLR	alphaTub	Tubulin alpha-2 chain	729.438	2	53.27	1768446	53.14	157727	0.11586
VGINYQPPTVVPGGDLAK	alphaTub	Tubulin alpha-2 chain	912.996	2	36.97	4567866	36.76	2064425	0.58709
AVCMLSNTTAIAEAWAR	alphaTub	Tubulin alpha-3 chain	940.953	2	39.84	709606	39.65	489135	0.89543
AVFVDLEPTVVDEVR	alphaTub	Tubulin alpha-3 chain	844.449	2	44.89	13338792	44.71	10363053	1.00924
AVFVDLEPTVVDEVR	alphaTub	Tubulin alpha-3 chain	563.302	3	44.92	3137291	44.67	2222947	0.92044
DVNAAIATIK	alphaTub	Tubulin alpha-3 chain	508.293	2	27.73	3407066	27.65	2606071	0.99364
FDGALNVDLTEFQTNLVPYPR	alphaTub	Tubulin alpha-3 chain	1205.11	2	53.41	332260	53.09	0	0
IHFPLVTYAPVISAEK	alphaTub	Tubulin alpha-3 chain	595.67	3	44.88	6502554	44.74	4535771	0.90613
LIGQIVSSITASLR	alphaTub	Tubulin alpha-3 chain	729.438	2	53.27	1768446	53.14	157727	0.11586
TIQFVDWCPTGFK	alphaTub	Tubulin alpha-3 chain	799.887	2	45.66	2155301	45.43	964521	0.58133
TVGGGDDSFNTFFSETGAGK	alphaTub	Tubulin alpha-3 chain	997.442	2	40.1	757733	39.88	250306	0.42912
VGINYQPPTVVPGGDLAK	alphaTub	Tubulin alpha-3 chain	912.996	2	36.97	4567866	36.76	2064425	0.58709
AQPGFGEDEVTIVLTGLPK	Yp3	Vitellogenin-3 (Vitellogenin III) (Yolk protein 3)	986.025	2	50.78	511312	50.51	698432	1.77444
YFAESVRPGSER	Yp3	Vitellogenin-3 (Vitellogenin III) (Yolk protein 3)	466.565	3	18.27	878476	18.27	1290612	1.90848
ALNLNPTLALIEK	Mlc1	Myosin light chain alkali	705.422	2	45.94	2478744	45.73	4382358	2.29667
LSEASQAADESER	Tm1	Tropomyosin-1, isoforms 9A/A/B (Cytoskeletal tropomyosin) (Tropomyosin II)	696.816	2	11.57	343010	11.67	695696	2.63472
VINDNFEIVEGLMTTVHATTATQK	Gapdh1	Glyceraldehyde-3-phosphate dehydrogenase 1 (EC 1.2.1.12) (Glyceraldehyde	883.446	3	44.43	15660984	44.21	14722617	1.2212
VPTPNVSVVDLTVR	Gapdh1	Glyceraldehyde-3-phosphate dehydrogenase 1 (EC 1.2.1.12) (Glyceraldehyde	748.428	2	39.69	2420706	39.45	1346589	0.72263
GAAQNIIPASTGAAK	Gapdh2	Glyceraldehyde-3-phosphate dehydrogenase 2 (EC 1.2.1.12) (Glyceraldehyde	685.375	2	22.79	1294864	22.83	1032530	1.03586
VINDNFEIVEGLMTTVHATTATQK	Gapdh2	Glyceraldehyde-3-phosphate dehydrogenase 2 (EC 1.2.1.12) (Glyceraldehyde	883.446	3	44.43	15660984	44.21	14722617	1.2212
VPTPNVSVVDLTVR	Gapdh2	Glyceraldehyde-3-phosphate dehydrogenase 2 (EC 1.2.1.12) (Glyceraldehyde	748.428	2	39.69	2420706	39.45	1346589	0.72263
ALQASVLR	Ald1	Fructose-bisphosphate aldolase (EC 4.1.2.13)	429.264	2	22.24	109791	22.21	356378	4.21664
IGGIGTVPVGR	eEF1alph	Elongation factor 1-alpha 1 (EF-1-alpha-1) (50 kDa female-specific protein)	513.309	2	26.78	8471779	26.71	7294916	1.11858
IHINIVVIGHVDSGK	eEF1alph	Elongation factor 1-alpha 1 (EF-1-alpha-1) (50 kDa female-specific protein)	534.311	3	34.63	1881392	34.42	1040176	0.71821
LPLQDVYK	eEF1alph	Elongation factor 1-alpha 1 (EF-1-alpha-1) (50 kDa female-specific protein)	488.279	2	28.46	1429878	28.37	739398	0.67174
SGDAAIVNLVPSKPLCVEAFQEFPP	eEF1alph	Elongation factor 1-alpha 1 (EF-1-alpha-1) (50 kDa female-specific protein)	1004.53	3	53.37	2199775	53.17	2605591	1.53869
STTTGHLIYK	eEF1alph	Elongation factor 1-alpha 1 (EF-1-alpha-1) (50 kDa female-specific protein)	560.803	2	15.84	2355586	15.79	1523568	0.8402
TLIDALDAILPPARPTDK	eEF1alph	Elongation factor 1-alpha 1 (EF-1-alpha-1) (50 kDa female-specific protein)	640.698	3	53.52	4586012	53.29	3427460	0.97087
VETGVLKPGTVVVFAPANITTEVK	eEF1alph	Elongation factor 1-alpha 1 (EF-1-alpha-1) (50 kDa female-specific protein)	823.804	3	44.29	5606537	44.1	4779433	1.1074
ALTVPELTQQMFDAK	betaTub6	Tubulin beta-3 chain (Beta-3-tubulin)	854.435	2	37.81	1590296	37.56	1317924	1.07655
IMNTYSVVPSPK	betaTub6	Tubulin beta-3 chain (Beta-3-tubulin)	668.352	2	29.06	1095355	28.94	858780	1.01847
LAVNMVPFPR	betaTub6	Tubulin beta-3 chain (Beta-3-tubulin)	572.321	2	40.41	1793279	40.19	932227	0.6753
LAVNMVPFPR	betaTub6	Tubulin beta-3 chain (Beta-3-tubulin)	580.318	2	34.29	768535	34.41	1250344	2.11343
LHFFMPGFAPLTSR	betaTub6	Tubulin beta-3 chain (Beta-3-tubulin)	540.95	3	48.83	2785496	48.88	1571753	0.733
LHFFMPGFAPLTSR	betaTub6	Tubulin beta-3 chain (Beta-3-tubulin)	546.282	3	42.62	1500055	42.47	820442	0.7105
NSSYFVEWIPNNVK	betaTub6	Tubulin beta-3 chain (Beta-3-tubulin)	848.92	2	44.43	6003407	44.1	632379	0.13684
IVSQHTSSSR	Lam	Lamin Dm0	551.286	2	7	45019	6.94	21557	0.62203
LATYIDR	Lam	Lamin Dm0	426.235	2	19.41	565173	19.45	354186	0.81409
LNITPATNTATVQSFSQSLR	Lam	Lamin Dm0	717.046	3	39.95	713399	39.69	972294	1.77047
TILLNSEGEAVANLDR	Lam	Lamin Dm0	857.952	2	36.67	1134982	36.48	1426663	1.63288
AGLQFPVGR	His2Av	Histone H2A.v (H2A.F/Z) (H2A.Z)	472.769	2	31.56	98633592	31.42	106838928	1.40711
ATIAGGGVIPHIHK	His2Av	Histone H2A.v (H2A.F/Z) (H2A.Z)	685.899	2	24.2	4969387	24.16	4711632	1.23166

ATIAGGGVIPHIHK	His2Av	Histone H2A.v (H2A.F/Z) (H2A.Z)	457.602	3	24.23	19229148	24.16	22945442	1.5501	
GDEELDSLIK	His2Av	Histone H2A.v (H2A.F/Z) (H2A.Z)	559.782	2	33.01	2609860	32.91	1105949	0.55048	
GNVILSQAY	His2Av	Histone H2A.v (H2A.F/Z) (H2A.Z)	482.759	2	31.72	3533263	31.6	3759713	1.3823	
HLQLAIR	His2Av	Histone H2A.v (H2A.F/Z) (H2A.Z)	425.767	2	22.72	21451580	22.66	9817742	0.59453	
VGATAAVYSAAILEYLTAEVLELAGI	His2Av	Histone H2A.v (H2A.F/Z) (H2A.Z)	965.85	3	65.73	7579277	65.74	7007764	1.20109	
VGATAAVYSAAILEYLTAEVLELAGI	His2Av	Histone H2A.v (H2A.F/Z) (H2A.Z)	724.639	4	65.73	1991616	65.74	2155629	1.40602	
LLTATESEVATQNR	Tm2	Tropomyosin-2 (Tropomyosin I)	766.899	2	21.98	140941	22.02	442612	4.07951	
ESTLHLVLR	Ubi-p63E	Polyubiquitin [Cleaved into: Ubiquitin]	534.314	2	29.06	19834334	28.96	12823394	0.83986	
ESTLHLVLR	Ubi-p63E	Polyubiquitin [Cleaved into: Ubiquitin]	356.545	3	29.02	13873703	28.96	8349692	0.78181	
ESTLHLVLR	Ubi-p63E	Polyubiquitin [Cleaved into: Ubiquitin]	534.314	2	29.06	19834334	28.96	12823394	0.83986	
ESTLHLVLR	Ubi-p63E	Polyubiquitin [Cleaved into: Ubiquitin]	356.545	3	29.02	13873703	28.96	8349692	0.78181	
ESTLHLVLR	Ubi-p63E	Polyubiquitin [Cleaved into: Ubiquitin]	534.314	2	29.06	19834334	28.96	12823394	0.83986	
ESTLHLVLR	Ubi-p63E	Polyubiquitin [Cleaved into: Ubiquitin]	356.545	3	29.02	13873703	28.96	8349692	0.78181	
ESTLHLVLR	Ubi-p63E	Polyubiquitin [Cleaved into: Ubiquitin]	534.314	2	29.06	19834334	28.96	12823394	0.83986	
ESTLHLVLR	Ubi-p63E	Polyubiquitin [Cleaved into: Ubiquitin]	356.545	3	29.02	13873703	28.96	8349692	0.78181	
ESTLHLVLR	Ubi-p63E	Polyubiquitin [Cleaved into: Ubiquitin]	534.314	2	29.06	19834334	28.96	12823394	0.83986	
ESTLHLVLR	Ubi-p63E	Polyubiquitin [Cleaved into: Ubiquitin]	356.545	3	29.02	13873703	28.96	8349692	0.78181	
ESTLHLVLR	Ubi-p63E	Polyubiquitin [Cleaved into: Ubiquitin]	534.314	2	29.06	19834334	28.96	12823394	0.83986	
ESTLHLVLR	Ubi-p63E	Polyubiquitin [Cleaved into: Ubiquitin]	356.545	3	29.02	13873703	28.96	8349692	0.78181	
ESTLHLVLR	Ubi-p63E	Polyubiquitin [Cleaved into: Ubiquitin]	534.314	2	29.06	19834334	28.96	12823394	0.83986	
ESTLHLVLR	Ubi-p63E	Polyubiquitin [Cleaved into: Ubiquitin]	356.545	3	29.02	13873703	28.96	8349692	0.78181	
ESTLHLVLR	Ubi-p63E	Polyubiquitin [Cleaved into: Ubiquitin]	534.314	2	29.06	19834334	28.96	12823394	0.83986	
ESTLHLVLR	Ubi-p63E	Polyubiquitin [Cleaved into: Ubiquitin]	356.545	3	29.02	13873703	28.96	8349692	0.78181	
ESTLHLVLR	Ubi-p63E	Polyubiquitin [Cleaved into: Ubiquitin]	534.314	2	29.06	19834334	28.96	12823394	0.83986	
ESTLHLVLR	Ubi-p63E	Polyubiquitin [Cleaved into: Ubiquitin]	356.545	3	29.02	13873703	28.96	8349692	0.78181	
ESTLHLVLR	Ubi-p63E	Polyubiquitin [Cleaved into: Ubiquitin]	534.314	2	29.06	19834334	28.96	12823394	0.83986	
ESTLHLVLR	Ubi-p63E	Polyubiquitin [Cleaved into: Ubiquitin]	356.545	3	29.02	13873703	28.96	8349692	0.78181	
MQIFVK	Ubi-p63E	Polyubiquitin [Cleaved into: Ubiquitin]	383.22	2	27.43	8697430	27.39	10348569	1.54565	
TITLEVEPSDTIENVK	Ubi-p63E	Polyubiquitin [Cleaved into: Ubiquitin]	894.467	2	36.85	41743372	36.64	47966256	1.49269	
TITLEVEPSDTIENVK	Ubi-p63E	Polyubiquitin [Cleaved into: Ubiquitin]	596.647	3	36.85	1563861	36.68	1963090	1.63066	
TITLEVEPSDTIENVK	Ubi-p63E	Polyubiquitin [Cleaved into: Ubiquitin]	894.467	2	36.85	41743372	36.64	47966256	1.49269	
TITLEVEPSDTIENVK	Ubi-p63E	Polyubiquitin [Cleaved into: Ubiquitin]	596.647	3	36.85	1563861	36.68	1963090	1.63066	
TITLEVEPSDTIENVK	Ubi-p63E	Polyubiquitin [Cleaved into: Ubiquitin]	894.467	2	36.85	41743372	36.64	47966256	1.49269	
TITLEVEPSDTIENVK	Ubi-p63E	Polyubiquitin [Cleaved into: Ubiquitin]	596.647	3	36.85	1563861	36.68	1963090	1.63066	
TITLEVEPSDTIENVK	Ubi-p63E	Polyubiquitin [Cleaved into: Ubiquitin]	894.467	2	36.85	41743372	36.64	47966256	1.49269	
TITLEVEPSDTIENVK	Ubi-p63E	Polyubiquitin [Cleaved into: Ubiquitin]	596.647	3	36.85	1563861	36.68	1963090	1.63066	
TITLEVEPSDTIENVK	Ubi-p63E	Polyubiquitin [Cleaved into: Ubiquitin]	894.467	2	36.85	41743372	36.64	47966256	1.49269	
TITLEVEPSDTIENVK	Ubi-p63E	Polyubiquitin [Cleaved into: Ubiquitin]	596.647	3	36.85	1563861	36.68	1963090	1.63066	
TITLEVEPSDTIENVK	Ubi-p63E	Polyubiquitin [Cleaved into: Ubiquitin]	894.467	2	36.85	41743372	36.64	47966256	1.49269	
TITLEVEPSDTIENVK	Ubi-p63E	Polyubiquitin [Cleaved into: Ubiquitin]	596.647	3	36.85	1563861	36.68	1963090	1.63066	
TITLEVEPSDTIENVK	Ubi-p63E	Polyubiquitin [Cleaved into: Ubiquitin]	894.467	2	36.85	41743372	36.64	47966256	1.49269	
TITLEVEPSDTIENVK	Ubi-p63E	Polyubiquitin [Cleaved into: Ubiquitin]	596.647	3	36.85	1563861	36.68	1963090	1.63066	
TITLEVEPSDTIENVK	Ubi-p63E	Polyubiquitin [Cleaved into: Ubiquitin]	894.467	2	36.85	41743372	36.64	47966256	1.49269	
TITLEVEPSDTIENVK	Ubi-p63E	Polyubiquitin [Cleaved into: Ubiquitin]	596.647	3	36.85	1563861	36.68	1963090	1.63066	
TITLEVEPSDTIENVK	Ubi-p63E	Polyubiquitin [Cleaved into: Ubiquitin]	894.467	2	36.85	41743372	36.64	47966256	1.49269	
TITLEVEPSDTIENVK	Ubi-p63E	Polyubiquitin [Cleaved into: Ubiquitin]	596.647	3	36.85	1563861	36.68	1963090	1.63066	
TITLEVEPSDTIENVK	Ubi-p63E	Polyubiquitin [Cleaved into: Ubiquitin]	894.467	2	36.85	41743372	36.64	47966256	1.49269	
TITLEVEPSDTIENVK	Ubi-p63E	Polyubiquitin [Cleaved into: Ubiquitin]	596.647	3	36.85	1563861	36.68	1963090	1.63066	
TLSDYNIQK	Ubi-p63E	Polyubiquitin [Cleaved into: Ubiquitin]	541.28	2	19.53	19914654	19.52	7860957	0.51277	
TLSDYNIQK	Ubi-p63E	Polyubiquitin [Cleaved into: Ubiquitin]	541.28	2	19.53	19914654	19.52	7860957	0.51277	
TLSDYNIQK	Ubi-p63E	Polyubiquitin [Cleaved into: Ubiquitin]	541.28	2	19.53	19914654	19.52	7860957	0.51277	
TLSDYNIQK	Ubi-p63E	Polyubiquitin [Cleaved into: Ubiquitin]	541.28	2	19.53	19914654	19.52	7860957	0.51277	
TLSDYNIQK	Ubi-p63E	Polyubiquitin [Cleaved into: Ubiquitin]	541.28	2	19.53	19914654	19.52	7860957	0.51277	

TLSDYNIQK	Ubi-p63E	Polyubiquitin [Cleaved into: Ubiquitin]	541.28	2	19.53	19914654	19.52	7860957	0.51277
TLSDYNIQK	Ubi-p63E	Polyubiquitin [Cleaved into: Ubiquitin]	541.28	2	19.53	19914654	19.52	7860957	0.51277
TLSDYNIQK	Ubi-p63E	Polyubiquitin [Cleaved into: Ubiquitin]	541.28	2	19.53	19914654	19.52	7860957	0.51277
TLSDYNIQK	Ubi-p63E	Polyubiquitin [Cleaved into: Ubiquitin]	541.28	2	19.53	19914654	19.52	7860957	0.51277
TLSDYNIQK	Ubi-p63E	Polyubiquitin [Cleaved into: Ubiquitin]	541.28	2	19.53	19914654	19.52	7860957	0.51277
AAIELNR	ninaC	Neither inactivation nor afterpotential protein C (EC 2.7.11.1)	393.727	2	17.87	27934492	17.87	8147882	0.3789
ADLEYKPR	ninaC	Neither inactivation nor afterpotential protein C (EC 2.7.11.1)	496.264	2	14.09	8517788	14.09	3261920	0.49747
ADVWALGITTIELADGKPPFADMHP	ninaC	Neither inactivation nor afterpotential protein C (EC 2.7.11.1)	974.832	3	55.59	2262995	55.61	1325024	0.76061
ADVWALGITTIELADGKPPFADMHP	ninaC	Neither inactivation nor afterpotential protein C (EC 2.7.11.1)	731.376	4	55.55	5864969	55.61	3224204	0.71413
ADVWALGITTIELADGKPPFADMHP	ninaC	Neither inactivation nor afterpotential protein C (EC 2.7.11.1)	980.164	3	53.25	2367700	53.26	1572627	0.86282
ADVWALGITTIELADGKPPFADMHP	ninaC	Neither inactivation nor afterpotential protein C (EC 2.7.11.1)	735.375	4	53.22	7293140	53.22	4530989	0.80705
AFTDINR	ninaC	Neither inactivation nor afterpotential protein C (EC 2.7.11.1)	418.717	2	17.64	9521909	17.64	2491073	0.33985
AILMLVNAGTPVNNDSTR	ninaC	Neither inactivation nor afterpotential protein C (EC 2.7.11.1)	943.494	2	39.58	4360804	39.35	1237694	0.3687
AILMLVNAGTPVNNDSTR	ninaC	Neither inactivation nor afterpotential protein C (EC 2.7.11.1)	629.332	3	39.61	1175945	39.7	524491	0.57939
ALGVLDTVIAR	ninaC	Neither inactivation nor afterpotential protein C (EC 2.7.11.1)	564.343	2	40.21	85712096	40.04	54023652	0.81877
AMFQIIR	ninaC	Neither inactivation nor afterpotential protein C (EC 2.7.11.1)	439.749	2	35.65	17816182	35.48	11806378	0.86084
AVFGDTNAIIIHDMFGFECFNR	ninaC	Neither inactivation nor afterpotential protein C (EC 2.7.11.1)	858.735	3	55.9	3358748	55.78	1202619	0.46513
CVLQYCLTFGK	ninaC	Neither inactivation nor afterpotential protein C (EC 2.7.11.1)	694.839	2	38.91	1761165	38.62	991756	0.73152
DAVASTLYSR	ninaC	Neither inactivation nor afterpotential protein C (EC 2.7.11.1)	541.777	2	22.04	13835789	22.06	3379994	0.31735
DDPEGNVER	ninaC	Neither inactivation nor afterpotential protein C (EC 2.7.11.1)	515.725	2	9.74	412063	9.86	43490	0.1371
DYCDHPNLPEFYGVYK	ninaC	Neither inactivation nor afterpotential protein C (EC 2.7.11.1)	1008.94	2	37.72	913930	37.56	316723	0.45018
DYCDHPNLPEFYGVYK	ninaC	Neither inactivation nor afterpotential protein C (EC 2.7.11.1)	672.965	3	37.72	4680463	37.56	1886732	0.52365
EQACQTNWDR	ninaC	Neither inactivation nor afterpotential protein C (EC 2.7.11.1)	654.275	2	14.22	2326577	14.16	675773	0.37732
EVNSSQLGPLPVPIK	ninaC	Neither inactivation nor afterpotential protein C (EC 2.7.11.1)	789.449	2	40.14	11621809	39.92	4206333	0.47017
FQDFVNLSQQVHIYNQR	ninaC	Neither inactivation nor afterpotential protein C (EC 2.7.11.1)	1068.53	2	39.17	2051933	39.04	701782	0.44428
FQDFVNLSQQVHIYNQR	ninaC	Neither inactivation nor afterpotential protein C (EC 2.7.11.1)	712.692	3	39.2	37242856	39	15147834	0.52836
FTCLTLLK	ninaC	Neither inactivation nor afterpotential protein C (EC 2.7.11.1)	498.283	2	37.02	3865875	36.79	785519	0.26396
GDNILLTK	ninaC	Neither inactivation nor afterpotential protein C (EC 2.7.11.1)	437.256	2	23.96	7840105	24.03	3269877	0.54179
GSDSGDQHTFQPPK	ninaC	Neither inactivation nor afterpotential protein C (EC 2.7.11.1)	750.839	2	15.01	635465	15.09	79069	0.16164
GSDSGDQHTFQPPK	ninaC	Neither inactivation nor afterpotential protein C (EC 2.7.11.1)	500.895	3	15.01	4659666	14.99	900055	0.25092
GSETNTYDFNNR	ninaC	Neither inactivation nor afterpotential protein C (EC 2.7.11.1)	709.303	2	17.48	9864913	17.48	4245090	0.55901
GYAAPPPPPPPMPSSNYYR	ninaC	Neither inactivation nor afterpotential protein C (EC 2.7.11.1)	1037.99	2	26.91	1737174	26.9	941907	0.70435
HLCYLGDGNR	ninaC	Neither inactivation nor afterpotential protein C (EC 2.7.11.1)	602.78	2	17.48	8493956	17.49	1859001	0.28431
HSQFVK	ninaC	Neither inactivation nor afterpotential protein C (EC 2.7.11.1)	373.203	2	9.26	4129579	9.38	1484137	0.46686
HTATEISVAHYTGR	ninaC	Neither inactivation nor afterpotential protein C (EC 2.7.11.1)	771.887	2	18.64	1826203	18.65	976342	0.6945
HTATEISVAHYTGR	ninaC	Neither inactivation nor afterpotential protein C (EC 2.7.11.1)	514.927	3	18.67	34603876	18.61	24736648	0.92862
INMNMSFPR	ninaC	Neither inactivation nor afterpotential protein C (EC 2.7.11.1)	555.265	2	33.61	1257006	33.45	473494	0.48933
INMNMSFPR	ninaC	Neither inactivation nor afterpotential protein C (EC 2.7.11.1)	563.263	2	24.81	6223789	24.72	1413809	0.29509
INMNMSFPR	ninaC	Neither inactivation nor afterpotential protein C (EC 2.7.11.1)	571.26	2	17.71	1092556	17.64	317990	0.37809
IQHYDEEHQVSIEEEYR	ninaC	Neither inactivation nor afterpotential protein C (EC 2.7.11.1)	735.335	3	21.82	9144805	21.85	3841429	0.54568
IQHYDEEHQVSIEEEYR	ninaC	Neither inactivation nor afterpotential protein C (EC 2.7.11.1)	551.753	4	21.82	11296823	21.81	5372007	0.61774
LDQIPFYDTQYMVDPANSISR	ninaC	Neither inactivation nor afterpotential protein C (EC 2.7.11.1)	825.062	3	47.1	965323	46.8	285191	0.38378
LDQIPFYDTQYMVDPANSISR	ninaC	Neither inactivation nor afterpotential protein C (EC 2.7.11.1)	1245.09	2	42.44	737594	42.18	108031	0.19026
LDQIPFYDTQYMVDPANSISR	ninaC	Neither inactivation nor afterpotential protein C (EC 2.7.11.1)	830.393	3	42.44	2133232	42.21	249429	0.15189
LPFDEFLR	ninaC	Neither inactivation nor afterpotential protein C (EC 2.7.11.1)	518.777	2	45.35	37304816	45.13	12833087	0.44688
LYELQVK	ninaC	Neither inactivation nor afterpotential protein C (EC 2.7.11.1)	446.761	2	26.36	7134839	26.31	1279726	0.233
MGESDNIYNQGYFR	ninaC	Neither inactivation nor afterpotential protein C (EC 2.7.11.1)	855.365	2	29.06	790199	28.98	104614	0.17198
MLSQNANLGVHFVR	ninaC	Neither inactivation nor afterpotential protein C (EC 2.7.11.1)	529.28	3	31.04	3025753	30.96	721657	0.30983
MLSQNANLGVHFVR	ninaC	Neither inactivation nor afterpotential protein C (EC 2.7.11.1)	534.612	3	28.76	906685	28.64	111527	0.15979
MYPEDLAALENPVDENIIESLR	ninaC	Neither inactivation nor afterpotential protein C (EC 2.7.11.1)	1266.12	2	55.67	1021590	55.37	543268	0.69081
MYPEDLAALENPVDENIIESLR	ninaC	Neither inactivation nor afterpotential protein C (EC 2.7.11.1)	844.416	3	55.74	3400744	55.49	1974218	0.75413
MYPEDLAALENPVDENIIESLR	ninaC	Neither inactivation nor afterpotential protein C (EC 2.7.11.1)	1274.12	2	53.98	1115935	53.78	538899	0.62732
MYPEDLAALENPVDENIIESLR	ninaC	Neither inactivation nor afterpotential protein C (EC 2.7.11.1)	849.748	3	54.05	4350341	53.78	2829471	0.8449

NEGDNSEDPPFNFK	ninaC	Neither inactivation nor afterpotential protein C (EC 2.7.11.1)	805.342	2	30.49	20135672	30.36	13465555	0.86872	
NHVLHR	ninaC	Neither inactivation nor afterpotential protein C (EC 2.7.11.1)	388.22	2	7.47	3112849	7.55	783942	0.32715	
NPPPTLMRPTNWSQQINDFISESLE	ninaC	Neither inactivation nor afterpotential protein C (EC 2.7.11.1)	1014.84	3	55.94	2051910	55.85	1795906	1.13697	
NPPPTLMRPTNWSQQINDFISESLE	ninaC	Neither inactivation nor afterpotential protein C (EC 2.7.11.1)	1020.17	3	52.77	5616241	52.6	3954417	0.91466	
NPSMTSCALTYNAYK	ninaC	Neither inactivation nor afterpotential protein C (EC 2.7.11.1)	860.887	2	28.39	475234	28.22	100326	0.27424	
NPSMTSCALTYNAYK	ninaC	Neither inactivation nor afterpotential protein C (EC 2.7.11.1)	868.885	2	23.43	1293065	23.38	266318	0.26755	
QAFPNQLLTQHMEDDEPWDSPLQI	ninaC	Neither inactivation nor afterpotential protein C (EC 2.7.11.1)	965.784	3	44.28	1072287	44.02	620225	0.75138	
QAFPNQLLTQHMEDDEPWDSPLQI	ninaC	Neither inactivation nor afterpotential protein C (EC 2.7.11.1)	971.115	3	41.07	2202046	40.9	1292612	0.76254	
QYTTEEAR	ninaC	Neither inactivation nor afterpotential protein C (EC 2.7.11.1)	499.233	2	9.23	582523	9.39	114303	0.2549	
SCQDQDLIMDR	ninaC	Neither inactivation nor afterpotential protein C (EC 2.7.11.1)	690.798	2	23.63	8294816	23.65	3307863	0.51804	
SCQDQDLIMDR	ninaC	Neither inactivation nor afterpotential protein C (EC 2.7.11.1)	698.795	2	18.85	5333858	18.8	2256882	0.54965	
SFEDSNAR	ninaC	Neither inactivation nor afterpotential protein C (EC 2.7.11.1)	463.204	2	9.94	841733	10.06	841427	1.29857	
SFEEAGSYVEEEIAPGITLSGYAVDI	ninaC	Neither inactivation nor afterpotential protein C (EC 2.7.11.1)	916.104	3	58.22	3614784	57.91	3303289	1.1871	
SFHSDVVQQQMK	ninaC	Neither inactivation nor afterpotential protein C (EC 2.7.11.1)	717.346	2	18.46	3748466	18.46	2122639	0.73561	
SFHSDVVQQQMK	ninaC	Neither inactivation nor afterpotential protein C (EC 2.7.11.1)	478.566	3	18.46	11674488	18.42	8562709	0.95279	
SFHSDVVQQQMK	ninaC	Neither inactivation nor afterpotential protein C (EC 2.7.11.1)	725.343	2	13.74	1001280	13.67	558782	0.72495	
SFHSDVVQQQMK	ninaC	Neither inactivation nor afterpotential protein C (EC 2.7.11.1)	483.898	3	13.74	2486371	13.7	1898734	0.99202	
SGQLNENTADFIRPFAK	ninaC	Neither inactivation nor afterpotential protein C (EC 2.7.11.1)	954.484	2	36.11	2672476	35.9	869006	0.42241	
SGQLNENTADFIRPFAK	ninaC	Neither inactivation nor afterpotential protein C (EC 2.7.11.1)	636.659	3	36.08	34215476	35.93	14653759	0.55635	
SIFQVLLHYR	ninaC	Neither inactivation nor afterpotential protein C (EC 2.7.11.1)	638.364	2	47.38	14050704	47.32	3586610	0.3316	
SIFQVLLHYR	ninaC	Neither inactivation nor afterpotential protein C (EC 2.7.11.1)	425.912	3	47.38	24262952	47.32	7253295	0.38834	
SSLDESIMLMFTNQLTK	ninaC	Neither inactivation nor afterpotential protein C (EC 2.7.11.1)	979.484	2	58.98	726179	58.79	934663	1.67199	
SSLDESIMLMFTNQLTK	ninaC	Neither inactivation nor afterpotential protein C (EC 2.7.11.1)	653.325	3	58.9	731597	58.75	1150737	2.04327	
SSLDESIMLMFTNQLTK	ninaC	Neither inactivation nor afterpotential protein C (EC 2.7.11.1)	987.482	2	52.23	1067574	51.98	1917715	2.3335	
SSLDESIMLMFTNQLTK	ninaC	Neither inactivation nor afterpotential protein C (EC 2.7.11.1)	658.657	3	52.23	889198	51.98	1157228	1.69061	
SSYPPSDPVR	ninaC	Neither inactivation nor afterpotential protein C (EC 2.7.11.1)	552.77	2	16.78	6761750	16.82	306942	0.05897	
SYALNTLSAGCISQVNNLR	ninaC	Neither inactivation nor afterpotential protein C (EC 2.7.11.1)	1041.03	2	38.84	1038066	38.54	265120	0.33177	
SYALNTLSAGCISQVNNLR	ninaC	Neither inactivation nor afterpotential protein C (EC 2.7.11.1)	694.353	3	38.76	1954606	38.58	912018	0.60613	
TALDNLLTKPDGLFYIIDDASR	ninaC	Neither inactivation nor afterpotential protein C (EC 2.7.11.1)	1226.14	2	58.41	1814072	58.25	838256	0.60027	
TALDNLLTKPDGLFYIIDDASR	ninaC	Neither inactivation nor afterpotential protein C (EC 2.7.11.1)	817.764	3	58.41	21971862	58.25	11525014	0.68139	
TLAANFR	ninaC	Neither inactivation nor afterpotential protein C (EC 2.7.11.1)	396.722	2	20.39	6184618	20.39	1161221	0.24391	
VATTDGTQHNFHIFYYFYDFINQQN	ninaC	Neither inactivation nor afterpotential protein C (EC 2.7.11.1)	863.914	4	56.09	1522872	55.61	34117	0.0291	
YQDDEGYNSSSGNYGVSR	ninaC	Neither inactivation nor afterpotential protein C (EC 2.7.11.1)	666.608	3	17.71	250320	17.76	340207	1.76551	
YYNDEFLAR	ninaC	Neither inactivation nor afterpotential protein C (EC 2.7.11.1)	595.777	2	28.3	10393152	28.23	1903322	0.2379	
AGFAGDDAPR	Act87E	Actin-87E	488.728	2	15.06	5346544	15.03	6072874	1.47551	
AVFPSIVGRPR	Act87E	Actin-87E	599.856	2	30.18	3527039	29.97	6233553	2.29587	
DSYVGDEAQSK	Act87E	Actin-87E	599.765	2	11.94	533801	12.02	513658	1.25002	
IWHHTFYNELR	Act87E	Actin-87E	505.921	3	25.48	1669489	25.49	2792816	2.17311	
SYELPDGQVITIGNER	Act87E	Actin-87E	895.95	2	39.17	5791673	39	8517636	1.91046	
SYELPDGQVITIGNER	Act87E	Actin-87E	597.635	3	39.17	427938	38.97	800279	2.42931	
TTGIVLDSGDGVSHTVPIYEGYALPI	Act87E	Actin-87E	1051.22	3	45.78	1749429	45.58	1016220	0.75459	
VAPEEHPVLLTEAPLNPK	Act87E	Actin-87E	977.536	2	34.25	620752	34.11	748708	1.56681	
VAPEEHPVLLTEAPLNPK	Act87E	Actin-87E	652.026	3	34.21	12031327	34.11	16999678	1.83548	
AGFAGDDAPR	Act5C	Actin-5C	488.728	2	15.06	5346544	15.03	6072874	1.47551	
AVFPSIVGRPR	Act5C	Actin-5C	599.856	2	30.18	3527039	29.97	6233553	2.29587	
DSYVGDEAQSK	Act5C	Actin-5C	599.765	2	11.94	533801	12.02	513658	1.25002	
IWHHTFYNELR	Act5C	Actin-5C	505.921	3	25.48	1669489	25.49	2792816	2.17311	
SYELPDGQVITIGNER	Act5C	Actin-5C	895.95	2	39.17	5791673	39	8517636	1.91046	
SYELPDGQVITIGNER	Act5C	Actin-5C	597.635	3	39.17	427938	38.97	800279	2.42931	
TTGIVLDSGDGVSHTVPIYEGYALPI	Act5C	Actin-5C	1051.22	3	45.78	1749429	45.58	1016220	0.75459	
VAPEEHPVLLTEAPLNPK	Act5C	Actin-5C	977.536	2	34.25	620752	34.11	748708	1.56681	
VAPEEHPVLLTEAPLNPK	Act5C	Actin-5C	652.026	3	34.21	12031327	34.11	16999678	1.83548	
FAPEEISSMVLTK	Hsc70-2	Heat shock 70 kDa protein cognate 2 (Heat shock 70 kDa protein 87D)	726.376	2	41.77	22231	41.79	11132469	650.511	

FAPEEISSMVLTK	Hsc70-2	Heat shock 70 kDa protein cognate 2 (Heat shock 70 kDa protein 87D)	484.587	3	41.77	0	41.79	276280	#DIV/0!
FAPEEISSMVLTK	Hsc70-2	Heat shock 70 kDa protein cognate 2 (Heat shock 70 kDa protein 87D)	734.374	2	35.12	297494	34.86	2047640	8.94124
QQQIFTTYSDNQNAVTIQVYEGER	Hsc70-2	Heat shock 70 kDa protein cognate 2 (Heat shock 70 kDa protein 87D)	944.786	3	42.73	477571	42.56	627649	1.70727
VEIIANDQGNR	Hsc70-2	Heat shock 70 kDa protein cognate 2 (Heat shock 70 kDa protein 87D)	614.818	2	18.24	20421124	18.27	30231408	1.9231
APAVGIDLGTTYSCVGVFQHGK	Hsc70-4	Heat shock 70 kDa protein cognate 4 (Heat shock 70 kDa protein 88E)	759.716	3	40.45	5148820	40.23	3011430	0.75978
ATLDEDNLK	Hsc70-4	Heat shock 70 kDa protein cognate 4 (Heat shock 70 kDa protein 88E)	509.756	2	17.44	12568892	17.45	9571772	0.98928
DAGTIAGLNVLR	Hsc70-4	Heat shock 70 kDa protein cognate 4 (Heat shock 70 kDa protein 88E)	600.341	2	38.18	41262500	38.03	54952584	1.73004
ELEGVCNPIITK	Hsc70-4	Heat shock 70 kDa protein cognate 4 (Heat shock 70 kDa protein 88E)	686.861	2	30.64	3930112	30.51	6693284	2.21236
ETAEAYLGK	Hsc70-4	Heat shock 70 kDa protein cognate 4 (Heat shock 70 kDa protein 88E)	491.248	2	18.78	1276400	18.74	1203743	1.22509
FEELNADLFR	Hsc70-4	Heat shock 70 kDa protein cognate 4 (Heat shock 70 kDa protein 88E)	627.312	2	40.11	5534845	39.92	4957492	1.16353
FELSGIPPAPR	Hsc70-4	Heat shock 70 kDa protein cognate 4 (Heat shock 70 kDa protein 88E)	592.327	2	35.41	8081636	34.58	6361236	1.0225
HWPFEVVSADGKPK	Hsc70-4	Heat shock 70 kDa protein cognate 4 (Heat shock 70 kDa protein 88E)	532.944	3	32.25	1810179	32.17	1759279	1.26251
LLQDLFNGK	Hsc70-4	Heat shock 70 kDa protein cognate 4 (Heat shock 70 kDa protein 88E)	524.295	2	37.39	815568	37.18	536116	0.85393
LVTHFVQEFK	Hsc70-4	Heat shock 70 kDa protein cognate 4 (Heat shock 70 kDa protein 88E)	416.564	3	29.17	6951855	29.06	4441078	0.82987
NQVAMNPTQTIFDAK	Hsc70-4	Heat shock 70 kDa protein cognate 4 (Heat shock 70 kDa protein 88E)	839.417	2	35.8	3572564	35.58	4997890	1.81731
NQVAMNPTQTIFDAK	Hsc70-4	Heat shock 70 kDa protein cognate 4 (Heat shock 70 kDa protein 88E)	847.414	2	30.99	17359498	30.83	24582760	1.83957
NVLIFDLGGGTFDVSILSIDDGIFEVI	Hsc70-4	Heat shock 70 kDa protein cognate 4 (Heat shock 70 kDa protein 88E)	961.839	3	65.61	296742	65.62	318618	1.39481
QTQTFTTYSDNQPGVLIQVYEGER	Hsc70-4	Heat shock 70 kDa protein cognate 4 (Heat shock 70 kDa protein 88E)	925.447	3	42.59	6743949	42.36	8544964	1.64596
SINPDEAVAYGAAVQAAILHGDK	Hsc70-4	Heat shock 70 kDa protein cognate 4 (Heat shock 70 kDa protein 88E)	770.729	3	49.12	5844145	48.87	6076260	1.35063
STAGDTHLGGEDFDNR	Hsc70-4	Heat shock 70 kDa protein cognate 4 (Heat shock 70 kDa protein 88E)	846.366	2	18.67	1657789	18.65	1831600	1.43524
STAGDTHLGGEDFDNR	Hsc70-4	Heat shock 70 kDa protein cognate 4 (Heat shock 70 kDa protein 88E)	564.58	3	18.67	10781481	18.68	14815577	1.7851
SVIHDIVLVGGSTR	Hsc70-4	Heat shock 70 kDa protein cognate 4 (Heat shock 70 kDa protein 88E)	726.912	2	33.42	16676664	33.29	17014050	1.32532
SVIHDIVLVGGSTR	Hsc70-4	Heat shock 70 kDa protein cognate 4 (Heat shock 70 kDa protein 88E)	484.944	3	33.42	25909346	33.29	28104442	1.4091
TFFPEEISSMVLTK	Hsc70-4	Heat shock 70 kDa protein cognate 4 (Heat shock 70 kDa protein 88E)	814.916	2	50.99	8350954	50.71	10307238	1.60335
TFFPEEISSMVLTK	Hsc70-4	Heat shock 70 kDa protein cognate 4 (Heat shock 70 kDa protein 88E)	543.613	3	50.99	1053530	50.71	1236310	1.52441
TFFPEEISSMVLTK	Hsc70-4	Heat shock 70 kDa protein cognate 4 (Heat shock 70 kDa protein 88E)	822.913	2	44.06	1805317	44.17	2932806	2.11034
TFFPEEISSMVLTK	Hsc70-4	Heat shock 70 kDa protein cognate 4 (Heat shock 70 kDa protein 88E)	548.944	3	43.99	365196	44.13	736202	2.61875
TLSSSTQASIEIDSLFEGTDFYTSIT	Hsc70-4	Heat shock 70 kDa protein cognate 4 (Heat shock 70 kDa protein 88E)	990.48	3	57.43	13069925	57.18	15772457	1.56765
TTPSYVAFTDTER	Hsc70-4	Heat shock 70 kDa protein cognate 4 (Heat shock 70 kDa protein 88E)	744.354	2	29.29	49722228	29.17	61082460	1.59584
TVTNAVITVPAYFNDSQR	Hsc70-4	Heat shock 70 kDa protein cognate 4 (Heat shock 70 kDa protein 88E)	998.51	2	40.66	11484469	40.5	14976923	1.69408
TVTNAVITVPAYFNDSQR	Hsc70-4	Heat shock 70 kDa protein cognate 4 (Heat shock 70 kDa protein 88E)	666.009	3	40.66	13980052	40.46	17266742	1.60444
VEIIANDQGNR	Hsc70-4	Heat shock 70 kDa protein cognate 4 (Heat shock 70 kDa protein 88E)	614.818	2	18.24	20421124	18.27	30231408	1.9231
ISGNHLTEIPDDAFTGLER	chp	Chaoptin (Photoreceptor cell-specific membrane protein)	695.68	3	38.69	287292	38.5	281624	1.27341
LLLTDNILSEIPYDALGPLK	chp	Chaoptin (Photoreceptor cell-specific membrane protein)	1099.62	2	62.1	575340	61.86	486645	1.09878
LLLTDNILSEIPYDALGPLK	chp	Chaoptin (Photoreceptor cell-specific membrane protein)	733.415	3	62.05	449702	61.83	498658	1.44046
AAASIILR	CCT1	T-complex protein 1 subunit alpha (TCP-1-alpha) (CCT-alpha) (Chaperonin co	407.761	2	25.72	2915044	25.64	1149857	0.51241
EQLAIAEFAK	CCT1	T-complex protein 1 subunit alpha (TCP-1-alpha) (CCT-alpha) (Chaperonin co	560.306	2	35.24	3285457	35.02	1749112	0.69158
FATEAAITILR	CCT1	T-complex protein 1 subunit alpha (TCP-1-alpha) (CCT-alpha) (Chaperonin co	603.348	2	39.36	10718412	39.2	13176747	1.59698
ICDDELILIK	CCT1	T-complex protein 1 subunit alpha (TCP-1-alpha) (CCT-alpha) (Chaperonin co	616.334	2	39.17	3199977	38.93	2711421	1.10071
IHPTSIISGYR	CCT1	T-complex protein 1 subunit alpha (TCP-1-alpha) (CCT-alpha) (Chaperonin co	622.343	2	25.75	1934865	25.67	1766943	1.1863
IIGADAEFFSAMVVDAAQSVK	CCT1	T-complex protein 1 subunit alpha (TCP-1-alpha) (CCT-alpha) (Chaperonin co	723.701	3	60.05	1786490	59.91	2285436	1.66185
IIGADAEFFSAMVVDAAQSVK	CCT1	T-complex protein 1 subunit alpha (TCP-1-alpha) (CCT-alpha) (Chaperonin co	729.033	3	49.26	1206828	48.93	1391766	1.49811
LLEVEHPAAK	CCT1	T-complex protein 1 subunit alpha (TCP-1-alpha) (CCT-alpha) (Chaperonin co	553.814	2	17.76	1488962	17.76	1720402	1.50096
LLEVEHPAAK	CCT1	T-complex protein 1 subunit alpha (TCP-1-alpha) (CCT-alpha) (Chaperonin co	369.545	3	17.76	5109354	17.76	6443911	1.63835
MLVDDIGDVTVTNDGATILR	CCT1	T-complex protein 1 subunit alpha (TCP-1-alpha) (CCT-alpha) (Chaperonin co	1059.54	2	45.59	805671	45.35	784141	1.26433
MLVDDIGDVTVTNDGATILR	CCT1	T-complex protein 1 subunit alpha (TCP-1-alpha) (CCT-alpha) (Chaperonin co	1067.54	2	42.51	681498	42.29	684194	1.30418
MLVDDIGDVTVTNDGATILR	CCT1	T-complex protein 1 subunit alpha (TCP-1-alpha) (CCT-alpha) (Chaperonin co	712.028	3	42.51	590691	42.33	540350	1.18833
SLLVIPK	CCT1	T-complex protein 1 subunit alpha (TCP-1-alpha) (CCT-alpha) (Chaperonin co	385.263	2	33.27	2316109	33.09	1393818	0.78175
SSLGPVGLDK	CCT1	T-complex protein 1 subunit alpha (TCP-1-alpha) (CCT-alpha) (Chaperonin co	486.772	2	24.74	2956948	24.72	3410829	1.49844
SYHNSSQTKPER	CCT1	T-complex protein 1 subunit alpha (TCP-1-alpha) (CCT-alpha) (Chaperonin co	717.342	2	6.8	51478	6.73	17702	0.44671
SYHNSSQTKPER	CCT1	T-complex protein 1 subunit alpha (TCP-1-alpha) (CCT-alpha) (Chaperonin co	478.564	3	6.8	142153	6.73	56320	0.51467
TQNVMAALSISNIVK	CCT1	T-complex protein 1 subunit alpha (TCP-1-alpha) (CCT-alpha) (Chaperonin co	794.94	2	46.95	1284833	46.68	1508805	1.52549
TQNVMAALSISNIVK	CCT1	T-complex protein 1 subunit alpha (TCP-1-alpha) (CCT-alpha) (Chaperonin co	802.937	2	39.73	2202017	39.48	2641875	1.55853

VLVELAQLQDEEVGDGTTSVVILAA	CCT1	T-complex protein 1 subunit alpha (TCP-1-alpha) (CCT-alpha) (Chaperonin cc	1018.23	3	65.61	1972773	65.17	0	0
WTGLDLIEGVVR	CCT1	T-complex protein 1 subunit alpha (TCP-1-alpha) (CCT-alpha) (Chaperonin cc	679.377	2	53.01	1351754	52.71	645340	0.62017
YFVEAGAMAVR	CCT1	T-complex protein 1 subunit alpha (TCP-1-alpha) (CCT-alpha) (Chaperonin cc	615.303	2	24.89	676486	24.87	566812	1.08844
DITEASIWDSYVLPK	RpS26	40S ribosomal protein S26 (DS31)	868.941	2	48.98	315622	48.7	205408	0.84542
ALLELQLDAEELYQTFQR	EF2	Elongation factor 2 (EF-2)	727.379	3	59.67	211967	59.47	377847	2.31563
AYLPVNESFGFTADLR	EF2	Elongation factor 2 (EF-2)	900.452	2	46.72	334189	46.49	1152789	4.48105
AYLPVNESFGFTADLR	EF2	Elongation factor 2 (EF-2)	600.637	3	46.72	103190	46.53	217303	2.73559
EGILADENLR	EF2	Elongation factor 2 (EF-2)	565.296	2	27.58	191418	27.42	395454	2.68371
GFLINLIDSPGHVDFSSEVTAALR	EF2	Elongation factor 2 (EF-2)	853.446	3	57.19	25314	56.52	168687	8.65652
GGGQIIPTTR	EF2	Elongation factor 2 (EF-2)	500.283	2	19.8	516572	19.79	630853	1.58643
LMEPVYLCEIQCPEVAVGGIYGVLN	EF2	Elongation factor 2 (EF-2)	993.831	3	57.43	170257	57	114365	0.87259
LMEPVYLCEIQCPEVAVGGIYGVLN	EF2	Elongation factor 2 (EF-2)	999.162	3	55.33	485439	54.81	219284	0.58681
NPADLPK	EF2	Elongation factor 2 (EF-2)	377.708	2	13.89	33881	13.79	44731	1.71504
STLTDSLVSK	EF2	Elongation factor 2 (EF-2)	525.788	2	24.62	115364	24.6	560125	6.30721
TGTITTFK	EF2	Elongation factor 2 (EF-2)	434.742	2	19.37	42040	19.33	38671	1.19494
VTDGALVVVDCVSGVCVQTETVLR	EF2	Elongation factor 2 (EF-2)	859.44	3	48.52	244266	48.19	220870	1.17462
YVEAIEDVPSGNICGLVGVDQFLVK	EF2	Elongation factor 2 (EF-2)	907.798	3	53.98	113966	53.65	365856	4.1702
ILPLDGLQAGYR	norpA	1-phosphatidylinositol 4,5-bisphosphate phosphodiesterase (EC 3.1.4.11) (1-p	658.372	2	39.05	1848340	38.89	1619786	1.13841
NDIEELFTSITK	norpA	1-phosphatidylinositol 4,5-bisphosphate phosphodiesterase (EC 3.1.4.11) (1-p	705.362	2	50.41	647218	50.1	519426	1.04255
VVLPDLAVLR	norpA	1-phosphatidylinositol 4,5-bisphosphate phosphodiesterase (EC 3.1.4.11) (1-p	547.85	2	45.88	627980	45.7	724721	1.49916
VAPVEAVTISSNR	mod	DNA-binding protein modulo	671.87	2	25.93	637081	25.91	601323	1.22613
EVSGDASEAALLK	Atpalpha	Sodium/potassium-transporting ATPase subunit alpha (Na(+)/K(+) ATPase alp	645.333	2	25.45	660865	25.35	279051	0.54852
IATLCNR	Atpalpha	Sodium/potassium-transporting ATPase subunit alpha (Na(+)/K(+) ATPase alp	424.226	2	14.34	183981	14.32	209877	1.48188
LIIVEGCQR	Atpalpha	Sodium/potassium-transporting ATPase subunit alpha (Na(+)/K(+) ATPase alp	544.3	2	24.31	677846	24.36	746923	1.43142
LNIPVSEVNPR	Atpalpha	Sodium/potassium-transporting ATPase subunit alpha (Na(+)/K(+) ATPase alp	619.349	2	30.64	1527141	30.51	1174989	0.99949
NLAFFSTNAVEGTAK	Atpalpha	Sodium/potassium-transporting ATPase subunit alpha (Na(+)/K(+) ATPase alp	785.399	2	38.55	1771624	38.01	15711079	11.5201
YHTEIVFAR	Atpalpha	Sodium/potassium-transporting ATPase subunit alpha (Na(+)/K(+) ATPase alp	568.298	2	25.19	792742	25.03	558537	0.91526
YHTEIVFAR	Atpalpha	Sodium/potassium-transporting ATPase subunit alpha (Na(+)/K(+) ATPase alp	379.201	3	25.23	368207	25.07	375193	1.32369
EIELYLFQER	ref(2)P	Protein ref(2)P (Refractory to sigma P)	670.348	2	45.17	146813	44.97	693000	6.13184
CNLTFVFSKPEEK	RpS27A	Ubiquitin-40S ribosomal protein S27a [Cleaved into: Ubiquitin; 40S ribosomal	533.601	3	33.88	499625	33.75	877919	2.28261
ESTLHLVLR	RpS27A	Ubiquitin-40S ribosomal protein S27a [Cleaved into: Ubiquitin; 40S ribosomal	534.314	2	29.06	19834334	28.96	12823394	0.83986
ESTLHLVLR	RpS27A	Ubiquitin-40S ribosomal protein S27a [Cleaved into: Ubiquitin; 40S ribosomal	356.545	3	29.02	13873703	28.96	8349692	0.78181
MQIFVK	RpS27A	Ubiquitin-40S ribosomal protein S27a [Cleaved into: Ubiquitin; 40S ribosomal	383.22	2	27.43	8697430	27.39	10348569	1.54565
TITLEVEPSDTIENVK	RpS27A	Ubiquitin-40S ribosomal protein S27a [Cleaved into: Ubiquitin; 40S ribosomal	894.467	2	36.85	41743372	36.64	47966256	1.49269
TITLEVEPSDTIENVK	RpS27A	Ubiquitin-40S ribosomal protein S27a [Cleaved into: Ubiquitin; 40S ribosomal	596.647	3	36.85	1563861	36.68	1963090	1.63066
TLSDYNIQK	RpS27A	Ubiquitin-40S ribosomal protein S27a [Cleaved into: Ubiquitin; 40S ribosomal	541.28	2	19.53	19914654	19.52	7860957	0.51277
AGIAVEGDIK	Arr1	Phosrestin-2 (Arrestin-1) (Arrestin-A) (Phosrestin II)	486.772	2	22.98	2931108	22.98	1880959	0.83362
ASDESQPCGVQYFVK	Arr1	Phosrestin-2 (Arrestin-1) (Arrestin-A) (Phosrestin II)	857.891	2	30.22	589087	30.08	894673	1.97291
DFLLSPGELELEVTLDK	Arr1	Phosrestin-2 (Arrestin-1) (Arrestin-A) (Phosrestin II)	959.506	2	56.3	265898	55.99	462292	2.25852
DFVDSVTQVEPIDGIIVLDDEYVR	Arr1	Phosrestin-2 (Arrestin-1) (Arrestin-A) (Phosrestin II)	1368.68	2	59.17	646283	58.87	419252	0.8427
DFVDSVTQVEPIDGIIVLDDEYVR	Arr1	Phosrestin-2 (Arrestin-1) (Arrestin-A) (Phosrestin II)	912.792	3	59.17	2018632	58.83	1330820	0.85642
IFTGDSDCDR	Arr1	Phosrestin-2 (Arrestin-1) (Arrestin-A) (Phosrestin II)	593.246	2	14.94	1443871	14.91	748462	0.67339
IFVQLVCNFR	Arr1	Phosrestin-2 (Arrestin-1) (Arrestin-A) (Phosrestin II)	648.35	2	42.99	1459871	42.8	1343715	1.19568
ISVNICVR	Arr1	Phosrestin-2 (Arrestin-1) (Arrestin-A) (Phosrestin II)	480.768	2	29.06	677107	28.92	402608	0.77241
QGIQPCTVVR	Arr1	Phosrestin-2 (Arrestin-1) (Arrestin-A) (Phosrestin II)	579.308	2	21.75	818570	21.73	482335	0.76545
STINLGIR	Arr1	Phosrestin-2 (Arrestin-1) (Arrestin-A) (Phosrestin II)	437.261	2	27.76	3970398	27.69	3553533	1.16265
VMYLVPTLVANCDR	Arr1	Phosrestin-2 (Arrestin-1) (Arrestin-A) (Phosrestin II)	825.921	2	44.34	873153	44.19	632809	0.94147
DSSLSSGALANILAIFK	Hn	Protein henna (EC 1.14.16.1) (EC 1.14.16.4) (Phe-4-monooxygenase) (Pheny	853.97	2	65.24	302707	65.18	21367	0.09169
FANSIPRPFGVR	Hn	Protein henna (EC 1.14.16.1) (EC 1.14.16.4) (Phe-4-monooxygenase) (Pheny	454.254	3	31.64	779696	31.6	818437	1.36359
DNYVPAVSALEQDIIEVDADTK	RpS17	40S ribosomal protein S17	1203.09	2	56.74	463234	56.49	231517	0.64924
GLQLTQPNTNNFGR	RpS17	40S ribosomal protein S17	780.4	2	29.1	12767330	29	13990296	1.42347
IAGYVTHLMGR	RpS17	40S ribosomal protein S17	609.327	2	29.32	3132626	29.21	1918585	0.7956
IAGYVTHLMGR	RpS17	40S ribosomal protein S17	406.553	3	29.32	3913691	29.24	2429526	0.80641

ICEEVAIIPTKPLR	RpS17	40S ribosomal protein S17	819.966	2	33.87	1546348	33.76	658387	0.55309
ICEEVAIIPTKPLR	RpS17	40S ribosomal protein S17	546.98	3	33.87	10439692	33.72	5906749	0.73499
LLDFHNIR	RpS17	40S ribosomal protein S17	514.288	2	30.03	8251491	29.93	7739377	1.21842
LTLDFHTNK	RpS17	40S ribosomal protein S17	363.529	3	26.47	1458287	26.42	1674230	1.4914
ESTLHLVLR	RpL40	Ubiquitin-60S ribosomal protein L40 (CEP52) [Cleaved into: Ubiquitin; 60S rib	534.314	2	29.06	19834334	28.96	12823394	0.83986
ESTLHLVLR	RpL40	Ubiquitin-60S ribosomal protein L40 (CEP52) [Cleaved into: Ubiquitin; 60S rib	356.545	3	29.02	13873703	28.96	8349692	0.78181
MQIFVK	RpL40	Ubiquitin-60S ribosomal protein L40 (CEP52) [Cleaved into: Ubiquitin; 60S rib	383.22	2	27.43	8697430	27.39	10348569	1.54565
TITLEVEPSDTIENVK	RpL40	Ubiquitin-60S ribosomal protein L40 (CEP52) [Cleaved into: Ubiquitin; 60S rib	894.467	2	36.85	41743372	36.64	47966256	1.49269
TITLEVEPSDTIENVK	RpL40	Ubiquitin-60S ribosomal protein L40 (CEP52) [Cleaved into: Ubiquitin; 60S rib	596.647	3	36.85	1563861	36.68	1963090	1.63066
TLSDYNIQK	RpL40	Ubiquitin-60S ribosomal protein L40 (CEP52) [Cleaved into: Ubiquitin; 60S rib	541.28	2	19.53	19914654	19.52	7860957	0.51277
AIETFHGTVQHVESR	ple	Tyrosine 3-monooxygenase (EC 1.14.16.2) (Protein Pale) (Tyrosine 3-hydroxy	570.957	3	18.83	39913	18.92	134503	4.37764
GNLLQLIR	ple	Tyrosine 3-monooxygenase (EC 1.14.16.2) (Protein Pale) (Tyrosine 3-hydroxy	463.793	2	37.15	0	41.79	173479	#DIV/0!
YGDPIPFIDYSDVEVK	ple	Tyrosine 3-monooxygenase (EC 1.14.16.2) (Protein Pale) (Tyrosine 3-hydroxy	928.951	2	47.5	0	47.3	113828	#DIV/0!
DFIDHIDYCDPVDGVIVVEPDYLK	Arr2	Phosrestin-1 (49 kDa arrestin-like protein) (Arrestin-2) (Arrestin-B) (Phosrestir	946.118	3	51.5	344194	51.28	451785	1.7051
GYYQDDDDNIVFEDFAK	Arr2	Phosrestin-1 (49 kDa arrestin-like protein) (Arrestin-2) (Arrestin-B) (Phosrestir	1027.44	2	42.84	1017665	42.59	957088	1.22171
HVAQLETK	Arr2	Phosrestin-1 (49 kDa arrestin-like protein) (Arrestin-2) (Arrestin-B) (Phosrestir	463.259	2	9.93	668361	10.05	276006	0.53645
LLQPAPGTIEK	Arr2	Phosrestin-1 (49 kDa arrestin-like protein) (Arrestin-2) (Arrestin-B) (Phosrestir	583.843	2	23.7	3216444	23.69	3105287	1.25415
STGDACGIVISYSVR	Arr2	Phosrestin-1 (49 kDa arrestin-like protein) (Arrestin-2) (Arrestin-B) (Phosrestir	792.888	2	33.08	1311769	32.98	1197629	1.18601
TFYLIPLAANNK	Arr2	Phosrestin-1 (49 kDa arrestin-like protein) (Arrestin-2) (Arrestin-B) (Phosrestir	682.882	2	45.25	741418	45.13	706529	1.23791
VFGQLATTYR	Arr2	Phosrestin-1 (49 kDa arrestin-like protein) (Arrestin-2) (Arrestin-B) (Phosrestir	578.311	2	28.69	3204435	28.59	3234217	1.31111
ALVDVLR	Rm62	ATP-dependent RNA helicase p62 (EC 3.6.4.13)	393.248	2	32.4	917820	32.25	781548	1.10617
EANQEINPALENLAR	Rm62	ATP-dependent RNA helicase p62 (EC 3.6.4.13)	841.429	2	32.48	1286441	32.37	785963	0.79366
ELAQQIQQVATEFGSSSYVR	Rm62	ATP-dependent RNA helicase p62 (EC 3.6.4.13)	747.71	3	48.34	417102	48.12	508191	1.58273
FGGGGGFGDR	Rm62	ATP-dependent RNA helicase p62 (EC 3.6.4.13)	463.709	2	17.67	571805	17.72	448173	1.01817
NTCVFGGAPK	Rm62	ATP-dependent RNA helicase p62 (EC 3.6.4.13)	525.755	2	19.91	235668	19.91	262752	1.44833
SNILVATDVAAR	Rm62	ATP-dependent RNA helicase p62 (EC 3.6.4.13)	615.346	2	29.95	1490640	29.85	1916326	1.67001
YVINFDYPQNSEDYIHR	Rm62	ATP-dependent RNA helicase p62 (EC 3.6.4.13)	725.004	3	37.84	686584	37.79	447734	0.84713
STFTVDITPLILAAHR	trp	Transient receptor potential protein	585.665	3	49.88	352438	49.67	830498	3.0611
AQELWELIVK	up	Troponin T, skeletal muscle (Protein intended thorax) (Protein upheld)	614.85	2	48.34	857786	48.15	594529	0.90036
DAGVLGLSSAAMER	up	Troponin T, skeletal muscle (Protein intended thorax) (Protein upheld)	696.843	2	30.26	1579477	30.2	1875543	1.54254
IKPLAIEGFGEAK	up	Troponin T, skeletal muscle (Protein intended thorax) (Protein upheld)	458.266	3	33.5	4523125	33.37	3961091	1.13762
ISLSFR	up	Troponin T, skeletal muscle (Protein intended thorax) (Protein upheld)	361.713	2	30.22	1860854	30.16	1791066	1.25032
ILEDSPNAR	Gs2	Glutamine synthetase 2 cytoplasmic (EC 6.3.1.2) (Glutamate--ammonia ligase	507.764	2	13.95	1451117	13.97	865640	0.77492
TLDFIPQSPK	Gs2	Glutamine synthetase 2 cytoplasmic (EC 6.3.1.2) (Glutamate--ammonia ligase	573.314	2	33.87	1671981	33.68	1493163	1.16011
YLSLPLQENIVQATYVWIDGTGEDL	Gs2	Glutamine synthetase 2 cytoplasmic (EC 6.3.1.2) (Glutamate--ammonia ligase	998.513	3	65.64	539196	65.12	224891	0.54181
IAFSPYGNITSAK	pAbp	Polyadenylate-binding protein (PABP) (Poly(A)-binding protein)	684.862	2	33.2	475743	33.09	1061132	2.89747
SLGYAYVNFQQPADAER	pAbp	Polyadenylate-binding protein (PABP) (Poly(A)-binding protein)	964.96	2	35.22	2374556	35.06	1626827	0.88998
IGTSEPIGR	Syt1	Synaptotagmin 1 (p65)	465.256	2	15.84	896749	15.87	539143	0.78101
TLSPVFNETFTFK	Syt1	Synaptotagmin 1 (p65)	765.896	2	45.21	1109199	44.93	1551467	1.817
AQYLQSVEDR	SdhB	Succinate dehydrogenase [ubiquinone] iron-sulfur subunit, mitochondrial (EC	604.799	2	21.3	641212	21.34	272433	0.55193
SGSGGSTGYVPTGRPR	D1	Chromosomal protein D1	512.59	3	14.38	493054	14.31	732667	1.93034
EFTLEFSR	SERCA	Calcium-transporting ATPase sarcoplasmic/endoplasmic reticulum type (EC 7	514.756	2	35.79	511756	35.66	541898	1.37555
GVVIGTGLSTAIGK	SERCA	Calcium-transporting ATPase sarcoplasmic/endoplasmic reticulum type (EC 7	636.88	2	34.79	1157677	34.58	1583799	1.77719
IDQSILTGESVSVIK	SERCA	Calcium-transporting ATPase sarcoplasmic/endoplasmic reticulum type (EC 7	794.943	2	37.8	1179386	37.6	1456916	1.60473
IGVFAEDEDTTGK	SERCA	Calcium-transporting ATPase sarcoplasmic/endoplasmic reticulum type (EC 7	691.328	2	25.89	1569911	25.87	1880132	1.55574
ITHIYSTTLR	SERCA	Calcium-transporting ATPase sarcoplasmic/endoplasmic reticulum type (EC 7	602.838	2	20.66	336218	20.64	473921	1.83108
ITHIYSTTLR	SERCA	Calcium-transporting ATPase sarcoplasmic/endoplasmic reticulum type (EC 7	402.228	3	20.66	1041150	20.64	1511216	1.88554
LNSFSVNK	SERCA	Calcium-transporting ATPase sarcoplasmic/endoplasmic reticulum type (EC 7	454.745	2	18.74	212026	18.69	338612	2.07461
NILFSGTNVAAGK	SERCA	Calcium-transporting ATPase sarcoplasmic/endoplasmic reticulum type (EC 7	646.354	2	32.18	877088	32.05	1429695	2.1175
TVEQSLNFFGTDPER	SERCA	Calcium-transporting ATPase sarcoplasmic/endoplasmic reticulum type (EC 7	870.415	2	39.92	733529	39.73	1152158	2.04041
AVTIFSPDGHLLQVEYAQEAVR	Prosalpha	Proteasome subunit alpha type-7-1 (EC 3.4.25.1) (PROS-Dm28.1) (Proteasor	815.092	3	44.54	28780	44.39	426811	19.2649
EAGPAASASEPEAK	Hsp67Bc	Heat shock protein 67B3 (Heat shock 18 kDa protein)	657.812	2	11.43	0	11.55	137626	#DIV/0!
NGELAALSR	Hsp67Bc	Heat shock protein 67B3 (Heat shock 18 kDa protein)	465.754	2	21.98	6343	21.77	75473	15.4568

RPATSGTGSVASSTAR	Map205	205 kDa microtubule-associated protein	753.387	2	9.41	74073	9.52	32583	0.57142
VFFDMTADNEPLGR	Cyp1	Peptidyl-prolyl cis-trans isomerase (PPIase) (EC 5.2.1.8) (Cyclophilin) (Cyclos	806.377	2	40.79	146730	40.66	612781	5.42511
VFFDMTADNEPLGR	Cyp1	Peptidyl-prolyl cis-trans isomerase (PPIase) (EC 5.2.1.8) (Cyclophilin) (Cyclos	814.375	2	34.05	24529	34.41	273040	14.46
VYSPHVLNLTLIDLPGLTK	shi	Dynamin (EC 3.6.5.5) (Protein shibire) (dDyn)	698.405	3	53.41	463587	53.25	477207	1.3372
SVTETGVELSNEER	14-3-3zet	14-3-3 protein zeta (14-3-3-like protein) (Protein Leonardo)	775.371	2	21.6	716717	21.65	1301291	2.35857
YLAEVATGDAR	14-3-3zet	14-3-3 protein zeta (14-3-3-like protein) (Protein Leonardo)	583.296	2	20.89	1053151	20.85	1412304	1.74205
LITPVVLQR	RpS6	40S ribosomal protein S6	519.837	2	34.36	2782314	34.22	2911970	1.35957
GQIHDIVLVGGSTR	Hsc70-1	Heat shock 70 kDa protein cognate 1 (Heat shock 70 kDa protein 70C)	726.402	2	27.66	141880	27.58	2013950	18.4395
GQIHDIVLVGGSTR	Hsc70-1	Heat shock 70 kDa protein cognate 1 (Heat shock 70 kDa protein 70C)	484.604	3	27.43	42940	27.54	4848927	146.692
SINPDEAVAYGAAVQAAILHGDK	Hsc70-1	Heat shock 70 kDa protein cognate 1 (Heat shock 70 kDa protein 70C)	770.729	3	49.12	5844145	48.87	6076260	1.35063
VEIIANDQGNR	Hsc70-1	Heat shock 70 kDa protein cognate 1 (Heat shock 70 kDa protein 70C)	614.818	2	18.24	20421124	18.27	30231408	1.9231
DLEAIVQPVIAK	Hsc70-3	Endoplasmic reticulum chaperone BiP (EC 3.6.4.10) (78 kDa glucose-regulate	648.382	2	41.32	544579	41.07	1292742	3.08371
ETAEAYLGK	Hsc70-3	Endoplasmic reticulum chaperone BiP (EC 3.6.4.10) (78 kDa glucose-regulate	491.248	2	18.78	1276400	18.74	1203743	1.22509
IEIESFFEGDDFSETLTR	Hsc70-3	Endoplasmic reticulum chaperone BiP (EC 3.6.4.10) (78 kDa glucose-regulate	1067.99	2	52.47	497399	52.17	1038290	2.71167
ITPSYVAFTADGER	Hsc70-3	Endoplasmic reticulum chaperone BiP (EC 3.6.4.10) (78 kDa glucose-regulate	763.878	2	32.4	751662	32.33	1189631	2.05595
IVITNDQNR	Hsc70-3	Endoplasmic reticulum chaperone BiP (EC 3.6.4.10) (78 kDa glucose-regulate	536.791	2	14.68	403680	14.64	383242	1.23327
NQLTTNPENTVFDAK	Hsc70-3	Endoplasmic reticulum chaperone BiP (EC 3.6.4.10) (78 kDa glucose-regulate	846.415	2	29.52	625548	29.44	1240840	2.57678
NSKPHISVDTSQGAK	Hsc70-3	Endoplasmic reticulum chaperone BiP (EC 3.6.4.10) (78 kDa glucose-regulate	523.606	3	12.41	133543	12.4	255862	2.4889
VEIIANDQGNR	Hsc70-3	Endoplasmic reticulum chaperone BiP (EC 3.6.4.10) (78 kDa glucose-regulate	614.818	2	18.24	20421124	18.27	30231408	1.9231
GSTGTLGNFAK	RpS2	40S ribosomal protein S2 (Protein strings of pearls)	526.772	2	21.67	1400654	21.69	1849774	1.71558
GTGIVSAPVPK	RpS2	40S ribosomal protein S2 (Protein strings of pearls)	513.303	2	23.48	3393633	23.45	3953923	1.51351
TYAYLTPDLWK	RpS2	40S ribosomal protein S2 (Protein strings of pearls)	685.853	2	43.29	689942	43.09	916755	1.72609
AVVQVFEGTSGIDAK	Vha55	V-type proton ATPase subunit B (V-ATPase subunit B) (V-ATPase 55 kDa sul	760.901	2	33.68	936310	33.53	864164	1.19894
DFISQPR	Vha55	V-type proton ATPase subunit B (V-ATPase subunit B) (V-ATPase 55 kDa sul	431.725	2	20.36	908159	20.31	646613	0.92492
FAEIVQLR	Vha55	V-type proton ATPase subunit B (V-ATPase subunit B) (V-ATPase 55 kDa sul	488.285	2	33.47	1109279	33.37	993806	1.16381
NFISQGNYENR	Vha55	V-type proton ATPase subunit B (V-ATPase subunit B) (V-ATPase 55 kDa sul	671.313	2	21.23	606233	21.27	286793	0.61454
NTLCEFTGDILR	Vha55	V-type proton ATPase subunit B (V-ATPase subunit B) (V-ATPase 55 kDa sul	719.853	2	41.77	1059567	41.56	793668	0.97304
TPVSEDMLGR	Vha55	V-type proton ATPase subunit B (V-ATPase subunit B) (V-ATPase 55 kDa sul	552.771	2	23.81	594688	23.84	576947	1.26029
GGGGGTGEAGFEVAK	128up	GTP-binding protein 128up	647.307	2	19.38	145884	19.38	205028	1.82569
APGIIPR	blw	ATP synthase subunit alpha, mitochondrial (Protein bellwether)	362.229	2	21.08	469561	21.05	342332	0.94706
AVDSLVPIGR	blw	ATP synthase subunit alpha, mitochondrial (Protein bellwether)	513.801	2	31.95	1671855	31.78	1623913	1.26179
GIRPAINVGLSVSR	blw	ATP synthase subunit alpha, mitochondrial (Protein bellwether)	480.288	3	31.51	1433876	31.42	1687483	1.5288
SAEISNILEER	blw	ATP synthase subunit alpha, mitochondrial (Protein bellwether)	630.825	2	33.08	2084754	32.98	1607500	1.00166
TALAIDTIINQK	blw	ATP synthase subunit alpha, mitochondrial (Protein bellwether)	650.877	2	38.45	1344625	38.31	1054061	1.01833
TGAIVDVPVGDELLGR	blw	ATP synthase subunit alpha, mitochondrial (Protein bellwether)	805.941	2	43.72	3128122	43.51	2122700	0.88151
TSEQALLDTIAK	blw	ATP synthase subunit alpha, mitochondrial (Protein bellwether)	645.351	2	35.43	1238241	35.22	1117479	1.17235
VLSIGDGIAR	blw	ATP synthase subunit alpha, mitochondrial (Protein bellwether)	500.793	2	29.32	2412863	29.21	2038012	1.09723
VVDALGNAIDGK	blw	ATP synthase subunit alpha, mitochondrial (Protein bellwether)	586.32	2	27.28	1696010	27.19	1089828	0.83474
HVVEQVHEEQER	Prm	Paramyosin, long form	506.915	3	9.49	191473	9.59	475223	3.22413
HVVEQVHEEQER	Prm	Paramyosin, short form (Miniparamyosin)	506.915	3	9.49	191473	9.59	475223	3.22413
AAEFNFR	wupA	Troponin I (Tn I) (Protein heldup) (Protein wings apart-A)	427.711	2	24.96	9042040	24.95	9905750	1.42313
GFMTPER	wupA	Troponin I (Tn I) (Protein heldup) (Protein wings apart-A)	419.2	2	19.67	1284815	19.67	1246317	1.26012
AIVAIDNPSDIFVISSRPIGQR	sta	40S ribosomal protein SA (K14) (Laminin receptor homolog) (Protein stubarist	790.104	3	47.14	1850721	46.91	2154167	1.51203
YTDTTPIAGR	sta	40S ribosomal protein SA (K14) (Laminin receptor homolog) (Protein stubarist	547.777	2	16.67	622037	16.64	752509	1.57151
ALQALEHAR	RpS19a	40S ribosomal protein S19a	504.783	2	16.22	955156	16.18	893690	1.21544
DIDQHAVTK	RpS19a	40S ribosomal protein S19a	513.764	2	10.4	2341073	10.53	719059	0.399
IANQIVFK	RpS19a	40S ribosomal protein S19a	466.782	2	26.32	23319114	26.31	14069064	0.78375
LSSIGQR	RpS19a	40S ribosomal protein S19a	380.719	2	11.74	2374571	11.84	1340591	0.73339
QTGPIVISK	RpS19a	40S ribosomal protein S19a	471.785	2	20.97	7892233	20.97	6770915	1.11447
SPAGVGSITK	RpS19a	40S ribosomal protein S19a	458.759	2	15.88	11010122	15.86	5559005	0.65588
DSQGHVFATR	RpS4	40S ribosomal protein S4	373.184	3	13.5	848798	13.47	498993	0.76368
ESLPLLIFLR	RpS4	40S ribosomal protein S4	600.871	2	59.87	978242	59.6	249332	0.3311
GNKPYISLPK	RpS4	40S ribosomal protein S4	372.885	3	24.24	1063638	24.28	968662	1.18304

GVPFLVTHDGR	RpS4	40S ribosomal protein S4	599.322	2	29.44	2161831	29.36	2769935	1.66445
LGGVFAPRPSTGPHK	RpS4	40S ribosomal protein S4	507.616	3	20.14	336675	20.13	222325	0.85783
LGGVFAPRPSTGPHK	RpS4	40S ribosomal protein S4	380.964	4	20.18	928142	20.13	480847	0.673
LSIAEER	RpS4	40S ribosomal protein S4	409.224	2	17.33	1731761	17.37	1338598	1.00412
LTNVFIIGK	RpS4	40S ribosomal protein S4	502.811	2	37.72	3811188	37.56	3884472	1.32402
TGEFFR	RpS4	40S ribosomal protein S4	378.687	2	22.32	2231185	22.28	2138263	1.24494
VGTVVNR	RpS4	40S ribosomal protein S4	372.722	2	10.66	487079	10.77	324263	0.86481
AAAPAAVASPAAAATSADASPSPAł	Pep	Zinc finger protein on ecdysone puffs	708.031	3	24.27	491341	24.2	1065912	2.81813
FFDTEVTAEIHSR	Pep	Zinc finger protein on ecdysone puffs	517.919	3	32.02	935298	31.86	1657140	2.30161
NQNPPSLLDLPR	Pep	Zinc finger protein on ecdysone puffs	682.37	2	37.75	928013	37.52	1703500	2.38457
HYWGLR	RpS18	40S ribosomal protein S18	416.217	2	21.34	2947345	21.35	3139903	1.38391
VPNWFLNR	RpS18	40S ribosomal protein S18	523.283	2	37.87	5287080	37.68	4566158	1.12191
YSNIVLK	RpS18	40S ribosomal protein S18	418.747	2	22.31	2725895	22.28	2149680	1.02444
LVTAIDEADLAR	eIF2alpha	Eukaryotic translation initiation factor 2 subunit 1 (Eukaryotic translation initiat	643.851	2	31.83	315858	31.67	469918	1.93265
AVFPSIVGRPR	Arp53D	Actin-like protein 53D	599.856	2	30.18	3527039	29.97	6233553	2.29587
AEEILER	RpL11	60S ribosomal protein L11	430.23	2	17.53	3556219	17.53	2139819	0.78165
IAVHCTVR	RpL11	60S ribosomal protein L11	478.261	2	12.42	1451582	12.46	1036746	0.9278
SGTVGFQHR	RpL11	60S ribosomal protein L11	494.752	2	11.45	1263829	11.54	829082	0.85218
VLEQLTGQQPVFSK	RpL11	60S ribosomal protein L11	787.433	2	33.08	9876999	32.94	10033931	1.31968
YDPSIGIYGLDFYVVLGRPGYNVNH	RpL11	60S ribosomal protein L11	746.882	4	53.64	810217	53.57	534216	0.85652
NFSGAELEGLVR	comt	Vesicle-fusing ATPase 1 (EC 3.6.4.6) (N-ethylmaleimide-sensitive fusion prote	646.336	2	38.11	673204	37.95	671954	1.29663
TVEVPSGYVGFSLVQR	comt	Vesicle-fusing ATPase 1 (EC 3.6.4.6) (N-ethylmaleimide-sensitive fusion prote	869.462	2	42.95	482529	43.06	1159918	3.12267
CGVISPR	RpS15Aa	40S ribosomal protein S15Aa	394.708	2	13.98	622408	13.82	550921	1.14984
FDVPINDIEK	RpS15Aa	40S ribosomal protein S15Aa	595.309	2	35.12	6034878	34.98	2750801	0.59212
HGYIGEFEIVDDHR	RpS15Aa	40S ribosomal protein S15Aa	562.934	3	31.11	2707218	31.08	907855	0.43563
MNVLADALK	RpS15Aa	40S ribosomal protein S15Aa	487.771	2	34.9	1815269	34.78	1716352	1.22825
QFGYVVLTTSGGIMDHEEAR	RpS15Aa	40S ribosomal protein S15Aa	737.356	3	39.87	1542845	39.68	2090883	1.76047
WTNNLLPSR	RpS15Aa	40S ribosomal protein S15Aa	550.796	2	30.38	2286379	30.28	1280460	0.72751
ECADLWPR	RpL23	60S ribosomal protein L23 (L17A)	523.74	2	28.3	1947834	28.17	1933021	1.28916
GSAITGPVAK	RpL23	60S ribosomal protein L23 (L17A)	450.761	2	15.45	462972	15.4	267152	0.74959
ISLGLPVGAVMNCADNTGAK	RpL23	60S ribosomal protein L23 (L17A)	994.501	2	44.45	1252962	44.18	868781	0.90073
LPAAGVGDMFVATVK	RpL23	60S ribosomal protein L23 (L17A)	738.4	2	42.18	1470091	42.02	283589	0.25059
LPAAGVGDMFVATVK	RpL23	60S ribosomal protein L23 (L17A)	746.397	2	34.67	839242	34.53	345373	0.53459
NLYVIAVHGIR	RpL23	60S ribosomal protein L23 (L17A)	627.869	2	34.45	2973190	34.35	2192327	0.95787
NLYVIAVHGIR	RpL23	60S ribosomal protein L23 (L17A)	418.915	3	34.45	3228524	34.42	2192238	0.88208
ALIELR	RpS25	40S ribosomal protein S25	357.729	2	28.69	15719181	28.59	8354136	0.69039
LITPSVVSER	RpS25	40S ribosomal protein S25	550.819	2	27.29	60807344	27.24	36641056	0.78277
LNNQVLFDK	RpS25	40S ribosomal protein S25	545.798	2	26.51	3604080	26.46	2295710	0.82746
QVVQHHSQVIYTR	RpS25	40S ribosomal protein S25	797.926	2	13.04	476611	12.99	214076	0.58348
QVVQHHSQVIYTR	RpS25	40S ribosomal protein S25	532.286	3	13.04	7237669	12.99	4618453	0.82893
LLLGGLGENPDR	Pu	GTP cyclohydrolase 1 (EC 3.5.4.16) (GTP cyclohydrolase I) (GTP-CH-I) (Prot	627.346	2	34.9	431350	34.7	325871	0.98138
TAVETAILLLR	CCT3	T-complex protein 1 subunit gamma (TCP-1-gamma) (CCT-gamma) (Chaperc	600.372	2	46.75	1365542	46.61	617422	0.58735
TLAQNCGANTIR	CCT3	T-complex protein 1 subunit gamma (TCP-1-gamma) (CCT-gamma) (Chaperc	659.83	2	15.65	496704	15.59	376804	0.98546
VFLGGLPSNVTETDLR	Hrb27C	Heterogeneous nuclear ribonucleoprotein 27C (Hrb27-C) (HRP48.1) (hnRNP	859.46	2	41.76	616996	41.61	670475	1.41164
LSEASQAADESER	Tm1	Tropomyosin-1, isoforms 33/34 (Tropomyosin II)	696.816	2	11.57	343010	11.67	695696	2.63472
AVYAHFPINCVTSENNTVIEIR	RpL9	60S ribosomal protein L9	849.76	3	40.81	1781795	40.62	1413499	1.03053
FLDGLYVSEK	RpL9	60S ribosomal protein L9	585.806	2	35.92	3763738	35.78	4648290	1.60434
GVTFGFQYK	RpL9	60S ribosomal protein L9	523.769	2	33.76	1916061	33.65	1180316	0.80022
TVCSHIENMIK	RpL9	60S ribosomal protein L9	666.326	2	25.86	3049601	26.46	614101	0.26159
VVTITGTR	RpL9	60S ribosomal protein L9	423.756	2	16.63	6749408	16.64	3559419	0.68507
VNNLGNNVTFER	RpL22	60S ribosomal protein L22	688.849	2	24.51	774262	24.48	737556	1.23745
AGFAGDDAPR	Act57B	Actin-57B	488.728	2	15.06	5346544	15.03	6072874	1.47551
AVFPSIVGRPR	Act57B	Actin-57B	599.856	2	30.18	3527039	29.97	6233553	2.29587

DSYVGDEAQSK	Act57B	Actin-57B	599.765	2	11.94	533801	12.02	513658	1.25002
EITSLAPSTIK	Act57B	Actin-57B	580.332	2	28.08	2654634	28	4111213	2.01181
IWHHTFYNELR	Act57B	Actin-57B	505.921	3	25.48	1669489	25.49	2792816	2.17311
SYELPDGQVITIGNER	Act57B	Actin-57B	895.95	2	39.17	5791673	39	8517636	1.91046
SYELPDGQVITIGNER	Act57B	Actin-57B	597.635	3	39.17	427938	38.97	800279	2.42931
TTGIVLDSGDGVSHTVPIYEGYALPI	Act57B	Actin-57B	1051.22	3	45.78	1749429	45.58	1016220	0.75459
VAPEEHPVLLTEAPLNPK	Act57B	Actin-57B	977.536	2	34.25	620752	34.11	748708	1.56681
VAPEEHPVLLTEAPLNPK	Act57B	Actin-57B	652.026	3	34.21	12031327	34.11	16999678	1.83548
SVYAAEER	Mlp60A	Muscle LIM protein 1	462.725	2	11.82	2588129	11.89	1417299	0.71137
NFSGAELEGLVR	Nsf2	Vesicle-fusing ATPase 2 (EC 3.6.4.6) (N-ethylmaleimide-sensitive fusion prote	646.336	2	38.11	673204	37.95	671954	1.29663
EAIVPFVPIFVEEADVIQGAK	eas	Ethanolamine kinase (EK) (EC 2.7.1.82) (Protein easily shocked)	757.747	3	63.84	23869	61.84	116025	6.31451
HPQLLYEAK	CkIalpha	Casein kinase I isoform alpha (CKI-alpha) (DmCK1) (EC 2.7.11.1)	549.801	2	20.1	592902	20.09	726533	1.59182
ILSGGVGFPR	CkIalpha	Casein kinase I isoform alpha (CKI-alpha) (DmCK1) (EC 2.7.11.1)	501.79	2	30.56	792616	30.4	1109698	1.81871
YASINAHLGIEQSR	CkIalpha	Casein kinase I isoform alpha (CKI-alpha) (DmCK1) (EC 2.7.11.1)	520.271	3	26.21	386181	26.15	468938	1.57742
DAGNYEMITGYR	Gdh	Glutamate dehydrogenase, mitochondrial (GDH) (EC 1.4.1.3)	695.309	2	29.45	100199	29.4	312114	4.04643
DAGNYEMITGYR	Gdh	Glutamate dehydrogenase, mitochondrial (GDH) (EC 1.4.1.3)	703.306	2	24.32	326038	24.28	360863	1.43779
TFIVQGFGNVGLHTTR	Gdh	Glutamate dehydrogenase, mitochondrial (GDH) (EC 1.4.1.3)	582.981	3	38.87	1874134	38.58	1474266	1.02187
DIEKPHVGDSASVHR	RpS20	40S ribosomal protein S20	549.613	3	14.1	1789285	14.02	1270258	0.92222
DIEKPHVGDSASVHR	RpS20	40S ribosomal protein S20	412.462	4	14.1	4796295	14.02	4014507	1.0873
IIDLHSPSEIVK	RpS20	40S ribosomal protein S20	675.885	2	30.68	1548028	30.56	1816547	1.52437
IIDLHSPSEIVK	RpS20	40S ribosomal protein S20	450.926	3	30.68	6406697	30.52	7870892	1.59592
ITSINIEPGVEVEVTIAN	RpS20	40S ribosomal protein S20	949.509	2	47.39	2652750	47.09	1755529	0.85967
SLENVCR	RpS20	40S ribosomal protein S20	439.213	2	13.35	4815301	13.38	2341853	0.63177
APNMFQTR	RpS3A	40S ribosomal protein S3a (C3 protein)	482.737	2	21.15	426234	21.11	434247	1.32346
LIAEDVQDR	RpS3A	40S ribosomal protein S3a (C3 protein)	529.777	2	18.13	939103	18.16	256307	0.35454
LLELHGDGGGK	RpS3A	40S ribosomal protein S3a (C3 protein)	548.293	2	18.13	889316	17.98	301503	0.44041
MTDIITNEVSGADLK	RpS3A	40S ribosomal protein S3a (C3 protein)	803.903	2	35.24	1723290	35.03	1709836	1.2889
SVEAVVSSEGAVIDRPEGYEPPVQI	RpS3A	40S ribosomal protein S3a (C3 protein)	938.466	3	40.14	2946991	39.92	2515191	1.1087
TVDGYLLR	RpS3A	40S ribosomal protein S3a (C3 protein)	468.761	2	27.97	1377497	27.93	1215401	1.14618
VFEVSLADLQK	RpS3A	40S ribosomal protein S3a (C3 protein)	624.845	2	40.52	2741802	40.31	2030446	0.96201
VVDPFSR	RpS3A	40S ribosomal protein S3a (C3 protein)	410.222	2	25.97	1581151	25.91	1298337	1.06669
WQTLIEAIVEAK	RpS3A	40S ribosomal protein S3a (C3 protein)	700.893	2	52.94	1141760	52.82	1124334	1.27921
LVAIVDVIDQNR	RpL14	60S ribosomal protein L14	677.888	2	42.05	2697914	41.86	1953720	0.94071
VLVDGPLTGVPR	RpL14	60S ribosomal protein L14	611.861	2	34.44	2925516	34.3	1598894	0.70997
IIGEYGLR	RpS9	40S ribosomal protein S9	460.764	2	25.97	1566590	25.94	2123615	1.76093
LFQGNALLR	RpS9	40S ribosomal protein S9	516.303	2	32.83	1000199	32.72	375882	0.48819
ALTVPELTQQMFDAK	betaTub8	Tubulin beta-2 chain (Beta-2-tubulin)	854.435	2	37.81	1590296	37.56	1317924	1.07655
GHYTEGAELVDSVLDVVR	betaTub8	Tubulin beta-2 chain (Beta-2-tubulin)	653.665	3	50.34	2775420	50.24	1100574	0.51513
LAVNMVPFPR	betaTub8	Tubulin beta-2 chain (Beta-2-tubulin)	572.321	2	40.41	1793279	40.19	932227	0.6753
LAVNMVPFPR	betaTub8	Tubulin beta-2 chain (Beta-2-tubulin)	580.318	2	34.29	768535	34.41	1250344	2.11343
LHFFMPGFAPLTSR	betaTub8	Tubulin beta-2 chain (Beta-2-tubulin)	540.95	3	48.83	2785496	48.88	1571753	0.733
LHFFMPGFAPLTSR	betaTub8	Tubulin beta-2 chain (Beta-2-tubulin)	546.282	3	42.62	1500055	42.47	820442	0.7105
YLTVAAIFR	betaTub8	Tubulin beta-2 chain (Beta-2-tubulin)	527.308	2	45.1	10561950	44.89	6760614	0.8315
DAGHIAGLNVLR	Hsp70Aa	Major heat shock 70 kDa protein Aa (Heat shock protein 70Aa) (HSP70-87A7	618.346	2	31.67	40614	31.54	13040718	417.108
DAGHIAGLNVLR	Hsp70Aa	Major heat shock 70 kDa protein Aa (Heat shock protein 70Aa) (HSP70-87A7	412.567	3	31.59	26572	31.54	33980956	1661.24
DNNALGTFDLSGIPPAPR	Hsp70Aa	Major heat shock 70 kDa protein Aa (Heat shock protein 70Aa) (HSP70-87A7	927.971	2	45.07	166587	43.39	30976200	241.551
DNNALGTFDLSGIPPAPR	Hsp70Aa	Major heat shock 70 kDa protein Aa (Heat shock protein 70Aa) (HSP70-87A7	618.983	3	45.21	24051	43.65	6711729	362.513
ETAEAYLGESITDAVITVPAYFNDSC	Hsp70Aa	Major heat shock 70 kDa protein Aa (Heat shock protein 70Aa) (HSP70-87A7	987.477	3	58.6	304104	58.36	1308713	5.59042
FAPEEISSMVLTK	Hsp70Aa	Major heat shock 70 kDa protein Aa (Heat shock protein 70Aa) (HSP70-87A7	726.376	2	41.77	22231	41.79	11132469	650.511
FAPEEISSMVLTK	Hsp70Aa	Major heat shock 70 kDa protein Aa (Heat shock protein 70Aa) (HSP70-87A7	484.587	3	41.77	0	41.79	276280	#DIV/0!
FAPEEISSMVLTK	Hsp70Aa	Major heat shock 70 kDa protein Aa (Heat shock protein 70Aa) (HSP70-87A7	734.374	2	35.12	297494	34.86	2047640	8.94124
FEELCADLFR	Hsp70Aa	Major heat shock 70 kDa protein Aa (Heat shock protein 70Aa) (HSP70-87A7	650.306	2	41.55	32123	41.36	4667637	188.757
GQIHDIVLVGGSTR	Hsp70Aa	Major heat shock 70 kDa protein Aa (Heat shock protein 70Aa) (HSP70-87A7	726.402	2	27.66	141880	27.58	2013950	18.4395

GQIHDIVLVGGSTR	Hsp70Aa	Major heat shock 70 kDa protein Aa (Heat shock protein 70Aa) (HSP70-87A7	484.604	3	27.43	42940	27.54	4848927	146.692
HCSPIMTK	Hsp70Aa	Major heat shock 70 kDa protein Aa (Heat shock protein 70Aa) (HSP70-87A7	487.233	2	11.2	35165	11.25	2786296	102.929
HCSPIMTK	Hsp70Aa	Major heat shock 70 kDa protein Aa (Heat shock protein 70Aa) (HSP70-87A7	495.231	2	9.26	11777	9.24	218205	24.0687
IQDVLLVDVAPLSLGIETAGGVMTK	Hsp70Aa	Major heat shock 70 kDa protein Aa (Heat shock protein 70Aa) (HSP70-87A7	1270.21	2	61.32	0	61.17	571901	#DIV/0!
IQDVLLVDVAPLSLGIETAGGVMTK	Hsp70Aa	Major heat shock 70 kDa protein Aa (Heat shock protein 70Aa) (HSP70-87A7	847.14	3	61.32	519	61.21	3335041	8347.49
IQDVLLVDVAPLSLGIETAGGVMTK	Hsp70Aa	Major heat shock 70 kDa protein Aa (Heat shock protein 70Aa) (HSP70-87A7	852.472	3	57.66	0	57.38	359486	#DIV/0!
LSQAEIDR	Hsp70Aa	Major heat shock 70 kDa protein Aa (Heat shock protein 70Aa) (HSP70-87A7	466.246	2	14.26	24538	14.28	4887219	258.729
NALESYVFNVK	Hsp70Aa	Major heat shock 70 kDa protein Aa (Heat shock protein 70Aa) (HSP70-87A7	642.335	2	40.6	35451	38.81	48323708	1770.74
NLNLSINPDEAVAYGAAVQAAILSGI	Hsp70Aa	Major heat shock 70 kDa protein Aa (Heat shock protein 70Aa) (HSP70-87A7	1493.76	2	58.87	44314	57.77	661805	19.4004
NLNLSINPDEAVAYGAAVQAAILSGI	Hsp70Aa	Major heat shock 70 kDa protein Aa (Heat shock protein 70Aa) (HSP70-87A7	996.175	3	58.34	121646	57.81	3315510	35.4058
NQVAMNPR	Hsp70Aa	Major heat shock 70 kDa protein Aa (Heat shock protein 70Aa) (HSP70-87A7	465.235	2	11.74	204889	11.84	365105	2.31484
STAGDTHLGGEDFDNR	Hsp70Aa	Major heat shock 70 kDa protein Aa (Heat shock protein 70Aa) (HSP70-87A7	846.366	2	18.67	1657789	18.65	1831600	1.43524
STAGDTHLGGEDFDNR	Hsp70Aa	Major heat shock 70 kDa protein Aa (Heat shock protein 70Aa) (HSP70-87A7	564.58	3	18.67	10781481	18.68	14815577	1.7851
TFSTYADNQPGVSIQVYEGER	Hsp70Aa	Major heat shock 70 kDa protein Aa (Heat shock protein 70Aa) (HSP70-87A7	787.705	3	34.9	43097	34.9	7443709	224.37
TLSSSTEATIEIDALFEGQDFYTK	Hsp70Aa	Major heat shock 70 kDa protein Aa (Heat shock protein 70Aa) (HSP70-87A7	1333.64	2	55.81	30514	54.36	4199024	178.76
TLSSSTEATIEIDALFEGQDFYTK	Hsp70Aa	Major heat shock 70 kDa protein Aa (Heat shock protein 70Aa) (HSP70-87A7	889.429	3	55.66	365012	54.36	14991373	53.3527
TTPSYVAFTDSER	Hsp70Aa	Major heat shock 70 kDa protein Aa (Heat shock protein 70Aa) (HSP70-87A7	737.346	2	28.48	159128	28.32	60403172	493.101
TTPSYVAFTDSER	Hsp70Aa	Major heat shock 70 kDa protein Aa (Heat shock protein 70Aa) (HSP70-87A7	491.9	3	28.44	0	28.4	323797	#DIV/0!
VEIIANDQGNR	Hsp70Aa	Major heat shock 70 kDa protein Aa (Heat shock protein 70Aa) (HSP70-87A7	614.818	2	18.24	20421124	18.27	30231408	1.9231
VQSLLQDFFHGK	Hsp70Aa	Major heat shock 70 kDa protein Aa (Heat shock protein 70Aa) (HSP70-87A7	709.875	2	45.25	30856	45.28	4190765	176.431
VQSLLQDFFHGK	Hsp70Aa	Major heat shock 70 kDa protein Aa (Heat shock protein 70Aa) (HSP70-87A7	473.586	3	45.21	0	45.24	14899044	#DIV/0!
AGFAGDDAPR	Act88F	Actin, indirect flight muscle (Actin-88F)	488.728	2	15.06	5346544	15.03	6072874	1.47551
AVFPSIVGRPR	Act88F	Actin, indirect flight muscle (Actin-88F)	599.856	2	30.18	3527039	29.97	6233553	2.29587
DSYVGDEAQSK	Act88F	Actin, indirect flight muscle (Actin-88F)	599.765	2	11.94	533801	12.02	513658	1.25002
IWHHTFYNELR	Act88F	Actin, indirect flight muscle (Actin-88F)	505.921	3	25.48	1669489	25.49	2792816	2.17311
SYELPDGQVITIGNER	Act88F	Actin, indirect flight muscle (Actin-88F)	895.95	2	39.17	5791673	39	8517636	1.91046
SYELPDGQVITIGNER	Act88F	Actin, indirect flight muscle (Actin-88F)	597.635	3	39.17	427938	38.97	800279	2.42931
VAPEEHPVLLTEAPLNPK	Act88F	Actin, indirect flight muscle (Actin-88F)	977.536	2	34.25	620752	34.11	748708	1.56681
VAPEEHPVLLTEAPLNPK	Act88F	Actin, indirect flight muscle (Actin-88F)	652.026	3	34.21	12031327	34.11	16999678	1.83548
DAVTYTEHAK	His4	Histone H4	567.775	2	11.73	1744246	11.83	635649	0.4734
DNIQGITKPAIR	His4	Histone H4	442.589	3	22.72	3459819	22.72	2375618	0.89196
ISGLIYEETR	His4	Histone H4	590.814	2	28.26	8240435	28.2	5262581	0.8296
VFLENVIR	His4	Histone H4	495.293	2	37.01	7324526	36.84	3166374	0.56157
AGLQFPVGR	His2A	Histone H2A	472.769	2	31.56	98633592	31.42	106838928	1.40711
HLQLAIR	His2A	Histone H2A	425.767	2	22.72	21451580	22.66	9817742	0.59453
LLSGVTIAQGGVLPNIQAVLLPK	His2A	Histone H2A	1151.2	2	58.27	5972554	58.05	3630529	0.78965
LLSGVTIAQGGVLPNIQAVLLPK	His2A	Histone H2A	767.802	3	58.3	10409686	58.05	8036129	1.00284
VGAGAPVYLAAVMEYLAAEVLELA(	His2A	Histone H2A	963.844	3	65.83	2552861	65.87	479648	0.24407
VGAGAPVYLAAVMEYLAAEVLELA(	His2A	Histone H2A	723.135	4	65.83	1259116	65.83	400141	0.41283
VGAGAPVYLAAVMEYLAAEVLELA(	His2A	Histone H2A	969.176	3	65.67	521759	65.67	545456	1.35804
FQSAAIGALQEASEAYLVGLFEDTN	His3.3A	Histone H3.3A	1146.57	3	65.53	385697	65.55	469966	1.58286
FQSAAIGALQEASEAYLVGLFEDTN	His3.3A	Histone H3.3A	860.182	4	65.53	371217	65.58	846592	2.96257
STELLIR	His3.3A	Histone H3.3A	416.25	2	25.89	5173880	25.9	6235095	1.56549
YRPGTVALR	His3.3A	Histone H3.3A	516.801	2	17.48	3862821	17.48	3541424	1.19096
ISGATTFDFIPQK	CaMKII	Calcium/calmodulin-dependent protein kinase type II alpha chain (CaM-kinase	712.875	2	38.6	1118955	38.42	1549927	1.79937
LVSQDNFGFDLAAVEAAAK	beta-Spec	Spectrin beta chain	983.499	2	48.76	272064	48.5	351415	1.67792
VAVVNQLAR	beta-Spec	Spectrin beta chain	485.296	2	22.75	392366	22.71	672076	2.2251
YATSQDESYR	beta-Spec	Spectrin beta chain	610.265	2	11.39	127721	11.46	145286	1.47769
AIYEQSR	hts	Protein hu-li tai shao (Adducin-like protein)	433.722	2	11.43	989317	11.54	662957	0.87051
DATVSQAGDHVVMMGAASK	hts	Protein hu-li tai shao (Adducin-like protein)	636.293	3	17.09	884028	17.06	1039090	1.5269
DGGAGASGILQQLSDIVGVPVSR	hts	Protein hu-li tai shao (Adducin-like protein)	1098.58	2	60.31	1852831	60.03	1591019	1.11548
DGGAGASGILQQLSDIVGVPVSR	hts	Protein hu-li tai shao (Adducin-like protein)	732.725	3	60.31	7576074	60.03	5964562	1.02272
FAAVAAAEDGAATAEK	hts	Protein hu-li tai shao (Adducin-like protein)	746.868	2	24.97	13124	25.1	319707	31.6452

LLDLYGWTQGLGAQITAR	hts	Protein hu-li tai shao (Adducin-like protein)	988.534	2	53.56	2279326	53.29	1565566	0.89225
LLDLYGWTQGLGAQITAR	hts	Protein hu-li tai shao (Adducin-like protein)	659.358	3	53.56	1975907	53.25	1298057	0.85339
LLPVGLDNLVLIPEESR	hts	Protein hu-li tai shao (Adducin-like protein)	626.363	3	55.71	938166	55.45	1069288	1.4806
MLDNAGYR	hts	Protein hu-li tai shao (Adducin-like protein)	470.221	2	16.11	947899	16.1	1485204	2.03538
NDVELPPAVSSLGYLLEEEELFR	hts	Protein hu-li tai shao (Adducin-like protein)	873.778	3	65.17	1366923	64.85	956167	0.90868
SHFVLHSVVHAARPDIR	hts	Protein hu-li tai shao (Adducin-like protein)	486.02	4	24.42	11051	23.97	0	0
SSNCMVPINDIR	hts	Protein hu-li tai shao (Adducin-like protein)	711.329	2	24.43	633585	24.05	1332102	2.73121
SVHGEYTDTDFSESEAVLQAGTK	hts	Protein hu-li tai shao (Adducin-like protein)	824.378	3	32.37	929683	32.29	1063232	1.48565
TGLLPLTK	hts	Protein hu-li tai shao (Adducin-like protein)	421.771	2	32.52	2862087	32.37	2725569	1.23708
TGYIYR	hts	Protein hu-li tai shao (Adducin-like protein)	386.703	2	16.34	1779020	16.29	796815	0.58183
VEVLETGTPDPK	hts	Protein hu-li tai shao (Adducin-like protein)	642.838	2	23.08	3487670	23.1	2329013	0.86748
VYSATISSGLDDSLDELDSLMSGLA	hts	Protein hu-li tai shao (Adducin-like protein)	797.385	4	54.66	1212756	54.41	1395124	1.49438
ILESGCHVVVGTPGR	eIF4A	Eukaryotic initiation factor 4A (EC 3.6.4.13) (ATP-dependent RNA helicase eI	527.612	3	22.54	32536	22.6	97954	3.91093
LGTLCDLYDTLSITQSVIFCNTR	eIF4A	Eukaryotic initiation factor 4A (EC 3.6.4.13) (ATP-dependent RNA helicase eI	897.444	3	65.14	15710	65.03	0	0
VLITTDLLAR	eIF4A	Eukaryotic initiation factor 4A (EC 3.6.4.13) (ATP-dependent RNA helicase eI	557.845	2	40.07	957169	39.92	1532706	2.08014
LQNLEDTNAGLNAR	LamC	Lamin-C (pG-IF)	764.889	2	23.51	123071	23.45	281522	2.97152
FLSQPFQVAEVFTGHAGK	ATPsynbe	ATP synthase subunit beta, mitochondrial (EC 7.1.2.2)	655.007	3	47.1	661096	46.95	927753	1.82301
LVLEVAQHLGENTVR	ATPsynbe	ATP synthase subunit beta, mitochondrial (EC 7.1.2.2)	839.468	2	34.87	556208	34.71	748475	1.74808
ACYGVLR	RpS3	40S ribosomal protein S3	419.716	2	20.14	3016355	20.13	2508723	1.08042
AELNEFLTR	RpS3	40S ribosomal protein S3	546.788	2	32.94	3256554	32.83	1378351	0.54982
ELAEDGYSGVEVR	RpS3	40S ribosomal protein S3	712.339	2	24.58	10406436	24.6	12489490	1.55907
FVDGLMIHSGDPCNDYVETATR	RpS3	40S ribosomal protein S3	838.374	3	33.19	433927	33.06	748456	2.24064
GCEVVVSGK	RpS3	40S ribosomal protein S3	467.737	2	13.15	569662	13.23	217126	0.49513
GLCAIAQAESLR	RpS3	40S ribosomal protein S3	644.838	2	32.06	4032273	31.93	2480082	0.79898
QGVLGIK	RpS3	40S ribosomal protein S3	357.729	2	22.9	1613732	22.87	2249317	1.81068
TEIIIMATK	RpS3	40S ribosomal protein S3	518.291	2	24	876921	24.27	939582	1.39186
VMLPYDPK	RpS3	40S ribosomal protein S3	481.754	2	28.33	612094	28.34	988565	2.09802
VMLPYDPK	RpS3	40S ribosomal protein S3	489.752	2	25.68	1125339	25.68	1698752	1.96096
AFIVKPIPSPEVIPVIVFINPK	rdgA	Eye-specific diacylglycerol kinase (DAG kinase 2) (DGK 2) (Diglyceride kinase	806.491	3	58.34	7861730	58.25	6537246	1.08019
AFIVKPIPSPEVIPVIVFINPK	rdgA	Eye-specific diacylglycerol kinase (DAG kinase 2) (DGK 2) (Diglyceride kinase	605.12	4	58.3	339552	58.29	268976	1.02903
ALGWGGGYTDEPIGK	rdgA	Eye-specific diacylglycerol kinase (DAG kinase 2) (DGK 2) (Diglyceride kinase	760.873	2	33.66	1454790	33.49	1166978	1.04204
ALHEQGYSLQSVNK	rdgA	Eye-specific diacylglycerol kinase (DAG kinase 2) (DGK 2) (Diglyceride kinase	525.27	3	20.1	844926	20.13	448176	0.68905
FEESISYPK	rdgA	Eye-specific diacylglycerol kinase (DAG kinase 2) (DGK 2) (Diglyceride kinase	550.269	2	25.08	699628	25.06	249796	0.46381
FQHLLNPR	rdgA	Eye-specific diacylglycerol kinase (DAG kinase 2) (DGK 2) (Diglyceride kinase	512.788	2	19.99	2182798	19.93	880908	0.52425
GDVQVNPLEK	rdgA	Eye-specific diacylglycerol kinase (DAG kinase 2) (DGK 2) (Diglyceride kinase	549.793	2	22.75	2434720	22.76	798326	0.42595
GPGSTDEDLQIGSKPIK	rdgA	Eye-specific diacylglycerol kinase (DAG kinase 2) (DGK 2) (Diglyceride kinase	581.304	3	23.2	1491415	23.18	425538	0.37065
LGNYAPIIVPPSWIVK	rdgA	Eye-specific diacylglycerol kinase (DAG kinase 2) (DGK 2) (Diglyceride kinase	884.014	2	52.63	3322956	52.44	1330008	0.51994
QVFDLTQGGPK	rdgA	Eye-specific diacylglycerol kinase (DAG kinase 2) (DGK 2) (Diglyceride kinase	595.314	2	28.22	3580063	28.2	2623698	0.95202
SFAAFSQR	rdgA	Eye-specific diacylglycerol kinase (DAG kinase 2) (DGK 2) (Diglyceride kinase	457.23	2	23.28	6655876	23.37	6106585	1.19183
TIPMQVDGEACR	rdgA	Eye-specific diacylglycerol kinase (DAG kinase 2) (DGK 2) (Diglyceride kinase	688.818	2	23.21	1029594	23.29	655361	0.82687
TIPMQVDGEACR	rdgA	Eye-specific diacylglycerol kinase (DAG kinase 2) (DGK 2) (Diglyceride kinase	696.816	2	17.7	628084	17.41	337537	0.69811
VKPSVIEIELLNK	rdgA	Eye-specific diacylglycerol kinase (DAG kinase 2) (DGK 2) (Diglyceride kinase	741.451	2	38.07	1106361	37.96	617198	0.72469
VKPSVIEIELLNK	rdgA	Eye-specific diacylglycerol kinase (DAG kinase 2) (DGK 2) (Diglyceride kinase	494.636	3	38.07	3645006	37.92	2591446	0.92356
VSQDWCFIDSCTAEHYFR	rdgA	Eye-specific diacylglycerol kinase (DAG kinase 2) (DGK 2) (Diglyceride kinase	774.33	3	41.88	500902	41.72	464633	1.20498
VTPNDDVTDDHVDR	rdgA	Eye-specific diacylglycerol kinase (DAG kinase 2) (DGK 2) (Diglyceride kinase	533.241	3	14.64	1483070	14.56	900190	0.78849
YIIASATR	rdgA	Eye-specific diacylglycerol kinase (DAG kinase 2) (DGK 2) (Diglyceride kinase	447.756	2	19.6	1081315	19.55	314198	0.37746
ILTGVVR	RpS11	40S ribosomal protein S11	379.25	2	21.96	1109408	21.94	1280841	1.49978
IQVNPNHEIIKPTTTR	DnaJ-1	DnaJ protein homolog 1 (DROJ1)	621.015	3	20.92	19679	20.89	261697	17.275
SLTIDNLDFLYEK	Stat92E	Signal transducer and transcription activator (d-STAT) (Protein marelle)	785.904	2	48.67	137787	48.23	187413	1.76691
FLINQR	RpL29	60S ribosomal protein L29 (L43)	395.732	2	22.84	98765624	22.79	117947512	1.55133
HESTLGMDVK	RpL29	60S ribosomal protein L29 (L43)	558.771	2	17.44	8388695	17.45	10347835	1.60242
HESTLGMDVK	RpL29	60S ribosomal protein L29 (L43)	566.769	2	11.21	6807770	11.36	4749146	0.90622
AQCPIVER	RpS5a	40S ribosomal protein S5a	486.75	2	14.71	806831	14.72	445526	0.71732

QAVDVSPLR	RpS5a	40S ribosomal protein S5a	492.777	2	23.02	630384	23.02	403036	0.83054
TIAECLADELINAAK	RpS5a	40S ribosomal protein S5a	816.419	2	52.59	3303911	52.37	1805891	0.71004
TIAECLADELINAAK	RpS5a	40S ribosomal protein S5a	544.615	3	52.55	1853788	52.37	881679	0.61784
WSCDDVTVNDISLQDYISVK	RpS5a	40S ribosomal protein S5a	1179.05	2	47.03	482748	46.74	433432	1.16633
LVPVAGLVDSFQK	ATPsynD	ATP synthase subunit d, mitochondrial (ATPase subunit d)	686.895	2	44.34	1462212	44.11	1311560	1.1652
CGGAVFAAEQQLSK	Mlp84B	Muscle LIM protein Mlp84B	733.359	2	29.7	610036	29.56	832171	1.77206
GFGYGHAPTLVSTSGESTIQFPDGI	Mlp84B	Muscle LIM protein Mlp84B	786.899	4	39.36	1207154	39.16	1556105	1.67455
GVGYGLGAGALTTF	Mlp84B	Muscle LIM protein Mlp84B	642.335	2	47.28	1954521	47.06	2621412	1.74228
GYGYGQGGGALQSDCYAHDDGAF	Mlp84B	Muscle LIM protein Mlp84B	905.061	3	26.86	601871	26.77	882779	1.90533
LAGGYVFHK	Mlp84B	Muscle LIM protein Mlp84B	496.272	2	20.86	564185	20.81	510898	1.17635
SVYAAEER	Mlp84B	Muscle LIM protein Mlp84B	462.725	2	11.82	2588129	11.89	1417299	0.71137
HSITSGGSGGGGFR	Rsf1	RNA-binding protein Rsf1 (RNA-binding protein Rox21)	638.805	2	12.65	1063621	12.67	1034087	1.26297
HSITSGGSGGGGFR	Rsf1	RNA-binding protein Rsf1 (RNA-binding protein Rox21)	426.206	3	12.65	1459305	12.67	1825302	1.62484
VYVGNLTDK	Rsf1	RNA-binding protein Rsf1 (RNA-binding protein Rox21)	504.772	2	21.41	1430452	21.39	1257685	1.14214
YSSGSSASYGR	Rsf1	RNA-binding protein Rsf1 (RNA-binding protein Rox21)	561.247	2	9.59	527731	9.71	255024	0.62776
ALTVPELTQQMFDAK	betaTub5	Tubulin beta-1 chain (Beta-1-tubulin)	854.435	2	37.81	1590296	37.56	1317924	1.07655
AVLVDLEPGTMDSVR	betaTub5	Tubulin beta-1 chain (Beta-1-tubulin)	801.414	2	39.24	2023515	39.04	2057238	1.32069
AVLVDLEPGTMDSVR	betaTub5	Tubulin beta-1 chain (Beta-1-tubulin)	809.411	2	34.48	5552510	34.3	4552577	1.0651
EAESCDCLQGFQLTHSLGGGTGS(	betaTub5	Tubulin beta-1 chain (Beta-1-tubulin)	1109.85	3	41.84	370508	41.64	177516	0.62239
GHYTEGAELVDSVLDVVR	betaTub5	Tubulin beta-1 chain (Beta-1-tubulin)	653.665	3	50.34	2775420	50.24	1100574	0.51513
IMNTYSVVPSPK	betaTub5	Tubulin beta-1 chain (Beta-1-tubulin)	668.352	2	29.06	1095355	28.94	858780	1.01847
INVYYNEASGGK	betaTub5	Tubulin beta-1 chain (Beta-1-tubulin)	657.82	2	21.12	4330833	21.13	2405958	0.72167
LAVNMVPFPR	betaTub5	Tubulin beta-1 chain (Beta-1-tubulin)	572.321	2	40.41	1793279	40.19	932227	0.6753
LAVNMVPFPR	betaTub5	Tubulin beta-1 chain (Beta-1-tubulin)	580.318	2	34.29	768535	34.41	1250344	2.11343
LHFFMPGFAPLTSR	betaTub5	Tubulin beta-1 chain (Beta-1-tubulin)	540.95	3	48.83	2785496	48.88	1571753	0.733
LHFFMPGFAPLTSR	betaTub5	Tubulin beta-1 chain (Beta-1-tubulin)	546.282	3	42.62	1500055	42.47	820442	0.7105
LTTPTYGDLNHLVSLTMSGVTTCLF	betaTub5	Tubulin beta-1 chain (Beta-1-tubulin)	917.467	3	55.9	882815	55.69	373168	0.54911
NSSYFVEWIPNNVK	betaTub5	Tubulin beta-1 chain (Beta-1-tubulin)	848.92	2	44.43	6003407	44.1	632379	0.13684
SGPFGQIFRPDNFVFGQSGAGNNV	betaTub5	Tubulin beta-1 chain (Beta-1-tubulin)	933.453	3	49.4	1384897	49.2	571844	0.53639
YLTVAAIFR	betaTub5	Tubulin beta-1 chain (Beta-1-tubulin)	527.308	2	45.1	10561950	44.89	6760614	0.8315
HPSKPEPSGECQPDLR	Caf1	Probable histone-binding protein Caf1 (Chromatin assembly factor 1 p55 subu	611.956	3	13	2959819	12.95	2946280	1.2931
IEIEIK	Caf1	Probable histone-binding protein Caf1 (Chromatin assembly factor 1 p55 subu	372.729	2	26.69	18490798	26.63	10128032	0.71153
IGEEQSTEDAEDGPPELLFIHGGHT	Caf1	Probable histone-binding protein Caf1 (Chromatin assembly factor 1 p55 subu	959.789	3	36.67	5605498	36.49	6740038	1.56196
IGEEQSTEDAEDGPPELLFIHGGHT	Caf1	Probable histone-binding protein Caf1 (Chromatin assembly factor 1 p55 subu	720.094	4	36.67	22180360	36.49	27435578	1.60682
INHEGEVNR	Caf1	Probable histone-binding protein Caf1 (Chromatin assembly factor 1 p55 subu	534.265	2	7.93	6273340	8.06	3016533	0.62464
INHEGEVNR	Caf1	Probable histone-binding protein Caf1 (Chromatin assembly factor 1 p55 subu	356.512	3	7.93	5037983	8.06	2395439	0.61766
LHVWDLSK	Caf1	Probable histone-binding protein Caf1 (Chromatin assembly factor 1 p55 subu	499.277	2	29.44	14247125	29.32	17649806	1.60929
LMIWDTR	Caf1	Probable histone-binding protein Caf1 (Chromatin assembly factor 1 p55 subu	467.744	2	35.19	7330851	35.02	7635260	1.35298
NIFTGHTAVVEDVAWHLLHESLFGS	Caf1	Probable histone-binding protein Caf1 (Chromatin assembly factor 1 p55 subu	1145.9	3	62.65	358249	62.54	531197	1.92616
NIFTGHTAVVEDVAWHLLHESLFGS	Caf1	Probable histone-binding protein Caf1 (Chromatin assembly factor 1 p55 subu	859.68	4	62.68	6111397	62.65	6672885	1.41839
SDNAAESFDDAVEER	Caf1	Probable histone-binding protein Caf1 (Chromatin assembly factor 1 p55 subu	827.845	2	26.73	15509184	26.66	5784847	0.48454
TVALWDLR	Caf1	Probable histone-binding protein Caf1 (Chromatin assembly factor 1 p55 subu	487.277	2	39.87	45980548	39.69	47007576	1.32805
VINEEYK	Caf1	Probable histone-binding protein Caf1 (Chromatin assembly factor 1 p55 subu	447.732	2	12.61	10431289	12.66	5073242	0.63179
YMPQNACVIATK	Caf1	Probable histone-binding protein Caf1 (Chromatin assembly factor 1 p55 subu	698.342	2	23.89	7995023	23.84	9614787	1.56222
YMPQNACVIATK	Caf1	Probable histone-binding protein Caf1 (Chromatin assembly factor 1 p55 subu	706.339	2	20.03	1455765	20.05	1599258	1.42708
AAYFGFYDTAR	sesB	ADP,ATP carrier protein (ADP/ATP translocase) (Adenine nucleotide transloc	641.299	2	36.23	8457992	36.06	4781556	0.73439
DFAAGGISAAVSK	sesB	ADP,ATP carrier protein (ADP/ATP translocase) (Adenine nucleotide transloc	597.312	2	28.61	8645959	28.51	9889000	1.4858
GFGVSVQGIIIYR	sesB	ADP,ATP carrier protein (ADP/ATP translocase) (Adenine nucleotide transloc	704.901	2	45.35	4833482	45.12	2059585	0.55353
SDGIVGLYR	sesB	ADP,ATP carrier protein (ADP/ATP translocase) (Adenine nucleotide transloc	490.264	2	28.91	1147390	28.83	342684	0.38798
TAVAPIER	sesB	ADP,ATP carrier protein (ADP/ATP translocase) (Adenine nucleotide transloc	428.748	2	16.27	5420824	16.21	2687378	0.644
TALTHYLEITAIPR	Cpr	NADPH--cytochrome P450 reductase (CPR) (P450R) (EC 1.6.2.4)	533.635	3	40.81	57301	40.62	150767	3.41796
EVDGSGTIASTNR	CG8635	Zinc finger CCCH domain-containing protein 15 homolog (DRG family regulat	653.815	2	13.51	1132231	13.47	535526	0.61442
FFLEAVEK	CG8635	Zinc finger CCCH domain-containing protein 15 homolog (DRG family regulat	491.766	2	36.01	903927	35.86	657062	0.94427

FIQQVQK	CG8635	Zinc finger CCCH domain-containing protein 15 homolog (DRG family regulat	445.758	2	14.57	1436865	14.52	689451	0.62332
LLDQATEAAK	CG8635	Zinc finger CCCH domain-containing protein 15 homolog (DRG family regulat	530.288	2	17.48	1942370	17.48	2050056	1.37106
VTLESFLAWK	CG8635	Zinc finger CCCH domain-containing protein 15 homolog (DRG family regulat	597.332	2	50.62	626652	50.28	644776	1.33661
CGVISPR	RpS15Ab	40S ribosomal protein S15Ab	394.708	2	13.98	622408	13.82	550921	1.14984
MNVLADALK	RpS15Ab	40S ribosomal protein S15Ab	487.771	2	34.9	1815269	34.78	1716352	1.22825
QFGYVVLTTSGGIMDHEEAR	RpS15Ab	40S ribosomal protein S15Ab	737.356	3	39.87	1542845	39.68	2090883	1.76047
WTNNLLPSR	RpS15Ab	40S ribosomal protein S15Ab	550.796	2	30.38	2286379	30.28	1280460	0.72751
LAMQVVLHTELER	TrpA1	Transient receptor potential cation channel subfamily A member 1 (dTRPA1) (	518.948	3	27.31	103016	27.24	1044228	13.1678
MLEQQHHLVR	TrpA1	Transient receptor potential cation channel subfamily A member 1 (dTRPA1) (	430.896	3	13.31	104134	13.27	657559	8.20284
NVIDIQQGGEHGR	TrpA1	Transient receptor potential cation channel subfamily A member 1 (dTRPA1) (	474.908	3	18.12	345514	18.16	3305973	12.4296
SPLLLAASR	TrpA1	Transient receptor potential cation channel subfamily A member 1 (dTRPA1) (	464.285	2	29.1	430144	29.02	1188200	3.58838
DAGHIAGLNVLR	Hsp70Ba	Major heat shock 70 kDa protein Ba (Heat shock protein 70Ba) (HSP70-87C1	618.346	2	31.67	40614	31.54	13040718	417.108
DAGHIAGLNVLR	Hsp70Ba	Major heat shock 70 kDa protein Ba (Heat shock protein 70Ba) (HSP70-87C1	412.567	3	31.59	26572	31.54	33980956	1661.24
DNNALGTFDLSGIPPAPR	Hsp70Ba	Major heat shock 70 kDa protein Ba (Heat shock protein 70Ba) (HSP70-87C1	927.971	2	45.07	166587	43.39	30976200	241.551
DNNALGTFDLSGIPPAPR	Hsp70Ba	Major heat shock 70 kDa protein Ba (Heat shock protein 70Ba) (HSP70-87C1	618.983	3	45.21	24051	43.65	6711729	362.513
ETAEAYLGESITDAVITVPAYFNDSC	Hsp70Ba	Major heat shock 70 kDa protein Ba (Heat shock protein 70Ba) (HSP70-87C1	987.477	3	58.6	304104	58.36	1308713	5.59042
FAPEEISSMVLTK	Hsp70Ba	Major heat shock 70 kDa protein Ba (Heat shock protein 70Ba) (HSP70-87C1	726.376	2	41.77	22231	41.79	11132469	650.511
FAPEEISSMVLTK	Hsp70Ba	Major heat shock 70 kDa protein Ba (Heat shock protein 70Ba) (HSP70-87C1	484.587	3	41.77	0	41.79	276280	#DIV/0!
FAPEEISSMVLTK	Hsp70Ba	Major heat shock 70 kDa protein Ba (Heat shock protein 70Ba) (HSP70-87C1	734.374	2	35.12	297494	34.86	2047640	8.94124
FEELCADLFR	Hsp70Ba	Major heat shock 70 kDa protein Ba (Heat shock protein 70Ba) (HSP70-87C1	650.306	2	41.55	32123	41.36	4667637	188.757
GQIHDIVLVGGSTR	Hsp70Ba	Major heat shock 70 kDa protein Ba (Heat shock protein 70Ba) (HSP70-87C1	726.402	2	27.66	141880	27.58	2013950	18.4395
GQIHDIVLVGGSTR	Hsp70Ba	Major heat shock 70 kDa protein Ba (Heat shock protein 70Ba) (HSP70-87C1	484.604	3	27.43	42940	27.54	4848927	146.692
HCSPIMTK	Hsp70Ba	Major heat shock 70 kDa protein Ba (Heat shock protein 70Ba) (HSP70-87C1	487.233	2	11.2	35165	11.25	2786296	102.929
HCSPIMTK	Hsp70Ba	Major heat shock 70 kDa protein Ba (Heat shock protein 70Ba) (HSP70-87C1	495.231	2	9.26	11777	9.24	218205	24.0687
IQDVLLVDVAPLSLGIETAGGVMTK	Hsp70Ba	Major heat shock 70 kDa protein Ba (Heat shock protein 70Ba) (HSP70-87C1	1270.21	2	61.32	0	61.17	571901	#DIV/0!
IQDVLLVDVAPLSLGIETAGGVMTK	Hsp70Ba	Major heat shock 70 kDa protein Ba (Heat shock protein 70Ba) (HSP70-87C1	847.14	3	61.32	519	61.21	3335041	8347.49
IQDVLLVDVAPLSLGIETAGGVMTK	Hsp70Ba	Major heat shock 70 kDa protein Ba (Heat shock protein 70Ba) (HSP70-87C1	852.472	3	57.66	0	57.38	359486	#DIV/0!
LSQAEIDR	Hsp70Ba	Major heat shock 70 kDa protein Ba (Heat shock protein 70Ba) (HSP70-87C1	466.246	2	14.26	24538	14.28	4887219	258.729
LVTHLAEEFK	Hsp70Ba	Major heat shock 70 kDa protein Ba (Heat shock protein 70Ba) (HSP70-87C1	593.827	2	25.94	371859	26.5	486802	1.70058
NALESYVFNVK	Hsp70Ba	Major heat shock 70 kDa protein Ba (Heat shock protein 70Ba) (HSP70-87C1	642.335	2	40.6	35451	38.81	48323708	1770.74
NLNLSINPDEAVAYGAAVQAAILSGI	Hsp70Ba	Major heat shock 70 kDa protein Ba (Heat shock protein 70Ba) (HSP70-87C1	1493.76	2	58.87	44314	57.77	661805	19.4004
NLNLSINPDEAVAYGAAVQAAILSGI	Hsp70Ba	Major heat shock 70 kDa protein Ba (Heat shock protein 70Ba) (HSP70-87C1	996.175	3	58.34	121646	57.81	3315510	35.4058
NQVAMNPR	Hsp70Ba	Major heat shock 70 kDa protein Ba (Heat shock protein 70Ba) (HSP70-87C1	465.235	2	11.74	204889	11.84	365105	2.31484
STAGDTHLGGEDFDNR	Hsp70Ba	Major heat shock 70 kDa protein Ba (Heat shock protein 70Ba) (HSP70-87C1	846.366	2	18.67	1657789	18.65	1831600	1.43524
STAGDTHLGGEDFDNR	Hsp70Ba	Major heat shock 70 kDa protein Ba (Heat shock protein 70Ba) (HSP70-87C1	564.58	3	18.67	10781481	18.68	14815577	1.7851
TFSTYSDNQPGVSIQVYEGER	Hsp70Ba	Major heat shock 70 kDa protein Ba (Heat shock protein 70Ba) (HSP70-87C1	793.036	3	34.64	0	34.11	7628120	#DIV/0!
TLSSSTEATIEIDALFEGQDFYTK	Hsp70Ba	Major heat shock 70 kDa protein Ba (Heat shock protein 70Ba) (HSP70-87C1	1333.64	2	55.81	30514	54.36	4199024	178.76
TLSSSTEATIEIDALFEGQDFYTK	Hsp70Ba	Major heat shock 70 kDa protein Ba (Heat shock protein 70Ba) (HSP70-87C1	889.429	3	55.66	365012	54.36	14991373	53.3527
TTPSYVAFTDSER	Hsp70Ba	Major heat shock 70 kDa protein Ba (Heat shock protein 70Ba) (HSP70-87C1	737.346	2	28.48	159128	28.32	60403172	493.101
TTPSYVAFTDSER	Hsp70Ba	Major heat shock 70 kDa protein Ba (Heat shock protein 70Ba) (HSP70-87C1	491.9	3	28.44	0	28.4	323797	#DIV/0!
VEIIANDQGNR	Hsp70Ba	Major heat shock 70 kDa protein Ba (Heat shock protein 70Ba) (HSP70-87C1	614.818	2	18.24	20421124	18.27	30231408	1.9231
VQSLLQEFFHGK	Hsp70Ba	Major heat shock 70 kDa protein Ba (Heat shock protein 70Ba) (HSP70-87C1	478.258	3	46.44	17592	46.3	17550354	1295.96
IADVVYNASNNELVR	RpS8	40S ribosomal protein S8	838.934	2	31.29	2587695	31.12	2542053	1.27613
IADVVYNASNNELVR	RpS8	40S ribosomal protein S8	559.625	3	31.29	1034232	31.16	1022478	1.28428
ILACISSRPGQCGR	RpS8	40S ribosomal protein S8	787.898	2	20.14	140708	20.13	87291	0.80588
ILACISSRPGQCGR	RpS8	40S ribosomal protein S8	525.601	3	20.1	386803	20.05	432135	1.45128
LETGNFAWASEGVAR	RpS8	40S ribosomal protein S8	804.394	2	35.85	1612600	35.66	1025388	0.82601
NSIVVIDATPFR	RpS8	40S ribosomal protein S8	666.37	2	40.3	3907103	40.11	3186895	1.05958
QWYEAHYVLPLGR	RpS8	40S ribosomal protein S8	544.616	3	39.06	708914	38.88	864527	1.58419
SDGYILEGK	RpS8	40S ribosomal protein S8	491.248	2	21.95	526219	21.94	571255	1.41022
VEQALEDQFTSGR	RpS8	40S ribosomal protein S8	740.357	2	25.93	843576	25.86	462117	0.71162
DGSLNYIEENDEVLVAGFGR	RpS23	40S ribosomal protein S23	1099.02	2	48.42	759053	48.15	930050	1.59168
DGSLNYIEENDEVLVAGFGR	RpS23	40S ribosomal protein S23	733.018	3	48.42	744083	48.11	1097192	1.91551

ITAFVPR	RpS23	40S ribosomal protein S23	402.242	2	24.96	1909159	24.94	2341284	1.59307
VANVSLLALYK	RpS23	40S ribosomal protein S23	595.861	2	41.4	2813053	41.22	3213274	1.48386
ATADEMTK	HDAC1	Histone deacetylase HDAC1 (HD) (EC 3.5.1.98)	433.7	2	9.11	2264200	9.21	405315	0.23254
CWTYETSVALAVEIANELPYNDYFE	HDAC1	Histone deacetylase HDAC1 (HD) (EC 3.5.1.98)	963.692	4	65.61	472255	65.23	0	0
DGMDDDAYESIFVPIISK	HDAC1	Histone deacetylase HDAC1 (HD) (EC 3.5.1.98)	1007.97	2	52.9	530494	52.56	138330	0.33873
FHSDEYVR	HDAC1	Histone deacetylase HDAC1 (HD) (EC 3.5.1.98)	526.743	2	13.32	709963	14.63	1351440	2.47277
FHSDEYVR	HDAC1	Histone deacetylase HDAC1 (HD) (EC 3.5.1.98)	351.498	3	13.32	673959	14.63	1582958	3.05111
FNVGEDCPVFDGLYEFCQLSAGGS	HDAC1	Histone deacetylase HDAC1 (HD) (EC 3.5.1.98)	1069.83	3	57.94	87424272	57.7	63756520	0.94736
FNVGEDCPVFDGLYEFCQLSAGGS	HDAC1	Histone deacetylase HDAC1 (HD) (EC 3.5.1.98)	802.624	4	57.94	1118260	57.66	844540	0.98107
LGCFNLTVK	HDAC1	Histone deacetylase HDAC1 (HD) (EC 3.5.1.98)	526.284	2	33.5	150463440	33.4	43651228	0.37687
LHISPSNMTNQNTSEYLEK	HDAC1	Histone deacetylase HDAC1 (HD) (EC 3.5.1.98)	736.02	3	28.84	82431888	28.73	40791888	0.64284
LHISPSNMTNQNTSEYLEK	HDAC1	Histone deacetylase HDAC1 (HD) (EC 3.5.1.98)	741.351	3	23.7	70432096	23.7	39591500	0.73022
MTHNLLLNYGLYR	HDAC1	Histone deacetylase HDAC1 (HD) (EC 3.5.1.98)	804.422	2	38.14	141822192	37.98	128141848	1.17373
MTHNLLLNYGLYR	HDAC1	Histone deacetylase HDAC1 (HD) (EC 3.5.1.98)	536.617	3	38.14	462744864	37.98	469054016	1.31675
MTHNLLLNYGLYR	HDAC1	Histone deacetylase HDAC1 (HD) (EC 3.5.1.98)	812.419	2	36.77	22296632	36.64	15969687	0.93042
MTHNLLLNYGLYR	HDAC1	Histone deacetylase HDAC1 (HD) (EC 3.5.1.98)	541.949	3	36.77	72824472	36.64	62372424	1.1126
QASEICINWGGGLHHAK	HDAC1	Histone deacetylase HDAC1 (HD) (EC 3.5.1.98)	939.457	2	29.1	2051706	29.03	1373282	0.86949
QASEICINWGGGLHHAK	HDAC1	Histone deacetylase HDAC1 (HD) (EC 3.5.1.98)	626.641	3	29.1	19598820	29.03	17632522	1.16871
QASEICINWGGGLHHAK	HDAC1	Histone deacetylase HDAC1 (HD) (EC 3.5.1.98)	470.232	4	29.14	19831466	29.03	18076356	1.18407
SEASGFCYVNDIVLGILELLK	HDAC1	Histone deacetylase HDAC1 (HD) (EC 3.5.1.98)	1170.61	2	65.78	2719398	65.77	2080468	0.99383
SEASGFCYVNDIVLGILELLK	HDAC1	Histone deacetylase HDAC1 (HD) (EC 3.5.1.98)	780.743	3	65.78	3096969	65.77	1401537	0.58788
SIRPDNMSEYNK	HDAC1	Histone deacetylase HDAC1 (HD) (EC 3.5.1.98)	727.341	2	16.04	53708104	16.04	37971828	0.91843
SIRPDNMSEYNK	HDAC1	Histone deacetylase HDAC1 (HD) (EC 3.5.1.98)	485.23	3	16.04	120756288	16.04	105525512	1.13519
SIRPDNMSEYNK	HDAC1	Histone deacetylase HDAC1 (HD) (EC 3.5.1.98)	735.338	2	10.87	23904354	10.96	16955166	0.9214
SIRPDNMSEYNK	HDAC1	Histone deacetylase HDAC1 (HD) (EC 3.5.1.98)	490.561	3	10.87	53564524	10.96	44713268	1.08438
VCYYYDSDIGNYYYGQGHPMKPHF	HDAC1	Histone deacetylase HDAC1 (HD) (EC 3.5.1.98)	746.579	4	29.87	4115821	29.76	3567074	1.12584
VCYYYDSDIGNYYYGQGHPMKPHF	HDAC1	Histone deacetylase HDAC1 (HD) (EC 3.5.1.98)	750.577	4	28.76	2696114	28.69	1881290	0.90644
VLYIDIDVHHGDGVEEAFYTTDR	HDAC1	Histone deacetylase HDAC1 (HD) (EC 3.5.1.98)	888.757	3	41.25	165468896	41.14	61172216	0.48024
VLYIDIDVHHGDGVEEAFYTTDR	HDAC1	Histone deacetylase HDAC1 (HD) (EC 3.5.1.98)	666.82	4	41.25	256402688	41.14	111283752	0.56381
VMETFQPAAVVLQCGADSLTGDR	HDAC1	Histone deacetylase HDAC1 (HD) (EC 3.5.1.98)	1233.09	2	49.57	27524708	49.28	8039522	0.37943
VMETFQPAAVVLQCGADSLTGDR	HDAC1	Histone deacetylase HDAC1 (HD) (EC 3.5.1.98)	822.398	3	49.57	84715408	49.28	27025732	0.41442
VMETFQPAAVVLQCGADSLTGDR	HDAC1	Histone deacetylase HDAC1 (HD) (EC 3.5.1.98)	827.73	3	46.69	8445326	46.45	2799609	0.43063
VMTVSFHK	HDAC1	Histone deacetylase HDAC1 (HD) (EC 3.5.1.98)	474.752	2	20.73	109722288	20.76	57390676	0.67947
VMTVSFHK	HDAC1	Histone deacetylase HDAC1 (HD) (EC 3.5.1.98)	482.75	2	15.64	6358404	15.58	2631531	0.53763
YGEYFPGTGDLR	HDAC1	Histone deacetylase HDAC1 (HD) (EC 3.5.1.98)	687.82	2	32.53	747701952	32.44	405453280	0.70442
YNLPFLMVGGGGYTIR	HDAC1	Histone deacetylase HDAC1 (HD) (EC 3.5.1.98)	879.456	2	52.58	179359136	52.33	115078000	0.83347
YNLPFLMVGGGGYTIR	HDAC1	Histone deacetylase HDAC1 (HD) (EC 3.5.1.98)	586.64	3	52.58	24277252	52.29	13448192	0.71959
YNLPFLMVGGGGYTIR	HDAC1	Histone deacetylase HDAC1 (HD) (EC 3.5.1.98)	887.453	2	49.33	155657392	49.1	55238892	0.461
YNLPFLMVGGGGYTIR	HDAC1	Histone deacetylase HDAC1 (HD) (EC 3.5.1.98)	591.971	3	49.29	5544385	48.91	3114041	0.72961
YYAVNIPLR	HDAC1	Histone deacetylase HDAC1 (HD) (EC 3.5.1.98)	554.811	2	35.58	11123592	35.45	2510918	0.29323
AAFGLVAEGFR	SdhA	Succinate dehydrogenase [ubiquinone] flavoprotein subunit, mitochondrial (E(	569.306	2	39.62	1069174	39.35	1016382	1.2349
LGANSLLDLVVFGR	SdhA	Succinate dehydrogenase [ubiquinone] flavoprotein subunit, mitochondrial (E(	737.425	2	59.1	1075521	58.95	1014957	1.22589
LTTNNFALGYTTK	porin	Voltage-dependent anion-selective channel (DmVDAC) (Porin)	722.378	2	31.55	326396	31.43	582312	2.31757
VFGSLETK	porin	Voltage-dependent anion-selective channel (DmVDAC) (Porin)	440.742	2	21.81	305180	21.82	380085	1.61788
VNNASQVGLGYQQK	porin	Voltage-dependent anion-selective channel (DmVDAC) (Porin)	753.389	2	19.18	385340	19.14	400935	1.35161
YQLDDDASVR	porin	Voltage-dependent anion-selective channel (DmVDAC) (Porin)	591.275	2	20.06	853548	20.05	1199040	1.82485
LFQVEYAIEAIK	Prosalpha	Proteasome subunit alpha type-5 (EC 3.4.25.1)	712.395	2	47.17	132055	46.95	351128	3.45408
DAGHIAGLNVLR	Hsp70Bc	Major heat shock 70 kDa protein Bc (Heat shock protein 70Bc) (HSP70-87C1	618.346	2	31.67	40614	31.54	13040718	417.108
DAGHIAGLNVLR	Hsp70Bc	Major heat shock 70 kDa protein Bc (Heat shock protein 70Bc) (HSP70-87C1	412.567	3	31.59	26572	31.54	33980956	1661.24
DNNALGTFDLSGIPPAPR	Hsp70Bc	Major heat shock 70 kDa protein Bc (Heat shock protein 70Bc) (HSP70-87C1	927.971	2	45.07	166587	43.39	30976200	241.551
DNNALGTFDLSGIPPAPR	Hsp70Bc	Major heat shock 70 kDa protein Bc (Heat shock protein 70Bc) (HSP70-87C1	618.983	3	45.21	24051	43.65	6711729	362.513
ETAEAYLGESITDAVITVPAYFNDSC	Hsp70Bc	Major heat shock 70 kDa protein Bc (Heat shock protein 70Bc) (HSP70-87C1	987.477	3	58.6	304104	58.36	1308713	5.59042
FAPEEISSMVLTK	Hsp70Bc	Major heat shock 70 kDa protein Bc (Heat shock protein 70Bc) (HSP70-87C1	726.376	2	41.77	22231	41.79	11132469	650.511

FAPEEISSMVLTK	Hsp70Bc	Major heat shock 70 kDa protein Bc (Heat shock protein 70Bc) (HSP70-87C1	484.587	3	41.77	0	41.79	276280	#DIV/0!
FAPEEISSMVLTK	Hsp70Bc	Major heat shock 70 kDa protein Bc (Heat shock protein 70Bc) (HSP70-87C1	734.374	2	35.12	297494	34.86	2047640	8.94124
FEELCADLFR	Hsp70Bc	Major heat shock 70 kDa protein Bc (Heat shock protein 70Bc) (HSP70-87C1	650.306	2	41.55	32123	41.36	4667637	188.757
GQIHDIVLVGGSTR	Hsp70Bc	Major heat shock 70 kDa protein Bc (Heat shock protein 70Bc) (HSP70-87C1	726.402	2	27.66	141880	27.58	2013950	18.4395
GQIHDIVLVGGSTR	Hsp70Bc	Major heat shock 70 kDa protein Bc (Heat shock protein 70Bc) (HSP70-87C1	484.604	3	27.43	42940	27.54	4848927	146.692
HCSPIMTK	Hsp70Bc	Major heat shock 70 kDa protein Bc (Heat shock protein 70Bc) (HSP70-87C1	487.233	2	11.2	35165	11.25	2786296	102.929
HCSPIMTK	Hsp70Bc	Major heat shock 70 kDa protein Bc (Heat shock protein 70Bc) (HSP70-87C1	495.231	2	9.26	11777	9.24	218205	24.0687
IQDVLLVDVAPLSLGIETAGGVMTK	Hsp70Bc	Major heat shock 70 kDa protein Bc (Heat shock protein 70Bc) (HSP70-87C1	1270.21	2	61.32	0	61.17	571901	#DIV/0!
IQDVLLVDVAPLSLGIETAGGVMTK	Hsp70Bc	Major heat shock 70 kDa protein Bc (Heat shock protein 70Bc) (HSP70-87C1	847.14	3	61.32	519	61.21	3335041	8347.49
IQDVLLVDVAPLSLGIETAGGVMTK	Hsp70Bc	Major heat shock 70 kDa protein Bc (Heat shock protein 70Bc) (HSP70-87C1	852.472	3	57.66	0	57.38	359486	#DIV/0!
LSQAEIDR	Hsp70Bc	Major heat shock 70 kDa protein Bc (Heat shock protein 70Bc) (HSP70-87C1	466.246	2	14.26	24538	14.28	4887219	258.729
LVTHLAEEFK	Hsp70Bc	Major heat shock 70 kDa protein Bc (Heat shock protein 70Bc) (HSP70-87C1	593.827	2	25.94	371859	26.5	486802	1.70058
NALESYVFNVK	Hsp70Bc	Major heat shock 70 kDa protein Bc (Heat shock protein 70Bc) (HSP70-87C1	642.335	2	40.6	35451	38.81	48323708	1770.74
NLNLSINPDEAVAYGAAVQAAILSGI	Hsp70Bc	Major heat shock 70 kDa protein Bc (Heat shock protein 70Bc) (HSP70-87C1	1493.76	2	58.87	44314	57.77	661805	19.4004
NLNLSINPDEAVAYGAAVQAAILSGI	Hsp70Bc	Major heat shock 70 kDa protein Bc (Heat shock protein 70Bc) (HSP70-87C1	996.175	3	58.34	121646	57.81	3315510	35.4058
NQVAMNPR	Hsp70Bc	Major heat shock 70 kDa protein Bc (Heat shock protein 70Bc) (HSP70-87C1	465.235	2	11.74	204889	11.84	365105	2.31484
STAGDTHLGGEDFDNR	Hsp70Bc	Major heat shock 70 kDa protein Bc (Heat shock protein 70Bc) (HSP70-87C1	846.366	2	18.67	1657789	18.65	1831600	1.43524
STAGDTHLGGEDFDNR	Hsp70Bc	Major heat shock 70 kDa protein Bc (Heat shock protein 70Bc) (HSP70-87C1	564.58	3	18.67	10781481	18.68	14815577	1.7851
TFSTYSDNQPGVSIQVYEGER	Hsp70Bc	Major heat shock 70 kDa protein Bc (Heat shock protein 70Bc) (HSP70-87C1	793.036	3	34.64	0	34.11	7628120	#DIV/0!
TLSSSTEATIEIDALFEGQDFYTK	Hsp70Bc	Major heat shock 70 kDa protein Bc (Heat shock protein 70Bc) (HSP70-87C1	1333.64	2	55.81	30514	54.36	4199024	178.76
TLSSSTEATIEIDALFEGQDFYTK	Hsp70Bc	Major heat shock 70 kDa protein Bc (Heat shock protein 70Bc) (HSP70-87C1	889.429	3	55.66	365012	54.36	14991373	53.3527
TTPSYVAFTDSER	Hsp70Bc	Major heat shock 70 kDa protein Bc (Heat shock protein 70Bc) (HSP70-87C1	737.346	2	28.48	159128	28.32	60403172	493.101
TTPSYVAFTDSER	Hsp70Bc	Major heat shock 70 kDa protein Bc (Heat shock protein 70Bc) (HSP70-87C1	491.9	3	28.44	0	28.4	323797	#DIV/0!
VEIIANDQGNR	Hsp70Bc	Major heat shock 70 kDa protein Bc (Heat shock protein 70Bc) (HSP70-87C1	614.818	2	18.24	20421124	18.27	30231408	1.9231
VQSLLQEFFHGK	Hsp70Bc	Major heat shock 70 kDa protein Bc (Heat shock protein 70Bc) (HSP70-87C1	478.258	3	46.44	17592	46.3	17550354	1295.96
DAGHIAGLNVLR	Hsp70Bb	Major heat shock 70 kDa protein Bb (Heat shock protein 70Bb) (HSP70-87C1	618.346	2	31.67	40614	31.54	13040718	417.108
DAGHIAGLNVLR	Hsp70Bb	Major heat shock 70 kDa protein Bb (Heat shock protein 70Bb) (HSP70-87C1	412.567	3	31.59	26572	31.54	33980956	1661.24
DNNALGTFDLSGIPPAPR	Hsp70Bb	Major heat shock 70 kDa protein Bb (Heat shock protein 70Bb) (HSP70-87C1	927.971	2	45.07	166587	43.39	30976200	241.551
DNNALGTFDLSGIPPAPR	Hsp70Bb	Major heat shock 70 kDa protein Bb (Heat shock protein 70Bb) (HSP70-87C1	618.983	3	45.21	24051	43.65	6711729	362.513
ETAEAYLGESITDAVITVPAYFNDSC	Hsp70Bb	Major heat shock 70 kDa protein Bb (Heat shock protein 70Bb) (HSP70-87C1	987.477	3	58.6	304104	58.36	1308713	5.59042
FAPEEISSMVLTK	Hsp70Bb	Major heat shock 70 kDa protein Bb (Heat shock protein 70Bb) (HSP70-87C1	726.376	2	41.77	22231	41.79	11132469	650.511
FAPEEISSMVLTK	Hsp70Bb	Major heat shock 70 kDa protein Bb (Heat shock protein 70Bb) (HSP70-87C1	484.587	3	41.77	0	41.79	276280	#DIV/0!
FAPEEISSMVLTK	Hsp70Bb	Major heat shock 70 kDa protein Bb (Heat shock protein 70Bb) (HSP70-87C1	734.374	2	35.12	297494	34.86	2047640	8.94124
FEELCADLFR	Hsp70Bb	Major heat shock 70 kDa protein Bb (Heat shock protein 70Bb) (HSP70-87C1	650.306	2	41.55	32123	41.36	4667637	188.757
GQIHDIVLVGGSTR	Hsp70Bb	Major heat shock 70 kDa protein Bb (Heat shock protein 70Bb) (HSP70-87C1	726.402	2	27.66	141880	27.58	2013950	18.4395
GQIHDIVLVGGSTR	Hsp70Bb	Major heat shock 70 kDa protein Bb (Heat shock protein 70Bb) (HSP70-87C1	484.604	3	27.43	42940	27.54	4848927	146.692
HCSPIMTK	Hsp70Bb	Major heat shock 70 kDa protein Bb (Heat shock protein 70Bb) (HSP70-87C1	487.233	2	11.2	35165	11.25	2786296	102.929
HCSPIMTK	Hsp70Bb	Major heat shock 70 kDa protein Bb (Heat shock protein 70Bb) (HSP70-87C1	495.231	2	9.26	11777	9.24	218205	24.0687
IQDVLLVDVAPLSLGIETAGGVMTK	Hsp70Bb	Major heat shock 70 kDa protein Bb (Heat shock protein 70Bb) (HSP70-87C1	1270.21	2	61.32	0	61.17	571901	#DIV/0!
IQDVLLVDVAPLSLGIETAGGVMTK	Hsp70Bb	Major heat shock 70 kDa protein Bb (Heat shock protein 70Bb) (HSP70-87C1	847.14	3	61.32	519	61.21	3335041	8347.49
IQDVLLVDVAPLSLGIETAGGVMTK	Hsp70Bb	Major heat shock 70 kDa protein Bb (Heat shock protein 70Bb) (HSP70-87C1	852.472	3	57.66	0	57.38	359486	#DIV/0!
LSQAEIDR	Hsp70Bb	Major heat shock 70 kDa protein Bb (Heat shock protein 70Bb) (HSP70-87C1	466.246	2	14.26	24538	14.28	4887219	258.729
LVTHLAEEFK	Hsp70Bb	Major heat shock 70 kDa protein Bb (Heat shock protein 70Bb) (HSP70-87C1	593.827	2	25.94	371859	26.5	486802	1.70058
NALESYVFNVK	Hsp70Bb	Major heat shock 70 kDa protein Bb (Heat shock protein 70Bb) (HSP70-87C1	642.335	2	40.6	35451	38.81	48323708	1770.74
NLNLSINPDEAVAYGAAVQAAILSGI	Hsp70Bb	Major heat shock 70 kDa protein Bb (Heat shock protein 70Bb) (HSP70-87C1	1493.76	2	58.87	44314	57.77	661805	19.4004
NLNLSINPDEAVAYGAAVQAAILSGI	Hsp70Bb	Major heat shock 70 kDa protein Bb (Heat shock protein 70Bb) (HSP70-87C1	996.175	3	58.34	121646	57.81	3315510	35.4058
NQVAMNPR	Hsp70Bb	Major heat shock 70 kDa protein Bb (Heat shock protein 70Bb) (HSP70-87C1	465.235	2	11.74	204889	11.84	365105	2.31484
STAGDTHLGGEDFDNR	Hsp70Bb	Major heat shock 70 kDa protein Bb (Heat shock protein 70Bb) (HSP70-87C1	846.366	2	18.67	1657789	18.65	1831600	1.43524
STAGDTHLGGEDFDNR	Hsp70Bb	Major heat shock 70 kDa protein Bb (Heat shock protein 70Bb) (HSP70-87C1	564.58	3	18.67	10781481	18.68	14815577	1.7851
TFSTYSDNQPGVSIQVYEGER	Hsp70Bb	Major heat shock 70 kDa protein Bb (Heat shock protein 70Bb) (HSP70-87C1	793.036	3	34.64	0	34.11	7628120	#DIV/0!
TLSSSTEATIEIDALFEGQDFYTK	Hsp70Bb	Major heat shock 70 kDa protein Bb (Heat shock protein 70Bb) (HSP70-87C1	1333.64	2	55.81	30514	54.36	4199024	178.76
TLSSSTEATIEIDALFEGQDFYTK	Hsp70Bb	Major heat shock 70 kDa protein Bb (Heat shock protein 70Bb) (HSP70-87C1	889.429	3	55.66	365012	54.36	14991373	53.3527
TTPSYVAFTDSER	Hsp70Bb	Major heat shock 70 kDa protein Bb (Heat shock protein 70Bb) (HSP70-87C1	737.346	2	28.48	159128	28.32	60403172	493.101

TTPSYVAFTDSER	Hsp70Bb	Major heat shock 70 kDa protein Bb (Heat shock protein 70Bb) (HSP70-87C1	491.9	3	28.44	0	28.4	323797	#DIV/0!
VEIIANDQGNR	Hsp70Bb	Major heat shock 70 kDa protein Bb (Heat shock protein 70Bb) (HSP70-87C1	614.818	2	18.24	20421124	18.27	30231408	1.9231
VQSLLQEFFHGK	Hsp70Bb	Major heat shock 70 kDa protein Bb (Heat shock protein 70Bb) (HSP70-87C1	478.258	3	46.44	17592	46.3	17550354	1295.96
HVELYNVGK	Jupiter	Microtubule-associated protein Jupiter	529.785	2	19.38	382263	19.41	547919	1.86199
SSIPFGQNTEAVAAQK	Jupiter	Microtubule-associated protein Jupiter	824.42	2	27.69	245096	27.65	643769	3.41206
LFITESLQIAK	eIF3h	Eukaryotic translation initiation factor 3 subunit H (eIF3h) (Eukaryotic translati	631.871	2	42.05	120480	41.83	127256	1.3721
TLIDDDNPPVSFVK	wds	Protein will die slowly	780.401	2	38.13	2624767	37.75	1560803	0.77247
DYGVLDEETGIPFR	Jafrac1	Peroxiredoxin 1 (EC 1.11.1.15) (Cytosolic thioredoxin peroxidase) (DPx-4783)	805.888	2	42.29	1389123	42.06	1525411	1.42649
SVEETLR	Jafrac1	Peroxiredoxin 1 (EC 1.11.1.15) (Cytosolic thioredoxin peroxidase) (DPx-4783)	417.222	2	14.57	798318	14.56	716777	1.16635
FTILPHDFSIPTGELGPTLK	bgm	Very long-chain-fatty-acid--CoA ligase bubblegum (EC 6.2.1.3)	728.396	3	49.95	906351	49.6	472601	0.67736
LCQGNADATIR	Aac11	Apoptosis inhibitor 5 homolog (API-5) (Antiapoptosis clone 11 protein homolog	609.798	2	14.57	226422	14.52	122831	0.70471
HAQDYSLEASAK	apolpp	Apolipophorins (Retinoid- and fatty acid-binding glycoprotein) [Cleaved into: A	660.315	2	14.87	331176	14.8	247926	0.97249
ECTIHLAK	RpL31	60S ribosomal protein L31	486.253	2	15.49	2304010	15.43	1332958	0.75154
EMGTTDVR	RpL31	60S ribosomal protein L31	454.711	2	11.66	962059	11.76	382662	0.5167
LYTYVTYVPVSTFK	RpL31	60S ribosomal protein L31	840.948	2	43.44	4801252	43.21	4022424	1.08832
NLQTENVESSDD	RpL31	60S ribosomal protein L31	675.786	2	15.99	4997861	15.98	2747102	0.71402
SAINEVVTR	RpL31	60S ribosomal protein L31	494.775	2	20.13	20293016	20.13	19123418	1.22417
VHNIGFK	RpL31	60S ribosomal protein L31	407.732	2	14.79	1318964	14.79	735432	0.72432
EQQYISSSVDPK	cora	Protein 4.1 homolog (Protein coracle)	690.836	2	19.72	1077568	19.73	711194	0.85736
IAPNQTAELEDK	cora	Protein 4.1 homolog (Protein coracle)	664.838	2	18.46	483773	18.46	266610	0.71591
ITIQSDGDPIDHDK	cora	Protein 4.1 homolog (Protein coracle)	518.586	3	21.49	444280	21.51	465036	1.35973
LDDTLGQIVCITPQDNPVVELTVITS	cora	Protein 4.1 homolog (Protein coracle)	999.525	3	60.96	477920	60.76	355750	0.96697
LGKPVSTPTVVK	cora	Protein 4.1 homolog (Protein coracle)	409.255	3	19.33	1398453	19.26	958082	0.88997
LGLVVTSGLGDNQQDQNLDEAAR	cora	Protein 4.1 homolog (Protein coracle)	805.07	3	37.08	2956134	36.91	3245204	1.42607
LIEVGQQIDPIVEVTSLAGK	cora	Protein 4.1 homolog (Protein coracle)	1055.09	2	52.4	1797413	52.06	1009797	0.72981
LIEVGQQIDPIVEVTSLAGK	cora	Protein 4.1 homolog (Protein coracle)	703.732	3	52.36	1187434	52.06	855614	0.93603
LILNYAQIDPSHFGK	cora	Protein 4.1 homolog (Protein coracle)	572.642	3	39.99	1926732	39.77	2328472	1.5699
LLQDPLLSPTTR	cora	Protein 4.1 homolog (Protein coracle)	677.39	2	36.71	1818393	36.6	2546599	1.81926
LQGSAVDAAAVPLSDSQK	cora	Protein 4.1 homolog (Protein coracle)	878.957	2	28.08	902207	28	1209649	1.74171
MFPVFGSTYR	cora	Protein 4.1 homolog (Protein coracle)	610.792	2	35.32	1647098	35.1	1475401	1.16362
NIDGVTHNVEEEVR	cora	Protein 4.1 homolog (Protein coracle)	805.892	2	25.19	772422	25.18	383894	0.64562
NIDGVTHNVEEEVR	cora	Protein 4.1 homolog (Protein coracle)	537.597	3	25.23	1472968	25.14	947257	0.8354
QAQVQTTTETVPITR	cora	Protein 4.1 homolog (Protein coracle)	836.947	2	22.76	1243364	22.75	1875309	1.95928
QAQVQTTTETVPITR	cora	Protein 4.1 homolog (Protein coracle)	558.3	3	22.72	338192	22.75	517745	1.98873
SLGVLNLDTGLLDTK	cora	Protein 4.1 homolog (Protein coracle)	779.938	2	48.49	4006855	48.22	3396143	1.10104
SPLFTTSATTGPHVESTR	cora	Protein 4.1 homolog (Protein coracle)	630.318	3	26.47	4223504	26.38	3945529	1.21354
SSSSSHGKPALAR	cora	Protein 4.1 homolog (Protein coracle)	642.836	2	8.36	115696	8.51	27955	0.31388
SSSSSHGKPALAR	cora	Protein 4.1 homolog (Protein coracle)	428.893	3	8.36	446429	8.49	229562	0.66799
TAQVETSGGQFDPK	cora	Protein 4.1 homolog (Protein coracle)	732.852	2	18.64	2712306	18.61	2930955	1.40376
TDTWPLTFAVK	cora	Protein 4.1 homolog (Protein coracle)	639.84	2	45.17	2241253	44.93	1441517	0.83551
TGNLHLVSGVADPK	cora	Protein 4.1 homolog (Protein coracle)	469.925	3	24.12	894256	24.12	482494	0.70089
TGYVITIYGYLDPK	cora	Protein 4.1 homolog (Protein coracle)	801.924	2	47.28	1483311	47.07	1539226	1.34801
TQAESTNTPVDR	cora	Protein 4.1 homolog (Protein coracle)	659.815	2	10.52	427177	10.65	96821	0.29443
TTATVTSGDQYQR	cora	Protein 4.1 homolog (Protein coracle)	714.342	2	13.07	702928	13.03	163813	0.30273
TTVTHTMTLTGEIDPVTGR	cora	Protein 4.1 homolog (Protein coracle)	677.346	3	32.9	1242941	32.67	1420656	1.48478
TVYVYNMIDPVTGEIVQVDPNDPR	cora	Protein 4.1 homolog (Protein coracle)	912.118	3	48.94	447629	48.63	338096	0.98117
VTLLDGSLLDVSIDR	cora	Protein 4.1 homolog (Protein coracle)	808.449	2	50.48	2980908	50.17	1512386	0.65908
VVLGEDTPGFSGHGEIISTQTVSSK	cora	Protein 4.1 homolog (Protein coracle)	849.098	3	34.36	2644169	34.26	2132465	1.04765
YGVIDPK	cora	Protein 4.1 homolog (Protein coracle)	396.219	2	18.74	1027816	18.73	968797	1.22445
SAFIQVLESSYR	aralar1	Calcium-binding mitochondrial carrier protein Aralar1	700.364	2	43.03	477153	42.8	602689	1.64081
AVIIYVPIPQQK	RpS7	40S ribosomal protein S7	684.916	2	41.28	8471675	41.1	6545686	1.00371
DLHITR	RpS7	40S ribosomal protein S7	377.714	2	14.71	21152176	14.72	16014808	0.98353
DVTFEFPDNYLNV	RpS7	40S ribosomal protein S7	786.864	2	53.99	23061740	53.69	18809548	1.05952

HVVVIAER	RpS7	40S ribosomal protein S7	461.777	2	15.49	20082808	15.46	11177676	0.72302
IIKPGGSDPDDFEK	RpS7	40S ribosomal protein S7	759.378	2	20.47	4039644	20.43	3343605	1.07521
IIKPGGSDPDDFEK	RpS7	40S ribosomal protein S7	506.588	3	20.47	25628294	20.43	25835260	1.30953
IQIILVR	RpS7	40S ribosomal protein S7	427.795	2	36.3	22111984	36.14	13910792	0.81723
SIAQALVELEANSDLKPYLR	RpS7	40S ribosomal protein S7	1115.61	2	52.69	3635581	52.52	3184821	1.13798
SIAQALVELEANSDLKPYLR	RpS7	40S ribosomal protein S7	744.074	3	52.73	38728332	52.56	37722616	1.26531
SIAQALVELEANSDLKPYLR	RpS7	40S ribosomal protein S7	558.307	4	52.69	4180332	52.56	3628128	1.12744
TLTAVYDAILEDLVFPAEIVGK	RpS7	40S ribosomal protein S7	1189.15	2	65.64	2919810	65.67	1234142	0.54908
TLTAVYDAILEDLVFPAEIVGK	RpS7	40S ribosomal protein S7	793.102	3	65.64	2850982	65.67	1260429	0.57431
VDTFTSVYK	RpS7	40S ribosomal protein S7	530.271	2	25.44	3847964	25.35	1682874	0.56812
LYPTVVVQPDGSTINIR	mRpL55	39S ribosomal protein L55, mitochondrial (L55mt) (MRP-L55)	936.515	2	39.28	1074393	39.08	1040329	1.25785
AQCPIVER	RpS5b	40S ribosomal protein S5b	486.75	2	14.71	806831	14.72	445526	0.71732
QAVDVSPLR	RpS5b	40S ribosomal protein S5b	492.777	2	23.02	630384	23.02	403036	0.83054
AMELYR	Cyp313a	Probable cytochrome P450 313a1 (EC 1.14.-.-) (CYPCCCXIIIA1)	391.697	2	20.4	76256	20.44	242871	4.13737
LIPAIPITAR	Cyp313a	Probable cytochrome P450 313a1 (EC 1.14.-.-) (CYPCCCXIIIA1)	532.845	2	37.91	41055	37.76	3413281	108.001
TDSDPESNIVINR	Cyp313a	Probable cytochrome P450 313a1 (EC 1.14.-.-) (CYPCCCXIIIA1)	730.355	2	24.08	276064	23.37	1685356	7.93057
VVEDIFSSPDCHNK	Cyp313a	Probable cytochrome P450 313a1 (EC 1.14.-.-) (CYPCCCXIIIA1)	549.587	3	26.43	0	26.43	281780	#DIV/0!
ASQELGLR	HtrA2	Serine protease HTRA2, mitochondrial (EC 3.4.21.108) (High temperature rec	437.243	2	17.76	957616	17.79	498328	0.676
TFPATIEDVDQTSDLATLR	HtrA2	Serine protease HTRA2, mitochondrial (EC 3.4.21.108) (High temperature rec	1047.02	2	42.81	1456762	42.55	805342	0.71815
TLDIVILR	HtrA2	Serine protease HTRA2, mitochondrial (EC 3.4.21.108) (High temperature rec	471.803	2	41.14	1392585	40.98	994282	0.92749
VTAGISFAIPIDYVK	HtrA2	Serine protease HTRA2, mitochondrial (EC 3.4.21.108) (High temperature rec	797.448	2	50.05	817270	49.8	294920	0.46877
DAGHIAGLNVLR	Hsp70Bb	Major heat shock 70 kDa protein Bbb (Heat shock protein 70Bbb) (HSP70-87	618.346	2	31.67	40614	31.54	13040718	417.108
DAGHIAGLNVLR	Hsp70Bb	Major heat shock 70 kDa protein Bbb (Heat shock protein 70Bbb) (HSP70-87	412.567	3	31.59	26572	31.54	33980956	1661.24
DNNALGTFDLSGIPPAPR	Hsp70Bb	Major heat shock 70 kDa protein Bbb (Heat shock protein 70Bbb) (HSP70-87	927.971	2	45.07	166587	43.39	30976200	241.551
DNNALGTFDLSGIPPAPR	Hsp70Bb	Major heat shock 70 kDa protein Bbb (Heat shock protein 70Bbb) (HSP70-87	618.983	3	45.21	24051	43.65	6711729	362.513
ETAEAYLGESITDAVITVPAYFNDS	Hsp70Bb	Major heat shock 70 kDa protein Bbb (Heat shock protein 70Bbb) (HSP70-87	987.477	3	58.6	304104	58.36	1308713	5.59042
FAPEEISSMVLTK	Hsp70Bb	Major heat shock 70 kDa protein Bbb (Heat shock protein 70Bbb) (HSP70-87	726.376	2	41.77	22231	41.79	11132469	650.511
FAPEEISSMVLTK	Hsp70Bb	Major heat shock 70 kDa protein Bbb (Heat shock protein 70Bbb) (HSP70-87	484.587	3	41.77	0	41.79	276280	#DIV/0!
FAPEEISSMVLTK	Hsp70Bb	Major heat shock 70 kDa protein Bbb (Heat shock protein 70Bbb) (HSP70-87	734.374	2	35.12	297494	34.86	2047640	8.94124
FEELCADLFR	Hsp70Bb	Major heat shock 70 kDa protein Bbb (Heat shock protein 70Bbb) (HSP70-87	650.306	2	41.55	32123	41.36	4667637	188.757
GQIHDIVLVGGSTR	Hsp70Bb	Major heat shock 70 kDa protein Bbb (Heat shock protein 70Bbb) (HSP70-87	726.402	2	27.66	141880	27.58	2013950	18.4395
GQIHDIVLVGGSTR	Hsp70Bb	Major heat shock 70 kDa protein Bbb (Heat shock protein 70Bbb) (HSP70-87	484.604	3	27.43	42940	27.54	4848927	146.692
HCSPIMTK	Hsp70Bb	Major heat shock 70 kDa protein Bbb (Heat shock protein 70Bbb) (HSP70-87	487.233	2	11.2	35165	11.25	2786296	102.929
HCSPIMTK	Hsp70Bb	Major heat shock 70 kDa protein Bbb (Heat shock protein 70Bbb) (HSP70-87	495.231	2	9.26	11777	9.24	218205	24.0687
IQDVLLVDVAPLSLGIETAGGVMTK	Hsp70Bb	Major heat shock 70 kDa protein Bbb (Heat shock protein 70Bbb) (HSP70-87	1270.21	2	61.32	0	61.17	571901	#DIV/0!
IQDVLLVDVAPLSLGIETAGGVMTK	Hsp70Bb	Major heat shock 70 kDa protein Bbb (Heat shock protein 70Bbb) (HSP70-87	847.14	3	61.32	519	61.21	3335041	8347.49
IQDVLLVDVAPLSLGIETAGGVMTK	Hsp70Bb	Major heat shock 70 kDa protein Bbb (Heat shock protein 70Bbb) (HSP70-87	852.472	3	57.66	0	57.38	359486	#DIV/0!
LSQAEIDR	Hsp70Bb	Major heat shock 70 kDa protein Bbb (Heat shock protein 70Bbb) (HSP70-87	466.246	2	14.26	24538	14.28	4887219	258.729
LVTHLAEEFK	Hsp70Bb	Major heat shock 70 kDa protein Bbb (Heat shock protein 70Bbb) (HSP70-87	593.827	2	25.94	371859	26.5	486802	1.70058
NALESYVFNVK	Hsp70Bb	Major heat shock 70 kDa protein Bbb (Heat shock protein 70Bbb) (HSP70-87	642.335	2	40.6	35451	38.81	48323708	1770.74
NLNLSINPDEAVAYGAAVQAAILSG	Hsp70Bb	Major heat shock 70 kDa protein Bbb (Heat shock protein 70Bbb) (HSP70-87	1493.76	2	58.87	44314	57.77	661805	19.4004
NLNLSINPDEAVAYGAAVQAAILSG	Hsp70Bb	Major heat shock 70 kDa protein Bbb (Heat shock protein 70Bbb) (HSP70-87	996.175	3	58.34	121646	57.81	3315510	35.4058
NQVAMNPR	Hsp70Bb	Major heat shock 70 kDa protein Bbb (Heat shock protein 70Bbb) (HSP70-87	465.235	2	11.74	204889	11.84	365105	2.31484
STAGDTHLGGEDFDNR	Hsp70Bb	Major heat shock 70 kDa protein Bbb (Heat shock protein 70Bbb) (HSP70-87	846.366	2	18.67	1657789	18.65	1831600	1.43524
STAGDTHLGGEDFDNR	Hsp70Bb	Major heat shock 70 kDa protein Bbb (Heat shock protein 70Bbb) (HSP70-87	564.58	3	18.67	10781481	18.68	14815577	1.7851
TFSTYSDNQPGVSIQVYEGER	Hsp70Bb	Major heat shock 70 kDa protein Bbb (Heat shock protein 70Bbb) (HSP70-87	793.036	3	34.64	0	34.11	7628120	#DIV/0!
TLSSSTEATIEIDALFEGQDFYTK	Hsp70Bb	Major heat shock 70 kDa protein Bbb (Heat shock protein 70Bbb) (HSP70-87	1333.64	2	55.81	30514	54.36	4199024	178.76
TLSSSTEATIEIDALFEGQDFYTK	Hsp70Bb	Major heat shock 70 kDa protein Bbb (Heat shock protein 70Bbb) (HSP70-87	889.429	3	55.66	365012	54.36	14991373	53.3527
TTPSYVAFTDSER	Hsp70Bb	Major heat shock 70 kDa protein Bbb (Heat shock protein 70Bbb) (HSP70-87	737.346	2	28.48	159128	28.32	60403172	493.101
TTPSYVAFTDSER	Hsp70Bb	Major heat shock 70 kDa protein Bbb (Heat shock protein 70Bbb) (HSP70-87	491.9	3	28.44	0	28.4	323797	#DIV/0!
VEIIANDQGNR	Hsp70Bb	Major heat shock 70 kDa protein Bbb (Heat shock protein 70Bbb) (HSP70-87	614.818	2	18.24	20421124	18.27	30231408	1.9231
VQSLLQEFFHGK	Hsp70Bb	Major heat shock 70 kDa protein Bbb (Heat shock protein 70Bbb) (HSP70-87	478.258	3	46.44	17592	46.3	17550354	1295.96
EYANDIGFK	RpS29	40S ribosomal protein S29	528.753	2	24.55	5298011	24.52	4571746	1.12096

YGLNICR	RpS29	40S ribosomal protein S29	448.226	2	23.96	2206338	23.96	2842385	1.67353
HVINFDLPSDVEEYVHR	bel	ATP-dependent RNA helicase bel (EC 3.6.4.13) (Protein belle)	690.341	3	42.33	52327	42.25	363307	9.01925
SGDCPILVATAVAAR	bel	ATP-dependent RNA helicase bel (EC 3.6.4.13) (Protein belle)	750.896	2	37.72	71006	37.48	451861	8.2667
GDSVILVLR	SmD2	Probable small nuclear ribonucleoprotein Sm D2 (Sm-D2) (snRNP core protei	486.298	2	36.9	739361	36.72	471765	0.82888
NNTQVLINCR	SmD2	Probable small nuclear ribonucleoprotein Sm D2 (Sm-D2) (snRNP core protei	616.314	2	20.03	394622	20.01	371111	1.22164
FAVNVDSPSR	hgo	Homogentisate 1,2-dioxygenase (EC 1.13.11.5) (Homogentisate oxygenase) (	546.278	2	23.05	15449	23.22	111077	9.33998
ELSNLESVNPK	KFase	Kynurenine formamidase (KFA) (KFase) (EC 3.5.1.9) (Arylformamidase) (N-fo	615.322	2	23.24	109557	23.14	186670	2.21338
HYADVLR	KFase	Kynurenine formamidase (KFA) (KFase) (EC 3.5.1.9) (Arylformamidase) (N-fo	437.233	2	16.26	45711	16.22	111969	3.18199
AAVEEGIVPGGGTALLR	Hsp60C	60 kDa heat shock protein homolog 2, mitochondrial (60 kDa chaperonin) (CP	805.449	2	37.57	3201747	37.41	3272869	1.3279
FGPEVR	Hsp60C	60 kDa heat shock protein homolog 2, mitochondrial (60 kDa chaperonin) (CP	352.69	2	17.41	1545828	17.45	1317497	1.10716
LVQDVANNTNEEAGDGTTTATVLAI	Hsp60C	60 kDa heat shock protein homolog 2, mitochondrial (60 kDa chaperonin) (CP	854.088	3	28.53	1313197	28.39	1332802	1.31843
TVAGGAWVYSTTAAASVR	RpL37A	60S ribosomal protein L37a	884.455	2	35.51	1995172	35.37	2313941	1.50659
TVAGGAWVYSTTAAASVR	RpL37A	60S ribosomal protein L37a	589.972	3	35.55	824979	35.33	1038904	1.63589
YTCSFCGK	RpL37A	60S ribosomal protein L37a	511.707	2	15.1	296011	15.08	174740	0.76684
AGIVVPFDQK	CG1218	Histone PARylation factor 1-like	537.303	2	33.47	6646903	33.37	2268859	0.44342
ENLLIAR	CG1218	Histone PARylation factor 1-like	414.751	2	25.57	440852	25.56	381848	1.12517
EYDNLCESNEFIR	CG1218	Histone PARylation factor 1-like	844.865	2	31.63	1959522	31.49	1062272	0.70422
FYYDPPEFQTIFVR	CG1218	Histone PARylation factor 1-like	911.446	2	50.48	8389040	50.25	4055532	0.628
FYYDPPEFQTIFVR	CG1218	Histone PARylation factor 1-like	607.966	3	50.51	2678767	50.25	1143147	0.55436
GSYEAETEELHK	CG1218	Histone PARylation factor 1-like	696.818	2	16.22	381862	16.21	235605	0.80149
GSYEAETEELHK	CG1218	Histone PARylation factor 1-like	464.881	3	16.26	1101486	16.21	539771	0.63658
GTGIHYGYWR	CG1218	Histone PARylation factor 1-like	403.865	3	25.27	2081514	25.22	1172175	0.73154
LGEPGDYLR	CG1218	Histone PARylation factor 1-like	510.261	2	26.16	9221286	26.15	4720985	0.66506
NYMEILEK	CG1218	Histone PARylation factor 1-like	528.258	2	23.59	1700695	23.69	1243872	0.9501
TEVGYRPLAVSDSELK	CG1218	Histone PARylation factor 1-like	588.644	3	29.33	2716172	29.24	976468	0.46701
VFQLQLVGPFEFLAGK	CG1218	Histone PARylation factor 1-like	897.003	2	62.82	2086123	62.5	439462	0.27366
YLSDNSLELAQLDR	CG1218	Histone PARylation factor 1-like	818.912	2	37.05	13109144	36.87	7556711	0.74883
AGGEVLTFDQLALR	RpL18	60S ribosomal protein L18	745.404	2	43.73	1193950	43.51	848280	0.92294
LTVCALHVTQTAR	RpL18	60S ribosomal protein L18	490.601	3	26.12	441082	25.98	561270	1.65301
GFLETVELQIGLK	RpL10Ab	60S ribosomal protein L10a-2	723.914	2	52.78	971368	52.52	1026785	1.37315
VCILGDQQHCDEAK	RpL10Ab	60S ribosomal protein L10a-2	558.252	3	18.98	72010	18.85	119507	2.15587
GDVGPGAGEVEFR	RpS10b	40S ribosomal protein S10b	645.31	2	25.28	985914	25.27	757708	0.99836
SETVRPRPAVGGPR	RpS10b	40S ribosomal protein S10b	493.611	3	12.12	3082161	12.12	2287000	0.9639
VAIYEYLFK	RpS10b	40S ribosomal protein S10b	573.316	2	46.67	2373270	46.49	2589560	1.41743
EFNLYANVRPCR	Idh3a	Probable isocitrate dehydrogenase [NAD] subunit alpha, mitochondrial (EC 1.	513.589	3	29.84	848933	29.81	651759	0.99732
FGIPQAAIDSVNTNK	Idh3a	Probable isocitrate dehydrogenase [NAD] subunit alpha, mitochondrial (EC 1.	787.912	2	35.39	2155730	35.25	1591635	0.95912
FGIPQAAIDSVNTNK	Idh3a	Probable isocitrate dehydrogenase [NAD] subunit alpha, mitochondrial (EC 1.	525.611	3	35.39	544207	35.25	192014	0.45834
SLNLALR	Idh3a	Probable isocitrate dehydrogenase [NAD] subunit alpha, mitochondrial (EC 1.	393.745	2	28.83	871937	28.75	602945	0.89829
VAEYAFQYAK	Idh3a	Probable isocitrate dehydrogenase [NAD] subunit alpha, mitochondrial (EC 1.	595.298	2	27.05	2240918	26.93	1780133	1.03193
YLTGDLGGR	Idh3a	Probable isocitrate dehydrogenase [NAD] subunit alpha, mitochondrial (EC 1.	476.248	2	21.45	1896980	21.42	1761025	1.20594
AAGITYIQYSNIAAR	sun	Protein stunted	806.428	2	34.52	1247977	34.41	1191664	1.24042
FAGVDIR	RpS16	40S ribosomal protein S16	389.216	2	25.71	4573283	25.68	6173631	1.75362
LQEPLLLLGK	RpS16	40S ribosomal protein S16	562.358	2	43.68	9036494	43.47	7008865	1.00756
FVNLPIFESTVLNCFVR	Rtf1	RNA polymerase-associated protein Rtf1 (dRtf1)	1028.04	2	65.6	62022	62.58	33158	0.69449
ALEIEPYSIEEAER	CG3224	Zinc finger protein 593 homolog	824.907	2	36.66	666577	36.48	735612	1.43358
FDPYIQQESLYR	mRpL33	39S ribosomal protein L33, mitochondrial (L33mt) (MRP-L33)	779.88	2	33.19	1291543	33.09	470049	0.47278
IMVVLESVVSGHQFNAFR	mRpL33	39S ribosomal protein L33, mitochondrial (L33mt) (MRP-L33)	678.359	3	47.98	1127158	47.81	717883	0.82735
FLYTLVVQDK	RpL38	60S ribosomal protein L38	613.345	2	38.8	2657415	38.5	2241895	1.09592
TSTYAVAIIASAFFFER	ox	Cytochrome b-c1 complex subunit 9 (Complex III subunit 9) (Complex III subu	947.491	2	65.61	437672	65.23	267890	0.79512
AFIVKPIPSPEVIPVIVFINPK	rdgA	Diacylglycerol kinase (DAG kinase) (EC 2.7.1.107)	806.491	3	58.34	7861730	58.25	6537246	1.08019
AFIVKPIPSPEVIPVIVFINPK	rdgA	Diacylglycerol kinase (DAG kinase) (EC 2.7.1.107)	605.12	4	58.3	339552	58.29	268976	1.02903
ALGWGGGYTDEPIGK	rdgA	Diacylglycerol kinase (DAG kinase) (EC 2.7.1.107)	760.873	2	33.66	1454790	33.49	1166978	1.04204
ALHEQGYSLQSVNK	rdgA	Diacylglycerol kinase (DAG kinase) (EC 2.7.1.107)	525.27	3	20.1	844926	20.13	448176	0.68905

FEESISYPK	rdgA	Diacylglycerol kinase (DAG kinase) (EC 2.7.1.107)	550.269	2	25.08	699628	25.06	249796	0.46381
FMHLEK	rdgA	Diacylglycerol kinase (DAG kinase) (EC 2.7.1.107)	410.705	2	11.03	406137	11.1	167691	0.53636
FQHLLNPR	rdgA	Diacylglycerol kinase (DAG kinase) (EC 2.7.1.107)	512.788	2	19.99	2182798	19.93	880908	0.52425
GDVQVNPLEK	rdgA	Diacylglycerol kinase (DAG kinase) (EC 2.7.1.107)	549.793	2	22.75	2434720	22.76	798326	0.42595
GPGSTDEDLQIGSKPIK	rdgA	Diacylglycerol kinase (DAG kinase) (EC 2.7.1.107)	581.304	3	23.2	1491415	23.18	425538	0.37065
LGNYAPIIVPPSWIVK	rdgA	Diacylglycerol kinase (DAG kinase) (EC 2.7.1.107)	884.014	2	52.63	3322956	52.44	1330008	0.51994
NANEIATYLESQER	rdgA	Diacylglycerol kinase (DAG kinase) (EC 2.7.1.107)	819.392	2	35.43	3962934	35.29	2961008	0.97061
NANEIATYLESQER	rdgA	Diacylglycerol kinase (DAG kinase) (EC 2.7.1.107)	546.597	3	35.43	1543644	35.29	934216	0.78618
QVFDLTQGGPK	rdgA	Diacylglycerol kinase (DAG kinase) (EC 2.7.1.107)	595.314	2	28.22	3580063	28.2	2623698	0.95202
SFAAFSQR	rdgA	Diacylglycerol kinase (DAG kinase) (EC 2.7.1.107)	457.23	2	23.28	6655876	23.37	6106585	1.19183
TIPMQVDGEACR	rdgA	Diacylglycerol kinase (DAG kinase) (EC 2.7.1.107)	688.818	2	23.21	1029594	23.29	655361	0.82687
TIPMQVDGEACR	rdgA	Diacylglycerol kinase (DAG kinase) (EC 2.7.1.107)	696.816	2	17.7	628084	17.41	337537	0.69811
VKPSVIEIELLNK	rdgA	Diacylglycerol kinase (DAG kinase) (EC 2.7.1.107)	741.451	2	38.07	1106361	37.96	617198	0.72469
VKPSVIEIELLNK	rdgA	Diacylglycerol kinase (DAG kinase) (EC 2.7.1.107)	494.636	3	38.07	3645006	37.92	2591446	0.92356
VSQDWCFIDSCTAEHYFR	rdgA	Diacylglycerol kinase (DAG kinase) (EC 2.7.1.107)	774.33	3	41.88	500902	41.72	464633	1.20498
VTPNDDVTDDHVDR	rdgA	Diacylglycerol kinase (DAG kinase) (EC 2.7.1.107)	533.241	3	14.64	1483070	14.56	900190	0.78849
YIIASATR	rdgA	Diacylglycerol kinase (DAG kinase) (EC 2.7.1.107)	447.756	2	19.6	1081315	19.55	314198	0.37746
VVYDPQTAEELSSK	ScsbetaA	Succinate--CoA ligase [ADP-forming] subunit beta, mitochondrial (EC 6.2.1.5)	783.388	2	26.05	1092441	25.98	763625	0.90804
ILFAFGPVVEPYTTEHTVVAK	alpha-Est	Carboxylic ester hydrolase (EC 3.1.1.-)	773.419	3	44.7	30930	48.23	75260	3.16087
AAIELNR	CG9492	Uncharacterized protein, isoform D (EC 3.6.1.3) (EC 3.6.4.5)	393.727	2	17.87	27934492	17.87	8147882	0.3789
ELAQQIQQVATEFGSSSYVR	Rm62	Rm62, isoform J	747.71	3	48.34	417102	48.12	508191	1.58273
FGGGGGFGDR	Rm62	Rm62, isoform J	463.709	2	17.67	571805	17.72	448173	1.01817
NTCVFGGAPK	Rm62	Rm62, isoform J	525.755	2	19.91	235668	19.91	262752	1.44833
SNILVATDVAAR	Rm62	Rm62, isoform J	615.346	2	29.95	1490640	29.85	1916326	1.67001
AAIELNR	CG9492	Uncharacterized protein, isoform F (EC 3.6.1.3) (EC 3.6.4.5)	393.727	2	17.87	27934492	17.87	8147882	0.3789
LSEASQAADESER	Tm1	Tropomyosin 1, isoform Q	696.816	2	11.57	343010	11.67	695696	2.63472
AQCPIVER	RpS5b	Ribosomal protein S5b, isoform B	486.75	2	14.71	806831	14.72	445526	0.71732
QAVDVSPLR	RpS5b	Ribosomal protein S5b, isoform B	492.777	2	23.02	630384	23.02	403036	0.83054
ELAQQIQQVATEFGSSSYVR	Rm62	Rm62, isoform L	747.71	3	48.34	417102	48.12	508191	1.58273
FGGGGGFGDR	Rm62	Rm62, isoform L	463.709	2	17.67	571805	17.72	448173	1.01817
NTCVFGGAPK	Rm62	Rm62, isoform L	525.755	2	19.91	235668	19.91	262752	1.44833
SNILVATDVAAR	Rm62	Rm62, isoform L	615.346	2	29.95	1490640	29.85	1916326	1.67001
ELAQQIQQVATEFGSSSYVR	Rm62	Rm62, isoform M	747.71	3	48.34	417102	48.12	508191	1.58273
FGGGGGFGDR	Rm62	Rm62, isoform M	463.709	2	17.67	571805	17.72	448173	1.01817
NTCVFGGAPK	Rm62	Rm62, isoform M	525.755	2	19.91	235668	19.91	262752	1.44833
SNILVATDVAAR	Rm62	Rm62, isoform M	615.346	2	29.95	1490640	29.85	1916326	1.67001
ILFAFGPVVEPYTTEHTVVAK	alpha-Est	Carboxylic ester hydrolase (EC 3.1.1.-)	773.419	3	44.7	30930	48.23	75260	3.16087
IADVVYNASNNELVR	RpS8	40S ribosomal protein S8	838.934	2	31.29	2587695	31.12	2542053	1.27613
IADVVYNASNNELVR	RpS8	40S ribosomal protein S8	559.625	3	31.29	1034232	31.16	1022478	1.28428
ILACISSRPGQCGR	RpS8	40S ribosomal protein S8	787.898	2	20.14	140708	20.13	87291	0.80588
ILACISSRPGQCGR	RpS8	40S ribosomal protein S8	525.601	3	20.1	386803	20.05	432135	1.45128
LETGNFAWASEGVAR	RpS8	40S ribosomal protein S8	804.394	2	35.85	1612600	35.66	1025388	0.82601
NSIVVIDATPFR	RpS8	40S ribosomal protein S8	666.37	2	40.3	3907103	40.11	3186895	1.05958
QWYEAHYVLPLGR	RpS8	40S ribosomal protein S8	544.616	3	39.06	708914	38.88	864527	1.58419
SDGYILEGK	RpS8	40S ribosomal protein S8	491.248	2	21.95	526219	21.94	571255	1.41022
VEQALEDQFTSGR	RpS8	40S ribosomal protein S8	740.357	2	25.93	843576	25.86	462117	0.71162
ELAQQIQQVATEFGSSSYVR	Rm62	Rm62, isoform K	747.71	3	48.34	417102	48.12	508191	1.58273
FGGGGGFGDR	Rm62	Rm62, isoform K	463.709	2	17.67	571805	17.72	448173	1.01817
NTCVFGGAPK	Rm62	Rm62, isoform K	525.755	2	19.91	235668	19.91	262752	1.44833
SNILVATDVAAR	Rm62	Rm62, isoform K	615.346	2	29.95	1490640	29.85	1916326	1.67001
SAFIQVLESSYR	aralar1	Aralar1, isoform E	700.364	2	43.03	477153	42.8	602689	1.64081
LSEASQAADESER	Tm1	Tropomyosin 1, isoform P	696.816	2	11.57	343010	11.67	695696	2.63472
AALDCVELFATEQR	Sin3A	Sin3A, isoform G	811.896	2	40.25	1722570	40.03	1260136	0.9503

AALDCVELFATEQR	Sin3A	Sin3A, isoform G	541.6	3	40.21	619337	39.99	590227	1.23798
ANTADGANTVQIHVTGGGAANNPA	Sin3A	Sin3A, isoform G	928.703	4	26.74	509613	26.67	542107	1.38187
DVSFSEASSK	Sin3A	Sin3A, isoform G	528.746	2	17.44	1467180	17.41	381916	0.33815
DYHAGLQQQAHQR	Sin3A	Sin3A, isoform G	517.919	3	11.22	2585659	11.33	2959684	1.48695
DYHAGLQQQAHQR	Sin3A	Sin3A, isoform G	388.691	4	11.22	864204	11.33	948677	1.42602
ESTATALR	Sin3A	Sin3A, isoform G	424.727	2	11.65	1476544	11.71	969884	0.85329
FHLDDHLGGTSQTIHQR	Sin3A	Sin3A, isoform G	654.657	3	23.08	1395287	23.06	756858	0.70465
FHLDDHLGGTSQTIHQR	Sin3A	Sin3A, isoform G	491.245	4	23.12	3523823	23.06	1769329	0.65225
FLEILHDYQR	Sin3A	Sin3A, isoform G	445.235	3	35.73	2969216	35.43	0	0
GTSHQSAAEYVQDVDLSSCK	Sin3A	Sin3A, isoform G	727.995	3	27.24	493988	27.08	189593	0.49857
ILTPQHGGAQTIAYLPSTTPTATNLK	Sin3A	Sin3A, isoform G	898.82	3	35.8	4998395	35.62	5283785	1.37321
LANPALGGGGNTGR	Sin3A	Sin3A, isoform G	627.831	2	17.75	10453019	17.75	7732981	0.96101
LGASYCALPQSTVPK	Sin3A	Sin3A, isoform G	796.411	2	28.68	1479395	28.59	1263101	1.10911
LHAILCDR	Sin3A	Sin3A, isoform G	499.266	2	19.41	1985761	19.38	633888	0.41468
LLEEQVR	Sin3A	Sin3A, isoform G	443.753	2	17.75	2718818	17.76	2609800	1.24695
MLTEQEVYTQVAK	Sin3A	Sin3A, isoform G	778.387	2	27.81	3209047	27.77	3556038	1.4395
NDILFEK	Sin3A	Sin3A, isoform G	439.735	2	27.05	2341755	27.01	1764770	0.97897
NPFVAVPIVLK	Sin3A	Sin3A, isoform G	598.874	2	46.1	1222769	45.94	767794	0.81569
QFVPDLFFAPR	Sin3A	Sin3A, isoform G	668.856	2	50.52	6756025	50.25	7385320	1.42004
SAHAAAIGNLSAVNTSVSIK	Sin3A	Sin3A, isoform G	956.018	2	31.61	170248	31.53	254171	1.9394
SAHAAAIGNLSAVNTSVSIK	Sin3A	Sin3A, isoform G	637.681	3	31.61	2399426	31.53	2575551	1.39439
SHCIDTPGVIER	Sin3A	Sin3A, isoform G	692.338	2	20.25	307279	20.24	237394	1.0036
SHCIDTPGVIER	Sin3A	Sin3A, isoform G	461.894	3	20.22	598355	20.24	340785	0.73985
SSPGISYATPPLPSGPHGQHNSGS.	Sin3A	Sin3A, isoform G	887.432	3	25.86	488181	25.83	202251	0.53819
TADQINASGWGSASK	Sin3A	Sin3A, isoform G	746.855	2	22.49	436619	22.54	444931	1.32377
TILDDAANLLIHHVK	Sin3A	Sin3A, isoform G	418.99	4	49.12	1380604	49.31	716808	0.67446
TISGTQTVATAVGNLATISQQQPVQ	Sin3A	Sin3A, isoform G	1041.89	3	45.65	3056186	45.35	3369934	1.4324
TYNNNQQQQNHVIGSGLNATR	Sin3A	Sin3A, isoform G	786.383	3	19.45	600429	19.45	504776	1.09209
VLENLQK	Sin3A	Sin3A, isoform G	422.25	2	16.41	3516505	16.41	2648950	0.97855
VTIELLDSEPEEVDKPAALK	Sin3A	Sin3A, isoform G	732.726	3	39.02	4118678	38.81	3130918	0.9875
YAHFQTYVNK	Sin3A	Sin3A, isoform G	635.814	2	19.83	763160	19.77	541027	0.92093
YAHFQTYVNK	Sin3A	Sin3A, isoform G	424.212	3	19.83	1837847	19.81	1859402	1.31428
YVGHAPNQNLTHGHNAK	Sin3A	Sin3A, isoform G	619.975	3	10.09	210214	10.13	163530	1.01055
AIYEQSR	hts	Hu li tai shao, isoform Q (Hu li tai shao, isoform R)	433.722	2	11.43	989317	11.54	662957	0.87051
DATVSQAGDHVVMMGAASK	hts	Hu li tai shao, isoform Q (Hu li tai shao, isoform R)	636.293	3	17.09	884028	17.06	1039090	1.5269
DGGAGASGILQQLSDIVGVPVSR	hts	Hu li tai shao, isoform Q (Hu li tai shao, isoform R)	1098.58	2	60.31	1852831	60.03	1591019	1.11548
DGGAGASGILQQLSDIVGVPVSR	hts	Hu li tai shao, isoform Q (Hu li tai shao, isoform R)	732.725	3	60.31	7576074	60.03	5964562	1.02272
FAAVAAAEDGAATAEK	hts	Hu li tai shao, isoform Q (Hu li tai shao, isoform R)	746.868	2	24.97	13124	25.1	319707	31.6452
LLDLYGWTQGLGAQITAR	hts	Hu li tai shao, isoform Q (Hu li tai shao, isoform R)	988.534	2	53.56	2279326	53.29	1565566	0.89225
LLDLYGWTQGLGAQITAR	hts	Hu li tai shao, isoform Q (Hu li tai shao, isoform R)	659.358	3	53.56	1975907	53.25	1298057	0.85339
LLPVGLDNLVLIPEESR	hts	Hu li tai shao, isoform Q (Hu li tai shao, isoform R)	626.363	3	55.71	938166	55.45	1069288	1.4806
MLDNAGYR	hts	Hu li tai shao, isoform Q (Hu li tai shao, isoform R)	470.221	2	16.11	947899	16.1	1485204	2.03538
NDVELPPAVSSLGYLLEEEELFR	hts	Hu li tai shao, isoform Q (Hu li tai shao, isoform R)	873.778	3	65.17	1366923	64.85	956167	0.90868
SHFVLHSVVHAARPDIR	hts	Hu li tai shao, isoform Q (Hu li tai shao, isoform R)	486.02	4	24.42	11051	23.97	0	0
SSNCMVPINDIR	hts	Hu li tai shao, isoform Q (Hu li tai shao, isoform R)	711.329	2	24.43	633585	24.05	1332102	2.73121
SVHGEYTDTDFSESEAVLQAGTK	hts	Hu li tai shao, isoform Q (Hu li tai shao, isoform R)	824.378	3	32.37	929683	32.29	1063232	1.48565
TGLLPLTK	hts	Hu li tai shao, isoform Q (Hu li tai shao, isoform R)	421.771	2	32.52	2862087	32.37	2725569	1.23708
TGYIYR	hts	Hu li tai shao, isoform Q (Hu li tai shao, isoform R)	386.703	2	16.34	1779020	16.29	796815	0.58183
VEVLETGTPDPK	hts	Hu li tai shao, isoform Q (Hu li tai shao, isoform R)	642.838	2	23.08	3487670	23.1	2329013	0.86748
GATALGPATTSGASER	PIP5K59E	Phosphatidylinositol 4-phosphate 5-kinase at 59B, isoform F (EC 2.7.1.-) (EC	723.863	2	16.98	232423	17.03	258407	1.44427
VAPVEAVTISSNR	mod	Modulo, isoform C	671.87	2	25.93	637081	25.91	601323	1.22613
NFPALTYFR	l(2)01289	Lethal (2) 01289, isoform M	564.795	2	42.88	433584	42.79	472450	1.41548
GATALGPATTSGASER	PIP5K59E	Phosphatidylinositol 4-phosphate 5-kinase at 59B, isoform H (EC 2.7.1.-) (EC	723.863	2	16.98	232423	17.03	258407	1.44427

NFPALTYFR	l(2)01289	Lethal (2) 01289, isoform L	564.795	2	42.88	433584	42.79	472450	1.41548
AIYEQSR	hts	Hu li tai shao, isoform O (Hu li tai shao, isoform S)	433.722	2	11.43	989317	11.54	662957	0.87051
DATVSQAGDHVVMMGAASK	hts	Hu li tai shao, isoform O (Hu li tai shao, isoform S)	636.293	3	17.09	884028	17.06	1039090	1.5269
DGGAGASGILQQLSDIVGVPVSR	hts	Hu li tai shao, isoform O (Hu li tai shao, isoform S)	1098.58	2	60.31	1852831	60.03	1591019	1.11548
DGGAGASGILQQLSDIVGVPVSR	hts	Hu li tai shao, isoform O (Hu li tai shao, isoform S)	732.725	3	60.31	7576074	60.03	5964562	1.02272
FAAVAAAEDGAATAEK	hts	Hu li tai shao, isoform O (Hu li tai shao, isoform S)	746.868	2	24.97	13124	25.1	319707	31.6452
LLDLYGWTQGLGAQITAR	hts	Hu li tai shao, isoform O (Hu li tai shao, isoform S)	988.534	2	53.56	2279326	53.29	1565566	0.89225
LLDLYGWTQGLGAQITAR	hts	Hu li tai shao, isoform O (Hu li tai shao, isoform S)	659.358	3	53.56	1975907	53.25	1298057	0.85339
LLPVGLDNLVLIPEESR	hts	Hu li tai shao, isoform O (Hu li tai shao, isoform S)	626.363	3	55.71	938166	55.45	1069288	1.4806
MLDNAGYR	hts	Hu li tai shao, isoform O (Hu li tai shao, isoform S)	470.221	2	16.11	947899	16.1	1485204	2.03538
NDVELPPAVSSLGYLLEEEELFR	hts	Hu li tai shao, isoform O (Hu li tai shao, isoform S)	873.778	3	65.17	1366923	64.85	956167	0.90868
SHFVLHSVVHAARPDIR	hts	Hu li tai shao, isoform O (Hu li tai shao, isoform S)	486.02	4	24.42	11051	23.97	0	0
SSNCMVPINDIR	hts	Hu li tai shao, isoform O (Hu li tai shao, isoform S)	711.329	2	24.43	633585	24.05	1332102	2.73121
SVHGEYTDTDFSESEAVLQAGTK	hts	Hu li tai shao, isoform O (Hu li tai shao, isoform S)	824.378	3	32.37	929683	32.29	1063232	1.48565
TGLLPLTK	hts	Hu li tai shao, isoform O (Hu li tai shao, isoform S)	421.771	2	32.52	2862087	32.37	2725569	1.23708
TGYIYR	hts	Hu li tai shao, isoform O (Hu li tai shao, isoform S)	386.703	2	16.34	1779020	16.29	796815	0.58183
VEVLETGTPDPK	hts	Hu li tai shao, isoform O (Hu li tai shao, isoform S)	642.838	2	23.08	3487670	23.1	2329013	0.86748
FLYTLVVQDK	RpL38	GEO09618p1 (Ribosomal protein L38, isoform B)	613.345	2	38.8	2657415	38.5	2241895	1.09592
SVTETGVELSNEER	14-3-3zet	14-3-3zeta, isoform K (14-3-3zeta, isoform L)	775.371	2	21.6	716717	21.65	1301291	2.35857
YLAEVATGDAR	14-3-3zet	14-3-3zeta, isoform K (14-3-3zeta, isoform L)	583.296	2	20.89	1053151	20.85	1412304	1.74205
SAGLTPDLSNFTETR	sbb	Scribbler, isoform J	804.897	2	36.67	741872	36.56	971672	1.70143
TSPGFGSVGVGAASSK	sbb	Scribbler, isoform J	704.857	2	24.77	1117813	24.76	1320748	1.53488
ASPSLYGTAER	jbug	Jitterbug, isoform M	576.288	2	18.78	139955	18.74	220071	2.04266
ASPSLYGTAER	jbug	Jitterbug, isoform N	576.288	2	18.78	139955	18.74	220071	2.04266
TVIIPSDVQVVSTK	CG16779	Uncharacterized protein, isoform D	743.43	2	34.9	984275	34.74	541335	0.71445
TVIIPSDVQVVSTK	CG16779	Uncharacterized protein, isoform C	743.43	2	34.9	984275	34.74	541335	0.71445
LQNLEDTNAGLNAR	LamC	Lamin C, isoform B	764.889	2	23.51	123071	23.45	281522	2.97152
DAVTYTEHAK	His4r	Histone H4	567.775	2	11.73	1744246	11.83	635649	0.4734
DNIQGITKPAIR	His4r	Histone H4	442.589	3	22.72	3459819	22.72	2375618	0.89196
ISGLIYEETR	His4r	Histone H4	590.814	2	28.26	8240435	28.2	5262581	0.8296
VFLENVIR	His4r	Histone H4	495.293	2	37.01	7324526	36.84	3166374	0.56157
LSEASQAADESER	Tm1	Tropomyosin 1, isoform S	696.816	2	11.57	343010	11.67	695696	2.63472
ASQELGLR	HtrA2	HTRA2-related serine protease, isoform B (EC 3.4.-.-) (EC 3.4.21.-)	437.243	2	17.76	957616	17.79	498328	0.676
TFPATIEDVDQTSDLATLR	HtrA2	HTRA2-related serine protease, isoform B (EC 3.4.-.-) (EC 3.4.21.-)	1047.02	2	42.81	1456762	42.55	805342	0.71815
TLDIVILR	HtrA2	HTRA2-related serine protease, isoform B (EC 3.4.-.-) (EC 3.4.21.-)	471.803	2	41.14	1392585	40.98	994282	0.92749
VTAGISFAIPIDYVK	HtrA2	HTRA2-related serine protease, isoform B (EC 3.4.-.-) (EC 3.4.21.-)	797.448	2	50.05	817270	49.8	294920	0.46877
AGSEPYVPPPSYWNR	Mf	Myofilin, isoform O	860.41	2	33.23	748487	33.06	1566838	2.71933
AIYDHHYSR	Mf	Myofilin, isoform O	581.275	2	11.31	475566	11.41	651391	1.77932
AIYDHHYSR	Mf	Myofilin, isoform O	387.853	3	11.31	2895978	11.41	4746762	2.12924
ASRPYSSYLDSPSYR	Mf	Myofilin, isoform O	874.916	2	25.08	1194562	25.03	1397150	1.51935
ASRPYSSYLDSPSYR	Mf	Myofilin, isoform O	583.613	3	25.12	5487893	25.03	8501748	2.01245
ASSLEPLDELFER	Mf	Myofilin, isoform O	753.378	2	48.45	4498040	48.19	5102070	1.47348
DKPLTPNSLVPLEYEPEDK	Mf	Myofilin, isoform O	728.707	3	38.98	567161	38.81	728559	1.66871
DSYLSPVK	Mf	Myofilin, isoform O	454.74	2	22.04	1523720	22.02	1430945	1.21994
DTYGYSPR	Mf	Myofilin, isoform O	479.717	2	14.86	2640775	14.83	2840777	1.39742
FWQSYIR	Mf	Myofilin, isoform O	500.256	2	32.37	739255	32.25	1687659	2.9656
GGPSVFDR	Mf	Myofilin, isoform O	417.709	2	19.86	5727839	19.85	7157511	1.62328
LLAELNK	Mf	Myofilin, isoform O	400.747	2	22.38	846200	22.35	1253332	1.92405
LPIFHPR	Mf	Myofilin, isoform O	440.264	2	24.47	512417	24.48	315832	0.80067
NHLEMIGR	Mf	Myofilin, isoform O	493.248	2	12.87	1554021	12.91	1080925	0.90357
SASPFRPAR	Mf	Myofilin, isoform O	494.77	2	13.46	1272464	13.42	1443660	1.47381
SIYDEPATANER	Mf	Myofilin, isoform O	683.318	2	18.42	9216683	18.42	15046478	2.12072
YGLYLR	Mf	Myofilin, isoform O	392.721	2	28.26	5499678	28.16	8903475	2.10303

LSEASQAADESER	Tm1	Tropomyosin 1, isoform R	696.816	2	11.57	343010	11.67	695696	2.63472
DITLFHAMNILHK	MTA1-like	Metastasis associated 1-like, isoform D	518.282	3	43.88	2053379	43.78	955106	0.60423
DLPNPLVQLADK	MTA1-like	Metastasis associated 1-like, isoform D	661.869	2	44.73	4867516	44.55	2835007	0.7566
ELTIIPTQATSTIR	MTA1-like	Metastasis associated 1-like, isoform D	772.438	2	37.83	11123584	37.63	12065403	1.40903
HYAQAHGIAISSGSPRPIMK	MTA1-like	Metastasis associated 1-like, isoform D	531.031	4	22.34	2400440	22.14	3061997	1.65705
HYAQAHGIAISSGSPRPIMK	MTA1-like	Metastasis associated 1-like, isoform D	713.037	3	18.86	1475021	18.76	1372104	1.2084
HYAQAHGIAISSGSPRPIMK	MTA1-like	Metastasis associated 1-like, isoform D	535.03	4	18.86	8277828	18.73	11433715	1.79429
IDQFLVVSR	MTA1-like	Metastasis associated 1-like, isoform D	538.809	2	33.15	2031489	33.09	2200379	1.40704
ILSAVDLR	MTA1-like	Metastasis associated 1-like, isoform D	443.771	2	29.06	3480815	28.94	3278001	1.22335
LCLSCWEYWR	MTA1-like	Metastasis associated 1-like, isoform D	736.826	2	45.03	1100791	44.85	1003854	1.18464
LGAPGSGPHEWLVLTPK	MTA1-like	Metastasis associated 1-like, isoform D	586.989	3	38.45	4162383	38.27	5011628	1.56408
LLLLKPK	MTA1-like	Metastasis associated 1-like, isoform D	412.802	2	25.48	7404797	25.43	7870932	1.38081
QIIEYYYMWK	MTA1-like	Metastasis associated 1-like, isoform D	718.85	2	43.1	621851	42.87	464183	0.96967
QPSLHMSAAAASR	MTA1-like	Metastasis associated 1-like, isoform D	448.223	3	11.24	631709	11.36	455066	0.93579
QSAYAINAMLVK	MTA1-like	Metastasis associated 1-like, isoform D	654.853	2	35.58	1490566	35.41	2100155	1.8303
QSAYAINAMLVK	MTA1-like	Metastasis associated 1-like, isoform D	662.85	2	29.32	5849027	29.21	6763609	1.50216
QVESIPATQIR	MTA1-like	Metastasis associated 1-like, isoform D	621.346	2	23.95	29583954	23.96	39601180	1.7389
SIGTFAR	MTA1-like	Metastasis associated 1-like, isoform D	376.208	2	18.93	21987752	18.88	27082934	1.60006
SSQWNSVSSGHSTSR	MTA1-like	Metastasis associated 1-like, isoform D	536.245	3	13.07	1701975	13.07	2157652	1.64684
SSSAASTPTATLAGLATTPTAVVDL	MTA1-like	Metastasis associated 1-like, isoform D	968.816	3	46.62	1694407	46.37	1458500	1.11818
TAFYLHTNPMTR	MTA1-like	Metastasis associated 1-like, isoform D	726.359	2	28.65	3357775	28.53	4332503	1.67614
TAFYLHTNPMTR	MTA1-like	Metastasis associated 1-like, isoform D	484.575	3	28.65	6289877	28.53	6959267	1.43729
TAFYLHTNPMTR	MTA1-like	Metastasis associated 1-like, isoform D	734.356	2	22.69	3508484	22.72	3475736	1.28691
TAFYLHTNPMTR	MTA1-like	Metastasis associated 1-like, isoform D	489.906	3	22.69	11884596	22.72	15231011	1.66482
TPVSEEQAAQAVLDPSIAALDEER	MTA1-like	Metastasis associated 1-like, isoform D	847.089	3	45.9	2579368	45.66	1415875	0.71307
VGDYVYVETTPNSPYLIR	MTA1-like	Metastasis associated 1-like, isoform D	1043.53	2	39.99	19490416	39.8	21930646	1.46168
VGDYVYVETTPNSPYLIR	MTA1-like	Metastasis associated 1-like, isoform D	696.021	3	39.99	8430076	39.8	9468280	1.45902
VMCFYR	MTA1-like	Metastasis associated 1-like, isoform D	446.196	2	18.26	2536584	18.3	3037729	1.55569
YQCDIPAK	MTA1-like	Metastasis associated 1-like, isoform D	497.737	2	14.9	10434692	14.9	5215218	0.64925
AAVSGAVEQATLR	P5cr-2	Pyrroline-5-carboxylate reductase (EC 1.5.1.2)	636.849	2	23.08	549773	23.1	334382	0.7901
IVIGAQPNDPVISEITDALLTSNYR	Stat92E	Signal transducer and activator of transcription	900.48	3	61.16	88166	60.86	149631	2.20467
SLTIDNLDFLYEK	Stat92E	Signal transducer and activator of transcription	785.904	2	48.67	137787	48.23	187413	1.76691
GYAFVTFTNR	Syp	Syncrip, isoform N (Syncrip, isoform P)	588.296	2	35.44	514118	35.33	604713	1.52795
EVSGDASEAALLK	Atpalpha	Sodium/potassium-transporting ATPase subunit alpha	645.333	2	25.45	660865	25.35	279051	0.54852
IATLCNR	Atpalpha	Sodium/potassium-transporting ATPase subunit alpha	424.226	2	14.34	183981	14.32	209877	1.48188
LIIVEGCQR	Atpalpha	Sodium/potassium-transporting ATPase subunit alpha	544.3	2	24.31	677846	24.36	746923	1.43142
LNIPVSEVNPR	Atpalpha	Sodium/potassium-transporting ATPase subunit alpha	619.349	2	30.64	1527141	30.51	1174989	0.99949
NLAFFSTNAVEGTAK	Atpalpha	Sodium/potassium-transporting ATPase subunit alpha	785.399	2	38.55	1771624	38.01	15711079	11.5201
YHTEIVFAR	Atpalpha	Sodium/potassium-transporting ATPase subunit alpha	568.298	2	25.19	792742	25.03	558537	0.91526
YHTEIVFAR	Atpalpha	Sodium/potassium-transporting ATPase subunit alpha	379.201	3	25.23	368207	25.07	375193	1.32369
IVIGAQPNDPVISEITDALLTSNYR	Stat92E	Signal transducer and activator of transcription	900.48	3	61.16	88166	60.86	149631	2.20467
SLTIDNLDFLYEK	Stat92E	Signal transducer and activator of transcription	785.904	2	48.67	137787	48.23	187413	1.76691
GYAFVTFTNR	Syp	Syncrip, isoform M	588.296	2	35.44	514118	35.33	604713	1.52795
GYAFVTFTNR	Syp	Syncrip, isoform R	588.296	2	35.44	514118	35.33	604713	1.52795
HVINFDLPSDVEEYVHR	bel	Belle, isoform B (EC 3.6.1.3)	690.341	3	42.33	52327	42.25	363307	9.01925
SGDCPILVATAVAAR	bel	Belle, isoform B (EC 3.6.1.3)	750.896	2	37.72	71006	37.48	451861	8.2667
ACHDSGEELDDISLPK	MBD-like	Methyl-CpG binding domain protein-like, isoform C	893.402	2	30.72	859129	30.56	663495	1.00323
ACHDSGEELDDISLPK	MBD-like	Methyl-CpG binding domain protein-like, isoform C	595.937	3	30.68	4227121	30.52	3334801	1.02482
TDVSLVPPIR	MBD-like	Methyl-CpG binding domain protein-like, isoform C	548.822	2	35.16	14387869	35.02	9195601	0.83024
TVGPNVNEQTVLQSVATALHMLNA	MBD-like	Methyl-CpG binding domain protein-like, isoform C	847.937	4	61.34	1644970	61.29	1228563	0.9702
TVGPNVNEQTVLQSVATALHMLNA	MBD-like	Methyl-CpG binding domain protein-like, isoform C	851.936	4	55.74	1113659	55.85	795373	0.92777
IVIGAQPNDPVISEITDALLTSNYR	Stat92E	Signal transducer and activator of transcription	900.48	3	61.16	88166	60.86	149631	2.20467
SLTIDNLDFLYEK	Stat92E	Signal transducer and activator of transcription	785.904	2	48.67	137787	48.23	187413	1.76691

GYAFVTFTNR	Syp	Syncrip, isoform I	588.296	2	35.44	514118	35.33	604713	1.52795	
GYAFVTFTNR	Syp	Syncrip, isoform O	588.296	2	35.44	514118	35.33	604713	1.52795	
AGLQFPVGR	His2Av	Histone H2A	472.769	2	31.56	98633592	31.42	106838928	1.40711	
ATIAGGGVIPHIHK	His2Av	Histone H2A	685.899	2	24.2	4969387	24.16	4711632	1.23166	
ATIAGGGVIPHIHK	His2Av	Histone H2A	457.602	3	24.23	19229148	24.16	22945442	1.5501	
GDEELDSLIK	His2Av	Histone H2A	559.782	2	33.01	2609860	32.91	1105949	0.55048	
GNVILSQAY	His2Av	Histone H2A	482.759	2	31.72	3533263	31.6	3759713	1.3823	
HLQLAIR	His2Av	Histone H2A	425.767	2	22.72	21451580	22.66	9817742	0.59453	
VGATAAVYSAAILEYLTAEVLELAGI	His2Av	Histone H2A	965.85	3	65.73	7579277	65.74	7007764	1.20109	
VGATAAVYSAAILEYLTAEVLELAGI	His2Av	Histone H2A	724.639	4	65.73	1991616	65.74	2155629	1.40602	
ISGNHLTEIPDDAFTGLER	chp	Chaoptin, isoform C	695.68	3	38.69	287292	38.5	281624	1.27341	
LLLTDNILSEIPYDALGPLK	chp	Chaoptin, isoform C	1099.62	2	62.1	575340	61.86	486645	1.09878	
LLLTDNILSEIPYDALGPLK	chp	Chaoptin, isoform C	733.415	3	62.05	449702	61.83	498658	1.44046	
GYAFVTFTNR	Syp	Syncrip, isoform K	588.296	2	35.44	514118	35.33	604713	1.52795	
STFTVDITPLILAAHR	trp	Transient receptor potential, isoform B	585.665	3	49.88	352438	49.67	830498	3.0611	
GYAFVTFTNR	Syp	Syncrip, isoform Q	588.296	2	35.44	514118	35.33	604713	1.52795	
LLTATESEVATQNR	Tm2	Tropomyosin 2, isoform E (Tropomyosin 2, isoform F) (Tropomyosin 2, isoforn	766.899	2	21.98	140941	22.02	442612	4.07951	
ISGNHLTEIPDDAFTGLER	chp	Chaoptin, isoform F	695.68	3	38.69	287292	38.5	281624	1.27341	
LLLTDNILSEIPYDALGPLK	chp	Chaoptin, isoform F	1099.62	2	62.1	575340	61.86	486645	1.09878	
LLLTDNILSEIPYDALGPLK	chp	Chaoptin, isoform F	733.415	3	62.05	449702	61.83	498658	1.44046	
VQVGHAPSPNLK	tau	Microtubule-associated protein	416.235	3	17.44	993769	17.41	1404801	1.83633	
SLTIDNLDFLYEK	Stat92E	Signal transducer and activator of transcription	785.904	2	48.67	137787	48.23	187413	1.76691	
SLTIDNLDFLYEK	Stat92E	Signal transducer and activator of transcription	785.904	2	48.67	137787	48.23	187413	1.76691	
GYAFVTFTNR	Syp	Syncrip, isoform J	588.296	2	35.44	514118	35.33	604713	1.52795	
SAFIQVLESSYR	aralar1	Aralar1, isoform F	700.364	2	43.03	477153	42.8	602689	1.64081	
ISGNHLTEIPDDAFTGLER	chp	Chaoptin, isoform D	695.68	3	38.69	287292	38.5	281624	1.27341	
LLLTDNILSEIPYDALGPLK	chp	Chaoptin, isoform D	1099.62	2	62.1	575340	61.86	486645	1.09878	
LLLTDNILSEIPYDALGPLK	chp	Chaoptin, isoform D	733.415	3	62.05	449702	61.83	498658	1.44046	
ISGNHLTEIPDDAFTGLER	chp	Chaoptin, isoform E	695.68	3	38.69	287292	38.5	281624	1.27341	
LLLTDNILSEIPYDALGPLK	chp	Chaoptin, isoform E	1099.62	2	62.1	575340	61.86	486645	1.09878	
LLLTDNILSEIPYDALGPLK	chp	Chaoptin, isoform E	733.415	3	62.05	449702	61.83	498658	1.44046	
VQVGHAPSPNLK	tau	Microtubule-associated protein	416.235	3	17.44	993769	17.41	1404801	1.83633	
RPATSGTGSVASSTAR	Map205	Microtubule-associated protein 205, isoform C	753.387	2	9.41	74073	9.52	32583	0.57142	
EVDGSGTIASTNR	CG8635	Uncharacterized protein, isoform B	653.815	2	13.51	1132231	13.47	535526	0.61442	
FFLEAVEK	CG8635	Uncharacterized protein, isoform B	491.766	2	36.01	903927	35.86	657062	0.94427	
FIQQVQK	CG8635	Uncharacterized protein, isoform B	445.758	2	14.57	1436865	14.52	689451	0.62332	
LLDQATEAAK	CG8635	Uncharacterized protein, isoform B	530.288	2	17.48	1942370	17.48	2050056	1.37106	
VTLESFLAWK	CG8635	Uncharacterized protein, isoform B	597.332	2	50.62	626652	50.28	644776	1.33661	
EAVGAFIVFDVTR	Rab32	Rab32, isoform H (Rab32, isoform J)	712.383	2	49.81	1401007	49.56	1390509	1.28931	
AALDCVELFATEQR	Sin3A	Sin3A, isoform H	811.896	2	40.25	1722570	40.03	1260136	0.9503	
AALDCVELFATEQR	Sin3A	Sin3A, isoform H	541.6	3	40.21	619337	39.99	590227	1.23798	
ANTADGANTVQIHVTGGGAANNPA	Sin3A	Sin3A, isoform H	928.703	4	26.74	509613	26.67	542107	1.38187	
DVSFSEASSK	Sin3A	Sin3A, isoform H	528.746	2	17.44	1467180	17.41	381916	0.33815	
DYHAGLQQQAHQR	Sin3A	Sin3A, isoform H	517.919	3	11.22	2585659	11.33	2959684	1.48695	
DYHAGLQQQAHQR	Sin3A	Sin3A, isoform H	388.691	4	11.22	864204	11.33	948677	1.42602	
ESTATALR	Sin3A	Sin3A, isoform H	424.727	2	11.65	1476544	11.71	969884	0.85329	
FHLDDHLGGTSQTIHQR	Sin3A	Sin3A, isoform H	654.657	3	23.08	1395287	23.06	756858	0.70465	
FHLDDHLGGTSQTIHQR	Sin3A	Sin3A, isoform H	491.245	4	23.12	3523823	23.06	1769329	0.65225	
FLEILHDYQR	Sin3A	Sin3A, isoform H	445.235	3	35.73	2969216	35.43	0	0	
GTSHQSAAEYVQDVDLSSCK	Sin3A	Sin3A, isoform H	727.995	3	27.24	493988	27.08	189593	0.49857	
ILTPQHGGAQTIAYLPSTTPTATNLK	Sin3A	Sin3A, isoform H	898.82	3	35.8	4998395	35.62	5283785	1.37321	
LANPALGGGGNTGR	Sin3A	Sin3A, isoform H	627.831	2	17.75	10453019	17.75	7732981	0.96101	
LGASYCALPQSTVPK	Sin3A	Sin3A, isoform H	796.411	2	28.68	1479395	28.59	1263101	1.10911	

LHAILCDR	Sin3A	Sin3A, isoform H	499.266	2	19.41	1985761	19.38	633888	0.41468
LLEEQVR	Sin3A	Sin3A, isoform H	443.753	2	17.75	2718818	17.76	2609800	1.24695
MLTEQEVYTQVAK	Sin3A	Sin3A, isoform H	778.387	2	27.81	3209047	27.77	3556038	1.4395
NDILFEK	Sin3A	Sin3A, isoform H	439.735	2	27.05	2341755	27.01	1764770	0.97897
NPFVAVPIVLK	Sin3A	Sin3A, isoform H	598.874	2	46.1	1222769	45.94	767794	0.81569
QFVPDLFFAPR	Sin3A	Sin3A, isoform H	668.856	2	50.52	6756025	50.25	7385320	1.42004
SAHAAAIGNLSAVNTSVSIK	Sin3A	Sin3A, isoform H	956.018	2	31.61	170248	31.53	254171	1.9394
SAHAAAIGNLSAVNTSVSIK	Sin3A	Sin3A, isoform H	637.681	3	31.61	2399426	31.53	2575551	1.39439
SHCIDTPGVIER	Sin3A	Sin3A, isoform H	692.338	2	20.25	307279	20.24	237394	1.0036
SHCIDTPGVIER	Sin3A	Sin3A, isoform H	461.894	3	20.22	598355	20.24	340785	0.73985
SSPGISYATPPLPSGPHGQHNSGS	Sin3A	Sin3A, isoform H	887.432	3	25.86	488181	25.83	202251	0.53819
TADQINASGWGSASK	Sin3A	Sin3A, isoform H	746.855	2	22.49	436619	22.54	444931	1.32377
TILDDAANLLIHHVK	Sin3A	Sin3A, isoform H	418.99	4	49.12	1380604	49.31	716808	0.67446
TISGTQTVATAVGNLATISQQQPVQ	Sin3A	Sin3A, isoform H	1041.89	3	45.65	3056186	45.35	3369934	1.4324
TYNNNQQQQNHVIGSGLNATR	Sin3A	Sin3A, isoform H	786.383	3	19.45	600429	19.45	504776	1.09209
VLENLQK	Sin3A	Sin3A, isoform H	422.25	2	16.41	3516505	16.41	2648950	0.97855
VTIELLDSEPEEVDKPAALK	Sin3A	Sin3A, isoform H	732.726	3	39.02	4118678	38.81	3130918	0.9875
YAHFQTYVNK	Sin3A	Sin3A, isoform H	635.814	2	19.83	763160	19.77	541027	0.92093
YAHFQTYVNK	Sin3A	Sin3A, isoform H	424.212	3	19.83	1837847	19.81	1859402	1.31428
YVGHAPNQNLTHGHNAK	Sin3A	Sin3A, isoform H	619.975	3	10.09	210214	10.13	163530	1.01055
AALDCVELFATEQR	Sin3A	Sin3A, isoform I	811.896	2	40.25	1722570	40.03	1260136	0.9503
AALDCVELFATEQR	Sin3A	Sin3A, isoform I	541.6	3	40.21	619337	39.99	590227	1.23798
ANTADGANTVQIHVTGGGAANNPA	Sin3A	Sin3A, isoform I	928.703	4	26.74	509613	26.67	542107	1.38187
DVSFSEASSK	Sin3A	Sin3A, isoform I	528.746	2	17.44	1467180	17.41	381916	0.33815
DYHAGLQQQAHQR	Sin3A	Sin3A, isoform I	517.919	3	11.22	2585659	11.33	2959684	1.48695
DYHAGLQQQAHQR	Sin3A	Sin3A, isoform I	388.691	4	11.22	864204	11.33	948677	1.42602
ESTATALR	Sin3A	Sin3A, isoform I	424.727	2	11.65	1476544	11.71	969884	0.85329
FHLDDHLGGTSQTIHQR	Sin3A	Sin3A, isoform I	654.657	3	23.08	1395287	23.06	756858	0.70465
FHLDDHLGGTSQTIHQR	Sin3A	Sin3A, isoform I	491.245	4	23.12	3523823	23.06	1769329	0.65225
FLEILHDYQR	Sin3A	Sin3A, isoform I	445.235	3	35.73	2969216	35.43	0	0
GTSHQSAAEYVQDVDLSSCK	Sin3A	Sin3A, isoform I	727.995	3	27.24	493988	27.08	189593	0.49857
ILTPQHGGAQTIAYLPSTTPTATNLK	Sin3A	Sin3A, isoform I	898.82	3	35.8	4998395	35.62	5283785	1.37321
LANPALGGGGNTGR	Sin3A	Sin3A, isoform I	627.831	2	17.75	10453019	17.75	7732981	0.96101
LGASYCALPQSTVPK	Sin3A	Sin3A, isoform I	796.411	2	28.68	1479395	28.59	1263101	1.10911
LHAILCDR	Sin3A	Sin3A, isoform I	499.266	2	19.41	1985761	19.38	633888	0.41468
MLTEQEVYTQVAK	Sin3A	Sin3A, isoform I	778.387	2	27.81	3209047	27.77	3556038	1.4395
NDILFEK	Sin3A	Sin3A, isoform I	439.735	2	27.05	2341755	27.01	1764770	0.97897
NPFVAVPIVLK	Sin3A	Sin3A, isoform I	598.874	2	46.1	1222769	45.94	767794	0.81569
QFVPDLFFAPR	Sin3A	Sin3A, isoform I	668.856	2	50.52	6756025	50.25	7385320	1.42004
SAHAAAIGNLSAVNTSVSIK	Sin3A	Sin3A, isoform I	956.018	2	31.61	170248	31.53	254171	1.9394
SAHAAAIGNLSAVNTSVSIK	Sin3A	Sin3A, isoform I	637.681	3	31.61	2399426	31.53	2575551	1.39439
SHCIDTPGVIER	Sin3A	Sin3A, isoform I	692.338	2	20.25	307279	20.24	237394	1.0036
SHCIDTPGVIER	Sin3A	Sin3A, isoform I	461.894	3	20.22	598355	20.24	340785	0.73985
SSPGISYATPPLPSGPHGQHNSGS	Sin3A	Sin3A, isoform I	887.432	3	25.86	488181	25.83	202251	0.53819
TILDDAANLLIHHVK	Sin3A	Sin3A, isoform I	418.99	4	49.12	1380604	49.31	716808	0.67446
TISGTQTVATAVGNLATISQQQPVQ	Sin3A	Sin3A, isoform I	1041.89	3	45.65	3056186	45.35	3369934	1.4324
TYNNNQQQQNHVIGSGLNATR	Sin3A	Sin3A, isoform I	786.383	3	19.45	600429	19.45	504776	1.09209
VLENLQK	Sin3A	Sin3A, isoform I	422.25	2	16.41	3516505	16.41	2648950	0.97855
VTIELLDSEPEEVDKPAALK	Sin3A	Sin3A, isoform I	732.726	3	39.02	4118678	38.81	3130918	0.9875
YVGHAPNQNLTHGHNAK	Sin3A	Sin3A, isoform I	619.975	3	10.09	210214	10.13	163530	1.01055
DGSLNYIEENDEVLVAGFGR	RpS23	GEO04460p1 (Ribosomal protein S23, isoform B)	1099.02	2	48.42	759053	48.15	930050	1.59168
DGSLNYIEENDEVLVAGFGR	RpS23	GEO04460p1 (Ribosomal protein S23, isoform B)	733.018	3	48.42	744083	48.11	1097192	1.91551
ITAFVPR	RpS23	GEO04460p1 (Ribosomal protein S23, isoform B)	402.242	2	24.96	1909159	24.94	2341284	1.59307

VANVSLLALYK	RpS23	GEO04460p1 (Ribosomal protein S23, isoform B)	595.861	2	41.4	2813053	41.22	3213274	1.48386
EQQYISSSVDPK	cora	Coracle, isoform F	690.836	2	19.72	1077568	19.73	711194	0.85736
IAPNQTAELEDK	cora	Coracle, isoform F	664.838	2	18.46	483773	18.46	266610	0.71591
ITIQSDGDPIDHDK	cora	Coracle, isoform F	518.586	3	21.49	444280	21.51	465036	1.35973
LDDTLGQIVCITPQDNPVVELTVITS	cora	Coracle, isoform F	999.525	3	60.96	477920	60.76	355750	0.96697
LGKPVSTPTVVK	cora	Coracle, isoform F	409.255	3	19.33	1398453	19.26	958082	0.88997
LGLVVTSGLGDNQQDQNLDEAAR	cora	Coracle, isoform F	805.07	3	37.08	2956134	36.91	3245204	1.42607
LIEVGQQIDPIVEVTSLAGK	cora	Coracle, isoform F	1055.09	2	52.4	1797413	52.06	1009797	0.72981
LIEVGQQIDPIVEVTSLAGK	cora	Coracle, isoform F	703.732	3	52.36	1187434	52.06	855614	0.93603
LILNYAQIDPSHFGK	cora	Coracle, isoform F	572.642	3	39.99	1926732	39.77	2328472	1.5699
LLQDPLLSPTTR	cora	Coracle, isoform F	677.39	2	36.71	1818393	36.6	2546599	1.81926
LQGSAVDAAAVPLSDSQK	cora	Coracle, isoform F	878.957	2	28.08	902207	28	1209649	1.74171
MFPVFGSTYR	cora	Coracle, isoform F	610.792	2	35.32	1647098	35.1	1475401	1.16362
NIDGVTHNVEEEVR	cora	Coracle, isoform F	805.892	2	25.19	772422	25.18	383894	0.64562
NIDGVTHNVEEEVR	cora	Coracle, isoform F	537.597	3	25.23	1472968	25.14	947257	0.8354
QAQVQTTTETVPITR	cora	Coracle, isoform F	836.947	2	22.76	1243364	22.75	1875309	1.95928
QAQVQTTTETVPITR	cora	Coracle, isoform F	558.3	3	22.72	338192	22.75	517745	1.98873
SLGVLNLDTGLLDTK	cora	Coracle, isoform F	779.938	2	48.49	4006855	48.22	3396143	1.10104
SPLFTTSATTGPHVESTR	cora	Coracle, isoform F	630.318	3	26.47	4223504	26.38	3945529	1.21354
SSSSSHGKPALAR	cora	Coracle, isoform F	642.836	2	8.36	115696	8.51	27955	0.31388
SSSSSHGKPALAR	cora	Coracle, isoform F	428.893	3	8.36	446429	8.49	229562	0.66799
TAQVETSGGQFDPK	cora	Coracle, isoform F	732.852	2	18.64	2712306	18.61	2930955	1.40376
TDTWPLTFAVK	cora	Coracle, isoform F	639.84	2	45.17	2241253	44.93	1441517	0.83551
TGNLHLVSGVADPK	cora	Coracle, isoform F	469.925	3	24.12	894256	24.12	482494	0.70089
TGYVITIYGYLDPK	cora	Coracle, isoform F	801.924	2	47.28	1483311	47.07	1539226	1.34801
TQAESTNTPVDR	cora	Coracle, isoform F	659.815	2	10.52	427177	10.65	96821	0.29443
TTVTHTMTLTGEIDPVTGR	cora	Coracle, isoform F	677.346	3	32.9	1242941	32.67	1420656	1.48478
TVYVYNMIDPVTGEIVQVDPNDPR	cora	Coracle, isoform F	912.118	3	48.94	447629	48.63	338096	0.98117
VTLLDGSLLDVSIDR	cora	Coracle, isoform F	808.449	2	50.48	2980908	50.17	1512386	0.65908
VVLGEDTPGFSGHGEIISTQTVSSK	cora	Coracle, isoform F	849.098	3	34.36	2644169	34.26	2132465	1.04765
YGVIDPK	cora	Coracle, isoform F	396.219	2	18.74	1027816	18.73	968797	1.22445
EQQYISSSVDPK	cora	Coracle, isoform G	690.836	2	19.72	1077568	19.73	711194	0.85736
IAPNQTAELEDK	cora	Coracle, isoform G	664.838	2	18.46	483773	18.46	266610	0.71591
ITIQSDGDPIDHDK	cora	Coracle, isoform G	518.586	3	21.49	444280	21.51	465036	1.35973
LDDTLGQIVCITPQDNPVVELTVITS	cora	Coracle, isoform G	999.525	3	60.96	477920	60.76	355750	0.96697
LGKPVSTPTVVK	cora	Coracle, isoform G	409.255	3	19.33	1398453	19.26	958082	0.88997
LGLVVTSGLGDNQQDQNLDEAAR	cora	Coracle, isoform G	805.07	3	37.08	2956134	36.91	3245204	1.42607
LIEVGQQIDPIVEVTSLAGK	cora	Coracle, isoform G	1055.09	2	52.4	1797413	52.06	1009797	0.72981
LIEVGQQIDPIVEVTSLAGK	cora	Coracle, isoform G	703.732	3	52.36	1187434	52.06	855614	0.93603
LILNYAQIDPSHFGK	cora	Coracle, isoform G	572.642	3	39.99	1926732	39.77	2328472	1.5699
LLQDPLLSPTTR	cora	Coracle, isoform G	677.39	2	36.71	1818393	36.6	2546599	1.81926
LQGSAVDAAAVPLSDSQK	cora	Coracle, isoform G	878.957	2	28.08	902207	28	1209649	1.74171
MFPVFGSTYR	cora	Coracle, isoform G	610.792	2	35.32	1647098	35.1	1475401	1.16362
NIDGVTHNVEEEVR	cora	Coracle, isoform G	805.892	2	25.19	772422	25.18	383894	0.64562
NIDGVTHNVEEEVR	cora	Coracle, isoform G	537.597	3	25.23	1472968	25.14	947257	0.8354
QAQVQTTTETVPITR	cora	Coracle, isoform G	836.947	2	22.76	1243364	22.75	1875309	1.95928
QAQVQTTTETVPITR	cora	Coracle, isoform G	558.3	3	22.72	338192	22.75	517745	1.98873
SLGVLNLDTGLLDTK	cora	Coracle, isoform G	779.938	2	48.49	4006855	48.22	3396143	1.10104
SPLFTTSATTGPHVESTR	cora	Coracle, isoform G	630.318	3	26.47	4223504	26.38	3945529	1.21354
SSSSSHGKPALAR	cora	Coracle, isoform G	642.836	2	8.36	115696	8.51	27955	0.31388
SSSSSHGKPALAR	cora	Coracle, isoform G	428.893	3	8.36	446429	8.49	229562	0.66799
TAQVETSGGQFDPK	cora	Coracle, isoform G	732.852	2	18.64	2712306	18.61	2930955	1.40376
TDTWPLTFAVK	cora	Coracle, isoform G	639.84	2	45.17	2241253	44.93	1441517	0.83551

TGNLHLVSGVADPK	cora	Coracle, isoform G	469.925	3	24.12	894256	24.12	482494	0.70089
TGYVITIYGYLDPK	cora	Coracle, isoform G	801.924	2	47.28	1483311	47.07	1539226	1.34801
TQAESTNTPVDR	cora	Coracle, isoform G	659.815	2	10.52	427177	10.65	96821	0.29443
TTVTHTMTLTGEIDPVTGR	cora	Coracle, isoform G	677.346	3	32.9	1242941	32.67	1420656	1.48478
TVYVYNMIDPVTGEIVQVDPNDPR	cora	Coracle, isoform G	912.118	3	48.94	447629	48.63	338096	0.98117
VTLLDGSLLDVSIDR	cora	Coracle, isoform G	808.449	2	50.48	2980908	50.17	1512386	0.65908
VVLGEDTPGFSGHGEIISTQTVSSK	cora	Coracle, isoform G	849.098	3	34.36	2644169	34.26	2132465	1.04765
YGVIDPK	cora	Coracle, isoform G	396.219	2	18.74	1027816	18.73	968797	1.22445
FAGVDIR	RpS16	GEO08239p1 (Ribosomal protein S16, isoform B)	389.216	2	25.71	4573283	25.68	6173631	1.75362
LQEPLLLLGK	RpS16	GEO08239p1 (Ribosomal protein S16, isoform B)	562.358	2	43.68	9036494	43.47	7008865	1.00756
EFTLEFSR	SERCA	Calcium-transporting ATPase (EC 7.2.2.10)	514.756	2	35.79	511756	35.66	541898	1.37555
GVVIGTGLSTAIGK	SERCA	Calcium-transporting ATPase (EC 7.2.2.10)	636.88	2	34.79	1157677	34.58	1583799	1.77719
IDQSILTGESVSVIK	SERCA	Calcium-transporting ATPase (EC 7.2.2.10)	794.943	2	37.8	1179386	37.6	1456916	1.60473
IGVFAEDEDTTGK	SERCA	Calcium-transporting ATPase (EC 7.2.2.10)	691.328	2	25.89	1569911	25.87	1880132	1.55574
ITHIYSTTLR	SERCA	Calcium-transporting ATPase (EC 7.2.2.10)	602.838	2	20.66	336218	20.64	473921	1.83108
ITHIYSTTLR	SERCA	Calcium-transporting ATPase (EC 7.2.2.10)	402.228	3	20.66	1041150	20.64	1511216	1.88554
LNSFSVNK	SERCA	Calcium-transporting ATPase (EC 7.2.2.10)	454.745	2	18.74	212026	18.69	338612	2.07461
NILFSGTNVAAGK	SERCA	Calcium-transporting ATPase (EC 7.2.2.10)	646.354	2	32.18	877088	32.05	1429695	2.1175
TVEQSLNFFGTDPER	SERCA	Calcium-transporting ATPase (EC 7.2.2.10)	870.415	2	39.92	733529	39.73	1152158	2.04041
SVYAAEER	Mlp60A	Muscle LIM protein at 60A, isoform F	462.725	2	11.82	2588129	11.89	1417299	0.71137
ALTVPELTQQMFDAK	betaTub6	Tubulin beta chain	854.435	2	37.81	1590296	37.56	1317924	1.07655
IMNTYSVVPSPK	betaTub6	Tubulin beta chain	668.352	2	29.06	1095355	28.94	858780	1.01847
LAVNMVPFPR	betaTub6	Tubulin beta chain	572.321	2	40.41	1793279	40.19	932227	0.6753
LAVNMVPFPR	betaTub6	Tubulin beta chain	580.318	2	34.29	768535	34.41	1250344	2.11343
LHFFMPGFAPLTSR	betaTub6	Tubulin beta chain	540.95	3	48.83	2785496	48.88	1571753	0.733
LHFFMPGFAPLTSR	betaTub6	Tubulin beta chain	546.282	3	42.62	1500055	42.47	820442	0.7105
NSSYFVEWIPNNVK	betaTub6	Tubulin beta chain	848.92	2	44.43	6003407	44.1	632379	0.13684
SIFSNFPQQVK	prom	Prominin, isoform E	647.843	2	36.49	1196743	36.37	1335180	1.44931
TLQEDQSIFR	prom	Prominin, isoform E	618.815	2	28.08	1390520	28.05	1856704	1.73455
VALQIQDVATSSR	prom	Prominin, isoform E	694.381	2	29.91	3286792	29.77	3985799	1.57531
AEEILER	RpL11	Ribosomal protein L11, isoform B	430.23	2	17.53	3556219	17.53	2139819	0.78165
IAVHCTVR	RpL11	Ribosomal protein L11, isoform B	478.261	2	12.42	1451582	12.46	1036746	0.9278
SGTVGFQHR	RpL11	Ribosomal protein L11, isoform B	494.752	2	11.45	1263829	11.54	829082	0.85218
VLEQLTGQQPVFSK	RpL11	Ribosomal protein L11, isoform B	787.433	2	33.08	9876999	32.94	10033931	1.31968
YDPSIGIYGLDFYVVLGRPGYNVNH	RpL11	Ribosomal protein L11, isoform B	746.882	4	53.64	810217	53.57	534216	0.85652
AAIELNR	DHC3B	Uncharacterized protein, isoform G (EC 3.6.1.3) (EC 3.6.4.5)	393.727	2	17.87	27934492	17.87	8147882	0.3789
AGFAGDDAPR	Act87E	Actin 87E, isoform C	488.728	2	15.06	5346544	15.03	6072874	1.47551
AVFPSIVGRPR	Act87E	Actin 87E, isoform C	599.856	2	30.18	3527039	29.97	6233553	2.29587
DSYVGDEAQSK	Act87E	Actin 87E, isoform C	599.765	2	11.94	533801	12.02	513658	1.25002
IWHHTFYNELR	Act87E	Actin 87E, isoform C	505.921	3	25.48	1669489	25.49	2792816	2.17311
SYELPDGQVITIGNER	Act87E	Actin 87E, isoform C	895.95	2	39.17	5791673	39	8517636	1.91046
SYELPDGQVITIGNER	Act87E	Actin 87E, isoform C	597.635	3	39.17	427938	38.97	800279	2.42931
TTGIVLDSGDGVSHTVPIYEGYALPI	Act87E	Actin 87E, isoform C	1051.22	3	45.78	1749429	45.58	1016220	0.75459
VAPEEHPVLLTEAPLNPK	Act87E	Actin 87E, isoform C	977.536	2	34.25	620752	34.11	748708	1.56681
VAPEEHPVLLTEAPLNPK	Act87E	Actin 87E, isoform C	652.026	3	34.21	12031327	34.11	16999678	1.83548
NFSGAELEGLVR	Nsf2	N-ethylmaleimide-sensitive factor 2, isoform B (N-ethylmaleimide-sensitive fac	646.336	2	38.11	673204	37.95	671954	1.29663
LVPVAGLVDSFQK	ATPsynD	ATP synthase subunit d, mitochondrial	686.895	2	44.34	1462212	44.11	1311560	1.1652
VQVGHAPSPNLK	tau	Tau, isoform H	416.235	3	17.44	993769	17.41	1404801	1.83633
ALNLNPTLALIEK	Mlc1	Myosin alkali light chain 1, isoform D	705.422	2	45.94	2478744	45.73	4382358	2.29667
VQVGHAPSPNLK	tau	Microtubule-associated protein	416.235	3	17.44	993769	17.41	1404801	1.83633
VQVGHAPSPNLK	tau	Microtubule-associated protein	416.235	3	17.44	993769	17.41	1404801	1.83633
VQVGHAPSPNLK	tau	Microtubule-associated protein	416.235	3	17.44	993769	17.41	1404801	1.83633
VQVGHAPSPNLK	tau	Microtubule-associated protein	416.235	3	17.44	993769	17.41	1404801	1.83633

TFIVQGFGNVGLHTTR	Gdh	Glutamate dehydrogenase	582.981	3	38.87	1874134	38.58	1474266	1.02187
EYANDIGFK	RpS29	GEO09636p1 (Ribosomal protein S29, isoform B)	528.753	2	24.55	5298011	24.52	4571746	1.12096
YGLNICR	RpS29	GEO09636p1 (Ribosomal protein S29, isoform B)	448.226	2	23.96	2206338	23.96	2842385	1.67353
ALDSMQASLEAEAK	Mhc	Myosin heavy chain, isoform V (EC 3.6.1.3)	732.356	2	29.64	321135	29.49	790332	3.19701
ALDSMQASLEAEAK	Mhc	Myosin heavy chain, isoform V (EC 3.6.1.3)	740.353	2	21.04	48359	20.88	260236	6.99057
DLEEANIQHESTLANLR	Mhc	Myosin heavy chain, isoform V (EC 3.6.1.3)	651.661	3	31.08	667254	31	1249882	2.43333
GALQDYQER	Mhc	Myosin heavy chain, isoform V (EC 3.6.1.3)	540.259	2	15.57	503772	15.55	1324414	3.41517
IEELEEEVEAER	Mhc	Myosin heavy chain, isoform V (EC 3.6.1.3)	737.849	2	27.66	871072	27.57	2436877	3.63414
LAEAEETIESLNQK	Mhc	Myosin heavy chain, isoform V (EC 3.6.1.3)	787.899	2	29.52	307761	29.44	1119309	4.72453
LAGADIETYLLEK	Mhc	Myosin heavy chain, isoform V (EC 3.6.1.3)	718.388	2	42.99	649408	42.76	1212276	2.42497
LTQEAVADLER	Mhc	Myosin heavy chain, isoform V (EC 3.6.1.3)	622.828	2	25.2	753282	25.18	1645891	2.83835
NYSTELFR	Mhc	Myosin heavy chain, isoform V (EC 3.6.1.3)	515.254	2	28.61	584482	28.59	1201453	2.67029
QIEEAEEIAALNLAK	Mhc	Myosin heavy chain, isoform V (EC 3.6.1.3)	821.438	2	39.84	244676	39.61	641209	3.40432
QLEEAESQVSQLSK	Mhc	Myosin heavy chain, isoform V (EC 3.6.1.3)	788.397	2	24.66	238416	24.6	763584	4.16048
TALLDSLSGEK	Mhc	Myosin heavy chain, isoform V (EC 3.6.1.3)	567.306	2	31.71	635833	31.55	1551318	3.16942
TLLEQADR	Mhc	Myosin heavy chain, isoform V (EC 3.6.1.3)	473.254	2	18.74	809820	18.73	1724785	2.76674
VIAYFATVGASK	Mhc	Myosin heavy chain, isoform V (EC 3.6.1.3)	613.843	2	33.65	620379	33.48	1435185	3.0052
AALDCVELFATEQR	Sin3A	Sin3A, isoform J	811.896	2	40.25	1722570	40.03	1260136	0.9503
AALDCVELFATEQR	Sin3A	Sin3A, isoform J	541.6	3	40.21	619337	39.99	590227	1.23798
ANTADGANTVQIHVTGGGAANNPA	Sin3A	Sin3A, isoform J	928.703	4	26.74	509613	26.67	542107	1.38187
DVSFSEASSK	Sin3A	Sin3A, isoform J	528.746	2	17.44	1467180	17.41	381916	0.33815
DYHAGLQQQAHQR	Sin3A	Sin3A, isoform J	517.919	3	11.22	2585659	11.33	2959684	1.48695
DYHAGLQQQAHQR	Sin3A	Sin3A, isoform J	388.691	4	11.22	864204	11.33	948677	1.42602
ESTATALR	Sin3A	Sin3A, isoform J	424.727	2	11.65	1476544	11.71	969884	0.85329
FHLDDHLGGTSQTIHQR	Sin3A	Sin3A, isoform J	654.657	3	23.08	1395287	23.06	756858	0.70465
FHLDDHLGGTSQTIHQR	Sin3A	Sin3A, isoform J	491.245	4	23.12	3523823	23.06	1769329	0.65225
FLEILHDYQR	Sin3A	Sin3A, isoform J	445.235	3	35.73	2969216	35.43	0	0
GTSHQSAAEYVQDVDLSSCK	Sin3A	Sin3A, isoform J	727.995	3	27.24	493988	27.08	189593	0.49857
ILTPQHGGAQTIAYLPSTTPTATNLK	Sin3A	Sin3A, isoform J	898.82	3	35.8	4998395	35.62	5283785	1.37321
LANPALGGGGNTGR	Sin3A	Sin3A, isoform J	627.831	2	17.75	10453019	17.75	7732981	0.96101
LGASYCALPQSTVPK	Sin3A	Sin3A, isoform J	796.411	2	28.68	1479395	28.59	1263101	1.10911
LHAILCDR	Sin3A	Sin3A, isoform J	499.266	2	19.41	1985761	19.38	633888	0.41468
MLTEQEVYTQVAK	Sin3A	Sin3A, isoform J	778.387	2	27.81	3209047	27.77	3556038	1.4395
NDILFEK	Sin3A	Sin3A, isoform J	439.735	2	27.05	2341755	27.01	1764770	0.97897
NPFVAVPIVLK	Sin3A	Sin3A, isoform J	598.874	2	46.1	1222769	45.94	767794	0.81569
QFVPDLFFAPR	Sin3A	Sin3A, isoform J	668.856	2	50.52	6756025	50.25	7385320	1.42004
SAHAAAIGNLSAVNTSVSIK	Sin3A	Sin3A, isoform J	956.018	2	31.61	170248	31.53	254171	1.9394
SAHAAAIGNLSAVNTSVSIK	Sin3A	Sin3A, isoform J	637.681	3	31.61	2399426	31.53	2575551	1.39439
SHCIDTPGVIER	Sin3A	Sin3A, isoform J	692.338	2	20.25	307279	20.24	237394	1.0036
SHCIDTPGVIER	Sin3A	Sin3A, isoform J	461.894	3	20.22	598355	20.24	340785	0.73985
SSPGISYATPPLPSGPHGQHNSGS.	Sin3A	Sin3A, isoform J	887.432	3	25.86	488181	25.83	202251	0.53819
TILDDAANLLIHHVK	Sin3A	Sin3A, isoform J	418.99	4	49.12	1380604	49.31	716808	0.67446
TISGTQTVATAVGNLATISQQQPVQ	Sin3A	Sin3A, isoform J	1041.89	3	45.65	3056186	45.35	3369934	1.4324
TYNNNQQQQNHVIGSGLNATR	Sin3A	Sin3A, isoform J	786.383	3	19.45	600429	19.45	504776	1.09209
VLENLQK	Sin3A	Sin3A, isoform J	422.25	2	16.41	3516505	16.41	2648950	0.97855
VTIELLDSEPEEVDKPAALK	Sin3A	Sin3A, isoform J	732.726	3	39.02	4118678	38.81	3130918	0.9875
YVGHAPNQNLTHGHNAK	Sin3A	Sin3A, isoform J	619.975	3	10.09	210214	10.13	163530	1.01055
VVYDPQTAEELSSK	ScsbetaA	Succinate--CoA ligase [ADP-forming] subunit beta, mitochondrial (EC 6.2.1.5)	783.388	2	26.05	1092441	25.98	763625	0.90804
VQVGHAPSPNLK	tau	Microtubule-associated protein	416.235	3	17.44	993769	17.41	1404801	1.83633
SIFSNFPQQVK	prom	IP13255p (Prominin, isoform B)	647.843	2	36.49	1196743	36.37	1335180	1.44931
TLQEDQSIFR	prom	IP13255p (Prominin, isoform B)	618.815	2	28.08	1390520	28.05	1856704	1.73455
VALQIQDVATSSR	prom	IP13255p (Prominin, isoform B)	694.381	2	29.91	3286792	29.77	3985799	1.57531
NFPALTYFR	l(2)01289	Lethal (2) 01289, isoform A	564.795	2	42.88	433584	42.79	472450	1.41548

DFYQIR	CG1620	Uncharacterized protein, isoform A (Uncharacterized protein, isoform C)	421.214	2	25.63	4058468	25.64	3651345	1.16873
DNEQALHLLVQCGYDFK	CG1620	Uncharacterized protein, isoform A (Uncharacterized protein, isoform C)	683.995	3	42.88	848275	42.64	432717	0.66266
ELVHFYYLWK	CG1620	Uncharacterized protein, isoform A (Uncharacterized protein, isoform C)	699.366	2	44.45	3217079	44.31	2152154	0.86903
ELVHFYYLWK	CG1620	Uncharacterized protein, isoform A (Uncharacterized protein, isoform C)	466.58	3	44.45	3541291	44.31	2675658	0.9815
SHLLDLYPDESFVDLAPTCGEETEF	CG1620	Uncharacterized protein, isoform A (Uncharacterized protein, isoform C)	965.111	3	46.58	8771567	46.33	5071262	0.75104
SSDHSQHSASEASADSGSLANSDL	CG1620	Uncharacterized protein, isoform A (Uncharacterized protein, isoform C)	740.089	4	22.78	177697	22.79	299818	2.19179
SYSIMTGSQAVGNPSGGNR	CG1620	Uncharacterized protein, isoform A (Uncharacterized protein, isoform C)	941.939	2	25.71	2675687	25.67	2818270	1.36826
SYSIMTGSQAVGNPSGGNR	CG1620	Uncharacterized protein, isoform A (Uncharacterized protein, isoform C)	628.295	3	25.75	615003	25.63	817516	1.7268
SYSIMTGSQAVGNPSGGNR	CG1620	Uncharacterized protein, isoform A (Uncharacterized protein, isoform C)	633.627	3	20.74	1262978	20.76	1244066	1.27959
YGGTAASPAMSSSNR	CG1620	Uncharacterized protein, isoform A (Uncharacterized protein, isoform C)	728.828	2	13.99	2532481	13.97	2794880	1.43364
YGGTAASPAMSSSNR	CG1620	Uncharacterized protein, isoform A (Uncharacterized protein, isoform C)	736.825	2	10.01	2951260	10.18	1942943	0.85521
LGGVPVGVIAVETR	ACC	Acetyl-CoA carboxylase, isoform A (EC 6.4.1.2)	683.906	2	38.38	348778	38.27	503425	1.87503
EAVGAFIVFDVTR	Rab32	FI09421p (Rab32, isoform D)	712.383	2	49.81	1401007	49.56	1390509	1.28931
EAVGAFIVFDVTR	Rab32	Rab32, isoform I	712.383	2	49.81	1401007	49.56	1390509	1.28931
FEVGSAVR	Rab32	Rab32, isoform I	432.732	2	20.96	1713425	21.01	2519725	1.91034
SPLSAYEAALAAAASQQPNSNEELS	Rab32	Rab32, isoform I	1026.49	3	47.1	622725	46.88	729415	1.5216
EAVGAFIVFDVTR	Rab32	Rab32, isoform B	712.383	2	49.81	1401007	49.56	1390509	1.28931
ESPSASPSPLITTTESTR	Rab32	Rab32, isoform B	930.963	2	27.24	387801	27.15	224693	0.75267
FEVGSAVR	Rab32	Rab32, isoform B	432.732	2	20.96	1713425	21.01	2519725	1.91034
SAEEEAQETPGELEDVASSISTDSG	Rab32	Rab32, isoform B	969.779	3	43.29	1179345	43.05	1501276	1.65364
SDTDYLTPAAEER	Rab32	Rab32, isoform B	734.334	2	23.54	1844209	23.46	1876636	1.32188
SPLSAYEAALAAAASQQPNSNEELS	Rab32	Rab32, isoform B	1026.49	3	47.1	622725	46.88	729415	1.5216
APNPPTPVR	Twdlbeta	Tweedlebeta	474.767	2	13.67	4226855	15.45	3159825	0.97111
QVLPPPVQDEHK	Twdlbeta	Tweedlebeta	462.917	3	19.53	1557367	19.59	3924824	3.2738
TLVYVLVK	Twdlbeta	Tweedlebeta	467.802	2	36.56	5020293	36.32	6917808	1.79004
AFSVALESIPLALAENSGLHPIETLSI	CCT5	Chaperonin containing TCP1 subunit 5, isoform B	1017.22	3	62.61	384652	62.45	401044	1.3544
FEELTPEK	CCT5	Chaperonin containing TCP1 subunit 5, isoform B	496.75	2	20.66	516313	20.64	311483	0.78369
IADGFELAAQCAIK	CCT5	Chaperonin containing TCP1 subunit 5, isoform B	753.885	2	37.31	382322	37.14	458415	1.55759
LGVAGLVR	CCT5	Chaperonin containing TCP1 subunit 5, isoform B	392.756	2	29.1	644815	29.01	620261	1.24957
ILTGVVR	RpS11	Ribosomal protein S11, isoform B	379.25	2	21.96	1109408	21.94	1280841	1.49978
AALDCVELFATEQR	Sin3A	Sin3A, isoform A (Sin3A, isoform F)	811.896	2	40.25	1722570	40.03	1260136	0.9503
AALDCVELFATEQR	Sin3A	Sin3A, isoform A (Sin3A, isoform F)	541.6	3	40.21	619337	39.99	590227	1.23798
ANTADGANTVQIHVTGGGAANNPA	Sin3A	Sin3A, isoform A (Sin3A, isoform F)	928.703	4	26.74	509613	26.67	542107	1.38187
DVSFSEASSK	Sin3A	Sin3A, isoform A (Sin3A, isoform F)	528.746	2	17.44	1467180	17.41	381916	0.33815
DYHAGLQQQAHQR	Sin3A	Sin3A, isoform A (Sin3A, isoform F)	517.919	3	11.22	2585659	11.33	2959684	1.48695
DYHAGLQQQAHQR	Sin3A	Sin3A, isoform A (Sin3A, isoform F)	388.691	4	11.22	864204	11.33	948677	1.42602
ESTATALR	Sin3A	Sin3A, isoform A (Sin3A, isoform F)	424.727	2	11.65	1476544	11.71	969884	0.85329
FHLDDHLGGTSQTIHQR	Sin3A	Sin3A, isoform A (Sin3A, isoform F)	654.657	3	23.08	1395287	23.06	756858	0.70465
FHLDDHLGGTSQTIHQR	Sin3A	Sin3A, isoform A (Sin3A, isoform F)	491.245	4	23.12	3523823	23.06	1769329	0.65225
FLEILHDYQR	Sin3A	Sin3A, isoform A (Sin3A, isoform F)	445.235	3	35.73	2969216	35.43	0	0
GTSHQSAAEYVQDVDLSSCK	Sin3A	Sin3A, isoform A (Sin3A, isoform F)	727.995	3	27.24	493988	27.08	189593	0.49857
ILTPQHGGAQTIAYLPSTTPTATNLK	Sin3A	Sin3A, isoform A (Sin3A, isoform F)	898.82	3	35.8	4998395	35.62	5283785	1.37321
LANPALGGGGNTGR	Sin3A	Sin3A, isoform A (Sin3A, isoform F)	627.831	2	17.75	10453019	17.75	7732981	0.96101
LGASYCALPQSTVPK	Sin3A	Sin3A, isoform A (Sin3A, isoform F)	796.411	2	28.68	1479395	28.59	1263101	1.10911
LHAILCDR	Sin3A	Sin3A, isoform A (Sin3A, isoform F)	499.266	2	19.41	1985761	19.38	633888	0.41468
MLTEQEVYTQVAK	Sin3A	Sin3A, isoform A (Sin3A, isoform F)	778.387	2	27.81	3209047	27.77	3556038	1.4395
NDILFEK	Sin3A	Sin3A, isoform A (Sin3A, isoform F)	439.735	2	27.05	2341755	27.01	1764770	0.97897
NPFVAVPIVLK	Sin3A	Sin3A, isoform A (Sin3A, isoform F)	598.874	2	46.1	1222769	45.94	767794	0.81569
QFVPDLFFAPR	Sin3A	Sin3A, isoform A (Sin3A, isoform F)	668.856	2	50.52	6756025	50.25	7385320	1.42004
SAHAAAIGNLSAVNTSVSIK	Sin3A	Sin3A, isoform A (Sin3A, isoform F)	956.018	2	31.61	170248	31.53	254171	1.9394
SAHAAAIGNLSAVNTSVSIK	Sin3A	Sin3A, isoform A (Sin3A, isoform F)	637.681	3	31.61	2399426	31.53	2575551	1.39439
SHCIDTPGVIER	Sin3A	Sin3A, isoform A (Sin3A, isoform F)	692.338	2	20.25	307279	20.24	237394	1.0036
SHCIDTPGVIER	Sin3A	Sin3A, isoform A (Sin3A, isoform F)	461.894	3	20.22	598355	20.24	340785	0.73985

SSPGISYATPPLPSGPHGQHNSGS.	Sin3A	Sin3A, isoform A (Sin3A, isoform F)	887.432	3	25.86	488181	25.83	202251	0.53819
TILDDAANLLIHHVK	Sin3A	Sin3A, isoform A (Sin3A, isoform F)	418.99	4	49.12	1380604	49.31	716808	0.67446
TISGTQTVATAVGNLATISQQQPVQ	Sin3A	Sin3A, isoform A (Sin3A, isoform F)	1041.89	3	45.65	3056186	45.35	3369934	1.4324
TYNNNQQQQNHVIGSGLNATR	Sin3A	Sin3A, isoform A (Sin3A, isoform F)	786.383	3	19.45	600429	19.45	504776	1.09209
VLENLQK	Sin3A	Sin3A, isoform A (Sin3A, isoform F)	422.25	2	16.41	3516505	16.41	2648950	0.97855
VTIELLDSEPEEVDKPAALK	Sin3A	Sin3A, isoform A (Sin3A, isoform F)	732.726	3	39.02	4118678	38.81	3130918	0.9875
YVGHAPNQNLTHGHNAK	Sin3A	Sin3A, isoform A (Sin3A, isoform F)	619.975	3	10.09	210214	10.13	163530	1.01055
AALDCVELFATEQR	Sin3A	Sin3A, isoform D	811.896	2	40.25	1722570	40.03	1260136	0.9503
AALDCVELFATEQR	Sin3A	Sin3A, isoform D	541.6	3	40.21	619337	39.99	590227	1.23798
ANTADGANTVQIHVTGGGAANNPA	Sin3A	Sin3A, isoform D	928.703	4	26.74	509613	26.67	542107	1.38187
DVSFSEASSK	Sin3A	Sin3A, isoform D	528.746	2	17.44	1467180	17.41	381916	0.33815
DYHAGLQQQAHQR	Sin3A	Sin3A, isoform D	517.919	3	11.22	2585659	11.33	2959684	1.48695
DYHAGLQQQAHQR	Sin3A	Sin3A, isoform D	388.691	4	11.22	864204	11.33	948677	1.42602
ESTATALR	Sin3A	Sin3A, isoform D	424.727	2	11.65	1476544	11.71	969884	0.85329
FHLDDHLGGTSQTIHQR	Sin3A	Sin3A, isoform D	654.657	3	23.08	1395287	23.06	756858	0.70465
FHLDDHLGGTSQTIHQR	Sin3A	Sin3A, isoform D	491.245	4	23.12	3523823	23.06	1769329	0.65225
FLEILHDYQR	Sin3A	Sin3A, isoform D	445.235	3	35.73	2969216	35.43	0	0
GTSHQSAAEYVQDVDLSSCK	Sin3A	Sin3A, isoform D	727.995	3	27.24	493988	27.08	189593	0.49857
ILTPQHGGAQTIAYLPSTTPTATNLK	Sin3A	Sin3A, isoform D	898.82	3	35.8	4998395	35.62	5283785	1.37321
LANPALGGGGNTGR	Sin3A	Sin3A, isoform D	627.831	2	17.75	10453019	17.75	7732981	0.96101
LGASYCALPQSTVPK	Sin3A	Sin3A, isoform D	796.411	2	28.68	1479395	28.59	1263101	1.10911
LHAILCDR	Sin3A	Sin3A, isoform D	499.266	2	19.41	1985761	19.38	633888	0.41468
MLTEQEVYTQVAK	Sin3A	Sin3A, isoform D	778.387	2	27.81	3209047	27.77	3556038	1.4395
NDILFEK	Sin3A	Sin3A, isoform D	439.735	2	27.05	2341755	27.01	1764770	0.97897
NPFVAVPIVLK	Sin3A	Sin3A, isoform D	598.874	2	46.1	1222769	45.94	767794	0.81569
QFVPDLFFAPR	Sin3A	Sin3A, isoform D	668.856	2	50.52	6756025	50.25	7385320	1.42004
SAHAAAIGNLSAVNTSVSIK	Sin3A	Sin3A, isoform D	956.018	2	31.61	170248	31.53	254171	1.9394
SAHAAAIGNLSAVNTSVSIK	Sin3A	Sin3A, isoform D	637.681	3	31.61	2399426	31.53	2575551	1.39439
SHCIDTPGVIER	Sin3A	Sin3A, isoform D	692.338	2	20.25	307279	20.24	237394	1.0036
SHCIDTPGVIER	Sin3A	Sin3A, isoform D	461.894	3	20.22	598355	20.24	340785	0.73985
SSPGISYATPPLPSGPHGQHNSGS.	Sin3A	Sin3A, isoform D	887.432	3	25.86	488181	25.83	202251	0.53819
TILDDAANLLIHHVK	Sin3A	Sin3A, isoform D	418.99	4	49.12	1380604	49.31	716808	0.67446
TISGTQTVATAVGNLATISQQQPVQ	Sin3A	Sin3A, isoform D	1041.89	3	45.65	3056186	45.35	3369934	1.4324
TYNNNQQQQNHVIGSGLNATR	Sin3A	Sin3A, isoform D	786.383	3	19.45	600429	19.45	504776	1.09209
VLENLQK	Sin3A	Sin3A, isoform D	422.25	2	16.41	3516505	16.41	2648950	0.97855
VTIELLDSEPEEVDKPAALK	Sin3A	Sin3A, isoform D	732.726	3	39.02	4118678	38.81	3130918	0.9875
YVGHAPNQNLTHGHNAK	Sin3A	Sin3A, isoform D	619.975	3	10.09	210214	10.13	163530	1.01055
GVDLDQLLDMPNNQLVELMHSR	RpS15	Ribosomal protein S15, isoform B (SD21317p)	846.421	3	56.93	2438393	56.76	3030472	1.61447
GVDLDQLLDMPNNQLVELMHSR	RpS15	Ribosomal protein S15, isoform B (SD21317p)	851.753	3	52.82	1993202	52.51	2732090	1.7806
GVDLDQLLDMPNNQLVELMHSR	RpS15	Ribosomal protein S15, isoform B (SD21317p)	857.084	3	46.75	939137	46.49	1012387	1.40036
HGRPGIGATHSSR	RpS15	Ribosomal protein S15, isoform B (SD21317p)	666.848	2	7.93	677685	8.06	679248	1.30204
HGRPGIGATHSSR	RpS15	Ribosomal protein S15, isoform B (SD21317p)	444.901	3	7.93	4672602	8.06	5517679	1.53398
DTAALLEGYVLIGLVPVEK	hnRNP	Uncharacterized protein, isoform B	1000.57	2	62.74	438531	62.45	679195	2.01195
DTAALLEGYVLIGLVPVEK	hnRNP	Uncharacterized protein, isoform B	667.382	3	62.73	263834	62.5	456206	2.24622
NFILDQTNVYASAQR	hnRNP	Uncharacterized protein, isoform B	870.439	2	36.23	646640	36.09	453785	0.91161
ALTVPELTQQMFDAK	betaTub5	Tubulin beta chain	854.435	2	37.81	1590296	37.56	1317924	1.07655
AVLVDLEPGTMDSVR	betaTub5	Tubulin beta chain	801.414	2	39.24	2023515	39.04	2057238	1.32069
AVLVDLEPGTMDSVR	betaTub5	Tubulin beta chain	809.411	2	34.48	5552510	34.3	4552577	1.0651
EAESCDCLQGFQLTHSLGGGTGSC	betaTub5	Tubulin beta chain	1109.85	3	41.84	370508	41.64	177516	0.62239
GHYTEGAELVDSVLDVVR	betaTub5	Tubulin beta chain	653.665	3	50.34	2775420	50.24	1100574	0.51513
IMNTYSVVPSPK	betaTub5	Tubulin beta chain	668.352	2	29.06	1095355	28.94	858780	1.01847
INVYYNEASGGK	betaTub5	Tubulin beta chain	657.82	2	21.12	4330833	21.13	2405958	0.72167
LAVNMVPFPR	betaTub5	Tubulin beta chain	572.321	2	40.41	1793279	40.19	932227	0.6753

LAVNMVPFPR	betaTub5	Tubulin beta chain	580.318	2	34.29	768535	34.41	1250344	2.11343	
LHFFMPGFAPLTSR	betaTub5	Tubulin beta chain	540.95	3	48.83	2785496	48.88	1571753	0.733	
LHFFMPGFAPLTSR	betaTub5	Tubulin beta chain	546.282	3	42.62	1500055	42.47	820442	0.7105	
LTTPTYGDLNHLVSLTMSGVTTCLR	betaTub5	Tubulin beta chain	917.467	3	55.9	882815	55.69	373168	0.54911	
NSSYFVEWIPNNVK	betaTub5	Tubulin beta chain	848.92	2	44.43	6003407	44.1	632379	0.13684	
SGPFGQIFRPDNFVFGQSGAGNNW	betaTub5	Tubulin beta chain	933.453	3	49.4	1384897	49.2	571844	0.53639	
YLTVAAIFR	betaTub5	Tubulin beta chain	527.308	2	45.1	10561950	44.89	6760614	0.8315	
ILIPSSIAGAVIGK	HnRNP-K	Heterogeneous nuclear ribonucleoprotein K, isoform C (RE05515p)	669.921	2	46.3	366013	46.1	1322236	4.69283	
ILIPSSIAGAVIGK	HnRNP-K	Heterogeneous nuclear ribonucleoprotein K, isoform A (Heterogeneous nucle	669.921	2	46.3	366013	46.1	1322236	4.69283	
ISGATTFDFIPQK	CaMKII	Calcium/calmodulin-dependent protein kinase II, isoform C (EC 2.7.11.-) (EC	712.875	2	38.6	1118955	38.42	1549927	1.79937	
ISGATTFDFIPQK	CaMKII	Calcium/calmodulin-dependent protein kinase II, isoform E (EC 2.7.11.-) (EC	712.875	2	38.6	1118955	38.42	1549927	1.79937	
ESTLHLVLR	Ubi-p63E	Ubiquitin-63E, isoform C (Ubiquitin-63E, isoform D)	534.314	2	29.06	19834334	28.96	12823394	0.83986	
ESTLHLVLR	Ubi-p63E	Ubiquitin-63E, isoform C (Ubiquitin-63E, isoform D)	356.545	3	29.02	13873703	28.96	8349692	0.78181	
ESTLHLVLR	Ubi-p63E	Ubiquitin-63E, isoform C (Ubiquitin-63E, isoform D)	534.314	2	29.06	19834334	28.96	12823394	0.83986	
ESTLHLVLR	Ubi-p63E	Ubiquitin-63E, isoform C (Ubiquitin-63E, isoform D)	356.545	3	29.02	13873703	28.96	8349692	0.78181	
ESTLHLVLR	Ubi-p63E	Ubiquitin-63E, isoform C (Ubiquitin-63E, isoform D)	534.314	2	29.06	19834334	28.96	12823394	0.83986	
ESTLHLVLR	Ubi-p63E	Ubiquitin-63E, isoform C (Ubiquitin-63E, isoform D)	356.545	3	29.02	13873703	28.96	8349692	0.78181	
ESTLHLVLR	Ubi-p63E	Ubiquitin-63E, isoform C (Ubiquitin-63E, isoform D)	534.314	2	29.06	19834334	28.96	12823394	0.83986	
ESTLHLVLR	Ubi-p63E	Ubiquitin-63E, isoform C (Ubiquitin-63E, isoform D)	356.545	3	29.02	13873703	28.96	8349692	0.78181	
ESTLHLVLR	Ubi-p63E	Ubiquitin-63E, isoform C (Ubiquitin-63E, isoform D)	534.314	2	29.06	19834334	28.96	12823394	0.83986	
ESTLHLVLR	Ubi-p63E	Ubiquitin-63E, isoform C (Ubiquitin-63E, isoform D)	356.545	3	29.02	13873703	28.96	8349692	0.78181	
ESTLHLVLR	Ubi-p63E	Ubiquitin-63E, isoform C (Ubiquitin-63E, isoform D)	534.314	2	29.06	19834334	28.96	12823394	0.83986	
ESTLHLVLR	Ubi-p63E	Ubiquitin-63E, isoform C (Ubiquitin-63E, isoform D)	356.545	3	29.02	13873703	28.96	8349692	0.78181	
ESTLHLVLR	Ubi-p63E	Ubiquitin-63E, isoform C (Ubiquitin-63E, isoform D)	534.314	2	29.06	19834334	28.96	12823394	0.83986	
ESTLHLVLR	Ubi-p63E	Ubiquitin-63E, isoform C (Ubiquitin-63E, isoform D)	356.545	3	29.02	13873703	28.96	8349692	0.78181	
ESTLHLVLR	Ubi-p63E	Ubiquitin-63E, isoform C (Ubiquitin-63E, isoform D)	534.314	2	29.06	19834334	28.96	12823394	0.83986	
ESTLHLVLR	Ubi-p63E	Ubiquitin-63E, isoform C (Ubiquitin-63E, isoform D)	356.545	3	29.02	13873703	28.96	8349692	0.78181	
ESTLHLVLR	Ubi-p63E	Ubiquitin-63E, isoform C (Ubiquitin-63E, isoform D)	534.314	2	29.06	19834334	28.96	12823394	0.83986	
ESTLHLVLR	Ubi-p63E	Ubiquitin-63E, isoform C (Ubiquitin-63E, isoform D)	356.545	3	29.02	13873703	28.96	8349692	0.78181	
ESTLHLVLR	Ubi-p63E	Ubiquitin-63E, isoform C (Ubiquitin-63E, isoform D)	534.314	2	29.06	19834334	28.96	12823394	0.83986	
ESTLHLVLR	Ubi-p63E	Ubiquitin-63E, isoform C (Ubiquitin-63E, isoform D)	356.545	3	29.02	13873703	28.96	8349692	0.78181	
MQIFVK	Ubi-p63E	Ubiquitin-63E, isoform C (Ubiquitin-63E, isoform D)	383.22	2	27.43	8697430	27.39	10348569	1.54565	
TITLEVEPSDTIENVK	Ubi-p63E	Ubiquitin-63E, isoform C (Ubiquitin-63E, isoform D)	894.467	2	36.85	41743372	36.64	47966256	1.49269	
TITLEVEPSDTIENVK	Ubi-p63E	Ubiquitin-63E, isoform C (Ubiquitin-63E, isoform D)	596.647	3	36.85	1563861	36.68	1963090	1.63066	
TITLEVEPSDTIENVK	Ubi-p63E	Ubiquitin-63E, isoform C (Ubiquitin-63E, isoform D)	894.467	2	36.85	41743372	36.64	47966256	1.49269	
TITLEVEPSDTIENVK	Ubi-p63E	Ubiquitin-63E, isoform C (Ubiquitin-63E, isoform D)	596.647	3	36.85	1563861	36.68	1963090	1.63066	
TITLEVEPSDTIENVK	Ubi-p63E	Ubiquitin-63E, isoform C (Ubiquitin-63E, isoform D)	894.467	2	36.85	41743372	36.64	47966256	1.49269	
TITLEVEPSDTIENVK	Ubi-p63E	Ubiquitin-63E, isoform C (Ubiquitin-63E, isoform D)	596.647	3	36.85	1563861	36.68	1963090	1.63066	
TITLEVEPSDTIENVK	Ubi-p63E	Ubiquitin-63E, isoform C (Ubiquitin-63E, isoform D)	894.467	2	36.85	41743372	36.64	47966256	1.49269	
TITLEVEPSDTIENVK	Ubi-p63E	Ubiquitin-63E, isoform C (Ubiquitin-63E, isoform D)	596.647	3	36.85	1563861	36.68	1963090	1.63066	
TITLEVEPSDTIENVK	Ubi-p63E	Ubiquitin-63E, isoform C (Ubiquitin-63E, isoform D)	894.467	2	36.85	41743372	36.64	47966256	1.49269	
TITLEVEPSDTIENVK	Ubi-p63E	Ubiquitin-63E, isoform C (Ubiquitin-63E, isoform D)	596.647	3	36.85	1563861	36.68	1963090	1.63066	
TITLEVEPSDTIENVK	Ubi-p63E	Ubiquitin-63E, isoform C (Ubiquitin-63E, isoform D)	894.467	2	36.85	41743372	36.64	47966256	1.49269	
TITLEVEPSDTIENVK	Ubi-p63E	Ubiquitin-63E, isoform C (Ubiquitin-63E, isoform D)	596.647	3	36.85	1563861	36.68	1963090	1.63066	
TITLEVEPSDTIENVK	Ubi-p63E	Ubiquitin-63E, isoform C (Ubiquitin-63E, isoform D)	894.467	2	36.85	41743372	36.64	47966256	1.49269	
TITLEVEPSDTIENVK	Ubi-p63E	Ubiquitin-63E, isoform C (Ubiquitin-63E, isoform D)	596.647	3	36.85	1563861	36.68	1963090	1.63066	
TITLEVEPSDTIENVK	Ubi-p63E	Ubiquitin-63E, isoform C (Ubiquitin-63E, isoform D)	894.467	2	36.85	41743372	36.64	47966256	1.49269	
TITLEVEPSDTIENVK	Ubi-p63E	Ubiquitin-63E, isoform C (Ubiquitin-63E, isoform D)	596.647	3	36.85	1563861	36.68	1963090	1.63066	
TITLEVEPSDTIENVK	Ubi-p63E	Ubiquitin-63E, isoform C (Ubiquitin-63E, isoform D)	894.467	2	36.85	41743372	36.64	47966256	1.49269	
TITLEVEPSDTIENVK	Ubi-p63E	Ubiquitin-63E, isoform C (Ubiquitin-63E, isoform D)	596.647	3	36.85	1563861	36.68	1963090	1.63066	
TLSDYNIQK	Ubi-p63E	Ubiquitin-63E, isoform C (Ubiquitin-63E, isoform D)	541.28	2	19.53	19914654	19.52	7860957	0.51277	

TLSDYNIQK	Ubi-p63E	Ubiquitin-63E, isoform C (Ubiquitin-63E, isoform D)	541.28	2	19.53	19914654	19.52	7860957	0.51277	
TLSDYNIQK	Ubi-p63E	Ubiquitin-63E, isoform C (Ubiquitin-63E, isoform D)	541.28	2	19.53	19914654	19.52	7860957	0.51277	
TLSDYNIQK	Ubi-p63E	Ubiquitin-63E, isoform C (Ubiquitin-63E, isoform D)	541.28	2	19.53	19914654	19.52	7860957	0.51277	
TLSDYNIQK	Ubi-p63E	Ubiquitin-63E, isoform C (Ubiquitin-63E, isoform D)	541.28	2	19.53	19914654	19.52	7860957	0.51277	
TLSDYNIQK	Ubi-p63E	Ubiquitin-63E, isoform C (Ubiquitin-63E, isoform D)	541.28	2	19.53	19914654	19.52	7860957	0.51277	
TLSDYNIQK	Ubi-p63E	Ubiquitin-63E, isoform C (Ubiquitin-63E, isoform D)	541.28	2	19.53	19914654	19.52	7860957	0.51277	
TLSDYNIQK	Ubi-p63E	Ubiquitin-63E, isoform C (Ubiquitin-63E, isoform D)	541.28	2	19.53	19914654	19.52	7860957	0.51277	
TLSDYNIQK	Ubi-p63E	Ubiquitin-63E, isoform C (Ubiquitin-63E, isoform D)	541.28	2	19.53	19914654	19.52	7860957	0.51277	
TLSDYNIQK	Ubi-p63E	Ubiquitin-63E, isoform C (Ubiquitin-63E, isoform D)	541.28	2	19.53	19914654	19.52	7860957	0.51277	
HVVEQVHEEQER	Prm	FI03688p (Paramyosin, isoform B)	506.915	3	9.49	191473	9.59	475223	3.22413	
HVVEQVHEEQER	Prm	Paramyosin, isoform D	506.915	3	9.49	191473	9.59	475223	3.22413	
SGSGGSTGYVPTGRPR	D1	D1 chromosomal protein, isoform C (D1 chromosomal protein, isoform D)	512.59	3	14.38	493054	14.31	732667	1.93034	
TAVETAILLLR	CCT3	T-complex protein 1 subunit gamma	600.372	2	46.75	1365542	46.61	617422	0.58735	
TLAQNCGANTIR	CCT3	T-complex protein 1 subunit gamma	659.83	2	15.65	496704	15.59	376804	0.98546	
GYAFVTFTNR	Syp	Syncrip, isoform F	588.296	2	35.44	514118	35.33	604713	1.52795	
AAASIILR	CCT1	Chaperonin containing TCP1 subunit 1, isoform B	407.761	2	25.72	2915044	25.64	1149857	0.51241	
EQLAIAEFAK	CCT1	Chaperonin containing TCP1 subunit 1, isoform B	560.306	2	35.24	3285457	35.02	1749112	0.69158	
FATEAAITILR	CCT1	Chaperonin containing TCP1 subunit 1, isoform B	603.348	2	39.36	10718412	39.2	13176747	1.59698	
ICDDELILIK	CCT1	Chaperonin containing TCP1 subunit 1, isoform B	616.334	2	39.17	3199977	38.93	2711421	1.10071	
IHPTSIISGYR	CCT1	Chaperonin containing TCP1 subunit 1, isoform B	622.343	2	25.75	1934865	25.67	1766943	1.1863	
IIGADAEFFSAMVVDAAQSVK	CCT1	Chaperonin containing TCP1 subunit 1, isoform B	723.701	3	60.05	1786490	59.91	2285436	1.66185	
IIGADAEFFSAMVVDAAQSVK	CCT1	Chaperonin containing TCP1 subunit 1, isoform B	729.033	3	49.26	1206828	48.93	1391766	1.49811	
LLEVEHPAAK	CCT1	Chaperonin containing TCP1 subunit 1, isoform B	553.814	2	17.76	1488962	17.76	1720402	1.50096	
LLEVEHPAAK	CCT1	Chaperonin containing TCP1 subunit 1, isoform B	369.545	3	17.76	5109354	17.76	6443911	1.63835	
MLVDDIGDVTVTNDGATILR	CCT1	Chaperonin containing TCP1 subunit 1, isoform B	1059.54	2	45.59	805671	45.35	784141	1.26433	
MLVDDIGDVTVTNDGATILR	CCT1	Chaperonin containing TCP1 subunit 1, isoform B	1067.54	2	42.51	681498	42.29	684194	1.30418	
MLVDDIGDVTVTNDGATILR	CCT1	Chaperonin containing TCP1 subunit 1, isoform B	712.028	3	42.51	590691	42.33	540350	1.18833	
SLLVIPK	CCT1	Chaperonin containing TCP1 subunit 1, isoform B	385.263	2	33.27	2316109	33.09	1393818	0.78175	
SSLGPVGLDK	CCT1	Chaperonin containing TCP1 subunit 1, isoform B	486.772	2	24.74	2956948	24.72	3410829	1.49844	
SYHNSSQTKPER	CCT1	Chaperonin containing TCP1 subunit 1, isoform B	717.342	2	6.8	51478	6.73	17702	0.44671	
SYHNSSQTKPER	CCT1	Chaperonin containing TCP1 subunit 1, isoform B	478.564	3	6.8	142153	6.73	56320	0.51467	
TQNVMAALSISNIVK	CCT1	Chaperonin containing TCP1 subunit 1, isoform B	794.94	2	46.95	1284833	46.68	1508805	1.52549	
TQNVMAALSISNIVK	CCT1	Chaperonin containing TCP1 subunit 1, isoform B	802.937	2	39.73	2202017	39.48	2641875	1.55853	
VLVELAQLQDEEVGDGTTSVVILAA	CCT1	Chaperonin containing TCP1 subunit 1, isoform B	1018.23	3	65.61	1972773	65.17	0	0	
WTGLDLIEGVVR	CCT1	Chaperonin containing TCP1 subunit 1, isoform B	679.377	2	53.01	1351754	52.71	645340	0.62017	
YFVEAGAMAVR	CCT1	Chaperonin containing TCP1 subunit 1, isoform B	615.303	2	24.89	676486	24.87	566812	1.08844	
ALQASVLR	Ald1	Fructose-bisphosphate aldolase (EC 4.1.2.13)	429.264	2	22.24	109791	22.21	356378	4.21664	
IGGIGTVPVGR	eEF1alph	Elongation factor 1-alpha	513.309	2	26.78	8471779	26.71	7294916	1.11858	
IHINIVVIGHVDSGK	eEF1alph	Elongation factor 1-alpha	534.311	3	34.63	1881392	34.42	1040176	0.71821	
LPLQDVYK	eEF1alph	Elongation factor 1-alpha	488.279	2	28.46	1429878	28.37	739398	0.67174	
STTTGHLIYK	eEF1alph	Elongation factor 1-alpha	560.803	2	15.84	2355586	15.79	1523568	0.8402	
IAAEVAAPLAK	Flo2	Flotillin 2, isoform F	527.319	2	25.68	101116	25.6	592504	7.61191	
VYSPHVLNLTLIDLPGLTK	shi	Shibire, isoform F (Shibire, isoform G) (Shibire, isoform J)	698.405	3	53.41	463587	53.25	477207	1.3372	
VYSPHVLNLTLIDLPGLTK	shi	Shibire, isoform C (Shibire, isoform E) (Shibire, isoform H) (Shibire, isoform I)	698.405	3	53.41	463587	53.25	477207	1.3372	
AAEFNFR	wupA	GH08696p (Wings up A, isoform F)	427.711	2	24.96	9042040	24.95	9905750	1.42313	
GFMTPER	wupA	GH08696p (Wings up A, isoform F)	419.2	2	19.67	1284815	19.67	1246317	1.26012	
LGGVPVGVIAVETR	ACC	Acetyl-CoA carboxylase, isoform E (EC 6.4.1.2)	683.906	2	38.38	348778	38.27	503425	1.87503	
EYTAAIEAK	Phb2	Prohibitin	498.256	2	17.18	20133	17.23	257332	16.6038	
IVQAEGEAEAAK	Phb2	Prohibitin	608.314	2	13.11	424400	13.11	493702	1.51116	
LGGIQSDIYSEGLHVR	Phb2	Prohibitin	581.972	3	33.76	567085	33.65	472144	1.08156	
EYTAAIEAK	Phb2	Prohibitin	498.256	2	17.18	20133	17.23	257332	16.6038	
IVQAEGEAEAAK	Phb2	Prohibitin	608.314	2	13.11	424400	13.11	493702	1.51116	
LGGIQSDIYSEGLHVR	Phb2	Prohibitin	581.972	3	33.76	567085	33.65	472144	1.08156	

AIYEQSR	hts	Hu li tai shao, isoform P	433.722	2	11.43	989317	11.54	662957	0.87051
DATVSQAGDHVVMMGAASK	hts	Hu li tai shao, isoform P	636.293	3	17.09	884028	17.06	1039090	1.5269
DGGAGASGILQQLSDIVGVPVSR	hts	Hu li tai shao, isoform P	1098.58	2	60.31	1852831	60.03	1591019	1.11548
DGGAGASGILQQLSDIVGVPVSR	hts	Hu li tai shao, isoform P	732.725	3	60.31	7576074	60.03	5964562	1.02272
FAAVAAAEDGAATAEK	hts	Hu li tai shao, isoform P	746.868	2	24.97	13124	25.1	319707	31.6452
LLDLYGWTQGLGAQITAR	hts	Hu li tai shao, isoform P	988.534	2	53.56	2279326	53.29	1565566	0.89225
LLDLYGWTQGLGAQITAR	hts	Hu li tai shao, isoform P	659.358	3	53.56	1975907	53.25	1298057	0.85339
LLPVGLDNLVLIPEESR	hts	Hu li tai shao, isoform P	626.363	3	55.71	938166	55.45	1069288	1.4806
MLDNAGYR	hts	Hu li tai shao, isoform P	470.221	2	16.11	947899	16.1	1485204	2.03538
NDVELPPAVSSLGYLLEEEELFR	hts	Hu li tai shao, isoform P	873.778	3	65.17	1366923	64.85	956167	0.90868
SHFVLHSVVHAARPDIR	hts	Hu li tai shao, isoform P	486.02	4	24.42	11051	23.97	0	0
SSNCMVPINDIR	hts	Hu li tai shao, isoform P	711.329	2	24.43	633585	24.05	1332102	2.73121
SVHGEYTDTDFSESEAVLQAGTK	hts	Hu li tai shao, isoform P	824.378	3	32.37	929683	32.29	1063232	1.48565
TGLLPLTK	hts	Hu li tai shao, isoform P	421.771	2	32.52	2862087	32.37	2725569	1.23708
TGYIYR	hts	Hu li tai shao, isoform P	386.703	2	16.34	1779020	16.29	796815	0.58183
VEVLETGTPDPK	hts	Hu li tai shao, isoform P	642.838	2	23.08	3487670	23.1	2329013	0.86748
DTAALLEGYVLIGLVPVEK	CG5477	GH01011p	1000.57	2	62.74	438531	62.45	679195	2.01195
DTAALLEGYVLIGLVPVEK	CG5477	GH01011p	667.382	3	62.73	263834	62.5	456206	2.24622
NFILDQTNVYASAQR	CG5477	GH01011p	870.439	2	36.23	646640	36.09	453785	0.91161
STSISNVAGASLTR	ens	Ensconsin, isoform F	682.362	2	25.86	816429	25.79	694557	1.10513
GYAFVTFTNR	Syp	Syncrip, isoform H	588.296	2	35.44	514118	35.33	604713	1.52795
HHDIDVVLIR	CG5028	MIP04243p	406.231	3	27.73	408874	27.61	208947	0.66385
HHDIDVVLIR	CG5028	Uncharacterized protein, isoform D (EC 1.1.1.41)	406.231	3	27.73	408874	27.61	208947	0.66385
FTILPHDFSIPTGELGPTLK	bgm	Bubblegum, isoform C (EC 6.2.1.3) (FI05443p)	728.396	3	49.95	906351	49.6	472601	0.67736
AIETFHGTVQHVESR	ple	FI04429p (Pale, isoform C) (EC 1.14.16.2)	570.957	3	18.83	39913	18.92	134503	4.37764
GNLLQLIR	ple	FI04429p (Pale, isoform C) (EC 1.14.16.2)	463.793	2	37.15	0	41.79	173479	#DIV/0!
YGDPIPFIDYSDVEVK	ple	FI04429p (Pale, isoform C) (EC 1.14.16.2)	928.951	2	47.5	0	47.3	113828	#DIV/0!
SIFSNFPQQVK	prom	Prominin, isoform D	647.843	2	36.49	1196743	36.37	1335180	1.44931
TLQEDQSIFR	prom	Prominin, isoform D	618.815	2	28.08	1390520	28.05	1856704	1.73455
VALQIQDVATSSR	prom	Prominin, isoform D	694.381	2	29.91	3286792	29.77	3985799	1.57531
DTSFEQLVLR	FASN1	Fatty acid synthase 1, isoform C (EC 1.-.-.-) (EC 2.3.1.85) (EC 3.1.-.-)	604.32	2	40.82	404038	40.66	103733	0.33352
LALVGGIFNLAAVLR	FASN1	Fatty acid synthase 1, isoform C (EC 1.-.-.-) (EC 2.3.1.85) (EC 3.1.-.-)	763.974	2	64.23	79694	63.78	0	0
NFDIVLSAQEIR	FASN1	Fatty acid synthase 1, isoform C (EC 1.-.-.-) (EC 2.3.1.85) (EC 3.1.-.-)	702.878	2	41.81	372388	41.64	404923	1.41253
DITLFHAMNILHK	MTA1-like	Metastasis associated 1-like, isoform C	518.282	3	43.88	2053379	43.78	955106	0.60423
DLPNPLVQLADK	MTA1-like	Metastasis associated 1-like, isoform C	661.869	2	44.73	4867516	44.55	2835007	0.7566
ELTIIPTQATSTIR	MTA1-like	Metastasis associated 1-like, isoform C	772.438	2	37.83	11123584	37.63	12065403	1.40903
HQPALDEER	MTA1-like	Metastasis associated 1-like, isoform C	547.765	2	10.77	1401862	10.88	390542	0.3619
HYAQAHGIAISSGSPRPIMK	MTA1-like	Metastasis associated 1-like, isoform C	531.031	4	22.34	2400440	22.14	3061997	1.65705
HYAQAHGIAISSGSPRPIMK	MTA1-like	Metastasis associated 1-like, isoform C	713.037	3	18.86	1475021	18.76	1372104	1.2084
HYAQAHGIAISSGSPRPIMK	MTA1-like	Metastasis associated 1-like, isoform C	535.03	4	18.86	8277828	18.73	11433715	1.79429
IDQFLVVSR	MTA1-like	Metastasis associated 1-like, isoform C	538.809	2	33.15	2031489	33.09	2200379	1.40704
ILSAVDLR	MTA1-like	Metastasis associated 1-like, isoform C	443.771	2	29.06	3480815	28.94	3278001	1.22335
LCLSCWEYWR	MTA1-like	Metastasis associated 1-like, isoform C	736.826	2	45.03	1100791	44.85	1003854	1.18464
LGAPGSGPHEWLVLTPK	MTA1-like	Metastasis associated 1-like, isoform C	586.989	3	38.45	4162383	38.27	5011628	1.56408
LLLLKPK	MTA1-like	Metastasis associated 1-like, isoform C	412.802	2	25.48	7404797	25.43	7870932	1.38081
QIIEYYYMWK	MTA1-like	Metastasis associated 1-like, isoform C	718.85	2	43.1	621851	42.87	464183	0.96967
QPSLHMSAAAASR	MTA1-like	Metastasis associated 1-like, isoform C	448.223	3	11.24	631709	11.36	455066	0.93579
QSAYAINAMLVK	MTA1-like	Metastasis associated 1-like, isoform C	654.853	2	35.58	1490566	35.41	2100155	1.8303
QSAYAINAMLVK	MTA1-like	Metastasis associated 1-like, isoform C	662.85	2	29.32	5849027	29.21	6763609	1.50216
QVESIPATQIR	MTA1-like	Metastasis associated 1-like, isoform C	621.346	2	23.95	29583954	23.96	39601180	1.7389
SIGTFAR	MTA1-like	Metastasis associated 1-like, isoform C	376.208	2	18.93	21987752	18.88	27082934	1.60006
SSQWNSVSSGHSTSR	MTA1-like	Metastasis associated 1-like, isoform C	536.245	3	13.07	1701975	13.07	2157652	1.64684
SSSAASTPTATLAGLATTPTAVVDL	MTA1-like	Metastasis associated 1-like, isoform C	968.816	3	46.62	1694407	46.37	1458500	1.11818

TAFYLHTNPMTR	MTA1-like	Metastasis associated 1-like, isoform C	726.359	2	28.65	3357775	28.53	4332503	1.67614	
TAFYLHTNPMTR	MTA1-like	Metastasis associated 1-like, isoform C	484.575	3	28.65	6289877	28.53	6959267	1.43729	
TAFYLHTNPMTR	MTA1-like	Metastasis associated 1-like, isoform C	734.356	2	22.69	3508484	22.72	3475736	1.28691	
TAFYLHTNPMTR	MTA1-like	Metastasis associated 1-like, isoform C	489.906	3	22.69	11884596	22.72	15231011	1.66482	
TLPIKPAAPEPSSRPIESQIVILD	MTA1-like	Metastasis associated 1-like, isoform C	857.818	3	41.39	34286420	41.22	42519524	1.61097	
VMCFYR	MTA1-like	Metastasis associated 1-like, isoform C	446.196	2	18.26	2536584	18.3	3037729	1.55569	
YQCDIPAK	MTA1-like	Metastasis associated 1-like, isoform C	497.737	2	14.9	10434692	14.9	5215218	0.64925	
AQELWELIVK	up	MIP03205p (Upheld, isoform M)	614.85	2	48.34	857786	48.15	594529	0.90036	
DAGLGLSSAAMER	up	MIP03205p (Upheld, isoform M)	647.309	2	23.24	1496095	23.26	1558348	1.35309	
IKPLAIEGFGEAK	up	MIP03205p (Upheld, isoform M)	458.266	3	33.5	4523125	33.37	3961091	1.13762	
ISLSFR	up	MIP03205p (Upheld, isoform M)	361.713	2	30.22	1860854	30.16	1791066	1.25032	
AIYDHHYSR	Mf	MIP03096p (Myofilin, isoform K) (Myofilin, isoform P)	581.275	2	11.31	475566	11.41	651391	1.77932	
AIYDHHYSR	Mf	MIP03096p (Myofilin, isoform K) (Myofilin, isoform P)	387.853	3	11.31	2895978	11.41	4746762	2.12924	
ASRPYSSYLDSPSYR	Mf	MIP03096p (Myofilin, isoform K) (Myofilin, isoform P)	874.916	2	25.08	1194562	25.03	1397150	1.51935	
ASRPYSSYLDSPSYR	Mf	MIP03096p (Myofilin, isoform K) (Myofilin, isoform P)	583.613	3	25.12	5487893	25.03	8501748	2.01245	
DTYGYSPR	Mf	MIP03096p (Myofilin, isoform K) (Myofilin, isoform P)	479.717	2	14.86	2640775	14.83	2840777	1.39742	
FWQSYIR	Mf	MIP03096p (Myofilin, isoform K) (Myofilin, isoform P)	500.256	2	32.37	739255	32.25	1687659	2.9656	
NHLEMIGR	Mf	MIP03096p (Myofilin, isoform K) (Myofilin, isoform P)	493.248	2	12.87	1554021	12.91	1080925	0.90357	
SIYDEPATANER	Mf	MIP03096p (Myofilin, isoform K) (Myofilin, isoform P)	683.318	2	18.42	9216683	18.42	15046478	2.12072	
AGSEPYVPPPSYWNR	Mf	MIP06826p (Myofilin, isoform F) (Myofilin, isoform J)	860.41	2	33.23	748487	33.06	1566838	2.71933	
AIYDHHYSR	Mf	MIP06826p (Myofilin, isoform F) (Myofilin, isoform J)	581.275	2	11.31	475566	11.41	651391	1.77932	
AIYDHHYSR	Mf	MIP06826p (Myofilin, isoform F) (Myofilin, isoform J)	387.853	3	11.31	2895978	11.41	4746762	2.12924	
ASRPYSSYLDSPSYR	Mf	MIP06826p (Myofilin, isoform F) (Myofilin, isoform J)	874.916	2	25.08	1194562	25.03	1397150	1.51935	
ASRPYSSYLDSPSYR	Mf	MIP06826p (Myofilin, isoform F) (Myofilin, isoform J)	583.613	3	25.12	5487893	25.03	8501748	2.01245	
ASSLEPLDELFER	Mf	MIP06826p (Myofilin, isoform F) (Myofilin, isoform J)	753.378	2	48.45	4498040	48.19	5102070	1.47348	
DKPLTPNSLVPLEYEPEDK	Mf	MIP06826p (Myofilin, isoform F) (Myofilin, isoform J)	728.707	3	38.98	567161	38.81	728559	1.66871	
DSYLSPVK	Mf	MIP06826p (Myofilin, isoform F) (Myofilin, isoform J)	454.74	2	22.04	1523720	22.02	1430945	1.21994	
DTYGYSPR	Mf	MIP06826p (Myofilin, isoform F) (Myofilin, isoform J)	479.717	2	14.86	2640775	14.83	2840777	1.39742	
FWQSYIR	Mf	MIP06826p (Myofilin, isoform F) (Myofilin, isoform J)	500.256	2	32.37	739255	32.25	1687659	2.9656	
GGPSVFDR	Mf	MIP06826p (Myofilin, isoform F) (Myofilin, isoform J)	417.709	2	19.86	5727839	19.85	7157511	1.62328	
LLAELNK	Mf	MIP06826p (Myofilin, isoform F) (Myofilin, isoform J)	400.747	2	22.38	846200	22.35	1253332	1.92405	
LPIFHPR	Mf	MIP06826p (Myofilin, isoform F) (Myofilin, isoform J)	440.264	2	24.47	512417	24.48	315832	0.80067	
NHLEMIGR	Mf	MIP06826p (Myofilin, isoform F) (Myofilin, isoform J)	493.248	2	12.87	1554021	12.91	1080925	0.90357	
SASPFRPAR	Mf	MIP06826p (Myofilin, isoform F) (Myofilin, isoform J)	494.77	2	13.46	1272464	13.42	1443660	1.47381	
SIYDEPATANER	Mf	MIP06826p (Myofilin, isoform F) (Myofilin, isoform J)	683.318	2	18.42	9216683	18.42	15046478	2.12072	
YGLYLR	Mf	MIP06826p (Myofilin, isoform F) (Myofilin, isoform J)	392.721	2	28.26	5499678	28.16	8903475	2.10303	
IIGEYGLR	RpS9	LD13662p (Ribosomal protein S9, isoform E)	460.764	2	25.97	1566590	25.94	2123615	1.76093	
LFQGNALLR	RpS9	LD13662p (Ribosomal protein S9, isoform E)	516.303	2	32.83	1000199	32.72	375882	0.48819	
ILDELEEEVYEINAEQQFLLLR	Lint-1	IP16414p (L(3)mbt interacting protein 1, isoform C) (L(3)mbt interacting protei	902.801	3	61.89	784107	61.7	561166	0.92969	
LNDSLQEFSVK	Lint-1	IP16414p (L(3)mbt interacting protein 1, isoform C) (L(3)mbt interacting protei	640.33	2	31.72	1131452	31.63	1444369	1.6583	
TQPEEYGQETFLGLFELYSPEVLK	Lint-1	IP16414p (L(3)mbt interacting protein 1, isoform C) (L(3)mbt interacting protei	939.8	3	64.56	308483	64.3	525500	2.21291	
TVQNASGVDFHYGQQLNAVDALVM	Lint-1	IP16414p (L(3)mbt interacting protein 1, isoform C) (L(3)mbt interacting protei	969.147	3	46.58	2486428	46.37	2112926	1.1039	
APAVGIDLGTTYSCVGVFQHGK	Hsc70-4	Heat shock protein cognate 4, isoform G (EC 3.6.1.3) (LD25749p)	759.716	3	40.45	5148820	40.23	3011430	0.75978	
ATLDEDNLK	Hsc70-4	Heat shock protein cognate 4, isoform G (EC 3.6.1.3) (LD25749p)	509.756	2	17.44	12568892	17.45	9571772	0.98928	
DAGTIAGLNVLR	Hsc70-4	Heat shock protein cognate 4, isoform G (EC 3.6.1.3) (LD25749p)	600.341	2	38.18	41262500	38.03	54952584	1.73004	
ELEGVCNPIITK	Hsc70-4	Heat shock protein cognate 4, isoform G (EC 3.6.1.3) (LD25749p)	686.861	2	30.64	3930112	30.51	6693284	2.21236	
ETAEAYLGK	Hsc70-4	Heat shock protein cognate 4, isoform G (EC 3.6.1.3) (LD25749p)	491.248	2	18.78	1276400	18.74	1203743	1.22509	
FEELNADLFR	Hsc70-4	Heat shock protein cognate 4, isoform G (EC 3.6.1.3) (LD25749p)	627.312	2	40.11	5534845	39.92	4957492	1.16353	
FELSGIPPAPR	Hsc70-4	Heat shock protein cognate 4, isoform G (EC 3.6.1.3) (LD25749p)	592.327	2	35.41	8081636	34.58	6361236	1.0225	
HWPFEVVSADGKPK	Hsc70-4	Heat shock protein cognate 4, isoform G (EC 3.6.1.3) (LD25749p)	532.944	3	32.25	1810179	32.17	1759279	1.26251	
LLQDLFNGK	Hsc70-4	Heat shock protein cognate 4, isoform G (EC 3.6.1.3) (LD25749p)	524.295	2	37.39	815568	37.18	536116	0.85393	
LVTHFVQEFK	Hsc70-4	Heat shock protein cognate 4, isoform G (EC 3.6.1.3) (LD25749p)	416.564	3	29.17	6951855	29.06	4441078	0.82987	
NQVAMNPTQTIFDAK	Hsc70-4	Heat shock protein cognate 4, isoform G (EC 3.6.1.3) (LD25749p)	839.417	2	35.8	3572564	35.58	4997890	1.81731	

NQVAMNPTQTIFDAK	Hsc70-4	Heat shock protein cognate 4, isoform G (EC 3.6.1.3) (LD25749p)	847.414	2	30.99	17359498	30.83	24582760	1.83957
NVLIFDLGGGTFDVSILSIDDGIFEVI	Hsc70-4	Heat shock protein cognate 4, isoform G (EC 3.6.1.3) (LD25749p)	961.839	3	65.61	296742	65.62	318618	1.39481
QTQTFTTYSDNQPGVLIQVYEGER	Hsc70-4	Heat shock protein cognate 4, isoform G (EC 3.6.1.3) (LD25749p)	925.447	3	42.59	6743949	42.36	8544964	1.64596
SINPDEAVAYGAAVQAAILHGDK	Hsc70-4	Heat shock protein cognate 4, isoform G (EC 3.6.1.3) (LD25749p)	770.729	3	49.12	5844145	48.87	6076260	1.35063
STAGDTHLGGEDFDNR	Hsc70-4	Heat shock protein cognate 4, isoform G (EC 3.6.1.3) (LD25749p)	846.366	2	18.67	1657789	18.65	1831600	1.43524
STAGDTHLGGEDFDNR	Hsc70-4	Heat shock protein cognate 4, isoform G (EC 3.6.1.3) (LD25749p)	564.58	3	18.67	10781481	18.68	14815577	1.7851
SVIHDIVLVGGSTR	Hsc70-4	Heat shock protein cognate 4, isoform G (EC 3.6.1.3) (LD25749p)	726.912	2	33.42	16676664	33.29	17014050	1.32532
SVIHDIVLVGGSTR	Hsc70-4	Heat shock protein cognate 4, isoform G (EC 3.6.1.3) (LD25749p)	484.944	3	33.42	25909346	33.29	28104442	1.4091
TFFPEEISSMVLTK	Hsc70-4	Heat shock protein cognate 4, isoform G (EC 3.6.1.3) (LD25749p)	814.916	2	50.99	8350954	50.71	10307238	1.60335
TFFPEEISSMVLTK	Hsc70-4	Heat shock protein cognate 4, isoform G (EC 3.6.1.3) (LD25749p)	543.613	3	50.99	1053530	50.71	1236310	1.52441
TFFPEEISSMVLTK	Hsc70-4	Heat shock protein cognate 4, isoform G (EC 3.6.1.3) (LD25749p)	822.913	2	44.06	1805317	44.17	2932806	2.11034
TFFPEEISSMVLTK	Hsc70-4	Heat shock protein cognate 4, isoform G (EC 3.6.1.3) (LD25749p)	548.944	3	43.99	365196	44.13	736202	2.61875
TLSSSTQASIEIDSLFEGTDFYTSITI	Hsc70-4	Heat shock protein cognate 4, isoform G (EC 3.6.1.3) (LD25749p)	990.48	3	57.43	13069925	57.18	15772457	1.56765
TTPSYVAFTDTER	Hsc70-4	Heat shock protein cognate 4, isoform G (EC 3.6.1.3) (LD25749p)	744.354	2	29.29	49722228	29.17	61082460	1.59584
TVTNAVITVPAYFNDSQR	Hsc70-4	Heat shock protein cognate 4, isoform G (EC 3.6.1.3) (LD25749p)	998.51	2	40.66	11484469	40.5	14976923	1.69408
TVTNAVITVPAYFNDSQR	Hsc70-4	Heat shock protein cognate 4, isoform G (EC 3.6.1.3) (LD25749p)	666.009	3	40.66	13980052	40.46	17266742	1.60444
VEIIANDQGNR	Hsc70-4	Heat shock protein cognate 4, isoform G (EC 3.6.1.3) (LD25749p)	614.818	2	18.24	20421124	18.27	30231408	1.9231
ALVDVLR	Rm62	LP18603p (Rm62, isoform I)	393.248	2	32.4	917820	32.25	781548	1.10617
EANQEINPALENLAR	Rm62	LP18603p (Rm62, isoform I)	841.429	2	32.48	1286441	32.37	785963	0.79366
ELAQQIQQVATEFGSSSYVR	Rm62	LP18603p (Rm62, isoform I)	747.71	3	48.34	417102	48.12	508191	1.58273
FGGGGGFGDR	Rm62	LP18603p (Rm62, isoform I)	463.709	2	17.67	571805	17.72	448173	1.01817
NTCVFGGAPK	Rm62	LP18603p (Rm62, isoform I)	525.755	2	19.91	235668	19.91	262752	1.44833
SNILVATDVAAR	Rm62	LP18603p (Rm62, isoform I)	615.346	2	29.95	1490640	29.85	1916326	1.67001
YVINFDYPQNSEDYIHR	Rm62	LP18603p (Rm62, isoform I)	725.004	3	37.84	686584	37.79	447734	0.84713
ALQASVLR	Ald1	Fructose-bisphosphate aldolase (EC 4.1.2.13)	429.264	2	22.24	109791	22.21	356378	4.21664
IAAEVAAPLAK	Flo2	Flo-2-PD (Flo-2-PH) (Flotillin 2, isoform H) (GH22754p)	527.319	2	25.68	101116	25.6	592504	7.61191
ILESGCHVVVGTPGR	eIF4A	Eukaryotic translation initiation factor 4A, isoform E (EC 2.7.7.-) (EC 3.6.1.3) (	527.612	3	22.54	32536	22.6	97954	3.91093
LGTLCDLYDTLSITQSVIFCNTR	eIF4A	Eukaryotic translation initiation factor 4A, isoform E (EC 2.7.7.-) (EC 3.6.1.3) (	897.444	3	65.14	15710	65.03	0	0
VLITTDLLAR	eIF4A	Eukaryotic translation initiation factor 4A, isoform E (EC 2.7.7.-) (EC 3.6.1.3) (	557.845	2	40.07	957169	39.92	1532706	2.08014
ISGATTFDFIPQK	CaMKII	Calcium/calmodulin-dependent protein kinase II, isoform I (EC 2.7.11.-) (EC 2	712.875	2	38.6	1118955	38.42	1549927	1.79937
ISGATTFDFIPQK	CaMKII	Calcium/calmodulin-dependent protein kinase II, isoform J (EC 2.7.11.-) (EC 2	712.875	2	38.6	1118955	38.42	1549927	1.79937
LLEANPNYSK	mfas	MIP15217p (Midline fasciclin, isoform Q)	574.801	2	18.6	422357	18.57	399268	1.22802
LNLYSTHALFSDVMNR	mfas	MIP15217p (Midline fasciclin, isoform Q)	632.981	3	38.73	372398	38.45	772695	2.6954
SAGLTPDLSNFTETR	sbb	Scribbler, isoform G	804.897	2	36.67	741872	36.56	971672	1.70143
TSPGFGSVGVGAASSK	sbb	Scribbler, isoform G	704.857	2	24.77	1117813	24.76	1320748	1.53488
DTAALLEGYVLIGLVPVEK	CG18610	Uncharacterized protein, isoform C	1000.57	2	62.74	438531	62.45	679195	2.01195
DTAALLEGYVLIGLVPVEK	CG18610	Uncharacterized protein, isoform C	667.382	3	62.73	263834	62.5	456206	2.24622
NFILDQTNVYASAQR	CG18610	Uncharacterized protein, isoform C	870.439	2	36.23	646640	36.09	453785	0.91161
ILIPSSIAGAVIGK	HnRNP-K	Heterogeneous nuclear ribonucleoprotein K, isoform E	669.921	2	46.3	366013	46.1	1322236	4.69283
NFPALTYFR	l(2)01289	Lethal (2) 01289, isoform J	564.795	2	42.88	433584	42.79	472450	1.41548
NFPALTYFR	l(2)01289	Lethal (2) 01289, isoform F	564.795	2	42.88	433584	42.79	472450	1.41548
NFPALTYFR	l(2)01289	Lethal (2) 01289, isoform G	564.795	2	42.88	433584	42.79	472450	1.41548
DFYQIR	CG1620	Uncharacterized protein, isoform B	421.214	2	25.63	4058468	25.64	3651345	1.16873
DNEQALHLLVQCGYDFK	CG1620	Uncharacterized protein, isoform B	683.995	3	42.88	848275	42.64	432717	0.66266
ERPLPADRPSK	CG1620	Uncharacterized protein, isoform B	422.57	3	9.9	322262	10.02	407848	1.64404
SHLLDLYPDESFVDLAPTCGEETEF	CG1620	Uncharacterized protein, isoform B	965.111	3	46.58	8771567	46.33	5071262	0.75104
SSDHSQHSASEASADSGSLANSDL	CG1620	Uncharacterized protein, isoform B	740.089	4	22.78	177697	22.79	299818	2.19179
YGGTAASPAMSSSNR	CG1620	Uncharacterized protein, isoform B	728.828	2	13.99	2532481	13.97	2794880	1.43364
YGGTAASPAMSSSNR	CG1620	Uncharacterized protein, isoform B	736.825	2	10.01	2951260	10.18	1942943	0.85521
VFLGGLPSNVTETDLR	Hrb27C	Heterogeneous nuclear ribonucleoprotein at 27C, isoform D (Heterogeneous ı	859.46	2	41.76	616996	41.61	670475	1.41164
ALDSMQASLEAEAK	Mhc	Myosin heavy chain, isoform O (EC 3.6.1.3)	732.356	2	29.64	321135	29.49	790332	3.19701
ALDSMQASLEAEAK	Mhc	Myosin heavy chain, isoform O (EC 3.6.1.3)	740.353	2	21.04	48359	20.88	260236	6.99057
DLEEANIQHESTLANLR	Mhc	Myosin heavy chain, isoform O (EC 3.6.1.3)	651.661	3	31.08	667254	31	1249882	2.43333

GALQDYQER	Mhc	Myosin heavy chain, isoform O (EC 3.6.1.3)	540.259	2	15.57	503772	15.55	1324414	3.41517
IEELEEEVEAER	Mhc	Myosin heavy chain, isoform O (EC 3.6.1.3)	737.849	2	27.66	871072	27.57	2436877	3.63414
LAEAEETIESLNQK	Mhc	Myosin heavy chain, isoform O (EC 3.6.1.3)	787.899	2	29.52	307761	29.44	1119309	4.72453
LAGADIETYLLEK	Mhc	Myosin heavy chain, isoform O (EC 3.6.1.3)	718.388	2	42.99	649408	42.76	1212276	2.42497
LTQEAVADLER	Mhc	Myosin heavy chain, isoform O (EC 3.6.1.3)	622.828	2	25.2	753282	25.18	1645891	2.83835
NYSTELFR	Mhc	Myosin heavy chain, isoform O (EC 3.6.1.3)	515.254	2	28.61	584482	28.59	1201453	2.67029
QIEEAEEIAALNLAK	Mhc	Myosin heavy chain, isoform O (EC 3.6.1.3)	821.438	2	39.84	244676	39.61	641209	3.40432
QLEEAESQVSQLSK	Mhc	Myosin heavy chain, isoform O (EC 3.6.1.3)	788.397	2	24.66	238416	24.6	763584	4.16048
TALLDSLSGEK	Mhc	Myosin heavy chain, isoform O (EC 3.6.1.3)	567.306	2	31.71	635833	31.55	1551318	3.16942
TLLEQADR	Mhc	Myosin heavy chain, isoform O (EC 3.6.1.3)	473.254	2	18.74	809820	18.73	1724785	2.76674
VIAYFATVGASK	Mhc	Myosin heavy chain, isoform O (EC 3.6.1.3)	613.843	2	33.65	620379	33.48	1435185	3.0052
ALDSMQASLEAEAK	Mhc	Myosin heavy chain, isoform N (EC 3.6.1.3)	732.356	2	29.64	321135	29.49	790332	3.19701
ALDSMQASLEAEAK	Mhc	Myosin heavy chain, isoform N (EC 3.6.1.3)	740.353	2	21.04	48359	20.88	260236	6.99057
DLEEANIQHESTLANLR	Mhc	Myosin heavy chain, isoform N (EC 3.6.1.3)	651.661	3	31.08	667254	31	1249882	2.43333
GALQDYQER	Mhc	Myosin heavy chain, isoform N (EC 3.6.1.3)	540.259	2	15.57	503772	15.55	1324414	3.41517
IEELEEEVEAER	Mhc	Myosin heavy chain, isoform N (EC 3.6.1.3)	737.849	2	27.66	871072	27.57	2436877	3.63414
LAEAEETIESLNQK	Mhc	Myosin heavy chain, isoform N (EC 3.6.1.3)	787.899	2	29.52	307761	29.44	1119309	4.72453
LAGADIETYLLEK	Mhc	Myosin heavy chain, isoform N (EC 3.6.1.3)	718.388	2	42.99	649408	42.76	1212276	2.42497
LTQEAVADLER	Mhc	Myosin heavy chain, isoform N (EC 3.6.1.3)	622.828	2	25.2	753282	25.18	1645891	2.83835
NYSTELFR	Mhc	Myosin heavy chain, isoform N (EC 3.6.1.3)	515.254	2	28.61	584482	28.59	1201453	2.67029
QIEEAEEIAALNLAK	Mhc	Myosin heavy chain, isoform N (EC 3.6.1.3)	821.438	2	39.84	244676	39.61	641209	3.40432
QLEEAESQVSQLSK	Mhc	Myosin heavy chain, isoform N (EC 3.6.1.3)	788.397	2	24.66	238416	24.6	763584	4.16048
TALLDSLSGEK	Mhc	Myosin heavy chain, isoform N (EC 3.6.1.3)	567.306	2	31.71	635833	31.55	1551318	3.16942
TLLEQADR	Mhc	Myosin heavy chain, isoform N (EC 3.6.1.3)	473.254	2	18.74	809820	18.73	1724785	2.76674
VIAYFATVGASK	Mhc	Myosin heavy chain, isoform N (EC 3.6.1.3)	613.843	2	33.65	620379	33.48	1435185	3.0052
ALDSMQASLEAEAK	Mhc	Myosin heavy chain, isoform P (EC 3.6.1.3)	732.356	2	29.64	321135	29.49	790332	3.19701
ALDSMQASLEAEAK	Mhc	Myosin heavy chain, isoform P (EC 3.6.1.3)	740.353	2	21.04	48359	20.88	260236	6.99057
DLEEANIQHESTLANLR	Mhc	Myosin heavy chain, isoform P (EC 3.6.1.3)	651.661	3	31.08	667254	31	1249882	2.43333
GALQDYQER	Mhc	Myosin heavy chain, isoform P (EC 3.6.1.3)	540.259	2	15.57	503772	15.55	1324414	3.41517
IEELEEEVEAER	Mhc	Myosin heavy chain, isoform P (EC 3.6.1.3)	737.849	2	27.66	871072	27.57	2436877	3.63414
LAEAEETIESLNQK	Mhc	Myosin heavy chain, isoform P (EC 3.6.1.3)	787.899	2	29.52	307761	29.44	1119309	4.72453
LAGADIETYLLEK	Mhc	Myosin heavy chain, isoform P (EC 3.6.1.3)	718.388	2	42.99	649408	42.76	1212276	2.42497
LTQEAVADLER	Mhc	Myosin heavy chain, isoform P (EC 3.6.1.3)	622.828	2	25.2	753282	25.18	1645891	2.83835
NYSTELFR	Mhc	Myosin heavy chain, isoform P (EC 3.6.1.3)	515.254	2	28.61	584482	28.59	1201453	2.67029
QIEEAEEIAALNLAK	Mhc	Myosin heavy chain, isoform P (EC 3.6.1.3)	821.438	2	39.84	244676	39.61	641209	3.40432
QLEEAESQVSQLSK	Mhc	Myosin heavy chain, isoform P (EC 3.6.1.3)	788.397	2	24.66	238416	24.6	763584	4.16048
TALLDSLSGEK	Mhc	Myosin heavy chain, isoform P (EC 3.6.1.3)	567.306	2	31.71	635833	31.55	1551318	3.16942
TLLEQADR	Mhc	Myosin heavy chain, isoform P (EC 3.6.1.3)	473.254	2	18.74	809820	18.73	1724785	2.76674
VIAYFATVGASK	Mhc	Myosin heavy chain, isoform P (EC 3.6.1.3)	613.843	2	33.65	620379	33.48	1435185	3.0052
ADNSAPAAQDDGSGAPVVR	Mi-2	Mi-2, isoform C (EC 3.6.1.3)	899.422	2	16.91	470838	16.88	285548	0.78783
AVAAATSAATGATGK	Mi-2	Mi-2, isoform C (EC 3.6.1.3)	624.333	2	13.11	1665996	13.11	983701	0.76703
HHYEQHQEDVGR	Mi-2	Mi-2, isoform C (EC 3.6.1.3)	512.231	3	7.62	201593	7.78	286609	1.84687
HHYEQHQEDVGR	Mi-2	Mi-2, isoform C (EC 3.6.1.3)	384.425	4	7.62	91019	7.78	138645	1.97877
IDGGITGTLR	Mi-2	Mi-2, isoform C (EC 3.6.1.3)	501.783	2	24.03	1258518	24.04	897889	0.9268
QAIDYYQDLR	Mi-2	Mi-2, isoform C (EC 3.6.1.3)	642.815	2	28.79	539792	28.68	906085	2.18054
SVTGDEPEEPR	Mi-2	Mi-2, isoform C (EC 3.6.1.3)	608.278	2	13.23	438146	13.27	556388	1.64961
VGGNIEVLGFNAR	Mi-2	Mi-2, isoform C (EC 3.6.1.3)	673.365	2	37.32	2342650	37.14	3356129	1.86103
AELDDNEDVGPR	CG32355	Uncharacterized protein, isoform E	665.299	2	17.49	854844	17.49	358372	0.54459
AGTNDTDPVIEVEPAGVR	CG32355	Uncharacterized protein, isoform E	920.458	2	31.26	799045	31.12	994850	1.61737
ELTPFEQELQR	CG32355	Uncharacterized protein, isoform E	695.354	2	34.45	1822534	34.23	951564	0.67824
FATLPSIR	CG32355	Uncharacterized protein, isoform E	452.766	2	32.1	2652917	31.93	2934962	1.43715
FHDLQDAQSTASSGK	CG32355	Uncharacterized protein, isoform E	531.25	3	15.41	391086	15.36	283768	0.94257
GADSLILDGHIK	CG32355	Uncharacterized protein, isoform E	413.563	3	29.68	1264479	29.53	1469523	1.50969

GFMASAQDR	CG32355	Uncharacterized protein, isoform E	499.722	2	11.62	285875	11.75	165984	0.75424
GISEDNIQLR	CG32355	Uncharacterized protein, isoform E	572.801	2	25.08	2302903	24.99	2379743	1.34238
NYQPDFDSQEFQR	CG32355	Uncharacterized protein, isoform E	837.363	2	28.43	615106	28.34	745801	1.57505
RPLGPDEQPEIVLLLR	CG32355	Uncharacterized protein, isoform E	615.691	3	45.58	1022269	45.43	642182	0.81605
SVSNTFLQPHEDVIVYR	CG32355	Uncharacterized protein, isoform E	668.678	3	35.81	2074033	35.7	1364163	0.85442
TDYEIHTSQVDSSALEELPLQPENR	CG32355	Uncharacterized protein, isoform E	957.793	3	39.43	686104	39.2	452230	0.85623
THEDAQSLPGNSANPSR	CG32355	Uncharacterized protein, isoform E	594.278	3	13.23	675781	13.53	14072906	27.052
STVPFATSESNR	Zasp66	Z band alternatively spliced PDZ-motif protein 66, isoform M	648.315	2	21.89	228788	21.86	230532	1.30894
AVVQVFEGTSGIDAK	Vha55	Vacuolar proton pump subunit B (V-ATPase subunit B) (Vacuolar proton pump	760.901	2	33.68	936310	33.53	864164	1.19894
DFISQPR	Vha55	Vacuolar proton pump subunit B (V-ATPase subunit B) (Vacuolar proton pump	431.725	2	20.36	908159	20.31	646613	0.92492
FAEIVQLR	Vha55	Vacuolar proton pump subunit B (V-ATPase subunit B) (Vacuolar proton pump	488.285	2	33.47	1109279	33.37	993806	1.16381
NFISQGNYENR	Vha55	Vacuolar proton pump subunit B (V-ATPase subunit B) (Vacuolar proton pump	671.313	2	21.23	606233	21.27	286793	0.61454
NTLCEFTGDILR	Vha55	Vacuolar proton pump subunit B (V-ATPase subunit B) (Vacuolar proton pump	719.853	2	41.77	1059567	41.56	793668	0.97304
TPVSEDMLGR	Vha55	Vacuolar proton pump subunit B (V-ATPase subunit B) (Vacuolar proton pump	552.771	2	23.81	594688	23.84	576947	1.26029
AGSEPYVPPPSYWNR	Mf	Myofilin, isoform H	860.41	2	33.23	748487	33.06	1566838	2.71933
AIYDHHYSR	Mf	Myofilin, isoform H	581.275	2	11.31	475566	11.41	651391	1.77932
AIYDHHYSR	Mf	Myofilin, isoform H	387.853	3	11.31	2895978	11.41	4746762	2.12924
ASRPYSSYLDSPSYR	Mf	Myofilin, isoform H	874.916	2	25.08	1194562	25.03	1397150	1.51935
ASRPYSSYLDSPSYR	Mf	Myofilin, isoform H	583.613	3	25.12	5487893	25.03	8501748	2.01245
DKPLTPNSLVPLEYEPEDK	Mf	Myofilin, isoform H	728.707	3	38.98	567161	38.81	728559	1.66871
DTYGYSPR	Mf	Myofilin, isoform H	479.717	2	14.86	2640775	14.83	2840777	1.39742
FWQSYIR	Mf	Myofilin, isoform H	500.256	2	32.37	739255	32.25	1687659	2.9656
GGPSVFDR	Mf	Myofilin, isoform H	417.709	2	19.86	5727839	19.85	7157511	1.62328
LLAELNK	Mf	Myofilin, isoform H	400.747	2	22.38	846200	22.35	1253332	1.92405
NHLEMIGR	Mf	Myofilin, isoform H	493.248	2	12.87	1554021	12.91	1080925	0.90357
SASPFRPAR	Mf	Myofilin, isoform H	494.77	2	13.46	1272464	13.42	1443660	1.47381
SIYDEPATANER	Mf	Myofilin, isoform H	683.318	2	18.42	9216683	18.42	15046478	2.12072
YGLYLR	Mf	Myofilin, isoform H	392.721	2	28.26	5499678	28.16	8903475	2.10303
AGSEPYVPPPSYWNR	Mf	Myofilin, isoform I	860.41	2	33.23	748487	33.06	1566838	2.71933
AIYDHHYSR	Mf	Myofilin, isoform I	581.275	2	11.31	475566	11.41	651391	1.77932
AIYDHHYSR	Mf	Myofilin, isoform I	387.853	3	11.31	2895978	11.41	4746762	2.12924
ASRPYSSYLDSPSYR	Mf	Myofilin, isoform I	874.916	2	25.08	1194562	25.03	1397150	1.51935
ASRPYSSYLDSPSYR	Mf	Myofilin, isoform I	583.613	3	25.12	5487893	25.03	8501748	2.01245
ASSLEPLDELFEL	Mf	Myofilin, isoform I	731.869	2	63.39	1138144	63.1	1815952	2.07267
DKPLTPNSLVPLEYEPEDK	Mf	Myofilin, isoform I	728.707	3	38.98	567161	38.81	728559	1.66871
DTYGYSPR	Mf	Myofilin, isoform I	479.717	2	14.86	2640775	14.83	2840777	1.39742
FWQSYIR	Mf	Myofilin, isoform I	500.256	2	32.37	739255	32.25	1687659	2.9656
GGPSVFDR	Mf	Myofilin, isoform I	417.709	2	19.86	5727839	19.85	7157511	1.62328
LLAELNK	Mf	Myofilin, isoform I	400.747	2	22.38	846200	22.35	1253332	1.92405
NHLEMIGR	Mf	Myofilin, isoform I	493.248	2	12.87	1554021	12.91	1080925	0.90357
SASPFRPAR	Mf	Myofilin, isoform I	494.77	2	13.46	1272464	13.42	1443660	1.47381
SIYDEPATANER	Mf	Myofilin, isoform I	683.318	2	18.42	9216683	18.42	15046478	2.12072
YGLYLR	Mf	Myofilin, isoform I	392.721	2	28.26	5499678	28.16	8903475	2.10303
HPSKPEPSGECQPDLR	Caf1-55	Chromatin assembly factor 1, p55 subunit, isoform B (SD22845p)	611.956	3	13	2959819	12.95	2946280	1.2931
IEIEIK	Caf1-55	Chromatin assembly factor 1, p55 subunit, isoform B (SD22845p)	372.729	2	26.69	18490798	26.63	10128032	0.71153
IGEEQSTEDAEDGPPELLFIHGGHT	Caf1-55	Chromatin assembly factor 1, p55 subunit, isoform B (SD22845p)	959.789	3	36.67	5605498	36.49	6740038	1.56196
IGEEQSTEDAEDGPPELLFIHGGHT	Caf1-55	Chromatin assembly factor 1, p55 subunit, isoform B (SD22845p)	720.094	4	36.67	22180360	36.49	27435578	1.60682
INHEGEVNR	Caf1-55	Chromatin assembly factor 1, p55 subunit, isoform B (SD22845p)	534.265	2	7.93	6273340	8.06	3016533	0.62464
INHEGEVNR	Caf1-55	Chromatin assembly factor 1, p55 subunit, isoform B (SD22845p)	356.512	3	7.93	5037983	8.06	2395439	0.61766
LHVWDLSK	Caf1-55	Chromatin assembly factor 1, p55 subunit, isoform B (SD22845p)	499.277	2	29.44	14247125	29.32	17649806	1.60929
LMIWDTR	Caf1-55	Chromatin assembly factor 1, p55 subunit, isoform B (SD22845p)	467.744	2	35.19	7330851	35.02	7635260	1.35298
NIFTGHTAVVEDVAWHLLHESLFGS	Caf1-55	Chromatin assembly factor 1, p55 subunit, isoform B (SD22845p)	1145.9	3	62.65	358249	62.54	531197	1.92616
NIFTGHTAVVEDVAWHLLHESLFGS	Caf1-55	Chromatin assembly factor 1, p55 subunit, isoform B (SD22845p)	859.68	4	62.68	6111397	62.65	6672885	1.41839

SDNAESFDDAVEER	Caf1-55	Chromatin assembly factor 1, p55 subunit, isoform B (SD22845p)	792.326	2	23.08	305817	23.18	177334	0.75327
TVALWDLR	Caf1-55	Chromatin assembly factor 1, p55 subunit, isoform B (SD22845p)	487.277	2	39.87	45980548	39.69	47007576	1.32805
VINEEYK	Caf1-55	Chromatin assembly factor 1, p55 subunit, isoform B (SD22845p)	447.732	2	12.61	10431289	12.66	5073242	0.63179
YMPQNACVIATK	Caf1-55	Chromatin assembly factor 1, p55 subunit, isoform B (SD22845p)	698.342	2	23.89	7995023	23.84	9614787	1.56222
YMPQNACVIATK	Caf1-55	Chromatin assembly factor 1, p55 subunit, isoform B (SD22845p)	706.339	2	20.03	1455765	20.05	1599258	1.42708
EVSGDASEAALLK	Atpalpha	Sodium/potassium-transporting ATPase subunit alpha	645.333	2	25.45	660865	25.35	279051	0.54852
IATLCNR	Atpalpha	Sodium/potassium-transporting ATPase subunit alpha	424.226	2	14.34	183981	14.32	209877	1.48188
LIIVEGCQR	Atpalpha	Sodium/potassium-transporting ATPase subunit alpha	544.3	2	24.31	677846	24.36	746923	1.43142
LNIPVSEVNPR	Atpalpha	Sodium/potassium-transporting ATPase subunit alpha	619.349	2	30.64	1527141	30.51	1174989	0.99949
NLAFFSTNAVEGTAK	Atpalpha	Sodium/potassium-transporting ATPase subunit alpha	785.399	2	38.55	1771624	38.01	15711079	11.5201
YHTEIVFAR	Atpalpha	Sodium/potassium-transporting ATPase subunit alpha	568.298	2	25.19	792742	25.03	558537	0.91526
YHTEIVFAR	Atpalpha	Sodium/potassium-transporting ATPase subunit alpha	379.201	3	25.23	368207	25.07	375193	1.32369
ALVDVLR	Rm62	Rm62, isoform H	393.248	2	32.4	917820	32.25	781548	1.10617
EANQEINPALENLAR	Rm62	Rm62, isoform H	841.429	2	32.48	1286441	32.37	785963	0.79366
ELAQQIQQVATEFGSSSYVR	Rm62	Rm62, isoform H	747.71	3	48.34	417102	48.12	508191	1.58273
FGGGGGFGDR	Rm62	Rm62, isoform H	463.709	2	17.67	571805	17.72	448173	1.01817
NTCVFGGAPK	Rm62	Rm62, isoform H	525.755	2	19.91	235668	19.91	262752	1.44833
SNILVATDVAAR	Rm62	Rm62, isoform H	615.346	2	29.95	1490640	29.85	1916326	1.67001
YVINFDYPQNSEDYIHR	Rm62	Rm62, isoform H	725.004	3	37.84	686584	37.79	447734	0.84713
VYSPHVLNLTLIDLPGLTK	shi	Shibire, isoform L	698.405	3	53.41	463587	53.25	477207	1.3372
VYSPHVLNLTLIDLPGLTK	shi	Shibire, isoform M	698.405	3	53.41	463587	53.25	477207	1.3372
ILPLDGLQAGYR	norpA	1-phosphatidylinositol 4,5-bisphosphate phosphodiesterase (EC 3.1.4.11)	658.372	2	39.05	1848340	38.89	1619786	1.13841
NDIEELFTSITK	norpA	1-phosphatidylinositol 4,5-bisphosphate phosphodiesterase (EC 3.1.4.11)	705.362	2	50.41	647218	50.1	519426	1.04255
VVLPDLAVLR	norpA	1-phosphatidylinositol 4,5-bisphosphate phosphodiesterase (EC 3.1.4.11)	547.85	2	45.88	627980	45.7	724721	1.49916
HPQLLYEAK	CkIalpha	Casein kinase ialpha, isoform D (EC 2.7.11.-) (Casein kinase ialpha, isoform E	549.801	2	20.1	592902	20.09	726533	1.59182
ILSGGVGFPR	CkIalpha	Casein kinase ialpha, isoform D (EC 2.7.11.-) (Casein kinase ialpha, isoform E	501.79	2	30.56	792616	30.4	1109698	1.81871
YASINAHLGIEQSR	CkIalpha	Casein kinase ialpha, isoform D (EC 2.7.11.-) (Casein kinase ialpha, isoform E	520.271	3	26.21	386181	26.15	468938	1.57742
IAAEVAAPLAK	Flo2	Flotillin 2, isoform G	527.319	2	25.68	101116	25.6	592504	7.61191
CGVISPR	RpS15Aa	GEO07866p1 (Ribosomal protein S15Aa, isoform F)	394.708	2	13.98	622408	13.82	550921	1.14984
FDVPINDIEK	RpS15Aa	GEO07866p1 (Ribosomal protein S15Aa, isoform F)	595.309	2	35.12	6034878	34.98	2750801	0.59212
HGYIGEFEIVDDHR	RpS15Aa	GEO07866p1 (Ribosomal protein S15Aa, isoform F)	562.934	3	31.11	2707218	31.08	907855	0.43563
MNVLADALK	RpS15Aa	GEO07866p1 (Ribosomal protein S15Aa, isoform F)	487.771	2	34.9	1815269	34.78	1716352	1.22825
QFGYVVLTTSGGIMDHEEAR	RpS15Aa	GEO07866p1 (Ribosomal protein S15Aa, isoform F)	737.356	3	39.87	1542845	39.68	2090883	1.76047
WTNNLLPSR	RpS15Aa	GEO07866p1 (Ribosomal protein S15Aa, isoform F)	550.796	2	30.38	2286379	30.28	1280460	0.72751
AQELWELIVK	up	Upheld, isoform H	614.85	2	48.34	857786	48.15	594529	0.90036
DAGVLGLSSAAMER	up	Upheld, isoform H	696.843	2	30.26	1579477	30.2	1875543	1.54254
IKPLAIEGFGEAK	up	Upheld, isoform H	458.266	3	33.5	4523125	33.37	3961091	1.13762
ISLSFR	up	Upheld, isoform H	361.713	2	30.22	1860854	30.16	1791066	1.25032
ALQALEHAR	RpS19a	Ribosomal protein S19a, isoform C (Ribosomal protein S19a, isoform D) (Ribo	504.783	2	16.22	955156	16.18	893690	1.21544
DIDQHAVTK	RpS19a	Ribosomal protein S19a, isoform C (Ribosomal protein S19a, isoform D) (Ribo	513.764	2	10.4	2341073	10.53	719059	0.399
IANQIVFK	RpS19a	Ribosomal protein S19a, isoform C (Ribosomal protein S19a, isoform D) (Ribo	466.782	2	26.32	23319114	26.31	14069064	0.78375
LSSIGQR	RpS19a	Ribosomal protein S19a, isoform C (Ribosomal protein S19a, isoform D) (Ribo	380.719	2	11.74	2374571	11.84	1340591	0.73339
QTGPIVISK	RpS19a	Ribosomal protein S19a, isoform C (Ribosomal protein S19a, isoform D) (Ribo	471.785	2	20.97	7892233	20.97	6770915	1.11447
SPAGVGSITK	RpS19a	Ribosomal protein S19a, isoform C (Ribosomal protein S19a, isoform D) (Ribo	458.759	2	15.88	11010122	15.86	5559005	0.65588
AAEFNFR	wupA	GH04081p (Wings up A, isoform H)	427.711	2	24.96	9042040	24.95	9905750	1.42313
GFMTPER	wupA	GH04081p (Wings up A, isoform H)	419.2	2	19.67	1284815	19.67	1246317	1.26012
IAAEVAAPLAK	Flo2	Flotillin 2, isoform K (LD15975p)	527.319	2	25.68	101116	25.6	592504	7.61191
STSISNVAGASLTR	ens	Ensconsin, isoform H (LD35939p)	682.362	2	25.86	816429	25.79	694557	1.10513
ALQASVLR	Ald1	Fructose-bisphosphate aldolase (EC 4.1.2.13)	429.264	2	22.24	109791	22.21	356378	4.21664
DLEAIVQPVIAK	Hsc70-3	Heat shock 70-kDa protein cognate 3, isoform E (EC 3.6.1.3) (LD03228p1)	648.382	2	41.32	544579	41.07	1292742	3.08371
ETAEAYLGK	Hsc70-3	Heat shock 70-kDa protein cognate 3, isoform E (EC 3.6.1.3) (LD03228p1)	491.248	2	18.78	1276400	18.74	1203743	1.22509
IEIESFFEGDDFSETLTR	Hsc70-3	Heat shock 70-kDa protein cognate 3, isoform E (EC 3.6.1.3) (LD03228p1)	1067.99	2	52.47	497399	52.17	1038290	2.71167
ITPSYVAFTADGER	Hsc70-3	Heat shock 70-kDa protein cognate 3, isoform E (EC 3.6.1.3) (LD03228p1)	763.878	2	32.4	751662	32.33	1189631	2.05595

IVITNDQNR	Hsc70-3	Heat shock 70-kDa protein cognate 3, isoform E (EC 3.6.1.3) (LD03228p1)	536.791	2	14.68	403680	14.64	383242	1.23327
NQLTTNPENTVFDAK	Hsc70-3	Heat shock 70-kDa protein cognate 3, isoform E (EC 3.6.1.3) (LD03228p1)	846.415	2	29.52	625548	29.44	1240840	2.57678
NSKPHISVDTSQGAK	Hsc70-3	Heat shock 70-kDa protein cognate 3, isoform E (EC 3.6.1.3) (LD03228p1)	523.606	3	12.41	133543	12.4	255862	2.4889
VEIIANDQGNR	Hsc70-3	Heat shock 70-kDa protein cognate 3, isoform E (EC 3.6.1.3) (LD03228p1)	614.818	2	18.24	20421124	18.27	30231408	1.9231
ACYGVLR	RpS3	Ribosomal protein S3, isoform B (EC 3.2.2.23) (EC 4.2.99.18) (RpS3-PA)	419.716	2	20.14	3016355	20.13	2508723	1.08042
AELNEFLTR	RpS3	Ribosomal protein S3, isoform B (EC 3.2.2.23) (EC 4.2.99.18) (RpS3-PA)	546.788	2	32.94	3256554	32.83	1378351	0.54982
ELAEDGYSGVEVR	RpS3	Ribosomal protein S3, isoform B (EC 3.2.2.23) (EC 4.2.99.18) (RpS3-PA)	712.339	2	24.58	10406436	24.6	12489490	1.55907
FVDGLMIHSGDPCNDYVETATR	RpS3	Ribosomal protein S3, isoform B (EC 3.2.2.23) (EC 4.2.99.18) (RpS3-PA)	838.374	3	33.19	433927	33.06	748456	2.24064
GCEVVVSGK	RpS3	Ribosomal protein S3, isoform B (EC 3.2.2.23) (EC 4.2.99.18) (RpS3-PA)	467.737	2	13.15	569662	13.23	217126	0.49513
GLCAIAQAESLR	RpS3	Ribosomal protein S3, isoform B (EC 3.2.2.23) (EC 4.2.99.18) (RpS3-PA)	644.838	2	32.06	4032273	31.93	2480082	0.79898
QGVLGIK	RpS3	Ribosomal protein S3, isoform B (EC 3.2.2.23) (EC 4.2.99.18) (RpS3-PA)	357.729	2	22.9	1613732	22.87	2249317	1.81068
TEIIIMATK	RpS3	Ribosomal protein S3, isoform B (EC 3.2.2.23) (EC 4.2.99.18) (RpS3-PA)	518.291	2	24	876921	24.27	939582	1.39186
VMLPYDPK	RpS3	Ribosomal protein S3, isoform B (EC 3.2.2.23) (EC 4.2.99.18) (RpS3-PA)	481.754	2	28.33	612094	28.34	988565	2.09802
VMLPYDPK	RpS3	Ribosomal protein S3, isoform B (EC 3.2.2.23) (EC 4.2.99.18) (RpS3-PA)	489.752	2	25.68	1125339	25.68	1698752	1.96096
LAMQVVLHTELER	TrpA1	TRPA1 isoform D (Transient receptor potential cation channel A1, isoform G)	518.948	3	27.31	103016	27.24	1044228	13.1678
MLEQQHHLVR	TrpA1	TRPA1 isoform D (Transient receptor potential cation channel A1, isoform G)	430.896	3	13.31	104134	13.27	657559	8.20284
NVIDIQQGGEHGR	TrpA1	TRPA1 isoform D (Transient receptor potential cation channel A1, isoform G)	474.908	3	18.12	345514	18.16	3305973	12.4296
SPLLLAASR	TrpA1	TRPA1 isoform D (Transient receptor potential cation channel A1, isoform G)	464.285	2	29.1	430144	29.02	1188200	3.58838
FVNLPIFESTVLNCFVR	Rtf1	FI16824p1 (Rtf1, isoform B)	1028.04	2	65.6	62022	62.58	33158	0.69449
LAMQVVLHTELER	TrpA1	TRPA1-C (Transient receptor potential cation channel A1, isoform H)	518.948	3	27.31	103016	27.24	1044228	13.1678
MLEQQHHLVR	TrpA1	TRPA1-C (Transient receptor potential cation channel A1, isoform H)	430.896	3	13.31	104134	13.27	657559	8.20284
NVIDIQQGGEHGR	TrpA1	TRPA1-C (Transient receptor potential cation channel A1, isoform H)	474.908	3	18.12	345514	18.16	3305973	12.4296
SPLLLAASR	TrpA1	TRPA1-C (Transient receptor potential cation channel A1, isoform H)	464.285	2	29.1	430144	29.02	1188200	3.58838
ISGATTFDFIPQK	CaMKII	Calcium/calmodulin-dependent protein kinase II, isoform M (EC 2.7.11.-) (EC	712.875	2	38.6	1118955	38.42	1549927	1.79937
ISGATTFDFIPQK	CaMKII	Calcium/calmodulin-dependent protein kinase II, isoform L (EC 2.7.11.-) (EC 2	712.875	2	38.6	1118955	38.42	1549927	1.79937
ISGATTFDFIPQK	CaMKII	Calcium/calmodulin-dependent protein kinase II, isoform K (EC 2.7.11.-) (EC 2	712.875	2	38.6	1118955	38.42	1549927	1.79937
HAQDYSLEASAK	apolpp	Apolipophorin, isoform B (Apolipophorin, isoform C) (Apolipophorin, isoform D	660.315	2	14.87	331176	14.8	247926	0.97249
FLSQPFQVAEVFTGHAGK	ATPsynbe	ATP synthase subunit beta (EC 7.1.2.2)	655.007	3	47.1	661096	46.95	927753	1.82301
LVLEVAQHLGENTVR	ATPsynbe	ATP synthase subunit beta (EC 7.1.2.2)	839.468	2	34.87	556208	34.71	748475	1.74808
ELEIEQR	eIF4B	Eukaryotic translation initiation factor 4B, isoform F	458.74	2	17.88	4591316	17.9	2801196	0.79255
QGGTSSLNVFGSAKPVDTAAR	eIF4B	Eukaryotic translation initiation factor 4B, isoform F	688.355	3	30.18	747833	30.12	321395	0.55829
TVAGGAWVYSTTAAASVR	RpL37A	GEO09637p1 (Ribosomal protein L37A, isoform C)	884.455	2	35.51	1995172	35.37	2313941	1.50659
TVAGGAWVYSTTAAASVR	RpL37A	GEO09637p1 (Ribosomal protein L37A, isoform C)	589.972	3	35.55	824979	35.33	1038904	1.63589
YTCSFCGK	RpL37A	GEO09637p1 (Ribosomal protein L37A, isoform C)	511.707	2	15.1	296011	15.08	174740	0.76684
AFIVKPIPSPEVIPVIVFINPK	rdgA	Diacylglycerol kinase (DAG kinase) (EC 2.7.1.107)	806.491	3	58.34	7861730	58.25	6537246	1.08019
AFIVKPIPSPEVIPVIVFINPK	rdgA	Diacylglycerol kinase (DAG kinase) (EC 2.7.1.107)	605.12	4	58.3	339552	58.29	268976	1.02903
ALGWGGGYTDEPIGK	rdgA	Diacylglycerol kinase (DAG kinase) (EC 2.7.1.107)	760.873	2	33.66	1454790	33.49	1166978	1.04204
ALHEQGYSLQSVNK	rdgA	Diacylglycerol kinase (DAG kinase) (EC 2.7.1.107)	525.27	3	20.1	844926	20.13	448176	0.68905
FEESISYPK	rdgA	Diacylglycerol kinase (DAG kinase) (EC 2.7.1.107)	550.269	2	25.08	699628	25.06	249796	0.46381
FQHLLNPR	rdgA	Diacylglycerol kinase (DAG kinase) (EC 2.7.1.107)	512.788	2	19.99	2182798	19.93	880908	0.52425
GDVQVNPLEK	rdgA	Diacylglycerol kinase (DAG kinase) (EC 2.7.1.107)	549.793	2	22.75	2434720	22.76	798326	0.42595
GPGSTDEDLQIGSKPIK	rdgA	Diacylglycerol kinase (DAG kinase) (EC 2.7.1.107)	581.304	3	23.2	1491415	23.18	425538	0.37065
LGNYAPIIVPPSWIVK	rdgA	Diacylglycerol kinase (DAG kinase) (EC 2.7.1.107)	884.014	2	52.63	3322956	52.44	1330008	0.51994
QVFDLTQGGPK	rdgA	Diacylglycerol kinase (DAG kinase) (EC 2.7.1.107)	595.314	2	28.22	3580063	28.2	2623698	0.95202
SFAAFSQR	rdgA	Diacylglycerol kinase (DAG kinase) (EC 2.7.1.107)	457.23	2	23.28	6655876	23.37	6106585	1.19183
TIPMQVDGEACR	rdgA	Diacylglycerol kinase (DAG kinase) (EC 2.7.1.107)	688.818	2	23.21	1029594	23.29	655361	0.82687
TIPMQVDGEACR	rdgA	Diacylglycerol kinase (DAG kinase) (EC 2.7.1.107)	696.816	2	17.7	628084	17.41	337537	0.69811
VKPSVIEIELLNK	rdgA	Diacylglycerol kinase (DAG kinase) (EC 2.7.1.107)	741.451	2	38.07	1106361	37.96	617198	0.72469
VKPSVIEIELLNK	rdgA	Diacylglycerol kinase (DAG kinase) (EC 2.7.1.107)	494.636	3	38.07	3645006	37.92	2591446	0.92356
VSQDWCFIDSCTAEHYFR	rdgA	Diacylglycerol kinase (DAG kinase) (EC 2.7.1.107)	774.33	3	41.88	500902	41.72	464633	1.20498
VTPNDDVTDDHVDR	rdgA	Diacylglycerol kinase (DAG kinase) (EC 2.7.1.107)	533.241	3	14.64	1483070	14.56	900190	0.78849
YIIASATR	rdgA	Diacylglycerol kinase (DAG kinase) (EC 2.7.1.107)	447.756	2	19.6	1081315	19.55	314198	0.37746
LAVAEFR	Hsc70Cb	Hsc70Cb, isoform G (Hsc70Cb, isoform H)	403.232	2	26.17	448094	26.11	568800	1.64897

TADSPVQISAVR	Hsc70Cb	Hsc70Cb, isoform G (Hsc70Cb, isoform H)	622.336	2	25.12	1436637	25.06	841173	0.76061
AFIVKPIPSPEVIPVIVFINPK	rdgA	Diacylglycerol kinase (DAG kinase) (EC 2.7.1.107)	806.491	3	58.34	7861730	58.25	6537246	1.08019
AFIVKPIPSPEVIPVIVFINPK	rdgA	Diacylglycerol kinase (DAG kinase) (EC 2.7.1.107)	605.12	4	58.3	339552	58.29	268976	1.02903
ALGWGGGYTDEPIGK	rdgA	Diacylglycerol kinase (DAG kinase) (EC 2.7.1.107)	760.873	2	33.66	1454790	33.49	1166978	1.04204
ALHEQGYSLQSVNK	rdgA	Diacylglycerol kinase (DAG kinase) (EC 2.7.1.107)	525.27	3	20.1	844926	20.13	448176	0.68905
FEESISYPK	rdgA	Diacylglycerol kinase (DAG kinase) (EC 2.7.1.107)	550.269	2	25.08	699628	25.06	249796	0.46381
FMHLEK	rdgA	Diacylglycerol kinase (DAG kinase) (EC 2.7.1.107)	410.705	2	11.03	406137	11.1	167691	0.53636
FQHLLNPR	rdgA	Diacylglycerol kinase (DAG kinase) (EC 2.7.1.107)	512.788	2	19.99	2182798	19.93	880908	0.52425
GDVQVNPLEK	rdgA	Diacylglycerol kinase (DAG kinase) (EC 2.7.1.107)	549.793	2	22.75	2434720	22.76	798326	0.42595
GPGSTDEDLQIGSKPIK	rdgA	Diacylglycerol kinase (DAG kinase) (EC 2.7.1.107)	581.304	3	23.2	1491415	23.18	425538	0.37065
LGNYAPIIVPPSWIVK	rdgA	Diacylglycerol kinase (DAG kinase) (EC 2.7.1.107)	884.014	2	52.63	3322956	52.44	1330008	0.51994
NANEIATYLESQER	rdgA	Diacylglycerol kinase (DAG kinase) (EC 2.7.1.107)	819.392	2	35.43	3962934	35.29	2961008	0.97061
NANEIATYLESQER	rdgA	Diacylglycerol kinase (DAG kinase) (EC 2.7.1.107)	546.597	3	35.43	1543644	35.29	934216	0.78618
QVFDLTQGGPK	rdgA	Diacylglycerol kinase (DAG kinase) (EC 2.7.1.107)	595.314	2	28.22	3580063	28.2	2623698	0.95202
SFAAFSQR	rdgA	Diacylglycerol kinase (DAG kinase) (EC 2.7.1.107)	457.23	2	23.28	6655876	23.37	6106585	1.19183
TIPMQVDGEACR	rdgA	Diacylglycerol kinase (DAG kinase) (EC 2.7.1.107)	688.818	2	23.21	1029594	23.29	655361	0.82687
TIPMQVDGEACR	rdgA	Diacylglycerol kinase (DAG kinase) (EC 2.7.1.107)	696.816	2	17.7	628084	17.41	337537	0.69811
VKPSVIEIELLNK	rdgA	Diacylglycerol kinase (DAG kinase) (EC 2.7.1.107)	741.451	2	38.07	1106361	37.96	617198	0.72469
VKPSVIEIELLNK	rdgA	Diacylglycerol kinase (DAG kinase) (EC 2.7.1.107)	494.636	3	38.07	3645006	37.92	2591446	0.92356
VSQDWCFIDSCTAEHYFR	rdgA	Diacylglycerol kinase (DAG kinase) (EC 2.7.1.107)	774.33	3	41.88	500902	41.72	464633	1.20498
VTPNDDVTDDHVDR	rdgA	Diacylglycerol kinase (DAG kinase) (EC 2.7.1.107)	533.241	3	14.64	1483070	14.56	900190	0.78849
YIIASATR	rdgA	Diacylglycerol kinase (DAG kinase) (EC 2.7.1.107)	447.756	2	19.6	1081315	19.55	314198	0.37746
ALDSMQASLEAEAK	Mhc	Myosin heavy chain, isoform S (EC 3.6.1.3)	732.356	2	29.64	321135	29.49	790332	3.19701
ALDSMQASLEAEAK	Mhc	Myosin heavy chain, isoform S (EC 3.6.1.3)	740.353	2	21.04	48359	20.88	260236	6.99057
DLEEANIQHESTLANLR	Mhc	Myosin heavy chain, isoform S (EC 3.6.1.3)	651.661	3	31.08	667254	31	1249882	2.43333
GALQDYQER	Mhc	Myosin heavy chain, isoform S (EC 3.6.1.3)	540.259	2	15.57	503772	15.55	1324414	3.41517
IEELEEEVEAER	Mhc	Myosin heavy chain, isoform S (EC 3.6.1.3)	737.849	2	27.66	871072	27.57	2436877	3.63414
LAEAEETIESLNQK	Mhc	Myosin heavy chain, isoform S (EC 3.6.1.3)	787.899	2	29.52	307761	29.44	1119309	4.72453
LAGADIETYLLEK	Mhc	Myosin heavy chain, isoform S (EC 3.6.1.3)	718.388	2	42.99	649408	42.76	1212276	2.42497
LTQEAVADLER	Mhc	Myosin heavy chain, isoform S (EC 3.6.1.3)	622.828	2	25.2	753282	25.18	1645891	2.83835
NYSTELFR	Mhc	Myosin heavy chain, isoform S (EC 3.6.1.3)	515.254	2	28.61	584482	28.59	1201453	2.67029
QIEEAEEIAALNLAK	Mhc	Myosin heavy chain, isoform S (EC 3.6.1.3)	821.438	2	39.84	244676	39.61	641209	3.40432
QLEEAESQVSQLSK	Mhc	Myosin heavy chain, isoform S (EC 3.6.1.3)	788.397	2	24.66	238416	24.6	763584	4.16048
TALLDSLSGEK	Mhc	Myosin heavy chain, isoform S (EC 3.6.1.3)	567.306	2	31.71	635833	31.55	1551318	3.16942
TLLEQADR	Mhc	Myosin heavy chain, isoform S (EC 3.6.1.3)	473.254	2	18.74	809820	18.73	1724785	2.76674
VIAYFATVGASK	Mhc	Myosin heavy chain, isoform S (EC 3.6.1.3)	613.843	2	33.65	620379	33.48	1435185	3.0052
AELDDNEDVGPR	lincRNA.4	Uncharacterized protein, isoform I	665.299	2	17.49	854844	17.49	358372	0.54459
EQRPSLLTTETQTSR	lincRNA.4	Uncharacterized protein, isoform I	582.971	3	21	714722	20.97	708419	1.28758
FATLPSIR	lincRNA.4	Uncharacterized protein, isoform I	452.766	2	32.1	2652917	31.93	2934962	1.43715
FHDLQDAQSTASSGK	lincRNA.4	Uncharacterized protein, isoform I	531.25	3	15.41	391086	15.36	283768	0.94257
GADSLILDGHIK	lincRNA.4	Uncharacterized protein, isoform I	413.563	3	29.68	1264479	29.53	1469523	1.50969
GFMASAQDR	lincRNA.4	Uncharacterized protein, isoform I	499.722	2	11.62	285875	11.75	165984	0.75424
GISEDNIQLR	lincRNA.4	Uncharacterized protein, isoform I	572.801	2	25.08	2302903	24.99	2379743	1.34238
LAELEVER	lincRNA.4	Uncharacterized protein, isoform I	479.764	2	23.2	1227100	23.26	1368562	1.44879
NYQPDFDSQEFQR	lincRNA.4	Uncharacterized protein, isoform I	837.363	2	28.43	615106	28.34	745801	1.57505
SNLQSGAVINK	lincRNA.4	Uncharacterized protein, isoform I	565.812	2	16.44	787854	16.41	462469	0.76253
STASEPTEAEVELK	lincRNA.4	Uncharacterized protein, isoform I	745.865	2	22.94	667731	22.98	801337	1.55896
SVEAAQEQDEVDGAR	lincRNA.4	Uncharacterized protein, isoform I	802.363	2	15.1	653414	15.03	773644	1.53807
SVSNTFLQPHEDVIVYR	lincRNA.4	Uncharacterized protein, isoform I	668.678	3	35.81	2074033	35.7	1364163	0.85442
TDYEIHTSQVDSSALEELPLQPENR	lincRNA.4	Uncharacterized protein, isoform I	957.793	3	39.43	686104	39.2	452230	0.85623
ALDSMQASLEAEAK	Mhc	Myosin heavy chain, isoform U (EC 3.6.1.3)	732.356	2	29.64	321135	29.49	790332	3.19701
ALDSMQASLEAEAK	Mhc	Myosin heavy chain, isoform U (EC 3.6.1.3)	740.353	2	21.04	48359	20.88	260236	6.99057
DLEEANIQHESTLANLR	Mhc	Myosin heavy chain, isoform U (EC 3.6.1.3)	651.661	3	31.08	667254	31	1249882	2.43333

GALQDYQER	Mhc	Myosin heavy chain, isoform U (EC 3.6.1.3)	540.259	2	15.57	503772	15.55	1324414	3.41517
IEELEEEVEAER	Mhc	Myosin heavy chain, isoform U (EC 3.6.1.3)	737.849	2	27.66	871072	27.57	2436877	3.63414
LAEAEETIESLNQK	Mhc	Myosin heavy chain, isoform U (EC 3.6.1.3)	787.899	2	29.52	307761	29.44	1119309	4.72453
LAGADIETYLLEK	Mhc	Myosin heavy chain, isoform U (EC 3.6.1.3)	718.388	2	42.99	649408	42.76	1212276	2.42497
LTQEAVADLER	Mhc	Myosin heavy chain, isoform U (EC 3.6.1.3)	622.828	2	25.2	753282	25.18	1645891	2.83835
NYSTELFR	Mhc	Myosin heavy chain, isoform U (EC 3.6.1.3)	515.254	2	28.61	584482	28.59	1201453	2.67029
QIEEAEEIAALNLAK	Mhc	Myosin heavy chain, isoform U (EC 3.6.1.3)	821.438	2	39.84	244676	39.61	641209	3.40432
QLEEAESQVSQLSK	Mhc	Myosin heavy chain, isoform U (EC 3.6.1.3)	788.397	2	24.66	238416	24.6	763584	4.16048
TALLDSLSGEK	Mhc	Myosin heavy chain, isoform U (EC 3.6.1.3)	567.306	2	31.71	635833	31.55	1551318	3.16942
TLLEQADR	Mhc	Myosin heavy chain, isoform U (EC 3.6.1.3)	473.254	2	18.74	809820	18.73	1724785	2.76674
VIAYFATVGASK	Mhc	Myosin heavy chain, isoform U (EC 3.6.1.3)	613.843	2	33.65	620379	33.48	1435185	3.0052
AASPASSVSSLSR	Gug	Grunge, isoform F (EC 3.5.1.98)	610.317	2	19.87	670193	19.91	1047675	2.03072
ALQALVK	Gug	Grunge, isoform F (EC 3.5.1.98)	371.745	2	22.24	1429410	22.24	802676	0.72947
ASPSVQISSSGGGPSGPGSVGPG(	Gug	Grunge, isoform F (EC 3.5.1.98)	862.663	4	27.81	128265	27.69	91817	0.9299
MYAPQPGQR	Gug	Grunge, isoform F (EC 3.5.1.98)	532.253	2	11.22	597570	11.23	396368	0.86165
QHQAAAAAAAQQHK	Gug	Grunge, isoform F (EC 3.5.1.98)	477.58	3	7.39	102108	7.46	61942	0.78804
QSLHDQYFR	Gug	Grunge, isoform F (EC 3.5.1.98)	398.528	3	18.86	599467	18.89	746793	1.61829
SQSAIFVR	Gug	Grunge, isoform F (EC 3.5.1.98)	454.253	2	22.57	2827859	22.59	2397174	1.10119
WSPGVVADGDLLMFLR	Gug	Grunge, isoform F (EC 3.5.1.98)	888.461	2	59.21	701351	58.95	433086	0.80216
WSPGVVADGDLLMFLR	Gug	Grunge, isoform F (EC 3.5.1.98)	896.459	2	56.45	1934292	56.29	935150	0.62803
YGDPLAAK	Gug	Grunge, isoform F (EC 3.5.1.98)	417.721	2	19.02	1065996	19	938830	1.14407
TLIDDDNPPVSFVK	wds	Will die slowly, isoform B	780.401	2	38.13	2624767	37.75	1560803	0.77247
AASPASSVSSLSR	Gug	Grunge, isoform G (EC 3.5.1.98)	610.317	2	19.87	670193	19.91	1047675	2.03072
ALQALVK	Gug	Grunge, isoform G (EC 3.5.1.98)	371.745	2	22.24	1429410	22.24	802676	0.72947
ASPSVQISSSGGGPSGPGSVGPG(	Gug	Grunge, isoform G (EC 3.5.1.98)	862.663	4	27.81	128265	27.69	91817	0.9299
MSYADQLQAAEFQR	Gug	Grunge, isoform G (EC 3.5.1.98)	829.386	2	32.37	172984	32.33	256318	1.92484
MYAPQPGQR	Gug	Grunge, isoform G (EC 3.5.1.98)	532.253	2	11.22	597570	11.23	396368	0.86165
QHQAAAAAAAQQHK	Gug	Grunge, isoform G (EC 3.5.1.98)	477.58	3	7.39	102108	7.46	61942	0.78804
QSLHDQYFR	Gug	Grunge, isoform G (EC 3.5.1.98)	398.528	3	18.86	599467	18.89	746793	1.61829
SQSAIFVR	Gug	Grunge, isoform G (EC 3.5.1.98)	454.253	2	22.57	2827859	22.59	2397174	1.10119
WSPGVVADGDLLMFLR	Gug	Grunge, isoform G (EC 3.5.1.98)	888.461	2	59.21	701351	58.95	433086	0.80216
WSPGVVADGDLLMFLR	Gug	Grunge, isoform G (EC 3.5.1.98)	896.459	2	56.45	1934292	56.29	935150	0.62803
YGDPLAAK	Gug	Grunge, isoform G (EC 3.5.1.98)	417.721	2	19.02	1065996	19	938830	1.14407
AELDDNEDVGPR	CG6239	Uncharacterized protein, isoform G	665.299	2	17.49	854844	17.49	358372	0.54459
EQRPSLLTTETQTSR	CG6239	Uncharacterized protein, isoform G	582.971	3	21	714722	20.97	708419	1.28758
FATLPSIR	CG6239	Uncharacterized protein, isoform G	452.766	2	32.1	2652917	31.93	2934962	1.43715
FHDLQDAQSTASSGK	CG6239	Uncharacterized protein, isoform G	531.25	3	15.41	391086	15.36	283768	0.94257
GADSLILDGHIK	CG6239	Uncharacterized protein, isoform G	413.563	3	29.68	1264479	29.53	1469523	1.50969
GFMASAQDR	CG6239	Uncharacterized protein, isoform G	499.722	2	11.62	285875	11.75	165984	0.75424
GISEDNIQLR	CG6239	Uncharacterized protein, isoform G	572.801	2	25.08	2302903	24.99	2379743	1.34238
LAELEVER	CG6239	Uncharacterized protein, isoform G	479.764	2	23.2	1227100	23.26	1368562	1.44879
NYQPDFDSQEFQR	CG6239	Uncharacterized protein, isoform G	837.363	2	28.43	615106	28.34	745801	1.57505
SNLQSGAVINK	CG6239	Uncharacterized protein, isoform G	565.812	2	16.44	787854	16.41	462469	0.76253
STASEPTEAEVELK	CG6239	Uncharacterized protein, isoform G	745.865	2	22.94	667731	22.98	801337	1.55896
SVEAAQEQDEVDGAR	CG6239	Uncharacterized protein, isoform G	802.363	2	15.1	653414	15.03	773644	1.53807
SVSNTFLQPHEDVIVYR	CG6239	Uncharacterized protein, isoform G	668.678	3	35.81	2074033	35.7	1364163	0.85442
TDYEIHTSQVDSSALEELPLQPENR	CG6239	Uncharacterized protein, isoform G	957.793	3	39.43	686104	39.2	452230	0.85623
DGFQVCMDVSHFKPSELVVK	Hsp23	Heat shock protein 23, isoform B	774.713	3	43.1	204592	43.01	339238	2.15396
DGFQVCMDVSHFKPSELVVK	Hsp23	Heat shock protein 23, isoform B	581.287	4	43.1	402779	42.97	317497	1.02399
DGFQVCMDVSHFKPSELVVK	Hsp23	Heat shock protein 23, isoform B	780.045	3	38.76	29062	38.74	599164	26.782
DGFQVCMDVSHFKPSELVVK	Hsp23	Heat shock protein 23, isoform B	585.286	4	38.73	18808	38.78	469777	32.4468
IVQIQQVGPAHLNVK	Hsp23	Heat shock protein 23, isoform B	822.483	2	30.45	27968	30.12	3476212	161.461
IVQIQQVGPAHLNVK	Hsp23	Heat shock protein 23, isoform B	548.658	3	30.33	1113	30.08	27440434	32027.1

MSMVPFYEPYYCQR	Hsp23	Heat shock protein 23, isoform B	935.902	2	43.25	0	43.16	737059	#DIV/0!
MSMVPFYEPYYCQR	Hsp23	Heat shock protein 23, isoform B	943.899	2	39.58	18216	39.45	774858	55.2575
MSMVPFYEPYYCQR	Hsp23	Heat shock protein 23, isoform B	943.899	2	37.24	37268	37.1	726973	25.3399
MSMVPFYEPYYCQR	Hsp23	Heat shock protein 23, isoform B	951.897	2	34.82	0	34.71	818198	#DIV/0!
NPYLALVGPMEQQLR	Hsp23	Heat shock protein 23, isoform B	864.959	2	46.34	474657	45.99	3156756	8.6394
NPYLALVGPMEQQLR	Hsp23	Heat shock protein 23, isoform B	576.975	3	46.1	37103	45.99	888801	31.1184
NPYLALVGPMEQQLR	Hsp23	Heat shock protein 23, isoform B	872.956	2	38.21	28503	37.96	3708824	169.032
NPYLALVGPMEQQLR	Hsp23	Heat shock protein 23, isoform B	582.307	3	38.46	368115	37.96	1423477	5.0233
QVGASSGSSGAVSK	Hsp23	Heat shock protein 23, isoform B	611.307	2	13.12	130999	8.97	467450	4.63542
VASTLSSDGVLTIK	Hsp23	Heat shock protein 23, isoform B	695.893	2	33.08	0	33.09	2054661	#DIV/0!
VQDNSVLVEGNHEER	Hsp23	Heat shock protein 23, isoform B	575.611	3	16.47	0	16.8	50272	#DIV/0!
IVSQHTSSSR	Lam	Lamin, isoform B (Lamin, isoform C) (Lamin, isoform D)	551.286	2	7	45019	6.94	21557	0.62203
LATYIDR	Lam	Lamin, isoform B (Lamin, isoform C) (Lamin, isoform D)	426.235	2	19.41	565173	19.45	354186	0.81409
LNITPATNTATVQSFSQSLR	Lam	Lamin, isoform B (Lamin, isoform C) (Lamin, isoform D)	717.046	3	39.95	713399	39.69	972294	1.77047
TILLNSEGEAVANLDR	Lam	Lamin, isoform B (Lamin, isoform C) (Lamin, isoform D)	857.952	2	36.67	1134982	36.48	1426663	1.63288
ALDSMQASLEAEAK	Mhc	Myosin heavy chain, isoform T (EC 3.6.1.3)	732.356	2	29.64	321135	29.49	790332	3.19701
ALDSMQASLEAEAK	Mhc	Myosin heavy chain, isoform T (EC 3.6.1.3)	740.353	2	21.04	48359	20.88	260236	6.99057
DLEEANIQHESTLANLR	Mhc	Myosin heavy chain, isoform T (EC 3.6.1.3)	651.661	3	31.08	667254	31	1249882	2.43333
GALQDYQER	Mhc	Myosin heavy chain, isoform T (EC 3.6.1.3)	540.259	2	15.57	503772	15.55	1324414	3.41517
IEELEEEVEAER	Mhc	Myosin heavy chain, isoform T (EC 3.6.1.3)	737.849	2	27.66	871072	27.57	2436877	3.63414
LAEAEETIESLNQK	Mhc	Myosin heavy chain, isoform T (EC 3.6.1.3)	787.899	2	29.52	307761	29.44	1119309	4.72453
LAGADIETYLLEK	Mhc	Myosin heavy chain, isoform T (EC 3.6.1.3)	718.388	2	42.99	649408	42.76	1212276	2.42497
LTQEAVADLER	Mhc	Myosin heavy chain, isoform T (EC 3.6.1.3)	622.828	2	25.2	753282	25.18	1645891	2.83835
NYSTELFR	Mhc	Myosin heavy chain, isoform T (EC 3.6.1.3)	515.254	2	28.61	584482	28.59	1201453	2.67029
QIEEAEEIAALNLAK	Mhc	Myosin heavy chain, isoform T (EC 3.6.1.3)	821.438	2	39.84	244676	39.61	641209	3.40432
QLEEAESQVSQLSK	Mhc	Myosin heavy chain, isoform T (EC 3.6.1.3)	788.397	2	24.66	238416	24.6	763584	4.16048
TALLDSLSGEK	Mhc	Myosin heavy chain, isoform T (EC 3.6.1.3)	567.306	2	31.71	635833	31.55	1551318	3.16942
TLLEQADR	Mhc	Myosin heavy chain, isoform T (EC 3.6.1.3)	473.254	2	18.74	809820	18.73	1724785	2.76674
VIAYFATVGASK	Mhc	Myosin heavy chain, isoform T (EC 3.6.1.3)	613.843	2	33.65	620379	33.48	1435185	3.0052
GDVGPGAGEVEFR	RpS10b	Ribosomal protein S10b, isoform D (Ribosomal protein S10b, isoform E)	645.31	2	25.28	985914	25.27	757708	0.99836
SETVRPRPAVGGPR	RpS10b	Ribosomal protein S10b, isoform D (Ribosomal protein S10b, isoform E)	493.611	3	12.12	3082161	12.12	2287000	0.9639
VAIYEYLFK	RpS10b	Ribosomal protein S10b, isoform D (Ribosomal protein S10b, isoform E)	573.316	2	46.67	2373270	46.49	2589560	1.41743
AGIAVEGDIK	Arr1	Arrestin 1, isoform B	486.772	2	22.98	2931108	22.98	1880959	0.83362
ASDESQPCGVQYFVK	Arr1	Arrestin 1, isoform B	857.891	2	30.22	589087	30.08	894673	1.97291
DFLLSPGELELEVTLDK	Arr1	Arrestin 1, isoform B	959.506	2	56.3	265898	55.99	462292	2.25852
DFVDSVTQVEPIDGIIVLDDEYVR	Arr1	Arrestin 1, isoform B	1368.68	2	59.17	646283	58.87	419252	0.8427
DFVDSVTQVEPIDGIIVLDDEYVR	Arr1	Arrestin 1, isoform B	912.792	3	59.17	2018632	58.83	1330820	0.85642
IFTGDSDCDR	Arr1	Arrestin 1, isoform B	593.246	2	14.94	1443871	14.91	748462	0.67339
IFVQLVCNFR	Arr1	Arrestin 1, isoform B	648.35	2	42.99	1459871	42.8	1343715	1.19568
ISVNICVR	Arr1	Arrestin 1, isoform B	480.768	2	29.06	677107	28.92	402608	0.77241
QGIQPCTVVR	Arr1	Arrestin 1, isoform B	579.308	2	21.75	818570	21.73	482335	0.76545
STINLGIR	Arr1	Arrestin 1, isoform B	437.261	2	27.76	3970398	27.69	3553533	1.16265
VMYLVPTLVANCDR	Arr1	Arrestin 1, isoform B	825.921	2	44.34	873153	44.19	632809	0.94147
ALDSMQASLEAEAK	Mhc	Myosin heavy chain, isoform R (EC 3.6.1.3)	732.356	2	29.64	321135	29.49	790332	3.19701
ALDSMQASLEAEAK	Mhc	Myosin heavy chain, isoform R (EC 3.6.1.3)	740.353	2	21.04	48359	20.88	260236	6.99057
DLEEANIQHESTLANLR	Mhc	Myosin heavy chain, isoform R (EC 3.6.1.3)	651.661	3	31.08	667254	31	1249882	2.43333
GALQDYQER	Mhc	Myosin heavy chain, isoform R (EC 3.6.1.3)	540.259	2	15.57	503772	15.55	1324414	3.41517
IEELEEEVEAER	Mhc	Myosin heavy chain, isoform R (EC 3.6.1.3)	737.849	2	27.66	871072	27.57	2436877	3.63414
LAEAEETIESLNQK	Mhc	Myosin heavy chain, isoform R (EC 3.6.1.3)	787.899	2	29.52	307761	29.44	1119309	4.72453
LAGADIETYLLEK	Mhc	Myosin heavy chain, isoform R (EC 3.6.1.3)	718.388	2	42.99	649408	42.76	1212276	2.42497
LTQEAVADLER	Mhc	Myosin heavy chain, isoform R (EC 3.6.1.3)	622.828	2	25.2	753282	25.18	1645891	2.83835
NYSTELFR	Mhc	Myosin heavy chain, isoform R (EC 3.6.1.3)	515.254	2	28.61	584482	28.59	1201453	2.67029
QIEEAEEIAALNLAK	Mhc	Myosin heavy chain, isoform R (EC 3.6.1.3)	821.438	2	39.84	244676	39.61	641209	3.40432

QLEEAESQVSQLSK	Mhc	Myosin heavy chain, isoform R (EC 3.6.1.3)	788.397	2	24.66	238416	24.6	763584	4.16048
TALLDSLSGEK	Mhc	Myosin heavy chain, isoform R (EC 3.6.1.3)	567.306	2	31.71	635833	31.55	1551318	3.16942
TLLEQADR	Mhc	Myosin heavy chain, isoform R (EC 3.6.1.3)	473.254	2	18.74	809820	18.73	1724785	2.76674
VIAYFATVGASK	Mhc	Myosin heavy chain, isoform R (EC 3.6.1.3)	613.843	2	33.65	620379	33.48	1435185	3.0052
AELDDNEDVGPR	CG32355	Uncharacterized protein, isoform J (Uncharacterized protein, isoform N)	665.299	2	17.49	854844	17.49	358372	0.54459
AGTNDTDPVIEVEPAGVR	CG32355	Uncharacterized protein, isoform J (Uncharacterized protein, isoform N)	920.458	2	31.26	799045	31.12	994850	1.61737
ELTPFEQELQR	CG32355	Uncharacterized protein, isoform J (Uncharacterized protein, isoform N)	695.354	2	34.45	1822534	34.23	951564	0.67824
EQRPSLLTTETQTSR	CG32355	Uncharacterized protein, isoform J (Uncharacterized protein, isoform N)	582.971	3	21	714722	20.97	708419	1.28758
FATLPSIR	CG32355	Uncharacterized protein, isoform J (Uncharacterized protein, isoform N)	452.766	2	32.1	2652917	31.93	2934962	1.43715
FHDLQDAQSTASSGK	CG32355	Uncharacterized protein, isoform J (Uncharacterized protein, isoform N)	531.25	3	15.41	391086	15.36	283768	0.94257
GADSLILDGHIK	CG32355	Uncharacterized protein, isoform J (Uncharacterized protein, isoform N)	413.563	3	29.68	1264479	29.53	1469523	1.50969
GFMASAQDR	CG32355	Uncharacterized protein, isoform J (Uncharacterized protein, isoform N)	499.722	2	11.62	285875	11.75	165984	0.75424
GISEDNIQLR	CG32355	Uncharacterized protein, isoform J (Uncharacterized protein, isoform N)	572.801	2	25.08	2302903	24.99	2379743	1.34238
LAELEVER	CG32355	Uncharacterized protein, isoform J (Uncharacterized protein, isoform N)	479.764	2	23.2	1227100	23.26	1368562	1.44879
NYQPDFDSQEFQR	CG32355	Uncharacterized protein, isoform J (Uncharacterized protein, isoform N)	837.363	2	28.43	615106	28.34	745801	1.57505
RPLGPDEQPEIVLLLR	CG32355	Uncharacterized protein, isoform J (Uncharacterized protein, isoform N)	615.691	3	45.58	1022269	45.43	642182	0.81605
SNLQSGAVINK	CG32355	Uncharacterized protein, isoform J (Uncharacterized protein, isoform N)	565.812	2	16.44	787854	16.41	462469	0.76253
STASEPTEAEVELK	CG32355	Uncharacterized protein, isoform J (Uncharacterized protein, isoform N)	745.865	2	22.94	667731	22.98	801337	1.55896
SVEAAQEQDEVDGAR	CG32355	Uncharacterized protein, isoform J (Uncharacterized protein, isoform N)	802.363	2	15.1	653414	15.03	773644	1.53807
SVSNTFLQPHEDVIVYR	CG32355	Uncharacterized protein, isoform J (Uncharacterized protein, isoform N)	668.678	3	35.81	2074033	35.7	1364163	0.85442
TDYEIHTSQVDSSALEELPLQPENR	CG32355	Uncharacterized protein, isoform J (Uncharacterized protein, isoform N)	957.793	3	39.43	686104	39.2	452230	0.85623
THEDAQSLPGNSANPSR	CG32355	Uncharacterized protein, isoform J (Uncharacterized protein, isoform N)	594.278	3	13.23	675781	13.53	14072906	27.052
AAEFNFR	wupA	Wings up A, isoform I	427.711	2	24.96	9042040	24.95	9905750	1.42313
AQEINDLNAQVNDLR	wupA	Wings up A, isoform I	856.932	2	30.64	781036	30.48	521347	0.86712
GFMTPER	wupA	Wings up A, isoform I	419.2	2	19.67	1284815	19.67	1246317	1.26012
NLSDASEAELQTICK	wupA	Wings up A, isoform I	839.901	2	30.84	2095930	30.72	1850935	1.14719
IGGVTLEILAK	GS	Uncharacterized protein, isoform F (EC 1.-.-.-) (EC 1.4.1.14) (Uncharacterizec	557.348	2	40.98	21782	40.9	251762	15.0146
LVSEVGVGVVASGVAK	GS	Uncharacterized protein, isoform F (EC 1.-.-.-) (EC 1.4.1.14) (Uncharacterizec	735.93	2	33.15	0	33.02	197421	#DIV/0!
AELDDNEDVGPR	CG17926	Uncharacterized protein, isoform H	665.299	2	17.49	854844	17.49	358372	0.54459
AGTNDTDPVIEVEPAGVR	CG17926	Uncharacterized protein, isoform H	920.458	2	31.26	799045	31.12	994850	1.61737
ELTPFEQELQR	CG17926	Uncharacterized protein, isoform H	695.354	2	34.45	1822534	34.23	951564	0.67824
EQRPSLLTTETQTSR	CG17926	Uncharacterized protein, isoform H	582.971	3	21	714722	20.97	708419	1.28758
FATLPSIR	CG17926	Uncharacterized protein, isoform H	452.766	2	32.1	2652917	31.93	2934962	1.43715
FHDLQDAQSTASSGK	CG17926	Uncharacterized protein, isoform H	531.25	3	15.41	391086	15.36	283768	0.94257
GADSLILDGHIK	CG17926	Uncharacterized protein, isoform H	413.563	3	29.68	1264479	29.53	1469523	1.50969
GFMASAQDR	CG17926	Uncharacterized protein, isoform H	499.722	2	11.62	285875	11.75	165984	0.75424
GISEDNIQLR	CG17926	Uncharacterized protein, isoform H	572.801	2	25.08	2302903	24.99	2379743	1.34238
LAELEVER	CG17926	Uncharacterized protein, isoform H	479.764	2	23.2	1227100	23.26	1368562	1.44879
NYQPDFDSQEFQR	CG17926	Uncharacterized protein, isoform H	837.363	2	28.43	615106	28.34	745801	1.57505
RPLGPDEQPEIVLLLR	CG17926	Uncharacterized protein, isoform H	615.691	3	45.58	1022269	45.43	642182	0.81605
SNLQSGAVINK	CG17926	Uncharacterized protein, isoform H	565.812	2	16.44	787854	16.41	462469	0.76253
STASEPTEAEVELK	CG17926	Uncharacterized protein, isoform H	745.865	2	22.94	667731	22.98	801337	1.55896
SVEAAQEQDEVDGAR	CG17926	Uncharacterized protein, isoform H	802.363	2	15.1	653414	15.03	773644	1.53807
SVSNTFLQPHEDVIVYR	CG17926	Uncharacterized protein, isoform H	668.678	3	35.81	2074033	35.7	1364163	0.85442
TDYEIHTSQVDSSALEELPLQPENR	CG17926	Uncharacterized protein, isoform H	957.793	3	39.43	686104	39.2	452230	0.85623
THEDAQSLPGNSANPSR	CG17926	Uncharacterized protein, isoform H	594.278	3	13.23	675781	13.53	14072906	27.052
AAAPAAVASPAAAATSADASPSPAI	Pep	Protein on ecdysone puffs, isoform D	708.031	3	24.27	491341	24.2	1065912	2.81813
FFDTEVTAEIHSR	Pep	Protein on ecdysone puffs, isoform D	517.919	3	32.02	935298	31.86	1657140	2.30161
NQNPPSLLDLPR	Pep	Protein on ecdysone puffs, isoform D	682.37	2	37.75	928013	37.52	1703500	2.38457
AQELWELIVK	up	Upheld, isoform N	614.85	2	48.34	857786	48.15	594529	0.90036
DAGLGLSSAAMER	up	Upheld, isoform N	647.309	2	23.24	1496095	23.26	1558348	1.35309
IKPLAIEGFGEAK	up	Upheld, isoform N	458.266	3	33.5	4523125	33.37	3961091	1.13762
ISLSFR	up	Upheld, isoform N	361.713	2	30.22	1860854	30.16	1791066	1.25032

AAEFNFR	wupA	Wings up A, isoform J	427.711	2	24.96	9042040	24.95	9905750	1.42313
GFMTPER	wupA	Wings up A, isoform J	419.2	2	19.67	1284815	19.67	1246317	1.26012
NLSDASEDTLK	wupA	Wings up A, isoform J	596.788	2	18.67	925293	18.66	786155	1.1037
DSSVLPVAEQPPAK	CoRest	CoRest, isoform J	719.383	2	26.69	3357320	26.66	3402373	1.31647
IALSTGGGSSVAEFLAN	CoRest	CoRest, isoform J	797.41	2	44.77	1725679	44.51	2728085	2.05362
TQTSSEELSAQK	CoRest	CoRest, isoform J	654.818	2	11.1	1476303	11.23	860048	0.75678
DGVLTVDAPLPALTAGETLIPIAHK	dHspB8	Uncharacterized protein, isoform C	838.139	3	55.9	27215	55.42	322725	15.4045
GVNPESIR	dHspB8	Uncharacterized protein, isoform C	436.235	2	16.4	162532	16.45	263810	2.10851
LALVGGIFNLAAVLR	FASN2	Fatty acid synthase 2, isoform B (EC 1.-.-.-) (EC 2.3.1.85) (EC 3.1.-.-)	763.974	2	64.23	79694	63.78	0	0
GSTGTLGNFAK	RpS2	Ribosomal protein S2, isoform B (Ribosomal protein S2, isoform C)	526.772	2	21.67	1400654	21.69	1849774	1.71558
GTGIVSAPVPK	RpS2	Ribosomal protein S2, isoform B (Ribosomal protein S2, isoform C)	513.303	2	23.48	3393633	23.45	3953923	1.51351
TYAYLTPDLWK	RpS2	Ribosomal protein S2, isoform B (Ribosomal protein S2, isoform C)	685.853	2	43.29	689942	43.09	916755	1.72609
ALLFIPR	Hsp83	Heat shock protein 83, isoform B	415.268	2	39.76	1322147	39.6	1321862	1.29876
FHTSASGDDFCSLADYVSR	Hsp83	Heat shock protein 83, isoform B	712.309	3	37.38	903559	37.17	836957	1.20329
HFSVEGQLEFR	Hsp83	Heat shock protein 83, isoform B	674.836	2	31.64	477696	31.52	426943	1.16102
HFSVEGQLEFR	Hsp83	Heat shock protein 83, isoform B	450.226	3	31.72	303941	31.52	380731	1.62724
STSISNVAGASLTR	ens	Ensconsin, isoform J	682.362	2	25.86	816429	25.79	694557	1.10513
DSSLSSGALANILAIFK	Hn	Henna, isoform C (EC 1.14.16.1)	853.97	2	65.24	302707	65.18	21367	0.09169
FANSIPRPFGVR	Hn	Henna, isoform C (EC 1.14.16.1)	454.254	3	31.64	779696	31.6	818437	1.36359
AELDDNEDVGPR	CG17926	Uncharacterized protein, isoform M	665.299	2	17.49	854844	17.49	358372	0.54459
AGTNDTDPVIEVEPAGVR	CG17926	Uncharacterized protein, isoform M	920.458	2	31.26	799045	31.12	994850	1.61737
ELTPFEQELQR	CG17926	Uncharacterized protein, isoform M	695.354	2	34.45	1822534	34.23	951564	0.67824
EQRPSLLTTETQTSR	CG17926	Uncharacterized protein, isoform M	582.971	3	21	714722	20.97	708419	1.28758
FHDLQDAQSTASSGK	CG17926	Uncharacterized protein, isoform M	531.25	3	15.41	391086	15.36	283768	0.94257
GADSLILDGHIK	CG17926	Uncharacterized protein, isoform M	413.563	3	29.68	1264479	29.53	1469523	1.50969
GFMASAQDR	CG17926	Uncharacterized protein, isoform M	499.722	2	11.62	285875	11.75	165984	0.75424
GISEDNIQLR	CG17926	Uncharacterized protein, isoform M	572.801	2	25.08	2302903	24.99	2379743	1.34238
LAELEVER	CG17926	Uncharacterized protein, isoform M	479.764	2	23.2	1227100	23.26	1368562	1.44879
NYQPDFDSQEFQR	CG17926	Uncharacterized protein, isoform M	837.363	2	28.43	615106	28.34	745801	1.57505
RPLGPDEQPEIVLLLR	CG17926	Uncharacterized protein, isoform M	615.691	3	45.58	1022269	45.43	642182	0.81605
SNLQSGAVINK	CG17926	Uncharacterized protein, isoform M	565.812	2	16.44	787854	16.41	462469	0.76253
STASEPTEAEVELK	CG17926	Uncharacterized protein, isoform M	745.865	2	22.94	667731	22.98	801337	1.55896
SVEAAQEQDEVDGAR	CG17926	Uncharacterized protein, isoform M	802.363	2	15.1	653414	15.03	773644	1.53807
SVSNTFLQPHEDVIVYR	CG17926	Uncharacterized protein, isoform M	668.678	3	35.81	2074033	35.7	1364163	0.85442
TDYEIHTSQVDSSALEELPLQPENR	CG17926	Uncharacterized protein, isoform M	957.793	3	39.43	686104	39.2	452230	0.85623
THEDAQSLPGNSANPSR	CG17926	Uncharacterized protein, isoform M	594.278	3	13.23	675781	13.53	14072906	27.052
LAMQVVLHTELER	TrpA1	Transient receptor potential cation channel A1, isoform K	518.948	3	27.31	103016	27.24	1044228	13.1678
MLEQQHHLVR	TrpA1	Transient receptor potential cation channel A1, isoform K	430.896	3	13.31	104134	13.27	657559	8.20284
NVIDIQQGGEHGR	TrpA1	Transient receptor potential cation channel A1, isoform K	474.908	3	18.12	345514	18.16	3305973	12.4296
SPLLLAASR	TrpA1	Transient receptor potential cation channel A1, isoform K	464.285	2	29.1	430144	29.02	1188200	3.58838
VSVNDFIIK	muc	Dihydrolipoamide acetyltransferase component of pyruvate dehydrogenase co	517.798	2	38.03	442234	37.88	706401	2.07502
VVDGAVAAR	muc	Dihydrolipoamide acetyltransferase component of pyruvate dehydrogenase co	429.246	2	12.17	236652	12.23	154993	0.85079
YEDIPVTNMR	muc	Dihydrolipoamide acetyltransferase component of pyruvate dehydrogenase co	627.295	2	20.92	490136	20.93	697704	1.84917
GGGSGTDFYNQQR	tral	Trailer hitch, isoform G	693.805	2	16.19	310314	16.14	351936	1.47328
DVLSVAFSADNR	Rack1	Receptor of activated protein kinase C 1, isoform C (Receptor of activated pro	647.325	2	37.28	534801	37.1	522154	1.26832
AIGAGGQSTQVR	Sap130	Sin3A-associated protein 130, isoform G	572.807	2	12.25	925618	12.32	536264	0.75261
GPGAGGTALTATNLPTTR	Sap130	Sin3A-associated protein 130, isoform G	828.439	2	27.36	569694	27.23	266622	0.60796
IGDQPATGGAGGR	Sap130	Sin3A-associated protein 130, isoform G	578.789	2	10.2	451645	10.33	79684	0.22919
IIQLQQPATGTTQQIIGSGAR	Sap130	Sin3A-associated protein 130, isoform G	727.738	3	34.41	1767358	34.26	2314474	1.70118
QLENVTATGR	Sap130	Sin3A-associated protein 130, isoform G	544.788	2	15.37	399235	15.31	452872	1.47356
SDGGGGISIAGTPILSGGTIK	Sap130	Sin3A-associated protein 130, isoform G	929.499	2	41.44	945778	41.22	943168	1.29545
TQITAIPAAPAR	Sap130	Sin3A-associated protein 130, isoform G	605.351	2	25.4	3213530	25.35	3375310	1.36444
VQPSLTITQLNPIGK	Sap130	Sin3A-associated protein 130, isoform G	804.97	2	41.17	1625853	40.98	1635254	1.30655

VTHGVASSAASVPATVGPVNSTR	Sap130	Sin3A-associated protein 130, isoform G	722.382	3	23.81	505024	23.8	708422	1.82223
VTTGAGNPQQATASGALVSSFMR	Sap130	Sin3A-associated protein 130, isoform G	756.374	3	32.37	491161	32.25	670045	1.77216
VSVNDFIIK	muc	Dihydrolipoamide acetyltransferase component of pyruvate dehydrogenase co	517.798	2	38.03	442234	37.88	706401	2.07502
VVDGAVAAR	muc	Dihydrolipoamide acetyltransferase component of pyruvate dehydrogenase co	429.246	2	12.17	236652	12.23	154993	0.85079
YEDIPVTNMR	muc	Dihydrolipoamide acetyltransferase component of pyruvate dehydrogenase co	627.295	2	20.92	490136	20.93	697704	1.84917
TALTHYLEITAIPR	Cpr	NADPH--cytochrome P450 reductase (CPR) (P450R) (EC 1.6.2.4)	533.635	3	40.81	57301	40.62	150767	3.41796
AATQEIAANVLR	Dlg5	Discs large 5, isoform C	628.852	2	39.24	4025067	39.04	5041733	1.62716
AAIELNR	ninaC	FI21215p1 (Neither inactivation nor afterpotential C, isoform C) (Neither inacti	393.727	2	17.87	27934492	17.87	8147882	0.3789
ADLEYKPR	ninaC	FI21215p1 (Neither inactivation nor afterpotential C, isoform C) (Neither inacti	496.264	2	14.09	8517788	14.09	3261920	0.49747
ADVWALGITTIELADGKPPFADMHP	ninaC	FI21215p1 (Neither inactivation nor afterpotential C, isoform C) (Neither inacti	974.832	3	55.59	2262995	55.61	1325024	0.76061
ADVWALGITTIELADGKPPFADMHP	ninaC	FI21215p1 (Neither inactivation nor afterpotential C, isoform C) (Neither inacti	731.376	4	55.55	5864969	55.61	3224204	0.71413
ADVWALGITTIELADGKPPFADMHP	ninaC	FI21215p1 (Neither inactivation nor afterpotential C, isoform C) (Neither inacti	980.164	3	53.25	2367700	53.26	1572627	0.86282
ADVWALGITTIELADGKPPFADMHP	ninaC	FI21215p1 (Neither inactivation nor afterpotential C, isoform C) (Neither inacti	735.375	4	53.22	7293140	53.22	4530989	0.80705
AFTDINR	ninaC	FI21215p1 (Neither inactivation nor afterpotential C, isoform C) (Neither inacti	418.717	2	17.64	9521909	17.64	2491073	0.33985
AILMLVNAGTPVNNDSTR	ninaC	FI21215p1 (Neither inactivation nor afterpotential C, isoform C) (Neither inacti	943.494	2	39.58	4360804	39.35	1237694	0.3687
AILMLVNAGTPVNNDSTR	ninaC	FI21215p1 (Neither inactivation nor afterpotential C, isoform C) (Neither inacti	629.332	3	39.61	1175945	39.7	524491	0.57939
ALGVLDTVIAR	ninaC	FI21215p1 (Neither inactivation nor afterpotential C, isoform C) (Neither inacti	564.343	2	40.21	85712096	40.04	54023652	0.81877
AMFQIIR	ninaC	FI21215p1 (Neither inactivation nor afterpotential C, isoform C) (Neither inacti	439.749	2	35.65	17816182	35.48	11806378	0.86084
AVFGDTNAIIIHDMFGFECFNR	ninaC	FI21215p1 (Neither inactivation nor afterpotential C, isoform C) (Neither inacti	858.735	3	55.9	3358748	55.78	1202619	0.46513
CVLQYCLTFGK	ninaC	FI21215p1 (Neither inactivation nor afterpotential C, isoform C) (Neither inacti	694.839	2	38.91	1761165	38.62	991756	0.73152
DAVASTLYSR	ninaC	FI21215p1 (Neither inactivation nor afterpotential C, isoform C) (Neither inacti	541.777	2	22.04	13835789	22.06	3379994	0.31735
DDPEGNVER	ninaC	FI21215p1 (Neither inactivation nor afterpotential C, isoform C) (Neither inacti	515.725	2	9.74	412063	9.86	43490	0.1371
DYCDHPNLPEFYGVYK	ninaC	FI21215p1 (Neither inactivation nor afterpotential C, isoform C) (Neither inacti	1008.94	2	37.72	913930	37.56	316723	0.45018
DYCDHPNLPEFYGVYK	ninaC	FI21215p1 (Neither inactivation nor afterpotential C, isoform C) (Neither inacti	672.965	3	37.72	4680463	37.56	1886732	0.52365
FTCLTLLK	ninaC	FI21215p1 (Neither inactivation nor afterpotential C, isoform C) (Neither inacti	498.283	2	37.02	3865875	36.79	785519	0.26396
GDNILLTK	ninaC	FI21215p1 (Neither inactivation nor afterpotential C, isoform C) (Neither inacti	437.256	2	23.96	7840105	24.03	3269877	0.54179
HLCYLGDGNR	ninaC	FI21215p1 (Neither inactivation nor afterpotential C, isoform C) (Neither inacti	602.78	2	17.48	8493956	17.49	1859001	0.28431
HSQFVK	ninaC	FI21215p1 (Neither inactivation nor afterpotential C, isoform C) (Neither inacti	373.203	2	9.26	4129579	9.38	1484137	0.46686
HTATEISVAHYTGR	ninaC	FI21215p1 (Neither inactivation nor afterpotential C, isoform C) (Neither inacti	771.887	2	18.64	1826203	18.65	976342	0.6945
HTATEISVAHYTGR	ninaC	FI21215p1 (Neither inactivation nor afterpotential C, isoform C) (Neither inacti	514.927	3	18.67	34603876	18.61	24736648	0.92862
INMNMSFPR	ninaC	FI21215p1 (Neither inactivation nor afterpotential C, isoform C) (Neither inacti	555.265	2	33.61	1257006	33.45	473494	0.48933
INMNMSFPR	ninaC	FI21215p1 (Neither inactivation nor afterpotential C, isoform C) (Neither inacti	563.263	2	24.81	6223789	24.72	1413809	0.29509
INMNMSFPR	ninaC	FI21215p1 (Neither inactivation nor afterpotential C, isoform C) (Neither inacti	571.26	2	17.71	1092556	17.64	317990	0.37809
IQHYDEEHQVSIEEEYR	ninaC	FI21215p1 (Neither inactivation nor afterpotential C, isoform C) (Neither inacti	735.335	3	21.82	9144805	21.85	3841429	0.54568
IQHYDEEHQVSIEEEYR	ninaC	FI21215p1 (Neither inactivation nor afterpotential C, isoform C) (Neither inacti	551.753	4	21.82	11296823	21.81	5372007	0.61774
LPFDEFLR	ninaC	FI21215p1 (Neither inactivation nor afterpotential C, isoform C) (Neither inacti	518.777	2	45.35	37304816	45.13	12833087	0.44688
LYELQVK	ninaC	FI21215p1 (Neither inactivation nor afterpotential C, isoform C) (Neither inacti	446.761	2	26.36	7134839	26.31	1279726	0.233
MDEALAAVR	ninaC	FI21215p1 (Neither inactivation nor afterpotential C, isoform C) (Neither inacti	496.247	2	19.06	919808	19.09	435944	0.61568
MLSQNANLGVHFVR	ninaC	FI21215p1 (Neither inactivation nor afterpotential C, isoform C) (Neither inacti	529.28	3	31.04	3025753	30.96	721657	0.30983
MLSQNANLGVHFVR	ninaC	FI21215p1 (Neither inactivation nor afterpotential C, isoform C) (Neither inacti	534.612	3	28.76	906685	28.64	111527	0.15979
MYPEDLAALENPVDENIIESLR	ninaC	FI21215p1 (Neither inactivation nor afterpotential C, isoform C) (Neither inacti	1266.12	2	55.67	1021590	55.37	543268	0.69081
MYPEDLAALENPVDENIIESLR	ninaC	FI21215p1 (Neither inactivation nor afterpotential C, isoform C) (Neither inacti	844.416	3	55.74	3400744	55.49	1974218	0.75413
MYPEDLAALENPVDENIIESLR	ninaC	FI21215p1 (Neither inactivation nor afterpotential C, isoform C) (Neither inacti	1274.12	2	53.98	1115935	53.78	538899	0.62732
MYPEDLAALENPVDENIIESLR	ninaC	FI21215p1 (Neither inactivation nor afterpotential C, isoform C) (Neither inacti	849.748	3	54.05	4350341	53.78	2829471	0.8449
NHVLHR	ninaC	FI21215p1 (Neither inactivation nor afterpotential C, isoform C) (Neither inacti	388.22	2	7.47	3112849	7.55	783942	0.32715
NPPPTLMRPTNWSQQINDFISESLE	ninaC	FI21215p1 (Neither inactivation nor afterpotential C, isoform C) (Neither inacti	1014.84	3	55.94	2051910	55.85	1795906	1.13697
NPPPTLMRPTNWSQQINDFISESLE	ninaC	FI21215p1 (Neither inactivation nor afterpotential C, isoform C) (Neither inacti	1020.17	3	52.77	5616241	52.6	3954417	0.91466
QYTTEEAR	ninaC	FI21215p1 (Neither inactivation nor afterpotential C, isoform C) (Neither inacti	499.233	2	9.23	582523	9.39	114303	0.2549
SCQDQDLIMDR	ninaC	FI21215p1 (Neither inactivation nor afterpotential C, isoform C) (Neither inacti	690.798	2	23.63	8294816	23.65	3307863	0.51804
SCQDQDLIMDR	ninaC	FI21215p1 (Neither inactivation nor afterpotential C, isoform C) (Neither inacti	698.795	2	18.85	5333858	18.8	2256882	0.54965
SFHSDVVQQQMK	ninaC	FI21215p1 (Neither inactivation nor afterpotential C, isoform C) (Neither inacti	717.346	2	18.46	3748466	18.46	2122639	0.73561
SFHSDVVQQQMK	ninaC	FI21215p1 (Neither inactivation nor afterpotential C, isoform C) (Neither inacti	478.566	3	18.46	11674488	18.42	8562709	0.95279
SFHSDVVQQQMK	ninaC	FI21215p1 (Neither inactivation nor afterpotential C, isoform C) (Neither inacti	725.343	2	13.74	1001280	13.67	558782	0.72495

SFHSDVVQQQMK	ninaC	FI21215p1 (Neither inactivation nor afterpotential C, isoform C) (Neither inacti	483.898	3	13.74	2486371	13.7	1898734	0.99202
SSLDESIMLMFTNQLTK	ninaC	FI21215p1 (Neither inactivation nor afterpotential C, isoform C) (Neither inacti	979.484	2	58.98	726179	58.79	934663	1.67199
SSLDESIMLMFTNQLTK	ninaC	FI21215p1 (Neither inactivation nor afterpotential C, isoform C) (Neither inacti	653.325	3	58.9	731597	58.75	1150737	2.04327
SSLDESIMLMFTNQLTK	ninaC	FI21215p1 (Neither inactivation nor afterpotential C, isoform C) (Neither inacti	987.482	2	52.23	1067574	51.98	1917715	2.3335
SSLDESIMLMFTNQLTK	ninaC	FI21215p1 (Neither inactivation nor afterpotential C, isoform C) (Neither inacti	658.657	3	52.23	889198	51.98	1157228	1.69061
SYALNTLSAGCISQVNNLR	ninaC	FI21215p1 (Neither inactivation nor afterpotential C, isoform C) (Neither inacti	1041.03	2	38.84	1038066	38.54	265120	0.33177
SYALNTLSAGCISQVNNLR	ninaC	FI21215p1 (Neither inactivation nor afterpotential C, isoform C) (Neither inacti	694.353	3	38.76	1954606	38.58	912018	0.60613
TALDNLLTKPDGLFYIIDDASR	ninaC	FI21215p1 (Neither inactivation nor afterpotential C, isoform C) (Neither inacti	1226.14	2	58.41	1814072	58.25	838256	0.60027
TALDNLLTKPDGLFYIIDDASR	ninaC	FI21215p1 (Neither inactivation nor afterpotential C, isoform C) (Neither inacti	817.764	3	58.41	21971862	58.25	11525014	0.68139
TLAANFR	ninaC	FI21215p1 (Neither inactivation nor afterpotential C, isoform C) (Neither inacti	396.722	2	20.39	6184618	20.39	1161221	0.24391
VATTDGTQHNFHIFYYFYDFINQQN	ninaC	FI21215p1 (Neither inactivation nor afterpotential C, isoform C) (Neither inacti	863.914	4	56.09	1522872	55.61	34117	0.0291
YYNDEFLAR	ninaC	FI21215p1 (Neither inactivation nor afterpotential C, isoform C) (Neither inacti	595.777	2	28.3	10393152	28.23	1903322	0.2379
VSVNDFIIK	muc	Midline uncoordinated, isoform D (EC 2.3.1.12)	517.798	2	38.03	442234	37.88	706401	2.07502
VVDGAVAAR	muc	Midline uncoordinated, isoform D (EC 2.3.1.12)	429.246	2	12.17	236652	12.23	154993	0.85079
YEDIPVTNMR	muc	Midline uncoordinated, isoform D (EC 2.3.1.12)	627.295	2	20.92	490136	20.93	697704	1.84917
IGGVTLEILAK	CG9674	Uncharacterized protein, isoform E (Uncharacterized protein, isoform H) (EC 1	557.348	2	40.98	21782	40.9	251762	15.0146
LVSEVGVGVVASGVAK	CG9674	Uncharacterized protein, isoform E (Uncharacterized protein, isoform H) (EC 1	735.93	2	33.15	0	33.02	197421	#DIV/0!
LTTNNFALGYTTK	porin	Porin, isoform D (Porin, isoform E)	722.378	2	31.55	326396	31.43	582312	2.31757
VFGSLETK	porin	Porin, isoform D (Porin, isoform E)	440.742	2	21.81	305180	21.82	380085	1.61788
VNNASQVGLGYQQK	porin	Porin, isoform D (Porin, isoform E)	753.389	2	19.18	385340	19.14	400935	1.35161
YQLDDDASVR	porin	Porin, isoform D (Porin, isoform E)	591.275	2	20.06	853548	20.05	1199040	1.82485
AATQEIAANVLR	Dlg5	Discs large 5, isoform D	628.852	2	39.24	4025067	39.04	5041733	1.62716
STSISNVAGASLTR	ens	Ensconsin, isoform K	682.362	2	25.86	816429	25.79	694557	1.10513
LSDVTVTPIPR	CDK2AP1	CDK2-associated protein 1, isoform B	599.346	2	29.17	1997051	29.1	2367705	1.54014
SQVQNFYNYQQQR	CDK2AP1	CDK2-associated protein 1, isoform B	851.9	2	23.81	499679	23.84	647495	1.68332
STSISNVAGASLTR	ens	Ensconsin, isoform I	682.362	2	25.86	816429	25.79	694557	1.10513
VTAVIPCFPYAR	Prps	Uncharacterized protein, isoform F (EC 2.7.6.1)	697.368	2	40.66	474085	40.36	413943	1.13424
VYAILTHGIFSGPAISR	Prps	Uncharacterized protein, isoform F (EC 2.7.6.1)	601.337	3	42.95	672496	42.83	628268	1.21361
AASPASSVSSLSR	Gug	Grunge, isoform I (EC 3.5.1.98)	610.317	2	19.87	670193	19.91	1047675	2.03072
ALQALVK	Gug	Grunge, isoform I (EC 3.5.1.98)	371.745	2	22.24	1429410	22.24	802676	0.72947
ASPSVQISSSGGGPSGPGSVGPGC	Gug	Grunge, isoform I (EC 3.5.1.98)	862.663	4	27.81	128265	27.69	91817	0.9299
MYAPQPGQR	Gug	Grunge, isoform I (EC 3.5.1.98)	532.253	2	11.22	597570	11.23	396368	0.86165
QHQAAAAAAAQQHK	Gug	Grunge, isoform I (EC 3.5.1.98)	477.58	3	7.39	102108	7.46	61942	0.78804
QSLHDQYFR	Gug	Grunge, isoform I (EC 3.5.1.98)	398.528	3	18.86	599467	18.89	746793	1.61829
SQSAIFVR	Gug	Grunge, isoform I (EC 3.5.1.98)	454.253	2	22.57	2827859	22.59	2397174	1.10119
WSPGVVADGDLLMFLR	Gug	Grunge, isoform I (EC 3.5.1.98)	888.461	2	59.21	701351	58.95	433086	0.80216
WSPGVVADGDLLMFLR	Gug	Grunge, isoform I (EC 3.5.1.98)	896.459	2	56.45	1934292	56.29	935150	0.62803
YGDPLAAK	Gug	Grunge, isoform I (EC 3.5.1.98)	417.721	2	19.02	1065996	19	938830	1.14407
AELDDNEDVGPR	CG32355	Uncharacterized protein, isoform K	665.299	2	17.49	854844	17.49	358372	0.54459
AGTNDTDPVIEVEPAGVR	CG32355	Uncharacterized protein, isoform K	920.458	2	31.26	799045	31.12	994850	1.61737
ELTPFEQELQR	CG32355	Uncharacterized protein, isoform K	695.354	2	34.45	1822534	34.23	951564	0.67824
EQRPSLLTTETQTSR	CG32355	Uncharacterized protein, isoform K	582.971	3	21	714722	20.97	708419	1.28758
FHDLQDAQSTASSGK	CG32355	Uncharacterized protein, isoform K	531.25	3	15.41	391086	15.36	283768	0.94257
GADSLILDGHIK	CG32355	Uncharacterized protein, isoform K	413.563	3	29.68	1264479	29.53	1469523	1.50969
GFMASAQDR	CG32355	Uncharacterized protein, isoform K	499.722	2	11.62	285875	11.75	165984	0.75424
GISEDNIQLR	CG32355	Uncharacterized protein, isoform K	572.801	2	25.08	2302903	24.99	2379743	1.34238
LAELEVER	CG32355	Uncharacterized protein, isoform K	479.764	2	23.2	1227100	23.26	1368562	1.44879
NYQPDFDSQEFQR	CG32355	Uncharacterized protein, isoform K	837.363	2	28.43	615106	28.34	745801	1.57505
RPLGPDEQPEIVLLLR	CG32355	Uncharacterized protein, isoform K	615.691	3	45.58	1022269	45.43	642182	0.81605
SNLQSGAVINK	CG32355	Uncharacterized protein, isoform K	565.812	2	16.44	787854	16.41	462469	0.76253
STASEPTEAEVELK	CG32355	Uncharacterized protein, isoform K	745.865	2	22.94	667731	22.98	801337	1.55896
SVEAAQEQDEVDGAR	CG32355	Uncharacterized protein, isoform K	802.363	2	15.1	653414	15.03	773644	1.53807
SVSNTFLQPHEDVIVYR	CG32355	Uncharacterized protein, isoform K	668.678	3	35.81	2074033	35.7	1364163	0.85442

TDYEIHTSQVDSSALEELPLQPENR	CG32355	Uncharacterized protein, isoform K	957.793	3	39.43	686104	39.2	452230	0.85623
THEDAQSLPGNSANPSR	CG32355	Uncharacterized protein, isoform K	594.278	3	13.23	675781	13.53	14072906	27.052
ANAVAAPFR	simj	Simjang, isoform D	458.754	2	21.85	720615	21.82	738030	1.33043
GSLSVTPTSAVPLPAHSK	simj	Simjang, isoform D	583.656	3	30.14	968499	29.97	1592607	2.13615
SSLPAGATLAAGTTITPATSSSHR	simj	Simjang, isoform D	752.392	3	30.52	3143778	30.4	4148506	1.7142
SSLSGSGGPAVSISATNSR	simj	Simjang, isoform D	867.934	2	24.08	1895042	24.04	2008027	1.37649
TNLPNLTITPSVTITPTSAPPSSLKPI	simj	Simjang, isoform D	935.19	3	42.52	2161882	42.25	1939128	1.16519
VTAVIPCFPYAR	Prps	Uncharacterized protein, isoform C (EC 2.7.6.1) (Uncharacterized protein, iso	697.368	2	40.66	474085	40.36	413943	1.13424
VYAILTHGIFSGPAISR	Prps	Uncharacterized protein, isoform C (EC 2.7.6.1) (Uncharacterized protein, iso	601.337	3	42.95	672496	42.83	628268	1.21361
GGGSGTDFYNQQR	tral	Trailer hitch, isoform C	693.805	2	16.19	310314	16.14	351936	1.47328
LVSQDNFGFDLAAVEAAAK	beta-Spec	Spectrin beta chain	983.499	2	48.76	272064	48.5	351415	1.67792
VAVVNQLAR	beta-Spec	Spectrin beta chain	485.296	2	22.75	392366	22.71	672076	2.2251
YATSQDESYR	beta-Spec	Spectrin beta chain	610.265	2	11.39	127721	11.46	145286	1.47769
GGGSGTDFYNQQR	tral	Trailer hitch, isoform H	693.805	2	16.19	310314	16.14	351936	1.47328
VTAVIPCFPYAR	Prps	Uncharacterized protein, isoform G (EC 2.7.6.1) (Uncharacterized protein, iso	697.368	2	40.66	474085	40.36	413943	1.13424
VYAILTHGIFSGPAISR	Prps	Uncharacterized protein, isoform G (EC 2.7.6.1) (Uncharacterized protein, iso	601.337	3	42.95	672496	42.83	628268	1.21361
ANAVAAPFR	simj	Simjang, isoform E	458.754	2	21.85	720615	21.82	738030	1.33043
GSLSVTPTSAVPLPAHSK	simj	Simjang, isoform E	583.656	3	30.14	968499	29.97	1592607	2.13615
SSLPAGATLAAGTTITPATSSSHR	simj	Simjang, isoform E	752.392	3	30.52	3143778	30.4	4148506	1.7142
SSLSGSGGPAVSISATNSR	simj	Simjang, isoform E	867.934	2	24.08	1895042	24.04	2008027	1.37649
TNLPNLTITPSVTITPTSAPPSSLKPI	simj	Simjang, isoform E	935.19	3	42.52	2161882	42.25	1939128	1.16519
GGGSGTDFYNQQR	tral	Trailer hitch, isoform E	693.805	2	16.19	310314	16.14	351936	1.47328
GGGSGTDFYNQQR	tral	Trailer hitch, isoform D	693.805	2	16.19	310314	16.14	351936	1.47328
GGGSGTDFYNQQR	tral	Trailer hitch, isoform I	693.805	2	16.19	310314	16.14	351936	1.47328
AGFAGDDAPR	Act79B	Actin 79B, isoform B	488.728	2	15.06	5346544	15.03	6072874	1.47551
AVFPSIVGRPR	Act79B	Actin 79B, isoform B	599.856	2	30.18	3527039	29.97	6233553	2.29587
SYELPDGQVITIGNER	Act79B	Actin 79B, isoform B	895.95	2	39.17	5791673	39	8517636	1.91046
SYELPDGQVITIGNER	Act79B	Actin 79B, isoform B	597.635	3	39.17	427938	38.97	800279	2.42931
TTGIVLDSGDGVSHTVPIYEGYALPI	Act79B	Actin 79B, isoform B	1051.22	3	45.78	1749429	45.58	1016220	0.75459
VAPEEHPVLLTEAPLNPK	Act79B	Actin 79B, isoform B	977.536	2	34.25	620752	34.11	748708	1.56681
VAPEEHPVLLTEAPLNPK	Act79B	Actin 79B, isoform B	652.026	3	34.21	12031327	34.11	16999678	1.83548
DITEASIWDSYVLPK	RpS26	40S ribosomal protein S26	868.941	2	48.98	315622	48.7	205408	0.84542
LTTSSHLTQFGTVENANTPGATAR	G9a	G9a, isoform B (EC 2.1.1.43)	825.414	3	27.32	294736	27.19	352075	1.55176
NFSGAELEGLVR	comt	Comatose, isoform B (EC 3.6.1.3)	646.336	2	38.11	673204	37.95	671954	1.29663
TVEVPSGYVGFSLVQR	comt	Comatose, isoform B (EC 3.6.1.3)	869.462	2	42.95	482529	43.06	1159918	3.12267
AFIVKPIPSPEVIPVIVFINPK	rdgA	Diacylglycerol kinase (DAG kinase) (EC 2.7.1.107)	806.491	3	58.34	7861730	58.25	6537246	1.08019
AFIVKPIPSPEVIPVIVFINPK	rdgA	Diacylglycerol kinase (DAG kinase) (EC 2.7.1.107)	605.12	4	58.3	339552	58.29	268976	1.02903
ALGWGGGYTDEPIGK	rdgA	Diacylglycerol kinase (DAG kinase) (EC 2.7.1.107)	760.873	2	33.66	1454790	33.49	1166978	1.04204
ALHEQGYSLQSVNK	rdgA	Diacylglycerol kinase (DAG kinase) (EC 2.7.1.107)	525.27	3	20.1	844926	20.13	448176	0.68905
FEESISYPK	rdgA	Diacylglycerol kinase (DAG kinase) (EC 2.7.1.107)	550.269	2	25.08	699628	25.06	249796	0.46381
FQHLLNPR	rdgA	Diacylglycerol kinase (DAG kinase) (EC 2.7.1.107)	512.788	2	19.99	2182798	19.93	880908	0.52425
GDVQVNPLEK	rdgA	Diacylglycerol kinase (DAG kinase) (EC 2.7.1.107)	549.793	2	22.75	2434720	22.76	798326	0.42595
GPGSTDEDLQIGSKPIK	rdgA	Diacylglycerol kinase (DAG kinase) (EC 2.7.1.107)	581.304	3	23.2	1491415	23.18	425538	0.37065
LGNYAPIIVPPSWIVK	rdgA	Diacylglycerol kinase (DAG kinase) (EC 2.7.1.107)	884.014	2	52.63	3322956	52.44	1330008	0.51994
QVFDLTQGGPK	rdgA	Diacylglycerol kinase (DAG kinase) (EC 2.7.1.107)	595.314	2	28.22	3580063	28.2	2623698	0.95202
SFAAFSQR	rdgA	Diacylglycerol kinase (DAG kinase) (EC 2.7.1.107)	457.23	2	23.28	6655876	23.37	6106585	1.19183
TIPMQVDGEACR	rdgA	Diacylglycerol kinase (DAG kinase) (EC 2.7.1.107)	688.818	2	23.21	1029594	23.29	655361	0.82687
TIPMQVDGEACR	rdgA	Diacylglycerol kinase (DAG kinase) (EC 2.7.1.107)	696.816	2	17.7	628084	17.41	337537	0.69811
VKPSVIEIELLNK	rdgA	Diacylglycerol kinase (DAG kinase) (EC 2.7.1.107)	741.451	2	38.07	1106361	37.96	617198	0.72469
VKPSVIEIELLNK	rdgA	Diacylglycerol kinase (DAG kinase) (EC 2.7.1.107)	494.636	3	38.07	3645006	37.92	2591446	0.92356
VSQDWCFIDSCTAEHYFR	rdgA	Diacylglycerol kinase (DAG kinase) (EC 2.7.1.107)	774.33	3	41.88	500902	41.72	464633	1.20498
VTPNDDVTDDHVDR	rdgA	Diacylglycerol kinase (DAG kinase) (EC 2.7.1.107)	533.241	3	14.64	1483070	14.56	900190	0.78849
YIIASATR	rdgA	Diacylglycerol kinase (DAG kinase) (EC 2.7.1.107)	447.756	2	19.6	1081315	19.55	314198	0.37746

STSISNVAGASLTR	ens	Ensconsin, isoform G	682.362	2	25.86	816429	25.79	694557	1.10513
AFIVKPIPSPEVIPVIVFINPK	rdgA	Diacylglycerol kinase (DAG kinase) (EC 2.7.1.107)	806.491	3	58.34	7861730	58.25	6537246	1.08019
AFIVKPIPSPEVIPVIVFINPK	rdgA	Diacylglycerol kinase (DAG kinase) (EC 2.7.1.107)	605.12	4	58.3	339552	58.29	268976	1.02903
ALHEQGYSLQSVNK	rdgA	Diacylglycerol kinase (DAG kinase) (EC 2.7.1.107)	525.27	3	20.1	844926	20.13	448176	0.68905
FEESISYPK	rdgA	Diacylglycerol kinase (DAG kinase) (EC 2.7.1.107)	550.269	2	25.08	699628	25.06	249796	0.46381
FQHLLNPR	rdgA	Diacylglycerol kinase (DAG kinase) (EC 2.7.1.107)	512.788	2	19.99	2182798	19.93	880908	0.52425
GDVQVNPLEK	rdgA	Diacylglycerol kinase (DAG kinase) (EC 2.7.1.107)	549.793	2	22.75	2434720	22.76	798326	0.42595
GPGSTDEDLQIGSKPIK	rdgA	Diacylglycerol kinase (DAG kinase) (EC 2.7.1.107)	581.304	3	23.2	1491415	23.18	425538	0.37065
LGNYAPIIVPPSWIVK	rdgA	Diacylglycerol kinase (DAG kinase) (EC 2.7.1.107)	884.014	2	52.63	3322956	52.44	1330008	0.51994
QVFDLTQGGPK	rdgA	Diacylglycerol kinase (DAG kinase) (EC 2.7.1.107)	595.314	2	28.22	3580063	28.2	2623698	0.95202
SFAAFSQR	rdgA	Diacylglycerol kinase (DAG kinase) (EC 2.7.1.107)	457.23	2	23.28	6655876	23.37	6106585	1.19183
TIPMQVDGEACR	rdgA	Diacylglycerol kinase (DAG kinase) (EC 2.7.1.107)	688.818	2	23.21	1029594	23.29	655361	0.82687
TIPMQVDGEACR	rdgA	Diacylglycerol kinase (DAG kinase) (EC 2.7.1.107)	696.816	2	17.7	628084	17.41	337537	0.69811
VKPSVIEIELLNK	rdgA	Diacylglycerol kinase (DAG kinase) (EC 2.7.1.107)	741.451	2	38.07	1106361	37.96	617198	0.72469
VKPSVIEIELLNK	rdgA	Diacylglycerol kinase (DAG kinase) (EC 2.7.1.107)	494.636	3	38.07	3645006	37.92	2591446	0.92356
VSQDWCFIDSCTAEHYFR	rdgA	Diacylglycerol kinase (DAG kinase) (EC 2.7.1.107)	774.33	3	41.88	500902	41.72	464633	1.20498
VTPNDDVTDDHVDR	rdgA	Diacylglycerol kinase (DAG kinase) (EC 2.7.1.107)	533.241	3	14.64	1483070	14.56	900190	0.78849
YIIASATR	rdgA	Diacylglycerol kinase (DAG kinase) (EC 2.7.1.107)	447.756	2	19.6	1081315	19.55	314198	0.37746
LVSQDNFGFDLAAVEAAAK	beta-Spec	Spectrin beta chain	983.499	2	48.76	272064	48.5	351415	1.67792
VAVVNQLAR	beta-Spec	Spectrin beta chain	485.296	2	22.75	392366	22.71	672076	2.2251
YATSQDESYR	beta-Spec	Spectrin beta chain	610.265	2	11.39	127721	11.46	145286	1.47769
AQELWELIVK	up	Upheld, isoform P	614.85	2	48.34	857786	48.15	594529	0.90036
DAGVLGLSSAAMER	up	Upheld, isoform P	696.843	2	30.26	1579477	30.2	1875543	1.54254
IKPLAIEGFGEAK	up	Upheld, isoform P	458.266	3	33.5	4523125	33.37	3961091	1.13762
ISLSFR	up	Upheld, isoform P	361.713	2	30.22	1860854	30.16	1791066	1.25032
IAAEVAAPLAK	Flo2	Flotillin 2, isoform J	527.319	2	25.68	101116	25.6	592504	7.61191
AGGEVLTFDQLALR	RpL18	Ribosomal protein L18, isoform B (Ribosomal protein L18, isoform C)	745.404	2	43.73	1193950	43.51	848280	0.92294
LTVCALHVTQTAR	RpL18	Ribosomal protein L18, isoform B (Ribosomal protein L18, isoform C)	490.601	3	26.12	441082	25.98	561270	1.65301
VYSPHVLNLTLIDLPGLTK	shi	Shibire, isoform N	698.405	3	53.41	463587	53.25	477207	1.3372
AQELWELIVK	up	Upheld, isoform O	614.85	2	48.34	857786	48.15	594529	0.90036
DAGVLGLSSAAMER	up	Upheld, isoform O	696.843	2	30.26	1579477	30.2	1875543	1.54254
IKPLAIEGFGEAK	up	Upheld, isoform O	458.266	3	33.5	4523125	33.37	3961091	1.13762
ISLSFR	up	Upheld, isoform O	361.713	2	30.22	1860854	30.16	1791066	1.25032
IAAEVAAPLAK	Flo2	Flotillin 2, isoform I	527.319	2	25.68	101116	25.6	592504	7.61191
AASPASSVSSLSR	Gug	Grunge, isoform J (EC 3.5.1.98)	610.317	2	19.87	670193	19.91	1047675	2.03072
ALQALVK	Gug	Grunge, isoform J (EC 3.5.1.98)	371.745	2	22.24	1429410	22.24	802676	0.72947
ASPSVQISSSGGGPSGPGSVGPG	Gug	Grunge, isoform J (EC 3.5.1.98)	862.663	4	27.81	128265	27.69	91817	0.9299
MYAPQPGQR	Gug	Grunge, isoform J (EC 3.5.1.98)	532.253	2	11.22	597570	11.23	396368	0.86165
QHQAAAAAAAQQHK	Gug	Grunge, isoform J (EC 3.5.1.98)	477.58	3	7.39	102108	7.46	61942	0.78804
QSLHDQYFR	Gug	Grunge, isoform J (EC 3.5.1.98)	398.528	3	18.86	599467	18.89	746793	1.61829
SQSAIFVR	Gug	Grunge, isoform J (EC 3.5.1.98)	454.253	2	22.57	2827859	22.59	2397174	1.10119
WSPGVVADGDLLMFLR	Gug	Grunge, isoform J (EC 3.5.1.98)	888.461	2	59.21	701351	58.95	433086	0.80216
WSPGVVADGDLLMFLR	Gug	Grunge, isoform J (EC 3.5.1.98)	896.459	2	56.45	1934292	56.29	935150	0.62803
YGDPLAAK	Gug	Grunge, isoform J (EC 3.5.1.98)	417.721	2	19.02	1065996	19	938830	1.14407
AELDDNEDVGPR	CG32352	Uncharacterized protein, isoform L	665.299	2	17.49	854844	17.49	358372	0.54459
AGTNDTDPVIEVEPAGVR	CG32352	Uncharacterized protein, isoform L	920.458	2	31.26	799045	31.12	994850	1.61737
ELTPFEQELQR	CG32352	Uncharacterized protein, isoform L	695.354	2	34.45	1822534	34.23	951564	0.67824
EQRPSLLTTETQTSR	CG32352	Uncharacterized protein, isoform L	582.971	3	21	714722	20.97	708419	1.28758
FATLPSIR	CG32352	Uncharacterized protein, isoform L	452.766	2	32.1	2652917	31.93	2934962	1.43715
FHDLQDAQSTASSGK	CG32352	Uncharacterized protein, isoform L	531.25	3	15.41	391086	15.36	283768	0.94257
GADSLILDGHIK	CG32352	Uncharacterized protein, isoform L	413.563	3	29.68	1264479	29.53	1469523	1.50969
GFMASAQDR	CG32352	Uncharacterized protein, isoform L	499.722	2	11.62	285875	11.75	165984	0.75424
GISEDNIQLR	CG32352	Uncharacterized protein, isoform L	572.801	2	25.08	2302903	24.99	2379743	1.34238

LAELEVER	CG32352	Uncharacterized protein, isoform L	479.764	2	23.2	1227100	23.26	1368562	1.44879
NYQPDFDSQEFQR	CG32352	Uncharacterized protein, isoform L	837.363	2	28.43	615106	28.34	745801	1.57505
RPLGPDEQPEIVLLLR	CG32352	Uncharacterized protein, isoform L	615.691	3	45.58	1022269	45.43	642182	0.81605
SNLQSGAVINK	CG32352	Uncharacterized protein, isoform L	565.812	2	16.44	787854	16.41	462469	0.76253
STASEPTEAEVELK	CG32352	Uncharacterized protein, isoform L	745.865	2	22.94	667731	22.98	801337	1.55896
SVEAAQEQDEVDGAR	CG32352	Uncharacterized protein, isoform L	802.363	2	15.1	653414	15.03	773644	1.53807
SVSNTFLQPHEDVIVYR	CG32352	Uncharacterized protein, isoform L	668.678	3	35.81	2074033	35.7	1364163	0.85442
TDYEIHTSQVDSSALEELPLQPENR	CG32352	Uncharacterized protein, isoform L	957.793	3	39.43	686104	39.2	452230	0.85623
THEDAQSLPGNSANPSR	CG32352	Uncharacterized protein, isoform L	594.278	3	13.23	675781	13.53	14072906	27.052
VTAVIPCFPYAR	Prps	Uncharacterized protein, isoform D (EC 2.7.6.1)	697.368	2	40.66	474085	40.36	413943	1.13424
VYAILTHGIFSGPAISR	Prps	Uncharacterized protein, isoform D (EC 2.7.6.1)	601.337	3	42.95	672496	42.83	628268	1.21361
GGGSGTDFYNQQR	tral	Trailer hitch, isoform F	693.805	2	16.19	310314	16.14	351936	1.47328
AIGAGGQSTQVR	Sap130	Sin3A-associated protein 130, isoform F	572.807	2	12.25	925618	12.32	536264	0.75261
GPGAGGTALTATNLPTTR	Sap130	Sin3A-associated protein 130, isoform F	828.439	2	27.36	569694	27.23	266622	0.60796
IGDQPATGGAGGR	Sap130	Sin3A-associated protein 130, isoform F	578.789	2	10.2	451645	10.33	79684	0.22919
IIQLQQPATGTTQQIIGSGAR	Sap130	Sin3A-associated protein 130, isoform F	727.738	3	34.41	1767358	34.26	2314474	1.70118
QLENVTATGR	Sap130	Sin3A-associated protein 130, isoform F	544.788	2	15.37	399235	15.31	452872	1.47356
SDGGGGISIAGTPILSGGTIK	Sap130	Sin3A-associated protein 130, isoform F	929.499	2	41.44	945778	41.22	943168	1.29545
TQITAIPAAPAR	Sap130	Sin3A-associated protein 130, isoform F	605.351	2	25.4	3213530	25.35	3375310	1.36444
VQPSLTITQLNPIGK	Sap130	Sin3A-associated protein 130, isoform F	804.97	2	41.17	1625853	40.98	1635254	1.30655
VTHGVASSAASVPATVGPVNSTR	Sap130	Sin3A-associated protein 130, isoform F	722.382	3	23.81	505024	23.8	708422	1.82223
VTTGAGNPQQATASGALVSSFMR	Sap130	Sin3A-associated protein 130, isoform F	756.374	3	32.37	491161	32.25	670045	1.77216
ADNSAPAAQDDGSGAPVVR	Mi-2	Mi-2, isoform D (EC 3.6.1.3)	899.422	2	16.91	470838	16.88	285548	0.78783
AVAAATSAATGATGK	Mi-2	Mi-2, isoform D (EC 3.6.1.3)	624.333	2	13.11	1665996	13.11	983701	0.76703
HHYEQHQEDVGR	Mi-2	Mi-2, isoform D (EC 3.6.1.3)	512.231	3	7.62	201593	7.78	286609	1.84687
HHYEQHQEDVGR	Mi-2	Mi-2, isoform D (EC 3.6.1.3)	384.425	4	7.62	91019	7.78	138645	1.97877
IDGGITGTLR	Mi-2	Mi-2, isoform D (EC 3.6.1.3)	501.783	2	24.03	1258518	24.04	897889	0.9268
QAIDYYQDLR	Mi-2	Mi-2, isoform D (EC 3.6.1.3)	642.815	2	28.79	539792	28.68	906085	2.18054
SVTGDEPEEPR	Mi-2	Mi-2, isoform D (EC 3.6.1.3)	608.278	2	13.23	438146	13.27	556388	1.64961
VGGNIEVLGFNAR	Mi-2	Mi-2, isoform D (EC 3.6.1.3)	673.365	2	37.32	2342650	37.14	3356129	1.86103
GAFGKPQGTVAR	RpL10	Ribosomal protein L10, isoform E	396.888	3	13.7	378695	13.67	452705	1.55292
IFDLGR	RpL10	Ribosomal protein L10, isoform E	360.706	2	28.76	454688	28.68	577700	1.65048
AQELWELIVK	up	Upheld, isoform Q (Upheld, isoform R) (Upheld, isoform S)	614.85	2	48.34	857786	48.15	594529	0.90036
DAGVLGLSSAAMER	up	Upheld, isoform Q (Upheld, isoform R) (Upheld, isoform S)	696.843	2	30.26	1579477	30.2	1875543	1.54254
IKPLAIEGFGEAK	up	Upheld, isoform Q (Upheld, isoform R) (Upheld, isoform S)	458.266	3	33.5	4523125	33.37	3961091	1.13762
ISLSFR	up	Upheld, isoform Q (Upheld, isoform R) (Upheld, isoform S)	361.713	2	30.22	1860854	30.16	1791066	1.25032
IAAEVAAPLAK	Flo2	Flotillin 2, isoform L	527.319	2	25.68	101116	25.6	592504	7.61191
GAAQNIIPASTGAAK	Gapdh2	Glyceraldehyde-3-phosphate dehydrogenase (EC 1.2.1.12)	685.375	2	22.79	1294864	22.83	1032530	1.03586
VINDNFEIVEGLMTTVHATTATQK	Gapdh2	Glyceraldehyde-3-phosphate dehydrogenase (EC 1.2.1.12)	883.446	3	44.43	15660984	44.21	14722617	1.2212
VPTPNVSVVDLTVR	Gapdh2	Glyceraldehyde-3-phosphate dehydrogenase (EC 1.2.1.12)	748.428	2	39.69	2420706	39.45	1346589	0.72263
VYSPHVLNLTLIDLPGLTK	shi	Shibire, isoform O	698.405	3	53.41	463587	53.25	477207	1.3372
AAEFNFR	wupA	Wings up A, isoform K (Wings up A, isoform L) (Wings up A, isoform M)	427.711	2	24.96	9042040	24.95	9905750	1.42313
GFMTPER	wupA	Wings up A, isoform K (Wings up A, isoform L) (Wings up A, isoform M)	419.2	2	19.67	1284815	19.67	1246317	1.26012
NLSDASEDTLK	wupA	Wings up A, isoform K (Wings up A, isoform L) (Wings up A, isoform M)	596.788	2	18.67	925293	18.66	786155	1.1037
ALLVWSPTK	CoRest	CoRest, isoform K	507.803	2	37.31	2318735	37.1	1637547	0.91741
SIASLVK	CoRest	CoRest, isoform K	359.229	2	23.99	840931	23.99	310644	0.47987
VLFEQAFQFHGK	CoRest	CoRest, isoform K	484.254	3	38.03	957244	37.88	274801	0.37292
SPVTNIAR	AP-2mu	Adaptor protein complex 2, mu subunit, isoform A (Adaptor protein complex 2	429.246	2	16.3	972986	16.1	1029269	1.37418
TFITQQGIK	AP-2mu	Adaptor protein complex 2, mu subunit, isoform A (Adaptor protein complex 2	518.295	2	24.31	387861	24.28	824058	2.75997
GIVPQFVTFLR	Kap-alpha	Importin subunit alpha	638.874	2	53.91	871	51.78	836549	1247.66
FQSLGVAFYR	Rab7	CG5915 protein (CG5915-PA) (GH03685p) (MIP21169p) (Rab7 protein) (Rab	594.314	2	37.76	127091	37.64	128722	1.31571
AAPLVAAGPAIVK	Ccp84Ag	Ccp84Ag (Cuticle protein)	589.369	2	31.83	1401640	31.7	1299475	1.20435
AAVAAPVVR	Ccp84Ag	Ccp84Ag (Cuticle protein)	427.266	2	18.94	2185353	18.93	1045509	0.62148

LMNAYCDR	smt3	Small ubiquitin-related modifier (SUMO)	521.726	2	16.48	162687	16.48	576360	4.60218
VLGQDNAVVQFK	smt3	Small ubiquitin-related modifier (SUMO)	659.362	2	30.18	2032621	29.97	8993196	5.74751
IGLIPLISDDVVER	RpII33	GH07456p (RNA polymerase II 33kD subunit) (EC 2.7.7.6) (RNA polymerase	769.943	2	51.98	343136	51.54	499279	1.89016
VTVAEEGAR	Mpcp2	MIP08013p1 (Mitochondrial phosphate carrier protein 2, isoform A) (Mitochon	466.246	2	11.65	204818	11.71	82620	0.52401
ANAVAAPFR	simj	Simjang, isoform A (Simjang, isoform B)	458.754	2	21.85	720615	21.82	738030	1.33043
GSLSVTPTSAVPLPAHSK	simj	Simjang, isoform A (Simjang, isoform B)	583.656	3	30.14	968499	29.97	1592607	2.13615
SSLPAGATLAAGTTITPATSSSHR	simj	Simjang, isoform A (Simjang, isoform B)	752.392	3	30.52	3143778	30.4	4148506	1.7142
SSLSGSGGPAVSISATNSR	simj	Simjang, isoform A (Simjang, isoform B)	867.934	2	24.08	1895042	24.04	2008027	1.37649
TNLPNLTITPSVTITPTSAPPSSLKPI	simj	Simjang, isoform A (Simjang, isoform B)	935.19	3	42.52	2161882	42.25	1939128	1.16519
GATALGPATTSGASER	PIP5K59E	Phosphatidylinositol 4-phosphate 5-kinase at 59B, isoform I (EC 2.7.1.-) (EC 2	723.863	2	16.98	232423	17.03	258407	1.44427
EYTAAIEAK	Phb2	Prohibitin	498.256	2	17.18	20133	17.23	257332	16.6038
IVQAEGEAEAAK	Phb2	Prohibitin	608.314	2	13.11	424400	13.11	493702	1.51116
LGGIQSDIYSEGLHVR	Phb2	Prohibitin	581.972	3	33.76	567085	33.65	472144	1.08156
NFPALTYFR	l(2)01289	Lethal (2) 01289, isoform K	564.795	2	42.88	433584	42.79	472450	1.41548
NFPALTYFR	l(2)01289	Lethal (2) 01289, isoform H	564.795	2	42.88	433584	42.79	472450	1.41548
AGLQFPVGR	His2A:CG	Histone H2A	472.769	2	31.56	98633592	31.42	106838928	1.40711
HLQLAIR	His2A:CG	Histone H2A	425.767	2	22.72	21451580	22.66	9817742	0.59453
LLSGVTIAQGGVLPNIQAVLLPK	His2A:CG	Histone H2A	1151.2	2	58.27	5972554	58.05	3630529	0.78965
LLSGVTIAQGGVLPNIQAVLLPK	His2A:CG	Histone H2A	767.802	3	58.3	10409686	58.05	8036129	1.00284
VGAGAPVYLAAVMEYLAAEVLELA(	His2A:CG	Histone H2A	963.844	3	65.83	2552861	65.87	479648	0.24407
VGAGAPVYLAAVMEYLAAEVLELA(	His2A:CG	Histone H2A	723.135	4	65.83	1259116	65.83	400141	0.41283
VGAGAPVYLAAVMEYLAAEVLELA(	His2A:CG	Histone H2A	969.176	3	65.67	521759	65.67	545456	1.35804
ILIQGNASGSDESSAER	mIF3	IP07950p (Mitochondrial translation initiation factor 3, isoform A) (Mitochondri	867.419	2	19.52	3032484	19.49	1576337	0.67526
ITEHDLSSR	mIF3	IP07950p (Mitochondrial translation initiation factor 3, isoform A) (Mitochondri	353.181	3	12.46	486310	12.51	535959	1.43166
ITLIQQNQSISITTLEEAQK	mIF3	IP07950p (Mitochondrial translation initiation factor 3, isoform A) (Mitochondri	753.413	3	42.15	498258	41.91	390505	1.01811
HAEIASSFIR	UQCR-6.4	GEO11443p1 (RH56961p) (Ubiquinol-cytochrome c reductase 6.4 kDa subun	565.801	2	24.81	2498313	24.75	1394569	0.72513
LVLQYVPIYGSK	UQCR-6.4	GEO11443p1 (RH56961p) (Ubiquinol-cytochrome c reductase 6.4 kDa subun	690.4	2	41.72	1373954	41.56	271010	0.25623
IQVNPNHEIIKPTTTR	DnaJ-1	DNAJ-1 (DnaJ-like-1, isoform B)	621.015	3	20.92	19679	20.89	261697	17.275
DSSLSSGALANILAIFK	Hn	Henna, isoform B (EC 1.14.16.1)	853.97	2	65.24	302707	65.18	21367	0.09169
FANSIPRPFGVR	Hn	Henna, isoform B (EC 1.14.16.1)	454.254	3	31.64	779696	31.6	818437	1.36359
ADNSAPAAQDDGSGAPVVR	Mi-2	Mi-2, isoform B (EC 3.6.1.3)	899.422	2	16.91	470838	16.88	285548	0.78783
AVAAATSAATGATGK	Mi-2	Mi-2, isoform B (EC 3.6.1.3)	624.333	2	13.11	1665996	13.11	983701	0.76703
HHYEQHQEDVGR	Mi-2	Mi-2, isoform B (EC 3.6.1.3)	512.231	3	7.62	201593	7.78	286609	1.84687
HHYEQHQEDVGR	Mi-2	Mi-2, isoform B (EC 3.6.1.3)	384.425	4	7.62	91019	7.78	138645	1.97877
IDGGITGTLR	Mi-2	Mi-2, isoform B (EC 3.6.1.3)	501.783	2	24.03	1258518	24.04	897889	0.9268
QAIDYYQDLR	Mi-2	Mi-2, isoform B (EC 3.6.1.3)	642.815	2	28.79	539792	28.68	906085	2.18054
SVTGDEPEEPR	Mi-2	Mi-2, isoform B (EC 3.6.1.3)	608.278	2	13.23	438146	13.27	556388	1.64961
VGGNIEVLGFNAR	Mi-2	Mi-2, isoform B (EC 3.6.1.3)	673.365	2	37.32	2342650	37.14	3356129	1.86103
AGTNDTDPVIEVEPAGVR	CG17926	GH14495p	920.458	2	31.26	799045	31.12	994850	1.61737
ELTPFEQELQR	CG17926	GH14495p	695.354	2	34.45	1822534	34.23	951564	0.67824
RPLGPDEQPEIVLLLR	CG17926	GH14495p	615.691	3	45.58	1022269	45.43	642182	0.81605
THEDAQSLPGNSANPSR	CG17926	GH14495p	594.278	3	13.23	675781	13.53	14072906	27.052
AALDCVELFATEQR	Sin3A	LD13852p (Sin3A, isoform B) (Sin3A, isoform C) (Sin3A, isoform E)	811.896	2	40.25	1722570	40.03	1260136	0.9503
AALDCVELFATEQR	Sin3A	LD13852p (Sin3A, isoform B) (Sin3A, isoform C) (Sin3A, isoform E)	541.6	3	40.21	619337	39.99	590227	1.23798
ANTADGANTVQIHVTGGGAANNPA	Sin3A	LD13852p (Sin3A, isoform B) (Sin3A, isoform C) (Sin3A, isoform E)	928.703	4	26.74	509613	26.67	542107	1.38187
DVSFSEASSK	Sin3A	LD13852p (Sin3A, isoform B) (Sin3A, isoform C) (Sin3A, isoform E)	528.746	2	17.44	1467180	17.41	381916	0.33815
DYHAGLQQQAHQR	Sin3A	LD13852p (Sin3A, isoform B) (Sin3A, isoform C) (Sin3A, isoform E)	517.919	3	11.22	2585659	11.33	2959684	1.48695
DYHAGLQQQAHQR	Sin3A	LD13852p (Sin3A, isoform B) (Sin3A, isoform C) (Sin3A, isoform E)	388.691	4	11.22	864204	11.33	948677	1.42602
ESTATALR	Sin3A	LD13852p (Sin3A, isoform B) (Sin3A, isoform C) (Sin3A, isoform E)	424.727	2	11.65	1476544	11.71	969884	0.85329
FHLDDHLGGTSQTIHQR	Sin3A	LD13852p (Sin3A, isoform B) (Sin3A, isoform C) (Sin3A, isoform E)	654.657	3	23.08	1395287	23.06	756858	0.70465
FHLDDHLGGTSQTIHQR	Sin3A	LD13852p (Sin3A, isoform B) (Sin3A, isoform C) (Sin3A, isoform E)	491.245	4	23.12	3523823	23.06	1769329	0.65225
FLEILHDYQR	Sin3A	LD13852p (Sin3A, isoform B) (Sin3A, isoform C) (Sin3A, isoform E)	445.235	3	35.73	2969216	35.43	0	0
GTSHQSAAEYVQDVDLSSCK	Sin3A	LD13852p (Sin3A, isoform B) (Sin3A, isoform C) (Sin3A, isoform E)	727.995	3	27.24	493988	27.08	189593	0.49857

ILTPQHGGAQTIAYLPSTTPTATNLK	Sin3A	LD13852p (Sin3A, isoform B) (Sin3A, isoform C) (Sin3A, isoform E)	898.82	3	35.8	4998395	35.62	5283785	1.37321
LANPALGGGGNTGR	Sin3A	LD13852p (Sin3A, isoform B) (Sin3A, isoform C) (Sin3A, isoform E)	627.831	2	17.75	10453019	17.75	7732981	0.96101
LGASYCALPQSTVPK	Sin3A	LD13852p (Sin3A, isoform B) (Sin3A, isoform C) (Sin3A, isoform E)	796.411	2	28.68	1479395	28.59	1263101	1.10911
LHAILCDR	Sin3A	LD13852p (Sin3A, isoform B) (Sin3A, isoform C) (Sin3A, isoform E)	499.266	2	19.41	1985761	19.38	633888	0.41468
LLEEQVR	Sin3A	LD13852p (Sin3A, isoform B) (Sin3A, isoform C) (Sin3A, isoform E)	443.753	2	17.75	2718818	17.76	2609800	1.24695
MLTEQEVYTQVAK	Sin3A	LD13852p (Sin3A, isoform B) (Sin3A, isoform C) (Sin3A, isoform E)	778.387	2	27.81	3209047	27.77	3556038	1.4395
NDILFEK	Sin3A	LD13852p (Sin3A, isoform B) (Sin3A, isoform C) (Sin3A, isoform E)	439.735	2	27.05	2341755	27.01	1764770	0.97897
NPFVAVPIVLK	Sin3A	LD13852p (Sin3A, isoform B) (Sin3A, isoform C) (Sin3A, isoform E)	598.874	2	46.1	1222769	45.94	767794	0.81569
QFVPDLFFAPR	Sin3A	LD13852p (Sin3A, isoform B) (Sin3A, isoform C) (Sin3A, isoform E)	668.856	2	50.52	6756025	50.25	7385320	1.42004
SAHAAAIGNLSAVNTSVSIK	Sin3A	LD13852p (Sin3A, isoform B) (Sin3A, isoform C) (Sin3A, isoform E)	956.018	2	31.61	170248	31.53	254171	1.9394
SAHAAAIGNLSAVNTSVSIK	Sin3A	LD13852p (Sin3A, isoform B) (Sin3A, isoform C) (Sin3A, isoform E)	637.681	3	31.61	2399426	31.53	2575551	1.39439
SHCIDTPGVIER	Sin3A	LD13852p (Sin3A, isoform B) (Sin3A, isoform C) (Sin3A, isoform E)	692.338	2	20.25	307279	20.24	237394	1.0036
SHCIDTPGVIER	Sin3A	LD13852p (Sin3A, isoform B) (Sin3A, isoform C) (Sin3A, isoform E)	461.894	3	20.22	598355	20.24	340785	0.73985
SSPGISYATPPLPSGPHGQHNSGS	Sin3A	LD13852p (Sin3A, isoform B) (Sin3A, isoform C) (Sin3A, isoform E)	887.432	3	25.86	488181	25.83	202251	0.53819
TADQINASGWGSASK	Sin3A	LD13852p (Sin3A, isoform B) (Sin3A, isoform C) (Sin3A, isoform E)	746.855	2	22.49	436619	22.54	444931	1.32377
TILDDAANLLIHHVK	Sin3A	LD13852p (Sin3A, isoform B) (Sin3A, isoform C) (Sin3A, isoform E)	418.99	4	49.12	1380604	49.31	716808	0.67446
TISGTQTVATAVGNLATISQQQPVQ	Sin3A	LD13852p (Sin3A, isoform B) (Sin3A, isoform C) (Sin3A, isoform E)	1041.89	3	45.65	3056186	45.35	3369934	1.4324
TYNNNQQQQNHVIGSGLNATR	Sin3A	LD13852p (Sin3A, isoform B) (Sin3A, isoform C) (Sin3A, isoform E)	786.383	3	19.45	600429	19.45	504776	1.09209
VLENLQK	Sin3A	LD13852p (Sin3A, isoform B) (Sin3A, isoform C) (Sin3A, isoform E)	422.25	2	16.41	3516505	16.41	2648950	0.97855
VTIELLDSEPEEVDKPAALK	Sin3A	LD13852p (Sin3A, isoform B) (Sin3A, isoform C) (Sin3A, isoform E)	732.726	3	39.02	4118678	38.81	3130918	0.9875
YAHFQTYVNK	Sin3A	LD13852p (Sin3A, isoform B) (Sin3A, isoform C) (Sin3A, isoform E)	635.814	2	19.83	763160	19.77	541027	0.92093
YAHFQTYVNK	Sin3A	LD13852p (Sin3A, isoform B) (Sin3A, isoform C) (Sin3A, isoform E)	424.212	3	19.83	1837847	19.81	1859402	1.31428
YVGHAPNQNLTHGHNAK	Sin3A	LD13852p (Sin3A, isoform B) (Sin3A, isoform C) (Sin3A, isoform E)	619.975	3	10.09	210214	10.13	163530	1.01055
LSEASQAADESER	Tm1	RE21974p (Tropomyosin 1, isoform N)	696.816	2	11.57	343010	11.67	695696	2.63472
AGSEPYVPPPSYWNR	Mf	Mf4 protein (Myofilin, isoform L) (Myofilin, isoform M)	860.41	2	33.23	748487	33.06	1566838	2.71933
AIYDHHYSR	Mf	Mf4 protein (Myofilin, isoform L) (Myofilin, isoform M)	581.275	2	11.31	475566	11.41	651391	1.77932
AIYDHHYSR	Mf	Mf4 protein (Myofilin, isoform L) (Myofilin, isoform M)	387.853	3	11.31	2895978	11.41	4746762	2.12924
ASRPYSSYLDSPSYR	Mf	Mf4 protein (Myofilin, isoform L) (Myofilin, isoform M)	874.916	2	25.08	1194562	25.03	1397150	1.51935
ASRPYSSYLDSPSYR	Mf	Mf4 protein (Myofilin, isoform L) (Myofilin, isoform M)	583.613	3	25.12	5487893	25.03	8501748	2.01245
ASSLEPLDELFER	Mf	Mf4 protein (Myofilin, isoform L) (Myofilin, isoform M)	753.378	2	48.45	4498040	48.19	5102070	1.47348
DKPLTPNSLVPLEYEPEDK	Mf	Mf4 protein (Myofilin, isoform L) (Myofilin, isoform M)	728.707	3	38.98	567161	38.81	728559	1.66871
DSYLSPVK	Mf	Mf4 protein (Myofilin, isoform L) (Myofilin, isoform M)	454.74	2	22.04	1523720	22.02	1430945	1.21994
DTYGYSPR	Mf	Mf4 protein (Myofilin, isoform L) (Myofilin, isoform M)	479.717	2	14.86	2640775	14.83	2840777	1.39742
FWQSYIR	Mf	Mf4 protein (Myofilin, isoform L) (Myofilin, isoform M)	500.256	2	32.37	739255	32.25	1687659	2.9656
GGPSVFDR	Mf	Mf4 protein (Myofilin, isoform L) (Myofilin, isoform M)	417.709	2	19.86	5727839	19.85	7157511	1.62328
LLAELNK	Mf	Mf4 protein (Myofilin, isoform L) (Myofilin, isoform M)	400.747	2	22.38	846200	22.35	1253332	1.92405
NHLEMIGR	Mf	Mf4 protein (Myofilin, isoform L) (Myofilin, isoform M)	493.248	2	12.87	1554021	12.91	1080925	0.90357
SASPFRPAR	Mf	Mf4 protein (Myofilin, isoform L) (Myofilin, isoform M)	494.77	2	13.46	1272464	13.42	1443660	1.47381
SIYDEPATANER	Mf	Mf4 protein (Myofilin, isoform L) (Myofilin, isoform M)	683.318	2	18.42	9216683	18.42	15046478	2.12072
WGPRPTEVAYDAEGLPIFHPR	Mf	Mf4 protein (Myofilin, isoform L) (Myofilin, isoform M)	602.809	4	43.06	371242	42.9	651591	2.28003
YGLYLR	Mf	Mf4 protein (Myofilin, isoform L) (Myofilin, isoform M)	392.721	2	28.26	5499678	28.16	8903475	2.10303
LGGVPVGVIAVETR	ACC	Acetyl-CoA carboxylase, isoform B (Acetyl-CoA carboxylase, isoform C) (EC 6	683.906	2	38.38	348778	38.27	503425	1.87503
DFPGPYCAR	Swim	RE01730p (Secreted Wg-interacting molecule, isoform A) (Secreted Wg-intera	541.74	2	24.28	1817528	24.24	1758109	1.25657
QQGCEGGHLDAAWR	Swim	RE01730p (Secreted Wg-interacting molecule, isoform A) (Secreted Wg-intera	528.904	3	20.29	345703	20.31	382594	1.43766
GISQEFAEK	P32	CG6459 protein (CG6459-PA) (LD29590p) (P32)	504.753	2	20.03	420230	20.01	235393	0.72766
LTGADVELTK	P32	CG6459 protein (CG6459-PA) (LD29590p) (P32)	523.79	2	22.57	348888	22.55	452549	1.68501
ESTLHLVLR	RpL40	RE10554p (Ribosomal protein L40, isoform B)	534.314	2	29.06	19834334	28.96	12823394	0.83986
ESTLHLVLR	RpL40	RE10554p (Ribosomal protein L40, isoform B)	356.545	3	29.02	13873703	28.96	8349692	0.78181
MQIFVK	RpL40	RE10554p (Ribosomal protein L40, isoform B)	383.22	2	27.43	8697430	27.39	10348569	1.54565
TITLEVEPSDTIENVK	RpL40	RE10554p (Ribosomal protein L40, isoform B)	894.467	2	36.85	41743372	36.64	47966256	1.49269
TITLEVEPSDTIENVK	RpL40	RE10554p (Ribosomal protein L40, isoform B)	596.647	3	36.85	1563861	36.68	1963090	1.63066
TLSDYNIQK	RpL40	RE10554p (Ribosomal protein L40, isoform B)	541.28	2	19.53	19914654	19.52	7860957	0.51277
GVDLDQLLDMPNNQLVELMHSR	RpS15	GEO07301p1 (RE08270p) (Ribosomal protein S15, isoform A)	846.421	3	56.93	2438393	56.76	3030472	1.61447

GVDLDQLLDMPNNQLVELMHSR	RpS15	GEO07301p1 (RE08270p) (Ribosomal protein S15, isoform A)	851.753	3	52.82	1993202	52.51	2732090	1.7806
GVDLDQLLDMPNNQLVELMHSR	RpS15	GEO07301p1 (RE08270p) (Ribosomal protein S15, isoform A)	857.084	3	46.75	939137	46.49	1012387	1.40036
HGRPGIGATHSSR	RpS15	GEO07301p1 (RE08270p) (Ribosomal protein S15, isoform A)	666.848	2	7.93	677685	8.06	679248	1.30204
HGRPGIGATHSSR	RpS15	GEO07301p1 (RE08270p) (Ribosomal protein S15, isoform A)	444.901	3	7.93	4672602	8.06	5517679	1.53398
GGCQVIADTGTSLIAAPLEEATSINC	cathD	CathD, isoform A (EC 3.4.23.-) (CathD, isoform B) (CathD, isoform C) (Cathe	915.464	3	49.08	208849	48.86	305991	1.90326
AFSVALESIPLALAENSGLHPIETLSI	CCT5	Chaperonin containing TCP1 subunit 5, isoform A (GM12270p)	1017.22	3	62.61	384652	62.45	401044	1.3544
FEELTPEK	CCT5	Chaperonin containing TCP1 subunit 5, isoform A (GM12270p)	496.75	2	20.66	516313	20.64	311483	0.78369
IADGFELAAQCAIK	CCT5	Chaperonin containing TCP1 subunit 5, isoform A (GM12270p)	753.885	2	37.31	382322	37.14	458415	1.55759
LGVAGLVR	CCT5	Chaperonin containing TCP1 subunit 5, isoform A (GM12270p)	392.756	2	29.1	644815	29.01	620261	1.24957
VAIIDLQDNLEEFVK	Pdh	Photoreceptor dehydrogenase, isoform C (EC 1.1.1.1) (EC 1.1.1.105) (Pigme	873.47	2	52.44	438704	52.21	466578	1.38158
GYAFVTFTNR	Syp	Syncrip, isoform B (Syncrip, isoform L)	588.296	2	35.44	514118	35.33	604713	1.52795
VSVNDFIIK	muc	Midline uncoordinated, isoform A (EC 2.3.1.12)	517.798	2	38.03	442234	37.88	706401	2.07502
VVDGAVAAR	muc	Midline uncoordinated, isoform A (EC 2.3.1.12)	429.246	2	12.17	236652	12.23	154993	0.85079
YEDIPVTNMR	muc	Midline uncoordinated, isoform A (EC 2.3.1.12)	627.295	2	20.92	490136	20.93	697704	1.84917
AASPASSVSSLSR	Gug	Grunge, isoform D (EC 3.5.1.98) (Grunge, isoform E)	610.317	2	19.87	670193	19.91	1047675	2.03072
ALQALVK	Gug	Grunge, isoform D (EC 3.5.1.98) (Grunge, isoform E)	371.745	2	22.24	1429410	22.24	802676	0.72947
ASPSVQISSSGGGPSGPGSVGPGC	Gug	Grunge, isoform D (EC 3.5.1.98) (Grunge, isoform E)	862.663	4	27.81	128265	27.69	91817	0.9299
MYAPQPGQR	Gug	Grunge, isoform D (EC 3.5.1.98) (Grunge, isoform E)	532.253	2	11.22	597570	11.23	396368	0.86165
QHQAAAAAAAQQHK	Gug	Grunge, isoform D (EC 3.5.1.98) (Grunge, isoform E)	477.58	3	7.39	102108	7.46	61942	0.78804
QSLHDQYFR	Gug	Grunge, isoform D (EC 3.5.1.98) (Grunge, isoform E)	398.528	3	18.86	599467	18.89	746793	1.61829
SQSAIFVR	Gug	Grunge, isoform D (EC 3.5.1.98) (Grunge, isoform E)	454.253	2	22.57	2827859	22.59	2397174	1.10119
WSPGVVADGDLLMFLR	Gug	Grunge, isoform D (EC 3.5.1.98) (Grunge, isoform E)	888.461	2	59.21	701351	58.95	433086	0.80216
WSPGVVADGDLLMFLR	Gug	Grunge, isoform D (EC 3.5.1.98) (Grunge, isoform E)	896.459	2	56.45	1934292	56.29	935150	0.62803
YGDPLAAK	Gug	Grunge, isoform D (EC 3.5.1.98) (Grunge, isoform E)	417.721	2	19.02	1065996	19	938830	1.14407
FLAPQAQGPVGLALDIPIGILR	BcDNA:R	Uncharacterized protein, isoform B (Uncharacterized protein, isoform E)	1130.16	2	63.35	1256442	63.16	1860146	1.92321
FLAPQAQGPVGLALDIPIGILR	BcDNA:R	Uncharacterized protein, isoform B (Uncharacterized protein, isoform E)	753.779	3	63.38	2942061	63.13	4898123	2.16272
SVQRPQAVNNR	BcDNA:R	Uncharacterized protein, isoform B (Uncharacterized protein, isoform E)	634.845	2	9.41	110272	9.55	122764	1.4462
SVQRPQAVNNR	BcDNA:R	Uncharacterized protein, isoform B (Uncharacterized protein, isoform E)	423.565	3	9.37	375735	9.52	435353	1.50516
TAGVGYAAAQANPSVGASALLGGS	BcDNA:R	Uncharacterized protein, isoform B (Uncharacterized protein, isoform E)	920.702	4	36.12	888234	35.9	1594106	2.33137
FVGPFTRPVVTIMTLR	Vap33	VAMP-associated protein 33kDa, isoform A	612.018	3	49.47	300313	49.3	756291	3.27143
SLFDLPLTIEPEHELR	Vap33	VAMP-associated protein 33kDa, isoform A	637.007	3	51.03	379872	50.9	998731	3.41534
ELEIEQR	eIF4B	Eukaryotic translation initiation factor 4B, isoform E	458.74	2	17.88	4591316	17.9	2801196	0.79255
QGGTSSLNVFGSAKPVDTAAR	eIF4B	Eukaryotic translation initiation factor 4B, isoform E	688.355	3	30.18	747833	30.12	321395	0.55829
ELEIEQR	eIF4B	Eukaryotic initiation factor 4B (Eukaryotic translation initiation factor 4B, isofor	458.74	2	17.88	4591316	17.9	2801196	0.79255
GQFNRPELLR	eIF4B	Eukaryotic initiation factor 4B (Eukaryotic translation initiation factor 4B, isofor	410.563	3	24.81	1619273	24.76	1465741	1.17587
QGGTSSLNVFGSAKPVDTAAR	eIF4B	Eukaryotic initiation factor 4B (Eukaryotic translation initiation factor 4B, isofor	688.355	3	30.18	747833	30.12	321395	0.55829
LLEANPNYSK	mfas	FI19380p1 (Midline fasciclin, isoform J)	574.801	2	18.6	422357	18.57	399268	1.22802
LNLYSTHALFSDVMNR	mfas	FI19380p1 (Midline fasciclin, isoform J)	632.981	3	38.73	372398	38.45	772695	2.6954
LLEANPNYSK	mfas	Midline fasciclin, isoform F (Midline fasciclin, isoform G) (Midline fasciclin, isof	574.801	2	18.6	422357	18.57	399268	1.22802
LNLYSTHALFSDVMNR	mfas	Midline fasciclin, isoform F (Midline fasciclin, isoform G) (Midline fasciclin, isof	632.981	3	38.73	372398	38.45	772695	2.6954
LLEANPNYSK	mfas	Midline fasciclin, isoform E (Midline fasciclin, isoform O) (RH53953p)	574.801	2	18.6	422357	18.57	399268	1.22802
LNLYSTHALFSDVMNR	mfas	Midline fasciclin, isoform E (Midline fasciclin, isoform O) (RH53953p)	632.981	3	38.73	372398	38.45	772695	2.6954
LLEANPNYSK	mfas	Midline fasciclin, isoform H (Midline fasciclin, isoform N)	574.801	2	18.6	422357	18.57	399268	1.22802
LNLYSTHALFSDVMNR	mfas	Midline fasciclin, isoform H (Midline fasciclin, isoform N)	632.981	3	38.73	372398	38.45	772695	2.6954
AASPASSVSSLSR	Gug	Grunge, isoform C (EC 3.5.1.98)	610.317	2	19.87	670193	19.91	1047675	2.03072
ALQALVK	Gug	Grunge, isoform C (EC 3.5.1.98)	371.745	2	22.24	1429410	22.24	802676	0.72947
ASPSVQISSSGGGPSGPGSVGPGC	Gug	Grunge, isoform C (EC 3.5.1.98)	862.663	4	27.81	128265	27.69	91817	0.9299
MYAPQPGQR	Gug	Grunge, isoform C (EC 3.5.1.98)	532.253	2	11.22	597570	11.23	396368	0.86165
QHQAAAAAAAQQHK	Gug	Grunge, isoform C (EC 3.5.1.98)	477.58	3	7.39	102108	7.46	61942	0.78804
QSLHDQYFR	Gug	Grunge, isoform C (EC 3.5.1.98)	398.528	3	18.86	599467	18.89	746793	1.61829
SQSAIFVR	Gug	Grunge, isoform C (EC 3.5.1.98)	454.253	2	22.57	2827859	22.59	2397174	1.10119
WSPGVVADGDLLMFLR	Gug	Grunge, isoform C (EC 3.5.1.98)	888.461	2	59.21	701351	58.95	433086	0.80216
WSPGVVADGDLLMFLR	Gug	Grunge, isoform C (EC 3.5.1.98)	896.459	2	56.45	1934292	56.29	935150	0.62803

YGDPLAAK	Gug	Grunge, isoform C (EC 3.5.1.98)	417.721	2	19.02	1065996	19	938830	1.14407
AIYDHHYSR	Mf	GH23305p (Mf2 protein) (Myofilin, isoform C) (Myofilin, isoform Q)	581.275	2	11.31	475566	11.41	651391	1.77932
AIYDHHYSR	Mf	GH23305p (Mf2 protein) (Myofilin, isoform C) (Myofilin, isoform Q)	387.853	3	11.31	2895978	11.41	4746762	2.12924
ASRPYSSYLDSPSYR	Mf	GH23305p (Mf2 protein) (Myofilin, isoform C) (Myofilin, isoform Q)	874.916	2	25.08	1194562	25.03	1397150	1.51935
ASRPYSSYLDSPSYR	Mf	GH23305p (Mf2 protein) (Myofilin, isoform C) (Myofilin, isoform Q)	583.613	3	25.12	5487893	25.03	8501748	2.01245
DKPLTPNSLVPLEYEPEDK	Mf	GH23305p (Mf2 protein) (Myofilin, isoform C) (Myofilin, isoform Q)	728.707	3	38.98	567161	38.81	728559	1.66871
DTYGYSPR	Mf	GH23305p (Mf2 protein) (Myofilin, isoform C) (Myofilin, isoform Q)	479.717	2	14.86	2640775	14.83	2840777	1.39742
FWQSYIR	Mf	GH23305p (Mf2 protein) (Myofilin, isoform C) (Myofilin, isoform Q)	500.256	2	32.37	739255	32.25	1687659	2.9656
LLAELNK	Mf	GH23305p (Mf2 protein) (Myofilin, isoform C) (Myofilin, isoform Q)	400.747	2	22.38	846200	22.35	1253332	1.92405
NHLEMIGR	Mf	GH23305p (Mf2 protein) (Myofilin, isoform C) (Myofilin, isoform Q)	493.248	2	12.87	1554021	12.91	1080925	0.90357
SIYDEPATANER	Mf	GH23305p (Mf2 protein) (Myofilin, isoform C) (Myofilin, isoform Q)	683.318	2	18.42	9216683	18.42	15046478	2.12072
YGLYLR	Mf	GH23305p (Mf2 protein) (Myofilin, isoform C) (Myofilin, isoform Q)	392.721	2	28.26	5499678	28.16	8903475	2.10303
GQIHDIVLVGGSTR	Hsc70-1	AT07372p (Heat shock protein cognate 1, isoform C) (Heat shock protein cog	726.402	2	27.66	141880	27.58	2013950	18.4395
GQIHDIVLVGGSTR	Hsc70-1	AT07372p (Heat shock protein cognate 1, isoform C) (Heat shock protein cog	484.604	3	27.43	42940	27.54	4848927	146.692
SINPDEAVAYGAAVQAAILHGDK	Hsc70-1	AT07372p (Heat shock protein cognate 1, isoform C) (Heat shock protein cog	770.729	3	49.12	5844145	48.87	6076260	1.35063
LSEASQAADESER	Tm1	SD21996p (Tropomyosin 1, isoform H)	696.816	2	11.57	343010	11.67	695696	2.63472
LSEASQAADESER	Tm1	RE08101p (Tropomyosin 1, isoform E)	696.816	2	11.57	343010	11.67	695696	2.63472
ESQNSVAIQGSFQK	CG32017	FI18814p1	761.878	2	21.85	406919	21.82	549063	1.75282
LGEFGSSASGLSHSR	CG32017	FI18814p1	497.911	3	21.34	530518	21.35	641053	1.5697
SIFFGSNR	CG32017	FI18814p1	464.238	2	29.33	812922	29.2	1231632	1.96813
TVGEVFVPK	CG32017	FI18814p1	488.279	2	27.47	636932	27.35	627150	1.27909
YYGAEMRPQTGASSIR	CG32017	FI18814p1	601.621	3	16.64	244775	16.57	232617	1.23452
RPATSGTGSVASSTAR	Map205	Microtubule-associated protein 205, isoform B	753.387	2	9.41	74073	9.52	32583	0.57142
AVIIYVPIPQQK	RpS7	40S ribosomal protein S7	684.916	2	41.28	8471675	41.1	6545686	1.00371
DLHITR	RpS7	40S ribosomal protein S7	377.714	2	14.71	21152176	14.72	16014808	0.98353
DVTFEFPDNYLNV	RpS7	40S ribosomal protein S7	786.864	2	53.99	23061740	53.69	18809548	1.05952
HVVVIAER	RpS7	40S ribosomal protein S7	461.777	2	15.49	20082808	15.46	11177676	0.72302
IIKPGGSDPDDFEK	RpS7	40S ribosomal protein S7	759.378	2	20.47	4039644	20.43	3343605	1.07521
IIKPGGSDPDDFEK	RpS7	40S ribosomal protein S7	506.588	3	20.47	25628294	20.43	25835260	1.30953
IQIILVR	RpS7	40S ribosomal protein S7	427.795	2	36.3	22111984	36.14	13910792	0.81723
SIAQALVELEANSDLKPYLR	RpS7	40S ribosomal protein S7	1115.61	2	52.69	3635581	52.52	3184821	1.13798
SIAQALVELEANSDLKPYLR	RpS7	40S ribosomal protein S7	744.074	3	52.73	38728332	52.56	37722616	1.26531
SIAQALVELEANSDLKPYLR	RpS7	40S ribosomal protein S7	558.307	4	52.69	4180332	52.56	3628128	1.12744
TLTAVYDAILEDLVFPAEIVGK	RpS7	40S ribosomal protein S7	1189.15	2	65.64	2919810	65.67	1234142	0.54908
TLTAVYDAILEDLVFPAEIVGK	RpS7	40S ribosomal protein S7	793.102	3	65.64	2850982	65.67	1260429	0.57431
VDTFTSVYK	RpS7	40S ribosomal protein S7	530.271	2	25.44	3847964	25.35	1682874	0.56812
HLPLSGTVGGGSSSSYLHK	Saf-B	Scaffold attachment factor B, isoform B	628.659	3	24.77	957648	24.64	1484691	2.01397
SLASQDRPR	Saf-B	Scaffold attachment factor B, isoform B	515.275	2	9.17	51712	9.24	185037	4.64825
STAAPGGATSSTASSAAGSK	Saf-B	Scaffold attachment factor B, isoform B	833.898	2	10.54	309614	10.69	137118	0.5753
TFIVQGFGNVGLHTTR	Gdh	Glutamate dehydrogenase	582.981	3	38.87	1874134	38.58	1474266	1.02187
GYAFVTFTNR	Syp	Syncrip, isoform A	588.296	2	35.44	514118	35.33	604713	1.52795
GYAFVTFTNR	Syp	Syncrip, isoform E	588.296	2	35.44	514118	35.33	604713	1.52795
AAVSGAVEQATLR	P5cr-2	Pyrroline-5-carboxylate reductase-like 2, isoform B (EC 1.5.1.2)	636.849	2	23.08	549773	23.1	334382	0.7901
AIYDHHYSR	Mf	Myofilin, isoform E	581.275	2	11.31	475566	11.41	651391	1.77932
AIYDHHYSR	Mf	Myofilin, isoform E	387.853	3	11.31	2895978	11.41	4746762	2.12924
ASRPYSSYLDSPSYR	Mf	Myofilin, isoform E	874.916	2	25.08	1194562	25.03	1397150	1.51935
ASRPYSSYLDSPSYR	Mf	Myofilin, isoform E	583.613	3	25.12	5487893	25.03	8501748	2.01245
DKPLTPNSLVPLEYEPEDK	Mf	Myofilin, isoform E	728.707	3	38.98	567161	38.81	728559	1.66871
DTYGYSPR	Mf	Myofilin, isoform E	479.717	2	14.86	2640775	14.83	2840777	1.39742
FWQSYIR	Mf	Myofilin, isoform E	500.256	2	32.37	739255	32.25	1687659	2.9656
LLAELNK	Mf	Myofilin, isoform E	400.747	2	22.38	846200	22.35	1253332	1.92405
NHLEMIGR	Mf	Myofilin, isoform E	493.248	2	12.87	1554021	12.91	1080925	0.90357
SIYDEPATANER	Mf	Myofilin, isoform E	683.318	2	18.42	9216683	18.42	15046478	2.12072

YGLYLR	Mf	Myofilin, isoform E	392.721	2	28.26	5499678	28.16	8903475	2.10303
HNILTVFPNR	CG10237	MIP27352p	404.228	3	27.09	502708	26.54	832902	2.15229
LLEAETDLLYPK	CG10237	MIP27352p	702.885	2	39.58	82213	39.35	456931	7.21992
GANAPEDLTLR	Mtpalpha	Mitochondrial trifunctional protein alpha subunit, isoform B (EC 1.1.1.211) (EC	578.801	2	24.55	462048	24.52	491502	1.38185
LGIVDLLVDPLGPGLQPAEQNTIEYL	Mtpalpha	Mitochondrial trifunctional protein alpha subunit, isoform B (EC 1.1.1.211) (EC	1012.22	3	65.71	94515	65.42	318192	4.37332
TAVQVANDLASGK	Mtpalpha	Mitochondrial trifunctional protein alpha subunit, isoform B (EC 1.1.1.211) (EC	637.341	2	24.73	1414991	24.64	1190450	1.0929
VNSLGSEVSDEFER	Mtpalpha	Mitochondrial trifunctional protein alpha subunit, isoform B (EC 1.1.1.211) (EC	784.365	2	31.55	326081	31.42	504128	2.00834
FVFDVFEFAQK	mmy	Mummy, isoform B (EC 2.7.7.23) (Mummy, isoform D)	688.848	2	54.52	25125	54.13	69150	3.57527
TALTHYLEITAIPR	Cpr	Cytochrome P450 reductase, isoform B (EC 1.-.-.-) (EC 1.6.2.4)	533.635	3	40.81	57301	40.62	150767	3.41796
VFSVHPVFR	pst	GH06117p (Pastrel, isoform F)	363.206	3	30.19	12544	30.2	609080	63.0755
VSQPIPGSHAGSGR	pst	GH06117p (Pastrel, isoform F)	675.35	2	15.96	162655	12.19	118004	0.94244
VFSVHPVFR	pst	Pastrel, isoform E (Pastrel, isoform G)	363.206	3	30.19	12544	30.2	609080	63.0755
VSQPIPGSHAGSGR	pst	Pastrel, isoform E (Pastrel, isoform G)	675.35	2	15.96	162655	12.19	118004	0.94244
AASPASSVSSLSR	Gug	Grunge, isoform A (EC 3.5.1.98)	610.317	2	19.87	670193	19.91	1047675	2.03072
ALQALVK	Gug	Grunge, isoform A (EC 3.5.1.98)	371.745	2	22.24	1429410	22.24	802676	0.72947
ASPSVQISSSGGGPSGPGSVGPGQ	Gug	Grunge, isoform A (EC 3.5.1.98)	862.663	4	27.81	128265	27.69	91817	0.9299
MSYADQLQAAEFQR	Gug	Grunge, isoform A (EC 3.5.1.98)	829.386	2	32.37	172984	32.33	256318	1.92484
MYAPQPGQR	Gug	Grunge, isoform A (EC 3.5.1.98)	532.253	2	11.22	597570	11.23	396368	0.86165
QHQAAAAAAAQQHK	Gug	Grunge, isoform A (EC 3.5.1.98)	477.58	3	7.39	102108	7.46	61942	0.78804
QSLHDQYFR	Gug	Grunge, isoform A (EC 3.5.1.98)	398.528	3	18.86	599467	18.89	746793	1.61829
SQSAIFVR	Gug	Grunge, isoform A (EC 3.5.1.98)	454.253	2	22.57	2827859	22.59	2397174	1.10119
WSPGVVADGDLLMFLR	Gug	Grunge, isoform A (EC 3.5.1.98)	888.461	2	59.21	701351	58.95	433086	0.80216
WSPGVVADGDLLMFLR	Gug	Grunge, isoform A (EC 3.5.1.98)	896.459	2	56.45	1934292	56.29	935150	0.62803
YGDPLAAK	Gug	Grunge, isoform A (EC 3.5.1.98)	417.721	2	19.02	1065996	19	938830	1.14407
STVPFATSESNR	Zasp66	Z band alternatively spliced PDZ-motif protein 66, isoform E	648.315	2	21.89	228788	21.86	230532	1.30894
AIGAGGQSTQVR	Sap130	Sin3A-associated protein 130, isoform B (Sin3A-associated protein 130, isofo	572.807	2	12.25	925618	12.32	536264	0.75261
GPGAGGTALTATNLPTTR	Sap130	Sin3A-associated protein 130, isoform B (Sin3A-associated protein 130, isofo	828.439	2	27.36	569694	27.23	266622	0.60796
IGDQPATGGAGGR	Sap130	Sin3A-associated protein 130, isoform B (Sin3A-associated protein 130, isofo	578.789	2	10.2	451645	10.33	79684	0.22919
IIQLQQPATGTTQQIIGSGAR	Sap130	Sin3A-associated protein 130, isoform B (Sin3A-associated protein 130, isofo	727.738	3	34.41	1767358	34.26	2314474	1.70118
QLENVTATGR	Sap130	Sin3A-associated protein 130, isoform B (Sin3A-associated protein 130, isofo	544.788	2	15.37	399235	15.31	452872	1.47356
SDGGGGISIAGTPILSGGTIK	Sap130	Sin3A-associated protein 130, isoform B (Sin3A-associated protein 130, isofo	929.499	2	41.44	945778	41.22	943168	1.29545
TQITAIPAAPAR	Sap130	Sin3A-associated protein 130, isoform B (Sin3A-associated protein 130, isofo	605.351	2	25.4	3213530	25.35	3375310	1.36444
VQPSLTITQLNPIGK	Sap130	Sin3A-associated protein 130, isoform B (Sin3A-associated protein 130, isofo	804.97	2	41.17	1625853	40.98	1635254	1.30655
VTHGVASSAASVPATVGPVNSTR	Sap130	Sin3A-associated protein 130, isoform B (Sin3A-associated protein 130, isofo	722.382	3	23.81	505024	23.8	708422	1.82223
VTTGAGNPQQATASGALVSSFMR	Sap130	Sin3A-associated protein 130, isoform B (Sin3A-associated protein 130, isofo	756.374	3	32.37	491161	32.25	670045	1.77216
AIGAGGQSTQVR	Sap130	Sin3A-associated protein 130, isoform C	572.807	2	12.25	925618	12.32	536264	0.75261
GPGAGGTALTATNLPTTR	Sap130	Sin3A-associated protein 130, isoform C	828.439	2	27.36	569694	27.23	266622	0.60796
IGDQPATGGAGGR	Sap130	Sin3A-associated protein 130, isoform C	578.789	2	10.2	451645	10.33	79684	0.22919
IIQLQQPATGTTQQIIGSGAR	Sap130	Sin3A-associated protein 130, isoform C	727.738	3	34.41	1767358	34.26	2314474	1.70118
QLENVTATGR	Sap130	Sin3A-associated protein 130, isoform C	544.788	2	15.37	399235	15.31	452872	1.47356
SDGGGGISIAGTPILSGGTIK	Sap130	Sin3A-associated protein 130, isoform C	929.499	2	41.44	945778	41.22	943168	1.29545
TQITAIPAAPAR	Sap130	Sin3A-associated protein 130, isoform C	605.351	2	25.4	3213530	25.35	3375310	1.36444
VQPSLTITQLNPIGK	Sap130	Sin3A-associated protein 130, isoform C	804.97	2	41.17	1625853	40.98	1635254	1.30655
VTHGVASSAASVPATVGPVNSTR	Sap130	Sin3A-associated protein 130, isoform C	722.382	3	23.81	505024	23.8	708422	1.82223
VTTGAGNPQQATASGALVSSFMR	Sap130	Sin3A-associated protein 130, isoform C	756.374	3	32.37	491161	32.25	670045	1.77216
DGVLTVDAPLPALTAGETLIPIAHK	HspB8	RE23625p (RE52196p)	838.139	3	55.9	27215	55.42	322725	15.4045
GVNPESIR	HspB8	RE23625p (RE52196p)	436.235	2	16.4	162532	16.45	263810	2.10851
STSISNVAGASLTR	ens	Ensconsin, isoform D	682.362	2	25.86	816429	25.79	694557	1.10513
STSISNVAGASLTR	ens	Ensconsin, isoform E (Ensconsin, isoform L) (LP21080p)	682.362	2	25.86	816429	25.79	694557	1.10513
STSISNVAGASLTR	ens	Ensconsin, isoform C	682.362	2	25.86	816429	25.79	694557	1.10513
YYLGIFFK	tfc	LP02252p (Triforce, isoform B)	525.787	2	48.48	464111	48.22	385306	1.07847
SIFSNFPQQVK	prom	Prominin, isoform C	647.843	2	36.49	1196743	36.37	1335180	1.44931
TLQEDQSIFR	prom	Prominin, isoform C	618.815	2	28.08	1390520	28.05	1856704	1.73455

VALQIQDVATSSR	prom	Prominin, isoform C	694.381	2	29.91	3286792	29.77	3985799	1.57531
LAVNMVPFPR	betaTub9	Tubulin beta chain	572.321	2	40.41	1793279	40.19	932227	0.6753
LAVNMVPFPR	betaTub9	Tubulin beta chain	580.318	2	34.29	768535	34.41	1250344	2.11343
NSSYFVEWIPNNVK	betaTub9	Tubulin beta chain	848.92	2	44.43	6003407	44.1	632379	0.13684
HHDIDVVLIR	CG5028	RH49423p	406.231	3	27.73	408874	27.61	208947	0.66385
FAPAPYFF	BcDNA:R	RE26528p	480.237	2	51.83	770165	51.51	848406	1.43101
FGFGPFAAPLAAPVPAPLAVAPK	BcDNA:R	RE26528p	736.086	3	58.91	2617017	58.71	3247207	1.61185
VAFAAPAPVAAPLGFAPAPAPVAFA	BcDNA:R	RE26528p	886.832	3	53.11	2925984	52.83	3692163	1.6392
MQGLGLQSLK	ND-MLRQ	GEO09626p1 (NADH dehydrogenase (Ubiquinone) MLRQ subunit, isoform A	545.8	2	28.03	3087470	28	3232792	1.36018
NPALIPLYVCVGAGAIGAVYYMAR	ND-MLRQ	GEO09626p1 (NADH dehydrogenase (Ubiquinone) MLRQ subunit, isoform A	847.112	3	63.46	114000	63.09	237220	2.70314
NPDVTWNR	ND-MLRQ	GEO09626p1 (NADH dehydrogenase (Ubiquinone) MLRQ subunit, isoform A	501.244	2	19.41	1214944	19.37	904787	0.96741
TSNPEPWQEYK	ND-MLRQ	GEO09626p1 (NADH dehydrogenase (Ubiquinone) MLRQ subunit, isoform A	689.817	2	25.23	750152	25.18	899126	1.55702
ELEIEQR	eIF4B	Eukaryotic initiation factor 4B (Eukaryotic translation initiation factor 4B, isofor	458.74	2	17.88	4591316	17.9	2801196	0.79255
QGGTSSLNVFGSAKPVDTAAR	eIF4B	Eukaryotic initiation factor 4B (Eukaryotic translation initiation factor 4B, isofor	688.355	3	30.18	747833	30.12	321395	0.55829
HSITSGGSGGGGFR	Rsf1	RE39606p (Repressor splicing factor 1, isoform B)	638.805	2	12.65	1063621	12.67	1034087	1.26297
HSITSGGSGGGGFR	Rsf1	RE39606p (Repressor splicing factor 1, isoform B)	426.206	3	12.65	1459305	12.67	1825302	1.62484
VYVGNLTDK	Rsf1	RE39606p (Repressor splicing factor 1, isoform B)	504.772	2	21.41	1430452	21.39	1257685	1.14214
YSSGSSASYGR	Rsf1	RE39606p (Repressor splicing factor 1, isoform B)	561.247	2	9.59	527731	9.71	255024	0.62776
TLVYVLVK	TwdlE	RE71854p (TweedleE)	467.802	2	36.56	5020293	36.32	6917808	1.79004
EAVGAFIVFDVTR	Rab32	LD04613p (Rab32, isoform C) (Rab32, isoform E)	712.383	2	49.81	1401007	49.56	1390509	1.28931
ALYSNEINQK	Mpp6	GEO07784p1 (M phase phosphoprotein 6) (M-phase phosphoprotein 6, isfor	590.304	2	18.79	615618	18.77	286252	0.60403
ATSPDSSDVIIAR	Cpr49Ae	Cuticular protein 49Ae (GEO04354p1) (LD46766p)	666.344	2	23.06	746167	23.1	1286669	2.24003
IIGEYGLR	RpS9	GEO04282p1 (LD32106p) (Ribosomal protein S9, isoform B)	460.764	2	25.97	1566590	25.94	2123615	1.76093
LFQGNALLR	RpS9	GEO04282p1 (LD32106p) (Ribosomal protein S9, isoform B)	516.303	2	32.83	1000199	32.72	375882	0.48819
LTTSSHLTQFGTVENANTPGATAR	G9a	G9a, isoform A (EC 2.1.1.43) (LD10743p)	825.414	3	27.32	294736	27.19	352075	1.55176
LSEASQAADESER	Tm1	GH09289p (LD11194p) (Tropomyosin 1, isoform C) (Tropomyosin 1, isoform I	696.816	2	11.57	343010	11.67	695696	2.63472
LITPVVLQR	RpS6	40S ribosomal protein S6	519.837	2	34.36	2782314	34.22	2911970	1.35957
GYAFVTFTNR	Syp	GH28335p (Syncrip, isoform C)	588.296	2	35.44	514118	35.33	604713	1.52795
STVPFATSESNR	Zasp66	GH19182p (Z band alternatively spliced PDZ-motif protein 66, isoform F)	648.315	2	21.89	228788	21.86	230532	1.30894
AGSEPYVPPPSYWNR	Mf	GH14252p (Mf3 protein) (Myofilin, isoform D) (RH68506p)	860.41	2	33.23	748487	33.06	1566838	2.71933
AIYDHHYSR	Mf	GH14252p (Mf3 protein) (Myofilin, isoform D) (RH68506p)	581.275	2	11.31	475566	11.41	651391	1.77932
AIYDHHYSR	Mf	GH14252p (Mf3 protein) (Myofilin, isoform D) (RH68506p)	387.853	3	11.31	2895978	11.41	4746762	2.12924
ASRPYSSYLDSPSYR	Mf	GH14252p (Mf3 protein) (Myofilin, isoform D) (RH68506p)	874.916	2	25.08	1194562	25.03	1397150	1.51935
ASRPYSSYLDSPSYR	Mf	GH14252p (Mf3 protein) (Myofilin, isoform D) (RH68506p)	583.613	3	25.12	5487893	25.03	8501748	2.01245
ASSLEPLDELFER	Mf	GH14252p (Mf3 protein) (Myofilin, isoform D) (RH68506p)	753.378	2	48.45	4498040	48.19	5102070	1.47348
DKPLTPNSLVPLEYEPEDK	Mf	GH14252p (Mf3 protein) (Myofilin, isoform D) (RH68506p)	728.707	3	38.98	567161	38.81	728559	1.66871
DSYLSPVK	Mf	GH14252p (Mf3 protein) (Myofilin, isoform D) (RH68506p)	454.74	2	22.04	1523720	22.02	1430945	1.21994
DTYGYSPR	Mf	GH14252p (Mf3 protein) (Myofilin, isoform D) (RH68506p)	479.717	2	14.86	2640775	14.83	2840777	1.39742
FWQSYIR	Mf	GH14252p (Mf3 protein) (Myofilin, isoform D) (RH68506p)	500.256	2	32.37	739255	32.25	1687659	2.9656
GGPSVFDR	Mf	GH14252p (Mf3 protein) (Myofilin, isoform D) (RH68506p)	417.709	2	19.86	5727839	19.85	7157511	1.62328
LLAELNK	Mf	GH14252p (Mf3 protein) (Myofilin, isoform D) (RH68506p)	400.747	2	22.38	846200	22.35	1253332	1.92405
LPIFHPR	Mf	GH14252p (Mf3 protein) (Myofilin, isoform D) (RH68506p)	440.264	2	24.47	512417	24.48	315832	0.80067
NHLEMIGR	Mf	GH14252p (Mf3 protein) (Myofilin, isoform D) (RH68506p)	493.248	2	12.87	1554021	12.91	1080925	0.90357
SASPFRPAR	Mf	GH14252p (Mf3 protein) (Myofilin, isoform D) (RH68506p)	494.77	2	13.46	1272464	13.42	1443660	1.47381
SIYDEPATANER	Mf	GH14252p (Mf3 protein) (Myofilin, isoform D) (RH68506p)	683.318	2	18.42	9216683	18.42	15046478	2.12072
YGLYLR	Mf	GH14252p (Mf3 protein) (Myofilin, isoform D) (RH68506p)	392.721	2	28.26	5499678	28.16	8903475	2.10303
VVYDPQTAEELSSK	ScsbetaA	Succinate--CoA ligase [ADP-forming] subunit beta, mitochondrial (EC 6.2.1.5)	783.388	2	26.05	1092441	25.98	763625	0.90804
ANAVAAPFR	simj	LD33361p (Simjang, isoform C)	458.754	2	21.85	720615	21.82	738030	1.33043
GSLSVTPTSAVPLPAHSK	simj	LD33361p (Simjang, isoform C)	583.656	3	30.14	968499	29.97	1592607	2.13615
SSLPAGATLAAGTTITPATSSSHR	simj	LD33361p (Simjang, isoform C)	752.392	3	30.52	3143778	30.4	4148506	1.7142
SSLSGSGGPAVSISATNSR	simj	LD33361p (Simjang, isoform C)	867.934	2	24.08	1895042	24.04	2008027	1.37649
TNLPNLTITPSVTITPTSAPPSSLKPI	simj	LD33361p (Simjang, isoform C)	935.19	3	42.52	2161882	42.25	1939128	1.16519
ILFAFGPVVEPYTTEHTVVAK	alpha-Est	Carboxylic ester hydrolase (EC 3.1.1.-)	773.419	3	44.7	30930	48.23	75260	3.16087

HNILTVFPNR	CG10237	Uncharacterized protein, isoform A (Uncharacterized protein, isoform D)	404.228	3	27.09	502708	26.54	832902	2.15229
LLEAETDLLYPK	CG10237	Uncharacterized protein, isoform A (Uncharacterized protein, isoform D)	702.885	2	39.58	82213	39.35	456931	7.21992
STSISNVAGASLTR	ens	Ensconsin, isoform A (LD09626p)	682.362	2	25.86	816429	25.79	694557	1.10513
SAGLTPDLSNFTETR	sbb	Brakeless-B (Scribbler long isoform) (Scribbler, isoform D) (Scribbler, isoform	804.897	2	36.67	741872	36.56	971672	1.70143
TSPGFGSVGVGAASSK	sbb	Brakeless-B (Scribbler long isoform) (Scribbler, isoform D) (Scribbler, isoform	704.857	2	24.77	1117813	24.76	1320748	1.53488
GANAPEDLTLR	Mtpalpha	BcDNA.GH12558 (Mitochondrial trifunctional protein alpha subunit, isoform A)	578.801	2	24.55	462048	24.52	491502	1.38185
LGIVDLLVDPLGPGLQPAEQNTIEYL	Mtpalpha	BcDNA.GH12558 (Mitochondrial trifunctional protein alpha subunit, isoform A)	1012.22	3	65.71	94515	65.42	318192	4.37332
TAVQVANDLASGK	Mtpalpha	BcDNA.GH12558 (Mitochondrial trifunctional protein alpha subunit, isoform A)	637.341	2	24.73	1414991	24.64	1190450	1.0929
VNSLGSEVSDEFER	Mtpalpha	BcDNA.GH12558 (Mitochondrial trifunctional protein alpha subunit, isoform A)	784.365	2	31.55	326081	31.42	504128	2.00834
LIVGNLDYGVSNTDIK	Ref1	LD24793p (RNA and export factor binding protein 1)	860.959	2	37.95	1201336	37.75	1078970	1.16672
RPVGGKPAAGGQR	Ref1	LD24793p (RNA and export factor binding protein 1)	625.857	2	7.23	50720	7.22	20708	0.53037
RPVGGKPAAGGQR	Ref1	LD24793p (RNA and export factor binding protein 1)	417.574	3	7.23	135020	7.2	45108	0.43399
SLGTADVIFER	Ref1	LD24793p (RNA and export factor binding protein 1)	604.32	2	35.16	5397913	34.98	6047044	1.45526
LNELLATPIFNER	REG	LD45860p (Proteasome regulator dREG) (REG, isoform A) (REG, isoform B)	765.42	2	44.66	196314	44.39	478969	3.16941
VPVPQPYEVIR	Vajk3	HL02234p (RH05666p) (Vajk3)	648.869	2	34.29	1028304	34.15	1020137	1.28872
VPVVSHSVVK	Vajk3	HL02234p (RH05666p) (Vajk3)	525.819	2	16.6	543779	16.57	824515	1.96969
VPVVSHSVVK	Vajk3	HL02234p (RH05666p) (Vajk3)	350.882	3	16.6	274491	16.57	456956	2.16256
ACHDSGEELDDISLPK	MBD-like	Methyl-CpG binding domain protein-like, isoform B (Methyl-CpG binding doma	893.402	2	30.72	859129	30.56	663495	1.00323
ACHDSGEELDDISLPK	MBD-like	Methyl-CpG binding domain protein-like, isoform B (Methyl-CpG binding doma	595.937	3	30.68	4227121	30.52	3334801	1.02482
TDVSLVPPIR	MBD-like	Methyl-CpG binding domain protein-like, isoform B (Methyl-CpG binding doma	548.822	2	35.16	14387869	35.02	9195601	0.83024
TVGPNVNEQTVLQSVATALHMLNA	MBD-like	Methyl-CpG binding domain protein-like, isoform B (Methyl-CpG binding doma	847.937	4	61.34	1644970	61.29	1228563	0.9702
TVGPNVNEQTVLQSVATALHMLNA	MBD-like	Methyl-CpG binding domain protein-like, isoform B (Methyl-CpG binding doma	851.936	4	55.74	1113659	55.85	795373	0.92777
GGPRPGGFGGGPR	vig	LD07162p (Vasa intronic gene, isoform A) (Vasa intronic gene, isoform B) (Va	390.204	3	13.98	2034032	13.94	2758189	1.76152
VLDIQFNFNDGR	vig	LD07162p (Vasa intronic gene, isoform A) (Vasa intronic gene, isoform B) (Va	719.36	2	42.3	932723	42.11	1042745	1.45227
LFLFVSQHTF	CT7856	RE68558p	619.832	2	48.93	1575091	48.71	1713018	1.41279
NFPALTYFR	l(2)01289	L(2)01289 long form (Lethal (2) 01289, isoform B)	564.795	2	42.88	433584	42.79	472450	1.41548
AHVHYVVTEHGIASLFGK	anon-WO	GM14349p	492.014	4	35.01	514891	35.22	487169	1.2291
FYSGFGGQVDFIR	anon-WO	GM14349p	746.865	2	42.05	2238795	41.9	2390076	1.38682
IVGSFLIGDK	anon-WO	GM14349p	524.805	2	36.77	1627498	36.56	1359424	1.08507
LIVAQINPK	anon-WO	GM14349p	498.316	2	27.09	1231167	26.93	998622	1.05367
SGDTVFAGGAAATPVALLNAMAK	anon-WO	GM14349p	711.705	3	54.28	110880	53.97	130931	1.53395
SHFDLAIEVTDDLPQHGTGK	anon-WO	GM14349p	727.359	3	37.31	553164	37.15	209091	0.49103
LAVNMVPFPR	betaTub9	Tubulin beta chain	572.321	2	40.41	1793279	40.19	932227	0.6753
LAVNMVPFPR	betaTub9	Tubulin beta chain	580.318	2	34.29	768535	34.41	1250344	2.11343
NSSYFVEWIPNNVK	betaTub9	Tubulin beta chain	848.92	2	44.43	6003407	44.1	632379	0.13684
SVVAVFAQGTEQR	FBXL8	FI05230p	696.368	2	30.76	1164648	30.64	1327554	1.48074
VQVGHAPSPNLK	tau	Microtubule-associated protein	416.235	3	17.44	993769	17.41	1404801	1.83633
VQVGHAPSPNLK	tau	Microtubule-associated protein	416.235	3	17.44	993769	17.41	1404801	1.83633
SLSAIQEQLQQR	l(3)mbt	LD05287p (Lethal (3) malignant brain tumor)	700.878	2	29.29	410006	29.17	437411	1.38587
TLVYVLVK	TwdlT	LP07342p (TweedleT)	467.802	2	36.56	5020293	36.32	6917808	1.79004
YTAHDISFEK	RpL27	60S ribosomal protein L27	404.196	3	20.71	543502	20.72	879409	2.1019
DLLHPLPAEEK	RpS27	40S ribosomal protein S27	421.231	3	30.37	2013778	30.24	3941259	2.54241
DLGGQSTTQDFTR	Idh3b	GH26270p (Isocitrate dehydrogenase 3b, isoform A) (EC 1.1.1.41) (Isocitrate	713.334	2	21.19	367303	21.19	267232	0.94512
GYAFVTFTNR	Syp	Syncrip, isoform D	588.296	2	35.44	514118	35.33	604713	1.52795
VVVQNHIEENLNR	CG6013	GH10002p	521.946	3	20.7	653310	20.72	528670	1.05121
LTSLTEDVAAVPEDAPFR	Wdr37	GH17724p	965.991	2	42.05	480078	41.83	526368	1.4243
AAVSGAVEQATLR	P5cr-2	Pyrroline-5-carboxylate reductase (EC 1.5.1.2)	636.849	2	23.08	549773	23.1	334382	0.7901
GLLLFVDEADAFLR	bor	ATPase family AAA domain-containing protein 3A homolog (Belphegor protei	789.93	2	61.99	401366	61.79	319540	1.03421
HSEQPAGVIVALTADQAHR	AOX3	Aldehyde oxidase 3, isoform A (EC 1.17.1.4) (EC 1.2.3.1) (Aldehyde oxidase	667.348	3	33.16	29495	33.17	355033	15.6366
AIYDHHYSR	Mf	MIP06926p (Myofilin, isoform A) (Myofilin, isoform G) (Myofilin, isoform N)	581.275	2	11.31	475566	11.41	651391	1.77932
AIYDHHYSR	Mf	MIP06926p (Myofilin, isoform A) (Myofilin, isoform G) (Myofilin, isoform N)	387.853	3	11.31	2895978	11.41	4746762	2.12924
ASRPYSSYLDSPSYR	Mf	MIP06926p (Myofilin, isoform A) (Myofilin, isoform G) (Myofilin, isoform N)	874.916	2	25.08	1194562	25.03	1397150	1.51935
ASRPYSSYLDSPSYR	Mf	MIP06926p (Myofilin, isoform A) (Myofilin, isoform G) (Myofilin, isoform N)	583.613	3	25.12	5487893	25.03	8501748	2.01245

DKPLTPNSLVPLEYEPEDK	Mf	MIP06926p (Myofilin, isoform A) (Myofilin, isoform G) (Myofilin, isoform N)	728.707	3	38.98	567161	38.81	728559	1.66871
DTYGYSPR	Mf	MIP06926p (Myofilin, isoform A) (Myofilin, isoform G) (Myofilin, isoform N)	479.717	2	14.86	2640775	14.83	2840777	1.39742
FWQSYIR	Mf	MIP06926p (Myofilin, isoform A) (Myofilin, isoform G) (Myofilin, isoform N)	500.256	2	32.37	739255	32.25	1687659	2.9656
LLAELNK	Mf	MIP06926p (Myofilin, isoform A) (Myofilin, isoform G) (Myofilin, isoform N)	400.747	2	22.38	846200	22.35	1253332	1.92405
NHLEMIGR	Mf	MIP06926p (Myofilin, isoform A) (Myofilin, isoform G) (Myofilin, isoform N)	493.248	2	12.87	1554021	12.91	1080925	0.90357
SIYDEPATANER	Mf	MIP06926p (Myofilin, isoform A) (Myofilin, isoform G) (Myofilin, isoform N)	683.318	2	18.42	9216683	18.42	15046478	2.12072
YGLYLR	Mf	MIP06926p (Myofilin, isoform A) (Myofilin, isoform G) (Myofilin, isoform N)	392.721	2	28.26	5499678	28.16	8903475	2.10303
AGSEPYVPPPSYWNR	Mf	Mf5 protein (Myofilin, isoform B) (RE24409p)	860.41	2	33.23	748487	33.06	1566838	2.71933
AIYDHHYSR	Mf	Mf5 protein (Myofilin, isoform B) (RE24409p)	581.275	2	11.31	475566	11.41	651391	1.77932
AIYDHHYSR	Mf	Mf5 protein (Myofilin, isoform B) (RE24409p)	387.853	3	11.31	2895978	11.41	4746762	2.12924
ASRPYSSYLDSPSYR	Mf	Mf5 protein (Myofilin, isoform B) (RE24409p)	874.916	2	25.08	1194562	25.03	1397150	1.51935
ASRPYSSYLDSPSYR	Mf	Mf5 protein (Myofilin, isoform B) (RE24409p)	583.613	3	25.12	5487893	25.03	8501748	2.01245
ASSLEPLDELFER	Mf	Mf5 protein (Myofilin, isoform B) (RE24409p)	753.378	2	48.45	4498040	48.19	5102070	1.47348
DKPLTPNSLVPLEYEPEDK	Mf	Mf5 protein (Myofilin, isoform B) (RE24409p)	728.707	3	38.98	567161	38.81	728559	1.66871
DSYLSPVK	Mf	Mf5 protein (Myofilin, isoform B) (RE24409p)	454.74	2	22.04	1523720	22.02	1430945	1.21994
DTYGYSPR	Mf	Mf5 protein (Myofilin, isoform B) (RE24409p)	479.717	2	14.86	2640775	14.83	2840777	1.39742
FWQSYIR	Mf	Mf5 protein (Myofilin, isoform B) (RE24409p)	500.256	2	32.37	739255	32.25	1687659	2.9656
GGPSVFDR	Mf	Mf5 protein (Myofilin, isoform B) (RE24409p)	417.709	2	19.86	5727839	19.85	7157511	1.62328
LLAELNK	Mf	Mf5 protein (Myofilin, isoform B) (RE24409p)	400.747	2	22.38	846200	22.35	1253332	1.92405
NHLEMIGR	Mf	Mf5 protein (Myofilin, isoform B) (RE24409p)	493.248	2	12.87	1554021	12.91	1080925	0.90357
SASPFRPAR	Mf	Mf5 protein (Myofilin, isoform B) (RE24409p)	494.77	2	13.46	1272464	13.42	1443660	1.47381
SIYDEPATANER	Mf	Mf5 protein (Myofilin, isoform B) (RE24409p)	683.318	2	18.42	9216683	18.42	15046478	2.12072
WGPRPTEVAYDAEGLPIFHPR	Mf	Mf5 protein (Myofilin, isoform B) (RE24409p)	602.809	4	43.06	371242	42.9	651591	2.28003
YGLYLR	Mf	Mf5 protein (Myofilin, isoform B) (RE24409p)	392.721	2	28.26	5499678	28.16	8903475	2.10303
GVTYDTGGADIK	Dip-B	Dipeptidase B, isoform A (Dipeptidase B, isoform B) (Dipeptidase B, isoform C	598.793	2	18.83	55238	18.98	102822	2.41808
FFGAGFGGSGGGR	Droj2	DnaJ-like-2, isoform A (DnaJ-like-2, isoform B) (DnaJ-like-2, isoform C) (DnaJ	587.275	2	30.15	714105	30.2	935327	1.70147
LLEANPNYSK	mfas	GH11519p (Midline fasciclin, isoform B) (Midline fasciclin, isoform D) (Midline	574.801	2	18.6	422357	18.57	399268	1.22802
LNLYSTHALFSDVMNR	mfas	GH11519p (Midline fasciclin, isoform B) (Midline fasciclin, isoform D) (Midline	632.981	3	38.73	372398	38.45	772695	2.6954
LLEANPNYSK	mfas	Midline fasciclin, isoform C (Midline fasciclin, isoform I) (Midline fasciclin, isofc	574.801	2	18.6	422357	18.57	399268	1.22802
LNLYSTHALFSDVMNR	mfas	Midline fasciclin, isoform C (Midline fasciclin, isoform I) (Midline fasciclin, isofc	632.981	3	38.73	372398	38.45	772695	2.6954
GSGVNILLITPEK	GC1	Glutamate carrier 1, isoform A (Glutamate carrier 1, isoform B) (SD15982p)	670.893	2	40.94	753169	40.74	667861	1.1519
VADFQIIDR	mRpL40	Mitochondrial ribosomal protein L40 (RE65766p)	538.79	2	31.75	1471897	31.63	1067453	0.94209
AAAPAAAPAPAAPK	alpha-KG	GM01350p (LP03989p)	587.833	2	15.61	635681	15.6	267047	0.54572
AAVENPAIIVAGL	alpha-KG	GM01350p (LP03989p)	619.361	2	50.09	768844	49.84	619275	1.04633
EAVLFLR	alpha-KG	GM01350p (LP03989p)	424.255	2	36.05	962959	35.86	525379	0.70874
AAIELNR	DHC3B	Uncharacterized protein, isoform E (EC 3.6.1.3) (EC 3.6.4.5)	393.727	2	17.87	27934492	17.87	8147882	0.3789
ACHDSGEELDDISLPK	MBD-like	LD22928p (Methyl-CpG binding domain protein-like, isoform A)	893.402	2	30.72	859129	30.56	663495	1.00323
ACHDSGEELDDISLPK	MBD-like	LD22928p (Methyl-CpG binding domain protein-like, isoform A)	595.937	3	30.68	4227121	30.52	3334801	1.02482
TDVSLVPPIR	MBD-like	LD22928p (Methyl-CpG binding domain protein-like, isoform A)	548.822	2	35.16	14387869	35.02	9195601	0.83024
TVGPNVNEQTVLQSVATALHMLNA	MBD-like	LD22928p (Methyl-CpG binding domain protein-like, isoform A)	847.937	4	61.34	1644970	61.29	1228563	0.9702
TVGPNVNEQTVLQSVATALHMLNA	MBD-like	LD22928p (Methyl-CpG binding domain protein-like, isoform A)	851.936	4	55.74	1113659	55.85	795373	0.92777
TVIIPSDVQVVSTK	CG16779	Uncharacterized protein, isoform A (Uncharacterized protein, isoform B) (Uncl	743.43	2	34.9	984275	34.74	541335	0.71445
VVYDPQTAEELSSK	ScsbetaA	Succinate--CoA ligase [ADP-forming] subunit beta, mitochondrial (EC 6.2.1.5)	783.388	2	26.05	1092441	25.98	763625	0.90804
HNILTVFPNR	CG10237	LD41874p	404.228	3	27.09	502708	26.54	832902	2.15229
LLEAETDLLYPK	CG10237	LD41874p	702.885	2	39.58	82213	39.35	456931	7.21992
LVLIASNTPALR	RpL30	RE25263p (Ribosomal protein L30, isoform B) (Ribosomal protein L30, isoforr	634.39	2	36.53	13305670	36.36	9153605	0.89367
TEVQHYSGTNIELGTACGK	RpL30	RE25263p (Ribosomal protein L30, isoform B) (Ribosomal protein L30, isoforr	688.993	3	23.73	1580231	23.72	1103695	0.9073
VCTLSITDPGDSDIIR	RpL30	RE25263p (Ribosomal protein L30, isoform B) (Ribosomal protein L30, isoforr	881.438	2	37.12	7196177	36.91	5238982	0.94573
YCLGYK	RpL30	RE25263p (Ribosomal protein L30, isoform B) (Ribosomal protein L30, isoforr	402.191	2	19.17	631184	19.17	219376	0.4515
INDTLYDLSDFAAR	CG17928	IP12339p	807.394	2	43.06	30666	45.24	52905	2.2411
AFADALEVIPSTLAENAGLNPIATVT	CCT4	T-complex protein 1 subunit delta	999.536	3	65.21	795472	65	511753	0.83571
AATQEIAANVLR	Dlg5	Discs large 5, isoform A (Discs large 5, isoform B) (LD32687p)	628.852	2	39.24	4025067	39.04	5041733	1.62716
SWYTVMGAGAR	CG6089	AT01533p	607.785	2	25.16	570186	25.18	436913	0.99541

SWYTVMGAGAR	CG6087	RH51633p	607.785	2	25.16	570186	25.18	436913	0.99541
DAQIQNVQEGIELGAYEQIPGNQR	CG17127	Uncharacterized protein, isoform A (Uncharacterized protein, isoform B)	890.775	3	39.99	5948109	39.77	10842609	2.36798
EGAAYTNEAIR	CG17127	Uncharacterized protein, isoform A (Uncharacterized protein, isoform B)	597.791	2	17.24	14578124	17.25	20886826	1.8612
QAQQTLLIPK	CG17127	Uncharacterized protein, isoform A (Uncharacterized protein, isoform B)	570.343	2	27.84	8634126	27.76	16636826	2.50308
AGTVWVNTYNVLAAQAPFGGYK	Aldh	Aldehyde dehydrogenase (Aldehyde dehydrogenase, isoform A) (EC 1.2.1.10	776.399	3	50.78	476997	50.55	100631	0.27406
ANNSEYGLAAAVFTK	Aldh	Aldehyde dehydrogenase (Aldehyde dehydrogenase, isoform A) (EC 1.2.1.10	778.391	2	38.76	362378	38.5	599018	2.14734
ANYIVGGLR	Aldh	Aldehyde dehydrogenase (Aldehyde dehydrogenase, isoform A) (EC 1.2.1.10	481.775	2	27.31	644317	27.19	494411	0.99681
IYDEFVER	Aldh	Aldehyde dehydrogenase (Aldehyde dehydrogenase, isoform A) (EC 1.2.1.10	535.761	2	26.32	269648	25.84	0	0
LIQLASGNTNLK	Aldh	Aldehyde dehydrogenase (Aldehyde dehydrogenase, isoform A) (EC 1.2.1.10	636.37	2	28.18	729317	28.09	380954	0.67854
VSVNDFIIK	muc	AT21758p (Midline uncoordinated, isoform B) (EC 2.3.1.12) (Midline uncoordi	517.798	2	38.03	442234	37.88	706401	2.07502
VVDGAVAAR	muc	AT21758p (Midline uncoordinated, isoform B) (EC 2.3.1.12) (Midline uncoordi	429.246	2	12.17	236652	12.23	154993	0.85079
YEDIPVTNMR	muc	AT21758p (Midline uncoordinated, isoform B) (EC 2.3.1.12) (Midline uncoordi	627.295	2	20.92	490136	20.93	697704	1.84917
CALMAR	rtp	MORN repeat-containing protein 4 homolog (Retinophilin) (undertaker)	361.178	2	14.53	575465	14.6	264197	0.59639
CPEVVQR	rtp	MORN repeat-containing protein 4 homolog (Retinophilin) (undertaker)	444.224	2	11.3	1459432	11.41	416922	0.3711
HGIGHLQFADGTR	rtp	MORN repeat-containing protein 4 homolog (Retinophilin) (undertaker)	470.241	3	21.24	17467458	21.2	4610221	0.34286
IWGLGLLTFQDFTHGFPR	rtp	MORN repeat-containing protein 4 homolog (Retinophilin) (undertaker)	702.37	3	62.01	229368	60.93	2241547	12.6951
NEGFFQDCR	rtp	MORN repeat-containing protein 4 homolog (Retinophilin) (undertaker)	586.743	2	24.15	21363690	24.2	9108685	0.55386
SQCEHPY	rtp	MORN repeat-containing protein 4 homolog (Retinophilin) (undertaker)	460.682	2	10.09	3548788	10.22	1264609	0.46291
YEGEFR	rtp	MORN repeat-containing protein 4 homolog (Retinophilin) (undertaker)	400.682	2	13.99	438716	14.08	69103	0.20461
YIGEWNQR	rtp	MORN repeat-containing protein 4 homolog (Retinophilin) (undertaker)	533.259	2	22.21	23276284	22.21	14982978	0.83619
SGPNPIAEEAIK	CG2931	RE50009p	613.325	2	25.08	260764	24.98	259468	1.29258
DITLFHAMNILHK	MTA1-like	Metastasis associated 1-like, isoform A	518.282	3	43.88	2053379	43.78	955106	0.60423
DLPNPLVQLADK	MTA1-like	Metastasis associated 1-like, isoform A	661.869	2	44.73	4867516	44.55	2835007	0.7566
ELTIIPTQATSTIR	MTA1-like	Metastasis associated 1-like, isoform A	772.438	2	37.83	11123584	37.63	12065403	1.40903
HYAQAHGIAISSGSPRPIMK	MTA1-like	Metastasis associated 1-like, isoform A	531.031	4	22.34	2400440	22.14	3061997	1.65705
HYAQAHGIAISSGSPRPIMK	MTA1-like	Metastasis associated 1-like, isoform A	713.037	3	18.86	1475021	18.76	1372104	1.2084
HYAQAHGIAISSGSPRPIMK	MTA1-like	Metastasis associated 1-like, isoform A	535.03	4	18.86	8277828	18.73	11433715	1.79429
IDQFLVVSR	MTA1-like	Metastasis associated 1-like, isoform A	538.809	2	33.15	2031489	33.09	2200379	1.40704
ILSAVDLR	MTA1-like	Metastasis associated 1-like, isoform A	443.771	2	29.06	3480815	28.94	3278001	1.22335
LCLSCWEYWR	MTA1-like	Metastasis associated 1-like, isoform A	736.826	2	45.03	1100791	44.85	1003854	1.18464
LGAPGSGPHEWLVLTPK	MTA1-like	Metastasis associated 1-like, isoform A	586.989	3	38.45	4162383	38.27	5011628	1.56408
LLLLKPK	MTA1-like	Metastasis associated 1-like, isoform A	412.802	2	25.48	7404797	25.43	7870932	1.38081
QIIEYYYMWK	MTA1-like	Metastasis associated 1-like, isoform A	718.85	2	43.1	621851	42.87	464183	0.96967
QPSLHMSAAAASR	MTA1-like	Metastasis associated 1-like, isoform A	448.223	3	11.24	631709	11.36	455066	0.93579
QSAYAINAMLVK	MTA1-like	Metastasis associated 1-like, isoform A	654.853	2	35.58	1490566	35.41	2100155	1.8303
QSAYAINAMLVK	MTA1-like	Metastasis associated 1-like, isoform A	662.85	2	29.32	5849027	29.21	6763609	1.50216
QVESIPATQIR	MTA1-like	Metastasis associated 1-like, isoform A	621.346	2	23.95	29583954	23.96	39601180	1.7389
SIGTFAR	MTA1-like	Metastasis associated 1-like, isoform A	376.208	2	18.93	21987752	18.88	27082934	1.60006
SSQWNSVSSGHSTSR	MTA1-like	Metastasis associated 1-like, isoform A	536.245	3	13.07	1701975	13.07	2157652	1.64684
SSSAASTPTATLAGLATTPTAVVDL	MTA1-like	Metastasis associated 1-like, isoform A	968.816	3	46.62	1694407	46.37	1458500	1.11818
TAFYLHTNPMTR	MTA1-like	Metastasis associated 1-like, isoform A	726.359	2	28.65	3357775	28.53	4332503	1.67614
TAFYLHTNPMTR	MTA1-like	Metastasis associated 1-like, isoform A	484.575	3	28.65	6289877	28.53	6959267	1.43729
TAFYLHTNPMTR	MTA1-like	Metastasis associated 1-like, isoform A	734.356	2	22.69	3508484	22.72	3475736	1.28691
TAFYLHTNPMTR	MTA1-like	Metastasis associated 1-like, isoform A	489.906	3	22.69	11884596	22.72	15231011	1.66482
TLPIKPAAPEPSSRPIESQIVILD	MTA1-like	Metastasis associated 1-like, isoform A	857.818	3	41.39	34286420	41.22	42519524	1.61097
TPVSEEQAAQAVLDPSIAALDEER	MTA1-like	Metastasis associated 1-like, isoform A	847.089	3	45.9	2579368	45.66	1415875	0.71307
VGDYVYVETTPNSPYLIR	MTA1-like	Metastasis associated 1-like, isoform A	1043.53	2	39.99	19490416	39.8	21930646	1.46168
VGDYVYVETTPNSPYLIR	MTA1-like	Metastasis associated 1-like, isoform A	696.021	3	39.99	8430076	39.8	9468280	1.45902
VMCFYR	MTA1-like	Metastasis associated 1-like, isoform A	446.196	2	18.26	2536584	18.3	3037729	1.55569
YQCDIPAK	MTA1-like	Metastasis associated 1-like, isoform A	497.737	2	14.9	10434692	14.9	5215218	0.64925
DITLFHAMNILHK	MTA1-like	Metastasis associated 1-like, isoform B	518.282	3	43.88	2053379	43.78	955106	0.60423
DLPNPLVQLADK	MTA1-like	Metastasis associated 1-like, isoform B	661.869	2	44.73	4867516	44.55	2835007	0.7566
ELTIIPTQATSTIR	MTA1-like	Metastasis associated 1-like, isoform B	772.438	2	37.83	11123584	37.63	12065403	1.40903

HQPALDEER	MTA1-like	Metastasis associated 1-like, isoform B	547.765	2	10.77	1401862	10.88	390542	0.3619
HYAQAHGIAISSGSPRPIMK	MTA1-like	Metastasis associated 1-like, isoform B	531.031	4	22.34	2400440	22.14	3061997	1.65705
HYAQAHGIAISSGSPRPIMK	MTA1-like	Metastasis associated 1-like, isoform B	713.037	3	18.86	1475021	18.76	1372104	1.2084
HYAQAHGIAISSGSPRPIMK	MTA1-like	Metastasis associated 1-like, isoform B	535.03	4	18.86	8277828	18.73	11433715	1.79429
IDQFLVVSR	MTA1-like	Metastasis associated 1-like, isoform B	538.809	2	33.15	2031489	33.09	2200379	1.40704
ILSAVDLR	MTA1-like	Metastasis associated 1-like, isoform B	443.771	2	29.06	3480815	28.94	3278001	1.22335
LCLSCWEYWR	MTA1-like	Metastasis associated 1-like, isoform B	736.826	2	45.03	1100791	44.85	1003854	1.18464
LGAPGSGPHEWLVLTPK	MTA1-like	Metastasis associated 1-like, isoform B	586.989	3	38.45	4162383	38.27	5011628	1.56408
LLLLKPK	MTA1-like	Metastasis associated 1-like, isoform B	412.802	2	25.48	7404797	25.43	7870932	1.38081
QIIEYYYMWK	MTA1-like	Metastasis associated 1-like, isoform B	718.85	2	43.1	621851	42.87	464183	0.96967
QPSLHMSAAAASR	MTA1-like	Metastasis associated 1-like, isoform B	448.223	3	11.24	631709	11.36	455066	0.93579
QSAYAINAMLVK	MTA1-like	Metastasis associated 1-like, isoform B	654.853	2	35.58	1490566	35.41	2100155	1.8303
QSAYAINAMLVK	MTA1-like	Metastasis associated 1-like, isoform B	662.85	2	29.32	5849027	29.21	6763609	1.50216
QVESIPATQIR	MTA1-like	Metastasis associated 1-like, isoform B	621.346	2	23.95	29583954	23.96	39601180	1.7389
SIGTFAR	MTA1-like	Metastasis associated 1-like, isoform B	376.208	2	18.93	21987752	18.88	27082934	1.60006
SSQWNSVSSGHSTSR	MTA1-like	Metastasis associated 1-like, isoform B	536.245	3	13.07	1701975	13.07	2157652	1.64684
SSSAASTPTATLAGLATTPTAVVDL	MTA1-like	Metastasis associated 1-like, isoform B	968.816	3	46.62	1694407	46.37	1458500	1.11818
TAFYLHTNPMTR	MTA1-like	Metastasis associated 1-like, isoform B	726.359	2	28.65	3357775	28.53	4332503	1.67614
TAFYLHTNPMTR	MTA1-like	Metastasis associated 1-like, isoform B	484.575	3	28.65	6289877	28.53	6959267	1.43729
TAFYLHTNPMTR	MTA1-like	Metastasis associated 1-like, isoform B	734.356	2	22.69	3508484	22.72	3475736	1.28691
TAFYLHTNPMTR	MTA1-like	Metastasis associated 1-like, isoform B	489.906	3	22.69	11884596	22.72	15231011	1.66482
TLPIKPAAPEPSSRPIESQIVILD	MTA1-like	Metastasis associated 1-like, isoform B	857.818	3	41.39	34286420	41.22	42519524	1.61097
VGDYVYVETTPNSPYLIR	MTA1-like	Metastasis associated 1-like, isoform B	1043.53	2	39.99	19490416	39.8	21930646	1.46168
VGDYVYVETTPNSPYLIR	MTA1-like	Metastasis associated 1-like, isoform B	696.021	3	39.99	8430076	39.8	9468280	1.45902
VMCFYR	MTA1-like	Metastasis associated 1-like, isoform B	446.196	2	18.26	2536584	18.3	3037729	1.55569
YQCDIPAK	MTA1-like	Metastasis associated 1-like, isoform B	497.737	2	14.9	10434692	14.9	5215218	0.64925
ASAVNTLADLLVSR	CG7470	Delta-1-pyrroline-5-carboxylate synthase [Includes: Glutamate 5-kinase (GK)	715.404	2	49.47	2562427	49.2	1828687	0.92707
FGLGAEVGISTAR	CG7470	Delta-1-pyrroline-5-carboxylate synthase [Includes: Glutamate 5-kinase (GK)	639.346	2	34.41	2134752	34.23	1487748	0.90532
GPVGVEGLLTTK	CG7470	Delta-1-pyrroline-5-carboxylate synthase [Includes: Glutamate 5-kinase (GK)	585.84	2	35.73	1330746	35.54	933874	0.91162
LASIVEQVAECHLEGR	CG7470	Delta-1-pyrroline-5-carboxylate synthase [Includes: Glutamate 5-kinase (GK)	604.309	3	37.17	750377	37.14	243617	0.42175
LNQQLTFGPPAAK	CG7470	Delta-1-pyrroline-5-carboxylate synthase [Includes: Glutamate 5-kinase (GK)	692.883	2	29.99	1289638	29.81	1025810	1.03329
LSLNPAK	CG7470	Delta-1-pyrroline-5-carboxylate synthase [Includes: Glutamate 5-kinase (GK)	371.727	2	19.02	496459	19.02	288630	0.75523
HFEVHQSPSASTVSPK	pzg	LD15904p (Putzig, isoform A) (Putzig, isoform B)	579.957	3	16.11	209908	16.02	226656	1.40269
GYDYPQLESYQR	mRpL48	Mitochondrial ribosomal protein L48, isoform A (Mitochondrial ribosomal prote	759.847	2	28.57	975481	28.48	964360	1.28423
NLQLNNVDAPVYPQFLR	mRpL48	Mitochondrial ribosomal protein L48, isoform A (Mitochondrial ribosomal prote	1001.03	2	45.83	870044	45.59	811963	1.21232
LALVGGIFNLAAVLR	FASN2	Fatty acid synthase 2, isoform A (EC 1.-.-.-) (EC 2.3.1.85) (EC 3.1.-.-) (GH222	763.974	2	64.23	79694	63.78	0	0
DTSFEQLVLR	FASN1	Fatty acid synthase 1, isoform A (EC 1.-.-.-) (EC 2.3.1.85) (EC 3.1.-.-)	604.32	2	40.82	404038	40.66	103733	0.33352
LALVGGIFNLAAVLR	FASN1	Fatty acid synthase 1, isoform A (EC 1.-.-.-) (EC 2.3.1.85) (EC 3.1.-.-)	763.974	2	64.23	79694	63.78	0	0
NFDIVLSAQEIR	FASN1	Fatty acid synthase 1, isoform A (EC 1.-.-.-) (EC 2.3.1.85) (EC 3.1.-.-)	702.878	2	41.81	372388	41.64	404923	1.41253
YAATPFAAPIAAPVAAAYTAPIAAAA	CG13674	Uncharacterized protein, isoform A (Uncharacterized protein, isoform B)	984.891	3	60.84	1512020	60.6	1459350	1.25379
GQYSLVEPDGSIR	Cpr66Cb	Cuticular protein 66Cb (GEO11540p1) (IP05056p)	710.857	2	29.1	764886	29.02	975676	1.65703
AASPASSVSSLSR	Gug	Grunge, isoform H (EC 3.5.1.98)	610.317	2	19.87	670193	19.91	1047675	2.03072
ALQALVK	Gug	Grunge, isoform H (EC 3.5.1.98)	371.745	2	22.24	1429410	22.24	802676	0.72947
ASPSVQISSSGGGPSGPGSVGPG(	Gug	Grunge, isoform H (EC 3.5.1.98)	862.663	4	27.81	128265	27.69	91817	0.9299
MSYADQLQAAEFQR	Gug	Grunge, isoform H (EC 3.5.1.98)	829.386	2	32.37	172984	32.33	256318	1.92484
MYAPQPGQR	Gug	Grunge, isoform H (EC 3.5.1.98)	532.253	2	11.22	597570	11.23	396368	0.86165
QHQAAAAAAAQQHK	Gug	Grunge, isoform H (EC 3.5.1.98)	477.58	3	7.39	102108	7.46	61942	0.78804
QSLHDQYFR	Gug	Grunge, isoform H (EC 3.5.1.98)	398.528	3	18.86	599467	18.89	746793	1.61829
SQSAIFVR	Gug	Grunge, isoform H (EC 3.5.1.98)	454.253	2	22.57	2827859	22.59	2397174	1.10119
WSPGVVADGDLLMFLR	Gug	Grunge, isoform H (EC 3.5.1.98)	888.461	2	59.21	701351	58.95	433086	0.80216
WSPGVVADGDLLMFLR	Gug	Grunge, isoform H (EC 3.5.1.98)	896.459	2	56.45	1934292	56.29	935150	0.62803
YGDPLAAK	Gug	Grunge, isoform H (EC 3.5.1.98)	417.721	2	19.02	1065996	19	938830	1.14407
AELDDNEDVGPR	CG32352	Uncharacterized protein, isoform B	665.299	2	17.49	854844	17.49	358372	0.54459

AGTNDTDPVIEVEPAGVR	CG32352	Uncharacterized protein, isoform B	920.458	2	31.26	799045	31.12	994850	1.61737
ELTPFEQELQR	CG32352	Uncharacterized protein, isoform B	695.354	2	34.45	1822534	34.23	951564	0.67824
FATLPSIR	CG32352	Uncharacterized protein, isoform B	452.766	2	32.1	2652917	31.93	2934962	1.43715
FHDLQDAQSTASSGK	CG32352	Uncharacterized protein, isoform B	531.25	3	15.41	391086	15.36	283768	0.94257
GADSLILDGHIK	CG32352	Uncharacterized protein, isoform B	413.563	3	29.68	1264479	29.53	1469523	1.50969
GFMASAQDR	CG32352	Uncharacterized protein, isoform B	499.722	2	11.62	285875	11.75	165984	0.75424
GISEDNIQLR	CG32352	Uncharacterized protein, isoform B	572.801	2	25.08	2302903	24.99	2379743	1.34238
NYQPDFDSQEFQR	CG32352	Uncharacterized protein, isoform B	837.363	2	28.43	615106	28.34	745801	1.57505
RPLGPDEQPEIVLLLR	CG32352	Uncharacterized protein, isoform B	615.691	3	45.58	1022269	45.43	642182	0.81605
SVSNTFLQPHEDVIVYR	CG32352	Uncharacterized protein, isoform B	668.678	3	35.81	2074033	35.7	1364163	0.85442
TDYEIHTSQVDSSALEELPLQPENR	CG32352	Uncharacterized protein, isoform B	957.793	3	39.43	686104	39.2	452230	0.85623
THEDAQSLPGNSANPSR	CG32352	Uncharacterized protein, isoform B	594.278	3	13.23	675781	13.53	14072906	27.052
AELDDNEDVGPR	CG32355	FI19449p1	665.299	2	17.49	854844	17.49	358372	0.54459
AGTNDTDPVIEVEPAGVR	CG32355	FI19449p1	920.458	2	31.26	799045	31.12	994850	1.61737
ELTPFEQELQR	CG32355	FI19449p1	695.354	2	34.45	1822534	34.23	951564	0.67824
FATLPSIR	CG32355	FI19449p1	452.766	2	32.1	2652917	31.93	2934962	1.43715
FHDLQDAQSTASSGK	CG32355	FI19449p1	531.25	3	15.41	391086	15.36	283768	0.94257
GADSLILDGHIK	CG32355	FI19449p1	413.563	3	29.68	1264479	29.53	1469523	1.50969
GFMASAQDR	CG32355	FI19449p1	499.722	2	11.62	285875	11.75	165984	0.75424
GISEDNIQLR	CG32355	FI19449p1	572.801	2	25.08	2302903	24.99	2379743	1.34238
NYQPDFDSQEFQR	CG32355	FI19449p1	837.363	2	28.43	615106	28.34	745801	1.57505
RPLGPDEQPEIVLLLR	CG32355	FI19449p1	615.691	3	45.58	1022269	45.43	642182	0.81605
SVSNTFLQPHEDVIVYR	CG32355	FI19449p1	668.678	3	35.81	2074033	35.7	1364163	0.85442
TDYEIHTSQVDSSALEELPLQPENR	CG32355	FI19449p1	957.793	3	39.43	686104	39.2	452230	0.85623
THEDAQSLPGNSANPSR	CG32355	FI19449p1	594.278	3	13.23	675781	13.53	14072906	27.052
AELDDNEDVGPR	CG17926	Uncharacterized protein, isoform C	665.299	2	17.49	854844	17.49	358372	0.54459
FATLPSIR	CG17926	Uncharacterized protein, isoform C	452.766	2	32.1	2652917	31.93	2934962	1.43715
FHDLQDAQSTASSGK	CG17926	Uncharacterized protein, isoform C	531.25	3	15.41	391086	15.36	283768	0.94257
GADSLILDGHIK	CG17926	Uncharacterized protein, isoform C	413.563	3	29.68	1264479	29.53	1469523	1.50969
GFMASAQDR	CG17926	Uncharacterized protein, isoform C	499.722	2	11.62	285875	11.75	165984	0.75424
GISEDNIQLR	CG17926	Uncharacterized protein, isoform C	572.801	2	25.08	2302903	24.99	2379743	1.34238
NYQPDFDSQEFQR	CG17926	Uncharacterized protein, isoform C	837.363	2	28.43	615106	28.34	745801	1.57505
SVSNTFLQPHEDVIVYR	CG17926	Uncharacterized protein, isoform C	668.678	3	35.81	2074033	35.7	1364163	0.85442
TDYEIHTSQVDSSALEELPLQPENR	CG17926	Uncharacterized protein, isoform C	957.793	3	39.43	686104	39.2	452230	0.85623
STVPFATSESNR	Zasp66	HL08110p (Z band alternatively spliced PDZ-motif protein 66, isoform A) (Z ba	648.315	2	21.89	228788	21.86	230532	1.30894
NAVQTVYWR	CG13315	GEO09635p1 (RH39779p)	568.796	2	27.31	3597957	27.28	4850267	1.75118
VNHLIGATTR	CG13315	GEO09635p1 (RH39779p)	541.309	2	14.79	1950017	14.75	2217894	1.47749
VNHLIGATTR	CG13315	GEO09635p1 (RH39779p)	361.209	3	14.79	3569957	14.75	4473470	1.62781
STSGAIDTLR	mRRF1	FI06490p (Mitochondrial ribosome recycling factor 1)	510.77	2	19.09	2925555	19.08	2125581	0.94383
VTAVIPCFPYAR	Prps	RE57174p	697.368	2	40.66	474085	40.36	413943	1.13424
VYAILTHGIFSGPAISR	Prps	RE57174p	601.337	3	42.95	672496	42.83	628268	1.21361
VTAVIPCFPYAR	Prps	Uncharacterized protein, isoform B (EC 2.7.6.1)	697.368	2	40.66	474085	40.36	413943	1.13424
VYAILTHGIFSGPAISR	Prps	Uncharacterized protein, isoform B (EC 2.7.6.1)	601.337	3	42.95	672496	42.83	628268	1.21361
YPQSQIPPQLQPYPQR	CG14153	RE35371p	647.339	3	28.49	4310	28.68	418214	126.05
GGGSGTDFYNQQR	tral	GH08269p (Trailer hitch, isoform A) (Trailer hitch, isoform B)	693.805	2	16.19	310314	16.14	351936	1.47328
AHEGYIQLSK	CG11279	GEO07182p1 (GM21080p)	573.301	2	19.18	1084597	19.28	1934844	2.31739
AHEGYIQLSK	CG11279	GEO07182p1 (GM21080p)	382.537	3	19.14	371205	19.14	545452	1.90882
SVESELR	CG11279	GEO07182p1 (GM21080p)	410.214	2	15.65	1324624	15.63	1386481	1.3597
LAVAEFR	Hsc70Cb	Hsc70Cb, isoform A (Hsc70Cb, isoform B) (Hsc70Cb, isoform C) (Hsc70Cb, is	403.232	2	26.17	448094	26.11	568800	1.64897
TADSPVQISAVR	Hsc70Cb	Hsc70Cb, isoform A (Hsc70Cb, isoform B) (Hsc70Cb, isoform C) (Hsc70Cb, is	622.336	2	25.12	1436637	25.06	841173	0.76061
LVDSGFHPEAFDVVGGQPTAVVDR	SsRbeta	Translocon-associated protein subunit beta (TRAP-beta) (Signal sequence re	838.087	3	40.66	2020733	40.46	1937125	1.24529
YTIFNVGSGAATK	SsRbeta	Translocon-associated protein subunit beta (TRAP-beta) (Signal sequence re	664.846	2	33.12	1236140	33.02	1805212	1.89707
NNYDGTLSSYTTAPFEFAGPYSSR	CG13047	Uncharacterized protein	882.398	3	45.57	1619853	45.35	1817136	1.45725

YVSGIPAAPAVVAK	CG13047	Uncharacterized protein	671.89	2	31.49	6867514	31.35	8839718	1.6721
VAIIDLQDNLEEFVK	Pdh	HL08057p (IP03491p) (Photoreceptor dehydrogenase, isoform A) (EC 1.1.1.1	873.47	2	52.44	438704	52.21	466578	1.38158
AIELVHK	UQCR-C2	AT02348p (Ubiquinol-cytochrome c reductase core protein 2, isoform A) (EC	405.248	2	16.79	126835	16.8	172499	1.76673
EALAFAILEQALGAK	UQCR-C2	AT02348p (Ubiquinol-cytochrome c reductase core protein 2, isoform A) (EC	772.938	2	62.65	170205	62.74	0	0
EALAFAILEQALGAK	UQCR-C2	AT02348p (Ubiquinol-cytochrome c reductase core protein 2, isoform A) (EC	515.628	3	62.58	74861	62.77	11133	0.19319
LAGGLSTQNSTAFAIAR	UQCR-C2	AT02348p (Ubiquinol-cytochrome c reductase core protein 2, isoform A) (EC	839.45	2	33.65	746600	33.41	2728467	4.74737
LAVGAIGHLANVPYASDLA	UQCR-C2	AT02348p (Ubiquinol-cytochrome c reductase core protein 2, isoform A) (EC	926.502	2	45.81	1586094	45.61	1624191	1.33024
LVVATADATLPVSR	UQCR-C2	AT02348p (Ubiquinol-cytochrome c reductase core protein 2, isoform A) (EC	706.909	2	32.76	1081464	32.64	965724	1.16001
NVLEADALLGAIDGISQSQVQEAAK	UQCR-C2	AT02348p (Ubiquinol-cytochrome c reductase core protein 2, isoform A) (EC	847.445	3	53.98	310764	53.7	726980	3.03888
LVPVATSHQFVTR	CG13026	Uncharacterized protein, isoform B (Uncharacterized protein, isoform C)	727.909	2	25.16	634571	25.1	470066	0.96228
IGGVTLEILAK	GS	GH26789p	557.348	2	40.98	21782	40.9	251762	15.0146
LVSEVGVGVVASGVAK	GS	GH26789p	735.93	2	33.15	0	33.02	197421	#DIV/0!
AFAGAISK	UQCR-Q	AT13736p (GEO08881p1) (Ubiquinol-cytochrome c reductase ubiquinone-bin	382.719	2	16.94	2457756	16.95	3271900	1.72935
LSPFEQR	UQCR-Q	AT13736p (GEO08881p1) (Ubiquinol-cytochrome c reductase ubiquinone-bin	438.732	2	20.96	3697092	20.97	5274297	1.85322
SNVFIVTPPFIVGYLIYDLTER	UQCR-Q	AT13736p (GEO08881p1) (Ubiquinol-cytochrome c reductase ubiquinone-bin	852.796	3	65.7	345422	65.71	194668	0.73209
VHGIVTYK	UQCR-Q	AT13736p (GEO08881p1) (Ubiquinol-cytochrome c reductase ubiquinone-bin	458.766	2	15.41	1114019	15.36	1240016	1.44596
DDEVQVIR	RpL26	GEO07453p1 (RE17611p) (Ribosomal protein L26, isoform A) (Ribosomal pro	487.251	2	20.33	646067	20.31	313988	0.63133
HFQAPSHIR	RpL26	GEO07453p1 (RE17611p) (Ribosomal protein L26, isoform A) (Ribosomal pro	364.861	3	13.63	2235615	13.59	2327669	1.35253
APAPVAFAAPVAAPLAAPILNR	CG14095	Uncharacterized protein	1049.61	2	51.53	517911	51.2	241907	0.60676
APAPVAFAAPVAAPLAAPILNR	CG14095	Uncharacterized protein	700.077	3	51.53	2270896	51.24	1187401	0.67924
VAPFAAPIATPFAAPYAHPYAAPVL/	CG14095	Uncharacterized protein	908.163	3	49.29	3508338	49.09	3146724	1.16514
DGVLTVDAPLPALTAGETLIPIAHK	dHspB8	LD37169p	838.139	3	55.9	27215	55.42	322725	15.4045
GVNPESIR	dHspB8	LD37169p	436.235	2	16.4	162532	16.45	263810	2.10851
ASAIQHILEHQSPAAGR	htk	Hat-trick, isoform D	595.983	3	25.4	244189	25.35	157660	0.83872
LQQQEELGQAQGQSK	htk	Hat-trick, isoform D	836.418	2	14.34	322759	14.31	224397	0.90315
ELIAICER	CG1461	Tyrosine aminotransferase (TAT) (EC 2.6.1.5)	502.266	2	27.85	2234	27.8	41760	24.2828
HYLPIIADEIYEHFVFPGSK	CG1461	Tyrosine aminotransferase (TAT) (EC 2.6.1.5)	792.407	3	56.7	42312	56.72	117214	3.59864
IVLTVPGAMIEEACSR	CG1461	Tyrosine aminotransferase (TAT) (EC 2.6.1.5)	873.45	2	44.08	2644	44.26	146014	71.739
NFIEHGLLDCDDVAF	CG1461	Tyrosine aminotransferase (TAT) (EC 2.6.1.5)	882.899	2	46.9	27995	46.68	41195	1.91155
INLIER	Brms1	BRMS1 (LD14895p)	379.232	2	23.44	2578008	23.49	2364046	1.19123
ILDELEEEVYEINAEQQFLLLR	Lint-1	L(3)mbt interacting protein 1, isoform A (L(3)mbt interacting protein 1, isoform	902.801	3	61.89	784107	61.7	561166	0.92969
LNDSLQEFSVK	Lint-1	L(3)mbt interacting protein 1, isoform A (L(3)mbt interacting protein 1, isoform	640.33	2	31.72	1131452	31.63	1444369	1.6583
TQPEEYGQETFLGLFELYSPEVLK	Lint-1	L(3)mbt interacting protein 1, isoform A (L(3)mbt interacting protein 1, isoform	939.8	3	64.56	308483	64.3	525500	2.21291
TVQNASGVDFHYGQQLNAVDALVM	Lint-1	L(3)mbt interacting protein 1, isoform A (L(3)mbt interacting protein 1, isoform	969.147	3	46.58	2486428	46.37	2112926	1.1039
LSDVTVTPIPR	CDK2AP1	CDK2-associated protein 1, isoform A (FI05233p)	599.346	2	29.17	1997051	29.1	2367705	1.54014
SQVQNFYNYQQQR	CDK2AP1	CDK2-associated protein 1, isoform A (FI05233p)	851.9	2	23.81	499679	23.84	647495	1.68332
LGAGAGTGYPYYNTR	CG2157	LP02042p	780.876	2	24.59	230436	24.56	514144	2.89839
DVPVAVAPVAPVLAK	Cpr64Ad	Cuticular protein 64Ad (RE69226p)	723.44	2	40.41	8575854	40.19	9120881	1.3816
FAYDVQDTLTGDSK	Cpr64Ad	Cuticular protein 64Ad (RE69226p)	780.365	2	32.1	2517516	31.94	1573502	0.81193
GSYSLIEPDGSR	Cpr64Ad	Cuticular protein 64Ad (RE69226p)	640.809	2	26.13	3158432	26.11	2432972	1.00066
TVAAPVAPVVAAAPAPVFAK	Cpr64Ad	Cuticular protein 64Ad (RE69226p)	924.043	2	41.95	5529904	41.71	6193381	1.4549
TVAAPVAPVVAAAPAPVFAK	Cpr64Ad	Cuticular protein 64Ad (RE69226p)	616.364	3	41.95	3358153	41.71	4283161	1.65686
TYAAAPFAAPFAAPVAPVAAR	Cpr64Ad	Cuticular protein 64Ad (RE69226p)	1015.55	2	46.4	6035925	46.19	6138683	1.32115
TYAAAPFAAPFAAPVAPVAAR	Cpr64Ad	Cuticular protein 64Ad (RE69226p)	677.367	3	46.4	6287974	46.15	6849990	1.41515
GSYSVVDADGSLR	Cpr64Ab	Cuticular protein 64Ab (GEO10707p1)	663.32	2	25.6	2217422	25.52	1531983	0.89749
STSISNVAGASLTR	ens	Ensconsin, isoform B	682.362	2	25.86	816429	25.79	694557	1.10513
AIGEFTVANEHLNR	BcDNA:R	CG16985 protein (CG16985-PA) (GEO07616p1) (RE30174p)	785.902	2	28.11	512160	28.08	482749	1.22444
AIGEFTVANEHLNR	BcDNA:R	CG16985 protein (CG16985-PA) (GEO07616p1) (RE30174p)	524.271	3	28.15	1306259	28.01	1428046	1.42015
GQYSLVEPDGSIR	Cpr62Bb	Cuticular protein 62Bb, isoform A (Cuticular protein 62Bb, isoform B) (Cuticula	710.857	2	29.1	764886	29.02	975676	1.65703
TVDYTADSIHGFNAVVTK	Cpr62Bb	Cuticular protein 62Bb, isoform A (Cuticular protein 62Bb, isoform B) (Cuticula	646.658	3	33.06	1350578	32.91	1897710	1.82529
YAFNYGVADHSTGDVK	Cpr62Bb	Cuticular protein 62Bb, isoform A (Cuticular protein 62Bb, isoform B) (Cuticula	581.937	3	25.93	1003839	25.87	1635503	2.11646
YYLGIFFK	tfc	RE15268p (Triforce, isoform A) (Triforce, isoform C)	525.787	2	48.48	464111	48.22	385306	1.07847
ALTTLNKPDLAPNSR	Crtp	Caldesmon-related protein (FI24430p1)	537.634	3	38.02	218088	37.87	927387	5.52397

CVGGEVGATSSLAPK	RpL12	GEO07602p1 (RE28824p) (Ribosomal protein L12, isoform A) (Ribosomal pro	716.859	2	22.65	976048	22.64	1029705	1.37045
HSGNIGFEDILAIAR	RpL12	GEO07602p1 (RE28824p) (Ribosomal protein L12, isoform A) (Ribosomal pro	806.926	2	46.59	1617829	46.41	1499515	1.20404
HSGNIGFEDILAIAR	RpL12	GEO07602p1 (RE28824p) (Ribosomal protein L12, isoform A) (Ribosomal pro	538.286	3	46.59	5548026	46.45	5434465	1.27245
IGPLGLSPK	RpL12	GEO07602p1 (RE28824p) (Ribosomal protein L12, isoform A) (Ribosomal pro	441.276	2	29.56	3940882	29.48	4841355	1.59586
STVDPNDPFAFEGPEIYK	Nap1	LD21576p (MIP15604p) (Nucleosome assembly protein 1, isoform A) (Nucleo	1013.48	2	42.58	250367	42.4	382398	1.98409
EAALAAGFSNK	Mtpbeta	GH13256p (Thiolase) (EC 1.1.1.211) (EC 2.3.1.16) (EC 4.2.1.17) (EC 4.2.1.74	539.78	2	22.68	1336355	22.68	566491	0.55067
GYFTDLVPFK	Mtpbeta	GH13256p (Thiolase) (EC 1.1.1.211) (EC 2.3.1.16) (EC 4.2.1.17) (EC 4.2.1.74	593.811	2	46.75	559291	46.49	310100	0.72025
HSLLSLLQK	Mtpbeta	GH13256p (Thiolase) (EC 1.1.1.211) (EC 2.3.1.16) (EC 4.2.1.17) (EC 4.2.1.74	519.819	2	36.67	1905280	36.52	1640452	1.11848
LASAFNVSR	Mtpbeta	GH13256p (Thiolase) (EC 1.1.1.211) (EC 2.3.1.16) (EC 4.2.1.17) (EC 4.2.1.74	482.764	2	23.13	1522885	23.18	826583	0.70509
SEQDEYALR	Mtpbeta	GH13256p (Thiolase) (EC 1.1.1.211) (EC 2.3.1.16) (EC 4.2.1.17) (EC 4.2.1.74	555.757	2	17.25	1380614	17.26	1028286	0.96753
YVGEIGDVVVAR	Rrp4	Rrp4, isoform A (Rrp4, isoform B)	638.848	2	32.53	400053	32.41	263223	0.85473
SLFHNPHVNALPDGYEH	levy	Levy, isoform A (Levy, isoform B) (RH03295p)	649.643	3	29.17	575588	29.09	809870	1.82779
FLAPQAQGPVGLALDIPIGILR	CG9812	Uncharacterized protein, isoform D	1130.16	2	63.35	1256442	63.16	1860146	1.92321
FLAPQAQGPVGLALDIPIGILR	CG9812	Uncharacterized protein, isoform D	753.779	3	63.38	2942061	63.13	4898123	2.16272
SVQRPQAVNNR	CG9812	Uncharacterized protein, isoform D	634.845	2	9.41	110272	9.55	122764	1.4462
SVQRPQAVNNR	CG9812	Uncharacterized protein, isoform D	423.565	3	9.37	375735	9.52	435353	1.50516
TAGVGYAAAQANPSVGASALLGGS	CG9812	Uncharacterized protein, isoform D	920.702	4	36.12	888234	35.9	1594106	2.33137
GATALGPATTSGASER	PIP5K59E	FI05352p (Phosphatidylinositol 4-phosphate 5-kinase at 59B, isoform A) (EC 2	723.863	2	16.98	232423	17.03	258407	1.44427
GATALGPATTSGASER	PIP5K59E	Phosphatidylinositol 4-phosphate 5-kinase at 59B, isoform E (EC 2.7.1.-) (EC	723.863	2	16.98	232423	17.03	258407	1.44427
ASPSLYGTAER	jbug	Jitterbug, isoform L	576.288	2	18.78	139955	18.74	220071	2.04266
ASPSLYGTAER	jbug	Jitterbug, isoform F	576.288	2	18.78	139955	18.74	220071	2.04266
HGLFEQK	RpS24	40S ribosomal protein S24	429.727	2	15.65	734143	15.55	688748	1.21871
VTPDVVFAFGFR	RpS24	40S ribosomal protein S24	677.861	2	51.03	3258168	50.75	2847528	1.13532
ILAAFFFPGK	Ugt49B1	UDP-glucuronosyltransferase (EC 2.4.1.17)	555.821	2	49.15	2262101	48.94	1079689	0.62002
NTVLEMTDLPTFAFLR	Ugt49B1	UDP-glucuronosyltransferase (EC 2.4.1.17)	942.482	2	53.08	2090973	52.94	532690	0.33094
SHFMMTNAIIR	Ugt49B1	UDP-glucuronosyltransferase (EC 2.4.1.17)	668.829	2	28.29	606574	28.21	392749	0.84111
VFLEVFGSLK	Ugt49B1	UDP-glucuronosyltransferase (EC 2.4.1.17)	569.829	2	47.97	3258128	47.71	3007730	1.1992
ATYAVMGLLIAYQVLKPK	Neb-cGP	Neb-cGP, isoform A (Neb-cGP, isoform B) (RE31692p) (RH45340p)	660.385	3	60.54	1171729	60.48	1047522	1.16134
AQAAIAVEVAEALVK	ATPsynd	ATP synthase, delta subunit, isoform A (ATP synthase, delta subunit, isoform	741.93	2	51.6	570354	51.28	878625	2.00116
ALLSLPTAIADNAGYDSAQLVSELR	CCT2	Chaperonin containing TCP1 subunit 2 (SD02216p)	1294.68	2	58.23	1928466	58.01	2488253	1.67612
ALLSLPTAIADNAGYDSAQLVSELR	CCT2	Chaperonin containing TCP1 subunit 2 (SD02216p)	863.457	3	58.19	9879444	58.01	10569201	1.38973
AVGVDNPAAK	CCT2	Chaperonin containing TCP1 subunit 2 (SD02216p)	471.256	2	13.5	877078	13.47	639345	0.94693
CNVFINR	CCT2	Chaperonin containing TCP1 subunit 2 (SD02216p)	461.732	2	21.15	360213	21.15	430311	1.55183
EAIAIEAFAR	CCT2	Chaperonin containing TCP1 subunit 2 (SD02216p)	545.798	2	36.37	1126825	36.16	819469	0.94471
IADLEMAEK	CCT2	Chaperonin containing TCP1 subunit 2 (SD02216p)	518.255	2	17.6	116035	17.64	184706	2.06783
ILVATGR	CCT2	Chaperonin containing TCP1 subunit 2 (SD02216p)	365.235	2	17.02	3243592	17.03	2722743	1.09044
LAYCTGGEIVSTFENPSLVK	CCT2	Chaperonin containing TCP1 subunit 2 (SD02216p)	1093.05	2	43.84	1333044	43.62	996915	0.97148
LAYCTGGEIVSTFENPSLVK	CCT2	Chaperonin containing TCP1 subunit 2 (SD02216p)	729.033	3	43.84	1612664	43.66	1524278	1.22784
LGEACTIVIR	CCT2	Chaperonin containing TCP1 subunit 2 (SD02216p)	566.313	2	28.26	3652809	28.2	3881738	1.38045
LGECDVIEQVMIGEDTLLR	CCT2	Chaperonin containing TCP1 subunit 2 (SD02216p)	730.698	3	56.69	321606	56.4	337532	1.36337
LGECDVIEQVMIGEDTLLR	CCT2	Chaperonin containing TCP1 subunit 2 (SD02216p)	736.029	3	51.31	385622	50.95	378485	1.275
LSSFIGAIAIGDLVK	CCT2	Chaperonin containing TCP1 subunit 2 (SD02216p)	752.443	2	54.88	9430067	54.6	11966694	1.64847
LSSFIGAIAIGDLVK	CCT2	Chaperonin containing TCP1 subunit 2 (SD02216p)	501.964	3	54.85	412679	54.6	596085	1.87637
NAGHVEVTNDGATILR	CCT2	Chaperonin containing TCP1 subunit 2 (SD02216p)	833.929	2	24.19	891284	24.2	1103077	1.60773
NAGHVEVTNDGATILR	CCT2	Chaperonin containing TCP1 subunit 2 (SD02216p)	556.288	3	24.23	4363636	24.2	5712773	1.70067
QATQVALEALTAAAQDNSSSDEK	CCT2	Chaperonin containing TCP1 subunit 2 (SD02216p)	783.379	3	39.58	351059	39.37	148801	0.55062
QLIYNYPEQLFADAR	CCT2	Chaperonin containing TCP1 subunit 2 (SD02216p)	614.312	3	46.95	441710	46.72	616657	1.81355
QTLGLDMELGK	CCT2	Chaperonin containing TCP1 subunit 2 (SD02216p)	602.816	2	36.05	342380	35.86	395495	1.50057
SGGTLGDSFLDEGFLLDK	CCT2	Chaperonin containing TCP1 subunit 2 (SD02216p)	935.957	2	51	555102	50.75	429057	1.00407
SLHDALCVLAATVK	CCT2	Chaperonin containing TCP1 subunit 2 (SD02216p)	499.941	3	45.14	803057	44.97	267410	0.43257
VDNIIR	CCT2	Chaperonin containing TCP1 subunit 2 (SD02216p)	365.216	2	16.71	2405682	16.72	1267340	0.68435
VMSIEHADFDGIER	CCT2	Chaperonin containing TCP1 subunit 2 (SD02216p)	809.88	2	31.34	278355	31.27	233245	1.08852
VQDEEVGDGTTSVTVLASELLR	CCT2	Chaperonin containing TCP1 subunit 2 (SD02216p)	1159.59	2	53.99	1961062	53.69	1720362	1.1396

VQDEEVGDGTTSVTVLASELLR	CCT2	Chaperonin containing TCP1 subunit 2 (SD02216p)	773.396	3	54.02	4045842	53.69	2249428	0.72225
YGVEVPK	Upf2	RE04053p (Upf2)	396.219	2	18.74	1025713	18.73	969049	1.22728
GTCELSIGSPAPVVISNFGSQR	CG2233	GH20802p	759.383	3	42.99	233628	42.76	526580	2.92794
SGTGQDEFVIIGHNSPFYR	CG2233	GH20802p	708.676	3	39.43	326208	39.27	483470	1.92529
FGCLSFVFVVPSVDLLQAQAR	PF1	GH06635p	785.084	3	63.1	388	62.89	561	1.87825
ESTLHLVLR	Ubi-p5E	GH17761p (Ubiquitin-5E, isoform A) (Ubiquitin-5E, isoform B)	534.314	2	29.06	19834334	28.96	12823394	0.83986
ESTLHLVLR	Ubi-p5E	GH17761p (Ubiquitin-5E, isoform A) (Ubiquitin-5E, isoform B)	356.545	3	29.02	13873703	28.96	8349692	0.78181
ESTLHLVLR	Ubi-p5E	GH17761p (Ubiquitin-5E, isoform A) (Ubiquitin-5E, isoform B)	534.314	2	29.06	19834334	28.96	12823394	0.83986
ESTLHLVLR	Ubi-p5E	GH17761p (Ubiquitin-5E, isoform A) (Ubiquitin-5E, isoform B)	356.545	3	29.02	13873703	28.96	8349692	0.78181
ESTLHLVLR	Ubi-p5E	GH17761p (Ubiquitin-5E, isoform A) (Ubiquitin-5E, isoform B)	534.314	2	29.06	19834334	28.96	12823394	0.83986
ESTLHLVLR	Ubi-p5E	GH17761p (Ubiquitin-5E, isoform A) (Ubiquitin-5E, isoform B)	356.545	3	29.02	13873703	28.96	8349692	0.78181
ESTLHLVLR	Ubi-p5E	GH17761p (Ubiquitin-5E, isoform A) (Ubiquitin-5E, isoform B)	534.314	2	29.06	19834334	28.96	12823394	0.83986
ESTLHLVLR	Ubi-p5E	GH17761p (Ubiquitin-5E, isoform A) (Ubiquitin-5E, isoform B)	356.545	3	29.02	13873703	28.96	8349692	0.78181
ESTLHLVLR	Ubi-p5E	GH17761p (Ubiquitin-5E, isoform A) (Ubiquitin-5E, isoform B)	534.314	2	29.06	19834334	28.96	12823394	0.83986
ESTLHLVLR	Ubi-p5E	GH17761p (Ubiquitin-5E, isoform A) (Ubiquitin-5E, isoform B)	356.545	3	29.02	13873703	28.96	8349692	0.78181
ESTLHLVLR	Ubi-p5E	GH17761p (Ubiquitin-5E, isoform A) (Ubiquitin-5E, isoform B)	534.314	2	29.06	19834334	28.96	12823394	0.83986
ESTLHLVLR	Ubi-p5E	GH17761p (Ubiquitin-5E, isoform A) (Ubiquitin-5E, isoform B)	356.545	3	29.02	13873703	28.96	8349692	0.78181
ESTLHLVLR	Ubi-p5E	GH17761p (Ubiquitin-5E, isoform A) (Ubiquitin-5E, isoform B)	534.314	2	29.06	19834334	28.96	12823394	0.83986
ESTLHLVLR	Ubi-p5E	GH17761p (Ubiquitin-5E, isoform A) (Ubiquitin-5E, isoform B)	356.545	3	29.02	13873703	28.96	8349692	0.78181
MQIFVK	Ubi-p5E	GH17761p (Ubiquitin-5E, isoform A) (Ubiquitin-5E, isoform B)	383.22	2	27.43	8697430	27.39	10348569	1.54565
TITLEVEPSDTIENVK	Ubi-p5E	GH17761p (Ubiquitin-5E, isoform A) (Ubiquitin-5E, isoform B)	894.467	2	36.85	41743372	36.64	47966256	1.49269
TITLEVEPSDTIENVK	Ubi-p5E	GH17761p (Ubiquitin-5E, isoform A) (Ubiquitin-5E, isoform B)	596.647	3	36.85	1563861	36.68	1963090	1.63066
TITLEVEPSDTIENVK	Ubi-p5E	GH17761p (Ubiquitin-5E, isoform A) (Ubiquitin-5E, isoform B)	894.467	2	36.85	41743372	36.64	47966256	1.49269
TITLEVEPSDTIENVK	Ubi-p5E	GH17761p (Ubiquitin-5E, isoform A) (Ubiquitin-5E, isoform B)	596.647	3	36.85	1563861	36.68	1963090	1.63066
TITLEVEPSDTIENVK	Ubi-p5E	GH17761p (Ubiquitin-5E, isoform A) (Ubiquitin-5E, isoform B)	894.467	2	36.85	41743372	36.64	47966256	1.49269
TITLEVEPSDTIENVK	Ubi-p5E	GH17761p (Ubiquitin-5E, isoform A) (Ubiquitin-5E, isoform B)	596.647	3	36.85	1563861	36.68	1963090	1.63066
TITLEVEPSDTIENVK	Ubi-p5E	GH17761p (Ubiquitin-5E, isoform A) (Ubiquitin-5E, isoform B)	894.467	2	36.85	41743372	36.64	47966256	1.49269
TITLEVEPSDTIENVK	Ubi-p5E	GH17761p (Ubiquitin-5E, isoform A) (Ubiquitin-5E, isoform B)	596.647	3	36.85	1563861	36.68	1963090	1.63066
TITLEVEPSDTIENVK	Ubi-p5E	GH17761p (Ubiquitin-5E, isoform A) (Ubiquitin-5E, isoform B)	894.467	2	36.85	41743372	36.64	47966256	1.49269
TITLEVEPSDTIENVK	Ubi-p5E	GH17761p (Ubiquitin-5E, isoform A) (Ubiquitin-5E, isoform B)	596.647	3	36.85	1563861	36.68	1963090	1.63066
TITLEVEPSDTIENVK	Ubi-p5E	GH17761p (Ubiquitin-5E, isoform A) (Ubiquitin-5E, isoform B)	894.467	2	36.85	41743372	36.64	47966256	1.49269
TITLEVEPSDTIENVK	Ubi-p5E	GH17761p (Ubiquitin-5E, isoform A) (Ubiquitin-5E, isoform B)	596.647	3	36.85	1563861	36.68	1963090	1.63066
TITLEVEPSDTIENVK	Ubi-p5E	GH17761p (Ubiquitin-5E, isoform A) (Ubiquitin-5E, isoform B)	894.467	2	36.85	41743372	36.64	47966256	1.49269
TITLEVEPSDTIENVK	Ubi-p5E	GH17761p (Ubiquitin-5E, isoform A) (Ubiquitin-5E, isoform B)	596.647	3	36.85	1563861	36.68	1963090	1.63066
TLSDYNIQK	Ubi-p5E	GH17761p (Ubiquitin-5E, isoform A) (Ubiquitin-5E, isoform B)	541.28	2	19.53	19914654	19.52	7860957	0.51277
TLSDYNIQK	Ubi-p5E	GH17761p (Ubiquitin-5E, isoform A) (Ubiquitin-5E, isoform B)	541.28	2	19.53	19914654	19.52	7860957	0.51277
TLSDYNIQK	Ubi-p5E	GH17761p (Ubiquitin-5E, isoform A) (Ubiquitin-5E, isoform B)	541.28	2	19.53	19914654	19.52	7860957	0.51277
TLSDYNIQK	Ubi-p5E	GH17761p (Ubiquitin-5E, isoform A) (Ubiquitin-5E, isoform B)	541.28	2	19.53	19914654	19.52	7860957	0.51277
TLSDYNIQK	Ubi-p5E	GH17761p (Ubiquitin-5E, isoform A) (Ubiquitin-5E, isoform B)	541.28	2	19.53	19914654	19.52	7860957	0.51277
TLSDYNIQK	Ubi-p5E	GH17761p (Ubiquitin-5E, isoform A) (Ubiquitin-5E, isoform B)	541.28	2	19.53	19914654	19.52	7860957	0.51277
TLSDYNIQK	Ubi-p5E	GH17761p (Ubiquitin-5E, isoform A) (Ubiquitin-5E, isoform B)	541.28	2	19.53	19914654	19.52	7860957	0.51277
FVGPFTRPVVTIMTLR	Vap33	LD30122p (VAMP-associated protein 33kDa, isoform B) (VAMP-associated pr	612.018	3	49.47	300313	49.3	756291	3.27143
SLFDLPLTIEPEHELR	Vap33	LD30122p (VAMP-associated protein 33kDa, isoform B) (VAMP-associated pr	637.007	3	51.03	379872	50.9	998731	3.41534
SALSELAEALK	CG14419	RH61722p	566.316	2	45.94	535910	45.71	806686	1.9554
VSDLLILPGVTPQGNK	CG3091	RH61816p	825.975	2	45.08	47759	44.02	510307	13.8803
LQRPVASTVGESASQSV	CG13377	RE15974p	858.45	2	21.39	46513	22.9	60244	1.68253
AIGAGGQSTQVR	Sap130	Sin3A-associated protein 130, isoform A	572.807	2	12.25	925618	12.32	536264	0.75261
GPGAGGTALTATNLPTTR	Sap130	Sin3A-associated protein 130, isoform A	828.439	2	27.36	569694	27.23	266622	0.60796
IGDQPATGGAGGR	Sap130	Sin3A-associated protein 130, isoform A	578.789	2	10.2	451645	10.33	79684	0.22919
IIQLQQPATGTTQQIIGSGAR	Sap130	Sin3A-associated protein 130, isoform A	727.738	3	34.41	1767358	34.26	2314474	1.70118
QLENVTATGR	Sap130	Sin3A-associated protein 130, isoform A	544.788	2	15.37	399235	15.31	452872	1.47356
SDGGGGISIAGTPILSGGTIK	Sap130	Sin3A-associated protein 130, isoform A	929.499	2	41.44	945778	41.22	943168	1.29545
TQITAIPAAPAR	Sap130	Sin3A-associated protein 130, isoform A	605.351	2	25.4	3213530	25.35	3375310	1.36444

VQPSLTITQLNPIGK	Sap130	Sin3A-associated protein 130, isoform A	804.97	2	41.17	1625853	40.98	1635254	1.30655
VTHGVASSAASVPATVGPVNSTR	Sap130	Sin3A-associated protein 130, isoform A	722.382	3	23.81	505024	23.8	708422	1.82223
VTTGAGNPQQATASGALVSSFMR	Sap130	Sin3A-associated protein 130, isoform A	756.374	3	32.37	491161	32.25	670045	1.77216
FVFDVFEFAQK	mmy	LD24639p (Mummy, isoform A) (EC 2.7.7.23)	688.848	2	54.52	25125	54.13	69150	3.57527
ESTLHLVLR	Ubi-p5E5	Uncharacterized protein	534.314	2	29.06	19834334	28.96	12823394	0.83986
ESTLHLVLR	Ubi-p5E5	Uncharacterized protein	356.545	3	29.02	13873703	28.96	8349692	0.78181
MQIFVK	Ubi-p5E5	Uncharacterized protein	383.22	2	27.43	8697430	27.39	10348569	1.54565
TITLEVEPSDTIENVK	Ubi-p5E5	Uncharacterized protein	894.467	2	36.85	41743372	36.64	47966256	1.49269
TITLEVEPSDTIENVK	Ubi-p5E5	Uncharacterized protein	596.647	3	36.85	1563861	36.68	1963090	1.63066
TITLEVEPSDTIENVK	Ubi-p5E5	Uncharacterized protein	894.467	2	36.85	41743372	36.64	47966256	1.49269
TITLEVEPSDTIENVK	Ubi-p5E5	Uncharacterized protein	596.647	3	36.85	1563861	36.68	1963090	1.63066
TLSDYNIQK	Ubi-p5E5	Uncharacterized protein	541.28	2	19.53	19914654	19.52	7860957	0.51277
TLSDYNIQK	Ubi-p5E5	Uncharacterized protein	541.28	2	19.53	19914654	19.52	7860957	0.51277
TLSDYNIQK	Ubi-p5E5	Uncharacterized protein	541.28	2	19.53	19914654	19.52	7860957	0.51277
TLSDYNIQK	Ubi-p5E5	Uncharacterized protein	541.28	2	19.53	19914654	19.52	7860957	0.51277
ASAIQHILEHQSPAAGR	htk	Hat-trick, isoform C	595.983	3	25.4	244189	25.35	157660	0.83872
LQQQEELGQAQGQSK	htk	Hat-trick, isoform C	836.418	2	14.34	322759	14.31	224397	0.90315
IGTSEPIGR	Syt1	Synaptotagmin 1, isoform I	465.256	2	15.84	896749	15.87	539143	0.78101
TLSPVFNETFTFK	Syt1	Synaptotagmin 1, isoform I	765.896	2	45.21	1109199	44.93	1551467	1.817
AGIAVEGDIK	Arr1	Arrestin 1, isoform C	486.772	2	22.98	2931108	22.98	1880959	0.83362
ASDESQPCGVQYFVK	Arr1	Arrestin 1, isoform C	857.891	2	30.22	589087	30.08	894673	1.97291
DFLLSPGELELEVTLDK	Arr1	Arrestin 1, isoform C	959.506	2	56.3	265898	55.99	462292	2.25852
IFTGDSDCDR	Arr1	Arrestin 1, isoform C	593.246	2	14.94	1443871	14.91	748462	0.67339
IFVQLVCNFR	Arr1	Arrestin 1, isoform C	648.35	2	42.99	1459871	42.8	1343715	1.19568
ISVNICVR	Arr1	Arrestin 1, isoform C	480.768	2	29.06	677107	28.92	402608	0.77241
QGIQPCTVVR	Arr1	Arrestin 1, isoform C	579.308	2	21.75	818570	21.73	482335	0.76545
STINLGIR	Arr1	Arrestin 1, isoform C	437.261	2	27.76	3970398	27.69	3553533	1.16265
VMYLVPTLVANCDR	Arr1	Arrestin 1, isoform C	825.921	2	44.34	873153	44.19	632809	0.94147
ELSNLESVNPK	KFase	Kynurenine formamidase (KFA) (KFase) (EC 3.5.1.9) (Arylformamidase) (N-fo	615.322	2	23.24	109557	23.14	186670	2.21338
HYADVLR	KFase	Kynurenine formamidase (KFA) (KFase) (EC 3.5.1.9) (Arylformamidase) (N-fo	437.233	2	16.26	45711	16.22	111969	3.18199
IGTSEPIGR	Syt1	Synaptotagmin 1, isoform H	465.256	2	15.84	896749	15.87	539143	0.78101
TLSPVFNETFTFK	Syt1	Synaptotagmin 1, isoform H	765.896	2	45.21	1109199	44.93	1551467	1.817
AAYFGFYDTAR	sesB	Stress-sensitive B, isoform E	641.299	2	36.23	8457992	36.06	4781556	0.73439
DFAAGGISAAVSK	sesB	Stress-sensitive B, isoform E	597.312	2	28.61	8645959	28.51	9889000	1.4858
GFGVSVQGIIIYR	sesB	Stress-sensitive B, isoform E	704.901	2	45.35	4833482	45.12	2059585	0.55353
SDGIVGLYR	sesB	Stress-sensitive B, isoform E	490.264	2	28.91	1147390	28.83	342684	0.38798
TAVAPIER	sesB	Stress-sensitive B, isoform E	428.748	2	16.27	5420824	16.21	2687378	0.644
AQELWELIVK	up	Upheld, isoform T	614.85	2	48.34	857786	48.15	594529	0.90036
DAGVLGLSSAAMER	up	Upheld, isoform T	696.843	2	30.26	1579477	30.2	1875543	1.54254
IKPLAIEGFGEAK	up	Upheld, isoform T	458.266	3	33.5	4523125	33.37	3961091	1.13762
ISLSFR	up	Upheld, isoform T	361.713	2	30.22	1860854	30.16	1791066	1.25032
ASGGPNALLPAVGK	Hsp27	Heat shock protein 27, isoform B	626.356	2	31.22	28381	31.28	8197708	375.221
DGFQVCMDVSQFKPNELTVK	Hsp27	Heat shock protein 27, isoform B	781.377	3	43.41	309810	43.2	1758866	7.37496
DGFQVCMDVSQFKPNELTVK	Hsp27	Heat shock protein 27, isoform B	786.708	3	39.05	29103	38.58	4310963	192.424
ELDHDYR	Hsp27	Heat shock protein 27, isoform B	474.215	2	10.24	25905	10.22	309944	15.5425
GFDPNEVVSTVSSDGVLTLK	Hsp27	Heat shock protein 27, isoform B	1032.53	2	49.31	578434	48.23	34145948	76.6845
GFDPNEVVSTVSSDGVLTLK	Hsp27	Heat shock protein 27, isoform B	688.688	3	49.28	243916	48.23	17596958	93.7173
IVQIQQTGPAHLSVK	Hsp27	Heat shock protein 27, isoform B	809.967	2	26.16	0	26.15	7420055	#DIV/0!
IVQIQQTGPAHLSVK	Hsp27	Heat shock protein 27, isoform B	540.314	3	26.32	125560	26.23	62417060	645.765
LLLPNTLGLGR	Hsp27	Heat shock protein 27, isoform B	583.866	2	42.95	0	42.98	26755810	#DIV/0!
MSIIPLLHLAR	Hsp27	Heat shock protein 27, isoform B	653.389	2	60.18	0	59.42	2492957	#DIV/0!
VVDNTVVVEGK	Hsp27	Heat shock protein 27, isoform B	579.822	2	18.32	82265	18.27	3377383	53.332
ILPLDGLQAGYR	norpA	1-phosphatidylinositol 4,5-bisphosphate phosphodiesterase (EC 3.1.4.11)	658.372	2	39.05	1848340	38.89	1619786	1.13841

NDIEELFTSITK	norpA	1-phosphatidylinositol 4,5-bisphosphate phosphodiesterase (EC 3.1.4.11)	705.362	2	50.41	647218	50.1	519426	1.04255
VVLPDLAVLR	norpA	1-phosphatidylinositol 4,5-bisphosphate phosphodiesterase (EC 3.1.4.11)	547.85	2	45.88	627980	45.7	724721	1.49916
AGFAGDDAPR	Act5C	Actin 5C, isoform E	488.728	2	15.06	5346544	15.03	6072874	1.47551
AVFPSIVGRPR	Act5C	Actin 5C, isoform E	599.856	2	30.18	3527039	29.97	6233553	2.29587
DSYVGDEAQSK	Act5C	Actin 5C, isoform E	599.765	2	11.94	533801	12.02	513658	1.25002
IWHHTFYNELR	Act5C	Actin 5C, isoform E	505.921	3	25.48	1669489	25.49	2792816	2.17311
SYELPDGQVITIGNER	Act5C	Actin 5C, isoform E	895.95	2	39.17	5791673	39	8517636	1.91046
SYELPDGQVITIGNER	Act5C	Actin 5C, isoform E	597.635	3	39.17	427938	38.97	800279	2.42931
TTGIVLDSGDGVSHTVPIYEGYALPI	Act5C	Actin 5C, isoform E	1051.22	3	45.78	1749429	45.58	1016220	0.75459
VAPEEHPVLLTEAPLNPK	Act5C	Actin 5C, isoform E	977.536	2	34.25	620752	34.11	748708	1.56681
VAPEEHPVLLTEAPLNPK	Act5C	Actin 5C, isoform E	652.026	3	34.21	12031327	34.11	16999678	1.83548
IEDVTPIPSDSTR	RpS14b	GEO12513p1 (Ribosomal protein S14b, isoform B)	715.362	2	24.62	3641721	24.6	2484539	0.88626
TLGITALHIK	RpS14b	GEO12513p1 (Ribosomal protein S14b, isoform B)	533.835	2	32.9	2242098	32.75	2243891	1.30008
TLGITALHIK	RpS14b	GEO12513p1 (Ribosomal protein S14b, isoform B)	356.225	3	32.86	2809070	32.75	2994782	1.38492
ILEDSPNAR	Gs2	Glutamine synthetase (EC 6.3.1.2)	507.764	2	13.95	1451117	13.97	865640	0.77492
TLDFIPQSPK	Gs2	Glutamine synthetase (EC 6.3.1.2)	573.314	2	33.87	1671981	33.68	1493163	1.16011
YLSLPLQENIVQATYVWIDGTGEDL	Gs2	Glutamine synthetase (EC 6.3.1.2)	998.513	3	65.64	539196	65.12	224891	0.54181
AVYAHFPINCVTSENNTVIEIR	RpL9	Ribosomal protein L9, isoform C	849.76	3	40.81	1781795	40.62	1413499	1.03053
FLDGLYVSEK	RpL9	Ribosomal protein L9, isoform C	585.806	2	35.92	3763738	35.78	4648290	1.60434
GVTFGFQYK	RpL9	Ribosomal protein L9, isoform C	523.769	2	33.76	1916061	33.65	1180316	0.80022
TVCSHIENMIK	RpL9	Ribosomal protein L9, isoform C	666.326	2	25.86	3049601	26.46	614101	0.26159
VVTITGTR	RpL9	Ribosomal protein L9, isoform C	423.756	2	16.63	6749408	16.64	3559419	0.68507
VYSPHVLNLTLIDLPGLTK	shi	Shibire, isoform P	698.405	3	53.41	463587	53.25	477207	1.3372
AFIVKPIPSPEVIPVIVFINPK	rdgA	Diacylglycerol kinase (DAG kinase) (EC 2.7.1.107)	806.491	3	58.34	7861730	58.25	6537246	1.08019
AFIVKPIPSPEVIPVIVFINPK	rdgA	Diacylglycerol kinase (DAG kinase) (EC 2.7.1.107)	605.12	4	58.3	339552	58.29	268976	1.02903
FEESISYPK	rdgA	Diacylglycerol kinase (DAG kinase) (EC 2.7.1.107)	550.269	2	25.08	699628	25.06	249796	0.46381
FQHLLNPR	rdgA	Diacylglycerol kinase (DAG kinase) (EC 2.7.1.107)	512.788	2	19.99	2182798	19.93	880908	0.52425
GDVQVNPLEK	rdgA	Diacylglycerol kinase (DAG kinase) (EC 2.7.1.107)	549.793	2	22.75	2434720	22.76	798326	0.42595
LGNYAPIIVPPSWIVK	rdgA	Diacylglycerol kinase (DAG kinase) (EC 2.7.1.107)	884.014	2	52.63	3322956	52.44	1330008	0.51994
QVFDLTQGGPK	rdgA	Diacylglycerol kinase (DAG kinase) (EC 2.7.1.107)	595.314	2	28.22	3580063	28.2	2623698	0.95202
SFAAFSQR	rdgA	Diacylglycerol kinase (DAG kinase) (EC 2.7.1.107)	457.23	2	23.28	6655876	23.37	6106585	1.19183
TIPMQVDGEACR	rdgA	Diacylglycerol kinase (DAG kinase) (EC 2.7.1.107)	688.818	2	23.21	1029594	23.29	655361	0.82687
TIPMQVDGEACR	rdgA	Diacylglycerol kinase (DAG kinase) (EC 2.7.1.107)	696.816	2	17.7	628084	17.41	337537	0.69811
VKPSVIEIELLNK	rdgA	Diacylglycerol kinase (DAG kinase) (EC 2.7.1.107)	741.451	2	38.07	1106361	37.96	617198	0.72469
VKPSVIEIELLNK	rdgA	Diacylglycerol kinase (DAG kinase) (EC 2.7.1.107)	494.636	3	38.07	3645006	37.92	2591446	0.92356
VSQDWCFIDSCTAEHYFR	rdgA	Diacylglycerol kinase (DAG kinase) (EC 2.7.1.107)	774.33	3	41.88	500902	41.72	464633	1.20498
VTPNDDVTDDHVDR	rdgA	Diacylglycerol kinase (DAG kinase) (EC 2.7.1.107)	533.241	3	14.64	1483070	14.56	900190	0.78849
LGAGAGTGYPYYNTR	CG2157	Uncharacterized protein, isoform B	780.876	2	24.59	230436	24.56	514144	2.89839
AFIVKPIPSPEVIPVIVFINPK	rdgA	Diacylglycerol kinase (DAG kinase) (EC 2.7.1.107)	806.491	3	58.34	7861730	58.25	6537246	1.08019
AFIVKPIPSPEVIPVIVFINPK	rdgA	Diacylglycerol kinase (DAG kinase) (EC 2.7.1.107)	605.12	4	58.3	339552	58.29	268976	1.02903
FEESISYPK	rdgA	Diacylglycerol kinase (DAG kinase) (EC 2.7.1.107)	550.269	2	25.08	699628	25.06	249796	0.46381
FQHLLNPR	rdgA	Diacylglycerol kinase (DAG kinase) (EC 2.7.1.107)	512.788	2	19.99	2182798	19.93	880908	0.52425
GDVQVNPLEK	rdgA	Diacylglycerol kinase (DAG kinase) (EC 2.7.1.107)	549.793	2	22.75	2434720	22.76	798326	0.42595
LGNYAPIIVPPSWIVK	rdgA	Diacylglycerol kinase (DAG kinase) (EC 2.7.1.107)	884.014	2	52.63	3322956	52.44	1330008	0.51994
QVFDLTQGGPK	rdgA	Diacylglycerol kinase (DAG kinase) (EC 2.7.1.107)	595.314	2	28.22	3580063	28.2	2623698	0.95202
SFAAFSQR	rdgA	Diacylglycerol kinase (DAG kinase) (EC 2.7.1.107)	457.23	2	23.28	6655876	23.37	6106585	1.19183
TIPMQVDGEACR	rdgA	Diacylglycerol kinase (DAG kinase) (EC 2.7.1.107)	688.818	2	23.21	1029594	23.29	655361	0.82687
TIPMQVDGEACR	rdgA	Diacylglycerol kinase (DAG kinase) (EC 2.7.1.107)	696.816	2	17.7	628084	17.41	337537	0.69811
VKPSVIEIELLNK	rdgA	Diacylglycerol kinase (DAG kinase) (EC 2.7.1.107)	741.451	2	38.07	1106361	37.96	617198	0.72469
VKPSVIEIELLNK	rdgA	Diacylglycerol kinase (DAG kinase) (EC 2.7.1.107)	494.636	3	38.07	3645006	37.92	2591446	0.92356
VSQDWCFIDSCTAEHYFR	rdgA	Diacylglycerol kinase (DAG kinase) (EC 2.7.1.107)	774.33	3	41.88	500902	41.72	464633	1.20498
VTPNDDVTDDHVDR	rdgA	Diacylglycerol kinase (DAG kinase) (EC 2.7.1.107)	533.241	3	14.64	1483070	14.56	900190	0.78849
AQPGFGEDEVTIVLTGLPK	Yp3	Yolk protein 3, isoform B (EC 3.1.1.-) (EC 3.1.1.1)	986.025	2	50.78	511312	50.51	698432	1.77444

YFAESVRPGSER	Yp3	Yolk protein 3, isoform B (EC 3.1.1.-) (EC 3.1.1.1)	466.565	3	18.27	878476	18.27	1290612	1.90848	
DYGVLDEETGIPFR	Jafrac1	Thioredoxin peroxidase 1, isoform C (Thioredoxin peroxidase 1, isoform D) (E	805.888	2	42.29	1389123	42.06	1525411	1.42649	
SVEETLR	Jafrac1	Thioredoxin peroxidase 1, isoform C (Thioredoxin peroxidase 1, isoform D) (E	417.222	2	14.57	798318	14.56	716777	1.16635	
EAIVPFVPIFVEEADVIQGAK	eas	Easily shocked, isoform J (EC 2.7.1.32) (EC 2.7.1.82)	757.747	3	63.84	23869	61.84	116025	6.31451	
LVTAIDEADLAR	eIF2alpha	Eukaryotic translation initiation factor 2 subunit alpha, isoform B	643.851	2	31.83	315858	31.67	469918	1.93265	
AEQVVSQLSSDGVLTVSIPKPQAVE	Hsp26	Heat shock protein 26, isoform B	942.169	3	41.6	19465	41.36	1034803	69.0598	
DGFQVCMDVAQFKPSELNVK	Hsp26	Heat shock protein 26, isoform B	771.373	3	44.11	213799	44.1	1638807	9.95737	
DGFQVCMDVAQFKPSELNVK	Hsp26	Heat shock protein 26, isoform B	776.705	3	39.94	53212	39.69	2583608	63.0724	
IIQIQQVGPAHLNVK	Hsp26	Heat shock protein 26, isoform B	829.491	2	32.72	98730	32.53	9251943	121.732	
IIQIQQVGPAHLNVK	Hsp26	Heat shock protein 26, isoform B	553.33	3	32.72	645047	32.53	78215392	157.515	
MSLSTLLSLVDELQEPR	Hsp26	Heat shock protein 26, isoform B	644.343	3	65.46	0	65.23	11197	#DIV/0!	
NDIHWPATAHVGK	Hsp26	Heat shock protein 26, isoform B	482.581	3	20.47	18499	23.18	478607	33.6088	
SPIYELGLGLHPHSR	Hsp26	Heat shock protein 26, isoform B	838.449	2	33.39	144972	33.4	4457974	39.9462	
SPIYELGLGLHPHSR	Hsp26	Heat shock protein 26, isoform B	559.302	3	33.64	373200	33.4	49626936	172.742	
SPIYELGLGLHPHSR	Hsp26	Heat shock protein 26, isoform B	419.728	4	33.54	257619	33.4	45925152	231.577	
VVDDSILVEGK	Hsp26	Heat shock protein 26, isoform B	587.322	2	27.24	288145	27.53	4657387	20.9968	
YVLPLGTQQR	Hsp26	Heat shock protein 26, isoform B	587.833	2	30.91	472461	30.72	95333624	262.121	
DSQGHVFATR	RpS4	40S ribosomal protein S4	373.184	3	13.5	848798	13.47	498993	0.76368	
ESLPLLIFLR	RpS4	40S ribosomal protein S4	600.871	2	59.87	978242	59.6	249332	0.3311	
GNKPYISLPK	RpS4	40S ribosomal protein S4	372.885	3	24.24	1063638	24.28	968662	1.18304	
GVPFLVTHDGR	RpS4	40S ribosomal protein S4	599.322	2	29.44	2161831	29.36	2769935	1.66445	
LGGVFAPRPSTGPHK	RpS4	40S ribosomal protein S4	507.616	3	20.14	336675	20.13	222325	0.85783	
LGGVFAPRPSTGPHK	RpS4	40S ribosomal protein S4	380.964	4	20.18	928142	20.13	480847	0.673	
LSIAEER	RpS4	40S ribosomal protein S4	409.224	2	17.33	1731761	17.37	1338598	1.00412	
LTNVFIIGK	RpS4	40S ribosomal protein S4	502.811	2	37.72	3811188	37.56	3884472	1.32402	
TGEFFR	RpS4	40S ribosomal protein S4	378.687	2	22.32	2231185	22.28	2138263	1.24494	
VGTVVNR	RpS4	40S ribosomal protein S4	372.722	2	10.66	487079	10.77	324263	0.86481	
FLSQPFQVAEVFTGHAGK	ATPsynbe	ATP synthase subunit beta (EC 7.1.2.2)	655.007	3	47.1	661096	46.95	927753	1.82301	
LVLEVAQHLGENTVR	ATPsynbe	ATP synthase subunit beta (EC 7.1.2.2)	839.468	2	34.87	556208	34.71	748475	1.74808	
ILEDSPNAR	Gs2	Glutamine synthetase (EC 6.3.1.2)	507.764	2	13.95	1451117	13.97	865640	0.77492	
TLDFIPQSPK	Gs2	Glutamine synthetase (EC 6.3.1.2)	573.314	2	33.87	1671981	33.68	1493163	1.16011	
YLSLPLQENIVQATYVWIDGTGEDL	Gs2	Glutamine synthetase (EC 6.3.1.2)	998.513	3	65.64	539196	65.12	224891	0.54181	
AQCPIVER	RpS5a	Ribosomal protein S5a, isoform B (Ribosomal protein S5a, isoform C) (Ribosc	486.75	2	14.71	806831	14.72	445526	0.71732	
QAVDVSPLR	RpS5a	Ribosomal protein S5a, isoform B (Ribosomal protein S5a, isoform C) (Ribosc	492.777	2	23.02	630384	23.02	403036	0.83054	
TIAECLADELINAAK	RpS5a	Ribosomal protein S5a, isoform B (Ribosomal protein S5a, isoform C) (Ribosc	816.419	2	52.59	3303911	52.37	1805891	0.71004	
TIAECLADELINAAK	RpS5a	Ribosomal protein S5a, isoform B (Ribosomal protein S5a, isoform C) (Ribosc	544.615	3	52.55	1853788	52.37	881679	0.61784	
WSCDDVTVNDISLQDYISVK	RpS5a	Ribosomal protein S5a, isoform B (Ribosomal protein S5a, isoform C) (Ribosc	1179.05	2	47.03	482748	46.74	433432	1.16633	

Supplementary Table 3 CoRest-C binding sites
The regions indicated by 'Start' and 'End' represent the called peak region.
The results of the peak-calling are indicated as 'Q score' and 'Fold enrichment'.
'Gene Symbol' indicates the annotated genes onverlapped with the peaks, or most closely located to the peaks.

Chromosome	Start	End	Gene Symbol	Distance from the center of the peaks to TSS (bp)	Q score (-log10(q value)	Fold change	Located within 500 bp from the CBP-peaks	Located within 500 bp from the Rpd3-peaks
chr2L	18218	19017	l(2)gl	159	6.36773	3.80	-	-
chr2L	128989	129432	CG3164	62	13.0863	5.43	+	+
chr2L	155007	155694	ND-15	97	4.51847	3.31	-	-
chr2L	160626	161623	spen	1848	7.51194	4.27	+	+
chr2L	221993	222545	kis	134	7.25765	4.27	+	-
chr2L	277160	277654	CG17078	95	4.72558	3.33	+	+
chr2L	377861	378197	al	69	9.607	5.58	+	+
chr2L	403954	404242	RpI135	163	4.81534	3.38	-	-
chr2L	414933	415214	ebi	22	8.60204	4.55	+	+
chr2L	478770	480420	cbt	602	5.62584	3.34	+	+
chr2L	490145	490499	ush	540	11.3163	5.26	+	-
chr2L	490145	490499	CR46258	540	11.3163	5.26	+	-
chr2L	735498	735952	Eaat2	6093	9.14311	5.14	+	-
chr2L	739959	740386	Eaat2	1557	19.8868	8.23	-	-
chr2L	850788	851212	drongo	19	10.4357	5.31	+	+
chr2L	865457	866512	snoRNA:Me28S-U123(	314	5.42437	3.75	+	+
chr2L	865457	866512	aru	54	5.42437	3.75	+	+
chr2L	866781	867498	aru	705	4.95707	3.36	+	+
chr2L	870495	871690	PNUTS	70	7.88864	4.21	+	-
chr2L	1118777	1119198	Pino	5	7.68006	4.23	+	+
chr2L	1157722	1158051	capt	329	5.20345	3.22	+	+
chr2L	1185971	1186370	CG5001	74	5.69393	3.91	+	-
chr2L	1611091	1611486	CG31935	259	6.13669	3.79	-	+
chr2L	1611091	1611486	CG14352	7	6.13669	3.79	-	+
chr2L	1714402	1714801	frtz	1220	5.91102	3.91	+	+
chr2L	1723699	1724530	Rim2	62	6.36773	3.92	+	+
chr2L	1728507	1728829	Rrp40	6	4.10003	3.15	+	-
chr2L	1729665	1730023	CG31937	54	6.87287	3.95	+	-
chr2L	1911363	1911876	CG7337	82	6.07228	3.88	+	+
chr2L	2009614	2010016	CG4238	93	11.246	5.23	+	-
chr2L	2037562	2038242	Su(dx)	8	4.74497	3.15	+	+
chr2L	2162575	2163021	aop	1202	4.53832	3.32	+	-
chr2L	2168859	2169278	aop	7442	5.4164	3.87	+	+
chr2L	2192255	2192536	CG10874	43	5.87999	3.46	-	+
chr2L	2193271	2193918	CG34172	748	13.5869	5.79	+	+
chr2L	2196402	2197363	CG34172	730	13.2231	5.21	+	+
chr2L	2200073	2200620	CG34172	2462	10.1441	5.01	+	+
chr2L	2202075	2203628	CG31668	2806	24.5937	7.85	+	+
chr2L	2221901	2222306	papi	41	4.14378	3.08	+	+
chr2L	2362885	2363598	Rab5	475	4.06253	3.04	+	+
chr2L	2374485	2374924	CG3609	41	9.10259	4.29	+	+
chr2L	2374485	2374924	CG15390	44	9.10259	4.29	+	+
chr2L	2376179	2376591	Atxn7	71	26.9117	8.77	+	+
chr2L	2428238	2428797	dpp	166	6.13849	4.18	+	+
chr2L	2492567	2493182	Slh	116	5.34846	3.21	+	+
chr2L	2492567	2493182	oaf	167	5.34846	3.21	+	+
chr2L	2769937	2770305	colt	30	5.4164	3.51	-	-
chr2L	2769937	2770305	CG2862	98	5.4164	3.51	-	-

chr2L	2874348	2875048	CG31694	7	6.32122	4.02	+	+
chr2L	2874348	2875048	CG8814	407	6.32122	4.02	+	+
chr2L	2878212	2878586	Prx6005	102	8.00356	4.04	-	+
chr2L	2878212	2878586	CG31950	9	8.00356	4.04	-	+
chr2L	2878931	2879286	betaggt-II	3	12.4006	5.29	-	+
chr2L	2884991	2885350	NTPase	40	6.02165	3.85	+	+
chr2L	2885690	2886015	NTPase	13	7.94962	4.36	+	+
chr2L	2885690	2886015	lilli	91	7.94962	4.36	+	+
chr2L	3465994	3466548	msl-2	93	9.69613	5.10	-	+
chr2L	3465994	3466548	ND-PDSW	49	9.69613	5.10	-	+
chr2L	3662332	3662706	CG34340	242	4.37187	3.13	+	-
chr2L	3713037	3713455	CG31955	24	5.3399	3.70	+	+
chr2L	3713037	3713455	CG2818	230	5.3399	3.70	+	+
chr2L	3771731	3772342	bowl	40	10.6764	5.43	+	+
chr2L	3772842	3773195	bowl	970	6.15777	4.35	+	-
chr2L	3778612	3778870	bowl	169	5.64314	4.01	+	-
chr2L	3786256	3787213	bark	75	8.45273	4.92	+	-
chr2L	3872290	3872743	CG31957	57	7.72842	4.38	-	+
chr2L	4187680	4188001	CG31961	12	5.32495	3.37	-	-
chr2L	4221419	4221890	ft	44	4.82013	3.39	+	-
chr2L	4333187	4333468	Atet	584	7.88864	4.09	+	+
chr2L	4333740	4334230	Atet	78	9.3363	5.24	+	+
chr2L	4340184	4340806	Atet	1602	5.34846	3.59	+	-
chr2L	4448719	4449024	MFS18	227	8.01306	4.84	-	-
chr2L	4458094	4458416	RpL27A	100	4.59498	3.16	-	+
chr2L	4594819	4595216	dpy	64	12.9819	5.69	+	+
chr2L	4614143	4614869	dpy	216	5.3399	3.70	+	+
chr2L	4810169	4810731	CR45297	1729	5.54211	4.46	+	+
chr2L	4811434	4812046	CR45297	94	8.67696	4.46	+	+
chr2L	4973794	4974248	l(2)05714	115	10.9463	5.57	-	+
chr2L	4973794	4974248	mRpL28	61	10.9463	5.57	-	+
chr2L	5000254	5000531	Rtnl1	72	4.49913	3.30	+	-
chr2L	5001158	5001609	Rtnl1	905	5.4978	3.85	+	-
chr2L	5009694	5009961	Rtnl1	11	5.12257	3.18	+	+
chr2L	5054789	5055792	CG14036	306	5.70738	3.57	-	-
chr2L	5054789	5055792	His3.3A	189	5.70738	3.57	-	-
chr2L	5096204	5096517	CG4230	4	13.0888	6.09	+	+
chr2L	5099788	5100323	ND-13A	4	9.12391	4.67	-	-
chr2L	5108337	5108607	Msp300	42	7.29631	4.43	+	+
chr2L	5301284	5301545	vri	1797	4.67589	3.84	+	-
chr2L	5325111	5325764	CR44985	1251	5.92284	3.80	+	+
chr2L	5326410	5326880	CR44985	247	7.09668	4.18	+	+
chr2L	5326410	5326880	Ncoa6	67	7.09668	4.18	+	+
chr2L	5441342	5441666	CR31647	2173	5.12257	3.96	-	-
chr2L	5799392	5800412	CG9171	39	6.59913	3.92	+	+
chr2L	5805242	5805506	CG11034	26	7.32245	4.17	+	-
chr2L	5835464	5835721	TrissinR	10847	5.83689	3.54	-	-
chr2L	5907669	5908055	bchs	617	6.44534	3.54	+	-
chr2L	5977314	5977827	chic	2449	5.09715	3.55	+	-
chr2L	5981461	5982205	eIF-4a	55	6.90281	3.96	+	+
chr2L	5986238	5986942	ifc	35	6.48358	4.11	-	+
chr2L	6037648	6038336	CG9117	104	6.147	3.69	-	-
chr2L	6037648	6038336	CG31643	318	6.147	3.69	-	-
chr2L	6078767	6079435	CR9162	743	11.1032	5.02	+	+
chr2L	6098887	6099303	CG9175	59	12.5821	5.52	+	+
chr2L	6341622	6341945	Cpr	5	5.79011	3.84	+	-
chr2L	6480055	6481048	Sec61alpha	325	19.1246	7.23	+	+
chr2L	6480055	6481048	Daxx	28	19.1246	7.23	+	+

chr2L	6480055	6481048	CR45463	30	19.1246	7.23	+	+
chr2L	6491037	6491307	CG31638	53	6.44806	3.96	+	+
chr2L	6610764	6611119	CG11050	27	10.7722	4.63	+	-
chr2L	6612561	6613024	CG11050	1214	4.97726	3.19	+	+
chr2L	6684048	6684619	Nhe3	42	8.00112	4.67	+	+
chr2L	6787262	6787949	nrv1	602	19.7228	7.48	+	+
chr2L	6918591	6918877	nop5	54	5.09715	3.34	+	+
chr2L	7010002	7010457	uif	194	6.57672	4.30	+	-
chr2L	7042081	7042519	milt	67	13.1724	5.46	+	-
chr2L	7306803	7307187	wg	172	6.13849	3.92	+	-
chr2L	7437363	7438363	CG5973	31	7.42025	3.88	+	+
chr2L	7438941	7439338	CG5973	1616	5.06525	3.77	-	-
chr2L	7496212	7496567	Ziz	40	6.61493	3.71	+	+
chr2L	7701451	7701761	Tep2	17	6.92889	4.51	+	+
chr2L	7702676	7702972	Tep3	34	11.0516	5.29	+	+
chr2L	7809435	7810072	cdc14	882	8.44855	4.35	+	+
chr2L	7827798	7828904	mts	556	11.5371	4.94	+	+
chr2L	7879109	7879761	spz3	273	4.89568	3.54	+	-
chr2L	7973637	7973921	Snoo	3338	4.29296	3.28	+	-
chr2L	7998697	7999171	Sirup	30	11.8109	5.49	+	+
chr2L	8042644	8042938	RpL36A	1	8.09036	4.43	-	+
chr2L	8156782	8157061	PAPLA1	220	7.52433	4.56	-	-
chr2L	8196966	8197433	CG8460	16	8.28003	4.83	-	-
chr2L	8307954	8308220	Ostgamma	141	7.44239	4.51	-	+
chr2L	8370384	8371068	CG17292	29	9.59942	5.21	+	+
chr2L	8370384	8371068	CR45341	112	9.59942	5.21	+	+
chr2L	8403398	8403732	emb	48	14.0562	5.99	+	+
chr2L	8415224	8416023	CG13398	449	9.18539	5.16	+	+
chr2L	8415224	8416023	Akap200	16	9.18539	5.16	+	+
chr2L	8449468	8449739	U26	26	4.54899	3.56	-	-
chr2L	8478069	8478954	d	503	5.56959	3.71	+	-
chr2L	8685914	8686347	PrBP	124	5.22851	3.63	-	-
chr2L	8685914	8686347	Trs23	151	5.22851	3.63	-	-
chr2L	8699573	8700191	Hnf4	192	5.79011	3.84	+	-
chr2L	8709049	8709586	Hnf4	203	8.37142	4.58	+	+
chr2L	8888293	8889093	CR44415	952	4.3399	3.42	-	-
chr2L	9387628	9387900	Aldh	227	6.96551	4.54	+	+
chr2L	9430875	9431434	FucTB	51	6.96551	4.54	-	+
chr2L	9430875	9431434	scat	403	6.96551	4.54	-	+
chr2L	9570192	9570490	CG3838	55	6.84115	4.05	+	-
chr2L	9678859	9679229	CR44150	7949	4.66658	3.64	-	-
chr2L	9699065	9699541	Pka-C1	52	13.3765	5.40	+	+
chr2L	9790633	9790969	CG4036	2	5.18403	3.39	+	+
chr2L	9947377	9947770	CG5853	72	7.91708	4.78	+	+
chr2L	10143217	10143979	CG4839	1367	25.1733	9.47	+	-
chr2L	10246973	10247249	chico	12	8.60204	4.55	+	-
chr2L	10334796	10335240	GATAd	1167	8.32052	4.70	+	+
chr2L	10506527	10507521	Myo31DF	101	15.5151	6.45	+	+
chr2L	10506527	10507521	CG6094	200	15.5151	6.45	+	+
chr2L	10661432	10662128	CG31871	474	17.4485	7.77	+	-
chr2L	10841971	10842626	CG6700	179	6.48117	3.86	+	+
chr2L	10976187	10976527	ATPsynG	12	11.0314	5.61	+	+
chr2L	11004093	11004428	RpL9	36	10.6791	5.80	-	+
chr2L	11106439	11106964	Reps	170	5.05193	3.42	+	+
chr2L	11106439	11106964	l(2)gd1	127	5.05193	3.42	+	+
chr2L	11155642	11155948	CG6287	0	7.36908	4.33	+	+
chr2L	11806300	11808218	crol	1119	10.3493	4.97	+	+
chr2L	11808765	11809331	crol	128	5.20345	3.22	+	+

chr2L	11948536	11948942	CG16964	757	7.41602	3.98	+	-
chr2L	11970870	11971358	CR43314	32	9.92817	5.22	+	+
chr2L	12003029	12003839	CG6746	1377	7.41602	3.98	+	+
chr2L	12004208	12005015	CG6746	2419	8.08878	4.57	+	+
chr2L	12005382	12006092	Rh5	2640	10.2459	5.22	+	+
chr2L	12007022	12007837	Rh5	602	11.9097	4.72	+	+
chr2L	12010219	12011178	Rh5	2136	14.959	5.73	+	+
chr2L	12025303	12025645	CR44587	204	7.21477	4.69	+	+
chr2L	12044721	12046297	CG6785	750	12.7778	5.60	+	+
chr2L	12044721	12046297	CG6770	168	12.7778	5.60	+	+
chr2L	12055692	12056427	JhI-21	32	9.67914	5.43	+	+
chr2L	12055692	12056427	RpL7-like	408	9.67914	5.43	+	+
chr2L	12055692	12056427	CG34164	430	9.67914	5.43	+	+
chr2L	12055692	12056427	RpL7-like	430	9.67914	5.43	+	+
chr2L	12108891	12109199	Rab6	97	9.0216	4.91	+	+
chr2L	12207710	12207992	CG31759	36	8.32052	4.70	+	-
chr2L	12418245	12418653	vir-1	3542	8.92434	5.37	+	-
chr2L	12434458	12434943	SC35	96	15.3456	6.38	+	+
chr2L	12434458	12434943	Elf	130	15.3456	6.38	+	+
chr2L	12520196	12520567	snoRNA:CG43051-a	12307	20.5171	7.83	+	-
chr2L	12529482	12529943	bun	11146	9.92817	5.22	+	+
chr2L	12718976	12719302	MRP	68	7.30083	4.04	+	+
chr2L	12974136	12975189	Vha68-2	1	4.95707	3.36	+	+
chr2L	13059830	13060523	CG9932	228	9.87653	4.89	+	+
chr2L	13156030	13156817	Pk34A	2123	5.75535	4.24	+	-
chr2L	13165407	13165749	Sirt1	20	4.59498	3.16	+	+
chr2L	13176557	13177298	Sfmbt	383	4.81682	3.49	-	+
chr2L	13176557	13177298	CG5439	19	4.81682	3.49	-	+
chr2L	13289890	13290277	CG31729	190	4.08231	3.42	+	+
chr2L	13549073	13549632	B4	57	10.7269	5.00	+	+
chr2L	13665415	13666281	CG18507	102	11.246	5.23	+	+
chr2L	13780501	13780803	p38b	51	10.4035	4.85	+	+
chr2L	13904246	13904763	Smg5	12	6.51044	3.67	+	+
chr2L	14022395	14022671	CR45352	525	4.89278	3.66	+	-
chr2L	14348252	14349004	mTTF	30	10.1156	5.31	-	+
chr2L	14385253	14385748	ppk	4371	8.72833	5.08	+	-
chr2L	14409517	14409833	elB	241	7.76958	3.93	+	-
chr2L	14409517	14409833	CR44733	36	7.76958	3.93	+	-
chr2L	15056984	15057309	ck	698	6.87287	4.19	+	+
chr2L	15060492	15060878	TfIIS	30	6.02165	3.85	-	+
chr2L	15498804	15499602	lace	186	8.47464	4.49	+	+
chr2L	15762377	15762932	Gli	52	12.4179	5.77	+	+
chr2L	15912400	15912903	CR44868	19	5.47582	3.55	+	-
chr2L	16268111	16268558	CG4935	6145	8.67696	4.46	+	-
chr2L	16269209	16269844	CG4935	7322	6.85483	3.73	+	-
chr2L	16272794	16273059	CG4935	10762	4.48694	3.40	+	+
chr2L	16287415	16287939	crp	7	11.1564	4.91	+	+
chr2L	16451012	16451278	Idgf3	73	5.36976	3.60	+	+
chr2L	16491159	16491641	Tpr2	216	10.1156	5.31	+	-
chr2L	16521415	16521926	CG5953	1628	7.8286	4.90	+	+
chr2L	16525971	16526469	mir-4943	2284	7.81438	4.30	+	-
chr2L	16532639	16533786	CG5953	663	9.12391	4.67	+	+
chr2L	16727536	16727912	VhaSFD	169	5.87999	3.46	+	-
chr2L	16880985	16881413	CG31743	36	5.05193	3.42	+	+
chr2L	16888342	16889316	tweek	16	9.22829	4.86	+	+
chr2L	16888342	16889316	CR44392	1	9.22829	4.86	+	+
chr2L	17427657	17428458	Dif	5	7.29631	4.29	+	+
chr2L	17500960	17501269	CG6412	54	5.32495	3.37	-	-

chr2L	18152193	18152622	Socs36E	25	6.25604	3.75	+	+
chr2L	18455643	18456411	let-7-C	473	9.76167	5.30	+	+
chr2L	18484264	18484601	CG10283	6	4.95707	3.36	+	+
chr2L	18572784	18573304	Pde11	816	7.14896	4.35	+	+
chr2L	18590756	18591111	CG15160	75	7.59497	4.60	-	+
chr2L	18677025	18678099	CG31751	374	6.8328	4.04	+	+
chr2L	18695265	18695918	CG10383	79	10.2459	4.78	+	-
chr2L	18695265	18695918	CG10338	18	10.2459	4.78	+	-
chr2L	18732531	18733077	CG10348	105	5.03438	3.63	+	+
chr2L	18753131	18753766	CG15167	13150	6.61192	4.05	+	-
chr2L	18810555	18811333	CG42502	151	8.44541	5.09	+	+
chr2L	18810555	18811333	CG10570	151	8.44541	5.09	+	+
chr2L	18810555	18811333	CG42502	115	8.44541	5.09	+	+
chr2L	18810555	18811333	CG10570	115	8.44541	5.09	+	+
chr2L	18830887	18831423	CG17597	11	8.63045	4.32	+	-
chr2L	18830887	18831423	ScpX	86	8.63045	4.32	+	-
chr2L	18839186	18839669	CG10600	349	6.19484	3.95	+	+
chr2L	18881358	18881644	tup	232	5.91102	3.91	+	+
chr2L	19003165	19003616	CG31793	38	19.5326	7.61	-	-
chr2L	19009071	19009581	RpL30	38	8.00766	3.94	+	+
chr2L	19054050	19054614	CG10492	321	12.7778	5.60	+	+
chr2L	19119985	19120411	Ddc	35	5.68721	3.66	+	-
chr2L	19158182	19158831	brat	218	6.8328	3.92	+	+
chr2L	19190247	19190555	drl	88	9.19661	5.00	+	+
chr2L	19422441	19422822	Pax	895	7.08802	4.46	+	-
chr2L	19426514	19426820	Pax	92	5.12257	3.57	+	-
chr2L	19508476	19508907	CG10188	171	5.5796	3.61	+	+
chr2L	19572268	19572650	spi	76	5.14201	3.27	+	+
chr2L	19865838	19866275	sick	147	5.37529	3.49	+	-
chr2L	19887543	19887840	CG10481	4902	13.5734	6.52	-	-
chr2L	19895094	19895515	sick	660	5.79011	3.72	+	-
chr2L	20084474	20085065	fok	451	6.95804	4.11	+	+
chr2L	20416937	20417552	Arpc2	18	9.71773	4.95	+	+
chr2L	20428851	20429298	CG31688	75	6.16217	4.06	+	-
chr2L	20656117	20656449	bru	17	4.16717	3.30	-	+
chr2L	20656117	20656449	CG2614	230	4.16717	3.30	-	+
chr2L	20758198	20758499	dia	218	6.53216	4.47	+	+
chr2L	20787466	20787727	Pomp	37	6.70936	3.98	-	+
chr2L	20793190	20794253	vari	44	11.8109	5.49	+	+
chr2L	20810943	20811473	CG9328	1258	5.69073	3.91	-	-
chr2L	20821922	20822313	CG9331	755	4.00061	3.09	+	-
chr2L	20823808	20824171	CG9331	428	10.1621	5.34	+	+
chr2L	20829865	20830211	CG31673	451	7.91904	4.63	+	-
chr2L	20922759	20923070	Mtp	65	5.84855	3.88	+	-
chr2L	21158507	21159155	CG9249	271	4.82977	3.62	-	-
chr2L	21158507	21159155	CG9248	21	4.82977	3.62	-	-
chr2L	21174836	21178612	caRNA:PsiU2-38.40.4	291	60.2138	16.78	+	+
chr2L	21174836	21178612	CR44911	942	60.2138	16.78	+	+
chr2L	21309348	21310227	Mondo	92	5.09715	3.55	+	+
chr2L	21314225	21314758	crc	19	8.17404	4.35	+	-
chr2L	22018870	22019307	tio	57	8.13971	4.21	+	-
chr2L	22018870	22019307	CR33987	57	8.13971	4.21	+	-
chr2R	870217	870586	CG40439	15548	101.096	11.79	-	+
chr2R	6036074	6036591	SCAP	114	7.45333	4.24	+	+
chr2R	6132826	6133990	EcR	1855	12.0441	5.15	+	+
chr2R	6151045	6151500	EcR	5852	6.6001	3.81	+	-
chr2R	6241853	6242304	Pld	8	5.75988	3.82	+	-
chr2R	6628150	6628630	CG33919	3931	5.81596	3.86	+	-

chr2R	6632325	6633291	Vha16-1	43	6.02268	3.74	+	+
chr2R	6647813	6649304	Debcl	516	5.5796	3.61	+	-
chr2R	6662714	6663238	Adf1	767	15.9765	6.29	+	+
chr2R	6741744	6742514	phtf	17	7.19385	4.11	+	+
chr2R	6872755	6873096	coro	242	5.31777	3.46	+	+
chr2R	6878255	6878798	Spn42Da	23	5.70738	3.57	+	+
chr2R	6923219	6923532	CG3358	18	11.8116	5.33	+	-
chr2R	6992499	6992856	Tsp42Ea	45	8.97314	4.60	+	+
chr2R	6992499	6992856	CG30159	40	8.97314	4.60	+	+
chr2R	6992499	6992856	Tsp42Ea	40	8.97314	4.60	+	+
chr2R	6992499	6992856	CG30159	40	8.97314	4.60	+	+
chr2R	6992499	6992856	Tsp42Ea	40	8.97314	4.60	+	+
chr2R	7045665	7046114	Tsp42El	165	4.58751	3.36	+	+
chr2R	7150565	7150924	pk	64	7.56768	4.74	+	+
chr2R	7300631	7301004	CG12164	10516	11.2057	6.09	+	-
chr2R	7387117	7387457	Dhx15	32	8.01338	4.15	-	+
chr2R	7418520	7418847	so	699	8.53386	4.66	+	+
chr2R	7464677	7465080	CG11127	1386	4.59814	3.47	+	-
chr2R	7477574	7477940	Aldh-III	589	7.07825	4.31	+	-
chr2R	7483395	7486102	wech	1931	22.9227	8.67	-	-
chr2R	7528985	7529379	Drat	123	9.5651	5.03	+	+
chr2R	7533560	7533949	Cyt-b5	251	5.90416	3.67	+	+
chr2R	7566604	7567141	boca	106	15.1723	6.13	+	+
chr2R	7566604	7567141	Vps13	36	15.1723	6.13	+	+
chr2R	7698885	7699640	LRR	479	12.9107	6.00	+	+
chr2R	7698885	7699640	CG30493	129	12.9107	6.00	+	+
chr2R	7698885	7699640	CG30496	118	12.9107	6.00	+	+
chr2R	7735432	7735690	CG18812	56	6.15082	4.19	+	+
chr2R	7767489	7767781	CG45093	8745	6.29928	4.14	+	-
chr2R	7767489	7767781	CG30497	8745	6.29928	4.14	+	-
chr2R	7812250	7812828	CG1882	40	11.378	4.88	+	-
chr2R	7962470	7962873	nito	228	4.24545	3.14	-	+
chr2R	7989679	7990041	ACC	120	9.5651	4.61	+	-
chr2R	7989679	7990041	Nup44A	24	9.5651	4.61	+	-
chr2R	8018545	8019173	CR45267	247	6.48358	4.11	+	+
chr2R	8068716	8069391	lig	330	4.77508	3.26	+	+
chr2R	8082823	8083420	sut1	1	6.48702	3.98	+	+
chr2R	8082823	8083420	slv	99	6.48702	3.98	+	+
chr2R	8131285	8131797	Pbp49	212	4.51847	3.12	+	+
chr2R	8131285	8131797	CG42516	212	4.51847	3.12	+	+
chr2R	8131285	8131797	Pbp49	212	4.51847	3.12	+	+
chr2R	8131285	8131797	Pabp2	105	4.51847	3.12	+	+
chr2R	8145382	8146482	Lpin	82	7.68006	4.23	+	+
chr2R	8328853	8329485	pdm3	1692	7.91708	4.78	+	-
chr2R	8608305	8608602	CG8642	5687	8.44541	5.09	+	-
chr2R	8696295	8696688	CG8738	34	4.13788	3.28	+	+
chr2R	8724100	8724595	gcl	33	4.49913	3.30	+	+
chr2R	9141106	9141388	CG44286	56	5.56646	3.71	+	-
chr2R	9148765	9149023	CG30345	229	4.37187	3.13	+	+
chr2R	9186089	9186341	Rab32	166	4.44932	3.17	+	+
chr2R	9251939	9252333	CG30343	146	11.8116	5.33	-	-
chr2R	9308273	9308685	Myd88	112	7.73054	4.25	+	+
chr2R	9426316	9426891	Pdk	80	6.30792	3.57	+	+
chr2R	9437884	9438133	ced-6	2907	4.02978	3.63	+	-
chr2R	9440708	9441180	ced-6	149	4.82013	3.39	+	+
chr2R	9445776	9446558	CR45321	260	11.7033	5.01	+	+
chr2R	9445776	9446558	Camta	9	11.7033	5.01	+	+
chr2R	9552810	9553117	CR43651	19	4.73151	3.06	+	+

chr2R	9553416	9554261	mir-14	36	7.54645	4.29	+	+
chr2R	9578769	9579081	CR46257	77	4.27117	3.26	+	+
chr2R	9578769	9579081	Not1	26	4.27117	3.26	+	+
chr2R	9593978	9594416	CG1902	204	12.2553	5.87	+	+
chr2R	9593978	9594416	CG30338	204	12.2553	5.87	+	+
chr2R	9834949	9835286	hebe	1560	6.13849	3.92	+	-
chr2R	9861303	9861859	PCB	161	4.36525	3.32	+	-
chr2R	9903318	9904297	CG1441	707	10.9077	5.93	+	-
chr2R	9910747	9911012	Etf-QO	19	5.79011	3.84	+	-
chr2R	9958143	9958909	Mef2	591	6.22782	3.97	+	+
chr2R	9959503	9960126	Pal1	211	6.25604	3.75	+	+
chr2R	9959503	9960126	CG15863	27	6.25604	3.75	+	+
chr2R	9959503	9960126	Pal1	318	6.25604	3.75	+	+
chr2R	9988269	9988820	TER94	692	6.02165	3.85	+	+
chr2R	10025047	10026214	Pka-R2	565	6.13669	3.91	+	+
chr2R	10100405	10102036	14-3-3zeta	447	7.81438	4.30	+	+
chr2R	10110843	10111198	Pfk	288	4.59498	3.16	+	+
chr2R	10134942	10135341	gem	70	4.13788	3.28	+	-
chr2R	10159471	10160213	mlt	720	10.2575	5.07	+	+
chr2R	10192264	10192588	KCNQ	59	5.01948	3.50	+	-
chr2R	10475230	10475876	whd	95	5.01885	3.40	+	+
chr2R	10554065	10554387	psq	3652	7.72842	4.38	+	-
chr2R	10645518	10645873	Elp2	72	6.96631	4.12	-	+
chr2R	10781696	10782078	CG33144	831	5.26331	3.54	+	-
chr2R	10809448	10809924	CG12935	77	7.87448	4.46	-	+
chr2R	10823250	10823924	CG12343	270	8.53386	4.66	-	+
chr2R	10823250	10823924	CG12325	48	8.53386	4.66	-	+
chr2R	10874052	10874894	CG30015	8	9.12391	4.67	+	+
chr2R	11066520	11066819	CG43188	19004	4.65183	3.63	-	-
chr2R	11149778	11150265	CG12391	35	6.71758	3.87	-	-
chr2R	11175626	11176152	shn	2196	6.29592	4.14	+	-
chr2R	11204976	11205356	shn	451	5.60713	3.52	+	-
chr2R	11379460	11380086	Tsp47F	765	6.15377	4.35	-	-
chr2R	11451767	11452902	E(Pc)	106	8.6267	5.02	+	+
chr2R	11547617	11547889	en	19600	4.52332	3.67	-	-
chr2R	11651571	11651948	CG9003	190	8.00356	4.04	+	-
chr2R	11651571	11651948	CG34228	50	8.00356	4.04	+	-
chr2R	11764192	11764916	CG13193	3759	5.94021	4.07	+	-
chr2R	11885392	11885764	CG13185	35	6.73958	4.12	-	+
chr2R	11891905	11893466	Ef1alpha48D	1116	10.313	4.68	+	+
chr2R	11891905	11893466	snoRNA:Me28S-A1322	115	10.313	4.68	+	+
chr2R	12037227	12037548	128up	55	4.89568	3.54	-	+
chr2R	12140472	12140751	CG8378	10	5.24882	3.90	-	-
chr2R	12168418	12169261	Oda	566	9.97354	4.93	+	-
chr2R	12168418	12169261	SmD3	324	9.97354	4.93	+	-
chr2R	12170222	12170830	mir-281-2	97	5.81596	3.86	+	+
chr2R	12170222	12170830	mir-281-1	121	5.81596	3.86	+	+
chr2R	12171138	12171675	Oda	181	6.02165	3.85	+	+
chr2R	12171138	12171675	CR46194	154	6.02165	3.85	+	+
chr2R	12343870	12344985	CG8839	201	10.4876	5.34	+	+
chr2R	12343870	12344985	ana3	566	10.4876	5.34	+	+
chr2R	12435756	12436092	DUBAI	8	5.42437	3.63	-	+
chr2R	12452950	12453452	Lac	76	12.013	5.27	+	-
chr2R	12461938	12462296	dgt5	5969	5.69073	3.91	+	-
chr2R	12528847	12529385	fra	35	4.45884	3.18	+	+
chr2R	12591310	12592314	Sin3A	1155	6.25152	3.86	+	+
chr2R	12593524	12593890	Amph	108	7.55162	4.17	+	-
chr2R	12593524	12593890	CR45279	116	7.55162	4.17	+	-

chr2R	12705069	12705397	CG42663	54	9.55589	5.19	+	+
chr2R	12755930	12756355	Taz	232	4.72558	3.33	+	+
chr2R	12916301	12916613	NAT1	344	5.57085	3.60	+	+
chr2R	12936211	12936775	CG13322	56	7.42562	4.10	+	+
chr2R	12955887	12956227	CG13321	2451	4.51847	3.12	+	-
chr2R	12990754	12991344	CG33798	2942	7.72842	4.38	+	+
chr2R	13015684	13016075	Su(z)2	18769	6.27495	3.65	+	-
chr2R	13168432	13168802	Spt-I	65	11.2867	5.75	+	-
chr2R	13429703	13430114	CG42321	140	10.6657	5.26	+	+
chr2R	13483020	13483428	arr	2	7.65493	4.48	+	+
chr2R	13494788	13495492	bbc	83	5.84855	3.88	+	+
chr2R	13545384	13545912	CG6145	111	10.0274	4.82	+	-
chr2R	13824080	13824850	fl(2)d	67	13.536	5.61	+	-
chr2R	13925326	13925641	shot	37	6.27495	3.65	+	-
chr2R	13942047	13942354	shot	30	4.53991	3.23	+	+
chr2R	13959457	13959820	Rpn13	586	4.92104	3.56	-	+
chr2R	14006779	14007272	mir-4978	947	10.6904	5.12	+	+
chr2R	14212298	14213036	Sox15	165	8.60204	4.55	+	+
chr2R	14270122	14270777	SelD	9	7.02504	4.02	+	+
chr2R	14270122	14270777	conv	79	7.02504	4.02	+	+
chr2R	14352149	14352522	CG8613	91	5.57085	3.83	-	+
chr2R	14513978	14514279	CG30197	22	8.48197	4.78	+	+
chr2R	14600873	14601126	Rpl1	211	4.08868	3.15	-	+
chr2R	14860481	14860866	U3-55K	135	4.27117	3.26	-	+
chr2R	14882035	14882343	Cyp6a20	45	6.50972	4.27	+	+
chr2R	15128223	15128953	chn	24	5.23108	3.89	+	+
chr2R	15511463	15512044	GalNAc-T1	30	17.5261	6.75	+	-
chr2R	15613294	15614784	CG42524	821	8.01716	4.53	+	+
chr2R	15675746	15676639	fus	99	6.73958	4.12	+	+
chr2R	15675746	15676639	CR45143	586	6.73958	4.12	+	+
chr2R	15735109	15735408	CG30089	252	7.84486	4.59	+	+
chr2R	15753983	15754548	CG30089	18949	4.29093	3.08	+	-
chr2R	15848536	15848892	tun	81	7.36908	4.47	+	+
chr2R	15861265	15862108	Gpo-1	83	11.3623	5.28	+	+
chr2R	15922511	15922787	sli	520	5.69073	3.91	-	-
chr2R	15951860	15952279	Strn-Mlck	128	8.07705	5.04	+	-
chr2R	16014712	16015037	Prosbeta1	47	8.97314	4.24	-	-
chr2R	16014712	16015037	Rrp42	73	8.97314	4.24	-	-
chr2R	16017612	16018402	CG8399	5	8.48197	4.78	+	+
chr2R	16098245	16098635	Dg	134	5.20345	3.22	+	-
chr2R	16102588	16103215	CG8414	471	6.30792	3.57	+	-
chr2R	16102588	16103215	Rho1	17	6.30792	3.57	+	-
chr2R	16129575	16130285	spin	4993	5.74412	3.49	+	-
chr2R	16138859	16139153	Got1	402	6.9485	3.98	+	+
chr2R	16196870	16197752	CG42837	40	12.803	5.78	+	+
chr2R	16196870	16197752	CG44243	40	12.803	5.78	+	+
chr2R	16336059	16336914	Hmgs	547	11.1801	5.20	+	-
chr2R	16336059	16336914	Vha44	1	11.1801	5.20	+	-
chr2R	16336059	16336914	Hmgs	123	11.1801	5.20	+	-
chr2R	16585695	16586283	RpLP2	43	5.37529	3.49	+	+
chr2R	16588894	16589356	CG8311	6	6.48702	3.76	+	-
chr2R	16818896	16819363	CR43417	181	8.01716	4.53	+	-
chr2R	16828829	16829391	CR43650	126	4.23076	3.14	+	-
chr2R	16830349	16831021	CR43650	218	6.71948	3.98	+	+
chr2R	17392239	17392870	CG10939	1917	8.23897	4.03	+	-
chr2R	17420041	17420854	l(2)k01209	185	5.6505	3.44	+	+
chr2R	17436318	17436794	CG18467	3784	7.94962	4.36	+	+
chr2R	17457346	17458052	CG14478	20	7.82408	4.89	+	+

chr2R	17469454	17469937	qkr54B	47	12.007	5.58	+	+
chr2R	17513780	17514308	CG4853	12	6.24359	4.25	-	+
chr2R	17547410	17548575	RpL18A	283	11.4464	5.65	+	+
chr2R	17547410	17548575	MESR4	23	11.4464	5.65	+	+
chr2R	17698261	17698517	rdgBbeta	15	4.57834	3.78	-	+
chr2R	17760903	17761777	CG5009	160	7.54642	3.84	+	+
chr2R	17789282	17789596	Ubc10	27	4.91748	3.44	+	+
chr2R	17800796	17801174	CR45270	50	8.37119	4.20	+	+
chr2R	17800796	17801174	grh	120	8.37119	4.20	+	+
chr2R	17823226	17823632	grh	80	4.57798	3.92	+	+
chr2R	17871931	17872228	Mapmodulin	102	4.77508	3.26	+	+
chr2R	18141469	18142206	pAbp	666	4.89278	3.43	+	+
chr2R	18183928	18184379	CG5721	86	8.6267	5.02	+	-
chr2R	18357494	18357861	sbb	358	8.53386	4.66	+	-
chr2R	18408795	18409114	GstE9	78	5.5796	3.61	+	-
chr2R	18440875	18441327	slim	76	6.02268	3.74	+	+
chr2R	18528733	18529086	CG10924	1457	7.78369	3.84	+	-
chr2R	18611470	18612057	GEFmeso	148	8.01306	4.53	+	+
chr2R	18687981	18688467	Sik3	27	8.23591	4.51	+	+
chr2R	18829499	18829795	CG15097	37	7.08208	4.45	+	+
chr2R	19253907	19254441	CG33453	827	9.74621	5.29	+	-
chr2R	19424856	19425475	hts	333	7.09903	3.95	+	+
chr2R	19424856	19425475	CalpA	124	7.09903	3.95	+	+
chr2R	19451109	19451800	betaTub56D	9	6.13669	3.79	+	+
chr2R	19462312	19462791	par-1	113	6.36773	3.80	+	-
chr2R	19468058	19468786	par-1	791	8.95443	4.59	+	-
chr2R	20118727	20119066	18w	7414	6.02165	3.85	-	-
chr2R	20531731	20532113	CR44651	71	4.9935	3.49	+	-
chr2R	20534161	20534529	CG18067	52	6.25964	3.99	+	+
chr2R	20551903	20552187	CG18065	29	5.69583	3.37	-	-
chr2R	20551903	20552187	CG13430	29	5.69583	3.37	-	-
chr2R	20618087	20618665	Hil	423	4.85366	3.30	+	-
chr2R	20618087	20618665	CG9945	12	4.85366	3.30	+	-
chr2R	20640751	20641173	maf-S	6	9.37364	5.09	-	+
chr2R	20670940	20671593	exu	150	10.1441	5.01	+	+
chr2R	20670940	20671593	CG30152	30	10.1441	5.01	+	+
chr2R	21000179	21000791	CG10543	168	7.19385	4.11	+	-
chr2R	21057566	21057953	cpa	58	11.1797	5.88	+	+
chr2R	21141946	21142261	Magi	140	5.23108	3.32	+	+
chr2R	21143521	21143982	Xbp1	129	6.84115	4.05	+	+
chr2R	21178794	21179145	Pu	176	13.5741	6.32	+	+
chr2R	21250917	21251693	ASPP	16	5.91104	3.68	+	-
chr2R	21295454	21296234	CG9752	51	12.8897	5.50	+	-
chr2R	21295454	21296234	CG42672	12	12.8897	5.50	+	-
chr2R	21323252	21324024	CG30394	402	9.97354	4.93	+	+
chr2R	21323252	21324024	dom	293	9.97354	4.93	+	+
chr2R	21365514	21365786	cv-2	13795	9.82691	5.71	+	-
chr2R	21382326	21382723	CG10795	1900	10.625	5.77	-	-
chr2R	21480772	21481185	Sdc	301	5.80675	3.62	+	+
chr2R	21501624	21502102	TAF1C-like	27	6.84122	3.93	+	-
chr2R	21501624	21502102	MESK2	337	6.84122	3.93	+	-
chr2R	21502426	21503008	MESK2	505	8.93304	4.72	+	-
chr2R	21535464	21535739	CG30286	1202	8.32052	4.70	+	-
chr2R	21612862	21613234	CG15673	45	9.00061	5.06	+	+
chr2R	21661971	21662428	pirk	86	6.87287	4.19	+	+
chr2R	21662947	21663538	pirk	235	10.9577	5.76	+	+
chr2R	21662947	21663538	CR44748	71	10.9577	5.76	+	+
chr2R	21702577	21703793	HmgZ	250	13.1208	5.75	+	+

chr2R	21704338	21704965	HmgZ	254	8.67696	4.46	+	+
chr2R	22060786	22061103	Snp	61	9.00104	4.36	+	-
chr2R	22311820	22312169	wdp	41	4.24601	3.25	+	+
chr2R	22317889	22318447	Gp150	277	4.91748	3.44	+	+
chr2R	22561791	22562346	px	13	20.0233	8.06	+	+
chr2R	22561791	22562346	CG11362	228	20.0233	8.06	+	+
chr2R	22570268	22571040	CG11362	8606	6.51367	4.13	+	-
chr2R	22641164	22641489	RpS24	119	6.13669	3.79	-	+
chr2R	22669217	22669533	CG13516	57	21.9012	8.70	+	+
chr2R	22686070	22686339	Ppa	2818	6.48117	3.86	+	+
chr2R	22688820	22689078	Ppa	57	7.59014	4.31	+	+
chr2R	22798747	22799188	blw	55	6.48117	3.86	+	+
chr2R	22853461	22854464	CG13531	467	10.5865	5.07	+	+
chr2R	22853461	22854464	RpL23	88	10.5865	5.07	+	+
chr2R	22895667	22896379	nahoda	17	7.59497	4.60	+	+
chr2R	22933691	22934563	CG3788	221	9.83027	5.17	+	+
chr2R	22998678	22998974	CG9896	74	5.62243	3.63	+	+
chr2R	23419368	23419886	CG9812	53	9.94006	5.07	+	+
chr2R	23432140	23432523	CG9815	189	4.62773	3.61	+	-
chr2R	23523521	23523949	RabX1	111	10.5424	5.05	+	+
chr2R	23523521	23523949	mi	131	10.5424	5.05	+	+
chr2R	23547664	23548506	wmd	54	8.23591	4.51	+	+
chr2R	23547664	23548506	levy	196	8.23591	4.51	+	+
chr2R	23564756	23565064	apt	15	9.00104	4.36	-	-
chr2R	23614698	23615025	CG4019	125	16.3675	7.47	+	-
chr2R	23711966	23712911	Sesn	1631	8.40087	4.74	+	+
chr2R	23713308	23714074	Sesn	124	5.24195	3.42	+	+
chr2R	23725974	23726554	CR45325	5017	4.06253	3.04	+	-
chr2R	23817681	23818698	CG4797	326	6.44897	4.09	+	-
chr2R	23871614	23872175	ken	1684	5.5796	3.84	+	+
chr2R	23872683	23873471	ken	2638	5.79209	3.61	+	+
chr2R	23898046	23898561	CG5339	8	5.09715	3.34	-	+
chr2R	23898046	23898561	Lpt	322	5.09715	3.34	-	+
chr2R	23978297	23979209	Sox14	353	26.5581	7.65	+	+
chr2R	23979702	23980220	Sox14	111	4.40661	3.24	+	+
chr2R	24026356	24026979	EbpIII	17	6.52753	4.14	+	+
chr2R	24342474	24342898	bs	187	8.48197	4.78	+	-
chr2R	24343588	24343930	bs	51	6.13669	3.79	-	-
chr2R	24402030	24402746	Slik	69	5.37529	3.49	+	+
chr2R	24571515	24571973	CG3511	270	4.68441	3.21	+	+
chr2R	24571515	24571973	Fcp1	75	4.68441	3.21	+	+
chr2R	24571515	24571973	CG3511	48	4.68441	3.21	+	+
chr2R	24580889	24581945	pio	84	7.36908	4.33	+	+
chr2R	24599695	24599959	ATPsynF	61	8.47464	4.49	+	+
chr2R	24599695	24599959	CG3548	64	8.47464	4.49	+	+
chr2R	24611609	24611888	Adck	6	4.60674	3.67	-	-
chr2R	24611609	24611888	CG4741	148	4.60674	3.67	-	-
chr2R	24611609	24611888	Adck	185	4.60674	3.67	-	-
chr2R	24669053	24669340	CG33228	13	6.58558	4.17	-	+
chr2R	24684813	24685387	Mmp1	61	5.97428	3.51	+	-
chr2R	24743668	24744083	Usp15-31	144	4.72492	3.62	+	-
chr2R	24767594	24768245	GstE12	142	9.76807	4.98	+	+
chr2R	24792849	24793673	Reg-5	121	6.84115	4.05	+	+
chr2R	24814374	24814916	Dll	31	4.63182	3.28	+	+
chr2R	24863773	24864039	CR44829	4544	4.3399	3.42	+	-
chr2R	24903697	24904105	RpL41	83	5.20345	3.22	+	-
chr2R	24927072	24927463	pain	992	8.18505	4.94	+	+
chr2R	24928040	24928527	pain	32	5.80675	3.62	+	+

chr2R	24940225	24940632	CG2811	45	8.24287	4.97	+	+
chr2R	24969423	24969892	RpL19	60	10.7476	4.87	+	+
chr2R	24969423	24969892	CG3776	70	10.7476	4.87	+	+
chr2R	24983201	24983845	emp	250	4.21193	3.03	+	+
chr2R	25011031	25011584	zip	52	9.69002	4.54	+	+
chr2R	25084029	25084640	gol	182	6.59913	3.92	+	-
chr3L	178389	178885	Vdup1	89	5.68721	3.66	+	-
chr3L	224111	225093	CG33229	84	7.29816	3.93	+	+
chr3L	224111	225093	CR42862	84	7.29816	3.93	+	+
chr3L	246412	247068	E(bx)	376	4.46194	3.38	+	+
chr3L	258169	258532	wac	30	6.49421	3.76	-	-
chr3L	292190	292723	RhoGEF3	240	6.36773	3.80	+	+
chr3L	319648	320010	fwd	73	7.50488	4.14	+	+
chr3L	340937	341689	mthl10	168	9.88804	5.04	+	+
chr3L	361409	361723	CG1233	139	7.00941	4.27	-	+
chr3L	540604	540865	klar	175	4.1344	3.25	+	+
chr3L	540604	540865	CG17180	61	4.1344	3.25	+	+
chr3L	564332	564711	Hipk	4837	7.59497	4.60	+	-
chr3L	603704	604042	CG13893	103	7.55162	4.17	+	+
chr3L	606524	607201	Reg-2	87	12.2846	6.07	+	+
chr3L	616417	617208	CR42719	414	7.65493	4.96	+	+
chr3L	627927	628890	CR43334	172	6.07228	3.88	+	+
chr3L	685409	685746	ebd1	4	6.84115	4.05	+	-
chr3L	753536	754244	hng3	1307	4.61041	3.37	+	-
chr3L	833021	833418	snoRNA:Psi18S-1086	23	5.4164	3.42	-	-
chr3L	833021	833418	CG13900	153	5.4164	3.42	-	-
chr3L	881562	882220	CG13907	48	9.12391	4.67	+	+
chr3L	895289	896062	CG34056	89	7.84551	4.74	+	+
chr3L	1204653	1204995	CG9119	37	5.00679	3.61	+	+
chr3L	1313561	1313996	CG9153	5	7.92301	3.81	+	-
chr3L	1334511	1335024	CG2211	329	7.68006	4.23	+	+
chr3L	1334511	1335024	l(3)02640	17	7.68006	4.23	+	+
chr3L	1517563	1518157	Psa	108	6.58558	3.92	+	-
chr3L	1568209	1568705	CG7879	253	6.03882	3.64	+	+
chr3L	1569019	1569339	CG12004	31	6.38648	4.06	+	+
chr3L	1576443	1576802	CG13917	98	6.19777	4.22	+	+
chr3L	1668596	1668858	RpL23A	82	8.17404	4.35	-	+
chr3L	1810411	1810676	GV1	33	8.96019	5.04	+	-
chr3L	1861638	1862294	Rap1	4	4.46194	3.38	+	+
chr3L	1865221	1865683	HBS1	211	5.22851	3.75	-	+
chr3L	1865221	1865683	CG12025	22	5.22851	3.75	-	+
chr3L	1884639	1885224	Aprt	5	8.90561	4.70	+	-
chr3L	1963928	1964664	mv	371	8.71301	4.91	-	+
chr3L	1963928	1964664	SCOT	91	8.71301	4.91	-	+
chr3L	2150264	2150679	zormin	1217	5.04262	3.52	+	-
chr3L	2151519	2151919	zormin	40	7.88522	3.98	+	-
chr3L	2388799	2389234	CG42787	208	6.63258	4.49	-	-
chr3L	2494396	2494728	CG45186	6	5.01948	3.50	+	-
chr3L	2531309	2531749	CG32295	4484	7.95686	4.65	-	-
chr3L	2554177	2554443	Spn	90	6.29592	4.14	+	+
chr3L	2589411	2590092	dos	56	4.81178	3.10	+	+
chr3L	2632723	2633238	MEP-1	709	22.8691	7.97	+	+
chr3L	2635032	2635879	MEP-1	296	4.14378	3.08	+	+
chr3L	2980819	2981427	CG2107	55	6.25604	3.75	-	-
chr3L	3038207	3038460	CG2162	14	5.32495	3.37	+	+
chr3L	3070658	3071363	CG32486	132	11.9284	5.39	+	+
chr3L	3070658	3071363	CG11486	161	11.9284	5.39	+	+
chr3L	3193187	3195585	Hsp83	198	8.90561	4.70	+	+

chr3L	3196011	3196754	CG14966	1000	5.23656	3.63	+	-
chr3L	3198836	3199451	CG32276	160	6.13669	3.79	+	+
chr3L	3198836	3199451	gry	202	6.13669	3.79	+	+
chr3L	3222795	3223072	RpL28	43	5.37529	3.49	-	+
chr3L	3336754	3337102	kst	81	7.81438	4.30	+	+
chr3L	3338481	3339063	kst	1539	15.463	7.03	+	+
chr3L	3418398	3418665	sty	589	12.2113	5.67	+	+
chr3L	3626006	3626466	dar1	156	13.0888	6.09	+	+
chr3L	3632189	3633161	dar1	6599	6.02165	3.85	+	+
chr3L	3787691	3788186	CG32264	99	4.95707	3.36	+	+
chr3L	3789123	3789452	CG32264	1303	10.5033	5.52	+	-
chr3L	3846037	3846426	Awh	29	6.7942	4.29	+	-
chr3L	4020977	4021595	Mul1	13	4.39075	3.34	-	-
chr3L	4092773	4093072	CG14990	309	10.5033	5.52	+	-
chr3L	4103644	4104162	Fit1	427	6.43458	3.84	+	+
chr3L	4228185	4228964	ImpL2	1359	10.8738	5.54	+	+
chr3L	4232182	4232568	ImpL2	3797	8.28003	4.83	+	-
chr3L	4233876	4234327	ImpL2	1980	10.5776	5.38	+	-
chr3L	4239498	4240020	CG14997	392	5.68721	3.66	+	+
chr3L	4253763	4254197	ago	35	5.05193	3.42	+	+
chr3L	4413842	4415413	DOR	289	11.8317	5.20	+	+
chr3L	4543875	4544188	CG11357	1904	5.4551	3.76	+	-
chr3L	4824581	4824837	Dhc64C	969	10.1878	4.89	+	+
chr3L	4825610	4826078	Dhc64C	91	8.26944	4.27	-	+
chr3L	4825610	4826078	Aats-leu	74	8.26944	4.27	-	+
chr3L	5145992	5146321	CG10671	68	8.24563	4.26	-	-
chr3L	5145992	5146321	Aats-ala-m	34	8.24563	4.26	-	-
chr3L	5355125	5355474	lama	48	10.3049	5.41	+	-
chr3L	5563670	5564046	Sucb	105	7.18203	3.98	-	+
chr3L	5563670	5564046	Sras	23	7.18203	3.98	-	+
chr3L	5577089	5577453	Msr-110	58	6.61192	4.05	+	+
chr3L	5610541	5610895	Eaf6	2389	5.22851	3.63	+	-
chr3L	5750609	5751697	DnaJ-1	738	7.09903	3.95	+	+
chr3L	5770215	5770897	scny	545	8.38038	4.58	+	+
chr3L	5869608	5869908	CG5568	1724	15.0827	7.06	+	-
chr3L	5902205	5902662	QC	95	7.95686	4.65	+	+
chr3L	5923535	5923827	Vap-33B	201	6.12636	4.18	+	+
chr3L	6184830	6185143	D19B	53	6.03882	3.64	+	-
chr3L	6728226	6728530	CR45417	113	5.33225	3.95	+	-
chr3L	6743587	6744140	dikar	605	25.2049	8.29	+	+
chr3L	6752132	6752666	velo	347	11.4263	5.31	+	+
chr3L	6977132	6977517	CR32385	1056	7.72614	4.83	+	-
chr3L	6979815	6980264	Galphai	79	7.65493	4.48	+	-
chr3L	7094658	7095398	form3	46	8.64661	4.87	+	+
chr3L	7134286	7134729	melt	68	11.8317	5.20	+	+
chr3L	7344888	7345333	CG8596	54	12.9481	6.21	+	+
chr3L	7360682	7361404	Sec63	684	11.0558	4.42	+	+
chr3L	7809673	7810191	CG32369	218	12.0387	5.76	+	+
chr3L	7864943	7865427	Pdp1	2363	4.8392	3.63	+	+
chr3L	7978923	7979335	nmo	70	9.94006	4.65	+	+
chr3L	8091688	8092019	CG7565	48	7.88864	4.09	+	-
chr3L	8151709	8152265	CG13676	304	5.12257	3.57	+	-
chr3L	8158529	8159040	CG42445	106	14.0823	6.56	-	-
chr3L	8186553	8187071	ERR	13	8.48501	4.64	+	+
chr3L	8186553	8187071	Atg18a	172	8.48501	4.64	+	+
chr3L	8309066	8309416	BI-1	315	4.87045	3.53	-	-
chr3L	8449799	8450616	Gug	85	6.61555	4.19	+	+
chr3L	8575806	8576141	CG6638	128	5.91102	3.91	-	-

chr3L	8576543	8576802	CG43078	88	4.89278	3.66	-	-
chr3L	8600752	8601125	Nelf-E	95	14.0777	5.68	-	-
chr3L	8600752	8601125	RpL14	31	14.0777	5.68	-	-
chr3L	8603894	8604255	CG5989	56	12.2553	5.87	+	+
chr3L	8675493	8676160	h	64	6.17125	3.60	+	+
chr3L	8766239	8766618	Fhos	13	7.80626	4.56	+	-
chr3L	8912414	8912677	Tsp66E	8	4.82013	3.39	+	-
chr3L	8912414	8912677	mfr	164	4.82013	3.39	+	-
chr3L	8913270	8913530	mfr	677	4.46194	3.38	+	-
chr3L	8997942	8998267	smg	130	5.87469	3.66	+	+
chr3L	8997942	8998267	CG5087	64	5.87469	3.66	+	+
chr3L	9041197	9041446	Doc1	20	4.39755	3.65	+	-
chr3L	9056443	9056809	Argk	981	12.2113	5.67	+	-
chr3L	9134274	9134735	bol	127	9.28024	4.74	+	-
chr3L	9134274	9134735	Dhpr	27	9.28024	4.74	+	-
chr3L	9335772	9336081	PGRP-LA	190	4.97968	3.59	+	+
chr3L	9349481	9349755	PGRP-LF	13	5.83689	3.54	+	+
chr3L	9358793	9359223	UGP	2	8.74927	4.63	+	+
chr3L	9358793	9359223	CG32039	65	8.74927	4.63	+	+
chr3L	9509170	9509674	CG3408	80	5.65147	3.64	+	+
chr3L	9511336	9511610	RpS9	50	4.74497	3.15	-	-
chr3L	9695033	9695591	CR45121	304	5.80675	3.62	+	+
chr3L	9695033	9695591	CG6767	95	5.80675	3.62	+	+
chr3L	9698322	9698617	Ubc4	159	5.72319	3.93	+	+
chr3L	9713802	9714825	SH3PX1	416	5.95149	3.81	+	+
chr3L	9713802	9714825	vsg	80	5.95149	3.81	+	+
chr3L	9853942	9854316	RasGAP1	1173	8.23591	4.51	+	+
chr3L	9854683	9855367	RasGAP1	432	8.63045	4.32	+	+
chr3L	9898263	9898578	CG6707	17	5.91102	3.91	-	+
chr3L	10325049	10325749	CR46006	1191	16.7495	6.80	+	-
chr3L	10332120	10332655	CR46006	85	9.71963	4.68	+	-
chr3L	10363956	10364230	CR46006	27	6.38228	3.70	+	-
chr3L	10630821	10631299	CG42535	92	9.81123	4.86	-	-
chr3L	10630821	10631299	CG34001	51	9.81123	4.86	-	-
chr3L	10630821	10631299	hay	51	9.81123	4.86	-	-
chr3L	10630821	10631299	CG34001	51	9.81123	4.86	-	-
chr3L	10665801	10666340	simj	1251	9.22216	5.36	+	+
chr3L	10775681	10776060	CG43245	4095	5.06126	3.65	+	+
chr3L	10867933	10868308	tna	1743	10.8979	5.38	+	+
chr3L	10993471	10993760	snoRNA:Me18S-G962	7881	6.51288	4.42	-	-
chr3L	11008961	11009537	klu	56	6.76943	4.14	+	+
chr3L	11010344	11011261	klu	1506	6.05096	4.13	+	+
chr3L	11062491	11062858	CG32069	4	4.7384	3.56	-	-
chr3L	11070389	11070803	Mocs1	9	7.75594	4.69	+	+
chr3L	11113458	11113846	Sod	25	5.91102	3.91	+	+
chr3L	11119627	11121292	FoxK	188	6.13849	3.92	+	+
chr3L	11119627	11121292	mRpL2	125	6.13849	3.92	+	+
chr3L	11210631	11210989	CG7600	87	4.89278	3.43	-	+
chr3L	11252114	11252493	scyl	100	5.68099	3.66	+	+
chr3L	11487507	11488052	chrb	201	7.06643	3.93	+	+
chr3L	11534537	11534862	Mob2	216	4.59498	3.16	+	-
chr3L	11552641	11553700	mir-4968	426	12.1726	5.34	+	-
chr3L	11552641	11553700	Mob2	7	12.1726	5.34	+	-
chr3L	11562206	11562842	Mob2	244	10.3493	4.97	+	-
chr3L	11562206	11562842	CG7394	195	10.3493	4.97	+	-
chr3L	11648500	11648781	CG7339	129	4.33525	3.20	-	+
chr3L	11696699	11697076	CG11652	16	6.17125	3.60	-	-
chr3L	11707877	11708560	CG11658	501	29.3999	10.08	+	+

chr3L	12081501	12081917	Sema-5c	63	7.23227	4.54	+	-
chr3L	12197572	12197872	CG11529	22	4.36525	3.32	+	-
chr3L	12408359	12409294	CG32103	52	8.90561	4.70	+	+
chr3L	12467983	12468381	eyg	86	9.87797	5.19	+	+
chr3L	12490078	12491267	CR45681	78	8.32052	4.70	+	+
chr3L	12490078	12491267	CG10660	461	8.32052	4.70	+	+
chr3L	12514694	12515273	sti	75	12.0348	5.76	+	+
chr3L	12514694	12515273	CR45168	119	12.0348	5.76	+	+
chr3L	12514694	12515273	tral	168	12.0348	5.76	+	+
chr3L	12526236	12527067	eIF-2beta	34	7.88206	4.61	-	+
chr3L	12580470	12580763	ara	58	5.37529	3.49	+	-
chr3L	12627762	12628135	CR32111	23	4.08868	3.15	+	-
chr3L	12692849	12693193	mirr	607	4.82013	3.39	+	+
chr3L	13036717	13036996	SRm160	4	6.00861	3.97	-	-
chr3L	13228449	13228873	caps	77	9.83808	4.37	+	-
chr3L	13235982	13236381	caps	7462	4.1784	3.20	+	-
chr3L	13389938	13390229	Poc1	44	5.14201	3.47	-	-
chr3L	13494488	13495556	Tgi	226	9.43589	4.82	+	+
chr3L	13497474	13498260	Tgi	2102	11.5649	5.54	+	+
chr3L	13502574	13502960	Spt20	62	12.6119	5.24	+	-
chr3L	13939124	13939491	CG32137	66	10.6455	5.98	+	+
chr3L	14004969	14006139	upSET	3184	7.65493	4.48	+	+
chr3L	14028553	14029107	Ptip	489	6.25152	3.86	+	+
chr3L	14053044	14053451	CG13484	241	7.78294	4.16	+	+
chr3L	14053044	14053451	CG32138	106	7.78294	4.16	+	+
chr3L	14071683	14072080	Pex1	42	11.6163	5.40	+	+
chr3L	14190332	14191002	nuf	66	9.06313	4.78	+	+
chr3L	14212316	14212690	CG7768	5701	15.3615	6.77	-	-
chr3L	14229796	14230107	nuf	1238	4.28201	3.70	-	-
chr3L	14409360	14409818	stwl	474	7.80626	4.56	+	-
chr3L	14427290	14427662	bbg	93	11.8109	5.49	+	+
chr3L	14476708	14477188	bbg	375	5.4551	3.76	+	-
chr3L	14537406	14537857	Mpcp	70	7.55162	4.17	+	+
chr3L	14544227	14544662	Gbs-70E	6403	5.37943	3.99	+	-
chr3L	14706605	14706961	ome	52	7.19385	4.11	+	+
chr3L	14785285	14785701	bmm	890	9.67245	4.93	+	+
chr3L	14804706	14805224	CG17839	18	4.14378	3.08	+	+
chr3L	14819714	14820036	CG17839	14739	7.09401	4.79	+	+
chr3L	15141831	15142559	CR45396	305	6.13669	3.79	+	+
chr3L	15143781	15144068	CR45397	126	6.94326	4.52	-	+
chr3L	15143781	15144068	CG32147	133	6.94326	4.52	-	+
chr3L	15532458	15532731	CG43248	1154	5.60713	3.52	+	-
chr3L	15982903	15983220	Arl1	97	5.79011	3.84	+	+
chr3L	16011216	16011886	mib1	196	5.83689	3.54	+	+
chr3L	16026345	16026647	Notum	2982	5.80996	3.99	+	+
chr3L	16026962	16027395	Notum	3641	9.18539	5.16	+	+
chr3L	16046607	16047011	Diap1	36	11.6163	5.40	+	+
chr3L	16113577	16113890	Taf4	53	9.06198	4.51	+	+
chr3L	16231734	16232242	PDCD-5	24	6.36773	3.80	-	+
chr3L	16231734	16232242	MED10	69	6.36773	3.80	-	+
chr3L	16233827	16234181	l(3)72Dp	196	6.36927	4.18	-	-
chr3L	16378226	16378681	roq	38	7.36908	4.33	+	+
chr3L	16381326	16381846	CG4753	89	8.37142	4.58	-	-
chr3L	16409124	16409563	fax	972	6.71948	3.98	+	+
chr3L	16574385	16574721	mbf1	71	7.65493	4.10	-	-
chr3L	16584095	16584485	UQCR-C2	12	8.16907	4.61	-	+
chr3L	16586855	16587388	Syx8	59	10.0899	4.99	-	-
chr3L	16586855	16587388	CG32163	40	10.0899	4.99	-	-

chr3L	16647701	16648121	Abl	72	6.25604	3.75	+	-
chr3L	16711411	16711828	Lasp	55	7.76862	4.04	+	-
chr3L	16711411	16711828	CG43954	55	7.76862	4.04	+	-
chr3L	16713479	16714333	Dab	255	4.72558	3.33	+	+
chr3L	16745797	16746422	CG42852	9568	6.36773	4.33	+	-
chr3L	16865205	16865696	Lmpt	57	6.48117	3.86	-	-
chr3L	16890936	16891445	Lmpt	18	6.41874	4.07	+	+
chr3L	16955364	16955710	Nc73EF	32	5.55991	3.39	-	-
chr3L	17010123	17010554	CG3764	2216	9.59887	4.90	+	+
chr3L	17012479	17012753	CG3764	7	6.77478	4.62	+	+
chr3L	17379290	17379671	Cad74A	99	5.79011	3.84	+	-
chr3L	17470020	17470573	CG7589	73	5.2975	3.80	+	-
chr3L	17475877	17476384	CG34250	87	12.6521	5.12	+	+
chr3L	17532908	17533738	Nedd4	47	9.21106	4.23	+	+
chr3L	17557352	17557900	CycT	239	9.23551	4.35	+	+
chr3L	17576241	17576545	Eip74EF	92	5.42437	3.63	+	-
chr3L	17577453	17578046	Eip74EF	1064	7.42562	4.10	+	-
chr3L	17584754	17585027	Eip74EF	8281	5.68721	3.66	+	-
chr3L	17603789	17604238	Eip74EF	15385	11.0768	5.31	+	-
chr3L	17612072	17612569	Eip74EF	6985	14.7673	5.81	+	-
chr3L	17644276	17645038	Pep	489	10.9423	5.24	+	+
chr3L	17653091	17653937	Krn	542	5.57085	3.49	+	+
chr3L	17653091	17653937	Ndfip	542	5.57085	3.49	+	+
chr3L	17659299	17659632	CG7484	4927	5.12257	3.57	+	-
chr3L	17841109	17841518	CG5577	119	9.19661	5.00	+	+
chr3L	17846186	17846636	MED19	65	5.06013	3.23	+	+
chr3L	18087971	18088397	CG34253	858	11.8279	5.66	+	-
chr3L	18105696	18106599	CG13698	121	9.37364	4.40	+	+
chr3L	18105696	18106599	mRpS26	550	9.37364	4.40	+	+
chr3L	18179659	18179993	hid	5911	4.44639	3.49	+	-
chr3L	18460390	18460646	CG32196	552	9.43589	4.82	-	-
chr3L	18625809	18626338	MYPT-75D	17	9.93609	5.39	+	+
chr3L	18633051	18633507	MYPT-75D	7427	9.24984	5.56	+	-
chr3L	18848408	18848720	Indy	2278	9.74226	5.29	+	-
chr3L	18869243	18869572	CG6852	46	7.59014	4.31	-	-
chr3L	18890135	18890492	CG3961	169	4.55905	3.45	+	+
chr3L	18897873	18898426	CG3902	57	10.8738	5.54	+	+
chr3L	18950488	18950816	CG6836	21	6.83727	4.62	-	-
chr3L	19015926	19016995	CG18136	13015	7.65493	4.48	+	+
chr3L	19062656	19063400	CG3797	120	17.9453	6.40	+	+
chr3L	19062656	19063400	CG6812	228	17.9453	6.40	+	+
chr3L	19294360	19295196	fal	30	11.4033	5.81	+	+
chr3L	19562674	19563036	CR45916	170	8.29452	4.40	+	+
chr3L	19585770	19586320	CG9231	51	9.74417	5.12	-	+
chr3L	19585770	19586320	CG9330	198	9.74417	5.12	-	+
chr3L	19618392	19618992	l(3)76BDm	321	6.7845	3.90	+	+
chr3L	19618392	19618992	asf1	51	6.7845	3.90	+	+
chr3L	19687183	19687651	CG8765	119	11.8131	6.02	-	+
chr3L	19687183	19687651	Gbeta76C	164	11.8131	6.02	-	+
chr3L	19791443	19792288	Gyc76C	1118	4.40661	3.24	+	-
chr3L	19791443	19792288	CG42637	1118	4.40661	3.24	+	-
chr3L	19793297	19794013	Gyc76C	157	5.27983	3.35	+	+
chr3L	19793297	19794013	CG42637	157	5.27983	3.35	+	+
chr3L	19793297	19794013	SREBP	522	5.27983	3.35	+	+
chr3L	19834063	19834487	trc	6	6.16217	4.06	+	+
chr3L	19861158	19861645	CR45433	23	9.06313	4.78	+	-
chr3L	19870816	19871586	Rab8	54	7.30664	3.83	+	+
chr3L	19870816	19871586	Usp32	114	7.30664	3.83	+	+

chr3L	19929927	19930211	RhoGDI	1304	9.69489	5.26	+	-
chr3L	20316795	20317447	Spn77Ba	81	10.7628	5.16	+	+
chr3L	20316795	20317447	CR43875	13	10.7628	5.16	+	+
chr3L	20353612	20354239	CG5618	98	6.47648	4.24	+	+
chr3L	20474856	20475167	isoQC	12	4.8964	4.01	-	+
chr3L	20525692	20526148	CG4825	424	5.14201	3.70	+	+
chr3L	20675084	20675328	kni	19316	4.02245	3.63	+	-
chr3L	20771338	20771812	CG4042	1	9.97354	4.93	+	+
chr3L	20771338	20771812	Pitslre	21	9.97354	4.93	+	+
chr3L	20785184	20786279	CG11399	463	8.51588	4.38	+	+
chr3L	21027766	21028117	CG10585	63	12.1967	5.50	+	+
chr3L	21049566	21049961	siz	1502	5.06013	3.23	+	-
chr3L	21056626	21057082	siz	2068	5.22851	3.63	+	-
chr3L	21057585	21058208	siz	737	6.42437	4.36	+	+
chr3L	21172782	21173450	chb	368	6.6517	4.21	+	-
chr3L	21172782	21173450	AsnS	103	6.6517	4.21	+	-
chr3L	21175704	21176080	chb	113	5.91102	3.91	+	+
chr3L	21285716	21286171	CG12974	34	11.3444	5.60	+	-
chr3L	21285716	21286171	pzg	102	11.3444	5.60	+	-
chr3L	21304303	21304966	CG11309	80	9.97354	4.93	+	-
chr3L	21304303	21304966	CG7632	42	9.97354	4.93	+	-
chr3L	21382370	21382674	rgn	44	4.23076	3.14	+	+
chr3L	21483906	21484303	Neu2	6513	12.5821	5.52	+	-
chr3L	21841021	21841385	P5CDh1	64	10.5632	4.92	+	+
chr3L	22262646	22263106	Rich	37	7.91708	4.78	-	+
chr3L	22460928	22461377	laza	138	4.72558	3.33	+	-
chr3L	22773397	22774043	SPoCk	227	8.53386	4.66	+	-
chr3L	22837865	22838679	CG45428	1273	6.30792	3.57	+	-
chr3L	22863670	22864159	CG33169	22	5.51221	3.46	+	+
chr3L	22894791	22895085	CR44688	1175	5.94021	4.07	+	-
chr3R	4174673	4175231	CG12581	327	6.75683	4.27	+	+
chr3R	4403372	4404089	lost	612	4.84254	3.51	+	+
chr3R	4659458	4659895	CG43427	216	6.147	3.69	+	+
chr3R	4717425	4718053	CR44923	1525	7.90024	4.94	-	-
chr3R	4952602	4952861	Ubc6	62	7.55162	4.17	+	+
chr3R	4955150	4955466	CG14661	3	5.57085	3.83	+	+
chr3R	5086838	5087479	corto	102	21.0133	6.88	+	+
chr3R	5363595	5363902	Cerk	1770	4.52176	3.42	+	-
chr3R	5371613	5371942	RpII18	34	12.0005	5.42	+	+
chr3R	5517043	5517747	Itp-r83A	205	4.43971	3.07	+	+
chr3R	5547238	5547847	CG2519	376	5.12257	3.57	+	+
chr3R	5600379	5601346	kra	175	6.48702	3.76	+	+
chr3R	5623998	5624716	Spec2	109	8.26944	4.27	+	+
chr3R	5623998	5624716	CG2911	109	8.26944	4.27	+	+
chr3R	5623998	5624716	RpL13A	239	8.26944	4.27	+	+
chr3R	5659119	5659540	CR44093	146	6.83727	3.82	+	-
chr3R	5670911	5671365	Atg17	1366	6.25712	3.98	+	+
chr3R	5672883	5673296	Atg17	3348	6.83727	3.82	+	-
chr3R	5742086	5742393	Sym	169	4.8392	3.40	+	+
chr3R	5778718	5779394	jagn	27	5.74412	3.49	+	+
chr3R	6405567	6406005	Dmtn	71	6.30792	3.57	+	+
chr3R	6406605	6406931	gpp	9	8.73586	4.62	+	-
chr3R	6646682	6647138	Taf1	8	5.70738	3.57	+	+
chr3R	7086369	7087143	alphaTub84B	78	6.58887	3.52	+	+
chr3R	7186602	7187239	CG10055	310	8.40095	4.46	+	+
chr3R	7186602	7187239	lap	10	8.40095	4.46	+	+
chr3R	7474472	7475074	snRNA:7SK	255	14.8638	6.17	+	+
chr3R	7515520	7516080	alpha-Est8	269	8.64661	4.87	+	+

chr3R	7523650	7523945	alpha-Est7	49	5.22851	3.63	+	-
chr3R	7886803	7887237	CG10092	8	7.90919	4.22	+	-
chr3R	7902520	7902782	mRpS9	14	6.79217	4.29	-	-
chr3R	7913038	7913314	CG2846	154	5.14201	3.47	+	+
chr3R	7947179	7947719	dsx	15230	9.43589	4.82	+	-
chr3R	8030598	8030862	CG2747	91	4.01981	3.10	+	+
chr3R	8030598	8030862	CG10903	127	4.01981	3.10	+	+
chr3R	8352915	8353666	Atg13	3466	7.51156	4.40	+	-
chr3R	8650761	8651096	Mkk4	79	6.02268	3.74	-	-
chr3R	8650761	8651096	SLIRP2	208	6.02268	3.74	-	-
chr3R	8820442	8820866	CG9821	16	6.36773	3.80	+	+
chr3R	8820442	8820866	CR43130	103	6.36773	3.80	+	+
chr3R	8846068	8846502	pyd	74	13.3765	5.70	+	+
chr3R	9047139	9048260	Rel	44	8.12213	4.09	+	-
chr3R	9053072	9054805	Kdm2	30	7.76862	4.04	+	+
chr3R	9157879	9158391	pum	391	5.37529	3.49	+	-
chr3R	9227699	9228033	CR45196	359	4.69719	3.54	+	-
chr3R	9328834	9329155	CG8861	508	12.8558	5.98	-	-
chr3R	9334687	9335408	CG8866	463	5.09775	3.67	+	+
chr3R	9334687	9335408	CG8121	8	5.09775	3.67	+	+
chr3R	9510106	9510503	rump	30	4.81178	3.10	-	+
chr3R	9515799	9516524	mRpL47	116	7.05922	4.16	+	+
chr3R	9515799	9516524	CG8176	18	7.05922	4.16	+	+
chr3R	9515799	9516524	JHDM2	18	7.05922	4.16	+	+
chr3R	9515799	9516524	CG8176	18	7.05922	4.16	+	+
chr3R	9516886	9517222	JHDM2	35	6.66726	4.21	+	+
chr3R	9516886	9517222	CG8176	35	6.66726	4.21	+	+
chr3R	9532799	9533089	by	65	7.00941	4.27	+	+
chr3R	9590757	9591304	bocks	8	8.47092	4.24	+	+
chr3R	9645384	9646019	Fst	541	11.7435	6.40	-	-
chr3R	9700348	9700674	Unc-115a	545	5.84173	4.15	-	-
chr3R	9704456	9705628	Unc-115a	213	7.68006	4.23	+	+
chr3R	9710899	9711331	trbd	11	8.74927	4.63	+	+
chr3R	9710899	9711331	dmt	115	8.74927	4.63	+	+
chr3R	9747192	9747740	CG12948	174	5.56959	3.71	+	+
chr3R	9932247	9932687	Glut4EF	3588	9.01905	4.91	+	+
chr3R	9946497	9946949	Glut4EF	17796	9.03519	4.92	+	+
chr3R	10134073	10134465	tws	113	7.85637	4.19	+	-
chr3R	10170344	10170884	Best1	244	9.2371	5.19	+	+
chr3R	10263954	10264638	CG43143	52	12.3778	4.90	+	+
chr3R	10331227	10331605	Bruce	10	7.19385	4.11	+	+
chr3R	10331227	10331605	CG12818	217	7.19385	4.11	+	+
chr3R	10349889	10350233	jumu	90	6.13669	3.79	+	+
chr3R	10377989	10378475	Rfx	54	5.2894	3.55	+	-
chr3R	10380296	10380575	Rfx	2313	4.91748	3.44	+	-
chr3R	10614040	10615018	hth	104	6.9485	3.87	+	-
chr3R	10665012	10665352	CG6465	12145	4.4393	3.68	+	-
chr3R	10862869	10863382	CG6574	208	10.9911	5.26	-	+
chr3R	10868374	10868984	SelR	68	11.6651	5.59	+	+
chr3R	10868374	10868984	Tsp86D	3	11.6651	5.59	+	+
chr3R	10881764	10882337	Tpc1	15	6.24636	4.11	+	+
chr3R	11210096	11210536	Tctp	17	5.27836	3.44	+	+
chr3R	11211276	11211721	Tctp	1351	7.55162	4.17	+	+
chr3R	11216033	11216427	RpS25	62	7.06643	3.93	-	+
chr3R	11243399	11245093	CG17734	300	9.06313	4.78	+	+
chr3R	11243399	11245093	CG5214	2	9.06313	4.78	+	+
chr3R	11431549	11431880	RpL24-like	3	5.14201	3.27	-	+
chr3R	11431549	11431880	CG5276	152	5.14201	3.27	-	+

chr3R	11581465	11582146	wkd	490	7.44239	4.51	+	+
chr3R	11749591	11750139	CG6923	166	7.42562	4.10	+	-
chr3R	11759422	11760009	Lk6	516	6.02268	3.74	+	+
chr3R	11763671	11764717	Lk6	90	9.19735	4.57	+	+
chr3R	11778095	11778517	l(3)neo38	77	10.3493	4.97	+	+
chr3R	11786521	11787138	CG17360	17	5.42437	3.75	+	+
chr3R	11796726	11797098	Sbf	34	7.91708	4.78	-	+
chr3R	11819041	11819569	glo	113	5.79209	3.61	+	+
chr3R	11819041	11819569	COX5A	174	5.79209	3.61	+	+
chr3R	11868765	11869518	CG6959	578	4.51847	3.12	+	+
chr3R	11888273	11888587	mthl5	1213	5.09715	3.34	+	-
chr3R	11949647	11950096	CG31211	48	6.13849	3.92	+	+
chr3R	11967396	11968027	CG12213	232	5.06852	3.53	+	+
chr3R	11967396	11968027	GC1	216	5.06852	3.53	+	+
chr3R	12090468	12090986	ATP8B	31	6.51367	4.13	+	+
chr3R	12121113	12121498	CG4702	6810	14.1977	6.42	-	+
chr3R	12368955	12369555	GstD1	134	8.23591	4.51	+	+
chr3R	12405654	12406058	Cyp9f2	141	7.45333	4.24	+	+
chr3R	12425625	12426279	Pp1-87B	48	10.4937	5.18	+	+
chr3R	12465179	12465508	CG5608	20	8.53386	4.66	-	-
chr3R	12505772	12506095	Hsp70Bb	134	70.845	11.29	+	-
chr3R	12857550	12857887	CG10126	45	9.00104	5.23	+	+
chr3R	12998027	12998441	CG7518	16	7.68006	4.23	+	+
chr3R	13015561	13016402	CtBP	864	6.17887	3.82	+	-
chr3R	13401489	13402042	GILT1	30	10.4876	5.34	+	+
chr3R	13644916	13646320	sqd	895	7.19385	4.11	+	+
chr3R	13661297	13661665	B52	33	10.6904	5.12	+	+
chr3R	13683639	13684448	flfl	130	7.55162	4.17	+	+
chr3R	13710627	13710994	primo-1	30	8.32052	4.70	-	-
chr3R	13782238	13782801	Dip-B	78	4.22062	3.04	+	-
chr3R	13794639	13795257	CR45590	8557	7.19385	4.68	+	-
chr3R	14223835	14224294	Sp212	147	6.02165	3.85	+	-
chr3R	14286566	14287083	trx	9	9.37364	4.40	+	-
chr3R	14308033	14308499	su(Hw)	419	6.36773	3.92	+	+
chr3R	14316006	14316417	Set8	142	10.0274	4.82	+	+
chr3R	14326482	14327632	Afti	336	9.42824	5.29	+	+
chr3R	14326482	14327632	ATPsynE	42	9.42824	5.29	+	+
chr3R	14511367	14511992	HtrA2	130	8.19003	4.35	-	+
chr3R	14511367	14511992	mRpL11	5	8.19003	4.35	-	+
chr3R	14569164	14569705	Cys	354	4.04281	3.12	+	+
chr3R	14714884	14715160	kibra	9183	4.6863	3.42	+	-
chr3R	14740250	14740840	eff	218	8.91381	4.57	+	+
chr3R	14741254	14741659	eff	124	6.8328	4.04	+	+
chr3R	14741254	14741659	jvl	138	6.8328	4.04	+	+
chr3R	14817894	14818338	btsz	248	5.47582	3.55	+	-
chr3R	14903841	14904341	VhaPPA1-1	30	8.85288	4.82	-	+
chr3R	15095561	15095980	CG6966	186	9.28177	5.05	+	+
chr3R	15146869	15147833	GlyS	147	4.77508	3.26	+	+
chr3R	15229162	15229574	Art3	55	5.90548	3.79	-	+
chr3R	15287795	15288153	Tm1	60	8.12089	3.98	+	-
chr3R	15290465	15290986	CG45218	145	6.61493	3.71	+	+
chr3R	15290465	15290986	Tm1	289	6.61493	3.71	+	+
chr3R	15328761	15329006	ea	105	5.27892	3.98	+	+
chr3R	15342559	15343019	CG6218	390	10.1441	5.01	+	+
chr3R	15356785	15357417	Atg4b	300	5.39664	4.05	+	+
chr3R	15356785	15357417	CG5044	4	5.39664	4.05	+	+
chr3R	15371754	15372419	Ccm3	49	9.19735	4.57	+	-
chr3R	15371754	15372419	CG6136	207	9.19735	4.57	+	-

chr3R	15547354	15547712	AOX3	217	4.24545	3.14	+	-
chr3R	15793362	15793792	nsl1	71	5.14201	3.27	+	+
chr3R	15804077	15804424	alpha-Man-IIb	8	7.55162	4.17	+	+
chr3R	15972696	15973873	mor	797	8.48178	4.37	+	+
chr3R	15972696	15973873	Hel89B	413	8.48178	4.37	+	+
chr3R	16099106	16100026	Akt1	696	4.82827	3.20	+	+
chr3R	16146289	16147250	CG5903	78	4.66368	3.11	-	+
chr3R	16146289	16147250	CG5916	364	4.66368	3.11	-	+
chr3R	16235359	16235977	tara	7271	12.596	5.68	+	+
chr3R	16236412	16237427	tara	6214	11.2747	5.10	+	+
chr3R	16242564	16243070	tara	145	4.66368	3.11	+	+
chr3R	16253100	16253483	tara	10476	6.33579	4.03	+	-
chr3R	16253974	16254815	bor	9769	6.15136	3.80	+	-
chr3R	16280101	16280560	gish	564	7.59225	4.60	+	-
chr3R	16305308	16306110	Zip89B	75	6.03471	3.99	+	+
chr3R	16356404	16357184	CG10311	146	9.12391	4.67	+	+
chr3R	16453473	16454035	Pak3	14	6.51367	4.13	+	+
chr3R	16798563	16798932	CR45750	1946	8.90561	4.70	+	-
chr3R	16969558	16970348	Abd-B	1101	10.301	5.24	+	-
chr3R	16981802	16982128	Ahcy89E	12	8.40095	4.33	+	-
chr3R	16987527	16987848	CG18622	43	7.18203	3.98	+	-
chr3R	17056563	17056990	Dad	1892	6.48211	4.25	+	-
chr3R	17065292	17065602	Ns1	7231	4.58751	3.36	+	-
chr3R	17385829	17386458	lute	471	4.04545	3.52	+	+
chr3R	17394138	17395200	CREG	61	5.27836	3.44	+	-
chr3R	17682133	17682737	alt	32	10.5865	5.07	+	-
chr3R	17682133	17682737	CG7655	121	10.5865	5.07	+	-
chr3R	17700065	17701802	CR46036	695	12.8558	5.98	+	+
chr3R	17703625	17704202	CR46035	204	11.5371	4.94	+	-
chr3R	18021486	18021880	CG7785	107	7.45333	4.24	+	-
chr3R	18121674	18122067	sr	243	6.50972	4.27	+	+
chr3R	18226238	18227111	Mdh2	573	6.96631	4.12	+	+
chr3R	18244265	18245314	14-3-3epsilon	2073	17.5507	6.41	+	+
chr3R	18310239	18311069	PKD	5	9.59887	4.90	+	+
chr3R	18396738	18397006	Vha100-2	111	5.49106	3.79	+	-
chr3R	18396738	18397006	Vti1b	177	5.49106	3.79	+	-
chr3R	18399544	18399882	Vti1b	17	8.68247	4.74	+	+
chr3R	18659220	18659608	CG31224	11	6.02165	3.85	+	+
chr3R	18914723	18915148	Xrp1	74	8.82148	4.96	+	+
chr3R	18918004	18919224	Xrp1	2	6.07228	3.55	+	+
chr3R	19027081	19027791	NP15.6	44	5.70738	3.92	-	+
chr3R	19096365	19097015	CG6040	131	6.48702	3.76	+	-
chr3R	19147494	19148207	Ppcs	115	11.3189	4.98	+	+
chr3R	19211481	19212467	cry	441	13.6308	5.98	+	+
chr3R	19491019	19491349	CG15025	68	10.2115	5.93	-	-
chr3R	19620996	19621358	CG6231	34	7.87163	4.46	+	+
chr3R	19641111	19641593	subdued	130	23.9909	7.62	+	+
chr3R	19643950	19644565	Vha13	187	8.53386	4.66	+	+
chr3R	19648094	19648950	CG6195	52	5.93986	3.93	-	+
chr3R	19648094	19648950	CG31220	128	5.93986	3.93	-	+
chr3R	20008290	20008964	Hs6st	62	4.51595	3.21	+	+
chr3R	20008290	20008964	MED25	496	4.51595	3.21	+	+
chr3R	20021042	20021405	CG4433	24	5.4551	3.76	+	+
chr3R	20252376	20252949	cic	132	13.8197	6.44	+	+
chr3R	20310328	20310603	CG42668	1096	5.97337	3.95	+	+
chr3R	20556240	20557842	att-ORFA	0	10.1188	5.15	+	+
chr3R	20579807	20580223	EloB	202	6.03882	3.64	-	+
chr3R	20579807	20580223	Srp14	62	6.03882	3.64	-	+

chr3R	20779834	20780156	Syp	286	4.78896	3.37	+	-
chr3R	20821587	20822150	CG17271	39	8.40095	4.33	+	+
chr3R	20850376	20850718	RpS30	14	7.09668	4.18	-	+
chr3R	20948659	20949388	Atpalpha	15	5.4551	3.76	+	+
chr3R	21064373	21064647	Cortactin	83	5.47157	3.91	+	-
chr3R	21101885	21102235	CG5745	47	8.64661	4.87	-	+
chr3R	21124308	21124790	slmb	2928	6.22041	4.23	+	-
chr3R	21127435	21127965	slmb	199	12.464	5.79	+	+
chr3R	21127435	21127965	CG5793	59	12.464	5.79	+	+
chr3R	21268830	21269206	Rab1	2	12.0929	5.03	+	+
chr3R	21296490	21298182	Hsromega	569	12.7762	5.45	+	+
chr3R	21376318	21377264	mod(mdg4)	280	6.01681	3.85	+	+
chr3R	21408297	21408680	CheB93b	553	34.9355	12.43	-	-
chr3R	21487642	21488132	Fancm	5507	7.80626	4.56	-	-
chr3R	21660788	21661156	E2f1	73	11.3189	4.98	+	-
chr3R	22019066	22019452	CG6439	39	8.74927	4.63	+	+
chr3R	22022832	22023776	Mitofilin	4	7.87163	4.46	+	+
chr3R	22022832	22023776	mRpL35	456	7.87163	4.46	+	+
chr3R	22035643	22036002	pit	14	7.39314	4.64	-	+
chr3R	22362430	22362973	Muted	2	7.18203	3.98	+	+
chr3R	22362430	22362973	CG7071	2	7.18203	3.98	+	+
chr3R	22362430	22362973	Muted	275	7.18203	3.98	+	+
chr3R	22470806	22473304	Nrx-1	1805	6.01219	4.25	+	-
chr3R	22478486	22479703	mir-4952	1434	9.607	5.58	-	-
chr3R	22482992	22483719	mir-4952	2786	4.55905	3.45	-	-
chr3R	22528024	22528716	CG5346	86	8.08878	4.88	+	+
chr3R	22547340	22548045	AdipoR	18	8.67696	4.46	-	-
chr3R	22639551	22639874	wake	15	4.58751	3.47	-	-
chr3R	22793961	22794303	CG7031	10135	14.3876	7.16	-	-
chr3R	23117628	23117954	Rassf	181	6.55247	3.79	+	+
chr3R	23157553	23158074	unk	253	4.13788	3.08	+	+
chr3R	23226255	23227136	cnc	127	12.7113	6.09	+	+
chr3R	23293038	23293451	pnt	2053	5.69583	3.37	+	-
chr3R	23295042	23295801	pnt	37	4.37187	3.44	+	-
chr3R	23348005	23348333	DNApol-epsilon255	65	6.07228	3.88	+	-
chr3R	23358774	23359127	RpS3	69	5.45744	3.65	-	+
chr3R	23374229	23374868	CG42828	741	4.77743	3.59	+	+
chr3R	23386079	23386541	CG4393	1571	7.65493	4.10	+	-
chr3R	23653778	23654680	CG31145	15362	10.1356	5.50	+	+
chr3R	23723175	23723452	CG10365	45	4.42754	3.25	+	+
chr3R	23819122	23819900	CG33111	26	5.27836	3.44	+	+
chr3R	23819122	23819900	eIF4G2	26	5.27836	3.44	+	+
chr3R	23819122	23819900	CG33111	26	5.27836	3.44	+	+
chr3R	23889974	23890264	Pli	53	5.26631	3.17	+	+
chr3R	23890670	23891161	TfIIA-S	99	6.02165	3.85	+	+
chr3R	23993088	23993762	Rab7	160	7.14896	4.35	+	+
chr3R	24031354	24032047	KrT95D	27	13.7872	5.72	+	+
chr3R	24050629	24051064	Atg6	119	4.77508	3.26	+	-
chr3R	24050629	24051064	CG5991	119	4.77508	3.26	+	-
chr3R	24130113	24130475	CG5463	31	7.01026	4.27	-	-
chr3R	24130113	24130475	Tsc1	20	7.01026	4.27	-	-
chr3R	24151918	24152611	p38a	148	4.91748	3.44	+	+
chr3R	24151918	24152611	CG6178	123	4.91748	3.44	+	+
chr3R	24259252	24259888	jar	26	5.97337	3.95	+	-
chr3R	24271098	24271504	jar	55	21.5335	7.83	+	+
chr3R	24555243	24556091	snRNA:U6:96Ac	684	43.9388	13.15	+	-
chr3R	24555243	24556091	snRNA:U6:96Ab	684	43.9388	13.15	+	-
chr3R	24555243	24556091	snRNA:U6:96Aa	684	43.9388	13.15	+	-

chr3R	24556748	24557525	snRNA:U6:96Ac	134	40.9327	12.43	+	-
chr3R	24556748	24557525	snRNA:U6:96Ab	134	40.9327	12.43	+	-
chr3R	24556748	24557525	snRNA:U6:96Aa	134	40.9327	12.43	+	-
chr3R	24581893	24582677	REPTOR	879	4.89568	3.54	+	+
chr3R	24586373	24586912	mld	1083	18.0638	6.97	+	+
chr3R	24862753	24863150	CG7016	19	6.29592	4.14	+	-
chr3R	24894432	24894878	CG31121	112	11.438	6.02	+	-
chr3R	24894432	24894878	CG11069	24	11.438	6.02	+	-
chr3R	24944704	24945380	ssh	298	5.93686	3.59	+	+
chr3R	24944704	24945380	Nmnat	18	5.93686	3.59	+	+
chr3R	25025027	25025576	Hr96	19	10.0673	5.13	+	+
chr3R	25049726	25050732	CG11791	222	5.21599	3.31	+	-
chr3R	25219076	25219394	CG31103	44	11.3207	5.96	+	+
chr3R	25247201	25247643	RpS27	1	10.5632	4.92	+	+
chr3R	25292723	25293079	CG31098	151	8.86712	4.99	+	+
chr3R	25328449	25329108	CHKov1	632	8.01716	4.53	+	-
chr3R	25482193	25482978	XNP	64	6.96631	4.12	+	+
chr3R	25490639	25491092	Dhap-at	35	13.2434	5.98	+	+
chr3R	25680395	25680797	CG4743	39	5.12257	3.57	-	+
chr3R	25740325	25740812	LpR2	26	5.12257	3.57	+	+
chr3R	26008399	26008940	CR45039	1485	8.31168	4.41	+	-
chr3R	26099125	26099630	dysf	569	4.23593	3.35	+	-
chr3R	26106790	26107239	CG31324	109	11.6163	5.40	+	+
chr3R	26440482	26441205	tx	231	6.84115	4.05	+	-
chr3R	26535953	26536582	scrib	32	11.6163	5.40	+	+
chr3R	26799041	26799334	Tl	148	9.49686	4.71	+	+
chr3R	26862320	26863605	Lerp	12	12.7053	5.57	+	-
chr3R	26862320	26863605	IntS12	257	12.7053	5.57	+	-
chr3R	26866612	26867513	ball	325	4.11881	3.07	+	+
chr3R	26866612	26867513	His2Av	204	4.11881	3.07	+	+
chr3R	26881312	26881739	T48	222	14.8429	6.16	+	+
chr3R	26988281	26988696	CG31075	12	12.2094	6.23	+	+
chr3R	27069778	27070439	spz	88	13.3508	6.61	+	+
chr3R	27071228	27071497	spz	1369	5.69073	3.91	+	-
chr3R	27090985	27091462	Cpr97Eb	92	7.40883	4.49	+	-
chr3R	27094359	27094847	CG14259	49	4.40587	3.58	+	+
chr3R	27149765	27150326	CG6051	864	5.04196	3.14	+	+
chr3R	27150695	27151115	CG6051	179	11.246	5.23	+	+
chr3R	27157763	27158150	grass	47	4.68441	3.21	+	-
chr3R	27165291	27165631	CG34293	49	4.24601	3.25	-	-
chr3R	27228365	27228650	CG42813	11	6.90459	4.21	-	-
chr3R	27706812	27707244	CG12877	56	5.32495	3.37	-	-
chr3R	27742321	27743323	Ets98B	704	6.97363	4.39	+	-
chr3R	28327972	28328569	larp	211	7.58117	4.59	+	-
chr3R	28544547	28544899	CG12413	38	9.34867	4.92	+	+
chr3R	28600343	28600753	Hrb98DE	238	6.96631	4.12	+	+
chr3R	28844967	28845281	spg	126	6.72338	3.87	+	+
chr3R	28858907	28859450	Inx3	97	7.14896	4.35	+	+
chr3R	28897846	28898385	Doa	4243	5.03438	3.63	-	-
chr3R	29122962	29123684	CG11880	33	11.0747	5.64	+	+
chr3R	29147851	29148542	CG11882	235	10.3385	5.26	+	+
chr3R	29147851	29148542	Pglym78	46	10.3385	5.26	+	+
chr3R	29199709	29200659	CG11899	183	10.7793	4.88	+	+
chr3R	29214328	29214715	CR31044	7	6.6001	3.81	+	+
chr3R	29219971	29220410	Ef1gamma	18	7.78294	4.16	-	+
chr3R	29493187	29493615	CG2310	102	7.94962	4.36	+	+
chr3R	29503632	29504016	Trc8	40	4.67071	3.52	+	+
chr3R	29723029	29723787	Pcd	15	6.61192	4.05	+	+

chr3R	29749024	29749400	ATPsyngamma	129	6.48702	3.98	+	+
chr3R	29749024	29749400	yata	74	6.48702	3.98	+	+
chr3R	29772137	29772478	kay	587	10.6196	5.24	+	+
chr3R	29772981	29773370	kay	259	7.86886	4.32	+	+
chr3R	29798970	29799899	CG7837	197	14.9577	6.39	-	+
chr3R	29798970	29799899	Drice	500	14.9577	6.39	-	+
chr3R	29841318	29841658	Cad99C	238	6.02268	3.74	+	+
chr3R	29861670	29862010	RpS8	37	9.47294	4.99	-	+
chr3R	29868746	29869379	Zip99C	347	5.7309	3.69	+	+
chr3R	29868746	29869379	CG34133	122	5.7309	3.69	+	+
chr3R	29876925	29877255	CG31038	2	5.6505	3.44	+	+
chr3R	30021918	30023092	Mgat2	580	6.38648	3.93	+	+
chr3R	30021918	30023092	RpS28a	153	6.38648	3.93	+	+
chr3R	30021918	30023092	Axn	40	6.38648	3.93	+	+
chr3R	30045981	30046859	RpL32	39	6.83727	3.82	+	+
chr3R	30045981	30046859	CG7943	269	6.83727	3.82	+	+
chr3R	30133932	30134677	Tpi	76	7.06643	3.93	+	-
chr3R	30171095	30171361	CG31028	32	4.22282	3.46	+	-
chr3R	30423778	30424067	CG15535	19	4.06253	3.04	-	+
chr3R	30423778	30424067	mRpS18C	25	4.06253	3.04	-	+
chr3R	30424931	30425380	CG15536	55	14.1559	6.81	-	-
chr3R	30459078	30459415	CG2224	71	11.4468	5.65	-	-
chr3R	30527053	30527505	jdp	167	9.187	4.70	+	-
chr3R	30563094	30563525	tmod	54	6.13669	3.91	+	+
chr3R	30803286	30803903	wts	3194	6.72171	4.25	+	-
chr3R	31130284	31130573	CG15556	68	4.95253	3.70	+	-
chr3R	31218417	31219849	ATPsynC	553	11.2747	5.10	+	+
chr3R	31218417	31219849	mir-4949	232	11.2747	5.10	+	+
chr3R	31218417	31219849	ATPsynC	26	11.2747	5.10	+	+
chr3R	31220630	31221880	CG12054	540	7.92	4.22	+	+
chr3R	31457979	31458351	lox	1705	9.43589	4.82	+	-
chr3R	31609473	31609890	CycG	67	6.46663	3.85	+	+
chr3R	31611193	31611487	Med	225	4.36525	3.32	+	+
chr3R	31711419	31712029	ttk	2274	4.55989	3.34	+	-
chr3R	31712342	31713670	ttk	791	8.63045	4.32	+	-
chr3R	31747462	31747961	nero	298	4.72558	3.33	+	+
chr3R	31747462	31747961	CG1910	214	4.72558	3.33	+	+
chr3R	31794362	31794850	Acf	806	4.03669	3.02	-	+
chr3R	32068301	32068690	Map205	27	6.41	3.62	+	+
chr4	68593	69049	pan	558	7.72842	4.38	+	-
chrX	493265	493935	CG17896	241	5.34678	3.83	+	+
chrX	529768	530009	CG4293	72	4.18334	3.76	+	+
chrX	758869	759399	CG5273	457	5.25105	3.64	+	+
chrX	764926	765429	RpL22	103	6.38648	4.06	+	+
chrX	764926	765429	CR44966	54	6.38648	4.06	+	+
chrX	1236472	1236865	CG3638	61	5.14201	3.47	+	-
chrX	1399346	1399962	CG11448	6739	9.00375	5.06	+	+
chrX	1466642	1467467	CG14777	49	7.44239	4.51	+	+
chrX	1512636	1513100	CG14795	179	5.69073	3.91	+	+
chrX	1512636	1513100	inc	87	5.69073	3.91	+	+
chrX	1618180	1618587	Mur2B	323	4.06794	3.24	+	-
chrX	1624677	1625220	br	2381	5.75535	4.24	-	-
chrX	1952954	1953303	Hr4	11250	8.60331	5.37	+	+
chrX	2040973	2041336	usp	114	7.12018	4.80	+	+
chrX	2041652	2042418	CG4313	295	8.28003	4.83	+	+
chrX	2041652	2042418	Actn	149	8.28003	4.83	+	+
chrX	2041652	2042418	usp	149	8.28003	4.83	+	+
chrX	2041652	2042418	Actn	149	8.28003	4.83	+	+

chrX	2050300	2050772	moody	361	8.64661	4.87	+	-
chrX	2073225	2073657	CG4199	5	6.25964	3.99	-	-
chrX	2094066	2094654	csw	223	8.28003	4.83	+	+
chrX	2123099	2123988	ph-d	6	16.2839	6.24	+	+
chrX	2134783	2135189	ph-p	1547	9.19816	5.34	+	+
chrX	2136724	2137118	ph-p	57	6.84115	4.05	+	+
chrX	2141344	2141933	CG3835	215	5.34678	3.83	+	+
chrX	2222003	2222686	Vinc	43	6.87287	4.19	+	+
chrX	2244424	2244757	Or2a	1641	4.52332	3.67	-	-
chrX	2253786	2254504	CG3091	249	4.66658	3.52	+	+
chrX	2317526	2317798	CR34052	4650	5.12257	3.96	+	-
chrX	2684522	2685719	per	98	5.75988	3.82	+	+
chrX	2789896	2793253	CG32795	145	316.742	40.40	+	-
chrX	2795911	2796788	w	49	316.742	35.45	+	+
chrX	2947899	2948160	CG3603	76	4.89568	3.54	-	-
chrX	3382131	3382871	CR44999	9249	6.01219	4.25	+	-
chrX	3382131	3382871	Myc	9249	6.01219	4.25	+	-
chrX	3719512	3720260	Rala	136	14.827	6.52	+	+
chrX	3719512	3720260	Tlk	235	14.827	6.52	+	+
chrX	3742344	3743425	Tlk	2726	7.94962	4.36	+	+
chrX	3811228	3811670	ec	332	6.2318	4.24	+	-
chrX	3880658	3881228	VhaAC39-1	213	6.07723	4.15	+	+
chrX	3965070	3965497	lva	11	10.7744	5.85	-	+
chrX	4426858	4427139	bi	195	4.37187	3.44	+	+
chrX	4684391	4684958	Pp2C1	2185	5.93686	3.59	+	-
chrX	4687998	4688464	ctp	27	4.40661	3.24	+	+
chrX	4956025	4956319	CG15468	4937	8.82833	5.31	+	-
chrX	5046385	5047324	ovo	1127	8.60331	5.37	+	-
chrX	5055245	5055533	ovo	7013	5.12257	3.96	+	-
chrX	5063632	5063928	ovo	75	7.84957	5.09	+	-
chrX	5063632	5063928	CR44833	100	7.84957	5.09	+	-
chrX	5311958	5312310	dhd	42	5.68104	3.78	+	+
chrX	5410578	5410846	NAAT1	43	5.75535	4.24	+	+
chrX	5420506	5420983	spoon	1	7.91708	4.78	+	+
chrX	5430079	5430647	CG15784	6	10.9186	5.74	+	+
chrX	5690042	5690528	cv	234	6.42063	4.52	+	+
chrX	5704918	5706203	CG4096	6525	9.39687	5.65	+	+
chrX	5737721	5738279	CG32758	69	5.00604	3.61	+	+
chrX	5745562	5745843	IntS6	676	4.77743	3.59	+	+
chrX	5755438	5755956	CG15771	43	7.20158	4.53	+	+
chrX	5907985	5908361	CG12236	51	13.6829	7.02	+	+
chrX	5916039	5916847	CG3011	36	4.08868	3.15	+	-
chrX	5988294	5988993	CG5966	49	7.84957	5.09	+	-
chrX	6001198	6001880	CR44498	238	6.42063	4.52	+	-
chrX	6278942	6279261	Ubi-p5E	193	5.54547	3.82	+	+
chrX	6295125	6295457	CG15891	43	6.73958	4.12	-	-
chrX	6295125	6295457	CG3815	0	6.73958	4.12	-	-
chrX	6346788	6347199	CG3847	63	6.57672	4.30	+	+
chrX	6743833	6744313	RpL17	197	5.47582	3.91	+	+
chrX	6749268	6749765	CR44964	1427	7.36422	4.62	+	-
chrX	6751742	6751999	CR44964	3870	4.09583	3.83	+	-
chrX	6770776	6771240	COQ7	131	4.44639	3.49	-	+
chrX	6998519	6999040	Inx2	20	5.66244	4.17	+	+
chrX	7109735	7110356	fz4	408	6.01219	4.25	+	+
chrX	7204556	7204947	CG9650	8672	8.60331	5.37	+	-
chrX	7285960	7286467	CG1677	27	14.9577	6.39	+	+
chrX	7285960	7286467	CG2059	189	14.9577	6.39	+	+
chrX	7300184	7300755	brk	7354	6.4369	4.22	+	+

chrX	7324357	7324621	Dok	277	6.81043	4.44	+	+
chrX	7920376	7920775	Ykt6	132	7.72398	5.01	+	+
chrX	7933368	7933854	RpS14a	62	6.78704	4.43	+	+
chrX	7944612	7945321	l(1)G0193	424	6.79217	4.29	+	-
chrX	8061017	8061547	fs(1)h	282	5.01885	3.40	+	+
chrX	8115403	8116051	Gclc	675	6.42063	4.52	+	+
chrX	8194557	8194883	sdt	1521	6.43139	4.22	+	-
chrX	8409839	8410133	lawc	165	9.28024	4.74	+	+
chrX	8561544	8562146	Trf4-1	336	6.51367	4.13	+	+
chrX	8574203	8574719	CG12065	23	4.82827	3.20	+	+
chrX	8863895	8864382	CG12075	39	10.2115	5.93	+	+
chrX	8904032	8904350	e(r)	1802	6.31356	4.45	+	-
chrX	9089565	9089866	CG7033	253	5.36569	3.71	+	+
chrX	9282966	9283314	c11.1	70	11.2406	6.11	+	+
chrX	9319044	9319320	c12.1	7	5.12257	3.57	-	+
chrX	9554556	9554849	RpS28b	18	4.89568	3.54	+	+
chrX	9579322	9580052	LPCAT	91	4.36525	3.32	+	-
chrX	9684397	9685123	nej	819	5.63369	3.87	+	+
chrX	9778499	9778938	CG1354	119	11.3623	5.98	+	+
chrX	9884312	9884610	CG9691	33	9.24984	5.56	+	+
chrX	10086208	10086820	RhoU	148	7.08649	4.61	+	+
chrX	10486366	10486722	CG32687	101	7.44239	4.51	+	+
chrX	10546572	10546872	spri	1	4.78896	3.48	+	+
chrX	10767001	10768583	Atg8a	70	12.007	5.58	+	+
chrX	10767001	10768583	BTBD9	128	12.007	5.58	+	+
chrX	10772286	10772562	CG15211	168	5.51837	4.08	+	+
chrX	10923748	10925693	v	308	49.6358	14.76	+	-
chrX	11108422	11109083	Hsp60	408	6.51367	4.13	+	+
chrX	11148337	11148645	Ran	29	5.37943	3.99	+	+
chrX	11339611	11340222	CG34348	192	5.14201	3.47	+	+
chrX	11339611	11340222	Kap3	20	5.14201	3.47	+	+
chrX	11350527	11351350	CG1737	574	4.23593	3.35	+	+
chrX	11423517	11424137	Gs2	280	9.00104	5.23	+	+
chrX	11427055	11427728	Gs2	3268	9.97354	5.60	+	+
chrX	11454144	11454402	Evi5	136	5.12257	3.96	+	+
chrX	11505491	11505744	Drak	11763	5.75432	4.23	+	-
chrX	11580132	11580878	nod	188	5.22851	3.63	-	+
chrX	11681949	11682261	bif	3168	7.21023	4.69	+	-
chrX	11694636	11695197	PhKgamma	65	9.99071	5.42	+	+
chrX	11792372	11792836	CkIIbeta	12	5.4551	3.76	+	+
chrX	12028765	12029189	Usp7	64	4.09583	3.83	+	+
chrX	12654095	12654397	CkIalpha	184	4.33525	3.20	+	-
chrX	12659053	12659573	Tis11	232	6.50972	4.27	+	+
chrX	12659053	12659573	CR33963	279	6.50972	4.27	+	+
chrX	12735108	12736247	CR43963	4406	6.67237	4.52	+	+
chrX	12753871	12754290	CR44619	2180	13.7266	6.81	+	+
chrX	12897270	12897535	Pde9	152	4.9935	3.86	-	-
chrX	12900901	12901256	CG32649	79	7.39314	4.64	-	-
chrX	13128124	13129083	snmRNA:204	408	6.88557	4.49	+	+
chrX	13128124	13129083	CG32638	572	6.88557	4.49	+	+
chrX	13128124	13129083	CG15717	729	6.88557	4.49	+	+
chrX	13128124	13129083	MFS10	795	6.88557	4.49	+	+
chrX	13128124	13129083	CG15717	795	6.88557	4.49	+	+
chrX	13131488	13131742	MFS10	136	4.52332	3.67	+	+
chrX	13257128	13257398	mew	140	6.58482	4.31	+	-
chrX	13329598	13330159	Jafrac1	71	6.6517	4.21	+	+
chrX	13404343	13405073	GstT4	173	17.7828	8.19	+	+
chrX	13624454	13624892	NFAT	13	6.36773	3.92	+	+

chrX	13641569	13642331	NFAT	88	4.4488	3.62	+	-
chrX	13739580	13740160	CG11151	80	6.25964	3.99	+	+
chrX	13821474	13822333	Clic	681	7.29747	4.74	+	+
chrX	13845798	13846108	AMPdeam	121	6.36773	4.33	+	-
chrX	13893540	13893838	mamo	39581	5.36268	3.97	+	-
chrX	13933060	13933509	mamo	22	7.72398	5.01	+	+
chrX	13933893	13934269	mamo	691	7.72614	4.83	+	+
chrX	13994927	13995435	ben	1254	5.09386	3.93	+	-
chrX	14261127	14261461	CG1461	35	4.52332	3.67	+	-
chrX	14825902	14826409	rut	20	7.03236	4.58	+	+
chrX	14839922	14840351	Flo2	175	13.7138	6.59	+	+
chrX	15074886	15075213	Lsd-2	1137	5.69393	3.91	+	+
chrX	15075935	15076295	Lsd-2	59	6.94241	4.69	+	+
chrX	15082643	15083066	CG33178	48	7.23227	4.54	+	+
chrX	15139177	15139840	RpL37a	78	10.8738	5.54	+	+
chrX	15303229	15303713	gce	2960	4.23593	3.35	+	-
chrX	15323267	15323760	Top1	78	24.5366	9.43	+	+
chrX	15465385	15465747	CG6299	13	8.8123	5.12	+	+
chrX	15811785	15812980	sd	82	7.32583	4.31	+	-
chrX	15852865	15853267	UBL3	5	5.34678	3.83	-	-
chrX	16104669	16104997	CG42353	37	6.25964	3.99	+	+
chrX	16104669	16104997	CG42354	37	6.25964	3.99	+	+
chrX	16104669	16104997	CG42353	37	6.25964	3.99	+	+
chrX	16270707	16271173	Mfe2	68	8.26282	4.98	+	-
chrX	16275512	16276027	kat80	269	8.32052	4.70	-	+
chrX	16281780	16282192	eas	3409	12.3623	6.52	+	-
chrX	16422641	16423207	hang	15	7.65493	4.48	+	+
chrX	16559623	16560143	Pp2B-14D	3	9.97001	5.79	+	+
chrX	16637152	16637509	Rok	97	5.06023	4.12	+	+
chrX	16637782	16638117	RpS19a	21	4.27117	3.26	+	+
chrX	16792184	16792636	CG4768	155	6.15777	4.35	+	+
chrX	17147944	17148499	RpS5a	1	6.87287	4.48	+	+
chrX	17148849	17149309	CG5010	96	7.12018	4.80	+	+
chrX	17349271	17349582	CR43491	29582	5.75535	4.24	+	-
chrX	17577479	17577796	CR44124	1057	5.86504	4.16	+	-
chrX	17579695	17580139	CG12991	11	6.01219	4.25	+	+
chrX	17581602	17581946	CG12991	1871	7.00632	4.73	+	-
chrX	17698338	17698850	chas	115	4.55905	3.57	+	+
chrX	17830733	17831189	CG6398	113	9.39687	5.65	+	+
chrX	17830733	17831189	CR44691	154	9.39687	5.65	+	+
chrX	17839717	17840051	e(y)1	2266	6.22041	4.23	+	-
chrX	17849997	17850393	CG15814	220	7.12536	4.64	-	+
chrX	17910757	17911082	CG6847	605	8.82833	5.31	+	+
chrX	17911354	17911712	CG6847	18	4.40661	3.24	+	+
chrX	18320980	18321477	CG6023	155	5.54547	3.82	+	+
chrX	18366196	18366666	CrebB	71	9.97354	4.93	+	-
chrX	18368428	18368947	CR43686	1522	6.08707	4.15	+	-
chrX	18370718	18371079	CrebB	90	6.76984	4.42	+	+
chrX	18385112	18385379	betaCOP	44	6.07228	3.55	-	-
chrX	18483493	18484002	Hayan	57	9.607	5.58	+	+
chrX	18489359	18489675	CG15046	32	4.1784	3.42	+	+
chrX	18692942	18693223	Cyp18a1	10	5.12257	3.96	+	-
chrX	18783532	18783898	CG18259	247	4.3399	3.42	-	-
chrX	18857131	18857725	CG7322	65	5.34678	3.83	+	-
chrX	19153057	19153835	RhoGAP18B	15	6.07723	4.15	+	+
chrX	19353637	19353994	kek5	492	6.15777	4.20	+	+
chrX	19495315	19496082	CG14200	169	6.03882	3.64	+	+
chrX	19495315	19496082	Pfrx	169	6.03882	3.64	+	+

chrX	19495315	19496082	CR44340	223	6.03882	3.64	+	+
chrX	19496858	19497188	Pfrx	305	7.09668	4.18	+	+
chrX	19498030	19498657	Pfrx	171	4.92486	3.68	+	-
chrX	19521746	19522441	l(1)G0156	347	10.247	5.56	+	+
chrX	19521746	19522441	CoRest	59	10.247	5.56	+	+
chrX	19681480	19682020	dome	1621	6.51288	4.42	+	-
chrX	19683001	19683496	dome	357	6.40503	4.20	+	+
chrX	19697320	19697644	Cdc42	401	5.71487	4.21	+	+
chrX	19743733	19744536	COX6B	305	6.93675	4.37	+	+
chrX	19743733	19744536	CG18809	18	6.93675	4.37	+	+
chrX	19743733	19744536	COX6B	18	6.93675	4.37	+	+
chrX	19743733	19744536	CG33932	77	6.93675	4.37	+	+
chrX	19743733	19744536	Rpp20	77	6.93675	4.37	+	+
chrX	19743733	19744536	CG33932	77	6.93675	4.37	+	+
chrX	19743733	19744536	Rpp20	77	6.93675	4.37	+	+
chrX	19848275	19848546	pico	98	4.46194	3.38	+	-
chrX	19851869	19852353	pico	146	7.33035	4.45	+	+
chrX	19851869	19852353	Nup205	8	7.33035	4.45	+	+
chrX	19865996	19866328	Hers	13	5.37529	3.49	+	+
chrX	19886941	19887669	Hers	330	8.45273	4.92	+	+
chrX	20468259	20468511	GstT3	17	4.4488	3.62	+	+
chrX	21042952	21043253	Mgstl	85	5.57529	3.97	+	+
chrX	21120391	21120725	CR44374	331	8.60331	5.37	+	-
chrX	21280753	21281166	CG32521	198	5.6063	4.14	+	+
chrX	21324073	21324579	CG1486	469	4.24144	3.35	+	+
chrX	21604776	21605054	GCS2alpha	69	4.3399	3.42	-	-
chrX	22372019	22372410	CG14621	72050	5.14201	3.27	-	-
chrX	22456147	22456492	Usp2	123	6.02165	3.85	+	+

Supplementary Table 4 CBP binding sites
The regions indicated by 'Start' and 'End' represent the called peak region, the results of which were indicated as 'Q score' and 'Fold enrichment'.

Chromosome	Start	End	Gene Symbol	Distance to TSS (bp)	Q score (-log10(q value)	Fold change
chr2L	5332	6132	CG11023	1778	226.615	18.04
chr2L	66317	67638	CR45340	649	316.742	21.51
chr2L	66317	67638	dbr	76	316.742	21.51
chr2L	72203	74153	galectin	149	316.742	22.14
chr2L	72203	74153	CR44987	1301	316.742	22.14
chr2L	72203	74153	galectin	1593	316.742	22.14
chr2L	107720	109527	Sam-S	22	158.614	11.71
chr2L	120439	120788	CG4822	58	13.0388	3.47
chr2L	128206	129673	CG3164	80	316.742	25.89
chr2L	131406	132265	Gs1	126	104.685	10.96
chr2L	158876	159432	spen	88	61.3134	6.93
chr2L	160407	162277	spen	2010	316.742	18.51
chr2L	165514	165797	spen	6636	8.63164	2.66
chr2L	166144	167226	spen	7933	196.427	14.25
chr2L	179139	179471	spen	3509	35.9383	5.73
chr2L	181230	181582	spen	1253	7.11056	2.53
chr2L	182744	183158	spen	38	12.7916	3.20
chr2L	220396	220969	CR44220	1039	226.442	14.52
chr2L	221261	224590	kis	260	316.742	30.04
chr2L	221261	224590	CR44222	1296	316.742	30.04
chr2L	246393	247901	CR44218	2150	316.742	26.67
chr2L	248173	248523	CR44218	614	14.2064	3.38
chr2L	248863	250978	CR44218	413	316.742	34.11
chr2L	248863	250978	kis	1502	316.742	34.11
chr2L	267178	268154	CG17075	394	45.0368	6.52
chr2L	277020	277364	CG17078	5	7.17004	2.57
chr2L	285492	285891	CG3625	31	46.9309	6.00
chr2L	286202	286721	CR46263	119	8.18511	2.60
chr2L	286202	286721	CG3625	268	8.18511	2.60
chr2L	291690	291962	CG3625	14	20.8614	4.62
chr2L	295042	295468	Hop	92	19.7395	4.20
chr2L	297824	298602	Pi3K21B	74	21.8615	3.85
chr2L	305824	307281	Plc21C	630	316.742	20.34
chr2L	309729	310560	Plc21C	71	316.742	24.52
chr2L	377523	378334	al	253	316.742	36.01
chr2L	412589	413626	AP-2alpha	48	66.8543	8.65
chr2L	414264	415290	AP-2alpha	70	42.8481	5.75
chr2L	414264	415290	ebi	219	42.8481	5.75
chr2L	430797	432053	ex	64	194.628	13.92
chr2L	434291	436390	ex	4654	269.922	17.96
chr2L	452867	453296	crq	91	8.4872	2.63
chr2L	452867	453296	CG4164	174	8.4872	2.63
chr2L	455388	455742	CG4133	8	42.3703	7.39
chr2L	470198	473172	CG4297	96	316.742	28.96
chr2L	470198	473172	MED15	716	316.742	28.96
chr2L	478696	482407	cbt	1212	316.742	19.78
chr2L	483275	483819	cbt	3860	87.0137	9.19
chr2L	484165	484989	cbt	4683	85.5559	8.64
chr2L	485478	485950	ush	5105	22.5253	3.95
chr2L	485478	485950	CR46258	5105	22.5253	3.95
chr2L	486945	487226	ush	3741	24.0577	4.61
chr2L	486945	487226	CR46258	3741	24.0577	4.61
chr2L	487705	488275	ush	2983	21.2144	4.37
chr2L	487705	488275	CR46258	2983	21.2144	4.37
chr2L	490115	491219	ush	96	316.742	47.32
chr2L	490115	491219	CR46258	96	316.742	47.32
chr2L	491915	492169	CR46258	158	17.9796	4.02
chr2L	516342	516949	ush	6735	38.4309	6.93
chr2L	523007	524028	ush	34	251.965	15.35
chr2L	548091	548466	Ets21C	1189	12.6052	3.70
chr2L	552260	552984	Ets21C	184	316.742	23.42
chr2L	559511	559914	Tspo	63	17.3478	3.74
chr2L	594516	595436	Gsc	223	198.202	20.66
chr2L	701561	702385	CR44988	8595	286.076	22.23
chr2L	710601	711063	CR44988	141	14.1753	3.56
chr2L	714750	715777	ds	305	316.742	30.61
chr2L	734796	736086	Eaat2	6039	64.5491	8.73
chr2L	741592	742017	Eaat2	35	49.9508	6.67
chr2L	821309	822133	CG3662	478	18.0985	3.88

chr2L	825802	826820	CG3862	727	99.2901	9.46
chr2L	825802	826820	dock	620	99.2901	9.46
chr2L	839365	841125	drongo	2015	65.7074	6.95
chr2L	843910	844782	drongo	1308	30.2056	4.93
chr2L	845191	846890	drongo	2824	139.737	12.48
chr2L	847600	847992	CR44060	2951	39.8637	6.14
chr2L	850649	851221	CR44060	326	55.208	6.21
chr2L	850649	851221	drongo	44	55.208	6.21
chr2L	852732	853148	kraken	41	28.9827	4.63
chr2L	865292	866457	snoRNA:Me28S-U123(	353	101.993	8.76
chr2L	865292	866457	aru	15	101.993	8.76
chr2L	866900	868680	aru	52	316.742	19.92
chr2L	870430	871981	PNUTS	584	40.1825	5.65
chr2L	922586	923059	CG4341	19	46.0093	7.65
chr2L	939774	940147	CG4341	17132	7.58498	2.88
chr2L	1043050	1043684	IA-2	284	23.1127	4.89
chr2L	1047034	1047394	IA-2	840	28.594	5.61
chr2L	1047798	1048334	IA-2	67	35.8015	5.93
chr2L	1048936	1049230	IA-2	1056	8.8973	2.90
chr2L	1076986	1078009	S	45	39.143	5.55
chr2L	1076986	1078009	ast	181	39.143	5.55
chr2L	1082680	1083038	Atg4a	1960	80.6791	9.00
chr2L	1102203	1102854	CG4629	28	166.71	13.62
chr2L	1108106	1108409	mtRNApol	104	12.2259	2.87
chr2L	1118643	1119914	Pino	31	316.742	42.69
chr2L	1125245	1126001	Pino	3158	22.7276	4.54
chr2L	1128347	1129004	Pino	400	31.2463	4.88
chr2L	1134306	1135036	CG4577	212	30.0512	5.84
chr2L	1136799	1137305	CG4577	2185	7.49255	2.81
chr2L	1156224	1156810	capt	341	14.2744	3.86
chr2L	1157333	1158255	capt	313	62.7435	6.87
chr2L	1185630	1186518	CG5001	115	316.742	27.36
chr2L	1255740	1256448	robo3	562	16.5777	4.14
chr2L	1346017	1346315	CR45006	149	29.7702	6.16
chr2L	1361512	1362039	NLaz	43	62.4554	6.53
chr2L	1361512	1362039	NLaz	43	62.4554	6.53
chr2L	1420218	1420761	robo2	11	48.7049	6.69
chr2L	1555014	1555300	haf	29	20.1295	4.06
chr2L	1617557	1618258	RFeSP	3756	142.735	12.83
chr2L	1644351	1644744	CR45695	2581	44.1942	5.60
chr2L	1649777	1650299	chinmo	1220	232.686	19.02
chr2L	1650757	1651500	chinmo	49	159.173	13.37
chr2L	1662549	1663922	chinmo	4566	118.863	14.14
chr2L	1669444	1670041	chinmo	1990	66.7121	8.83
chr2L	1714362	1714874	frtz	1214	26.345	4.92
chr2L	1723195	1724520	Rim2	528	31.9969	4.55
chr2L	1727062	1728481	Eno	366	210.606	15.23
chr2L	1729725	1730197	CG31937	66	17.7837	3.56
chr2L	1732355	1732743	CG17652	150	29.7318	4.85
chr2L	1732355	1732743	CG17646	17	29.7318	4.85
chr2L	1736976	1737790	CG17646	29	316.742	25.14
chr2L	1748609	1748917	CG17712	3454	7.68684	2.77
chr2L	1774156	1774483	Gr22e	9767	17.4777	4.42
chr2L	1808717	1809054	Gr22a	13964	35.5108	6.39
chr2L	1836193	1836759	wry	28	78.7891	10.14
chr2L	1884507	1885207	CG7337	138	65.9089	7.93
chr2L	1902753	1903376	CG7337	8786	49.182	6.85
chr2L	1909723	1910204	CG7337	1770	139.86	12.71
chr2L	1911162	1911944	CG7337	70	52.8402	5.80
chr2L	1914354	1914702	CG7337	2745	14.9793	3.44
chr2L	1927341	1928011	CG7337	107	316.742	20.00
chr2L	1930719	1931199	CG7337	3134	16.0908	3.44
chr2L	1970396	1970995	erm	272	113.607	11.80
chr2L	1972602	1973214	Der-1	1042	163.308	14.71
chr2L	1980706	1981260	CG15356	27	13.8643	3.40
chr2L	1980706	1981260	CG15362	393	13.8643	3.40
chr2L	1987210	1988822	Got2	527	299.789	16.60
chr2L	1987210	1988822	Npc2a	4	299.789	16.60
chr2L	2009722	2010065	CG4238	35	18.8793	3.51
chr2L	2037532	2038672	Su(dx)	373	35.7647	5.04
chr2L	2130088	2131154	GlyP	370	316.742	48.91
chr2L	2161011	2164130	aop	1514	316.742	23.68
chr2L	2165649	2166340	aop	4507	87.0012	9.66
chr2L	2168768	2169475	aop	7505	39.352	4.95
chr2L	2173492	2173752	aop	5078	9.63106	2.95

chr2L	2175129	2176908	aop	3102	316.742	19.10
chr2L	2177975	2179069	aop	26	316.742	19.56
chr2L	2179869	2180255	aop	1349	24.7197	4.94
chr2L	2193378	2193895	CG34172	576	103.042	8.76
chr2L	2196290	2198237	CG34172	760	243.137	12.91
chr2L	2198528	2199013	CG34172	900	58.5422	6.80
chr2L	2199946	2203850	CG34172	5519	316.742	35.41
chr2L	2221400	2222442	papi	66	316.742	27.93
chr2L	2226620	2226916	sau	226	11.8981	2.87
chr2L	2236622	2236972	Sec24CD	28	17.8969	3.95
chr2L	2242033	2242658	CG4267	81	316.742	27.27
chr2L	2279358	2279721	CR43753	144	15.5302	4.09
chr2L	2311415	2311823	eys	91	25.2621	5.13
chr2L	2362632	2365766	Rab5	210	316.742	24.72
chr2L	2362632	2365766	Axud1	2353	316.742	24.72
chr2L	2373486	2374159	CG3609	126	133.887	13.12
chr2L	2374477	2374762	CG3609	8	27.6826	4.85
chr2L	2374477	2374762	CG15390	93	27.6826	4.85
chr2L	2375790	2376637	Atxn7	46	142.555	11.99
chr2L	2423076	2423350	CG44139	1545	11.5963	3.12
chr2L	2427678	2428747	CR44912	254	43.4598	5.67
chr2L	2427678	2428747	dpp	236	43.4598	5.67
chr2L	2433418	2434181	dpp	5168	7.93292	2.58
chr2L	2434570	2434997	dpp	6398	60.2213	7.50
chr2L	2450665	2451483	dpp	180	316.742	27.54
chr2L	2454317	2455100	dpp	66	57.2987	8.72
chr2L	2465563	2466059	CR45286	7247	28.2939	5.16
chr2L	2492630	2493518	Slh	329	52.7812	6.06
chr2L	2492630	2493518	oaf	46	52.7812	6.06
chr2L	2530407	2530670	CR44112	3123	9.17898	3.28
chr2L	2576600	2577008	insv	86	44.3111	6.64
chr2L	2584540	2585088	Drp1	599	15.6134	3.60
chr2L	2748498	2748830	Pgk	186	21.3956	4.23
chr2L	2749292	2755902	Pgk	2178	316.742	42.37
chr2L	2749292	2755902	Bacc	368	316.742	42.37
chr2L	2749292	2755902	Pgk	99	316.742	42.37
chr2L	2749292	2755902	Bacc	65	316.742	42.37
chr2L	2797342	2797808	CR43821	269	71.2184	9.83
chr2L	2798579	2799327	Syt1	927	128.962	13.94
chr2L	2799676	2800273	Syt1	98	80.8249	10.42
chr2L	2804793	2805259	daw	253	41.1871	6.64
chr2L	2807983	2808550	daw	20	27.779	5.35
chr2L	2829985	2831065	Duox	454	78.4866	10.71
chr2L	2855172	2856297	CG3104	320	56.9395	7.05
chr2L	2855172	2856297	RpS21	12	56.9395	7.05
chr2L	2857999	2858445	CG2991	1018	50.0262	6.68
chr2L	2861379	2862772	CG2991	74	313.971	21.95
chr2L	2874175	2875236	CG31694	406	151.896	13.36
chr2L	2874175	2875236	CG8814	6	151.896	13.36
chr2L	2884788	2885378	NTPase	51	54.1035	5.81
chr2L	2885755	2887313	NTPase	929	316.742	30.54
chr2L	2885755	2887313	lilli	851	316.742	30.54
chr2L	2887999	2889594	lilli	680	316.742	26.89
chr2L	2897185	2897516	lilli	7272	8.4325	2.92
chr2L	2900144	2900770	lilli	10137	32.1549	5.02
chr2L	2908761	2909120	lilli	18761	28.5896	4.72
chr2L	2924217	2925754	lilli	21855	277.124	19.82
chr2L	2945336	2945929	lilli	1099	15.796	3.96
chr2L	2955647	2958306	Rbp9	871	272.574	22.98
chr2L	2958868	2959158	Rbp9	113	24.7797	5.03
chr2L	2976851	2977355	CG9641	57	10.6341	3.18
chr2L	2976851	2977355	CG3165	202	10.6341	3.18
chr2L	2993183	2993515	CG3558	398	14.8975	3.35
chr2L	3056256	3056954	FASN1	46	316.742	17.10
chr2L	3058355	3059610	FASN1	2422	49.3544	6.17
chr2L	3088247	3088535	toc	1905	15.3953	3.76
chr2L	3094929	3095747	toc	611	87.2633	8.97
chr2L	3107874	3108614	toc	80	242.002	17.00
chr2L	3111338	3111750	toc	798	65.9727	8.49
chr2L	3122075	3122584	CG15403	6249	62.4232	7.53
chr2L	3124470	3125166	CG15403	3838	316.742	29.12
chr2L	3144630	3145027	toc	50	71.6306	7.71
chr2L	3159191	3159628	Mad	258	26.9525	4.51
chr2L	3165290	3165698	Hydr2	1870	52.5118	7.27
chr2L	3172786	3173124	CG31698	884	13.2341	3.03

chr2L	3301643	3303545	CG9663	76	293.897	19.46
chr2L	3348270	3348764	E23	6347	34.811	6.06
chr2L	3354564	3355517	E23	86	316.742	46.77
chr2L	3371128	3371608	CG34394	7	37.6469	5.21
chr2L	3425916	3426402	pgant2	591	179.547	16.53
chr2L	3426733	3427122	pgant2	237	55.4961	6.97
chr2L	3476434	3476786	CR43928	246	26.5367	4.71
chr2L	3477742	3478726	Thor	53	316.742	29.90
chr2L	3479730	3480112	Thor	1487	14.1573	3.37
chr2L	3482350	3482674	Thor	3997	14.2304	3.67
chr2L	3507051	3508233	tim	92	102.265	9.89
chr2L	3533788	3534793	Che-13	3585	316.742	35.32
chr2L	3538426	3540305	drm	155	316.742	25.83
chr2L	3545061	3545830	drm	5715	316.742	39.94
chr2L	3573275	3574014	CR43822	1479	19.1864	4.68
chr2L	3580532	3581374	sob	117	316.742	60.90
chr2L	3606202	3607078	odd	112	235.217	22.66
chr2L	3618951	3619404	Dot	62	45.5576	6.62
chr2L	3628879	3629257	for	3172	14.3347	3.49
chr2L	3631474	3633259	for	22	316.742	20.63
chr2L	3639994	3640351	mir-4972	3084	13.1443	3.71
chr2L	3645957	3646236	for	28	12.7984	3.24
chr2L	3648487	3648822	for	2501	32.6669	5.42
chr2L	3655164	3657095	for	1002	316.742	23.41
chr2L	3657384	3658462	for	1102	39.7209	6.62
chr2L	3662015	3662816	CG34340	284	244.486	14.60
chr2L	3699001	3699322	CG10031	76	26.5212	5.17
chr2L	3703279	3703772	CG2816	87	25.1003	4.69
chr2L	3712717	3713850	CG31955	484	12.173	2.83
chr2L	3712717	3713850	CG2818	230	12.173	2.83
chr2L	3717975	3718567	Shaw	54	40.3311	6.75
chr2L	3729576	3730674	CG10019	212	196.677	16.96
chr2L	3756849	3757458	CG43707	43	90.6763	11.92
chr2L	3771005	3773267	bowl	60	316.742	28.45
chr2L	3776845	3777121	bowl	1938	18.1679	4.06
chr2L	3778476	3779004	bowl	104	25.6344	4.85
chr2L	3786265	3787543	bark	55	316.742	33.93
chr2L	3789698	3789977	bark	171	11.7704	3.39
chr2L	3803069	3806746	Reph	370	316.742	22.84
chr2L	3825309	3825900	slp1	86	111.408	13.35
chr2L	3835203	3835724	slp2	1447	65.2134	8.26
chr2L	3836369	3836963	slp2	162	162.532	14.83
chr2L	3888033	3889712	capu	70	312.407	21.31
chr2L	3895595	3896379	capu	85	92.2678	10.68
chr2L	3991575	3992051	fred	156	27.3298	5.59
chr2L	3992396	3992747	fred	882	41.9596	6.99
chr2L	4030191	4030767	ed	963	39.1347	5.32
chr2L	4031111	4032049	ed	7	108.653	10.77
chr2L	4197152	4198191	CG3714	68	316.742	32.99
chr2L	4220331	4220654	ft	1343	21.8111	4.06
chr2L	4221565	4222175	ft	79	155.659	13.72
chr2L	4222448	4222702	ft	773	17.7908	4.22
chr2L	4281817	4282239	Art2	673	86.7371	10.97
chr2L	4282768	4283894	tutl	88	246.873	19.35
chr2L	4301459	4301748	tutl	17366	14.4039	3.96
chr2L	4310136	4310525	bdl	14184	14.0411	4.07
chr2L	4324216	4324645	bdl	33	12.198	3.54
chr2L	4331850	4334906	Atet	50	264.434	14.94
chr2L	4340009	4340807	Atet	1738	147.44	10.69
chr2L	4371608	4372253	Traf4	86	316.742	33.02
chr2L	4375037	4375504	Traf4	3303	119.659	10.11
chr2L	4419181	4419689	CG43055	8327	12.9246	3.40
chr2L	4440692	4441509	CG15431	39	105.348	11.18
chr2L	4456622	4456881	Gs1l	12	11.7751	3.24
chr2L	4476778	4477294	ine	129	243.695	23.05
chr2L	4591749	4592456	dpy	170	260.244	19.94
chr2L	4594695	4595498	dpy	135	316.742	34.76
chr2L	4614083	4614852	dpy	170	316.742	22.31
chr2L	4696177	4696544	CG34351	1734	11.6988	3.59
chr2L	4793804	4794204	CG15630	100	15.8376	3.29
chr2L	4810071	4811042	CR45297	1720	91.2744	10.73
chr2L	4811385	4812040	CR45297	235	31.2678	4.33
chr2L	4820373	4822000	CG15628	939	66.7764	9.52
chr2L	4846633	4847229	mxt	76	43.5404	5.99
chr2L	4852620	4853097	pog	38	32.5612	5.47

chr2L	4891735	4892373	CG3036	40	71.9273	7.61
chr2L	4928242	4928592	hoe1	30	17.7804	4.21
chr2L	4936146	4936774	hoe2	58	27.0358	5.43
chr2L	4981482	4981918	CG8892	99	17.6046	3.74
chr2L	4981482	4981918	CG34126	78	17.6046	3.74
chr2L	4982187	4982520	CG34126	807	9.40479	2.86
chr2L	4997212	4998216	Rtnl1	406	316.742	21.86
chr2L	4998975	4999517	Rtnl1	423	18.347	3.88
chr2L	5000074	5001629	Rtnl1	33	37.2168	5.31
chr2L	5002984	5003422	Rtnl1	1748	17.1546	3.75
chr2L	5004633	5005721	Rtnl1	63	127.32	11.15
chr2L	5009536	5009924	Rtnl1	80	9.64745	2.65
chr2L	5037375	5038024	SP555	158	95.3883	10.79
chr2L	5037375	5038024	CG14042	158	95.3883	10.79
chr2L	5037375	5038024	SP555	247	95.3883	10.79
chr2L	5037375	5038024	CG14042	247	95.3883	10.79
chr2L	5043403	5043969	CG31650	144	10.7923	2.69
chr2L	5044328	5044638	CG44000	61	13.3343	3.53
chr2L	5044328	5044638	CG33995	61	13.3343	3.53
chr2L	5044328	5044638	CG44001	61	13.3343	3.53
chr2L	5052567	5052904	eIF-3p40	43	26.345	4.92
chr2L	5052567	5052904	CG14040	105	26.345	4.92
chr2L	5062706	5063129	qtc	36	49.907	7.33
chr2L	5063886	5064437	qtc	173	39.5918	6.25
chr2L	5070882	5071194	RpL37A	50	17.1398	3.96
chr2L	5070882	5071194	CR45305	60	17.1398	3.96
chr2L	5079213	5079686	CR45305	8357	125.903	12.26
chr2L	5092789	5094066	pgant5	117	171.06	12.49
chr2L	5092789	5094066	CG5828	500	171.06	12.49
chr2L	5096561	5097168	CG4230	612	56.4115	6.97
chr2L	5107374	5109478	Msp300	158	316.742	28.40
chr2L	5144985	5146658	Msp300	389	316.742	22.46
chr2L	5147451	5147952	Msp300	1342	121.737	9.77
chr2L	5152392	5152751	Msp300	829	32.3351	5.50
chr2L	5237120	5237970	tkv	93	316.742	46.29
chr2L	5278216	5278669	Bsg25D	24	20.0558	4.74
chr2L	5292964	5293412	vri	4221	67.446	7.32
chr2L	5296809	5297398	CR45730	828	256.108	20.58
chr2L	5300956	5301906	vri	1742	123.55	12.81
chr2L	5302664	5304025	vri	2177	39.9971	5.08
chr2L	5305089	5306007	vri	63	57.4454	6.58
chr2L	5306303	5307121	vri	984	316.742	25.09
chr2L	5314629	5315183	CG14024	44	121.926	13.43
chr2L	5324906	5326889	CR44985	993	79.4015	8.23
chr2L	5324906	5326889	Ncoa6	1173	79.4015	8.23
chr2L	5342085	5342638	CG12512	55	22.1428	3.85
chr2L	5403438	5404100	H15	550	226.442	14.52
chr2L	5421477	5422074	CR43713	10121	67.4938	8.97
chr2L	5438216	5438481	snoRNA:Me28S-A248	648	13.7746	3.68
chr2L	5460972	5461793	mid	239	316.742	24.76
chr2L	5492112	5492375	CR45287	5356	10.6016	3.08
chr2L	5542642	5543575	Lam	41	316.742	18.70
chr2L	5546669	5547526	Oscillin	28	235.331	13.93
chr2L	5617898	5618312	DIP-eta	87	17.8289	4.23
chr2L	5687357	5688029	CR44819	2838	22.432	4.68
chr2L	5724548	5725269	CG11030	410	29.2682	4.48
chr2L	5730926	5731256	CG14006	2343	10.1277	3.30
chr2L	5740647	5741234	CG11147	14	122.315	11.28
chr2L	5742579	5743019	CG11147	1569	24.7797	5.03
chr2L	5799192	5800466	CG9171	39	316.742	24.14
chr2L	5804864	5805768	CG11034	54	316.742	33.49
chr2L	5879231	5880145	rau	62	316.742	23.12
chr2L	5906959	5907977	bchs	227	316.742	21.64
chr2L	5923620	5924025	CG9016	136	16.7612	3.73
chr2L	5925025	5925601	dsf	514	23.3434	5.36
chr2L	5943505	5945016	Gpdh	701	316.742	42.41
chr2L	5945642	5945945	Gpdh	1810	14.8583	2.94
chr2L	5973794	5974457	CR43465	3094	54.1486	6.76
chr2L	5977230	5978127	chic	2484	316.742	17.55
chr2L	5978799	5981116	chic	143	316.742	41.42
chr2L	5981445	5983687	eIF-4a	279	316.742	30.47
chr2L	5997001	5997496	lid	615	10.6257	3.17
chr2L	5997791	5998372	lid	270	37.4162	5.93
chr2L	6006465	6006879	Gal	166	30.3392	5.57
chr2L	6019790	6020130	H2.0	615	19.5844	4.87

chr2L	6045754	6046242	ade2	2	20.7853	3.89
chr2L	6047838	6048388	slmo	18	10.0967	2.70
chr2L	6047838	6048388	CG12393	49	10.0967	2.70
chr2L	6051135	6051668	CG9135	473	39.734	5.10
chr2L	6054016	6054498	CG13995	78	50.3978	7.15
chr2L	6062564	6062864	ND-51	49	10.9339	3.03
chr2L	6070998	6071311	Kr-h2	35	7.46815	2.46
chr2L	6078289	6080731	CR9162	797	316.742	36.37
chr2L	6078289	6080731	CR44773	514	316.742	36.37
chr2L	6081116	6081751	Kr-h1	1213	39.4449	5.79
chr2L	6082179	6083711	Kr-h1	831	239.658	15.85
chr2L	6088081	6089365	CG45075	2886	316.742	28.65
chr2L	6088081	6089365	Kr-h1	2886	316.742	28.65
chr2L	6098839	6099281	CG9175	34	20.9703	4.06
chr2L	6100143	6100539	stai	67	40.6851	6.54
chr2L	6109051	6109857	stai	145	78.4866	10.71
chr2L	6178545	6179444	CG34380	37	174.735	16.83
chr2L	6186401	6186830	CG34380	7481	28.6864	5.90
chr2L	6252273	6253225	Ddr	629	140.945	14.23
chr2L	6323632	6324836	PDZ-GEF	770	316.742	22.39
chr2L	6338697	6339811	Cpr	572	316.742	27.68
chr2L	6341521	6342017	Cpr	50	45.3359	5.88
chr2L	6423243	6423828	Tig	314	44.1311	6.08
chr2L	6424224	6424694	Fic	42	21.8908	4.33
chr2L	6447532	6448076	CG9527	18	63.9562	7.78
chr2L	6455014	6456567	retm	676	155.05	12.11
chr2L	6455014	6456567	frj	798	155.05	12.11
chr2L	6465458	6465880	CG16947	3	68.7154	8.05
chr2L	6471889	6472214	mmy	68	25.8828	4.83
chr2L	6473396	6473928	mmy	118	7.21123	2.38
chr2L	6479241	6481868	Sec61alpha	979	189.731	12.55
chr2L	6479241	6481868	Daxx	1441	189.731	12.55
chr2L	6479241	6481868	CR45463	1443	189.731	12.55
chr2L	6490780	6491303	CG9548	316	12.5313	3.19
chr2L	6490780	6491303	CG31638	99	12.5313	3.19
chr2L	6494272	6494849	CG9547	44	316.742	21.63
chr2L	6498464	6498887	CG31637	25	32.3271	5.23
chr2L	6525257	6525514	eya	5135	11.9218	3.27
chr2L	6535793	6536541	eya	215	159.813	13.15
chr2L	6546908	6547230	eya	55	28.2939	5.16
chr2L	6600869	6601722	CG11319	111	224.893	21.87
chr2L	6610993	6611650	CG11050	97	9.32917	2.65
chr2L	6612345	6613165	CG11050	903	112.001	10.99
chr2L	6644457	6644807	CG31635	3508	30.1806	4.93
chr2L	6647100	6649439	CG31635	657	258.137	15.97
chr2L	6647100	6649439	Tango1	1899	258.137	15.97
chr2L	6667630	6668246	Galt	98	199.406	13.26
chr2L	6672929	6673971	Spn27A	10	316.742	24.52
chr2L	6672929	6673971	CG34310	626	316.742	24.52
chr2L	6684081	6684647	Nhe3	45	16.3189	3.65
chr2L	6709036	6709342	Tsp	67	15.6368	3.71
chr2L	6722930	6723326	homer	81	12.288	2.90
chr2L	6723990	6724552	Liprin-alpha	447	32.272	4.55
chr2L	6742034	6742591	CG11221	380	18.301	4.40
chr2L	6773814	6774254	CG17378	95	13.0059	3.74
chr2L	6775059	6775336	CG17378	537	9.17898	3.28
chr2L	6784020	6784613	nrv1	2710	23.1959	4.51
chr2L	6785233	6785531	nrv1	1494	19.2268	3.68
chr2L	6786498	6788210	nrv1	929	316.742	30.94
chr2L	6791749	6792540	nrv2	2437	316.742	21.27
chr2L	6793650	6795265	nrv2	345	261.655	18.10
chr2L	6797504	6797766	nrv2	573	18.3301	3.54
chr2L	6817361	6817785	sens-2	4339	59.5991	7.82
chr2L	6821679	6822035	sens-2	74	16.6579	4.45
chr2L	6833469	6834156	sens-2	11457	88.982	12.00
chr2L	6873849	6875342	Nlg2	676	56.5397	8.56
chr2L	6918690	6919661	nop5	700	13.5923	3.31
chr2L	6920775	6922452	Hrb27C	475	51.7489	6.03
chr2L	6948523	6948995	ihog	21	29.6506	4.96
chr2L	6967408	6967722	smt3	85	18.347	4.21
chr2L	6971555	6972049	snRNP-U1-70K	198	28.1655	4.50
chr2L	6990179	6990541	CR44326	4413	34.7274	4.99
chr2L	7000562	7000891	uif	9190	26.1113	4.64
chr2L	7009774	7010480	uif	163	73.5273	9.14
chr2L	7033100	7033511	Caper	621	22.0647	4.25

chr2L	7037810	7038686	milt	642	15.6523	3.66
chr2L	7042284	7042577	milt	85	10.4108	2.63
chr2L	7048912	7050933	milt	62	296.33	16.23
chr2L	7056274	7056897	Mnn1	467	14.7124	3.67
chr2L	7084436	7084908	Pvf2	44	316.742	30.93
chr2L	7121995	7122324	Pvf3	11360	23.0575	4.88
chr2L	7187941	7189085	CG4496	703	316.742	19.99
chr2L	7199706	7200249	CG4502	4338	92.2678	10.68
chr2L	7203516	7204351	CG4502	450	35.1091	5.27
chr2L	7219385	7220178	CG13784	506	26.1527	4.53
chr2L	7245311	7245767	Ndae1	92	124.278	11.18
chr2L	7249252	7249853	Ndae1	196	182.206	16.30
chr2L	7306756	7307297	wg	203	71.7856	9.04
chr2L	7388052	7388383	CG5149	3	16.9601	3.59
chr2L	7402916	7403311	CG5171	30	48.6953	7.38
chr2L	7418118	7418943	CR43857	5167	316.742	30.29
chr2L	7419670	7421099	CR43857	3764	35.9951	6.40
chr2L	7422368	7422901	CR43857	1116	110.447	11.95
chr2L	7423406	7425672	CR43857	1244	279.499	17.58
chr2L	7436885	7437955	CG5973	80	316.742	20.28
chr2L	7451019	7451338	santa-maria	63	33.4109	5.71
chr2L	7494555	7494876	Ziz	1603	51.4229	7.62
chr2L	7495249	7496519	Ziz	730	316.742	29.27
chr2L	7529998	7531239	RapGAP1	392	316.742	30.71
chr2L	7534919	7535315	RapGAP1	4531	56.8377	6.68
chr2L	7576399	7577373	RapGAP1	409	79.5215	7.95
chr2L	7597991	7598867	CR45370	5620	65.111	8.33
chr2L	7608036	7608427	CG6739	184	41.3576	6.86
chr2L	7627278	7627574	CR44799	8713	17.3282	4.06
chr2L	7674683	7675447	Slob	129	61.9574	8.34
chr2L	7701330	7701962	Tep2	82	316.742	22.41
chr2L	7702597	7703191	Tep3	75	66.0871	6.88
chr2L	7711019	7711610	Ntl	97	17.4612	4.21
chr2L	7790355	7790683	CG7149	18	8.503	2.81
chr2L	7799418	7800217	Herp	1	40.8927	5.41
chr2L	7799418	7800217	r2d2	298	40.8927	5.41
chr2L	7808980	7810830	cdc14	919	316.742	27.64
chr2L	7811298	7811676	LanB1	13	47.0061	6.56
chr2L	7827780	7829417	mts	512	316.742	29.82
chr2L	7832762	7833176	mts	5157	38.1633	5.64
chr2L	7851397	7851718	CG14535	3698	19.7607	4.83
chr2L	7854912	7855571	CG14535	92	76.8981	9.04
chr2L	7878990	7879918	spz3	108	316.742	30.35
chr2L	7880317	7880997	spz3	1166	316.742	26.68
chr2L	7936119	7936767	Snoo	40556	21.4555	4.42
chr2L	7956698	7957083	Snoo	20129	11.0815	3.38
chr2L	7961168	7961553	Snoo	15710	93.1491	9.42
chr2L	7965792	7966143	Snoo	11034	21.9618	4.79
chr2L	7966483	7967237	Snoo	10235	47.7951	7.30
chr2L	7967564	7968088	Snoo	9290	45.136	6.24
chr2L	7968644	7968954	Snoo	8221	16.6402	4.15
chr2L	7973352	7973936	Snoo	3343	82.8517	9.56
chr2L	7976661	7977133	Snoo	171	197.511	15.58
chr2L	7984037	7984524	Snoo	83	316.742	24.56
chr2L	7984966	7985490	CG7231	50	230.412	17.47
chr2L	7991427	7991969	pes	143	91.1054	10.36
chr2L	7993127	7994718	pes	14	130.926	12.52
chr2L	7998768	7999173	Sirup	26	44.9382	5.35
chr2L	8000467	8000965	Sirup	1711	16.5566	3.69
chr2L	8006091	8007865	Spn28Dc	133	316.742	32.67
chr2L	8009203	8009890	CG12560	34	120.784	9.58
chr2L	8010476	8010741	CG34010	55	17.7496	3.92
chr2L	8012401	8013058	Tg	74	303.065	18.10
chr2L	8030744	8032242	Cka	807	316.742	29.02
chr2L	8035152	8035518	Cka	4369	12.9987	3.32
chr2L	8083308	8085012	Bsg	342	316.742	20.86
chr2L	8085480	8086009	Bsg	2267	58.5422	6.80
chr2L	8088164	8088741	Bsg	252	50.4674	7.25
chr2L	8097159	8098105	Bsg	8471	29.697	5.68
chr2L	8100020	8100431	Bsg	11145	34.9379	5.92
chr2L	8103932	8105019	Bsg	15110	316.742	24.15
chr2L	8148492	8149135	CG43394	93	120.798	12.95
chr2L	8149730	8150231	CG43394	968	21.7866	4.25
chr2L	8162510	8163901	Piezo	16	316.742	24.55
chr2L	8171663	8172010	Piezo	8753	18.0696	4.10

chr2L	8204741	8205713	SLC5A11	314	316.742	20.58
chr2L	8204741	8205713	CR44120	202	316.742	20.58
chr2L	8212905	8213191	CG8360	45	9.31732	2.92
chr2L	8233659	8233996	Pvr	3164	11.436	3.26
chr2L	8239495	8239968	Pvr	42	26.4389	4.33
chr2L	8257284	8258181	CG8086	13	31.4312	4.96
chr2L	8265479	8266240	Btk29A	293	40.9377	6.34
chr2L	8277022	8278497	Btk29A	323	316.742	31.52
chr2L	8300514	8301097	Btk29A	52	79.5215	7.95
chr2L	8324449	8325679	CG7781	672	36.6054	5.39
chr2L	8328685	8329219	CG14275	66	316.742	19.90
chr2L	8346067	8346787	CG7778	122	316.742	24.20
chr2L	8364388	8364863	RpS13	8	17.7837	3.56
chr2L	8364388	8364863	CSN8	237	17.7837	3.56
chr2L	8365933	8366605	Pp2A-29B	0	13.4933	3.42
chr2L	8370336	8370903	CG17292	24	59.8898	7.00
chr2L	8370336	8370903	CR45341	117	59.8898	7.00
chr2L	8384371	8384721	CG13384	57	14.7878	3.45
chr2L	8403536	8404055	emb	206	9.20515	2.60
chr2L	8403536	8404055	CR44991	146	9.20515	2.60
chr2L	8411472	8411864	CG13397	64	79.7819	9.54
chr2L	8415634	8417103	Akap200	784	94.1541	8.47
chr2L	8419649	8420653	Akap200	505	298.054	18.53
chr2L	8424686	8425194	Akap200	1766	60.9294	8.15
chr2L	8463796	8464147	fu12	4	38.2014	4.85
chr2L	8478070	8479012	d	35	223.555	18.51
chr2L	8521934	8522288	Acer	179	39.4752	6.39
chr2L	8528992	8529317	CG17834	50	11.2178	3.01
chr2L	8534287	8536576	CG17834	636	316.742	34.51
chr2L	8542063	8544413	Sema-1a	830	190.08	13.26
chr2L	8619435	8619836	CR43855	25098	26.5164	5.32
chr2L	8690962	8692063	Hnf4	511	316.742	28.52
chr2L	8692458	8692923	Hnf4	305	60.2566	7.19
chr2L	8699589	8700155	Hnf4	74	13.7402	3.08
chr2L	8707582	8707971	Hnf4	1279	50.9627	7.35
chr2L	8708429	8709755	Hnf4	96	316.742	26.69
chr2L	8717270	8718585	raw	3534	316.742	41.50
chr2L	8735513	8736154	raw	38	316.742	22.62
chr2L	8739826	8740736	raw	394	316.742	21.64
chr2L	8824977	8825815	SoxN	233	250.337	19.77
chr2L	8898064	8899098	CG34398	124	316.742	24.88
chr2L	8937038	8937951	Tsp29Fb	74	154.628	12.52
chr2L	8949718	8950357	C1GalTA	1206	316.742	21.71
chr2L	8950931	8951888	C1GalTA	28	173.914	12.94
chr2L	8955701	8956132	CG31886	69	39.065	6.23
chr2L	8957632	8958583	CG31886	13	140.821	12.23
chr2L	8984623	8985168	CG13101	62	166.286	13.67
chr2L	8988954	8989614	rost	5	185.019	18.32
chr2L	9125600	9125978	CG32982	56	31.4312	4.96
chr2L	9164050	9164322	CG13108	16	15.3953	3.76
chr2L	9166830	9167332	tai	280	7.87529	2.46
chr2L	9175105	9177146	tai	126	316.742	41.63
chr2L	9178352	9179698	tai	2340	163.435	12.22
chr2L	9182480	9183072	tai	6431	186.913	18.03
chr2L	9203302	9204431	tai	27335	270.012	19.68
chr2L	9204986	9205960	tai	28971	316.742	23.62
chr2L	9224159	9224963	aust	26622	100.326	10.45
chr2L	9225225	9225708	aust	25495	50.359	8.15
chr2L	9228292	9228941	aust	22397	189.435	13.45
chr2L	9229741	9230265	aust	21090	138.684	11.28
chr2L	9231873	9232306	aust	18977	25.7773	5.09
chr2L	9237612	9238243	aust	13248	36.8152	6.21
chr2L	9256429	9257016	Ggamma30A	98	55.8418	7.18
chr2L	9272055	9272622	Ggamma30A	128	100.429	10.67
chr2L	9333376	9333893	Eaat1	57	35.7141	5.03
chr2L	9386347	9386614	Aldh	932	12.4526	2.96
chr2L	9387438	9388474	Aldh	101	90.3296	9.01
chr2L	9415080	9415454	Shawl	81	28.2308	5.89
chr2L	9416097	9416683	Mco1	176	129.581	13.92
chr2L	9437230	9437747	numb	29	91.545	11.15
chr2L	9446524	9447017	numb	3006	56.9927	7.54
chr2L	9448046	9450206	numb	53	316.742	33.39
chr2L	9493808	9494941	Gdi	70	76.355	7.94
chr2L	9495418	9495686	CG33298	61	10.054	2.47
chr2L	9496274	9496738	CG33298	1017	100.783	8.89

chr2L	9497148	9497733	CG33298	1963	196.498	12.30
chr2L	9521124	9521930	Oatp30B	476	143.16	11.70
chr2L	9522675	9523322	Oatp30B	1792	298.375	19.24
chr2L	9560413	9560784	jp	176	14.7145	4.10
chr2L	9566177	9567026	jp	14	51.5768	7.02
chr2L	9567411	9569212	CR44149	1037	259.362	16.02
chr2L	9567411	9569212	CG3838	1141	259.362	16.02
chr2L	9569633	9570936	CG3838	168	102.628	10.94
chr2L	9576109	9576881	Mtpalpha	419	61.0323	6.76
chr2L	9613205	9614148	Trx-2	4	316.742	25.90
chr2L	9616638	9617190	GlcAT-S	64	155.672	14.83
chr2L	9698152	9699519	Pka-C1	23	77.9396	9.02
chr2L	9702156	9702421	pelo	1494	17.9748	3.86
chr2L	9705252	9706497	CG31710	72	218.303	17.39
chr2L	9728714	9729267	CG13116	851	12.8646	3.33
chr2L	9729656	9730102	CG13116	36	23.0887	4.17
chr2L	9734803	9735123	CG13117	1716	20.6898	4.06
chr2L	9736470	9736797	CG13117	4	23.665	4.37
chr2L	9743717	9744254	Nckx30C	2509	47.4605	6.71
chr2L	9744632	9745145	Nckx30C	1671	119.078	12.89
chr2L	9746003	9747050	Nckx30C	306	47.0783	6.81
chr2L	9749920	9750319	Cyp4e3	58	39.894	6.31
chr2L	9757908	9759181	zf30C	306	63.1089	6.04
chr2L	9779129	9779858	IP3K1	2970	22.763	4.61
chr2L	9781991	9783032	IP3K1	115	316.742	34.53
chr2L	9783293	9783598	IP3K1	1146	8.89369	2.83
chr2L	9790447	9790815	CG4036	214	25.1315	4.22
chr2L	9863536	9863904	CR43766	15413	38.9732	6.85
chr2L	9886080	9886501	nAChRalpha6	26	12.7847	3.41
chr2L	9906850	9908081	CG13124	980	42.169	6.10
chr2L	9908354	9908976	CG13124	68	316.742	25.16
chr2L	9915003	9915599	yip2	36	83.6948	7.63
chr2L	9938679	9938953	Apf	23	18.3845	3.66
chr2L	9938679	9938953	CG4619	49	18.3845	3.66
chr2L	9947138	9947788	CG5853	65	57.9021	7.61
chr2L	9948061	9948458	CG5853	659	35.3484	6.00
chr2L	9967009	9967459	Dref	40	39.5223	4.68
chr2L	9967009	9967459	RpL13	43	39.5223	4.68
chr2L	10009349	10010693	Trp1	301	316.742	20.37
chr2L	10032519	10032899	obst-B	15	31.3617	5.61
chr2L	10143280	10143755	CG4839	1324	18.5762	4.39
chr2L	10180213	10180588	CG44153	149	19.4026	4.37
chr2L	10208911	10209287	CR44845	88	72.9821	8.75
chr2L	10220700	10221041	Npc1a	69	48.2371	6.40
chr2L	10246807	10247060	chico	200	11.7412	3.11
chr2L	10321457	10321959	CG5381	22	57.6177	7.31
chr2L	10334388	10335251	GATAd	986	49.0881	6.39
chr2L	10376301	10376671	dpr19	10	17.8936	4.11
chr2L	10395917	10396306	Mdh1	40	12.4855	3.26
chr2L	10457325	10457978	LManII	8	316.742	24.91
chr2L	10474174	10474573	RluA-1	35	33.6222	6.26
chr2L	10506537	10507037	Myo31DF	27	33.7275	4.13
chr2L	10516380	10517444	Fatp	122	20.9703	4.06
chr2L	10516380	10517444	Lrr47	788	20.9703	4.06
chr2L	10661967	10662297	CG31871	53	10.1241	2.97
chr2L	10686832	10687433	CG7296	30	146.619	12.67
chr2L	10693978	10694795	CG17108	544	15.3558	3.70
chr2L	10755939	10756626	CG17124	34	168.066	11.87
chr2L	10756942	10757570	CG17124	895	152.698	11.90
chr2L	10760062	10760451	CG6495	850	11.6566	3.22
chr2L	10800902	10801385	CG31869	53	43.9241	6.73
chr2L	10842244	10842561	CG6700	207	11.0603	3.09
chr2L	10850045	10850851	CG43129	570	12.0506	3.06
chr2L	10850045	10850851	porin	138	12.0506	3.06
chr2L	10856381	10856646	Dnz1	169	5.97227	2.22
chr2L	10967158	10967979	CG33129	732	237.9	16.34
chr2L	10976937	10977353	SCAR	214	9.98162	2.87
chr2L	11044445	11045674	CG18666	10534	254.257	18.88
chr2L	11052808	11053210	Samuel	4474	14.1546	3.96
chr2L	11057092	11058724	Samuel	388	291.343	22.96
chr2L	11061203	11061454	Samuel	3765	8.525	2.74
chr2L	11066271	11067333	Samuel	50	288.39	20.57
chr2L	11086491	11086956	CG16854	44	65.1514	7.73
chr2L	11106675	11107044	l(2)gd1	6	16.1019	3.33
chr2L	11112914	11113603	CG6201	9	18.3114	3.92

chr2L	11130650	11131139	CG4751	260	24.119	4.82
chr2L	11136206	11136465	hgo	50	19.3066	4.71
chr2L	11155346	11156100	CG6287	69	316.742	29.98
chr2L	11167243	11168429	Ca-beta	250	31.0719	5.48
chr2L	11168708	11169213	Ca-beta	1002	68.2663	8.32
chr2L	11182600	11182951	CG4788	3741	21.487	5.00
chr2L	11203403	11204219	Ca-beta	384	26.4355	4.81
chr2L	11210397	11211287	ab	469	13.0311	3.32
chr2L	11220014	11220347	ab	8364	13.3929	3.97
chr2L	11221096	11221705	ab	9608	176.725	12.17
chr2L	11271538	11272343	cana	16	316.742	27.79
chr2L	11278008	11278426	CG33695	64	43.1781	5.57
chr2L	11278008	11278426	CG33695	64	43.1781	5.57
chr2L	11329508	11329945	CR44584	2977	8.4006	2.75
chr2L	11358362	11359128	salr	166	316.742	23.71
chr2L	11445421	11446501	salm	244	316.742	22.50
chr2L	11447226	11448175	salm	2273	316.742	33.15
chr2L	11501542	11501920	spz4	36	35.7647	6.85
chr2L	11516279	11517367	CG6509	289	140.498	11.99
chr2L	11527975	11528458	CG31705	14	246.277	16.28
chr2L	11528757	11529015	CG31705	696	32.297	5.43
chr2L	11581638	11582197	kek2	58	65.56	9.04
chr2L	11804377	11805295	crol	4091	259.14	17.31
chr2L	11805990	11809548	crol	1991	316.742	33.38
chr2L	11842485	11843234	CG14933	100	28.07	5.34
chr2L	11842485	11843234	CG42486	100	28.07	5.34
chr2L	11874410	11875234	Pde1c	619	62.2723	7.93
chr2L	11878871	11879383	Pde1c	1878	88.6157	8.68
chr2L	11903448	11903821	Pde1c	6860	51.2876	7.70
chr2L	11948558	11949316	CG16964	1137	89.052	10.33
chr2L	11970271	11971572	CR43314	167	316.742	31.90
chr2L	12002994	12007865	Rh5	647	197.847	14.85
chr2L	12010013	12012722	CR44588	2866	114.498	8.73
chr2L	12025279	12025754	CR44587	307	39.491	5.79
chr2L	12044589	12046618	CG6785	957	217.998	13.81
chr2L	12044589	12046618	CG6770	39	217.998	13.81
chr2L	12044589	12046618	CR44589	284	217.998	13.81
chr2L	12055710	12056387	JhI-21	1	316.742	32.97
chr2L	12055710	12056387	RpL7-like	339	316.742	32.97
chr2L	12055710	12056387	CG34164	361	316.742	32.97
chr2L	12055710	12056387	RpL7-like	361	316.742	32.97
chr2L	12085846	12086097	prd	156	19.4412	4.52
chr2L	12108648	12109200	Rab6	134	86.1631	9.93
chr2L	12133937	12134960	rho-6	717	112.688	9.38
chr2L	12142489	12142923	CG31760	39	48.5443	7.45
chr2L	12177495	12178008	atilla	104	36.385	6.04
chr2L	12207643	12208099	CG31759	86	37.8802	5.53
chr2L	12286778	12287383	aret	229	65.0141	8.41
chr2L	12288516	12288996	aret	1500	180.45	16.00
chr2L	12294607	12295123	aret	7687	18.9856	4.49
chr2L	12298012	12298311	aret	10913	14.2996	4.00
chr2L	12341720	12342031	bru-2	2354	19.7607	4.83
chr2L	12364354	12364622	bru-2	1861	18.5777	4.40
chr2L	12365363	12366621	bru-2	147	316.742	34.37
chr2L	12367324	12367685	bru-2	1116	22.3043	4.00
chr2L	12369728	12370108	bru-2	17	9.07242	3.08
chr2L	12404387	12404774	vir-1	72	42.1634	5.54
chr2L	12418167	12418595	vir-1	3505	13.0722	3.69
chr2L	12421622	12422215	vir-1	78	316.742	24.13
chr2L	12433652	12434050	SC35	103	12.7152	3.07
chr2L	12434389	12435083	Elf	338	40.7545	5.80
chr2L	12434389	12435083	SC35	124	40.7545	5.80
chr2L	12434389	12435083	Elf	102	40.7545	5.80
chr2L	12436320	12436696	Elf	230	27.0549	4.53
chr2L	12459781	12461863	mir-967	1243	316.742	45.90
chr2L	12459781	12461863	bun	87	316.742	45.90
chr2L	12482780	12484735	bun	407	316.742	33.68
chr2L	12492001	12492622	bun	405	146.912	14.63
chr2L	12504121	12505628	bun	114	260.795	19.21
chr2L	12507451	12508320	CR43051	157	316.742	30.42
chr2L	12507451	12508320	snoRNA:CG43051-a	326	316.742	30.42
chr2L	12520887	12522082	snoRNA:CG43051-a	13135	316.742	31.58
chr2L	12527583	12528618	bun	13139	29.6459	5.58
chr2L	12528945	12530788	bun	11031	316.742	32.39
chr2L	12535197	12536012	bun	5260	52.925	8.48

chr2L	12540086	12540597	bun	377	14.4479	3.43
chr2L	12542948	12544320	CR42746	148	316.742	56.16
chr2L	12545505	12546812	bun	750	316.742	34.55
chr2L	12586731	12587610	nub	456	316.742	26.13
chr2L	12617769	12618884	nub	114	316.742	32.29
chr2L	12655562	12656282	pdm2	1247	316.742	40.62
chr2L	12678203	12678650	pdm2	167	41.5816	7.11
chr2L	12700513	12700771	CG17036	34	19.4412	4.52
chr2L	12704421	12704870	CG5776	121	11.2158	2.97
chr2L	12704421	12704870	spict	152	11.2158	2.97
chr2L	12718579	12719382	MRP	73	316.742	18.86
chr2L	12719988	12720462	MRP	1154	26.8074	4.76
chr2L	12721235	12721538	Pih1D1	520	11.4543	3.31
chr2L	12822483	12823070	kek1	18	79.0479	9.41
chr2L	12854509	12854914	CR44971	13175	71.5795	9.66
chr2L	12973997	12975492	Vha68-2	76	294.494	20.03
chr2L	13003693	13004128	Vha68-1	251	21.0477	4.12
chr2L	13021092	13021380	A16	161	7.99966	2.56
chr2L	13032593	13032947	A16	11442	27.7607	5.51
chr2L	13035713	13036330	A16	14728	108.123	11.66
chr2L	13059904	13060766	CG9932	187	316.742	32.10
chr2L	13069100	13069513	CG9932	9221	16.9722	3.98
chr2L	13089649	13090034	CR44197	3	20.8104	4.68
chr2L	13090829	13091122	CR44197	1175	19.2011	4.46
chr2L	13097999	13098889	CR45447	1353	316.742	19.65
chr2L	13099420	13099847	CR45447	2626	26.8822	4.40
chr2L	13101509	13101819	CR45461	3038	9.9796	2.74
chr2L	13113646	13113927	CR44198	5535	14.8931	3.88
chr2L	13139409	13140254	CG9928	2881	15.1828	3.66
chr2L	13156051	13156915	Pk34A	2533	34.841	5.41
chr2L	13165422	13166307	Sirt1	298	316.742	19.80
chr2L	13165422	13166307	DnaJ-H	211	316.742	19.80
chr2L	13191182	13191738	CG16974	570	17.2664	3.73
chr2L	13203596	13204295	Pect	166	165.441	11.42
chr2L	13205075	13205558	Pect	1488	23.7739	4.06
chr2L	13206165	13206628	Pect	2511	16.1395	3.61
chr2L	13207153	13207467	Pect	3554	7.87137	2.70
chr2L	13215710	13216117	snRNA:U2:34ABb	60	43.9502	6.05
chr2L	13215710	13216117	snRNA:U2:34ABc	60	43.9502	6.05
chr2L	13240008	13240284	CG5945	38	9.98625	3.16
chr2L	13290245	13291313	CG31729	196	316.742	30.11
chr2L	13295914	13297128	CG31729	552	121.76	10.39
chr2L	13330922	13331517	CG43778	84	122.937	12.70
chr2L	13331798	13332404	CG43778	928	160.027	13.83
chr2L	13354496	13354817	CG9395	390	23.5235	4.98
chr2L	13362993	13363305	CG9377	1638	32.4941	4.91
chr2L	13373517	13373791	CG9305	227	13.3268	3.26
chr2L	13400493	13400866	CR44600	787	13.9887	3.73
chr2L	13415012	13415575	CG42784	127	90.2117	9.16
chr2L	13415012	13415575	CG43850	127	90.2117	9.16
chr2L	13418949	13419395	CG43850	60	44.4712	7.32
chr2L	13460672	13461928	CG42784	395	119.952	14.70
chr2L	13485855	13486323	CG42784	10019	66.3745	8.18
chr2L	13511273	13511764	CG42784	15512	42.6146	6.49
chr2L	13512615	13514898	CG33640	14356	316.742	47.65
chr2L	13547590	13548154	B4	65	25.0419	5.01
chr2L	13548449	13549509	B4	78	94.0342	8.07
chr2L	13550003	13551096	kuz	700	316.742	30.57
chr2L	13571758	13572203	CR44973	10524	39.3302	7.25
chr2L	13641044	13641310	ics	126	19.0085	4.29
chr2L	13664752	13666476	CG18507	637	316.742	29.96
chr2L	13717175	13717582	CG31814	80	30.6062	6.26
chr2L	13779853	13780890	CG9008	66	58.1228	7.16
chr2L	13779853	13780890	p38b	589	58.1228	7.16
chr2L	13793539	13793853	Sec71	207	12.6962	3.00
chr2L	13802123	13802658	CAH1	1242	61.9953	7.03
chr2L	13803184	13804060	CAH1	102	316.742	28.43
chr2L	13820849	13821514	b	42	43.899	7.10
chr2L	13835441	13835748	CenG1A	36	24.2942	4.23
chr2L	13877860	13878403	CenG1A	809	139.506	11.05
chr2L	13878732	13879248	CenG1A	43	142.029	11.55
chr2L	13904067	13904698	Smg5	54	32.2279	4.93
chr2L	13905150	13905899	Ance	6	248.549	16.68
chr2L	13934663	13934987	CG16885	124	29.2681	5.94
chr2L	13976995	13977548	Cyp28a5	46	210.164	19.67

chr2L	14021502	14022761	CR45352	202	109.537	9.50
chr2L	14023603	14024607	CR31840	213	306.772	19.42
chr2L	14038676	14039195	CR45284	655	41.2305	5.75
chr2L	14039673	14040570	nAChRalpha5	105	40.905	6.96
chr2L	14049728	14050148	NimC4	2326	46.585	8.01
chr2L	14054200	14054734	NimC4	6859	48.2567	7.59
chr2L	14094644	14094957	CG16879	9639	32.9577	4.83
chr2L	14110517	14111259	CG31769	356	25.2963	4.66
chr2L	14115772	14116547	CG31769	5603	39.5286	6.67
chr2L	14133307	14134611	CG17341	325	131.876	15.62
chr2L	14150397	14150841	CG43332	12671	10.9793	3.53
chr2L	14204149	14204576	Dyrk2	6662	9.68554	3.30
chr2L	14225869	14226201	Dyrk2	8081	38.1318	5.28
chr2L	14227633	14227960	Dyrk2	6277	21.3932	4.53
chr2L	14228275	14228563	Dyrk2	5675	17.7712	3.52
chr2L	14229950	14230561	Dyrk2	3868	126.946	12.10
chr2L	14233890	14234893	Dyrk2	20	103.13	8.70
chr2L	14233890	14234893	CR44727	607	103.13	8.70
chr2L	14244235	14244628	CR44216	3279	30.3262	5.14
chr2L	14287938	14288230	CR43639	3104	26.9717	4.79
chr2L	14334009	14334352	CG33090	14	19.5723	3.77
chr2L	14355022	14355832	l(2)34Fd	430	119.669	10.20
chr2L	14355022	14355832	Rab14	371	119.669	10.20
chr2L	14385044	14385684	ppk	4210	219.955	13.62
chr2L	14409303	14410698	elB	182	316.742	45.00
chr2L	14409303	14410698	CR44733	23	316.742	45.00
chr2L	14458533	14458860	CR44730	1529	9.05003	2.90
chr2L	14489925	14491142	noc	116	316.742	41.67
chr2L	14529648	14529908	CR44852	3747	9.67825	3.19
chr2L	14533249	14534721	CR44852	956	22.432	4.68
chr2L	14614724	14615734	Adhr	2	96.6725	8.87
chr2L	14614724	14615734	Adh	2	96.6725	8.87
chr2L	14632008	14632355	Adhr	14622	19.0028	4.42
chr2L	14688830	14689815	osp	30	131.629	11.39
chr2L	14706240	14706596	CG15282	6064	40.3339	4.99
chr2L	14797508	14797977	CG18420	1088	76.9565	8.95
chr2L	14851647	14851959	CG15279	49	14.1584	3.51
chr2L	14972107	14972641	CG42313	90	72.6947	9.20
chr2L	14995383	14996135	mol	1904	238.689	17.70
chr2L	14997300	14997954	mol	25	316.742	24.92
chr2L	15011880	15012315	CR43856	579	17.3238	3.74
chr2L	15013487	15013912	CR43856	1008	33.0161	6.04
chr2L	15016243	15016501	GABA-B-R1	9	11.6988	3.59
chr2L	15035269	15035829	yellow-c	12	57.1183	8.03
chr2L	15039415	15039891	Su(H)	238	28.2173	4.83
chr2L	15056622	15057802	ck	732	316.742	35.69
chr2L	15067770	15068266	vig	15	26.2698	4.40
chr2L	15074424	15074908	CG15270	83	53.4937	6.66
chr2L	15109005	15109557	CG15269	72	110.244	13.26
chr2L	15113290	15113628	stc	62	13.686	3.41
chr2L	15200304	15200821	CG4168	115	30.1853	6.16
chr2L	15264293	15265017	yuri	26	316.742	40.30
chr2L	15332515	15332948	esg	1136	129.57	11.38
chr2L	15333277	15334172	esg	259	316.742	25.10
chr2L	15339423	15340058	esg	5847	164.988	15.24
chr2L	15425635	15426443	wor	431	316.742	24.13
chr2L	15491563	15492542	CR45293	6214	79.9154	9.16
chr2L	15498837	15499906	lace	480	316.742	20.49
chr2L	15552449	15552901	kek3	62	43.5716	7.23
chr2L	15745456	15746957	CR44974	521	127.747	12.45
chr2L	15745456	15746957	CycE	875	127.747	12.45
chr2L	15747298	15748431	CR44866	359	284.14	17.04
chr2L	15747298	15748431	CycE	509	284.14	17.04
chr2L	15761873	15763091	Gli	24	316.742	21.98
chr2L	15911612	15912987	CR44867	259	316.742	31.60
chr2L	15911612	15912987	CR44868	608	316.742	31.60
chr2L	15913289	15913888	CR44868	1077	61.4595	7.16
chr2L	15962332	15962670	beat-Ib	69	31.2105	5.83
chr2L	16000156	16000592	beat-Ic	90	66.7095	10.03
chr2L	16049613	16050192	beat-Ia	62	73.6935	9.28
chr2L	16168954	16169275	Ca-alpha1D	745	24.8247	5.11
chr2L	16198191	16198600	CR44735	2119	44.7029	7.81
chr2L	16231147	16231735	CG42817	46	96.2634	10.50
chr2L	16234911	16235253	CG42817	3612	19.1368	4.59
chr2L	16251440	16252128	PRL-1	57	316.742	32.62

chr2L	16256958	16257669	PRL-1	43	17.3792	3.85
chr2L	16267902	16270110	CG4935	7528	316.742	39.81
chr2L	16271206	16271915	CG4935	9439	316.742	22.80
chr2L	16273503	16274513	CG4935	11555	40.7026	5.86
chr2L	16276787	16277245	crp	10773	49.343	7.13
chr2L	16279588	16280284	crp	7656	91.1664	11.47
chr2L	16284200	16285269	crp	2941	316.742	40.87
chr2L	16285955	16288883	crp	929	316.742	26.21
chr2L	16285955	16288883	pkaap	1488	316.742	26.21
chr2L	16299284	16299909	heix	224	249.174	15.48
chr2L	16315004	16315362	fzy	4204	30.4622	4.92
chr2L	16318263	16318540	cact	6999	15.6177	3.70
chr2L	16324809	16326259	cact	130	195.97	11.24
chr2L	16350243	16351445	chif	757	64.8459	7.45
chr2L	16351704	16353326	CG42231	372	36.8578	5.37
chr2L	16351704	16353326	chif	372	36.8578	5.37
chr2L	16351704	16353326	CG4455	677	36.8578	5.37
chr2L	16356514	16356880	CaBP1	54	39.1347	5.32
chr2L	16365676	16365979	jhamt	80	17.9011	4.31
chr2L	16422498	16423462	snoRNA:Me28S-A2958	4953	316.742	32.52
chr2L	16447051	16447592	Idgf2	34	66.6063	8.04
chr2L	16447932	16449379	CG5888	537	295.804	20.74
chr2L	16447932	16449379	Idgf2	18	295.804	20.74
chr2L	16450814	16451550	Idgf3	140	316.742	31.12
chr2L	16485515	16487019	dac	476	316.742	19.03
chr2L	16491245	16492078	Tpr2	40	19.7065	3.46
chr2L	16492514	16492991	Tpr2	1100	12.5392	3.15
chr2L	16496434	16496944	Tpr2	94	229.01	19.28
chr2L	16501174	16501568	Tpr2	524	59.1122	8.11
chr2L	16519954	16522722	CG5953	116	316.742	24.40
chr2L	16523030	16523608	CG5953	3130	81.2483	8.11
chr2L	16525857	16526710	mir-4943	2104	316.742	37.02
chr2L	16532594	16533772	CG5953	184	316.742	28.44
chr2L	16534053	16534628	CR45354	191	316.742	20.19
chr2L	16534912	16535218	CR45355	176	18.0307	3.92
chr2L	16535501	16536420	CR45355	565	293.895	21.84
chr2L	16543865	16546313	CG42389	108	316.742	20.38
chr2L	16548003	16548516	CG5968	1618	296.507	23.69
chr2L	16587736	16589225	CG42389	13714	316.742	27.72
chr2L	16601337	16602630	CG42389	225	316.742	24.75
chr2L	16679994	16680527	grp	351	16.6485	3.45
chr2L	16679994	16680527	squ	351	16.6485	3.45
chr2L	16679994	16680527	grp	351	16.6485	3.45
chr2L	16679994	16680527	squ	351	16.6485	3.45
chr2L	16684794	16685709	grp	60	197.851	15.29
chr2L	16689669	16690821	grp	5146	54.1303	7.39
chr2L	16715590	16716879	Cyt-c-d	217	316.742	24.29
chr2L	16715590	16716879	CG31808	18	316.742	24.29
chr2L	16718528	16718989	Cyt-c-p	1100	33.3385	5.77
chr2L	16719745	16721544	Cyt-c-p	1270	138.069	10.05
chr2L	16727458	16727851	VhaSFD	77	16.1989	3.72
chr2L	16743050	16743684	LSm7	452	26.7167	4.33
chr2L	16743050	16743684	BuGZ	148	26.7167	4.33
chr2L	16754066	16754546	yellow-b	52	65.012	7.78
chr2L	16791265	16792369	CG17928	47	316.742	23.96
chr2L	16798700	16799267	Ugt36Bc	56	316.742	28.10
chr2L	16816519	16816873	mdy	30	65.4828	7.62
chr2L	16824879	16825201	Cas	176	11.3159	3.33
chr2L	16852195	16852452	CG31810	51	13.7402	3.08
chr2L	16858098	16859603	CG31809	87	241.484	23.18
chr2L	16862601	16863022	CR43858	1352	28.2939	5.16
chr2L	16877582	16878021	CG32832	1520	10.8303	3.17
chr2L	16880795	16881479	CG31743	122	316.742	24.26
chr2L	16887714	16888081	CG6115	43	24.3905	4.29
chr2L	16888380	16889080	tweek	46	11.5829	2.97
chr2L	16888380	16889080	CR44392	29	11.5829	2.97
chr2L	16972720	16973298	beat-IIIb	118	72.3722	8.38
chr2L	17102366	17102746	CR45448	2638	56.4866	9.04
chr2L	17130802	17131322	beat-IIIa	28	66.2073	7.73
chr2L	17130802	17131322	CG34106	28	66.2073	7.73
chr2L	17382649	17383064	Lrch	1273	26.8457	5.09
chr2L	17383852	17384255	Lrch	51	44.9666	5.26
chr2L	17384600	17385525	CLIP-190	323	38.9044	5.57
chr2L	17387695	17388300	CLIP-190	1252	30.8981	4.58
chr2L	17388967	17389589	CLIP-190	63	316.742	25.41

chr2L	17410375	17410646	Rpb11	9	6.34221	2.33
chr2L	17410375	17410646	CG15141	225	6.34221	2.33
chr2L	17427423	17428507	Dif	27	316.742	26.32
chr2L	17429689	17430190	CR44409	221	166.127	13.75
chr2L	17430954	17431376	CR44409	1447	104.541	10.72
chr2L	17433086	17433438	Dif	135	23.3675	4.88
chr2L	17448066	17448808	dl	91	94.2182	9.41
chr2L	17449493	17450738	dl	120	253.921	16.33
chr2L	17449493	17450738	CR46204	9	253.921	16.33
chr2L	17450999	17451312	CR46204	646	32.3855	5.66
chr2L	17466208	17466899	Sgt	5270	9.40611	3.12
chr2L	17471676	17472026	Sgt	241	31.3675	5.07
chr2L	17472474	17473355	BicD	491	30.4622	4.92
chr2L	17558401	17558794	CR45919	2254	13.3343	3.53
chr2L	17560279	17560980	CR45919	107	39.2811	6.19
chr2L	17574504	17574971	mir-287	221	31.0683	5.20
chr2L	17575334	17575735	CR45358	11	22.3373	4.80
chr2L	17589190	17589499	Oli	35	16.8563	4.14
chr2L	17592245	17592579	Sytalpha	138	13.0631	3.63
chr2L	17592245	17592579	CG6870	98	13.0631	3.63
chr2L	17621211	17621581	CR44413	4238	27.0784	4.69
chr2L	17735489	17736012	CadN	16595	98.2341	12.51
chr2L	17751037	17751697	CadN	1105	53.2496	8.09
chr2L	17752283	17752976	CadN	326	237.514	23.54
chr2L	17754009	17754536	CadN	1968	33.1559	6.56
chr2L	17778272	17778576	CadN	86	18.301	4.40
chr2L	17843489	17843945	CadN2	98	31.3257	5.19
chr2L	17963195	17963639	CG5674	17	20.7834	4.35
chr2L	18141543	18141974	CR44478	3372	51.473	6.85
chr2L	18149979	18150894	Socs36E	1742	62.739	8.49
chr2L	18151234	18152725	Socs36E	811	104.432	9.45
chr2L	18155751	18156593	CG5783	283	175.025	15.49
chr2L	18184428	18185415	Ptp36E	104	147.696	16.12
chr2L	18275602	18276166	CG42750	184	47.7485	7.92
chr2L	18313600	18314310	CR44489	229	38.7035	5.73
chr2L	18319904	18320350	Fas3	37	90.8996	8.55
chr2L	18326995	18328030	Fas3	7158	68.9155	8.00
chr2L	18395301	18395682	CR44486	5010	16.3847	3.53
chr2L	18455240	18456551	let-7-C	112	316.742	22.62
chr2L	18456961	18457331	let-7-C	1442	30.3802	5.42
chr2L	18458483	18459158	let-7-C	2950	25.8391	4.64
chr2L	18459694	18460486	let-7-C	4257	300.994	19.35
chr2L	18465164	18466173	mir-100	5571	84.0753	9.09
chr2L	18469478	18470043	mir-100	1627	107.456	10.39
chr2L	18483796	18484750	CG10283	15	316.742	17.78
chr2L	18485172	18485587	CG10176	25	42.8099	5.87
chr2L	18509339	18509631	CG10178	61	10.7391	2.95
chr2L	18517132	18517629	CG10211	145	89.052	10.33
chr2L	18553985	18554248	Pde11	9108	10.1941	3.21
chr2L	18557473	18558538	Pde11	5133	186.269	15.79
chr2L	18561690	18563604	Pde11	116	159.579	10.37
chr2L	18569961	18570311	Pde11	2161	33.3861	5.78
chr2L	18571575	18573297	Pde11	3	252.466	16.55
chr2L	18604738	18605048	CG10413	183	10.6639	3.01
chr2L	18617069	18617522	MESR3	5	58.8054	8.36
chr2L	18629005	18629655	MESR3	12111	316.742	26.44
chr2L	18677205	18677898	CG31751	255	37.2295	5.04
chr2L	18695289	18695782	CG10383	43	14.4224	3.26
chr2L	18695289	18695782	CG10338	104	14.4224	3.26
chr2L	18708087	18708696	Faf2	243	24.7008	4.39
chr2L	18728598	18729088	CG10348	2560	68.7037	9.21
chr2L	18731731	18731997	CG10348	333	5.94722	2.32
chr2L	18732317	18733370	CR44768	700	97.151	9.94
chr2L	18732317	18733370	CG10348	639	97.151	9.94
chr2L	18735556	18736113	CR44769	202	27.8114	5.52
chr2L	18737296	18737583	CR44769	1710	10.8049	3.26
chr2L	18748995	18749535	CG15167	8874	72.9341	8.05
chr2L	18753337	18753875	CG15167	13285	102.986	11.82
chr2L	18782829	18783794	ham	962	31.3926	6.24
chr2L	18795748	18796931	CR44904	77	316.742	36.86
chr2L	18809519	18809844	CG42502	1235	7.71266	2.48
chr2L	18809519	18809844	CG10570	1235	7.71266	2.48
chr2L	18809519	18809844	CG42502	1235	7.71266	2.48
chr2L	18810790	18811805	CG42502	221	65.7224	8.96
chr2L	18810790	18811805	CG10570	221	65.7224	8.96

chr2L	18810790	18811805	CG42502	45	65.7224	8.96
chr2L	18810790	18811805	CG10570	45	65.7224	8.96
chr2L	18813039	18813398	CG42305	341	8.98115	2.93
chr2L	18813039	18813398	CG17325	341	8.98115	2.93
chr2L	18831083	18831618	CG17597	161	25.837	4.48
chr2L	18831083	18831618	ScpX	86	25.837	4.48
chr2L	18838695	18839905	CG10600	125	41.1335	4.96
chr2L	18844488	18845577	CG33120	559	303.162	26.59
chr2L	18844488	18845577	CG17321	45	303.162	26.59
chr2L	18846801	18847317	CG17321	1880	103.636	11.95
chr2L	18850779	18851186	CG17321	5763	15.6489	4.05
chr2L	18881096	18881977	tup	237	316.742	22.05
chr2L	18936044	18936548	ssp3	7702	152.785	13.28
chr2L	18943911	18944530	ssp3	341	44.7231	5.68
chr2L	18965948	18966916	Nak	1976	26.6638	4.46
chr2L	18968454	18968780	Nak	190	10.8708	2.91
chr2L	18973911	18974409	CG10639	15	68.7592	7.58
chr2L	18985485	18986262	Swip-1	1686	11.7131	3.19
chr2L	18987017	18987390	Swip-1	53	24.766	4.16
chr2L	19008979	19009528	RpL30	35	23.8466	4.11
chr2L	19011893	19013272	CR45292	950	25.7809	5.50
chr2L	19014925	19015446	CR45292	3310	10.3226	3.48
chr2L	19021337	19021890	CG15172	690	17.4341	4.57
chr2L	19023946	19024387	CR43700	240	13.756	3.80
chr2L	19024724	19025273	CR43700	427	29.7189	5.86
chr2L	19027078	19027994	Sidpn	75	31.2105	5.83
chr2L	19045254	19045722	Acn	20	16.1194	3.56
chr2L	19054099	19054774	CG10492	344	63.743	7.14
chr2L	19083274	19084003	Lim3	154	28.5332	5.28
chr2L	19108581	19108871	CR44905	32	14.7	4.17
chr2L	19119998	19121150	Ddc	7	155.368	13.56
chr2L	19133523	19134489	brat	294	12.4855	3.26
chr2L	19134842	19135630	l(2)37Cg	177	83.295	8.80
chr2L	19157909	19159293	brat	134	316.742	21.07
chr2L	19161371	19161832	CR44977	2350	22.2417	4.57
chr2L	19164708	19166384	brat	555	148.859	13.45
chr2L	19190141	19190618	drl	50	62.6653	6.50
chr2L	19361905	19363407	dnt	248	316.742	19.09
chr2L	19389632	19390269	CG17549	94	113.656	10.57
chr2L	19397902	19399347	CG17544	523	316.742	32.78
chr2L	19413863	19414752	Pax	379	109.191	10.96
chr2L	19416180	19416764	Pax	13	26.3157	4.51
chr2L	19422361	19422873	Pax	897	59.9528	7.62
chr2L	19423214	19424314	Pax	49	316.742	38.79
chr2L	19423214	19424314	CG16771	448	316.742	38.79
chr2L	19423214	19424314	CG13085	448	316.742	38.79
chr2L	19425076	19425507	CG13085	1013	35.8682	6.11
chr2L	19425076	19425507	CG16771	1013	35.8682	6.11
chr2L	19426134	19426832	CG13085	310	128.909	9.76
chr2L	19426134	19426832	CG16771	152	128.909	9.76
chr2L	19426134	19426832	Pax	11	128.909	9.76
chr2L	19433627	19433896	Rab9	1178	9.90151	2.67
chr2L	19437859	19438628	CG10237	523	13.2303	3.13
chr2L	19440731	19441579	CG10237	28	14.7065	3.48
chr2L	19441962	19442416	RanGAP	156	9.8176	2.99
chr2L	19444686	19445114	Hs2st	270	10.3923	2.82
chr2L	19444686	19445114	RanGAP	27	10.3923	2.82
chr2L	19454077	19455117	CG10026	87	82.8306	8.81
chr2L	19463788	19464418	tj	257	71.5856	9.55
chr2L	19479545	19480047	CG10195	4883	31.4312	4.96
chr2L	19508299	19508809	CG10188	122	16.4803	3.72
chr2L	19518638	19519108	CG10132	56	47.2097	6.52
chr2L	19519537	19520035	CG10132	893	106.414	10.33
chr2L	19528009	19528949	CG10165	8	230.768	16.46
chr2L	19542459	19542847	ref(2)P	189	21.4591	3.95
chr2L	19548113	19548713	Tep4	84	228.289	16.34
chr2L	19549385	19549937	Tep4	32	45.136	6.24
chr2L	19570937	19573362	spi	644	316.742	30.94
chr2L	19574772	19577292	spi	587	316.742	27.22
chr2L	19587182	19587570	Lar	120	18.4366	4.00
chr2L	19590620	19591066	Lar	3597	38.4774	6.64
chr2L	19605329	19605895	CR44978	173	63.4793	7.54
chr2L	19606234	19606656	CG46244	145	45.1666	6.71
chr2L	19606234	19606656	Lar	145	45.1666	6.71
chr2L	19735613	19736100	CG10462	108	33.3953	4.79

chr2L	19735613	19736100	Nf-YB	215	33.3953	4.79
chr2L	19756066	19757064	CG10631	10	37.9558	5.10
chr2L	19764572	19764844	fbp	568	15.7254	3.83
chr2L	19791103	19791866	CdGAPr	89	9.34759	2.74
chr2L	19809587	19809965	snRNA:U4:38AB	945	40.6205	5.98
chr2L	19819836	19820394	snRNA:U5:38ABb	3654	64.7608	8.67
chr2L	19865806	19866178	sick	205	18.8892	3.55
chr2L	19895167	19895572	sick	590	25.9722	5.63
chr2L	19895952	19896279	sick	62	11.3602	3.29
chr2L	19917066	19917388	sick	944	25.1028	4.88
chr2L	19917939	19918245	sick	34	23.9447	4.72
chr2L	19935034	19935600	sick	7	91.9537	9.89
chr2L	19948945	19949222	COX4	9578	11.3342	3.56
chr2L	19962316	19962865	CG13965	266	47.6234	6.44
chr2L	19962316	19962865	CG16772	60	47.6234	6.44
chr2L	19963997	19964370	CG10680	37	17.5331	3.98
chr2L	19963997	19964370	CR45701	138	17.5331	3.98
chr2L	20066428	20066861	bwa	351	10.5121	2.98
chr2L	20075014	20075778	pr	353	11.1002	3.19
chr2L	20075014	20075778	neb	651	11.1002	3.19
chr2L	20080716	20081043	fok	677	13.9129	3.46
chr2L	20082465	20082776	fok	2394	11.7751	3.24
chr2L	20084720	20085698	fok	11	128.133	9.35
chr2L	20086280	20086579	fok	1335	19.8529	3.78
chr2L	20120855	20121308	CG10651	13856	120.084	9.16
chr2L	20242804	20243092	CR40341	5052	30.0859	6.03
chr2L	20290758	20291024	CG12617	3795	13.4655	3.67
chr2L	20310673	20311464	spir	13	158.285	11.43
chr2L	20321218	20321818	CR43606	3951	50.9105	7.93
chr2L	20341609	20342134	spir	6	40.4196	5.24
chr2L	20350308	20350611	RtGEF	3	19.3445	4.07
chr2L	20382139	20382595	CG16798	45	80.7893	8.35
chr2L	20416966	20418024	Arpc2	384	307.857	19.12
chr2L	20425970	20426381	CG31688	36	22.8586	4.91
chr2L	20428429	20429422	CG31688	72	43.4236	5.34
chr2L	20561273	20562404	CR45347	61	41.1492	7.45
chr2L	20561273	20562404	mir-4973	112	41.1492	7.45
chr2L	20638204	20638722	CG31680	1692	60.0564	8.19
chr2L	20671422	20671734	CG31678	3482	18.1774	4.30
chr2L	20700878	20701152	CR44187	9020	19.3733	4.43
chr2L	20705812	20706440	CR44187	4008	133.565	14.69
chr2L	20714332	20715109	Hr38	104	316.742	34.35
chr2L	20715833	20716642	Hr38	1577	149.878	13.98
chr2L	20719950	20720600	CR45306	1734	41.5796	6.22
chr2L	20758091	20759060	dia	661	55.5616	6.50
chr2L	20759454	20759786	dia	653	16.9601	3.59
chr2L	20770302	20770820	cad	168	145.293	12.48
chr2L	20783123	20783466	Pomp	4380	38.0233	6.20
chr2L	20793470	20794419	vari	64	316.742	23.89
chr2L	20797561	20797962	CG9328	32	42.1713	4.48
chr2L	20803700	20804030	CG9328	5990	8.66683	2.98
chr2L	20808050	20809353	CG9328	843	29.5898	5.07
chr2L	20821469	20822125	CheB38c	882	288.135	19.15
chr2L	20822537	20823186	CG9331	51	148.882	12.80
chr2L	20823516	20825786	CG9331	5	209.133	18.37
chr2L	20829391	20830183	CG31673	20	97.036	11.20
chr2L	20831089	20831570	CG31674	35	116.644	10.37
chr2L	20840401	20840928	CG31676	56	43.8783	6.25
chr2L	20858990	20859402	CG9336	20	35.3581	5.25
chr2L	20864578	20865161	CG9338	358	23.7283	4.44
chr2L	20869834	20870214	CG14401	248	9.45705	3.08
chr2L	20877952	20878326	CR44981	3892	11.3468	3.07
chr2L	20885729	20886461	sky	223	189.067	12.17
chr2L	20890135	20890793	sky	40	316.742	23.83
chr2L	20904443	20904721	sky	12808	12.327	3.27
chr2L	20917139	20918461	sky	29	78.6001	7.89
chr2L	20922602	20923133	Mtp	57	44.3727	6.19
chr2L	21051726	21052355	CG42238	89	93.4442	9.74
chr2L	21065872	21066270	CG9265	3887	17.0726	4.32
chr2L	21071423	21071700	CG9265	51	17.9567	4.32
chr2L	21075107	21075560	tadr	16	15.6368	3.71
chr2L	21096515	21097164	CG34136	140	274.858	23.40
chr2L	21141697	21142443	Dap160	436	63.9201	6.49
chr2L	21162858	21163123	Nbr	85	18.0307	3.92
chr2L	21175829	21176177	caRNA:PsiU2-38.40.4	1113	9.31732	2.92

chr2L	21177283	21177612	caRNA:PsiU2-38.40.4	220	14.9772	3.40
chr2L	21178475	21179691	bur	793	67.8798	8.17
chr2L	21218318	21218963	CG8679	206	92.2177	11.15
chr2L	21219604	21220225	Atg18b	691	17.6046	3.74
chr2L	21221286	21222290	CG8677	662	316.742	26.17
chr2L	21237072	21238333	Hr39	606	316.742	29.09
chr2L	21250467	21251163	l(2)k14505	10306	34.3409	5.45
chr2L	21309166	21310286	Mondo	54	107.438	9.61
chr2L	21310811	21311183	crc	129	17.2033	3.43
chr2L	21312924	21313524	crc	1082	302.842	19.25
chr2L	21313795	21314756	crc	32	316.742	33.63
chr2L	21344384	21345209	Tsp39D	272	41.9156	5.56
chr2L	21379973	21380253	nrv3	10	18.394	4.28
chr2L	21381116	21381652	nrv3	1367	18.7317	4.29
chr2L	21572774	21573094	Lamp1	469	50.6563	7.47
chr2L	21618615	21619185	CG2201	59	90.0037	9.67
chr2L	21623425	21623913	CG2201	62	203.552	12.76
chr2L	21627290	21627566	CR44993	1032	32.1549	5.02
chr2L	21628419	21630153	CR44993	1468	184.001	12.22
chr2L	21628419	21630153	Df31	3	184.001	12.22
chr2L	21632385	21633403	Ac3	483	27.5095	4.76
chr2L	21639633	21639965	Ac3	7183	27.8653	5.71
chr2L	21676457	21677009	CG2225	516	150.937	12.11
chr2L	21681693	21682222	EF2	34	28.4374	4.64
chr2L	21683163	21684063	nolo	273	34.3257	4.69
chr2L	21753505	21754213	step	110	307.442	20.22
chr2L	21757183	21757559	step	105	12.5392	3.15
chr2L	21794550	21795628	CG31612	665	316.742	25.61
chr2L	21819448	21819743	CR44786	81	18.301	4.40
chr2L	21827944	21829142	tsh	63	316.742	27.56
chr2L	21830336	21831588	tsh	2194	79.8711	9.25
chr2L	21863651	21863960	CR44918	2482	15.2491	3.96
chr2L	21866406	21866808	CR44918	297	55.2324	8.18
chr2L	21899045	21899912	CG11629	1182	316.742	42.63
chr2L	22018531	22019559	tio	64	316.742	54.26
chr2L	22018531	22019559	CR33987	64	316.742	54.26
chr2L	22018531	22019559	tio	178	316.742	54.26
chr2L	22024046	22024998	tio	5163	316.742	26.53
chr2L	22108498	22109130	CG6675	8	24.1886	4.52
chr2L	22117460	22118136	CG3651	3489	118.915	12.36
chr2L	22121898	22122182	CG3651	195	9.18791	2.81
chr2L	22133979	22134637	CG42748	58	316.742	30.99
chr2L	22142632	22142942	CG42748	2695	18.4702	4.44
chr2L	22143942	22144459	CG42748	4108	194.67	13.47
chr2L	22145534	22145941	CG42748	5614	35.8523	5.45
chr2L	22148717	22149096	CG34137	2821	106.406	9.63
chr2L	22419904	22420179	CG17018	65662	22.5747	5.26
chr2L	22549775	22550348	CG17493	36	146.268	14.79
chr2L	22756176	22756643	CG40006	68	167.391	12.12
chr2R	601773	602097	CG45781	169651	87.2863	11.92
chr2R	4270533	4271053	IntS3	27832	49.9213	6.45
chr2R	5413446	5413842	laccase2	106	90.4687	8.87
chr2R	5495689	5496760	CG30440	32	26.9524	4.97
chr2R	5495689	5496760	CG30438	844	26.9524	4.97
chr2R	5608615	5608882	CG17508	113	27.1281	4.70
chr2R	5726702	5727309	ap	206	316.742	22.34
chr2R	5772969	5773421	scaRNA:PsiU2-35.45	8016	165.667	16.06
chr2R	5782085	5783091	ZnT41F	472	276.945	18.11
chr2R	5804293	5804843	dpr12	161	67.1236	7.79
chr2R	5981909	5982695	Src42A	366	85.0153	9.24
chr2R	5992498	5993243	Src42A	178	316.742	21.85
chr2R	6022987	6023443	Strica	40	52.5089	8.39
chr2R	6035267	6036564	SCAP	361	24.1333	4.35
chr2R	6040197	6040530	CG14591	38	23.2788	4.31
chr2R	6102136	6103300	Ars2	16962	316.742	27.25
chr2R	6103810	6104492	Ars2	18569	48.3168	7.12
chr2R	6120307	6121026	EcR	11266	316.742	17.98
chr2R	6125055	6125423	EcR	6596	20.9884	4.16
chr2R	6131640	6132467	EcR	179	316.742	33.23
chr2R	6132734	6134865	EcR	1246	316.742	29.49
chr2R	6142446	6143007	CG14589	5496	51.8744	6.17
chr2R	6145615	6146292	CG14589	8739	18.1656	4.11
chr2R	6151076	6151517	EcR	5864	28.441	4.49
chr2R	6156189	6157464	EcR	435	316.742	20.41
chr2R	6157951	6160083	CR43904	480	316.742	20.09

chr2R	6168258	6169290	EcR	3	101.246	9.83
chr2R	6173586	6173913	Cyp6w1	50	26.4355	4.81
chr2R	6195838	6196610	Ptr	0	209.828	19.12
chr2R	6214565	6214836	bin3	94	15.2728	3.30
chr2R	6220180	6222241	bin3	577	316.742	18.83
chr2R	6235319	6236430	Pld	5895	316.742	34.21
chr2R	6241794	6242519	Pld	48	316.742	22.94
chr2R	6245275	6245564	Pld	104	17.7908	4.22
chr2R	6500450	6502303	CR46075	9	254.816	14.89
chr2R	6500450	6502303	jing	224	254.816	14.89
chr2R	6505496	6505948	jing	3793	11.3812	3.20
chr2R	6518521	6519110	CR45127	8509	153.022	14.01
chr2R	6519939	6520584	CR45127	10036	21.2502	4.06
chr2R	6574697	6575583	CG15233	25843	93.1871	9.34
chr2R	6610101	6611246	jing	104	97.6167	11.07
chr2R	6612871	6613829	jing	612	83.4579	8.66
chr2R	6627707	6628992	CG33919	3799	316.742	38.54
chr2R	6629572	6633303	Vha16-1	105	316.742	40.07
chr2R	6644901	6645466	Fmo-2	86	47.2418	6.92
chr2R	6648443	6649332	Debcl	109	217.696	17.17
chr2R	6661973	6663528	Dpit47	1046	93.2309	9.26
chr2R	6661973	6663528	Adf1	895	93.2309	9.26
chr2R	6673151	6673660	Hsepi	26	19.5354	4.16
chr2R	6673151	6673660	PGAP3	257	19.5354	4.16
chr2R	6696560	6696864	Mob4	10	13.3609	3.19
chr2R	6696560	6696864	Rab2	67	13.3609	3.19
chr2R	6715196	6716003	CG3409	44	35.5974	5.35
chr2R	6715196	6716003	CG45092	44	35.5974	5.35
chr2R	6741818	6742584	phtf	22	10.7626	2.80
chr2R	6749155	6749677	Eb1	53	22.3717	4.06
chr2R	6750002	6750513	Eb1	766	22.7933	4.75
chr2R	6758622	6759129	CG9436	16	33.0765	5.30
chr2R	6781416	6782110	Cyp6a2	184	59.1555	7.93
chr2R	6798817	6799188	Epac	29	34.6748	5.72
chr2R	6799812	6800140	Epac	1020	25.6968	5.66
chr2R	6807681	6807955	SdhB	252	12.4792	3.50
chr2R	6810932	6811320	koi	36	24.8525	4.22
chr2R	6828049	6828600	CG15236	32	164.714	17.97
chr2R	6871394	6873138	coro	586	282.026	16.42
chr2R	6878196	6879031	Spn42Da	66	316.742	28.63
chr2R	6880752	6881120	Spn42Db	173	26.5566	5.03
chr2R	6880752	6881120	Spn42Dc	51	26.5566	5.03
chr2R	6882837	6883665	Spn42Dd	58	316.742	28.17
chr2R	6885504	6885894	Spn42De	181	14.7609	3.63
chr2R	6923112	6923547	CG3358	88	48.259	6.33
chr2R	6970973	6971735	mim	46	16.6496	3.38
chr2R	6970973	6971735	CheB42c	46	16.6496	3.38
chr2R	6970973	6971735	mim	46	16.6496	3.38
chr2R	6985111	6985807	CG30156	161	20.4882	4.12
chr2R	6985111	6985807	vimar	161	20.4882	4.12
chr2R	6991834	6993130	Tsp42Ea	156	247.711	17.11
chr2R	6991834	6993130	CG30159	151	247.711	17.11
chr2R	6991834	6993130	Tsp42Ea	151	247.711	17.11
chr2R	6991834	6993130	CG30159	151	247.711	17.11
chr2R	6991834	6993130	Tsp42Ea	151	247.711	17.11
chr2R	6991834	6993130	CG30159	88	247.711	17.11
chr2R	7012038	7013532	Tsp42Ee	114	82.6389	7.62
chr2R	7014484	7015123	Tsp42Ee	2163	16.7281	3.68
chr2R	7024793	7025653	Tsp42Ef	273	316.742	25.73
chr2R	7028632	7029104	Tsp42Eg	91	50.6989	6.87
chr2R	7031290	7031735	Tsp42Eh	2053	35.0921	5.39
chr2R	7034878	7035551	Tsp42Ei	45	64.161	8.26
chr2R	7039840	7040459	Tsp42Ej	66	141.426	13.30
chr2R	7045158	7046116	Tsp42El	28	92.5482	8.77
chr2R	7049507	7049942	lbm	64	88.1761	10.87
chr2R	7106266	7106838	esn	146	108.252	11.56
chr2R	7129612	7129945	Cyp9b1	47	32.5306	5.85
chr2R	7131863	7132788	Cyp9b2	194	316.742	23.10
chr2R	7146716	7147274	Spn43Aa	698	264.225	19.60
chr2R	7150246	7150981	pk	76	316.742	33.06
chr2R	7153436	7153844	Spn43Ab	5	36.1627	6.08
chr2R	7155224	7156567	Spn43Ad	128	316.742	29.96
chr2R	7157956	7158692	nec	98	316.742	28.45
chr2R	7187088	7187485	pk	46	36.9617	5.64
chr2R	7245301	7246106	Ady43A	119	138.227	7.39

chr2R	7248553	7249425	Gadd45	77	316.742	40.42
chr2R	7252144	7252421	CR45280	2325	8.66213	2.77
chr2R	7252974	7253332	CR45280	1484	16.1583	4.11
chr2R	7253886	7254340	CR45280	475	61.3346	8.04
chr2R	7300721	7301025	CG12164	10539	55.0748	7.56
chr2R	7352179	7353062	CR44071	9267	316.742	27.65
chr2R	7371285	7371539	Dscam1	10442	23.2184	5.16
chr2R	7373866	7374250	Dscam1	7868	20.2224	4.55
chr2R	7381387	7382236	Dscam1	60	316.742	33.01
chr2R	7407210	7407928	Eaf	329	10.1626	2.94
chr2R	7418381	7419623	so	671	244.226	19.49
chr2R	7427336	7427732	CG11145	967	19.2306	4.41
chr2R	7429141	7429783	CR44203	665	20.5555	4.78
chr2R	7457357	7459229	Inos	74	316.742	28.88
chr2R	7464785	7465278	CG11127	1592	25.6008	4.91
chr2R	7470889	7471587	Aldh-III	2885	13.4928	3.51
chr2R	7473539	7474196	Aldh-III	230	29.3219	4.96
chr2R	7474454	7474747	Aldh-III	54	22.1667	4.12
chr2R	7477587	7477908	Aldh-III	603	12.7744	3.23
chr2R	7478642	7479145	Aldh-III	1643	15.5592	3.55
chr2R	7487883	7489270	wech	139	311.451	21.04
chr2R	7489579	7490867	wech	734	256.155	16.02
chr2R	7491999	7493347	Coop	108	248.968	19.69
chr2R	7491999	7493347	CR43723	57	248.968	19.69
chr2R	7491999	7493347	Coop	169	248.968	19.69
chr2R	7500035	7500425	dpa	24	9.15002	2.50
chr2R	7500035	7500425	didum	25	9.15002	2.50
chr2R	7528978	7529777	Drat	43	60.1817	6.71
chr2R	7530635	7531250	Drat	99	214.11	13.78
chr2R	7533327	7533968	CR44209	143	25.3252	4.16
chr2R	7533327	7533968	Cyt-b5	135	25.3252	4.16
chr2R	7543998	7544510	Corin	815	54.6975	8.29
chr2R	7544932	7545555	Corin	61	60.6501	8.62
chr2R	7566661	7567014	boca	112	27.0657	4.53
chr2R	7566661	7567014	Vps13	42	27.0657	4.53
chr2R	7586844	7587397	blow	56	105.379	11.77
chr2R	7590042	7590367	CG1360	97	13.3	3.14
chr2R	7590042	7590367	scra	5	13.3	3.14
chr2R	7620586	7621070	CG1358	57	27.0355	5.13
chr2R	7622941	7623480	CG43340	73	90.3671	10.92
chr2R	7632352	7633416	CG43341	137	316.742	42.49
chr2R	7636158	7637127	CG43340	32	39.641	6.18
chr2R	7663553	7664025	CG2065	53	63.6563	9.11
chr2R	7665597	7666063	CG2064	3	160.93	11.91
chr2R	7692740	7693211	LRR	2459	28.0323	5.66
chr2R	7697785	7698170	LRR	854	26.09	4.94
chr2R	7698808	7699722	LRR	576	79.6269	7.56
chr2R	7698808	7699722	CG30493	226	79.6269	7.56
chr2R	7698808	7699722	CG30496	21	79.6269	7.56
chr2R	7709644	7710135	CG1941	21	75.976	8.11
chr2R	7722345	7723498	CG18812	96	316.742	32.30
chr2R	7730358	7730747	CG18812	3076	13.681	3.61
chr2R	7731168	7731697	CG18812	3939	32	4.69
chr2R	7735013	7735712	CG18812	130	49.182	6.85
chr2R	7742923	7743292	CG45093	881	8.73854	2.95
chr2R	7742923	7743292	CG30497	881	8.73854	2.95
chr2R	7745051	7745868	CG30497	88	316.742	27.53
chr2R	7745051	7745868	CG45093	88	316.742	27.53
chr2R	7758521	7759318	CG45093	6	88.798	8.56
chr2R	7758521	7759318	CG30497	6	88.798	8.56
chr2R	7766323	7767934	CG45093	8778	143.846	14.35
chr2R	7766323	7767934	CG30497	8778	143.846	14.35
chr2R	7779348	7780107	CG30497	3005	238.253	18.67
chr2R	7779348	7780107	CG45093	3005	238.253	18.67
chr2R	7782389	7783358	CG30497	570	277.622	19.64
chr2R	7782389	7783358	CG45093	570	277.622	19.64
chr2R	7782389	7783358	CR45108	126	277.622	19.64
chr2R	7785985	7786540	CanB2	297	35.6154	5.23
chr2R	7785985	7786540	mir-4980	15	35.6154	5.23
chr2R	7792982	7793369	Gapdh1	24	27.8127	5.07
chr2R	7805241	7806543	Nop17l	74	240.193	16.75
chr2R	7805241	7806543	sax	843	240.193	16.75
chr2R	7812322	7813129	CG1882	97	97.3296	9.19
chr2R	7885791	7886454	Dgk	119	289.18	21.10
chr2R	7888106	7888469	CG30377	1736	42.8731	6.71

chr2R	7916774	7917705	Cul1	184	39.818	5.92
chr2R	7955478	7955971	CG14764	64	12.7948	3.32
chr2R	7959344	7960049	CG2915	260	316.742	27.19
chr2R	7983698	7984573	ACC	98	316.742	40.61
chr2R	7989032	7989934	ACC	461	32.2991	4.99
chr2R	7989032	7989934	Nup44A	605	32.2991	4.99
chr2R	7991896	7992168	ACC	167	15.2212	3.89
chr2R	8001951	8002749	CG11191	58	28.9637	5.69
chr2R	8012987	8013923	CG14762	12	32.8839	5.09
chr2R	8018657	8019420	CR45267	192	245.146	15.19
chr2R	8024363	8024760	CR43724	4909	17.2529	3.98
chr2R	8029360	8029736	CR43724	94	29.7942	5.31
chr2R	8030635	8031702	Optix	258	89.7191	10.02
chr2R	8051287	8051545	CG12769	7148	13.7162	3.11
chr2R	8060293	8062042	CG12769	2656	316.742	39.53
chr2R	8062358	8063370	CG12769	2963	151.056	14.05
chr2R	8063805	8064439	CG12769	1479	61.0223	7.80
chr2R	8064743	8065880	CG12769	9	316.742	22.48
chr2R	8067906	8069440	lig	383	107.363	9.45
chr2R	8082774	8083653	sut1	35	136.542	12.10
chr2R	8082774	8083653	slv	65	136.542	12.10
chr2R	8121483	8121840	Asap	1340	40.2793	6.73
chr2R	8122707	8123090	Asap	68	10.8256	3.22
chr2R	8122707	8123090	Nup50	102	10.8256	3.22
chr2R	8131276	8132252	Pbp49	563	185.257	11.69
chr2R	8131276	8132252	CG42516	563	185.257	11.69
chr2R	8131276	8132252	Pbp49	563	185.257	11.69
chr2R	8131276	8132252	Pabp2	456	185.257	11.69
chr2R	8143079	8143590	Lpin	831	76.8981	9.04
chr2R	8145392	8146599	Lpin	66	316.742	24.39
chr2R	8150731	8151075	kermit	1417	9.04564	2.90
chr2R	8151782	8152494	kermit	2769	268.687	18.25
chr2R	8156271	8156746	Lpin	176	31.0557	5.34
chr2R	8156271	8156746	kermit	176	31.0557	5.34
chr2R	8173975	8175189	LRP1	32	68.1589	6.05
chr2R	8236775	8238268	pnut	80	264.55	15.98
chr2R	8272209	8273221	CG30371	3523	316.742	39.44
chr2R	8288150	8288466	mir-280	9764	14.3778	3.88
chr2R	8288785	8289085	mir-280	9187	14.8847	3.94
chr2R	8317433	8318167	CR44450	6284	85.4805	10.97
chr2R	8326691	8327769	pdm3	232	316.742	34.99
chr2R	8329018	8329363	pdm3	1658	16.9067	3.91
chr2R	8335950	8336430	pdm3	8703	18.8732	3.93
chr2R	8336736	8337340	pdm3	9555	187.148	14.32
chr2R	8340431	8340805	pdm3	13172	12.1994	3.43
chr2R	8396605	8397160	pdm3	9449	36.1316	5.20
chr2R	8411085	8411492	CG2121	66	24.31	4.93
chr2R	8444515	8444933	Cyp4e2	8	25.3299	5.00
chr2R	8447260	8447680	Cyp4e1	18	44.8909	7.20
chr2R	8462223	8462920	Mal-A5	293	154.96	14.45
chr2R	8516518	8516873	mtt	15626	10.3226	3.48
chr2R	8570262	8570757	Cyp6a13	2	105.363	13.45
chr2R	8608107	8608613	CG8642	5827	40.392	6.76
chr2R	8614510	8615328	Cirl	59	22.1903	3.90
chr2R	8632490	8632818	CG8635	707	24.2061	5.14
chr2R	8647322	8648342	ptc	1851	316.742	34.26
chr2R	8649177	8650495	ptc	133	316.742	42.43
chr2R	8665735	8666975	Acsl	77	316.742	32.92
chr2R	8667397	8668152	Acsl	1537	316.742	32.54
chr2R	8668576	8669634	Acsl	1846	316.742	38.76
chr2R	8670008	8672312	Acsl	147	272.124	18.97
chr2R	8695577	8696841	CG8586	145	316.742	21.12
chr2R	8695577	8696841	CG8738	37	316.742	21.12
chr2R	8723650	8724821	CG14767	696	310.761	18.41
chr2R	8723650	8724821	gcl	278	310.761	18.41
chr2R	8725428	8725722	snoRNA:gcl-a	649	25.1762	4.06
chr2R	8733175	8733564	stmA	37	13.8792	3.28
chr2R	8735308	8736541	CG8740	101	316.742	30.55
chr2R	8746516	8747110	CG8740	2076	52.8269	7.80
chr2R	8774199	8774464	CR44278	5547	15.6489	4.05
chr2R	8798044	8798741	sns	93	302.52	17.35
chr2R	8859502	8859772	RyR	748	16.0637	3.90
chr2R	8860195	8860467	RyR	28	9.43558	3.13
chr2R	8862409	8862805	RyR	156	40.9662	6.60
chr2R	8891164	8891471	CG8272	46	16.2393	3.78

chr2R	8901270	8901802	MrgBP	22	16.7281	3.68
chr2R	8901270	8901802	Ggamma1	6	16.7281	3.68
chr2R	8906788	8907056	CG8258	7	10.8746	3.09
chr2R	8913471	8914047	Pgi	115	27.8929	4.66
chr2R	8928032	8928415	CG8235	47	8.49472	2.47
chr2R	8928032	8928415	PAN2	16	8.49472	2.47
chr2R	8943543	8943852	CR44274	224	7.95929	2.50
chr2R	8944566	8944882	CR44274	1243	12.7505	3.36
chr2R	8947102	8947464	CG8230	62	11.239	3.01
chr2R	8949020	8949564	CG8229	1791	24.6197	4.66
chr2R	8949020	8949564	CG33199	1791	24.6197	4.66
chr2R	8949020	8949564	CG8229	1791	24.6197	4.66
chr2R	8949020	8949564	CG33199	1791	24.6197	4.66
chr2R	8950686	8951213	CG8229	163	40.9377	5.21
chr2R	8950686	8951213	CG33199	163	40.9377	5.21
chr2R	8950686	8951213	CG8229	163	40.9377	5.21
chr2R	8950686	8951213	CG33199	163	40.9377	5.21
chr2R	8967962	8968417	CG8216	50	18.4422	4.66
chr2R	9039462	9039750	Ance-4	2542	29.3291	5.09
chr2R	9059766	9060457	CG13743	324	54.4517	7.27
chr2R	9092604	9093206	CG8080	14	43.3746	5.96
chr2R	9095134	9095515	Uhg4	37	18.0948	3.99
chr2R	9095134	9095515	snoRNA:Psi28S-2949	62	18.0948	3.99
chr2R	9116087	9116709	Su(var)2-10	1	16.4803	3.72
chr2R	9126127	9126396	GstE13	3941	17.4372	3.97
chr2R	9130440	9130723	CG18659	1347	10.0177	2.78
chr2R	9131226	9131836	CG18659	482	30.6476	5.19
chr2R	9132371	9132724	Hydr1	42	9.20515	2.60
chr2R	9137060	9138040	alc	108	215.892	14.91
chr2R	9138324	9138602	CG8788	17	15.8592	3.61
chr2R	9138324	9138602	CG44286	17	15.8592	3.61
chr2R	9138896	9139278	CG44286	18	25.3317	4.73
chr2R	9138896	9139278	CG8788	18	25.3317	4.73
chr2R	9140721	9141386	CG44286	163	85.7419	8.51
chr2R	9144808	9145467	CG30344	261	34.119	5.17
chr2R	9147536	9147936	CG30345	952	62.3725	8.04
chr2R	9148408	9148917	CG30345	72	90.0037	9.67
chr2R	9181145	9181400	Rab32	40	10.7012	3.15
chr2R	9184679	9184957	CR44284	1398	25.6254	4.85
chr2R	9185770	9186493	Rab32	38	42.2411	5.14
chr2R	9191815	9192227	CG8026	43	41.7763	5.08
chr2R	9191815	9192227	CG45085	43	41.7763	5.08
chr2R	9191815	9192227	CG8026	43	41.7763	5.08
chr2R	9194749	9195269	unpg	229	59.6809	8.53
chr2R	9207059	9207861	CG8027	213	44.1734	5.49
chr2R	9207059	9207861	VhaAC45	223	44.1734	5.49
chr2R	9239912	9240295	Cyp4p1	54	10.8746	3.09
chr2R	9247046	9247686	hig	5	41.9921	7.20
chr2R	9274761	9275927	Pkn	29	316.742	22.10
chr2R	9278690	9279391	Pkn	3958	210.653	17.36
chr2R	9284024	9285194	CR45132	564	316.742	24.06
chr2R	9284024	9285194	Pkn	817	316.742	24.06
chr2R	9292100	9293019	Drep2	885	316.742	20.97
chr2R	9293409	9294819	Drep2	131	37.1145	5.47
chr2R	9303144	9303598	Myd88	4961	19.5076	3.68
chr2R	9308004	9308843	Myd88	97	287.349	18.69
chr2R	9376355	9377019	CG13954	11431	51.85	8.02
chr2R	9388091	9388482	CG13954	43	14.659	3.89
chr2R	9408733	9409971	wun	488	316.742	28.02
chr2R	9413216	9413729	wun	11	122.419	11.40
chr2R	9414084	9414474	wun2	61	74.0384	9.92
chr2R	9426293	9427367	Pdk	97	148.303	10.04
chr2R	9427868	9428276	Pdk	1583	28.319	4.73
chr2R	9430000	9431714	Pdk	4387	165.186	12.02
chr2R	9437693	9438122	ced-6	2930	39.1275	5.43
chr2R	9440602	9443971	ced-6	81	316.742	30.16
chr2R	9444822	9445138	ced-6	92	15.5732	3.64
chr2R	9445971	9446801	Camta	317	47.1618	5.97
chr2R	9503450	9503980	brp	72	88.0073	10.25
chr2R	9504518	9504807	brp	894	16.4023	4.23
chr2R	9506167	9506614	brp	2605	59.47	7.89
chr2R	9515629	9515938	brp	682	14.4217	3.42
chr2R	9519409	9519825	brp	3156	24.0474	4.95
chr2R	9540389	9540769	CG1888	6377	119.073	13.16
chr2R	9542533	9543158	CG1888	4083	17.2898	3.69

chr2R	9544073	9544723	CG1888	2685	21.1815	4.00
chr2R	9546511	9547362	CG1888	101	40.0501	5.89
chr2R	9547995	9548486	CG1888	117	43.0252	6.33
chr2R	9549910	9550470	CG1888	2131	26.1709	4.70
chr2R	9550840	9551317	CR43651	1712	16.7012	3.63
chr2R	9551768	9555635	CR43651	80	225.341	13.26
chr2R	9551768	9555635	mir-14	838	225.341	13.26
chr2R	9556076	9556824	mir-14	2617	29.9046	5.19
chr2R	9564883	9565788	CR45281	102	99.9817	8.72
chr2R	9577264	9579059	CG1814	107	139.709	12.17
chr2R	9577264	9579059	Not1	477	139.709	12.17
chr2R	9577264	9579059	CR46257	1371	139.709	12.17
chr2R	9577264	9579059	Not1	1474	139.709	12.17
chr2R	9579415	9579728	CG1814	86	12.0706	3.35
chr2R	9594042	9594443	CG1902	235	18.8182	3.87
chr2R	9594042	9594443	CG30338	235	18.8182	3.87
chr2R	9658006	9658426	Mmp2	18	33.1096	5.58
chr2R	9683689	9684132	Mmp2	74	71.3206	8.38
chr2R	9691870	9693420	Uba1	588	155.724	13.18
chr2R	9711346	9712993	dap	69	316.742	25.45
chr2R	9806716	9807038	CG1688	16	17.3282	4.06
chr2R	9813040	9813333	CG1648	371	14.76	3.49
chr2R	9814074	9814453	lectin-46Cb	1264	13.589	3.44
chr2R	9819823	9820436	CG1648	112	183.957	12.78
chr2R	9833282	9834227	hebe	71	56.4004	7.36
chr2R	9835055	9835404	hebe	1438	21.1199	4.47
chr2R	9836449	9836988	hebe	31	13.0667	3.21
chr2R	9836449	9836988	CG1663	373	13.0667	3.21
chr2R	9860048	9862298	PCB	66	316.742	21.64
chr2R	9860048	9862298	CR44293	319	316.742	21.64
chr2R	9860048	9862298	PCB	718	316.742	21.64
chr2R	9875049	9875475	cbx	175	68.0132	7.48
chr2R	9875049	9875475	CG12744	33	68.0132	7.48
chr2R	9881945	9882308	Sec24AB	210	22.7232	4.31
chr2R	9903061	9904222	CG1441	574	178.731	15.71
chr2R	9910759	9911112	Etf-QO	30	13.6026	3.27
chr2R	9929498	9930356	Mef2	246	131.568	14.59
chr2R	9942777	9943103	Mef2	1799	27.1007	4.39
chr2R	9945723	9946002	Mef2	1118	20.3396	3.99
chr2R	9949233	9950060	Mef2	4813	316.742	27.65
chr2R	9954598	9955998	Mef2	3562	316.742	41.57
chr2R	9957416	9959063	Mef2	664	316.742	34.92
chr2R	9959914	9960305	Pal1	13	8.75836	2.55
chr2R	9978925	9979452	eve	76	63.5953	9.73
chr2R	9988170	9989312	TER94	645	206.194	17.31
chr2R	9988170	9989312	CR45324	772	206.194	17.31
chr2R	10003136	10003552	mir-4967	8899	9.69091	3.36
chr2R	10025088	10026376	Pka-R2	49	316.742	31.62
chr2R	10039940	10040749	oys	610	14.7196	3.28
chr2R	10045843	10046097	CR44295	1936	15.0172	3.58
chr2R	10047971	10048684	COX7C	168	20.4149	3.79
chr2R	10047971	10048684	oys	435	20.4149	3.79
chr2R	10052312	10053031	magu	7	198.449	15.96
chr2R	10052312	10053031	CR44297	448	198.449	15.96
chr2R	10076354	10076652	CG1371	825	15.6368	3.71
chr2R	10077034	10077388	egr	1152	18.8173	4.02
chr2R	10078030	10078664	egr	24	316.742	28.06
chr2R	10087364	10087794	CG2269	13	72.6341	10.00
chr2R	10096940	10097339	Jra	161	12.8188	2.93
chr2R	10099507	10102475	RpLP0-like	1365	316.742	32.89
chr2R	10099507	10102475	14-3-3zeta	80	316.742	32.89
chr2R	10103171	10103661	14-3-3zeta	290	24.7219	4.74
chr2R	10110879	10111273	Pfk	256	15.6407	3.32
chr2R	10123601	10125408	gem	84	316.742	28.79
chr2R	10125712	10126159	gem	9	36.6575	5.65
chr2R	10129559	10129976	gem	3802	30.8116	5.22
chr2R	10134566	10135377	gem	497	316.742	24.33
chr2R	10155930	10156695	KCNQ	1235	72.0408	8.59
chr2R	10158015	10158939	mlt	21	237.216	15.74
chr2R	10159339	10160429	mlt	951	316.742	40.11
chr2R	10192077	10192872	KCNQ	2	140.742	11.29
chr2R	10210808	10211795	Hr46	171	203.068	16.93
chr2R	10213850	10214579	Hr46	2901	316.742	25.83
chr2R	10224580	10225613	Hr46	2780	216.931	19.30
chr2R	10227785	10228573	Hr46	221	271.012	23.21

chr2R	10233550	10235072	CG12912	299	32.6254	5.63
chr2R	10233550	10235072	Hr46	299	32.6254	5.63
chr2R	10233550	10235072	CG12912	299	32.6254	5.63
chr2R	10238029	10238755	CR45265	308	127.783	13.85
chr2R	10252196	10252450	Syb	83	12.0465	3.25
chr2R	10270853	10271937	CAP	654	45.5752	6.18
chr2R	10275318	10275814	CAP	95	27.6062	4.96
chr2R	10302548	10303630	CAP	40	316.742	25.54
chr2R	10304274	10304638	CAP	1224	10.3833	3.16
chr2R	10350016	10350305	CG43172	1783	19.2862	4.28
chr2R	10383488	10383847	Ir47a	3565	23.2452	5.34
chr2R	10395081	10396034	CG42732	127	30.6062	6.26
chr2R	10417875	10418993	Prx2540-2	529	316.742	24.44
chr2R	10420844	10422310	CG11825	461	316.742	26.63
chr2R	10420844	10422310	CG33474	175	316.742	26.63
chr2R	10420844	10422310	CG11825	378	316.742	26.63
chr2R	10420844	10422310	CG33474	661	316.742	26.63
chr2R	10423102	10424609	CG11825	200	316.742	29.02
chr2R	10423102	10424609	Prx2540-1	585	316.742	29.02
chr2R	10427765	10429323	CG12896	558	316.742	35.26
chr2R	10429666	10431065	RanBPM	576	316.742	31.81
chr2R	10432271	10432792	RanBPM	2526	316.742	20.66
chr2R	10437725	10438608	Galphao	36	316.742	30.76
chr2R	10443898	10444508	Galphao	70	109.65	11.93
chr2R	10444908	10445237	Galphao	57	36.7662	6.20
chr2R	10473858	10474120	whd	1475	17.5704	3.69
chr2R	10474745	10475876	whd	13	297.464	18.67
chr2R	10476821	10477190	CR45134	989	37.5628	5.88
chr2R	10477565	10478811	CR45134	102	316.742	42.60
chr2R	10477565	10478811	whd	627	316.742	42.60
chr2R	10530511	10530901	lola	3610	29.0691	5.10
chr2R	10534042	10535526	lola	120	316.742	37.71
chr2R	10536024	10536446	snoRNA:lola-a	455	27.9106	5.02
chr2R	10537757	10538596	snoRNA:lola-d	695	38.9093	5.76
chr2R	10540284	10543655	CR45135	133	316.742	23.20
chr2R	10540284	10543655	lola	57	316.742	23.20
chr2R	10551600	10552263	acal	4718	214.27	20.82
chr2R	10552986	10553301	psq	4757	29.3832	5.69
chr2R	10553801	10554667	psq	3648	316.742	25.41
chr2R	10555073	10555738	psq	2522	316.742	27.39
chr2R	10557593	10559201	psq	927	316.742	20.87
chr2R	10559538	10560090	psq	1947	180.411	14.11
chr2R	10562782	10563052	psq	5021	16.6337	4.03
chr2R	10564116	10564880	psq	4405	87.2156	10.93
chr2R	10565971	10567254	psq	2307	45.8395	7.40
chr2R	10568487	10569817	psq	304	127.376	12.17
chr2R	10583227	10583738	psq	95	14.7609	3.63
chr2R	10617347	10618900	CG11883	910	316.742	41.79
chr2R	10730750	10731102	CG12338	19	51.1752	6.44
chr2R	10733354	10733881	CG7220	1064	7.99721	2.66
chr2R	10735190	10735889	CG7220	88	52.6062	6.81
chr2R	10776921	10777279	CG33144	5766	27.4159	4.87
chr2R	10781295	10783122	CG33144	11	316.742	32.79
chr2R	10781295	10783122	CR44299	36	316.742	32.79
chr2R	10798548	10799005	CG33144	77	74.1146	7.99
chr2R	10806391	10807623	wde	128	230.87	16.59
chr2R	10819785	10820048	dgo	48	6.97395	2.36
chr2R	10819785	10820048	Prosbeta5	45	6.97395	2.36
chr2R	10857772	10858118	CR44302	1708	27.0358	5.43
chr2R	10861188	10862199	CG30015	64	40.4055	5.56
chr2R	10871384	10871781	CG30015	3256	14.1549	3.66
chr2R	10874269	10874952	CG30015	79	60.6996	6.15
chr2R	10918283	10918595	Obp47a	8501	26.4968	5.76
chr2R	11062271	11062858	CG43188	22850	20.1295	4.06
chr2R	11065494	11065996	CG43188	19856	50.3624	6.74
chr2R	11105627	11106975	luna	27	28.0128	4.25
chr2R	11107407	11107824	luna	773	110.006	12.25
chr2R	11115591	11116807	luna	109	103.322	11.76
chr2R	11172152	11172503	CG13229	84	16.9067	3.91
chr2R	11173349	11174108	shn	80	316.742	26.12
chr2R	11175125	11176259	shn	1581	95.116	9.72
chr2R	11178206	11179120	shn	4303	38.639	6.32
chr2R	11181765	11182085	shn	1364	12.4032	3.53
chr2R	11183943	11184443	shn	1070	21.8989	4.64
chr2R	11185515	11186084	shn	2612	153.155	12.23

chr2R	11194252	11194743	shn	3890	22.2864	4.72
chr2R	11196636	11197736	shn	950	316.742	22.88
chr2R	11198081	11199305	shn	667	165.025	13.81
chr2R	11202120	11204138	shn	2210	316.742	23.18
chr2R	11204460	11205769	shn	443	28.8746	4.42
chr2R	11206364	11207057	shn	2317	11.4567	3.17
chr2R	11219565	11220220	CG9084	176	69.7502	7.96
chr2R	11219565	11220220	Syx6	14	69.7502	7.96
chr2R	11222422	11222960	CG7737	225	110.31	11.58
chr2R	11231804	11233162	metro	464	95.2095	12.61
chr2R	11279495	11280038	CG9067	20	20.1481	3.83
chr2R	11279495	11280038	Vhl	21	20.1481	3.83
chr2R	11283649	11284291	CG9062	32	173.475	13.25
chr2R	11283649	11284291	CG13220	343	173.475	13.25
chr2R	11288841	11289795	Fbl6	360	116.587	9.95
chr2R	11297121	11297535	CG42336	252	13.589	3.44
chr2R	11307209	11307650	Fpps	70	27.5095	4.76
chr2R	11364309	11367792	CG13204	803	316.742	28.50
chr2R	11370774	11371254	Tapdelta	214	12.6248	3.06
chr2R	11383619	11384631	Sod3	93	316.742	42.39
chr2R	11388118	11388415	CG30022	471	19.6611	3.88
chr2R	11397174	11398142	sprt	48	316.742	24.33
chr2R	11451793	11452856	E(Pc)	27	33.8783	5.13
chr2R	11469400	11469811	inv	4881	42.2208	6.85
chr2R	11475276	11476011	inv	44	316.742	29.78
chr2R	11527700	11528698	en	17	316.742	31.99
chr2R	11529365	11529992	en	1471	316.742	19.80
chr2R	11534151	11534699	en	6155	33.644	6.08
chr2R	11581921	11582328	tou	521	24.1126	4.29
chr2R	11592292	11594564	tou	66	316.742	25.23
chr2R	11596621	11597092	tou	3739	16.9525	3.98
chr2R	11597462	11598088	tou	4501	25.287	4.49
chr2R	11600695	11601878	tou	3207	24.9208	4.78
chr2R	11603101	11603891	tou	1374	316.742	25.80
chr2R	11604461	11605577	tou	196	316.742	37.47
chr2R	11607153	11607896	tou	2368	15.9713	3.94
chr2R	11613978	11614530	tou	1911	18.0359	4.27
chr2R	11615087	11615362	tou	868	11.3133	3.24
chr2R	11615733	11616592	tou	41	167.14	12.61
chr2R	11615733	11616592	CR43907	108	167.14	12.61
chr2R	11625678	11627029	CG9005	5908	316.742	32.10
chr2R	11628918	11630307	CG9005	2758	266.455	14.22
chr2R	11630728	11631987	CG9005	1388	92.6411	8.78
chr2R	11632261	11633850	CG9005	40	135.185	11.61
chr2R	11634978	11635541	CG9005	2802	215.707	16.04
chr2R	11637081	11637946	CG9005	3263	316.742	25.02
chr2R	11639478	11639769	CG9005	1317	23.9393	3.74
chr2R	11640234	11641071	CG9005	319	16.1019	3.33
chr2R	11648224	11648957	CG9003	93	316.742	28.30
chr2R	11650729	11651801	CG9003	590	169.161	11.92
chr2R	11656002	11656628	CG13198	1409	28.0734	5.05
chr2R	11657366	11658318	CG13198	3127	181.473	15.52
chr2R	11661152	11661683	Tret1-1	4343	16.3667	3.85
chr2R	11662785	11663090	Tret1-1	2831	22.276	4.58
chr2R	11665267	11665939	Tret1-1	118	316.742	24.40
chr2R	11666325	11667092	Tret1-1	1015	316.742	23.14
chr2R	11711971	11712444	pyr	52	48.2621	7.59
chr2R	11756338	11756877	ths	187	78.1386	8.78
chr2R	11757620	11758063	ths	1003	68.4814	8.18
chr2R	11764432	11764879	CG13193	3793	31.5835	5.17
chr2R	11765336	11765694	CG13193	2878	12.7916	3.20
chr2R	11789929	11790251	ths	269	33.8429	5.24
chr2R	11831930	11832299	CG13192	6868	9.54414	3.32
chr2R	11833601	11833912	Sobp	5777	28.5531	5.28
chr2R	11836615	11837085	Sobp	2683	32.7609	4.90
chr2R	11845141	11845768	Sln	39	26.1906	5.50
chr2R	11890259	11891086	ERp60	39	27.492	4.45
chr2R	11891916	11893508	Ef1alpha48D	882	138.451	11.16
chr2R	11891916	11893508	snoRNA:Me28S-A1322	119	138.451	11.16
chr2R	11899428	11899981	Zip48C	82	22.8209	4.56
chr2R	11912719	11913007	reb	52	13.7071	3.72
chr2R	11930260	11930613	CR44312	661	9.7279	2.97
chr2R	12019615	12020085	otk	60	171.997	12.43
chr2R	12025025	12025448	otk2	29	59.2552	7.50
chr2R	12027646	12028250	Mppe	107	9.72121	2.72

chr2R	12027646	12028250	pds5	170	9.72121	2.72
chr2R	12033894	12034633	CG8321	142	27.7337	4.26
chr2R	12044389	12045419	Drep1	21	301.229	18.50
chr2R	12050205	12050471	CG30039	1437	21.5535	5.01
chr2R	12054710	12055190	Drep3	163	71.6257	9.33
chr2R	12118810	12119599	jeb	54	145.74	15.12
chr2R	12120271	12120806	jeb	1160	23.3434	5.36
chr2R	12121162	12121854	jeb	2181	20.2306	4.36
chr2R	12150341	12151085	CG8878	193	17.0425	3.73
chr2R	12168475	12169232	Oda	270	40.69	5.67
chr2R	12168475	12169232	SmD3	28	40.69	5.67
chr2R	12169716	12171793	mir-281-2	992	42.4941	5.76
chr2R	12169716	12171793	mir-281-1	774	42.4941	5.76
chr2R	12169716	12171793	Oda	49	42.4941	5.76
chr2R	12169716	12171793	CR46194	76	42.4941	5.76
chr2R	12172843	12174453	Oda	42	48.7559	5.69
chr2R	12252385	12253223	CG13168	1016	196.101	16.73
chr2R	12259061	12259765	Cam	46	257.382	16.07
chr2R	12259061	12259765	CR43900	57	257.382	16.07
chr2R	12261074	12262060	Cam	1390	316.742	22.48
chr2R	12279303	12280000	CG42700	292	63.9562	7.78
chr2R	12306267	12306592	CG13164	199	38.8508	6.27
chr2R	12306267	12306592	CG17739	59	38.8508	6.27
chr2R	12313923	12314617	CG30046	32	30.6062	6.26
chr2R	12326149	12326510	CG8841	72	8.50399	2.61
chr2R	12326149	12326510	garz	29	8.50399	2.61
chr2R	12337729	12338370	Den1	231	29.5442	4.47
chr2R	12343958	12344700	CG8839	122	48.5722	5.82
chr2R	12401727	12402108	Cpr49Ae	385	18.8588	4.77
chr2R	12437298	12438178	Dyb	204	15.871	3.23
chr2R	12438720	12439161	Dyb	1589	101.556	9.14
chr2R	12452963	12454565	Lac	999	316.742	42.58
chr2R	12461854	12463109	dgt5	6042	109.965	11.19
chr2R	12471718	12472072	dgt5	143	28.6653	4.91
chr2R	12471718	12472072	CG8550	4	28.6653	4.91
chr2R	12473951	12474676	Dh44-R2	156	316.742	26.08
chr2R	12504709	12505324	fdl	298	40.3218	6.23
chr2R	12506952	12507287	fdl	175	13.0667	3.21
chr2R	12528888	12529528	fra	116	40.6785	5.22
chr2R	12534758	12535014	fra	5880	7.43779	2.72
chr2R	12566007	12566329	Cyp301a1	14	27.854	4.89
chr2R	12572096	12572559	ClC-b	198	21.0983	3.94
chr2R	12586489	12590467	Sin3A	1973	316.742	26.39
chr2R	12586489	12590467	CR30055	2059	316.742	26.39
chr2R	12591128	12592933	Sin3A	1124	316.742	21.10
chr2R	12593454	12593901	Amph	71	42.3811	4.98
chr2R	12593454	12593901	CR45279	153	42.3811	4.98
chr2R	12597636	12599054	CR43909	3204	42.9689	6.47
chr2R	12601008	12601346	CR43909	5541	28.165	4.65
chr2R	12602351	12602634	CR43909	6856	15.2586	3.45
chr2R	12612318	12613575	CG45086	26	16.9541	3.29
chr2R	12612318	12613575	Galphaq	26	16.9541	3.29
chr2R	12670039	12670359	nemy	33	17.7519	4.41
chr2R	12679423	12679911	GLS	64	67.4896	9.55
chr2R	12686581	12687496	CG13148	102	80.1568	8.83
chr2R	12704680	12705469	CG42663	91	278.309	18.52
chr2R	12742877	12743486	Mos	9693	238.664	18.47
chr2R	12756065	12756368	Taz	125	8.53222	2.71
chr2R	12780162	12780708	sca	93	130.986	13.90
chr2R	12868555	12868937	CR45140	577	16.1658	3.66
chr2R	12870148	12870467	bic	57	14.7065	3.48
chr2R	12884255	12884784	vg	92	112.359	11.81
chr2R	12904199	12904918	CR44461	1871	222.991	18.70
chr2R	12915996	12917893	NAT1	208	73.5637	7.55
chr2R	12936133	12937056	CG13322	79	53.1436	7.76
chr2R	12936133	12937056	Sans	276	53.1436	7.76
chr2R	12955760	12956465	CG13321	2447	167.938	11.78
chr2R	12958717	12959261	CG13321	5539	28.5935	5.52
chr2R	12962852	12963150	CG13321	9416	14.5516	3.74
chr2R	12979700	12981552	Psc	116	316.742	38.28
chr2R	12984452	12986237	CR44339	509	316.742	21.77
chr2R	12990478	12992380	CG33798	2746	272.176	20.01
chr2R	12995944	12997835	Su(z)2	13	316.742	23.67
chr2R	12998461	12998918	Su(z)2	1580	38.7872	5.88
chr2R	13006058	13006321	Su(z)2	8996	16.3449	3.91

chr2R	13009129	13009709	Su(z)2	12321	96.4672	11.86
chr2R	13014535	13016299	Su(z)2	18638	316.742	50.65
chr2R	13025265	13025598	CG13323	21814	12.2121	3.49
chr2R	13031943	13032568	CG13323	15007	131.839	15.28
chr2R	13034910	13035813	CG13323	11819	316.742	19.13
chr2R	13038573	13039739	CG13323	8346	313.271	18.20
chr2R	13041891	13042662	CG13323	5059	210.893	12.30
chr2R	13079332	13079841	Drl-2	28	51.9646	7.94
chr2R	13157986	13158665	TppII	79	41.4952	6.28
chr2R	13157986	13158665	ND-MWFE	447	41.4952	6.28
chr2R	13167807	13168085	GLaz	45	12.4855	3.26
chr2R	13171116	13171729	AGBE	29	54.3955	6.16
chr2R	13189062	13189589	Kdm4B	71	143.75	15.18
chr2R	13189868	13190475	CG17724	145	87.2378	10.81
chr2R	13189868	13190475	Kdm4B	145	87.2378	10.81
chr2R	13189868	13190475	seq	145	87.2378	10.81
chr2R	13189868	13190475	CG17724	145	87.2378	10.81
chr2R	13189868	13190475	seq	145	87.2378	10.81
chr2R	13191406	13191989	CG17724	1530	88.1637	9.86
chr2R	13191406	13191989	Kdm4B	1530	88.1637	9.86
chr2R	13191406	13191989	seq	1530	88.1637	9.86
chr2R	13191406	13191989	CG17724	1530	88.1637	9.86
chr2R	13191406	13191989	seq	1530	88.1637	9.86
chr2R	13192842	13193141	CG17724	2877	17.814	3.98
chr2R	13192842	13193141	Kdm4B	2877	17.814	3.98
chr2R	13192842	13193141	seq	2877	17.814	3.98
chr2R	13192842	13193141	CG17724	2877	17.814	3.98
chr2R	13192842	13193141	seq	2877	17.814	3.98
chr2R	13218996	13219624	CG4630	77	269.519	18.84
chr2R	13222326	13222974	Fsn	89	10.6058	2.86
chr2R	13222326	13222974	Dp	5	10.6058	2.86
chr2R	13222326	13222974	Fsn	32	10.6058	2.86
chr2R	13223236	13224413	Dp	179	50.3879	5.91
chr2R	13337270	13337626	CR44206	102	34.9991	5.86
chr2R	13344637	13345245	CR44206	118	87.4288	11.11
chr2R	13362669	13363268	CR45312	119	65.4676	9.89
chr2R	13424599	13425265	CG42321	271	163.543	14.03
chr2R	13428564	13430227	CG42321	28	308.235	17.43
chr2R	13449146	13450772	cnn	478	316.742	25.95
chr2R	13449146	13450772	cbs	588	316.742	25.95
chr2R	13469450	13469718	CG43192	7786	10.6469	3.38
chr2R	13482604	13483394	arr	63	59.5359	6.44
chr2R	13494464	13494935	bbc	197	23.665	4.37
chr2R	13501734	13502978	mars	3	316.742	27.75
chr2R	13501734	13502978	drk	215	316.742	27.75
chr2R	13501734	13502978	mars	850	316.742	27.75
chr2R	13506983	13507511	EfTuM	463	10.805	3.22
chr2R	13539506	13540200	CG6145	177	316.742	22.67
chr2R	13545210	13545699	CG6145	9	28.8448	4.56
chr2R	13554016	13554529	Roe1	219	15.163	3.82
chr2R	13574059	13575184	CG42808	74	57.1433	7.48
chr2R	13574059	13575184	CR44365	581	57.1433	7.48
chr2R	13576194	13576960	CR44366	1401	58.1723	8.34
chr2R	13577522	13578276	CR44366	275	14.7001	3.43
chr2R	13587381	13587672	CG6191	5850	16.1048	3.52
chr2R	13587381	13587672	CG45088	5850	16.1048	3.52
chr2R	13589584	13589887	CG6191	3590	10.7	2.98
chr2R	13589584	13589887	CG45088	3590	10.7	2.98
chr2R	13591841	13593448	CG6191	242	225.903	15.11
chr2R	13591841	13593448	CG45088	242	225.903	15.11
chr2R	13620144	13620594	fas	2649	33.3586	5.16
chr2R	13622658	13623259	fas	128	229.006	22.15
chr2R	13805825	13806240	stj	110	22.0038	4.59
chr2R	13822190	13823126	fl(2)d	1681	294.14	18.04
chr2R	13823915	13824666	fl(2)d	247	230.83	14.59
chr2R	13826137	13827092	CG6329	175	141.825	12.91
chr2R	13829034	13829606	CG6329	101	104.483	10.61
chr2R	13839720	13840337	CG6357	45	316.742	20.62
chr2R	13845736	13846319	Syngr	374	10.0569	3.04
chr2R	13853958	13854443	CG6543	35	19.726	3.47
chr2R	13855491	13855921	CG6553	47	38.2278	5.78
chr2R	13896269	13897032	shot	1207	46.2171	6.73
chr2R	13897399	13898133	shot	45	316.742	35.08
chr2R	13899000	13899376	shot	1478	17.4684	3.92
chr2R	13912725	13913203	shot	2080	19.163	4.09

chr2R	13918650	13920036	shot	6617	81.9046	7.85
chr2R	13923921	13924472	shot	1332	235.044	16.83
chr2R	13925223	13926263	shot	92	316.742	54.23
chr2R	13934294	13935116	shot	7281	119.008	10.98
chr2R	13937340	13937792	shot	4621	10.6309	3.04
chr2R	13939697	13940075	shot	2214	66.497	7.62
chr2R	13940933	13942376	shot	894	316.742	21.80
chr2R	13953538	13954292	AGO1	1	149.543	11.84
chr2R	13953538	13954292	mRpL53	168	149.543	11.84
chr2R	13953538	13954292	CG33155	168	149.543	11.84
chr2R	13956798	13958456	AGO1	88	316.742	24.28
chr2R	13960499	13961388	Cp1	186	32.9772	4.47
chr2R	13979430	13979904	CG6701	8	41.2305	5.75
chr2R	13990229	13990785	RN-tre	176	90.3789	8.27
chr2R	13991063	13992642	mam	686	316.742	20.21
chr2R	13995501	13996212	mam	4717	76.9015	8.94
chr2R	14000683	14001241	mir-4978	5012	109.096	11.37
chr2R	14005596	14005863	mir-4978	334	8.6116	2.80
chr2R	14006599	14007490	mir-4978	845	316.742	33.82
chr2R	14008031	14009504	mam	770	316.742	30.76
chr2R	14011632	14011947	mam	2586	14.48	3.73
chr2R	14023897	14025124	CG18371	3843	316.742	35.63
chr2R	14026867	14027184	CG18371	1603	29.5898	5.07
chr2R	14030133	14030808	mam	1089	32.1474	5.69
chr2R	14031155	14031892	mam	114	63.4115	8.52
chr2R	14041022	14041684	mam	9629	85.955	10.22
chr2R	14113878	14114359	CG42288	23219	15.2794	3.54
chr2R	14139831	14141665	Prosap	41	238.436	15.79
chr2R	14164452	14164769	Tango7	11	25.1636	4.42
chr2R	14170368	14170623	CG8331	28	13.6102	3.31
chr2R	14170981	14171469	CG8331	62	16.0288	3.79
chr2R	14173579	14175908	mRpS16	1214	97.8409	9.18
chr2R	14173579	14175908	cg	1118	97.8409	9.18
chr2R	14184922	14185715	CG30069	142	188.679	14.53
chr2R	14185997	14186952	CG30069	1421	273.258	18.65
chr2R	14212126	14213195	Sox15	48	284.067	18.60
chr2R	14216329	14216771	RpS23	0	17.8234	3.65
chr2R	14255443	14256529	Hsc70-5	54	73.7474	8.69
chr2R	14255443	14256529	CG8531	485	73.7474	8.69
chr2R	14265371	14266125	CG8547	1666	316.742	27.32
chr2R	14267570	14268295	CG8547	102	316.742	19.20
chr2R	14270078	14270982	SelD	332	50.5884	6.56
chr2R	14270078	14270982	conv	402	50.5884	6.56
chr2R	14275135	14275609	Ih	719	68.1794	7.80
chr2R	14275894	14276229	Ih	46	13.8687	3.45
chr2R	14276663	14276989	Ih	729	13.8951	3.55
chr2R	14288968	14289774	Ih	9	21.2502	4.06
chr2R	14306863	14308507	Shroom	803	316.742	39.47
chr2R	14309721	14310003	Shroom	1722	14.4803	3.62
chr2R	14332041	14332622	Shroom	101	117.942	10.92
chr2R	14359973	14360434	Arc1	23	316.742	24.93
chr2R	14361707	14362626	Arc2	21	308.157	18.00
chr2R	14363003	14363294	Tfb1	63	12.1935	3.33
chr2R	14432079	14432469	phyl	28	37.2	6.76
chr2R	14435602	14435947	Oaz	91	32.9389	5.77
chr2R	14437425	14437763	Oaz	1675	23.4925	5.31
chr2R	14448937	14449317	Oaz	9394	47.5554	8.11
chr2R	14456150	14456450	Oaz	2193	21.5869	4.51
chr2R	14457821	14458633	Oaz	328	217.445	19.08
chr2R	14481135	14482481	PRAS40	390	38.1658	5.18
chr2R	14503766	14504541	Asx	64	45.4571	5.62
chr2R	14513863	14514368	CG30197	31	57.7597	7.68
chr2R	14526133	14527290	ttv	456	182.703	13.71
chr2R	14574294	14576133	LamC	81	156.045	11.24
chr2R	14576626	14577323	LamC	1204	316.742	21.73
chr2R	14618629	14618958	Sec61beta	50	17.7712	3.52
chr2R	14621527	14622142	Uhg5	377	25.4402	4.41
chr2R	14621527	14622142	snoRNA:Me18S-C136	196	25.4402	4.41
chr2R	14621527	14622142	noRNA:Me28S-C3227	110	25.4402	4.41
chr2R	14624534	14624859	snoRNA:Or-CD4	469	33.5636	4.87
chr2R	14713364	14713931	mspo	62	154.26	14.09
chr2R	14715728	14716171	mspo	2324	165.302	18.28
chr2R	14745189	14745611	CG12858	90	35.6367	5.61
chr2R	14746702	14747165	AttA	502	61.6968	8.61
chr2R	14755711	14756273	CG10151	15	20.3396	3.99

chr2R	14765471	14766077	Spred	3744	30.3755	5.02
chr2R	14768342	14769312	Spred	627	316.742	23.30
chr2R	14790423	14791080	kn	6296	115.271	11.41
chr2R	14799002	14799855	kn	719	67.8565	7.52
chr2R	14829173	14829489	CG10205	3509	24.9044	5.56
chr2R	14848234	14848519	CG10209	6	22.2765	4.79
chr2R	14870543	14870927	Pcf11	401	16.226	3.19
chr2R	14871600	14871956	Cyp6a22	101	26.7911	4.53
chr2R	14873683	14874204	Cyp6a17	14	285.949	18.51
chr2R	14875638	14876046	Cyp6a23	1	90.0218	9.57
chr2R	14881078	14881627	Cyp6a9	13	154.601	14.27
chr2R	14881913	14882350	Cyp6a20	4	84.9398	9.42
chr2R	14886705	14887093	Cyp6a21	55	33.5987	5.52
chr2R	14898895	14899622	Cyp317a1	2	268.583	20.28
chr2R	14907946	14908522	Kank	334	79.0434	8.92
chr2R	14917156	14917894	Kank	10	70.4573	9.44
chr2R	14921136	14921539	Kank	3974	42.6616	5.50
chr2R	14927978	14928613	Kank	165	176.216	14.59
chr2R	14932792	14933539	CG10253	6	49.343	7.13
chr2R	14932792	14933539	CG12853	204	49.343	7.13
chr2R	14933838	14934205	CG12853	482	34.5396	5.93
chr2R	14935411	14936089	Lap1	178	72.1138	6.82
chr2R	14954295	14954935	aPKC	3692	109.078	12.33
chr2R	14958249	14959408	aPKC	712	91.1141	9.38
chr2R	14962167	14963475	aPKC	440	185.836	14.66
chr2R	14962167	14963475	ckn	762	185.836	14.66
chr2R	14964006	14965922	ckn	989	316.742	24.30
chr2R	14970001	14970471	ckn	4554	95.5762	10.18
chr2R	14973154	14973876	ckn	2562	45.5752	6.18
chr2R	14981716	14982062	pcs	663	30.0418	4.80
chr2R	15010525	15011507	hbs	545	166.127	13.75
chr2R	15109416	15109772	chn	5734	7.05692	2.40
chr2R	15114976	15116647	chn	931	95.6614	11.19
chr2R	15127893	15129084	chn	453	167.293	13.82
chr2R	15135036	15135934	chn	6505	316.742	26.92
chr2R	15155117	15155600	igl	96	70.5594	9.58
chr2R	15231778	15232133	CR44370	5035	10.3226	3.48
chr2R	15241374	15242115	Fs	13	30.3392	5.57
chr2R	15248275	15249422	scb	304	143.298	14.80
chr2R	15252265	15252915	scb	90	316.742	45.59
chr2R	15290557	15291905	Trpm	16	144.172	12.42
chr2R	15307942	15309565	Trpm	32	206.375	14.47
chr2R	15322389	15323260	Arf51F	53	177.691	12.97
chr2R	15329130	15329771	CG8160	1185	49.7513	7.39
chr2R	15330608	15331237	CR44371	411	51.547	8.30
chr2R	15349360	15349905	unc-5	487	209.376	17.70
chr2R	15380745	15381108	dup	0	23.0776	4.55
chr2R	15400817	15401295	CG43729	101	69.5108	9.85
chr2R	15424611	15425443	CG43729	122	250.279	21.13
chr2R	15474369	15474905	CG8180	8289	13.3157	3.30
chr2R	15482372	15483105	CG8180	20	269.927	17.63
chr2R	15511541	15511984	GalNAc-T1	30	25.1116	4.01
chr2R	15531377	15532301	Khc-73	660	27.779	4.75
chr2R	15541312	15541616	CG8187	83	16.4803	3.72
chr2R	15541312	15541616	Vha36-1	12	16.4803	3.72
chr2R	15569118	15569649	Flo1	34	63.9863	6.85
chr2R	15600018	15600805	CG42524	13362	316.742	24.65
chr2R	15609884	15610346	CG42524	3701	67.6925	9.59
chr2R	15610752	15611591	CG42524	2794	277.84	24.90
chr2R	15613133	15615237	CG42524	102	316.742	40.28
chr2R	15634487	15634950	CG42524	20884	67.4896	9.55
chr2R	15648779	15649166	mir-278	8157	16.68	4.23
chr2R	15657725	15658163	mir-278	866	33.2774	5.92
chr2R	15660742	15661050	mir-278	3795	18.7317	4.29
chr2R	15668740	15669082	fus	3826	12.6549	3.54
chr2R	15674828	15676926	fus	167	316.742	31.50
chr2R	15674828	15676926	CR45143	320	316.742	31.50
chr2R	15674828	15676926	fus	549	316.742	31.50
chr2R	15699084	15699364	CG42662	1	8.36912	2.95
chr2R	15727123	15727519	CG30089	87	40.2833	5.86
chr2R	15734785	15735816	CG30089	113	316.742	32.40
chr2R	15754130	15754774	CG30089	19136	13.91	3.21
chr2R	15816693	15817423	Zasp52	88	316.742	24.34
chr2R	15835061	15835393	Poxn	171	15.9714	4.35
chr2R	15843354	15843878	CG8249	3789	119.931	10.84

chr2R	15847163	15847553	CG8249	41	31.1159	5.81
chr2R	15847992	15849002	tun	43	27.4159	4.87
chr2R	15851266	15851641	tun	2884	22.0066	4.41
chr2R	15861108	15862204	Gpo-1	13	316.742	18.54
chr2R	15862711	15863213	Gpo-1	88	12.99	3.51
chr2R	15930995	15932074	bdg	481	28.0734	5.05
chr2R	15951826	15952482	Strn-Mlck	35	199.925	18.25
chr2R	16000103	16000514	ATPCL	13	14.9793	3.36
chr2R	16007484	16007794	CG8386	1	15.2663	3.49
chr2R	16017551	16018387	CG8399	1	80.6848	8.41
chr2R	16030918	16032186	SP2353	629	137.394	13.99
chr2R	16047464	16047903	CG8405	37	33.8889	5.37
chr2R	16070815	16071324	mir-137	5804	17.7362	4.56
chr2R	16097963	16098717	Dg	244	249.603	15.62
chr2R	16102484	16105085	CG8414	1879	316.742	34.69
chr2R	16102484	16105085	Rho1	1022	316.742	34.69
chr2R	16109792	16110044	Ric	46	8.84149	2.98
chr2R	16124845	16125739	spin	291	193.78	13.54
chr2R	16126179	16126762	spin	1710	9.26653	2.67
chr2R	16128990	16130681	spin	5289	316.742	39.84
chr2R	16135488	16135782	Got1	2679	14.6183	3.07
chr2R	16138707	16139243	Got1	299	71.4231	8.31
chr2R	16149980	16151246	lbk	879	123.05	10.92
chr2R	16159945	16160982	CG10734	1616	11.8463	3.51
chr2R	16178762	16180664	Lis-1	793	153.581	12.67
chr2R	16187058	16188220	clu	85	294.166	19.61
chr2R	16196927	16197324	CG42837	97	30.6421	4.73
chr2R	16196927	16197324	CG44243	97	30.6421	4.73
chr2R	16209892	16210827	mrj	110	316.742	23.79
chr2R	16220778	16221880	CR43898	333	189.701	14.05
chr2R	16220778	16221880	mrj	491	189.701	14.05
chr2R	16226028	16226752	CG15706	259	129.221	10.97
chr2R	16226028	16226752	Tsf3	11	129.221	10.97
chr2R	16259670	16259995	CngA	22	19.5844	4.87
chr2R	16270254	16271027	Khc	1417	225.137	14.82
chr2R	16289091	16289954	JhI-26	36	316.742	18.90
chr2R	16335696	16337321	Hmgs	89	214.477	15.98
chr2R	16335696	16337321	Vha44	637	214.477	15.98
chr2R	16335696	16337321	Hmgs	759	214.477	15.98
chr2R	16386261	16387003	Sema-2b	33	38.7872	5.88
chr2R	16398634	16399106	Sema-2b	45	56.433	7.80
chr2R	16499718	16500347	Sema-2a	158	142.656	14.27
chr2R	16517026	16517339	Sema-2a	734	24.2433	5.23
chr2R	16551935	16552493	Syn2	63	97.1091	8.57
chr2R	16579309	16581144	Cdk4	29	47.2493	5.70
chr2R	16583155	16584347	CR44375	558	316.742	20.23
chr2R	16585536	16586064	RpLP2	373	144.173	13.28
chr2R	16588953	16589279	CG8311	30	13.3419	3.22
chr2R	16593262	16593554	CG8306	1251	18.0307	3.92
chr2R	16594022	16594993	CG8306	276	159.783	13.25
chr2R	16604008	16604616	CG8303	62	39.4994	6.16
chr2R	16607634	16609640	CG5065	72	124.771	12.53
chr2R	16637893	16638566	Alk	278	45.3252	6.42
chr2R	16641004	16641354	gprs	67	10.6309	3.04
chr2R	16658984	16659424	Picot	16	37.2017	5.75
chr2R	16667543	16668171	Picot	339	29.3301	5.68
chr2R	16689417	16689954	CG5210	1359	49.4883	6.83
chr2R	16691454	16691934	CG5210	32	59.5922	6.82
chr2R	16731106	16731724	CG30463	57	70.2912	7.81
chr2R	16764302	16764794	CG42392	1447	60.6546	7.83
chr2R	16764302	16764794	unc-104	1447	60.6546	7.83
chr2R	16765830	16766137	CG42392	2	15.0084	3.97
chr2R	16765830	16766137	unc-104	2	15.0084	3.97
chr2R	16772437	16772721	unc-104	23	11.1296	2.77
chr2R	16818932	16819594	CR43417	76	42.6855	5.61
chr2R	16828712	16829165	CR43650	131	52.4296	6.43
chr2R	16830423	16830995	CR43650	41	57.1021	6.93
chr2R	16837028	16837474	mir-8	5672	8.05875	2.64
chr2R	16856072	16857493	Dek	48	316.742	22.71
chr2R	16856072	16857493	snoRNA:Dek-a	819	316.742	22.71
chr2R	16869157	16869674	CG6426	15	153.425	10.00
chr2R	16872079	16872485	CG6421	5	21.2502	4.06
chr2R	16873698	16874019	CG6435	131	23.5543	4.58
chr2R	16895242	16896036	mir-990	370	49.1016	7.66
chr2R	16895242	16896036	CG8910	26	49.1016	7.66

chr2R	16896685	16897079	CG8910	1168	23.942	4.37
chr2R	16924486	16925077	Pkc53E	131	93.0923	9.59
chr2R	16960892	16961212	CG9010	859	28.5973	5.69
chr2R	16973697	16974063	Cbp53E	23	20.5066	4.35
chr2R	17020187	17020761	Dark	380	62.5097	7.97
chr2R	17026589	17028694	CR44390	1415	316.742	25.90
chr2R	17026589	17028694	RhoGEF2	1219	316.742	25.90
chr2R	17048898	17049296	CG9646	10	21.8867	4.12
chr2R	17084778	17085360	CG30460	2499	22.0912	4.06
chr2R	17087397	17087845	CG30460	33	73.2108	9.41
chr2R	17091586	17092010	Sply	133	12.5232	3.31
chr2R	17091586	17092010	CG6984	158	12.5232	3.31
chr2R	17097084	17098810	GstS1	23	316.742	33.31
chr2R	17099847	17100554	CG30456	68	316.742	27.99
chr2R	17101810	17102082	CG30456	997	19.0419	4.30
chr2R	17102590	17103262	CG30456	74	55.2928	7.98
chr2R	17108484	17109082	CG15611	5	152.205	14.12
chr2R	17133786	17134384	CG11400	66	316.742	21.55
chr2R	17136139	17136734	Gbp	52	316.742	21.01
chr2R	17139636	17140191	CG43103	17	166.212	12.67
chr2R	17140617	17140917	CG43107	104	25.3684	4.87
chr2R	17215599	17216476	mbl	705	219.286	18.95
chr2R	17261644	17262202	CR45997	455	108.538	9.45
chr2R	17264899	17265782	mbl	65	316.742	19.86
chr2R	17392202	17392806	CG10939	2028	30.26	4.40
chr2R	17394369	17395027	CG10939	91	152.785	13.28
chr2R	17401120	17401634	CG6568	5715	55.6727	7.23
chr2R	17406832	17407423	CG6568	221	15.6041	3.65
chr2R	17420091	17422461	l(2)k01209	454	221.247	13.29
chr2R	17427446	17428043	CG6550	11	11.3602	3.29
chr2R	17427446	17428043	Mtap	294	11.3602	3.29
chr2R	17435160	17437258	CG18467	3804	105.51	9.36
chr2R	17438992	17439734	CG18467	782	32.2511	5.71
chr2R	17440130	17440679	CG18467	92	30.0675	5.16
chr2R	17444462	17445134	EDTP	150	39.1347	5.32
chr2R	17457590	17458973	CG14478	91	39.2552	5.82
chr2R	17462186	17462581	CG14478	1766	67.1263	9.01
chr2R	17469545	17470202	qkr54B	71	13.6168	3.23
chr2R	17484592	17485145	NT5E-2	196	111.46	13.36
chr2R	17535003	17535941	Patronin	69	134.568	11.25
chr2R	17536280	17536588	eIF3-S9	59	15.3705	3.15
chr2R	17547626	17548690	MESR4	83	316.742	27.11
chr2R	17592807	17593742	Smurf	384	165.363	13.97
chr2R	17623066	17623347	CG44403	5357	12.8238	3.63
chr2R	17639028	17639368	CG10936	71	34.1305	5.62
chr2R	17665505	17665888	eIF3-S8	2	10.8708	2.75
chr2R	17682959	17684053	Sema-1b	322	65.9017	7.46
chr2R	17682959	17684053	HPS4	248	65.9017	7.46
chr2R	17686380	17686923	HPS4	41	183.957	12.78
chr2R	17708874	17709456	CG6424	57	173.694	13.89
chr2R	17716169	17718021	CG6424	261	316.742	27.60
chr2R	17719046	17719851	CG6424	54	153.901	12.71
chr2R	17721411	17722104	CG10934	706	42.0397	6.55
chr2R	17726632	17726919	Snx16	146	8.31446	2.66
chr2R	17726632	17726919	CG4984	212	8.31446	2.66
chr2R	17726632	17726919	CG4975	212	8.31446	2.66
chr2R	17726632	17726919	CG4984	212	8.31446	2.66
chr2R	17739626	17740046	CG6401	286	9.84442	2.88
chr2R	17739626	17740046	CG4996	71	9.84442	2.88
chr2R	17750049	17750705	CG5002	85	273.179	17.31
chr2R	17760506	17761570	CG5009	201	157.986	12.41
chr2R	17766694	17767018	CR44416	15	8.71584	2.75
chr2R	17789010	17789502	Ubc10	210	21.1336	4.19
chr2R	17792405	17793107	Dhit	132	204.802	13.81
chr2R	17793828	17794594	Dhit	1387	116.793	12.73
chr2R	17795919	17796582	Dhit	3339	149.399	15.37
chr2R	17800759	17803186	CR45270	56	316.742	22.28
chr2R	17800759	17803186	grh	14	316.742	22.28
chr2R	17800759	17803186	CR45270	352	316.742	22.28
chr2R	17800759	17803186	grh	1128	316.742	22.28
chr2R	17803950	17804809	grh	1420	307.564	19.64
chr2R	17807158	17807747	grh	4720	9.56063	3.06
chr2R	17813317	17814371	grh	270	162.315	12.81
chr2R	17814801	17815145	grh	600	38.6821	6.08
chr2R	17820869	17821151	grh	2551	20.2185	4.48

chr2R	17821993	17822858	grh	1034	60.4654	7.46
chr2R	17823223	17824205	grh	66	53.9056	7.10
chr2R	17832162	17832836	grh	8828	47.8726	6.20
chr2R	17843283	17843549	olf186-F	7	11.6404	3.41
chr2R	17845853	17847502	olf186-M	91	316.742	23.51
chr2R	17850805	17851841	olf186-F	720	316.742	25.54
chr2R	17857175	17857542	olf186-F	2116	40.5659	7.09
chr2R	17869022	17869741	CR44417	332	123.89	9.53
chr2R	17870089	17872136	Mapmodulin	1200	35.258	4.96
chr2R	17873625	17874053	Elk	83	23.0009	4.31
chr2R	18007697	18008179	dpr13	90	47.6938	8.14
chr2R	18008479	18008862	dpr13	778	85.0551	11.72
chr2R	18125655	18125958	stau	41	26.2595	4.66
chr2R	18128328	18128869	Dlip3	45	43.0266	6.40
chr2R	18133550	18134705	Hsf	878	67.583	7.87
chr2R	18135449	18135855	Pcl	235	41.982	6.37
chr2R	18140592	18142341	pAbp	99	110.283	10.35
chr2R	18151201	18152037	CG5742	393	23.8345	4.30
chr2R	18151201	18152037	adp	50	23.8345	4.30
chr2R	18156162	18158331	lolal	79	316.742	21.97
chr2R	18156162	18158331	CG10914	1285	316.742	21.97
chr2R	18179751	18180084	CG5726	162	7.77854	2.76
chr2R	18183897	18184291	CG5721	41	25.9006	4.84
chr2R	18229747	18230042	CR45311	1336	21.9962	4.87
chr2R	18231010	18231526	CR45311	48	143.239	11.71
chr2R	18232393	18233007	fj	93	309.138	24.73
chr2R	18288680	18288977	CG14502	120	30.0639	5.67
chr2R	18288680	18288977	sbb	54	30.0639	5.67
chr2R	18289910	18290372	CG14502	809	10.4857	3.23
chr2R	18309447	18310037	Tango8	2007	132.695	13.68
chr2R	18317647	18318222	Tango8	6152	13.8951	3.55
chr2R	18331823	18332297	Tango8	20240	50.7172	7.49
chr2R	18334811	18335658	sbb	22179	67.8253	8.72
chr2R	18342676	18343435	sbb	14405	37.7772	6.50
chr2R	18351537	18352187	sbb	5457	35.5209	5.46
chr2R	18352821	18354256	sbb	3259	37.9558	5.10
chr2R	18354676	18358109	sbb	361	316.742	36.15
chr2R	18374313	18375335	CR42736	2397	17.4341	4.57
chr2R	18381757	18382069	CG43202	65	18.1418	4.67
chr2R	18384427	18384682	CG18107	3	14.7	4.17
chr2R	18387696	18388299	IM3	56	68.8714	8.71
chr2R	18398279	18398673	GstE1	78	71.1315	8.53
chr2R	18398279	18398673	GstE10	49	71.1315	8.53
chr2R	18400413	18401093	GstE3	33	316.742	21.49
chr2R	18401703	18402290	GstE4	1259	291.003	17.46
chr2R	18404448	18405462	GstE5	38	75.075	8.73
chr2R	18405886	18407240	GstE6	897	125.937	10.61
chr2R	18405886	18407240	GstE7	16	125.937	10.61
chr2R	18407534	18408018	GstE8	16	7.93136	2.79
chr2R	18409030	18409328	GstE9	9	9.18791	2.81
chr2R	18411837	18413818	Dp1	640	316.742	26.98
chr2R	18411837	18413818	imd	44	316.742	26.98
chr2R	18433552	18434290	CG30118	6457	13.3268	3.26
chr2R	18435290	18435606	CG30118	5047	20.34	4.32
chr2R	18439779	18440579	CG30118	63	30.5855	5.12
chr2R	18440848	18441417	slim	192	44.3158	6.18
chr2R	18442392	18443301	slim	1721	227.143	16.41
chr2R	18497733	18498370	CG30116	59	308.281	20.46
chr2R	18503316	18504560	GstE11	34	277.896	16.78
chr2R	18514251	18515201	CG43066	379	53.4733	6.46
chr2R	18517968	18518469	CG43066	3670	102.066	11.90
chr2R	18528337	18529250	CG10924	1520	223.126	14.05
chr2R	18536245	18536998	CG45087	9	70.6231	9.25
chr2R	18536245	18536998	Pepck	9	70.6231	9.25
chr2R	18583208	18583550	GEFmeso	68	27.4601	5.36
chr2R	18610982	18612175	GEFmeso	70	316.742	29.19
chr2R	18621465	18621738	CG5335	16	13.2204	3.41
chr2R	18638466	18639399	CG30122	683	14.168	3.26
chr2R	18652533	18653297	SP2637	189	316.742	40.79
chr2R	18672936	18673827	edl	10	316.742	33.54
chr2R	18688019	18689303	Sik3	403	78.2167	8.70
chr2R	18689758	18690299	Sik3	1390	140.018	13.08
chr2R	18690670	18691021	Sik3	2117	10.2439	3.28
chr2R	18698008	18698580	CG15071	1845	19.5823	4.48
chr2R	18717194	18718358	Hs3st-A	66	176.518	16.14

chr2R	18754813	18755543	Cyp12b2	45	197.827	13.95
chr2R	18760606	18762046	Jabba	71	126.887	13.55
chr2R	18760606	18762046	Mctp	604	126.887	13.55
chr2R	18766793	18767105	Mctp	152	12.4721	3.34
chr2R	18770650	18771706	Mctp	537	64.0229	8.96
chr2R	18776721	18777629	CG15093	36	61.9837	8.77
chr2R	18799183	18799832	MFS15	2763	133.947	13.77
chr2R	18800275	18800959	MFS14	1860	316.742	27.26
chr2R	18801408	18803246	MFS14	363	316.742	31.10
chr2R	18805847	18806798	CR44468	704	316.742	27.21
chr2R	18814917	18815230	CR43418	129	22.3489	5.12
chr2R	18814917	18815230	CG15096	47	22.3489	5.12
chr2R	18829351	18829915	CG15097	35	110.572	9.48
chr2R	18834616	18835662	CG15098	526	47.3942	5.95
chr2R	18834616	18835662	CG15083	39	47.3942	5.95
chr2R	18846030	18846708	CG15099	281	273.449	16.74
chr2R	18854902	18855637	Jheh1	118	30.101	4.75
chr2R	18857751	18858163	Jheh2	29	55.7713	6.47
chr2R	18877791	18878629	sano	345	114.707	10.63
chr2R	18947126	18947530	sano	18	30.2186	4.67
chr2R	18972972	18973497	Topors	292	137.449	11.03
chr2R	19066899	19067299	5-HT1A	81	30.5289	5.95
chr2R	19125723	19126460	CG15117	314	165.334	12.12
chr2R	19136793	19138039	CG15118	472	33.9974	5.15
chr2R	19136793	19138039	CG15111	270	33.9974	5.15
chr2R	19141480	19142514	ena	472	104.813	9.83
chr2R	19142943	19143339	CR43399	1405	42.0459	6.81
chr2R	19154010	19155533	ena	37	316.742	27.51
chr2R	19157417	19158073	ena	2354	41.1468	6.82
chr2R	19163987	19164709	CG10737	42	35.8511	5.33
chr2R	19166604	19167325	CG10737	2605	31.2708	5.52
chr2R	19168961	19169279	CG10737	1112	19.929	4.12
chr2R	19178532	19179450	hppy	464	183.216	12.36
chr2R	19191781	19192351	hppy	13318	26.1416	5.58
chr2R	19226166	19226437	CG7137	1042	23.4423	3.85
chr2R	19228754	19230220	cora	36	316.742	23.32
chr2R	19230931	19231954	cora	2373	316.742	22.46
chr2R	19249468	19249758	wbl	161	17.3726	4.33
chr2R	19253919	19254503	CG33453	1001	123.748	10.09
chr2R	19268688	19269318	rib	1043	105.355	9.73
chr2R	19275290	19275883	rib	167	227.566	16.47
chr2R	19290001	19290544	FK506-bp2	183	36.0308	5.90
chr2R	19292582	19294074	Tab2	690	284.591	18.11
chr2R	19325335	19325791	Rgk1	50	65.6671	9.93
chr2R	19356134	19358555	Rgk1	79	107.64	10.91
chr2R	19356134	19358555	CG11961	927	107.64	10.91
chr2R	19394914	19395521	SdhA	813	232.981	17.12
chr2R	19411246	19411650	hts	42	67.4938	8.97
chr2R	19418247	19419237	snoRNA:hts-a	4479	121.565	11.57
chr2R	19423518	19425347	hts	106	244.642	16.80
chr2R	19423518	19425347	CalpA	892	244.642	16.80
chr2R	19436643	19437199	Fak	208	26.2698	4.40
chr2R	19442207	19442547	RpL11	13	19.2912	3.77
chr2R	19446007	19446356	EloC	67	14.743	3.58
chr2R	19449394	19451779	betaTub56D	201	316.742	22.52
chr2R	19455596	19457469	par-1	948	316.742	21.41
chr2R	19455596	19457469	mei-W68	948	316.742	21.41
chr2R	19455596	19457469	par-1	948	316.742	21.41
chr2R	19461381	19463947	par-1	607	282.664	17.87
chr2R	19466981	19469139	par-1	808	316.742	35.71
chr2R	19617752	19618201	sm	12296	11.3812	3.20
chr2R	19618632	19618996	sm	11565	22.5484	4.57
chr2R	19626573	19627107	sm	3613	51.0646	7.75
chr2R	19630403	19632629	sm	481	316.742	34.07
chr2R	19668909	19669938	hrg	88	27.8904	4.51
chr2R	19691873	19692357	CR44506	24	27.8127	5.07
chr2R	19825492	19826892	Toll-7	102	316.742	34.68
chr2R	20007025	20007571	CR44628	254	90.3966	8.94
chr2R	20106201	20106959	18w	4953	316.742	44.62
chr2R	20110596	20111758	18w	265	316.742	49.80
chr2R	20119609	20119884	18w	8210	12.4007	3.38
chr2R	20245262	20245591	CG11208	13	20.7834	4.35
chr2R	20257051	20257429	Ate1	82	21.7866	4.25
chr2R	20263885	20264211	RpS18	16	14.4217	3.42
chr2R	20274252	20274639	CG10444	41	51.9797	6.01

chr2R	20274252	20274639	CG11788	138	51.9797	6.01
chr2R	20307238	20307533	CG11200	1025	9.31732	2.92
chr2R	20313301	20313923	CG13868	21	213.138	14.66
chr2R	20319565	20320866	CG8920	313	316.742	36.77
chr2R	20327198	20328042	CG8929	374	316.742	19.26
chr2R	20423733	20424507	CG12484	123	65.9727	8.49
chr2R	20503382	20503652	Obp57c	33	11.3227	3.38
chr2R	20531218	20531939	CR44651	198	280.319	18.79
chr2R	20533065	20533474	CG30154	100	46.2171	6.73
chr2R	20534104	20534694	CG18067	107	164.151	13.26
chr2R	20581507	20583171	qsm	623	316.742	25.58
chr2R	20587541	20588794	HnRNP-K	527	316.742	22.36
chr2R	20595959	20596425	HnRNP-K	2213	20.986	4.96
chr2R	20613163	20613509	Hil	4864	23.5235	4.98
chr2R	20617719	20618599	Hil	11	106.651	9.21
chr2R	20617719	20618599	CG9945	296	106.651	9.21
chr2R	20646165	20646501	Lrt	26	27.2721	5.25
chr2R	20654807	20655415	CG16799	17	140.27	13.85
chr2R	20665532	20665941	Fem-1	142	43.0566	6.18
chr2R	20666408	20667409	Fem-1	616	97.2081	8.51
chr2R	20671227	20671557	CG30152	143	11.0655	2.74
chr2R	20697810	20698651	dpr1	73	50.4129	8.40
chr2R	20808949	20809207	CG15225	16126	17.8465	4.59
chr2R	20830386	20830746	sktl	1902	17.5204	3.60
chr2R	20831661	20832541	sktl	507	126.593	10.62
chr2R	20835773	20836695	sktl	56	316.742	21.35
chr2R	20835773	20836695	insc	56	316.742	21.35
chr2R	20869141	20869502	IM4	38	21.8908	4.33
chr2R	20870201	20870741	IM14	55	11.3812	3.20
chr2R	20884435	20884826	Cib2	4727	45.5018	5.73
chr2R	20903363	20903942	otp	245	225.331	17.97
chr2R	20913850	20914607	Rx	2485	133.27	13.39
chr2R	20916164	20916846	Rx	75	30.8834	5.68
chr2R	20935172	20935452	Act57B	8644	9.14346	3.15
chr2R	20938091	20938525	Act57B	5798	35.2316	5.82
chr2R	20960907	20961411	hbn	254	52.4802	7.63
chr2R	20963228	20963651	hbn	2466	23.5869	4.85
chr2R	20975318	20975583	CG34115	42	25.4009	5.41
chr2R	20996206	20999572	CG10543	1502	316.742	19.35
chr2R	20999969	21000918	CG10543	243	316.742	29.58
chr2R	21047643	21048425	shg	9305	210.658	16.12
chr2R	21056360	21057315	shg	358	150.052	11.39
chr2R	21057690	21057947	cpa	6	10.6309	3.04
chr2R	21072493	21073155	CG10527	72	316.742	18.86
chr2R	21073660	21074137	Treh	46	15.9182	3.71
chr2R	21075160	21077120	Treh	355	316.742	23.52
chr2R	21084194	21084525	Treh	50	13.8647	3.36
chr2R	21101539	21102114	ktub	129	143.014	15.65
chr2R	21125336	21125600	CG4050	2116	10.5548	3.15
chr2R	21126135	21126695	CG4050	1161	47.0783	6.81
chr2R	21127475	21128535	CG4050	433	197.012	12.59
chr2R	21141780	21142345	Magi	22	59.5991	7.82
chr2R	21143490	21144317	Xbp1	142	52.6004	5.82
chr2R	21158366	21158761	CG30389	595	21.7477	4.67
chr2R	21159708	21160506	CG30389	460	45.2997	5.88
chr2R	21174925	21175509	Pu	113	316.742	30.16
chr2R	21176866	21177125	CG4286	1684	15.011	3.85
chr2R	21178701	21179473	Pu	44	316.742	24.57
chr2R	21180720	21181249	Pu	1988	27.5095	4.76
chr2R	21193296	21193598	Lapsyn	129	20.3795	4.66
chr2R	21199537	21201547	CG44245	556	277.377	15.99
chr2R	21199537	21201547	Glycogenin	556	277.377	15.99
chr2R	21202612	21203024	Glycogenin	554	91.0529	9.55
chr2R	21214868	21215178	CG4302	1	22.7936	4.90
chr2R	21234729	21235066	ASPP	11086	20.0809	4.45
chr2R	21248710	21251726	ASPP	21	316.742	26.71
chr2R	21293580	21293899	Sgf29	94	32.3271	5.23
chr2R	21293580	21293899	RpL29	27	32.3271	5.23
chr2R	21295578	21296097	CG9752	113	9.34759	2.74
chr2R	21295578	21296097	CG42672	50	9.34759	2.74
chr2R	21323245	21323852	CG30394	299	33.2377	4.72
chr2R	21323245	21323852	dom	190	33.2377	4.72
chr2R	21361896	21363349	CG17974	12238	115.633	10.41
chr2R	21365468	21365799	cv-2	13855	22.3172	4.95
chr2R	21379153	21379729	cv-2	4	276.308	17.60

chr2R	21386474	21386848	Acox57D-p	20	25.0252	4.67
chr2R	21389225	21389500	Acox57D-d	62	16.8016	3.79
chr2R	21416925	21417207	Sdc	0	15.7254	3.83
chr2R	21474994	21477104	Sdc	4285	71.0245	7.55
chr2R	21480417	21481259	Sdc	297	316.742	23.49
chr2R	21496597	21497860	Fkbp14	323	316.742	31.29
chr2R	21501910	21503340	MESK2	587	294.166	19.61
chr2R	21505911	21506566	MESK2	258	18.8597	4.14
chr2R	21522150	21523078	Egfr	95	127.901	9.72
chr2R	21535440	21535769	CG30286	1181	41.3129	5.89
chr2R	21540937	21541618	CG30283	710	316.742	28.22
chr2R	21545233	21547494	Egfr	161	316.742	29.79
chr2R	21549910	21550751	Egfr	3769	275.507	19.33
chr2R	21552448	21553210	Egfr	6178	140.435	15.29
chr2R	21563825	21564269	twz	7451	43.2363	6.36
chr2R	21571381	21571755	twz	88	22.8708	4.77
chr2R	21606656	21607396	CG10433	86	316.742	38.77
chr2R	21611707	21612098	CG15673	1161	13.0667	3.21
chr2R	21612756	21613390	CG15673	36	198.449	15.96
chr2R	21624029	21624455	PTP-ER	227	31.7954	5.40
chr2R	21634557	21635218	CG10321	17	47.8446	6.07
chr2R	21645429	21647108	CG10082	68	316.742	24.26
chr2R	21649007	21649497	CG30284	2551	9.00951	2.93
chr2R	21661969	21662523	pirk	12	89.9897	9.86
chr2R	21663057	21663521	CR44748	46	18.1042	3.66
chr2R	21669848	21670244	CG9865	139	12.5161	3.07
chr2R	21669848	21670244	CG42379	139	12.5161	3.07
chr2R	21669848	21670244	CG42380	139	12.5161	3.07
chr2R	21669848	21670244	CG42381	139	12.5161	3.07
chr2R	21669848	21670244	CG9865	139	12.5161	3.07
chr2R	21669848	21670244	NC2alpha	96	12.5161	3.07
chr2R	21688083	21689027	CG30285	49	296.886	18.82
chr2R	21688083	21689027	CG10307	276	296.886	18.82
chr2R	21690824	21691161	Tim10	216	17.5689	3.83
chr2R	21690824	21691161	CG42497	216	17.5689	3.83
chr2R	21690824	21691161	Tim10	216	17.5689	3.83
chr2R	21700282	21701965	HmgD	66	77.28	8.00
chr2R	21702511	21705095	HmgZ	262	294.296	17.17
chr2R	21705786	21706377	HmgZ	1531	108.425	9.75
chr2R	21706737	21707243	HmgZ	872	31.5094	5.35
chr2R	21709151	21709839	CR44749	24	316.742	22.74
chr2R	21711540	21712246	CG30403	370	28.0489	4.74
chr2R	21712955	21715018	HmgD	86	316.742	26.95
chr2R	21720307	21720953	LBR	76	211.454	18.48
chr2R	21871554	21872231	Fili	242	149.454	10.63
chr2R	22039183	22039932	CG34370	112	90.2834	10.79
chr2R	22058923	22059187	CG13500	1397	24.4843	4.70
chr2R	22060791	22061182	Snp	13	24.1572	3.91
chr2R	22075903	22076243	GlcT-1	42	15.5373	3.46
chr2R	22080009	22080718	Synj	17	316.742	18.98
chr2R	22099128	22099769	CG11073	71	127.512	11.37
chr2R	22109016	22109298	Vrp1	3618	20.2385	4.36
chr2R	22116155	22116662	Vrp1	73	130.452	11.23
chr2R	22119460	22120070	mei-S332	2537	10.4012	3.03
chr2R	22122282	22122570	Vrp1	164	10.5547	2.76
chr2R	22136664	22137250	a	30	316.742	47.02
chr2R	22179372	22180667	CG11275	51	219.099	14.93
chr2R	22193164	22193461	Swim	162	22.3464	5.03
chr2R	22212590	22213546	ari-2	230	159.057	13.79
chr2R	22241832	22244202	dve	1726	316.742	33.59
chr2R	22270388	22271183	dve	146	316.742	31.14
chr2R	22271528	22271941	dve	773	22.0982	5.06
chr2R	22302432	22302772	wdp	1186	13.7502	3.68
chr2R	22311674	22312426	wdp	96	316.742	28.19
chr2R	22317485	22318924	Gp150	62	154.421	11.77
chr2R	22323329	22324514	Gp150	738	24.0577	4.61
chr2R	22354427	22355057	Liprin-gamma	47	94.2794	10.93
chr2R	22372800	22374096	Liprin-gamma	199	316.742	31.35
chr2R	22382679	22383478	CG13506	117	316.742	44.72
chr2R	22401909	22402334	babos	66	44.6026	6.86
chr2R	22403265	22404294	CG6044	580	249.519	21.16
chr2R	22426728	22427078	CG10344	64	25.328	4.35
chr2R	22454587	22454936	CG10384	14	20.8799	4.85
chr2R	22478131	22478620	CG4752	8	48.1198	7.17
chr2R	22513887	22514999	px	341	182.421	15.93

chr2R	22519795	22521676	px	7280	67.4883	9.67
chr2R	22546262	22546994	px	4	43.8403	6.47
chr2R	22561718	22562109	px	91	24.9393	4.49
chr2R	22569509	22571123	CG11362	7526	316.742	34.51
chr2R	22571598	22572159	CG11362	9606	138.158	11.75
chr2R	22576874	22577274	CG11362	14724	12.1308	3.77
chr2R	22592678	22593460	dnr1	13	316.742	25.66
chr2R	22606015	22606318	RpS16	40	16.1194	3.56
chr2R	22614173	22614602	CG33143	41	39.3398	6.73
chr2R	22646010	22646479	CG2852	61	22.7232	4.31
chr2R	22649236	22649967	CG3746	28	27.304	5.86
chr2R	22666985	22667469	CG30273	36	30.5536	5.38
chr2R	22666985	22667469	CG30269	36	30.5536	5.38
chr2R	22669167	22669625	CG13516	30	99.9817	8.72
chr2R	22670627	22671030	RYBP	53	18.1042	3.66
chr2R	22676267	22677384	CR44761	3368	55.6727	7.23
chr2R	22678002	22679095	CR44761	4916	316.742	37.96
chr2R	22680880	22681255	CR44761	7331	38.5336	6.30
chr2R	22683114	22683860	Ppa	5417	49.45	7.34
chr2R	22685674	22687236	Ppa	2992	316.742	20.87
chr2R	22688396	22690044	Ppa	93	316.742	31.41
chr2R	22688396	22690044	CR44762	14	316.742	31.41
chr2R	22688396	22690044	CR44763	414	316.742	31.41
chr2R	22700485	22701126	robo1	49	53.7436	8.20
chr2R	22728944	22729691	jbug	72	316.742	63.16
chr2R	22729975	22730234	jbug	824	11.4179	3.58
chr2R	22736674	22737493	jbug	58	316.742	21.27
chr2R	22740185	22740924	jbug	82	190.983	13.20
chr2R	22796838	22797243	CG42260	1275	52.1778	7.30
chr2R	22798169	22798587	CG42260	154	22.6627	3.81
chr2R	22799005	22799726	blw	545	11.1351	2.89
chr2R	22854206	22854558	RpL23	38	8.38578	2.56
chr2R	22860074	22860503	inaD	1515	28.9159	5.77
chr2R	22867584	22868032	fd59A	301	42.6774	7.14
chr2R	22874577	22874979	PIP5K59B	50	17.4341	4.57
chr2R	22894211	22894750	CG3700	23	316.742	22.47
chr2R	22895910	22896594	nahoda	22	316.742	26.15
chr2R	22901541	22902265	nahoda	5747	8.525	2.74
chr2R	22924736	22925340	Nup214	402	25.5623	4.49
chr2R	22924736	22925340	Reep1	38	25.5623	4.49
chr2R	22926118	22926981	Reep1	7	186.828	15.26
chr2R	22933621	22934944	CG3788	280	316.742	31.85
chr2R	22936586	22937132	CG3800	52	8.75696	2.63
chr2R	22940760	22941376	CG3831	23	24.1684	5.13
chr2R	22998614	22999038	CG9896	34	38.8965	5.57
chr2R	23042743	23043491	CG42741	152	70.5886	8.18
chr2R	23054912	23055238	CG44252	399	11.0587	3.55
chr2R	23067282	23067768	l(2)k09913	229	9.77495	2.62
chr2R	23203753	23204210	CG34371	14	89.947	8.49
chr2R	23328037	23328420	tbrd-3	706	28.5455	5.43
chr2R	23376693	23377056	Golgin245	217	26.1113	4.64
chr2R	23395206	23395578	CG3530	2	48.9415	7.63
chr2R	23395900	23396679	CG3530	483	9.9796	2.74
chr2R	23419229	23419836	CG9812	9	48.7559	5.69
chr2R	23425729	23426825	CG9815	97	316.742	26.87
chr2R	23427301	23427759	CG9815	1424	11.6945	3.14
chr2R	23428685	23429021	CG9815	2840	26.7911	4.53
chr2R	23431935	23432675	CG9815	320	85.9513	10.69
chr2R	23443745	23444306	CG43795	174	53.3608	8.71
chr2R	23523289	23523950	RabX1	224	19.0512	3.82
chr2R	23523289	23523950	mi	18	19.0512	3.82
chr2R	23547590	23548354	wmd	45	18.0413	3.39
chr2R	23547590	23548354	levy	205	18.0413	3.39
chr2R	23551245	23551995	Dcp-1	229	41.4703	5.73
chr2R	23551245	23551995	pita	10	41.4703	5.73
chr2R	23566971	23567343	apt	919	15.298	3.98
chr2R	23569812	23570480	apt	1955	91.0129	9.83
chr2R	23573051	23573700	apt	5240	95.0124	12.12
chr2R	23580049	23581347	apt	301	316.742	43.49
chr2R	23614666	23615034	CG4019	125	23.514	4.63
chr2R	23621322	23621792	CG17664	4806	73.0528	10.63
chr2R	23632115	23632592	retn	233	90.4961	8.21
chr2R	23652345	23652638	Pde8	5061	23.6142	4.21
chr2R	23657110	23657703	Pde8	6	13.5553	3.75
chr2R	23658916	23660212	Pde8	92	316.742	42.60

chr2R	23666239	23668053	Pde8	158	316.742	53.60
chr2R	23671690	23672876	Pde8	170	69.0167	8.09
chr2R	23683079	23683645	St3	26	186.499	14.02
chr2R	23687450	23687843	Pal2	412	12.7061	3.73
chr2R	23697490	23698389	CG30183	26	179.198	11.76
chr2R	23697490	23698389	mRpL43	254	179.198	11.76
chr2R	23706926	23707286	l(2)tid	36	31.3675	5.07
chr2R	23711974	23714241	Sesn	390	316.742	23.81
chr2R	23725132	23726212	CR45325	4969	74.327	8.61
chr2R	23726960	23727548	CR45325	6114	22.809	4.18
chr2R	23744501	23745231	egl	315	22.0066	4.41
chr2R	23745942	23746919	egl	138	31.3435	4.79
chr2R	23782843	23783111	CG15800	8303	12.3507	3.76
chr2R	23796248	23796739	CG9850	1879	123.778	9.80
chr2R	23797785	23798613	CG9850	84	15.2882	3.79
chr2R	23817956	23820592	CG4797	1927	57.4883	7.81
chr2R	23817956	23820592	CG5549	99	57.4883	7.81
chr2R	23826950	23827525	CG4763	2429	12.5224	3.68
chr2R	23839909	23840276	snoRNA:Psi18S-176	62	43.9234	5.51
chr2R	23862127	23862544	TBPH	76	7.71759	2.67
chr2R	23864862	23865394	CG4585	495	5.57776	2.21
chr2R	23870096	23870827	ken	122	316.742	20.74
chr2R	23871372	23874223	ken	1723	316.742	27.19
chr2R	23909995	23910649	kcc	17	121.988	10.86
chr2R	23932363	23933790	SERCA	23	18.1713	3.59
chr2R	23940258	23940641	Taldo	28	19.9971	3.94
chr2R	23977802	23980210	Sox14	133	316.742	17.56
chr2R	23982047	23982590	Sox14	2477	13.9685	3.67
chr2R	23988303	23988592	Phm	33	13.0059	3.20
chr2R	23994732	23995339	CG3860	71	76.3037	7.59
chr2R	24021721	24022132	CG3907	272	24.1126	4.29
chr2R	24026090	24026905	EbpIII	47	83.2666	9.43
chr2R	24033678	24034034	Chi	62	15.2491	3.41
chr2R	24033678	24034034	MAN1	92	15.2491	3.41
chr2R	24045405	24045842	tsr	1324	6.73657	2.33
chr2R	24046225	24046925	tsr	50	14.9988	3.53
chr2R	24116546	24116839	CG3257	57	20.6205	4.00
chr2R	24131171	24131853	tamo	286	302.147	21.28
chr2R	24133106	24134047	tamo	43	31.8561	4.62
chr2R	24133106	24134047	Zfrp8	480	31.8561	4.62
chr2R	24138752	24139643	Dat	575	61.2192	8.11
chr2R	24145900	24146241	Dat	790	12.2707	3.40
chr2R	24146575	24147108	Dat	42	273.442	26.02
chr2R	24156341	24156658	DnaJ-60	2740	9.48323	3.04
chr2R	24156341	24156658	CG42568	2740	9.48323	3.04
chr2R	24156341	24156658	DnaJ-60	2740	9.48323	3.04
chr2R	24161776	24162924	CG3328	8	316.742	22.37
chr2R	24197696	24197967	CG3356	12	13.91	3.21
chr2R	24229606	24230104	CG30419	75	60.6096	8.50
chr2R	24257773	24258132	CG3376	32	29.3863	4.97
chr2R	24275432	24276103	nvy	128	28.5196	5.77
chr2R	24291170	24291552	CG3394	2797	19.4026	4.37
chr2R	24293237	24294909	CG3394	62	316.742	35.12
chr2R	24293237	24294909	mir-4985	196	316.742	35.12
chr2R	24342440	24343052	bs	88	245.348	16.68
chr2R	24366383	24366849	CR44811	3059	23.514	4.63
chr2R	24369466	24370207	CR44811	89	316.742	24.03
chr2R	24388759	24390633	mAChR-A	672	316.742	23.99
chr2R	24401590	24403142	Slik	465	113.456	10.10
chr2R	24401590	24403142	Rpn8	1085	113.456	10.10
chr2R	24514420	24515137	Letm1	93	10.3923	2.82
chr2R	24525591	24526281	CG4612	272	133.494	11.20
chr2R	24525591	24526281	Brca2	354	133.494	11.20
chr2R	24531454	24532487	CG13585	32	52.6981	7.31
chr2R	24537921	24539232	ITP	1631	24.0583	4.95
chr2R	24543336	24543843	ITP	86	153.022	14.01
chr2R	24547559	24547837	ITP	4065	12.5022	3.45
chr2R	24549432	24550182	ITP	6083	25.4581	4.75
chr2R	24550650	24551405	CG4622	5194	126.246	12.67
chr2R	24571045	24571804	CG3511	285	92.0504	8.48
chr2R	24571045	24571804	Fcp1	319	92.0504	8.48
chr2R	24580826	24581643	pio	87	258.76	18.29
chr2R	24587400	24587779	pio	123	10.9047	3.34
chr2R	24599534	24600243	ATPsynF	5	25.0005	3.92
chr2R	24599534	24600243	CG3548	130	25.0005	3.92

chr2R	24606947	24607257	CG4707	4	11.4666	3.02
chr2R	24620899	24621464	CG4741	5904	33.9361	6.06
chr2R	24630808	24631366	Cyp9c1	9	297.631	19.97
chr2R	24638408	24638806	CG13594	27	36.5313	6.93
chr2R	24684885	24685573	Mmp1	24	102.729	9.00
chr2R	24687334	24688274	Mmp1	178	77.6321	8.36
chr2R	24694079	24694380	ST6Gal	305	5.66044	2.20
chr2R	24712474	24713413	ST6Gal	18524	225.551	21.09
chr2R	24727259	24727916	Mid1	8961	130.966	15.59
chr2R	24736441	24737000	Mid1	88	86.9516	9.55
chr2R	24739262	24739685	CR44828	57	26.9859	5.42
chr2R	24743706	24744512	Usp15-31	481	109.429	9.88
chr2R	24747883	24748368	Usp15-31	4488	23.9546	4.79
chr2R	24749446	24750197	Usp15-31	6006	201.054	14.87
chr2R	24757133	24757466	spz6	66	14.1049	3.76
chr2R	24765916	24766345	GstE12	1478	15.0172	3.58
chr2R	24767314	24768612	GstE12	68	316.742	42.33
chr2R	24793022	24794049	Reg-5	59	316.742	38.32
chr2R	24793022	24794049	CR43257	337	316.742	38.32
chr2R	24813340	24814998	Dll	998	316.742	28.00
chr2R	24823668	24824056	Dll	9047	40.9846	7.41
chr2R	24829522	24829863	Dll	14905	25.0042	5.32
chr2R	24860281	24860704	CR44829	1216	14.8275	3.75
chr2R	24863672	24863961	CR44829	4507	9.94473	3.26
chr2R	24874706	24875064	CG16896	34	27.1759	4.26
chr2R	24876241	24876816	CG44247	234	24.6023	4.60
chr2R	24881584	24882973	CR43466	840	95.5526	8.95
chr2R	24881584	24882973	NKAIN	189	95.5526	8.95
chr2R	24891268	24892151	NaCP60E	126	62.4538	8.87
chr2R	24903156	24904135	RpL41	559	28.754	4.80
chr2R	24903156	24904135	NaCP60E	740	28.754	4.80
chr2R	24927099	24927481	pain	1029	15.9801	3.94
chr2R	24928045	24928417	pain	29	23.4933	4.06
chr2R	24937268	24937594	CG30427	76	25.8836	4.66
chr2R	24940258	24940580	CG2811	24	19.7879	3.69
chr2R	24942157	24943429	Tina-1	767	266.339	16.87
chr2R	24948320	24948800	CG2790	173	11.2832	2.82
chr2R	24948320	24948800	CG12851	93	11.2832	2.82
chr2R	24962389	24962853	CG2765	10	21.6922	4.18
chr2R	24963213	24965313	CG2765	664	315.505	21.23
chr2R	24969361	24969982	RpL19	4	7.00964	2.23
chr2R	24969361	24969982	CG3776	126	7.00964	2.23
chr2R	24971709	24972090	Phk-3	54	50.9627	7.35
chr2R	24979530	24979909	emp	1353	7.78566	2.47
chr2R	24982876	24983653	emp	90	61.0288	7.89
chr2R	24985804	24987326	CG3829	900	316.742	21.96
chr2R	24985804	24987326	emp	873	316.742	21.96
chr2R	25010468	25011527	zip	443	177.695	13.34
chr2R	25011951	25012280	zip	140	23.3473	4.12
chr2R	25011951	25012280	uzip	44	23.3473	4.12
chr2R	25031775	25032599	Nplp1	51	101.641	10.99
chr2R	25052228	25052778	gsb-n	154	57.6512	8.80
chr2R	25061549	25062065	gsb	149	224.04	19.48
chr2R	25084087	25084674	gol	43	148.303	10.04
chr2R	25099699	25100732	lov	952	21.3548	5.05
chr2R	25114257	25114571	CG30430	15016	38.4295	7.15
chr2R	25115289	25115846	CG30430	13859	77.9997	8.94
chr2R	25215030	25215516	Kr	11324	53.9056	7.10
chr2R	25279662	25280231	CG9380	24587	94.0675	12.52
chr2R	25285105	25285976	CG9380	30308	271.254	15.01
chr3L	39962	40846	CG43149	14289	193.462	13.87
chr3L	128249	129942	Pdk1	39	316.742	28.07
chr3L	130823	132782	CR45805	97	316.742	32.86
chr3L	130823	132782	Pdk1	337	316.742	32.86
chr3L	140688	141460	Pdk1	66	316.742	29.71
chr3L	152632	152893	p130CAS	128	22.6806	5.20
chr3L	160651	161066	p130CAS	8	48.0535	6.58
chr3L	166058	166817	p130CAS	5116	20.4405	4.01
chr3L	178366	179162	Vdup1	29	316.742	20.06
chr3L	179819	180574	CG13875	86	316.742	21.02
chr3L	199039	199812	rno	445	82.7421	6.78
chr3L	203647	204316	NitFhit	313	61.5834	7.72
chr3L	205533	206202	Gyk	49	46.579	6.41
chr3L	205533	206202	CG13876	373	46.579	6.41
chr3L	222986	223241	pyx	1	14.834	3.55

chr3L	223839	225490	CG33229	53	200.73	13.86
chr3L	223839	225490	CR42862	53	200.73	13.86
chr3L	246420	247114	E(bx)	52	21.2997	3.84
chr3L	262674	263154	snoRNA:Psi18S-996	825	11.0211	2.81
chr3L	263817	264542	Tudor-SN	43	25.4402	4.41
chr3L	267456	267922	miple1	100	72.2204	8.62
chr3L	270864	272032	miple2	33	213.97	14.57
chr3L	277087	277693	RhoGEF3	74	84.5995	9.56
chr3L	282186	282639	RhoGEF3	4945	36.6169	5.27
chr3L	291748	292858	RhoGEF3	268	23.665	4.37
chr3L	311444	312014	fwd	98	80.2248	7.70
chr3L	315078	315405	CG34264	1857	26.9727	4.91
chr3L	319072	320041	fwd	48	38.2324	5.09
chr3L	332071	332441	mthl9	77	13.9596	3.73
chr3L	341215	341839	mthl10	65	60.0608	7.24
chr3L	346730	348069	mth	917	67.6254	7.02
chr3L	346730	348069	Ptpmeg	416	67.6254	7.02
chr3L	377785	378508	trh	881	316.742	22.99
chr3L	432595	433030	CG13891	1100	51.1014	7.19
chr3L	439533	439822	klar	1490	13.5724	3.48
chr3L	440698	442000	klar	45	316.742	33.46
chr3L	448000	448286	klar	13	33.644	6.08
chr3L	490528	491031	klar	28	165.214	15.69
chr3L	505812	506403	CR46002	3080	38.6252	5.35
chr3L	539402	540733	klar	343	50.8555	6.39
chr3L	542908	544743	Cypl	528	316.742	33.49
chr3L	542908	544743	Hipk	400	316.742	33.49
chr3L	545416	546516	Hipk	73	155.079	14.47
chr3L	550763	551025	CR46249	116	17.0481	4.06
chr3L	557053	557447	CR45808	4003	6.05588	2.22
chr3L	561046	561481	CR45808	8000	21.8225	4.62
chr3L	561771	562090	Hipk	7367	24.4843	4.70
chr3L	563050	564879	Hipk	5582	43.0447	5.85
chr3L	593210	593546	MED14	146	15.2794	3.54
chr3L	603707	604422	CG13893	51	316.742	25.13
chr3L	606314	607243	Reg-2	84	33.0999	5.18
chr3L	616338	617310	CR42719	197	316.742	29.55
chr3L	626007	627215	CR43334	1556	135.399	13.26
chr3L	627756	630392	CR43334	1667	283.94	17.93
chr3L	633385	633721	CR43423	4493	18.0995	4.22
chr3L	648404	649154	Gale	87	316.742	17.69
chr3L	662690	663040	Rev1	8516	21.2307	5.02
chr3L	673242	673809	CG17129	6767	98.4378	10.76
chr3L	681829	682775	CG3386	54	90.6169	8.16
chr3L	684974	685880	ebd1	40	316.742	25.69
chr3L	696754	697096	CG13894	2340	7.79166	2.80
chr3L	707698	708564	CG13895	36	316.742	25.34
chr3L	738299	739918	hng3	137	316.742	40.45
chr3L	748323	749737	emc	55	316.742	24.67
chr3L	751474	752267	emc	2371	89.9769	10.38
chr3L	753603	754316	hng3	1436	83.3739	8.73
chr3L	766037	766790	hng3	10917	13.6387	3.45
chr3L	769037	769687	hng3	13935	316.742	21.95
chr3L	774699	775349	hng3	19787	8.71584	2.75
chr3L	777277	777933	hng3	22144	19.008	4.23
chr3L	784003	784841	CG13898	19640	49.7132	6.49
chr3L	805021	805392	CG13898	1119	41.4594	7.19
chr3L	809249	809698	CR44537	3822	84.8759	9.81
chr3L	837505	837954	CG13901	34	11.7095	2.96
chr3L	837505	837954	CG13887	5	11.7095	2.96
chr3L	857049	857882	dpr20	25	44.7875	6.81
chr3L	871810	872773	Usp10	250	71.5426	7.84
chr3L	875612	875991	Usp10	2941	13.0938	3.48
chr3L	880774	882518	CG13907	31	316.742	51.77
chr3L	882800	883184	CG13907	1003	29.077	4.75
chr3L	885830	887081	Usp10	1416	67.6144	7.28
chr3L	895304	896227	CG34056	15	125.988	14.08
chr3L	913796	915200	Glut1	348	39.2811	6.19
chr3L	958970	960367	Glut1	66	316.742	33.79
chr3L	1000512	1001100	trio	251	121.988	10.86
chr3L	1000512	1001100	CR45811	208	121.988	10.86
chr3L	1007629	1008449	trio	403	200.671	18.08
chr3L	1022770	1023238	trio	220	24.2433	5.23
chr3L	1034220	1035260	CG9205	55	18.0426	3.49
chr3L	1034220	1035260	trio	124	18.0426	3.49

chr3L	1034220	1035260	CG9205	433	18.0426	3.49
chr3L	1098359	1099192	bab1	2197	316.742	22.99
chr3L	1101459	1103092	bab1	1438	316.742	31.66
chr3L	1177023	1178797	bab2	91	316.742	27.34
chr3L	1195975	1196471	Aplip1	107	68.5105	9.64
chr3L	1202669	1203088	CG32335	137	37.1888	6.37
chr3L	1204109	1205025	CG9119	511	25.7802	4.63
chr3L	1208061	1208668	mwh	81	280.769	19.58
chr3L	1256136	1256872	CG9134	1	240.309	19.00
chr3L	1274705	1276210	CG32333	19	83.9855	10.43
chr3L	1298481	1298963	scf	21	174.185	15.10
chr3L	1307125	1307385	CG2469	61	9.7112	2.64
chr3L	1313004	1313884	CG9186	298	25.2826	3.57
chr3L	1313004	1313884	CG9153	20	25.2826	3.57
chr3L	1334627	1336534	l(3)02640	634	182.733	12.80
chr3L	1334627	1336534	CG2211	163	182.733	12.80
chr3L	1349536	1350049	Ptp61F	218	18.3573	3.93
chr3L	1366801	1367434	ru	3592	107.414	9.69
chr3L	1379195	1379458	ru	8680	12.7271	3.45
chr3L	1463462	1464280	rho	27	151.849	11.13
chr3L	1489364	1489922	stet	93	114.553	10.17
chr3L	1502079	1502599	pUf68	278	12.3213	3.07
chr3L	1515723	1516027	cue	1436	15.1495	3.71
chr3L	1516328	1518102	cue	57	22.1008	3.81
chr3L	1516328	1518102	Psa	285	22.1008	3.81
chr3L	1536420	1536911	CG12091	87	13.3419	3.22
chr3L	1537181	1537773	CG7852	100	16.3747	3.48
chr3L	1546005	1547054	Cct1	328	88.672	7.74
chr3L	1549716	1550077	Cct1	50	20.9206	4.32
chr3L	1549716	1550077	CR45813	29	20.9206	4.32
chr3L	1557989	1558778	Pex10	229	35.3943	5.14
chr3L	1557989	1558778	CG12099	28	35.3943	5.14
chr3L	1568174	1568724	CG7879	119	26.2485	4.21
chr3L	1569078	1569437	CG12004	53	16.3667	3.85
chr3L	1569730	1570088	CG12004	679	17.2405	3.59
chr3L	1576515	1576829	CG13917	6	17.2241	3.55
chr3L	1577331	1577806	CG13917	865	44.195	6.45
chr3L	1586247	1587020	CG13917	94	316.742	54.04
chr3L	1591195	1591521	CG13917	4708	22.8209	4.56
chr3L	1592809	1593529	CG13917	6392	18.4479	3.76
chr3L	1626848	1627361	CG13928	1259	112.024	11.20
chr3L	1628027	1628717	CG13928	21	70.6617	9.14
chr3L	1629093	1629540	GC	3	32.8668	5.60
chr3L	1636752	1637822	nSyb	537	106.31	12.74
chr3L	1648703	1649086	CG13920	18	48.2803	7.11
chr3L	1652876	1653347	CG7967	268	22.5848	4.00
chr3L	1652876	1653347	CG7970	164	22.5848	4.00
chr3L	1661917	1662370	CG7971	2108	37.5953	6.28
chr3L	1663579	1663878	CG7971	524	9.56063	3.06
chr3L	1664152	1664518	CG7971	120	58.2368	7.34
chr3L	1730028	1730536	drpr	712	10.3923	2.82
chr3L	1730855	1731248	drpr	17	22.9262	4.46
chr3L	1738571	1739345	Gk	51	22.835	4.39
chr3L	1739919	1740178	Gk	729	7.39486	2.47
chr3L	1741642	1742062	Gk	66	25.5623	4.49
chr3L	1746189	1747224	CG13921	19	220.231	20.58
chr3L	1748347	1748820	CG13921	283	57.6881	6.74
chr3L	1755282	1756040	Cht2	105	154.756	11.90
chr3L	1794096	1795681	alpha-Spec	520	316.742	18.78
chr3L	1794096	1795681	dlt	520	316.742	18.78
chr3L	1794096	1795681	Cdc37	583	316.742	18.78
chr3L	1802915	1803289	CG42676	79	22.0647	4.25
chr3L	1810389	1810666	GV1	48	14.5688	3.29
chr3L	1831480	1832304	Cpr62Ba	145	13.5937	3.31
chr3L	1859624	1859992	Rap1	2017	36.3006	6.19
chr3L	1861244	1862380	Rap1	133	283.59	16.88
chr3L	1876107	1876433	dre4	44	12.7539	3.31
chr3L	1880264	1881040	CG13937	2180	11.0348	3.17
chr3L	1882549	1883558	CG13937	205	182.8	13.44
chr3L	1885326	1885789	Tmhs	65	66.4723	9.00
chr3L	1926105	1926842	CR45826	1837	10.6639	3.01
chr3L	1967290	1968532	CG1927	532	288.227	20.15
chr3L	1969487	1970249	CG1927	54	92.8131	8.65
chr3L	2150123	2150902	zormin	1194	316.742	22.50
chr3L	2151454	2152287	zormin	132	316.742	37.92

chr3L	2152646	2153046	zormin	1217	12.8751	3.34
chr3L	2163664	2164008	CG15822	41	25.856	4.89
chr3L	2168991	2169274	CG15879	1	22.0711	4.81
chr3L	2191946	2192551	CG5707	53	316.742	22.26
chr3L	2228353	2229546	Oseg2	473	212.201	18.76
chr3L	2237177	2237515	ACXD	365	7.8707	2.67
chr3L	2244110	2244378	CG32301	246	17.4916	3.52
chr3L	2255748	2257963	CG1275	52	149.8	12.73
chr3L	2255748	2257963	CG2034	1393	149.8	12.73
chr3L	2282397	2282998	MsR2	242	39.3302	7.25
chr3L	2322415	2322747	MsR1	148	13.319	3.48
chr3L	2387864	2388173	CG45186	2	24.2064	4.17
chr3L	2411303	2412284	CG45186	72	83.9024	9.95
chr3L	2417247	2417519	CG45186	594	20.0607	4.74
chr3L	2428651	2429067	CG45186	50	36.9101	5.15
chr3L	2433976	2434378	CG45186	4975	15.5019	3.90
chr3L	2466526	2467059	CG45186	97	273.148	16.33
chr3L	2478315	2479105	Cyp4d20	125	97.2944	9.35
chr3L	2488676	2489048	CG1146	47	31.5617	5.29
chr3L	2489880	2490441	CG1146	95	316.742	32.07
chr3L	2494231	2494713	CG45186	18	47.9695	6.28
chr3L	2494980	2495364	CG45186	639	32.9726	5.78
chr3L	2516800	2517124	CR45170	158	19.2862	4.28
chr3L	2554168	2554426	Spn	20	14.2045	3.10
chr3L	2581055	2581467	msn	4982	35.9886	6.22
chr3L	2584415	2584876	msn	1666	23.7963	4.76
chr3L	2585549	2586704	msn	227	316.742	27.01
chr3L	2589311	2590309	dos	654	130.649	11.83
chr3L	2599121	2599688	CG16985	12	24.8525	4.22
chr3L	2599121	2599688	CG12182	295	24.8525	4.22
chr3L	2631915	2633314	MEP-1	716	307.178	22.19
chr3L	2634786	2636550	MEP-1	281	149.303	12.56
chr3L	2711547	2712141	Fife	96	79.263	8.06
chr3L	2712518	2712961	Fife	1064	66.3745	8.18
chr3L	2734636	2734907	Mrtf	107	20.1656	4.54
chr3L	2766061	2766386	Mrtf	283	8.34871	2.50
chr3L	2773935	2774638	CG12093	451	14.7196	3.28
chr3L	2773935	2774638	Atg2	121	14.7196	3.28
chr3L	2820737	2821508	Tet	123	316.742	28.68
chr3L	2823204	2824099	Tet	2517	163.364	15.93
chr3L	2824401	2825146	Tet	3789	64.3803	8.11
chr3L	2828511	2829140	Tet	7883	44.9356	7.31
chr3L	2891935	2892509	spz5	59	312.813	22.34
chr3L	2894894	2895186	Shab	12	21.825	4.69
chr3L	2896378	2896958	Shab	92	83.8549	9.73
chr3L	3035172	3036180	CG2162	327	70.1853	6.60
chr3L	3036605	3036878	CG2162	1558	14.7593	3.32
chr3L	3037469	3038672	CG2162	90	96.6376	11.13
chr3L	3044813	3045469	scramb2	1725	86.793	8.51
chr3L	3046492	3046870	scramb2	63	14.2848	3.28
chr3L	3059788	3061324	CG16753	5669	137.213	10.68
chr3L	3069866	3071185	CG32486	74	316.742	17.72
chr3L	3069866	3071185	CG11486	367	316.742	17.72
chr3L	3071754	3072040	CG11486	636	21.614	4.33
chr3L	3077559	3078199	CG11486	59	258.49	14.71
chr3L	3080307	3081892	CG11486	273	302.037	20.60
chr3L	3090499	3090925	snRNA:U5:63BC	97	43.5001	6.12
chr3L	3100936	3101273	Cht7	5826	27.5697	5.55
chr3L	3123218	3123636	prominin-like	277	20.5148	4.55
chr3L	3133969	3134483	CG11537	74	218.101	18.89
chr3L	3139741	3140689	CG11537	546	23.665	4.37
chr3L	3143047	3143522	CG11537	42	10.1241	2.97
chr3L	3148155	3148426	Usp5	105	12.8367	3.13
chr3L	3148704	3149929	BtbVII	364	316.742	21.57
chr3L	3165169	3165470	CG32280	67	11.0069	2.97
chr3L	3178443	3178710	CG14964	5	12.8569	3.29
chr3L	3193023	3193496	Hsp83	4	69.3674	7.13
chr3L	3193964	3194495	Hsp83	899	17.7496	3.92
chr3L	3197113	3197583	CG14966	91	6.95269	2.20
chr3L	3198657	3199497	CG32276	59	25.969	4.21
chr3L	3198657	3199497	gry	101	25.969	4.21
chr3L	3224853	3226743	CG11505	241	316.742	43.80
chr3L	3246517	3246866	CG43389	1322	31.3617	5.61
chr3L	3249435	3250897	CR43626	108	316.742	45.62
chr3L	3267027	3267593	CG34265	1184	275.703	17.42

chr3L	3300937	3301579	CG14968	127	316.742	25.64
chr3L	3303684	3304315	ZnT63C	203	24.9883	4.07
chr3L	3319501	3319811	CG12016	105	9.45636	2.91
chr3L	3319501	3319811	CG42456	105	9.45636	2.91
chr3L	3325664	3326498	Strip	384	24.7659	3.66
chr3L	3325664	3326498	PHGPx	66	24.7659	3.66
chr3L	3336685	3337628	kst	80	264.426	15.61
chr3L	3338120	3338969	kst	1525	173.616	14.54
chr3L	3373933	3374361	YT521-B	20	17.0425	3.73
chr3L	3373933	3374361	CG12010	91	17.0425	3.73
chr3L	3417141	3420806	sty	554	316.742	23.49
chr3L	3424879	3425502	sty	394	28.793	5.41
chr3L	3427054	3427437	sty	2359	18.252	4.13
chr3L	3436931	3437188	sty	12170	14.2475	3.92
chr3L	3515667	3516119	Eip63E	125	38.8193	4.97
chr3L	3542289	3543921	Eip63E	813	316.742	25.87
chr3L	3544821	3545391	Eip63E	176	91.1141	9.38
chr3L	3616406	3616702	CG10357	1155	26.3999	4.81
chr3L	3624732	3625331	dar1	1329	102.986	11.82
chr3L	3625855	3626562	dar1	164	316.742	30.99
chr3L	3632191	3633372	dar1	6681	203.636	17.57
chr3L	3746502	3746962	CG32264	145	57.3921	7.89
chr3L	3747318	3747669	CG32264	896	51.6622	6.80
chr3L	3749488	3749802	CG32264	3063	21.5869	4.51
chr3L	3756669	3757129	ppk27	1751	50.9998	6.70
chr3L	3757734	3758155	CG32264	1237	75.3551	9.94
chr3L	3763147	3763535	CG32264	2358	60.2837	9.00
chr3L	3765563	3766216	CG32264	127	316.742	24.93
chr3L	3771269	3771537	CG32264	5775	8.4006	2.75
chr3L	3773861	3774265	CG32264	8243	18.0691	4.21
chr3L	3784049	3784343	CG32264	3715	11.3812	3.20
chr3L	3787047	3788239	CG32264	26	94.3781	9.10
chr3L	3789058	3789526	CG32264	1321	172.37	12.65
chr3L	3801064	3801828	CG32264	34	64.4974	8.04
chr3L	3802247	3802540	CG32264	1111	16.3449	3.91
chr3L	3814172	3814566	Scsalpha	146	11.7477	3.07
chr3L	3822710	3823387	Rdh	2458	89.0967	10.45
chr3L	3824844	3825974	Rdh	84	161.955	15.81
chr3L	3826315	3827153	Rdh	1134	31.0683	5.20
chr3L	3827993	3828497	enc	686	93.4102	10.89
chr3L	3830059	3830442	enc	1251	33.8181	6.40
chr3L	3845693	3846528	Awh	22	123.09	12.61
chr3L	3878826	3879173	CR43917	5757	14.76	3.49
chr3L	3901940	3902819	Ubi-p63E	25	49.5072	6.53
chr3L	3908987	3909335	mge	511	9.19162	2.97
chr3L	3925541	3926653	Eip63F-1	828	65.1514	7.73
chr3L	3942185	3943144	CG10863	18	41.6685	5.58
chr3L	3942185	3943144	CG14982	262	41.6685	5.58
chr3L	3952080	3952517	CG14984	192	11.636	3.35
chr3L	3960854	3961214	CG12605	956	13.4742	3.79
chr3L	3963330	3964420	CG12605	234	85.9274	10.10
chr3L	3964760	3965387	CG12605	412	22.4866	4.91
chr3L	3983150	3984033	scrt	59	116.234	12.63
chr3L	4025768	4026262	CG11594	23	227.222	17.49
chr3L	4062018	4062369	dib	79	86.2945	10.28
chr3L	4068429	4068740	CG32259	811	16.1276	3.37
chr3L	4070127	4070690	CG32259	894	240.702	18.39
chr3L	4071116	4072166	wit	83	316.742	43.75
chr3L	4073694	4074200	Faa	16	272.176	20.01
chr3L	4088843	4089176	CG14989	143	20.9712	5.04
chr3L	4092751	4093090	CG14990	283	55.1986	6.78
chr3L	4102258	4102945	CG14995	1	316.742	20.68
chr3L	4103445	4104344	Fit1	185	163.236	12.74
chr3L	4108167	4108818	Ack	276	31.045	4.51
chr3L	4109295	4109734	Ack	1294	29.5525	5.33
chr3L	4122009	4122579	Chd64	48	47.3088	5.82
chr3L	4133738	4134552	ens	321	241.939	15.18
chr3L	4138776	4139498	Rop	307	21.3272	4.12
chr3L	4138776	4139498	Ras64B	228	21.3272	4.12
chr3L	4147216	4147612	CG1299	1146	9.00951	2.93
chr3L	4148338	4149083	CG1299	64	197.248	13.70
chr3L	4170335	4170607	Ero1L	31	14.1002	3.32
chr3L	4178148	4178574	Teh4	24	14.0377	3.80
chr3L	4185096	4185742	Teh3	754	77.9396	9.02
chr3L	4187756	4188629	Teh2	104	46.2025	6.90

chr3L	4187756	4188629	tipE	371	46.2025	6.90
chr3L	4221312	4221625	CR32252	1235	20.9767	4.38
chr3L	4225601	4225961	ImpL2	79	12.8627	3.70
chr3L	4227077	4227661	ImpL2	80	201.422	16.29
chr3L	4227936	4229322	ImpL2	1609	155.787	14.34
chr3L	4232242	4232631	ImpL2	3673	29.2779	5.59
chr3L	4233609	4234376	ImpL2	2201	316.742	20.01
chr3L	4239376	4240419	CG14997	645	13.4374	3.10
chr3L	4252077	4252470	ago	251	52.7133	6.82
chr3L	4253325	4254400	ago	186	53.5625	7.20
chr3L	4286360	4286734	TfIIEbeta	514	28.441	4.49
chr3L	4288778	4289269	Hexo1	105	80.8688	9.32
chr3L	4360309	4360728	Cip4	3446	60.9484	7.30
chr3L	4363028	4364235	Cip4	323	106.466	9.48
chr3L	4372046	4372362	CG11342	4817	13.8213	4.01
chr3L	4380160	4380717	DopEcR	81	252.165	17.15
chr3L	4406809	4407378	DOR	78	158.544	14.67
chr3L	4409457	4410315	DOR	2903	35.1385	5.52
chr3L	4412124	4412455	DOR	2083	12.2014	2.98
chr3L	4412805	4413244	DOR	1250	21.2219	3.75
chr3L	4413734	4415591	DOR	166	316.742	20.33
chr3L	4416788	4417168	DOR	1719	27.5045	4.82
chr3L	4430342	4430880	nAChRbeta1	1379	31.816	5.27
chr3L	4431213	4431662	nAChRbeta1	2104	49.0457	7.76
chr3L	4436495	4436804	CG15021	33	19.1267	4.38
chr3L	4531853	4532423	Tie	24	173.384	14.74
chr3L	4541650	4544252	CG11357	15	316.742	23.90
chr3L	4556853	4557467	CG42540	158	35.5835	6.22
chr3L	4574409	4575211	CG42540	318	51.6407	8.20
chr3L	4624452	4624793	Src64B	1528	47.0028	6.34
chr3L	4625058	4626375	Src64B	24	155.466	11.65
chr3L	4626688	4627313	HDAC1	475	10.0788	2.82
chr3L	4626688	4627313	CR45438	305	10.0788	2.82
chr3L	4685012	4685275	axo	2131	9.82837	3.08
chr3L	4687272	4687591	axo	131	22.0038	4.59
chr3L	4692016	4693046	RhoGEF64C	485	316.742	22.55
chr3L	4708011	4708512	CG13712	1675	52.7313	8.10
chr3L	4800638	4801058	CR45740	846	43.8783	6.25
chr3L	4824435	4824840	Dhc64C	1026	21.1203	3.94
chr3L	4975971	4976790	Con	188	111.706	11.94
chr3L	5079010	5079404	Con	174	22.4872	5.07
chr3L	5126279	5126705	CG4597	3199	31.4493	6.36
chr3L	5152535	5152857	Srp54k	44	12.4529	3.00
chr3L	5180879	5181369	shep	3759	53.4551	7.44
chr3L	5184178	5185366	shep	100	316.742	39.28
chr3L	5186577	5187434	shep	1979	46.9023	6.69
chr3L	5206228	5206671	shep	4926	16.5777	4.14
chr3L	5243291	5243792	shep	4827	31.7491	5.94
chr3L	5247879	5249506	shep	801	278.846	17.71
chr3L	5273245	5273589	shep	138	20.8549	4.19
chr3L	5276531	5278297	shep	700	316.742	26.25
chr3L	5329279	5330017	CR45742	5294	64.4974	8.04
chr3L	5354592	5355998	lama	169	133.004	10.40
chr3L	5360010	5360487	Cyt-c1	18	70.4467	7.40
chr3L	5365358	5366227	Uev1A	156	40.2026	5.16
chr3L	5575648	5576000	Msr-110	648	15.0379	3.74
chr3L	5577162	5578076	Msr-110	38	30.8454	5.10
chr3L	5583633	5583962	l(3)psg2	28	10.2175	3.17
chr3L	5610425	5611093	Eaf6	2440	316.742	24.94
chr3L	5611636	5612459	Eaf6	3770	265.262	19.23
chr3L	5629707	5631212	Blimp-1	316	316.742	44.36
chr3L	5632403	5633020	Blimp-1	1610	57.4755	7.13
chr3L	5634062	5634609	Blimp-1	1618	69.535	8.18
chr3L	5635136	5635453	Blimp-1	720	26.4501	4.88
chr3L	5639466	5640541	Blimp-1	1861	316.742	21.05
chr3L	5642763	5643236	Blimp-1	628	29.0227	4.79
chr3L	5657454	5658043	sif	149	155.149	11.37
chr3L	5664059	5665018	sif	331	69.1224	8.47
chr3L	5665397	5665863	sif	525	51.9747	7.01
chr3L	5678215	5679084	sif	122	28.9673	5.44
chr3L	5688723	5689084	sif	2580	22.2685	4.94
chr3L	5690632	5691666	sif	107	96.8609	11.55
chr3L	5751474	5752349	DnaJ-1	29	67.1378	6.90
chr3L	5752958	5753717	Usp47	272	31.0738	4.80
chr3L	5768948	5769687	scny	189	83.0346	9.11

chr3L	5770127	5771501	scny	243	139.122	11.70
chr3L	5774653	5775206	vito	2510	20.3392	4.38
chr3L	5801892	5802276	CG42272	871	15.3421	4.05
chr3L	5807348	5808495	S6k	2909	81.5911	8.31
chr3L	5809749	5811126	S6k	29	17.6046	3.74
chr3L	5809749	5811126	CG5537	70	17.6046	3.74
chr3L	5845457	5845989	vn	19	90.3461	11.85
chr3L	5869633	5869949	CG5568	1754	28.0734	5.05
chr3L	5902148	5902789	QC	35	176.421	13.17
chr3L	5920787	5921417	CG10483	321	22.1626	4.76
chr3L	5922043	5922755	CG32407	76	227.566	16.47
chr3L	5923645	5924229	Vap-33B	27	37.6449	6.29
chr3L	5926348	5927054	Vap-33B	171	223.418	17.29
chr3L	5997033	5997375	PVRAP	66	23.2633	5.00
chr3L	6076473	6076880	ssp6	27	54.9599	7.11
chr3L	6082629	6083181	CG13293	73	19.7607	4.83
chr3L	6110746	6111406	Ets65A	578	17.1528	4.34
chr3L	6124698	6124997	CR46005	553	25.4402	4.41
chr3L	6184821	6185261	D19B	135	82.1764	8.88
chr3L	6184821	6185261	CG43293	133	82.1764	8.88
chr3L	6192958	6194093	CG13298	745	35.4255	4.54
chr3L	6192958	6194093	Trn	179	35.4255	4.54
chr3L	6218437	6218983	LanA	680	214.6	16.82
chr3L	6249053	6249336	Mdr65	3497	13.2742	3.68
chr3L	6253685	6253940	Best2	2843	11.7262	3.07
chr3L	6256355	6257305	Best2	7	316.742	23.73
chr3L	6262452	6263255	ImpL3	24	74.2693	9.72
chr3L	6263629	6264785	CR44522	496	243.873	18.48
chr3L	6352331	6352641	CG13300	67	17.8883	4.06
chr3L	6458427	6458740	CG10147	29870	14.3186	3.81
chr3L	6495514	6496961	sfl	674	237.114	16.89
chr3L	6550128	6551383	CG18769	339	53.288	6.30
chr3L	6552402	6552889	CG18769	1927	52.4677	6.25
chr3L	6570479	6570734	jv	18905	12.5936	3.64
chr3L	6605970	6606228	zpg	44	12.9286	3.04
chr3L	6605970	6606228	CG8368	102	12.9286	3.04
chr3L	6608882	6610023	CR45418	121	75.9139	8.96
chr3L	6608882	6610023	CG8398	207	75.9139	8.96
chr3L	6623630	6624219	GluRIA	166	122.027	12.79
chr3L	6703365	6703761	SP1173	8	38.6416	6.40
chr3L	6719397	6719784	ple	76	14.4789	3.20
chr3L	6728111	6728395	CR45417	94	14.4387	3.51
chr3L	6742129	6744129	dikar	578	160.344	11.44
chr3L	6747149	6747470	velo	65	46.3516	7.02
chr3L	6752188	6752867	velo	289	53.0335	5.68
chr3L	6789488	6790429	vvl	148	316.742	25.59
chr3L	6807882	6808277	vvl	17847	18.4702	4.44
chr3L	6863535	6863999	CR45115	2722	15.0895	4.05
chr3L	6866135	6866990	CR45115	70	16.4111	3.98
chr3L	6868090	6868441	CR45115	1828	18.5762	4.39
chr3L	6899313	6899839	Prat2	14417	16.5214	4.34
chr3L	6903753	6905107	Prat2	9695	316.742	40.30
chr3L	6942665	6943873	tow	680	145.827	14.04
chr3L	6964047	6964943	sgl	64	101.273	9.66
chr3L	6969597	6969896	CG9953	67	27.8127	5.07
chr3L	6977206	6977955	CR32385	1077	38.6704	6.08
chr3L	6979429	6980207	Galphai	142	12.6592	3.03
chr3L	6997419	6997878	Mp	1205	79.3605	10.50
chr3L	6998725	6999030	Mp	53	23.9919	5.25
chr3L	7013688	7014297	Mp	126	109.454	10.23
chr3L	7077362	7077692	CG8641	62	22.8458	4.99
chr3L	7094583	7095163	form3	7	109.455	9.72
chr3L	7131529	7131982	Dbi	78	80.9146	9.04
chr3L	7133863	7135275	melt	85	316.742	31.23
chr3L	7156815	7157298	corn	24	158.059	12.06
chr3L	7189553	7190180	Dscam2	4	95.5586	11.05
chr3L	7215896	7216237	CR45410	6685	27.3428	4.98
chr3L	7225263	7225886	CR45410	2752	49.7435	8.02
chr3L	7263496	7264362	CG14830	118	316.742	43.95
chr3L	7312790	7313244	unc-13-4A	186	40.2075	6.82
chr3L	7313689	7314157	unc-13-4A	1041	30.8834	5.68
chr3L	7328481	7328931	CG8602	41	17.7837	3.56
chr3L	7339466	7339785	lark	172	11.0059	2.81
chr3L	7344575	7345486	CG8596	55	316.742	19.19
chr3L	7347903	7348644	Srp19	460	26.2698	4.40

chr3L	7347903	7348644	qm	182	26.2698	4.40
chr3L	7359876	7361753	pst	1178	316.742	17.17
chr3L	7359876	7361753	Sec63	881	316.742	17.17
chr3L	7370038	7370763	akirin	16	37.2017	5.75
chr3L	7381202	7381851	SMSr	28	316.742	24.05
chr3L	7381202	7381851	smid	111	316.742	24.05
chr3L	7433426	7435699	Rac2	148	316.742	37.41
chr3L	7495932	7496460	Cyp4d8	11	109.965	11.19
chr3L	7498331	7498789	CG8539	51	82.8388	11.52
chr3L	7510937	7511434	CG7546	204	43.0447	5.85
chr3L	7518680	7519141	ltl	84	29.4923	5.90
chr3L	7528694	7529546	lqf	408	41.698	5.70
chr3L	7604933	7605254	CG33275	76	16.7679	4.05
chr3L	7614536	7614929	CG33275	9764	18.0593	4.65
chr3L	7711525	7711920	Ank2	1899	34.0192	6.65
chr3L	7713440	7713857	Ank2	31	14.2699	3.74
chr3L	7717962	7718539	Ank2	270	10.3968	3.21
chr3L	7719119	7719711	CG12262	16	38.908	4.90
chr3L	7747009	7747447	CG13685	121	53.7436	8.20
chr3L	7760294	7760722	Hn	11	32.7752	5.66
chr3L	7771986	7772832	Clk	3266	316.742	26.30
chr3L	7773404	7773920	Clk	1881	80.2876	10.07
chr3L	7775049	7776067	Clk	74	316.742	29.03
chr3L	7781542	7782130	Clk	6241	214.469	14.35
chr3L	7782969	7783324	Clk	7574	13.3343	3.53
chr3L	7787788	7788238	Clk	12418	23.4066	4.74
chr3L	7790062	7790810	CG32369	14235	204.256	15.21
chr3L	7804558	7805842	CG32369	813	248.197	14.42
chr3L	7808615	7810260	CG32369	54	316.742	32.69
chr3L	7811655	7811911	CG32369	1966	13.8647	3.36
chr3L	7826557	7827174	Pdp1	1178	139.709	12.17
chr3L	7827781	7828550	Pdp1	143	244.904	18.90
chr3L	7829725	7830014	Pdp1	1888	10.029	2.85
chr3L	7832673	7834421	Pdp1	822	316.742	21.67
chr3L	7834928	7836049	Pdp1	2361	108.226	11.01
chr3L	7836778	7837500	Pdp1	4170	316.742	20.28
chr3L	7847573	7848239	Pdp1	7684	133.012	13.87
chr3L	7854317	7857038	Pdp1	439	316.742	20.78
chr3L	7858614	7858973	Pdp1	3173	8.56262	2.82
chr3L	7862107	7862654	Pdp1	4990	58.1228	7.16
chr3L	7863200	7864491	Pdp1	3732	281.39	17.82
chr3L	7865142	7865680	Pdp1	1891	84.4217	8.72
chr3L	7866824	7867846	Pdp1	251	316.742	26.04
chr3L	7935504	7936137	syd	426	21.539	3.80
chr3L	7947866	7948314	exex	101	88.0073	10.25
chr3L	7968628	7968964	RNaseX25	1773	28.119	5.96
chr3L	7978866	7980165	nmo	51	147.968	12.88
chr3L	8024335	8024697	CR45867	31332	16.5938	3.65
chr3L	8056176	8056811	bip1	105	316.742	18.12
chr3L	8057332	8057902	CR45868	105	56.9395	7.05
chr3L	8058496	8058976	bip1	49	124.785	10.22
chr3L	8063218	8064120	Ect4	40	65.8869	8.57
chr3L	8065973	8066439	Ect4	2326	14.4543	3.10
chr3L	8071685	8072057	Ect4	900	31.5557	5.90
chr3L	8072643	8073363	Ect4	1247	25.3857	5.32
chr3L	8081465	8082299	CR43974	1186	316.742	25.82
chr3L	8091552	8092122	CG7565	95	308.871	18.49
chr3L	8095671	8096742	Ect4	422	163.671	12.99
chr3L	8102274	8102637	Ect4	1492	12.8505	3.48
chr3L	8115853	8116351	Arp3	259	13.7093	3.11
chr3L	8115853	8116351	msk	17	13.7093	3.11
chr3L	8116624	8117144	msk	889	10.1605	2.98
chr3L	8127767	8129041	Uba2	719	88.8879	8.01
chr3L	8127767	8129041	CdsA	484	88.8879	8.01
chr3L	8151717	8152407	CG13676	282	302.964	22.86
chr3L	8153172	8154013	CG13676	1919	28.2939	5.16
chr3L	8186617	8187498	ERR	344	63.9555	6.34
chr3L	8186617	8187498	Atg18a	21	63.9555	6.34
chr3L	8197706	8198042	UbcE2M	6	14.1991	3.61
chr3L	8240858	8241485	Dscam4	54	59.8558	8.35
chr3L	8285299	8285664	cert	965	29.4304	5.62
chr3L	8299442	8299828	Arl5	229	10.7303	2.82
chr3L	8314991	8315526	CG7185	213	86.2195	8.99
chr3L	8346621	8346976	GAPcenA	192	33.9473	6.24
chr3L	8358520	8359347	Idh	43	25.837	4.48

chr3L	8360189	8361084	Idh	74	178.074	13.48
chr3L	8395867	8396375	CG7120	77	22.9723	4.66
chr3L	8424857	8425508	Cbl	329	78.3002	8.63
chr3L	8427669	8428024	Cbl	2909	53.0283	7.03
chr3L	8441216	8441516	Unr	331	38.9058	6.05
chr3L	8441825	8442510	Unr	31	23.7366	4.23
chr3L	8449774	8451117	Gug	296	152.211	12.28
chr3L	8453525	8453821	Gug	3032	12.1589	2.97
chr3L	8454976	8455369	Gug	4521	19.3941	4.44
chr3L	8457544	8458308	Gug	7374	185.253	16.68
chr3L	8506529	8507352	CG43163	4	71.1808	7.50
chr3L	8512426	8513557	CG6765	73	122.844	13.61
chr3L	8518641	8519211	GstO3	95	264.002	15.47
chr3L	8523275	8523582	GstO2	49	23.5371	4.77
chr3L	8525168	8525926	foi	101	25.2255	4.28
chr3L	8528565	8528937	foi	42	51.1616	7.67
chr3L	8534904	8535345	ergic53	189	26.1113	4.64
chr3L	8546227	8546937	rhea	82	316.742	28.15
chr3L	8547381	8547757	rhea	921	24.6689	4.29
chr3L	8559727	8560414	rhea	128	131.689	13.49
chr3L	8583200	8583991	CG43078	138	31.9554	5.50
chr3L	8604049	8604852	CG5989	484	47.2525	6.68
chr3L	8604049	8604852	CG6282	23	47.2525	6.68
chr3L	8672691	8673179	h	2805	298.97	24.83
chr3L	8675226	8676242	h	51	135.434	12.04
chr3L	8692422	8692857	CR44526	8	16.7281	3.68
chr3L	8693725	8694279	CR44526	1255	116.553	12.31
chr3L	8696487	8696929	CR44526	3938	32.8225	5.32
chr3L	8697887	8698387	CR44526	5369	23.4481	4.56
chr3L	8700096	8701230	CR44526	7886	31.08	5.72
chr3L	8741081	8741430	Prm	2342	16.4023	4.23
chr3L	8765786	8766689	Fhos	96	213.731	16.94
chr3L	8766995	8768208	Fhos	752	22.0721	4.36
chr3L	8827148	8828125	dally	79	316.742	22.38
chr3L	8912381	8913003	Tsp66E	162	165.759	11.68
chr3L	8912381	8913003	mfr	32	165.759	11.68
chr3L	8913703	8914322	CR43970	1160	208.666	14.97
chr3L	8952508	8953397	CG43783	4	22.835	4.39
chr3L	8952508	8953397	orb2	4	22.835	4.39
chr3L	8954839	8955177	GNBP3	6	27.2021	4.60
chr3L	8968500	8968979	CG5026	153	7.88806	2.51
chr3L	8974361	8974839	pix	115	50.9213	6.99
chr3L	8978628	8979166	CG5644	956	212.096	16.69
chr3L	8979593	8980224	CG5644	102	125.055	13.34
chr3L	8996937	8998469	smg	26	144.166	12.22
chr3L	8996937	8998469	CG5087	911	144.166	12.22
chr3L	9004641	9005405	Doc3	176	164.677	15.60
chr3L	9019072	9019702	Doc2	147	118.181	12.99
chr3L	9040981	9041560	Doc1	143	67.6608	8.04
chr3L	9056169	9056915	Argk	977	17.8861	3.75
chr3L	9057499	9058406	Argk	166	122.403	11.29
chr3L	9059623	9060385	Argk	2345	22.6881	4.36
chr3L	9064338	9064994	Argk	1268	78.9815	9.00
chr3L	9065426	9066456	Argk	3	161.013	14.33
chr3L	9072169	9072669	CG4911	216	8.03147	2.49
chr3L	9124874	9125839	bol	108	316.742	29.16
chr3L	9126898	9128248	bol	2579	316.742	21.83
chr3L	9128734	9129105	bol	1416	35.5108	6.39
chr3L	9129364	9129913	bol	584	15.7078	4.07
chr3L	9134268	9135007	bol	365	59.4095	7.45
chr3L	9134268	9135007	Dhpr	4	59.4095	7.45
chr3L	9137531	9138041	nwk	74	64.1011	7.06
chr3L	9171877	9172338	Rdl	5342	11.6516	3.46
chr3L	9179142	9179910	Rdl	106	189.765	19.81
chr3L	9181899	9182582	Rdl	100	22.432	4.68
chr3L	9188132	9188599	Rdl	6222	104.276	11.69
chr3L	9190057	9190589	Rdl	8211	114.872	12.02
chr3L	9251611	9252123	GluRIB	107	115.657	14.09
chr3L	9333764	9334513	PGRP-LA	62	143.209	13.13
chr3L	9335391	9336249	PGRP-LA	12	316.742	26.28
chr3L	9337782	9338351	PGRP-LC	737	225.151	18.21
chr3L	9339445	9341335	PGRP-LC	55	316.742	30.59
chr3L	9349274	9349883	PGRP-LF	37	43.1682	5.36
chr3L	9357482	9359092	UGP	427	115.458	8.78
chr3L	9357482	9359092	CG32039	490	115.458	8.78

chr3L	9360246	9360792	Pdxk	182	27.4894	4.10
chr3L	9366884	9367803	Klp67A	22	15.1828	3.66
chr3L	9366884	9367803	CG4452	403	15.1828	3.66
chr3L	9373384	9374204	Hsp22	310	316.742	31.32
chr3L	9377093	9377932	Hsp26	16	312.006	20.48
chr3L	9378480	9378752	CR43481	434	13.0509	3.25
chr3L	9383381	9384272	Hsp27	49	75.095	7.63
chr3L	9398565	9399358	CG4080	39	33.0413	5.87
chr3L	9400834	9402553	eIF-4E	163	316.742	32.55
chr3L	9410053	9411208	CG4022	591	15.5923	3.43
chr3L	9410053	9411208	CG3689	796	15.5923	3.43
chr3L	9419800	9420909	CG3967	393	235.998	15.69
chr3L	9421814	9422304	aay	1193	65.5209	6.99
chr3L	9422608	9423529	aay	44	237.097	14.20
chr3L	9426326	9427026	CG3967	71	13.5865	3.35
chr3L	9426326	9427026	Shc	403	13.5865	3.35
chr3L	9429634	9430647	RpS17	653	18.6655	3.67
chr3L	9429634	9430647	MTF-1	163	18.6655	3.67
chr3L	9446842	9447853	CG3529	215	63.8743	8.50
chr3L	9451924	9452208	ghi	110	7.05709	2.29
chr3L	9455443	9456365	phol	654	26.9291	4.61
chr3L	9455443	9456365	CG3552	690	26.9291	4.61
chr3L	9455443	9456365	CR45873	711	26.9291	4.61
chr3L	9460858	9461311	CG44838	68	19.2027	3.64
chr3L	9461865	9462996	CG44838	404	316.742	22.57
chr3L	9465434	9466508	CG44838	2778	142.623	14.00
chr3L	9471575	9472003	Uch-L5	5140	17.5268	4.29
chr3L	9477336	9478033	Jarid2	484	23.769	4.62
chr3L	9503579	9503950	path	1620	40.5664	6.99
chr3L	9505023	9506191	path	36	149.841	15.61
chr3L	9508946	9509709	CG3408	305	172.694	11.49
chr3L	9546721	9547476	CG42673	124	316.742	29.58
chr3L	9614558	9615081	LanB2	56	58.3536	7.44
chr3L	9675541	9676016	fry	306	68.7237	8.59
chr3L	9678140	9678772	fry	748	129.809	13.82
chr3L	9689795	9690122	CR45804	3831	18.5598	4.13
chr3L	9692224	9695772	CR45804	1057	316.742	37.52
chr3L	9692224	9695772	CG6767	1694	316.742	37.52
chr3L	9692224	9695772	CR45121	2279	316.742	37.52
chr3L	9692224	9695772	CG6767	2678	316.742	37.52
chr3L	9696541	9697293	CG6767	258	176.174	15.17
chr3L	9698342	9698905	Ubc4	273	35.9945	5.67
chr3L	9704333	9704708	CG16711	262	21.3369	4.34
chr3L	9713501	9714911	SH3PX1	496	43.2363	6.36
chr3L	9713501	9714911	vsg	0	43.2363	6.36
chr3L	9716809	9717312	snmRNA:838	461	9.52296	2.69
chr3L	9751350	9751882	CG42268	126	92.0504	8.48
chr3L	9760193	9760709	CG42268	89	138.598	12.11
chr3L	9761800	9762425	CR45407	1185	14.4441	3.27
chr3L	9773939	9774399	CG8177	34	17.5222	4.36
chr3L	9786844	9787206	CG32052	37	12.1308	3.77
chr3L	9851789	9852272	RasGAP1	3350	10.8746	3.09
chr3L	9853638	9855389	RasGAP1	1187	126.833	9.87
chr3L	9927157	9927676	CG34356	65	22.3373	4.80
chr3L	9974406	9974787	dpr6	33	31.6293	5.05
chr3L	10185987	10186396	dpr10	85	40.2833	5.86
chr3L	10224722	10225212	scramb1	350	10.6639	3.01
chr3L	10226004	10226445	scramb1	920	53.8471	8.11
chr3L	10258848	10259119	can	8118	10.3226	3.48
chr3L	10289241	10289838	Or67d	16447	58.8054	8.36
chr3L	10306130	10306908	CR46004	288	77.1282	10.56
chr3L	10307928	10308495	CR46003	240	98.4315	11.99
chr3L	10321836	10322340	CR46006	1389	40.1394	5.35
chr3L	10324887	10326072	CR46006	1425	316.742	25.45
chr3L	10331494	10332706	CR46006	128	88.5249	7.78
chr3L	10354758	10355086	CR46006	9059	19.0903	4.13
chr3L	10363716	10364213	CR46006	2	71.8886	6.39
chr3L	10368791	10369485	mir-276a	3949	45.7154	7.08
chr3L	10407611	10408417	CR44543	2975	18.9633	4.34
chr3L	10435481	10435742	Ir67c	1504	9.50575	3.26
chr3L	10452801	10453119	Gem3	10146	22.7222	4.81
chr3L	10500570	10500989	S-Lap4	9941	72.7103	10.28
chr3L	10515790	10516965	A2bp1	32	133.494	11.20
chr3L	10517476	10517885	A2bp1	1784	15.3953	3.76
chr3L	10521524	10521929	A2bp1	5724	30.3821	5.57

chr3L	10522425	10522806	A2bp1	6638	32.1019	5.13
chr3L	10526575	10526867	A2bp1	10764	18.1596	4.37
chr3L	10655744	10656045	CG32066	4100	20.5555	4.78
chr3L	10658800	10659527	CG32066	1021	33.3017	6.01
chr3L	10660240	10661136	CG32066	38	38.7872	5.88
chr3L	10664398	10667033	simj	770	316.742	19.49
chr3L	10669790	10670469	simj	5231	23.514	4.63
chr3L	10674083	10674584	simj	9604	127.262	12.84
chr3L	10675844	10676250	simj	11287	40.8499	5.95
chr3L	10678574	10679128	CG11811	12015	23.9005	4.71
chr3L	10680669	10681355	CG11811	9984	316.742	25.83
chr3L	10681651	10684040	CG11811	9053	316.742	31.16
chr3L	10696636	10697736	NijA	3	218.496	16.67
chr3L	10708413	10709113	NijA	11914	53.8471	8.11
chr3L	10724811	10725328	NijA	28002	10.5384	3.20
chr3L	10736708	10740801	CG43245	34717	316.742	47.44
chr3L	10742448	10743326	CG43245	29014	316.742	21.94
chr3L	10748083	10748471	CG43245	23630	34.7685	5.89
chr3L	10752792	10753160	CG43245	18915	32.385	6.08
chr3L	10756059	10756603	CG43245	15546	21.0144	4.88
chr3L	10775631	10777620	CG43245	4227	316.742	22.40
chr3L	10810885	10811503	CG42831	3674	57.7597	7.68
chr3L	10813314	10813577	CG42831	1365	11.822	3.69
chr3L	10816864	10817356	CR45247	558	21.8144	5.16
chr3L	10852202	10853510	CR45169	1787	187.888	15.55
chr3L	10853906	10854857	tna	3589	47.4773	7.14
chr3L	10856355	10858135	tna	1032	316.742	28.54
chr3L	10866647	10868420	tna	1939	288.401	17.96
chr3L	10869039	10869485	tna	588	19.1824	4.20
chr3L	10881950	10882314	CG6409	66	16.4323	3.98
chr3L	10885940	10886202	CG6404	2	17.4372	3.97
chr3L	10897939	10899513	Aps	122	144.074	11.22
chr3L	10970231	10970502	CG14147	2263	16.6768	4.31
chr3L	10990011	10990667	snoRNA:Me18S-G962	10966	9.34759	2.74
chr3L	11008817	11009762	klu	101	316.742	30.25
chr3L	11010100	11011183	klu	1238	316.742	41.18
chr3L	11046447	11047018	CG43693	96	232.613	19.75
chr3L	11047962	11048221	CG43693	858	21.9712	4.18
chr3L	11050276	11050905	CR45119	156	48.851	7.31
chr3L	11050276	11050905	CG43693	123	48.851	7.31
chr3L	11070298	11071103	Mocs1	41	145.717	12.05
chr3L	11084297	11085199	JIL-1	6925	185.136	14.78
chr3L	11092316	11093274	JIL-1	277	74.685	6.96
chr3L	11096251	11096980	CG33947	7	316.742	24.13
chr3L	11113306	11114038	Sod	247	29.1313	4.70
chr3L	11118402	11120903	FoxK	405	145.606	11.00
chr3L	11118402	11120903	mRpL2	1975	145.606	11.00
chr3L	11123908	11124283	NaPi-III	743	42.734	5.74
chr3L	11135851	11136268	CG32082	27	61.8447	7.77
chr3L	11170021	11170308	CG7616	127	12.0718	2.87
chr3L	11205685	11206136	Plod	166	49.1249	5.90
chr3L	11214409	11216078	CG42671	13	316.742	24.84
chr3L	11239378	11239718	CR44718	1948	24.8381	4.90
chr3L	11242615	11243191	CR44713	4070	316.742	37.01
chr3L	11245858	11246657	CR44713	787	316.742	27.68
chr3L	11247343	11248536	CR44715	351	316.742	22.17
chr3L	11247343	11248536	CR44717	382	316.742	22.17
chr3L	11251781	11252573	scyl	108	316.742	42.85
chr3L	11275622	11276357	CR44720	1660	32.8668	5.60
chr3L	11285529	11285969	CR45404	1038	44.0719	6.43
chr3L	11291080	11291632	CG6175	3415	43.664	7.15
chr3L	11292033	11292656	CG6175	2457	53.2638	6.49
chr3L	11293408	11293924	CG6175	1180	40.3218	6.23
chr3L	11294453	11296114	CG6175	130	316.742	28.13
chr3L	11296387	11297448	CG6175	709	229.958	19.60
chr3L	11298834	11299418	CG6175	3059	20.4943	4.54
chr3L	11364792	11365964	CG6163	716	316.742	22.64
chr3L	11366418	11366897	CG6163	1816	35.153	5.66
chr3L	11428282	11428860	CG7560	3426	246.395	15.61
chr3L	11431023	11432202	CG6149	4086	30.3262	5.14
chr3L	11487147	11488199	chrb	106	316.742	31.06
chr3L	11492533	11492946	chrb	1098	24.5089	4.83
chr3L	11493348	11493849	chrb	299	109.504	9.57
chr3L	11523331	11523661	CG11714	773	37.3645	5.45
chr3L	11523331	11523661	Fuca	773	37.3645	5.45

chr3L	11523331	11523661	CG11714	773	37.3645	5.45
chr3L	11532518	11533161	Mob2	1658	17.2058	3.47
chr3L	11534219	11535442	Mob2	113	316.742	40.31
chr3L	11536891	11537556	Mob2	2628	149.682	11.94
chr3L	11552874	11553790	Mob2	298	316.742	32.50
chr3L	11556130	11556462	Mob2	2422	20.7671	4.53
chr3L	11557199	11557686	Mob2	1293	134.543	12.88
chr3L	11561872	11562291	CG7394	141	17.7074	4.08
chr3L	11561872	11562291	Mob2	97	17.7074	4.08
chr3L	11586616	11588094	rt	840	316.742	26.86
chr3L	11588475	11588911	rt	864	19.8065	4.46
chr3L	11591728	11592796	CR45442	766	42.061	7.43
chr3L	11591728	11592796	CR45672	773	42.061	7.43
chr3L	11596451	11597380	CG7368	499	140.352	11.87
chr3L	11599090	11600056	CG14137	620	35.1247	6.04
chr3L	11601049	11601671	CG7368	97	87.978	10.14
chr3L	11601992	11602419	CG7368	783	50.5102	7.54
chr3L	11623429	11623939	CG6084	2	118.639	10.14
chr3L	11629549	11629876	mir-3642	201	14.6003	3.81
chr3L	11656470	11656984	mir-274	195	41.1492	7.45
chr3L	11680134	11680644	CG32085	128	45.3758	7.40
chr3L	11683958	11684493	Sugb	271	16.9441	3.54
chr3L	11707750	11708628	CG11658	291	316.742	40.74
chr3L	11714999	11715635	CG14132	42	316.742	31.53
chr3L	11764932	11765465	CG6024	620	77.725	9.59
chr3L	11765722	11766376	CG6024	3	20.1137	4.46
chr3L	11776749	11777173	CG14131	83	81.4122	10.93
chr3L	11788963	11790091	Pi3K68D	755	90.6577	8.89
chr3L	11819475	11820285	CG5946	75	316.742	22.31
chr3L	11821627	11822311	CG11597	183	316.742	23.66
chr3L	11821627	11822311	CG5946	183	316.742	23.66
chr3L	11821627	11822311	CG11597	183	316.742	23.66
chr3L	11821627	11822311	CG5946	253	316.742	23.66
chr3L	11821627	11822311	CG11597	253	316.742	23.66
chr3L	11821627	11822311	CG5946	253	316.742	23.66
chr3L	11862761	11863699	CG44837	4	34.9443	6.66
chr3L	11918290	11918552	CG11588	3115	18.7107	4.11
chr3L	11927227	11927669	byn	233	44.0855	7.34
chr3L	11937258	11937513	CG44837	1458	17.6329	4.46
chr3L	12002407	12003231	Pop2	148	31.0683	5.20
chr3L	12002407	12003231	CG6928	387	31.0683	5.20
chr3L	12040310	12040735	CG6793	1919	16.8118	3.89
chr3L	12056186	12056871	rols	22	316.742	33.96
chr3L	12081552	12082866	Sema-5c	25	309.214	20.10
chr3L	12110993	12111308	l(3)j2D3	4921	40.9426	7.29
chr3L	12124135	12124620	yps	819	55.7668	6.01
chr3L	12124909	12125197	yps	56	5.20568	2.08
chr3L	12146566	12147371	Nrx-IV	633	267.108	18.27
chr3L	12163684	12164145	CG9760	203	138.931	16.12
chr3L	12175413	12175833	Rh7	131	20.3795	4.66
chr3L	12179769	12180091	CAH2	100	14.7276	3.97
chr3L	12197519	12198018	CG11529	92	62.2997	8.95
chr3L	12203334	12204067	Adk1	47	26.1125	4.47
chr3L	12223240	12223494	app	919	7.78943	2.99
chr3L	12265284	12266818	app	186	64.1322	6.63
chr3L	12277474	12277857	CG10426	14	29.9229	5.33
chr3L	12296144	12296439	CG4328	144	22.5747	5.26
chr3L	12328807	12329200	CG32105	278	18.5969	4.62
chr3L	12391230	12391593	Ncc69	13	42.0673	7.55
chr3L	12407028	12407730	CG32103	1250	196.712	13.45
chr3L	12408361	12408993	CG32103	24	231.735	17.17
chr3L	12432440	12433108	toe	168	76.7118	9.51
chr3L	12433632	12434923	toe	1948	194.764	15.76
chr3L	12467795	12468545	eyg	152	249.174	15.48
chr3L	12489810	12491328	CG10660	107	316.742	34.94
chr3L	12489810	12491328	CR45681	111	316.742	34.94
chr3L	12489810	12491328	CG10660	494	316.742	34.94
chr3L	12503851	12504518	CG10646	376	28.9242	5.28
chr3L	12503851	12504518	vih	21	28.9242	5.28
chr3L	12514707	12516124	sti	1027	282.775	18.54
chr3L	12514707	12516124	CR45168	833	282.775	18.54
chr3L	12514707	12516124	tral	784	282.775	18.54
chr3L	12529666	12530181	nst	134	13.8873	3.41
chr3L	12580090	12580916	ara	181	264.55	15.98
chr3L	12609093	12609845	caup	133	316.742	25.06

chr3L	12627446	12628204	CR32111	83	316.742	28.56
chr3L	12653083	12653550	CR43431	253	110.612	9.41
chr3L	12692651	12693900	mirr	212	316.742	29.49
chr3L	12788510	12788846	CG42709	1505	22.3373	4.80
chr3L	12789812	12790520	CG42709	0	316.742	20.76
chr3L	12790948	12791460	CG42709	32	316.742	23.05
chr3L	12798506	12799478	Atg1	6211	14.4209	3.38
chr3L	12804128	12805244	Atg1	570	316.742	29.23
chr3L	12805892	12806741	Sap130	57	112.489	8.69
chr3L	12832336	12832817	CG10960	32	87.1962	11.46
chr3L	12834105	12834663	CG10960	1884	16.0203	4.14
chr3L	12842864	12843691	CG10960	15	31.4376	5.88
chr3L	12849853	12850318	CG10948	93	13.3268	3.26
chr3L	12849853	12850318	Wbp2	46	13.3268	3.26
chr3L	12892409	12892922	CG14118	29	115.985	12.09
chr3L	12913797	12914285	CG32115	392	316.742	22.18
chr3L	13022384	13022798	AdenoK	155	20.4566	3.83
chr3L	13023251	13023577	RpS12	73	12.1589	2.97
chr3L	13032081	13032684	CR45157	529	22.3712	5.04
chr3L	13044238	13044777	Syx13	32	30.4913	4.87
chr3L	13113658	13114570	trn	151	316.742	25.90
chr3L	13227469	13229009	caps	120	316.742	31.25
chr3L	13235927	13237234	caps	8363	32.1482	5.33
chr3L	13403439	13403925	sens	2568	73.6749	10.09
chr3L	13426383	13426812	CG14105	36	12.1935	3.38
chr3L	13435045	13435600	CG10713	173	77.2515	8.63
chr3L	13442537	13442886	cmb	57	12.5224	3.68
chr3L	13474674	13475162	CG10089	45	54.2892	7.33
chr3L	13476474	13478666	stv	186	316.742	47.72
chr3L	13479519	13480824	stv	120	165.562	13.46
chr3L	13494351	13495445	Tgi	476	90.7138	8.67
chr3L	13497327	13498509	Tgi	2127	131.436	11.18
chr3L	13502577	13503021	Spt20	188	10.1576	2.69
chr3L	13666721	13668092	bru-3	719	101.273	10.20
chr3L	13803270	13804277	bru-3	663	24.7508	5.18
chr3L	13840726	13841125	CR46266	280	28.5516	5.14
chr3L	13901741	13902477	dysc	1139	102.715	11.06
chr3L	13902969	13903885	dysc	41	52.5077	7.19
chr3L	13904314	13904953	dysc	1399	130.365	11.13
chr3L	13917630	13918349	Rgl	1741	9.98162	2.87
chr3L	13919310	13920775	Rgl	77	316.742	39.58
chr3L	13922116	13922474	Rgl	14	20.7834	4.35
chr3L	13922804	13923215	Rgl	684	89.9897	9.86
chr3L	13923634	13924273	Rgl	1222	45.6556	6.89
chr3L	13925059	13925706	Rgl	89	316.742	29.86
chr3L	13934829	13935357	CG32137	4196	172.805	15.91
chr3L	13938327	13939553	CG32137	636	286.554	19.66
chr3L	13991231	13991951	CG17364	1882	105.849	11.15
chr3L	13993209	13993747	CG17364	134	125.501	12.31
chr3L	14002050	14003283	upSET	815	150.784	12.00
chr3L	14003558	14007009	upSET	2092	116.618	8.27
chr3L	14028552	14029119	Ptip	418	11.597	2.82
chr3L	14030984	14031694	endos	147	30.3217	4.95
chr3L	14030984	14031694	CG6650	229	30.3217	4.95
chr3L	14036945	14038666	Hsc70Cb	1114	316.742	25.37
chr3L	14052999	14053504	CG13484	169	32.6391	5.10
chr3L	14052999	14053504	CG32138	34	32.6391	5.10
chr3L	14055041	14055411	CR46067	933	45.6428	7.06
chr3L	14055709	14056332	CR46067	1767	226.124	20.34
chr3L	14059622	14059964	CR46067	5516	22.6835	4.48
chr3L	14063716	14064341	Pex1	7764	65.6742	7.98
chr3L	14071686	14072115	Pex1	50	44.3082	5.85
chr3L	14109771	14110163	Sox21a	1835	30.6062	6.26
chr3L	14131223	14132186	Sox21b	481	206.658	15.01
chr3L	14177391	14178407	D	157	316.742	21.75
chr3L	14190715	14191070	nuf	28	23.3849	3.67
chr3L	14200902	14201408	CG32141	2104	180.075	17.68
chr3L	14205087	14205597	nuf	139	185.253	16.68
chr3L	14219557	14220072	nuf	94	144.088	11.56
chr3L	14221043	14221540	nuf	102	45.6836	7.26
chr3L	14272098	14273467	fz	1353	316.742	27.39
chr3L	14273864	14274608	fz	126	316.742	59.79
chr3L	14409367	14410963	stwl	533	316.742	21.79
chr3L	14409367	14410963	CG3919	153	316.742	21.79
chr3L	14409367	14410963	CR45233	226	316.742	21.79

chr3L	14427139	14427774	bbg	142	189.099	13.78
chr3L	14448389	14448989	bbg	127	316.742	31.70
chr3L	14449388	14449709	bbg	979	20.9131	4.43
chr3L	14450540	14450883	bbg	2146	25.3934	5.59
chr3L	14476600	14477403	bbg	280	316.742	32.84
chr3L	14537459	14537976	Mpcp	5	30.6488	4.45
chr3L	14544000	14544309	Mpcp	6421	14.0377	3.80
chr3L	14550709	14551614	Gbs-70E	359	170.068	13.38
chr3L	14552712	14553065	Gbs-70E	1902	16.7774	4.12
chr3L	14578881	14579355	CG42758	11700	65.7447	7.51
chr3L	14599506	14599893	HGTX	134	38.288	6.70
chr3L	14600628	14600971	HGTX	1229	32.7876	6.27
chr3L	14613329	14613788	shd	804	14.4803	3.62
chr3L	14614075	14614516	shd	94	21.1336	4.19
chr3L	14622469	14623462	CG9628	27	138.18	10.57
chr3L	14624314	14625360	CG9628	1786	135.029	12.50
chr3L	14632465	14632773	dlp	1	31.0549	5.41
chr3L	14672592	14673025	ome	51	89.295	10.84
chr3L	14681563	14682145	CG5048	3016	111.154	11.38
chr3L	14706341	14707031	ome	70	316.742	17.60
chr3L	14731566	14731851	CG13471	1825	16.2987	4.14
chr3L	14743195	14743697	CG13471	9958	60.7841	7.35
chr3L	14754695	14760494	Trl	782	316.742	47.51
chr3L	14754695	14760494	CG42507	1408	316.742	47.51
chr3L	14754695	14760494	Trl	1408	316.742	47.51
chr3L	14775680	14776173	mop	213	88.1317	9.19
chr3L	14781091	14781410	mop	5124	36.1832	6.09
chr3L	14785280	14786897	bmm	840	304.972	18.02
chr3L	14785280	14786897	Tdrd3	1218	304.972	18.02
chr3L	14804680	14805336	CG17839	11	67.6809	6.62
chr3L	14819217	14819892	CG17839	14630	19.9971	3.94
chr3L	14843512	14843905	CG17839	38648	18.7287	4.06
chr3L	14984734	14985119	mnd	188	41.8878	6.60
chr3L	14986960	14987660	mnd	121	118.302	11.37
chr3L	14992031	14992384	Zip71B	75	44.1734	7.46
chr3L	15006482	15006952	Prosbeta2	6891	11.9679	3.48
chr3L	15079281	15079741	Plp	78	101.329	12.73
chr3L	15094147	15094743	sstn	283	88.1004	7.92
chr3L	15094147	15094743	Plp	288	88.1004	7.92
chr3L	15098533	15099785	CR43973	216	316.742	23.78
chr3L	15101484	15101901	CR43973	2164	30.1541	5.86
chr3L	15104094	15105933	CTPsyn	1401	80.8688	9.32
chr3L	15104094	15105933	CG45071	1401	80.8688	9.32
chr3L	15107549	15108549	CR43971	812	11.3903	3.51
chr3L	15125188	15125619	CG16959	74	38.7959	6.91
chr3L	15141344	15142796	CR45396	212	275.764	17.63
chr3L	15141344	15142796	Pdi	674	275.764	17.63
chr3L	15146691	15147263	FucTA	279	57.3599	7.88
chr3L	15231046	15231427	Tollo	4378	26.1893	5.17
chr3L	15234003	15234829	Tollo	1114	243.413	18.15
chr3L	15235174	15235910	Tollo	46	316.742	28.68
chr3L	15304067	15304737	CG7255	6	25.2621	5.13
chr3L	15329507	15329976	CR43247	2538	111.13	12.33
chr3L	15336238	15336861	Toll-6	91	57.7848	6.96
chr3L	15493857	15494306	CG12355	236	80.8129	10.06
chr3L	15500737	15501908	CG7272	131	148.657	10.98
chr3L	15507376	15508474	CG7841	587	316.742	23.19
chr3L	15510633	15511288	Eip71CD	75	316.742	25.68
chr3L	15511607	15512140	Eip71CD	16	207.132	15.61
chr3L	15514302	15514819	CG13454	2498	316.742	27.62
chr3L	15532148	15532846	CG43248	1040	316.742	19.58
chr3L	15533457	15536069	CrebA	66	316.742	29.22
chr3L	15540497	15541411	CrebA	5671	86.0194	10.23
chr3L	15554126	15554584	AGO2	282	18.1042	3.66
chr3L	15566018	15566986	dop	822	24.3352	4.44
chr3L	15588041	15588475	CG7656	53	93.6892	8.77
chr3L	15588732	15589395	RhoGAP71E	284	82.8228	7.91
chr3L	15593441	15593726	RhoGAP71E	4702	19.9542	4.36
chr3L	15619609	15620268	CG7372	1	233.347	20.04
chr3L	15625193	15626553	CR45977	3089	316.742	35.55
chr3L	15647032	15647363	comm3	593	18.7287	4.06
chr3L	15683643	15684947	pgant8	439	316.742	26.94
chr3L	15699506	15700444	comm2	91	246.936	21.51
chr3L	15728130	15728792	comm	6	316.742	20.02
chr3L	15729121	15729808	comm	1015	316.742	35.95

chr3L	15743424	15743684	CR45240	3860	14.0698	3.75
chr3L	15745264	15745683	CR45240	1858	19.5329	4.47
chr3L	15815183	15816430	fwe	660	200.718	15.59
chr3L	15824496	15824791	DCP2	445	10.9339	3.03
chr3L	15825423	15826483	DCP2	654	316.742	19.98
chr3L	15826904	15827162	dbo	105	10.3932	2.99
chr3L	15898813	15899189	pHCl	46	27.8702	4.95
chr3L	15920930	15921329	sff	3739	32.2995	6.46
chr3L	15924494	15925177	sff	55	148.112	14.71
chr3L	15975266	15975588	CG17026	72	19.4612	4.20
chr3L	15976168	15976489	CG17029	540	29.3863	4.97
chr3L	15976816	15977130	CG17029	31	36.0308	5.90
chr3L	15979379	15980569	CG17027	87	118.716	9.62
chr3L	15982645	15983281	brm	231	22.2506	4.18
chr3L	15982645	15983281	Arl1	34	22.2506	4.18
chr3L	15988226	15988702	Hip14	235	82.9844	9.19
chr3L	15996508	15996820	CG5830	8110	27.5528	5.09
chr3L	15997095	15997374	CG5830	7530	14.2819	3.80
chr3L	16000869	16002977	CG5830	3567	121.735	10.31
chr3L	16004120	16004771	CG5830	235	42.9314	5.59
chr3L	16011069	16011939	mib1	337	42.6855	5.61
chr3L	16023115	16023686	Notum	107	304.374	17.56
chr3L	16025953	16026550	Notum	2659	84.7575	10.22
chr3L	16026827	16027597	Notum	3888	48.851	7.31
chr3L	16028855	16029494	mir-4941	1991	142.137	15.48
chr3L	16041283	16041697	CR45889	843	15.0681	3.69
chr3L	16045755	16046022	Diap1	944	16.5691	3.84
chr3L	16046299	16047497	Diap1	125	235.22	14.66
chr3L	16049848	16051121	Diap1	106	155.032	12.77
chr3L	16051802	16053313	Mbs	600	316.742	28.79
chr3L	16113190	16113945	Taf4	28	15.2559	3.41
chr3L	16128480	16128793	Pgm	103	10.0813	2.70
chr3L	16149332	16151060	CG5151	223	35.8567	5.65
chr3L	16151573	16152022	CG5151	2491	14.3655	3.70
chr3L	16153134	16153674	CG5151	4078	79.0434	8.92
chr3L	16162075	16162479	CG5151	2425	23.7856	4.62
chr3L	16163961	16165057	CG5151	338	316.742	31.59
chr3L	16166158	16166673	CG5151	1720	61.9574	8.34
chr3L	16191009	16191296	CR45437	6033	8.39622	2.96
chr3L	16241191	16241769	l(3)72Dr	3720	64.6194	7.97
chr3L	16252218	16252646	CG13055	94	86.9274	9.95
chr3L	16254837	16255266	CG13054	2115	63.4027	9.43
chr3L	16257081	16257507	CG13054	100	29.8684	6.08
chr3L	16298819	16299432	CG4982	2284	21.8144	5.16
chr3L	16317753	16318106	Nplp3	90	31.0002	5.78
chr3L	16334806	16335165	CG13056	1683	27.3778	4.38
chr3L	16368170	16368556	CG4842	49	40.4034	7.17
chr3L	16377807	16378669	roq	502	52.2193	6.47
chr3L	16385346	16385902	CG4729	282	28.0227	4.58
chr3L	16392527	16392952	CG44836	940	8.5771	3.16
chr3L	16407987	16412025	fax	391	298.178	17.72
chr3L	16451938	16452385	dsx-c73A	208	61.8783	9.48
chr3L	16478753	16479069	aos	4720	12.0357	3.62
chr3L	16480677	16481155	aos	2649	33.0785	5.43
chr3L	16482925	16484510	aos	577	316.742	23.12
chr3L	16572059	16572692	Mipp1	11	316.742	25.60
chr3L	16588161	16588578	l(3)73Ah	140	10.3415	2.84
chr3L	16590854	16591121	tra	101	12.359	3.64
chr3L	16644564	16645008	Abl	3064	65.3291	8.37
chr3L	16645517	16646048	Abl	2113	46.1634	6.49
chr3L	16646892	16648046	Abl	157	97.6439	8.92
chr3L	16659899	16661864	Baldspot	823	237.216	15.74
chr3L	16680628	16680886	CG13032	6792	15.6905	3.83
chr3L	16710600	16710942	Lasp	914	17.9513	3.81
chr3L	16710600	16710942	CG43954	914	17.9513	3.81
chr3L	16711367	16711849	Lasp	19	42.1942	5.23
chr3L	16711367	16711849	CG43954	19	42.1942	5.23
chr3L	16713502	16714431	Dab	216	270.962	16.64
chr3L	16733325	16733677	CG9701	20	37.3852	6.33
chr3L	16745651	16746061	CG42852	9712	40.9662	6.60
chr3L	16746389	16746843	CG42852	8847	24.119	4.82
chr3L	16765614	16766350	Nrt	9	92.0459	9.71
chr3L	16780069	16780829	CG9705	66	30.5855	5.12
chr3L	16796431	16796858	CG9674	3529	56.1154	7.56
chr3L	16799956	16800776	CG9674	82	316.742	29.03

chr3L	16801413	16801733	CG9674	1367	29.2914	5.36
chr3L	16805812	16806808	nudC	1512	61.4595	7.16
chr3L	16807133	16807950	CG9674	30	73.3768	8.12
chr3L	16811821	16812250	CG13024	31	41.8221	6.68
chr3L	16835157	16835631	CG13024	6392	17.7908	4.22
chr3L	16845155	16845860	CG9715	34	145.297	11.72
chr3L	16853710	16854175	sina	116	6.11748	2.25
chr3L	16877030	16877661	CG32170	12	90.1613	9.24
chr3L	16886452	16886878	Lmpt	484	35.5108	6.39
chr3L	16890792	16891441	Lmpt	95	98.6901	8.91
chr3L	16910990	16911317	Lmpt	144	29.0461	5.98
chr3L	16915978	16916469	Lmpt	49	142.965	14.19
chr3L	16969577	16970212	Exn	120	256.641	21.29
chr3L	16985632	16987198	Exn	2	316.742	28.31
chr3L	16989441	16989751	Exn	2973	17.5552	3.78
chr3L	17003623	17005389	CG3764	2194	82.1817	8.17
chr3L	17009983	17010603	CG3764	2276	209.338	12.56
chr3L	17010922	17012718	CG3764	441	135.323	10.93
chr3L	17015750	17016031	CG6664	187	10.3936	2.95
chr3L	17031919	17032484	Ilp8	99	211.635	19.13
chr3L	17051241	17051510	rogdi	49	13.8108	3.27
chr3L	17147816	17148371	Rbp6	184	78.7529	9.56
chr3L	17148717	17149197	Rbp6	973	191.3	16.08
chr3L	17149662	17150377	Rbp6	2029	105.354	10.17
chr3L	17153269	17153563	Rbp6	5401	9.74428	3.38
chr3L	17211574	17212060	Rbp6	26416	89.0402	11.30
chr3L	17237928	17238685	Rbp6	228	154.827	15.51
chr3L	17379275	17379806	Cad74A	39	143.877	11.36
chr3L	17400536	17401720	noe	190	41.1495	6.30
chr3L	17415538	17416121	blot	10	101.376	10.52
chr3L	17421186	17422088	blot	41	316.742	29.87
chr3L	17432903	17433201	CG32174	200	9.07558	2.63
chr3L	17432903	17433201	frc	200	9.07558	2.63
chr3L	17438592	17439508	CG18265	81	70.8229	8.86
chr3L	17444628	17444924	CG18265	1734	12.7565	3.87
chr3L	17469948	17470537	CG7589	41	42.2125	6.58
chr3L	17474926	17475280	UQCR-Q	4	12.7356	2.98
chr3L	17475873	17476285	CG34250	220	14.4244	3.34
chr3L	17485832	17486087	CG6333	318	7.79053	2.50
chr3L	17486820	17487830	Oatp74D	191	316.742	23.73
chr3L	17499378	17501014	edin	5540	316.742	27.22
chr3L	17529647	17530245	Edc3	218	13.0314	3.13
chr3L	17529647	17530245	Nedd4	1	13.0314	3.13
chr3L	17529647	17530245	CR45165	123	13.0314	3.13
chr3L	17532828	17534084	Nedd4	50	226.326	14.08
chr3L	17534407	17534898	Nedd4	931	61.5213	7.47
chr3L	17551067	17551442	CG7542	1419	26.8073	5.15
chr3L	17552166	17552556	CG7542	2481	20.8397	3.94
chr3L	17557067	17557817	CycT	526	35.079	5.58
chr3L	17563234	17564317	snoRNA:Me28S-A576	2373	316.742	45.49
chr3L	17566419	17567433	snoRNA:Me28S-A576	564	146.226	12.41
chr3L	17569922	17570415	snoRNA:Me28S-A576	3982	79.2696	10.11
chr3L	17571899	17572708	Eip74EF	3976	316.742	23.03
chr3L	17573360	17574422	Eip74EF	2287	316.742	26.18
chr3L	17576088	17578493	Eip74EF	1266	316.742	31.84
chr3L	17584080	17585147	Eip74EF	8258	169.386	13.29
chr3L	17586198	17586813	Eip74EF	9954	34.15	6.10
chr3L	17588132	17588799	Eip74EF	11741	77.1631	9.82
chr3L	17590257	17591311	Eip74EF	14243	316.742	41.08
chr3L	17602102	17602379	Eip74EF	17041	20.758	4.53
chr3L	17603682	17604402	Eip74EF	15372	278.846	17.71
chr3L	17606611	17607412	Eip74EF	12318	316.742	30.87
chr3L	17611642	17612692	Eip74EF	7163	316.742	35.53
chr3L	17616674	17617458	Eip74EF	2198	269.404	17.15
chr3L	17618995	17619764	Eip74EF	129	316.742	38.67
chr3L	17638089	17638369	CG7497	4768	17.3238	3.74
chr3L	17644203	17645105	Pep	536	35.0192	4.74
chr3L	17653126	17654420	Krn	735	133.901	11.16
chr3L	17653126	17654420	Ndfip	735	133.901	11.16
chr3L	17658285	17659777	CG7484	4642	105.34	10.08
chr3L	17665085	17665858	snmRNA:641	3246	17.2947	3.83
chr3L	17667860	17669812	snmRNA:641	245	127.869	11.72
chr3L	17667860	17669812	Crtc	259	127.869	11.72
chr3L	17740358	17740888	CG7460	24	44.4712	7.32
chr3L	17841009	17841658	CG5577	109	316.742	30.73

chr3L	17843071	17843467	CG5567	428	10.52	3.24
chr3L	17846149	17846573	MED19	95	29.2213	4.30
chr3L	17846991	17847342	CG7430	110	11.4471	3.09
chr3L	17856516	17857654	CG5535	623	187.66	13.59
chr3L	17878799	17879221	CG7408	103	87.9623	9.62
chr3L	17895567	17896219	Tsp74F	61	178.412	14.25
chr3L	17907548	17907956	Prestin	43	40.7545	5.80
chr3L	17913960	17914492	CG5290	1666	316.742	23.85
chr3L	17922287	17922627	CG5290	9802	6.6678	2.67
chr3L	17927875	17928513	CR43174	5662	19.9502	4.56
chr3L	17929985	17930571	CR43174	3681	54.102	6.55
chr3L	17947173	17947767	CR45393	300	287.378	19.90
chr3L	17949109	17949489	CR45393	1570	23.548	5.32
chr3L	17956288	17957404	Eip75B	5146	132.1	10.68
chr3L	17957740	17959085	Eip75B	3480	144.932	12.23
chr3L	17961407	17962719	Eip75B	60	316.742	18.89
chr3L	17964660	17965650	Eip75B	3154	188.937	13.21
chr3L	17968534	17969110	Eip75B	2576	85.9738	9.40
chr3L	17970934	17971621	Eip75B	131	266.869	19.74
chr3L	17972367	17973467	Eip75B	1176	270.268	19.08
chr3L	17975649	17976019	Eip75B	4341	20.5555	4.78
chr3L	17999375	18000071	Eip75B	239	82.8388	11.52
chr3L	18005292	18005708	snoRNA:Me28S-A30	2388	72.6663	10.14
chr3L	18020129	18020464	Eip75B	5711	26.5164	5.32
chr3L	18020894	18021495	Eip75B	4955	147.725	14.50
chr3L	18021974	18023094	Eip75B	3177	176.141	16.08
chr3L	18029359	18029912	Eip75B	3611	281.968	20.28
chr3L	18032806	18034742	CR45922	5179	316.742	17.31
chr3L	18036135	18036712	CR45922	1881	45.9767	7.32
chr3L	18044236	18044914	CG42393	1882	62.1708	7.26
chr3L	18045553	18045940	CG42393	3064	13.6168	3.23
chr3L	18056949	18057416	Eip75B	2419	98.1772	10.01
chr3L	18061492	18062305	Eip75B	455	316.742	31.18
chr3L	18062761	18063715	Eip75B	1297	316.742	35.38
chr3L	18064643	18065225	Eip75B	252	316.742	25.35
chr3L	18066390	18066836	Eip75B	1892	47.5754	7.45
chr3L	18069819	18070452	CG44006	1	316.742	39.17
chr3L	18073586	18074076	CG44004	26	254.52	21.24
chr3L	18074837	18075331	CG44004	1195	187.094	17.00
chr3L	18077660	18078635	CR45392	421	86.3472	11.02
chr3L	18082029	18082411	CR43253	3184	8.93907	3.00
chr3L	18087079	18087613	CG34253	61	77.2515	8.63
chr3L	18105463	18106372	CG13698	423	277.529	18.30
chr3L	18114234	18114811	CG32195	193	95.4907	7.93
chr3L	18138971	18139673	geko	94	316.742	31.01
chr3L	18141641	18142112	geko	2371	15.5456	3.50
chr3L	18167075	18167421	CG13699	4706	43.9209	7.75
chr3L	18172395	18172680	CG13699	10009	18.3971	4.42
chr3L	18179669	18180007	hid	5824	16.7679	4.05
chr3L	18185392	18187199	hid	100	316.742	29.19
chr3L	18213858	18214260	CG7320	12574	55.5708	8.47
chr3L	18234033	18235029	CR45937	414	316.742	26.52
chr3L	18304279	18304972	grim	177	76.6982	9.62
chr3L	18398324	18398688	rpr	110	24.9044	5.56
chr3L	18439764	18440317	skl	90	137.289	14.53
chr3L	18539753	18540141	CG32198	12962	64.6289	9.83
chr3L	18559883	18560285	AstC-R1	210	39.3302	7.25
chr3L	18618163	18618487	CG13380	387	35.1247	6.04
chr3L	18618163	18618487	CG4174	387	35.1247	6.04
chr3L	18618163	18618487	CG13380	387	35.1247	6.04
chr3L	18618163	18618487	CG4174	387	35.1247	6.04
chr3L	18621040	18622170	not	788	316.742	22.04
chr3L	18625872	18626148	MYPT-75D	47	37.1113	5.35
chr3L	18627520	18628020	MYPT-75D	1938	8.27928	2.93
chr3L	18628488	18628870	MYPT-75D	2747	24.5199	5.05
chr3L	18633070	18633566	MYPT-75D	7380	66.084	9.63
chr3L	18640161	18641638	MYPT-75D	553	316.742	40.00
chr3L	18672334	18673335	Capr	662	39.9071	6.08
chr3L	18748637	18748905	Atg3	74	15.9198	3.41
chr3L	18753738	18754211	CR45938	118	230.328	19.36
chr3L	18756792	18757685	CR45938	3196	316.742	24.62
chr3L	18758847	18759781	CR45938	5227	277.626	15.43
chr3L	18760612	18761153	ftz-f1	4147	160.34	13.99
chr3L	18765432	18766252	ftz-f1	195	316.742	24.73
chr3L	18771124	18772245	ftz-f1	5891	316.742	25.86

chr3L	18781582	18782092	ftz-f1	15943	19.6611	3.88
chr3L	18783618	18784262	ftz-f1	17447	20.3193	4.97
chr3L	18787861	18788155	ftz-f1	13285	26.0364	5.07
chr3L	18790832	18791792	ftz-f1	9797	107.067	8.84
chr3L	18792671	18793392	ftz-f1	8331	316.742	28.10
chr3L	18795195	18796103	ftz-f1	5453	72.733	9.54
chr3L	18799952	18801709	ftz-f1	864	316.742	18.74
chr3L	18799952	18801709	CR45939	867	316.742	18.74
chr3L	18812029	18812348	CG14073	8414	14.4561	3.57
chr3L	18813928	18814684	CG14073	6248	120.782	10.51
chr3L	18817310	18817775	CG14073	3100	24.6114	4.43
chr3L	18819710	18820800	CG14073	286	43.7038	5.65
chr3L	18822490	18823163	Cat	248	191.664	13.02
chr3L	18834678	18835022	Indy	5670	50.2466	7.90
chr3L	18840247	18840884	Indy	96	244.426	17.81
chr3L	18845442	18846573	Indy	51	289.991	19.05
chr3L	18848353	18848710	Indy	2274	82.2316	10.96
chr3L	18867470	18868033	Dysb	9	315.446	22.17
chr3L	18890102	18891443	CG3961	549	316.742	27.32
chr3L	18895116	18895530	snoRNA:Psi28S-2566	1157	43.0266	6.40
chr3L	18896330	18896813	snoRNA:Psi28S-2566	101	76.8462	9.12
chr3L	18898025	18898428	CG3902	70	37.4321	5.12
chr3L	18945916	18946247	CG32204	8	26.6186	4.91
chr3L	18946954	18947275	CG32204	1084	67.689	9.72
chr3L	18949112	18949546	CG6836	1320	18.2556	3.86
chr3L	18994531	18994967	CG18135	849	11.9688	3.15
chr3L	18995596	18996068	CG18135	25	40.3367	5.87
chr3L	18996370	18997112	CG18135	1012	152.39	10.14
chr3L	19015831	19017150	CG18136	13125	166.999	13.67
chr3L	19044849	19045748	nkd	30	316.742	20.53
chr3L	19062619	19063513	CG3797	375	22.4999	3.56
chr3L	19062619	19063513	CG6812	27	22.4999	3.56
chr3L	19072203	19072749	Mkp3	6	58.7158	8.45
chr3L	19081358	19081748	Mkp3	4875	76.9565	8.95
chr3L	19085989	19087713	Mkp3	52	316.742	24.47
chr3L	19091506	19092195	MESR6	760	61.4394	6.35
chr3L	19170298	19172457	fz2	1197	316.742	46.85
chr3L	19177888	19178202	CR45941	6728	9.87221	3.24
chr3L	19218605	19219610	CR43893	10343	17.289	4.24
chr3L	19230673	19231067	CR43893	1795	40.905	6.96
chr3L	19234450	19234750	fz2	764	21.3369	4.34
chr3L	19235129	19235648	fz2	33	71.4745	7.15
chr3L	19237934	19238205	CG33647	1693	13.1054	3.64
chr3L	19260243	19260716	nes	226	12.0842	3.03
chr3L	19292072	19293649	Gbs-76A	111	316.742	23.59
chr3L	19294320	19295300	fal	223	128.959	10.51
chr3L	19294320	19295300	CR46048	209	128.959	10.51
chr3L	19412772	19413305	CG32206	117	112.992	12.17
chr3L	19432132	19432654	CR45732	8812	121.374	13.06
chr3L	19482019	19482315	brv1	4323	49.9213	6.45
chr3L	19500145	19500977	CG9451	514	17.6934	4.13
chr3L	19528260	19529168	Cpr76Bd	3978	41.7058	6.66
chr3L	19562091	19563790	CR45916	301	315.49	19.99
chr3L	19566066	19566700	mir-9a	1343	111.575	11.79
chr3L	19580745	19581094	Shal	3668	35.153	5.66
chr3L	19583478	19584738	Shal	55	26.6186	4.91
chr3L	19605944	19606616	lush	101	54.4436	6.95
chr3L	19605944	19606616	CG9372	16	54.4436	6.95
chr3L	19618190	19618934	l(3)76BDm	235	16.7281	3.68
chr3L	19618190	19618934	asf1	607	16.7281	3.68
chr3L	19632693	19633402	wnd	24	316.742	27.39
chr3L	19635068	19636127	wnd	55	316.742	33.18
chr3L	19649673	19650031	Oat	34	24.9044	5.56
chr3L	19663611	19664072	tey	146	36.1065	5.84
chr3L	19682888	19683372	CR44678	2963	30.9021	4.99
chr3L	19694927	19695452	verm	33	22.9723	4.66
chr3L	19707436	19708187	serp	62	23.7842	4.45
chr3L	19787670	19788042	Gyc76C	3192	49.3026	7.60
chr3L	19787670	19788042	CG42637	3192	49.3026	7.60
chr3L	19789956	19792297	Gyc76C	549	316.742	33.04
chr3L	19789956	19792297	CG42637	549	316.742	33.04
chr3L	19792754	19794505	Gyc76C	120	142.87	12.03
chr3L	19792754	19794505	CG42637	120	142.87	12.03
chr3L	19792754	19794505	SREBP	245	142.87	12.03
chr3L	19813150	19813580	Cyp305a1	4	51.3979	6.61

chr3L	19815517	19815859	cyc	275	20.8343	3.56
chr3L	19818700	19819231	Deaf1	167	46.4249	6.53
chr3L	19833168	19833623	trc	902	87.1435	9.13
chr3L	19847908	19848319	Papss	28	35.7647	6.85
chr3L	19861307	19861626	CR45433	28	13.0311	3.32
chr3L	19870519	19871203	Rab8	270	55.1986	6.78
chr3L	19870519	19871203	Usp32	330	55.1986	6.78
chr3L	19871648	19871999	Usp32	661	12.1207	3.00
chr3L	19894918	19897921	CR32218	118	316.742	29.57
chr3L	19899290	19899716	CR32218	3202	12.3895	3.01
chr3L	19900223	19900890	CR32218	4312	63.743	7.14
chr3L	19903407	19903690	CR43889	5789	26.7863	4.64
chr3L	19904658	19905064	CR43889	4448	32.4941	4.91
chr3L	19905626	19906348	CR43889	3566	32.737	5.30
chr3L	19906728	19907696	CR43889	2212	211.032	16.29
chr3L	19908112	19910491	CR43889	398	316.742	20.67
chr3L	19908112	19910491	Su(Tpl)	444	316.742	20.67
chr3L	19908112	19910491	Mi-2	1249	316.742	20.67
chr3L	19908112	19910491	Prp3	1333	316.742	20.67
chr3L	19929026	19929490	RhoGDI	559	37.0387	4.97
chr3L	19929830	19930390	RhoGDI	1395	169.412	15.34
chr3L	19932992	19933361	CR45678	570	34.4769	4.76
chr3L	19938911	19939465	Ac76E	114	316.742	22.48
chr3L	20002710	20003100	CG7668	190	41.9034	6.20
chr3L	20006311	20006648	kug	154	25.3684	4.87
chr3L	20088873	20089197	sNPF-R	65	9.74428	3.38
chr3L	20103487	20105540	CG14186	522	60.8395	8.24
chr3L	20103487	20105540	CR44679	153	60.8395	8.24
chr3L	20185083	20185408	CG42674	148	17.8883	4.06
chr3L	20185924	20187040	CG42674	1344	248.992	16.12
chr3L	20197664	20198636	CG42674	51	266.533	15.93
chr3L	20230408	20230687	CG13813	1192	10.0788	2.82
chr3L	20241343	20241601	CG17233	13	9.56822	2.82
chr3L	20241343	20241601	CG6951	57	9.56822	2.82
chr3L	20241343	20241601	Clc	57	9.56822	2.82
chr3L	20247572	20248354	rdgC	171	100.956	11.82
chr3L	20295028	20295499	gogo	164	68.2663	8.32
chr3L	20302123	20302689	Pex23	127	10.0522	3.04
chr3L	20302123	20302689	CG6597	162	10.0522	3.04
chr3L	20306087	20306530	CG32225	71	56.8284	8.18
chr3L	20316802	20317737	Spn77Ba	70	316.742	21.27
chr3L	20316802	20317737	CR43875	164	316.742	21.27
chr3L	20341237	20341508	Spn77Bc	1301	17.2351	4.22
chr3L	20348263	20349837	eRF1	267	316.742	19.61
chr3L	20353418	20354362	CG5618	122	87.6548	8.63
chr3L	20366852	20367578	in	84	164.23	12.07
chr3L	20376130	20376577	RhoBTB	16	10.029	2.85
chr3L	20385732	20386367	fbl	260	64.0799	7.26
chr3L	20397628	20399823	CR45677	2934	191.225	13.31
chr3L	20400089	20400579	trbl	1356	32.6458	5.71
chr3L	20401083	20402072	trbl	116	316.742	42.00
chr3L	20439673	20440004	CG11796	27	23.7399	4.88
chr3L	20439673	20440004	CR43943	108	23.7399	4.88
chr3L	20473036	20473497	CG32428	21	20.1186	4.27
chr3L	20493750	20494027	ZnT77C	397	27.638	5.48
chr3L	20497316	20497771	CG32425	203	23.5033	4.51
chr3L	20507983	20508668	CG32425	1458	117.685	10.43
chr3L	20509211	20510019	CG32425	90	103.374	12.72
chr3L	20517321	20517829	CG5059	274	143.649	12.76
chr3L	20518449	20519461	CG5059	761	23.7434	4.28
chr3L	20525911	20526212	CG4825	33	6.98208	2.53
chr3L	20540177	20540748	Rcd2	42	316.742	21.21
chr3L	20620403	20622427	knrl	1093	316.742	26.37
chr3L	20674922	20675847	kni	19378	27.1135	4.87
chr3L	20695154	20695929	kni	152	267.406	21.41
chr3L	20719038	20719446	cmpy	11	36.6468	6.95
chr3L	20729940	20730897	CG13252	148	316.742	33.92
chr3L	20733813	20734231	CG13252	3430	47.1603	7.27
chr3L	20769304	20769605	CG4074	29	11.5443	3.00
chr3L	20769304	20769605	CG4186	74	11.5443	3.00
chr3L	20771351	20771876	CG4042	96	9.50205	2.81
chr3L	20771351	20771876	Pitslre	76	9.50205	2.81
chr3L	20784371	20786296	CG11399	752	316.742	35.67
chr3L	20808985	20809340	CG3634	7	9.55401	2.78
chr3L	20837826	20838789	CG13255	234	77.6819	7.84

chr3L	20837826	20838789	CR45943	567	77.6819	7.84
chr3L	20884749	20885529	CG32432	7	21.0402	4.52
chr3L	20933158	20933463	CG10589	5472	9.74428	3.38
chr3L	20948981	20950786	fng	150	316.742	43.36
chr3L	20948981	20950786	CR45243	1121	316.742	43.36
chr3L	21007576	21010180	scaRNA:PsiU6-40	2516	316.742	35.13
chr3L	21010877	21012239	scaRNA:PsiU6-40	4646	316.742	24.27
chr3L	21014415	21014966	scaRNA:PsiU6-40	8281	162.841	17.23
chr3L	21023394	21023881	skd	2657	310.689	27.09
chr3L	21024706	21027511	skd	211	316.742	38.60
chr3L	21027776	21028310	CG10585	111	61.8142	7.07
chr3L	21034418	21034781	siz	73	35.4504	5.04
chr3L	21042192	21043034	siz	5814	55.0806	7.21
chr3L	21043526	21043909	siz	4413	12.0088	3.55
chr3L	21047203	21048190	siz	292	88.4047	9.80
chr3L	21049367	21050038	siz	1441	118.202	10.86
chr3L	21055897	21056200	siz	2735	9.26653	2.67
chr3L	21056610	21059563	siz	15	316.742	38.25
chr3L	21060028	21060637	siz	1522	182.83	12.73
chr3L	21063619	21063951	CG43072	2074	31.8918	5.04
chr3L	21150649	21151075	Ac78C	233	38.8308	7.13
chr3L	21172702	21173405	chb	30	92.0847	9.53
chr3L	21172702	21173405	AsnS	235	92.0847	9.53
chr3L	21175733	21176810	chb	215	27.3656	4.21
chr3L	21185704	21186916	CG42337	6688	72.6134	9.87
chr3L	21191772	21192465	CG42337	1314	52.9192	7.43
chr3L	21193136	21193992	CG42337	291	99.2228	10.17
chr3L	21202405	21203366	CG33054	43	316.742	19.96
chr3L	21202405	21203366	CG33056	43	316.742	19.96
chr3L	21202405	21203366	CG10512	6	316.742	19.96
chr3L	21202405	21203366	CG33056	307	316.742	19.96
chr3L	21205265	21205733	CG10512	23	16.3449	3.91
chr3L	21210028	21210963	CG10510	145	36.6054	5.39
chr3L	21210028	21210963	CG10508	15	36.6054	5.39
chr3L	21219213	21220348	CG43219	975	91.4418	8.92
chr3L	21239777	21240612	Eip78C	252	316.742	29.17
chr3L	21276637	21277383	AcCoAS	786	252.993	19.91
chr3L	21279720	21280598	AcCoAS	1	160.692	10.62
chr3L	21285684	21286346	CG12974	133	44.4803	5.70
chr3L	21285684	21286346	pzg	3	44.4803	5.70
chr3L	21304737	21305018	CG7632	134	10.7193	2.91
chr3L	21317773	21318100	Pc	36	6.82529	2.28
chr3L	21318530	21318889	CR45674	36	35.6154	5.23
chr3L	21318530	21318889	Rab26	87	35.6154	5.23
chr3L	21319267	21319944	Rab26	523	54.2892	7.33
chr3L	21322175	21322465	Rab26	69	14.3285	3.93
chr3L	21346689	21347390	ebd2	124	38.1633	5.64
chr3L	21381635	21382774	rgn	40	316.742	23.67
chr3L	21433319	21434374	Aef1	306	35.8731	5.22
chr3L	21476333	21477268	croc	464	316.742	30.49
chr3L	21479450	21480147	croc	3321	274.867	20.85
chr3L	21483939	21484504	Neu2	6440	75.9772	8.88
chr3L	21485038	21485831	Neu2	5087	316.742	22.22
chr3L	21497131	21497501	Hr78	148	13.8792	3.28
chr3L	21498806	21499586	CR43933	1473	37.0454	6.63
chr3L	21507468	21508199	Glg1	462	23.3677	4.17
chr3L	21507468	21508199	M6	149	23.3677	4.17
chr3L	21509608	21509968	M6	99	45.2998	7.39
chr3L	21516218	21516541	Cdk12	10	13.9818	3.12
chr3L	21536966	21537253	Rpn10	18	22.835	4.39
chr3L	21584723	21585125	TfAP-2	1935	86.456	11.17
chr3L	21598266	21598721	TfAP-2	351	28.119	5.96
chr3L	21640744	21641129	Atox1	111	16.3449	3.91
chr3L	21644586	21645155	CG43980	139	62.4965	8.76
chr3L	21738238	21738610	CG14567	1	59.2347	8.66
chr3L	21769406	21770969	CG7370	764	262.794	15.43
chr3L	21769406	21770969	Syn1	44	262.794	15.43
chr3L	21807928	21808503	eg	453	129.968	10.48
chr3L	21818716	21819215	CycH	3055	42.8436	7.07
chr3L	21839998	21841457	P5CDh1	404	197.046	13.58
chr3L	21843345	21843726	CG14563	1133	41.5677	6.46
chr3L	21844424	21844956	mub	14	16.9779	4.04
chr3L	21879211	21880910	mub	1068	316.742	26.03
chr3L	21883057	21883519	mub	3615	15.0422	3.64
chr3L	21884045	21884395	CR43878	2818	22.2894	4.46

chr3L	21886186	21887427	CR43878	33	199.307	14.14
chr3L	21889602	21890234	CR43878	2929	29.3938	4.54
chr3L	21890507	21891384	CR43878	3803	148.882	12.80
chr3L	21892335	21893021	CR43878	5540	82.2486	8.81
chr3L	21954777	21955423	CG7458	47	316.742	23.38
chr3L	22034835	22035100	Oct-TyrR	13	16.9306	4.44
chr3L	22070675	22070980	CG7139	1052	20.4581	4.22
chr3L	22075955	22076292	RpLP0	41	14.9633	3.32
chr3L	22132740	22133147	olf413	3168	75.4293	10.20
chr3L	22134052	22135257	olf413	1362	153.699	12.59
chr3L	22135752	22136379	olf413	47	316.742	30.22
chr3L	22267189	22267991	Csp	112	24.8876	4.42
chr3L	22273788	22274122	Srpk79D	42	18.8111	4.13
chr3L	22276365	22276707	CR45960	866	13.0895	3.76
chr3L	22346706	22347083	CR45963	1534	24.6583	5.32
chr3L	22407525	22408570	CR45962	408	316.742	37.18
chr3L	22407525	22408570	Ten-m	92	316.742	37.18
chr3L	22460819	22461487	laza	49	95.7768	11.34
chr3L	22465768	22466907	CG11438	540	45.9767	7.32
chr3L	22470424	22471090	CG11426	61	27.0891	4.93
chr3L	22474233	22474507	CR45382	64	15.2586	3.45
chr3L	22571696	22572857	Trxr-2	21713	45.5576	6.62
chr3L	22642520	22642827	CR45381	1234	19.9701	4.36
chr3L	22720513	22721171	mael	202	25.7575	5.24
chr3L	22727843	22728440	CG11367	5	316.742	18.43
chr3L	22738203	22738754	CG11241	57	214.394	17.56
chr3L	22738203	22738754	CR45163	230	214.394	17.56
chr3L	22741165	22742549	l(3)04053	435	23.427	4.00
chr3L	22741165	22742549	CG7369	31	23.427	4.00
chr3L	22773288	22774212	SPoCk	21	173.495	12.70
chr3L	22822338	22825379	jim	107	316.742	40.16
chr3L	22830576	22830844	jim	2684	12.2753	3.56
chr3L	22835000	22836151	jim	351	316.742	40.48
chr3L	22837523	22838913	CG45428	794	316.742	28.86
chr3L	22839224	22840313	CR45659	468	316.742	24.82
chr3L	22840772	22841155	CR45659	84	17.6934	3.86
chr3L	22851033	22852186	CR43426	747	316.742	32.52
chr3L	22863698	22863988	CG33169	51	14.9859	3.32
chr3L	22864655	22865103	CG33170	10	64.5611	6.41
chr3L	22870979	22872373	Arf79F	341	60.0756	7.17
chr3L	22870979	22872373	CR45969	253	60.0756	7.17
chr3L	22870979	22872373	Arf79F	13	60.0756	7.17
chr3L	22870979	22872373	CG11109	134	60.0756	7.17
chr3L	22870979	22872373	Arf79F	166	60.0756	7.17
chr3L	22870979	22872373	CG11109	192	60.0756	7.17
chr3L	22878641	22879127	Chro	34	35.001	5.37
chr3L	22878641	22879127	Ssl1	286	35.001	5.37
chr3L	22880807	22881291	slif	57	32.272	4.55
chr3L	22894706	22895208	CR44688	1136	20.5586	3.94
chr3L	22910173	22910654	CG12768	68	101.836	11.25
chr3L	22945248	22945723	Mes2	174	78.8346	9.27
chr3L	22948317	22948994	Mes2	2558	39.3585	5.98
chr3L	22960270	22961108	CR45758	373	90.0378	11.79
chr3L	22998180	22998562	nrm	9	23.4066	4.74
chr3L	24723745	24724078	scro	222	27.5095	4.76
chr3L	26012525	26012890	CG40178	1005	64.8389	8.58
chr3R	4077617	4078125	CG42402	87	114.661	10.53
chr3R	4083619	4084149	CG42402	219	54.498	8.84
chr3R	4130120	4130797	Gfat1	142	87.318	11.35
chr3R	4174259	4175927	CG12581	81	316.742	34.88
chr3R	4177276	4177753	CG12581	2915	12.2088	3.24
chr3R	4186709	4187645	Dsk	3382	39.904	5.27
chr3R	4196975	4197577	beta-Man	42	199.079	18.40
chr3R	4235003	4235333	Gel	45	32.4034	4.61
chr3R	4236079	4237199	Gel	77	243.549	15.36
chr3R	4280047	4280423	CR45597	199	13.4179	3.66
chr3R	4280047	4280423	cpx	36	13.4179	3.66
chr3R	4281052	4282015	cpx	333	36.371	5.54
chr3R	4284486	4284983	cpx	2181	23.9095	5.23
chr3R	4291248	4292036	cpx	200	53.0987	6.74
chr3R	4307484	4308246	MP1	91	316.742	28.73
chr3R	4311509	4311764	CG9791	30	8.38584	2.62
chr3R	4319958	4320222	CG9795	364	7.54905	2.59
chr3R	4337617	4338050	Skp2	122	32.9664	4.73
chr3R	4337617	4338050	CG1103	42	32.9664	4.73

chr3R	4359623	4361281	CG1090	1136	271.197	17.69
chr3R	4378606	4379467	CG11739	52	316.742	24.77
chr3R	4387586	4387894	tub	4	16.6471	3.42
chr3R	4402962	4404004	lost	632	62.5136	7.17
chr3R	4419405	4419856	CG32944	74	66.3745	8.18
chr3R	4433171	4434058	eIF3-S10	232	208.397	14.44
chr3R	4433171	4434058	CG1074	301	208.397	14.44
chr3R	4449275	4449659	CG31522	1395	27.7117	5.42
chr3R	4452260	4454093	CR43629	454	316.742	26.52
chr3R	4452260	4454093	CG31522	75	316.742	26.52
chr3R	4452260	4454093	CR43629	159	316.742	26.52
chr3R	4468867	4469204	CG31523	417	14.6993	3.39
chr3R	4470231	4470592	CG31523	882	17.6452	3.80
chr3R	4471064	4471654	CG31523	16	52.2481	6.28
chr3R	4478136	4479300	CG31523	453	297.895	21.46
chr3R	4599662	4600018	CR44317	65	40.6205	5.98
chr3R	4612611	4612972	5-HT2A	46	40.7545	5.80
chr3R	4649307	4650273	Karybeta3	683	44.6372	5.96
chr3R	4654736	4655269	spartin	115	39.3957	4.75
chr3R	4659370	4660310	CG43427	48	87.2818	7.30
chr3R	4731727	4732108	Nep2	1	32.1019	5.13
chr3R	4738732	4740992	tacc	75	316.742	36.36
chr3R	4746259	4746761	tacc	136	87.1611	10.67
chr3R	4803060	4803798	ctrip	519	316.742	26.70
chr3R	4804271	4804629	ctrip	1609	19.849	4.06
chr3R	4805989	4807216	ctrip	86	316.742	18.21
chr3R	4807575	4808092	ctrip	1373	258.955	19.33
chr3R	4811814	4812163	ctrip	61	12.6592	3.03
chr3R	4811814	4812163	Hus1-like	61	12.6592	3.03
chr3R	4811814	4812163	ctrip	61	12.6592	3.03
chr3R	4811814	4812163	Hus1-like	61	12.6592	3.03
chr3R	4820991	4821425	CG14657	199	42.1634	5.54
chr3R	4835284	4835758	CG14659	4215	72.9341	8.05
chr3R	4852208	4852944	opa	324	294.633	16.05
chr3R	4910413	4911449	Cdep	514	316.742	17.03
chr3R	4951707	4952801	Ubc6	700	19.163	4.03
chr3R	4955082	4955764	CG14661	205	292.769	23.71
chr3R	4999688	5000589	CG2022	10618	33.5632	5.74
chr3R	5021179	5022015	CR45585	5880	116.36	12.40
chr3R	5042789	5043473	CR43635	2939	58.6731	7.33
chr3R	5074746	5075109	corto	11778	14.3799	3.76
chr3R	5081640	5082066	corto	4828	35.0776	4.89
chr3R	5086414	5088382	corto	148	316.742	49.28
chr3R	5150610	5150917	dpr16	130	18.347	4.21
chr3R	5215679	5217534	cno	1025	316.742	22.99
chr3R	5221621	5222205	CG2604	1491	26.3541	4.92
chr3R	5223093	5223431	CG2604	25	28.8448	4.56
chr3R	5231639	5232427	Kat60	82	12.8951	3.07
chr3R	5251136	5251841	CG12163	174	47.9443	6.88
chr3R	5257038	5257748	Hph	496	43.3581	6.38
chr3R	5263891	5265239	CR45793	398	316.742	20.57
chr3R	5263891	5265239	Hph	29	316.742	20.57
chr3R	5279712	5280233	mtd	113	114.93	12.03
chr3R	5282508	5283634	mtd	303	316.742	26.64
chr3R	5287144	5287613	mtd	84	9.55719	2.79
chr3R	5315253	5315612	mtd	7397	12.1831	3.24
chr3R	5319663	5320013	mtd	4091	13.0388	3.47
chr3R	5346663	5347087	mtd	3143	35.3551	5.49
chr3R	5349755	5350226	mtd	56	64.7608	8.67
chr3R	5350491	5350963	mtd	325	54.9599	7.11
chr3R	5361639	5362179	Cerk	44	22.559	4.39
chr3R	5362938	5363857	Cerk	1596	14.9793	3.36
chr3R	5367508	5368390	Cerk	27	27.6062	4.96
chr3R	5371673	5372003	RpII18	55	16.5691	3.84
chr3R	5413557	5414641	CG14669	173	188.858	19.62
chr3R	5464456	5464716	PEK	98	8.29734	2.80
chr3R	5508387	5508712	CG31547	5827	28.8275	6.03
chr3R	5517045	5518122	Itp-r83A	151	70.7791	7.31
chr3R	5518591	5519151	Itp-r83A	1728	222.307	14.95
chr3R	5519736	5520159	Itp-r83A	2825	23.0437	4.81
chr3R	5523017	5523622	CR43845	325	37.804	6.32
chr3R	5523017	5523622	Nmdar1	117	37.804	6.32
chr3R	5547107	5547919	CG2519	118	61.3134	6.93
chr3R	5564838	5565406	CG12746	84	32.2279	4.93
chr3R	5571124	5571437	CRMP	211	9.92322	2.82

chr3R	5587985	5588364	CG2926	262	10.5759	3.11
chr3R	5591832	5592457	Vha26	603	10.8707	2.87
chr3R	5599096	5601172	kra	30	132.906	10.16
chr3R	5612299	5613173	Rga	242	111.747	9.27
chr3R	5623153	5624578	Spec2	173	58.3215	6.28
chr3R	5623153	5624578	CG2911	173	58.3215	6.28
chr3R	5623153	5624578	RpL13A	521	58.3215	6.28
chr3R	5647654	5649147	MTA1-like	201	25.287	4.49
chr3R	5647654	5649147	Sec23	527	25.287	4.49
chr3R	5654601	5654981	elm	41	16.9268	3.46
chr3R	5659064	5659935	CR44093	235	23.5664	4.11
chr3R	5659064	5659935	CG2182	671	23.5664	4.11
chr3R	5668945	5671538	Xe7	916	73.7602	7.14
chr3R	5668945	5671538	Atg17	538	73.7602	7.14
chr3R	5672376	5673287	Atg17	2945	314.503	21.09
chr3R	5685874	5686281	plx	160	7.64017	2.49
chr3R	5701053	5701497	plx	1354	60.4003	7.96
chr3R	5742366	5742890	Sym	253	23.4605	4.45
chr3R	5742366	5742890	Madm	220	23.4605	4.45
chr3R	5771785	5772266	CG2082	192	50.2304	7.21
chr3R	5778903	5779504	jagn	23	20.8953	4.14
chr3R	5781436	5782167	kat-60L1	99	67.0045	9.70
chr3R	5801734	5802489	CG11000	38	27.7809	5.20
chr3R	5812404	5812728	CG42675	1022	32.4734	5.84
chr3R	5833841	5834416	CG1208	749	15.6368	3.71
chr3R	5861930	5862383	CG2017	206	11.5184	3.15
chr3R	5959409	5959810	CG34113	44	26.1148	5.67
chr3R	6007335	6008719	Rm62	148	174.979	12.89
chr3R	6097158	6097424	Gasp	3575	10.3286	3.06
chr3R	6100809	6101286	Gasp	191	40.5391	6.19
chr3R	6112555	6112956	Obp83g	490	23.8339	5.05
chr3R	6198577	6198926	Osi24	43	20.9461	5.04
chr3R	6343948	6344271	CG17919	115	27.8114	5.52
chr3R	6350971	6351726	Pak	503	43.5188	5.79
chr3R	6353238	6354132	Pak	634	66.8543	8.65
chr3R	6373375	6373826	CRAT	18	107.876	10.55
chr3R	6382964	6383600	godzilla	239	14.9876	3.68
chr3R	6405157	6406021	Dmtn	423	164.05	11.04
chr3R	6406694	6408051	gpp	846	268.003	15.40
chr3R	6413861	6414827	CR44017	5041	30.3392	5.57
chr3R	6419872	6420622	CR44017	10472	35.2316	5.82
chr3R	6421600	6422166	CR44017	12380	50.9213	6.99
chr3R	6424124	6424473	CR44017	14735	16.7357	4.47
chr3R	6430133	6430487	CR44017	20745	10.3226	3.48
chr3R	6435307	6435562	CG9727	20949	9.04704	2.98
chr3R	6474481	6474823	CG15186	3671	43.3746	5.96
chr3R	6593171	6593747	dpr11	164	28.9409	6.06
chr3R	6646751	6647132	Taf1	51	17.7837	3.56
chr3R	6678457	6678866	lab	84	55.1862	7.40
chr3R	6741568	6742399	pb	274	55.3146	7.02
chr3R	6791328	6792065	Dfd	180	316.742	22.71
chr3R	6815670	6815980	CR42651	561	10.9113	3.57
chr3R	6848404	6849095	Scr	314	169.267	16.73
chr3R	6849865	6850329	Scr	137	61.712	8.40
chr3R	6892971	6893815	CR45559	126	191.477	13.43
chr3R	6912230	6913231	CR43252	8256	51.6622	6.80
chr3R	6930391	6930892	Antp	2187	83.0015	10.24
chr3R	6958515	6958915	Antp	25929	21.1799	4.42
chr3R	6966703	6967068	Antp	32334	27.8702	4.95
chr3R	7000505	7001385	CR45899	524	313.678	20.10
chr3R	7037089	7037973	CR44933	390	270.82	18.91
chr3R	7067042	7067402	CG1105	194	6.75781	2.45
chr3R	7067042	7067402	CG1965	70	6.75781	2.45
chr3R	7075252	7076072	CR45908	208	24.4862	4.17
chr3R	7076122	7076866	CG1943	7	8.25322	2.53
chr3R	7086341	7087467	alphaTub84B	40	117.939	9.25
chr3R	7089519	7090630	alphaTub84B	3844	38.4648	5.76
chr3R	7103368	7103674	Alh	4883	30.2699	5.80
chr3R	7107480	7110283	Alh	55	316.742	32.79
chr3R	7107480	7110283	CR45906	1532	316.742	32.79
chr3R	7122177	7122448	Alh	1365	9.59059	2.59
chr3R	7122817	7124804	Alh	200	113.726	9.37
chr3R	7122817	7124804	CG10098	609	113.726	9.37
chr3R	7129323	7129763	CG31248	27	19.2889	4.06
chr3R	7162354	7163257	sas	39	316.742	39.13

chr3R	7162354	7163257	CR45909	168	316.742	39.13
chr3R	7167949	7168451	CR45909	5357	35.9426	6.12
chr3R	7186376	7187696	CG10055	578	316.742	22.94
chr3R	7186376	7187696	lap	258	316.742	22.94
chr3R	7187967	7188339	lap	1152	45.9589	6.53
chr3R	7223956	7224385	CG14608	299	32.2995	6.46
chr3R	7265749	7266062	Syt4	64	8.34778	2.77
chr3R	7266427	7266720	Syt4	447	14.4282	3.96
chr3R	7306631	7307252	CG42544	2340	105.341	9.82
chr3R	7309588	7310107	rn	219	136.571	15.30
chr3R	7310528	7311027	rn	1121	150.897	16.13
chr3R	7312879	7313593	CR44334	408	316.742	21.73
chr3R	7394018	7394825	CG42650	2830	55.25	7.87
chr3R	7474534	7474978	snRNA:7SK	240	58.3215	6.28
chr3R	7491226	7491961	alpha-Est10	65	316.742	34.04
chr3R	7506535	7506995	alpha-Est9	1650	116.793	12.73
chr3R	7508488	7508766	alpha-Est9	3593	13.589	3.44
chr3R	7512882	7513222	alpha-Est8	2637	12.2876	3.50
chr3R	7515324	7516164	alpha-Est8	29	96.2573	9.36
chr3R	7523488	7524160	alpha-Est7	37	50.0299	6.08
chr3R	7536147	7536497	alpha-Est5	53	26.2428	4.55
chr3R	7541054	7541506	alpha-Est3	19	20.6741	4.27
chr3R	7548858	7549356	alpha-Est1	103	23.2998	4.31
chr3R	7639277	7639806	Nlg3	131	115.391	11.54
chr3R	7749818	7750361	pyd3	38	266.354	18.89
chr3R	7753506	7753930	pyd3	3637	60.3113	7.51
chr3R	7760728	7761457	Sp7	26	316.742	29.77
chr3R	7793731	7794324	CG2641	47	316.742	20.44
chr3R	7809485	7810653	CG2993	315	45.4509	5.32
chr3R	7809485	7810653	sgll	202	45.4509	5.32
chr3R	7813694	7813974	CG34384	534	22.4092	4.48
chr3R	7821386	7822168	CG34384	6	20.6741	4.27
chr3R	7884151	7884764	CR46127	142	49.303	7.70
chr3R	7886078	7887137	CG17816	61	101.939	10.31
chr3R	7886078	7887137	CG10092	250	101.939	10.31
chr3R	7907074	7907691	Taf7	227	12.5892	3.28
chr3R	7907074	7907691	EMC1	196	12.5892	3.28
chr3R	7907074	7907691	Taf7	78	12.5892	3.28
chr3R	7913017	7913332	CG2846	74	13.8647	3.36
chr3R	7947123	7947872	dsx	15319	316.742	25.57
chr3R	7962515	7964035	dsx	206	141.387	12.63
chr3R	7964810	7965243	dsx	2270	34.5905	5.43
chr3R	7966200	7968371	dsx	536	316.742	38.43
chr3R	7968891	7969625	dsx	1738	46.8918	6.86
chr3R	7977264	7977774	CD98hc	95	12.354	3.04
chr3R	7985369	7985764	Sfp84E	5375	6.58803	2.41
chr3R	7993524	7994068	CG2781	2017	77.725	9.59
chr3R	7995628	7996328	CG2781	152	189.043	14.69
chr3R	8001399	8001864	CG2698	16	16.0637	3.90
chr3R	8026974	8027886	CG2747	439	166.195	13.24
chr3R	8030179	8030766	CG10903	54	15.813	3.39
chr3R	8059077	8059371	mAChR-B	121	9.74428	3.38
chr3R	8094027	8094364	CG7900	35	48.6506	8.24
chr3R	8105217	8106862	puc	235	316.742	28.35
chr3R	8108780	8109321	puc	2548	72.1761	7.24
chr3R	8111772	8112305	CR44331	1981	26.0501	5.00
chr3R	8113341	8114612	CR44331	430	37.5292	5.54
chr3R	8116224	8116584	CR44331	2375	19.2554	4.10
chr3R	8119648	8120325	CG7878	2687	316.742	25.90
chr3R	8122507	8122906	CG7878	56	29.2625	4.83
chr3R	8126543	8127220	Gie	239	134.4	11.06
chr3R	8181283	8182209	grn	302	279.57	17.28
chr3R	8190711	8191324	CR43302	6693	275.8	19.76
chr3R	8219976	8220239	CG18249	93	12.6703	3.39
chr3R	8233342	8234053	DNApol-iota	12	15.8377	3.80
chr3R	8233342	8234053	CR45549	119	15.8377	3.80
chr3R	8240240	8240582	Ada2b	116	19.3532	4.36
chr3R	8250324	8250621	mtg	69	15.6469	3.81
chr3R	8251631	8251924	mtg	1300	14.6471	3.61
chr3R	8261425	8262147	RpA-70	2755	250.384	15.53
chr3R	8308688	8309096	tex	230	30.8454	5.10
chr3R	8317255	8318214	Ctr1B	311	293.588	18.50
chr3R	8333726	8334652	Poxm	169	61.4519	9.01
chr3R	8349760	8350111	Atg13	119	16.7012	3.63
chr3R	8353067	8353592	Atg13	3452	23.087	3.95

chr3R	8355832	8356109	stck	992	12.9984	3.16
chr3R	8357127	8357796	stck	69	11.2653	2.80
chr3R	8517752	8518447	CG11737	267	16.2529	3.55
chr3R	8658598	8660530	bel	1487	316.742	33.20
chr3R	8663424	8663843	p	277	18.1042	3.66
chr3R	8669016	8670884	CG8036	70	241.948	13.27
chr3R	8669016	8670884	p	1137	241.948	13.27
chr3R	8697572	8698305	hb	178	52.0851	7.86
chr3R	8746999	8748299	CG9801	45	38.6252	5.35
chr3R	8746999	8748299	CG8223	167	38.6252	5.35
chr3R	8792340	8792942	Pif1A	121	205.007	16.85
chr3R	8792340	8792942	Pif1B	121	205.007	16.85
chr3R	8805396	8805796	CG9839	36	31.1664	4.98
chr3R	8819664	8820941	CG9821	4	100.111	8.16
chr3R	8819664	8820941	CR43130	83	100.111	8.16
chr3R	8821662	8822115	CG8369	97	44.9389	6.51
chr3R	8823307	8823613	IscU	738	15.8558	3.56
chr3R	8823932	8824202	IscU	105	11.2848	3.06
chr3R	8846053	8847133	pyd	5	62.2729	7.59
chr3R	8854812	8855210	pyd	2627	28.3346	5.03
chr3R	8857516	8857906	pyd	84	17.7496	3.92
chr3R	8930846	8932101	pyd	23	127.929	9.65
chr3R	8938628	8939365	skap	255	122.751	10.28
chr3R	8955593	8955885	CG11966	7277	18.1418	4.67
chr3R	8980292	8981474	CAHbeta	54	316.742	24.58
chr3R	9013555	9014918	CG11984	149	75.5704	7.83
chr3R	9019668	9020034	tgo	228	15.3773	3.65
chr3R	9033536	9034464	neur	427	211.454	18.48
chr3R	9047455	9048938	Rel	1	87.4689	7.78
chr3R	9047455	9048938	Mst85C	814	87.4689	7.78
chr3R	9047455	9048938	Nmdmc	821	87.4689	7.78
chr3R	9052360	9055728	Kdm2	86	316.742	34.37
chr3R	9056124	9058268	Kdm2	4015	316.742	41.59
chr3R	9157735	9158636	pum	279	316.742	31.26
chr3R	9160167	9160716	pum	2504	160.716	13.08
chr3R	9214812	9215311	pum	11806	67.1236	7.79
chr3R	9226224	9228454	pum	377	316.742	28.23
chr3R	9226224	9228454	CR45196	915	316.742	28.23
chr3R	9228933	9230687	CR45196	1528	316.742	23.17
chr3R	9235906	9237806	pum	82	316.742	35.22
chr3R	9239730	9240269	D1	273	146.669	11.99
chr3R	9334494	9334814	CG8866	69	18.3726	3.88
chr3R	9338855	9339182	CR43301	986	23.5552	4.35
chr3R	9339563	9339961	CR43301	289	8.72213	2.57
chr3R	9340743	9342793	CG45050	819	220.576	12.99
chr3R	9350577	9351185	noRNA:Me18S-G1358	3432	210.369	17.45
chr3R	9351539	9354193	noRNA:Me18S-G1358	1109	316.742	33.10
chr3R	9359359	9359785	CG45050	3623	113.796	13.54
chr3R	9359359	9359785	CG43675	3623	113.796	13.54
chr3R	9393511	9393871	CR46145	229	18.6407	4.21
chr3R	9394143	9394864	FER	63	316.742	27.98
chr3R	9400969	9401399	RpL34b	9	16.1275	3.27
chr3R	9417455	9419779	ps	95	316.742	45.89
chr3R	9420330	9421399	ps	1144	124.592	11.52
chr3R	9422080	9422715	ps	2712	13.3521	2.96
chr3R	9427925	9428940	ps	135	316.742	42.45
chr3R	9429976	9430454	ps	1766	13.3836	3.15
chr3R	9432823	9433287	ps	472	61.8142	7.07
chr3R	9434939	9437099	ps	96	316.742	27.90
chr3R	9437479	9437826	ps	791	7.25069	2.50
chr3R	9459489	9460127	GstZ2	508	18.6954	4.35
chr3R	9460557	9460975	GstZ2	126	72.7217	7.38
chr3R	9491025	9491568	CG16779	2316	36.1316	6.34
chr3R	9493579	9494060	CG16779	131	99.1978	9.98
chr3R	9501678	9503154	RhoL	41	316.742	28.51
chr3R	9512124	9513382	Ras85D	225	316.742	24.95
chr3R	9512124	9513382	Rlb1	484	316.742	24.95
chr3R	9515988	9516777	CG8176	116	14.0432	3.07
chr3R	9515988	9516777	JHDM2	116	14.0432	3.07
chr3R	9515988	9516777	CG8176	116	14.0432	3.07
chr3R	9528470	9528800	by	4417	14.0984	3.70
chr3R	9532148	9534205	by	607	316.742	36.62
chr3R	9534546	9535099	by	1794	316.742	21.53
chr3R	9542614	9542875	mura	8543	15.5456	3.50
chr3R	9543460	9543969	mura	7614	103.314	9.70

chr3R	9550518	9551895	mura	470	316.742	47.60
chr3R	9550518	9551895	CR42549	489	316.742	47.60
chr3R	9550518	9551895	mura	938	316.742	47.60
chr3R	9550518	9551895	CR42549	938	316.742	47.60
chr3R	9559640	9560008	MBD-like	27	9.92342	2.76
chr3R	9570403	9571730	CG9396	1052	25.012	4.50
chr3R	9573244	9574273	CG9399	69	316.742	21.42
chr3R	9577439	9578086	Kap-alpha3	4	10.7472	2.95
chr3R	9577439	9578086	CG8273	304	10.7472	2.95
chr3R	9581126	9581691	CG8301	62	98.5129	10.89
chr3R	9586747	9587178	P58IPK	35	13.2052	3.06
chr3R	9591161	9591539	CG8312	86	20.9105	3.71
chr3R	9593647	9593956	CG8312	1938	8.77179	2.73
chr3R	9603380	9603948	CG8312	11676	23.2866	4.11
chr3R	9630015	9631085	Calr	13	51.2475	5.61
chr3R	9704136	9705712	Unc-115a	493	316.742	20.84
chr3R	9710424	9711154	trbd	7	17.4916	3.52
chr3R	9710424	9711154	dmt	111	17.4916	3.52
chr3R	9725364	9725676	hyd	143	8.42048	2.46
chr3R	9739987	9740509	CG12950	411	13.0388	3.47
chr3R	9747067	9747587	CG12948	173	49.5782	6.14
chr3R	9762807	9763088	Snap24	202	15.4574	3.83
chr3R	9767726	9768059	MED6	72	10.2455	2.82
chr3R	9767726	9768059	CG8481	54	10.2455	2.82
chr3R	9784485	9784905	CG8500	45	19.4237	4.51
chr3R	9800797	9801135	CG9467	0	13.0387	3.28
chr3R	9808703	9809413	FBXO11	237	65.5175	6.17
chr3R	9865688	9866722	Teh1	2038	245.168	18.42
chr3R	9867956	9868859	Teh1	209	88.1412	9.46
chr3R	9872221	9872817	Glut4EF	2185	134.018	10.37
chr3R	9873374	9875238	Glut4EF	74	316.742	25.07
chr3R	9877467	9878257	CR45029	632	185.576	14.20
chr3R	9884470	9884952	CR45029	6210	60.3113	7.51
chr3R	9906999	9907554	Glut4EF	21792	18.5328	3.96
chr3R	9928264	9929253	Glut4EF	183	316.742	36.14
chr3R	9931673	9933696	Glut4EF	3711	165.3	13.32
chr3R	9946281	9947055	Glut4EF	17855	92.1503	9.36
chr3R	9947557	9948060	Glut4EF	18959	14.2573	3.20
chr3R	9949651	9950505	Glut4EF	21162	316.742	22.87
chr3R	9950914	9951405	Glut4EF	22339	16.5971	3.75
chr3R	9951688	9952216	Glut4EF	23031	23.665	4.37
chr3R	9952506	9953438	Glut4EF	24183	148.583	11.89
chr3R	9954292	9955290	Glut4EF	26086	35.258	4.96
chr3R	9978918	9979688	Art4	6823	81.1517	9.08
chr3R	9982444	9982862	Art4	3496	14.1991	3.61
chr3R	9985734	9986343	Art4	53	15.535	3.38
chr3R	10035116	10035552	Mical	7426	46.7085	6.91
chr3R	10038976	10039304	Mical	3646	18.6178	4.09
chr3R	10042518	10043022	Mical	37	25.6344	4.85
chr3R	10042518	10043022	Mical	37	25.6344	4.85
chr3R	10048599	10048955	CG12811	15	51.1752	6.44
chr3R	10070222	10070557	nerfin-2	734	19.8065	4.46
chr3R	10077347	10079891	CG5361	4075	316.742	29.53
chr3R	10080365	10081259	CR45054	2540	148.299	10.10
chr3R	10081529	10083519	CR45054	375	316.742	23.09
chr3R	10084016	10085397	CR45054	963	316.742	21.49
chr3R	10086104	10086538	CR45054	2920	22.0936	3.97
chr3R	10086937	10088207	mir-317	3844	316.742	25.10
chr3R	10088912	10090455	mir-317	1707	316.742	40.23
chr3R	10093188	10095224	CR43459	128	316.742	22.45
chr3R	10109060	10110308	Fmr1	161	316.742	32.14
chr3R	10111972	10112395	CG3940	68	98.2742	11.31
chr3R	10124884	10125153	knk	648	22.3929	4.48
chr3R	10133067	10134495	tws	433	106.852	9.95
chr3R	10139546	10140478	tws	473	280.265	19.76
chr3R	10144544	10145016	Invadolysin	83	62.4795	6.37
chr3R	10170179	10170894	Best1	66	316.742	32.55
chr3R	10188914	10191295	CG12814	77	313.645	16.86
chr3R	10188914	10191295	Syn	1008	313.645	16.86
chr3R	10212792	10213947	Timp	26	65.2944	7.67
chr3R	10226377	10227024	CG42795	85	29.8095	5.17
chr3R	10264063	10265349	CG43143	82	123.499	9.69
chr3R	10267028	10267552	CG43143	3014	156.047	13.45
chr3R	10268196	10268560	CG43143	4123	43.776	6.79
chr3R	10311443	10311855	CG6293	43	100.984	11.45

chr3R	10331179	10331436	Bruce	82	8.03147	2.49
chr3R	10349990	10351117	jumu	608	82.6389	7.62
chr3R	10351867	10352434	jumu	1973	55.3146	7.02
chr3R	10356034	10356815	jumu	6394	81.1455	10.00
chr3R	10368300	10368692	Rfx	1048	35.1247	6.04
chr3R	10378323	10378587	Rfx	413	12.1332	3.15
chr3R	10379823	10380575	Rfx	2362	12.5094	2.94
chr3R	10380974	10381278	CR44938	2650	29.0413	5.38
chr3R	10384390	10384941	CR44938	880	289.558	20.21
chr3R	10387005	10387452	CR44938	237	316.742	47.15
chr3R	10388126	10389467	cwo	17	249.801	24.32
chr3R	10393375	10393771	cwo	5351	21.3272	4.12
chr3R	10434714	10435258	Cyp12e1	289	56.2627	7.77
chr3R	10527614	10528024	mir-4944	14664	49.5146	7.08
chr3R	10535658	10536211	mir-4944	22802	316.742	24.46
chr3R	10546583	10547173	mir-4944	33914	16.0637	3.90
chr3R	10556749	10557686	mir-4944	43860	115.431	12.23
chr3R	10567432	10567866	hth	46697	9.67102	2.88
chr3R	10587968	10589176	hth	25339	32.277	5.35
chr3R	10592942	10593349	hth	21118	20.5466	4.49
chr3R	10613982	10615176	hth	241	316.742	24.38
chr3R	10616433	10617540	hth	2989	241.443	16.81
chr3R	10622062	10622685	hth	8065	316.742	19.98
chr3R	10638707	10640035	CR44018	400	65.7345	8.75
chr3R	10638707	10640035	hth	690	65.7345	8.75
chr3R	10640560	10641095	hth	1310	49.182	6.85
chr3R	10664838	10665672	CG6465	12024	172.827	14.42
chr3R	10680875	10681134	CG14688	48	16.8016	3.79
chr3R	10686728	10686995	Skeletor	91	18.9621	4.63
chr3R	10696679	10697118	CG14683	428	9.58397	2.86
chr3R	10696679	10697118	pug	428	9.58397	2.86
chr3R	10699378	10699760	pug	58	34.0246	6.35
chr3R	10761088	10761681	Cap-H2	16	32.8175	4.67
chr3R	10826567	10827382	Cad86C	88	316.742	44.26
chr3R	10849596	10850631	CG42394	16	87.9453	8.44
chr3R	10852261	10852590	CG4565	242	16.4955	4.00
chr3R	10859984	10860245	CG14694	333	15.8227	4.03
chr3R	10868193	10868866	SelR	20	23.1791	4.24
chr3R	10868193	10868866	Tsp86D	85	23.1791	4.24
chr3R	10872095	10872514	Fdh	87	36.6054	5.39
chr3R	10875822	10876544	Sodh-2	334	81.8076	9.79
chr3R	10881839	10882344	Tpc1	30	12.5931	3.09
chr3R	10884887	10885994	CG14696	551	193.03	13.18
chr3R	10884887	10885994	Leash	13	193.03	13.18
chr3R	10886379	10887375	Leash	1527	316.742	30.65
chr3R	10888535	10889726	Adk3	142	316.742	46.58
chr3R	10970886	10971841	CG34114	729	44.8251	7.72
chr3R	10972112	10972508	CG34114	44	22.6835	4.48
chr3R	11128155	11128642	Ugt86Dd	70	39.9166	6.57
chr3R	11156625	11157429	Ugt86Da	111	316.742	61.20
chr3R	11172828	11173188	Ugt35a	365	60.4003	7.96
chr3R	11196823	11198396	cu	113	306.554	19.06
chr3R	11199948	11200906	cu	208	316.742	25.16
chr3R	11210073	11210497	Tctp	6	35.1771	4.81
chr3R	11211251	11211867	Tctp	1350	31.2463	4.88
chr3R	11221452	11222042	CG6693	221	24.5734	4.23
chr3R	11221452	11222042	RpL3	33	24.5734	4.23
chr3R	11243445	11244616	CG17734	299	20.8254	3.52
chr3R	11243445	11244616	CG5214	3	20.8254	3.52
chr3R	11254450	11254866	CR43283	1479	79.5904	9.20
chr3R	11281561	11282144	CR31386	23848	33.1559	6.56
chr3R	11302809	11303394	CR31386	2527	107.394	13.42
chr3R	11312262	11312621	CR45055	1790	39.5395	7.19
chr3R	11330577	11331898	pros	1717	92.1126	10.32
chr3R	11334298	11334884	pros	397	32.606	6.22
chr3R	11335530	11336312	CR46008	581	35.813	6.76
chr3R	11341199	11341506	CR46008	5051	9.69643	3.19
chr3R	11352952	11353725	KP78a	106	220.792	19.37
chr3R	11352952	11353725	KP78b	106	220.792	19.37
chr3R	11371816	11372930	pros	759	41.9596	6.99
chr3R	11458083	11458338	PGRP-LB	189	23.3221	4.87
chr3R	11483376	11483780	CR45581	3232	49.539	8.20
chr3R	11499784	11500149	dpr5	28	35.3264	6.54
chr3R	11542614	11543505	dpr4	104	86.1693	10.73
chr3R	11557750	11558334	CG10898	271	37.9558	5.10

chr3R	11561862	11563539	sea	408	202.664	16.36
chr3R	11566449	11567528	fabp	64	316.742	19.90
chr3R	11566449	11567528	sea	64	316.742	19.90
chr3R	11568598	11570204	Mrp4	86	316.742	44.44
chr3R	11581438	11582340	wkd	218	147.059	12.95
chr3R	11582981	11583407	wkd	268	58.431	7.89
chr3R	11589783	11590089	CG6791	119	5.95359	2.27
chr3R	11604231	11605254	Jupiter	140	316.742	42.23
chr3R	11619680	11619956	Jupiter	62	9.93728	2.53
chr3R	11646778	11647418	Csk	146	176.596	13.78
chr3R	11647741	11648193	Csk	1045	25.1315	4.22
chr3R	11648713	11649255	Csk	1726	15.629	3.87
chr3R	11650343	11651056	Csk	116	25.7164	4.41
chr3R	11650343	11651056	Elp1	380	25.7164	4.41
chr3R	11655722	11657075	CG42327	18	316.742	19.04
chr3R	11661071	11661448	CG42327	5108	25.4032	4.87
chr3R	11669524	11670070	CG14715	4541	23.2431	4.65
chr3R	11684036	11684413	CG6830	80	24.1324	4.76
chr3R	11687964	11688368	CG6834	66	21.7007	4.53
chr3R	11689685	11691475	CG6908	1150	99.2472	10.27
chr3R	11694704	11695429	Fer3	895	37.6451	6.05
chr3R	11749742	11750115	CG6923	137	12.6214	3.13
chr3R	11756784	11757046	Lk6	3142	16.9534	3.77
chr3R	11758280	11762137	Lk6	85	316.742	27.18
chr3R	11762438	11762871	Lk6	1819	43.4259	5.55
chr3R	11763427	11764686	Lk6	541	253.707	15.92
chr3R	11770842	11771123	l(3)neo38	610	13.1443	3.71
chr3R	11773331	11774201	l(3)neo38	303	316.742	24.68
chr3R	11777766	11779345	l(3)neo38	55	140.653	11.20
chr3R	11779608	11781114	CR45595	82	316.742	20.49
chr3R	11786612	11787167	CG17360	112	6.68471	2.46
chr3R	11812725	11813586	ClC-a	5	111.608	9.47
chr3R	11819046	11819675	glo	62	14.4244	3.34
chr3R	11819046	11819675	COX5A	81	14.4244	3.34
chr3R	11865080	11865495	CG6959	4093	27.8929	4.66
chr3R	11868486	11869676	CG6959	37	97.255	9.42
chr3R	11869953	11870372	CG6959	571	10.4133	2.70
chr3R	11886723	11888580	mthl5	45	316.742	48.34
chr3R	11923244	11923623	CG14731	7872	32.8269	6.00
chr3R	11948617	11949342	Cad87A	44	196.193	12.13
chr3R	11949640	11950025	CG31211	99	12.0506	3.06
chr3R	11958078	11958761	Hsp70Aa	14	316.742	23.73
chr3R	11958078	11958761	Hsp70Ab	13	316.742	23.73
chr3R	11967620	11968215	CG12213	276	55.5753	7.14
chr3R	11967620	11968215	GC1	260	55.5753	7.14
chr3R	11980057	11980594	CG18547	55	272.345	22.27
chr3R	11981925	11983043	CG12224	281	33.6709	5.04
chr3R	11984186	11985138	CG3397	43	50.3978	7.15
chr3R	11990283	11990540	mfas	360	14.8813	3.82
chr3R	11992919	11993662	mfas	59	288.907	19.37
chr3R	12078654	12081096	ATP8B	860	316.742	22.90
chr3R	12090180	12090984	ATP8B	96	316.742	21.44
chr3R	12158497	12158792	dpr15	51	14.6003	3.81
chr3R	12182351	12182657	CG4810	12524	42.0673	7.55
chr3R	12221293	12221564	CG4860	46	16.6802	3.58
chr3R	12244364	12244934	CR45582	365	172.306	14.23
chr3R	12278780	12279513	svp	1945	250.907	17.09
chr3R	12280554	12281334	svp	144	316.742	26.38
chr3R	12367482	12368024	GstD9	120	114.186	11.34
chr3R	12368934	12369759	GstD1	64	316.742	27.93
chr3R	12370369	12371135	CR46015	415	94.3243	10.71
chr3R	12398768	12399057	lig3	2971	11.3914	3.12
chr3R	12405624	12406119	Cyp9f2	121	34.5905	5.43
chr3R	12406585	12407463	CG5167	308	14.7626	3.73
chr3R	12425870	12426286	Pp1-87B	255	13.34	3.34
chr3R	12442679	12443374	Desat1	650	121.756	8.74
chr3R	12443834	12444238	Desat1	19	13.4897	3.17
chr3R	12448785	12450079	CG18549	250	107.239	8.73
chr3R	12451849	12452325	CG5509	263	27.4601	5.36
chr3R	12467676	12468089	Hsp70Ba	123	316.742	50.73
chr3R	12477327	12477759	agamma-element:CR3	790	316.742	29.71
chr3R	12505774	12506064	Hsp70Bb	126	110.041	6.33
chr3R	12595403	12596261	Octbeta2R	296	255.361	17.14
chr3R	12626613	12628881	Vha55	655	27.1007	4.39
chr3R	12626613	12628881	Snx3	984	27.1007	4.39

chr3R	12642466	12642777	CG6753	90	10.3226	3.48
chr3R	12653530	12654707	CG6225	83	55.0313	7.46
chr3R	12689217	12689639	kar	78	62.8958	8.52
chr3R	12717138	12717874	Men	2107	31.1506	5.29
chr3R	12718202	12718713	Men	1299	41.3993	5.37
chr3R	12719387	12720255	Men	152	316.742	28.50
chr3R	12720622	12721068	Men	72	52.4145	7.52
chr3R	12783145	12783628	beat-Vc	273	22.3373	4.80
chr3R	12848370	12848743	beat-Va	169	32.1474	5.69
chr3R	12857113	12857971	CG10126	48	149.343	13.08
chr3R	12915801	12916210	beat-Vb	86	28.5516	5.14
chr3R	12961485	12961890	grsm	77	28.0128	4.25
chr3R	12961485	12961890	Spc25	24	28.0128	4.25
chr3R	12974326	12974699	CG7381	72	32.4941	6.11
chr3R	12981019	12981332	Paip2	21	8.13585	2.54
chr3R	12981990	12982411	Paip2	1055	8.64876	2.48
chr3R	12982436	12983394	Paip2	2186	10.0372	2.72
chr3R	12994650	12995362	yellow-f2	2	73.7425	7.72
chr3R	12997995	12998369	CG7518	53	12.8367	3.13
chr3R	13010992	13011263	CG8031	274	13.2696	3.25
chr3R	13011659	13013354	CtBP	987	308.214	20.21
chr3R	13015419	13017016	CtBP	1003	51.1702	6.95
chr3R	13017484	13018026	CtBP	557	26.7911	4.53
chr3R	13032467	13032736	ry	17	8.50399	2.61
chr3R	13051159	13051721	CG7966	0	316.445	17.89
chr3R	13052032	13052696	pic	253	75.6889	7.20
chr3R	13063978	13064682	sim	1881	149.072	11.43
chr3R	13155533	13155968	2mit	82	52.6919	8.20
chr3R	13155533	13155968	CR45109	82	52.6919	8.20
chr3R	13208301	13208704	CG8483	5937	56.639	6.72
chr3R	13232864	13233158	CG8476	12423	9.62707	2.91
chr3R	13240394	13240983	Ace	18154	113.464	11.66
chr3R	13258526	13259672	Ace	103	260.113	16.60
chr3R	13260173	13260616	CG11686	33	35.7141	5.03
chr3R	13304567	13305077	CG32473	18	95.1742	10.32
chr3R	13318189	13318760	CG45080	845	316.742	22.50
chr3R	13364048	13365405	poly	535	316.742	33.05
chr3R	13364048	13365405	Dic1	186	316.742	33.05
chr3R	13379289	13380000	CG9813	21	86.0104	9.31
chr3R	13379289	13380000	CG8870	45	86.0104	9.31
chr3R	13382037	13383064	Droj2	9	102.172	8.65
chr3R	13401091	13402100	GILT1	43	237.808	20.04
chr3R	13434123	13435166	yrt	24	15.2632	3.33
chr3R	13450231	13450722	CG14372	113	37.5953	6.28
chr3R	13466008	13466594	CR17025	164	22.0711	4.81
chr3R	13466008	13466594	CR45914	224	22.0711	4.81
chr3R	13538748	13539320	CG31337	6464	44.8909	7.20
chr3R	13554054	13554361	CG31337	8821	35.0866	6.30
chr3R	13644638	13646324	sqd	1040	316.742	28.58
chr3R	13646851	13648155	rin	891	109.777	9.17
chr3R	13660176	13660607	Hrb87F	71	20.0988	3.74
chr3R	13661202	13661810	B52	111	17.5197	3.28
chr3R	13674425	13674761	Task6	93	24.9208	4.78
chr3R	13676490	13676771	Lkb1	45	10.0339	3.08
chr3R	13676490	13676771	CG9588	88	10.0339	3.08
chr3R	13676490	13676771	Lkb1	95	10.0339	3.08
chr3R	13683623	13685506	flfl	292	316.742	32.28
chr3R	13686110	13686981	omd	471	116.65	9.65
chr3R	13714951	13715524	CG34383	50	211.763	16.16
chr3R	13735333	13735684	CG34383	51	19.4612	4.20
chr3R	13782286	13783037	Dip-B	34	225.514	14.96
chr3R	13794338	13795411	CR45590	8417	316.742	36.78
chr3R	13796572	13797075	CR45590	10248	50.8044	6.74
chr3R	13797837	13798401	CR45590	11728	10.713	3.10
chr3R	13800029	13801320	tal-2A	12414	26.8125	5.39
chr3R	13800029	13801320	tal-1A	12414	26.8125	5.39
chr3R	13800029	13801320	tal-AA	12414	26.8125	5.39
chr3R	13800029	13801320	tal-3A	12414	26.8125	5.39
chr3R	13802970	13803825	tal-2A	10015	14.6003	3.81
chr3R	13802970	13803825	tal-1A	10015	14.6003	3.81
chr3R	13802970	13803825	tal-AA	10015	14.6003	3.81
chr3R	13802970	13803825	tal-3A	10015	14.6003	3.81
chr3R	13804330	13805419	tal-2A	7905	26.5212	5.17
chr3R	13804330	13805419	tal-1A	7905	26.5212	5.17
chr3R	13804330	13805419	tal-AA	7905	26.5212	5.17

chr3R	13804330	13805419	tal-3A	7905	26.5212	5.17
chr3R	13806108	13806542	tal-2A	6718	8.6007	3.11
chr3R	13806108	13806542	tal-1A	6718	8.6007	3.11
chr3R	13806108	13806542	tal-AA	6718	8.6007	3.11
chr3R	13806108	13806542	tal-3A	6718	8.6007	3.11
chr3R	13812534	13813363	tal-2A	172	316.742	46.68
chr3R	13812534	13813363	tal-1A	172	316.742	46.68
chr3R	13812534	13813363	tal-AA	172	316.742	46.68
chr3R	13812534	13813363	tal-3A	172	316.742	46.68
chr3R	13814381	13815608	CR43641	58	316.742	46.14
chr3R	13831280	13832262	Mst87F	3578	221.565	17.01
chr3R	13874769	13875642	E5	376	316.742	34.16
chr3R	13877009	13877662	E5	2362	265.509	18.28
chr3R	13901344	13902082	ems	98	300.785	18.99
chr3R	13904857	13905222	ems	3178	23.7851	4.97
chr3R	13958626	13958944	lpp	18	24.0577	4.61
chr3R	13975995	13976650	rdx	4499	316.742	20.39
chr3R	13983081	13984198	rdx	501	316.742	26.25
chr3R	14011094	14011589	rdx	14691	11.3812	3.20
chr3R	14014568	14014951	rdx	11192	36.7406	6.03
chr3R	14017476	14018784	rdx	8120	316.742	46.70
chr3R	14020434	14021258	rdx	5061	316.742	27.37
chr3R	14024058	14026966	rdx	945	230.972	13.86
chr3R	14027403	14027668	rdx	1642	13.8687	3.45
chr3R	14028877	14029303	Cyp6d5	67	22.9491	4.41
chr3R	14031638	14032884	rdx	132	22.382	3.81
chr3R	14031638	14032884	CG3061	445	22.382	3.81
chr3R	14050634	14053777	foxo	4276	181.16	12.37
chr3R	14055804	14058028	foxo	280	316.742	27.89
chr3R	14059658	14060285	foxo	2070	102.312	10.88
chr3R	14061296	14062647	foxo	3829	32.5695	5.34
chr3R	14064588	14064948	foxo	6861	22.9585	4.47
chr3R	14088591	14089495	Npc2b	445	14.9456	3.62
chr3R	14090653	14090964	Npc2b	48	22.9574	4.36
chr3R	14098623	14098939	CG9920	690	25.328	4.35
chr3R	14100315	14101025	PK1-R	2164	245.71	17.51
chr3R	14102945	14103274	PK1-R	31	20.8328	4.84
chr3R	14108260	14108842	CG12402	234	115.162	11.29
chr3R	14140923	14141324	CG14356	9054	64.1906	8.99
chr3R	14170528	14170786	CG3199	7898	8.16286	2.90
chr3R	14214634	14215181	Dop1R1	132	74.2808	8.51
chr3R	14216895	14218088	CG9649	5	66.0618	7.41
chr3R	14219919	14220525	CG31326	88	247.194	20.59
chr3R	14223912	14224372	Sp212	64	36.1251	5.56
chr3R	14226467	14227081	CG9631	45	234.467	15.89
chr3R	14277555	14279678	trx	8047	316.742	21.70
chr3R	14280036	14280326	trx	6714	10.3178	2.98
chr3R	14282473	14287025	trx	387	316.742	34.83
chr3R	14288881	14289533	CG12207	70	205.899	14.70
chr3R	14298062	14298649	CG12207	55	97.3296	9.19
chr3R	14307987	14308425	su(Hw)	448	19.1502	3.45
chr3R	14316000	14316605	Set8	3	171.357	11.20
chr3R	14326346	14326736	Afti	78	10.6118	2.74
chr3R	14342315	14342591	spn-B	14310	7.69742	2.82
chr3R	14346728	14347427	HEATR2	14759	198.264	13.21
chr3R	14363210	14363576	HEATR2	1606	13.8618	3.49
chr3R	14364019	14364401	HEATR2	2399	13.9547	3.15
chr3R	14365033	14365516	HEATR2	3298	10.0569	3.04
chr3R	14369716	14370668	NK7.1	275	257.382	16.07
chr3R	14372821	14374011	NK7.1	720	301.046	16.05
chr3R	14382049	14383254	soti	846	316.742	25.15
chr3R	14391008	14391600	mir-2281	5059	73.9437	7.34
chr3R	14478756	14479251	cv-c	2683	129.234	13.85
chr3R	14481521	14482058	cv-c	172	21.913	4.57
chr3R	14557776	14558826	Pde6	110	301.181	17.58
chr3R	14567202	14567712	CG31313	46	108.626	9.31
chr3R	14568180	14569888	CG8066	802	218.967	13.20
chr3R	14568180	14569888	CR44095	688	218.967	13.20
chr3R	14568180	14569888	Cys	5	218.967	13.20
chr3R	14574705	14576028	CG7987	904	121.21	12.17
chr3R	14608709	14609815	CG14853	553	108.023	12.01
chr3R	14624216	14624811	put	1160	305.245	21.19
chr3R	14625143	14626130	put	31	10.5364	2.67
chr3R	14625143	14626130	His4r	20	10.5364	2.67
chr3R	14630082	14630633	Cad88C	125	117.429	13.26

chr3R	14649238	14650510	l(3)L1231	5517	47.114	5.08
chr3R	14652344	14652819	l(3)L1231	2606	60.9484	7.30
chr3R	14653086	14653551	l(3)L1231	1919	38.7035	5.73
chr3R	14654446	14655787	l(3)L1231	75	151.382	9.98
chr3R	14656170	14656540	l(3)L1231	648	16.1275	3.27
chr3R	14657215	14657865	CG3505	31	101.273	9.66
chr3R	14659416	14659733	Hexim	146	11.3043	2.72
chr3R	14696735	14697466	Meltrin	277	316.742	25.20
chr3R	14711386	14712199	kibra	12320	51.8059	7.23
chr3R	14714108	14715230	kibra	9773	216.6	19.24
chr3R	14722090	14722982	kibra	1822	316.742	28.98
chr3R	14723563	14724446	kibra	384	316.742	26.25
chr3R	14728990	14729260	CG7530	35	16.9722	3.98
chr3R	14730556	14731831	CG7530	60	196.662	12.40
chr3R	14734768	14735049	CG7530	3422	25.0324	4.94
chr3R	14737967	14738341	eff	2653	20.2289	4.29
chr3R	14738628	14741116	eff	1505	251.076	14.33
chr3R	14741467	14742155	jvl	70	9.55401	2.78
chr3R	14743502	14743773	jvl	2048	16.8476	3.90
chr3R	14747832	14748251	smp-30	634	22.1347	4.49
chr3R	14748774	14749838	smp-30	488	316.742	31.64
chr3R	14748774	14749838	jvl	87	316.742	31.64
chr3R	14776597	14776922	jvl	11189	24.31	4.93
chr3R	14796051	14796499	CG14855	37	92.9285	12.42
chr3R	14817671	14818677	btsz	92	316.742	37.11
chr3R	14819784	14820131	btsz	1174	26.7672	5.30
chr3R	14844384	14844733	CG3610	447	24.5085	5.37
chr3R	14853653	14854406	btsz	106	316.742	32.02
chr3R	14873243	14873680	btsz	73	20.8219	4.76
chr3R	14875419	14876314	btsz	602	87.5447	11.26
chr3R	14986089	14986552	CR45599	18630	115.652	12.40
chr3R	15077003	15077610	dpr9	93	73.8896	8.91
chr3R	15092727	15093028	CG6966	2769	28.8594	5.00
chr3R	15093816	15095911	CG6966	942	164.05	11.04
chr3R	15128634	15128931	mir-2493	2073	22.1018	4.98
chr3R	15130676	15132096	mir-2493	975	256.743	13.37
chr3R	15130676	15132096	CG42788	92	256.743	13.37
chr3R	15146726	15147833	GlyS	10	130.872	10.60
chr3R	15149950	15150354	Gyc88E	306	10.9947	3.41
chr3R	15166639	15166944	Mf	12669	22.3717	4.06
chr3R	15196460	15196867	CG42542	39	14.0871	3.36
chr3R	15206220	15206575	Spn88Ea	28	41.9016	5.44
chr3R	15242411	15243987	Hsc70-4	434	61.0323	6.76
chr3R	15250196	15250878	CG42404	410	54.9599	7.11
chr3R	15285953	15287105	Tm1	206	137.923	13.96
chr3R	15287745	15288136	Tm1	80	27.0657	4.53
chr3R	15289883	15292244	CG45218	951	316.742	26.20
chr3R	15289883	15292244	Tm1	805	316.742	26.20
chr3R	15293207	15293614	Tm1	2430	31.3519	5.54
chr3R	15328575	15329136	ea	38	49.182	6.85
chr3R	15342506	15343207	CG6218	314	101.402	9.35
chr3R	15345192	15345798	Surf4	631	52.9671	6.72
chr3R	15357129	15357410	CG5044	39	17.8002	4.10
chr3R	15371950	15372551	Ccm3	297	100.732	10.41
chr3R	15371950	15372551	CG6136	41	100.732	10.41
chr3R	15374379	15374865	Rbp	10	69.997	8.62
chr3R	15375222	15376000	Rbp	911	15.7831	3.90
chr3R	15378971	15379495	h-cup	3735	34.7685	5.89
chr3R	15401411	15401870	CR46058	1421	34.369	4.84
chr3R	15408524	15409543	Atx2	541	35.8167	5.57
chr3R	15419088	15419678	CR45911	1002	55.9527	8.11
chr3R	15420260	15420978	CR45911	2	162.693	14.13
chr3R	15476101	15476737	AdamTS-A	313	138.11	14.98
chr3R	15536916	15537685	AOX1	77	316.742	31.48
chr3R	15547333	15548467	AOX3	87	114.661	10.53
chr3R	15664664	15665201	pxb	1240	97.4174	8.41
chr3R	15665832	15666400	pxb	117	118.445	13.18
chr3R	15793366	15794743	nsl1	756	111.471	10.00
chr3R	15804097	15804441	alpha-Man-IIb	9	18.0383	3.46
chr3R	15810481	15810835	alpha-Man-IIb	31	14.7623	4.11
chr3R	15875547	15875915	CG31183	189	14.1844	3.22
chr3R	15897899	15898988	CG14877	2689	215.201	18.25
chr3R	15899423	15900075	glob1	1402	10.903	3.06
chr3R	15900961	15901657	glob1	36	316.742	40.44
chr3R	15908310	15908593	CG3303	30	47.6938	8.14

chr3R	15919744	15921591	Fbxl7	21	316.742	29.28
chr3R	15923466	15923900	CR46028	485	46.8806	7.41
chr3R	15935141	15936250	Sap47	918	103.293	10.05
chr3R	15937670	15938229	Sap47	2806	71.8668	8.85
chr3R	15973068	15974795	Hel89B	471	316.742	27.61
chr3R	15993122	15993605	srp	770	72.6947	9.20
chr3R	16025588	16026353	pnr	182	316.742	28.77
chr3R	16050788	16051754	msps	666	153.202	12.92
chr3R	16071915	16072521	Mhcl	224	111.58	11.35
chr3R	16093906	16094271	Mhcl	36	11.9227	3.47
chr3R	16094657	16095160	Mhcl	917	6.42196	2.32
chr3R	16097565	16098813	Mhcl	416	44.1942	5.60
chr3R	16099118	16100221	Akt1	681	85.5537	8.41
chr3R	16128398	16128911	Sb	61	170.34	15.76
chr3R	16155710	16156086	CG6006	879	8.50861	2.78
chr3R	16157756	16158387	CG6006	122	252.849	17.03
chr3R	16171770	16172129	CG8925	31	43.5497	6.84
chr3R	16186781	16187606	Bin1	149	44.7083	5.27
chr3R	16186781	16187606	sra	48	44.7083	5.27
chr3R	16201384	16201724	CG6126	1473	18.1679	4.06
chr3R	16202805	16203425	CG6126	44	316.742	23.55
chr3R	16202805	16203425	CG31287	243	316.742	23.55
chr3R	16205347	16205755	mtSSB	294	46.0052	5.20
chr3R	16207132	16207462	CR45640	1269	10.9759	3.35
chr3R	16212623	16213901	CR45642	78	70.5646	9.82
chr3R	16214236	16214506	CR45641	26	7.45565	2.76
chr3R	16218100	16218541	CR45641	3847	20.3383	4.65
chr3R	16221237	16221823	CR45643	1883	24.5402	4.77
chr3R	16222259	16222753	CR45643	872	26.5466	4.39
chr3R	16224126	16225134	tara	910	316.742	28.92
chr3R	16225663	16226137	tara	251	19.7279	3.94
chr3R	16228910	16229339	tara	3487	17.5784	4.05
chr3R	16231455	16232008	tara	5986	12.7744	3.23
chr3R	16235073	16237854	tara	6207	316.742	18.37
chr3R	16239488	16240173	tara	2909	24.3346	4.06
chr3R	16240513	16241760	tara	1566	316.742	22.41
chr3R	16242181	16243115	tara	190	316.742	33.33
chr3R	16244514	16245115	tara	2063	103.832	10.31
chr3R	16247017	16248921	tara	5208	82.3264	8.74
chr3R	16249519	16250991	tara	7426	316.742	25.25
chr3R	16253095	16253469	tara	10448	22.8209	4.56
chr3R	16253812	16255341	bor	9931	36.371	5.54
chr3R	16272077	16272452	Mat89Ba	148	10.6058	2.86
chr3R	16272077	16272452	gish	184	10.6058	2.86
chr3R	16272928	16273887	gish	500	162.41	11.68
chr3R	16274620	16274997	gish	1786	49.7546	6.72
chr3R	16279928	16282411	gish	483	316.742	23.00
chr3R	16284863	16285417	gish	4166	105.374	9.66
chr3R	16305234	16306262	Zip89B	76	316.742	23.75
chr3R	16338305	16338725	Sulf1	42	33.8429	5.24
chr3R	16341647	16342075	SF2	93	24.0155	4.23
chr3R	16349107	16349432	CG14879	5	17.6046	3.74
chr3R	16349107	16349432	Manf	172	17.6046	3.74
chr3R	16355380	16355737	CG10311	1459	32.3618	5.12
chr3R	16356105	16357374	CG10311	22	316.742	18.51
chr3R	16403371	16404176	ss	0	81.2025	8.82
chr3R	16431697	16431955	CG31279	330	20.0832	3.86
chr3R	16449115	16449875	Pak3	4163	38.45	6.45
chr3R	16452764	16453969	Pak3	711	316.742	23.00
chr3R	16592566	16592922	CG14892	1611	22.276	4.58
chr3R	16604683	16604997	CG10345	16	19.0509	4.37
chr3R	16625144	16626259	Fas1	42	41.5832	5.01
chr3R	16647644	16648284	Cctgamma	396	52.0108	6.50
chr3R	16655082	16656030	modSP	338	151.74	11.95
chr3R	16702575	16703438	Ubx	31412	316.742	43.70
chr3R	16719732	16720337	Ubx	14432	203.687	18.01
chr3R	16734359	16734776	Ubx	104	68.2663	8.32
chr3R	16763462	16764559	bxd	8973	316.742	21.88
chr3R	16798547	16798966	CR45750	1810	9.09143	2.55
chr3R	16811718	16812254	CR45751	10689	103.301	13.31
chr3R	16829978	16830511	abd-A	204	220.208	17.69
chr3R	16847120	16847692	iab-8	1282	148.19	14.19
chr3R	16857555	16857991	mir-iab-8	1447	42.9912	7.65
chr3R	16869223	16869807	mir-iab-8	13155	209.241	14.36
chr3R	16882360	16882905	iab-8	9347	63.7911	8.59

chr3R	16898792	16900184	iab-8	628	266.354	18.89
chr3R	16919016	16919367	iab-8	3954	28.4374	4.64
chr3R	16920389	16920772	iab-8	2521	33.6275	6.36
chr3R	16934225	16935016	Abd-B	265	82.7846	11.36
chr3R	16948509	16949088	CR46267	3129	224.04	19.48
chr3R	16957794	16958773	Abd-B	2304	58.6334	8.54
chr3R	16969394	16970341	Abd-B	1077	58.988	6.20
chr3R	16981758	16982529	Ahcy89E	46	76.8462	9.12
chr3R	16986251	16986775	CG18622	332	16.7267	3.87
chr3R	16987427	16988147	CG18622	62	316.742	22.50
chr3R	16996368	16996700	CG3534	72	25.4096	4.63
chr3R	17002517	17003185	Irc	22	119.696	9.74
chr3R	17020732	17021202	AdSL	84	37.6147	5.43
chr3R	17053427	17054881	Dad	46	316.742	26.43
chr3R	17055969	17057061	Dad	1572	316.742	20.34
chr3R	17059004	17060167	Dad	4469	73.3219	7.88
chr3R	17064723	17065879	Ns1	7203	316.742	38.67
chr3R	17067668	17068165	Ns1	4773	35.0921	5.39
chr3R	17077782	17078396	Keap1	1	127.73	12.80
chr3R	17079187	17080261	Keap1	528	33.1458	5.07
chr3R	17079187	17080261	kuk	826	33.1458	5.07
chr3R	17081035	17081573	kuk	563	11.8142	3.01
chr3R	17082407	17082784	kuk	272	11.9329	3.23
chr3R	17116425	17116700	cher	2579	9.90867	3.05
chr3R	17118896	17119406	cher	81	316.742	19.73
chr3R	17149090	17149388	m-cup	195	16.6617	3.81
chr3R	17151740	17152093	CG5292	16	13.8647	3.36
chr3R	17264967	17265333	beat-IIb	126	37.5351	7.05
chr3R	17264967	17265333	CG44257	102	37.5351	7.05
chr3R	17366020	17366502	beat-IIa	124	50.8072	7.41
chr3R	17377565	17377949	lute	2204	37.8189	6.50
chr3R	17386007	17386514	lute	395	28.0014	4.85
chr3R	17394313	17395157	CREG	30	21.1336	4.19
chr3R	17402392	17402786	Sur-8	118	36.3954	5.89
chr3R	17505577	17506173	Dscam3	272	64.4822	9.17
chr3R	17534462	17534756	CG5823	28	26.9717	4.79
chr3R	17557332	17558030	Hmx	1193	171.404	15.28
chr3R	17558568	17558942	Hmx	154	35.0684	6.20
chr3R	17566686	17567380	Hmx	8079	96.7467	11.27
chr3R	17603703	17604180	sll	2286	20.4094	4.06
chr3R	17604504	17605649	sll	1413	22.0711	4.81
chr3R	17615847	17616287	CG14327	2229	14.6441	3.42
chr3R	17653613	17654470	CR45223	409	122.56	12.05
chr3R	17665940	17666931	CR45045	1370	156.491	15.68
chr3R	17670619	17670875	CG7523	170	9.40479	2.72
chr3R	17679302	17679860	alt	432	21.0538	4.34
chr3R	17680331	17682727	alt	991	140.216	11.76
chr3R	17680331	17682727	CG7655	1416	140.216	11.76
chr3R	17699823	17701964	CR46036	244	125.793	10.04
chr3R	17702280	17704890	CR46035	364	94.7138	8.21
chr3R	17706753	17707259	CR46035	3245	19.2889	4.06
chr3R	17711418	17711756	osa	6807	13.9734	3.79
chr3R	17712635	17713463	osa	5231	36.501	5.69
chr3R	17716835	17718608	osa	1092	316.742	39.92
chr3R	17738069	17738417	TyrRII	706	16.6683	4.45
chr3R	17738995	17739396	TyrRII	260	75.2	10.83
chr3R	17774845	17777561	CG43102	107	120.056	14.24
chr3R	17798126	17798390	CG7379	26	15.2794	3.54
chr3R	17897859	17898175	CG12347	8398	9.87475	3.19
chr3R	17900876	17901651	CG12347	5401	44.1544	7.05
chr3R	17902211	17902665	CG12347	4063	50.5792	8.44
chr3R	17911933	17912366	CG43445	768	47.0028	6.34
chr3R	17915190	17915573	Rim	1181	76.6935	9.73
chr3R	17915853	17916995	Rim	141	68.5549	9.29
chr3R	17919091	17920682	cpo	653	85.0551	11.72
chr3R	17943545	17944412	cpo	177	316.742	42.67
chr3R	17948835	17950773	cpo	5238	71.0781	7.93
chr3R	17964112	17964710	cpo	20442	28.4184	5.19
chr3R	18017334	18017650	DNaseII	1835	45.6556	6.89
chr3R	18021208	18021495	CG7785	273	19.9971	3.94
chr3R	18063232	18063614	htl	10387	45.7966	7.95
chr3R	18089115	18089804	sr	1315	149.043	12.23
chr3R	18111555	18112120	sr	10266	76.5914	9.94
chr3R	18118318	18118681	sr	3523	18.9331	4.27
chr3R	18121276	18122276	sr	138	316.742	19.87

chr3R	18160274	18160741	CR45104	617	34.9032	6.08
chr3R	18166044	18166811	Prx5	29	67.2226	8.51
chr3R	18167625	18170429	PP2A-B'	70	316.742	37.86
chr3R	18171990	18172989	PP2A-B'	552	140.019	12.96
chr3R	18196701	18199895	Ssdp	704	316.742	41.91
chr3R	18196701	18199895	CG7985	965	316.742	41.91
chr3R	18200180	18200450	CG7985	30	10.2268	2.60
chr3R	18226064	18227154	CG7168	893	44.4467	7.11
chr3R	18226064	18227154	Mdh2	580	44.4467	7.11
chr3R	18242528	18245476	14-3-3epsilon	2004	316.742	32.88
chr3R	18245818	18246428	14-3-3epsilon	3564	34.6304	5.37
chr3R	18265103	18265667	CG18599	2328	28.2806	5.30
chr3R	18274677	18275551	eIF-1A	487	316.742	25.89
chr3R	18298078	18298950	l(3)05822	486	246.757	15.38
chr3R	18310295	18312080	PKD	730	316.742	25.39
chr3R	18314181	18315782	PKD	193	316.742	19.67
chr3R	18360493	18361199	CG15803	153	111.602	10.55
chr3R	18360493	18361199	CR45634	101	111.602	10.55
chr3R	18371488	18371766	gl	1119	14.2996	4.00
chr3R	18372751	18373323	CG14310	24	16.0475	4.37
chr3R	18379456	18379762	CG7675	1081	15.535	3.38
chr3R	18380931	18381559	CG7675	149	103.43	10.54
chr3R	18396700	18399778	Vha100-2	556	316.742	33.64
chr3R	18396700	18399778	Vti1b	490	316.742	33.64
chr3R	18396700	18399778	Vha100-2	1685	316.742	33.64
chr3R	18396700	18399778	Vti1b	2224	316.742	33.64
chr3R	18402509	18402907	CG12333	11	43.4598	5.67
chr3R	18412446	18413023	CG31122	47	40.1667	6.05
chr3R	18436520	18437381	fru	1703	316.742	25.52
chr3R	18439230	18439588	fru	566	19.7632	3.65
chr3R	18445098	18445636	fru	2059	20.6741	4.27
chr3R	18447385	18447752	fru	250	59.2177	9.27
chr3R	18480720	18480979	fru	184	17.4341	4.57
chr3R	18481601	18482040	fru	1193	18.4544	4.30
chr3R	18545487	18546097	fru	58	19.5329	4.47
chr3R	18546791	18547312	fru	1450	51.6407	8.20
chr3R	18548819	18549122	fru	3369	8.08695	3.08
chr3R	18572927	18574414	fray	656	316.742	34.15
chr3R	18572927	18574414	CG7694	656	316.742	34.15
chr3R	18572927	18574414	fray	656	316.742	34.15
chr3R	18583362	18584920	CG7694	405	316.742	31.72
chr3R	18583362	18584920	fray	405	316.742	31.72
chr3R	18585266	18585931	fray	228	316.742	27.04
chr3R	18658607	18659406	CG31224	532	65.4411	7.61
chr3R	18670884	18671204	CG31230	644	22.9723	4.66
chr3R	18680367	18680736	CG7708	1	17.444	4.14
chr3R	18698958	18699589	CR45996	2042	30.5536	5.38
chr3R	18705171	18705662	ChAT	54	28.2992	5.46
chr3R	18705171	18705662	VAChT	54	28.2992	5.46
chr3R	18705171	18705662	ChAT	54	28.2992	5.46
chr3R	18739153	18740726	Mekk1	75	316.742	20.09
chr3R	18741133	18741700	Mekk1	1296	101.556	9.14
chr3R	18742525	18743770	Mekk1	742	316.742	33.34
chr3R	18798069	18798499	CG7720	3133	44.6278	7.25
chr3R	18849080	18849659	CG18208	184	151.896	13.36
chr3R	18906539	18907122	EndoA	248	36.7603	5.88
chr3R	18912689	18913264	CG14291	63	260.939	22.05
chr3R	18913781	18914257	Xrp1	930	233.602	17.45
chr3R	18914580	18915186	Xrp1	96	138.011	12.43
chr3R	18916747	18917334	Xrp1	1153	129.917	10.32
chr3R	18917855	18919278	Xrp1	74	151.507	10.65
chr3R	18922928	18924709	Xrp1	680	316.742	18.84
chr3R	18929085	18929787	CG42613	212	73.6634	9.38
chr3R	18931411	18932147	CG42613	2714	74.5581	10.28
chr3R	18935082	18935687	CG42613	6144	59.7648	8.44
chr3R	18949103	18949459	CG43732	917	31.6752	5.76
chr3R	18949103	18949459	CG42613	917	31.6752	5.76
chr3R	18949103	18949459	CG43732	917	31.6752	5.76
chr3R	18949997	18950373	CG43732	17	40.2102	7.34
chr3R	18949997	18950373	CG42613	17	40.2102	7.34
chr3R	18949997	18950373	CG43732	17	40.2102	7.34
chr3R	18983834	18984142	gukh	85	9.64745	2.65
chr3R	19017391	19018279	CG6005	6157	95.0569	10.96
chr3R	19018681	19019121	CG6005	5111	26.2963	5.62
chr3R	19023953	19024273	CG6005	74	21.1815	4.00

chr3R	19023953	19024273	CG14286	13	21.1815	4.00
chr3R	19057523	19057827	CR46041	333	13.8097	3.53
chr3R	19073043	19073492	CR46041	15076	10.6247	3.32
chr3R	19093603	19094400	cdi	42	27.3219	4.37
chr3R	19096507	19097194	CG6040	165	316.742	22.73
chr3R	19111090	19111643	CG6040	14533	18.8413	4.45
chr3R	19134861	19135512	Cyp12a4	30	300.792	21.21
chr3R	19147302	19147991	Ppcs	288	316.742	27.22
chr3R	19148762	19149098	CR44107	197	15.1049	3.65
chr3R	19153455	19153849	CG5835	25	53.8245	7.51
chr3R	19162049	19162332	Nsun5	113	11.0211	2.81
chr3R	19162049	19162332	CG42359	113	11.0211	2.81
chr3R	19163818	19165105	sqz	89	316.742	24.23
chr3R	19165516	19166164	sqz	1618	316.742	26.10
chr3R	19211311	19213408	cry	16	236.548	13.60
chr3R	19225276	19226763	vib	693	316.742	26.70
chr3R	19321255	19322559	Dl	4127	316.742	42.29
chr3R	19325971	19326786	Dl	60	197.036	14.95
chr3R	19411424	19411788	CG31221	1103	34.2762	6.51
chr3R	19412321	19412798	CG31221	117	53.7324	7.15
chr3R	19460480	19461361	Dys	408	103.179	11.99
chr3R	19463189	19464535	Dys	103	316.742	21.85
chr3R	19465589	19466017	Dys	2283	72.0504	9.52
chr3R	19476645	19476905	Dys	50	18.2248	4.31
chr3R	19495100	19495682	CG15025	4266	98.1772	10.01
chr3R	19498647	19500088	Dys	4287	112.705	10.71
chr3R	19503205	19503747	Dys	356	62.5419	8.89
chr3R	19508770	19509361	Dys	266	110.015	13.06
chr3R	19568762	19569370	Dys	128	177.814	13.35
chr3R	19620934	19621447	CG6231	27	29.9813	5.74
chr3R	19623288	19623880	CG6231	109	126.609	13.10
chr3R	19624243	19624924	CG6231	46	205.589	17.08
chr3R	19627501	19627785	CG6231	9	6.59139	2.57
chr3R	19628042	19628442	CG6231	477	63.0083	8.34
chr3R	19632559	19633139	CG6231	103	123.852	12.62
chr3R	19640924	19641650	subdued	25	316.742	20.56
chr3R	19641997	19642817	subdued	240	84.0584	8.58
chr3R	19643968	19644586	Vha13	78	52.3017	5.74
chr3R	19656448	19656916	CG6184	239	89.1968	11.47
chr3R	19671316	19671795	Naam	39	32.7609	4.90
chr3R	19719348	19719722	CG34286	3610	45.7572	7.82
chr3R	19730220	19731716	GluClalpha	118	121.829	10.57
chr3R	19781063	19781451	CG11407	103	13.999	3.79
chr3R	19786900	19787395	CG11453	3807	7.85955	2.46
chr3R	19793666	19793965	CG11453	10298	23.7601	5.28
chr3R	19802256	19802805	CG11453	19085	130.653	11.44
chr3R	19836689	19837192	bnl	120	149.135	15.78
chr3R	19859618	19860082	Ire1	1460	19.8876	4.23
chr3R	19860683	19861572	Ire1	223	292.66	16.70
chr3R	19863944	19864517	CG4572	128	33.43	3.72
chr3R	19863944	19864517	CG4686	230	33.43	3.72
chr3R	19875092	19875725	CG4562	88	80.3179	8.06
chr3R	19889215	19891583	CG4733	578	316.742	30.25
chr3R	19902817	19903534	CG4538	220	16.0954	3.48
chr3R	19905052	19905485	RhoGAP92B	6	20.1758	3.83
chr3R	20007649	20008686	Hs6st	427	229.603	15.98
chr3R	20021608	20022102	psidin	61	45.1017	5.91
chr3R	20035667	20036034	CG10889	234	19.4026	4.37
chr3R	20080124	20080704	Rh3	1996	66.7121	8.83
chr3R	20204399	20205003	Nlg4	110	118.09	13.68
chr3R	20252547	20255036	cic	1233	316.742	37.97
chr3R	20261181	20262267	cic	9114	47.1618	5.97
chr3R	20283888	20284602	cic	8959	35.1389	6.71
chr3R	20292897	20293313	cic	94	64.6896	8.76
chr3R	20309180	20310872	CG42668	146	112.068	8.96
chr3R	20311366	20311687	CG42668	2178	15.6368	3.71
chr3R	20317423	20318542	CG42668	387	48.9103	7.33
chr3R	20324452	20324799	CR45800	883	24.5502	4.01
chr3R	20327738	20328095	CG4360	46	20.6898	4.06
chr3R	20449594	20449961	Gfrl	95	27.304	5.86
chr3R	20484346	20484901	Gfrl	66	30.7009	6.18
chr3R	20546482	20547071	Stat92E	4285	29.2443	4.15
chr3R	20549742	20551264	Stat92E	1074	70.6203	8.18
chr3R	20555560	20557922	att-ORFA	1422	129.154	10.87
chr3R	20564061	20564372	CG10877	181	14.043	3.58

chr3R	20565318	20566128	CG5191	39	245.953	15.10
chr3R	20574152	20574990	snoRNA:Psi18S-301	212	92.0359	9.90
chr3R	20585461	20585833	CG16953	65	12.5445	3.41
chr3R	20592966	20593697	bon	239	62.3409	7.22
chr3R	20594262	20595170	bon	1738	316.742	23.85
chr3R	20604087	20604386	bon	11136	14.4803	3.62
chr3R	20621904	20622379	H	235	27.7919	4.64
chr3R	20634012	20634281	Pi3K92E	628	10.0736	2.82
chr3R	20658684	20658949	Nep4	802	13.0473	3.52
chr3R	20677173	20677477	CG4000	41	12.5391	3.51
chr3R	20678100	20678449	CR43452	130	17.6883	4.33
chr3R	20739841	20740387	CG42322	54	55.0107	8.03
chr3R	20741368	20741842	CG42322	1520	105.539	10.68
chr3R	20745126	20745590	CG31200	8	93.2309	9.26
chr3R	20777988	20779124	Syp	288	316.742	28.08
chr3R	20779444	20780829	Syp	475	284.838	16.93
chr3R	20781533	20785559	Syp	205	316.742	38.42
chr3R	20790375	20790781	Syp	548	26.7911	4.53
chr3R	20821825	20822231	CG17271	81	10.7923	2.69
chr3R	20821825	20822231	RpS20	251	10.7923	2.69
chr3R	20829014	20830794	AdSS	1307	316.742	24.13
chr3R	20832703	20833600	Synd	534	47.7003	5.93
chr3R	20836942	20837376	Synd	2658	13.8927	3.24
chr3R	20838708	20839396	CG15695	2996	27.9498	4.84
chr3R	20866038	20866431	CG3822	19	24.1886	4.52
chr3R	20890482	20891766	CR44947	3107	70.3707	8.31
chr3R	20897159	20897660	Ktl	78	62.577	7.48
chr3R	20913998	20914971	hdly	54	194.764	15.76
chr3R	20945453	20945946	CG31191	168	34.4192	6.07
chr3R	20948755	20950156	Atpalpha	586	316.742	20.88
chr3R	20956727	20959255	Atpalpha	671	316.742	32.27
chr3R	20959901	20960552	Atpalpha	2519	19.6281	3.97
chr3R	20960968	20961331	Atpalpha	3456	18.055	3.78
chr3R	20962806	20963385	Atpalpha	5535	18.2527	4.07
chr3R	20977711	20978371	Calx	70	76.3037	7.59
chr3R	21046803	21047106	CG31207	35	16.4465	3.82
chr3R	21048541	21049002	CG7079	49	25.4334	4.52
chr3R	21059945	21061061	Mvl	41	100.762	10.84
chr3R	21064952	21066090	AnxB9	9	255.641	14.81
chr3R	21090142	21090399	CR44178	1503	12.9593	3.67
chr3R	21096579	21097127	RhoGAP93B	968	29.6547	5.67
chr3R	21097550	21097859	RhoGAP93B	109	10.4133	2.70
chr3R	21111378	21113356	Rab11	1637	132.054	12.59
chr3R	21124114	21124589	slmb	3063	31.0549	5.41
chr3R	21126740	21128021	slmb	259	111.516	9.68
chr3R	21126740	21128021	CG5793	1	111.516	9.68
chr3R	21141307	21141651	SNF4Agamma	689	21.2771	4.87
chr3R	21152361	21153495	SNF4Agamma	199	316.742	24.75
chr3R	21180527	21180839	SNF4Agamma	124	30.1339	5.95
chr3R	21186613	21187887	SNF4Agamma	1380	201.81	16.86
chr3R	21188383	21189112	SNF4Agamma	151	278.847	18.03
chr3R	21195411	21196112	SNF4Agamma	3289	20.9075	4.56
chr3R	21198681	21199740	SNF4Agamma	49	82.1895	7.76
chr3R	21200095	21200985	SNF4Agamma	263	168.264	12.66
chr3R	21203144	21205276	SNF4Agamma	75	316.742	24.78
chr3R	21205824	21206083	SNF4Agamma	1023	22.6016	4.58
chr3R	21206516	21207059	SNF4Agamma	1688	114.391	11.82
chr3R	21223522	21224622	CG3337	805	92.0359	9.90
chr3R	21223522	21224622	Nelf-A	686	92.0359	9.90
chr3R	21223522	21224622	CR44265	200	92.0359	9.90
chr3R	21237060	21237527	e	127	28.594	5.61
chr3R	21246629	21247012	ETHR	178	19.7632	3.65
chr3R	21268172	21268438	Rab1	784	16.9468	3.67
chr3R	21268924	21269213	Rab1	26	16.9331	3.50
chr3R	21275994	21276529	CG3301	38	47.4343	6.27
chr3R	21289048	21289615	SIFaR	4663	153.964	16.59
chr3R	21293863	21294256	SIFaR	54	22.9585	4.47
chr3R	21296268	21298239	Hsromega	57	241.282	14.58
chr3R	21348475	21349883	CG16791	9	316.742	18.95
chr3R	21376227	21377487	mod(mdg4)	956	126.906	10.49
chr3R	21378683	21379118	tin	116	64.0767	8.34
chr3R	21433913	21434780	lbl	175	316.742	28.46
chr3R	21446147	21446902	lbe	39	316.742	32.94
chr3R	21460019	21460285	CR43453	5533	18.1418	4.67
chr3R	21480925	21481263	Fancm	1256	11.3088	3.33

chr3R	21499149	21500003	CR44032	233	113.517	10.03
chr3R	21499149	21500003	C15	347	113.517	10.03
chr3R	21561422	21562013	slou	4522	118.04	12.09
chr3R	21581398	21582449	InR	8427	48.3177	6.14
chr3R	21589771	21590467	InR	164	222.118	17.60
chr3R	21591163	21591456	InR	855	9.92342	2.76
chr3R	21591789	21592571	InR	278	43.2564	6.08
chr3R	21591789	21592571	CR43653	50	43.2564	6.08
chr3R	21598652	21598910	CR43653	6413	21.9567	4.65
chr3R	21604591	21606090	CR44034	10134	250.716	16.33
chr3R	21606599	21607022	CR44034	8919	42.3433	5.93
chr3R	21613233	21613643	CR44034	2296	46.0093	7.14
chr3R	21616097	21616614	CR44034	670	40.5183	5.83
chr3R	21618158	21619557	InR	587	316.742	30.67
chr3R	21626373	21627044	mir-11	4200	121.515	12.22
chr3R	21628088	21629045	E2f1	3971	14.9957	3.68
chr3R	21629786	21631257	E2f1	1945	316.742	19.35
chr3R	21632174	21633994	E2f1	460	316.742	25.23
chr3R	21632174	21633994	CR46055	449	316.742	25.23
chr3R	21632174	21633994	E2f1	80	316.742	25.23
chr3R	21659873	21660858	E2f1	425	10.7809	2.92
chr3R	21719396	21720842	CG31176	108	233.136	15.43
chr3R	21782344	21782708	tsl	26	27.621	4.67
chr3R	21794009	21794351	mir-999	4167	25.6968	5.66
chr3R	21807860	21809044	CASK	65	173.546	15.77
chr3R	21814544	21814995	CASK	6081	56.4866	9.04
chr3R	21821629	21822779	CASK	837	34.8621	5.54
chr3R	21829320	21829676	Gr93a	3025	18.44	4.50
chr3R	21848765	21849560	Gr93d	2316	39.9142	5.61
chr3R	21852359	21853104	glec	3713	25.9153	4.78
chr3R	21853621	21854567	glec	2304	316.742	23.00
chr3R	21855850	21857088	glec	34	316.742	17.84
chr3R	21874915	21875602	CG6656	85	316.742	33.31
chr3R	21930324	21930674	CG17843	16835	13.0345	3.29
chr3R	21948058	21949707	Eip93F	170	316.742	44.24
chr3R	21957925	21958224	Eip93F	8880	11.1824	2.87
chr3R	21961877	21962133	Eip93F	10420	11.7756	3.34
chr3R	21964460	21964788	Eip93F	7714	14.0334	3.80
chr3R	21965767	21966119	Eip93F	6411	9.71851	3.20
chr3R	21970603	21972636	Eip93F	209	316.742	29.65
chr3R	21985571	21985982	Eip93F	13434	10.903	3.06
chr3R	22018941	22019240	CG6439	102	8.87506	2.82
chr3R	22022893	22023161	Mitofilin	61	10.296	2.74
chr3R	22041581	22043092	how	540	316.742	28.06
chr3R	22053931	22054345	how	12013	19.8561	4.61
chr3R	22086164	22086605	CG5778	84	23.3428	4.54
chr3R	22133536	22134127	SKIP	28	56.8965	7.20
chr3R	22324091	22324673	CG34377	424	14.0411	4.07
chr3R	22356516	22357602	Sar1	362	31.4472	4.56
chr3R	22362317	22362848	PSR	288	26.897	4.26
chr3R	22362317	22362848	Muted	40	26.897	4.26
chr3R	22362317	22362848	CG7071	40	26.897	4.26
chr3R	22367185	22370071	CG5380	2461	130.47	13.15
chr3R	22367185	22370071	PyK	553	130.47	13.15
chr3R	22447927	22448291	CG34376	41	30.076	4.60
chr3R	22464708	22465119	CG7054	106	17.4391	3.63
chr3R	22469040	22469558	Nrx-1	18	13.0315	3.68
chr3R	22469943	22470550	Nrx-1	791	40.7714	6.65
chr3R	22476679	22477051	mir-4952	3977	26.1495	5.49
chr3R	22504928	22505382	Octbeta1R	7902	28.4446	5.94
chr3R	22526599	22527055	CG5346	1924	22.3929	4.48
chr3R	22527545	22528020	CG5346	974	13.9247	3.61
chr3R	22528283	22528730	CG5346	116	55.5103	7.73
chr3R	22539813	22540185	CG5326	9	20.9594	4.51
chr3R	22539813	22540185	CR45646	85	20.9594	4.51
chr3R	22570010	22570375	CG33110	43	13.0388	3.47
chr3R	22571955	22572256	CG33110	1958	12.8238	3.63
chr3R	22578904	22579485	CSN6	1609	39.818	5.92
chr3R	22587643	22588150	Efa6	56	50.8385	6.14
chr3R	22597350	22597932	Efa6	9794	13.756	3.80
chr3R	22598196	22598699	Efa6	10694	110.71	10.79
chr3R	22624757	22625236	loco	1592	27.0898	5.36
chr3R	22626067	22627494	loco	330	316.742	22.57
chr3R	22632960	22633397	loco	140	51.85	8.02
chr3R	22666254	22668199	wake	326	316.742	19.50

chr3R	22684182	22684555	Gclm	2127	26.2552	4.84
chr3R	22686156	22686702	Gclm	34	112.519	10.23
chr3R	22698160	22698847	Cyp6d4	156	210.043	17.95
chr3R	22726921	22727493	CG45049	202	127.74	11.60
chr3R	22750710	22751414	CG7029	296	16.3998	3.57
chr3R	22750710	22751414	Usp12-46	296	16.3998	3.57
chr3R	22752794	22753180	CG7029	1593	36.4994	6.07
chr3R	22753753	22755632	CG7029	1	143.16	13.71
chr3R	22767646	22767993	CG13841	108	27.4159	4.87
chr3R	22781367	22781898	CR43654	157	110.554	10.48
chr3R	22781367	22781898	CG7029	105	110.554	10.48
chr3R	22898466	22899441	klg	63	84.3983	11.15
chr3R	22998186	22998481	Nha2	246	11.9203	3.42
chr3R	23046313	23046579	CG17121	1723	14.7363	3.57
chr3R	23048019	23048499	CG17121	16	35.8167	5.57
chr3R	23048881	23049155	CG17121	853	19.8068	4.15
chr3R	23057383	23057749	CG17119	3	39.8056	6.46
chr3R	23058446	23059307	CG17119	25	177.82	15.31
chr3R	23066206	23066691	CG17111	746	28.0128	4.25
chr3R	23067040	23068202	CG17111	107	316.742	27.71
chr3R	23091639	23091968	CG17110	81	24.2141	4.71
chr3R	23091639	23091968	CG6726	28	24.2141	4.71
chr3R	23112568	23113330	Cow	84	67.4279	8.18
chr3R	23117495	23118016	Rassf	124	40.0881	5.70
chr3R	23156770	23157966	unk	575	86.0425	8.12
chr3R	23173419	23174212	Irk1	206	174.539	13.90
chr3R	23175085	23175583	Irk1	1438	19.4026	4.37
chr3R	23190198	23191574	cnc	170	316.742	26.27
chr3R	23200934	23201584	cnc	543	176.666	13.30
chr3R	23214814	23215327	cnc	494	59.6655	6.13
chr3R	23220226	23220480	cnc	1591	17.8883	4.06
chr3R	23221089	23222127	cnc	490	242.341	16.39
chr3R	23225300	23227293	cnc	94	316.742	30.35
chr3R	23238922	23240423	CG4467	91	143.704	14.60
chr3R	23248788	23249384	CG4467	166	189.701	14.05
chr3R	23278846	23279508	orb	1611	100.553	12.71
chr3R	23280674	23281226	orb	47	79.934	7.80
chr3R	23280674	23281226	Cdc16	297	79.934	7.80
chr3R	23292912	23293659	pnt	1849	316.742	25.32
chr3R	23295070	23295942	pnt	285	316.742	25.77
chr3R	23314151	23314745	pnt	121	232.487	18.57
chr3R	23323330	23323861	pnt	9384	15.7062	3.94
chr3R	23334253	23334971	CR46090	773	316.742	31.96
chr3R	23345949	23347876	pnt	96	179.148	12.97
chr3R	23372487	23372778	CR44322	46	10.155	3.26
chr3R	23374304	23375154	CG42828	892	23.1465	4.00
chr3R	23383633	23384162	CG4393	950	88.992	10.10
chr3R	23384535	23385596	CG4393	420	157.804	12.57
chr3R	23385950	23386498	CG4393	1448	126.494	8.99
chr3R	23419694	23419996	CG43694	6253	13.8304	3.75
chr3R	23472080	23473122	CG4374	452	316.742	21.32
chr3R	23519376	23519705	Irk2	117	18.4482	4.06
chr3R	23557158	23557574	beat-IV	65	50.3686	7.61
chr3R	23601386	23601933	CG10183	2685	49.9213	6.45
chr3R	23654141	23654726	CG31145	15325	35.8515	5.21
chr3R	23666884	23667464	CG31145	2616	50.5791	7.27
chr3R	23667820	23668148	CG31145	1710	12.0718	2.87
chr3R	23668546	23668835	CG31145	945	17.4126	3.85
chr3R	23669342	23670538	CG31145	481	316.742	32.97
chr3R	23686024	23687010	SPE	476	208.953	20.30
chr3R	23722546	23724181	CG10365	581	316.742	27.35
chr3R	23727991	23728603	CG10365	178	142.375	13.96
chr3R	23729874	23730395	RNaseMRP:RNA	231	11.6945	3.14
chr3R	23773867	23774250	CG10208	35	36.6094	6.01
chr3R	23781528	23783390	Nup98-96	1021	120.687	10.17
chr3R	23781528	23783390	mbc	878	120.687	10.17
chr3R	23786704	23787297	mbc	5227	35.1018	5.27
chr3R	23818631	23819427	CG33111	156	296.886	18.82
chr3R	23818631	23819427	eIF4G2	156	296.886	18.82
chr3R	23818631	23819427	CG33111	262	296.886	18.82
chr3R	23818631	23819427	eIF4G2	262	296.886	18.82
chr3R	23818631	23819427	CG33111	262	296.886	18.82
chr3R	23859375	23859875	snRNA:U1:21D	47	72.9341	8.05
chr3R	23859375	23859875	snRNA:U1:95Cb	47	72.9341	8.05
chr3R	23859375	23859875	snRNA:U1:95Ca	47	72.9341	8.05

chr3R	23886964	23887617	Pli	2673	54.6282	6.91
chr3R	23889794	23890197	Pli	49	20.6603	3.74
chr3R	23909714	23910039	sba	13139	15.5784	3.60
chr3R	23912925	23913450	sba	9730	12.8033	3.37
chr3R	23921861	23923127	sba	189	316.742	22.10
chr3R	23933962	23934334	CG5854	137	9.73415	2.86
chr3R	23942849	23943445	Gdh	54	316.742	18.47
chr3R	23948280	23948614	CG12268	65	8.7402	2.91
chr3R	23951201	23951473	CG12268	2890	12.9814	3.46
chr3R	23958287	23958729	Aats-glupro	39	13.9129	3.46
chr3R	23958287	23958729	AP-1sigma	220	13.9129	3.46
chr3R	23962729	23963390	CG31140	207	176.44	17.47
chr3R	23963734	23964400	CG31140	756	30.0512	5.84
chr3R	23965020	23965674	CG31140	2227	91.0422	10.14
chr3R	23986483	23987038	CG13603	241	41.7978	6.19
chr3R	23986483	23987038	CG5902	169	41.7978	6.19
chr3R	23992790	23993753	CG13604	221	41.7114	5.70
chr3R	23992790	23993753	Rab7	41	41.7114	5.70
chr3R	24006341	24007267	KrT95D	106	316.742	26.50
chr3R	24008150	24008436	KrT95D	1747	14.9793	3.44
chr3R	24031036	24031978	KrT95D	399	316.742	28.17
chr3R	24037544	24037877	Miro	22	16.9814	3.63
chr3R	24038145	24038514	spas	79	23.094	4.55
chr3R	24046451	24048426	Rox8	34	201.036	14.37
chr3R	24046451	24048426	CG5986	455	201.036	14.37
chr3R	24046451	24048426	Rox8	656	201.036	14.37
chr3R	24046451	24048426	CG5986	963	201.036	14.37
chr3R	24050666	24051093	Atg6	87	40.3887	5.45
chr3R	24050666	24051093	CG5991	87	40.3887	5.45
chr3R	24051547	24051851	CG5991	8	22.4092	4.48
chr3R	24057139	24057553	Hsp68	41	48.8687	7.23
chr3R	24092566	24093459	Syx1A	12974	76.3813	8.50
chr3R	24104663	24106392	Syx1A	788	119.664	9.88
chr3R	24108657	24109217	4EHP	25	27.6152	4.51
chr3R	24140927	24141568	Kal1	34	67.4523	9.67
chr3R	24152312	24152786	p38a	236	42.4062	5.41
chr3R	24152312	24152786	CG6178	211	42.4062	5.41
chr3R	24218512	24219181	CG17786	173	316.742	21.71
chr3R	24221018	24222138	cav	873	37.3645	5.45
chr3R	24221018	24222138	twin	540	37.3645	5.45
chr3R	24221018	24222138	cav	445	37.3645	5.45
chr3R	24221018	24222138	twin	10	37.3645	5.45
chr3R	24230555	24231493	mask	264	53.059	6.27
chr3R	24230555	24231493	CR44092	360	53.059	6.27
chr3R	24230555	24231493	CR45217	539	53.059	6.27
chr3R	24233536	24233921	mask	1363	62.7912	8.11
chr3R	24234333	24234672	mask	642	16.7357	3.20
chr3R	24259118	24260827	jar	79	316.742	30.93
chr3R	24263669	24264913	jar	45	41.6379	6.56
chr3R	24270979	24271449	jar	25	68.1794	7.80
chr3R	24272423	24273365	Orct2	100	69.1758	8.67
chr3R	24276295	24276963	Orct	2	111.313	12.76
chr3R	24279384	24279656	CG13611	59	15.7851	4.09
chr3R	24283812	24284065	CG6356	8	9.05671	3.13
chr3R	24294877	24296178	crb	831	252.81	15.63
chr3R	24318916	24320690	BRWD3	1565	24.3346	4.06
chr3R	24318916	24320690	CR46093	782	24.3346	4.06
chr3R	24348421	24348794	CG5746	142	12.4763	3.30
chr3R	24457034	24458003	nAChRalpha1	180	78.7625	10.01
chr3R	24485000	24486087	nAChRalpha2	222	101.299	10.31
chr3R	24514615	24515133	nAChRbeta2	274	23.2138	5.07
chr3R	24518261	24518747	Pp1alpha-96A	6	5.55156	2.31
chr3R	24543026	24544055	CG5789	57	78.5812	8.42
chr3R	24544382	24544923	Esyt2	110	84.3417	8.79
chr3R	24548765	24549178	Esyt2	23	80.1568	8.83
chr3R	24555515	24555957	snRNA:U6:96Ac	399	13.3134	3.47
chr3R	24555515	24555957	snRNA:U6:96Ab	399	13.3134	3.47
chr3R	24555515	24555957	snRNA:U6:96Aa	399	13.3134	3.47
chr3R	24556873	24557468	snRNA:U6:96Ac	79	84.1719	8.22
chr3R	24556873	24557468	snRNA:U6:96Ab	79	84.1719	8.22
chr3R	24556873	24557468	snRNA:U6:96Aa	79	84.1719	8.22
chr3R	24565443	24566294	REPTOR	6093	316.742	28.48
chr3R	24566614	24567155	REPTOR	5164	95.3197	10.66
chr3R	24571649	24573220	REPTOR	265	316.742	27.23
chr3R	24577394	24577717	REPTOR	55	19.1872	4.39

chr3R	24581840	24583029	REPTOR	815	87.5762	8.11
chr3R	24586197	24587033	mld	1051	316.742	31.83
chr3R	24588788	24589414	mld	61	264.81	15.06
chr3R	24625604	24626146	atl	129	21.9943	4.00
chr3R	24626682	24626982	atl	1142	19.4612	4.20
chr3R	24629842	24630869	Wsck	453	244.2	17.54
chr3R	24629842	24630869	atl	62	244.2	17.54
chr3R	24649686	24650250	puf	13	78.6763	8.60
chr3R	24649686	24650250	ash2	94	78.6763	8.60
chr3R	24651004	24651764	ash2	59	316.742	35.05
chr3R	24662178	24662676	slo	22	13.9567	3.84
chr3R	24719877	24720251	tok	3	35.7603	5.43
chr3R	24767537	24769790	CR45650	258	316.742	23.52
chr3R	24767537	24769790	CG13631	608	316.742	23.52
chr3R	24770585	24771635	CG42331	822	215.768	17.25
chr3R	24787077	24787484	CG5805	136	17.6934	3.86
chr3R	24798767	24800014	Ude	13	316.742	36.62
chr3R	24813757	24814034	CG5807	61	16.2756	3.60
chr3R	24860204	24860940	CG13639	143	215.156	14.62
chr3R	24862573	24863235	CG7016	59	316.742	21.02
chr3R	24875945	24876739	CG3744	468	40.1874	5.98
chr3R	24894323	24895404	CG31121	95	316.742	35.64
chr3R	24894323	24895404	CG11069	41	316.742	35.64
chr3R	24944399	24945081	ssh	9	11.6946	3.06
chr3R	24984184	24984461	tnc	72	10.8025	3.36
chr3R	24985184	24985751	tnc	187	94.2217	11.17
chr3R	25025100	25025610	Hr96	187	23.0297	4.31
chr3R	25033065	25033534	Smg6	200	14.7192	3.44
chr3R	25037987	25038506	CG6422	590	17.3238	3.74
chr3R	25047521	25047813	CG11790	45	20.2486	4.30
chr3R	25049605	25050555	CG11791	95	251.226	16.71
chr3R	25056745	25057248	Unc-13-4B	64	116.218	10.58
chr3R	25080364	25080897	fd96Ca	2072	85.2416	8.92
chr3R	25094512	25095129	fd96Cb	156	103.335	9.62
chr3R	25122492	25123015	CG31108	165	56.626	6.34
chr3R	25122492	25123015	CG31510	165	56.626	6.34
chr3R	25137711	25138162	danr	159	86.9989	10.29
chr3R	25196580	25197260	CG13653	1508	70.593	9.35
chr3R	25199507	25199983	CG13654	19	51.2862	6.89
chr3R	25205200	25205972	Cad96Ca	2590	15.8396	3.56
chr3R	25207343	25208885	Cad96Ca	119	316.742	40.79
chr3R	25219052	25219723	CG31103	28	130.374	12.10
chr3R	25247258	25247623	RpS27	10	12.0506	3.06
chr3R	25258702	25259349	CG11857	28	56.5488	6.99
chr3R	25265597	25265967	Aats-gln	30	14.5646	3.41
chr3R	25265597	25265967	RIOK2	92	14.5646	3.41
chr3R	25292693	25293221	CG31098	7	143.003	11.50
chr3R	25295210	25295811	CG31102	31	74.8234	10.47
chr3R	25303918	25304374	CG31436	34	137.501	12.79
chr3R	25305648	25306047	CG31436	1720	30.4911	4.66
chr3R	25308239	25308553	CG10550	7	42.4062	5.41
chr3R	25315929	25316401	CG10559	71	72.5432	7.76
chr3R	25322540	25322919	CG10562	2	18.8892	3.55
chr3R	25328422	25330125	CHKov1	549	316.742	37.83
chr3R	25331025	25331351	CHKov2	31	25.1315	4.22
chr3R	25438354	25438653	Fur1	34547	21.7323	4.60
chr3R	25471879	25473277	Fur1	334	267.182	15.83
chr3R	25482325	25483024	XNP	27	121.334	10.68
chr3R	25490605	25491019	Dhap-at	8	17.083	3.79
chr3R	25491337	25491718	CG5112	6	20.1186	4.27
chr3R	25515315	25516403	msi	464	316.742	35.17
chr3R	25517928	25518516	msi	100	192.903	14.08
chr3R	25526653	25527031	msi	8549	22.0038	4.59
chr3R	25532723	25533145	msi	14629	43.899	7.10
chr3R	25588378	25589925	mir-1000	492	54.7032	7.77
chr3R	25588378	25589925	CR45910	271	54.7032	7.77
chr3R	25613508	25613800	CG5079	46	17.9011	4.31
chr3R	25626724	25627031	Ssadh	14	20.6194	4.04
chr3R	25646833	25648314	jigr1	97	254.294	13.62
chr3R	25652168	25652444	mir-92b	882	21.104	3.53
chr3R	25661766	25662199	RASSF8	14	9.09143	2.55
chr3R	25719578	25720301	LpR2	8	33.2496	5.20
chr3R	25735449	25736237	CR46102	2344	123.786	10.33
chr3R	25740218	25741029	LpR2	178	316.742	27.00
chr3R	25752780	25753166	LpR1	14	24.6015	4.92

chr3R	25756602	25757116	LpR1	3582	16.1587	3.98
chr3R	25760055	25760758	LpR1	116	316.742	27.49
chr3R	25782957	25783315	CG4960	4136	76.7957	9.75
chr3R	25855903	25856266	CG5890	28	22.2828	4.40
chr3R	25877517	25878012	Tsp96F	3674	12.5966	3.20
chr3R	25880300	25881579	Tsp96F	597	116.14	11.24
chr3R	25882015	25882386	SppL	88	11.1242	3.15
chr3R	25883918	25884267	SppL	1528	31.3617	5.61
chr3R	25888334	25889095	Lnk	219	28.4877	4.36
chr3R	25893377	25893682	CG5913	28	13.8687	3.45
chr3R	25969855	25970211	CG42261	87	25.2963	4.66
chr3R	25983168	25983715	Nf1	437	161.911	12.56
chr3R	26005806	26006656	E(spl)mbeta-HLH	100	128.394	11.21
chr3R	26007053	26007398	CR45039	196	10.3591	3.02
chr3R	26008141	26009043	CR45039	1354	140.919	12.99
chr3R	26017276	26017854	E(spl)m2-BFM	93	84.9881	10.38
chr3R	26020526	26022001	CR43644	662	253.52	18.64
chr3R	26020526	26022001	E(spl)m3-HLH	84	253.52	18.64
chr3R	26036806	26037094	E(spl)m7-HLH	77	20.2088	4.12
chr3R	26041584	26043014	gro	49	316.742	29.61
chr3R	26055176	26055544	Dak1	80	26.2698	4.40
chr3R	26098445	26099657	dysf	563	97.1587	9.76
chr3R	26105631	26107577	CG31324	80	130.953	10.37
chr3R	26117401	26117969	CR46060	7735	250.907	17.09
chr3R	26224756	26225082	CG12290	5371	17.2612	3.93
chr3R	26227189	26227471	CG12290	2908	11.597	2.82
chr3R	26229430	26230520	CG12290	67	316.742	29.34
chr3R	26250036	26250367	Ald	8130	12.4894	3.61
chr3R	26258441	26259288	Ald	570	225.303	17.70
chr3R	26260556	26261710	CR44321	7	214.327	14.71
chr3R	26260556	26261710	Ald	504	214.327	14.71
chr3R	26262147	26262841	CG6154	17	44.1161	6.68
chr3R	26318075	26318622	CG33970	49	25.7691	4.81
chr3R	26325831	26326238	CR42765	693	18.8895	4.03
chr3R	26347859	26348249	CG6036	2670	8.72603	3.09
chr3R	26438277	26438660	tx	2301	15.4439	3.83
chr3R	26440329	26441203	tx	150	135.958	12.43
chr3R	26461099	26461929	CG5455	74	316.742	26.74
chr3R	26462206	26462841	CG5455	1033	316.742	34.13
chr3R	26464513	26464845	CG5455	840	11.7794	3.04
chr3R	26534118	26537400	plum	1966	207.717	13.72
chr3R	26534118	26537400	scrib	834	207.717	13.72
chr3R	26541312	26541634	CR46084	1706	27.0802	5.00
chr3R	26543159	26543443	CR46084	103	12.2343	3.49
chr3R	26547552	26548207	CR46084	4767	19.1687	3.98
chr3R	26566811	26567243	scrib	1598	42.1964	5.66
chr3R	26567620	26569067	scrib	104	316.742	22.56
chr3R	26681868	26682340	beat-VII	104	64.3803	8.11
chr3R	26713906	26714445	amon	26	18.9036	4.21
chr3R	26747949	26748356	CR45562	9796	53.14	7.86
chr3R	26797753	26800158	Tl	774	316.742	16.91
chr3R	26801123	26802035	Tl	2404	175.05	12.99
chr3R	26862518	26863270	Lerp	92	137.054	10.21
chr3R	26866533	26867575	ball	62	49.5976	5.85
chr3R	26866533	26867575	His2Av	59	49.5976	5.85
chr3R	26875338	26875794	Rb97D	83	10.0788	2.82
chr3R	26881269	26881899	T48	209	53.1251	6.82
chr3R	26896120	26896529	T48	14670	14.0843	3.81
chr3R	26915628	26915962	sda	37	24.6023	4.60
chr3R	26916648	26917111	sda	1108	50.8071	7.91
chr3R	26958287	26958729	CG6330	8	36.0308	5.90
chr3R	26960524	26960966	CG6330	2240	35.1018	5.27
chr3R	26966888	26967404	CG5521	275	33.8783	5.13
chr3R	26980216	26980727	CG14253	55	138.928	14.27
chr3R	26988202	26988992	CG31075	6	122.22	11.17
chr3R	27067447	27067781	CG43935	2148	22.697	4.67
chr3R	27069635	27070489	spz	73	316.742	33.07
chr3R	27070947	27071541	spz	1147	73.8892	7.67
chr3R	27086670	27086990	Cpr97Ea	219	23.7295	5.19
chr3R	27091056	27091457	Cpr97Eb	82	20.8105	4.18
chr3R	27094318	27094773	CG14259	27	72.4897	9.27
chr3R	27111831	27112583	gb	188	40.2093	6.13
chr3R	27111831	27112583	CG5815	483	40.2093	6.13
chr3R	27129398	27130125	CG6059	4972	176.692	14.32
chr3R	27149659	27152191	CG6051	825	316.742	30.89

chr3R	27149659	27152191	Men-b	1417	316.742	30.89
chr3R	27153090	27153462	Men-b	1615	40.0501	5.89
chr3R	27157007	27158230	grass	231	242.52	17.90
chr3R	27160445	27161435	CG5909	470	129.798	11.61
chr3R	27193992	27194600	Ser	38	183.62	16.00
chr3R	27244489	27245847	CG18766	1	179.104	12.37
chr3R	27244489	27245847	CR46085	82	179.104	12.37
chr3R	27246519	27246843	CG5984	1325	33.3622	6.30
chr3R	27253368	27253662	TfIIA-L	42	14.4274	3.47
chr3R	27263473	27264193	woc	371	30.4083	5.28
chr3R	27270303	27270762	l(3)mbt	2726	24.6114	4.43
chr3R	27274799	27275474	CG5938	107	19.1689	3.56
chr3R	27279623	27280045	mrt	593	33.0108	5.49
chr3R	27280761	27282715	mrt	381	316.742	27.03
chr3R	27288235	27289056	bigmax	39	16.4422	3.72
chr3R	27288235	27289056	Hmu	281	16.4422	3.72
chr3R	27292076	27292372	CG3348	22	12.9883	3.51
chr3R	27437347	27437772	side	64	18.6255	4.21
chr3R	27463019	27463374	CG13978	966	16.0475	4.37
chr3R	27558925	27559323	Klp98A	13	22.9002	4.24
chr3R	27561036	27561428	CG5646	20	21.0603	4.34
chr3R	27579528	27579800	wdb	1854	15.6621	3.62
chr3R	27580043	27582331	wdb	212	316.742	21.58
chr3R	27605181	27605761	dsd	2072	13.0316	3.46
chr3R	27607235	27607502	dsd	10	15.6621	3.62
chr3R	27608072	27608607	Tusp	4	38.8796	5.33
chr3R	27635980	27636982	CG5611	280	258.474	17.44
chr3R	27635980	27636982	Mtl	71	258.474	17.44
chr3R	27654384	27655262	tau	2137	58.5422	6.26
chr3R	27656596	27657276	tau	119	34.4626	5.00
chr3R	27662061	27663076	unc80	86	40.8008	7.15
chr3R	27677376	27677797	CG5590	77	16.6485	3.45
chr3R	27699576	27700524	ALiX	80	94.956	9.70
chr3R	27699576	27700524	btz	330	94.956	9.70
chr3R	27736232	27737198	Ets98B	61	316.742	19.22
chr3R	27742371	27743431	Ets98B	218	39.1275	5.43
chr3R	27905711	27906233	CG34353	153	60.0564	8.19
chr3R	27922707	27923070	RpL4	21	24.3164	4.73
chr3R	27924759	27926073	mino	383	316.742	27.81
chr3R	27924759	27926073	CR45566	205	316.742	27.81
chr3R	27924759	27926073	mino	91	316.742	27.81
chr3R	27947759	27948120	mfrn	71	15.5019	3.90
chr3R	27948542	27948982	Gp93	68	91.1543	9.30
chr3R	27987255	27989518	betaTub97EF	849	316.742	30.36
chr3R	28025084	28027012	CG34362	447	103.387	12.16
chr3R	28047224	28047718	CG34362	11	30.5821	5.54
chr3R	28146336	28146921	CG34354	324	75.7022	9.44
chr3R	28324772	28325256	larp	1229	151.906	13.95
chr3R	28325913	28327178	larp	89	316.742	17.90
chr3R	28328183	28328784	larp	99	88.0499	8.38
chr3R	28330621	28331408	larp	339	242.818	14.75
chr3R	28332025	28332365	larp	1003	38.7283	5.94
chr3R	28335300	28336612	larp	191	52.4933	6.58
chr3R	28339489	28340587	Gfat2	35	316.742	31.03
chr3R	28339489	28340587	Moca-cyp	533	316.742	31.03
chr3R	28348509	28348881	Or98b	3569	26.5164	5.32
chr3R	28417941	28418305	beat-VI	28	20.3193	4.97
chr3R	28544516	28545160	CG12413	41	21.8927	4.70
chr3R	28585236	28585804	fkh	247	60.9276	7.70
chr3R	28600333	28600612	Hrb98DE	76	14.1844	3.22
chr3R	28634204	28635356	CG10011	513	137.172	10.60
chr3R	28654555	28654974	CG31050	4294	73.8299	9.00
chr3R	28672094	28672617	Sid	1694	131.097	10.71
chr3R	28681850	28682553	CG9990	96	144.698	12.39
chr3R	28683353	28683925	CG9990	1451	8.19301	2.71
chr3R	28690563	28690906	CG9990	1012	12.0918	3.46
chr3R	28735321	28735836	AstA-R2	9208	31.1266	6.08
chr3R	28811061	28811407	wdn	99	13.6351	3.20
chr3R	28830946	28831937	WASp	59	90.1063	10.75
chr3R	28844305	28845464	Apc	527	40.4212	4.91
chr3R	28844305	28845464	spg	6	40.4212	4.91
chr3R	28850111	28850529	spg	4956	29.9462	4.68
chr3R	28857630	28859703	Inx3	91	316.742	36.80
chr3R	28860527	28861953	Inx3	1963	316.742	38.85
chr3R	28879888	28880487	CG14523	31	70.7619	8.95

chr3R	28883439	28883798	CG14526	112	37.7441	6.49
chr3R	28888739	28889856	Doa	514	70.0158	6.79
chr3R	28891650	28892210	Doa	16	52.9048	6.78
chr3R	28970201	28970712	DIP-gamma	43	52.3432	7.70
chr3R	28970201	28970712	CR45669	125	52.3432	7.70
chr3R	28990026	28991066	mir-4947	17999	85.9925	10.34
chr3R	29039014	29040389	Slu7	1014	73.2334	7.58
chr3R	29039014	29040389	Pkc98E	256	73.2334	7.58
chr3R	29062866	29065142	Cul5	1777	316.742	35.07
chr3R	29062866	29065142	CG11873	925	316.742	35.07
chr3R	29069601	29070003	CG11873	6009	23.1017	4.68
chr3R	29073821	29074618	CG11873	10342	287.943	16.27
chr3R	29083872	29084520	CG11873	4075	79.7819	9.54
chr3R	29087209	29089065	CG11873	73	139.896	14.04
chr3R	29122613	29123628	yem	483	34.4378	5.75
chr3R	29122613	29123628	CG11880	7	34.4378	5.75
chr3R	29138712	29139030	CG14516	32	19.2173	3.99
chr3R	29148144	29148565	CG11882	221	22.3763	4.74
chr3R	29148144	29148565	Pglym78	32	22.3763	4.74
chr3R	29157233	29158530	CG11897	21	316.742	22.95
chr3R	29194849	29195282	CG14509	27	20.4943	4.54
chr3R	29199850	29200756	CG11899	94	87.6915	9.98
chr3R	29206505	29207794	Cyt-c1L	2634	316.742	20.90
chr3R	29214004	29216157	CR31044	566	218.288	15.02
chr3R	29214004	29216157	mir-279	457	218.288	15.02
chr3R	29229155	29229490	Usp1	90	9.47267	2.84
chr3R	29238555	29238993	CG11951	21	88.3075	8.96
chr3R	29244330	29244765	CG31445	53	217.364	20.72
chr3R	29250194	29250928	SP1029	84	259.815	17.18
chr3R	29318635	29319667	Cnx99A	158	22.7232	4.31
chr3R	29377109	29377766	Ptp99A	276	72.6134	9.87
chr3R	29457190	29458507	Ptp99A	84	270.82	18.91
chr3R	29478993	29479692	Ptp99A	85	197.939	17.03
chr3R	29493228	29493660	CG2310	56	28.5967	5.02
chr3R	29503429	29503704	Trc8	55	12.1207	3.00
chr3R	29555850	29556546	Dr	184	316.742	23.42
chr3R	29571910	29572482	Dr	15845	28.332	5.39
chr3R	29591676	29592475	alph	271	52.185	6.27
chr3R	29618537	29619414	CG1907	79	316.742	21.60
chr3R	29659930	29660305	Dop1R2	145	16.4023	4.23
chr3R	29681578	29682815	CG7582	76	316.742	23.89
chr3R	29681578	29682815	Gycalpha99B	148	316.742	23.89
chr3R	29691005	29691555	dmrt99B	1282	260.743	21.37
chr3R	29716965	29717370	Obp99b	229	26.4821	4.21
chr3R	29718582	29718889	CG15506	116	15.3558	3.70
chr3R	29723130	29723740	Pcd	283	91.3273	7.37
chr3R	29725690	29726791	Kul	614	127.81	12.35
chr3R	29736657	29737004	CG7601	142	15.1029	3.65
chr3R	29736657	29737004	CIA30	77	15.1029	3.65
chr3R	29748663	29749340	ATPsyngamma	13	14.7001	3.43
chr3R	29748663	29749340	yata	362	14.7001	3.43
chr3R	29759942	29760356	CR44953	1101	128.498	15.40
chr3R	29761694	29762023	CR44953	2819	31.292	5.94
chr3R	29764025	29764684	kay	1515	66.5543	9.12
chr3R	29771528	29775044	kay	161	316.742	38.93
chr3R	29778426	29779051	fig	1797	138.634	13.20
chr3R	29782018	29783021	kay	88	316.742	25.54
chr3R	29788496	29789150	kay	194	198.544	17.27
chr3R	29802783	29803174	CG7834	8	25.8391	4.64
chr3R	29806760	29807427	ca	472	16.7979	3.74
chr3R	29806760	29807427	ncd	155	16.7979	3.74
chr3R	29806760	29807427	ca	45	16.7979	3.74
chr3R	29829272	29829712	Atg16	3824	18.252	4.13
chr3R	29841190	29841989	Cad99C	28	104.608	10.84
chr3R	29868602	29868966	Zip99C	29	13.8698	3.32
chr3R	29876240	29877443	CG31038	2	316.742	40.47
chr3R	29877876	29878548	CG31038	1121	72.0038	7.28
chr3R	29927086	29927463	CG15522	50	29.3832	5.69
chr3R	29934599	29934869	eIF2B-alpha	1141	11.2142	2.97
chr3R	29997875	29998226	CG11504	161	15.2096	3.44
chr3R	30014472	30015379	CG7920	53	46.2298	5.67
chr3R	30021839	30022948	Mgat2	27	192.723	12.50
chr3R	30021839	30022948	RpS28a	454	192.723	12.50
chr3R	30021839	30022948	Axn	647	192.723	12.50
chr3R	30046104	30046453	RpL32	20	11.9505	2.59

chr3R	30058225	30058559	sima	74	23.9297	3.96
chr3R	30064909	30065196	sima	2127	13.5986	3.49
chr3R	30084359	30084649	sima	15017	23.1387	4.76
chr3R	30090126	30090793	sima	9020	116.768	11.88
chr3R	30095537	30095914	sima	3782	22.4629	4.90
chr3R	30098247	30098526	sima	1105	18.3114	3.92
chr3R	30099115	30099863	sima	93	316.742	36.13
chr3R	30100342	30101052	sima	1292	40.2833	5.86
chr3R	30105165	30105424	CR46112	1732	17.6173	4.31
chr3R	30108415	30108677	CR46112	1531	12.1935	3.33
chr3R	30114482	30114771	CR46112	7632	23.0009	4.31
chr3R	30134010	30134716	Tpi	52	39.8854	5.37
chr3R	30150498	30150879	AdoR	1116	30.556	5.24
chr3R	30151357	30152419	CG15529	246	27.4159	4.87
chr3R	30160279	30160769	CG11498	85	63.5953	9.73
chr3R	30170509	30171237	CG31028	533	35.6411	6.72
chr3R	30201006	30201503	CG9743	1724	19.678	4.37
chr3R	30202678	30204181	CG9743	797	316.742	26.96
chr3R	30214641	30215293	CecB	1352	24.2103	3.96
chr3R	30237683	30237951	CG43448	98	21.5423	4.56
chr3R	30243418	30243818	CG9737	297	14.2841	3.24
chr3R	30246602	30246967	CG9733	90	16.5691	3.84
chr3R	30271036	30271412	CR46113	2272	20.0809	4.45
chr3R	30272773	30273211	CR46113	637	27.8114	5.52
chr3R	30277848	30278186	hdc	36	22.9723	4.66
chr3R	30281808	30282330	hdc	4151	23.8303	4.46
chr3R	30282682	30282954	hdc	4860	15.5881	3.56
chr3R	30293310	30293669	CG34300	13186	52.3385	6.91
chr3R	30296623	30297109	CG34300	9760	52.6469	6.60
chr3R	30325737	30326278	CG34300	19433	66.4818	9.85
chr3R	30337957	30338929	hdc	19575	316.742	27.83
chr3R	30344067	30345222	hdc	13321	225.602	18.85
chr3R	30345538	30345916	hdc	12131	19.3343	4.64
chr3R	30386505	30389569	Fer1HCH	1681	316.742	25.23
chr3R	30386505	30389569	Fer2LCH	1497	316.742	25.23
chr3R	30386505	30389569	Fer1HCH	1122	316.742	25.23
chr3R	30386505	30389569	Fer2LCH	162	316.742	25.23
chr3R	30427952	30428243	CG2218	13	13.3157	3.30
chr3R	30449874	30451229	aralar1	62	316.742	50.42
chr3R	30452687	30454617	aralar1	680	316.742	23.33
chr3R	30465658	30466311	PH4alphaEFB	71	316.742	16.27
chr3R	30526663	30527846	jdp	54	316.742	21.04
chr3R	30532200	30532663	tmod	167	104.279	12.61
chr3R	30563111	30563970	tmod	489	185.528	14.73
chr3R	30587033	30587731	CG9717	156	144.206	11.66
chr3R	30599732	30601089	CG2246	495	316.742	19.62
chr3R	30616136	30617852	CR45571	4439	105.419	12.70
chr3R	30764026	30764586	zfh1	1601	189.147	16.52
chr3R	30765457	30766129	zfh1	184	149.016	14.60
chr3R	30781485	30782274	zfh1	1262	86.2342	10.62
chr3R	30800440	30800770	wts	5981	21.5869	4.51
chr3R	30803053	30804522	wts	3016	316.742	25.74
chr3R	30805624	30806679	wts	678	316.742	21.32
chr3R	30808410	30809203	cindr	68	51.8043	6.54
chr3R	30815076	30815580	cindr	2300	59.8715	7.69
chr3R	30821958	30822522	cindr	175	59.1048	7.56
chr3R	30827514	30827950	CG15544	50	31.5835	5.17
chr3R	30874604	30875148	CG15546	1231	55.3422	7.70
chr3R	30876074	30876529	CG15546	25	15.534	3.42
chr3R	30880742	30881402	CG12071	163	96.5014	10.87
chr3R	30888124	30889186	Sap-r	58	86.1179	7.56
chr3R	30909001	30909361	Ptx1	3563	23.7856	4.62
chr3R	30912211	30912836	Ptx1	214	316.742	21.19
chr3R	31016736	31017197	5-HT7	72	69.8943	7.99
chr3R	31058760	31059019	dco	2328	17.0112	3.88
chr3R	31059393	31059888	dco	1528	59.8898	7.00
chr3R	31060189	31061699	dco	273	64.5592	7.12
chr3R	31086381	31086713	CG11317	10813	22.4583	3.90
chr3R	31097120	31097565	CG11317	12	73.4654	10.31
chr3R	31119795	31120158	Prosalpha3T	3448	32.1474	5.69
chr3R	31130222	31130612	CG15556	20	35.1117	6.04
chr3R	31134087	31134508	Gycbeta100B	139	72.628	8.51
chr3R	31168478	31169287	stops	19	45.7966	7.95
chr3R	31218389	31219712	ATPsynC	429	35.8344	4.72
chr3R	31218389	31219712	mir-4949	108	35.8344	4.72

chr3R	31218389	31219712	ATPsynC	79	35.8344	4.72
chr3R	31220594	31223109	CG12054	1084	102.329	9.56
chr3R	31310413	31311336	CG34347	139	73.0528	10.63
chr3R	31381654	31382224	CG1607	64	32.2279	4.93
chr3R	31384073	31384656	CG1607	18	183.103	14.38
chr3R	31405086	31405904	Gprk2	154	15.8127	3.43
chr3R	31405086	31405904	CG11337	54	15.8127	3.43
chr3R	31437662	31437942	lox	22109	8.21781	2.55
chr3R	31441780	31442166	lox	17919	18.1167	4.05
chr3R	31457993	31458555	lox	1540	36.65	5.17
chr3R	31462780	31463327	CG11334	169	14.4384	3.56
chr3R	31462780	31463327	CG11333	94	14.4384	3.56
chr3R	31592666	31593075	CG1774	62	69.7793	8.88
chr3R	31605871	31606201	CycG	3793	16.341	3.85
chr3R	31607879	31609285	CycG	746	137.807	10.75
chr3R	31609640	31609988	CycG	28	25.5276	4.11
chr3R	31610394	31611629	CycG	152	14.462	2.99
chr3R	31610394	31611629	Med	85	14.462	2.99
chr3R	31621797	31622307	kek6	106	101.951	11.39
chr3R	31672431	31673440	CG1815	3285	61.2302	6.73
chr3R	31688889	31689848	CR45565	572	316.742	31.18
chr3R	31709173	31709604	CR34046	19	8.02872	2.74
chr3R	31711424	31714312	ttk	279	316.742	45.71
chr3R	31714544	31714966	ttk	846	25.1436	4.64
chr3R	31715253	31715966	ttk	1708	142.555	11.03
chr3R	31720112	31720441	ttk	4749	20.9051	4.43
chr3R	31721374	31722385	ttk	2943	231.771	18.32
chr3R	31722928	31723222	ttk	1929	12.4007	3.38
chr3R	31723481	31726335	ttk	187	316.742	18.44
chr3R	31738869	31739765	Ef1alpha100E	383	54.65	8.17
chr3R	31748070	31748745	CG1910	118	60.3675	6.49
chr3R	31777552	31778320	faf	556	178.632	12.08
chr3R	31805056	31805473	CG42233	68	8.10853	2.39
chr3R	31805056	31805473	CG1971	121	8.10853	2.39
chr3R	31809474	31810630	CG11576	38	243.84	17.25
chr3R	31812453	31813738	RhoGAP100F	54	38.4703	4.84
chr3R	31822526	31823032	RhoGAP100F	3055	79.9318	10.49
chr3R	31825452	31825994	RhoGAP100F	91	30.0976	5.60
chr3R	31937136	31938663	heph	1032	22.5954	5.01
chr3R	31945702	31946055	heph	8473	17.2351	4.22
chr3R	31985505	31985949	heph	50	53.391	6.93
chr3R	31986743	31987151	CG2003	72	61.7771	8.52
chr3R	32015165	32016140	heph	60	104.394	11.24
chr3R	32015165	32016140	CR45557	360	104.394	11.24
chr3R	32067984	32068482	Map205	245	63.3416	6.02
chr3R	32071953	32072304	Map205	3631	22.9723	4.66
chr4	56190	56626	ci	32	59.1772	7.18
chr4	56190	56626	CR43957	133	59.1772	7.18
chr4	69291	69740	pan	1	12.6962	3.00
chr4	276333	276654	Nfl	179	32.3271	5.23
chr4	276333	276654	CR44023	26	32.3271	5.23
chr4	385215	385503	dati	2984	23.4619	5.05
chr4	388500	389347	dati	733	102.719	12.44
chr4	404334	404647	dati	8840	29.5424	5.33
chr4	419659	419946	CR43437	6292	16.1249	4.24
chr4	426938	427467	CR44024	79	24.9044	5.56
chr4	501329	502092	zfh2	48	316.742	36.56
chr4	502590	503214	zfh2	1117	58.8379	7.27
chr4	504841	505906	zfh2	3495	284.705	17.20
chr4	506955	508907	zfh2	5958	316.742	29.25
chr4	554873	555277	Thd1	2760	28.165	4.65
chr4	554873	555277	CR43958	2760	28.165	4.65
chr4	554873	555277	Thd1	2760	28.165	4.65
chr4	557086	558287	Thd1	526	243.085	15.98
chr4	557086	558287	CR43958	526	243.085	15.98
chr4	557086	558287	Thd1	526	243.085	15.98
chr4	557086	558287	Pur-alpha	687	243.085	15.98
chr4	660221	660628	gw	204	21.8111	4.06
chr4	703825	704809	ey	136	90.7138	8.67
chr4	715628	716175	ey	8619	11.9158	3.32
chr4	807658	808696	Sox102F	636	187.965	15.09
chr4	833899	834767	Sox102F	120	125.803	10.19
chr4	844499	844806	fd102C	118	11.9203	3.42
chr4	904850	905586	unc-13	554	120.923	10.80
chr4	989328	989929	toy	126	268.962	19.01

chr4	1031721	1032031	CG11076	131	18.9364	3.99
chr4	1031721	1032031	ATPsynbeta	25	18.9364	3.99
chr4	1088458	1088792	sv	146	14.1991	3.61
chr4	1293056	1293476	Cadps	61308	110.467	9.78
chr4	1306022	1306852	Cadps	74651	156.224	17.58
chr4	1318384	1319384	Cadps	87123	316.742	13.55
chrX	58441	59519	CR40469	63605	316.742	36.04
chrX	245888	246423	G9a	286	30.8834	5.68
chrX	275990	276673	ewg	194	57.3916	8.86
chrX	279277	279558	ewg	226	10.8049	3.26
chrX	334678	335077	CG32816	70	84.8484	11.67
chrX	492914	495130	CG17896	0	112.296	11.04
chrX	492914	495130	CG17778	1118	112.296	11.04
chrX	497445	497795	CR44891	254	11.6375	3.30
chrX	498382	500389	svr	221	316.742	28.77
chrX	498382	500389	CR44892	45	316.742	28.77
chrX	498382	500389	svr	456	316.742	28.77
chrX	519988	520976	elav	432	53.8103	8.10
chrX	521278	523972	elav	105	316.742	37.97
chrX	530225	531336	CG4293	112	25.3684	4.87
chrX	530225	531336	Appl	97	25.3684	4.87
chrX	541718	542401	Appl	10731	49.6123	8.34
chrX	590510	590826	vnd	418	12.1935	3.38
chrX	616880	617540	CG13366	516	27.5528	5.09
chrX	621005	621315	CG13365	3	12.4611	3.34
chrX	628935	629524	RpL36	141	49.4311	7.33
chrX	635889	637323	su(sable)	214	98.9188	10.32
chrX	654181	656733	Hmt4-20	100	286.896	20.09
chrX	654181	656733	SkpA	1075	286.896	20.09
chrX	686304	687930	sdk	584	316.742	25.62
chrX	755695	756557	CG5254	240	180.801	15.93
chrX	758853	759778	CG5273	572	13.1368	3.11
chrX	761590	762204	CR44965	23	13.1443	3.71
chrX	764822	765370	RpL22	16	17.9731	4.13
chrX	764822	765370	CR44966	141	17.9731	4.13
chrX	768311	769186	fz3	1110	19.5637	4.28
chrX	769775	770228	fz3	32	31.5617	5.29
chrX	796399	796872	CG16989	3417	15.2882	3.79
chrX	840455	841401	CG43867	196	44.7887	7.18
chrX	884459	885063	CG43867	507	10.9134	3.58
chrX	900733	901486	CG43867	1756	316.742	26.15
chrX	908698	909383	CG43867	14	34.7685	5.89
chrX	934990	936115	TRAM	5	85.9	9.88
chrX	940596	940935	CG3704	18	18.3298	4.63
chrX	945540	945880	CG3703	62	24.263	5.41
chrX	1119203	1119945	CG3655	15337	30.5821	5.54
chrX	1134046	1135349	CG3655	49	44.7051	7.16
chrX	1232525	1233063	CG3638	2268	76.7338	9.41
chrX	1233912	1236050	CG3638	55	316.742	30.50
chrX	1236414	1236795	CG3638	61	10.7	2.98
chrX	1241878	1242454	CG11403	2263	216.17	16.57
chrX	1251849	1252693	CG11403	12237	204.397	20.46
chrX	1261250	1261589	Atf3	13851	18.8588	4.77
chrX	1274735	1277882	Atf3	1222	316.742	30.93
chrX	1308347	1308841	DAAM	153	55.0107	8.03
chrX	1310542	1310909	DAAM	2239	9.74428	3.38
chrX	1321714	1322476	DAAM	343	11.6561	3.65
chrX	1342030	1342413	CG11412	14	17.4341	4.57
chrX	1370330	1370865	ssx	41	19.9394	4.30
chrX	1375236	1375893	AMPKalpha	44	121.065	11.00
chrX	1380015	1380326	CG3719	450	7.09214	2.79
chrX	1380594	1381107	CG32813	21	59.47	7.89
chrX	1387153	1389249	CG32813	350	316.742	31.27
chrX	1392947	1393336	CG11448	181	8.47249	2.89
chrX	1397838	1398266	CG11448	4786	5.58338	2.45
chrX	1398705	1400120	CG11448	6094	316.742	24.46
chrX	1408963	1409876	futsch	639	38.2918	7.12
chrX	1410241	1410544	futsch	1340	12.8627	3.70
chrX	1411244	1411926	futsch	198	24.9044	5.56
chrX	1466558	1467426	CG14777	221	104.434	10.51
chrX	1478495	1479098	rush	171	28.0948	5.42
chrX	1483086	1483406	sta	29	12.891	3.43
chrX	1507595	1508727	Nmdar2	198	68.8071	10.23
chrX	1512778	1513122	CG14795	119	21.066	3.89
chrX	1512778	1513122	inc	147	21.066	3.89

chrX	1517587	1517983	inc	4733	16.7357	4.47
chrX	1535303	1536108	inc	22427	14.0411	4.07
chrX	1578472	1579217	br	3920	19.5844	4.87
chrX	1614350	1614800	br	937	10.2503	3.23
chrX	1616862	1621015	Mur2B	1048	316.742	38.95
chrX	1623148	1623557	br	610	73.7649	10.37
chrX	1656418	1656689	dor	8493	8.95959	3.16
chrX	1773794	1774761	Adar	68	21.5498	4.44
chrX	1793858	1794502	CG32806	4517	39.3302	7.25
chrX	1809712	1810066	CG14810	10513	12.1308	3.77
chrX	1811235	1811625	CG14810	8943	17.4341	4.57
chrX	1824554	1825084	CG42666	106	22.8586	4.91
chrX	1828212	1828523	CG42666	1040	16.6813	4.30
chrX	1830578	1831604	CG42666	78	316.742	26.82
chrX	1842899	1843554	CG42666	182	85.9092	9.99
chrX	1861690	1862014	deltaCOP	32	13.449	3.78
chrX	1893586	1893985	mRpL16	3509	28.2992	5.46
chrX	1899362	1900228	arm	2	96.1881	9.53
chrX	1907726	1908147	Edem1	0	13.1054	3.64
chrX	1908734	1909211	Edem1	66	18.8732	3.93
chrX	1908734	1909211	Rbcn-3B	26	18.8732	3.93
chrX	1922847	1923404	eIF2B-epsilon	320	9.08777	3.04
chrX	1922847	1923404	Ocrl	214	9.08777	3.04
chrX	1941667	1942081	Hr4	79	93.9409	12.33
chrX	1943159	1943643	Hr4	1453	70.9218	10.43
chrX	1946958	1948611	Hr4	6308	316.742	32.06
chrX	1951685	1952079	Hr4	9847	15.3684	4.27
chrX	1953014	1953276	Hr4	11237	11.239	3.01
chrX	1963566	1964484	Hr4	21938	30.5433	6.14
chrX	1964795	1965389	Hr4	23096	85.9738	9.40
chrX	1967072	1967655	Hr4	24899	47.6938	8.14
chrX	1975656	1976719	Hr4	15962	316.742	50.80
chrX	1981748	1982316	Hr4	10117	12.7565	3.87
chrX	1988451	1989403	Hr4	3634	66.7095	10.03
chrX	1991080	1992378	Hr4	169	64.6289	9.83
chrX	2009841	2010933	CR45478	354	261.564	16.38
chrX	2009841	2010933	mir-2496	11	261.564	16.38
chrX	2009841	2010933	east	549	261.564	16.38
chrX	2011457	2013767	east	15	173.001	13.00
chrX	2031798	2032250	mir-4954	2899	34.8887	6.75
chrX	2036344	2036633	Actn	12	17.958	4.62
chrX	2040913	2042463	usp	479	36.371	5.54
chrX	2040913	2042463	CG4313	76	36.371	5.54
chrX	2040913	2042463	Actn	520	36.371	5.54
chrX	2040913	2042463	usp	520	36.371	5.54
chrX	2040913	2042463	Actn	520	36.371	5.54
chrX	2043640	2044015	CG4325	14	20.6918	4.45
chrX	2048931	2050780	moody	239	316.742	36.27
chrX	2060586	2062128	Sik2	781	47.0061	6.56
chrX	2070008	2071099	CG4199	40	50.3436	6.38
chrX	2071689	2072283	CR44382	306	100.023	11.16
chrX	2071689	2072283	CG4199	50	100.023	11.16
chrX	2080292	2080893	CG16903	259	45.9589	6.53
chrX	2080292	2080893	Unc-76	55	45.9589	6.53
chrX	2083459	2084311	Unc-76	3241	316.742	40.06
chrX	2094089	2094892	csw	297	149.519	15.11
chrX	2098473	2098800	snoRNA:Me18S-C102	3345	16.7966	4.33
chrX	2102059	2103182	csw	84	306.722	26.31
chrX	2106511	2106927	csw	1526	58.4408	8.73
chrX	2107488	2109272	csw	40	26.9399	4.46
chrX	2112729	2113375	csw	4979	9.74428	3.38
chrX	2121724	2124818	ph-d	127	316.742	21.60
chrX	2125462	2125968	ph-d	2428	108.411	9.83
chrX	2134464	2138847	ph-p	138	316.742	23.21
chrX	2140459	2142134	CG3835	115	283.023	22.34
chrX	2145214	2146508	Pgd	11	27.0191	5.27
chrX	2155826	2156408	wapl	175	218.524	18.67
chrX	2157896	2159069	wapl	888	46.3942	6.24
chrX	2175788	2176291	Cyp4d2	25	74.6483	9.68
chrX	2176566	2177098	Cyp4d2	95	69.5886	9.27
chrX	2194292	2194662	Mct1	97	45.7966	7.95
chrX	2221362	2222790	Vinc	25	68.5012	7.33
chrX	2253808	2255773	CG3091	1063	316.742	34.40
chrX	2259721	2260102	CG3078	63	11.9218	3.27
chrX	2269520	2270914	CG2924	554	59.9876	8.08

chrX	2269520	2270914	CR45480	276	59.9876	8.08
chrX	2269520	2270914	CG2924	490	59.9876	8.08
chrX	2274733	2275202	CG2918	57	25.0781	5.02
chrX	2286244	2286723	CG2865	6980	34.0192	6.65
chrX	2292614	2295017	CG2865	97	316.742	35.01
chrX	2295684	2296767	Raf	640	316.742	26.64
chrX	2297175	2297677	Raf	2039	50.8336	7.51
chrX	2300379	2302532	Raf	5675	316.742	35.85
chrX	2303272	2304531	Raf	8465	316.742	33.56
chrX	2305106	2307173	Raf	10563	316.742	23.78
chrX	2317344	2318020	CR34052	4591	316.742	31.00
chrX	2323868	2324240	CG14050	1711	21.8144	5.16
chrX	2325554	2326245	CG14050	65	160.1	17.88
chrX	2334091	2334940	Ilp6	413	21.8144	5.16
chrX	2362412	2362702	CG2841	3799	24.7558	5.34
chrX	2366091	2366825	CG2841	52	95.7768	11.34
chrX	2383274	2383950	PsGEF	3936	40.2365	7.35
chrX	2447908	2448370	z	273	14.8847	3.94
chrX	2469630	2470317	boi	13	110.455	9.86
chrX	2545406	2545775	trol	195	28.9409	6.06
chrX	2588502	2589634	egh	480	278.312	17.23
chrX	2592638	2593630	egh	683	22.8458	4.99
chrX	2633910	2635003	sgg	484	47.7951	7.30
chrX	2635935	2636375	sgg	1233	16.7357	4.47
chrX	2636948	2637309	sgg	276	29.2914	5.36
chrX	2642296	2643921	sgg	76	316.742	40.24
chrX	2645673	2646869	sgg	497	273.117	17.61
chrX	2647196	2647558	sgg	734	36.7831	6.67
chrX	2652512	2652818	sgg	6028	24.8157	5.54
chrX	2658275	2659008	sgg	114	316.742	26.74
chrX	2684630	2686204	per	67	316.742	29.70
chrX	2687277	2687621	per	1879	24.2657	5.41
chrX	2694109	2694557	CG2650	413	29.7702	6.16
chrX	2703704	2704005	Csat	14	15.1847	4.15
chrX	2741943	2742942	Syx4	707	145.827	14.04
chrX	2743391	2744899	Syx4	268	288.637	25.48
chrX	2786522	2787664	CG32795	2040	30.703	4.64
chrX	2789588	2793248	CG32795	190	316.742	10.55
chrX	2795944	2796788	w	24	136.694	7.18
chrX	3099006	3099706	kirre	256	189.62	17.60
chrX	3134660	3135933	N	248	81.8273	10.02
chrX	3136336	3136813	N	1736	90.6608	12.22
chrX	3161866	3163305	CG18508	10201	273.984	19.88
chrX	3175956	3176621	dnc	154	316.742	25.31
chrX	3189747	3190022	dnc	13412	17.4341	4.57
chrX	3190750	3191397	dnc	14505	83.9855	10.43
chrX	3206031	3206434	dnc	29829	22.5747	5.26
chrX	3288317	3289638	dnc	53	196.021	17.85
chrX	3294573	3295807	dnc	329	109.096	11.37
chrX	3297964	3298320	dnc	2886	27.6526	5.75
chrX	3332519	3332964	dnc	1107	65.6671	9.93
chrX	3369534	3369855	CR44999	3469	16.68	4.23
chrX	3369534	3369855	Myc	3469	16.68	4.23
chrX	3372706	3373436	CR44999	64	316.742	22.51
chrX	3372706	3373436	Myc	64	316.742	22.51
chrX	3376554	3376994	CR44999	3595	17.8021	4.58
chrX	3376554	3376994	Myc	3595	17.8021	4.58
chrX	3377336	3378124	CR44999	4708	312.351	20.05
chrX	3377336	3378124	Myc	4708	312.351	20.05
chrX	3379304	3379813	CR44999	6446	40.4411	7.28
chrX	3379304	3379813	Myc	6446	40.4411	7.28
chrX	3381397	3382685	CR44999	9129	241.091	19.26
chrX	3381397	3382685	Myc	9129	241.091	19.26
chrX	3427536	3427968	CG14269	8150	14.9061	4.15
chrX	3433732	3434116	CG14269	1902	15.3684	4.27
chrX	3440730	3441389	CG12535	1076	17.8883	4.06
chrX	3446146	3446602	CG12535	1190	13.3263	3.95
chrX	3451172	3451667	CG12535	2526	191.432	14.81
chrX	3452152	3452605	CG12535	3434	34.8887	6.75
chrX	3482277	3482567	CG10804	127	27.8127	5.07
chrX	3545941	3546715	MRE16	360	164.093	13.78
chrX	3570355	3570676	ppk8	1000	13.5986	3.49
chrX	3672016	3673059	Mnt	486	316.742	29.77
chrX	3673604	3674271	Mnt	2185	220.417	15.81
chrX	3675578	3675999	Mnt	3990	95.1045	9.91

chrX	3676573	3678815	Mnt	5903	316.742	42.65
chrX	3679470	3680988	Mnt	8049	92.6302	11.23
chrX	3681594	3683002	Mnt	5553	275.136	18.55
chrX	3684828	3685939	Mnt	3142	40.0501	5.89
chrX	3686705	3689566	Mnt	89	316.742	34.29
chrX	3691287	3692758	Mnt	3129	204.391	18.29
chrX	3694067	3696084	Mnt	6304	316.742	32.03
chrX	3696781	3697302	CR45102	4802	104.777	12.16
chrX	3718486	3720912	Rala	849	316.742	38.41
chrX	3718486	3720912	Tlk	1220	316.742	38.41
chrX	3722942	3723464	Tlk	1418	291.842	19.91
chrX	3740582	3741378	Tlk	986	316.742	44.89
chrX	3741732	3745156	Tlk	4750	316.742	26.11
chrX	3747134	3747483	Tlk	208	10.2392	3.18
chrX	3747954	3748265	Tlk	925	10.5226	3.35
chrX	3748530	3749303	Tlk	67	316.742	39.03
chrX	3750104	3750411	Tlk	1157	10.9134	3.58
chrX	3761207	3761646	Tlk	11162	20.5555	4.78
chrX	3772765	3773887	Tlk	669	127.747	12.45
chrX	3775729	3781351	Tlk	4280	316.742	34.57
chrX	3775729	3781351	mir-4962	2957	316.742	34.57
chrX	3775729	3781351	Tlk	196	316.742	34.57
chrX	3811243	3812244	ec	59	291.888	21.24
chrX	3835170	3836434	ec	475	316.742	30.41
chrX	3841075	3841524	ec	4634	36.7705	6.47
chrX	3845352	3846160	ec	100	316.742	29.99
chrX	3861135	3861505	roX1	744	32.1681	6.13
chrX	3866152	3866992	yin	246	264.203	22.14
chrX	3868154	3869066	yin	240	219.964	20.02
chrX	3868154	3869066	CG2930	401	219.964	20.02
chrX	3872270	3872598	CG2930	23	18.3275	4.63
chrX	3880676	3881006	VhaAC39-1	37	12.493	3.50
chrX	3930764	3931164	CG42541	444	16.9306	4.44
chrX	3949025	3950336	Vap-33A	593	180.962	16.22
chrX	4105833	4106703	cib	22	58.8156	7.68
chrX	4107201	4107668	cib	637	15.6041	3.65
chrX	4116949	4117491	Tip60	166	18.1042	3.66
chrX	4116949	4117491	tyf	96	18.1042	3.66
chrX	4116949	4117491	Tip60	327	18.1042	3.66
chrX	4169906	4170232	Fas2	35996	11.4567	3.42
chrX	4203773	4204331	Fas2	1966	103.027	13.25
chrX	4205416	4206272	Fas2	88	235.503	17.73
chrX	4322520	4323190	norpA	25	11.4471	2.88
chrX	4323802	4324182	norpA	447	23.2357	5.16
chrX	4324472	4325635	norpA	1266	316.742	30.75
chrX	4411199	4411964	bi	1275	272.363	21.48
chrX	4412601	4413306	bi	76	17.4341	4.57
chrX	4423809	4425575	bi	2006	212.096	16.69
chrX	4426526	4427360	bi	153	316.742	37.08
chrX	4438527	4440172	bi	12295	13.319	3.48
chrX	4444748	4445161	bi	17816	13.7183	3.67
chrX	4508116	4508378	CG3556	21963	14.5571	3.93
chrX	4589744	4590545	CG12684	18369	34.0192	6.65
chrX	4596399	4597028	CR32773	14728	129.272	14.74
chrX	4617202	4617774	peb	71	305.101	27.61
chrX	4667005	4667334	CG3009	474	22.8209	4.56
chrX	4678216	4679561	rap	527	203.752	16.78
chrX	4684247	4686964	Pp2C1	2400	298.065	17.32
chrX	4687950	4688491	ctp	59	63.3289	7.60
chrX	4689690	4690069	ctp	1750	12.5816	3.69
chrX	4690833	4691279	ctp	2840	24.1197	5.46
chrX	4694053	4694394	ctp	6026	15.8482	4.24
chrX	4702392	4702808	l(1)G0334	484	5.20293	2.20
chrX	4702847	4703235	l(1)G0334	795	7.56884	2.51
chrX	4707645	4707916	Proc-R	140	13.0509	3.25
chrX	4840660	4840914	CG42594	54	13.3929	3.97
chrX	4908856	4909413	CG32772	52	36.3765	6.58
chrX	4909885	4911044	CG32772	1395	93.9849	10.88
chrX	4918207	4920042	CG4041	1401	39.4599	6.00
chrX	4918207	4920042	CG6903	977	39.4599	6.00
chrX	4930007	4930830	CG44774	527	25.4777	5.34
chrX	4936149	4936533	CG44774	198	30.8834	5.68
chrX	4937183	4938131	CG44774	493	74.2808	8.51
chrX	4939106	4940625	Ptp4E	815	101.245	10.01
chrX	4956021	4956463	CG15468	4957	11.9329	3.23

chrX	5044835	5045175	ovo	3254	12.1308	3.77
chrX	5045619	5045944	ovo	2581	8.62644	3.18
chrX	5046270	5048458	ovo	258	316.742	39.45
chrX	5052789	5053236	ovo	4753	65.4457	9.49
chrX	5055129	5055447	ovo	7031	22.5747	5.26
chrX	5056190	5056885	ovo	7568	13.9887	3.73
chrX	5063314	5063906	ovo	332	15.163	3.82
chrX	5063314	5063906	CR44833	357	15.163	3.82
chrX	5081891	5083158	CG32767	251	46.0209	6.11
chrX	5083424	5084817	CG32767	301	85.0153	9.24
chrX	5085155	5087670	rg	828	316.742	31.23
chrX	5085155	5087670	CG15465	828	316.742	31.23
chrX	5085155	5087670	rg	828	316.742	31.23
chrX	5164255	5164586	CG15465	937	21.0625	5.07
chrX	5164255	5164586	rg	937	21.0625	5.07
chrX	5164850	5165362	CG15465	172	12.7942	3.57
chrX	5164850	5165362	rg	172	12.7942	3.57
chrX	5279257	5279520	CG42749	16704	14.0411	4.07
chrX	5294215	5294636	CG42749	1714	18.1418	4.67
chrX	5311931	5312344	dhd	58	11.6566	3.22
chrX	5322499	5322801	Rnp4F	92	9.7279	2.89
chrX	5332477	5332979	CanB	28	70.7112	10.38
chrX	5339994	5340443	SK	67	73.0528	10.63
chrX	5363142	5363519	SK	1878	24.9044	5.56
chrX	5363971	5364444	SK	959	66.4907	9.34
chrX	5410624	5411381	NAAT1	25	35.3264	6.54
chrX	5417831	5419288	spoon	451	225.448	16.86
chrX	5419601	5420031	spoon	538	23.4903	5.14
chrX	5420577	5420967	spoon	30	12.2653	3.13
chrX	5428221	5428706	CR45517	43	129.272	14.74
chrX	5429881	5430780	CG15784	93	215.362	18.72
chrX	5432822	5433499	CR44471	196	52.0185	7.36
chrX	5515287	5516142	CG12730	931	39.4994	6.16
chrX	5516450	5518645	CG12730	60	316.742	46.44
chrX	5516450	5518645	CR45518	302	316.742	46.44
chrX	5524454	5524705	snoRNA:Psi28S-3342	148	9.54155	3.16
chrX	5560373	5561127	Vsx2	535	212.561	17.67
chrX	5593784	5594417	Vsx1	211	131.69	14.32
chrX	5621949	5622692	mir-3645	293	15.3558	3.70
chrX	5621949	5622692	Mlc-c	416	15.3558	3.70
chrX	5621949	5622692	l(1)G0045	524	15.3558	3.70
chrX	5636518	5637160	CG42492	851	88.9279	11.69
chrX	5637446	5638063	CG42492	185	132.91	15.02
chrX	5648336	5648710	CG42492	6	14.4387	3.51
chrX	5649339	5649658	CG42492	981	21.0625	5.07
chrX	5649959	5650482	CG42492	1734	42.3588	7.28
chrX	5661997	5663172	CG42265	491	70.9218	10.43
chrX	5661997	5663172	CR45520	743	70.9218	10.43
chrX	5664126	5664629	CR45520	1278	114.368	13.22
chrX	5666048	5666314	CG4119	54	5.71079	2.35
chrX	5671570	5671890	Rab18	51	13.3929	3.97
chrX	5672470	5672793	RpL35	30	13.0535	3.52
chrX	5679766	5680121	Tre1	8	30.3398	5.21
chrX	5689912	5691282	cv	58	78.7418	9.67
chrX	5689912	5691282	CR45525	795	78.7418	9.67
chrX	5696447	5696844	CR42794	429	24.1197	5.46
chrX	5698390	5698778	CG4096	20	23.2633	5.00
chrX	5705061	5706491	CG4096	6960	69.606	10.13
chrX	5715399	5716256	CG33080	145	16.0203	4.14
chrX	5717663	5718007	CG33080	57	31.0588	6.16
chrX	5724245	5724792	CG32758	953	23.4066	4.74
chrX	5734907	5735861	CG15773	2987	22.2306	5.09
chrX	5737147	5738181	CG32758	650	68.1853	9.33
chrX	5744269	5744698	IntS6	1862	58.0488	7.55
chrX	5745120	5747456	IntS6	287	316.742	34.44
chrX	5754906	5756478	lin-52	320	316.742	34.64
chrX	5754906	5756478	CG15771	295	316.742	34.64
chrX	5763303	5764146	MAPk-Ak2	281	14.1426	3.41
chrX	5770708	5771131	CG3097	131	58.958	7.62
chrX	5818425	5818833	CG15765	42	24.1197	5.46
chrX	5879812	5880959	CG16721	273	23.8345	4.30
chrX	5885432	5887357	CG16721	6195	316.742	29.84
chrX	5895874	5896497	CR45668	3873	29.0751	5.54
chrX	5899164	5901179	CR45668	20	316.742	27.82
chrX	5899164	5901179	Act5C	851	316.742	27.82

chrX	5901463	5902439	Act5C	173	316.742	31.62
chrX	5904186	5904988	CG4020	1218	279.742	20.62
chrX	5908032	5908474	CG12236	45	8.97238	2.96
chrX	5912181	5912532	CR44108	2057	19.2591	4.48
chrX	5916070	5916508	CG3011	23	14.0872	3.54
chrX	5916852	5917396	CG3726	24	87.4049	8.83
chrX	5918474	5919312	CG3726	1649	316.742	26.03
chrX	5935088	5935465	CG12728	7250	18.1418	4.67
chrX	5987720	5988995	CG5966	434	158.042	16.28
chrX	5992237	5992491	CG5966	3578	25.7773	5.09
chrX	6000539	6001920	CG4766	501	266.366	24.55
chrX	6000539	6001920	CR44498	212	266.366	24.55
chrX	6012990	6013800	mab-21	181	316.742	23.30
chrX	6012990	6013800	CR44499	345	316.742	23.30
chrX	6037194	6037467	rux	56	8.56807	2.69
chrX	6050762	6051342	CG5921	227	17.019	4.24
chrX	6068850	6069210	CG5928	1093	34.0192	6.65
chrX	6075020	6075339	Tsp5D	307	21.8144	5.16
chrX	6077154	6077645	CG4666	71	53.476	8.49
chrX	6081264	6081681	CG4660	105	21.8144	5.16
chrX	6089602	6090138	CR44959	29	46.579	6.41
chrX	6093740	6094076	Nep1	63	28.0546	5.49
chrX	6193229	6193914	Ca-alpha1T	1074	28.5455	5.43
chrX	6219901	6220467	Efr	122	66.905	7.18
chrX	6224189	6225065	sqh	60	139.122	11.70
chrX	6225505	6226053	CG14446	49	23.7856	4.62
chrX	6254433	6254824	Rbcn-3A	945	14.0411	4.07
chrX	6262986	6263240	CG3781	1933	23.3834	5.11
chrX	6266116	6266446	schlank	969	16.9573	4.23
chrX	6267714	6268566	Spt6	112	15.3558	3.70
chrX	6278899	6279873	Ubi-p5E	144	316.742	26.66
chrX	6278899	6279873	CR45530	261	316.742	26.66
chrX	6281971	6282433	CG11700	1320	74.1242	10.73
chrX	6283381	6283880	CG11700	69	119.156	12.17
chrX	6287764	6288237	Top3beta	18	11.2818	3.18
chrX	6287764	6288237	wuho	91	11.2818	3.18
chrX	6317091	6318038	CG15894	106	46.695	7.91
chrX	6330124	6331167	CG42240	25	316.742	31.30
chrX	6331554	6332303	CG42240	1299	17.9731	4.13
chrX	6332974	6333413	CG42240	2314	86.7176	11.50
chrX	6338737	6341365	CG3842	445	172.404	17.17
chrX	6341710	6342289	CG3842	855	108.127	13.27
chrX	6343638	6345038	CG3847	3092	316.742	24.42
chrX	6346879	6347505	CG3847	107	39.6833	6.35
chrX	6351949	6353571	kdn	863	103.483	11.92
chrX	6354160	6354481	kdn	2044	31.3542	6.13
chrX	6357519	6357789	kdn	5393	12.7565	3.87
chrX	6365918	6366462	Marf	264	25.9686	4.56
chrX	6524229	6524730	CG42340	60	12.1138	3.32
chrX	6524229	6524730	CG3918	80	12.1138	3.32
chrX	6533964	6534625	dx	279	293.118	20.61
chrX	6543907	6544232	CG34417	2069	14.0411	4.07
chrX	6545999	6546313	CG34417	51	14.2507	4.05
chrX	6547263	6547545	CG34417	1224	11.9203	3.42
chrX	6549415	6550095	CG34417	3501	135.281	14.58
chrX	6550757	6551241	CG34417	4891	139.687	16.29
chrX	6590788	6591221	Pat1	285	11.9854	3.33
chrX	6599158	6599513	pigs	733	26.5212	5.17
chrX	6599794	6600280	pigs	58	148.392	13.73
chrX	6601383	6602518	pigs	35	22.2685	4.94
chrX	6618847	6619265	CG14443	14983	14.9468	4.09
chrX	6624052	6624525	CG14443	9715	53.14	7.86
chrX	6643156	6643414	CR44817	6145	16.6919	3.98
chrX	6654583	6654893	Cdc7	52	12.1882	3.33
chrX	6656022	6657539	Cdc7	2646	58.7989	8.57
chrX	6661627	6662815	Ctr1A	947	36.4947	5.83
chrX	6668999	6669377	CG3198	4775	54.498	8.84
chrX	6678135	6678926	ND-ASHI	550	10.8116	3.42
chrX	6678135	6678926	l(1)G0255	387	10.8116	3.42
chrX	6679415	6679717	l(1)G0255	85	18.1418	4.67
chrX	6683845	6684466	Pink1	57	23.8527	4.83
chrX	6694041	6694721	CG14442	787	72.9111	8.83
chrX	6698093	6698607	CG14440	219	44.1161	6.68
chrX	6699150	6700894	CG14441	1078	316.742	24.71
chrX	6702582	6703067	CG14441	3300	33.0785	5.43

chrX	6703528	6703998	CG14441	4208	35.6367	5.61
chrX	6706795	6707270	CG14441	2029	37.5924	6.55
chrX	6707916	6709672	CG14441	946	140.27	13.85
chrX	6711323	6711727	CG14441	2313	13.3929	3.97
chrX	6726991	6727481	CG3168	35	20.9195	4.63
chrX	6743595	6744298	snoRNA:Psi28S-1232	375	11.9203	3.42
chrX	6743595	6744298	RpL17	19	11.9203	3.42
chrX	6747054	6747861	CG14439	217	89.7324	11.44
chrX	6749651	6750254	CR44964	1791	14.3186	3.81
chrX	6751623	6752019	CR44964	3795	14.9277	4.16
chrX	6756365	6756807	CG14438	228	26.9291	4.61
chrX	6757288	6757553	CG14438	1047	16.6813	4.30
chrX	6779556	6780296	shf	428	143.965	15.85
chrX	6791103	6792167	C3G	548	316.742	24.64
chrX	6793501	6793848	C3G	1689	22.4872	5.07
chrX	6800305	6801243	C3G	231	180.708	17.03
chrX	6802790	6805168	C3G	1142	316.742	25.28
chrX	6802790	6805168	pod1	249	316.742	25.28
chrX	6805571	6805847	pod1	313	15.9289	4.05
chrX	6853413	6853834	CG14434	746	23.7415	5.28
chrX	6855641	6856107	CG14434	2913	35.2967	6.25
chrX	6860135	6860955	CR45531	34	121.927	10.76
chrX	6861324	6862025	CR45531	1302	296.002	25.77
chrX	6862405	6863324	CG43736	985	68.7154	8.05
chrX	6865132	6865481	CG43736	1178	18.2469	4.46
chrX	6865959	6866489	CG14434	957	58.2813	8.81
chrX	6867083	6867641	CG14434	208	29.5442	4.47
chrX	6867927	6868922	CG43736	24	316.742	28.52
chrX	6871771	6872070	CG43736	3407	18.5016	4.60
chrX	6874992	6875411	CG43736	736	19.929	4.12
chrX	6875782	6876210	CG43736	13	85.0153	9.24
chrX	6969395	6969907	CG4586	319	30.6062	6.26
chrX	6980847	6982188	ogre	110	316.742	45.45
chrX	6985518	6987882	ogre	1640	197.308	17.93
chrX	6985518	6987882	bou	823	197.308	17.93
chrX	6997265	6997867	CR43837	390	10.9134	3.58
chrX	6998247	6999121	Inx2	54	316.742	46.09
chrX	7000432	7000803	Inx2	1948	44.3195	7.39
chrX	7008065	7009086	cm	2531	24.1043	5.28
chrX	7068232	7068496	CG4607	1073	8.98509	2.97
chrX	7068894	7069612	CG4607	255	122.809	14.19
chrX	7095221	7095535	Sxl	1730	10.029	3.08
chrX	7095819	7096393	Sxl	970	46.5283	6.96
chrX	7096745	7098258	Sxl	236	295.804	20.74
chrX	7101339	7102395	CG4615	225	29.7942	5.31
chrX	7109667	7110432	fz4	362	68.3494	10.13
chrX	7110863	7111219	fz4	661	16.2826	4.21
chrX	7193787	7194332	CG9650	1908	113.936	14.20
chrX	7194598	7195051	CG9650	1339	11.516	3.68
chrX	7195868	7196514	CG9650	154	116.334	14.40
chrX	7204019	7205336	CG9650	8920	139.687	16.29
chrX	7242060	7242415	CG9650	15849	14.7	4.17
chrX	7285280	7286893	CG1677	642	238.93	23.64
chrX	7285280	7286893	CG2059	804	238.93	23.64
chrX	7293474	7294190	unc-119	2748	11.516	3.68
chrX	7299523	7302270	brk	8035	316.742	33.98
chrX	7305695	7308362	brk	235	316.742	33.47
chrX	7317263	7317638	Atg5	6327	40.027	6.87
chrX	7324196	7325034	Dok	336	23.1117	4.38
chrX	7445022	7445398	CR45533	145	35.7647	6.85
chrX	7604363	7605107	ct	4389	316.742	25.99
chrX	7605653	7606099	ct	3216	26.0188	5.38
chrX	7607855	7608685	ct	841	316.742	27.62
chrX	7626611	7627694	ct	5070	316.742	32.82
chrX	7690677	7693542	CHES-1-like	2241	316.742	32.07
chrX	7694184	7696146	CHES-1-like	196	316.742	28.46
chrX	7696498	7697716	CHES-1-like	118	316.742	38.59
chrX	7698569	7698985	CHES-1-like	2089	68.7592	7.58
chrX	7702434	7703099	CHES-1-like	2201	316.742	49.76
chrX	7703396	7703848	CHES-1-like	1374	90.6624	10.86
chrX	7704853	7707534	CHES-1-like	346	316.742	25.43
chrX	7709172	7710343	CHES-1-like	113	316.742	21.12
chrX	7728712	7729141	CG12155	190	30.5821	5.54
chrX	7901093	7901418	snoRNA:Or-aca2	104	18.0266	4.41
chrX	7901093	7901418	RpS6	24	18.0266	4.41

chrX	7907664	7908173	dpr14	253	18.4273	4.36
chrX	7908627	7909151	CG1444	120	14.1627	3.66
chrX	7912112	7912417	snz	51	7.30252	2.68
chrX	7920388	7920786	Ykt6	54	10.5982	3.21
chrX	7930067	7931487	CG10777	727	316.742	47.86
chrX	7933039	7933922	CG10778	38	10.3591	3.02
chrX	7933039	7933922	RpS14a	447	10.3591	3.02
chrX	7935787	7936306	Ubr3	102	12.3183	3.51
chrX	7937537	7938985	Ubr3	2742	256.108	20.58
chrX	7944405	7945631	l(1)G0193	466	316.742	22.61
chrX	7946735	7947334	l(1)G0193	136	101.273	10.20
chrX	7959081	7959595	CR45620	85	19.5844	4.87
chrX	7959081	7959595	sws	181	19.5844	4.87
chrX	7967111	7968389	sws	366	231.883	18.75
chrX	7969457	7969962	sn	19	95.2615	10.54
chrX	7970978	7971237	sn	1405	8.99161	3.17
chrX	7995384	7995784	Tbh	130	30.5289	5.95
chrX	8049323	8049597	fs(1)h	6665	14.0411	4.07
chrX	8056236	8059297	fs(1)h	394	316.742	30.77
chrX	8059700	8062621	fs(1)h	174	316.742	44.11
chrX	8059700	8062621	mys	496	316.742	44.11
chrX	8063075	8063554	mys	1628	55.9369	8.00
chrX	8081266	8082667	CG2254	26	29.0751	5.54
chrX	8090449	8093967	UbcE2H	130	316.742	31.16
chrX	8099888	8100586	CG2258	304	67.7561	10.13
chrX	8101178	8101953	CG2258	55	74.5806	8.30
chrX	8113817	8114131	Gclc	2158	23.2431	4.65
chrX	8114730	8116255	Gclc	680	89.7291	10.57
chrX	8141496	8142332	Smox	425	76.8462	9.12
chrX	8157346	8157914	Traf6	282	25.2837	5.47
chrX	8157346	8157914	GIIIspla2	41	25.2837	5.47
chrX	8176373	8177001	slpr	600	31.08	5.72
chrX	8177777	8178216	sdt	565	27.304	5.86
chrX	8178517	8178920	sdt	132	21.0625	5.07
chrX	8192889	8193501	sdt	36	204.371	19.57
chrX	8194376	8194970	sdt	1539	110.184	11.44
chrX	8195890	8196219	sdt	2869	30.5378	6.05
chrX	8196635	8197045	sdt	3692	22.5308	4.92
chrX	8202332	8202898	sdt	9531	15.3684	4.27
chrX	8211584	8211846	sdt	4408	10.3009	3.19
chrX	8213909	8214433	sdt	1972	117.325	13.82
chrX	8215862	8216227	sdt	72	27.8114	5.52
chrX	8228816	8229671	sdt	1123	11.3577	3.34
chrX	8230145	8230822	sdt	163	310.502	26.27
chrX	8242363	8243843	Trxr-1	1022	84.3271	10.37
chrX	8242363	8243843	sni	189	84.3271	10.37
chrX	8242363	8243843	Trxr-1	32	84.3271	10.37
chrX	8244175	8244517	Trxr-1	66	7.16552	2.61
chrX	8271281	8271869	Corp	207	95.2095	12.61
chrX	8275996	8276432	CG15343	2380	62.8255	8.51
chrX	8409108	8412044	Trf2	1283	316.742	30.20
chrX	8409108	8412044	lawc	456	316.742	30.20
chrX	8412340	8413153	lawc	1243	156.047	13.45
chrX	8418388	8418799	Trf2	2754	58.9015	8.27
chrX	8419581	8419884	Trf2	1664	25.7575	5.24
chrX	8420837	8421604	Trf2	78	316.742	41.85
chrX	8436523	8436879	CG12125	114	15.0422	3.64
chrX	8437147	8438034	CG12125	85	46.0271	6.87
chrX	8464183	8465155	Sptr	254	316.742	23.44
chrX	8510799	8511108	CG33181	1274	39.3302	7.25
chrX	8517236	8518925	Nrg	922	115.047	12.17
chrX	8533821	8534392	PIP82	4405	171.859	18.77
chrX	8558670	8558999	IntS4	47	20.0809	4.45
chrX	8560519	8562578	Trf4-1	43	207.757	15.93
chrX	8566848	8567146	CR46195	1839	16.271	4.28
chrX	8569002	8569417	CG12111	1726	48.6506	8.24
chrX	8574179	8575108	CG12065	411	30.7794	5.29
chrX	8588745	8589192	CG12081	140	18.1774	4.30
chrX	8624671	8625299	oc	18143	240.669	22.25
chrX	8650448	8651694	oc	270	316.742	24.02
chrX	8656942	8657479	oc	6584	54.498	8.84
chrX	8676618	8677333	CG12772	137	73.996	9.56
chrX	8681439	8682425	CG11284	548	82.6544	10.53
chrX	8709366	8709871	Hexo2	47	66.189	9.65
chrX	8714335	8714735	CG2004	135	21.9632	3.95

chrX	8757689	8758223	Lim1	27376	285.163	26.82
chrX	8785705	8786210	Lim1	659	82.3404	11.13
chrX	8805585	8807168	Lim1	946	287.954	18.77
chrX	8863720	8864694	CG12075	56	110.363	13.91
chrX	8869963	8870428	Moe	5829	129.731	15.49
chrX	8873829	8874097	Moe	2066	9.83025	3.23
chrX	8877365	8877654	Moe	413	8.62644	3.18
chrX	8888204	8888481	Moe	3499	13.0212	3.81
chrX	8891340	8892173	Moe	93	316.742	29.94
chrX	8893731	8895075	Moe	443	131.168	13.94
chrX	8895683	8896528	Moe	1826	21.0402	4.52
chrX	8897272	8898388	Moe	78	59.4645	7.38
chrX	8903331	8904408	e(r)	1785	28.119	5.96
chrX	8905595	8906208	e(r)	239	30.5821	5.54
chrX	8906897	8907355	rdgA	29	36.8999	5.63
chrX	8924588	8925274	rdgA	213	142.202	16.49
chrX	9029747	9030496	Nost	157	316.742	38.84
chrX	9039751	9040593	Nost	309	133.276	14.63
chrX	9041449	9041738	Nost	1074	7.0135	2.73
chrX	9042029	9042968	Nost	110	316.742	70.14
chrX	9047322	9047654	CR43836	1548	28.9409	6.06
chrX	9067707	9068167	CG15365	4	74.895	10.62
chrX	9078278	9078599	CG44325	66	15.4124	3.40
chrX	9089806	9090123	CG7033	370	8.18818	2.78
chrX	9109417	9110924	AP-1gamma	25	218.76	19.16
chrX	9118679	9119295	CR45539	1755	15.3684	4.27
chrX	9134270	9135220	fend	19	43.823	7.50
chrX	9152157	9152542	His3.3B	2666	14.7014	3.35
chrX	9155576	9156801	His3.3B	260	76.9488	8.86
chrX	9163266	9164665	mei-P26	20	316.742	27.66
chrX	9201800	9202058	su(r)	89	12.7565	3.87
chrX	9214763	9215113	Obp8a	11	19.8762	4.62
chrX	9247452	9247873	Dsor1	45	29.4923	5.90
chrX	9250523	9250781	CG17754	268	10.5548	3.15
chrX	9251167	9251448	CG17754	921	16.4111	3.98
chrX	9251965	9252450	CG17754	8	87.2863	11.92
chrX	9282438	9283309	c11.1	101	21.1167	4.91
chrX	9283835	9284737	lz	244	316.742	36.16
chrX	9305221	9306472	CR44534	8678	225.331	17.97
chrX	9310228	9310521	CR44534	4321	12.6307	3.77
chrX	9355519	9356257	CG34449	494	62.669	8.69
chrX	9400208	9400944	BCL7-like	43715	13.3929	3.97
chrX	9466200	9467713	CG44815	20309	291.373	21.44
chrX	9490925	9491288	mgl	4752	18.1418	4.67
chrX	9495518	9496587	mgl	54	316.742	25.98
chrX	9496932	9497651	mgl	1134	230.185	22.59
chrX	9499853	9500674	mgl	28	69.7441	9.08
chrX	9553259	9553993	l(1)G0320	0	42.6889	6.05
chrX	9554522	9554857	RpS28b	25	15.5019	3.90
chrX	9578791	9580112	LPCAT	950	37.4936	5.03
chrX	9583364	9586573	CR45541	1607	316.742	29.42
chrX	9583364	9586573	Hex-A	106	316.742	29.42
chrX	9588555	9588850	CR45542	1922	37.5164	6.54
chrX	9590249	9590754	CR45542	92	22.9723	4.66
chrX	9595734	9596211	CR45543	910	13.0388	3.47
chrX	9599222	9600692	Gga	1034	157.615	12.45
chrX	9601021	9601611	Gga	66	12.386	3.28
chrX	9608689	9609315	Aats-lys	226	17.7294	4.41
chrX	9608689	9609315	CG42797	165	17.7294	4.41
chrX	9638901	9639506	Ptpmeg2	171	316.742	28.07
chrX	9640343	9641422	Ptpmeg2	58	276.64	20.01
chrX	9646075	9646470	Ptpmeg2	304	17.8958	4.53
chrX	9650808	9651307	Ptpmeg2	338	33.0315	6.53
chrX	9657975	9658985	Ptpmeg2	744	86.9274	9.95
chrX	9666452	9666753	CG3106	3716	31.3452	6.04
chrX	9684032	9687228	nej	194	316.742	30.42
chrX	9687812	9688120	CR42657	251	30.5855	5.12
chrX	9692674	9693036	CR44016	445	37.4225	6.08
chrX	9693603	9694351	btd	209	316.742	30.80
chrX	9729011	9729474	Sp1	421	25.6968	5.66
chrX	9778477	9779108	CG1354	26	46.579	6.41
chrX	9864837	9865400	Ser7	111	57.6177	7.31
chrX	9881655	9882001	CG9689	166	25.6968	5.66
chrX	9884137	9884726	CG9691	16	169.366	14.82
chrX	9930747	9931354	Idgf4	96	316.742	28.62

chrX	9931708	9932070	Idgf4	994	19.5844	4.87
chrX	9964783	9965195	CG1791	9	10.0834	3.24
chrX	9989455	9989838	CG32694	57	26.3157	5.45
chrX	9992108	9992377	CG32694	2526	12.7565	3.87
chrX	10071434	10071734	CR45602	189	12.7565	3.87
chrX	10086336	10086797	RhoU	49	14.4387	3.51
chrX	10209591	10210024	CG15309	96	57.513	8.65
chrX	10211423	10212436	CG15309	690	257.191	18.07
chrX	10224670	10225077	CG34408	1857	62.5419	8.89
chrX	10243533	10243878	Hk	809	20.3193	4.97
chrX	10245205	10245650	Hk	838	70.8907	8.77
chrX	10247327	10247761	Hk	2976	76.2796	10.93
chrX	10250505	10250811	CR43959	5999	24.4015	5.44
chrX	10260956	10261688	Hk	159	87.3268	10.96
chrX	10263646	10264015	Hk	344	22.559	4.39
chrX	10265130	10266175	CG12643	512	72.8476	10.05
chrX	10307826	10308215	alpha-Man-Ia	1127	8.63625	3.02
chrX	10308872	10309441	alpha-Man-Ia	49	316.742	30.91
chrX	10319256	10319645	alpha-Man-Ia	6523	38.8796	5.33
chrX	10322247	10322557	alpha-Man-Ia	3444	9.68765	3.24
chrX	10324409	10324836	alpha-Man-Ia	1296	34.4738	6.45
chrX	10325159	10326023	alpha-Man-Ia	395	150.119	14.66
chrX	10329494	10329969	Gip	93	107.38	9.93
chrX	10333333	10333804	CG43740	99	63.5953	9.73
chrX	10344900	10345249	snoRNA:U3:9B	3071	56.4866	9.04
chrX	10365710	10366382	l(1)G0289	57	23.5664	4.11
chrX	10369744	10370194	CG17841	1997	33.6494	5.68
chrX	10370580	10371230	CG17841	2737	15.013	3.79
chrX	10385835	10386944	Psf3	184	62.669	8.69
chrX	10385835	10386944	flw	86	62.669	8.69
chrX	10472556	10473199	nocte	822	79.1851	8.77
chrX	10473524	10474804	nocte	781	233.669	17.10
chrX	10473524	10474804	CG2889	431	233.669	17.10
chrX	10473524	10474804	nocte	307	233.669	17.10
chrX	10473524	10474804	CG2889	347	233.669	17.10
chrX	10478683	10479600	PPP4R2r	115	112.981	12.67
chrX	10486141	10487214	CG32687	41	170.798	13.48
chrX	10544301	10544632	spri	2313	24.4963	5.20
chrX	10546486	10548369	spri	108	316.742	28.64
chrX	10585233	10585600	spri	41	20.8219	4.76
chrX	10595713	10596271	Hmr	255	62.7656	7.84
chrX	10595713	10596271	CG2124	11	62.7656	7.84
chrX	10601492	10601890	CG1628	90	44.856	7.85
chrX	10602258	10602659	CG1628	280	20.3193	4.97
chrX	10613550	10613946	CG43347	6470	13.3929	3.97
chrX	10619637	10620718	CG43347	285	82.8388	11.52
chrX	10620989	10621841	CG43347	1353	139.687	16.29
chrX	10627476	10628807	X11Lbeta	65	51.8632	7.42
chrX	10729957	10730379	ras	14342	108.942	12.71
chrX	10734818	10735224	ras	9510	18.5762	4.39
chrX	10735549	10736316	ras	8641	316.742	31.22
chrX	10737500	10738384	ras	6337	16.862	4.20
chrX	10738704	10740233	ras	5476	316.742	30.54
chrX	10742078	10743306	ras	1393	68.8667	9.35
chrX	10744000	10745703	ras	389	92.2404	9.20
chrX	10744000	10745703	CR44766	383	92.2404	9.20
chrX	10744000	10745703	CG32676	53	92.2404	9.20
chrX	10744000	10745703	ras	543	92.2404	9.20
chrX	10757474	10758190	Rph	14	16.0475	4.37
chrX	10758467	10758836	Rph	867	10.1287	3.31
chrX	10763165	10764485	Tango5	803	75.5278	8.37
chrX	10767193	10768754	Atg8a	238	93.0738	9.68
chrX	10767193	10768754	BTBD9	992	93.0738	9.68
chrX	10771757	10772729	CG15211	83	316.742	30.41
chrX	10783553	10787044	sesB	124	316.742	38.66
chrX	10783553	10787044	Ant2	2325	316.742	38.66
chrX	10783553	10787044	sesB	2325	316.742	38.66
chrX	10801022	10802583	Imp	4566	316.742	23.88
chrX	10805332	10807162	Imp	30	302.017	24.33
chrX	10832423	10832729	CG15210	6665	34.0192	6.65
chrX	10857664	10858281	CG2186	396	15.4323	3.94
chrX	10906380	10906867	CG43901	880	23.3434	5.36
chrX	10923856	10925642	v	32	20.9337	4.71
chrX	10927251	10927667	CG2145	43	22.4872	5.07
chrX	10929766	10930073	Myo10A	927	21.8144	5.16

chrX	10930484	10931661	Myo10A	62	316.742	26.00
chrX	11081825	11082094	CR44358	2682	19.0085	4.29
chrX	11086087	11086533	sev	3	28.5967	5.88
chrX	11108429	11109245	Hsp60	507	24.8304	5.19
chrX	11124191	11124836	CG11122	107	15.2982	3.59
chrX	11136248	11136996	Klp10A	129	41.7978	6.19
chrX	11142230	11142748	CG15202	20	17.0481	4.06
chrX	11143794	11144309	CG15201	138	42.0673	7.55
chrX	11146532	11146884	Ran	136	11.3652	3.63
chrX	11147577	11147971	Ran	849	9.14346	3.15
chrX	11148333	11148643	Ran	44	13.8868	3.96
chrX	11154013	11155361	Dlic	617	316.742	27.51
chrX	11155692	11156213	Dlic	1597	57.7597	7.68
chrX	11293629	11293973	CG44422	65	19.3066	4.71
chrX	11325274	11326894	l(1)10Bb	1369	36.0308	5.90
chrX	11325274	11326894	CG1657	1293	36.0308	5.90
chrX	11338914	11341164	Kap3	1366	90.2114	10.65
chrX	11338914	11341164	CG34348	1082	90.2114	10.65
chrX	11338914	11341164	Kap3	870	90.2114	10.65
chrX	11350451	11351537	CG1737	263	137.082	12.50
chrX	11355875	11356376	dsh	241	33.0513	5.96
chrX	11359222	11359491	Pa1	70	7.61925	2.68
chrX	11370139	11370799	dlg1	695	52.2062	8.44
chrX	11372751	11373152	dlg1	73	70.9218	10.43
chrX	11389639	11390395	dlg1	54	316.742	23.05
chrX	11423198	11424210	Gs2	415	316.742	30.21
chrX	11426137	11426719	Gs2	2423	102.959	11.57
chrX	11427073	11427763	Gs2	3490	95.1742	10.32
chrX	11442160	11442757	Sk1	26	50.9627	7.35
chrX	11454015	11454441	Evi5	148	20.8219	4.76
chrX	11493054	11493486	Drak	69	35.5835	6.22
chrX	11494879	11495476	Drak	1394	92.4249	10.95
chrX	11505512	11505775	Drak	11840	16.3497	3.57
chrX	11518401	11518711	Drak	24761	15.4627	4.15
chrX	11559932	11560881	PGRP-SA	1438	72.6131	9.52
chrX	11570062	11570684	RpII215	57	22.9663	5.10
chrX	11570062	11570684	CG11699	351	22.9663	5.10
chrX	11599717	11600042	rho-4	35	22.5747	5.26
chrX	11600743	11601530	rho-4	1105	52.7684	7.50
chrX	11616182	11616776	Met	419	133.224	11.91
chrX	11621788	11623148	Ptp10D	872	316.742	23.78
chrX	11623544	11623841	Ptp10D	1674	31.1217	5.65
chrX	11678937	11679853	bif	683	24.5777	5.14
chrX	11681646	11682346	bif	3007	316.742	28.63
chrX	11683266	11683883	bif	4583	28.2173	4.83
chrX	11684480	11684909	bif	5706	34.0192	6.65
chrX	11686060	11686808	bif	7390	316.742	42.27
chrX	11694966	11696151	PhKgamma	218	36.1768	5.71
chrX	11710417	11710786	FucT6	659	58.8424	7.96
chrX	11713566	11717211	Amun	40	316.742	29.70
chrX	11713566	11717211	CR45618	2833	316.742	29.70
chrX	11713566	11717211	prtp	2868	316.742	29.70
chrX	11729709	11730220	inaF-D	846	58.4945	9.24
chrX	11764329	11765082	CG9360	60	174.952	17.34
chrX	11791448	11793401	CR44962	30	316.742	22.64
chrX	11791448	11793401	CkIIbeta	785	316.742	22.64
chrX	11805003	11806513	Hsc70-3	111	212.339	16.73
chrX	11806960	11807821	Hsc70-3	398	316.742	33.35
chrX	11815634	11819264	CG1578	1309	316.742	25.60
chrX	11815634	11819264	rudhira	476	316.742	25.60
chrX	11819901	11821134	rudhira	2469	94.3979	10.84
chrX	11821842	11822203	rudhira	3709	40.5664	6.99
chrX	11823031	11823659	rudhira	4982	316.742	23.84
chrX	11828667	11828971	CR43908	8832	34.0192	6.65
chrX	11854929	11855225	CG10347	110	13.3869	3.65
chrX	11854929	11855225	ATP7	20	13.3869	3.65
chrX	11873146	11873522	CG15739	19	21.7466	4.98
chrX	11914722	11915679	pot	2235	139.687	16.29
chrX	11917038	11917661	pot	136	316.742	31.42
chrX	11922703	11923085	Sclp	134	59.4736	8.83
chrX	12003722	12004109	fw	15	14.7	4.17
chrX	12006707	12008053	CG1806	133	316.742	45.23
chrX	12014802	12015524	regucalcin	387	19.9502	4.56
chrX	12018569	12019137	CG15735	277	39.7982	6.83
chrX	12028310	12029066	Usp7	141	13.6388	3.77

chrX	12031256	12031512	Cyp311a1	43	16.0074	4.21
chrX	12085768	12086297	CG15734	1169	16.0475	4.37
chrX	12128189	12128449	Ten-a	62	10.3226	3.48
chrX	12203339	12204011	Ten-a	155	269.478	22.76
chrX	12389145	12389541	Ir11a	11559	26.4968	5.76
chrX	12463496	12464363	CG42258	123	13.0309	3.42
chrX	12470056	12470956	CG42258	1225	316.742	25.52
chrX	12471307	12473018	CG42258	81	286.917	22.85
chrX	12482997	12483555	Cpr11A	3577	24.1197	5.46
chrX	12546095	12548174	CG43921	601	89.4868	11.13
chrX	12567214	12568743	CG43921	450	46.8918	6.86
chrX	12574594	12576059	CG43921	6506	185.81	15.11
chrX	12578891	12579479	CG11138	3896	57.4829	7.54
chrX	12579824	12580944	CG11138	3051	175.18	14.10
chrX	12582771	12583801	CG11138	107	147.059	12.95
chrX	12591128	12592156	LIMK1	391	23.6409	4.54
chrX	12595165	12597029	CG1824	839	270.376	20.14
chrX	12595165	12597029	Sec16	212	270.376	20.14
chrX	12601029	12601444	Sec16	4336	47.5554	8.11
chrX	12612197	12612757	CG2543	36	55.4106	8.67
chrX	12613097	12613506	CG2543	823	76.2796	10.93
chrX	12615765	12616055	CR45621	155	14.0411	4.07
chrX	12631603	12632357	Tomosyn	214	27.6062	4.96
chrX	12633289	12634474	Tomosyn	7	23.4631	5.13
chrX	12653614	12654860	CkIalpha	72	23.7739	4.06
chrX	12658999	12659663	Tis11	69	49.5158	6.32
chrX	12658999	12659663	CR33963	22	49.5158	6.32
chrX	12660065	12660719	Tis11	67	154.619	14.92
chrX	12661447	12661978	Tis11	1127	188.85	19.08
chrX	12664955	12666626	Tis11	4697	316.742	38.45
chrX	12667528	12668163	Tis11	7440	23.2357	5.16
chrX	12669611	12670026	Tis11	9245	35.5741	5.97
chrX	12671686	12671976	Tis11	11306	16.7264	4.17
chrX	12672510	12673133	Tis11	12282	17.814	3.98
chrX	12673627	12674477	Tis11	13378	159.985	15.89
chrX	12699324	12699768	CG15725	6349	54.498	8.84
chrX	12715949	12716323	CR43963	2410	14.9811	3.90
chrX	12716808	12717079	CR43963	1575	11.6708	3.53
chrX	12720013	12720560	CR43963	1830	81.8905	10.89
chrX	12721068	12721775	CR43963	2941	45.9589	6.53
chrX	12724190	12724757	CR43963	6047	61.4917	9.26
chrX	12725017	12725854	CR43963	5699	183.255	17.65
chrX	12727582	12728173	CR43963	3213	16.5248	4.06
chrX	12734394	12734709	CR43963	3605	20.8219	4.76
chrX	12734995	12736549	CR43963	4316	270.661	17.20
chrX	12737038	12737901	Smr	4689	182.472	17.05
chrX	12741835	12742563	Smr	46	39.2811	6.19
chrX	12741835	12742563	CR45623	522	39.2811	6.19
chrX	12750232	12752429	CR44619	160	245.168	18.42
chrX	12753597	12756792	CR44619	2245	316.742	27.62
chrX	12760276	12760808	ade5	81	127.462	13.65
chrX	12765228	12765839	CG3812	409	13.3929	3.97
chrX	12784559	12785161	CG32647	29	109.537	9.50
chrX	12869423	12870036	Pde9	1041	86.7176	11.50
chrX	12890201	12890575	Pde9	6948	65.4488	9.75
chrX	12905248	12906236	CG4404	112	40.3927	6.02
chrX	12917207	12917536	fne	119	10.9134	3.58
chrX	12925426	12926452	fne	8254	153.652	17.38
chrX	12929786	12930286	fne	8466	67.5523	10.09
chrX	12938020	12938579	fne	224	110.363	13.91
chrX	12988162	12988754	rad	366	12.2997	3.69
chrX	13087421	13087823	hep	2741	29.6697	6.13
chrX	13089708	13090504	hep	299	147.312	14.84
chrX	13090984	13091338	lic	32	31.3257	5.19
chrX	13128000	13129174	snmRNA:204	283	43.8783	6.25
chrX	13128000	13129174	CG32638	447	43.8783	6.25
chrX	13128000	13129174	CG15717	604	43.8783	6.25
chrX	13128000	13129174	MFS10	670	43.8783	6.25
chrX	13128000	13129174	CG15717	670	43.8783	6.25
chrX	13130829	13132062	MFS10	216	316.742	35.08
chrX	13138847	13139241	CG32645	157	41.1492	7.45
chrX	13208468	13208974	sno	2942	56.9036	8.76
chrX	13209666	13209948	sno	1938	9.45705	3.08
chrX	13211390	13211728	sno	123	19.1824	4.20
chrX	13237597	13237992	CG32639	1229	79.542	11.22

chrX	13238261	13238575	CG32639	1823	21.8144	5.16
chrX	13256955	13257292	mew	66	18.1596	4.37
chrX	13272647	13273076	HDAC4	106	53.5107	8.74
chrX	13273367	13273652	HDAC4	763	14.7	4.17
chrX	13277071	13279106	HDAC4	6071	246.039	21.69
chrX	13280394	13280719	HDAC4	5038	17.6934	4.13
chrX	13284373	13285600	HDAC4	922	167.951	15.62
chrX	13288004	13288536	CG15744	252	37.2267	6.47
chrX	13300574	13301461	CG1764	1177	161.046	16.39
chrX	13302117	13303025	CG1764	342	75.427	8.43
chrX	13303823	13304588	CR45018	80	145.691	16.72
chrX	13310886	13311212	IP3K2	4029	15.2212	3.89
chrX	13314889	13315291	IP3K2	94	21.487	5.00
chrX	13319472	13320123	IP3K2	1557	316.742	37.34
chrX	13320510	13321676	IP3K2	11	105.937	13.11
chrX	13322249	13322942	IP3K2	1213	227.658	22.06
chrX	13329592	13330285	Jafrac1	107	152.945	14.49
chrX	13332061	13333758	CR32636	337	19.9394	4.30
chrX	13358334	13358592	Syt12	797	13.6626	3.91
chrX	13359059	13359388	Syt12	28	14.0411	4.07
chrX	13371063	13371605	CG1640	331	12.914	3.49
chrX	13373178	13374744	CG1640	52	85.0628	9.16
chrX	13384664	13384958	Rbp1-like	49	16.1989	3.72
chrX	13386792	13387467	Yippee	414	30.556	5.24
chrX	13394876	13395388	CR44621	721	105.642	13.51
chrX	13396625	13397095	CG1673	65	146.313	16.51
chrX	13403770	13405127	GstT4	122	81.8514	10.14
chrX	13411065	13411869	CG1998	10	108.155	12.42
chrX	13412622	13413494	CG1998	5	126.536	11.42
chrX	13412622	13413494	Set2	162	126.536	11.42
chrX	13563511	13564359	Tango13	63	28.3996	4.92
chrX	13615993	13616511	tth	172	26.064	5.15
chrX	13624303	13625853	CG2691	654	237.401	16.17
chrX	13624303	13625853	NFAT	318	237.401	16.17
chrX	13626916	13627204	NFAT	2358	21.437	4.82
chrX	13627628	13628104	NFAT	3103	18.2075	3.81
chrX	13630148	13630951	NFAT	5980	39.9166	6.57
chrX	13636755	13637078	NFAT	5278	14.6478	4.16
chrX	13640068	13641030	NFAT	1427	277.21	22.30
chrX	13641487	13642439	NFAT	194	316.742	34.64
chrX	13696803	13697259	NnaD	344	22.5747	5.26
chrX	13703258	13703533	NnaD	131	20.3193	4.97
chrX	13718036	13718352	CG9940	26	10.0647	3.35
chrX	13736088	13736396	g	1	10.9947	3.41
chrX	13739359	13740513	CG11151	50	216.344	14.68
chrX	13747000	13748593	CR42861	2418	316.742	32.45
chrX	13754500	13755586	CR43833	45	96.1322	9.79
chrX	13754500	13755586	Pdcd4	40	96.1322	9.79
chrX	13761659	13763501	rdgB	469	281.104	20.02
chrX	13763874	13764321	rdgB	1358	131.69	14.32
chrX	13781791	13782466	CtsB1	127	43.0447	5.85
chrX	13781791	13782466	CG11103	415	43.0447	5.85
chrX	13783526	13784596	inaE	66	126.609	13.10
chrX	13805493	13806104	CG11095	5983	95.7323	12.13
chrX	13815809	13816111	CG11095	4235	15.6485	3.82
chrX	13821471	13822458	Clic	55	37.6335	5.55
chrX	13829975	13830261	jub	182	11.3718	3.45
chrX	13839041	13839366	AMPdeam	819	19.5844	4.87
chrX	13844404	13844850	AMPdeam	1206	16.553	4.20
chrX	13845744	13846011	AMPdeam	36	13.8792	3.28
chrX	13879250	13879814	AMPdeam	33548	19.5844	4.87
chrX	13888867	13890764	mamo	43787	151.593	13.77
chrX	13893360	13894160	mamo	39662	102.183	12.61
chrX	13933096	13934426	mamo	710	182.601	17.38
chrX	13935339	13936992	mamo	2798	204.576	18.78
chrX	13963960	13964429	CG11068	8980	49.6123	8.34
chrX	13986315	13987062	mamo	314	316.742	22.42
chrX	13988641	13989394	mamo	276	107.976	13.08
chrX	13990411	13991214	mamo	386	221.494	17.37
chrX	13990411	13991214	CR44655	181	221.494	17.37
chrX	13994652	13995494	ben	1361	316.742	23.27
chrX	13995979	13997222	ben	273	20.7834	4.35
chrX	14037875	14038593	Ste:CG33247	9143	149.454	17.01
chrX	14037875	14038593	Ste:CG33237	9143	149.454	17.01
chrX	14082591	14083275	CR46242	14714	316.742	35.12

chrX	14083611	14085007	CR46242	13771	83.4293	11.22
chrX	14091034	14091345	CR46242	6344	14.5249	4.05
chrX	14208459	14209269	l(1)G0469	2732	58.7158	8.45
chrX	14211164	14212652	l(1)G0469	849	316.742	23.27
chrX	14211164	14212652	l(1)G0007	108	316.742	23.27
chrX	14213800	14214154	l(1)G0007	1538	10.2804	3.14
chrX	14214712	14215575	l(1)G0007	2636	41.2284	6.93
chrX	14217979	14219221	l(1)G0007	5931	316.742	25.71
chrX	14222029	14222446	CG11674	3660	53.3377	8.70
chrX	14233701	14234161	CG42271	171	14.7609	3.63
chrX	14261015	14261406	CG1461	104	11.516	3.68
chrX	14321885	14322657	dpr8	153	23.3434	5.36
chrX	14483337	14483658	CG9411	3376	22.5747	5.26
chrX	14591446	14591898	CG9413	162	36.6468	6.95
chrX	14602360	14602789	CG9413	11	39.2756	6.72
chrX	14653864	14654208	NetA	107	36.5721	6.52
chrX	14748872	14749876	NetB	67	119.332	13.07
chrX	14824843	14826307	rut	41	29.8308	4.76
chrX	14831773	14832066	CG14408	123	7.48484	2.61
chrX	14832510	14832844	CG14411	46	19.8065	4.46
chrX	14839600	14840417	Flo2	78	213.914	16.97
chrX	14929510	14929939	Flo2	77	9.68765	3.24
chrX	14947339	14947791	pdgy	3168	57.513	8.65
chrX	14948693	14949205	pdgy	1691	12.7565	3.87
chrX	14950314	14951764	pdgy	410	207.734	18.73
chrX	14952081	14952799	pdgy	344	316.742	23.34
chrX	14953108	14953882	pdgy	115	97.4456	10.81
chrX	14953108	14953882	CR44831	50	97.4456	10.81
chrX	15022141	15022592	be	2093	59.5055	9.34
chrX	15023679	15025083	be	532	269.073	25.73
chrX	15025661	15026157	be	1506	175.93	18.73
chrX	15043188	15044575	CG5541	398	316.742	28.38
chrX	15048754	15049123	CG5541	52	26.011	5.21
chrX	15062668	15063469	hiw	589	21.8144	5.16
chrX	15068554	15068845	dob	1674	7.85541	2.91
chrX	15069175	15070075	dob	2473	110.363	13.91
chrX	15070713	15071431	dob	4372	10.5286	3.41
chrX	15072840	15073156	Lsd-2	3169	8.62644	3.18
chrX	15074529	15076973	Lsd-2	979	257.88	20.42
chrX	15082580	15083078	CG33178	3	49.3464	8.16
chrX	15086127	15086422	CG5599	100	10.2627	3.18
chrX	15089433	15089836	Rab3-GEF	85	25.5273	5.44
chrX	15139087	15139622	RpL37a	111	14.4561	3.57
chrX	15162931	15163324	CG9095	104	47.6938	8.14
chrX	15175257	15175694	CG43737	24	14.4475	3.96
chrX	15297381	15299438	gce	1080	316.742	37.22
chrX	15297381	15299438	CR44429	628	316.742	37.22
chrX	15297381	15299438	gce	163	316.742	37.22
chrX	15301123	15301715	gce	843	316.742	26.99
chrX	15302262	15302808	gce	2067	191.533	17.49
chrX	15303162	15303887	gce	3079	274.103	21.93
chrX	15306241	15306787	gce	5990	316.742	26.17
chrX	15311853	15312950	CR44961	5820	56.7054	8.37
chrX	15313545	15314498	CR44961	4129	91.6606	11.30
chrX	15319288	15321170	Top1	3	26.5472	5.50
chrX	15319288	15321170	mir-4957	549	26.5472	5.50
chrX	15319288	15321170	Top1	880	26.5472	5.50
chrX	15323255	15323844	Top1	98	52.8514	7.08
chrX	15333053	15333851	CG9114	231	316.742	33.86
chrX	15333053	15333851	HDAC6	76	316.742	33.86
chrX	15356285	15356805	Pp1-13C	4505	48.6506	8.24
chrX	15379267	15379790	acj6	443	23.3434	5.36
chrX	15441091	15441740	Cngl	180	28.9409	6.06
chrX	15443439	15443823	Cngl	2352	16.0475	4.37
chrX	15449374	15450968	Scamp	220	47.0783	6.81
chrX	15449374	15450968	Ahcy13	597	47.0783	6.81
chrX	15452369	15452776	CG11655	868	107.802	12.62
chrX	15453182	15453814	CG11655	21	236.113	19.45
chrX	15465205	15465893	CG6299	110	237.514	23.54
chrX	15483182	15483514	CG6340	980	23.0575	4.88
chrX	15484635	15485041	Gmap	2078	50.3686	7.61
chrX	15497632	15498042	CR44888	33	66.7095	10.03
chrX	15498754	15499671	Gmap	20	42.6146	6.49
chrX	15543367	15543984	CR44354	3922	19.1317	4.32
chrX	15544906	15545469	CR44354	2466	17.4341	4.57

chrX	15545737	15546004	CR44354	1870	13.6062	3.96
chrX	15554148	15554436	Ac13E	5	17.0088	3.68
chrX	15592957	15594617	CG9220	218	316.742	24.48
chrX	15598581	15598843	CG9220	5112	7.58498	2.88
chrX	15601638	15601999	CG9220	8251	20.7519	4.90
chrX	15626215	15626718	sog	21	123.092	13.52
chrX	15631940	15632271	sog	1367	32.2437	5.88
chrX	15633209	15633893	sog	157	316.742	47.95
chrX	15697561	15697940	CG8128	372	5.47695	2.26
chrX	15709647	15710819	CG9281	17	108.391	11.71
chrX	15709647	15710819	CG15601	124	108.391	11.71
chrX	15712600	15713690	CG8184	283	45.9589	6.53
chrX	15760396	15760654	Graf	129	11.516	3.68
chrX	15803330	15803789	PGRP-LE	255	39.3523	6.37
chrX	15804066	15805274	sd	44	316.742	24.58
chrX	15811463	15813914	sd	257	316.742	42.46
chrX	15834456	15835426	Chc	26	316.742	25.46
chrX	15844131	15844400	Aats-arg	170	6.21352	2.45
chrX	15860385	15860902	Gbeta13F	164	88.665	9.84
chrX	15867746	15868494	Gapdh2	17	229.803	17.25
chrX	15886593	15887433	tay	511	139.156	14.89
chrX	15892095	15892385	shi	3	9.07558	2.63
chrX	15892573	15892858	shi	220	11.034	3.13
chrX	15973056	15973645	mmd	223	43.8983	7.63
chrX	15973056	15973645	CR45527	172	43.8983	7.63
chrX	16063521	16063863	dpr18	18	14.3778	3.88
chrX	16072908	16073594	Nipsnap	11	14.8813	3.82
chrX	16085842	16086603	Tob	1289	140.497	14.56
chrX	16087438	16087882	Tob	292	128.066	14.50
chrX	16089051	16089787	Tob	75	316.742	22.92
chrX	16091304	16091925	Tob	183	229.803	17.25
chrX	16092508	16092921	Tob	1195	61.5998	9.16
chrX	16101697	16102019	CG42353	3089	12.8627	3.70
chrX	16101697	16102019	CG42354	3089	12.8627	3.70
chrX	16101697	16102019	CG42353	3089	12.8627	3.70
chrX	16104330	16105184	CG42353	8	69.3184	8.32
chrX	16104330	16105184	CG42354	8	69.3184	8.32
chrX	16104330	16105184	CG42353	8	69.3184	8.32
chrX	16147432	16148556	disco-r	1135	157.807	14.30
chrX	16148826	16149587	disco-r	281	316.742	29.51
chrX	16150399	16151085	disco-r	1796	204.391	18.29
chrX	16216461	16217961	disco	240	316.742	39.60
chrX	16270765	16271228	Mfe2	6	27.8127	5.07
chrX	16278245	16278600	eas	35	16.1275	3.27
chrX	16281671	16282192	eas	3328	61.8639	8.43
chrX	16296247	16297263	CG9911	60	7.81799	2.53
chrX	16296247	16297263	CG3632	23	7.81799	2.53
chrX	16296247	16297263	CG9911	238	7.81799	2.53
chrX	16296247	16297263	CG3632	435	7.81799	2.53
chrX	16319267	16320408	Cyp1	877	19.0757	4.12
chrX	16332549	16333940	CG9921	71	316.742	32.20
chrX	16332549	16333940	Dsp1	82	316.742	32.20
chrX	16334365	16334875	Dsp1	1065	10.6058	2.86
chrX	16372463	16373648	Fur2	38	20.8549	4.19
chrX	16385251	16385927	CG4239	54	110.556	11.07
chrX	16421789	16423387	AnxB11	810	57.4362	8.30
chrX	16421789	16423387	hang	245	57.4362	8.30
chrX	16424287	16425541	hang	2484	98.3183	10.53
chrX	16441724	16442568	rngo	489	70.7211	9.61
chrX	16448532	16449384	Rbp2	105	45.8078	5.83
chrX	16526072	16526595	Cnx14D	899	165.302	18.28
chrX	16531071	16531814	para	1746	28.8275	6.03
chrX	16532857	16533562	para	16	24.1197	5.46
chrX	16552805	16554001	Arp2	228	157.768	13.17
chrX	16559242	16560215	Pp2B-14D	403	316.742	28.15
chrX	16562331	16562626	Pp2B-14D	2457	20.9195	4.63
chrX	16579413	16580016	CanA-14F	6802	18.3275	4.63
chrX	16581979	16582458	CanA-14F	4534	30.6062	6.26
chrX	16585301	16587268	CanA-14F	1153	40.4034	7.17
chrX	16599112	16600106	CG9784	698	54.3603	7.52
chrX	16599112	16600106	Nup153	673	54.3603	7.52
chrX	16607895	16608253	mir-4965	1971	35.2967	6.25
chrX	16610555	16611282	mbt	358	20.0006	4.89
chrX	16637041	16637441	Rok	183	31.5667	5.43
chrX	16637749	16638387	RpS19a	31	232.686	19.02

chrX	16640702	16641186	mthl1	30	36.4947	5.83
chrX	16643494	16643749	CR46147	2572	9.54821	2.97
chrX	16648354	16648675	CR46147	2344	12.1308	3.77
chrX	16650508	16650933	CG42512	571	10.2284	3.33
chrX	16650508	16650933	CG32573	571	10.2284	3.33
chrX	16655007	16656093	CG9723	726	72.7521	9.21
chrX	16655007	16656093	r	272	72.7521	9.21
chrX	16683498	16684080	Axs	96	12.0506	3.06
chrX	16687324	16687870	44078	66	72.8378	10.58
chrX	16694587	16695250	44078	16	21.6072	4.12
chrX	16695773	16696158	CG4678	255	12.2121	3.49
chrX	16696679	16697041	CG4678	769	12.1308	3.77
chrX	16723330	16723870	CG34325	2161	152.007	17.20
chrX	16750725	16751977	goe	489	159.021	15.42
chrX	16773207	16773735	if	7596	118.743	14.60
chrX	16780183	16780729	if	584	175.594	17.15
chrX	16782167	16782826	if	25	103.699	11.01
chrX	16787701	16788030	Sap30	83	16.8268	4.41
chrX	16792139	16792506	CG4768	235	8.21781	2.55
chrX	16835259	16836596	CG13004	764	316.742	35.06
chrX	16844228	16844695	RSG7	16	55.49	8.94
chrX	16897685	16898099	CG33252	702	60.5212	9.44
chrX	16912900	16913735	CG4928	309	170.49	17.28
chrX	16916070	16916367	CG4928	3071	22.982	5.19
chrX	16930655	16931544	CG4928	6209	43.9502	6.05
chrX	16943266	16944582	RhoGAP15B	468	117.874	11.95
chrX	16945134	16945426	RhoGAP15B	2000	11.9316	3.65
chrX	16946747	16947047	CG13000	1576	21.3477	4.40
chrX	16964021	16964420	Ubr1	4459	53.2082	6.98
chrX	16966574	16966896	Ubr1	2055	7.47705	2.76
chrX	16967574	16968790	Ubr1	443	24.4843	4.89
chrX	17047677	17048107	CG45002	15188	36.6468	6.95
chrX	17076542	17078153	CG8949	7	95.5526	8.95
chrX	17091143	17091989	CG16700	553	83.3132	11.06
chrX	17091143	17091989	CG4991	553	83.3132	11.06
chrX	17107535	17107882	CG5004	64	16.447	3.67
chrX	17147738	17148571	RpS5a	78	29.8085	5.46
chrX	17148891	17149417	CG5010	32	27.5697	5.55
chrX	17159872	17160820	baz	568	136.707	14.14
chrX	17164609	17165054	baz	4837	48.6506	8.24
chrX	17165926	17166585	baz	6402	14.8813	3.82
chrX	17170241	17171131	baz	10758	316.742	34.76
chrX	17178473	17179007	CR44026	4413	44.856	7.85
chrX	17299168	17300198	Fim	798	107.426	10.30
chrX	17300476	17301339	Fim	74	28.91	5.59
chrX	17301663	17302029	Fim	38	28.3996	4.92
chrX	17302483	17303009	CG5445	176	24.1122	4.89
chrX	17313256	17313813	B-H2	1005	78.7724	9.46
chrX	17314127	17314820	B-H2	195	99.7442	12.25
chrX	17345839	17346473	B-H2	31652	83.9031	10.18
chrX	17349248	17349676	CR43491	29564	40.2365	7.35
chrX	17397027	17397652	B-H1	180	182.649	17.86
chrX	17423365	17423949	CG5613	188	51.5514	8.54
chrX	17493794	17494284	CR44692	2747	22.479	5.24
chrX	17506246	17507883	CG43658	56	306.918	23.36
chrX	17531794	17532474	CG43658	525	26.4968	5.76
chrX	17577488	17577971	CR44124	963	84.1127	10.70
chrX	17578725	17579102	Ankle2	80	9.83586	2.95
chrX	17579648	17580142	CG12991	27	36.6575	5.65
chrX	17581560	17581989	CG12991	1924	103.042	11.46
chrX	17611499	17611817	X11L	11664	17.4341	4.57
chrX	17624974	17625816	stas	2673	47.0783	6.81
chrX	17627149	17627905	stas	451	7.29525	2.57
chrX	17650825	17652611	RhoGAPp190	263	316.742	23.52
chrX	17657247	17659553	beta-Spec	1696	316.742	29.88
chrX	17680069	17680474	chas	2659	74.7195	10.18
chrX	17682631	17683180	chas	11	40.2461	7.24
chrX	17683534	17683923	chas	799	62.376	9.46
chrX	17694076	17695001	chas	127	48.3816	7.62
chrX	17697989	17699940	chas	49	314.753	21.65
chrX	17702700	17703034	CG8188	111	13.4534	3.61
chrX	17709872	17711182	CG42684	825	134.897	14.36
chrX	17722830	17723511	CG42684	156	46.6065	6.64
chrX	17726074	17726443	CG42684	3084	30.6062	6.26
chrX	17733704	17734160	CG42684	4485	120.529	13.87

chrX	17737950	17739443	CG42684	786	154.936	17.48
chrX	17742509	17743122	CG42684	4495	164.677	15.60
chrX	17767967	17768784	unc-4	194	316.742	28.98
chrX	17788951	17790038	OdsH	1340	150.777	17.12
chrX	17802070	17802756	OdsH	11513	15.7831	3.90
chrX	17826560	17827665	Socs16D	370	316.742	24.33
chrX	17830499	17831237	CG6398	79	150.165	15.23
chrX	17830499	17831237	CR44691	188	150.165	15.23
chrX	17834000	17834356	CR44691	3099	18.0702	4.65
chrX	17839494	17840075	e(y)1	2284	30.5289	5.95
chrX	17856841	17857334	CG6762	27	14.7928	3.69
chrX	17871021	17871531	mnb	3774	71.5856	9.55
chrX	17877945	17879920	mnb	3694	316.742	32.87
chrX	17880407	17880938	CG12985	3000	89.9897	9.86
chrX	17881399	17881968	CG12985	2113	40.6205	5.98
chrX	17885534	17887668	mnb	484	188.351	15.74
chrX	17907582	17908038	Vps4	8	8.525	2.74
chrX	17910621	17911742	CG6847	583	198.265	18.89
chrX	17963019	17964276	Sh	3722	77.363	11.03
chrX	17966917	17967475	Sh	28	21.5412	4.85
chrX	17995595	17995967	Sh	195	18.774	4.75
chrX	17997246	17997530	Sh	1782	16.0475	4.37
chrX	18018818	18019079	Sh	3741	10.3226	3.48
chrX	18023638	18024147	CG6867	7	50.4975	8.30
chrX	18063236	18063716	Sh	305	18.8588	4.77
chrX	18084900	18085665	CG7536	11	17.608	4.06
chrX	18091248	18091525	scu	159	14.8275	3.75
chrX	18101054	18101379	CG7192	83	16.5691	3.84
chrX	18136576	18137933	ari-1	58	127.74	11.60
chrX	18154906	18155540	Frq1	309	20.3193	4.97
chrX	18214433	18214839	Him	5212	22.2306	5.09
chrX	18243175	18244213	upd2	254	316.742	30.78
chrX	18275822	18277012	upd3	939	104.47	13.41
chrX	18282293	18282950	upd3	5333	185.156	17.54
chrX	18312381	18313146	CR45609	839	316.742	48.50
chrX	18318802	18319156	CG6023	2213	39.3302	7.25
chrX	18319901	18320338	CG6023	1111	9.89413	2.93
chrX	18320611	18321650	CG6023	322	209.043	18.81
chrX	18365882	18369118	CrebB	107	238.274	19.82
chrX	18369750	18370133	CR43686	156	25.5054	5.26
chrX	18370827	18371871	CrebB	18	316.742	27.10
chrX	18374391	18375050	CrebB	3460	70.1728	8.46
chrX	18386065	18386627	Tsf1	784	45.1666	6.71
chrX	18387640	18388008	Tsf1	56	25.1395	5.43
chrX	18404355	18405268	CG32549	89	316.742	36.96
chrX	18406581	18406992	CG32549	2237	33.8473	5.95
chrX	18409469	18410467	CG32549	82	101.245	10.01
chrX	18479369	18480030	CG32547	260	84.3983	11.15
chrX	18483409	18484142	Hayan	68	187.125	15.67
chrX	18486552	18487231	psh	14	181.681	18.85
chrX	18489334	18490050	CG15046	93	316.742	38.29
chrX	18499523	18499917	GalNAc-T2	167	13.0285	3.37
chrX	18506826	18507337	Wnt5	1738	76.5372	9.27
chrX	18512091	18512622	Ggt-1	32	35.2316	5.82
chrX	18515329	18516893	Aats-his	647	64.7459	7.91
chrX	18515329	18516893	Bx	647	64.7459	7.91
chrX	18515329	18516893	Aats-his	647	64.7459	7.91
chrX	18534213	18535706	Bx	150	316.742	33.42
chrX	18544164	18544525	Bx	9636	20.1354	4.84
chrX	18545733	18547257	Bx	11484	34.9991	5.86
chrX	18558402	18558714	Bx	7729	14.7	4.17
chrX	18575189	18575608	Bx	9199	12.8627	3.70
chrX	18587267	18587649	CR43492	264	44.7029	7.81
chrX	18634622	18635118	wgn	2	217.798	16.81
chrX	18636291	18636653	wgn	1573	23.2208	4.92
chrX	18645946	18646295	Rip11	1431	22.2685	4.94
chrX	18646737	18647726	Rip11	288	117.329	11.42
chrX	18665784	18666209	CR45514	79	23.3434	5.36
chrX	18679313	18679630	CR45710	2679	10.3226	3.48
chrX	18692701	18693435	Cyp18a1	188	25.6968	5.66
chrX	18789173	18790305	S6KL	60	84.8759	9.81
chrX	18793711	18794008	Atg101	2104	7.56868	2.93
chrX	18797510	18797869	bnb	30	29.8347	5.88
chrX	18802841	18803192	CR44841	3296	22.4872	5.07
chrX	18806422	18806795	CG7058	113	53.5107	8.74

chrX	18813010	18813848	CG42450	74	43.9209	7.75
chrX	18814327	18814658	CG42450	1137	13.3929	3.97
chrX	18832224	18833108	Pvf1	154	316.742	47.41
chrX	18843450	18843803	CG34422	67	21.5869	4.51
chrX	18848421	18848712	CG7280	30	11.9067	3.71
chrX	18857299	18857815	CG7322	97	109.078	12.33
chrX	18863805	18865320	CG34401	1074	77.7171	9.70
chrX	18884590	18884982	CG7332	253	16.2083	4.05
chrX	18899567	18900678	CG7378	105	226.604	20.26
chrX	18902547	18902975	CG7378	2288	23.7462	5.11
chrX	18905273	18905850	CG7378	5374	7.05037	2.88
chrX	18929052	18929406	CG43759	1087	22.7445	5.05
chrX	18937839	18938539	CG43759	7639	18.8665	4.61
chrX	19146638	19146912	RhoGAP18B	6692	16.0475	4.37
chrX	19152949	19156244	RhoGAP18B	359	316.742	38.82
chrX	19167268	19167604	RhoGAP18B	373	30.6062	6.26
chrX	19168074	19168406	mir-4959	1019	22.2685	4.94
chrX	19174747	19175132	CG33939	58	21.1897	4.62
chrX	19187567	19188258	Mec2	93	114.096	13.03
chrX	19201090	19201845	CG7990	134	13.3929	3.97
chrX	19220387	19220685	CG7884	88	18.1418	4.67
chrX	19248476	19248964	Hs3st-B	37	35.9255	6.38
chrX	19259898	19260392	rictor	228	58.4945	9.24
chrX	19260667	19261317	rictor	384	28.0078	5.93
chrX	19261874	19262276	Vav	279	11.0815	3.38
chrX	19297637	19299163	CG8034	637	316.742	25.71
chrX	19315500	19315983	out	6376	180.892	17.84
chrX	19317203	19317762	out	4553	52.7684	7.50
chrX	19321733	19324288	out	490	316.742	24.33
chrX	19348156	19348574	nAChRalpha7	60	17.4146	4.13
chrX	19353017	19354006	kek5	509	316.742	27.18
chrX	19360938	19361593	kek5	8114	316.742	23.66
chrX	19395103	19395417	kek5	42114	8.5523	3.00
chrX	19398463	19398849	kek5	45503	11.4211	3.12
chrX	19479921	19480797	Nat1	426	19.4415	4.14
chrX	19479921	19480797	mRpS14	171	19.4415	4.14
chrX	19479921	19480797	Nat1	151	19.4415	4.14
chrX	19495390	19497225	CG14200	1224	277.132	21.21
chrX	19495390	19497225	Pfrx	1224	277.132	21.21
chrX	19495390	19497225	CR44340	832	277.132	21.21
chrX	19495390	19497225	Pfrx	54	277.132	21.21
chrX	19498004	19499368	Pfrx	723	100.754	11.17
chrX	19503342	19503913	Pfrx	836	82.3699	10.86
chrX	19504213	19504615	Pfrx	49	15.0743	3.69
chrX	19505779	19506207	Pfrx	108	57.2932	8.49
chrX	19520968	19523035	l(1)G0156	1063	43.2617	6.69
chrX	19520968	19523035	CoRest	775	43.2617	6.69
chrX	19546084	19547031	CG32532	630	23.3434	5.36
chrX	19565292	19565800	Grip84	107	49.6896	7.02
chrX	19578363	19579873	Tao	431	24.8752	4.59
chrX	19597756	19598125	RpS10b	33	7.89471	2.87
chrX	19604336	19604669	CG14207	722	28.658	5.99
chrX	19605055	19605441	CG14207	43	33.0785	5.43
chrX	19605852	19606217	CG14207	766	13.1132	3.48
chrX	19608001	19608734	CG14207	1721	90.1	10.41
chrX	19631234	19632028	CG14212	5822	316.742	25.66
chrX	19636829	19638861	e(y)3	1247	121.065	11.00
chrX	19636829	19638861	Rcd-1	1644	121.065	11.00
chrX	19643310	19643584	Sec61gamma	143	10.8303	3.17
chrX	19652234	19652551	Ranbp21	89	9.05003	2.90
chrX	19652234	19652551	CG14215	54	9.05003	2.90
chrX	19662700	19663838	CG14223	303	316.742	31.03
chrX	19665442	19666220	Ssu72	1334	245.399	19.98
chrX	19667035	19668877	Zw	1195	96.2193	10.39
chrX	19667035	19668877	CR44512	700	96.2193	10.39
chrX	19669426	19669852	Zw	205	12.6166	3.59
chrX	19681494	19682177	dome	1547	25.3684	4.87
chrX	19682732	19684209	dome	390	316.742	46.59
chrX	19682732	19684209	Ubqn	679	316.742	46.59
chrX	19693172	19693463	Mer	191	9.73133	3.10
chrX	19696895	19697896	Cdc42	23	107.291	11.63
chrX	19712560	19713055	CR44889	45	31.5663	5.99
chrX	19712560	19713055	meso18E	157	31.5663	5.99
chrX	19718270	19719065	CG12531	379	98.4315	11.99
chrX	19735362	19735617	CG14234	27	13.3263	3.95

chrX	19744020	19744522	COX6B	216	26.2963	5.05
chrX	19744020	19744522	CG18809	71	26.2963	5.05
chrX	19744020	19744522	COX6B	71	26.2963	5.05
chrX	19744020	19744522	CG33932	166	26.2963	5.05
chrX	19744020	19744522	Rpp20	166	26.2963	5.05
chrX	19744020	19744522	CG33932	166	26.2963	5.05
chrX	19744020	19744522	Rpp20	166	26.2963	5.05
chrX	19750642	19751304	Pmp70	286	140.598	14.04
chrX	19753858	19755177	Pmp70	612	223.089	19.94
chrX	19846774	19847382	pico	1317	36.9723	6.42
chrX	19847943	19848743	pico	11	222.824	17.07
chrX	19850863	19852438	pico	649	183.298	13.99
chrX	19850863	19852438	Nup205	803	183.298	13.99
chrX	19865925	19867345	Hers	434	316.742	30.06
chrX	19874799	19875989	Hers	290	109.404	13.38
chrX	19877040	19877468	Hers	1537	78.4506	11.12
chrX	19886703	19888089	Hers	308	316.742	27.08
chrX	19889284	19889601	amn	558	14.5206	4.12
chrX	19961054	19961827	Nep3	114	23.1387	4.76
chrX	20147794	20148286	CG9572	49	115.134	14.30
chrX	20158832	20159990	Rab35	733	316.742	32.02
chrX	20173229	20174117	AnxB10	37	316.742	21.22
chrX	20236227	20236485	sw	58	21.0144	4.88
chrX	20251235	20251690	Rab10	209	13.8643	3.40
chrX	20253726	20254053	Rab10	2579	21.487	5.00
chrX	20256886	20257214	CG17065	45	17.9731	4.13
chrX	20330301	20330637	CG1504	68	16.7357	4.47
chrX	20352440	20352702	CG1504	22178	14.0411	4.07
chrX	20411611	20412062	CG15459	1741	128.498	15.40
chrX	20419365	20420046	Obp19d	424	6.59975	2.58
chrX	20430088	20430418	mal	75	20.2224	4.55
chrX	20433799	20434714	CG1695	510	26.5472	5.50
chrX	20436832	20437405	CG1695	3128	67.4896	9.55
chrX	20468084	20468662	GstT3	54	129.731	15.49
chrX	20483196	20483499	CG15461	2781	18.1418	4.67
chrX	20488944	20489326	RhoGAP19D	2079	60.5212	9.44
chrX	20489640	20490006	RhoGAP19D	1379	63.4004	9.69
chrX	20510454	20510868	RhoGAP19D	22	10.1576	2.69
chrX	20511482	20511784	CG1812	73	11.2051	3.04
chrX	20524566	20525325	Tak1	240	24.1197	5.46
chrX	20656129	20657715	Cyp6v1	43	316.742	25.38
chrX	20656129	20657715	CR45466	470	316.742	25.38
chrX	20656129	20657715	Cyp6v1	508	316.742	25.38
chrX	20656129	20657715	CR45466	600	316.742	25.38
chrX	20760357	20760751	CR43838	419	33.845	5.50
chrX	20924889	20925403	shakB	109	44.856	7.85
chrX	21010260	21011149	r-cup	14917	34.8887	6.75
chrX	21028120	21028433	CG1532	87	24.7547	4.68
chrX	21035405	21035986	Ntf-2	161	15.0084	3.97
chrX	21042493	21043393	Mgstl	97	127.602	12.20
chrX	21045044	21045584	Cbs	50	40.3169	6.47
chrX	21053080	21055533	CG1518	521	316.742	28.84
chrX	21053080	21055533	bves	87	316.742	28.84
chrX	21067355	21068601	CG32512	104	197.663	19.99
chrX	21071587	21072260	CG32512	4437	145.49	12.61
chrX	21120377	21120828	CR44374	334	19.9502	4.56
chrX	21123697	21123973	CG34120	208	10.8116	3.42
chrX	21127972	21128345	CG34120	4207	10.3723	3.37
chrX	21214847	21215232	CG32523	2783	14.0411	4.07
chrX	21231345	21232189	CG32523	14082	57.4883	7.81
chrX	21280625	21281495	CG32521	73	203.631	15.90
chrX	21306423	21307152	CG1718	63	281.818	19.24
chrX	21319094	21319410	CG33713	76	15.3953	3.76
chrX	21319094	21319410	SLIRP1	76	15.3953	3.76
chrX	21319094	21319410	CG33713	7	15.3953	3.76
chrX	21319094	21319410	SLIRP1	7	15.3953	3.76
chrX	21323964	21324756	Dd	304	20.2236	3.88
chrX	21323964	21324756	CG1486	247	20.2236	3.88
chrX	21338049	21338625	tty	655	35.3032	6.54
chrX	21339584	21339981	tty	118	16.7966	4.33
chrX	21339584	21339981	flil	54	16.7966	4.33
chrX	21350135	21350836	sol	51	13.3929	3.97
chrX	21358183	21358660	44075	74	15.4354	4.07
chrX	21366218	21369020	bbx	187	292.689	20.15
chrX	21369344	21369807	bbx	86	54.7871	7.17

chrX	21373878	21374558	sIgA	80	89.0802	9.33
chrX	21373878	21374558	CR45611	186	89.0802	9.33
chrX	21375358	21375924	sIgA	249	199.079	18.40
chrX	21378435	21378755	sIgA	2507	15.163	3.82
chrX	21629546	21629968	DIP1	531	8.54165	3.00
chrX	21634116	21634447	flam	2388	52.5284	8.64
chrX	22449159	22449563	CG14615	10	26.4968	5.76
chrX	22455764	22456999	Usp2	109	51.2354	7.05
chrX	22478336	22478741	CG12576	911	20.3795	4.66
chrX	22480724	22482045	CG12576	723	114.186	11.34
chrX	22487106	22488479	l(1)G0196	965	88.5078	8.16
chrX	22490653	22491800	CR43493	603	208.461	20.74
chrX	22490653	22491800	l(1)G0196	119	208.461	20.74
chrX	22539946	22540376	CG17598	227	48.5443	7.45
chrX	22854983	22855471	fog	85	211.496	19.76
chrX	22950024	22950385	CG41106	4916	61.4394	6.35

Supplementary Table 5 Rpd3 binding sites
The regions indicated by 'Start' and 'End' represent the called peak region,
the results of which were indicated as 'Q score' and 'Fold enrichment'.

Chromosome	Start	End	Gene Symbol	Distance to TSS (bp)	Q score (-log10(q value)	Fold enrichment
chr2L	72320	74246	galectin	396	15.5557	3.36
chr2L	72320	74246	CR44987	1054	15.5557	3.36
chr2L	72320	74246	galectin	1346	15.5557	3.36
chr2L	127740	129684	CG3164	65	16.7973	3.46
chr2L	158469	159458	CG33635	426	30.3276	4.81
chr2L	158469	159458	spen	59	30.3276	4.81
chr2L	160433	162171	spen	1928	16.4431	3.59
chr2L	246733	247126	CR44218	1965	19.8919	3.95
chr2L	250406	251200	kis	15	16.9888	3.42
chr2L	252025	252432	Rpp30	54	13.4834	3.46
chr2L	273218	274300	mbm	172	14.7199	3.72
chr2L	273218	274300	CG11555	292	14.7199	3.72
chr2L	276875	278204	CG17078	127	30.7884	4.93
chr2L	276875	278204	smo	496	30.7884	4.93
chr2L	282753	283462	CG11601	89	16.4388	4.04
chr2L	285247	285875	CG3625	58	19.9791	3.83
chr2L	295047	295606	Hop	186	12.6967	3.08
chr2L	297428	298615	Pi3K21B	151	29.5482	4.78
chr2L	309899	310479	Plc21C	98	11.4702	3.02
chr2L	377674	378390	al	137	19.3012	4.70
chr2L	414129	415684	AP-2alpha	264	20.8154	3.97
chr2L	414129	415684	ebi	25	20.8154	3.97
chr2L	419838	421715	RpLP1	162	24.478	4.17
chr2L	419838	421715	CG11885	1337	24.478	4.17
chr2L	430872	431822	ex	54	21.5451	4.29
chr2L	471537	472619	CG4297	6	29.2808	4.34
chr2L	478740	480262	cbt	40	72.5606	7.23
chr2L	523305	523959	ush	85	35.6204	5.06
chr2L	541427	542643	lwr	149	25.3044	4.71
chr2L	544090	544781	Spp	147	15.4097	3.66
chr2L	559427	560471	Tspo	43	19.8093	3.85
chr2L	559427	560471	rempA	656	19.8093	3.85
chr2L	567683	568930	Nle	116	23.1472	4.54
chr2L	567683	568930	CG2807	334	23.1472	4.54
chr2L	819093	820536	CG3876	658	23.0519	4.58
chr2L	819093	820536	Clp	203	23.0519	4.58
chr2L	825144	826779	CG3862	266	21.6471	3.90
chr2L	825144	826779	dock	159	21.6471	3.90
chr2L	850159	851230	CR44060	250	27.7017	4.80
chr2L	850159	851230	drongo	120	27.7017	4.80
chr2L	852251	853282	CG4291	239	35.9999	5.22
chr2L	852251	853282	kraken	122	35.9999	5.22
chr2L	865310	866438	snoRNA:Me28S-U123(	608	15.7576	3.64
chr2L	865310	866438	aru	240	15.7576	3.64
chr2L	866812	868466	aru	77	38.1834	5.95
chr2L	1077490	1078431	S	185	33.2559	4.76
chr2L	1077490	1078431	ast	53	33.2559	4.76
chr2L	1107876	1108674	mtRNApol	91	76.7397	7.27
chr2L	1107876	1108674	CG14339	257	76.7397	7.27

chr2L	1117859	1119416	Pino	200	12.5755	3.19
chr2L	1128249	1129799	Pino	122	29.2758	5.27
chr2L	1128249	1129799	CG4552	484	29.2758	5.27
chr2L	1157337	1158289	capt	204	38.487	4.99
chr2L	1158608	1159377	Tango14	192	25.6982	4.61
chr2L	1158608	1159377	CR43767	503	25.6982	4.61
chr2L	1164298	1164971	IntS14	204	14.4125	3.70
chr2L	1169519	1170560	Plap	143	21.2132	4.64
chr2L	1169519	1170560	CG31922	381	21.2132	4.64
chr2L	1169519	1170560	Plap	566	21.2132	4.64
chr2L	1420214	1420666	robo2	53	16.8296	3.85
chr2L	1610764	1611703	CG31935	104	16.3945	3.72
chr2L	1610764	1611703	CG14352	356	16.3945	3.72
chr2L	1613483	1614368	RFeSP	98	19.2849	3.88
chr2L	1644388	1644775	CR45695	2636	12.1351	3.61
chr2L	1709774	1711177	CG17660	174	28.9759	5.08
chr2L	1709774	1711177	mRpL48	134	28.9759	5.08
chr2L	1714362	1716151	frtz	199	13.0377	3.41
chr2L	1722500	1724601	Rim2	106	43.8516	6.30
chr2L	1751096	1752259	CG17712	135	11.783	3.47
chr2L	1884610	1884993	CG7337	150	20.2246	3.91
chr2L	1902889	1903421	CG7337	8662	11.4242	3.24
chr2L	1911695	1911964	CG7337	116	13.0103	3.40
chr2L	1972661	1973271	Der-1	1038	13.4627	3.70
chr2L	1973954	1974851	Der-1	196	24.1656	4.21
chr2L	1987250	1987946	Got2	108	44.5065	5.23
chr2L	2037504	2039068	Su(dx)	123	38.8667	5.44
chr2L	2144663	2145218	tho2	272	38.4074	5.72
chr2L	2151714	2152260	AIF	256	12.7602	3.19
chr2L	2168902	2169505	aop	7583	12.8637	3.51
chr2L	2178067	2178869	aop	42	33.4869	4.43
chr2L	2191511	2192688	CG10874	97	16.5352	3.61
chr2L	2193382	2193904	CG34172	631	65.5038	7.66
chr2L	2196365	2198316	CG34172	1204	35.3983	4.88
chr2L	2198700	2199031	CG34172	946	10.2141	3.02
chr2L	2199685	2200547	CG34172	2403	19.3052	3.89
chr2L	2201294	2203604	CG31668	3478	36.7097	5.27
chr2L	2216634	2217889	CG42371	111	28.4603	4.76
chr2L	2216634	2217889	CG15386	111	28.4603	4.76
chr2L	2216634	2217889	mio	68	28.4603	4.76
chr2L	2221816	2222430	papi	35	22.7821	4.12
chr2L	2362235	2365895	Rab5	314	27.3933	4.69
chr2L	2362235	2365895	Axud1	937	27.3933	4.69
chr2L	2375109	2376639	CR44982	784	46.9461	6.33
chr2L	2375109	2376639	Atxn7	34	46.9461	6.33
chr2L	2427957	2429162	dpp	14	9.23195	3.09
chr2L	2492231	2493395	Slh	329	23.3105	3.71
chr2L	2492231	2493395	oaf	46	23.3105	3.71
chr2L	2584691	2585600	Drp1	126	13.0517	3.33
chr2L	2747850	2748787	Pgk	49	36.6381	5.61
chr2L	2749311	2751615	Pgk	274	14.2988	3.12
chr2L	2752147	2754633	Bacc	513	42.583	5.30
chr2L	2752147	2754633	Pgk	244	42.583	5.30
chr2L	2752147	2754633	Bacc	80	42.583	5.30
chr2L	2759139	2760443	Snapin	193	19.9342	3.91
chr2L	2759139	2760443	CG9960	193	19.9342	3.91

chr2L	2759139	2760443	Trn-SR	439	19.9342	3.91
chr2L	2765165	2766680	CG3077	766	27.4579	4.87
chr2L	2765165	2766680	aph-1	199	27.4579	4.87
chr2L	2767447	2768566	Taf10b	85	13.4155	3.32
chr2L	2767447	2768566	Taf10	41	13.4155	3.32
chr2L	2855514	2857459	CG3104	377	35.8458	4.99
chr2L	2855514	2857459	RpS21	45	35.8458	4.99
chr2L	2855514	2857459	CG2991	1008	35.8458	4.99
chr2L	2873718	2875617	CG31694	205	24.5309	4.46
chr2L	2873718	2875617	CG8814	605	24.5309	4.46
chr2L	2877851	2879462	Prx6005	519	24.1296	4.12
chr2L	2877851	2879462	CG31950	232	24.1296	4.12
chr2L	2877851	2879462	betaggt-II	360	24.1296	4.12
chr2L	2884346	2885421	NTPase	54	50.5133	6.63
chr2L	2885745	2886809	NTPase	156	22.7821	4.12
chr2L	2885745	2886809	lilli	78	22.7821	4.12
chr2L	2955992	2957022	Rbp9	9	9.51149	3.07
chr2L	2992599	2993758	okr	469	26.8997	4.56
chr2L	2992599	2993758	CG3558	256	26.8997	4.56
chr2L	3145303	3145866	ND-B14.5B	109	15.7576	3.64
chr2L	3163617	3164015	Hydr2	141	24.559	5.40
chr2L	3301771	3303814	CG9663	956	27.9865	4.97
chr2L	3354625	3355123	E23	45	17.1334	3.86
chr2L	3465496	3466862	msl-2	254	12.7616	3.31
chr2L	3465496	3466862	ND-PDSW	210	12.7616	3.31
chr2L	3477985	3480135	Thor	15	21.6997	3.72
chr2L	3507085	3507521	tim	111	14.7932	3.17
chr2L	3631725	3632870	for	75	31.0824	5.62
chr2L	3656672	3657015	for	64	32.7114	5.65
chr2L	3712645	3713839	CG31955	362	17.797	3.94
chr2L	3712645	3713839	CG2818	108	17.797	3.94
chr2L	3771083	3772324	bowl	108	13.6339	3.69
chr2L	3803123	3804139	Reph	347	17.7161	3.62
chr2L	3805496	3806630	Reph	33	33.7434	5.50
chr2L	3812942	3813597	CG3407	49	19.4031	3.78
chr2L	3872016	3872949	CG31957	228	42.6021	6.45
chr2L	4031273	4031885	ed	82	14.4751	3.52
chr2L	4197175	4198173	CG3714	221	12.5115	3.08
chr2L	4226442	4227037	CG3702	98	13.7586	3.38
chr2L	4332678	4334486	Atet	67	58.3048	8.15
chr2L	4393482	4394760	CG3652	423	17.678	4.08
chr2L	4393482	4394760	l(2)k05819	104	17.678	4.08
chr2L	4457375	4458428	snoRNA:Me18S-C183[illegible]	582	40.7365	5.85
chr2L	4457375	4458428	RpL27A	61	40.7365	5.85
chr2L	4459231	4460255	mRpL27	214	15.382	3.66
chr2L	4459231	4460255	CG15435	340	15.382	3.66
chr2L	4476750	4477187	ine	34	23.4616	5.07
chr2L	4591785	4592261	dpy	5	24.6768	5.52
chr2L	4594738	4595179	dpy	82	35.6212	5.38
chr2L	4614087	4614727	dpy	147	23.445	5.14
chr2L	4810061	4811089	CR45297	1682	109.174	11.51
chr2L	4811395	4812060	CR45297	153	20.0579	3.93
chr2L	4837073	4838646	tank	197	23.4245	4.05
chr2L	4837073	4838646	CG12194	544	23.4245	4.05
chr2L	4846628	4848354	mxt	127	13.233	3.32
chr2L	4846628	4848354	CG11927	597	13.233	3.32

chr2L	4849967	4850385	mRpS2	151	9.71429	3.12
chr2L	4891900	4892371	CG3036	60	12.6879	3.34
chr2L	4928125	4928642	hoe1	7	18.825	4.45
chr2L	4944919	4946271	mRpL24	342	20.5596	3.89
chr2L	4944919	4946271	betaggt-I	125	20.5596	3.89
chr2L	4949321	4949815	jet	195	20.4022	4.35
chr2L	4965653	4966361	CG34125	435	11.9605	3.46
chr2L	4967176	4967806	Trip1	233	12.5073	3.34
chr2L	4971016	4972303	l(2)05714	363	13.5982	3.31
chr2L	4971016	4972303	CG14043	147	13.5982	3.31
chr2L	4973748	4974594	l(2)05714	213	20.28	4.32
chr2L	4973748	4974594	mRpL28	159	20.28	4.32
chr2L	4976456	4977588	Gmd	96	16.6352	3.59
chr2L	4976456	4977588	CG3792	384	16.6352	3.59
chr2L	4980569	4982418	CG8892	132	16.6443	3.37
chr2L	4980569	4982418	CG34126	111	16.6443	3.37
chr2L	5009417	5010176	Rtnl1	7	19.496	3.47
chr2L	5043078	5044044	CG31650	363	15.2265	3.56
chr2L	5043078	5044044	CG44001	252	15.2265	3.56
chr2L	5043078	5044044	CG44000	252	15.2265	3.56
chr2L	5043078	5044044	CG44001	252	15.2265	3.56
chr2L	5043078	5044044	CG33995	252	15.2265	3.56
chr2L	5043078	5044044	CG31650	487	15.2265	3.56
chr2L	5052064	5053220	eIF-3p40	98	61.6356	6.90
chr2L	5052064	5053220	CG14040	160	61.6356	6.90
chr2L	5052064	5053220	eIF-3p40	412	61.6356	6.90
chr2L	5070877	5071965	RpL37A	192	20.5163	4.92
chr2L	5070877	5071965	CR45305	82	20.5163	4.92
chr2L	5096301	5096853	CG4230	169	14.1855	3.24
chr2L	5107535	5109184	Msp300	90	116.655	11.66
chr2L	5278939	5279569	Bub1	201	19.3828	4.09
chr2L	5303226	5303957	vri	2231	21.3578	4.61
chr2L	5314557	5315037	CG14024	55	11.421	3.38
chr2L	5324661	5326880	CR44985	1082	28.7933	4.99
chr2L	5324661	5326880	Ncoa6	1262	28.7933	4.99
chr2L	5461111	5461882	mid	239	28.3503	4.33
chr2L	5519352	5520801	CG7382	670	15.0625	3.28
chr2L	5519352	5520801	cl	307	15.0625	3.28
chr2L	5541625	5543759	Hel25E	439	33.5262	5.66
chr2L	5541625	5543759	Lam	25	33.5262	5.66
chr2L	5740662	5741161	CG11147	75	12.1149	3.35
chr2L	5799177	5800405	CG9171	63	35.5342	5.43
chr2L	5801345	5802626	CG14005	167	17.4445	3.65
chr2L	5801345	5802626	CG7239	411	17.4445	3.65
chr2L	5943579	5945296	Gpdh	45	40.6741	5.38
chr2L	5945565	5946602	Gpdh	2158	14.3258	3.63
chr2L	5949173	5949958	CG9044	184	44.3212	6.17
chr2L	5979775	5981203	chic	139	45.3174	5.24
chr2L	5981470	5983231	eIF-4a	164	36.2081	5.14
chr2L	5986164	5987183	ifc	146	25.4504	4.92
chr2L	5998474	5999713	lid	227	27.8429	4.94
chr2L	6000177	6000775	Tsp26A	214	22.1747	4.85
chr2L	6046922	6049318	slmo	188	39.6662	5.68
chr2L	6046922	6049318	CG12393	255	39.6662	5.68
chr2L	6046922	6049318	CG34179	960	39.6662	5.68
chr2L	6046922	6049318	slmo	960	39.6662	5.68

chr2L	6046922	6049318	CG34179	960	39.6662	5.68
chr2L	6046922	6049318	slmo	960	39.6662	5.68
chr2L	6050702	6051761	CG9135	59	23.7979	4.85
chr2L	6068939	6069606	CG13993	7	18.6029	4.10
chr2L	6070134	6071921	CG9154	701	19.3727	4.14
chr2L	6070134	6071921	Kr-h2	319	19.3727	4.14
chr2L	6078434	6079635	CR9162	555	30.2527	4.47
chr2L	6082212	6083632	Kr-h1	47	15.4841	3.08
chr2L	6098171	6099170	CG9175	198	27.9616	4.90
chr2L	6125062	6125737	Arpc4	270	15.191	4.06
chr2L	6323578	6324887	PDZ-GEF	339	22.4931	3.83
chr2L	6337916	6339762	Pez	232	22.5672	3.92
chr2L	6337916	6339762	Cpr	68	22.5672	3.92
chr2L	6455052	6456896	retm	120	20.4089	3.64
chr2L	6455052	6456896	frj	242	20.4089	3.64
chr2L	6465472	6465913	CG16947	66	15.9777	3.73
chr2L	6467452	6467851	CG9531	8	12.3916	3.20
chr2L	6473362	6474021	mmy	187	31.7094	4.92
chr2L	6476396	6477076	CG9536	183	12.8698	3.33
chr2L	6478928	6482252	Sec61alpha	278	44.0676	6.27
chr2L	6478928	6482252	Daxx	75	44.0676	6.27
chr2L	6478928	6482252	CR45463	77	44.0676	6.27
chr2L	6487953	6489826	KFase	74	13.5778	3.39
chr2L	6487953	6489826	epsilonCOP	548	13.5778	3.39
chr2L	6490369	6492068	CG9548	404	42.224	6.31
chr2L	6490369	6492068	CG31638	187	42.224	6.31
chr2L	6498426	6498949	CG31637	78	24.6739	4.04
chr2L	6557818	6558425	COX5B	190	22.9185	4.61
chr2L	6561029	6561735	CG9596	85	11.4366	3.29
chr2L	6562095	6562738	CG9596	16	12.6775	3.42
chr2L	6562095	6562738	Ent2	19	12.6775	3.42
chr2L	6612649	6613379	CG11050	1211	32.6178	4.55
chr2L	6647435	6649971	CG31635	416	21.6959	4.12
chr2L	6647435	6649971	Tango1	143	21.6959	4.12
chr2L	6673843	6674355	CG34310	140	18.3627	3.86
chr2L	6683696	6684901	Nhe3	139	22.7867	4.65
chr2L	6722809	6723446	homer	159	43.9002	5.63
chr2L	6723882	6724942	Liprin-alpha	287	44.4018	6.06
chr2L	6786590	6787969	nrv1	60	32.1995	4.80
chr2L	6794259	6795008	nrv2	47	20.833	3.77
chr2L	6853997	6854526	l(2)k09022	0	14.4751	3.52
chr2L	6913492	6914249	Wee1	297	19.3781	4.26
chr2L	6918084	6918933	nop5	48	28.8943	4.54
chr2L	6919371	6920304	x16	513	16.6155	3.47
chr2L	6920654	6922337	Hrb27C	70	20.8154	3.97
chr2L	6948275	6948909	ihog	42	20.9064	4.79
chr2L	6949510	6950948	Gas41	424	16.8251	4.25
chr2L	6949510	6950948	SA	211	16.8251	4.25
chr2L	6966736	6967735	smt3	10	16.9888	3.42
chr2L	6971179	6972052	snRNP-U1-70K	151	18.9749	3.83
chr2L	7026579	7028024	CG31908	157	40.4123	5.40
chr2L	7026579	7028024	CG3476	395	40.4123	5.40
chr2L	7031891	7033575	Rab30	259	18.2727	3.67
chr2L	7031891	7033575	Caper	145	18.2727	3.67
chr2L	7056147	7057135	Mnn1	109	39.9996	6.52
chr2L	7219781	7220222	CG13784	38	19.1359	3.48

chr2L	7412261	7412930	chm	199	46.1596	6.86
chr2L	7418162	7418769	CR43857	5185	13.9434	3.55
chr2L	7423454	7425210	CR43857	37	29.6758	4.75
chr2L	7426999	7427663	CG5261	234	11.0989	3.02
chr2L	7437378	7438215	CG5973	43	15.7978	3.27
chr2L	7450966	7451314	santa-maria	77	12.8698	3.33
chr2L	7495463	7496487	Ziz	54	25.2495	4.20
chr2L	7701388	7701835	Tep2	15	25.6301	5.24
chr2L	7702188	7703266	Tep3	29	11.5205	3.14
chr2L	7782764	7783258	CG7154	100	29.8446	5.31
chr2L	7809442	7810828	cdc14	44	30.659	5.33
chr2L	7826546	7827396	Rack1	36	25.1121	4.36
chr2L	7827717	7829441	mts	547	23.5825	3.85
chr2L	7885194	7886093	SmE	12	10.9657	3.14
chr2L	7885194	7886093	CG34132	64	10.9657	3.14
chr2L	7976472	7977037	Snoo	132	10.2469	3.52
chr2L	7993906	7994354	pes	70	19.6858	4.10
chr2L	7998747	7999515	Sirup	89	33.9269	4.81
chr2L	7999839	8001027	Sirup	1487	12.7356	3.44
chr2L	8009115	8009706	CG12560	52	29.3214	5.15
chr2L	8030723	8032364	Cka	98	17.8941	3.40
chr2L	8043289	8044052	CG7466	166	17.3411	3.48
chr2L	8071155	8071733	poe	130	23.157	3.97
chr2L	8072255	8073349	MED20	206	18.0528	3.89
chr2L	8072255	8073349	Trf	160	18.0528	3.89
chr2L	8082331	8084719	CR45257	649	24.3601	4.48
chr2L	8082331	8084719	Mcr	525	24.3601	4.48
chr2L	8082331	8084719	Bsg	88	24.3601	4.48
chr2L	8162886	8163653	Piezo	40	39.34	5.25
chr2L	8239364	8240233	Pvr	94	21.3964	4.32
chr2L	8307661	8308945	Ostgamma	145	19.218	4.17
chr2L	8307661	8308945	CG7818	379	19.218	4.17
chr2L	8320197	8320875	CG7787	24	8.67665	3.09
chr2L	8363882	8365587	RpS13	62	51.8015	6.43
chr2L	8363882	8365587	CSN8	320	51.8015	6.43
chr2L	8365881	8366651	Pp2A-29B	250	27.1219	4.36
chr2L	8370341	8371115	CG17292	78	29.1348	5.31
chr2L	8370341	8371115	CR45341	63	29.1348	5.31
chr2L	8383566	8384899	l(2)k12914	438	16.0949	3.96
chr2L	8383566	8384899	CG13384	121	16.0949	3.96
chr2L	8402784	8404331	fy	408	31.1443	4.35
chr2L	8402784	8404331	emb	116	31.1443	4.35
chr2L	8402784	8404331	CR44991	236	31.1443	4.35
chr2L	8415696	8417015	Akap200	730	10.1684	3.01
chr2L	8511471	8512071	CG13096	13	12.3916	3.20
chr2L	8527867	8528634	ImgB	158	13.9982	3.77
chr2L	8527867	8528634	ImgA	281	13.9982	3.77
chr2L	8527867	8528634	ImgB	281	13.9982	3.77
chr2L	8527867	8528634	ImgA	281	13.9982	3.77
chr2L	8527867	8528634	ImgB	394	13.9982	3.77
chr2L	8527867	8528634	ImgA	394	13.9982	3.77
chr2L	8708483	8709699	Hnf4	195	12.3502	3.27
chr2L	8735428	8735918	raw	50	15.5832	3.65
chr2L	8988956	8989517	rost	13	15.2879	3.96
chr2L	9125610	9126003	CG32982	79	10.4114	3.15
chr2L	9176066	9176678	tai	56	17.3411	3.59

chr2L	9333414	9333944	Eaat1	78	24.5856	4.95
chr2L	9387475	9388393	Aldh	201	33.7456	6.09
chr2L	9430864	9431916	FucTB	641	10.4114	3.15
chr2L	9430864	9431916	scat	289	10.4114	3.15
chr2L	9449115	9450131	numb	57	31.1502	5.56
chr2L	9493687	9494986	Gdi	261	33.6148	5.06
chr2L	9495381	9495815	CG33298	80	16.0484	3.84
chr2L	9496108	9496770	CG33298	1018	11.9547	3.08
chr2L	9613279	9614068	Trx-2	79	38.0204	6.38
chr2L	9698615	9701190	Pka-C1	25	41.3098	5.47
chr2L	9698615	9701190	hoip	468	41.3098	5.47
chr2L	9698615	9701190	pelo	1487	41.3098	5.47
chr2L	9705667	9706138	CG31710	12	24.2448	3.98
chr2L	9710852	9711359	CG4364	62	21.8608	4.65
chr2L	9757958	9759272	zf30C	696	19.9791	3.83
chr2L	9762924	9764008	Taf11	413	27.0287	4.74
chr2L	9762924	9764008	und	141	27.0287	4.74
chr2L	9782135	9783366	IP3K1	53	16.3486	3.26
chr2L	9790386	9791407	CG4036	282	15.3751	3.41
chr2L	9890384	9891211	CG34183	161	15.7359	3.39
chr2L	9890384	9891211	CG4537	273	15.7359	3.39
chr2L	9897002	9897667	RpS2	134	35.6017	6.49
chr2L	9911988	9913365	Etl1	509	13.8173	3.24
chr2L	9911988	9913365	Srp54	207	13.8173	3.24
chr2L	9915122	9915853	yip2	123	20.2246	3.91
chr2L	9917373	9919280	CG5885	126	30.3917	4.12
chr2L	9917373	9919280	CG4598	567	30.3917	4.12
chr2L	9947156	9947737	CG5853	21	37.2231	6.30
chr2L	9956755	9958321	CG5846	513	19.5803	4.36
chr2L	9956755	9958321	CG4658	401	19.5803	4.36
chr2L	9956755	9958321	CG5846	295	19.5803	4.36
chr2L	9956755	9958321	CG4658	238	19.5803	4.36
chr2L	9963438	9963768	CG5850	193	9.85543	3.06
chr2L	9966650	9968453	Dref	218	13.2533	3.08
chr2L	9966650	9968453	RpL13	118	13.2533	3.08
chr2L	10003692	10004891	CG4747	538	20.8513	4.27
chr2L	10009276	10011055	Trp1	141	17.7161	3.62
chr2L	10200846	10201877	Ripalpha	89	22.9909	3.98
chr2L	10200846	10201877	RpL7	36	22.9909	3.98
chr2L	10220269	10222026	Npc1a	360	24.5309	4.46
chr2L	10220269	10222026	Prosalpha6	226	24.5309	4.46
chr2L	10232500	10232792	eEF1delta	145	14.1855	3.24
chr2L	10303212	10304209	nmd	63	15.1939	3.41
chr2L	10311134	10312193	l(2)SH0834	98	16.3088	3.95
chr2L	10331551	10332087	CG5037	212	14.702	4.00
chr2L	10333773	10335247	GATAd	269	17.72	3.93
chr2L	10375450	10376818	CG33303	61	14.113	3.45
chr2L	10375450	10376818	dpr19	471	14.113	3.45
chr2L	10408277	10408950	RpS27A	4	16.0484	3.84
chr2L	10408277	10408950	lp259	4	16.0484	3.84
chr2L	10430462	10431806	Bug22	8	19.0787	4.04
chr2L	10430462	10431806	TfIIB	411	19.0787	4.04
chr2L	10474136	10474435	RluA-1	90	17.1676	4.27
chr2L	10488057	10489217	CG7456	149	22.4983	4.03
chr2L	10488057	10489217	CG6144	220	22.4983	4.03
chr2L	10506516	10507595	Myo31DF	58	20.1481	3.82

chr2L	10506516	10507595	CG6094	359	20.1481	3.82
chr2L	10515965	10517656	Fatp	42	31.7926	4.69
chr2L	10515965	10517656	Lrr47	708	31.7926	4.69
chr2L	10735718	10736085	Nup107	179	11.5289	3.37
chr2L	10743376	10744051	CG6729	144	16.1486	3.72
chr2L	10756092	10756484	CG17124	76	18.2465	3.59
chr2L	10769470	10770108	CG6724	177	26.3442	5.40
chr2L	10800904	10801363	CG31869	42	11.6017	3.37
chr2L	10841701	10842638	CG6700	208	24.4817	4.31
chr2L	10849885	10851012	CG43129	399	37.7304	5.61
chr2L	10849885	10851012	porin	199	37.7304	5.61
chr2L	10856152	10856997	Dnz1	158	22.3955	4.51
chr2L	10858461	10858940	Stam	237	10.5404	3.01
chr2L	10967230	10968163	CG33129	254	38.9571	6.02
chr2L	10975881	10977531	ATPsynG	571	31.7231	5.83
chr2L	10975881	10977531	SCAR	199	31.7231	5.83
chr2L	11003659	11004277	RpL9	209	22.0143	4.08
chr2L	11004858	11005290	Nup154	224	17.5212	4.10
chr2L	11057160	11058288	Samuel	98	27.2386	5.36
chr2L	11066754	11067097	Samuel	118	17.0389	3.89
chr2L	11106065	11107685	Reps	34	30.6721	4.97
chr2L	11106065	11107685	l(2)gd1	263	30.6721	4.97
chr2L	11130627	11131588	RfC38	330	31.6553	5.25
chr2L	11130627	11131588	CG4751	239	31.6553	5.25
chr2L	11155413	11155959	CG6287	45	58.935	7.72
chr2L	11277913	11278616	CG33695	43	13.94	3.38
chr2L	11280130	11280933	Ppt2	8	19.8396	4.24
chr2L	11280130	11280933	CR44182	288	19.8396	4.24
chr2L	11317047	11317313	CR44583	1778	11.6017	3.37
chr2L	11516265	11517419	CG6509	192	30.3205	4.75
chr2L	11516265	11517419	CG4970	501	30.3205	4.75
chr2L	11807381	11808303	crol	932	25.041	4.55
chr2L	11808777	11809546	crol	70	26.1705	3.92
chr2L	11813146	11813872	CycY	381	16.8296	3.85
chr2L	11970639	11971371	CR43314	128	18.4467	4.12
chr2L	11987433	11988628	CG6686	270	16.1055	3.96
chr2L	11987433	11988628	CG34163	500	16.1055	3.96
chr2L	12002974	12007926	Rh5	622	48.1726	4.98
chr2L	12009998	12012720	CR44588	1111	25.2747	4.09
chr2L	12025360	12025678	CR44587	324	15.3164	4.09
chr2L	12038012	12038538	Tom70	51	12.8577	3.42
chr2L	12044690	12047044	CG6785	864	23.2622	4.07
chr2L	12044690	12047044	CG6770	54	23.2622	4.07
chr2L	12044690	12047044	CR44589	377	23.2622	4.07
chr2L	12048269	12048962	Plzf	109	18.8102	4.38
chr2L	12055490	12056832	JhI-21	31	37.327	6.41
chr2L	12055490	12056832	RpL7-like	407	37.327	6.41
chr2L	12055490	12056832	CG34164	429	37.327	6.41
chr2L	12055490	12056832	RpL7-like	429	37.327	6.41
chr2L	12093579	12094124	Pex19	21	16.5551	4.26
chr2L	12095946	12096899	CG6712	178	19.409	4.51
chr2L	12095946	12096899	Mt2	191	19.409	4.51
chr2L	12103058	12104411	Patsas	655	31.8157	5.16
chr2L	12103058	12104411	Aats-thr	190	31.8157	5.16
chr2L	12108527	12109217	Rab6	98	29.4241	5.10
chr2L	12421649	12422034	vir-1	23	11.2123	3.08

chr2L	12427308	12427775	CG5446	23	16.7534	4.05
chr2L	12433501	12435929	SC35	105	37.5165	5.29
chr2L	12433501	12435929	Elf	518	37.5165	5.29
chr2L	12433501	12435929	SC35	732	37.5165	5.29
chr2L	12433501	12435929	Elf	958	37.5165	5.29
chr2L	12436316	12436822	Elf	93	15.2975	3.84
chr2L	12529469	12529898	bun	11194	12.1432	3.35
chr2L	12692090	12693163	CG5525	330	33.4714	5.21
chr2L	12692090	12693163	CG6153	388	33.4714	5.21
chr2L	12704089	12705150	CG5776	257	14.9226	3.23
chr2L	12704089	12705150	spict	16	14.9226	3.23
chr2L	12718864	12719349	MRP	31	32.3973	4.98
chr2L	12721165	12722648	Pih1D1	125	17.5402	3.63
chr2L	12721165	12722648	MRP	554	17.5402	3.63
chr2L	12974112	12975574	Vha68-2	62	20.0928	3.73
chr2L	13004465	13004955	Tor	175	27.9529	4.73
chr2L	13018252	13019300	CG9934	13	13.233	3.32
chr2L	13020567	13021509	A16	16	12.7404	3.77
chr2L	13059813	13060465	CG9932	43	22.9909	3.98
chr2L	13165408	13166671	Sirt1	421	30.6156	4.57
chr2L	13165408	13166671	DnaJ-H	88	30.6156	4.57
chr2L	13176212	13177743	Sfmbt	413	29.9359	5.26
chr2L	13176212	13177743	CG43925	373	29.9359	5.26
chr2L	13176212	13177743	Sfmbt	10	29.9359	5.26
chr2L	13176212	13177743	CG5439	354	29.9359	5.26
chr2L	13179161	13179668	CG5287	218	20.4553	4.14
chr2L	13191594	13192601	CG16974	235	9.6957	3.03
chr2L	13191594	13192601	Edem2	14	9.6957	3.03
chr2L	13203708	13204711	Pect	231	29.5482	4.78
chr2L	13215742	13216139	snRNA:U2:34ABb	118	38.6289	5.19
chr2L	13215742	13216139	snRNA:U2:34ABc	118	38.6289	5.19
chr2L	13219169	13220390	CG9426	35	25.9709	4.55
chr2L	13219169	13220390	CG5705	90	25.9709	4.55
chr2L	13285950	13287259	Drep4	532	15.2967	3.79
chr2L	13285950	13287259	Uvrag	218	15.2967	3.79
chr2L	13289931	13291445	CG31729	6	32.0617	4.64
chr2L	13373106	13374672	CG9305	147	46.134	6.34
chr2L	13373106	13374672	CG6565	341	46.134	6.34
chr2L	13378198	13379335	CG9302	374	17.3643	3.64
chr2L	13378198	13379335	beta'COP	11	17.3643	3.64
chr2L	13394321	13395275	RpL24	97	26.0908	5.05
chr2L	13512760	13513778	CG33640	14494	20.6511	3.99
chr2L	13548604	13549632	B4	86	34.1336	5.04
chr2L	13550082	13550836	kuz	159	15.0394	3.12
chr2L	13665427	13666258	CG18507	148	15.9181	3.39
chr2L	13779844	13781110	CG9008	49	83.3352	9.96
chr2L	13779844	13781110	p38b	572	83.3352	9.96
chr2L	13793429	13793983	Sec71	165	34.2783	5.73
chr2L	13803105	13803864	CAH1	20	13.0413	3.50
chr2L	13813710	13814441	Sos	392	20.3428	4.23
chr2L	13820898	13821395	b	33	9.59119	3.23
chr2L	13832672	13833842	CG33649	77	14.8491	3.35
chr2L	13832672	13833842	DNApol-gamma35	77	14.8491	3.35
chr2L	13832672	13833842	RpII33	308	14.8491	3.35
chr2L	13903864	13904774	Smg5	80	30.6156	4.57
chr2L	14110354	14110723	CG31769	200	9.68089	3.37

chr2L	14233872	14234287	Dyrk2	43	18.3864	3.86
chr2L	14348243	14348909	mTTF	47	21.1978	4.57
chr2L	14354278	14355681	l(2)34Fd	111	39.3626	5.63
chr2L	14354278	14355681	Rab14	52	39.3626	5.63
chr2L	14362011	14362928	spel1	100	20.1429	4.61
chr2L	14614722	14615945	Adhr	8	13.5752	3.48
chr2L	14614722	14615945	Adh	8	13.5752	3.48
chr2L	14689039	14689402	osp	92	19.6821	3.60
chr2L	15007839	15008380	NC2beta	218	18.4788	3.97
chr2L	15009314	15010359	l(2)35Bc	393	12.2069	3.20
chr2L	15009314	15010359	l(2)35Bd	179	12.2069	3.20
chr2L	15037950	15038874	CIAPIN1	173	15.9328	3.63
chr2L	15039341	15039834	Su(H)	126	21.0887	4.06
chr2L	15057022	15057994	ck	30	12.7728	3.03
chr2L	15059821	15061056	TfIIS	194	23.5836	4.68
chr2L	15271228	15272130	Cul3	161	35.5264	4.86
chr2L	15498889	15499805	lace	166	27.1785	4.50
chr2L	15747250	15748015	CR44866	476	29.4241	4.82
chr2L	15750577	15751211	ND-B17	199	39.1036	5.84
chr2L	15752213	15752824	l(2)35Df	236	16.4711	3.93
chr2L	15762385	15762831	Gli	91	20.2994	4.16
chr2L	16049667	16050049	beat-Ia	188	10.7856	3.56
chr2L	16257209	16258423	PRL-1	157	15.0109	3.50
chr2L	16257209	16258423	EndoGI	238	15.0109	3.50
chr2L	16272844	16273826	CG4935	11543	10.6984	3.21
chr2L	16286602	16288900	crp	112	53.6466	6.06
chr2L	16286602	16288900	pkaap	671	53.6466	6.06
chr2L	16299144	16300517	heix	396	26.4398	5.05
chr2L	16299144	16300517	CG17328	480	26.4398	5.05
chr2L	16324685	16326291	cact	52	22.2338	3.95
chr2L	16328571	16329262	mRpL4	184	9.54246	3.07
chr2L	16350675	16353352	CG42231	331	12.9588	3.03
chr2L	16350675	16353352	chif	331	12.9588	3.03
chr2L	16350675	16353352	CG4455	636	12.9588	3.03
chr2L	16356248	16356896	CaBP1	53	20.8903	4.71
chr2L	16450849	16451333	Idgf3	49	22.3256	4.28
chr2L	16485689	16486905	dac	492	23.5522	4.16
chr2L	16521147	16521904	CG5953	1467	11.7447	3.41
chr2L	16532596	16533703	CG5953	12	21.6997	3.72
chr2L	16601640	16602120	CG42389	28	36.3471	5.44
chr2L	16715639	16716242	Cyt-c-d	72	15.9106	3.62
chr2L	16715639	16716242	CG31808	163	15.9106	3.62
chr2L	16719602	16721257	Cyt-c-p	26	17.2519	3.43
chr2L	16732568	16733098	CG17904	130	13.0517	3.33
chr2L	16742706	16743928	LSm7	459	24.5074	4.68
chr2L	16742706	16743928	BuGZ	155	24.5074	4.68
chr2L	16791448	16791993	CG17928	71	15.7576	3.64
chr2L	16816491	16816875	mdy	26	29.3431	5.03
chr2L	16824827	16825298	Cas	111	20.2104	3.51
chr2L	16826969	16827810	CG31739	81	24.6971	4.90
chr2L	16881062	16881709	CG31743	25	22.9612	4.39
chr2L	16887376	16889196	CG6115	106	33.3852	4.74
chr2L	16887376	16889196	tweek	629	33.3852	4.74
chr2L	16887376	16889196	CR44392	646	33.3852	4.74
chr2L	17383666	17384235	Lrch	63	16.6352	3.59
chr2L	17427397	17428409	Dif	33	15.7273	3.47

chr2L	17477259	17477997	Aac11	63	13.933	3.46
chr2L	17963196	17963638	CG5674	27	19.6406	3.86
chr2L	18137559	18138169	kel	165	11.2866	3.21
chr2L	18151215	18152731	Socs36E	51	25.0413	4.26
chr2L	18442511	18443430	RpS26	62	27.4214	4.32
chr2L	18448479	18449008	ncm	241	31.3024	5.45
chr2L	18454223	18454778	bsf	84	17.1899	3.65
chr2L	18454223	18454778	Ntf-2r	145	17.1899	3.65
chr2L	18455560	18456456	let-7-C	21	17.9766	3.79
chr2L	18483872	18484615	CG10283	32	53.4167	6.48
chr2L	18572077	18572397	Pde11	50	13.4499	3.25
chr2L	18590688	18591546	CG15160	38	28.0496	5.38
chr2L	18604414	18605230	CG10413	135	28.7495	4.76
chr2L	18611875	18612240	Atac2	195	16.6352	3.59
chr2L	18677054	18677668	CG31751	196	22.9612	4.39
chr2L	18698598	18699477	CG10341	121	45.0318	6.51
chr2L	18702698	18704053	CG10376	173	13.933	3.46
chr2L	18702698	18704053	CG10343	540	13.933	3.46
chr2L	18705395	18705951	Jwa	117	9.17445	3.12
chr2L	18707759	18709132	Faf2	186	31.9271	4.66
chr2L	18707759	18709132	Grip71	432	31.9271	4.66
chr2L	18732417	18733259	CR44768	449	14.3258	3.63
chr2L	18732417	18733259	CG10348	388	14.3258	3.63
chr2L	18808233	18809967	CG42502	1198	28.1561	5.56
chr2L	18808233	18809967	CG10570	1198	28.1561	5.56
chr2L	18808233	18809967	CG42502	1198	28.1561	5.56
chr2L	18810480	18811330	CG42502	26	16.5868	4.20
chr2L	18810480	18811330	CG10570	26	16.5868	4.20
chr2L	18810480	18811330	CG42502	26	16.5868	4.20
chr2L	18810480	18811330	CG10570	240	16.5868	4.20
chr2L	18838698	18839854	CG10600	270	38.7204	5.97
chr2L	18858766	18859392	CG10602	196	28.5004	4.82
chr2L	18858766	18859392	mRpL13	196	28.5004	4.82
chr2L	18881084	18881797	tup	223	42.8757	6.42
chr2L	18968208	18969356	Nak	144	17.532	3.41
chr2L	18968208	18969356	Tango6	450	17.532	3.41
chr2L	18986993	18987947	Swip-1	51	25.041	4.55
chr2L	18986993	18987947	CG15168	477	25.041	4.55
chr2L	19008795	19009473	RpL30	116	43.4925	5.28
chr2L	19034366	19035301	mib2	422	25.5629	4.49
chr2L	19034366	19035301	CG31800	289	25.5629	4.49
chr2L	19034366	19035301	mib2	225	25.5629	4.49
chr2L	19051830	19052411	Rpn3	229	21.4678	4.50
chr2L	19054046	19055023	CG10492	406	57.6078	7.19
chr2L	19131356	19132037	Aats-asn	199	19.218	4.17
chr2L	19132387	19134497	l(2)37Cg	439	15.1713	3.49
chr2L	19132387	19134497	brat	107	15.1713	3.49
chr2L	19158119	19158986	brat	66	18.1434	3.29
chr2L	19190221	19190674	drl	73	19.218	4.17
chr2L	19362665	19363231	dnt	249	13.7537	3.47
chr2L	19398788	19399243	CG17544	538	50.1838	6.79
chr2L	19444096	19445239	Hs2st	137	17.8796	3.53
chr2L	19444096	19445239	RanGAP	160	17.8796	3.53
chr2L	19492382	19493940	CG10189	537	11.4702	3.02
chr2L	19492382	19493940	swm	250	11.4702	3.02
chr2L	19492382	19493940	CG10189	247	11.4702	3.02

chr2L	19492382	19493940	swm	67	11.4702	3.02
chr2L	19508148	19509095	CG10188	248	22.4738	4.31
chr2L	19529817	19530339	CG10165	215	18.8647	4.53
chr2L	19549369	19549971	Tep4	57	17.6365	3.81
chr2L	19571790	19572747	spi	77	46.1145	5.58
chr2L	19575156	19577393	spi	175	16.2832	3.61
chr2L	19582013	19582558	Hakai	181	35.8458	4.99
chr2L	19583240	19584811	CG10262	103	13.6415	3.54
chr2L	19583240	19584811	CG10366	347	13.6415	3.54
chr2L	19586570	19587675	Lar	152	13.4393	3.08
chr2L	19756063	19757010	CG10631	59	14.7396	3.08
chr2L	19791477	19792466	CdGAPr	58	23.9142	4.00
chr2L	19791477	19792466	CG10466	279	23.9142	4.00
chr2L	19935107	19935745	sick	94	13.9265	3.60
chr2L	19958727	19959528	COX4	114	23.9828	4.89
chr2L	20066155	20067085	bwa	22	28.4819	4.61
chr2L	20074900	20076049	pr	243	13.8675	3.18
chr2L	20074900	20076049	neb	541	13.8675	3.18
chr2L	20084216	20085719	fok	31	19.3273	3.61
chr2L	20090841	20091939	CG10747	362	12.2143	3.02
chr2L	20090841	20091939	CG10721	99	12.2143	3.02
chr2L	20349080	20350861	La	233	39.3626	6.65
chr2L	20349080	20350861	RtGEF	654	39.3626	6.65
chr2L	20416378	20417782	CG10949	305	48.7397	6.05
chr2L	20416378	20417782	Arpc2	134	48.7397	6.05
chr2L	20655594	20656817	bru	236	28.1646	5.26
chr2L	20655594	20656817	CG2614	483	28.1646	5.26
chr2L	20720092	20720639	CR45306	1666	19.565	3.98
chr2L	20757274	20758937	CG9323	218	23.1868	4.55
chr2L	20757274	20758937	dia	482	23.1868	4.55
chr2L	20770343	20770725	cad	134	12.1669	3.27
chr2L	20787496	20788169	Pomp	162	24.577	3.95
chr2L	20793596	20794262	vari	29	12.0756	3.13
chr2L	20823723	20824114	CG9331	368	15.9094	3.81
chr2L	20917184	20917621	sky	13	12.7437	3.54
chr2L	21051206	21052206	CG42238	33	24.0111	4.23
chr2L	21143149	21143762	CG9253	115	38.9204	6.08
chr2L	21162605	21163172	Nbr	77	26.1078	4.53
chr2L	21163520	21164181	CG9246	90	13.7586	3.38
chr2L	21168344	21169485	CG43345	344	19.5999	3.94
chr2L	21168344	21169485	CG43346	344	19.5999	3.94
chr2L	21168344	21169485	Acon	124	19.5999	3.94
chr2L	21168344	21169485	CG43346	112	19.5999	3.94
chr2L	21168344	21169485	CG43345	112	19.5999	3.94
chr2L	21168344	21169485	Acon	43	19.5999	3.94
chr2L	21178504	21179744	bur	128	19.7049	3.67
chr2L	21219636	21220686	Atg18b	110	11.0265	3.08
chr2L	21237087	21238276	Hr39	128	23.3233	3.96
chr2L	21261008	21261537	l(2)k14505	244	11.124	3.53
chr2L	21308788	21310267	Mondo	160	48.5632	6.97
chr2L	21310751	21311417	crc	88	29.4241	4.82
chr2L	21629167	21630105	Df31	39	48.8395	5.95
chr2L	21681275	21682251	EF2	4	16.9985	3.83
chr2L	21757017	21757586	step	119	12.7602	3.19
chr2L	21828397	21829108	tsh	112	14.2928	3.44
chr2L	22121758	22122298	CG3651	196	34.3815	5.18

chr2L	22247224	22247815	RpL21	253	18.2193	3.88
chr2L	22535850	22536462	RpL5	440	8.86652	3.04
chr2R	870235	870529	CG40439	15553	31.9031	3.53
chr2R	5608573	5609326	CG17508	83	18.6029	4.10
chr2R	5754501	5755033	l(2)09851	188	22.3955	4.51
chr2R	5760809	5761225	Gp210	127	21.4885	4.77
chr2R	6017550	6018317	Pngl	122	18.0567	4.41
chr2R	6035259	6036704	SCAP	23	18.9749	3.83
chr2R	6075669	6077354	CG7845	77	28.222	5.27
chr2R	6075669	6077354	CG7849	619	28.222	5.27
chr2R	6084864	6085499	Ars2	220	11.118	3.06
chr2R	6132915	6133903	EcR	1265	22.3765	3.62
chr2R	6173564	6173989	Cyp6w1	38	12.3786	3.67
chr2R	6213715	6214821	l(2)k14710	520	15.5781	3.33
chr2R	6213715	6214821	bin3	75	15.5781	3.33
chr2R	6501539	6502362	CR46075	246	20.4891	3.79
chr2R	6501539	6502362	jing	13	20.4891	3.79
chr2R	6631360	6633288	Vha16-1	185	48.7877	6.00
chr2R	6635692	6637111	Trap1	478	41.9091	6.11
chr2R	6635692	6637111	Bap170	229	41.9091	6.11
chr2R	6655851	6656463	geminin	67	10.8054	3.50
chr2R	6661228	6663906	Dpit47	964	29.6618	4.80
chr2R	6661228	6663906	Adf1	813	29.6618	4.80
chr2R	6672820	6673794	Hsepi	244	13.4155	3.32
chr2R	6672820	6673794	PGAP3	13	13.4155	3.32
chr2R	6741721	6742738	phtf	293	20.1634	4.04
chr2R	6749096	6749729	Eb1	107	16.6246	3.51
chr2R	6807581	6808187	SdhB	213	17.6909	3.92
chr2R	6810906	6811952	koi	44	23.1018	4.08
chr2R	6871525	6873292	coro	99	43.0133	5.46
chr2R	6871525	6873292	CR44169	529	43.0133	5.46
chr2R	6878343	6878997	Spn42Da	43	17.5225	3.55
chr2R	6985255	6985722	CG30156	128	15.3956	3.34
chr2R	6985255	6985722	vimar	128	15.3956	3.34
chr2R	6986021	6986475	CG17002	228	13.8233	3.40
chr2R	6992505	6993267	Tsp42Ea	67	19.2393	4.02
chr2R	6992505	6993267	CG30159	62	19.2393	4.02
chr2R	6992505	6993267	Tsp42Ea	62	19.2393	4.02
chr2R	6992505	6993267	CG30159	62	19.2393	4.02
chr2R	6992505	6993267	Tsp42Ea	62	19.2393	4.02
chr2R	6992505	6993267	CG30159	177	19.2393	4.02
chr2R	7012095	7013246	Tsp42Ee	81	17.3508	3.41
chr2R	7024850	7025516	Tsp42Ef	20	32.3734	5.32
chr2R	7044959	7046122	Tsp42El	135	12.1669	3.27
chr2R	7150682	7151027	pk	59	12.0224	3.53
chr2R	7157860	7158333	nec	92	13.0517	3.33
chr2R	7277666	7278319	Br140	57	31.425	4.93
chr2R	7352212	7352800	CR44071	9078	29.8606	5.00
chr2R	7381516	7382094	Dscam1	58	24.9643	4.39
chr2R	7386648	7388062	cos	350	14.9226	3.23
chr2R	7386648	7388062	Dhx15	142	14.9226	3.23
chr2R	7395584	7396051	Glo1	81	22.6738	4.45
chr2R	7406677	7407951	mir-4977	385	17.678	4.08
chr2R	7406677	7407951	Eaf	597	17.678	4.08
chr2R	7419170	7419576	so	36	12.9822	3.71
chr2R	7458434	7459045	Inos	47	26.2299	4.71

chr2R	7465971	7466953	p47	243	21.7465	3.80
chr2R	7473721	7474756	Aldh-III	67	20.3191	3.81
chr2R	7491964	7493472	Coop	55	29.752	4.98
chr2R	7491964	7493472	CR43723	106	29.752	4.98
chr2R	7491964	7493472	Coop	332	29.752	4.98
chr2R	7495898	7496417	CG1620	196	14.5117	3.29
chr2R	7528977	7529840	Drat	13	16.5842	3.80
chr2R	7533239	7534433	CR44209	105	29.792	4.63
chr2R	7533239	7534433	Cyt-b5	97	29.792	4.63
chr2R	7539080	7540003	CG1598	112	22.227	4.48
chr2R	7565044	7567467	CG30499	1104	52.3598	6.52
chr2R	7565044	7567467	boca	117	52.3598	6.52
chr2R	7565044	7567467	Vps13	47	52.3598	6.52
chr2R	7632422	7633346	CG43341	89	12.2143	3.02
chr2R	7636777	7637158	CG43340	39	14.6489	3.81
chr2R	7668310	7669021	DCTN4-p62	196	15.1939	3.41
chr2R	7673966	7674923	mRpL52	214	14.6359	3.56
chr2R	7673966	7674923	CG12107	263	14.6359	3.56
chr2R	7699175	7699955	CG30493	54	14.455	3.73
chr2R	7699175	7699955	CG30496	193	14.455	3.73
chr2R	7735010	7735743	CG18812	303	20.6778	4.47
chr2R	7793575	7793907	mus205	145	14.85	3.60
chr2R	7805201	7807291	Nop17l	95	26.2481	4.50
chr2R	7805201	7807291	sax	822	26.2481	4.50
chr2R	7822973	7823634	cathD	187	35.0342	5.80
chr2R	7938657	7939544	CG8728	51	19.8396	4.24
chr2R	7955055	7956121	CG14764	103	23.2132	4.80
chr2R	7962430	7963155	nito	161	23.7056	4.27
chr2R	7971114	7972077	CG8726	72	18.4659	4.30
chr2R	7972485	7972977	CSN4	58	14.9618	3.82
chr2R	7983307	7984484	ACC	54	23.157	3.97
chr2R	8001633	8002662	CG11191	95	14.4324	4.07
chr2R	8018728	8019285	CR45267	198	19.0787	4.04
chr2R	8030627	8031683	Optix	331	14.6897	3.88
chr2R	8067881	8069468	lig	68	25.2747	4.09
chr2R	8081811	8083557	slv	219	21.0719	4.21
chr2R	8081811	8083557	sut1	974	21.0719	4.21
chr2R	8081811	8083557	slv	1074	21.0719	4.21
chr2R	8098713	8099153	CG11210	318	11.157	3.03
chr2R	8122471	8123473	Asap	177	18.0528	3.89
chr2R	8122471	8123473	Nup50	211	18.0528	3.89
chr2R	8127502	8128086	Socs44A	101	11.9082	3.40
chr2R	8131271	8132613	Pbp49	783	34.1874	5.45
chr2R	8131271	8132613	CG42516	783	34.1874	5.45
chr2R	8131271	8132613	Pbp49	783	34.1874	5.45
chr2R	8131271	8132613	Pabp2	676	34.1874	5.45
chr2R	8145720	8146460	Lpin	50	24.478	4.17
chr2R	8163067	8163658	RagC-D	127	17.4043	3.73
chr2R	8163067	8163658	Rs1	190	17.4043	3.73
chr2R	8570521	8570791	Cyp6a13	130	11.4281	3.49
chr2R	8599307	8600055	CG8735	72	16.2837	3.84
chr2R	8600329	8600682	rgr	87	20.0567	3.65
chr2R	8614555	8615311	Cirl	192	15.03	3.35
chr2R	8696309	8697201	CG8738	50	12.5105	3.54
chr2R	8723214	8725011	CG14767	527	31.5738	5.50
chr2R	8723214	8725011	gcl	109	31.5738	5.50

chr2R	8723214	8725011	snoRNA:gcl-a	538	31.5738	5.50
chr2R	8732820	8734382	stmA	113	21.0887	4.06
chr2R	8735340	8736314	CG8740	83	23.1448	4.72
chr2R	8899898	8901942	ana2	1143	24.1873	4.09
chr2R	8899898	8901942	CG13751	1114	24.1873	4.09
chr2R	8899898	8901942	MrgBP	21	24.1873	4.09
chr2R	8899898	8901942	Ggamma1	49	24.1873	4.09
chr2R	8906495	8907076	CG8258	171	14.653	3.51
chr2R	8907404	8907915	CG30349	127	17.1899	3.65
chr2R	8914440	8915182	lin	293	38.5999	6.50
chr2R	8922402	8923141	Spt	137	14.24	3.72
chr2R	8922402	8923141	CG8243	166	14.24	3.72
chr2R	8927732	8928737	CG8235	29	25.3185	4.50
chr2R	8927732	8928737	PAN2	92	25.3185	4.50
chr2R	8946897	8947511	CG8230	57	23.4625	4.48
chr2R	8950323	8952795	CG8229	111	20.4414	3.71
chr2R	8950323	8952795	CG33199	111	20.4414	3.71
chr2R	8950323	8952795	CG8229	111	20.4414	3.71
chr2R	8950323	8952795	CG33199	111	20.4414	3.71
chr2R	8950323	8952795	Tom7	619	20.4414	3.71
chr2R	8950323	8952795	babo	1363	20.4414	3.71
chr2R	9087818	9088392	Dbp45A	209	26.3075	4.96
chr2R	9092605	9093281	CG8080	9	12.8698	3.33
chr2R	9095076	9095857	Uhg4	106	27.7658	3.90
chr2R	9095076	9095857	snoRNA:Psi28S-2949	7	27.7658	3.90
chr2R	9095076	9095857	snoRNA:Or-aca5	306	27.7658	3.90
chr2R	9110693	9111281	tsu	173	53.0031	7.71
chr2R	9113586	9114875	Mys45A	90	22.3397	4.75
chr2R	9113586	9114875	Phax	412	22.3397	4.75
chr2R	9115721	9117493	Su(var)2-10	239	23.0792	4.36
chr2R	9131111	9131942	CG18659	190	20.5859	3.63
chr2R	9132335	9133206	Hydr1	16	15.2023	3.58
chr2R	9136682	9138101	alc	91	55.8552	6.41
chr2R	9148540	9149155	CG30345	33	23.0834	4.22
chr2R	9176839	9177469	Rme-8	169	27.2964	5.16
chr2R	9185528	9186579	Rab32	41	29.6845	4.56
chr2R	9191246	9192191	CG8026	79	54.4604	6.06
chr2R	9191246	9192191	CG45085	79	54.4604	6.06
chr2R	9191246	9192191	CG8026	79	54.4604	6.06
chr2R	9206671	9208141	CG8027	502	20.4089	3.64
chr2R	9206671	9208141	VhaAC45	66	20.4089	3.64
chr2R	9239907	9240292	Cyp4p1	20	11.3973	3.08
chr2R	9286271	9287116	CG2063	256	14.7199	3.72
chr2R	9286271	9287116	CG1968	64	14.7199	3.72
chr2R	9308141	9308892	Myd88	99	17.3503	3.63
chr2R	9398186	9399434	Non1	214	25.8329	4.58
chr2R	9398186	9399434	l(2)k10201	559	25.8329	4.58
chr2R	9408979	9409789	wun	67	38.6211	5.35
chr2R	9423961	9424700	prel	38	23.1698	5.08
chr2R	9426322	9427164	Pdk	292	16.6246	3.51
chr2R	9440614	9441734	ced-6	81	20.5596	3.89
chr2R	9445824	9446739	Camta	147	17.1625	3.60
chr2R	9552693	9554923	CR43651	59	19.5315	3.68
chr2R	9552693	9554923	mir-14	859	19.5315	3.68
chr2R	9577292	9579140	CG1814	200	32.0708	5.15
chr2R	9577292	9579140	Not1	384	32.0708	5.15

chr2R	9577292	9579140	CR46257	1278	32.0708	5.15
chr2R	9577292	9579140	Not1	1381	32.0708	5.15
chr2R	9579424	9580185	CG1814	288	37.592	5.71
chr2R	9584497	9585265	CG1868	153	13.1096	3.57
chr2R	9584497	9585265	Updo	56	13.1096	3.57
chr2R	9586163	9586759	CG12929	71	13.3314	3.67
chr2R	9587090	9587618	RpL31	7	15.676	3.87
chr2R	9593919	9594545	CG1902	190	27.9181	5.01
chr2R	9593919	9594545	CG30338	190	27.9181	5.01
chr2R	9606005	9606587	GstT1	322	10.4185	3.24
chr2R	9691913	9693490	Uba1	643	40.1528	4.67
chr2R	9712121	9712861	dap	132	16.989	3.57
chr2R	9835915	9837244	hebe	22	19.7164	4.39
chr2R	9835915	9837244	CG1663	364	19.7164	4.39
chr2R	9839418	9839862	Lsm11	15	11.0265	3.08
chr2R	9839418	9839862	CG1665	260	11.0265	3.08
chr2R	9891195	9891789	CG1513	262	13.0467	3.28
chr2R	9957969	9959036	Mef2	184	27.793	4.65
chr2R	9959409	9960945	Pal1	517	13.4499	3.25
chr2R	9959409	9960945	CG15863	333	13.4499	3.25
chr2R	9959409	9960945	Pal1	12	13.4499	3.25
chr2R	9968973	9969740	Adam	213	28.0815	4.70
chr2R	9988948	9989628	TER94	42	18.4231	3.44
chr2R	9988948	9989628	CR45324	85	18.4231	3.44
chr2R	9988948	9989628	TER94	115	18.4231	3.44
chr2R	10025211	10027478	Pka-R2	127	31.547	4.57
chr2R	10025211	10027478	CR44294	843	31.547	4.57
chr2R	10025211	10027478	CG12128	923	31.547	4.57
chr2R	10032346	10033031	CG1407	82	17.4043	3.73
chr2R	10047994	10048807	COX7C	226	30.3476	4.81
chr2R	10047994	10048807	oys	41	30.3476	4.81
chr2R	10049412	10050151	COX7C	181	22.4738	4.31
chr2R	10051608	10052845	sel	441	21.8115	4.57
chr2R	10051608	10052845	magu	68	21.8115	4.57
chr2R	10096790	10098009	Jra	164	26.3346	4.20
chr2R	10098869	10102314	RpLP0-like	327	40.8609	5.31
chr2R	10098869	10102314	14-3-3zeta	241	40.8609	5.31
chr2R	10110388	10111769	Pfk	141	38.6211	5.57
chr2R	10123831	10125436	gem	50	30.7863	4.99
chr2R	10142073	10142780	CR43467	308	13.3967	3.49
chr2R	10142073	10142780	CG18011	45	13.3967	3.49
chr2R	10157999	10158818	mlt	92	33.3728	5.56
chr2R	10159478	10160372	mlt	905	24.9184	4.43
chr2R	10251760	10252517	Syb	106	27.6043	5.15
chr2R	10309337	10310819	Jhl-1	416	20.3588	3.98
chr2R	10309337	10310819	CG12909	98	20.3588	3.98
chr2R	10427885	10428681	CG12896	498	14.3584	3.46
chr2R	10474767	10475761	whd	74	49.5565	6.05
chr2R	10534635	10535296	lola	56	22.9272	4.10
chr2R	10541452	10542555	CR45135	47	15.4794	3.68
chr2R	10541452	10542555	lola	29	15.4794	3.68
chr2R	10543112	10543587	lola	79	14.4914	3.89
chr2R	10617459	10618737	CG11883	41	29.5482	4.78
chr2R	10644980	10646144	Git	231	13.0829	3.25
chr2R	10644980	10646144	Elp2	76	13.0829	3.25
chr2R	10808276	10809096	wde	33	17.0617	3.75

chr2R	10809437	10811286	CG12935	1051	24.3368	4.59
chr2R	10809437	10811286	CG7637	207	24.3368	4.59
chr2R	10809437	10811286	mms4	348	24.3368	4.59
chr2R	10822585	10824003	CG12343	134	16.4459	3.52
chr2R	10822585	10824003	CG12325	166	16.4459	3.52
chr2R	10838378	10838906	CG7686	27	20.2723	4.57
chr2R	10873963	10875092	CG30015	74	43.0761	5.97
chr2R	10875787	10876481	nclb	278	17.8858	3.91
chr2R	10879116	10880262	Taf5	215	17.7005	3.47
chr2R	10879116	10880262	Pex6	515	17.7005	3.47
chr2R	10898543	10899221	Rpb5	289	15.4097	3.66
chr2R	11105673	11106993	luna	35	17.2315	3.98
chr2R	11173478	11174033	shn	19	23.2628	4.15
chr2R	11218972	11220476	CG9084	57	15.2101	4.43
chr2R	11218972	11220476	Syx6	354	15.2101	4.43
chr2R	11218972	11220476	CG9084	587	15.2101	4.43
chr2R	11218972	11220476	Syx6	749	15.2101	4.43
chr2R	11222765	11223150	CG7737	56	10.9184	3.22
chr2R	11279367	11280186	CG9067	113	20.7679	4.25
chr2R	11279367	11280186	Vhl	72	20.7679	4.25
chr2R	11283526	11284864	CG9062	110	24.3459	4.54
chr2R	11283526	11284864	CG13220	421	24.3459	4.54
chr2R	11289041	11290007	Fbl6	509	25.0015	4.01
chr2R	11306923	11308464	Fpps	108	38.0486	6.22
chr2R	11306923	11308464	CG7745	458	38.0486	6.22
chr2R	11358737	11359247	CG12384	124	22.6239	4.14
chr2R	11359792	11360209	ix	169	17.1334	3.86
chr2R	11366273	11366822	CG13204	110	30.4094	5.49
chr2R	11397266	11397556	sprt	41	8.46252	3.18
chr2R	11451790	11453421	E(Pc)	51	26.492	4.64
chr2R	11604430	11605359	tou	127	15.3692	3.48
chr2R	11631639	11632853	CG9005	52	29.9359	4.70
chr2R	11852549	11853109	Mtor	176	12.3502	3.27
chr2R	11885149	11885884	CG13185	65	57.7513	8.15
chr2R	11890237	11891102	ERp60	215	18.098	3.68
chr2R	11891937	11893567	Ef1alpha48D	85	40.9057	5.04
chr2R	11891937	11893567	snoRNA:Me28S-A1322	916	40.9057	5.04
chr2R	11891937	11893567	Ef1alpha48D	1335	40.9057	5.04
chr2R	11899432	11900040	Zip48C	104	23.7089	4.14
chr2R	11903590	11903987	MCPH1	150	10.7353	3.22
chr2R	12027346	12028965	Mppe	456	37.2866	5.60
chr2R	12027346	12028965	pds5	179	37.2866	5.60
chr2R	12033851	12034869	CG8321	56	22.3101	4.18
chr2R	12037215	12037908	128up	100	15.8437	3.85
chr2R	12044520	12044945	Drep1	110	14.4835	3.12
chr2R	12145666	12146061	Hen1	167	22.5331	4.72
chr2R	12150237	12151188	CG8878	210	25.041	4.55
chr2R	12162437	12163148	Prp8	64	20.9293	4.08
chr2R	12170333	12171737	mir-281-1	710	25.9379	4.77
chr2R	12170333	12171737	Oda	113	25.9379	4.77
chr2R	12170333	12171737	CR46194	140	25.9379	4.77
chr2R	12172698	12174433	Oda	49	22.2338	3.95
chr2R	12200027	12200955	RpS11	209	36.5996	6.10
chr2R	12259113	12259692	Cam	55	18.8931	3.82
chr2R	12259113	12259692	CR43900	44	18.8931	3.82
chr2R	12325665	12326859	CG8841	106	21.8188	4.24

chr2R	12325665	12326859	garz	5	21.8188	4.24
chr2R	12337675	12338577	Den1	80	22.3401	4.62
chr2R	12343791	12345000	CG8839	146	27.0287	4.74
chr2R	12343791	12345000	ana3	511	27.0287	4.74
chr2R	12423489	12423971	CG8520	158	12.5329	3.26
chr2R	12430635	12431075	Nup54	323	8.98345	3.07
chr2R	12435193	12438259	DUBAI	1594	25.1125	4.10
chr2R	12435193	12438259	CG30051	1311	25.1125	4.10
chr2R	12435193	12438259	DUBAI	853	25.1125	4.10
chr2R	12435193	12438259	Dyb	86	25.1125	4.10
chr2R	12468567	12469117	CG8545	125	23.603	4.46
chr2R	12474105	12474519	Dh44-R2	132	20.9293	4.08
chr2R	12518563	12519534	CG8818	259	10.1185	3.12
chr2R	12518563	12519534	CG8569	6	10.1185	3.12
chr2R	12528711	12529514	fra	55	22.4665	4.16
chr2R	12572040	12572459	ClC-b	135	13.4096	3.17
chr2R	12586702	12590387	Sin3A	96	26.0154	4.24
chr2R	12586702	12590387	CR30055	10	26.0154	4.24
chr2R	12591122	12593008	Sin3A	1114	14.4835	3.36
chr2R	12612459	12613497	CG45086	409	18.0897	3.85
chr2R	12612459	12613497	Galphaq	409	18.0897	3.85
chr2R	12635723	12637259	CG33672	740	19.4731	3.87
chr2R	12635723	12637259	CG33671	740	19.4731	3.87
chr2R	12635723	12637259	Iswi	211	19.4731	3.87
chr2R	12692395	12692761	CG8768	76	8.08118	3.07
chr2R	12705000	12705416	CG42663	63	20.2554	4.38
chr2R	12755854	12758159	Taz	1377	28.6233	4.79
chr2R	12755854	12758159	mRpL18	1358	28.6233	4.79
chr2R	12755854	12758159	ox	262	28.6233	4.79
chr2R	12755854	12758159	Dgkepsilon	162	28.6233	4.79
chr2R	12760217	12760969	Nacalpha	1	21.8608	4.65
chr2R	12870107	12870993	bic	185	40.3316	5.55
chr2R	12880134	12880682	Nmda1	148	18.0261	4.27
chr2R	12880134	12880682	Aats-asp	53	18.0261	4.27
chr2R	12915989	12917087	NAT1	512	14.3293	3.30
chr2R	12935962	12936957	CG13322	59	20.5596	3.89
chr2R	12935962	12936957	Sans	414	20.5596	3.89
chr2R	12980194	12981171	Psc	72	34.8511	4.64
chr2R	12990761	12991365	CG33798	2863	15.5555	3.40
chr2R	12996143	12997419	Su(z)2	5	21.2917	3.48
chr2R	13133128	13133496	Cap-G	188	10.8909	3.21
chr2R	13152146	13152705	Ack-like	297	18.0222	4.09
chr2R	13158695	13159102	ND-MWFE	211	12.5329	3.26
chr2R	13170999	13172078	AGBE	48	27.289	4.10
chr2R	13198830	13199299	Aats-val	90	10.0428	3.15
chr2R	13221858	13223917	Fsn	64	28.6173	5.08
chr2R	13221858	13223917	Dp	30	28.6173	5.08
chr2R	13221858	13223917	Fsn	57	28.6173	5.08
chr2R	13221858	13223917	Dp	1188	28.6173	5.08
chr2R	13429653	13430341	CG42321	5	29.9528	4.90
chr2R	13482445	13483366	arr	101	29.275	4.64
chr2R	13484865	13485353	cbc	204	12.5329	3.26
chr2R	13494425	13495461	bbc	146	22.7821	4.12
chr2R	13506387	13508051	mip120	1362	24.478	4.17
chr2R	13506387	13508051	EfTuM	235	24.478	4.17
chr2R	13592286	13593447	CG6191	117	14.4835	3.12

chr2R	13592286	13593447	CG45088	117	14.4835	3.12
chr2R	13821837	13823035	fl(2)d	1680	18.4467	4.12
chr2R	13853683	13854539	CG6543	60	60.914	7.12
chr2R	13859008	13859380	Uba3	221	9.31717	3.26
chr2R	13897464	13898030	shot	5	29.1409	4.13
chr2R	13941030	13942333	shot	28	34.4785	4.89
chr2R	13953648	13954947	AGO1	90	22.7768	4.26
chr2R	13953648	13954947	mRpL53	259	22.7768	4.26
chr2R	13953648	13954947	CG33155	259	22.7768	4.26
chr2R	13956794	13958311	AGO1	169	22.1547	4.20
chr2R	13959795	13961383	Rpn13	420	28.2129	4.67
chr2R	13959795	13961383	Cp1	150	28.2129	4.67
chr2R	13991045	13991393	mam	15	16.4597	3.60
chr2R	14006781	14007223	mir-4978	1018	24.6353	4.15
chr2R	14140307	14141622	Prosap	38	23.107	4.37
chr2R	14152053	14153605	Rcd1	106	27.9015	5.42
chr2R	14152053	14153605	pea	550	27.9015	5.42
chr2R	14164202	14165573	Tango7	65	13.933	3.46
chr2R	14164202	14165573	CG8323	494	13.933	3.46
chr2R	14170322	14171369	CG8331	76	14.4838	3.51
chr2R	14170322	14171369	CR44368	380	14.4838	3.51
chr2R	14170322	14171369	CG8331	622	14.4838	3.51
chr2R	14185039	14186845	CG30069	55	38.6997	5.53
chr2R	14212176	14213115	Sox15	45	12.5551	3.45
chr2R	14215921	14217160	RpS23	103	18.0578	3.52
chr2R	14234915	14235486	Opa1	243	16.1486	3.72
chr2R	14239288	14240433	CG8485	86	15.1903	3.49
chr2R	14239288	14240433	Usp20-33	425	15.1903	3.49
chr2R	14255511	14256948	Hsc70-5	162	37.0135	5.16
chr2R	14255511	14256948	CG8531	593	37.0135	5.16
chr2R	14258883	14259416	beta4GalNAcTA	12	19.6847	4.21
chr2R	14270250	14271003	SelD	234	16.8083	3.43
chr2R	14270250	14271003	conv	304	16.8083	3.43
chr2R	14288894	14289776	Ih	49	18.0222	4.09
chr2R	14352130	14352756	CG8613	75	22.4738	4.65
chr2R	14356647	14357420	CG8617	160	13.7586	3.38
chr2R	14360036	14360418	Arc1	47	31.7752	5.76
chr2R	14361821	14362478	Arc2	62	28.0496	5.38
chr2R	14362943	14363464	Tfb1	111	24.4817	4.31
chr2R	14457906	14458367	Oaz	361	8.93717	3.16
chr2R	14503033	14504796	Cpsf160	563	33.7434	5.50
chr2R	14503033	14504796	Asx	94	33.7434	5.50
chr2R	14513885	14514406	CG30197	70	15.0256	3.59
chr2R	14575123	14575868	LamC	61	14.5418	3.54
chr2R	14600631	14601740	Rpl1	107	40.019	6.21
chr2R	14600631	14601740	Blos1	168	40.019	6.21
chr2R	14603968	14604383	tra2	164	12.9449	3.19
chr2R	14617509	14618242	Su(var)2-HP2	202	22.6589	4.69
chr2R	14618514	14619291	Sec61beta	245	43.8895	5.98
chr2R	14621000	14622165	CG12863	310	31.2751	4.80
chr2R	14621000	14622165	Uhg5	30	31.2751	4.80
chr2R	14621000	14622165	snoRNA:Me18S-C136(	151	31.2751	4.80
chr2R	14621000	14622165	noRNA:Me28S-C3227	457	31.2751	4.80
chr2R	14645114	14645737	CG10139	262	13.5778	3.39
chr2R	14751505	14752786	Rpn6	96	14.2378	3.18
chr2R	14751505	14752786	ave	345	14.2378	3.18

chr2R	14756233	14757181	CG10151	320	26.4811	4.54
chr2R	14756233	14757181	ND-B15	93	26.4811	4.54
chr2R	14757445	14757896	Trs31	147	10.5965	3.14
chr2R	14770375	14772145	BEAF-32	1125	22.4744	4.59
chr2R	14770375	14772145	CR42254	703	22.4744	4.59
chr2R	14770375	14772145	BEAF-32	678	22.4744	4.59
chr2R	14770375	14772145	CR42254	298	22.4744	4.59
chr2R	14770375	14772145	BEAF-32	293	22.4744	4.59
chr2R	14847912	14848520	CG10209	23	16.6008	3.91
chr2R	14854036	14855076	Ciao1	148	15.7576	3.64
chr2R	14855384	14856181	HPS1	244	12.9449	3.19
chr2R	14860351	14860896	U3-55K	141	20.0324	4.39
chr2R	14870471	14871090	Pcf11	358	15.3956	3.34
chr2R	14881995	14882473	Cyp6a20	80	30.1036	5.50
chr2R	14928003	14928533	Kank	54	15.6701	4.04
chr2R	14962135	14963609	aPKC	4	17.5225	3.55
chr2R	14962135	14963609	ckn	326	17.5225	3.55
chr2R	15127984	15129013	chn	53	22.8377	5.00
chr2R	15216073	15216674	CG11808	113	16.6664	3.68
chr2R	15216996	15217999	mRpL41	87	50.1621	6.51
chr2R	15252467	15252950	scb	51	17.9766	3.79
chr2R	15310763	15311190	CG8152	100	13.3967	3.49
chr2R	15321794	15323247	CG8155	642	38.1784	5.56
chr2R	15321794	15323247	Arf51F	11	38.1784	5.56
chr2R	15482139	15482970	CG8180	105	15.8056	3.74
chr2R	15538013	15538655	CG30467	178	11.2123	3.08
chr2R	15540945	15543002	CG8187	151	18.4894	3.75
chr2R	15540945	15543002	Vha36-1	80	18.4894	3.75
chr2R	15540945	15543002	eIF2B-gamma	1146	18.4894	3.75
chr2R	15565080	15565786	CG8195	189	21.1537	4.50
chr2R	15573542	15574115	CG8204	9	19.8434	4.08
chr2R	15613484	15614366	CG42524	24	20.2357	4.44
chr2R	15675450	15676815	fus	94	12.7164	3.26
chr2R	15675450	15676815	CR45143	581	12.7164	3.26
chr2R	15675450	15676815	fus	810	12.7164	3.26
chr2R	15682703	15683236	Ranbp11	254	16.0217	4.13
chr2R	15734964	15735664	CG30089	182	10.0304	3.25
chr2R	15816798	15817404	Zasp52	61	9.82793	3.30
chr2R	15847878	15849073	tun	480	24.3303	3.88
chr2R	15861316	15862226	Gpo-1	113	26.2481	4.50
chr2R	15931238	15932127	bdg	57	11.8392	3.20
chr2R	15991875	15992875	Pex11	7	13.4155	3.32
chr2R	15991875	15992875	CG8320	39	13.4155	3.32
chr2R	16000022	16001357	ATPCL	37	15.0109	3.50
chr2R	16000022	16001357	CG8370	930	15.0109	3.50
chr2R	16000022	16001357	ATPCL	1040	15.0109	3.50
chr2R	16001710	16002392	CG8370	171	17.3003	3.84
chr2R	16007315	16007851	CG8386	69	13.4155	3.32
chr2R	16017576	16018761	CG8399	80	35.3711	5.52
chr2R	16047317	16048060	CG8405	257	10.346	3.32
chr2R	16109177	16110029	Ric	205	23.1868	4.55
chr2R	16124845	16125627	spin	182	24.161	4.35
chr2R	16138563	16139250	Got1	111	32.4772	5.16
chr2R	16142221	16143485	Aats-cys	134	13.266	3.25
chr2R	16148990	16149624	CG10731	222	13.1958	3.59
chr2R	16150002	16151108	Ibk	102	25.666	3.96

chr2R	16186550	16188122	clu	211	14.113	3.45
chr2R	16195759	16197905	calypso	827	43.6796	6.57
chr2R	16195759	16197905	CG44242	777	43.6796	6.57
chr2R	16195759	16197905	CG42837	119	43.6796	6.57
chr2R	16195759	16197905	CG44243	119	43.6796	6.57
chr2R	16226330	16226856	Tsf3	42	13.7974	3.67
chr2R	16289212	16289587	Jhl-26	65	20.9201	4.23
chr2R	16293807	16294901	CG7747	386	37.592	5.71
chr2R	16293807	16294901	Atg9	117	37.592	5.71
chr2R	16585759	16586867	RpLP2	120	25.5629	4.30
chr2R	16593952	16594946	CG8306	197	59.8005	7.03
chr2R	16640864	16641366	gprs	152	14.9177	4.06
chr2R	16691510	16692017	CG5210	46	11.9617	3.36
chr2R	16773302	16773878	Sod2	385	10.5518	3.22
chr2R	16830193	16830916	CR43650	56	13.0829	3.25
chr2R	16856082	16857273	Dek	55	26.2299	4.71
chr2R	17015123	17016165	Ehbp1	105	13.8173	3.24
chr2R	17018634	17019572	CG8963	127	35.0912	5.74
chr2R	17027123	17028442	RhoGEF2	528	14.363	3.42
chr2R	17048873	17049650	CG9646	45	21.5388	4.14
chr2R	17062107	17062839	CG8950	103	47.101	6.87
chr2R	17062107	17062839	CG6967	243	47.101	6.87
chr2R	17097189	17097778	GstS1	4	29.0614	4.92
chr2R	17412712	17413207	Prosalpha5	77	22.4946	4.47
chr2R	17418946	17419686	cnk	61	17.8796	3.53
chr2R	17427186	17427571	CG6550	55	10.4574	3.17
chr2R	17430634	17431570	Bap55	255	23.795	4.72
chr2R	17430634	17431570	Lhr	187	23.795	4.72
chr2R	17436353	17436933	CG18467	3658	19.6821	3.60
chr2R	17444133	17445129	EDTP	160	19.4731	3.87
chr2R	17457466	17459018	CG14478	216	19.8396	4.24
chr2R	17469522	17470219	qkr54B	188	28.3467	4.64
chr2R	17484918	17485169	NT5E-2	32	9.62378	3.35
chr2R	17514124	17514815	CG4853	108	16.148	3.97
chr2R	17536288	17536905	eIF3-S9	173	24.478	4.17
chr2R	17546354	17548736	RpL18A	703	49.9338	6.41
chr2R	17546354	17548736	MESR4	126	49.9338	6.41
chr2R	17568036	17569199	Ns2	378	18.9129	4.22
chr2R	17568036	17569199	POSH	103	18.9129	4.22
chr2R	17592759	17593672	Smurf	151	35.222	5.63
chr2R	17665435	17666535	eIF3-S8	168	31.4773	4.99
chr2R	17672029	17672621	P32	217	16.4597	3.60
chr2R	17682548	17684043	Sema-1b	284	18.9556	3.49
chr2R	17682548	17684043	HPS4	210	18.9556	3.49
chr2R	17697554	17698495	rdgBbeta	70	10.9124	3.02
chr2R	17739202	17740463	CG6401	277	14.8323	3.42
chr2R	17739202	17740463	CG4996	62	14.8323	3.42
chr2R	17750189	17750523	CG5002	84	16.1486	3.72
chr2R	17760988	17761983	CG5009	277	46.1917	5.54
chr2R	17788688	17790109	Ubc10	151	31.7667	5.27
chr2R	17788688	17790109	CG5033	419	31.7667	5.27
chr2R	17800780	17801965	CR45270	55	22.1547	3.86
chr2R	17800780	17801965	grh	125	22.1547	3.86
chr2R	17800780	17801965	CR45270	463	22.1547	3.86
chr2R	17823262	17824178	grh	62	18.4922	4.51
chr2R	17846319	17847069	olf186-M	60	13.0185	3.36

chr2R	17850645	17851695	olf186-F	566	17.0617	3.75
chr2R	17869032	17869695	CR44417	359	25.4181	3.89
chr2R	17870200	17872224	Mapmodulin	42	22.7522	4.25
chr2R	18094531	18095562	CG5757	272	21.5388	4.14
chr2R	18094531	18095562	CG5098	232	21.5388	4.14
chr2R	18125551	18126516	stau	10	20.6326	4.08
chr2R	18125551	18126516	Spn55B	577	20.6326	4.08
chr2R	18133640	18134703	Hsf	197	18.9194	4.06
chr2R	18140615	18142382	pAbp	189	18.6225	3.65
chr2R	18150817	18152158	CG5742	424	26.3597	4.67
chr2R	18150817	18152158	adp	81	26.3597	4.67
chr2R	18156299	18158496	lolal	58	28.1065	4.51
chr2R	18156299	18158496	CG10914	1422	28.1065	4.51
chr2R	18179607	18180367	CG5726	261	15.9481	3.72
chr2R	18387767	18388286	IM3	112	8.36405	3.11
chr2R	18387767	18388286	IM3	112	8.36405	3.11
chr2R	18410813	18413119	imd	647	64.5252	7.51
chr2R	18410813	18413119	Dp1	153	64.5252	7.51
chr2R	18439690	18441497	CG30118	648	32.1714	5.05
chr2R	18439690	18441497	slim	159	32.1714	5.05
chr2R	18611293	18611927	GEFmeso	45	18.9979	4.07
chr2R	18638479	18639461	CG30122	291	58.6836	6.62
chr2R	18687967	18689298	Sik3	65	29.4678	4.44
chr2R	18754865	18755394	Cyp12b2	97	22.4738	4.31
chr2R	18776641	18777047	CG15093	43	12.3103	3.60
chr2R	18796277	18796913	MFS15	189	19.2325	3.79
chr2R	18801849	18802787	MFS14	178	16.1001	3.38
chr2R	18819452	18819941	Phb2	228	28.6174	4.59
chr2R	18829148	18829806	CG15097	106	18.2193	3.88
chr2R	18834340	18835794	CG15098	547	31.7752	5.76
chr2R	18834340	18835794	CG15083	60	31.7752	5.76
chr2R	18846147	18846741	CG15099	182	28.0815	4.70
chr2R	18850142	18850609	MetRS	174	14.5491	3.73
chr2R	18857637	18858110	Jheh2	24	21.8472	4.24
chr2R	19129058	19129983	CG15117	405	12.8577	3.42
chr2R	19129058	19129983	botv	117	12.8577	3.42
chr2R	19136617	19138370	CG15118	109	25.4357	4.46
chr2R	19136617	19138370	CG15111	399	25.4357	4.46
chr2R	19141512	19142670	ena	55	17.8061	3.80
chr2R	19155141	19155472	ena	54	10.9857	3.44
chr2R	19228916	19230102	cora	45	14.5532	3.08
chr2R	19289910	19290737	FK506-bp2	121	34.1874	5.45
chr2R	19305113	19306002	CG7461	59	10.7267	3.01
chr2R	19357485	19358628	CG11961	134	17.6476	3.86
chr2R	19390075	19390531	CG11257	227	11.0619	3.40
chr2R	19424025	19425479	hts	52	27.5357	4.33
chr2R	19424025	19425479	CalpA	261	27.5357	4.33
chr2R	19436489	19437300	Fak	128	26.4027	4.98
chr2R	19442109	19442887	RpL11	62	33.8336	4.51
chr2R	19450698	19451758	betaTub56D	38	60.0998	7.14
chr2R	19496375	19497025	hpo	32	14.6522	3.43
chr2R	19668908	19670174	hrg	4	23.6983	4.47
chr2R	20256815	20258212	Ate1	258	18.0528	3.89
chr2R	20256815	20258212	Hsl	517	18.0528	3.89
chr2R	20274005	20274868	CG10444	68	55.28	6.06
chr2R	20274005	20274868	CG11788	165	55.28	6.06

chr2R	20286261	20287183	CG16868	108	18.4461	4.65
chr2R	20312997	20313806	CG13868	68	15.3316	3.75
chr2R	20319522	20321002	CG8920	88	28.0503	4.37
chr2R	20327178	20328055	CG8929	153	35.7473	5.94
chr2R	20534214	20534724	CG18067	15	14.8511	3.91
chr2R	20622774	20624007	CG11180	4	31.3656	5.03
chr2R	20622774	20624007	FAM21	113	31.3656	5.03
chr2R	20640249	20642037	rig	366	25.4822	5.00
chr2R	20640249	20642037	maf-S	72	25.4822	5.00
chr2R	20640249	20642037	CG11110	694	25.4822	5.00
chr2R	20643311	20644519	CG33786	448	14.1514	3.65
chr2R	20643311	20644519	CG33785	448	14.1514	3.65
chr2R	20643311	20644519	DMAP1	198	14.1514	3.65
chr2R	20666327	20667401	Fem-1	193	37.3549	6.25
chr2R	20671093	20671668	CG30152	148	17.9766	3.79
chr2R	20831724	20833148	sktl	315	13.0413	3.50
chr2R	20994755	20998551	Prosalpha3	1068	19.3915	3.78
chr2R	20994755	20998551	CG10543	252	19.3915	3.78
chr2R	21016845	21017474	CG3295	232	15.8278	4.02
chr2R	21056514	21057368	shg	49	36.4258	5.08
chr2R	21057657	21058411	cpa	56	25.8869	5.15
chr2R	21060246	21061131	CG15653	437	14.313	3.68
chr2R	21060246	21061131	mRpL54	196	14.313	3.68
chr2R	21072111	21072992	CG10527	49	27.5549	4.61
chr2R	21141718	21142327	Magi	31	21.4109	4.03
chr2R	21143409	21144341	Xbp1	96	17.2105	3.73
chr2R	21159993	21160656	CG30389	15	20.3132	3.93
chr2R	21170946	21171357	Xpd	64	8.88712	3.00
chr2R	21178816	21180021	Pu	21	23.603	4.46
chr2R	21183268	21183735	tud	203	13.4598	3.41
chr2R	21292758	21294359	Sgf29	220	21.7383	3.99
chr2R	21292758	21294359	RpL29	99	21.7383	3.99
chr2R	21322745	21324479	CG30394	346	32.0617	5.09
chr2R	21322745	21324479	dom	237	32.0617	5.09
chr2R	21379012	21379637	cv-2	75	16.2152	3.83
chr2R	21385925	21386913	EfSec	466	13.233	3.32
chr2R	21385925	21386913	Acox57D-p	62	13.233	3.32
chr2R	21480725	21481333	Sdc	46	21.5388	4.14
chr2R	21496629	21497717	Fkbp14	48	20.0928	3.73
chr2R	21522252	21522930	Egfr	76	15.7598	3.33
chr2R	21546324	21546937	Egfr	84	13.2485	3.71
chr2R	21611422	21612252	CG15673	1274	9.6128	3.09
chr2R	21612632	21613281	CG15673	51	17.0699	4.00
chr2R	21616268	21616910	CG18870	217	23.8572	4.25
chr2R	21623919	21624536	PTP-ER	181	30.2028	4.64
chr2R	21633906	21635347	CR42547	628	13.6828	3.18
chr2R	21633906	21635347	CG10321	10	13.6828	3.18
chr2R	21645895	21646799	CG10082	49	19.1506	3.62
chr2R	21662026	21662905	pirk	42	8.91069	3.06
chr2R	21685155	21685747	CG10306	224	14.7514	3.83
chr2R	21687111	21687760	Tbp	83	18.3198	4.34
chr2R	21688084	21688937	CG30285	37	39.3626	5.63
chr2R	21688084	21688937	CG10307	264	39.3626	5.63
chr2R	21690702	21691295	Tim10	189	26.3532	4.85
chr2R	21690702	21691295	CG42497	189	26.3532	4.85
chr2R	21690702	21691295	Tim10	189	26.3532	4.85

chr2R	21690702	21691295	CG42496	344	26.3532	4.85
chr2R	21690702	21691295	Ppcdc	344	26.3532	4.85
chr2R	21696712	21697145	CngB	243	15.9866	4.00
chr2R	21702691	21705057	HmgZ	409	15.6213	3.53
chr2R	21973027	21973556	CG9304	143	17.5212	4.10
chr2R	22075845	22076511	GlcT-1	69	35.9049	5.75
chr2R	22080037	22080699	Synj	96	15.4662	3.83
chr2R	22099164	22099716	CG11073	33	32.1714	5.05
chr2R	22174139	22175220	CG3045	61	9.61014	3.01
chr2R	22174139	22175220	CG6758	291	9.61014	3.01
chr2R	22185981	22186434	Vps35	141	10.8826	3.18
chr2R	22243723	22244180	dve	54	17.3931	3.96
chr2R	22311665	22312258	wdp	35	22.1008	4.45
chr2R	22317419	22319021	Gp150	66	29.8249	4.54
chr2R	22410989	22412227	qkr58E-3	93	17.0617	3.75
chr2R	22410989	22412227	Mes4	336	17.0617	3.75
chr2R	22415098	22416348	qkr58E-1	197	29.752	4.98
chr2R	22415098	22416348	qkr58E-2	238	29.752	4.98
chr2R	22483247	22483628	CG4554	57	8.95089	3.07
chr2R	22561415	22562233	px	86	47.7586	6.99
chr2R	22592414	22593078	dnr1	22	16.1079	3.62
chr2R	22605965	22606697	RpS16	161	44.8735	6.87
chr2R	22637295	22637856	Cdk9	161	16.8296	3.85
chr2R	22637295	22637856	bonsai	203	16.8296	3.85
chr2R	22640663	22641489	RpS24	147	34.593	5.11
chr2R	22645591	22646494	CG2852	64	38.5457	5.62
chr2R	22667074	22667556	CG30273	44	18.4467	4.12
chr2R	22667074	22667556	CG30269	44	18.4467	4.12
chr2R	22669256	22669908	CG13516	80	20.4687	4.62
chr2R	22670490	22671390	RYBP	22	26.6086	4.56
chr2R	22678083	22678673	CR44761	4654	15.4302	3.28
chr2R	22686055	22686424	Ppa	2810	11.8909	3.14
chr2R	22688372	22689218	Ppa	3	18.0528	3.89
chr2R	22688372	22689218	CR44762	104	18.0528	3.89
chr2R	22740179	22740951	jbug	40	16.2152	3.83
chr2R	22798772	22799971	blw	105	28.596	4.79
chr2R	22853449	22853892	CG13531	16	25.9773	4.78
chr2R	22885484	22887020	CG3499	447	27.7017	4.80
chr2R	22885484	22887020	asrij	134	27.7017	4.80
chr2R	22895997	22896753	nahoda	50	20.8177	4.43
chr2R	22933587	22934960	CG3788	162	23.9355	4.63
chr2R	22935646	22937045	CG3800	198	37.0943	5.28
chr2R	22998730	22999012	CG9896	17	12.9449	3.19
chr2R	23356618	23357567	CG3500	77	16.7675	3.89
chr2R	23365859	23366320	vir	238	16.8032	3.43
chr2R	23366796	23367454	Ice1	125	17.9236	3.69
chr2R	23376421	23377098	Golgin245	228	19.2325	3.79
chr2R	23385782	23386776	CG30415	337	22.3997	3.93
chr2R	23385782	23386776	eIF2B-delta	426	22.3997	3.93
chr2R	23419060	23419891	CG9812	49	14.8323	3.51
chr2R	23523056	23524254	RabX1	170	22.1754	4.06
chr2R	23523056	23524254	mi	72	22.1754	4.06
chr2R	23546776	23548786	wmd	216	31.9013	5.43
chr2R	23546776	23548786	levy	129	31.9013	5.43
chr2R	23560402	23561021	Pi3K59F	117	19.8396	4.24
chr2R	23667061	23667413	Pde8	115	16.7966	3.79

chr2R	23701504	23702848	sigmar	137	18.6132	4.27
chr2R	23701504	23702848	l(2)dtl	207	18.6132	4.27
chr2R	23706807	23707527	l(2)tid	161	23.1868	4.55
chr2R	23709408	23710081	l(2)not	33	30.2242	4.84
chr2R	23712019	23714184	Sesn	0	41.4038	5.77
chr2R	23745780	23746969	egl	91	32.0738	4.83
chr2R	23849836	23851156	eIF6	589	18.098	3.68
chr2R	23849836	23851156	CR45928	561	18.098	3.68
chr2R	23849836	23851156	ytr	183	18.098	3.68
chr2R	23851480	23851896	ytr	193	14.85	3.60
chr2R	23851480	23851896	gbb	112	14.85	3.60
chr2R	23856554	23857083	Pym	12	29.6618	4.80
chr2R	23864658	23865824	CG4585	18	18.4788	3.97
chr2R	23864658	23865824	CG5569	3	18.4788	3.97
chr2R	23868156	23868682	Thiolase	153	19.402	4.00
chr2R	23871524	23872297	ken	1892	9.7724	3.04
chr2R	23881981	23882514	CG4882	147	13.2926	3.72
chr2R	23897837	23899018	CG5339	513	23.7122	4.65
chr2R	23897837	23899018	Lpt	199	23.7122	4.65
chr2R	23906552	23907413	Nap1	30	58.1477	7.76
chr2R	23932797	23933885	SERCA	209	27.4635	4.46
chr2R	23939136	23940804	CG3735	717	23.9784	4.58
chr2R	23939136	23940804	Taldo	62	23.9784	4.58
chr2R	23969827	23970831	CG10904	494	17.0541	4.00
chr2R	23969827	23970831	CG3065	263	17.0541	4.00
chr2R	23978301	23979939	Sox14	272	25.5873	3.88
chr2R	24021716	24022670	CG3907	281	23.6413	4.76
chr2R	24021716	24022670	CG11388	484	23.6413	4.76
chr2R	24026337	24027348	EbpIII	68	21.6308	4.42
chr2R	24028436	24028885	HSPC300	138	21.2486	4.04
chr2R	24029191	24029773	CG3163	51	18.1467	3.77
chr2R	24045323	24046941	tsr	104	30.2242	4.84
chr2R	24057647	24058248	eIF-5A	175	19.2693	3.72
chr2R	24062057	24062446	RpL39	188	14.85	3.60
chr2R	24086308	24087085	snama	129	21.2353	3.83
chr2R	24097901	24098671	gek	180	33.096	5.38
chr2R	24132489	24134481	tamo	173	36.5598	5.42
chr2R	24132489	24134481	Zfrp8	610	36.5598	5.42
chr2R	24173802	24175588	mRpS17	481	24.0111	4.23
chr2R	24173802	24175588	Nop60B	46	24.0111	4.23
chr2R	24186307	24187190	ocm	61	15.1909	3.62
chr2R	24197700	24198228	CG3356	72	14.1207	3.37
chr2R	24401664	24403388	Slik	207	35.6204	5.06
chr2R	24401664	24403388	Rpn8	827	35.6204	5.06
chr2R	24514428	24515389	Letm1	154	14.1855	3.24
chr2R	24531464	24532566	CG13585	176	19.5406	4.29
chr2R	24560596	24561147	Nurf-38	98	20.8154	3.97
chr2R	24570868	24572068	CG3511	140	30.2937	5.26
chr2R	24570868	24572068	Fcp1	174	30.2937	5.26
chr2R	24570868	24572068	CG3511	362	30.2937	5.26
chr2R	24580948	24581762	pio	132	19.5406	4.29
chr2R	24598883	24600340	ATPsynF	257	29.5256	4.78
chr2R	24598883	24600340	CG3548	132	29.5256	4.78
chr2R	24603525	24603975	CG3570	105	14.3921	3.60
chr2R	24606554	24608033	CG4707	202	35.7108	5.34
chr2R	24606554	24608033	CG42360	445	35.7108	5.34

chr2R	24606554	24608033	CG42361	851	35.7108	5.34
chr2R	24664402	24665098	ND-19	224	32.6609	5.19
chr2R	24668753	24669868	CG33228	24	13.1871	3.44
chr2R	24668753	24669868	Pof	90	13.1871	3.44
chr2R	24762732	24763558	CG12848	183	30.659	5.33
chr2R	24762732	24763558	CG3894	111	30.659	5.33
chr2R	24767315	24768346	GstE12	89	32.8696	5.11
chr2R	24774654	24775251	Eps-15	181	13.7586	3.38
chr2R	24793072	24794205	Reg-5	171	22.1773	4.36
chr2R	24793072	24794205	CR43257	225	22.1773	4.36
chr2R	24797098	24797478	Orc4	93	17.835	4.05
chr2R	24814431	24815024	Dll	60	13.3129	3.19
chr2R	24927876	24928436	pain	65	15.3751	3.41
chr2R	24940045	24940650	CG2811	44	23.0834	4.22
chr2R	24947998	24948828	CG2790	39	21.9015	3.98
chr2R	24947998	24948828	CG12851	305	21.9015	3.98
chr2R	24963959	24965300	CG2765	43	30.659	5.33
chr2R	24969077	24970098	RpL19	21	27.2717	4.34
chr2R	24969077	24970098	CG3776	189	27.2717	4.34
chr2R	24982734	24983523	emp	64	26.4932	4.87
chr2R	25011063	25011664	zip	105	40.9057	5.04
chr3L	129319	129711	Pdk1	55	20.267	3.72
chr3L	198753	200926	rno	379	67.4611	6.41
chr3L	198753	200926	mri	790	67.4611	6.41
chr3L	211026	211756	CG7028	202	29.4799	5.24
chr3L	224013	226488	CG33229	179	37.5101	5.13
chr3L	224013	226488	CR42862	179	37.5101	5.13
chr3L	246139	247140	E(bx)	260	15.2362	3.68
chr3L	262090	265260	Tudor-SN	177	22.0067	3.51
chr3L	262090	265260	mRpL17	575	22.0067	3.51
chr3L	291811	293087	RhoGEF3	119	27.9244	4.73
chr3L	319052	320062	fwd	37	29.2582	4.64
chr3L	340651	341598	mthl10	19	17.1334	3.86
chr3L	360877	362123	Cdc5	141	26.8078	4.99
chr3L	360877	362123	CG1233	53	26.8078	4.99
chr3L	377819	378433	trh	875	46.9396	6.77
chr3L	539876	541348	klar	54	36.7239	4.89
chr3L	539876	541348	CG17180	290	36.7239	4.89
chr3L	593105	593675	MED14	161	38.5457	5.62
chr3L	603319	604200	CG13893	81	31.0275	4.87
chr3L	606203	607007	Reg-2	30	25.5021	4.93
chr3L	616589	617216	CR42719	393	21.1452	4.92
chr3L	627825	628819	CR43334	68	22.6849	4.34
chr3L	653415	654648	MED30	144	16.9176	3.68
chr3L	653415	654648	Rev1	417	16.9176	3.68
chr3L	681567	682785	CG3386	20	20.7279	3.87
chr3L	837081	838469	CG13901	27	20.4297	4.30
chr3L	837081	838469	CG13887	256	20.4297	4.30
chr3L	881438	882328	CG13907	105	17.0617	3.75
chr3L	894871	896297	CG34056	49	23.603	4.46
chr3L	1034274	1035884	CG9205	84	20.7897	3.69
chr3L	1034274	1035884	trio	95	20.7897	3.69
chr3L	1034274	1035884	CG9205	404	20.7897	3.69
chr3L	1204482	1205192	CG9119	37	12.9163	3.48
chr3L	1247383	1248697	Psf1	382	24.6739	4.44
chr3L	1247383	1248697	Sac1	40	24.6739	4.44

chr3L	1306800	1307837	CG2277	52	14.2623	3.03
chr3L	1306800	1307837	CG2469	163	14.2623	3.03
chr3L	1306800	1307837	CG2277	358	14.2623	3.03
chr3L	1306800	1307837	CG2469	487	14.2623	3.03
chr3L	1329654	1330996	ND-ACP	219	20.5848	4.12
chr3L	1334102	1335877	CG2211	333	19.4731	3.87
chr3L	1334102	1335877	l(3)02640	21	19.4731	3.87
chr3L	1334102	1335877	CG2211	450	19.4731	3.87
chr3L	1341744	1342554	CG2199	172	16.6352	3.59
chr3L	1349603	1350742	Ptp61F	303	29.0355	4.51
chr3L	1351096	1351537	312	99	23.4355	4.92
chr3L	1463520	1464101	rho	50	19.7164	4.39
chr3L	1501839	1502819	pUf68	173	17.3447	3.55
chr3L	1533535	1533994	Iml1	166	9.98653	3.18
chr3L	1536125	1537909	CG12091	435	41.5133	5.85
chr3L	1536125	1537909	CG7852	134	41.5133	5.85
chr3L	1545982	1546857	Cct1	159	23.5867	4.03
chr3L	1557598	1559155	Pex10	662	31.5922	4.96
chr3L	1557598	1559155	CG12099	405	31.5922	4.96
chr3L	1557598	1559155	Pex10	108	31.5922	4.96
chr3L	1568119	1568732	CG7879	117	16.6278	3.44
chr3L	1569077	1570245	CG12004	181	20.9201	4.23
chr3L	1576533	1577308	CG13917	9	20.2969	4.58
chr3L	1629071	1629421	GC	24	15.0539	4.16
chr3L	1644778	1645650	alphaCOP	32	18.0528	3.89
chr3L	1644778	1645650	CG13919	299	18.0528	3.89
chr3L	1661782	1662398	CG7971	2273	22.0549	4.83
chr3L	1663497	1665467	CG7971	255	17.72	3.93
chr3L	1663497	1665467	CG7974	328	17.72	3.93
chr3L	1667983	1669029	RpL23A	109	20.6511	3.99
chr3L	1730853	1731360	drpr	92	13.7504	3.34
chr3L	1741459	1741938	Gk	39	9.53515	3.17
chr3L	1861188	1862391	Rap1	163	29.8651	4.95
chr3L	1864518	1866093	HBS1	336	25.3073	4.90
chr3L	1864518	1866093	CG12025	147	25.3073	4.90
chr3L	1875936	1876494	dre4	142	21.8472	4.24
chr3L	1947057	1948107	mu2	479	21.5451	4.29
chr3L	1947057	1948107	Cnb	200	21.5451	4.29
chr3L	1963406	1965178	CR45828	745	21.3964	4.32
chr3L	1963406	1965178	mv	267	21.3964	4.32
chr3L	1963406	1965178	SCOT	13	21.3964	4.32
chr3L	2255697	2256987	CG1275	45	33.9493	5.60
chr3L	2257274	2258454	CG1275	456	20.9706	4.29
chr3L	2257274	2258454	CG2034	237	20.9706	4.29
chr3L	2373180	2374320	mRpL23	146	27.2728	4.57
chr3L	2373180	2374320	CG9004	123	27.2728	4.57
chr3L	2377152	2377803	CG15877	202	13.3968	3.40
chr3L	2377152	2377803	CG45186	300	13.3968	3.40
chr3L	2466733	2467338	CG45186	78	24.2416	4.13
chr3L	2553402	2554517	Spn	3	13.6339	3.24
chr3L	2585453	2586711	msn	105	43.92	5.43
chr3L	2587310	2588680	RpL8	168	17.8061	3.80
chr3L	2589252	2590344	dos	58	26.9692	4.13
chr3L	2598924	2600325	CG16985	152	17.8061	3.80
chr3L	2598924	2600325	CG12182	131	17.8061	3.80
chr3L	2630990	2633262	MEP-1	1193	27.0287	4.74

chr3L	2630990	2633262	CG42245	1193	27.0287	4.74
chr3L	2630990	2633262	MEP-1	633	27.0287	4.74
chr3L	2634936	2636427	MEP-1	162	24.0111	4.23
chr3L	2771771	2772418	CG9977	202	25.9862	5.10
chr3L	2773205	2775140	CG12093	496	42.3659	5.86
chr3L	2773205	2775140	Atg2	166	42.3659	5.86
chr3L	3035184	3036122	CG2162	684	14.4835	3.36
chr3L	3037856	3038829	CG2162	91	13.1443	3.03
chr3L	3045697	3047581	scramb2	187	38.0636	5.79
chr3L	3045697	3047581	Sk2	461	38.0636	5.79
chr3L	3050222	3050757	CG32485	36	19.8968	4.56
chr3L	3052118	3052974	CG1271	4	19.7391	4.33
chr3L	3059751	3061291	CG16753	5768	30.4474	5.04
chr3L	3069924	3071484	CG32486	112	44.755	5.93
chr3L	3069924	3071484	CG11486	405	44.755	5.93
chr3L	3090548	3090949	snRNA:U5:63BC	27	15.4198	4.11
chr3L	3127362	3127847	prominin-like	98	21.4787	3.91
chr3L	3142665	3143535	CG11537	171	17.9761	4.32
chr3L	3147752	3150066	Usp5	178	33.2775	5.29
chr3L	3147752	3150066	BtbVII	465	33.2775	5.29
chr3L	3159925	3160532	CG15812	121	14.85	3.60
chr3L	3192195	3193533	CG14965	764	61.7706	7.18
chr3L	3192195	3193533	Hsp83	40	61.7706	7.18
chr3L	3193915	3194438	Hsp83	840	14.4693	3.66
chr3L	3198358	3199834	CG32276	124	44.9986	5.74
chr3L	3198358	3199834	gry	166	44.9986	5.74
chr3L	3222620	3223996	RpL28	661	24.7935	4.14
chr3L	3222620	3223996	CG17737	281	24.7935	4.14
chr3L	3301057	3301418	CG14968	60	13.8742	3.80
chr3L	3318810	3320310	PIG-C	136	25.9435	4.55
chr3L	3318810	3320310	CG12016	458	25.9435	4.55
chr3L	3318810	3320310	CG42456	458	25.9435	4.55
chr3L	3325510	3326829	Strip	411	51.2958	5.96
chr3L	3325510	3326829	PHGPx	93	51.2958	5.96
chr3L	3336703	3337411	kst	57	23.4985	4.33
chr3L	3338044	3338969	kst	1618	14.3326	3.79
chr3L	3373619	3374520	YT521-B	56	31.5929	5.01
chr3L	3373619	3374520	CG12010	167	31.5929	5.01
chr3L	3418374	3418885	sty	591	18.0252	3.89
chr3L	3460143	3460804	eIF5B	216	17.3447	3.55
chr3L	3625991	3626476	dar1	24	15.882	3.86
chr3L	3632492	3633132	dar1	6575	10.8404	3.08
chr3L	3765710	3766085	CG32264	173	12.2143	3.02
chr3L	3787570	3788235	CG32264	165	15.2023	3.58
chr3L	3809792	3810416	CG32262	166	14.684	3.71
chr3L	3811167	3811521	CG12006	35	14.4266	4.07
chr3L	3814112	3814732	Scsalpha	225	15.3789	3.57
chr3L	3902117	3902854	Ubi-p63E	12	22.6242	4.28
chr3L	3903235	3903836	Sc2	107	25.1778	4.75
chr3L	3952243	3952905	CG14984	5	10.0365	3.52
chr3L	4039731	4040904	CG11593	180	29.9825	4.91
chr3L	4039731	4040904	Rcd5	134	29.9825	4.91
chr3L	4070490	4071968	wit	49	30.5158	4.31
chr3L	4102072	4102850	CG14995	58	39.7681	5.41
chr3L	4103501	4104289	Fit1	205	15.03	3.35
chr3L	4108157	4109950	Ack	254	24.095	3.81

chr3L	4133900	4134584	ens	140	31.1894	5.00
chr3L	4138503	4140096	Rop	257	47.7477	6.39
chr3L	4138503	4140096	Ras64B	178	47.7477	6.39
chr3L	4148585	4149061	CG1299	163	16.6234	4.14
chr3L	4170246	4170800	Ero1L	79	30.4875	5.43
chr3L	4227064	4227616	ImpL2	32	14.7874	3.96
chr3L	4227982	4229078	ImpL2	1580	11.6551	3.21
chr3L	4240049	4240587	CG14997	26	11.2415	3.39
chr3L	4240049	4240587	CR45738	172	11.2415	3.39
chr3L	4253074	4254382	ago	130	26.5345	4.45
chr3L	4282065	4282933	CG11583	209	16.2051	3.93
chr3L	4291986	4292898	CG15012	455	7.84582	3.00
chr3L	4291986	4292898	Fdx2	220	7.84582	3.00
chr3L	4318773	4319366	PMP34	245	19.3485	4.57
chr3L	4363463	4364919	Cip4	60	36.4258	5.08
chr3L	4363463	4364919	mRpS6	507	36.4258	5.08
chr3L	4403247	4403950	Syx17	40	22.0322	4.38
chr3L	4406965	4407288	DOR	44	10.4692	3.08
chr3L	4413849	4414957	DOR	36	25.7973	4.63
chr3L	4625771	4626313	Src64B	2	25.1121	4.36
chr3L	4626638	4627562	HDAC1	511	185.334	14.92
chr3L	4626638	4627562	CR45438	341	185.334	14.92
chr3L	4824410	4826427	Dhc64C	982	89.9665	8.84
chr3L	4824410	4826427	Aats-leu	1147	89.9665	8.84
chr3L	5134183	5134837	CG4603	246	12.1407	3.08
chr3L	5152464	5153091	Srp54k	81	17.3508	3.41
chr3L	5359007	5359481	Klp64D	110	20.9647	3.68
chr3L	5360105	5361083	Cyt-c1	153	46.134	6.34
chr3L	5365404	5367013	Uev1A	216	25.6982	4.61
chr3L	5365404	5367013	Membrin	677	25.6982	4.61
chr3L	5563342	5564199	Sucb	183	16.725	3.65
chr3L	5563342	5564199	Sras	101	16.725	3.65
chr3L	5577054	5577520	Msr-110	51	12.2069	3.20
chr3L	5583075	5584539	l(3)psg2	11	13.5778	3.39
chr3L	5751558	5752531	DnaJ-1	115	18.0765	3.39
chr3L	5752903	5753635	Usp47	96	34.3815	5.18
chr3L	5765010	5766363	CR43603	348	46.0031	6.83
chr3L	5765010	5766363	CG10576	107	46.0031	6.83
chr3L	5765010	5766363	Txl	451	46.0031	6.83
chr3L	5768411	5771962	Ppat-Dpck	491	32.0617	4.64
chr3L	5768411	5771962	scny	163	32.0617	4.64
chr3L	5779938	5780493	Pmi	159	11.1593	3.10
chr3L	5779938	5780493	PGRP-LD	54	11.1593	3.10
chr3L	5809835	5811662	S6k	21	20.833	3.77
chr3L	5809835	5811662	CG5537	20	20.833	3.77
chr3L	5902132	5902707	QC	19	30.659	5.33
chr3L	5923533	5924228	Vap-33B	37	22.3255	4.33
chr3L	5931822	5932900	Rcc1	160	17.4043	3.73
chr3L	6094687	6095429	CG10467	269	21.0657	4.37
chr3L	6094687	6095429	loj	30	21.0657	4.37
chr3L	6192739	6194390	CG13298	617	31.6553	5.45
chr3L	6192739	6194390	Trn	51	31.6553	5.45
chr3L	6218608	6218944	LanA	726	21.7452	5.15
chr3L	6256085	6256849	Best2	27	16.2152	3.83
chr3L	6488040	6489071	CG10147	255	18.3414	4.05
chr3L	6488040	6489071	CG8270	1	18.3414	4.05

chr3L	6494827	6496646	CG10144	437	19.858	3.59
chr3L	6494827	6496646	sfl	55	19.858	3.59
chr3L	6689008	6689335	SP1173	3484	21.6959	4.27
chr3L	6742067	6744120	dikar	102	30.0689	4.14
chr3L	6746867	6747469	velo	40	13.3967	3.49
chr3L	6751874	6753533	velo	475	28.6233	4.79
chr3L	6751874	6753533	CG8549	160	28.6233	4.79
chr3L	6755633	6756616	CG10103	166	15.5333	3.47
chr3L	6964326	6964963	sgl	99	52.0155	6.05
chr3L	7094688	7095188	form3	88	27.0049	5.03
chr3L	7124982	7125683	msl-3	145	22.5335	4.48
chr3L	7133516	7134780	melt	91	17.1671	3.56
chr3L	7189495	7190044	Dscam2	279	12.3339	3.55
chr3L	7238571	7239046	BHD	196	20.175	4.36
chr3L	7240746	7241460	RpL18	72	23.5542	4.29
chr3L	7248737	7249463	Cdc27	117	11.8019	3.28
chr3L	7249980	7250543	MED4	223	29.085	5.04
chr3L	7328115	7329657	CG8602	93	46.9675	6.27
chr3L	7328115	7329657	mus312	382	46.9675	6.27
chr3L	7328115	7329657	CG42307	382	46.9675	6.27
chr3L	7328115	7329657	mus312	382	46.9675	6.27
chr3L	7328115	7329657	CG42307	382	46.9675	6.27
chr3L	7328115	7329657	mus312	382	46.9675	6.27
chr3L	7339403	7340320	lark	209	15.6608	3.22
chr3L	7344663	7345368	CG8596	74	50.3757	7.22
chr3L	7347494	7348728	Srp19	414	39.7681	5.41
chr3L	7347494	7348728	qm	136	39.7681	5.41
chr3L	7358800	7361550	pst	471	106.387	8.28
chr3L	7358800	7361550	Sec63	174	106.387	8.28
chr3L	7369762	7370967	akirin	187	18.4168	3.51
chr3L	7435025	7435530	Rac2	24	11.7751	3.27
chr3L	7510958	7511582	CG7546	179	17.5225	3.55
chr3L	7653849	7654844	CG7492	220	22.1249	4.58
chr3L	7719183	7719731	CG12262	43	30.0537	4.67
chr3L	7809515	7810197	CG32369	256	22.227	4.48
chr3L	7864012	7864444	Pdp1	3124	13.9636	3.30
chr3L	7935588	7936822	syd	182	11.8019	3.28
chr3L	7935588	7936822	Srp9	434	11.8019	3.28
chr3L	7978252	7979436	CG8038	455	27.9025	4.39
chr3L	7978252	7979436	nmo	60	27.9025	4.39
chr3L	8115546	8116995	Arp3	314	25.7144	4.28
chr3L	8115546	8116995	msk	38	25.7144	4.28
chr3L	8127440	8128935	CdsA	454	38.9555	5.40
chr3L	8127440	8128935	Uba2	397	38.9555	5.40
chr3L	8127440	8128935	CdsA	162	38.9555	5.40
chr3L	8177600	8178487	Nmt	263	20.6883	4.74
chr3L	8185377	8187787	ERR	320	35.1798	5.37
chr3L	8185377	8187787	Atg18a	3	35.1798	5.37
chr3L	8191335	8191693	MED24	256	9.72728	3.07
chr3L	8197454	8198573	UbcE2M	3	12.3773	3.41
chr3L	8197454	8198573	CG8005	346	12.3773	3.41
chr3L	8297124	8297672	Rab19	76	13.2179	3.49
chr3L	8298932	8300522	Arl5	149	18.2372	3.52
chr3L	8298932	8300522	CG13671	373	18.2372	3.52
chr3L	8314897	8315582	CG7185	204	64.0809	6.68
chr3L	8345812	8347121	CG7182	376	46.3365	5.99

chr3L	8345812	8347121	GAPcenA	198	46.3365	5.99
chr3L	8395890	8396689	CG7120	81	13.8175	3.98
chr3L	8404745	8405897	mkg-p	214	31.4096	5.47
chr3L	8404745	8405897	CG33057	214	31.4096	5.47
chr3L	8404745	8405897	mkg-p	214	31.4096	5.47
chr3L	8404745	8405897	CG33057	214	31.4096	5.47
chr3L	8404745	8405897	CG13667	390	31.4096	5.47
chr3L	8424797	8425836	Cbl	111	24.3896	4.49
chr3L	8441794	8442540	Unr	1	24.5309	4.46
chr3L	8449736	8450607	Gug	88	39.4226	6.17
chr3L	8525126	8525791	foi	72	22.7821	4.12
chr3L	8534823	8535529	ergic53	125	38.3271	5.30
chr3L	8603687	8604272	CG5989	168	28.4819	4.61
chr3L	8675452	8676308	h	78	22.517	4.21
chr3L	8693467	8694243	CR44526	1215	10.9722	3.23
chr3L	8827398	8827757	dally	35	13.5922	3.58
chr3L	8968423	8969129	CG5026	61	16.8023	3.90
chr3L	8973875	8975561	pix	169	45.3947	6.97
chr3L	8973875	8975561	Srp68	449	45.3947	6.97
chr3L	8984972	8985712	Galk	38	14.1464	3.30
chr3L	8996487	8998741	smg	202	20.3097	3.80
chr3L	8996487	8998741	CG5087	735	20.3097	3.80
chr3L	9072083	9072747	CG4911	147	13.5578	3.13
chr3L	9335641	9336228	PGRP-LA	147	18.2908	4.15
chr3L	9349464	9349866	PGRP-LF	54	26.0427	4.37
chr3L	9357560	9359221	UGP	402	12.8827	3.08
chr3L	9357560	9359221	CG32039	465	12.8827	3.08
chr3L	9360204	9360827	Pdxk	296	14.6662	3.36
chr3L	9366912	9368221	Klp67A	493	30.7884	4.93
chr3L	9366912	9368221	CG4452	163	30.7884	4.93
chr3L	9370463	9371065	Fdx1	137	11.9836	3.28
chr3L	9401314	9402617	eIF-4E	116	13.9288	3.13
chr3L	9409847	9411533	CG4022	242	22.225	3.69
chr3L	9409847	9411533	CG3689	37	22.225	3.69
chr3L	9426041	9427833	CG3967	316	34.281	5.22
chr3L	9426041	9427833	Shc	65	34.281	5.22
chr3L	9428940	9429842	snoRNA:Psi28S-612	114	16.2152	3.83
chr3L	9428940	9429842	RpS17	189	16.2152	3.83
chr3L	9430121	9431088	MTF-1	169	18.642	3.95
chr3L	9441601	9442408	Nf-YA	67	21.9923	4.61
chr3L	9441601	9442408	Bet3	67	21.9923	4.61
chr3L	9446594	9447804	CG3529	240	55.1234	6.25
chr3L	9451235	9452698	CG3448	104	10.9657	3.14
chr3L	9451235	9452698	ghi	407	10.9657	3.14
chr3L	9455426	9456981	phol	411	34.1225	4.99
chr3L	9455426	9456981	CG3552	447	34.1225	4.99
chr3L	9455426	9456981	CR45873	468	34.1225	4.99
chr3L	9476582	9477998	Uch-L5	73	32.9787	5.07
chr3L	9476582	9477998	Jarid2	1	32.9787	5.07
chr3L	9505039	9505398	path	69	20.6778	4.47
chr3L	9508971	9509950	CG3408	277	24.6739	4.44
chr3L	9546816	9547509	CG42673	180	21.3521	3.74
chr3L	9629956	9630650	CG8336	115	37.8858	5.51
chr3L	9643664	9644144	CNMaR	174	12.6775	3.42
chr3L	9694184	9694674	CG6767	61	24.9643	4.39
chr3L	9698098	9698978	Ubc4	171	18.0941	4.16

chr3L	9704230	9705174	CG16711	245	29.752	4.98
chr3L	9713301	9713873	SH3PX1	139	27.4635	4.46
chr3L	9714165	9714965	vsg	44	16.989	3.57
chr3L	9723335	9723894	defl	70	14.9886	3.78
chr3L	9726905	9727236	Naa60	195	15.9487	4.05
chr3L	9729927	9730787	ATPsynB	132	25.1778	4.75
chr3L	9853949	9855407	RasGAP1	548	23.1047	4.17
chr3L	9863841	9865346	iPLA2-VIA	412	19.6858	4.10
chr3L	9863841	9865346	CG8108	123	19.6858	4.10
chr3L	9888113	9889575	Taf2	82	14.85	3.60
chr3L	9888113	9889575	CalpB	160	14.85	3.60
chr3L	9898089	9899696	CG6707	41	18.6029	4.10
chr3L	9898089	9899696	nudE	225	18.6029	4.10
chr3L	9968016	9968700	CG6685	39	17.7005	3.54
chr3L	9968016	9968700	Dronc	200	17.7005	3.54
chr3L	9971718	9973082	CG6674	310	17.4681	3.83
chr3L	9971718	9973082	vnc	53	17.4681	3.83
chr3L	9971718	9973082	CG42455	53	17.4681	3.83
chr3L	9971718	9973082	vnc	53	17.4681	3.83
chr3L	9971718	9973082	CG42455	391	17.4681	3.83
chr3L	9971718	9973082	vnc	391	17.4681	3.83
chr3L	10224780	10225338	scramb1	119	15.0256	3.59
chr3L	10515799	10516257	A2bp1	46	25.9862	5.10
chr3L	10637334	10638677	E(z)	175	13.4156	3.59
chr3L	10637334	10638677	CG8009	441	13.4156	3.59
chr3L	10660468	10661109	CG32066	113	16.7173	3.26
chr3L	10661504	10661876	CG8003	139	13.7753	4.04
chr3L	10664539	10666655	simj	20	34.1839	5.26
chr3L	10683068	10683903	CG11811	7788	14.1207	3.54
chr3L	10775675	10777306	CG43245	4831	10.8635	3.12
chr3L	10857720	10858173	tna	41	22.3363	4.05
chr3L	10867780	10868298	tna	1753	23.5552	4.50
chr3L	11008873	11009783	klu	39	21.8736	4.35
chr3L	11010283	11011148	klu	1535	8.72283	3.01
chr3L	11070314	11071177	Mocs1	95	55.2595	7.26
chr3L	11073845	11074437	l(3)01239	194	18.3864	3.86
chr3L	11113254	11114007	Sod	226	27.1138	4.99
chr3L	11120013	11121307	FoxK	59	46.1145	6.26
chr3L	11120013	11121307	mRpL2	122	46.1145	6.26
chr3L	11122537	11124099	Ufd1-like	121	17.66	4.07
chr3L	11122537	11124099	NaPi-III	10	17.66	4.07
chr3L	11135873	11136427	CG32082	69	23.8306	5.40
chr3L	11169142	11170377	wls	534	14.2378	3.18
chr3L	11169142	11170377	CG7616	86	14.2378	3.18
chr3L	11205751	11206162	Plod	7	30.4405	5.81
chr3L	11209867	11211269	Ube3a	395	23.8415	4.45
chr3L	11209867	11211269	CG7600	193	23.8415	4.45
chr3L	11252109	11252947	scyl	65	40.8563	5.75
chr3L	11431227	11431607	CG6149	4104	14.1387	3.93
chr3L	11487390	11488048	chrb	43	24.5103	4.05
chr3L	11586703	11588168	rt	862	14.6738	3.61
chr3L	11601199	11601688	CG7368	105	10.664	3.46
chr3L	11627891	11628509	CG18815	58	19.8017	4.23
chr3L	11648026	11649026	RIOK1	122	20.6127	4.12
chr3L	11648026	11649026	CG7339	403	20.6127	4.12
chr3L	11681783	11682494	TfIIEalpha	5	24.5856	4.95

chr3L	11683670	11684751	Sugb	270	29.752	4.98
chr3L	11690966	11691277	Pallidin	232	12.0224	3.53
chr3L	11707917	11708628	CG11658	155	47.972	5.99
chr3L	11714980	11715351	CG14132	138	21.0828	4.75
chr3L	11822709	11823465	RpL10Ab	30	20.1634	4.04
chr3L	11822709	11823465	snoRNA:Psi18S-920	103	20.1634	4.04
chr3L	12002451	12003174	Pop2	173	25.4357	4.07
chr3L	12002451	12003174	CG6928	412	25.4357	4.07
chr3L	12118208	12118814	ssp	173	24.5856	4.95
chr3L	12124067	12125341	yps	187	23.2622	4.07
chr3L	12137904	12139335	ND-SGDH	151	21.2264	4.18
chr3L	12137904	12139335	RhoGAP68F	545	21.2264	4.18
chr3L	12145688	12146101	CG5642	250	11.2866	3.21
chr3L	12270844	12271396	Pbgs	119	15.8889	3.97
chr3L	12276899	12278211	CG4300	485	31.1502	5.56
chr3L	12276899	12278211	CG10426	82	31.1502	5.56
chr3L	12408318	12408959	CG32103	4	22.6239	4.14
chr3L	12414145	12414974	CG32103	23	14.4704	3.62
chr3L	12416767	12417643	ArfGAP1	253	16.8406	3.67
chr3L	12467979	12468405	eyg	193	14.8187	3.79
chr3L	12471735	12472161	CG10616	210	17.0699	4.00
chr3L	12489727	12491229	CG10660	322	11.2614	3.21
chr3L	12489727	12491229	CR45681	104	11.2614	3.21
chr3L	12489727	12491229	CG10660	279	11.2614	3.21
chr3L	12500761	12501285	CG10638	309	12.9163	3.48
chr3L	12514479	12516189	sti	379	22.3255	4.33
chr3L	12514479	12516189	CR45168	185	22.3255	4.33
chr3L	12514479	12516189	tral	136	22.3255	4.33
chr3L	12526113	12526827	eIF-2beta	172	56.7985	6.20
chr3L	12533041	12534847	Tsf2	164	15.4794	3.68
chr3L	12533041	12534847	CG34242	435	15.4794	3.68
chr3L	12533041	12534847	CG10688	1029	15.4794	3.68
chr3L	12609289	12609953	caup	148	23.1868	4.55
chr3L	12692813	12693780	mirr	432	26.1078	4.53
chr3L	12734315	12735168	SmD1	75	21.8543	4.10
chr3L	12734315	12735168	Ptp69D	408	21.8543	4.10
chr3L	12750530	12750856	CG10984	190	11.0265	3.08
chr3L	12804395	12805166	Atg1	128	19.7277	3.96
chr3L	12805980	12806586	Sap130	55	18.8931	3.82
chr3L	12814414	12814841	CG42588	432	13.1898	3.51
chr3L	12849501	12850494	CG10948	129	18.1467	3.77
chr3L	12849501	12850494	Wbp2	82	18.1467	3.77
chr3L	13015966	13016580	CG11267	146	17.72	3.93
chr3L	13021643	13022815	AdenoK	129	43.4375	5.68
chr3L	13023205	13023883	RpS12	24	23.603	4.46
chr3L	13023205	13023883	snoRNA:Me28S-A774b	204	23.603	4.46
chr3L	13041572	13042368	RpS4	84	49.0167	5.81
chr3L	13044283	13045415	Syx13	1	28.0815	4.70
chr3L	13114082	13114523	trn	64	19.6858	4.10
chr3L	13442435	13443528	cmb	259	22.8516	5.25
chr3L	13477152	13478546	stv	83	24.1656	4.21
chr3L	13485775	13486363	Abp1	116	28.7239	5.04
chr3L	13494531	13495005	Tgi	785	25.6156	4.89
chr3L	13497557	13498452	Tgi	2276	19.2127	4.01
chr3L	13512713	13513970	Vps36	52	13.94	3.38
chr3L	13512713	13513970	Liprin-beta	193	13.94	3.38

chr3L	13928573	13930216	CG8833	602	34.0434	6.07
chr3L	13928573	13930216	DCTN1-p150	216	34.0434	6.07
chr3L	13938969	13939722	CG32137	54	15.3605	4.10
chr3L	13964283	13964842	Meics	43	13.0305	3.60
chr3L	13965126	13965723	ssp2	276	17.8922	3.61
chr3L	13965126	13965723	Nxf3	276	17.8922	3.61
chr3L	14002025	14003273	upSET	82	26.492	4.18
chr3L	14003573	14006188	upSET	1925	17.604	3.56
chr3L	14028264	14029284	Ptip	455	38.2857	5.83
chr3L	14031047	14031826	endos	265	15.9328	3.63
chr3L	14031047	14031826	CG6650	183	15.9328	3.63
chr3L	14031047	14031826	endos	74	15.9328	3.63
chr3L	14036892	14038559	Hsc70Cb	91	17.5907	3.29
chr3L	14050428	14051787	CG6833	289	27.2872	4.67
chr3L	14052431	14053580	CG13484	212	42.6974	5.62
chr3L	14052431	14053580	CG32138	77	42.6974	5.62
chr3L	14071695	14072558	Pex1	47	16.8125	3.58
chr3L	14190246	14191648	nuf	21	25.3185	4.50
chr3L	14274073	14274657	fz	41	18.8362	4.26
chr3L	14427161	14427638	bbg	34	12.261	3.13
chr3L	14537461	14538491	Mpcp	16	27.5782	4.83
chr3L	14552726	14552996	Gbs-70E	1864	10.6523	3.40
chr3L	14622728	14623435	CG9628	128	11.5271	3.22
chr3L	14624394	14625259	CG9628	1751	12.3257	3.35
chr3L	14706514	14707012	ome	58	46.9461	6.33
chr3L	14785435	14787467	bmm	107	22.324	3.85
chr3L	14785435	14787467	Tdrd3	485	22.324	3.85
chr3L	14789835	14791080	CG9425	297	13.7431	3.13
chr3L	14801566	14802314	NHP2	193	26.8929	5.14
chr3L	14804644	14805627	CG17839	249	38.9571	6.02
chr3L	14819628	14819959	CG17839	14615	11.9299	3.74
chr3L	15093908	15095553	sstn	571	12.9588	3.03
chr3L	15093908	15095553	Plp	566	12.9588	3.03
chr3L	15093908	15095553	Aats-gly	149	12.9588	3.03
chr3L	15141259	15142753	CR45396	378	46.2899	6.04
chr3L	15141259	15142753	Pdi	84	46.2899	6.04
chr3L	15143546	15144853	CR45397	705	51.0415	6.94
chr3L	15143546	15144853	CG32147	446	51.0415	6.94
chr3L	15143546	15144853	Pex3	151	51.0415	6.94
chr3L	15162971	15163704	CG7011	181	22.6738	4.45
chr3L	15498844	15499597	CG7275	410	9.6395	3.14
chr3L	15500794	15501696	CG7272	35	23.1053	3.65
chr3L	15554074	15554591	AGO2	254	23.1356	3.72
chr3L	15579874	15581306	mrn	62	15.5555	3.40
chr3L	15579874	15581306	CG12301	324	15.5555	3.40
chr3L	15588744	15589441	RhoGAP71E	115	43.8895	5.98
chr3L	15609891	15610339	CG7650	264	13.4156	3.59
chr3L	15825428	15826489	DCP2	136	14.5117	3.29
chr3L	15826858	15827259	dbo	132	22.3255	4.33
chr3L	15832172	15832792	CG12713	133	16.1079	3.62
chr3L	15835154	15835451	CG18081	51	13.1236	3.93
chr3L	15956555	15957215	elgi	142	27.6085	4.43
chr3L	15967389	15968700	l(3)72Ab	456	22.8075	4.37
chr3L	15967389	15968700	CG10516	125	22.8075	4.37
chr3L	15976900	15977444	CG17029	96	12.5105	3.54
chr3L	15982252	15983510	brm	78	22.7768	4.26

chr3L	15982252	15983510	Arl1	187	22.7768	4.26
chr3L	15988145	15988841	Hip14	228	20.3801	4.40
chr3L	16001039	16002316	CG5830	3442	9.98231	3.01
chr3L	16011267	16011937	mib1	217	40.15	5.26
chr3L	16023329	16023630	Notum	24	15.3316	3.75
chr3L	16027053	16027642	mir-4941	3713	11.7823	3.37
chr3L	16045958	16047339	Diap1	46	20.6154	4.22
chr3L	16049766	16051134	Diap1	155	26.5363	4.88
chr3L	16087035	16087619	Tasp1	144	31.1502	4.83
chr3L	16090350	16091054	CG5235	258	14.113	3.45
chr3L	16090350	16091054	ClC-c	195	14.113	3.45
chr3L	16113141	16114382	Taf4	206	48.0787	5.90
chr3L	16129535	16130076	SsRbeta	98	32.9295	5.48
chr3L	16228146	16228823	l(3)72Dn	270	21.067	4.61
chr3L	16231538	16232605	PDCD-5	65	21.8502	4.40
chr3L	16231538	16232605	MED10	20	21.8502	4.40
chr3L	16377787	16378706	roq	384	19.5296	4.12
chr3L	16385351	16385977	CG4729	128	20.9293	4.08
chr3L	16408631	16409552	fax	718	14.9922	3.45
chr3L	16409939	16410700	fax	1247	10.1972	3.14
chr3L	16410984	16411938	fax	19	14.5117	3.29
chr3L	16418825	16419380	Aats-tyr	99	17.5769	3.71
chr3L	16483407	16483821	aos	40	16.3829	3.81
chr3L	16572116	16572553	Mipp1	16	14.8323	3.51
chr3L	16582978	16583901	Rpn12	209	24.1656	4.21
chr3L	16584214	16584864	UQCR-C2	278	21.8012	4.40
chr3L	16588099	16589187	l(3)73Ah	153	13.3304	3.03
chr3L	16590766	16591169	tra	13	10.2836	3.08
chr3L	16611560	16612876	Su(P)	218	23.8086	4.73
chr3L	16611560	16612876	CG4101	504	23.8086	4.73
chr3L	16611560	16612876	CR45865	846	23.8086	4.73
chr3L	16660052	16661785	Baldspot	70	30.0491	4.25
chr3L	16713512	16714429	Dab	531	16.4711	3.93
chr3L	16765653	16766275	Nrt	34	17.8061	3.80
chr3L	16780049	16780848	CG9705	114	25.9862	5.10
chr3L	16786640	16787114	Int6	107	17.191	3.36
chr3L	16808173	16809062	nudC	142	19.402	4.00
chr3L	16845343	16845949	CG9715	264	32.8528	5.54
chr3L	16890925	16891463	Lmpt	48	19.27	4.07
chr3L	16986459	16987008	Exn	18	21.7548	4.63
chr3L	17010030	17010575	CG3764	2283	14.113	3.45
chr3L	17010980	17012736	CG3764	82	28.946	4.96
chr3L	17015543	17016130	CG6664	131	32.4667	5.27
chr3L	17016513	17016955	CG7728	170	26.201	4.10
chr3L	17035661	17036002	beg	146	10.1684	3.01
chr3L	17042180	17043089	scaf6	90	12.3631	3.51
chr3L	17042180	17043089	Papst2	312	12.3631	3.51
chr3L	17051111	17051957	rogdi	95	15.2459	3.28
chr3L	17474881	17476663	UQCR-Q	160	32.2254	5.49
chr3L	17474881	17476663	CG34250	1013	32.2254	5.49
chr3L	17513433	17514741	U4-U6-60K	255	17.2105	3.73
chr3L	17513433	17514741	CG7564	640	17.2105	3.73
chr3L	17532810	17534112	Nedd4	4	19.3273	3.61
chr3L	17557062	17557865	CycT	403	22.4593	3.90
chr3L	17621120	17622224	Vps60	259	25.8948	4.39
chr3L	17621120	17622224	CG7510	61	25.8948	4.39

chr3L	17644073	17645129	Pep	577	39.8758	5.84
chr3L	17653138	17654400	Krn	112	24.5103	4.05
chr3L	17653138	17654400	Ndfip	112	24.5103	4.05
chr3L	17667854	17669357	snmRNA:641	398	20.392	3.90
chr3L	17667854	17669357	Crtc	106	20.392	3.90
chr3L	17841148	17841662	CG5577	47	14.6738	3.61
chr3L	17845808	17846658	MED19	146	55.383	5.97
chr3L	17846954	17847739	CG7430	161	27.9181	5.01
chr3L	17849672	17850292	CG7441	43	11.1149	3.36
chr3L	17895565	17896008	Tsp74F	61	13.933	3.46
chr3L	17907520	17908645	Prestin	47	31.9551	5.13
chr3L	17907520	17908645	CG14353	377	31.9551	5.13
chr3L	17961423	17961952	Eip75B	163	26.3411	4.33
chr3L	18105527	18107147	CG13698	56	70.1723	7.35
chr3L	18105527	18107147	mRpS26	485	70.1723	7.35
chr3L	18139091	18139952	geko	39	10.2553	3.47
chr3L	18185430	18186303	hid	29	21.9846	3.87
chr3L	18621079	18622203	not	195	20.2246	3.91
chr3L	18625867	18626951	MYPT-75D	54	22.372	4.83
chr3L	18672229	18673409	Capr	459	48.5993	6.27
chr3L	18800010	18801671	ftz-f1	28	16.827	3.37
chr3L	18800010	18801671	CR45939	25	16.827	3.37
chr3L	18822507	18823029	Cat	147	24.478	4.17
chr3L	18865883	18866690	CR45912	314	14.113	3.45
chr3L	18865883	18866690	CG14074	197	14.113	3.45
chr3L	18890112	18891341	CG3961	73	16.8917	3.77
chr3L	18897933	18898438	CG3902	103	15.2023	3.58
chr3L	18898725	18899790	RpL26	105	16.7783	4.24
chr3L	18904205	18904859	CSN1b	281	15.3824	4.04
chr3L	18905387	18905924	CG6841	173	31.4721	5.35
chr3L	18995155	18995935	CG18135	44	13.2295	3.66
chr3L	19015854	19016544	CG18136	13088	14.4751	3.52
chr3L	19044993	19045623	nkd	50	22.4983	4.03
chr3L	19062051	19063714	CG3797	384	18.3564	3.68
chr3L	19062051	19063714	CG6812	36	18.3564	3.68
chr3L	19086033	19086571	Mkp3	41	17.5402	3.63
chr3L	19170049	19170770	fz2	81	15.733	3.55
chr3L	19260108	19260770	nes	233	27.5782	4.83
chr3L	19261228	19262006	Bet1	224	16.6352	3.91
chr3L	19263585	19264810	CG42374	137	24.0358	4.38
chr3L	19263585	19264810	CG9666	137	24.0358	4.38
chr3L	19263585	19264810	Max	282	24.0358	4.38
chr3L	19263585	19264810	CG9666	501	24.0358	4.38
chr3L	19263585	19264810	CG42374	501	24.0358	4.38
chr3L	19294281	19295218	fal	64	34.2199	5.79
chr3L	19294281	19295218	CR46048	368	34.2199	5.79
chr3L	19551823	19552855	Ltn1	83	44.0519	5.82
chr3L	19551823	19552855	CG9300	212	44.0519	5.82
chr3L	19562203	19563533	CR45916	75	13.6828	3.18
chr3L	19585413	19586546	CG9231	296	14.499	3.62
chr3L	19585413	19586546	CG9330	47	14.499	3.62
chr3L	19589931	19590764	CG14100	80	16.1486	3.72
chr3L	19598342	19599501	ash1	209	18.2322	4.03
chr3L	19598342	19599501	Taf6	541	18.2322	4.03
chr3L	19618033	19618557	l(3)76BDm	197	29.3222	4.47
chr3L	19635252	19636010	wnd	49	37.8033	4.94

chr3L	19686937	19687677	CG8765	20	19.0643	4.19
chr3L	19686937	19687677	Gbeta76C	303	19.0643	4.19
chr3L	19793036	19794464	Gyc76C	97	18.4168	3.51
chr3L	19793036	19794464	CG42637	97	18.4168	3.51
chr3L	19793036	19794464	SREBP	462	18.4168	3.51
chr3L	19833367	19835109	trc	78	34.6369	5.46
chr3L	19833367	19835109	CG32221	443	34.6369	5.46
chr3L	19870785	19871953	Rab8	189	21.4687	3.91
chr3L	19870785	19871953	Usp32	129	21.4687	3.91
chr3L	19908592	19910910	CR43889	581	17.3508	3.41
chr3L	19908592	19910910	Su(Tpl)	535	17.3508	3.41
chr3L	19908592	19910910	Mi-2	270	17.3508	3.41
chr3L	19908592	19910910	Prp3	354	17.3508	3.41
chr3L	19914853	19915766	Rpn1	265	20.5222	4.06
chr3L	19922785	19924093	Su(z)12	328	22.2457	4.22
chr3L	19922785	19924093	CG7770	536	22.2457	4.22
chr3L	19924841	19925276	Grasp65	135	39.2229	6.21
chr3L	19939094	19939372	Ac76E	81	13.5778	3.39
chr3L	20001079	20001644	Tom20	239	23.1394	4.66
chr3L	20198030	20198434	CG42674	111	26.8798	4.82
chr3L	20242484	20243111	Clc	267	18.3175	3.76
chr3L	20301759	20302872	Pex23	471	42.6024	5.66
chr3L	20301759	20302872	CG6597	182	42.6024	5.66
chr3L	20316870	20317581	Spn77Ba	92	16.4431	3.59
chr3L	20316870	20317581	CR43875	2	16.4431	3.59
chr3L	20347111	20349380	Rbbp5	543	30.2321	5.13
chr3L	20347111	20349380	eRF1	131	30.2321	5.13
chr3L	20353692	20354359	CG5618	258	14.3736	3.80
chr3L	20367023	20367616	in	69	10.2525	3.20
chr3L	20375392	20376698	Ide	540	31.1484	5.11
chr3L	20375392	20376698	RhoBTB	195	31.1484	5.11
chr3L	20401325	20401710	trbl	89	14.0011	3.24
chr3L	20427224	20427712	CG5274	53	13.6339	3.69
chr3L	20432127	20432951	Las	422	14.8323	3.51
chr3L	20432127	20432951	Psn	145	14.8323	3.51
chr3L	20452363	20453531	CG5199	440	11.7823	3.37
chr3L	20452363	20453531	Uhg8	12	11.7823	3.37
chr3L	20472647	20473487	CG32428	109	15.2976	3.08
chr3L	20473766	20475438	CG5969	197	17.5561	3.94
chr3L	20473766	20475438	isoQC	486	17.5561	3.94
chr3L	20525584	20526889	CG4825	178	30.571	5.38
chr3L	20525584	20526889	CG4858	579	30.571	5.38
chr3L	20770885	20772018	CG4042	144	44.9986	5.74
chr3L	20770885	20772018	Pitslre	124	44.9986	5.74
chr3L	20777844	20778509	Pex16	137	14.1132	3.80
chr3L	20785132	20786360	CG11399	149	33.2559	4.76
chr3L	20787761	20788267	CG11396	37	14.3011	3.90
chr3L	20808825	20810191	CG3634	117	23.3237	4.51
chr3L	20808825	20810191	HIPP1	538	23.3237	4.51
chr3L	20841034	20841713	CSN3	85	21.0719	4.21
chr3L	20949029	20950115	fng	40	21.1032	4.37
chr3L	21025702	21027516	skd	254	15.8437	3.85
chr3L	21027797	21028538	CG10585	129	37.683	4.97
chr3L	21058588	21059007	siz	26	14.2988	3.12
chr3L	21135588	21136173	CG10565	207	25.1125	4.10
chr3L	21175432	21176821	chb	142	40.5164	6.15

chr3L	21202298	21202959	CG33054	167	12.3743	3.19
chr3L	21202298	21202959	CG33056	167	12.3743	3.19
chr3L	21202298	21202959	CG10512	204	12.3743	3.19
chr3L	21216471	21217251	CG12975	50	25.0089	4.77
chr3L	21216471	21217251	Ilk	111	25.0089	4.77
chr3L	21279720	21280132	AcCoAS	48	17.6368	3.24
chr3L	21317470	21318172	Pc	44	17.8796	3.53
chr3L	21342931	21343816	CG7324	150	23.2622	4.07
chr3L	21382355	21382807	rgn	62	38.9571	6.02
chr3L	21432735	21434303	barc	521	22.7835	4.41
chr3L	21432735	21434303	Aef1	217	22.7835	4.41
chr3L	21497185	21497605	Hr78	49	19.7277	3.96
chr3L	21497185	21497605	CR43933	91	19.7277	3.96
chr3L	21507267	21508213	Glg1	58	20.0863	3.98
chr3L	21507267	21508213	M6	371	20.0863	3.98
chr3L	21536952	21537778	Rpn10	171	28.5549	5.27
chr3L	21538974	21540394	COX8	148	33.8965	5.05
chr3L	21538974	21540394	VhaM9.7-b	562	33.8965	5.05
chr3L	21554094	21555088	Wnk	76	13.0377	3.41
chr3L	21568335	21568971	CRIF	192	16.9672	3.88
chr3L	21626252	21626690	CG11306	201	22.1747	4.85
chr3L	21635261	21635759	ORMDL	142	15.9328	3.63
chr3L	21679202	21679796	Als2	158	12.6879	3.34
chr3L	21821920	21822283	CycH	130	14.3784	3.75
chr3L	21824658	21825794	CG7414	171	20.4553	4.14
chr3L	21832587	21833373	Nopp140	28	27.6043	5.15
chr3L	21840735	21841472	P5CDh1	45	26.6451	4.29
chr3L	21879490	21880777	mub	111	29.5476	4.54
chr3L	22070925	22071940	CG7139	96	16.9964	3.65
chr3L	22262181	22262989	Rich	144	27.5071	5.20
chr3L	22266140	22266786	CG11523	107	25.9862	5.10
chr3L	22267209	22267963	Csp	158	21.7012	4.22
chr3L	22287618	22288240	Hem	161	15.2023	3.58
chr3L	22288501	22289566	Aats-ile	61	34.0473	6.07
chr3L	22740898	22742571	l(3)04053	481	45.2205	6.06
chr3L	22740898	22742571	CG7369	77	45.2205	6.06
chr3L	22863575	22865155	CG33169	14	38.0523	5.42
chr3L	22863575	22865155	CG33170	750	38.0523	5.42
chr3L	22870775	22872264	Arf79F	158	16.4922	3.31
chr3L	22870775	22872264	CR45969	567	16.4922	3.31
chr3L	22870775	22872264	Arf79F	807	16.4922	3.31
chr3L	22870775	22872264	CG11109	954	16.4922	3.31
chr3L	22870775	22872264	Arf79F	986	16.4922	3.31
chr3L	22870775	22872264	CG11109	1012	16.4922	3.31
chr3L	25216937	25217361	UQCR-11	96335	12.0224	3.20
chr3R	4130250	4130952	Gfat1	67	11.3549	3.65
chr3R	4174386	4176171	CG12581	42	11.8909	3.14
chr3R	4233011	4233461	abs	217	14.499	3.62
chr3R	4311495	4311944	CG9791	102	19.6858	4.10
chr3R	4319454	4320179	CG9795	113	17.3106	3.75
chr3R	4333472	4334208	CG9776	146	18.9722	3.92
chr3R	4378724	4379502	CG11739	64	42.4039	5.10
chr3R	4397341	4397897	CG9855	246	18.5168	4.38
chr3R	4399283	4400039	CG9853	190	28.6173	5.08
chr3R	4400417	4401166	CG14647	103	12.5864	3.03
chr3R	4402910	4404279	CR45187	253	47.3611	5.41

chr3R	4402910	4404279	lost	106	47.3611	5.41
chr3R	4428546	4429506	srl	129	29.1296	4.35
chr3R	4433572	4434225	eIF3-S10	75	33.0896	5.03
chr3R	4433572	4434225	CG1074	144	33.0896	5.03
chr3R	4436757	4437801	CG9804	206	11.8909	3.14
chr3R	4436757	4437801	CG14650	149	11.8909	3.14
chr3R	4470938	4471424	CG31523	51	17.7143	3.83
chr3R	4484997	4485422	Fip1	218	21.3712	4.82
chr3R	4649300	4650552	Karybeta3	287	29.8249	4.54
chr3R	4659415	4660411	CG43427	99	37.361	4.68
chr3R	4713751	4714529	CG9769	111	24.7707	4.61
chr3R	4739142	4739886	tacc	41	19.0499	4.03
chr3R	4811633	4812260	ctrip	90	14.9783	3.17
chr3R	4811633	4812260	Hus1-like	90	14.9783	3.17
chr3R	4811633	4812260	ctrip	90	14.9783	3.17
chr3R	4811633	4812260	Hus1-like	90	14.9783	3.17
chr3R	4813236	4813700	CG1129	98	16.3088	3.32
chr3R	4820642	4821509	CG14657	197	31.7094	4.92
chr3R	4852191	4852862	opa	361	29.4106	4.61
chr3R	4910420	4911457	Cdep	84	31.4773	4.43
chr3R	4951669	4952897	Ubc6	3	19.1506	3.62
chr3R	4955017	4955729	CG14661	31	23.4571	4.72
chr3R	5086403	5087403	corto	113	36.0295	5.28
chr3R	5216180	5217667	cno	50	33.6668	5.55
chr3R	5222557	5223482	CG2604	72	24.9643	4.39
chr3R	5231311	5233041	Kat60	140	42.9923	6.09
chr3R	5231311	5233041	Mms19	473	42.9923	6.09
chr3R	5251157	5251884	CG12163	222	26.0427	4.37
chr3R	5355079	5355586	CG1161	241	20.4879	4.00
chr3R	5357543	5358272	Prosbeta7	142	16.9818	3.49
chr3R	5367536	5368448	Cerk	82	21.2353	3.83
chr3R	5371616	5372245	RpII18	46	25.6037	4.44
chr3R	5463292	5463643	PEK	344	14.9618	3.82
chr3R	5463968	5464781	PEK	167	19.0643	4.19
chr3R	5466635	5467191	RpL35A	189	19.496	4.12
chr3R	5516536	5517710	Itp-r83A	160	24.6739	4.04
chr3R	5547091	5547944	CG2519	246	42.9571	6.15
chr3R	5564364	5565427	CG14671	183	20.4414	3.71
chr3R	5564364	5565427	CG12746	134	20.4414	3.71
chr3R	5567945	5569693	CG2931	251	14.8243	3.59
chr3R	5567945	5569693	Rheb	556	14.8243	3.59
chr3R	5587579	5588503	CG2926	259	36.3471	5.44
chr3R	5590960	5592437	noi	124	21.2264	4.18
chr3R	5590960	5592437	Vha26	427	21.2264	4.18
chr3R	5599147	5602054	kra	98	31.9333	4.85
chr3R	5599147	5602054	CG1427	394	31.9333	4.85
chr3R	5623087	5625068	CG2911	1498	33.3852	4.74
chr3R	5623087	5625068	Spec2	524	33.3852	4.74
chr3R	5623087	5625068	CG2911	524	33.3852	4.74
chr3R	5623087	5625068	RpL13A	176	33.3852	4.74
chr3R	5646746	5649828	MTA1-like	254	30.1024	4.87
chr3R	5646746	5649828	Sec23	580	30.1024	4.87
chr3R	5653946	5655651	elm	4	28.8775	4.73
chr3R	5653946	5655651	MED27	281	28.8775	4.73
chr3R	5660349	5661260	CG1109	364	18.8931	3.82
chr3R	5668672	5671189	Xe7	488	16.7173	3.26

chr3R	5668672	5671189	Atg17	110	16.7173	3.26
chr3R	5736721	5737040	CG1236	120	14.4751	3.52
chr3R	5741805	5743485	Sym	292	22.4665	4.16
chr3R	5741805	5743485	Madm	259	22.4665	4.16
chr3R	5758266	5759157	Sec8	194	33.096	5.38
chr3R	5778816	5780095	jagn	235	23.5522	4.16
chr3R	6007387	6008751	Rm62	149	33.6771	4.87
chr3R	6345259	6346466	SmD2	169	17.8229	4.16
chr3R	6345259	6346466	CG18048	368	17.8229	4.16
chr3R	6367592	6368334	CG42724	128	16.1486	3.72
chr3R	6368902	6369398	CG10286	128	24.577	3.95
chr3R	6382834	6383657	godzilla	232	28.0294	4.97
chr3R	6405161	6406058	Dmtn	110	29.4318	4.20
chr3R	6646778	6647217	Taf1	159	28.8775	4.73
chr3R	6657080	6657491	CG1307	228	9.43592	3.35
chr3R	6657791	6658336	twr	227	14.4428	3.76
chr3R	7066732	7067802	CG1105	452	19.1732	3.70
chr3R	7066732	7067802	CG1965	188	19.1732	3.70
chr3R	7073887	7075658	CG1104	178	21.5745	4.01
chr3R	7073887	7075658	CG1943	584	21.5745	4.01
chr3R	7073887	7075658	CR45908	1213	21.5745	4.01
chr3R	7079746	7080425	Ref1	284	36.4986	5.72
chr3R	7084994	7087817	Tailor	1154	51.5374	5.79
chr3R	7084994	7087817	CR45907	1058	51.5374	5.79
chr3R	7084994	7087817	Tailor	634	51.5374	5.79
chr3R	7084994	7087817	alphaTub84B	119	51.5374	5.79
chr3R	7123459	7125086	Alh	192	37.8062	5.56
chr3R	7123459	7125086	CG10098	554	37.8062	5.56
chr3R	7185653	7187596	CG10055	208	42.3901	5.37
chr3R	7185653	7187596	lap	528	42.3901	5.37
chr3R	7474540	7475038	snRNA:7SK	228	29.0081	4.70
chr3R	7491236	7491858	alpha-Est10	36	42.9923	6.09
chr3R	7515166	7516118	alpha-Est8	42	16.4711	3.93
chr3R	7760910	7761396	Sp7	65	18.1543	4.01
chr3R	7911320	7911830	Prat	152	13.1443	3.03
chr3R	7912931	7913326	CG2846	193	13.94	3.38
chr3R	8001272	8002417	Kmn2	272	11.7057	3.14
chr3R	8001272	8002417	CG2698	20	11.7057	3.14
chr3R	8029941	8031318	CG10903	237	21.3939	4.26
chr3R	8029941	8031318	CG2747	143	21.3939	4.26
chr3R	8029941	8031318	CG10903	179	21.3939	4.26
chr3R	8032612	8033784	CG11035	409	11.7057	3.14
chr3R	8032612	8033784	unc-45	118	11.7057	3.14
chr3R	8125898	8127383	CG9667	350	32.5623	5.23
chr3R	8125898	8127383	Gie	103	32.5623	5.23
chr3R	8240267	8240631	Ada2b	86	14.2928	3.44
chr3R	8243715	8244584	CG9636	428	14.3111	3.85
chr3R	8243715	8244584	CG33722	166	14.3111	3.85
chr3R	8243715	8244584	CG18749	166	14.3111	3.85
chr3R	8243715	8244584	CG33722	166	14.3111	3.85
chr3R	8243715	8244584	CG18749	166	14.3111	3.85
chr3R	8243715	8244584	CG33722	73	14.3111	3.85
chr3R	8301294	8301671	mRpL1	3	10.7872	3.23
chr3R	8313107	8313690	eIF4AIII	180	18.6368	4.16
chr3R	8356828	8358212	stck	104	12.2801	3.10
chr3R	8517735	8518295	CG11737	100	12.261	3.13

chr3R	8652328	8653601	Dhod	298	20.8513	4.27
chr3R	8652328	8653601	CG11753	277	20.8513	4.27
chr3R	8652328	8653601	Dhod	181	20.8513	4.27
chr3R	8652328	8653601	CG11753	87	20.8513	4.27
chr3R	8659451	8660696	bel	59	27.6913	4.59
chr3R	8663405	8664895	p	84	11.9822	3.12
chr3R	8663405	8664895	CG8032	299	11.9822	3.12
chr3R	8669086	8670221	CG8036	43	58.4172	6.25
chr3R	8732037	8732579	CG8202	111	12.0756	3.13
chr3R	8746955	8748374	CG9801	23	24.9526	4.12
chr3R	8746955	8748374	CG8223	145	24.9526	4.12
chr3R	8801380	8802061	Tcp-1eta	212	14.6522	3.43
chr3R	8819765	8820934	CG9821	33	37.157	5.46
chr3R	8819765	8820934	CR43130	120	37.157	5.46
chr3R	8823491	8824955	IscU	146	17.9766	3.62
chr3R	8823491	8824955	CG8379	379	17.9766	3.62
chr3R	8846139	8846497	pyd	45	12.0756	3.13
chr3R	8931634	8932130	pyd	42	41.0683	5.60
chr3R	8983668	8984293	RagA-B	245	12.4986	3.43
chr3R	8984645	8985353	CG11970	76	16.4711	3.93
chr3R	8992436	8993597	E(var)3-9	389	16.4459	3.52
chr3R	8992436	8993597	CG11975	86	16.4459	3.52
chr3R	9013507	9015344	CG11984	103	32.1788	5.11
chr3R	9013507	9015344	CG11985	1412	32.1788	5.11
chr3R	9019420	9020116	tgo	176	33.7854	4.66
chr3R	9052365	9054936	Kdm2	29	19.8808	3.66
chr3R	9064176	9065121	beag	152	16.9799	3.93
chr3R	9260574	9261246	VhaM8.9	158	24.3896	4.49
chr3R	9266313	9267653	Aats-trp	232	14.1853	3.56
chr3R	9266313	9267653	Vps16A	118	14.1853	3.56
chr3R	9334419	9335680	CG8866	466	18.9129	4.22
chr3R	9334419	9335680	CG8121	11	18.9129	4.22
chr3R	9400404	9401584	RpL34b	83	49.3511	6.51
chr3R	9400404	9401584	CG8132	391	49.3511	6.51
chr3R	9502125	9502673	RhoL	120	12.9588	3.03
chr3R	9509778	9510480	rump	329	16.615	3.54
chr3R	9515383	9516620	mRpL47	145	21.4013	4.12
chr3R	9515383	9516620	CG8176	47	21.4013	4.12
chr3R	9515383	9516620	JHDM2	47	21.4013	4.12
chr3R	9515383	9516620	CG8176	47	21.4013	4.12
chr3R	9532117	9533446	by	66	40.8658	6.14
chr3R	9550938	9552511	mura	299	24.2448	3.98
chr3R	9550938	9552511	CR42549	111	24.2448	3.98
chr3R	9550938	9552511	mura	169	24.2448	3.98
chr3R	9550938	9552511	CR42549	169	24.2448	3.98
chr3R	9550938	9552511	RnpS1	396	24.2448	3.98
chr3R	9559143	9560567	AP-1mu	421	48.6978	6.10
chr3R	9559143	9560567	MBD-like	187	48.6978	6.10
chr3R	9562913	9564649	CG9393	63	19.8434	4.08
chr3R	9562913	9564649	Vps45	313	19.8434	4.08
chr3R	9573236	9574282	CG9399	77	42.6828	5.27
chr3R	9577127	9578527	Kap-alpha3	15	23.6052	4.21
chr3R	9577127	9578527	CG8273	323	23.6052	4.21
chr3R	9590558	9591645	bocks	202	28.2367	4.48
chr3R	9590558	9591645	CG8312	508	28.2367	4.48
chr3R	9629720	9631125	Calr	59	54.5095	6.01

chr3R	9631562	9632350	SpdS	26	29.9385	5.47
chr3R	9636689	9637693	Scm	74	16.8561	4.19
chr3R	9677732	9678589	CG16817	245	31.3656	5.03
chr3R	9704104	9705722	Unc-115a	45	35.8458	4.99
chr3R	9710680	9711538	trbd	81	14.6954	3.29
chr3R	9710680	9711538	dmt	185	14.6954	3.29
chr3R	9746244	9747686	CG34409	971	39.0009	5.95
chr3R	9746244	9747686	CG12948	168	39.0009	5.95
chr3R	9762411	9763268	Snap24	214	28.0095	5.23
chr3R	9767362	9768506	MED6	91	30.3917	5.22
chr3R	9767362	9768506	CG8481	73	30.3917	5.22
chr3R	9767362	9768506	MED6	204	30.3917	5.22
chr3R	9777737	9778673	RpS29	214	22.4983	4.03
chr3R	9777737	9778673	snoRNA:Psi18S-1275	87	22.4983	4.03
chr3R	9808478	9809540	FBXO11	295	31.7402	5.40
chr3R	9928568	9929376	Glut4EF	22	48.3256	6.35
chr3R	9932065	9933732	Glut4EF	3713	26.0427	4.37
chr3R	9946326	9947030	Glut4EF	17919	19.7343	4.16
chr3R	9949778	9950402	Glut4EF	21072	18.6024	3.57
chr3R	10042347	10043029	Mical	15	29.752	4.98
chr3R	10144514	10145133	Invadolysin	88	42.3045	4.92
chr3R	10170203	10170830	Best1	42	19.6243	3.86
chr3R	10189353	10189894	CG12814	26	14.8797	3.28
chr3R	10264064	10264880	CG43143	69	35.9049	4.78
chr3R	10330818	10331831	Bruce	149	51.7897	6.49
chr3R	10330818	10331831	CG12818	376	51.7897	6.49
chr3R	10349878	10350855	jumu	67	23.7056	4.27
chr3R	10388205	10389382	cwo	119	17.0617	3.75
chr3R	10760591	10761858	MED7	446	19.3567	3.69
chr3R	10760591	10761858	Cap-H2	169	19.3567	3.69
chr3R	10760591	10761858	CR45016	319	19.3567	3.69
chr3R	10787711	10788576	Art1	231	9.10851	3.11
chr3R	10787711	10788576	Mcm5	528	9.10851	3.11
chr3R	10854154	10854954	CG6567	185	11.6869	3.13
chr3R	10854154	10854954	CG4511	234	11.6869	3.13
chr3R	10862631	10863715	CG6574	54	18.1467	3.77
chr3R	10868051	10869552	SelR	183	26.8679	4.76
chr3R	10868051	10869552	Tsp86D	118	26.8679	4.76
chr3R	10881748	10882476	Tpc1	135	26.5433	5.07
chr3R	10888822	10889492	Adk3	82	25.6342	4.35
chr3R	11157049	11157367	Ugt86Da	104	13.4156	3.59
chr3R	11209166	11210902	SdhC	472	21.0064	3.76
chr3R	11209166	11210902	Tctp	75	21.0064	3.76
chr3R	11215508	11217089	RpS25	135	23.1018	4.08
chr3R	11215508	11217089	CG4820	382	23.1018	4.08
chr3R	11220859	11222459	CG6693	490	36.522	5.35
chr3R	11220859	11222459	RpL3	236	36.522	5.35
chr3R	11220859	11222459	snoRNA:Me18S-C109	275	36.522	5.35
chr3R	11234483	11236478	Arfip	496	12.3947	3.32
chr3R	11234483	11236478	CG17721	127	12.3947	3.32
chr3R	11234483	11236478	CG31441	776	12.3947	3.32
chr3R	11243443	11245011	CG17734	209	16.0879	3.45
chr3R	11243443	11245011	CG5214	511	16.0879	3.45
chr3R	11407862	11408255	mRpL40	120	30.8654	4.69
chr3R	11409439	11410495	mgr	168	17.5561	3.94
chr3R	11409439	11410495	Irbp	339	17.5561	3.94

chr3R	11430854	11432350	RpL24-like	187	37.8066	5.18
chr3R	11430854	11432350	CG5276	342	37.8066	5.18
chr3R	11435109	11436440	CG6744	63	12.393	3.46
chr3R	11435109	11436440	CG5281	305	12.393	3.46
chr3R	11563016	11563513	sea	56	15.7576	3.64
chr3R	11566653	11567186	fabp	133	29.9359	4.70
chr3R	11566653	11567186	sea	133	29.9359	4.70
chr3R	11581312	11582623	wkd	256	26.7784	4.81
chr3R	11589651	11590331	CG6791	118	18.4532	4.07
chr3R	11619595	11620533	Jupiter	65	17.7161	3.62
chr3R	11619595	11620533	CG14710	248	17.7161	3.62
chr3R	11622991	11623752	CG6808	407	17.0664	3.80
chr3R	11622991	11623752	CG14711	166	17.0664	3.80
chr3R	11628529	11629147	CG14712	128	14.3728	3.60
chr3R	11655917	11656449	CG42327	37	19.2515	3.68
chr3R	11669745	11670273	CG14715	4105	13.2485	3.71
chr3R	11758944	11760182	Lk6	619	24.8825	4.52
chr3R	11763401	11764744	Lk6	211	28.1953	4.62
chr3R	11777878	11779177	l(3)neo38	56	82.9838	8.26
chr3R	11786341	11787477	CG17360	248	16.1055	3.96
chr3R	11786341	11787477	HisCl1	649	16.1055	3.96
chr3R	11796367	11797090	Sbf	86	15.5555	3.56
chr3R	11818696	11820196	glo	480	33.0659	5.03
chr3R	11818696	11820196	COX5A	221	33.0659	5.03
chr3R	11868622	11869663	CG6959	44	48.6278	6.21
chr3R	11882028	11883239	CG6962	420	24.1873	4.09
chr3R	11882028	11883239	CG31368	165	24.1873	4.09
chr3R	11886670	11887321	mthl5	61	16.148	3.97
chr3R	11948573	11949310	Cad87A	60	62.5446	7.09
chr3R	11949588	11950285	CG31211	302	28.946	4.96
chr3R	11958107	11958673	Hsp70Aa	19	29.9106	4.38
chr3R	11958107	11958673	Hsp70Ab	18	29.9106	4.38
chr3R	11967026	11968056	CG12213	225	22.4665	4.16
chr3R	11967026	11968056	GC1	475	22.4665	4.16
chr3R	12016137	12016927	CG3313	671	7.50858	3.01
chr3R	12017200	12017492	Spt3	238	12.6847	3.44
chr3R	12090203	12090909	ATP8B	14	27.1138	4.99
chr3R	12121196	12121501	CG4702	6843	20.8177	4.43
chr3R	12355784	12356339	MBD-R2	126	28.8775	4.73
chr3R	12369000	12369615	GstD1	11	71.3124	8.50
chr3R	12405586	12406016	Cyp9f2	158	16.0888	3.53
chr3R	12425247	12426316	Pp1-87B	197	21.8876	4.41
chr3R	12429002	12429386	CG5641	169	10.3353	3.01
chr3R	12431016	12431709	Prosalpha2	206	16.6278	3.44
chr3R	12459694	12460167	CG5961	189	11.8446	3.33
chr3R	12719314	12720099	Men	67	22.4738	4.31
chr3R	12857277	12857815	CG10126	36	17.6866	4.26
chr3R	12985960	12986752	CG31342	90	21.6057	4.47
chr3R	12997131	12997671	CG44194	74	16.2998	4.07
chr3R	12997131	12997671	CG17327	74	16.2998	4.07
chr3R	12997946	12998639	CG7518	93	44.8686	5.83
chr3R	13010735	13012871	CG8031	164	31.7926	4.69
chr3R	13010735	13012871	CtBP	502	31.7926	4.69
chr3R	13051923	13052726	pic	217	26.6031	4.77
chr3R	13260155	13260921	CG11686	1	31.5922	4.96
chr3R	13263669	13264635	Ravus	400	14.8323	3.42

chr3R	13263669	13264635	Su(var)3-7	98	14.8323	3.42
chr3R	13364372	13365293	poly	362	16.6246	3.51
chr3R	13364372	13365293	Dic1	13	16.6246	3.51
chr3R	13388208	13388811	CG9799	194	24.9643	4.39
chr3R	13401227	13401990	GILT1	72	28.7239	5.04
chr3R	13434310	13434975	yrt	206	14.2378	3.18
chr3R	13644865	13646304	sqd	22	42.4869	5.76
chr3R	13660084	13660665	Hrb87F	151	41.3964	5.43
chr3R	13661181	13661889	B52	131	56.83	7.53
chr3R	13683661	13685346	flfl	257	18.9737	3.63
chr3R	13715075	13715494	CG34383	41	17.2252	3.30
chr3R	13831400	13831820	Mst87F	3679	9.81937	3.24
chr3R	13837494	13838179	Nsf2	137	19.3727	4.14
chr3R	13874811	13875550	E5	243	16.8023	3.90
chr3R	14024837	14026078	rdx	900	37.8858	5.51
chr3R	14051634	14053697	foxo	3801	12.2258	3.28
chr3R	14056760	14057197	foxo	46	26.6266	4.61
chr3R	14217035	14217937	CG9649	130	12.0756	3.13
chr3R	14308037	14308580	su(Hw)	301	10.7267	3.01
chr3R	14309011	14309570	RpII15	72	16.721	3.78
chr3R	14315980	14316711	Set8	127	21.0665	3.85
chr3R	14326051	14327687	Afti	187	31.7094	4.92
chr3R	14326051	14327687	ATPsynE	444	31.7094	4.92
chr3R	14511028	14512145	HtrA2	24	15.1903	3.49
chr3R	14511028	14512145	mRpL11	401	15.1903	3.49
chr3R	14557840	14558732	Pde6	14	17.707	4.14
chr3R	14569193	14569804	Cys	8	21.7383	3.99
chr3R	14625006	14626439	put	262	22.324	3.85
chr3R	14625006	14626439	His4r	313	22.324	3.85
chr3R	14696995	14697486	Meltrin	49	18.7755	3.50
chr3R	14723556	14724491	kibra	108	24.478	4.17
chr3R	14739170	14741812	eff	119	14.2988	3.12
chr3R	14739170	14741812	jvl	909	14.2988	3.12
chr3R	14853666	14854205	btsz	61	18.4177	4.29
chr3R	14889793	14890809	CycC	78	16.3203	3.70
chr3R	14889793	14890809	UQCR-C1	53	16.3203	3.70
chr3R	14903546	14904265	VhaPPA1-1	128	29.0724	5.23
chr3R	15094533	15096081	CG6966	205	46.7035	6.88
chr3R	15131593	15132065	CG42788	61	34.2191	4.00
chr3R	15146623	15147789	GlyS	16	22.4983	4.03
chr3R	15195663	15197114	CG6752	532	19.4731	3.87
chr3R	15195663	15197114	CG42542	145	19.4731	3.87
chr3R	15206167	15206581	Spn88Ea	90	27.9181	5.01
chr3R	15209078	15209887	CG4203	229	40.7673	5.35
chr3R	15218487	15219790	SIDL	532	14.6954	3.29
chr3R	15218487	15219790	CG12241	151	14.6954	3.29
chr3R	15225084	15225971	CG31344	29	21.6471	3.90
chr3R	15225084	15225971	Caf1-55	217	21.6471	3.90
chr3R	15228989	15229440	Art3	160	13.5752	3.48
chr3R	15242153	15243988	tefu	1363	28.7545	4.56
chr3R	15242153	15243988	Hsc70-4	402	28.7545	4.56
chr3R	15262747	15264132	eIF-2gamma	221	33.2065	5.53
chr3R	15262747	15264132	Su(var)3-9	221	33.2065	5.53
chr3R	15262747	15264132	eIF-2gamma	221	33.2065	5.53
chr3R	15262747	15264132	Set	314	33.2065	5.53
chr3R	15274525	15276090	Cp190	28	21.4234	4.62

chr3R	15274525	15276090	CG4338	308	21.4234	4.62
chr3R	15289968	15292034	CG45218	170	21.7383	3.99
chr3R	15289968	15292034	Tm1	24	21.7383	3.99
chr3R	15328100	15329137	CG6236	551	20.1403	4.04
chr3R	15328100	15329137	ea	92	20.1403	4.04
chr3R	15330591	15331108	mRpL9	71	11.8887	3.44
chr3R	15332600	15334068	FK506-bp1	313	38.826	5.86
chr3R	15332600	15334068	Sra-1	181	38.826	5.86
chr3R	15342623	15343636	CG6218	142	25.2495	4.20
chr3R	15345075	15346487	Surf4	68	16.9985	3.83
chr3R	15345075	15346487	CG31301	96	16.9985	3.83
chr3R	15347827	15348339	CG42727	37	13.5778	3.39
chr3R	15347827	15348339	CG42726	37	13.5778	3.39
chr3R	15356262	15357662	Atg4b	447	24.6336	4.28
chr3R	15356262	15357662	CG5044	143	24.6336	4.28
chr3R	15408108	15409444	Atx2	123	22.4946	4.47
chr3R	15792588	15794983	Acyp2	1054	30.0605	4.36
chr3R	15792588	15794983	nsl1	40	30.0605	4.36
chr3R	15804022	15804607	alpha-Man-IIb	53	22.3363	4.05
chr3R	15836514	15837142	ND-23	128	28.7003	5.60
chr3R	15933782	15934369	blp	146	13.7673	3.56
chr3R	15972297	15974262	mor	326	57.6622	7.20
chr3R	15972297	15974262	Hel89B	710	57.6622	7.20
chr3R	16050659	16051754	msps	306	19.3052	3.89
chr3R	16097713	16098791	Mhcl	130	11.8427	3.02
chr3R	16099087	16100279	Akt1	151	24.5103	4.05
chr3R	16146090	16147463	CG5903	341	23.839	4.25
chr3R	16146090	16147463	CG5916	168	23.839	4.25
chr3R	16186552	16187299	Bin1	150	24.3115	4.33
chr3R	16186552	16187299	sra	49	24.3115	4.33
chr3R	16202781	16203203	CG6126	67	29.7954	5.51
chr3R	16235279	16237906	tara	6319	17.9766	3.79
chr3R	16240528	16241730	tara	1494	16.8586	3.31
chr3R	16242187	16243113	tara	71	28.9631	4.25
chr3R	16263389	16264963	bor	244	26.5163	4.45
chr3R	16263389	16264963	asun	419	26.5163	4.45
chr3R	16272978	16273669	gish	329	13.068	3.61
chr3R	16305043	16306082	Zip89B	90	14.499	3.62
chr3R	16341357	16342790	SF2	398	29.1399	4.67
chr3R	16341357	16342790	pad	69	29.1399	4.67
chr3R	16356241	16357140	CG10311	61	12.4008	3.02
chr3R	16442517	16443354	CG14894	93	17.5054	4.22
chr3R	16442517	16443354	CG14882	292	17.5054	4.22
chr3R	16452797	16453986	Pak3	55	31.4721	5.35
chr3R	16455995	16456906	CG14883	147	11.0619	3.40
chr3R	16469128	16469780	CG42232	56	18.1777	4.37
chr3R	16613985	16614485	CG10340	100	13.0163	3.55
chr3R	16655163	16655781	modSP	80	33.0429	5.50
chr3R	17002073	17003144	CG16941	579	17.5761	4.00
chr3R	17002073	17003144	lrc	60	17.5761	4.00
chr3R	17020704	17021228	AdSL	147	14.6522	3.43
chr3R	17042694	17044050	CG3995	642	19.8919	3.95
chr3R	17042694	17044050	Patr-1	215	19.8919	3.95
chr3R	17053916	17054337	Dad	32	19.6406	3.86
chr3R	17072059	17073130	Ns1	70	15.7186	3.17
chr3R	17072059	17073130	mRpS11	14	15.7186	3.17

chr3R	17079097	17081598	Keap1	2	34.6523	5.01
chr3R	17079097	17081598	kuk	300	34.6523	5.01
chr3R	17148889	17149886	m-cup	43	13.068	3.08
chr3R	17148889	17149886	Det	297	13.068	3.08
chr3R	17151495	17152274	CG5292	199	20.2994	4.16
chr3R	17385675	17386856	lute	345	27.0287	4.74
chr3R	17390772	17391469	Brf	27	13.0377	3.41
chr3R	17391753	17392067	CR44160	314	10.4692	3.08
chr3R	17539514	17540012	l(3)07882	127	17.4043	3.73
chr3R	17540400	17540963	SF1	162	18.642	3.95
chr3R	17546179	17546575	Prx3	239	8.78992	3.17
chr3R	17670372	17670987	CG7523	51	29.0331	5.29
chr3R	17699347	17701820	CR46036	326	25.1778	4.75
chr3R	17716795	17718668	osa	1050	22.0143	4.08
chr3R	17797871	17798550	CG7379	185	14.4751	3.52
chr3R	17810607	17811811	CG18012	163	16.3203	3.70
chr3R	17810607	17811811	tinc	569	16.3203	3.70
chr3R	18121444	18122126	sr	75	20.766	4.75
chr3R	18164016	18164984	CG7218	94	22.1773	4.36
chr3R	18164016	18164984	Cbp20	357	22.1773	4.36
chr3R	18165983	18166638	Prx5	134	21.0679	4.06
chr3R	18167672	18169446	PP2A-B'	168	35.1548	5.68
chr3R	18182867	18183573	cdm	159	17.1671	3.56
chr3R	18185612	18186174	CG7208	82	16.6414	4.03
chr3R	18185612	18186174	Arp5	60	16.6414	4.03
chr3R	18197872	18200451	Ssdp	156	17.8739	3.53
chr3R	18197872	18200451	CG7985	417	17.8739	3.53
chr3R	18223075	18224086	mTerf5	152	17.7039	4.20
chr3R	18223075	18224086	Non3	113	17.7039	4.20
chr3R	18226034	18227149	CG7168	404	38.2318	5.34
chr3R	18226034	18227149	Mdh2	91	38.2318	5.34
chr3R	18242445	18245398	14-3-3epsilon	169	31.4773	4.43
chr3R	18273925	18275481	Vps39	443	36.2081	5.14
chr3R	18273925	18275481	eIF-1A	192	36.2081	5.14
chr3R	18298191	18299726	l(3)05822	276	32.598	4.86
chr3R	18298191	18299726	Dlc90F	625	32.598	4.86
chr3R	18301232	18301854	CG18600	39	21.6811	4.55
chr3R	18310522	18311127	PKD	34	19.6858	4.10
chr3R	18314109	18314803	PKD	13	16.1079	3.62
chr3R	18360498	18360870	CG15803	41	11.0619	3.40
chr3R	18366438	18366952	Nup43	203	11.4702	3.02
chr3R	18380849	18381461	CG7675	35	17.4679	3.98
chr3R	18398700	18400102	Vha100-2	652	25.7482	4.42
chr3R	18398700	18400102	Vti1b	113	25.7482	4.42
chr3R	18402278	18402926	CG12333	2	43.8895	5.98
chr3R	18412211	18413217	CG31122	6	25.041	4.55
chr3R	18584415	18584735	CG7694	56	13.1871	3.13
chr3R	18584415	18584735	fray	56	13.1871	3.13
chr3R	18658540	18660111	CG31224	115	24.0271	4.10
chr3R	18658540	18660111	CstF-64	423	24.0271	4.10
chr3R	18739319	18740493	Mekk1	91	38.6857	5.41
chr3R	18742579	18743949	Mekk1	97	19.3143	3.54
chr3R	18758365	18759010	CG14299	107	12.9859	3.23
chr3R	18906463	18907148	EndoA	271	22.1547	4.20
chr3R	18914718	18915138	Xrp1	19	10.9657	3.14
chr3R	18917953	18919258	Xrp1	119	18.9556	3.49

chr3R	18928216	18928788	Mpc1	134	28.0587	5.10
chr3R	18983114	18984314	snRNP-U1-C	435	42.2101	5.45
chr3R	18983114	18984314	gukh	61	42.2101	5.45
chr3R	19023711	19024587	CG6005	192	12.6315	3.13
chr3R	19023711	19024587	CG14286	131	12.6315	3.13
chr3R	19027066	19029325	NP15.6	206	23.1448	4.72
chr3R	19027066	19029325	GatA	1284	23.1448	4.72
chr3R	19027066	19029325	P5cr	1357	23.1448	4.72
chr3R	19030814	19031253	Prp18	259	18.1375	4.17
chr3R	19031522	19031932	CG6013	188	14.1207	3.37
chr3R	19093513	19095809	cdi	41	24.577	3.95
chr3R	19093513	19095809	mRpL55	676	24.577	3.95
chr3R	19093513	19095809	ATPsynD	770	24.577	3.95
chr3R	19147330	19148202	Ppcs	44	15.5781	3.33
chr3R	19156402	19157095	CG11779	55	25.041	4.55
chr3R	19161481	19162567	Nsun5	191	19.5751	3.77
chr3R	19161481	19162567	CG42359	191	19.5751	3.77
chr3R	19163826	19164948	sqz	87	14.3728	3.60
chr3R	19211382	19212461	cry	100	22.3363	4.05
chr3R	19246094	19246708	unc79	363	41.2653	6.36
chr3R	19325985	19326339	Dl	37	12.9535	3.88
chr3R	19402779	19403746	Ino80	36	17.0627	3.54
chr3R	19402779	19403746	CG5316	337	17.0627	3.54
chr3R	19620947	19621228	CG6231	42	15.4097	3.66
chr3R	19640946	19641631	subdued	62	26.6451	4.29
chr3R	19642103	19644589	subdued	1486	25.8329	4.58
chr3R	19642103	19644589	Vha13	178	25.8329	4.58
chr3R	19645057	19645681	Nup58	230	24.5074	4.68
chr3R	19647945	19648913	CG6195	150	22.3101	4.18
chr3R	19647945	19648913	CG31220	226	22.3101	4.18
chr3R	19860763	19862161	Ire1	117	30.5252	5.00
chr3R	19860763	19862161	CG11447	435	30.5252	5.00
chr3R	19875199	19875815	CG4562	20	7.76419	3.08
chr3R	19879662	19880178	Ask1	233	24.5074	4.68
chr3R	19902384	19903818	CG4538	254	33.9599	5.34
chr3R	19904253	19906217	Surf6	105	21.0887	4.06
chr3R	19904253	19906217	RhoGAP92B	400	21.0887	4.06
chr3R	20008099	20009283	Hs6st	98	44.9986	5.74
chr3R	20008099	20009283	MED25	532	44.9986	5.74
chr3R	20020537	20021468	CG4433	55	16.5152	4.40
chr3R	20021762	20022442	psidin	263	19.6858	4.10
chr3R	20030749	20031401	trem	251	15.0256	3.59
chr3R	20035590	20036288	CG10889	23	25.8329	4.58
chr3R	20057091	20058145	CG4390	166	17.9783	4.14
chr3R	20057091	20058145	mdlc	423	17.9783	4.14
chr3R	20252559	20253986	cic	109	32.3973	4.98
chr3R	20308973	20310711	CG42668	244	22.5758	3.68
chr3R	20326846	20328150	CG4360	191	106.518	10.11
chr3R	20549765	20551288	Stat92E	62	35.6432	4.66
chr3R	20551576	20552493	Stat92E	11	19.496	4.12
chr3R	20556217	20557888	att-ORFA	917	45.4338	6.09
chr3R	20558159	20558588	CG5180	8	14.2251	3.66
chr3R	20579364	20580492	EloB	8	26.8997	4.56
chr3R	20579364	20580492	Srp14	256	26.8997	4.56
chr3R	20585008	20585900	CG16953	196	20.6165	4.28
chr3R	20587972	20588433	CR46044	233	22.9408	4.10

chr3R	20587972	20588433	44076	121	22.9408	4.10
chr3R	20592982	20593463	bon	166	28.3782	4.86
chr3R	20621782	20622833	H	257	18.4033	3.91
chr3R	20633962	20634754	Pi3K92E	221	16.9389	3.73
chr3R	20635331	20636164	Lrrk	153	16.8125	3.58
chr3R	20778558	20779116	Syp	63	25.0925	4.22
chr3R	20783601	20785297	Syp	1791	20.6607	3.78
chr3R	20818605	20819033	CG17270	46	17.8922	3.61
chr3R	20821083	20822754	CG17271	256	41.4484	5.97
chr3R	20821083	20822754	RpS20	588	41.4484	5.97
chr3R	20821083	20822754	snoRNA:Psi18S-1295	1017	41.4484	5.97
chr3R	20829766	20830780	AdSS	280	23.824	3.92
chr3R	20832683	20833869	Synd	206	37.592	5.71
chr3R	20850382	20851132	RpS30	37	17.6365	3.81
chr3R	20948632	20950009	Atpalpha	142	34.4586	5.91
chr3R	21102160	21102781	CG5745	158	15.0256	3.59
chr3R	21108343	21108708	rtet	55	19.2253	4.34
chr3R	21126480	21128158	slmb	243	16.5514	3.80
chr3R	21126480	21128158	CG5793	15	16.5514	3.80
chr3R	21155900	21156782	SNF4Agamma	362	12.4735	3.53
chr3R	21188601	21189081	SNF4Agamma	11	26.3346	4.20
chr3R	21204779	21205192	SNF4Agamma	18	11.6615	3.34
chr3R	21215008	21215527	CG5862	305	10.9098	3.27
chr3R	21223186	21224592	CG3337	201	27.4289	4.50
chr3R	21223186	21224592	Nelf-A	82	27.4289	4.50
chr3R	21223186	21224592	CR44265	804	27.4289	4.50
chr3R	21268181	21270452	Rab1	77	12.0279	3.02
chr3R	21268181	21270452	AP-2sigma	1092	12.0279	3.02
chr3R	21268181	21270452	CG5919	1142	12.0279	3.02
chr3R	21273216	21273915	CG3308	378	12.817	3.13
chr3R	21296402	21298127	Hsromega	67	39.0931	5.42
chr3R	21348536	21349766	CG16791	40	30.4766	4.59
chr3R	21376267	21377510	mod(mdg4)	52	23.0834	4.22
chr3R	21446406	21446951	lbe	53	21.7452	4.44
chr3R	21520726	21521418	CG7956	47	19.6885	4.26
chr3R	21618324	21620041	InR	42	37.4501	5.03
chr3R	21662630	21662994	CG6353	130	27.1573	5.42
chr3R	21948300	21949421	Eip93F	275	12.8757	3.08
chr3R	22018630	22019428	CG6439	138	26.0427	4.37
chr3R	22022427	22024120	Mitofilin	180	46.6812	6.80
chr3R	22022427	22024120	mRpL35	632	46.6812	6.80
chr3R	22033726	22034908	CG45099	324	13.0517	3.33
chr3R	22033726	22034908	CG6015	324	13.0517	3.33
chr3R	22033726	22034908	pit	335	13.0517	3.33
chr3R	22033726	22034908	Fadd	492	13.0517	3.33
chr3R	22035593	22036312	pit	88	23.6489	5.03
chr3R	22356396	22357539	Sar1	204	34.6583	5.23
chr3R	22361887	22363317	PSR	13	23.2593	4.29
chr3R	22361887	22363317	Muted	261	23.2593	4.29
chr3R	22361887	22363317	CG7071	261	23.2593	4.29
chr3R	22361887	22363317	Muted	538	23.2593	4.29
chr3R	22420351	22421637	Nop56	159	27.2259	4.95
chr3R	22420351	22421637	mats	251	27.2259	4.95
chr3R	22424452	22425000	pinta	35	17.8061	3.80
chr3R	22461519	22462067	Rpn7	199	16.4711	3.93
chr3R	22462479	22462883	AP-2mu	190	13.0829	3.25

chr3R	22487055	22487748	CG7048	157	19.5363	3.36
chr3R	22490990	22491687	T-cp1	175	15.5832	3.65
chr3R	22526671	22528730	CG5346	189	15.882	3.86
chr3R	22539742	22540290	CG5326	60	9.53515	3.17
chr3R	22539742	22540290	CR45646	136	9.53515	3.17
chr3R	22587418	22587979	Efa6	46	21.5761	3.81
chr3R	22666446	22667564	wake	91	21.5802	4.41
chr3R	22685630	22687012	Gclm	106	32.4667	5.27
chr3R	22685630	22687012	CG17625	444	32.4667	5.27
chr3R	22689929	22691036	wfs1	319	10.1335	3.32
chr3R	22689929	22691036	Nup133	93	10.1335	3.32
chr3R	22725746	22726465	CG4813	318	17.2061	4.09
chr3R	22726990	22727854	CG45049	342	14.921	3.36
chr3R	23066316	23067448	CG17111	89	11.3107	3.52
chr3R	23117293	23118113	Rassf	64	16.9775	3.46
chr3R	23127175	23128118	CG31457	205	10.9657	3.14
chr3R	23156796	23158145	unk	514	32.1832	4.75
chr3R	23226014	23227482	cnc	22	35.6818	5.71
chr3R	23257789	23258665	wda	30	17.5761	4.00
chr3R	23257789	23258665	CG13827	307	17.5761	4.00
chr3R	23356485	23357003	Sec13	171	34.8581	6.07
chr3R	23358699	23359540	RpS3	96	25.041	4.55
chr3R	23374308	23374831	CG42828	761	10.4114	3.15
chr3R	23472133	23472683	CG4374	165	10.6553	3.08
chr3R	23653829	23654571	CG31145	15272	12.0224	3.53
chr3R	23669318	23670159	CG31145	41	15.5487	3.47
chr3R	23686164	23687054	SPE	66	17.72	3.93
chr3R	23723174	23724078	CG10365	60	19.3567	3.69
chr3R	23732350	23733552	Rpn9	36	17.835	4.05
chr3R	23732350	23733552	Hmgcr	348	17.835	4.05
chr3R	23732350	23733552	Rpn9	487	17.835	4.05
chr3R	23752183	23753305	RanBP3	20	11.1508	3.14
chr3R	23752183	23753305	Rpt5	61	11.1508	3.14
chr3R	23780819	23783065	Nup98-96	219	37.9264	5.14
chr3R	23780819	23783065	mbc	76	37.9264	5.14
chr3R	23818922	23819403	CG33111	152	18.6618	3.74
chr3R	23818922	23819403	eIF4G2	152	18.6618	3.74
chr3R	23818922	23819403	CG33111	152	18.6618	3.74
chr3R	23859294	23859809	snRNA:U1:21D	108	65.4163	7.57
chr3R	23859294	23859809	snRNA:U1:95Cb	108	65.4163	7.57
chr3R	23859294	23859809	snRNA:U1:95Ca	108	65.4163	7.57
chr3R	23890528	23891198	TfIIA-S	125	18.5546	3.84
chr3R	23921959	23923231	sba	18	26.4216	3.99
chr3R	23927220	23927688	CG13601	111	15.5832	3.65
chr3R	23933773	23934740	CG5854	283	25.2747	4.09
chr3R	23942546	23943317	Gdh	38	31.9098	4.27
chr3R	23957686	23959312	Aats-glupro	147	30.4474	5.04
chr3R	23957686	23959312	AP-1sigma	406	30.4474	5.04
chr3R	23986246	23987388	CG13603	270	46.919	6.54
chr3R	23986246	23987388	CG5902	198	46.919	6.54
chr3R	23992268	23994121	CG13604	317	26.7628	4.58
chr3R	23992268	23994121	Rab7	55	26.7628	4.58
chr3R	24008097	24008388	KrT95D	1734	7.68125	3.06
chr3R	24031125	24032618	KrT95D	101	35.8963	5.10
chr3R	24031125	24032618	Ime4	430	35.8963	5.10
chr3R	24036995	24039376	Miro	180	37.4926	5.97

chr3R	24036995	24039376	spas	664	37.4926	5.97
chr3R	24036995	24039376	Miro	1297	37.4926	5.97
chr3R	24046622	24048648	Rox8	117	24.4639	4.30
chr3R	24046622	24048648	CG5986	185	24.4639	4.30
chr3R	24046622	24048648	Rox8	386	24.4639	4.30
chr3R	24046622	24048648	CG5986	693	24.4639	4.30
chr3R	24105028	24106492	Syx1A	37	22.7821	4.12
chr3R	24108388	24109719	4EHP	437	12.0756	3.13
chr3R	24108388	24109719	CG18428	31	12.0756	3.13
chr3R	24152281	24152936	p38a	160	19.7277	3.96
chr3R	24152281	24152936	CG6178	135	19.7277	3.96
chr3R	24163153	24163600	mRpS24	180	31.8322	5.70
chr3R	24169070	24169687	CG5510	74	17.8941	3.40
chr3R	24218354	24218925	CG17786	15	15.2599	3.78
chr3R	24230424	24231516	mask	213	22.4665	4.16
chr3R	24230424	24231516	CR44092	411	22.4665	4.16
chr3R	24230424	24231516	CR45217	590	22.4665	4.16
chr3R	24252309	24252817	beta-PheRS	75	16.6352	3.91
chr3R	24270947	24271444	jar	61	16.6352	3.59
chr3R	24272649	24273288	Orct2	40	11.1508	3.14
chr3R	24289892	24290602	CG6364	170	17.5816	3.76
chr3R	24294866	24295882	crb	17	21.6471	3.90
chr3R	24318969	24319988	BRWD3	176	22.8258	4.00
chr3R	24318969	24319988	CR46093	607	22.8258	4.00
chr3R	24332790	24333590	CG5728	23	21.8156	3.88
chr3R	24332790	24333590	Dis3	139	21.8156	3.88
chr3R	24348204	24349606	CG5746	85	33.096	5.38
chr3R	24348204	24349606	CG6454	475	33.096	5.38
chr3R	24357495	24358002	Golgin84	216	11.2415	3.39
chr3R	24359836	24360287	CG18528	65	9.91295	3.07
chr3R	24518186	24518998	Pp1alpha-96A	211	24.9643	4.39
chr3R	24528021	24528545	CG6607	85	10.9184	3.22
chr3R	24542994	24544055	CG5789	54	81.3599	8.80
chr3R	24581899	24582989	REPTOR	72	13.889	3.03
chr3R	24584736	24586884	CG13625	1526	15.8772	3.76
chr3R	24584736	24586884	mld	1034	15.8772	3.76
chr3R	24625065	24626320	Syx18	330	21.2353	3.83
chr3R	24625065	24626320	atl	222	21.2353	3.83
chr3R	24629728	24630935	Wsck	230	27.5782	4.83
chr3R	24629728	24630935	atl	15	27.5782	4.83
chr3R	24649221	24651803	puf	290	21.8736	4.35
chr3R	24649221	24651803	ash2	209	21.8736	4.35
chr3R	24654592	24655489	CG31125	379	15.1903	3.49
chr3R	24654592	24655489	CG6695	40	15.1903	3.49
chr3R	24718933	24720327	CG13630	748	10.744	3.06
chr3R	24718933	24720327	tok	56	10.744	3.06
chr3R	24786676	24787563	CG5805	60	28.4819	4.61
chr3R	24799152	24800158	Ude	69	18.3864	3.86
chr3R	24804966	24805344	polybromo	75	17.5761	4.00
chr3R	24813356	24814557	CG5807	100	23.5522	4.16
chr3R	24813356	24814557	CG34150	349	23.5522	4.16
chr3R	24817468	24818012	Saf-B	212	21.3964	4.32
chr3R	24860211	24860623	CG13639	31	9.91295	3.07
chr3R	24875964	24877558	CG3744	314	26.4777	4.81
chr3R	24875964	24877558	CG31381	704	26.4777	4.81
chr3R	24875964	24877558	CG11089	704	26.4777	4.81

chr3R	24875964	24877558	CG31381	704	26.4777	4.81
chr3R	24917306	24918786	sud1	97	18.2908	4.15
chr3R	24917306	24918786	PIG-S	97	18.2908	4.15
chr3R	24917306	24918786	CG11168	338	18.2908	4.15
chr3R	24931989	24932454	CG11771	171	21.8876	4.41
chr3R	24937694	24938405	Osbp	44	20.2001	4.77
chr3R	24944380	24945709	ssh	242	30.0685	4.67
chr3R	24944380	24945709	Nmnat	522	30.0685	4.67
chr3R	25024587	25025775	CG11781	445	25.041	4.55
chr3R	25024587	25025775	Hr96	228	25.041	4.55
chr3R	25032859	25033600	Smg6	185	21.8156	3.88
chr3R	25038179	25039669	CG6422	159	15.5555	3.40
chr3R	25038179	25039669	bai	35	15.5555	3.40
chr3R	25047678	25048171	CG11790	125	15.635	3.76
chr3R	25069285	25070048	OstStt3	256	9.35909	3.03
chr3R	25069285	25070048	CG11839	545	9.35909	3.03
chr3R	25122443	25123028	CG31108	121	30.0324	5.21
chr3R	25122443	25123028	CG31510	121	30.0324	5.21
chr3R	25123350	25124238	vig2	42	20.1448	4.19
chr3R	25219195	25220004	CG31103	85	21.5451	4.65
chr3R	25246999	25247636	RpS27	228	18.4241	3.58
chr3R	25249242	25250014	Nup358	478	14.1332	3.30
chr3R	25258230	25259714	CG11857	271	30.4474	5.04
chr3R	25265130	25266349	Aats-gln	127	27.8259	4.76
chr3R	25265130	25266349	RIOK2	5	27.8259	4.76
chr3R	25292701	25293024	CG31098	160	10.7814	3.14
chr3R	25308231	25308510	CG10550	7	20.5506	4.32
chr3R	25472772	25474092	Fur1	56	26.5254	4.07
chr3R	25472772	25474092	Vps33B	487	26.5254	4.07
chr3R	25482124	25483720	XNP	82	40.8563	5.75
chr3R	25482124	25483720	CG42488	437	40.8563	5.75
chr3R	25482124	25483720	CG5116	437	40.8563	5.75
chr3R	25489969	25491056	Dhap-at	171	12.3502	3.27
chr3R	25624087	25624814	Npl4	80	12.3116	3.30
chr3R	25646858	25647584	jigr1	123	14.2008	3.35
chr3R	25660778	25661423	Tnks	3	11.1508	3.14
chr3R	25661690	25662957	RASSF8	137	34.0553	4.79
chr3R	25661690	25662957	CR45208	732	34.0553	4.79
chr3R	25680232	25681306	CG4743	337	14.3293	3.30
chr3R	25680232	25681306	CG5028	43	14.3293	3.30
chr3R	25684721	25685217	RpL27	87	26.3903	4.47
chr3R	25740243	25740614	LpR2	46	18.0528	3.89
chr3R	25880823	25881504	Tsp96F	207	14.1853	3.56
chr3R	25888291	25889184	Lnk	214	17.6476	3.86
chr3R	25982838	25983754	Nf1	363	30.9988	4.67
chr3R	26005771	26006721	E(spl)mbeta-HLH	80	10.3353	3.01
chr3R	26041527	26043348	gro	124	50.3757	6.05
chr3R	26051904	26052334	Exo84	71	13.0829	3.25
chr3R	26052641	26053404	Vps2	152	26.9372	5.15
chr3R	26054682	26055622	Dak1	36	33.6772	5.13
chr3R	26058472	26059953	RpL34a	280	10.5404	3.01
chr3R	26058472	26059953	CG14544	508	10.5404	3.01
chr3R	26106637	26107155	CG31324	4	33.6187	5.41
chr3R	26188262	26189007	CG6073	134	53.7841	6.80
chr3R	26261053	26261668	Ald	59	23.0896	4.08
chr3R	26452514	26453039	CG5447	189	23.6737	4.70

chr3R	26536152	26536986	scrib	130	32.3288	4.77
chr3R	26717863	26718393	CG5484	241	18.8066	3.94
chr3R	26798928	26800110	Tl	68	15.3751	3.41
chr3R	26866282	26867662	ball	317	25.5629	4.49
chr3R	26866282	26867662	His2Av	196	25.5629	4.49
chr3R	26875174	26876889	Rb97D	70	24.818	4.41
chr3R	26875174	26876889	ms(3)K81	179	24.818	4.41
chr3R	26875174	26876889	CG5500	957	24.818	4.41
chr3R	26881143	26882091	ro	542	18.7233	3.83
chr3R	26881143	26882091	T48	20	18.7233	3.83
chr3R	26912273	26912985	Ets97D	51	28.7166	5.44
chr3R	26966842	26967382	CG5521	203	31.5922	4.96
chr3R	26988266	26988864	CG31075	40	50.7049	7.65
chr3R	27069762	27070364	spz	43	17.5391	4.29
chr3R	27094362	27094884	CG14259	101	27.7617	5.64
chr3R	27111655	27112876	gb	177	29.6402	5.02
chr3R	27111655	27112876	CG5815	472	29.6402	5.02
chr3R	27149802	27151249	CG6051	118	41.6892	5.88
chr3R	27151535	27152303	Men-b	52	21.6959	4.27
chr3R	27244482	27245811	CG18766	92	27.6913	4.59
chr3R	27244482	27245811	CR46085	606	27.6913	4.59
chr3R	27263280	27264220	woc	214	33.317	4.97
chr3R	27272298	27273985	l(3)mbt	436	14.1036	3.59
chr3R	27272298	27273985	CG5934	5	14.1036	3.59
chr3R	27274516	27275769	CG5938	165	19.3567	3.69
chr3R	27283703	27284336	CG3368	140	18.7614	4.08
chr3R	27287686	27289188	bigmax	150	56.9171	7.63
chr3R	27287686	27289188	Hmu	392	56.9171	7.63
chr3R	27580052	27582322	wdb	114	17.3643	3.64
chr3R	27582883	27583422	pins	324	11.1508	3.14
chr3R	27635630	27636891	CG5611	48	21.6914	4.49
chr3R	27635630	27636891	Mtl	303	21.6914	4.49
chr3R	27662338	27663462	unc80	632	11.2968	3.35
chr3R	27677178	27678044	CG5590	237	15.9094	3.81
chr3R	27699601	27700920	ALiX	253	20.2246	3.91
chr3R	27699601	27700920	btz	503	20.2246	3.91
chr3R	27916113	27916568	CG5003	177	16.4597	3.60
chr3R	27916865	27917362	mRpS22	148	25.5021	4.93
chr3R	27920561	27921088	CG12259	144	20.4017	4.47
chr3R	27921930	27922348	BCAS2	137	9.45755	3.10
chr3R	27922700	27923498	RpL4	172	36.1072	5.59
chr3R	27924472	27925859	mino	40	23.5836	4.68
chr3R	27924472	27925859	CR45566	138	23.5836	4.68
chr3R	27924472	27925859	mino	434	23.5836	4.68
chr3R	27937281	27938165	Mes-4	10	15.0256	3.59
chr3R	27938519	27939208	CG5514	206	23.7459	4.43
chr3R	27947256	27948156	mfrn	182	27.811	5.06
chr3R	27948570	27949261	Gp93	73	57.6144	7.12
chr3R	27987580	27988487	betaTub97EF	33	26.6968	5.17
chr3R	28325942	28326873	larp	22	21.7979	4.57
chr3R	28335028	28336651	larp	243	33.5549	4.90
chr3R	28339551	28339935	Gfat2	22	17.6365	3.81
chr3R	28340306	28340903	Moca-cyp	186	31.4025	4.77
chr3R	28340306	28340903	CR46105	281	31.4025	4.77
chr3R	28544465	28544911	CG12413	149	22.3903	4.40
chr3R	28598818	28599292	Noa36	184	14.4223	3.18

chr3R	28599760	28601050	Hrb98DE	32	19.7277	3.96
chr3R	28681950	28682625	CG9990	37	14.3026	3.37
chr3R	28810788	28811455	wdn	166	46.1965	7.13
chr3R	28812021	28812792	CG1646	88	28.9394	5.02
chr3R	28812021	28812792	snoRNA:291	315	28.9394	5.02
chr3R	28831237	28832188	WASp	240	23.4475	4.43
chr3R	28844258	28845557	Apc	562	14.9783	3.17
chr3R	28844258	28845557	spg	11	14.9783	3.17
chr3R	28858666	28859391	Inx3	64	16.4597	3.60
chr3R	28887464	28889825	CG33203	1573	15.5094	3.29
chr3R	28887464	28889825	Doa	262	15.5094	3.29
chr3R	28891605	28892122	Doa	21	14.1207	3.37
chr3R	29038487	29040504	Slu7	130	22.7821	4.12
chr3R	29038487	29040504	Pkc98E	46	22.7821	4.12
chr3R	29062653	29063391	Cul5	160	18.069	3.60
chr3R	29062653	29063391	CG11873	96	18.069	3.60
chr3R	29063655	29065090	CG11873	120	35.8037	5.30
chr3R	29112297	29113686	alpha-Man-Ib	533	31.4096	5.15
chr3R	29112297	29113686	CG11876	148	31.4096	5.15
chr3R	29115958	29116805	Atg14	115	11.4905	3.30
chr3R	29122320	29123677	yem	462	15.8437	3.85
chr3R	29122320	29123677	CG11880	28	15.8437	3.85
chr3R	29131227	29131947	Vha100-1	134	15.466	3.77
chr3R	29138084	29139156	CG14512	71	22.4096	4.77
chr3R	29138084	29139156	CG14516	17	22.4096	4.77
chr3R	29146530	29146991	CG14511	15	12.1149	3.35
chr3R	29147729	29148608	CG11882	235	30.0867	5.16
chr3R	29147729	29148608	Pglym78	46	30.0867	5.16
chr3R	29152885	29154064	Slbp	21	19.2127	4.01
chr3R	29152885	29154064	Rpn2	278	19.2127	4.01
chr3R	29157730	29158637	CG11897	43	27.0376	4.91
chr3R	29199883	29200872	CG11899	74	34.2059	5.16
chr3R	29214360	29215403	CR31044	26	19.6243	3.86
chr3R	29219971	29220645	Ef1gamma	4	20.5596	3.89
chr3R	29222998	29224074	Cisd2	147	28.5642	5.57
chr3R	29222998	29224074	Brd8	91	28.5642	5.57
chr3R	29229050	29230162	Usp1	0	13.5778	3.39
chr3R	29318703	29319677	Cnx99A	174	19.5751	3.77
chr3R	29478901	29479944	Ptp99A	159	13.4959	3.66
chr3R	29490614	29491222	Spase12	97	19.3727	4.14
chr3R	29493129	29493672	CG2310	139	29.8651	4.95
chr3R	29503103	29504010	Trc8	241	13.2973	3.33
chr3R	29591725	29592646	alph	374	18.5546	3.84
chr3R	29723105	29723803	Pcd	337	21.6959	4.12
chr3R	29724508	29727115	CG7593	918	19.402	4.00
chr3R	29724508	29727115	CG18731	842	19.402	4.00
chr3R	29724508	29727115	Kul	39	19.402	4.00
chr3R	29736428	29737203	CG7601	55	23.4087	4.71
chr3R	29736428	29737203	CIA30	274	23.4087	4.71
chr3R	29744876	29745671	CG7609	271	22.6849	4.34
chr3R	29744876	29745671	IntS11	27	22.6849	4.34
chr3R	29748199	29749827	ATPsyngamma	197	42.2628	5.97
chr3R	29748199	29749827	yata	142	42.2628	5.97
chr3R	29772838	29774398	kay	259	25.7065	4.46
chr3R	29782533	29783044	kay	71	13.7274	3.22
chr3R	29798974	29800216	CG7837	118	19.0787	4.04

chr3R	29798974	29800216	Drice	421	19.0787	4.04
chr3R	29802315	29803633	CG7834	5	22.4147	4.35
chr3R	29802315	29803633	CG7789	346	22.4147	4.35
chr3R	29806516	29807934	ncd	359	20.525	4.37
chr3R	29806516	29807934	ca	296	20.525	4.37
chr3R	29806516	29807934	ncd	21	20.525	4.37
chr3R	29806516	29807934	ca	131	20.525	4.37
chr3R	29834598	29835154	Atg16	181	34.6005	4.86
chr3R	29841451	29841861	Cad99C	39	14.8243	3.59
chr3R	29856980	29857551	dgt1	203	20.8122	4.16
chr3R	29859696	29862407	CG15514	534	32.3734	5.32
chr3R	29859696	29862407	RpS8	132	32.3734	5.32
chr3R	29868587	29869826	Zip99C	145	22.9272	4.10
chr3R	29868587	29869826	CG34133	614	22.9272	4.10
chr3R	29876868	29877493	CG31038	44	23.4245	4.05
chr3R	29934738	29936326	eIF2B-alpha	80	14.6662	3.36
chr3R	29934738	29936326	CG15523	169	14.6662	3.36
chr3R	29997144	29998408	CG15525	362	17.4005	4.26
chr3R	29997144	29998408	CG11504	139	17.4005	4.26
chr3R	30004182	30005543	Nph	25	18.7624	4.25
chr3R	30004182	30005543	Nlp	110	18.7624	4.25
chr3R	30004182	30005543	Nph	187	18.7624	4.25
chr3R	30004182	30005543	Nlp	276	18.7624	4.25
chr3R	30014546	30015539	CG7920	52	20.267	3.72
chr3R	30021605	30023538	Mgat2	525	28.1955	4.77
chr3R	30021605	30023538	RpS28a	98	28.1955	4.77
chr3R	30021605	30023538	Axn	95	28.1955	4.77
chr3R	30039359	30040158	janA	195	11.8019	3.28
chr3R	30039359	30040158	Sry-beta	10	11.8019	3.28
chr3R	30039359	30040158	Sry-alpha	10	11.8019	3.28
chr3R	30043205	30044005	Sry-delta	185	42.6243	5.67
chr3R	30045485	30047502	RpL32	23	36.614	5.31
chr3R	30045485	30047502	CG7943	352	36.614	5.31
chr3R	30052482	30053150	CG7946	199	21.5849	4.20
chr3R	30056817	30057371	CG34317	189	12.8577	3.42
chr3R	30056817	30057371	CG7950	189	12.8577	3.42
chr3R	30325806	30326094	CG34300	19301	7.81828	3.22
chr3R	30386766	30389254	Fer1HCH	562	27.6128	4.07
chr3R	30386766	30389254	Fer2LCH	765	27.6128	4.07
chr3R	30386766	30389254	Fer1HCH	822	27.6128	4.07
chr3R	30386766	30389254	Fer2LCH	1237	27.6128	4.07
chr3R	30423471	30424383	CG15535	198	11.1508	3.14
chr3R	30423471	30424383	mRpS18C	242	11.1508	3.14
chr3R	30563131	30563622	tmod	208	26.9969	4.73
chr3R	30805671	30806759	wts	209	15.733	3.55
chr3R	30808347	30809235	cindr	12	23.2622	4.07
chr3R	30888000	30889129	Sap-r	47	22.9408	4.10
chr3R	31213169	31213869	Zwilch	131	11.3423	3.22
chr3R	31213169	31213869	CG1542	50	11.3423	3.22
chr3R	31215487	31216102	CG15561	222	11.7057	3.14
chr3R	31218301	31219865	ATPsynC	448	62.4217	6.70
chr3R	31218301	31219865	mir-4949	127	62.4217	6.70
chr3R	31218301	31219865	ATPsynC	60	62.4217	6.70
chr3R	31220894	31222919	CG12054	1244	44.9501	6.14
chr3R	31254764	31255510	CG1750	367	12.8391	3.56
chr3R	31254764	31255510	spn-F	141	12.8391	3.56

chr3R	31384157	31384690	CG1607	41	30.8453	5.23
chr3R	31404197	31405797	CG11337	597	18.8304	3.99
chr3R	31404197	31405797	Gprk2	93	18.8304	3.99
chr3R	31404197	31405797	CG11337	115	18.8304	3.99
chr3R	31572091	31572459	gammaCOP	117	9.42371	3.00
chr3R	31595567	31596330	RpL6	148	46.6345	5.75
chr3R	31609438	31610012	CycG	41	19.9791	3.83
chr3R	31610342	31611805	CycG	64	30.5858	4.75
chr3R	31610342	31611805	Med	131	30.5858	4.75
chr3R	31736489	31737300	CstF-50	174	18.1467	3.77
chr3R	31736489	31737300	CG11563	245	18.1467	3.77
chr3R	31743298	31744664	CG1890	231	10.6553	3.08
chr3R	31743298	31744664	CG1896	561	10.6553	3.08
chr3R	31745359	31746401	awd	50	50.17	6.44
chr3R	31746926	31748796	nero	796	33.5913	5.22
chr3R	31746926	31748796	CG1910	204	33.5913	5.22
chr3R	31776932	31778362	faf	58	39.676	6.22
chr3R	31793333	31794877	CG2118	248	14.3026	3.37
chr3R	31793333	31794877	Acf	175	14.3026	3.37
chr3R	31811792	31812293	FeCH	43	17.1427	4.02
chr3R	32051237	32052058	krz	116	30.659	5.33
chr3R	32052567	32053070	mod	159	40.9227	5.70
chr3R	32067950	32068677	Map205	58	14.1138	3.13
chr4	313289	313922	Zip102B	164	13.2179	3.49
chr4	507758	508843	zfh2	6882	19.7502	4.46
chr4	570367	570786	ND-49	191	13.4156	3.59
chr4	671098	671461	CG11360	145	12.5105	3.54
chr4	1030502	1030927	CG11076	254	20.6511	3.99
chrX	245550	246293	CG3038	106	10.941	3.32
chrX	245550	246293	G9a	15	10.941	3.32
chrX	492733	494077	CG17896	52	25.6291	5.03
chrX	499863	500271	svr	52	11.0265	3.08
chrX	522057	523675	elav	94	11.1149	3.36
chrX	529470	530246	CG4293	178	31.964	5.58
chrX	620671	621889	CG13365	96	29.7833	5.83
chrX	620671	621889	mod(r)	410	29.7833	5.83
chrX	625603	625909	CG17829	192	9.55894	3.28
chrX	628900	630024	RpL36	83	29.1703	4.94
chrX	628900	630024	l(1)1Bi	439	29.1703	4.94
chrX	647161	647781	Roc1a	176	15.191	4.06
chrX	653847	656682	Hmt4-20	538	17.0146	3.66
chrX	653847	656682	SkpA	637	17.0146	3.66
chrX	686707	687768	sdk	55	17.849	4.00
chrX	758826	760154	CG5273	15	12.1639	3.23
chrX	764416	765406	RpL22	58	47.7368	6.46
chrX	764416	765406	CR44966	215	47.7368	6.46
chrX	927178	928579	CG3711	156	15.6161	3.91
chrX	927178	928579	CG43867	549	15.6161	3.91
chrX	927178	928579	MED22	712	15.6161	3.91
chrX	940368	941426	CG3704	157	9.81937	3.24
chrX	940368	941426	mus81	426	9.81937	3.24
chrX	1026478	1027066	CG32814	81	10.0988	3.07
chrX	1026478	1027066	CG11638	84	10.0988	3.07
chrX	1026478	1027066	CG3021	171	10.0988	3.07
chrX	1041113	1041393	CG42259	4956	9.68089	3.37
chrX	1341979	1342909	CG11412	133	19.0787	4.04

chrX	1353450	1354372	CG12773	98	13.2926	3.72
chrX	1353450	1354372	CG11417	130	13.2926	3.72
chrX	1370347	1371361	ssx	180	36.7171	6.46
chrX	1398729	1400057	CG11448	6734	48.6447	7.16
chrX	1460284	1460955	Lrpprc2	71	12.1957	3.46
chrX	1463681	1464494	Ns3	193	12.6775	3.42
chrX	1465660	1467498	O-fut2	898	15.733	3.55
chrX	1465660	1467498	CG14777	146	15.733	3.55
chrX	1465660	1467498	CG14778	598	15.733	3.55
chrX	1465660	1467498	CG14777	598	15.733	3.55
chrX	1470237	1471331	pck	110	20.0096	4.33
chrX	1476173	1477334	Rab27	412	9.85778	3.15
chrX	1476173	1477334	mei-38	500	9.85778	3.15
chrX	1478483	1479824	rush	159	21.0887	4.06
chrX	1482628	1483620	snoRNA:Me28S-G980	623	42.8867	6.35
chrX	1482628	1483620	sta	40	42.8867	6.35
chrX	1482628	1483620	inc	226	42.8867	6.35
chrX	1512864	1513514	inc	198	18.642	3.95
chrX	1695163	1695927	a6	232	14.2059	3.82
chrX	1772611	1772957	CG42666	934	17.6866	4.26
chrX	1773664	1774365	CG42666	227	18.9129	4.22
chrX	1773664	1774365	Adar	221	18.9129	4.22
chrX	1858503	1859197	CG14812	2	8.93717	3.16
chrX	1861251	1862532	deltaCOP	211	14.852	3.98
chrX	1873215	1873575	Pgam5	144	11.2123	3.08
chrX	1873832	1874520	Vps26	202	21.6098	4.73
chrX	1876805	1877724	CG14817	265	20.753	4.68
chrX	1876805	1877724	CG14805	219	20.753	4.68
chrX	1880100	1881014	CG14806	189	19.5797	4.77
chrX	1889379	1890782	trr	369	17.8858	3.91
chrX	1889379	1890782	mRpL16	674	17.8858	3.91
chrX	1899411	1900682	arm	209	40.9794	6.63
chrX	1907354	1909326	Edem1	976	21.7452	5.15
chrX	1907354	1909326	mip130	859	21.7452	5.15
chrX	1907354	1909326	Edem1	270	21.7452	5.15
chrX	1907354	1909326	Rbcn-3B	178	21.7452	5.15
chrX	1922335	1923729	eIF2B-epsilon	282	25.7891	5.28
chrX	1922335	1923729	Ocrl	176	25.7891	5.28
chrX	1953003	1953330	Hr4	11203	10.7856	3.56
chrX	2008712	2009411	CG4406	247	12.4986	3.43
chrX	2012071	2013675	east	107	12.5755	3.19
chrX	2040722	2042380	usp	753	34.2199	5.79
chrX	2040722	2042380	CG4313	198	34.2199	5.79
chrX	2040722	2042380	Actn	246	34.2199	5.79
chrX	2040722	2042380	usp	246	34.2199	5.79
chrX	2040722	2042380	Actn	246	34.2199	5.79
chrX	2061469	2062068	Sik2	49	15.13	3.86
chrX	2077007	2077490	CG4025	191	8.32326	3.09
chrX	2079909	2080964	CG16903	303	19.0631	3.80
chrX	2079909	2080964	Unc-76	99	19.0631	3.80
chrX	2094019	2094996	csw	407	11.9836	3.28
chrX	2102381	2103301	csw	300	15.3504	4.03
chrX	2122443	2124026	ph-d	56	32.0738	4.83
chrX	2134331	2138879	ph-p	97	29.4106	4.61
chrX	2141319	2141995	CG3835	5	12.8637	3.51
chrX	2157960	2159146	wapl	163	27.2509	5.08

chrX	2176732	2177108	Cyp4d2	34	7.60598	3.09
chrX	2184224	2184815	Nmd3	197	26.1322	5.20
chrX	2184224	2184815	CG17776	253	26.1322	5.20
chrX	2221958	2222798	Vinc	101	13.217	3.40
chrX	2225159	2225846	pcx	162	20.8177	4.43
chrX	2253552	2254467	CG3091	73	12.1432	3.35
chrX	2262262	2263303	temp	506	23.3632	5.12
chrX	2262262	2263303	CG3071	185	23.3632	5.12
chrX	2274599	2275189	CG2918	54	15.466	3.77
chrX	2366144	2366727	CG2841	15	13.0536	3.91
chrX	2447869	2448366	boi	259	13.9783	3.66
chrX	2447869	2448366	z	196	13.9783	3.66
chrX	2584973	2585412	CG13760	126	17.0686	4.31
chrX	2585703	2586397	wds	149	29.8446	5.31
chrX	2603768	2604543	Klp3A	180	9.14444	3.27
chrX	2603768	2604543	Zw10	36	9.14444	3.27
chrX	2609391	2610383	eIF3ga	151	24.6768	5.52
chrX	2684547	2686416	per	31	22.4738	4.31
chrX	2703595	2704042	Csat	15	13.5922	3.58
chrX	2704591	2705190	eIF2B-beta	153	9.85778	3.15
chrX	2731688	2732981	ttm50	217	22.9958	5.28
chrX	2731688	2732981	CG2712	495	22.9958	5.28
chrX	2796009	2796777	w	3	47.1086	4.28
chrX	3485001	3486009	CG14270	230	20.5028	4.77
chrX	3485001	3486009	CG10803	501	20.5028	4.77
chrX	3670927	3671721	Parg	18	16.0163	4.26
chrX	3719261	3720597	Rala	29	18.3414	4.05
chrX	3719261	3720597	Tlk	400	18.3414	4.05
chrX	3742057	3744163	Tlk	3278	30.5465	5.24
chrX	3779696	3780340	Tlk	198	17.5015	4.56
chrX	3880648	3881320	VhaAC39-1	393	20.3253	4.96
chrX	3949028	3950290	Vap-33A	770	11.637	3.60
chrX	3964820	3965450	lva	88	20.0324	4.39
chrX	4103570	4104866	Xpac	138	12.7367	3.49
chrX	4103570	4104866	brn	282	12.7367	3.49
chrX	4105827	4107456	cib	58	19.866	4.03
chrX	4116247	4118314	Tip60	469	18.3175	3.76
chrX	4116247	4118314	tyf	399	18.3175	3.76
chrX	4116247	4118314	Tip60	24	18.3175	3.76
chrX	4116247	4118314	tyf	131	18.3175	3.76
chrX	4321828	4323301	mRpL33	414	10.9124	3.02
chrX	4321828	4323301	norpA	20	10.9124	3.02
chrX	4411458	4411808	bi	1259	14.9821	4.21
chrX	4426827	4427327	bi	83	19.7391	4.33
chrX	4540231	4541407	rb	451	24.7879	4.92
chrX	4540231	4541407	CHOp24	170	24.7879	4.92
chrX	4551523	4552191	CG11436	128	13.5572	3.78
chrX	4551523	4552191	CG3527	222	13.5572	3.78
chrX	4553682	4554348	CG11444	18	10.346	3.32
chrX	4649510	4650326	Torsin	69	10.2275	3.46
chrX	4649510	4650326	Cbp80	250	10.2275	3.46
chrX	4649510	4650326	Torsin	268	10.2275	3.46
chrX	4649510	4650326	Cbp80	285	10.2275	3.46
chrX	4687952	4688478	ctp	64	15.7576	3.64
chrX	4908940	4910941	CG32772	1116	10.9124	3.02
chrX	4929884	4930909	CG44774	237	15.8322	4.22

chrX	4939049	4939730	Ptp4E	72	9.91295	3.07
chrX	5048116	5048385	ovo	7	15.6189	4.30
chrX	5082021	5084870	CG32767	124	30.1444	5.74
chrX	5085610	5085942	rg	43	13.4519	3.76
chrX	5085610	5085942	CG15465	43	13.4519	3.76
chrX	5085610	5085942	rg	43	13.4519	3.76
chrX	5311862	5312468	TrxT	398	13.2685	3.90
chrX	5311862	5312468	dhd	67	13.2685	3.90
chrX	5322172	5322884	Rnp4F	64	15.515	4.07
chrX	5328859	5329475	CG3309	130	16.2668	4.40
chrX	5410537	5411213	NAAT1	32	16.8387	4.47
chrX	5419663	5421100	spoon	186	56.23	8.25
chrX	5429931	5430766	CG15784	67	66.7423	9.61
chrX	5516592	5518272	CG12730	221	13.2179	3.49
chrX	5516592	5518272	CR45518	21	13.2179	3.49
chrX	5560518	5561018	Vsx2	497	14.3258	3.63
chrX	5622023	5622790	mir-3645	127	13.9434	3.55
chrX	5622023	5622790	Mlc-c	250	13.9434	3.55
chrX	5622023	5622790	l(1)G0045	358	13.9434	3.55
chrX	5665657	5666677	Sirt4	197	26.1451	5.00
chrX	5665657	5666677	CG4119	86	26.1451	5.00
chrX	5671131	5671948	Rab18	146	33.0101	5.43
chrX	5672411	5673260	RpL35	66	29.4241	5.10
chrX	5689125	5689559	CG3160	134	13.9783	3.66
chrX	5689853	5691300	cv	212	23.1987	5.33
chrX	5689853	5691300	CR45525	959	23.1987	5.33
chrX	5705476	5706249	CG4096	6561	15.6189	4.30
chrX	5737178	5738247	CG32758	42	26.3075	4.96
chrX	5745491	5747422	IntS6	126	21.5745	4.01
chrX	5747676	5748431	CG4078	199	9.95205	3.27
chrX	5754582	5756457	lin-52	798	33.8227	5.94
chrX	5754582	5756457	CG15771	9	33.8227	5.94
chrX	5763379	5765082	MAPk-Ak2	62	24.0119	4.83
chrX	5763379	5765082	CG42699	411	24.0119	4.83
chrX	5879415	5880906	Usp30	582	16.2668	3.52
chrX	5879415	5880906	CG16721	164	16.2668	3.52
chrX	5899186	5901144	CR45668	903	31.0886	5.55
chrX	5899186	5901144	Act5C	32	31.0886	5.55
chrX	5901701	5902282	Act5C	38	33.0896	5.03
chrX	5904339	5904735	CG4020	1189	21.7352	4.69
chrX	5907930	5908629	CG12236	58	16.2668	4.40
chrX	6036762	6038338	rux	3	13.5744	3.62
chrX	6036762	6038338	MCTS1	632	13.5744	3.62
chrX	6081149	6081630	CG4660	48	10.8995	3.53
chrX	6224014	6225127	sqh	205	16.3829	3.81
chrX	6225545	6226075	CG14446	142	16.1486	3.72
chrX	6254523	6256242	Rbcn-3A	42	14.9177	4.06
chrX	6254523	6256242	Spx	376	14.9177	4.06
chrX	6267684	6268502	Spt6	148	21.0887	4.06
chrX	6279055	6279676	Ubi-p5E	36	15.9106	3.54
chrX	6287511	6288671	Top3beta	223	25.4332	4.68
chrX	6287511	6288671	wuho	114	25.4332	4.68
chrX	6290983	6291782	Rpt4	281	11.7447	3.41
chrX	6290983	6291782	CG15896	224	11.7447	3.41
chrX	6346293	6347582	CG3847	67	22.8018	4.92
chrX	6351904	6352670	kdn	43	20.5412	4.50

chrX	6372426	6373214	PpV	82	14.1035	3.80
chrX	6524098	6524889	CG42340	272	14.3584	3.50
chrX	6524098	6524889	CG3918	132	14.3584	3.50
chrX	6532567	6533470	RpL7A	38	20.9605	4.40
chrX	6533973	6534653	dx	263	14.3784	3.75
chrX	6590681	6591163	Pat1	219	17.6365	4.07
chrX	6653909	6655295	CG3226	567	12.286	3.43
chrX	6653909	6655295	Cdc7	137	12.286	3.43
chrX	6660790	6662462	CG3224	313	26.1028	4.74
chrX	6660790	6662462	Ctr1A	129	26.1028	4.74
chrX	6677557	6678892	ND-ASHI	71	25.7891	5.28
chrX	6677557	6678892	l(1)G0255	333	25.7891	5.28
chrX	6697891	6698628	CG14440	95	26.425	5.34
chrX	6699181	6699768	CG14441	59	39.0495	5.89
chrX	6727146	6727477	CG3168	16	10.4114	3.15
chrX	6743298	6744438	snoRNA:Psi28S-1232	360	22.6589	4.69
chrX	6743298	6744438	RpL17	4	22.6589	4.69
chrX	6756071	6756801	CG14438	244	11.7823	3.37
chrX	6770663	6771833	COQ7	28	13.6339	3.69
chrX	6820319	6821550	Nf-YC	169	17.2519	4.04
chrX	6820319	6821550	xit	265	17.2519	4.04
chrX	6824181	6824890	nonC	193	9.42371	3.00
chrX	6843120	6844913	CG14435	544	25.4297	5.61
chrX	6843120	6844913	CG4557	125	25.4297	5.61
chrX	6849911	6850338	CG4558	352	10.5716	3.44
chrX	6868022	6868897	CG43736	40	18.2727	3.67
chrX	6968197	6968806	CG32732	219	12.7209	3.64
chrX	6980924	6981759	ogre	72	12.5492	3.66
chrX	6998389	6999106	Inx2	57	25.7081	5.50
chrX	7008518	7008933	cm	2438	10.6603	3.40
chrX	7010719	7012216	cm	165	16.3496	4.34
chrX	7010719	7012216	CG42308	376	16.3496	4.34
chrX	7010719	7012216	CG32736	376	16.3496	4.34
chrX	7010719	7012216	CG42308	456	16.3496	4.34
chrX	7010719	7012216	CG32736	456	16.3496	4.34
chrX	7097697	7098289	Sxl	60	38.0315	6.30
chrX	7101455	7102446	CG4615	180	51.236	7.29
chrX	7109686	7110437	fz4	217	22.6538	5.04
chrX	7285580	7286974	CG1677	20	19.6858	4.10
chrX	7285580	7286974	CG2059	182	19.6858	4.10
chrX	7290832	7291422	unc-119	99	14.2987	3.53
chrX	7300905	7301376	brk	6766	25.5319	5.38
chrX	7323408	7323902	Atg5	59	15.515	4.07
chrX	7696447	7696902	CHES-1-like	25	17.0945	4.25
chrX	7706772	7707363	CR43287	621	11.6023	3.48
chrX	7728026	7729355	NELF-B	184	16.721	3.78
chrX	7728026	7729355	CG12155	577	16.721	3.78
chrX	7737265	7737958	Tom40	183	12.4662	3.76
chrX	7888213	7888901	CG10932	156	18.9699	4.70
chrX	7900874	7902420	snoRNA:Or-aca2	800	27.5071	5.20
chrX	7900874	7902420	RpS6	672	27.5071	5.20
chrX	7900874	7902420	bys	124	27.5071	5.20
chrX	7920045	7921104	hdm	302	16.921	4.49
chrX	7920045	7921104	Ykt6	25	16.921	4.49
chrX	7930088	7931455	CG10777	125	24.0119	4.83
chrX	7932752	7934130	CG10778	226	15.0627	3.69

chrX	7932752	7934130	RpS14a	635	15.0627	3.69
chrX	7967600	7968374	sws	319	11.9324	3.35
chrX	8058444	8059189	fs(1)h	471	11.421	3.38
chrX	8059686	8062929	fs(1)h	175	13.9973	3.47
chrX	8059686	8062929	mys	33	13.9973	3.47
chrX	8074919	8075982	Upf2	14	13.9778	3.95
chrX	8074919	8075982	CG1571	424	13.9778	3.95
chrX	8092663	8094061	UbcE2H	233	12.3103	3.60
chrX	8114753	8116235	Gclc	106	20.8124	4.69
chrX	8120500	8121185	CG2260	184	20.5412	4.50
chrX	8141414	8142406	Smox	446	42.0974	6.76
chrX	8143030	8143640	CG17982	231	9.17076	3.07
chrX	8152763	8153290	CG18262	21	14.9618	3.82
chrX	8156596	8158152	Traf6	97	19.6995	4.45
chrX	8156596	8158152	GIIIspla2	144	19.6995	4.45
chrX	8176223	8176970	slpr	626	17.707	4.14
chrX	8193091	8193544	sdt	79	23.8306	5.40
chrX	8242201	8244468	Trxr-1	101	15.5555	3.56
chrX	8242201	8244468	sni	732	15.5555	3.56
chrX	8242201	8244468	Trxr-1	953	15.5555	3.56
chrX	8242201	8244468	sni	1388	15.5555	3.56
chrX	8242201	8244468	Trxr-1	1560	15.5555	3.56
chrX	8251101	8251714	ND-75	26	17.5015	4.56
chrX	8294939	8296040	CG15343	21699	10.7958	3.82
chrX	8398582	8399675	CG10555	147	20.175	4.36
chrX	8408917	8410860	Trf2	67	29.5906	5.71
chrX	8408917	8410860	lawc	760	29.5906	5.71
chrX	8436414	8438193	CG12125	149	21.7352	4.69
chrX	8436414	8438193	CG1440	404	21.7352	4.69
chrX	8436414	8438193	CG12125	1168	21.7352	4.69
chrX	8461565	8461921	Es2	231	9.14444	3.27
chrX	8463951	8464790	Sptr	84	10.8995	3.53
chrX	8517233	8517967	Nrg	18	32.4667	5.27
chrX	8558366	8558939	IntS4	122	43.0198	7.23
chrX	8559743	8562488	CG12112	1718	22.2165	4.72
chrX	8559743	8562488	Trf4-1	216	22.2165	4.72
chrX	8574177	8575339	CG12065	126	31.0275	4.87
chrX	8591155	8591783	CG12659	121	29.752	4.98
chrX	8592040	8592934	Crag	205	12.1588	3.56
chrX	8650559	8651489	oc	282	39.6378	6.45
chrX	8676747	8677568	CG12772	137	27.4579	4.87
chrX	8681302	8682267	CG11284	65	32.4478	6.44
chrX	8714325	8715492	CG2004	59	24.5682	5.24
chrX	8714325	8715492	CG1785	48	24.5682	5.24
chrX	8720045	8721310	l(1)G0020	428	18.94	4.77
chrX	8720045	8721310	CG1789	186	18.94	4.77
chrX	8805643	8806142	Lim1	170	15.8056	3.74
chrX	8806561	8807016	Lim1	958	15.224	3.94
chrX	8863686	8864339	CG12075	30	17.5838	4.58
chrX	8891448	8891965	Moe	58	19.6514	4.79
chrX	8899856	8900384	CG1885	155	17.898	4.44
chrX	8924676	8925115	rdgA	291	9.80303	3.34
chrX	9029851	9030503	Nost	31	19.8903	4.42
chrX	9042212	9042948	Nost	19	16.4087	4.29
chrX	9080778	9081541	CG7246	382	10.6871	3.26
chrX	9080778	9081541	HP1b	186	10.6871	3.26

chrX	9085843	9086338	CG32708	208	12.3339	3.55
chrX	9089131	9090186	CG7033	77	24.9047	4.58
chrX	9103277	9103802	CG7766	148	21.1032	4.37
chrX	9107008	9107597	Arfrp1	190	17.7622	4.48
chrX	9148658	9149871	fh	430	27.7043	5.71
chrX	9148658	9149871	Bap111	193	27.7043	5.71
chrX	9155496	9156699	His3.3B	163	21.5849	4.20
chrX	9163167	9164428	mei-P26	367	13.5509	3.52
chrX	9196050	9196509	CG12118	193	14.9821	4.21
chrX	9247457	9247938	Dsor1	3	10.9352	3.48
chrX	9250361	9251078	CG17754	133	18.7624	4.25
chrX	9282382	9283295	c11.1	51	30.8649	5.90
chrX	9318653	9319810	c12.1	294	22.3255	4.33
chrX	9318653	9319810	c12.2	159	22.3255	4.33
chrX	9355690	9357106	CG34449	60	14.8323	3.51
chrX	9355690	9357106	BCL7-like	466	14.8323	3.51
chrX	9553259	9553968	l(1)G0320	73	69.4842	8.20
chrX	9554425	9555234	RpS28b	151	23.2554	4.34
chrX	9598841	9601699	Gga	212	22.6127	4.55
chrX	9608137	9609450	Aats-lys	249	30.571	5.38
chrX	9608137	9609450	CG42797	188	30.571	5.38
chrX	9640111	9641118	Ptpmeg2	98	10.8826	3.41
chrX	9658228	9659121	Ptpmeg2	327	15.9777	3.73
chrX	9673106	9674337	CG3106	11142	14.8323	3.42
chrX	9684689	9686065	nej	103	11.0762	3.21
chrX	9693726	9694165	btd	237	17.5212	4.10
chrX	9778524	9779223	CG1354	49	33.3526	5.85
chrX	9865036	9865503	Ser7	35	10.2275	3.46
chrX	9884373	9884807	CG9691	70	13.9778	3.95
chrX	10081926	10082547	ZAP3	149	11.9836	3.57
chrX	10083133	10083624	CG2972	206	20.0324	4.39
chrX	10085571	10086873	CG2974	410	19.1053	4.38
chrX	10085571	10086873	RhoU	55	19.1053	4.38
chrX	10220023	10220443	CG34408	110	17.2105	4.15
chrX	10325330	10326253	alpha-Man-Ia	75	22.6095	4.67
chrX	10326579	10326841	CG2909	284	8.93717	3.16
chrX	10472601	10475226	nocte	686	14.2928	3.44
chrX	10472601	10475226	CG2889	1036	14.2928	3.44
chrX	10472601	10475226	nocte	1774	14.2928	3.44
chrX	10472601	10475226	CG2889	1814	14.2928	3.44
chrX	10486169	10486981	CG32687	224	13.9434	3.55
chrX	10546519	10546966	spri	34	24.6971	4.90
chrX	10735622	10736122	ras	8664	32.5898	5.77
chrX	10744063	10745734	ras	1026	40.1538	4.82
chrX	10744063	10745734	CR44766	1020	40.1538	4.82
chrX	10744063	10745734	CG32676	584	40.1538	4.82
chrX	10744063	10745734	ras	94	40.1538	4.82
chrX	10757433	10758162	Rph	41	13.734	4.02
chrX	10763227	10764512	Tango5	42	22.4665	4.16
chrX	10767241	10769037	Atg8a	155	24.3336	4.65
chrX	10767241	10769037	BTBD9	353	24.3336	4.65
chrX	10772176	10772728	CG15211	254	14.7377	4.08
chrX	10783668	10784728	sesB	15	12.8698	3.33
chrX	10785648	10787000	sesB	618	27.1138	4.99
chrX	10785648	10787000	Ant2	618	27.1138	4.99
chrX	10785648	10787000	sesB	618	27.1138	4.99

chrX	10846489	10847714	sbr	222	12.3502	3.27
chrX	10846489	10847714	CG17333	567	12.3502	3.27
chrX	10848682	10849361	CG2202	208	29.3303	5.73
chrX	10857152	10858655	sofe	648	18.4788	3.97
chrX	10857152	10858655	CG2186	405	18.4788	3.97
chrX	10927308	10927721	CG2145	48	12.5236	3.84
chrX	11086024	11086476	sev	44	14.9821	4.21
chrX	11107962	11109226	Hsp60	164	39.7015	6.15
chrX	11125134	11125603	Rpt3	148	16.7534	4.05
chrX	11128293	11129197	Gtp-bp	54	23.1987	5.33
chrX	11135944	11138489	Klp10A	1020	22.4738	4.31
chrX	11135944	11138489	CDK2AP1	280	22.4738	4.31
chrX	11147554	11148702	Ran	182	31.9551	5.13
chrX	11154010	11155287	Dlic	138	18.8066	3.94
chrX	11338987	11340317	Kap3	207	41.2653	6.36
chrX	11338987	11340317	CG34348	491	41.2653	6.36
chrX	11338987	11340317	Kap3	703	41.2653	6.36
chrX	11348999	11349435	CG1738	110	8.42918	3.07
chrX	11349850	11351869	CG11752	664	25.3355	4.97
chrX	11349850	11351869	CG1737	54	25.3355	4.97
chrX	11355852	11356484	dsh	363	13.8063	3.67
chrX	11367505	11368629	hop	68	19.8195	4.54
chrX	11367505	11368629	Tim8	414	19.8195	4.54
chrX	11389615	11390390	dlg1	119	11.8392	3.20
chrX	11423419	11424279	Gs2	67	24.98	5.18
chrX	11426191	11427744	Gs2	3514	28.5116	5.90
chrX	11453816	11454479	Evi5	19	13.1236	3.93
chrX	11454814	11455318	HP5	164	11.3549	3.65
chrX	11569756	11570879	RpII215	40	33.5262	5.66
chrX	11569756	11570879	CG11699	334	33.5262	5.66
chrX	11580132	11581084	nod	388	14.7954	3.84
chrX	11599797	11600059	rho-4	45	11.3549	3.65
chrX	11610923	11612453	CG2247	273	19.1053	4.38
chrX	11610923	11612453	CG1703	547	19.1053	4.38
chrX	11621380	11622423	Ptp10D	65	27.1453	5.13
chrX	11694936	11695760	PhKgamma	142	27.3397	4.91
chrX	11709639	11710445	FucT6	10	16.4711	3.93
chrX	11720142	11720952	Tango10	44	14.587	4.04
chrX	11791850	11793355	CkIIbeta	24	25.5021	4.93
chrX	11804999	11806576	Hsc70-3	31	19.565	3.98
chrX	11815697	11818595	CG1578	72	21.6098	4.73
chrX	11815697	11818595	rudhira	418	21.6098	4.73
chrX	11852133	11852647	p24-1	216	12.3195	3.44
chrX	11854363	11855698	CG10347	174	15.8278	4.02
chrX	11854363	11855698	ATP7	84	15.8278	4.02
chrX	11886854	11887607	CG10353	14	14.4125	3.70
chrX	11886854	11887607	SelG	135	14.4125	3.70
chrX	12003649	12004198	fw	57	11.2909	3.63
chrX	12018564	12019041	CG15735	221	17.678	4.08
chrX	12028290	12029128	Usp7	373	14.6472	4.05
chrX	12031013	12031480	Cyp311a1	169	10.1855	3.23
chrX	12203601	12204031	Ten-a	42	9.85778	3.15
chrX	12463442	12464156	CG42258	73	16.614	4.27
chrX	12579004	12579590	CG11138	3956	16.9084	4.21
chrX	12582618	12583873	CG11138	170	23.4245	4.05
chrX	12595040	12596797	CG1824	119	32.6478	5.71

chrX	12595040	12596797	Sec16	541	32.6478	5.71
chrX	12610907	12612700	CG1463	354	12.7164	3.26
chrX	12610907	12612700	CG2543	155	12.7164	3.26
chrX	12631517	12632304	Tomosyn	155	23.0519	4.58
chrX	12658946	12659684	Tis11	84	12.0224	3.20
chrX	12658946	12659684	CR33963	37	12.0224	3.20
chrX	12665185	12665555	Tis11	4810	9.74536	3.12
chrX	12735098	12736400	CR43963	4931	17.835	4.23
chrX	12753674	12754711	CR44619	2210	15.9634	4.11
chrX	12755039	12756002	CR44619	3851	13.6339	3.69
chrX	12764764	12765502	CG3812	54	19.0922	4.65
chrX	12902779	12903646	CG4407	120	21.3118	4.41
chrX	12905267	12906120	CG4404	226	21.4109	4.03
chrX	12909182	12909685	Brms1	200	9.43051	3.19
chrX	12909995	12910455	CG4645	246	19.1801	4.40
chrX	13089785	13090533	hep	158	17.6365	3.81
chrX	13091037	13091353	lic	7	11.3009	3.51
chrX	13112200	13112717	CG43313	42	13.734	4.02
chrX	13128036	13129380	snmRNA:204	135	33.7484	5.78
chrX	13128036	13129380	CG32638	29	33.7484	5.78
chrX	13128036	13129380	CG15717	186	33.7484	5.78
chrX	13128036	13129380	MFS10	252	33.7484	5.78
chrX	13128036	13129380	CG15717	252	33.7484	5.78
chrX	13130978	13131972	MFS10	131	26.3442	5.40
chrX	13154789	13155267	CG4318	85	12.1405	3.45
chrX	13154789	13155267	CG4332	28	12.1405	3.45
chrX	13159099	13160138	Bap60	60	13.9434	3.55
chrX	13159099	13160138	CG12096	35	13.9434	3.55
chrX	13211088	13211746	sno	62	17.7622	4.48
chrX	13212245	13212615	REG	74	17.5838	4.58
chrX	13285954	13286447	CG15743	42	18.94	4.77
chrX	13287893	13288667	CG15744	177	22.9185	4.61
chrX	13296358	13297611	CG1764	668	22.553	5.09
chrX	13296358	13297611	CG1622	238	22.553	5.09
chrX	13314899	13315191	IP3K2	67	7.0378	3.08
chrX	13329605	13330243	Jafrac1	47	29.3214	5.15
chrX	13337058	13337842	RpS15Aa	110	25.8329	4.58
chrX	13338231	13338765	CG15747	138	10.7044	3.42
chrX	13383912	13385665	Rbp1-like	461	14.5114	3.73
chrX	13383912	13385665	Tim9a	793	14.5114	3.73
chrX	13386822	13388275	Yippee	191	29.3896	5.66
chrX	13386822	13388275	CG1662	336	29.3896	5.66
chrX	13403830	13405012	GstT4	84	18.94	4.77
chrX	13412643	13413726	CG1998	197	31.5662	6.24
chrX	13412643	13413726	Set2	30	31.5662	6.24
chrX	13563248	13564185	Tango13	248	24.3799	4.72
chrX	13615840	13616753	tth	145	21.8608	4.65
chrX	13623825	13625645	CG2691	386	24.1136	4.55
chrX	13623825	13625645	NFAT	50	24.1136	4.55
chrX	13717917	13718382	CG9940	186	17.3931	4.32
chrX	13735977	13736419	g	33	9.01856	3.13
chrX	13739567	13740433	CG11151	2	33.2876	5.48
chrX	13754601	13755544	CR43833	49	13.4499	3.25
chrX	13754601	13755544	Pdcd4	36	13.4499	3.25
chrX	13757107	13757613	Rtc1	111	18.642	3.95
chrX	13762579	13763105	rdgB	46	15.3789	3.57

chrX	13781639	13782336	CtsB1	279	13.7206	3.84
chrX	13783434	13784669	inaE	134	23.2554	4.34
chrX	13821517	13822469	Clic	75	18.642	3.95
chrX	13829686	13830329	jub	227	12.8577	3.42
chrX	13933860	13934265	mamo	740	13.734	4.02
chrX	14210944	14211630	l(1)G0469	59	9.42371	3.00
chrX	14233541	14234184	CG42271	142	20.5412	4.50
chrX	14237750	14238705	Fbxl4	561	9.31717	3.26
chrX	14237750	14238705	CG1434	170	9.31717	3.26
chrX	14580449	14580928	CG14414	70	23.2683	5.18
chrX	14582662	14583072	mRpS25	84	11.0212	3.15
chrX	14825521	14826280	rut	17	27.8952	5.50
chrX	14831627	14832168	CG14408	32	19.6294	4.87
chrX	14832509	14833415	CG14411	205	39.1945	7.20
chrX	14837694	14840285	CG14407	1194	14.4125	3.70
chrX	14837694	14840285	CG32590	286	14.4125	3.70
chrX	14837694	14840285	Flo2	17	14.4125	3.70
chrX	14950403	14950870	pdgy	84	17.835	4.23
chrX	14955201	14955726	eag	89	13.734	4.02
chrX	15063254	15064365	hiw	679	18.7614	4.08
chrX	15063254	15064365	ND-B18	190	18.7614	4.08
chrX	15066033	15066465	opm	183	10.2275	3.46
chrX	15074575	15076352	Lsd-2	897	10.8909	3.30
chrX	15082648	15083080	CG33178	47	18.4659	4.30
chrX	15083978	15086466	CG9065	1585	23.8946	4.87
chrX	15083978	15086466	CG15027	457	23.8946	4.87
chrX	15083978	15086466	CG5599	160	23.8946	4.87
chrX	15138720	15139829	RpL37a	150	27.0049	5.03
chrX	15311821	15312278	CR44961	5851	10.2209	3.29
chrX	15313702	15314447	CR44961	3745	17.6866	4.26
chrX	15319316	15321085	Top1	487	15.8498	3.70
chrX	15319316	15321085	mir-4957	59	15.8498	3.70
chrX	15319316	15321085	Top1	390	15.8498	3.70
chrX	15323091	15323885	Top1	175	35.968	5.69
chrX	15333214	15334175	HDAC6	98	12.3916	3.20
chrX	15449127	15450690	Scamp	461	20.175	4.36
chrX	15449127	15450690	Ahcy13	84	20.175	4.36
chrX	15453271	15453852	CG11655	123	20.5698	4.78
chrX	15462065	15463140	shtd	418	28.7812	5.46
chrX	15462065	15463140	CG6299	138	28.7812	5.46
chrX	15462065	15463140	CG6294	138	28.7812	5.46
chrX	15465413	15465885	CG6299	72	12.1432	3.35
chrX	15471533	15472597	Grip128	193	10.3937	3.34
chrX	15471533	15472597	CG6308	149	10.3937	3.34
chrX	15498810	15499645	Gmap	72	10.346	3.32
chrX	15577855	15578643	cerv	205	15.0627	3.69
chrX	15577855	15578643	CG7872	172	15.0627	3.69
chrX	15582284	15583202	CG9215	330	11.6551	3.39
chrX	15582284	15583202	CG8097	614	11.6551	3.39
chrX	15593367	15593711	CG9220	10	15.224	3.94
chrX	15697403	15698460	CG8128	205	11.8019	3.28
chrX	15702997	15704465	Pis	207	18.1375	4.17
chrX	15702997	15704465	CG8134	561	18.1375	4.17
chrX	15709645	15711374	CG9281	48	49.8226	7.57
chrX	15709645	15711374	CG15601	155	49.8226	7.57
chrX	15732199	15732450	CG8191	128	10.1855	3.23

chrX	15733318	15734617	CG12379	248	10.346	3.32
chrX	15733318	15734617	l(1)G0136	25	10.346	3.32
chrX	15738545	15739907	CG11679	359	18.7546	4.19
chrX	15738545	15739907	CG8206	84	18.7546	4.19
chrX	15745745	15746291	CG8944	231	12.8637	3.51
chrX	15746656	15747567	Tcp-1zeta	224	27.7043	5.71
chrX	15749039	15749460	CG8239	90	23.5836	4.68
chrX	15761124	15761533	mRpL3	27	15.7165	3.88
chrX	15762856	15763418	CG17209	361	8.80992	3.18
chrX	15785443	15786434	Paf-AHalpha	636	10.5272	3.32
chrX	15785443	15786434	mRpS30	173	10.5272	3.32
chrX	15857271	15857885	Myb	112	15.635	3.76
chrX	15859979	15860918	Gbeta13F	199	22.4738	4.31
chrX	15868050	15868767	Gapdh2	62	27.8259	4.76
chrX	15909405	15909886	CycD	125	10.3036	3.60
chrX	15986435	15986830	ND-20	131	16.614	4.27
chrX	15996969	15997499	CG8939	200	14.7199	3.72
chrX	16104496	16105080	CG42353	43	36.0359	5.64
chrX	16104496	16105080	CG42354	43	36.0359	5.64
chrX	16104496	16105080	CG42353	43	36.0359	5.64
chrX	16244768	16245105	snRNA:U5:14B	9190	31.686	6.36
chrX	16275391	16276774	kat80	259	33.9995	6.23
chrX	16275391	16276774	Prosalpha4	170	33.9995	6.23
chrX	16277908	16278616	eas	57	23.3237	4.51
chrX	16286184	16286814	UQCR-14	45	19.6294	4.87
chrX	16287923	16289401	CG32576	263	13.4499	3.25
chrX	16287923	16289401	caz	539	13.4499	3.25
chrX	16296181	16297368	CG9911	99	11.6319	3.20
chrX	16296181	16297368	CG3632	182	11.6319	3.20
chrX	16296181	16297368	CG9911	397	11.6319	3.20
chrX	16296181	16297368	CG3632	594	11.6319	3.20
chrX	16319276	16320553	Cyp1	85	24.0787	5.21
chrX	16325605	16326067	CalpC	62	9.27259	3.25
chrX	16331597	16332077	CG9921	223	20.6778	4.47
chrX	16384775	16385919	CG9992	580	19.6294	4.87
chrX	16384775	16385919	CG4239	11	19.6294	4.87
chrX	16391007	16392010	TH1	401	12.5236	3.84
chrX	16391007	16392010	mei-41	142	12.5236	3.84
chrX	16421705	16423804	AnxB11	41	34.0898	5.31
chrX	16421705	16423804	hang	629	34.0898	5.31
chrX	16440750	16442524	CG9947	407	11.0265	3.08
chrX	16440750	16442524	rngo	151	11.0265	3.08
chrX	16446551	16447194	eIF-2alpha	119	16.148	3.97
chrX	16447957	16449618	CG34015	598	36.1934	6.10
chrX	16447957	16449618	Rbp2	236	36.1934	6.10
chrX	16552784	16553892	Arp2	830	10.9184	3.22
chrX	16558994	16560123	Pp2B-14D	218	34.8939	6.00
chrX	16593032	16594342	SMC3	503	16.2668	4.40
chrX	16593032	16594342	Ubc7	266	16.2668	4.40
chrX	16597648	16598587	CG9784	53	11.1508	3.14
chrX	16599058	16600135	CG9784	168	24.5309	4.46
chrX	16599058	16600135	Nup153	143	24.5309	4.46
chrX	16610579	16611285	mbt	69	15.3692	3.48
chrX	16636708	16638735	Rok	144	48.7269	7.17
chrX	16636708	16638735	RpS19a	623	48.7269	7.17
chrX	16640778	16641738	mthl1	33	18.9645	4.41

chrX	16751315	16752129	goe	32	25.4047	5.61
chrX	16783091	16784459	if	722	18.8924	4.61
chrX	16783091	16784459	mRpL22	224	18.8924	4.61
chrX	16787725	16788373	Sap30	159	13.8063	3.67
chrX	16792027	16793303	CG4768	25	12.5236	3.84
chrX	16803039	16803361	CG9125	170	8.98547	3.07
chrX	16930676	16931525	CG4928	6136	11.9344	3.74
chrX	17107376	17107984	CG5004	73	31.7752	5.76
chrX	17147704	17148622	RpS5a	69	48.6746	7.08
chrX	17148970	17149444	CG5010	84	21.0332	5.05
chrX	17301567	17302004	Fim	45	18.1543	4.01
chrX	17302391	17303294	CG5445	165	39.7343	6.08
chrX	17314192	17314898	B-H2	142	10.7257	3.54
chrX	17397212	17397579	B-H1	74	13.1236	3.93
chrX	17423396	17423887	CG5613	26	11.0619	3.40
chrX	17506756	17507270	CG43658	43	15.8056	3.74
chrX	17579669	17580439	CG12991	66	15.477	4.27
chrX	17635504	17636849	CG8289	433	28.7812	5.46
chrX	17635504	17636849	CG5800	197	28.7812	5.46
chrX	17651089	17652660	RhoGAPp190	149	17.2322	3.74
chrX	17656398	17656883	IntS2	169	20.6165	4.28
chrX	17657294	17657675	beta-Spec	136	20.6778	4.47
chrX	17694522	17694885	chas	275	10.7044	3.42
chrX	17698187	17698897	chas	73	33.2663	5.99
chrX	17702326	17703065	CG8188	61	11.8107	3.71
chrX	17738162	17739275	CG42684	109	11.3549	3.65
chrX	17768094	17768686	unc-4	243	31.4721	5.35
chrX	17826535	17827759	Socs16D	31	47.3886	7.56
chrX	17830693	17831183	CG6398	97	16.921	4.49
chrX	17830693	17831183	CR44691	170	16.921	4.49
chrX	17844528	17845606	CG8142	21	12.0148	3.42
chrX	17844528	17845606	Ucp4A	265	12.0148	3.42
chrX	17849802	17850709	CG15814	229	18.2484	4.38
chrX	17849802	17850709	Spt7	346	18.2484	4.38
chrX	17860458	17861547	Arp8	530	11.6191	3.28
chrX	17860458	17861547	CG6769	329	11.6191	3.28
chrX	17886468	17887499	mnb	159	27.5781	5.36
chrX	17907571	17908146	Vps4	110	17.5838	4.58
chrX	17911330	17911680	CG6847	54	14.6738	3.61
chrX	18084872	18086217	CG7536	5	21.5521	4.72
chrX	18084872	18086217	Ada3	358	21.5521	4.72
chrX	18090099	18090544	CG7206	139	10.4692	3.08
chrX	18091257	18091748	scu	171	15.5517	3.90
chrX	18100913	18101429	CG7192	74	41.2947	6.78
chrX	18101916	18102449	Taf8	203	26.1898	5.71
chrX	18136460	18137961	ari-1	191	29.3858	5.29
chrX	18320873	18321263	CG6023	168	9.13261	3.27
chrX	18370678	18371763	CrebB	130	14.4751	3.52
chrX	18483484	18484105	Hayan	44	31.5825	6.15
chrX	18489278	18489838	CG15046	17	9.08306	3.15
chrX	18499471	18500235	GalNAc-T2	136	21.8876	4.41
chrX	18499471	18500235	Rad51D	390	21.8876	4.41
chrX	18512162	18512614	Ggt-1	60	11.2548	3.29
chrX	18515251	18516205	Aats-his	44	19.2473	4.35
chrX	18515251	18516205	Bx	44	19.2473	4.35
chrX	18515251	18516205	Aats-his	44	19.2473	4.35

chrX	18646817	18649003	Rip11	108	25.041	4.55
chrX	18646817	18649003	CG6540	722	25.041	4.55
chrX	18652596	18653209	Ing3	42	9.2139	3.18
chrX	18787906	18789682	CG6961	508	17.3003	3.84
chrX	18787906	18789682	S6KL	255	17.3003	3.84
chrX	18790091	18790445	S6KL	35	13.0113	3.77
chrX	18793645	18794103	Atg101	2092	10.5272	3.32
chrX	18830509	18831061	CG7101	111	15.9106	3.54
chrX	18843359	18844504	CG34422	89	27.2259	4.95
chrX	18848029	18849062	CG7280	43	15.224	3.94
chrX	18848029	18849062	CG34422	490	15.224	3.94
chrX	18863578	18864340	CG7326	281	16.5217	3.79
chrX	18863578	18864340	CG34401	108	16.5217	3.79
chrX	18893074	18893738	CG7358	105	14.587	4.04
chrX	19152939	19154057	RhoGAP18B	63	37.1354	6.64
chrX	19261710	19262624	Vav	171	18.258	4.68
chrX	19268387	19268960	CG8010	199	10.0988	3.07
chrX	19276384	19276872	CG32537	84	17.985	4.20
chrX	19353050	19353652	kek5	149	22.2268	5.10
chrX	19478790	19481074	CG32533	1291	29.6609	5.72
chrX	19478790	19481074	mRpS14	785	29.6609	5.72
chrX	19478790	19481074	Nat1	719	29.6609	5.72
chrX	19478790	19481074	mRpS14	464	29.6609	5.72
chrX	19478790	19481074	Nat1	444	29.6609	5.72
chrX	19491616	19493015	pcm	614	24.3799	4.72
chrX	19491616	19493015	ND-18	205	24.3799	4.72
chrX	19494169	19497208	CG12204	1304	20.3191	3.81
chrX	19494169	19497208	ksh	713	20.3191	3.81
chrX	19494169	19497208	CG14200	201	20.3191	3.81
chrX	19494169	19497208	Pfrx	201	20.3191	3.81
chrX	19494169	19497208	CR44340	191	20.3191	3.81
chrX	19494169	19497208	Pfrx	969	20.3191	3.81
chrX	19520537	19523122	l(1)G0156	1060	64.8876	9.58
chrX	19520537	19523122	CoRest	772	64.8876	9.58
chrX	19565363	19566669	Grip84	532	19.6885	4.26
chrX	19565363	19566669	car	114	19.6885	4.26
chrX	19578010	19580056	Tao	166	20.4891	3.79
chrX	19597687	19598733	RpS10b	156	29.4783	6.03
chrX	19605073	19606026	CG14207	25	20.2323	4.94
chrX	19613644	19614310	Shawn	142	13.1236	3.93
chrX	19616120	19616783	CG14210	159	17.5561	3.94
chrX	19631256	19631978	CG14212	6086	21.6057	4.47
chrX	19636938	19639220	e(y)3	595	29.3132	4.96
chrX	19636938	19639220	Rcd-1	992	29.3132	4.96
chrX	19662697	19663172	CG14223	433	26.6562	4.84
chrX	19664520	19666029	Ssu72	169	13.6935	4.01
chrX	19667072	19668823	Zw	27	22.4096	4.77
chrX	19667072	19668823	CR44512	468	22.4096	4.77
chrX	19683030	19684456	dome	247	21.9998	4.22
chrX	19683030	19684456	Ubqn	536	21.9998	4.22
chrX	19692909	19693555	Mer	209	16.7602	4.24
chrX	19696913	19697774	Cdc42	168	30.7569	6.24
chrX	19703583	19704722	CG14232	152	17.0945	4.25
chrX	19703583	19704722	CG14230	174	17.0945	4.25
chrX	19703583	19704722	CG14232	258	17.0945	4.25
chrX	19743526	19745093	COX6B	257	31.0824	5.62

chrX	19743526	19745093	CG18809	30	31.0824	5.62
chrX	19743526	19745093	COX6B	30	31.0824	5.62
chrX	19743526	19745093	CG33932	125	31.0824	5.62
chrX	19743526	19745093	Rpp20	125	31.0824	5.62
chrX	19743526	19745093	CG33932	125	31.0824	5.62
chrX	19743526	19745093	Rpp20	125	31.0824	5.62
chrX	19746036	19746913	parvin	154	12.8996	3.26
chrX	19746036	19746913	CR45501	254	12.8996	3.26
chrX	19750682	19751615	Pmp70	135	30.0491	5.89
chrX	19851361	19852480	pico	213	22.0322	4.38
chrX	19851361	19852480	Nup205	59	22.0322	4.38
chrX	19865199	19865609	CG15618	62	21.7445	5.06
chrX	19886894	19887924	Hers	21	47.0308	7.03
chrX	20158932	20160157	Rab35	337	20.6778	4.47
chrX	20235762	20236484	sw	31	12.2069	3.69
chrX	20250580	20252306	CG17068	170	21.1677	4.44
chrX	20250580	20252306	Rab10	414	21.1677	4.44
chrX	20391289	20392079	l(1)G0004	111	25.041	4.55
chrX	20392660	20393842	Syx16	50	11.9344	3.74
chrX	20394221	20394994	HERC2	201	13.3788	3.86
chrX	20423368	20424625	Pp4-19C	74	20.4022	4.35
chrX	20423368	20424625	cactin	346	20.4022	4.35
chrX	20429914	20430592	mal	145	14.8918	3.70
chrX	20468278	20468627	GstT3	82	16.921	4.49
chrX	20510418	20510919	RhoGAP19D	13	16.148	3.97
chrX	20524462	20525233	Tak1	80	23.2908	5.11
chrX	21028011	21028425	CG1532	96	14.8918	3.70
chrX	21031652	21032091	CG1529	90	16.9743	4.43
chrX	21035379	21036027	Ntf-2	259	16.911	4.03
chrX	21042453	21043386	Mgstl	109	19.1053	4.38
chrX	21045067	21045676	Cbs	24	40.4539	6.61
chrX	21052605	21054216	CG1518	754	26.1078	5.35
chrX	21052605	21054216	bves	779	26.1078	5.35
chrX	21116909	21117520	unc	261	18.6029	4.10
chrX	21116909	21117520	CG15445	15	18.6029	4.10
chrX	21280701	21281503	CG32521	43	44.4478	7.55
chrX	21315916	21316352	Rpt6	194	11.8446	3.54
chrX	21318914	21319378	CG33713	17	18.7624	4.25
chrX	21318914	21319378	SLIRP1	17	18.7624	4.25
chrX	21318914	21319378	CG33713	100	18.7624	4.25
chrX	21318914	21319378	SLIRP1	100	18.7624	4.25
chrX	21323102	21324999	Dd	477	20.2357	4.44
chrX	21323102	21324999	CG1486	420	20.2357	4.44
chrX	21339573	21340343	tty	156	19.9533	4.64
chrX	21339573	21340343	flil	92	19.9533	4.64
chrX	21345173	21345848	dod	171	34.1384	6.64
chrX	21350164	21350866	sol	255	20.3253	4.96
chrX	21358190	21358659	44075	61	17.5402	3.63
chrX	21362928	21363546	waw	149	37.9402	6.63
chrX	21362928	21363546	bbx	149	37.9402	6.63
chrX	21373964	21375798	sIgA	60	19.4731	3.87
chrX	21373964	21375798	CR45611	46	19.4731	3.87
chrX	21373964	21375798	sIgA	395	19.4731	3.87
chrX	21387535	21387992	Hlc	111	13.7673	3.56
chrX	21629443	21630100	DIP1	177	10.1212	3.43
chrX	22444671	22445432	CG14621	236	20.3253	4.96

chrX	22454880	22455371	wap	288	11.6551	3.21
chrX	22455661	22457150	Usp2	123	15.9328	3.63
chrX	22471609	22472049	CG14613	217	16.2668	4.40
chrX	22481034	22481509	CG12576	753	20.6311	4.46
chrX	22481769	22482119	CG12576	32	12.0148	3.42
chrX	22481769	22482119	Cp110	86	12.0148	3.42
chrX	22529908	22530389	S6kII	170	13.1236	3.93
chrX	22539726	22540403	CG17598	131	15.882	3.86
chrX	23096302	23096683	CG17162	258	10.8826	3.41
chrX	23097437	23098006	su(f)	239	10.8826	3.41

Supplementary Table 6 Genes showing overlapped binding of CoRest-C, CBP, and Rpd3

Chromosom	Gene Symbol
chr2L	Akap200
chr2L	al
chr2L	Aldh
chr2L	aop
chr2L	Arpc2
chr2L	aru
chr2L	Atet
chr2L	ATPsynG
chr2L	Atxn7
chr2L	B4
chr2L	bowl
chr2L	brat
chr2L	bun
chr2L	capt
chr2L	cbt
chr2L	cdc14
chr2L	CG10188
chr2L	CG10283
chr2L	CG10348
chr2L	CG10492
chr2L	CG10570
chr2L	CG10600
chr2L	CG11050
chr2L	CG13398
chr2L	CG15390
chr2L	CG17078
chr2L	CG17292
chr2L	CG18507
chr2L	CG2818
chr2L	CG31638
chr2L	CG3164
chr2L	CG31668
chr2L	CG31694
chr2L	CG31729
chr2L	CG31743
chr2L	CG31751
chr2L	CG31955
chr2L	CG34164
chr2L	CG34172
chr2L	CG3609
chr2L	CG4036
chr2L	CG4230
chr2L	CG42502
chr2L	CG4935
chr2L	CG5853
chr2L	CG5953
chr2L	CG5973
chr2L	CG6094
chr2L	CG6287
chr2L	CG6700
chr2L	CG6746
chr2L	CG6770
chr2L	CG6785
chr2L	CG7337

chr2L	CG8814
chr2L	CG9171
chr2L	CG9175
chr2L	CG9331
chr2L	CG9932
chr2L	ck
chr2L	CR43314
chr2L	CR44392
chr2L	CR44587
chr2L	CR44911
chr2L	CR44985
chr2L	CR45297
chr2L	CR45341
chr2L	CR45463
chr2L	CR9162
chr2L	crol
chr2L	crp
chr2L	Daxx
chr2L	dia
chr2L	Dif
chr2L	dpp
chr2L	dpy
chr2L	drl
chr2L	drongo
chr2L	ebi
chr2L	eIF-4a
chr2L	Elf
chr2L	emb
chr2L	fok
chr2L	frtz
chr2L	GATAd
chr2L	Gli
chr2L	Hnf4
chr2L	Idgf3
chr2L	JhI-21
chr2L	l(2)gd1
chr2L	lace
chr2L	let-7-C
chr2L	lilli
chr2L	Mondo
chr2L	MRP
chr2L	Msp300
chr2L	mts
chr2L	Myo31DF
chr2L	Ncoa6
chr2L	Nhe3
chr2L	nop5
chr2L	nrv1
chr2L	NTPase
chr2L	oaf
chr2L	p38b
chr2L	papi
chr2L	Pde11
chr2L	Pino
chr2L	Pka-C1
chr2L	Rab5

chr2L	Rab6
chr2L	Reps
chr2L	Rh5
chr2L	Rim2
chr2L	RpL30
chr2L	RpL7-like
chr2L	Rtnl1
chr2L	SC35
chr2L	caRNA:PsiU2-38.40.42
chr2L	Sec61alpha
chr2L	Sirt1
chr2L	Sirup
chr2L	Slh
chr2L	Smg5
chr2L	smo
chr2L	snoRNA:Me28S-U1230
chr2L	Socs36E
chr2L	spen
chr2L	spi
chr2L	Su(dx)
chr2L	Tep2
chr2L	Tep3
chr2L	tup
chr2L	tweek
chr2L	vari
chr2L	Vha68-2
chr2L	Ziz
chr2R	14-3-3zeta
chr2R	Adf1
chr2R	ana3
chr2R	arr
chr2R	ATPsynF
chr2R	bbc
chr2R	betaTub56D
chr2R	blw
chr2R	boca
chr2R	CalpA
chr2R	Camta
chr2R	ced-6
chr2R	CG11362
chr2R	CG13322
chr2R	CG13516
chr2R	CG13531
chr2R	CG14478
chr2R	CG15097
chr2R	CG15673
chr2R	CG15863
chr2R	CG18067
chr2R	CG18467
chr2R	CG18812
chr2R	CG1902
chr2R	CG2811
chr2R	CG30015
chr2R	CG30089
chr2R	CG30152
chr2R	CG30159

chr2R	CG30197
chr2R	CG30338
chr2R	CG30345
chr2R	CG30394
chr2R	CG30493
chr2R	CG30496
chr2R	CG33798
chr2R	CG3511
chr2R	CG3548
chr2R	CG3776
chr2R	CG3788
chr2R	CG42321
chr2R	CG42516
chr2R	CG42524
chr2R	CG42663
chr2R	CG42837
chr2R	CG44243
chr2R	CG5009
chr2R	CG8399
chr2R	CG8738
chr2R	CG8839
chr2R	CG9812
chr2R	CG9896
chr2R	chn
chr2R	conv
chr2R	coro
chr2R	cpa
chr2R	CR43650
chr2R	CR43651
chr2R	CR44748
chr2R	CR45143
chr2R	CR45267
chr2R	CR45270
chr2R	CR45321
chr2R	CR46194
chr2R	CR46257
chr2R	Cyp6a20
chr2R	Cyt-b5
chr2R	Dll
chr2R	dom
chr2R	Drat
chr2R	E(Pc)
chr2R	EbpIII
chr2R	EcR
chr2R	Ef1alpha48D
chr2R	emp
chr2R	exu
chr2R	Fcp1
chr2R	fra
chr2R	fus
chr2R	gcl
chr2R	GEFmeso
chr2R	Got1
chr2R	Gp150
chr2R	Gpo-1
chr2R	grh

chr2R	GstE12
chr2R	HmgZ
chr2R	hts
chr2R	ken
chr2R	l(2)k01209
chr2R	levy
chr2R	lig
chr2R	Lpin
chr2R	LRR
chr2R	Magi
chr2R	Mapmodulin
chr2R	Mef2
chr2R	MESR4
chr2R	mi
chr2R	mir-14
chr2R	mir-281-1
chr2R	mir-281-2
chr2R	mir-4978
chr2R	mlt
chr2R	Myd88
chr2R	nahoda
chr2R	NAT1
chr2R	Not1
chr2R	Oda
chr2R	pAbp
chr2R	Pabp2
chr2R	pain
chr2R	Pal1
chr2R	Pbp49
chr2R	Pdk
chr2R	Pfk
chr2R	phtf
chr2R	pio
chr2R	pirk
chr2R	pk
chr2R	Pka-R2
chr2R	Ppa
chr2R	Pu
chr2R	px
chr2R	qkr54B
chr2R	Rab32
chr2R	RabX1
chr2R	Reg-5
chr2R	RpL18A
chr2R	RpL19
chr2R	RpL23
chr2R	RpLP2
chr2R	SCAP
chr2R	Sdc
chr2R	SelD
chr2R	Sesn
chr2R	shot
chr2R	Sik3
chr2R	Sin3A
chr2R	Slik
chr2R	slim

chr2R	slv
chr2R	snoRNA:Me28S-A1322
chr2R	so
chr2R	Sox14
chr2R	Sox15
chr2R	Spn42Da
chr2R	sut1
chr2R	Taz
chr2R	TER94
chr2R	Tsp42Ea
chr2R	Tsp42El
chr2R	tun
chr2R	Ubc10
chr2R	Vha16-1
chr2R	Vps13
chr2R	wdp
chr2R	whd
chr2R	wmd
chr2R	Xbp1
chr2R	zip
chr3L	ago
chr3L	Arl1
chr3L	asf1
chr3L	Atg18a
chr3L	bbg
chr3L	bmm
chr3L	CG10585
chr3L	CG10660
chr3L	CG11399
chr3L	CG11486
chr3L	CG11658
chr3L	CG12004
chr3L	CG13484
chr3L	CG13698
chr3L	CG13893
chr3L	CG13907
chr3L	CG13917
chr3L	CG14997
chr3L	CG17180
chr3L	CG17839
chr3L	CG18136
chr3L	CG2162
chr3L	CG2211
chr3L	CG32039
chr3L	CG32103
chr3L	CG32137
chr3L	CG32138
chr3L	CG32264
chr3L	CG32276
chr3L	CG32369
chr3L	CG32486
chr3L	CG33169
chr3L	CG33229
chr3L	CG34056
chr3L	CG3408
chr3L	CG34250

chr3L	CG3764
chr3L	CG3797
chr3L	CG3902
chr3L	CG3961
chr3L	CG4042
chr3L	CG42637
chr3L	CG43245
chr3L	CG4825
chr3L	CG5087
chr3L	CG5577
chr3L	CG5618
chr3L	CG5989
chr3L	CG6767
chr3L	CG6812
chr3L	CG7879
chr3L	CG8596
chr3L	CG9119
chr3L	chb
chr3L	chrb
chr3L	CR42719
chr3L	CR42862
chr3L	CR43334
chr3L	CR43875
chr3L	CR45121
chr3L	CR45168
chr3L	CR45396
chr3L	CR45681
chr3L	CR45916
chr3L	CycT
chr3L	Dab
chr3L	dar1
chr3L	Dhc64C
chr3L	Diap1
chr3L	dikar
chr3L	DnaJ-1
chr3L	DOR
chr3L	dos
chr3L	E(bx)
chr3L	ERR
chr3L	eyg
chr3L	fal
chr3L	fax
chr3L	Fit1
chr3L	form3
chr3L	FoxK
chr3L	fwd
chr3L	gry
chr3L	Gug
chr3L	Gyc76C
chr3L	h
chr3L	Hsp83
chr3L	ImpL2
chr3L	klar
chr3L	klu
chr3L	Krn
chr3L	kst

chr3L	l(3)02640
chr3L	l(3)76BDm
chr3L	Lmpt
chr3L	MED19
chr3L	melt
chr3L	MEP-1
chr3L	mib1
chr3L	mirr
chr3L	Mocs1
chr3L	Mpcp
chr3L	mRpL2
chr3L	mRpS26
chr3L	Msr-110
chr3L	mthl10
chr3L	MYPT-75D
chr3L	Ndfip
chr3L	Nedd4
chr3L	nmo
chr3L	Notum
chr3L	nuf
chr3L	ome
chr3L	P5CDh1
chr3L	Pdp1
chr3L	Pep
chr3L	Pex1
chr3L	PGRP-LA
chr3L	PGRP-LF
chr3L	Pitslre
chr3L	Ptip
chr3L	QC
chr3L	Rab8
chr3L	Rap1
chr3L	RasGAP1
chr3L	Reg-2
chr3L	rgn
chr3L	RhoGEF3
chr3L	roq
chr3L	scny
chr3L	scyl
chr3L	Sec63
chr3L	SH3PX1
chr3L	simj
chr3L	siz
chr3L	smg
chr3L	Sod
chr3L	Spn
chr3L	Spn77Ba
chr3L	SREBP
chr3L	sti
chr3L	sty
chr3L	Taf4
chr3L	Tgi
chr3L	tna
chr3L	tral
chr3L	trc
chr3L	Ubc4

chr3L	UGP
chr3L	upSET
chr3L	Usp32
chr3L	Vap-33B
chr3L	velo
chr3L	vsg
chr3R	14-3-3epsilon
chr3R	Afti
chr3R	Akt1
chr3R	alpha-Est8
chr3R	alpha-Man-IIb
chr3R	alphaTub84B
chr3R	Atg17
chr3R	Atg4b
chr3R	ATP8B
chr3R	Atpalpha
chr3R	ATPsynC
chr3R	ATPsynE
chr3R	ATPsyngamma
chr3R	att-ORFA
chr3R	Axn
chr3R	B52
chr3R	ball
chr3R	Best1
chr3R	bocks
chr3R	Bruce
chr3R	by
chr3R	Cad99C
chr3R	CG10055
chr3R	CG10126
chr3R	CG10311
chr3R	CG10365
chr3R	CG10903
chr3R	CG11880
chr3R	CG11882
chr3R	CG11899
chr3R	CG12054
chr3R	CG12213
chr3R	CG12413
chr3R	CG12581
chr3R	CG12818
chr3R	CG12948
chr3R	CG14259
chr3R	CG14661
chr3R	CG17271
chr3R	CG17360
chr3R	CG17734
chr3R	CG1910
chr3R	CG2310
chr3R	CG2519
chr3R	CG2747
chr3R	CG2846
chr3R	CG2911
chr3R	CG31038
chr3R	CG31075
chr3R	CG31098

chr3R	CG31103
chr3R	CG31145
chr3R	CG31211
chr3R	CG31224
chr3R	CG31324
chr3R	CG33111
chr3R	CG34133
chr3R	CG42668
chr3R	CG42828
chr3R	CG43143
chr3R	CG43427
chr3R	CG4433
chr3R	CG45218
chr3R	CG5044
chr3R	CG5214
chr3R	CG5346
chr3R	CG5793
chr3R	CG6051
chr3R	CG6178
chr3R	CG6218
chr3R	CG6231
chr3R	CG6439
chr3R	CG6959
chr3R	CG6966
chr3R	CG7071
chr3R	CG7518
chr3R	CG7943
chr3R	CG8121
chr3R	CG8176
chr3R	CG8866
chr3R	CG9821
chr3R	cic
chr3R	cnc
chr3R	corto
chr3R	COX5A
chr3R	CR31044
chr3R	CR43130
chr3R	CR46036
chr3R	cry
chr3R	CycG
chr3R	Cyp9f2
chr3R	Cys
chr3R	Dhap-at
chr3R	dmt
chr3R	Dmtn
chr3R	ea
chr3R	eff
chr3R	eIF4G2
chr3R	flfl
chr3R	GC1
chr3R	GILT1
chr3R	glo
chr3R	Glut4EF
chr3R	GlyS
chr3R	GstD1
chr3R	Hel89B

chr3R	His2Av
chr3R	Hr96
chr3R	Hrb98DE
chr3R	Hs6st
chr3R	Hsromega
chr3R	Inx3
chr3R	Itp-r83A
chr3R	jagn
chr3R	jar
chr3R	JHDM2
chr3R	jumu
chr3R	jvl
chr3R	kay
chr3R	Kdm2
chr3R	kra
chr3R	KrT95D
chr3R	l(3)neo38
chr3R	lap
chr3R	Lk6
chr3R	lost
chr3R	LpR2
chr3R	lute
chr3R	Map205
chr3R	Mdh2
chr3R	Med
chr3R	MED25
chr3R	Mgat2
chr3R	mir-4949
chr3R	Mitofilin
chr3R	mld
chr3R	mod(mdg4)
chr3R	mor
chr3R	mRpL35
chr3R	mRpL47
chr3R	Muted
chr3R	nero
chr3R	Nmnat
chr3R	nsl1
chr3R	p38a
chr3R	Pak3
chr3R	Pcd
chr3R	Pglym78
chr3R	PKD
chr3R	Pli
chr3R	Pp1-87B
chr3R	Ppcs
chr3R	pyd
chr3R	Rab1
chr3R	Rab7
chr3R	Rassf
chr3R	REPTOR
chr3R	RpII18
chr3R	RpL13A
chr3R	RpL32
chr3R	RpS27
chr3R	RpS28a

chr3R	scrib
chr3R	SelR
chr3R	Set8
chr3R	slmb
chr3R	snRNA:7SK
chr3R	Spec2
chr3R	spg
chr3R	spz
chr3R	sqd
chr3R	sr
chr3R	ssh
chr3R	su(Hw)
chr3R	subdued
chr3R	Sym
chr3R	T48
chr3R	Taf1
chr3R	tara
chr3R	Tctp
chr3R	TfIIA-S
chr3R	Tl
chr3R	Tm1
chr3R	tmod
chr3R	Tpc1
chr3R	trbd
chr3R	Trc8
chr3R	Tsp86D
chr3R	Ubc6
chr3R	Unc-115a
chr3R	unk
chr3R	Vha13
chr3R	Vti1b
chr3R	wkd
chr3R	XNP
chr3R	Xrp1
chr3R	yata
chr3R	Zip89B
chr3R	Zip99C
chrX	Actn
chrX	Atg8a
chrX	bi
chrX	brk
chrX	BTBD9
chrX	c11.1
chrX	Cdc42
chrX	CG11151
chrX	CG11448
chrX	CG12065
chrX	CG12075
chrX	CG12236
chrX	CG12991
chrX	CG1354
chrX	CG14200
chrX	CG14777
chrX	CG14795
chrX	CG1486
chrX	CG15046

chrX	CG15211
chrX	CG15717
chrX	CG15771
chrX	CG15784
chrX	CG1677
chrX	CG1737
chrX	CG17896
chrX	CG18809
chrX	CG2059
chrX	CG3091
chrX	CG32521
chrX	CG32638
chrX	CG32687
chrX	CG32758
chrX	CG33178
chrX	CG33932
chrX	CG34348
chrX	CG3835
chrX	CG3847
chrX	CG4096
chrX	CG42353
chrX	CG42354
chrX	CG4293
chrX	CG4313
chrX	CG4768
chrX	CG5010
chrX	CG5273
chrX	CG6023
chrX	CG6299
chrX	CG6398
chrX	CG6847
chrX	CG7033
chrX	CG9691
chrX	chas
chrX	CkIIbeta
chrX	Clic
chrX	CoRest
chrX	COX6B
chrX	CR33963
chrX	CR43963
chrX	CR44340
chrX	CR44619
chrX	CR44691
chrX	CR44966
chrX	CrebB
chrX	csw
chrX	ctp
chrX	cv
chrX	dhd
chrX	Dok
chrX	dome
chrX	Evi5
chrX	Flo2
chrX	fs(1)h
chrX	fz4
chrX	Gclc

chrX	Gs2
chrX	GstT3
chrX	GstT4
chrX	hang
chrX	Hayan
chrX	Hers
chrX	Hr4
chrX	Hsp60
chrX	inc
chrX	IntS6
chrX	Inx2
chrX	Jafrac1
chrX	Kap3
chrX	kek5
chrX	l(1)G0156
chrX	lawc
chrX	Lsd-2
chrX	mamo
chrX	MFS10
chrX	Mgstl
chrX	NAAT1
chrX	nej
chrX	NFAT
chrX	Nup205
chrX	per
chrX	Pfrx
chrX	ph-d
chrX	PhKgamma
chrX	ph-p
chrX	pico
chrX	Pp2B-14D
chrX	Rala
chrX	Ran
chrX	RhoGAP18B
chrX	RhoU
chrX	Rok
chrX	RpL17
chrX	RpL22
chrX	RpL37a
chrX	Rpp20
chrX	RpS14a
chrX	RpS19a
chrX	RpS28b
chrX	RpS5a
chrX	rut
chrX	snmRNA:204
chrX	spoon
chrX	spri
chrX	Tis11
chrX	Tlk
chrX	Top1
chrX	Trf4-1
chrX	Ubi-p5E
chrX	usp
chrX	Usp2
chrX	Usp7

chrX	VhaAC39-1
chrX	Vinc
chrX	w
chrX	Ykt6

Supplementary Table 7 Genes related to the enriched GO terms

The genes categolized in each GO term were shown. The genes showing significant increase after 5-min optogenetic activation followed by 10-min rest were indicated in red.

Enriched GO term (molecular functions)	Genes
protein domain specific binding	Su(dx);aop;Sirt1;Myd88;14-3-3zeta;fra;dos;Hsp83;Arl1;Dab;Nedd4;Ndfip;Ubc6;pyd;14-3-3epsilon;mod(mdg4);Tl;Rala;ctp;pico
DNA-binding transcription factor activity	cbt;aop;lilli;bowl;dpy;Hnf4;GATAd;bun;CG9932;Sirt1;crp;Dif;tup;Mondo;EcR;phtf;so;Camta;Mef2;Sox15;chn;MESR4;grh;Xbp1;ken;Sox14;dar1;Pdp1;ERR;h;klu;FoxK;eyg;mirr;Taf4;SREBP;jumu;l(3)neo38;sr;CG31224;cic;cnc;REPTOR;Hr96;kay;Med;Hr4;usp;bi;CG12236;brk;NFAT;CrebB;CoRest
enzyme binding	aru;capt;Msp300;CG9175;Ziz;emb;CG6746;Sirt1;CG10283;drl;CG10188;dia;CG30496;Ubc10;GEFmeso;Magi;RhoGEF3;ago;Ubc4;tral;nuf;Diap1;CycT;MYPT-75D;siz;Spec2;Atg17;pyd;Tctp;wkd;Lk6;CG17360;eff;nsl1;Pak3;Mitofilin;ball;spg;CG34133;Axn;CycG;CG11448;ph-p;Ubi-p5E;nej;RhoU;spri;Atg8a;Evi5;Rok;RhoGAP18B;dome;Cdc42;pico
transcription factor binding	ebi;lilli;Sirt1;tup;Mondo;EcR;so;14-3-3zeta;HmgZ;E(bx);DOR;DnaJ-1;Gug;h;Tgi;Taf4;MED19;asf1;Taf1;14-3-3epsilon;cic;TfIIA-S;Hr96;kay;per;nej;Usp7;NFAT;CoRest
sequence-specific DNA binding	al;cbt;aop;bowl;Hnf4;GATAd;CG9932;Smg5;crp;Dif;tup;EcR;so;Pbp49;Camta;Mef2;chn;MESR4;grh;Xbp1;ken;Sox14;Dll;dar1;Pdp1;ERR;h;klu;FoxK;eyg;mirr;Taf1;JHDM2;jumu;sqd;su(Hw);CG31224;cic;Hr96;Hrb98DE;kay;Med;Hr4;usp;per;bi;CG3847;brk;NFAT;mamo;CrebB;Hers
protein dimerization activity	smo;dpp;bun;crp;drl;CG9331;Mondo;EcR;phtf;Camta;Mef2;14-3-3zeta;Sin3A;grh;exu;cpa;Hsp83;h;mirr;Taf4;SREBP;corto;Pak3;14-3-3epsilon;subdued;slmb;mod(mdg4);cnc;REPTOR;His2Av;spz;kay;inc;usp;per;ctp;dome
cytoskeletal protein binding	aru;capt;Msp300;Myo31DF;Rab6;ck;crp;Arpc2;dia;shot;Mapmodulin;CG15097;hts;cpa;RpL23;zip;klar;Spn;kst;CG32264;form3;smg;nuf;Nedd4;chb;CG8176;by;Unc-115a;Tm1;jar;ssh;Cad99C;tmod;Map205;inc;Actn;Vinc;Kap3
lipid binding	smo;l(2)gd1;dia;SCAP;EcR;CG30493;Myd88;CG5009;kst;melt;ERR;Atg18a;SH3PX1;Itp-r83A;lap;CG8176;Akt1;CG42668;ATPsynC;usp;CG32758;Clic;CG6299
protein-containing complex binding	dpp;Slh;Msp300;Smg5;ck;Arpc2;hts;cpa;mi;CG3776;Spn;CG5989;Ubc4;Taf4;chb;kra;CG8176;Unc-115a;Afti;Tm1;Rab7;jar;LpR2;His2Av;Actn;Vinc;CG1354;Usp2
kinase activity	Akap200;Pka-C1;p38b;drl;Pdk;Pfk;SelD;l(2)k01209;Sik3;Magi;Slik;fwd;CG11486;nmo;sti;CG42637;trc;Pitslre;Taf1;CG2846;pyd;CG43143;Lk6;CG6218;Akt1;Pak3;PKD;CG31145;p38a;ball;yata;kay;CycG;Tlk;Dok;PhKgamma;CkIIbeta;Rok;Pfrx

Enriched GO term (biological process)	Genes
wing disc development	spen;smo;ebi;cbt;capt;frtz;Su(dx);Rab5;dpp;Slh;lilli;bowl;dpy;mts;Pka-C1;l(2)gd1;crol;p38b;ck;Gli;Socs36E;tup;spi;EcR;pk;Lpin;Pal1;Sin3A;arr;shot;Sox15;GEFmeso;cpa;dom;px;wmd;pio;Dll;zip;sty;nmo;Gug;RasGAP1;eyg;mirr;nuf;Arl1;Notum;Nedd4;Ndfip;MYPT-75D;trc;CG11399;corto;pyd;by;jumu;Cyp9f2;mor;tara;14-3-3epsilon;Ppcs;Hs6st;cic;unk;Rab7;scrib;kay;RpS28a;Axn;Med;bi;ctp;brk;Dok;Gclc;Pp2B-14D;Rok;dome;Cdc42
post-embryonic animal organ development	spen;smo;al;ebi;cbt;frtz;Su(dx);Rab5;dpp;bowl;dpy;Msp300;Pka-C1;CG4036;Myo31DF;l(2)gd1;crol;p38b;ck;Gli;crp;Dif;Socs36E;spi;EcR;pk;so;Lpin;Pal1;Sin3A;arr;shot;chn;GEFmeso;px;wmd;pio;Dll;zip;klar;sty;nmo;Gug;RasGAP1;mirr;Diap1;Nedd4;MYPT-75D;CG42637;trc;corto;Kdm2;by;jumu;nsl1;mor;Ppcs;Hs6st;cic;unk;jar;scrib;Tl;spz;kay;Axn;Med;bi;ctp;brk;Dok;Gclc;Rok;dome;Cdc42
regionalization	spen;smo;cbt;capt;Su(dx);Rab5;dpp;Slh;lilli;bowl;mts;Pka-C1;Rab6;ck;Gli;tup;brat;EcR;pk;lig;Lpin;gcl;Myd88;TER94;fra;arr;pAbp;GEFmeso;CalpA;exu;dom;Dll;zip;klar;dos;Hsp83;sty;Dhc64C;nmo;Gug;h;RasGAP1;klu;Ptip;nuf;Notum;CG11399;lost;corto;Kdm2;wkd;glo;sqd;jvl;Tm1;ea;mor;tara;14-3-3epsilon;cic;slmb;cnc;Rab7;scrib;Tl;spz;Axn;Med;csw;bi;spoon;brk;fs(1)h;CkIIbeta;dome
metamorphosis	spen;smo;al;ebi;cbt;frtz;Su(dx);Rab5;dpp;bowl;dpy;Pka-C1;Myo31DF;l(2)gd1;crol;p38b;ck;Gli;Dif;Socs36E;spi;EcR;pk;so;Lpin;Pal1;TER94;arr;shot;GEFmeso;px;wmd;Sox14;EbpIII;pio;Dll;zip;sty;nmo;Atg18a;Gug;RasGAP1;mirr;Diap1;Nedd4;MYPT-75D;trc;corto;Itp-r83A;Atg17;by;jumu;mor;Mdh2;Ppcs;Hs6st;cic;unk;jar;scrib;kay;Axn;Med;usp;bi;ctp;brk;Dok;Gclc;Atg8a;Rok;dome;Cdc42
appendage development	spen;smo;al;ebi;frtz;Su(dx);Rab5;dpp;bowl;dpy;Pka-C1;l(2)gd1;crol;p38b;ck;Gli;Socs36E;drl;spi;EcR;pk;Lpin;Pal1;arr;shot;GEFmeso;px;wmd;pio;Dll;zip;sty;nmo;Gug;RasGAP1;eyg;mirr;Diap1;Nedd4;MYPT-75D;trc;corto;by;jumu;mor;Ppcs;Hs6st;cic;unk;scrib;Axn;Med;bi;ctp;brk;Dok;Gclc;Rok;Cdc42
sensory organ development	spen;smo;al;ebi;cbt;capt;aop;dpp;lilli;mts;Sirup;Pka-C1;l(2)gd1;Rab6;bun;ck;Socs36E;tup;spi;pk;so;fra;NAT1;arr;shot;Sox15;chn;grh;pAbp;Gp150;zip;klar;Rap1;dos;sty;melt;nmo;Gug;h;RasGAP1;klu;eyg;mirr;nuf;mib1;Diap1;Dab;Krn;trc;RpL13A;jagn;pyd;jumu;SelR;Afti;eff;Ppcs;Hs6st;cic;unk;ssh;scrib;Hrb98DE;spg;kay;csw;Rala;bi;ctp;nej;Rok;dome;Cdc42
epithelial tube morphogenesis	spen;smo;al;ebi;cbt;frtz;Su(dx);Rab5;dpp;bowl;dpy;Pka-C1;Myo31DF;l(2)gd1;crol;p38b;ck;Gli;Socs36E;spi;EcR;pk;so;Lpin;Pal1;arr;shot;GEFmeso;px;wmd;Slik;pio;Dll;zip;sty;ago;form3;nmo;Gug;RasGAP1;mirr;Diap1;Nedd4;MYPT-75D;CG42637;trc;corto;pyd;by;dmt;jumu;mor;Ppcs;Vha13;Hs6st;cic;unk;jar;scrib;kay;Axn;Med;VhaAC39-1;bi;ctp;brk;Dok;Gclc;Rok;dome;Cdc42
post-embryonic animal morphogenesis	spen;smo;al;ebi;cbt;frtz;Su(dx);Rab5;dpp;bowl;dpy;Pka-C1;Myo31DF;l(2)gd1;crol;p38b;ck;Gli;Dif;Socs36E;spi;EcR;pk;so;Lpin;Pal1;TER94;arr;shot;GEFmeso;px;wmd;pio;Dll;zip;sty;nmo;Atg18a;Gug;RasGAP1;mirr;Diap1;Nedd4;MYPT-75D;trc;corto;Itp-r83A;Atg17;by;jumu;mor;Mdh2;Ppcs;Hs6st;cic;unk;jar;scrib;kay;Axn;Med;bi;ctp;brk;Dok;Gclc;Atg8a;Rok;dome;Cdc42
negative regulation of response to stimulus	ebi;Su(dx);aop;mts;Akap200;Pka-C1;l(2)gd1;crol;bun;Socs36E;drl;pk;LRR;lig;Lpin;gcl;TER94;Sin3A;MESR4;Sik3;cpa;pirk;wdp;Sesn;ken;E(bx);sty;ImpL2;ago;DnaJ-1;scny;nmo;Atg18a;Gug;PGRP-LA;PGRP-LF;RasGAP1;scyl;chrb;Notum;Nedd4;asf1;Spn77Ba;chb;Ubc6;Lk6;eff;CG5044;Akt1;slmb;Rassf;Pli;p38a;ssh;Axn;Hr4;Rala;CG4096;brk;nej;NFAT;Rok;kek5;Usp2
regulation of intracellular signal transduction	smo;ebi;aru;dpp;Ziz;mts;Akap200;Socs36E;drl;CG10188;spi;LRR;lig;Lpin;Myd88;14-3-3zeta;MESR4;slim;GEFmeso;Sik3;cpa;wdp;Sesn;ken;E(bx);RhoGEF3;Rap1;dos;sty;ImpL2;melt;Atg18a;PGRP-LF;scyl;chrb;siz;chb;Ubc6;Spec2;pyd;Lk6;Pp1-87B;Akt1;Pak3;14-3-3epsilon;slmb;Rassf;ssh;XNP;scrib;spg;CycG;Rala;spoon;spri;NFAT;rut;Cdc42

Supplementary Table 8 Genes showing increase by optogenetic activation.
The genes showed statistically significant increase after 5 min light illumination followed by 10 min rest were determined by DESeq2.

Gene Symbol	q.value	log2	CoRest-C bound (338/1582)	CBP bound (1055/1582)	Rpd3 bound (606/1582)	all colocalized (235/1582)
DnaJ-1	0	3.4216922	+	+	+	+
Hsp83	0	2.6751262	+	+	+	+
Hsromega	2.44E-140	1.9288177	+	+	+	+
bmm	3.302E-88	1.8142158	+	+	+	+
Pdk	4.202E-95	1.7280181	+	+	+	+
Lsd-2	9.295E-52	1.3953836	+	+	+	+
CG14259	1.982E-52	1.3404234	+	+	+	+
CG7033	5.116E-36	1.2501684	+	+	+	+
ken	7.744E-10	1.1476486	+	+	+	+
h	2.18E-22	1.115911	+	+	+	+
whd	1.532E-25	1.0969557	+	+	+	+
CG6785	1.689E-08	1.093644	+	+	+	+
ced-6	1.262E-27	1.0754023	+	+	+	+
CG10348	4.727E-15	1.0753352	+	+	+	+
att-ORFA	3.543E-09	1.0159564	+	+	+	+
CG13893	3.223E-18	0.993227	+	+	+	+
CG15673	5.487E-23	0.9441159	+	+	+	+
ck	5.712E-10	0.9175971	+	+	+	+
cbt	1.519E-16	0.8846943	+	+	+	+
klu	8.112E-06	0.8797964	+	+	+	+
dmt	2.104E-06	0.8791368	+	+	+	+
Tis11	1.835E-16	0.8779546	+	+	+	+
CG10600	8.807E-12	0.8645571	+	+	+	+
chas	1.174E-09	0.8202687	+	+	+	+
Hers	9.244E-06	0.8139233	+	+	+	+
CR43314	5.287E-10	0.8107388	+	+	+	+
MRP	6.597E-11	0.7933821	+	+	+	+
CG9812	7.846E-18	0.7800547	+	+	+	+
ImpL2	2.256E-17	0.7770316	+	+	+	+
CG5953	1.022E-09	0.7554061	+	+	+	+
Notum	0.0072918	0.7553727	+	+	+	+
Jafrac1	9.437E-16	0.7545314	+	+	+	+
CG6767	3.801E-10	0.737051	+	+	+	+
emp	7.295E-12	0.7361563	+	+	+	+
pk	2.217E-07	0.7289841	+	+	+	+
Pino	9.015E-15	0.7228043	+	+	+	+
cdc14	5.957E-10	0.7226957	+	+	+	+
grh	7.262E-12	0.7128377	+	+	+	+
CG5973	5.417E-18	0.6958718	+	+	+	+
Dll	4.677E-08	0.6939546	+	+	+	+
CG9932	5.662E-11	0.6800857	+	+	+	+
drongo	2.219E-06	0.6744075	+	+	+	+
CG32103	2.548E-13	0.6731429	+	+	+	+
CG33178	7.109E-09	0.6681518	+	+	+	+
nahoda	7.739E-06	0.6679851	+	+	+	+
CG6231	1.662E-07	0.667901	+	+	+	+
CG43427	1.57E-06	0.6634732	+	+	+	+
siz	5.773E-06	0.6631415	+	+	+	+
bowl	0.0005089	0.6603608	+	+	+	+
RhoU	0.0053187	0.6603003	+	+	+	+
Cad99C	9.818E-06	0.6532968	+	+	+	+

CG17896	1.477E-12	0.651285	+	+	+	+
Cyt-b5	4.734E-14	0.6487456	+	+	+	+
GstD1	8.775E-16	0.6476504	+	+	+	+
mirr	0.0002295	0.6414534	+	+	+	+
CG6959	2.661E-07	0.6397509	+	+	+	+
wdp	5.122E-08	0.6370918	+	+	+	+
Hnf4	2.343E-08	0.6365088	+	+	+	+
bbg	0.0003063	0.6362856	+	+	+	+
fus	4.955E-06	0.633616	+	+	+	+
CR42862	3.058E-10	0.6314933	+	+	+	+
MYPT-75D	5.884E-08	0.6307827	+	+	+	+
GstT3	8.01E-06	0.6307402	+	+	+	+
CG8596	5.09E-07	0.626466	+	+	+	+
Tgi	5.584E-08	0.6251766	+	+	+	+
mthl10	0.0005955	0.6210502	+	+	+	+
CG5346	6.686E-08	0.61158	+	+	+	+
Ziz	5.451E-06	0.6088291	+	+	+	+
RasGAP1	0.0005179	0.6020787	+	+	+	+
CG32276	6.971E-08	0.6001167	+	+	+	+
CG12075	0.0004445	0.5992837	+	+	+	+
dpp	5.099E-06	0.5935855	+	+	+	+
LpR2	5.777E-05	0.5916319	+	+	+	+
Lmpt	3.996E-11	0.5864842	+	+	+	+
PGRP-LF	0.0005137	0.5861127	+	+	+	+
sr	0.0092355	0.5854005	+	+	+	+
GstT4	5.303E-10	0.5793712	+	+	+	+
Sox14	0.0003545	0.5783105	+	+	+	+
fra	0.0006242	0.5754807	+	+	+	+
bi	0.0023647	0.575342	+	+	+	+
Sesn	7.287E-09	0.5718267	+	+	+	+
shot	1.143E-10	0.5683081	+	+	+	+
pirk	0.0232515	0.5668801	+	+	+	+
kay	9.796E-05	0.5653496	+	+	+	+
CG14200	0.0001536	0.5638811	+	+	+	+
CG17734	9.348E-08	0.5630141	+	+	+	+
pio	8.052E-09	0.5629224	+	+	+	+
Gli	0.0024634	0.562179	+	+	+	+
Itp-r83A	0.0002069	0.559498	+	+	+	+
Sik3	0.0023034	0.5547132	+	+	+	+
CG12581	4.579E-05	0.5493135	+	+	+	+
CG17839	2.035E-06	0.5482652	+	+	+	+
CG33111	0.0001189	0.546928	+	+	+	+
Mocs1	2.79E-05	0.5455171	+	+	+	+
aop	5.71E-05	0.5452879	+	+	+	+
papi	3.64E-05	0.5448706	+	+	+	+
CG7337	1.409E-06	0.5439602	+	+	+	+
nrv1	5.214E-05	0.5432995	+	+	+	+
CG12065	0.000631	0.5406569	+	+	+	+
Diap1	3.734E-07	0.5402352	+	+	+	+
GILT1	3.55E-07	0.5365792	+	+	+	+
so	0.0006961	0.5364171	+	+	+	+
jvl	1.748E-06	0.528381	+	+	+	+
CG8399	5.308E-06	0.5275453	+	+	+	+
CG3164	1.82E-06	0.5216386	+	+	+	+
Msp300	3.081E-06	0.5162733	+	+	+	+
CG9119	0.0018638	0.5137569	+	+	+	+
w	0.0002118	0.5088074	+	+	+	+
CG4313	0.0032588	0.5074011	+	+	+	+

CG5009	5.387E-07	0.5057667	+	+	+	+
CG31955	0.0058591	0.5044311	+	+	+	+
Gp150	5.009E-08	0.5006437	+	+	+	+
Pde11	4.113E-06	0.4992967	+	+	+	+
Magi	0.0020443	0.4989076	+	+	+	+
EcR	0.0003491	0.4984095	+	+	+	+
pico	0.0004338	0.4956375	+	+	+	+
CalpA	0.0003149	0.4955735	+	+	+	+
spg	0.0098288	0.4945892	+	+	+	+
Fit1	0.0008709	0.4935844	+	+	+	+
CG6770	0.0004034	0.4900305	+	+	+	+
lilli	0.0007066	0.4895356	+	+	+	+
NFAT	5.841E-05	0.487447	+	+	+	+
Zip89B	0.0056126	0.4849378	+	+	+	+
form3	2.231E-05	0.4832382	+	+	+	+
vari	0.0001577	0.4794743	+	+	+	+
CycT	7.955E-06	0.478123	+	+	+	+
T48	0.0017636	0.4779023	+	+	+	+
Dif	3.648E-05	0.4753232	+	+	+	+
Oda	1.266E-09	0.4738718	+	+	+	+
alpha-Est8	6.68E-05	0.4726668	+	+	+	+
cnc	2.784E-06	0.4725418	+	+	+	+
CG18812	6.869E-05	0.4717637	+	+	+	+
Trf4-1	3.634E-06	0.4697376	+	+	+	+
CG32369	0.0138384	0.4676699	+	+	+	+
spz	0.003605	0.4664343	+	+	+	+
zip	4.806E-05	0.4634142	+	+	+	+
CG5853	4.603E-06	0.4622885	+	+	+	+
CG2519	1.583E-05	0.460666	+	+	+	+
by	8.803E-05	0.4596426	+	+	+	+
pain	1.258E-05	0.4596156	+	+	+	+
fz4	0.0478105	0.4594165	+	+	+	+
Nedd4	0.0017262	0.4593378	+	+	+	+
Ppa	0.0068902	0.4584259	+	+	+	+
scyl	3.154E-06	0.4530075	+	+	+	+
CG6700	0.0005154	0.4517847	+	+	+	+
CG5618	3.218E-05	0.4463937	+	+	+	+
Gug	0.000681	0.4445698	+	+	+	+
scrib	5.074E-06	0.4444619	+	+	+	+
CG9171	0.0044949	0.4432674	+	+	+	+
fwd	0.0014688	0.4399271	+	+	+	+
CG7879	0.004631	0.4310095	+	+	+	+
CG6398	0.0014074	0.4295643	+	+	+	+
CG6178	0.0232285	0.427362	+	+	+	+
CG11151	0.0007449	0.426495	+	+	+	+
cry	0.000375	0.4241443	+	+	+	+
CG3091	0.0006492	0.4233705	+	+	+	+
spri	0.0080477	0.4211938	+	+	+	+
mld	0.0016039	0.4200796	+	+	+	+
CG14661	4.139E-07	0.4199988	+	+	+	+
Reg-5	2.892E-05	0.4163615	+	+	+	+
kst	8.569E-05	0.4159107	+	+	+	+
CG32137	0.0016876	0.4114021	+	+	+	+
CG5010	2.25E-05	0.4102484	+	+	+	+
cic	0.0122121	0.4068925	+	+	+	+
Drat	1.704E-06	0.4007799	+	+	+	+
cv	0.0007887	0.4007598	+	+	+	+
pyd	0.0006544	0.3991508	+	+	+	+

Myo31DF	0.010445	0.3975111	+	+	+	+
eIF4G2	8.361E-05	0.3972229	+	+	+	+
Pu	1.043E-05	0.3891995	+	+	+	+
CG34348	0.0090145	0.3883874	+	+	+	+
CG12991	0.0015823	0.3872764	+	+	+	+
CG32138	0.0012816	0.3864364	+	+	+	+
Xrp1	5.961E-05	0.3863007	+	+	+	+
ATP8B	0.0018609	0.3860515	+	+	+	+
Tl	0.0060302	0.3857363	+	+	+	+
Gclc	0.0247775	0.3854937	+	+	+	+
Vap-33B	0.0071502	0.3840271	+	+	+	+
hang	0.0006183	0.3836463	+	+	+	+
CG32486	5.612E-05	0.3831538	+	+	+	+
lawc	0.0038117	0.3793063	+	+	+	+
CG6051	0.0373158	0.3779457	+	+	+	+
Vinc	0.0065335	0.3758479	+	+	+	+
Zip99C	0.0018561	0.3757047	+	+	+	+
Tep3	0.0038753	0.3724598	+	+	+	+
CG31211	0.0002411	0.3691363	+	+	+	+
inc	0.004709	0.3680213	+	+	+	+
CG8738	0.0242539	0.3640502	+	+	+	+
Ef1alpha48D	4.276E-06	0.3640362	+	+	+	+
Msr-110	0.0014011	0.3614324	+	+	+	+
Cyp9f2	9.796E-05	0.355976	+	+	+	+
PKD	0.0218887	0.3553139	+	+	+	+
Pfrx	0.0012816	0.353133	+	+	+	+
Dmtn	0.0135603	0.3527875	+	+	+	+
fok	0.001745	0.3519137	+	+	+	+
Tm1	0.0008001	0.3491019	+	+	+	+
CG8839	0.0029073	0.3484305	+	+	+	+
simj	0.0178637	0.34752	+	+	+	+
CG18507	0.0025541	0.3469172	+	+	+	+
RhoGAP18B	0.016123	0.3364359	+	+	+	+
Rok	0.0270608	0.3354933	+	+	+	+
Flo2	0.0402892	0.3353432	+	+	+	+
Ran	0.0033338	0.3349718	+	+	+	+
corto	0.0168375	0.3329261	+	+	+	+
Unc-115a	0.0281107	0.3322192	+	+	+	+
Lpin	0.0010321	0.327714	+	+	+	+
CG8814	0.0167564	0.3250776	+	+	+	+
CG9821	0.0072009	0.3231785	+	+	+	+
Glut4EF	0.0015335	0.3116447	+	+	+	+
jar	0.0040599	0.3108267	+	+	+	+
CG31145	0.0103098	0.3102253	+	+	+	+
TER94	0.0111149	0.3078768	+	+	+	+
CG31103	0.0087696	0.306157	+	+	+	+
tna	0.0032185	0.3040672	+	+	+	+
CG3961	0.0260779	0.2954547	+	+	+	+
GEFmeso	0.0392546	0.2919441	+	+	+	+
hts	0.0212407	0.2914401	+	+	+	+
CG42668	0.0156173	0.2816664	+	+	+	+
CG6966	0.0277288	0.280558	+	+	+	+
CG32521	0.0407854	0.279576	+	+	+	+
sqd	0.005177	0.278261	+	+	+	+
pAbp	0.0423732	0.2758755	+	+	+	+
Vha68-2	0.0220193	0.2757904	+	+	+	+
Inx3	0.014601	0.2750954	+	+	+	+
eff	0.0454723	0.2745361	+	+	+	+

Actn	0.0318768	0.2652867	+	+	+	+
nuf	0.0186262	0.2613968	+	+	+	+
Xbp1	0.0040064	0.2605162	+	+	+	+
csw	0.0197929	0.2546066	+	+	+	+
CG31038	0.044706	0.2491072	+	+	+	+
Mgstl	0.0382923	0.2460354	+	+	+	+
Sod	0.0265701	0.2315371	+	+	+	+
SelR	0.0437858	0.2152736	+	+	+	+
ovo	6.082E-14	1.0049452	+	+	+	-
Aldh-III	1.81E-15	0.721551	+	+	+	-
cv-2	4.523E-06	0.7156691	+	+	+	-
Snoo	5.75E-07	0.693172	+	+	+	-
Vha100-2	1.664E-10	0.6755643	+	+	+	-
eas	1.022E-09	0.6491915	+	+	+	-
chic	2.13E-13	0.612756	+	+	+	-
Dad	0.0007159	0.5972617	+	+	+	-
Doa	3.853E-08	0.5969499	+	+	+	-
Cerk	5.714E-10	0.5742689	+	+	+	-
CG45186	1.206E-06	0.5495608	+	+	+	-
shn	7.135E-06	0.5315449	+	+	+	-
hid	0.0004053	0.5185574	+	+	+	-
Cpr	5.652E-08	0.5056746	+	+	+	-
Syp	2.036E-06	0.4874295	+	+	+	-
kibra	0.0001547	0.4718546	+	+	+	-
crc	8.315E-06	0.4313451	+	+	+	-
gish	9.768E-06	0.4270215	+	+	+	-
Gbs-70E	0.0006659	0.3421022	+	+	+	-
vir-1	0.0062317	0.2773876	+	+	+	-
gem	0.0135603	0.2576038	+	+	+	-
Dok	0.0003415	0.6914229	+	+	-	+
Hsp70Bb	8.796E-10	1.7989018	+	+	-	-
CG14966	7.218E-10	1.4243405	+	+	-	-
Cpr97Eb	5.365E-54	1.2822401	+	+	-	-
CG5966	6.518E-17	1.2764771	+	+	-	-
CG13676	1.597E-16	1.0407076	+	+	-	-
ttk	1.043E-17	1.0144604	+	+	-	-
Cad74A	9.277E-07	1.0131313	+	+	-	-
ec	7.175E-15	1.0005762	+	+	-	-
CG31028	3.154E-06	0.9551563	+	+	-	-
dsx	1.966E-16	0.9413354	+	+	-	-
spz3	8.543E-06	0.8877477	+	+	-	-
CG9153	2.778E-12	0.8322067	+	+	-	-
CG1441	1.003E-17	0.8314957	+	+	-	-
GV1	3.028E-12	0.8213351	+	+	-	-
Drak	1.556E-09	0.8059434	+	+	-	-
Vdup1	3.55E-18	0.8041286	+	+	-	-
d	0.002006	0.7978247	+	+	-	-
CR32385	0.0046411	0.789117	+	+	-	-
CG6040	4.888E-12	0.7890818	+	+	-	-
CG11791	1.826E-14	0.7781757	+	+	-	-
ASPP	9.822E-06	0.7640733	+	+	-	-
CG11069	1.985E-05	0.7543739	+	+	-	-
wg	0.0085692	0.7391351	+	+	-	-
Ets98B	5.287E-11	0.7217472	+	+	-	-
AOX3	1.955E-12	0.7176692	+	+	-	-
Eip74EF	3.063E-08	0.7088742	+	+	-	-
trx	1.728E-07	0.6852514	+	+	-	-
Indy	4.797E-13	0.6594897	+	+	-	-

uif	1.12E-07	0.6563893	+	+	-	-
pan	8.465E-14	0.6561491	+	+	-	-
CG3011	7.594E-10	0.6487406	+	+	-	-
ft	0.0032021	0.6424647	+	+	-	-
CR32111	0.001799	0.6153138	+	+	-	-
CG6923	0.0005648	0.6143827	+	+	-	-
Lasp	5.685E-05	0.607702	+	+	-	-
mew	0.0008518	0.6005281	+	+	-	-
ScpX	7.822E-07	0.5961853	+	+	-	-
gce	3.426E-08	0.5834436	+	+	-	-
caps	0.0014446	0.5621391	+	+	-	-
zormin	8.386E-07	0.5574447	+	+	-	-
Mfe2	9.588E-07	0.5554243	+	+	-	-
Snp	8.316E-07	0.5527218	+	+	-	-
pdm3	4.291E-05	0.5272688	+	+	-	-
hth	1.027E-07	0.5188299	+	+	-	-
Fhos	0.0004589	0.5173864	+	+	-	-
sd	7.258E-06	0.5036785	+	+	-	-
Hil	3.273E-05	0.5018236	+	+	-	-
tx	0.0226937	0.4859953	+	+	-	-
Amph	2.649E-07	0.4855182	+	+	-	-
Pld	0.040752	0.4763237	+	+	-	-
Galphai	0.0009653	0.4692141	+	+	-	-
MESK2	2.176E-05	0.4668025	+	+	-	-
par-1	8.66E-07	0.456008	+	+	-	-
tws	0.0007867	0.4388666	+	+	-	-
CG15556	0.020556	0.4347112	+	+	-	-
Lac	0.0033115	0.4251853	+	+	-	-
CG7016	0.0141972	0.4151349	+	+	-	-
Atg13	0.009355	0.4072138	+	+	-	-
CREG	0.0373865	0.3950782	+	+	-	-
Ddc	0.0005154	0.3775674	+	+	-	-
SmD3	0.007532	0.3597041	+	+	-	-
CG31121	0.0168727	0.3588209	+	+	-	-
CG33144	0.0110567	0.3428259	+	+	-	-
Strn-Mlck	0.0080688	0.3339349	+	+	-	-
Tpr2	0.0082458	0.3337223	+	+	-	-
gpp	0.0160453	0.3183263	+	+	-	-
GstE9	0.0448536	0.3118141	+	+	-	-
hipk	0.0103194	0.3105552	+	+	-	-
CG43078	0.0066585	0.3052688	+	+	-	-
grass	0.0262768	0.2956737	+	+	-	-
Mob2	0.0043836	0.2699402	+	+	-	-
Rho1	0.0192993	0.2593045	+	+	-	-
SPoCk	0.01925	0.2447858	+	+	-	-
psq	0.0294297	0.2435207	+	+	-	-
RhoGDI	0.0420851	0.2238574	+	+	-	-
bif	0.0354498	0.2130016	+	+	-	-
CG13531	0.0051238	0.4501562	+	-	+	+
Drice	0.0252799	0.4733977	+	-	+	-
Srp14	0.0458412	0.4449812	+	-	+	-
mbf1	4.463E-31	1.2538188	+	-	-	-
CG5608	4.07E-11	1.0487676	+	-	-	-
l(2)gl	0.0093824	0.4062685	+	-	-	-
sli	0.0114538	0.3783871	+	-	-	-
Hsp70Aa	1.54E-182	5.249882	-	+	+	-
Hsp70Ab	8.73E-225	5.0693914	-	+	+	-
stv	0	3.5873949	-	+	+	-

Hsc70Cb	2.005E-93	1.881285	-	+	+	-
tra	3.782E-68	1.833602	-	+	+	-
Hop	2.744E-80	1.7868571	-	+	+	-
CR44768	3.262E-12	1.5496639	-	+	+	-
Nop17l	1.14E-35	1.5210371	-	+	+	-
LamC	3.544E-27	1.3917414	-	+	+	-
IP3K2	5.542E-41	1.3774959	-	+	+	-
GIIIspla2	1.483E-30	1.3079519	-	+	+	-
CG13639	0.0003527	1.1792888	-	+	+	-
foxo	5.662E-21	1.1488679	-	+	+	-
sgl	1.035E-22	1.1361298	-	+	+	-
CG16721	1.936E-23	1.1257728	-	+	+	-
nkd	1.022E-09	1.0741936	-	+	+	-
Hsc70-4	2.79E-24	1.0578546	-	+	+	-
B-H2	1.208E-06	1.031323	-	+	+	-
CG8258	3.198E-20	1.024122	-	+	+	-
Trxr-1	1.285E-36	1.0236963	-	+	+	-
CG8312	6.492E-18	1.0038387	-	+	+	-
CR45668	3.072E-21	0.9907806	-	+	+	-
Gfat1	2.935E-16	0.949564	-	+	+	-
sni	3.644E-08	0.9267861	-	+	+	-
alpha-Est10	1.7E-11	0.9137846	-	+	+	-
PRL-1	1.909E-10	0.8997211	-	+	+	-
CG17928	5.662E-21	0.8963167	-	+	+	-
ena	1.215E-09	0.8882745	-	+	+	-
Ubi-p63E	1.26E-22	0.8631942	-	+	+	-
CR45646	0.01242	0.850061	-	+	+	-
olf186-M	2.271E-12	0.8380825	-	+	+	-
CG9990	8.392E-13	0.8380228	-	+	+	-
Eip93F	9.759E-18	0.8302103	-	+	+	-
RluA-1	1.245E-07	0.8229066	-	+	+	-
caup	0.0003323	0.8183087	-	+	+	-
Ude	1.047E-05	0.8160931	-	+	+	-
Adh	1.851E-06	0.8121782	-	+	+	-
CG5326	2.403E-12	0.8096399	-	+	+	-
CG9705	3.859E-15	0.7892203	-	+	+	-
CG11686	7.832E-06	0.7785866	-	+	+	-
dp	1.339E-16	0.7674886	-	+	+	-
opa	0.0056448	0.7668649	-	+	+	-
Cat	3.578E-19	0.7661664	-	+	+	-
dl	2.582E-09	0.7563253	-	+	+	-
Irc	4.515E-15	0.7513175	-	+	+	-
Tdrd3	7.105E-06	0.7505402	-	+	+	-
CG7139	1.941E-10	0.74799	-	+	+	-
for	8.032E-11	0.7479647	-	+	+	-
in	0.0203775	0.744439	-	+	+	-
CG8306	8.379E-12	0.741502	-	+	+	-
l(1)G0469	1.099E-07	0.7370894	-	+	+	-
Uhg5	4.547E-12	0.7342015	-	+	+	-
CG11073	8.044E-10	0.7333379	-	+	+	-
CG32082	1.571E-13	0.7204372	-	+	+	-
Exn	1.289E-09	0.7138623	-	+	+	-
CG4928	4.074E-10	0.7112306	-	+	+	-
CG2604	9.123E-13	0.7066824	-	+	+	-
Keap1	1.468E-05	0.7032758	-	+	+	-
Ugt86Da	7.039E-06	0.6987454	-	+	+	-
InR	7.47E-12	0.6931608	-	+	+	-
fw	5.08E-08	0.6910808	-	+	+	-

CG4996	3.055E-05	0.6893097	-	+	+	-
CG14207	3.787E-14	0.6872168	-	+	+	-
Plod	6.677E-10	0.6833275	-	+	+	-
b	1.409E-06	0.6804272	-	+	+	-
jbug	6.145E-08	0.6690862	-	+	+	-
CG4455	0.0051448	0.6614674	-	+	+	-
tsh	0.001494	0.6527549	-	+	+	-
Rac2	2.507E-06	0.6524446	-	+	+	-
Hsc70-5	1.075E-11	0.6400902	-	+	+	-
wit	3.846E-07	0.6382869	-	+	+	-
Ant2	0.0356908	0.6301566	-	+	+	-
CG14223	0.0011303	0.6274368	-	+	+	-
CR43257	0.0220568	0.626195	-	+	+	-
CG8128	7.258E-06	0.6228314	-	+	+	-
CG4404	3.091E-08	0.6200191	-	+	+	-
CG17754	1.46E-06	0.614028	-	+	+	-
CG9795	4.738E-05	0.6075542	-	+	+	-
CG7675	5.557E-11	0.6062252	-	+	+	-
Nak	0.0001487	0.6041964	-	+	+	-
Acox57D-p	7.258E-06	0.6032624	-	+	+	-
sra	2.859E-05	0.5953362	-	+	+	-
Hsc70-3	4.276E-10	0.5924434	-	+	+	-
PEK	2.88E-06	0.5912829	-	+	+	-
cno	8.877E-08	0.5886729	-	+	+	-
Ptp10D	1.012E-05	0.5799094	-	+	+	-
CR43900	0.0343551	0.5785068	-	+	+	-
Ald	1.257E-13	0.5769548	-	+	+	-
ed	9.119E-06	0.5736184	-	+	+	-
shg	0.0015671	0.5711902	-	+	+	-
mnb	6.813E-06	0.5697004	-	+	+	-
Samuel	0.0080166	0.5678974	-	+	+	-
mura	1.302E-08	0.5658221	-	+	+	-
osp	4.257E-05	0.5649408	-	+	+	-
Nrt	9.796E-05	0.5633737	-	+	+	-
modSP	1.619E-06	0.5631844	-	+	+	-
CG11147	0.0002449	0.5592623	-	+	+	-
mal	0.0188409	0.5560346	-	+	+	-
Mipp1	3.191E-06	0.5557857	-	+	+	-
fray	0.0033217	0.5557517	-	+	+	-
CG42238	7.728E-07	0.5543571	-	+	+	-
sba	8.306E-05	0.5529294	-	+	+	-
koi	0.0002645	0.5496788	-	+	+	-
Hr39	0.0012906	0.5445955	-	+	+	-
Piezo	5.647E-05	0.5427962	-	+	+	-
if	1.223E-06	0.542646	-	+	+	-
CG34126	0.0016562	0.5397336	-	+	+	-
CG33298	0.0007251	0.5380044	-	+	+	-
Tsp42Ef	0.0001127	0.5364684	-	+	+	-
fz2	9.203E-05	0.5361058	-	+	+	-
ckn	0.0002006	0.5320132	-	+	+	-
CG32676	4.706E-05	0.5224279	-	+	+	-
mys	2.607E-05	0.5220167	-	+	+	-
CG11655	0.0024283	0.5176053	-	+	+	-
sprt	0.0017987	0.5172794	-	+	+	-
msn	1.313E-06	0.5169251	-	+	+	-
ex	0.0005749	0.5156776	-	+	+	-
CG43736	1.737E-06	0.5143951	-	+	+	-
yrt	0.0004395	0.5128683	-	+	+	-

gk	0.0010298	0.5101069	-	+	+	-
CG42666	0.0209259	0.5086366	-	+	+	-
wun	4.738E-05	0.5059959	-	+	+	-
CG11737	0.0005139	0.5008492	-	+	+	-
CG4374	0.0006785	0.5004855	-	+	+	-
CG11284	0.0156394	0.4998879	-	+	+	-
sdk	3.505E-05	0.4998774	-	+	+	-
Uba2	0.000476	0.4997751	-	+	+	-
Shc	0.0036331	0.4984085	-	+	+	-
CG4538	5.824E-05	0.4980224	-	+	+	-
CG4297	0.003361	0.4967544	-	+	+	-
mdy	1.753E-05	0.4911343	-	+	+	-
Eaat1	1.606E-06	0.4906966	-	+	+	-
magu	0.01075	0.4895909	-	+	+	-
Rab10	0.0017482	0.4888028	-	+	+	-
CG9005	9.203E-05	0.4886054	-	+	+	-
Mical	7.274E-05	0.4849189	-	+	+	-
dnr1	0.0001105	0.4833135	-	+	+	-
CG14441	0.0005046	0.4809023	-	+	+	-
Smg6	4.04E-05	0.4778722	-	+	+	-
CG43341	0.0044865	0.4743451	-	+	+	-
Ntf-2	0.0003708	0.4712048	-	+	+	-
olf186-F	0.000508	0.4688443	-	+	+	-
skd	0.0001577	0.4675407	-	+	+	-
trbl	0.001504	0.465898	-	+	+	-
Gprk2	5.209E-05	0.4642505	-	+	+	-
Kat60	0.0016333	0.4631748	-	+	+	-
fng	1.952E-05	0.4623251	-	+	+	-
hoe1	3.8E-05	0.4622511	-	+	+	-
Sec16	0.0130657	0.461385	-	+	+	-
Adk3	0.0120007	0.4605739	-	+	+	-
Dp1	0.0025337	0.4596623	-	+	+	-
Ptp4E	8.821E-05	0.4582798	-	+	+	-
Tep4	6.972E-07	0.4569915	-	+	+	-
MED14	0.0021041	0.4554292	-	+	+	-
CG3552	0.0241007	0.4537702	-	+	+	-
CG1299	0.001274	0.4525208	-	+	+	-
M6	0.0008249	0.4507138	-	+	+	-
CG30069	0.0040637	0.4500206	-	+	+	-
Wbp2	6.694E-06	0.4493808	-	+	+	-
CG31869	0.0115319	0.4476886	-	+	+	-
CG5002	0.0231813	0.446085	-	+	+	-
CR44987	0.0014752	0.445606	-	+	+	-
CG34383	3.395E-05	0.4443835	-	+	+	-
Tsf3	0.004513	0.437176	-	+	+	-
Jheh2	1.838E-05	0.436019	-	+	+	-
CG6791	0.0144301	0.4358688	-	+	+	-
rngo	0.0242684	0.4350527	-	+	+	-
CG31523	0.0001268	0.4343054	-	+	+	-
Eaf	0.0072899	0.4339682	-	+	+	-
Ubqn	5.892E-05	0.4335246	-	+	+	-
CG8740	0.0008117	0.4333806	-	+	+	-
Hmt4-20	0.0031209	0.4328067	-	+	+	-
Cip4	6.855E-05	0.4310866	-	+	+	-
gukh	0.0141228	0.4307226	-	+	+	-
Unr	9.088E-06	0.4273297	-	+	+	-
CR44961	0.0473922	0.4254417	-	+	+	-
Cyp4d2	0.0002476	0.4242929	-	+	+	-

Df31	0.0040933	0.4208842	-	+	+	-
Egfr	0.0002349	0.4192998	-	+	+	-
oc	0.0025589	0.418064	-	+	+	-
Pdk1	0.0001156	0.4170662	-	+	+	-
CG11873	0.0053774	0.4166441	-	+	+	-
cact	5.317E-05	0.4157714	-	+	+	-
CG2469	1.986E-05	0.4147823	-	+	+	-
Cad87A	0.0092355	0.4135454	-	+	+	-
CG12262	2.206E-05	0.4131324	-	+	+	-
Zasp52	0.0045784	0.412787	-	+	+	-
CG15093	0.0039379	0.4098144	-	+	+	-
Su(Tpl)	2.065E-05	0.4096957	-	+	+	-
mbc	0.0075458	0.4096805	-	+	+	-
drpr	0.0051566	0.4067672	-	+	+	-
Ctr1A	0.0061074	0.401328	-	+	+	-
CG4022	0.0448201	0.4007161	-	+	+	-
Gp93	0.0020931	0.4006387	-	+	+	-
CG10527	0.0001403	0.3989528	-	+	+	-
CG9008	0.0104263	0.3984762	-	+	+	-
CG14132	0.0009844	0.3983986	-	+	+	-
CG3036	2.972E-05	0.3967624	-	+	+	-
Atg1	0.0183153	0.3966232	-	+	+	-
CG42674	0.0014733	0.3958351	-	+	+	-
Pmp70	0.0006932	0.3934101	-	+	+	-
l(3)73Ah	0.0117112	0.3930298	-	+	+	-
rdgA	0.0003019	0.3924688	-	+	+	-
Karybeta3	0.0001567	0.3911129	-	+	+	-
mthl1	0.0329454	0.3898251	-	+	+	-
SF2	0.0003219	0.3877224	-	+	+	-
Fur1	0.0028202	0.3859217	-	+	+	-
Pvr	0.00659	0.3855978	-	+	+	-
CG15083	0.0131398	0.3848805	-	+	+	-
CG33056	0.001136	0.3842157	-	+	+	-
pdgy	9.203E-05	0.3835886	-	+	+	-
Dscam1	0.0022198	0.3833331	-	+	+	-
Sxl	0.0002851	0.3805278	-	+	+	-
CG11883	0.0185765	0.3803713	-	+	+	-
Zw	0.0069858	0.3794308	-	+	+	-
CG4452	0.0145945	0.3754543	-	+	+	-
luna	0.0284211	0.3743154	-	+	+	-
Pih1D1	0.001373	0.3723038	-	+	+	-
CG14995	0.0464527	0.3712098	-	+	+	-
Adar	0.0007793	0.3705458	-	+	+	-
AdSS	0.0004035	0.3697445	-	+	+	-
step	0.0227925	0.3656496	-	+	+	-
ATP7	0.0287819	0.3633549	-	+	+	-
Top3beta	0.0336099	0.3593776	-	+	+	-
tai	0.0038458	0.3578428	-	+	+	-
RhoGAP71E	0.0101838	0.3570789	-	+	+	-
CG42389	0.0074149	0.3539747	-	+	+	-
CG8229	0.0107626	0.3527221	-	+	+	-
Efa6	0.0146164	0.348348	-	+	+	-
CG17111	0.0011293	0.3483286	-	+	+	-
CG18659	0.0181231	0.3465942	-	+	+	-
aos	0.019858	0.3449385	-	+	+	-
CG42327	0.0129434	0.3444921	-	+	+	-
Tfb1	0.0451081	0.3422246	-	+	+	-
ctrip	0.0473114	0.3406872	-	+	+	-

SP1173	0.0246141	0.3396982	-	+	+	-
G9a	0.0284243	0.339654	-	+	+	-
CG12814	0.0315971	0.3386827	-	+	+	-
CG2186	0.0087301	0.3385836	-	+	+	-
Mhcl	0.0151955	0.3379993	-	+	+	-
Ac76E	0.0447842	0.3355584	-	+	+	-
CG8929	0.0250018	0.335098	-	+	+	-
CG30122	0.0009184	0.3350818	-	+	+	-
crb	0.0137464	0.3333743	-	+	+	-
CG8036	0.0006367	0.3333596	-	+	+	-
Jra	0.0182879	0.3311418	-	+	+	-
Hsf	0.0073962	0.3267705	-	+	+	-
Hr78	0.0133798	0.3260839	-	+	+	-
Rip11	0.0057738	0.3257971	-	+	+	-
atl	0.0029513	0.3245508	-	+	+	-
CG34408	0.0182837	0.3243915	-	+	+	-
EDTP	0.0076934	0.3226624	-	+	+	-
Cnx99A	0.0026257	0.3198436	-	+	+	-
CG5674	0.020951	0.3194991	-	+	+	-
Kul	0.0173402	0.3181252	-	+	+	-
NT5E-2	0.0243281	0.3173268	-	+	+	-
CG31635	0.0150149	0.3149449	-	+	+	-
CG10413	0.0228834	0.3137972	-	+	+	-
ftz-f1	0.0069507	0.3137656	-	+	+	-
GlcT-1	0.0179434	0.3114423	-	+	+	-
put	0.0321457	0.309728	-	+	+	-
Gmap	0.0228509	0.3080185	-	+	+	-
Meltrin	0.0299397	0.3074113	-	+	+	-
Cirl	0.0109797	0.3012602	-	+	+	-
Mi-2	0.024575	0.2996105	-	+	+	-
Stat92E	0.0023591	0.2978677	-	+	+	-
Hmu	0.0330124	0.2972497	-	+	+	-
CG31808	0.0171898	0.2970161	-	+	+	-
DIP1	0.0325744	0.2964103	-	+	+	-
Vav	0.0273106	0.2943913	-	+	+	-
rdx	0.0248813	0.2941334	-	+	+	-
ens	0.0234177	0.2932286	-	+	+	-
pr	0.0223375	0.2911234	-	+	+	-
Eip75B	0.046874	0.2904037	-	+	+	-
CG42542	0.0230092	0.2895571	-	+	+	-
elav	0.0093667	0.2885592	-	+	+	-
CG10082	0.0021936	0.2880516	-	+	+	-
Kr-h2	0.0335569	0.2879505	-	+	+	-
Trx-2	0.0250891	0.2875986	-	+	+	-
SkpA	0.0232438	0.2873464	-	+	+	-
RhoGEF2	0.0103318	0.287069	-	+	+	-
Pde8	0.0260883	0.2866307	-	+	+	-
CG3168	0.0178218	0.2860125	-	+	+	-
CG14438	0.0201502	0.2818079	-	+	+	-
BtbVII	0.0092316	0.2808659	-	+	+	-
Rbp1-like	0.0062521	0.2769478	-	+	+	-
santa-maria	0.0111271	0.2725392	-	+	+	-
inaE	0.0468789	0.268687	-	+	+	-
Trf2	0.0365487	0.2655432	-	+	+	-
Tsp74F	0.034017	0.2649102	-	+	+	-
Prosap	0.0152221	0.2601801	-	+	+	-
SNF4Agamma	0.0210231	0.2600521	-	+	+	-
Got2	0.021016	0.2549364	-	+	+	-

AnxB11	0.0454723	0.2541368	-	+	+	-
ine	0.0287819	0.2519909	-	+	+	-
Cp1	0.0045575	0.2488228	-	+	+	-
Men	0.0169475	0.2486861	-	+	+	-
Dek	0.0400732	0.2455529	-	+	+	-
Xe7	0.0427523	0.2326145	-	+	+	-
Bsg	0.0159937	0.2324672	-	+	+	-
lolal	0.0472853	0.2286844	-	+	+	-
CG16953	0.0496454	0.2063358	-	+	+	-
alphagamma-el	0	5.5928615	-	+	-	-
Hsp27	0	5.0253504	-	+	-	-
Hsp68	0	4.8393569	-	+	-	-
Hsp26	0	4.6307071	-	+	-	-
CG5290	1.4E-171	3.5131249	-	+	-	-
Hsp70Ba	1.084E-33	3.3897953	-	+	-	-
CR42765	5.386E-29	2.7200533	-	+	-	-
Pa1	4.23E-83	2.2451208	-	+	-	-
CR43465	4.07E-11	2.0835189	-	+	-	-
verm	1.199E-47	1.9414917	-	+	-	-
Faa	1.338E-64	1.8731707	-	+	-	-
CG12643	1.15E-21	1.8604524	-	+	-	-
Cpr97Ea	8.283E-57	1.8122014	-	+	-	-
Cyp4e3	1.155E-46	1.6620536	-	+	-	-
CG8483	9.434E-22	1.6015253	-	+	-	-
CG14186	2.206E-55	1.5966348	-	+	-	-
CG16885	9.648E-75	1.5770275	-	+	-	-
Mrp4	3.917E-54	1.569465	-	+	-	-
CG11700	6.59E-07	1.5458309	-	+	-	-
GstE8	6.196E-12	1.4866883	-	+	-	-
Cht7	6.054E-27	1.4634153	-	+	-	-
serp	4.843E-59	1.4590785	-	+	-	-
CecB	2.222E-05	1.4476136	-	+	-	-
GstE10	2.709E-08	1.3982027	-	+	-	-
GstE7	1.921E-20	1.3584838	-	+	-	-
GstE3	4.785E-37	1.3511733	-	+	-	-
CG2065	1.084E-33	1.3431674	-	+	-	-
ref(2)P	1.003E-58	1.3393737	-	+	-	-
CG12290	1.614E-31	1.3073736	-	+	-	-
CG2650	5.637E-39	1.2711035	-	+	-	-
CG12224	3.819E-23	1.2616691	-	+	-	-
Cctgamma	4.096E-24	1.2560014	-	+	-	-
GstE5	1.916E-11	1.2362135	-	+	-	-
dlt	1.619E-06	1.2354436	-	+	-	-
Gasp	9.429E-28	1.1860175	-	+	-	-
GstE1	1.458E-25	1.1776774	-	+	-	-
CG16959	7.046E-10	1.1727819	-	+	-	-
CR43481	7.825E-13	1.1719157	-	+	-	-
bnl	1.035E-12	1.1644436	-	+	-	-
CG11453	1.599E-13	1.1623828	-	+	-	-
ds	5.705E-13	1.1372773	-	+	-	-
CR44769	0.0001413	1.1342809	-	+	-	-
mtg	3.485E-13	1.0980119	-	+	-	-
Clk	5.011E-22	1.0927158	-	+	-	-
Taf7	1.927E-08	1.0844412	-	+	-	-
CR43838	0.0002299	1.0677175	-	+	-	-
CG6675	2.799E-09	1.0673209	-	+	-	-
ade2	1.317E-28	1.0653249	-	+	-	-
CG10936	1.542E-12	1.0646238	-	+	-	-

CG9850	7.105E-16	1.0538095	-	+	-	-
spz5	1.824E-10	1.0242657	-	+	-	-
laccase2	3.522E-13	1.0170436	-	+	-	-
CG12985	2.883E-05	1.0103231	-	+	-	-
CG6739	1.676E-14	1.0043856	-	+	-	-
CG30438	1.263E-11	0.9814959	-	+	-	-
CR45018	4.41E-05	0.9787725	-	+	-	-
sas	4.219E-18	0.9614223	-	+	-	-
comm2	1.594E-13	0.9605781	-	+	-	-
obst-B	1.157E-10	0.9556665	-	+	-	-
CG17834	2.097E-11	0.9356763	-	+	-	-
CG40006	1.8E-19	0.9271956	-	+	-	-
CG14892	1.547E-21	0.9252123	-	+	-	-
mthl9	0.0047485	0.9244752	-	+	-	-
knk	2.128E-07	0.9240843	-	+	-	-
mfas	5.099E-27	0.9226872	-	+	-	-
CG30427	3.981E-15	0.9214581	-	+	-	-
CG15544	9.627E-11	0.9139956	-	+	-	-
lush	0.0001456	0.9119765	-	+	-	-
CG15021	2.367E-08	0.911581	-	+	-	-
CR32636	0.0076432	0.9079393	-	+	-	-
AOX1	1.386E-16	0.9066481	-	+	-	-
CG9095	0.004041	0.9059822	-	+	-	-
CR44108	0.0001653	0.8959079	-	+	-	-
CG34137	0.0064917	0.8932339	-	+	-	-
Obp83g	0.0001161	0.8927817	-	+	-	-
CG42240	2.266E-13	0.8925185	-	+	-	-
Cyp301a1	0.0083446	0.8917789	-	+	-	-
CG10026	4.987E-10	0.8843974	-	+	-	-
Cdc37	1.813E-15	0.8832901	-	+	-	-
Cyp6a21	8.916E-08	0.8799998	-	+	-	-
CG32816	1.416E-05	0.8783899	-	+	-	-
CG10737	3.175E-14	0.8556403	-	+	-	-
rols	1.082E-07	0.848987	-	+	-	-
CG15879	9.738E-07	0.8473892	-	+	-	-
CAHbeta	7.592E-08	0.8450072	-	+	-	-
CG5509	0.0103318	0.8430245	-	+	-	-
CG4982	0.0257582	0.8429829	-	+	-	-
CG34347	7.578E-08	0.8363936	-	+	-	-
CG4733	5.412E-10	0.8261195	-	+	-	-
CG6908	4.806E-18	0.8260895	-	+	-	-
CR44321	0.0001586	0.8229063	-	+	-	-
CG15506	1.159E-06	0.8221923	-	+	-	-
sv	5.484E-12	0.8210823	-	+	-	-
mol	7.592E-08	0.8207019	-	+	-	-
CG10237	2.587E-15	0.8120552	-	+	-	-
tnc	1.619E-06	0.8090915	-	+	-	-
CG13116	5.379E-11	0.8023001	-	+	-	-
CG17919	2.088E-15	0.8012881	-	+	-	-
CG32170	7.03E-05	0.798325	-	+	-	-
CG1673	2.181E-10	0.7923418	-	+	-	-
CG10345	2.373E-20	0.7919249	-	+	-	-
Cad86C	1.606E-05	0.7911811	-	+	-	-
CR43452	0.0002911	0.7910683	-	+	-	-
Nplp3	1.264E-24	0.7902551	-	+	-	-
haf	6.562E-06	0.7894126	-	+	-	-
GstE6	3.695E-10	0.789296	-	+	-	-
Mtap	1.092E-06	0.7857927	-	+	-	-

CG14879	0.0211635	0.7832064	-	+	-	-
CG30440	6.444E-05	0.7817463	-	+	-	-
CG15695	0.0024545	0.7796991	-	+	-	-
CG7778	4.806E-18	0.7754118	-	+	-	-
pot	2.974E-09	0.7723883	-	+	-	-
ple	6.173E-12	0.771165	-	+	-	-
Cyp6a2	0.0047447	0.7696916	-	+	-	-
how	4.247E-10	0.7689678	-	+	-	-
bnb	1.216E-11	0.764796	-	+	-	-
AnxB9	6.677E-18	0.7642017	-	+	-	-
CG7079	1.021E-13	0.7564588	-	+	-	-
CG34010	0.0480098	0.7459189	-	+	-	-
ci	1.379E-05	0.7424252	-	+	-	-
bru-2	2.034E-06	0.7423179	-	+	-	-
CG2781	3.821E-17	0.7364618	-	+	-	-
CG9701	1.258E-12	0.7340687	-	+	-	-
r-cup	0.0409064	0.7336965	-	+	-	-
lab	0.0001668	0.7329577	-	+	-	-
Myo10A	3.362E-05	0.7310936	-	+	-	-
CG34398	7.759E-07	0.7297255	-	+	-	-
CG4267	2.204E-05	0.7290675	-	+	-	-
CG42808	2.109E-05	0.7287408	-	+	-	-
CG13055	0.006723	0.7254071	-	+	-	-
plum	4.536E-06	0.7252248	-	+	-	-
CG15546	2.616E-05	0.7209164	-	+	-	-
yin	5.557E-11	0.716081	-	+	-	-
GstO3	3.074E-07	0.7131412	-	+	-	-
Osi24	0.0046276	0.7075549	-	+	-	-
CG7900	0.0113808	0.7050043	-	+	-	-
Skeletor	7.697E-07	0.7033575	-	+	-	-
Cyp6a23	8.962E-07	0.6956725	-	+	-	-
Fmo-2	5.287E-10	0.6934381	-	+	-	-
CG5191	8.178E-08	0.6931521	-	+	-	-
alpha-Est1	2.875E-10	0.6917654	-	+	-	-
CG5381	1.967E-05	0.691517	-	+	-	-
CG34120	3.249E-13	0.6895306	-	+	-	-
trol	1.526E-16	0.6871058	-	+	-	-
CG2816	0.0456069	0.6856384	-	+	-	-
vn	0.0004328	0.6766202	-	+	-	-
CG15365	0.0002163	0.6764484	-	+	-	-
yellow-c	1.972E-13	0.6752654	-	+	-	-
CG31207	9.35E-12	0.6707971	-	+	-	-
CG5921	0.0002681	0.6707576	-	+	-	-
CG5835	1.348E-09	0.6648609	-	+	-	-
qsm	4.734E-11	0.6628839	-	+	-	-
drm	0.0029931	0.662144	-	+	-	-
Idh	1.444E-14	0.660498	-	+	-	-
CG9186	3.435E-09	0.6534534	-	+	-	-
CG3655	4.344E-07	0.6518977	-	+	-	-
insc	0.0013264	0.6493008	-	+	-	-
CG33970	1.68E-12	0.6492287	-	+	-	-
CG42272	0.0109278	0.6490793	-	+	-	-
kermit	0.0267936	0.6415413	-	+	-	-
CG31522	3.719E-10	0.641026	-	+	-	-
Tollo	0.0019992	0.6400584	-	+	-	-
CG30460	6.681E-05	0.6398759	-	+	-	-
hdly	2.014E-11	0.6379159	-	+	-	-
CG14694	8.787E-05	0.637685	-	+	-	-

Droj2	3.488E-10	0.6308438	-	+	-	-
CG30284	0.0156213	0.6295461	-	+	-	-
CG12535	0.003832	0.6287417	-	+	-	-
CG4000	5.336E-11	0.6285094	-	+	-	-
Phk-3	7.021E-05	0.6267343	-	+	-	-
CG9527	1.837E-05	0.6249093	-	+	-	-
Sulf1	3.776E-09	0.6241955	-	+	-	-
CG6175	0.0014446	0.6221244	-	+	-	-
CG3842	1.062E-09	0.6199013	-	+	-	-
CG16772	7.885E-10	0.6197183	-	+	-	-
mth	0.0003868	0.6137387	-	+	-	-
dgo	0.0103318	0.6132597	-	+	-	-
CG42748	2.698E-10	0.6099793	-	+	-	-
Ect4	3.204E-06	0.6093222	-	+	-	-
CR43253	0.0110046	0.608221	-	+	-	-
RhoGAP15B	0.0012036	0.6060581	-	+	-	-
CG8303	0.0159321	0.6054081	-	+	-	-
CG31809	0.000268	0.6032121	-	+	-	-
CG33275	3.154E-05	0.6009215	-	+	-	-
spir	3.55E-07	0.6009198	-	+	-	-
out	6.601E-07	0.5992142	-	+	-	-
CG43394	4.2E-05	0.5967208	-	+	-	-
CG17778	4.299E-06	0.5946642	-	+	-	-
CG43935	0.0031706	0.5945218	-	+	-	-
CG43693	4.502E-07	0.5944055	-	+	-	-
CG17027	0.0003377	0.5916549	-	+	-	-
CG34325	2.813E-05	0.5860245	-	+	-	-
PGRP-LC	2.25E-08	0.582703	-	+	-	-
slp2	0.0207613	0.5821416	-	+	-	-
fog	0.0001175	0.5769196	-	+	-	-
be	0.0006098	0.5741767	-	+	-	-
Tie	6.313E-07	0.5740678	-	+	-	-
CG10176	0.0002255	0.5735205	-	+	-	-
Cad96Ca	2.228E-05	0.5721527	-	+	-	-
CG44245	1.8E-07	0.5714285	-	+	-	-
aay	5.08E-08	0.5707895	-	+	-	-
Cpr49Ae	1.701E-09	0.5695502	-	+	-	-
Spn28Dc	6.216E-07	0.5684292	-	+	-	-
ptr	0.0001078	0.5682956	-	+	-	-
CG2254	1.145E-12	0.5674568	-	+	-	-
fj	0.0118203	0.5654112	-	+	-	-
Pvf1	0.0001108	0.5618899	-	+	-	-
CG31176	0.0133131	0.5613816	-	+	-	-
CG14696	0.0095325	0.5579837	-	+	-	-
Shroom	4.947E-06	0.5552596	-	+	-	-
CR43459	0.0378271	0.5528282	-	+	-	-
SKIP	0.0193687	0.549375	-	+	-	-
CG1774	0.011487	0.5480201	-	+	-	-
CG30456	0.0015636	0.5460598	-	+	-	-
CG9839	0.0014564	0.5441365	-	+	-	-
Rgl	2.499E-05	0.5404627	-	+	-	-
CG34417	5.66E-08	0.5396082	-	+	-	-
AdamTS-A	0.018961	0.5371941	-	+	-	-
mab-21	0.0013413	0.5367397	-	+	-	-
lz	0.001523	0.5366465	-	+	-	-
disco-r	0.0004655	0.5362016	-	+	-	-
Ten-m	3.614E-06	0.5360491	-	+	-	-
CG1927	7.582E-07	0.5340236	-	+	-	-

CG16700	0.0001839	0.5305821	-	+	-	-
CG43163	0.0161519	0.5291225	-	+	-	-
hgo	4.402E-08	0.5289256	-	+	-	-
CG13366	0.0004748	0.5289131	-	+	-	-
CG11951	0.0002973	0.5279105	-	+	-	-
Irk1	0.0041423	0.5264047	-	+	-	-
Ance	0.0003066	0.5220812	-	+	-	-
Dys	9.818E-06	0.5201036	-	+	-	-
NijA	0.0003005	0.5194875	-	+	-	-
Cht2	0.0001571	0.5184685	-	+	-	-
msi	3.997E-07	0.5180119	-	+	-	-
CG7970	0.0011274	0.5176979	-	+	-	-
Btk29A	0.0071342	0.516955	-	+	-	-
Thd1	0.0007296	0.5153564	-	+	-	-
Fs	0.0001487	0.5151192	-	+	-	-
CG10562	0.0022198	0.5147479	-	+	-	-
GstZ2	7.374E-05	0.5104062	-	+	-	-
CG34371	0.0005471	0.5098835	-	+	-	-
5-HT2A	2.499E-05	0.5098718	-	+	-	-
CG13101	0.0007217	0.5090778	-	+	-	-
Fas3	8.117E-07	0.5090153	-	+	-	-
RhoGEF64C	3.811E-05	0.5082591	-	+	-	-
e	0.000159	0.5071865	-	+	-	-
CG3328	0.002006	0.5062039	-	+	-	-
CG9920	0.0055458	0.5043537	-	+	-	-
LRP1	2.023E-05	0.5037887	-	+	-	-
dib	0.0040599	0.5034958	-	+	-	-
CLIP-190	2.581E-06	0.5019507	-	+	-	-
sog	0.0003635	0.5008039	-	+	-	-
Nha2	0.0007189	0.4974974	-	+	-	-
ZnT41F	0.0015394	0.4861877	-	+	-	-
Cyp6a9	0.0003913	0.4813822	-	+	-	-
CG7497	0.0062521	0.4811295	-	+	-	-
cnn	7.739E-06	0.4805082	-	+	-	-
MESR3	0.0062521	0.4796747	-	+	-	-
MESR6	0.0084089	0.4794829	-	+	-	-
CG6701	0.0015998	0.4775821	-	+	-	-
CG9733	0.0118861	0.4772688	-	+	-	-
CG8086	5.24E-06	0.4748265	-	+	-	-
Rcd2	0.0349117	0.4738251	-	+	-	-
Mst85C	0.005813	0.4726815	-	+	-	-
Act57B	0.0001373	0.4723659	-	+	-	-
toy	0.0011027	0.4705037	-	+	-	-
CG5945	8.361E-05	0.4703405	-	+	-	-
Mvl	0.0033625	0.4689976	-	+	-	-
CR44107	0.0030268	0.4663297	-	+	-	-
Ac13E	2.972E-05	0.4649026	-	+	-	-
nrm	2.357E-05	0.4638584	-	+	-	-
CG13024	0.003832	0.4633966	-	+	-	-
Tg	0.0025337	0.4618901	-	+	-	-
GstO2	0.002535	0.4615879	-	+	-	-
CG14830	0.0001329	0.4615414	-	+	-	-
cana	0.040752	0.4612876	-	+	-	-
Ddr	0.0496087	0.4610161	-	+	-	-
cv-c	3.072E-05	0.459328	-	+	-	-
slif	0.0109726	0.458305	-	+	-	-
Nep2	0.0058258	0.4582911	-	+	-	-
Pgd	0.0001325	0.4564212	-	+	-	-

CG3301	4.571E-06	0.4559595	-	+	-	-
Pur-alpha	2.721E-05	0.4547085	-	+	-	-
CG2930	0.0015831	0.4521299	-	+	-	-
CG7668	0.0044981	0.4520458	-	+	-	-
Tsp	2.76E-06	0.451901	-	+	-	-
Trpm	0.0002802	0.4497341	-	+	-	-
Prm	0.0280896	0.4468614	-	+	-	-
CG11407	1.245E-06	0.4466752	-	+	-	-
NKAIN	0.0001057	0.446182	-	+	-	-
CG4991	0.0011875	0.4459171	-	+	-	-
CHKov2	0.0020397	0.4429944	-	+	-	-
CG1640	6.689E-06	0.4402522	-	+	-	-
ps	1.72E-06	0.4348859	-	+	-	-
gl	0.0014915	0.4338231	-	+	-	-
l(1)G0289	0.0001801	0.4313631	-	+	-	-
CG10462	0.0043075	0.4281103	-	+	-	-
CG32407	0.0030059	0.4276557	-	+	-	-
CG1806	0.0119252	0.4241041	-	+	-	-
toe	0.0442117	0.424032	-	+	-	-
plx	0.0025982	0.4219732	-	+	-	-
CG13895	0.0005307	0.4207779	-	+	-	-
mim	0.0002147	0.4191308	-	+	-	-
Atf3	0.0018041	0.4186316	-	+	-	-
CG5541	0.0219816	0.418602	-	+	-	-
CR45102	0.0215529	0.4172679	-	+	-	-
comm3	0.0289323	0.4161241	-	+	-	-
CG14439	6.269E-05	0.4153909	-	+	-	-
r2d2	0.0142575	0.4089042	-	+	-	-
SLC5A11	0.0016333	0.4084176	-	+	-	-
CG3829	0.0003491	0.4076016	-	+	-	-
app	2.607E-05	0.405568	-	+	-	-
Dfd	0.0249007	0.4049162	-	+	-	-
GLaz	9.209E-05	0.404467	-	+	-	-
blot	0.0031243	0.4040957	-	+	-	-
CG5909	0.0297268	0.40341	-	+	-	-
Jheh1	0.0007093	0.402776	-	+	-	-
Cdk4	0.0329536	0.4017297	-	+	-	-
CG1648	3.849E-06	0.4004259	-	+	-	-
CanB2	0.0002443	0.4000916	-	+	-	-
yuri	0.0119372	0.4000121	-	+	-	-
ITP	0.0008664	0.3985354	-	+	-	-
CG42671	0.0001253	0.3981851	-	+	-	-
CrebA	0.0359706	0.3978726	-	+	-	-
Src42A	0.0149663	0.395986	-	+	-	-
LanB2	0.0387998	0.3958905	-	+	-	-
mwh	0.0137922	0.3958436	-	+	-	-
dsd	0.0175257	0.3952503	-	+	-	-
CAP	9.86E-05	0.3948931	-	+	-	-
atilla	0.0006167	0.3932919	-	+	-	-
CG1888	0.0086544	0.3922634	-	+	-	-
CG15279	0.0032314	0.3908165	-	+	-	-
Cyp305a1	0.0016284	0.3907098	-	+	-	-
Pgi	2.691E-05	0.3851836	-	+	-	-
CG18769	0.0014876	0.3850103	-	+	-	-
Blimp-1	0.0066697	0.3790249	-	+	-	-
CG2182	0.0016071	0.3772734	-	+	-	-
CG34394	0.0001399	0.3755504	-	+	-	-
CG11426	0.0111271	0.3755198	-	+	-	-

SP1029	0.0120826	0.3730945	-	+	-	-
CG2993	0.0007449	0.3722214	-	+	-	-
RNaseX25	0.0099335	0.3708575	-	+	-	-
CG9360	0.0385388	0.3696556	-	+	-	-
ey	0.0017013	0.3673028	-	+	-	-
cpo	0.000486	0.3670864	-	+	-	-
Klp98A	0.0083561	0.3661744	-	+	-	-
C3G	0.0010023	0.3643575	-	+	-	-
Patronin	0.001403	0.3619348	-	+	-	-
sano	0.0325575	0.3615196	-	+	-	-
Mbs	0.0007225	0.3606376	-	+	-	-
CG17119	0.0388647	0.3598503	-	+	-	-
CG9717	0.0045063	0.3586177	-	+	-	-
baz	0.0015696	0.3583474	-	+	-	-
Nfl	0.0039548	0.3581251	-	+	-	-
CG9727	0.0277272	0.3581032	-	+	-	-
FER	0.0004118	0.3564893	-	+	-	-
pgant5	0.0040599	0.3563914	-	+	-	-
Kal1	0.0047339	0.3562286	-	+	-	-
ths	0.003605	0.3560268	-	+	-	-
Bsg25D	0.0262311	0.3542318	-	+	-	-
Chd64	0.0001329	0.3535138	-	+	-	-
CG4619	0.0018311	0.3506797	-	+	-	-
Cpr76Bd	0.0274383	0.3506225	-	+	-	-
Csk	0.0333147	0.3442583	-	+	-	-
Cyp4d20	0.000434	0.3422401	-	+	-	-
NK7.1	0.016123	0.3414556	-	+	-	-
Mf	0.0223839	0.3377615	-	+	-	-
unc-13	0.0006866	0.334777	-	+	-	-
Ca-beta	0.029781	0.3345227	-	+	-	-
CG34380	0.0153187	0.3342135	-	+	-	-
Nrx-IV	0.01075	0.3338862	-	+	-	-
CG42784	0.0008249	0.3319673	-	+	-	-
Cyp6d5	0.0013251	0.3319618	-	+	-	-
CG4080	0.0062051	0.3292463	-	+	-	-
Sod3	0.003066	0.3277797	-	+	-	-
CG31200	0.002006	0.322797	-	+	-	-
CG34376	0.0131188	0.3212085	-	+	-	-
CG32280	0.024575	0.320953	-	+	-	-
Pak	0.0117148	0.3186768	-	+	-	-
mgl	0.0196658	0.3185499	-	+	-	-
Chro	0.0311292	0.3154825	-	+	-	-
sm	0.0046186	0.3140033	-	+	-	-
CG33110	0.0042753	0.3129929	-	+	-	-
ClC-a	0.003714	0.3128044	-	+	-	-
Ace	0.0017376	0.3109281	-	+	-	-
drk	0.0228834	0.3096385	-	+	-	-
AP-1gamma	0.0054171	0.3095854	-	+	-	-
CG8184	0.0184085	0.3088541	-	+	-	-
capu	0.0062435	0.3062919	-	+	-	-
blow	0.03694	0.303811	-	+	-	-
CG10178	0.0110523	0.3015974	-	+	-	-
Cyp6a17	0.0228016	0.3012266	-	+	-	-
Hex-A	0.0137922	0.296618	-	+	-	-
CR44390	0.0373395	0.2962209	-	+	-	-
CG12207	0.0241118	0.2905275	-	+	-	-
NaCP60E	0.0035355	0.2898364	-	+	-	-
CG31705	0.0049551	0.2883585	-	+	-	-

rin	0.0100708	0.2883034	-	+	-	-
CG10680	0.0197029	0.2859156	-	+	-	-
SP2637	0.0289143	0.2855726	-	+	-	-
nAChRalpha5	0.0160453	0.2835679	-	+	-	-
hppy	0.020697	0.2832923	-	+	-	-
CG45050	0.0382827	0.2823186	-	+	-	-
Calx	0.0218517	0.2820105	-	+	-	-
Cyp12a4	0.0240956	0.281587	-	+	-	-
CG9572	0.0493555	0.2775515	-	+	-	-
Acsl	0.0087696	0.2757455	-	+	-	-
ct	0.0428134	0.2703958	-	+	-	-
roX1	0.0101008	0.2697012	-	+	-	-
HDAC4	0.0392269	0.2694408	-	+	-	-
l(1)G0196	0.0054092	0.268298	-	+	-	-
Fas1	0.0321111	0.2642022	-	+	-	-
cg	0.0435249	0.2637331	-	+	-	-
RN-tre	0.0462309	0.2564625	-	+	-	-
Sh	0.0371534	0.2538248	-	+	-	-
Tret1-1	0.0182371	0.2528711	-	+	-	-
CG9674	0.023574	0.2469898	-	+	-	-
Octbeta2R	0.0331445	0.2453472	-	+	-	-
lqf	0.0258593	0.2433771	-	+	-	-
sima	0.0340149	0.2433152	-	+	-	-
CG17549	0.0225304	0.2254686	-	+	-	-
CG6409	0.0409064	0.1979628	-	+	-	-
CG3226	7.654E-13	1.1126849	-	-	+	-
CG34409	7.966E-11	0.9753952	-	-	+	-
mus205	8.037E-11	0.9301904	-	-	+	-
CG43313	2.228E-07	0.9174168	-	-	+	-
CG5525	8.499E-18	0.9171983	-	-	+	-
T-cp1	3.096E-10	0.911344	-	-	+	-
CG42360	2.521E-05	0.8155903	-	-	+	-
Tcp-1eta	0.0003904	0.6573091	-	-	+	-
Tcp-1zeta	1.974E-06	0.6502018	-	-	+	-
rtet	0.0005228	0.6415176	-	-	+	-
CG11089	4.502E-07	0.6404973	-	-	+	-
CIAPIN1	2.138E-12	0.6351842	-	-	+	-
nclb	0.0060139	0.6252714	-	-	+	-
CG3038	0.0034936	0.6202165	-	-	+	-
Rpn6	0.0006167	0.5676282	-	-	+	-
Abp1	0.0024919	0.5621353	-	-	+	-
CG10576	0.0001525	0.5523558	-	-	+	-
ZAP3	7.587E-08	0.5233941	-	-	+	-
Crag	0.0001127	0.514296	-	-	+	-
CG11360	3.199E-07	0.5135391	-	-	+	-
CG14353	0.0457725	0.5024869	-	-	+	-
unc-45	0.0030674	0.4858353	-	-	+	-
CG8944	0.0226515	0.4856579	-	-	+	-
sbr	5.016E-07	0.4477293	-	-	+	-
Mio	0.0001663	0.4323495	-	-	+	-
CycD	0.0417854	0.4302206	-	-	+	-
CG3308	0.000311	0.4248183	-	-	+	-
CG10516	0.004512	0.4084588	-	-	+	-
CG7766	1.266E-05	0.4014969	-	-	+	-
CG7461	0.0048853	0.3921067	-	-	+	-
CG1109	0.0270608	0.3733685	-	-	+	-
CG6967	0.0420137	0.3708813	-	-	+	-
vig2	0.0197762	0.3344718	-	-	+	-

Thiolase	0.01242	0.3313541	-	-	+	-
CG9776	0.0019625	0.3285483	-	-	+	-
Mcr	0.0385644	0.325058	-	-	+	-
Ehbp1	0.0174917	0.3006158	-	-	+	-
Aac11	0.011688	0.2976636	-	-	+	-
rempA	0.0133481	0.2889218	-	-	+	-
babo	0.0278498	0.2887342	-	-	+	-
CG10103	0.0325744	0.2865724	-	-	+	-
CG10803	0.0398706	0.272477	-	-	+	-
CG7206	0.0155923	0.2713552	-	-	+	-
CG16817	0.0271012	0.2623899	-	-	+	-
tud	0.0390162	0.249814	-	-	+	-
eag	0.0366914	0.2398633	-	-	+	-
Nmda1	0.0344096	0.2325934	-	-	+	-
Hsp70Bc	0	5.488537	-	-	-	-
Hsp70Bbb	1.36E-128	5.0005527	-	-	-	-
Hsp23	4.07E-208	2.7410785	-	-	-	-
Hsp67Bc	1.909E-78	2.700527	-	-	-	-
CG13043	2.131E-62	2.5648242	-	-	-	-
Cpr66D	7.291E-38	2.5606357	-	-	-	-
CG13044	1.618E-24	2.4848187	-	-	-	-
CG5172	5.519E-53	2.4392272	-	-	-	-
Cpr49Ac	6.801E-14	2.240673	-	-	-	-
Pxd	2.546E-32	2.1836092	-	-	-	-
CR43482	4.888E-12	2.1692669	-	-	-	-
CG17298	3.091E-57	2.1124869	-	-	-	-
CG3281	6.415E-48	2.0731571	-	-	-	-
CR45890	9.804E-14	2.0406351	-	-	-	-
CG10625	6.001E-17	1.8424178	-	-	-	-
Cpr62Bc	3.26E-50	1.7999574	-	-	-	-
CG43965	1.555E-15	1.7906257	-	-	-	-
CG8736	6.235E-96	1.7638316	-	-	-	-
CG6511	3.492E-28	1.74732	-	-	-	-
CG34232	5.71E-22	1.7181148	-	-	-	-
CG12998	1.198E-10	1.7021855	-	-	-	-
Ccp84Aa	1.199E-12	1.681184	-	-	-	-
Ir21a	9.176E-16	1.6676356	-	-	-	-
CG13926	2.258E-22	1.6620241	-	-	-	-
CR43609	6.316E-12	1.6590687	-	-	-	-
CG34217	9.653E-09	1.6433014	-	-	-	-
Acp1	1.449E-62	1.6117895	-	-	-	-
Ucp4B	1.82E-07	1.5885935	-	-	-	-
CG16884	4.115E-54	1.5646132	-	-	-	-
CG13722	5.462E-38	1.5420302	-	-	-	-
ninaD	3.277E-06	1.5394591	-	-	-	-
CR45814	1.323E-15	1.5325867	-	-	-	-
Ccp84Ab	1.164E-29	1.5271595	-	-	-	-
CG1416	5.804E-59	1.525152	-	-	-	-
CG8908	2.341E-12	1.5249625	-	-	-	-
snoRNA:Psi28S	1.44E-07	1.5040413	-	-	-	-
CG7130	6.561E-17	1.4849583	-	-	-	-
CG1979	3.454E-07	1.4582586	-	-	-	-
kkv	2.755E-25	1.4536861	-	-	-	-
Cpr100A	1.713E-27	1.4111099	-	-	-	-
CG31296	4.896E-05	1.3832859	-	-	-	-
fzo	2.087E-05	1.368624	-	-	-	-
Cct5	2.484E-32	1.3675507	-	-	-	-
yellow-k	7.588E-05	1.353758	-	-	-	-

CG16719	2.532E-05	1.3536516	-	-	-	-
CG14961	8.332E-09	1.3334226	-	-	-	-
CG42323	4.854E-12	1.3250793	-	-	-	-
Cp18	2.172E-06	1.3237462	-	-	-	-
CG13063	0.0001264	1.3201163	-	-	-	-
CG3777	9.972E-18	1.3113966	-	-	-	-
Cpr92F	8.498E-09	1.3074914	-	-	-	-
CG9698	0.0001664	1.299397	-	-	-	-
AOX2	0.0001316	1.2936922	-	-	-	-
CG7203	9.438E-63	1.2820094	-	-	-	-
obst-A	1.482E-17	1.2781386	-	-	-	-
CR44647	0.0002497	1.2675798	-	-	-	-
yellow-d2	8.1E-11	1.2566325	-	-	-	-
CR44157	0.0001704	1.2543964	-	-	-	-
CG2444	6.05E-12	1.2455427	-	-	-	-
CG12824	0.0003353	1.244251	-	-	-	-
CG10445	2.726E-05	1.2441222	-	-	-	-
CG31415	3.518E-08	1.2433485	-	-	-	-
CR43159	0.0003655	1.239354	-	-	-	-
nab	0.0001403	1.2311241	-	-	-	-
CHORD	5.032E-15	1.2270464	-	-	-	-
CG16710	0.0002189	1.2250789	-	-	-	-
CG17189	1.057E-18	1.2249256	-	-	-	-
CG13065	3.515E-05	1.2141489	-	-	-	-
CecA1	0.0005932	1.2098714	-	-	-	-
pip	3.02E-17	1.2019837	-	-	-	-
CG11693	0.0002229	1.1997395	-	-	-	-
CG14752	0.0001805	1.1960598	-	-	-	-
CG15517	0.0006944	1.188152	-	-	-	-
Cpr50Cb	0.0002803	1.1747705	-	-	-	-
CG5873	0.0008365	1.1599803	-	-	-	-
spag	2.456E-09	1.1509393	-	-	-	-
CG40198	7.175E-15	1.1376845	-	-	-	-
CG17991	0.0013094	1.1241303	-	-	-	-
CG30178	0.0008244	1.1200442	-	-	-	-
CG16786	1.84E-13	1.1186492	-	-	-	-
CR44135	0.0014781	1.1144428	-	-	-	-
Cda5	1.032E-20	1.1112645	-	-	-	-
CG15547	1.405E-20	1.1094287	-	-	-	-
CG32189	0.0020625	1.1050983	-	-	-	-
CG14258	6.25E-07	1.0979759	-	-	-	-
CG31320	0.0001306	1.0934223	-	-	-	-
CG15414	3.554E-19	1.0927136	-	-	-	-
Cda4	3.637E-12	1.0917749	-	-	-	-
CR45807	7.975E-20	1.0907214	-	-	-	-
CG32855	0.0008916	1.0880765	-	-	-	-
mira	0.0021021	1.0867095	-	-	-	-
CG8907	3.671E-05	1.0795849	-	-	-	-
CR18228	3.106E-27	1.0731965	-	-	-	-
iav	0.0001571	1.0699389	-	-	-	-
CG7214	3.398E-27	1.0640346	-	-	-	-
CG14770	0.0010726	1.0630521	-	-	-	-
CG13713	0.0021928	1.0494338	-	-	-	-
CG31904	8.015E-06	1.038233	-	-	-	-
CG7896	1.361E-05	1.0321213	-	-	-	-
CG1698	3.05E-09	1.0276509	-	-	-	-
CG9782	5.485E-10	1.0268152	-	-	-	-
CR44662	1.736E-08	1.0229924	-	-	-	-

CG13183	2.661E-07	1.0207685	-	-	-	-
CG15545	9.397E-08	1.0191961	-	-	-	-
Cht5	5.17E-09	1.017668	-	-	-	-
CG2837	1.499E-10	1.0100452	-	-	-	-
l(1)G0148	8.545E-12	1.005065	-	-	-	-
Ance-2	0.0032771	1.0038085	-	-	-	-
CR45024	2.722E-35	1.0027764	-	-	-	-
Ccp84Ad	0.0051238	1.0011268	-	-	-	-
Hsp67Ba	7.302E-13	0.9983563	-	-	-	-
CG10359	2.548E-06	0.9973654	-	-	-	-
CG18031	0.0012755	0.9946981	-	-	-	-
CG7945	2.496E-25	0.9899398	-	-	-	-
CG9447	0.0074029	0.9846641	-	-	-	-
tRNA:Y1:22Fb	0.0037932	0.9809551	-	-	-	-
slx1	0.0014948	0.9793072	-	-	-	-
scaRNA:PsiU1-	0.0037289	0.9682843	-	-	-	-
CG13426	0.0085506	0.9681703	-	-	-	-
CG34141	0.0056515	0.9674096	-	-	-	-
CG13796	3.125E-09	0.96261	-	-	-	-
CG11327	0.0057738	0.9570007	-	-	-	-
CG17028	0.0057738	0.9548141	-	-	-	-
CG18130	0.0056334	0.9529365	-	-	-	-
spdo	0.0066697	0.9460031	-	-	-	-
CG3713	0.0008566	0.9444214	-	-	-	-
Cht6	1.293E-12	0.9437166	-	-	-	-
Sgs1	0.0001103	0.9372516	-	-	-	-
CG15023	0.0108705	0.9344172	-	-	-	-
CecA2	0.0047423	0.9337694	-	-	-	-
CG10337	2.318E-09	0.9329518	-	-	-	-
CG12398	0.0112722	0.9309801	-	-	-	-
CR32745	0.0041438	0.9291197	-	-	-	-
Nep5	0.0031793	0.9287769	-	-	-	-
CG15080	1.468E-05	0.9232588	-	-	-	-
CG10973	8.236E-10	0.9184633	-	-	-	-
CR43243	0.000312	0.9163246	-	-	-	-
CG32364	5.292E-05	0.9153021	-	-	-	-
Clect27	6.792E-24	0.9128076	-	-	-	-
CG8785	2.515E-05	0.9071602	-	-	-	-
CG4613	0.0152681	0.9066747	-	-	-	-
tld	8.361E-05	0.9059374	-	-	-	-
CR32194	0.0001498	0.9053254	-	-	-	-
Ccp84Ac	0.0048111	0.9029752	-	-	-	-
CR46038	1.54E-06	0.9027152	-	-	-	-
obst-E	3.022E-16	0.9012115	-	-	-	-
CR33294	4.457E-07	0.8943715	-	-	-	-
CG15369	0.0119996	0.8942585	-	-	-	-
CG8292	0.0049098	0.8933453	-	-	-	-
mthl4	0.0138596	0.8924174	-	-	-	-
Ubc84D	0.0024176	0.891544	-	-	-	-
NimA	0.0089435	0.8889829	-	-	-	-
CG8252	0.0162936	0.8865505	-	-	-	-
CG41520	2.251E-16	0.8859804	-	-	-	-
CG14490	0.0084744	0.883004	-	-	-	-
CG15515	0.0109355	0.8752539	-	-	-	-
Dhc93AB	0.0004083	0.8751399	-	-	-	-
CG13449	1.409E-06	0.8710465	-	-	-	-
CG11852	0.0066819	0.8706171	-	-	-	-
CR44648	0.0183625	0.8696203	-	-	-	-

CR45985	0.008652	0.8677686	-	-	-	-
CG17707	9.364E-06	0.8637108	-	-	-	-
CG8630	0.0002072	0.8604579	-	-	-	-
CG13082	0.0058098	0.8585436	-	-	-	-
ppk22	0.0007279	0.8511233	-	-	-	-
CG15322	0.0150654	0.8501501	-	-	-	-
CG8219	0.0186979	0.8452853	-	-	-	-
CG11913	0.0142575	0.8386232	-	-	-	-
CG8927	4.888E-09	0.8338071	-	-	-	-
btl	0.0030589	0.8333549	-	-	-	-
snoRNA:Psi18S	8.994E-08	0.8318425	-	-	-	-
to	4.706E-16	0.831328	-	-	-	-
CG43646	0.0284371	0.8300229	-	-	-	-
CG42368	2.787E-09	0.8259624	-	-	-	-
CG15861	0.0003188	0.8217635	-	-	-	-
CG5987	0.0005968	0.8128887	-	-	-	-
CG34375	0.0003188	0.806558	-	-	-	-
CG5327	1.929E-07	0.8043976	-	-	-	-
CG30380	0.0352612	0.8036731	-	-	-	-
CG16926	1.614E-05	0.8017806	-	-	-	-
CG34327	2.012E-05	0.7987035	-	-	-	-
CR43483	0.0341252	0.7948631	-	-	-	-
CR41601	0.0331806	0.7947222	-	-	-	-
CG31205	1.811E-11	0.7915891	-	-	-	-
CG13003	6.458E-05	0.7897247	-	-	-	-
Sgt1	6.066E-09	0.7884722	-	-	-	-
Ets96B	1.985E-05	0.7882906	-	-	-	-
CG9509	0.0054123	0.7814828	-	-	-	-
CG14304	0.0109726	0.7811625	-	-	-	-
CG31189	6.137E-09	0.7802033	-	-	-	-
sad	0.0430397	0.7792498	-	-	-	-
CR43906	6.085E-12	0.7785324	-	-	-	-
Ag5r	0.0392546	0.7774368	-	-	-	-
CG9293	3.934E-08	0.7769647	-	-	-	-
wb	0.0001247	0.7762965	-	-	-	-
CG6790	0.016547	0.7756565	-	-	-	-
CG43062	0.0337159	0.7745227	-	-	-	-
CG12206	0.0406122	0.7732974	-	-	-	-
yellow-h	6.925E-12	0.7714112	-	-	-	-
CG15368	0.0109726	0.7710843	-	-	-	-
CR44096	0.0410328	0.7708392	-	-	-	-
CG15337	0.0470134	0.765757	-	-	-	-
CG13618	0.0102691	0.7655666	-	-	-	-
CG11777	7.836E-09	0.7620068	-	-	-	-
CG4914	3.293E-08	0.7620016	-	-	-	-
CR45949	0.0468626	0.7610684	-	-	-	-
CG11458	0.0220193	0.7592116	-	-	-	-
CG12992	0.046525	0.7573876	-	-	-	-
CR44161	0.0473114	0.7549534	-	-	-	-
CG8192	0.0118343	0.7491931	-	-	-	-
CG12825	0.0009874	0.7491561	-	-	-	-
CR45964	0.0045492	0.747411	-	-	-	-
Cyp313a1	8.134E-16	0.7450991	-	-	-	-
CG11619	0.0090973	0.7450226	-	-	-	-
snoRNA:684	0.042806	0.744687	-	-	-	-
CG11437	0.0428826	0.744141	-	-	-	-
CG11353	0.0339262	0.742275	-	-	-	-
bond	2.76E-06	0.7408082	-	-	-	-

tw	3.716E-06	0.739456	-	-	-	-
TTLL3B	0.0499875	0.7392267	-	-	-	-
CG13377	1.764E-09	0.7384041	-	-	-	-
CG33137	0.0495938	0.7377829	-	-	-	-
snoRNA:Me28S	0.0495938	0.7374387	-	-	-	-
CG45067	0.0437888	0.7301882	-	-	-	-
CG6074	0.0086073	0.7274385	-	-	-	-
CG1136	2.747E-07	0.7253582	-	-	-	-
Cyp6a19	3.883E-05	0.7247903	-	-	-	-
hh	0.0347716	0.7240514	-	-	-	-
CG33978	4.704E-06	0.7232239	-	-	-	-
CR44224	0.0174917	0.7225574	-	-	-	-
CG11345	0.0178999	0.7200752	-	-	-	-
CG13606	3.254E-13	0.7120533	-	-	-	-
CG9624	0.0028323	0.710471	-	-	-	-
CG17760	0.009416	0.7090776	-	-	-	-
CG7133	2.388E-05	0.70523	-	-	-	-
CG31998	4.465E-08	0.7019142	-	-	-	-
CR43472	0.0401604	0.7016494	-	-	-	-
pgc	0.0042753	0.7007469	-	-	-	-
CG34454	6.735E-05	0.7002958	-	-	-	-
CG15145	0.0278576	0.695729	-	-	-	-
Ugt86De	0.0303877	0.6938895	-	-	-	-
CG4576	0.0389371	0.6936025	-	-	-	-
CG33993	0.0008328	0.6933065	-	-	-	-
CR44894	0.0448091	0.6932658	-	-	-	-
CG44163	0.0041803	0.6922435	-	-	-	-
CG32365	0.0083752	0.6917019	-	-	-	-
l(2)34Fc	1.105E-09	0.6882811	-	-	-	-
Peritrophin-A	1.289E-09	0.6873164	-	-	-	-
Or65c	0.0281268	0.6840714	-	-	-	-
T3dh	0.0026046	0.6835891	-	-	-	-
CG16978	1.836E-05	0.6820876	-	-	-	-
CG42329	0.0008709	0.6810571	-	-	-	-
CrzR	4.221E-06	0.6800896	-	-	-	-
CR44172	0.0351683	0.6796597	-	-	-	-
CR44029	6.212E-08	0.6796145	-	-	-	-
CG2812	0.0014712	0.6652083	-	-	-	-
CR44779	2.81E-05	0.66387	-	-	-	-
Cht3	0.0180285	0.6619859	-	-	-	-
CG5958	4.985E-06	0.6596615	-	-	-	-
rdo	6.162E-08	0.6591501	-	-	-	-
CG31999	4.693E-08	0.6558828	-	-	-	-
yellow-e	2.058E-06	0.6554924	-	-	-	-
CR44119	0.0157791	0.6554551	-	-	-	-
TwdlT	0.0079665	0.6532474	-	-	-	-
CG10077	3.735E-09	0.65317	-	-	-	-
CG42494	0.007825	0.6512487	-	-	-	-
CR46065	0.0122433	0.650982	-	-	-	-
E(spl)mdelta-HL	0.0294006	0.6499998	-	-	-	-
Rpt4R	0.0003399	0.647109	-	-	-	-
CG34138	0.0419599	0.6469816	-	-	-	-
CG17104	0.0003635	0.6409957	-	-	-	-
CG7065	3.101E-05	0.6402409	-	-	-	-
GCC185	0.0022976	0.639831	-	-	-	-
CR44850	2.071E-05	0.6392069	-	-	-	-
Sip1	2.734E-06	0.634054	-	-	-	-
CR44960	1.47E-10	0.6339367	-	-	-	-

snoRNA:Me28S	0.0222164	0.6310605	-	-	-	-
CG14301	0.0015556	0.6227886	-	-	-	-
slow	7.279E-06	0.6220897	-	-	-	-
Vm34Ca	0.043487	0.6210349	-	-	-	-
CR43864	0.0192643	0.6210217	-	-	-	-
CG4221	0.0006687	0.6208443	-	-	-	-
CG16749	0.0495109	0.6204999	-	-	-	-
CR43170	0.0149589	0.6182392	-	-	-	-
Cpr64Aa	0.0007648	0.6158149	-	-	-	-
CG18155	0.049034	0.6135614	-	-	-	-
CG13437	0.0446674	0.611761	-	-	-	-
CG32793	0.0486795	0.6104001	-	-	-	-
CG5493	0.0006436	0.6094728	-	-	-	-
CG11413	0.0076186	0.6084175	-	-	-	-
CR45185	8.577E-05	0.6009167	-	-	-	-
CG32801	0.0346815	0.5996874	-	-	-	-
CG34461	0.0053321	0.5987041	-	-	-	-
Cralbp	0.0025074	0.5983043	-	-	-	-
FoxP	3.167E-06	0.5972337	-	-	-	-
l(2)01289	0.0151181	0.5952788	-	-	-	-
dmGlut	0.0019282	0.5924498	-	-	-	-
sage	0.0059674	0.5817529	-	-	-	-
Cpr67B	7.691E-08	0.5782376	-	-	-	-
Osi9	0.0423936	0.5759417	-	-	-	-
CR31514	0.024355	0.575189	-	-	-	-
dsb	2.858E-06	0.5739531	-	-	-	-
CR44986	5.054E-09	0.5704676	-	-	-	-
CG13188	4.091E-06	0.5660526	-	-	-	-
CG17032	0.000461	0.5649563	-	-	-	-
CG16789	0.0027901	0.5582603	-	-	-	-
CG8854	0.0009291	0.5548993	-	-	-	-
net	0.0167797	0.5489787	-	-	-	-
CG5482	5.832E-06	0.5487248	-	-	-	-
ckd	0.0280282	0.5484434	-	-	-	-
CG42249	0.0139852	0.5452406	-	-	-	-
wat	1.06E-06	0.5431667	-	-	-	-
Dyrk3	6.39E-07	0.5379421	-	-	-	-
CG10553	0.0016439	0.5372748	-	-	-	-
CG10175	0.0005648	0.5357213	-	-	-	-
Cpr47Ef	0.0273791	0.5318535	-	-	-	-
CG32243	0.0170282	0.5308973	-	-	-	-
CG12428	0.0061814	0.5306202	-	-	-	-
SAK	0.000103	0.5292731	-	-	-	-
Cpr64Ac	0.0384637	0.5290126	-	-	-	-
CG42846	0.0030706	0.5285191	-	-	-	-
gt	0.0045492	0.5253903	-	-	-	-
GstD10	0.0435543	0.5236712	-	-	-	-
l(2)efl	0.0146638	0.5216287	-	-	-	-
Cpr47Ee	0.0015177	0.5211642	-	-	-	-
CG13793	0.0260779	0.5211606	-	-	-	-
CG6332	0.0316971	0.5196039	-	-	-	-
CG3267	0.0450102	0.5195788	-	-	-	-
Tm2	0.000464	0.5144335	-	-	-	-
Mlf	9.244E-06	0.5116847	-	-	-	-
ana1	0.0032529	0.5091097	-	-	-	-
Cog7	0.0002815	0.5068511	-	-	-	-
CG3984	0.0122348	0.5046454	-	-	-	-
CG17912	1.302E-08	0.5046214	-	-	-	-

Mitf	1.474E-06	0.5037994	-	-	-	-
CG3921	0.0102519	0.5036626	-	-	-	-
Cyp6t3	0.018884	0.5028446	-	-	-	-
Cyp28d2	0.0133307	0.5023763	-	-	-	-
CG15449	0.0154251	0.4996563	-	-	-	-
CG15479	0.0176316	0.4966364	-	-	-	-
CR44704	1.501E-07	0.496077	-	-	-	-
CG5458	0.0242446	0.4951541	-	-	-	-
CR46089	0.0002355	0.4936433	-	-	-	-
b6	0.0063636	0.486088	-	-	-	-
CR44969	7.67E-07	0.4837203	-	-	-	-
CR45713	0.0416927	0.4836999	-	-	-	-
CG17211	0.0001962	0.4826557	-	-	-	-
Sir2	0.0002172	0.4810991	-	-	-	-
sxe2	1.186E-07	0.4783955	-	-	-	-
Rbm13	0.0025092	0.4777112	-	-	-	-
mGluR	0.0003013	0.4771569	-	-	-	-
CG12661	0.0356532	0.4722997	-	-	-	-
CG8671	0.0005091	0.4722558	-	-	-	-
CG44085	0.0001173	0.4688447	-	-	-	-
CG9519	0.0109167	0.4676666	-	-	-	-
Ccs	0.0245849	0.4646258	-	-	-	-
Buffy	0.0222213	0.4629119	-	-	-	-
CR42491	0.0133814	0.4601922	-	-	-	-
CG13833	0.000611	0.4599915	-	-	-	-
CG13025	0.0084719	0.459727	-	-	-	-
CG32579	0.0278498	0.45501	-	-	-	-
rtv	0.0101704	0.4548423	-	-	-	-
CG6845	0.0026622	0.4545612	-	-	-	-
CG7457	1.782E-05	0.4538208	-	-	-	-
CG34057	0.0282083	0.4530421	-	-	-	-
Mlc2	6.847E-06	0.4488486	-	-	-	-
CG9747	0.0018214	0.4476455	-	-	-	-
r-l	0.0052137	0.4459699	-	-	-	-
Cyp4d21	0.0005166	0.4448861	-	-	-	-
CG4266	0.001367	0.4437907	-	-	-	-
CG4294	0.0038753	0.4415685	-	-	-	-
CR43989	0.0494059	0.4414023	-	-	-	-
drd	0.0089435	0.4404595	-	-	-	-
asparagine-syn	0.000573	0.4377089	-	-	-	-
CG13624	0.0004748	0.4374424	-	-	-	-
CG9400	0.0341402	0.4343294	-	-	-	-
CR43361	0.0170271	0.4343017	-	-	-	-
CG7402	0.0074663	0.4328881	-	-	-	-
CR45350	0.0006945	0.4320364	-	-	-	-
Cep135	0.0340052	0.4299423	-	-	-	-
CG9297	0.0201509	0.4284883	-	-	-	-
CR45007	0.0294849	0.4263765	-	-	-	-
Ephrin	0.0014948	0.4262495	-	-	-	-
Rhp	0.0304784	0.4218299	-	-	-	-
CG8665	0.0014186	0.4183863	-	-	-	-
scaf	0.0026661	0.4170545	-	-	-	-
FKBP59	0.0035815	0.4162016	-	-	-	-
CG9640	0.0133104	0.4150049	-	-	-	-
nAChRalpha4	0.001347	0.4145998	-	-	-	-
Cf2	0.0012729	0.4099199	-	-	-	-
CR42860	0.0344932	0.4076586	-	-	-	-
Mlp84B	0.0035355	0.4015474	-	-	-	-

Obp57b	0.0002189	0.4014088	-	-	-	-
ABCB7	9.203E-05	0.4013452	-	-	-	-
CG5867	5.139E-06	0.3999117	-	-	-	-
CG9512	0.0150285	0.3963588	-	-	-	-
Zasp66	0.0109278	0.3956866	-	-	-	-
CG6912	0.000396	0.3926816	-	-	-	-
CG12567	0.0156213	0.3926436	-	-	-	-
CG2789	0.0005571	0.3891366	-	-	-	-
CR43470	0.0030189	0.3823516	-	-	-	-
CG13794	0.0047228	0.378702	-	-	-	-
Obp49a	0.007837	0.378694	-	-	-	-
CG1889	0.0403675	0.375135	-	-	-	-
kon	0.043487	0.3738164	-	-	-	-
CR43425	0.0001531	0.3737367	-	-	-	-
TBCB	0.0110046	0.3671772	-	-	-	-
CG42388	0.0099113	0.3662493	-	-	-	-
CG12090	0.0017386	0.365294	-	-	-	-
Ada2a	0.0267936	0.3651984	-	-	-	-
MED26	0.0006503	0.3650063	-	-	-	-
CG1316	0.0135073	0.3641103	-	-	-	-
Psi	0.0027733	0.3635087	-	-	-	-
Drsl5	0.0286325	0.3631775	-	-	-	-
Rbp1	0.000742	0.3603868	-	-	-	-
Drep-1	0.0316593	0.3601786	-	-	-	-
CG17167	0.0247587	0.3595363	-	-	-	-
CG34207	0.0239756	0.3545999	-	-	-	-
CG1041	0.0109278	0.3539631	-	-	-	-
CG3793	0.0180215	0.3534665	-	-	-	-
CG8317	0.0056798	0.3513187	-	-	-	-
DIP2	0.0154645	0.3482253	-	-	-	-
CG41099	0.0078113	0.3480682	-	-	-	-
CG8475	0.0008993	0.3475596	-	-	-	-
CR44990	0.0392269	0.3466239	-	-	-	-
CG1674	0.0342754	0.3460804	-	-	-	-
Sh3beta	0.0075425	0.3425209	-	-	-	-
CG1124	0.0018767	0.3420107	-	-	-	-
Set1	0.0101509	0.3406345	-	-	-	-
gfzf	0.0377792	0.3405052	-	-	-	-
CG31321	0.0099555	0.3394276	-	-	-	-
MSBP	0.0025862	0.3393996	-	-	-	-
CG10249	0.0104975	0.3337272	-	-	-	-
Mlc1	0.0497008	0.3328105	-	-	-	-
Cyp6g1	0.0003323	0.3305547	-	-	-	-
CG2278	0.0159078	0.3293326	-	-	-	-
myo	0.0232111	0.3258258	-	-	-	-
CG3209	0.0017362	0.323553	-	-	-	-
onecut	0.0118886	0.3225305	-	-	-	-
CG11414	0.0477183	0.3220051	-	-	-	-
CG3191	0.0220193	0.3217604	-	-	-	-
ade3	0.0054517	0.3213964	-	-	-	-
CG8079	0.0158333	0.3207189	-	-	-	-
CG15661	0.0055458	0.3192079	-	-	-	-
CG43092	0.0105374	0.3176296	-	-	-	-
Ubp64E	0.0363619	0.3158031	-	-	-	-
PebIII	0.0135572	0.3099721	-	-	-	-
CR43964	0.040752	0.3036579	-	-	-	-
sowah	0.0496675	0.3004368	-	-	-	-
conu	0.0205902	0.2972424	-	-	-	-

CR45682	0.0486244	0.2966368	-	-	-	-
CG43291	0.0405267	0.2961615	-	-	-	-
CG31712	0.0128124	0.2924603	-	-	-	-
CG12582	0.0277849	0.2907358	-	-	-	-
CG42235	0.0484066	0.288166	-	-	-	-
CG31199	0.0270116	0.2849924	-	-	-	-
CR44690	0.0102519	0.2797072	-	-	-	-
roX2	0.0237091	0.2796652	-	-	-	-
alpha-Man-I	0.0145618	0.2780122	-	-	-	-
su(w[a])	0.0288496	0.266999	-	-	-	-
CaMKII	0.0278709	0.2612034	-	-	-	-
CG32000	0.0311922	0.2588601	-	-	-	-
nonA	0.0216287	0.2558683	-	-	-	-
CR45601	0.0232351	0.2469571	-	-	-	-
CG2076	0.0472667	0.2455416	-	-	-	-
CG8974	0.0492926	0.2454298	-	-	-	-
Ank	0.0220338	0.2449155	-	-	-	-
CG2321	0.0443043	0.2448766	-	-	-	-
Yp1	0.0111271	0.2440312	-	-	-	-
CG1632	0.0269572	0.2407545	-	-	-	-
CG17698	0.0398031	0.2170329	-	-	-	-
Inr-a	0.0377257	0.2080048	-	-	-	-
Yp2	0.0252767	0.2018107	-	-	-	-

Supplementary Table 9 Mapping summary in ChIP-seq and nuclear RNA-seq analysis

ChIP-seq	Input reads	Uniquely mapped reads	Mapping rate
CBP_naive1	11,622,571	10,140,169	87.25%
CBP_naive2	12,883,750	10,378,327	80.55%
CBP_naive3	13,984,685	10,971,584	78.45%
CBP_5minLight1	14,670,203	11,920,429	81.26%
CBP_5minLight2	16,387,875	13,735,653	83.82%
CBP_5minLight3	15,386,636	12,059,922	78.38%
CoRest-C_naive1	14,044,052	5,035,629	35.86%
CoRest-C_naive2	13,624,091	5,997,394	44.02%
CoRest-C_naive3	14,068,291	4,155,139	29.54%
CoRest-C_5minLight1	7,710,889	3,637,016	47.17%
CoRest-C_5minLight2	12,693,280	7,499,826	59.09%
CoRest-C_5minLight3	14,840,103	7,262,224	48.94%
Rpd3_naive1	12,663,514	9,146,812	72.23%
Rpd3_naive2	11,736,300	7,749,778	66.03%
Rpd3_naive3	13,181,257	9,542,370	72.39%
Rpd3_5minLight1	13,263,141	5,872,746	44.28%
Rpd3_5minLight2	12,567,451	8,256,347	65.70%
Rpd3_5minLight3	13,711,110	10,429,489	76.07%
Input	12,836,771	10,449,877	81.41%

Nuclear RNA-seq	Input reads	Uniquely mapped reads	Mapping rate
nRNAseq_naive1	19,712,179	18,107,447	91.86%
nRNAseq_naive2	16,929,889	15,134,101	89.39%
nRNAseq_naive3	13,661,735	12,522,852	91.66%
nRNAseq_5minLight1	17,183,396	15,698,394	91.36%
nRNAseq_5minLight2	21,433,850	19,660,967	91.73%
nRNAseq_5minLight3	22,045,647	19,982,508	90.64%
nRNAseq_5minLight5minRest1	21,681,759	19,178,118	88.45%
nRNAseq_5minLight5minRest2	21,127,023	18,810,955	89.04%
nRNAseq_5minLight5minRest3	19,066,142	16,945,663	88.88%
nRNAseq_5minLight10minRest1	22,215,469	19,782,388	89.05%
nRNAseq_5minLight10minRest2	17,872,798	16,048,670	89.79%
nRNAseq_5minLight10minRest3	19,468,450	17,479,866	89.79%
nRNAseq_5minLight15minRest1	12,174,444	11,020,456	90.52%
nRNAseq_5minLight15minRest2	14,977,933	13,563,166	90.55%
nRNAseq_5minLight15minRest3	18,535,205	16,866,439	91.00%
