# Supplementary material for: Rpd3/CoRest-mediated activity-dependent transcription regulates the flexibility in memory updating in Drosophila
Source: Nat Commun. 2021 Jan 27;12:628. doi: 10.1038/s41467-021-20898-x (PMC7840730; doi:10.1038/s41467-021-20898-x)
Supplement: Supplementary file 3 — Reporting Summary [file 41467_2021_20898_MOESM3_ESM.pdf]

nature research

Corresponding author(s): Yukinori Hirano

Last updated by author(s): Dec 8, 2020

Reporting Summary

Nature Research wishes to improve the reproducibility of the work that we publish. This form provides structure for consistency and transparency in reporting. For further information on Nature Research policies, seeAuthors & Referees and theEditorial Policy Checklist .

Statistics

For all statistical analyses, confirm that the following items are present in the figure legend, table legend, main text, or Methods section.

n/a	Confirmed	
☐	☒	The exact sample size (*n*) for each experimental group/condition, given as a discrete number and unit of measurement
☐	☒	A statement on whether measurements were taken from distinct samples or whether the same sample was measured repeatedly
☐	☒	The statistical test(s) used AND whether they are one- or two-sided<br>*Only common tests should be described solely by name; describe more complex techniques in the Methods section.*
☒	☐	A description of all covariates tested
☐	☒	A description of any assumptions or corrections, such as tests of normality and adjustment for multiple comparisons
☐	☒	A full description of the statistical parameters including central tendency (e.g. means) or other basic estimates (e.g. regression coefficient) AND variation (e.g. standard deviation) or associated estimates of uncertainty (e.g. confidence intervals)
☐	☒	For null hypothesis testing, the test statistic (e.g. *F*, *t*, *r*) with confidence intervals, effect sizes, degrees of freedom and *P* value noted<br>*Give P values as exact values whenever suitable.*
☒	☐	For Bayesian analysis, information on the choice of priors and Markov chain Monte Carlo settings
☒	☐	For hierarchical and complex designs, identification of the appropriate level for tests and full reporting of outcomes
☒	☐	Estimates of effect sizes (e.g. Cohen's *d*, Pearson's *r*), indicating how they were calculated

Our web collection on statistics for biologists contains articles on many of the points above.

Software and code

Policy information about availability of computer code

Data collection	No software was used for data collection.
Data analysis	Prism version 5.0, R3.3.2, StrandNGS 2.6, Treeview version 1.1.6r4, trimommatic-0.36 (version 3), STAR_2.5.3a, HTseq-count_0.6.1, DESeq2 version 1.12.4, MACS2, MASCOT version 2.6, skyline, Proteome discoverer 2.1. FastQC version 0.11.4, Image J version 1.53e

For manuscripts utilizing custom algorithms or software that are central to the research but not yet described in published literature, software must be made available to editors/reviewers. We strongly encourage code deposition in a community repository (e.g. GitHub). See the Nature Research guidelines for submitting code & software for further information.

Data

Policy information about availability of data

All manuscripts must include a data availability statement. This statement should provide the following information, where applicable:

- Accession codes, unique identifiers, or web links for publicly available datasets
- A list of figures that have associated raw data
- A description of any restrictions on data availability

All data generated or analyzed during this study are included in this published article (and its Supplementary Information Files) or deposited. The GEO accession number for the ChIP-seq and RNA-seq data reported in this paper is GSE150642 [https://www.ncbi.nlm.nih.gov/geo/query/acc.cgi?acc=GSE150642]. All mass spectrometry data have been deposited to ProteomeXchange Consortium via jPOST with the accession number PXD021294 [http://proteomecentral.proteomexchange.org/cgi/GetDataset?ID=PXD021294] and JPST000948 [https://repository.jpostdb.org/entry/JPST000948], respectively. Source data are provided as a Source Data File.

Field-specific reporting

Please select the one below that is the best fit for your research. If you are not sure, read the appropriate sections before making your selection.

- [x] Life sciences
- [ ] Behavioural & social sciences
- [ ] Ecological, evolutionary & environmental sciences

For a reference copy of the document with all sections, see nature.com/documents/nr-reporting-summary-flat.pdf

Life sciences study design

All studies must disclose on these points even when the disclosure is negative.

Sample size

No sample size calculations were performed. Our sample sizes are similar to those generally used in this field of research.

Data exclusions

No data were excluded from the analyses.

Replication

Each experiment was repeated from at least two independent crosses ,except Mass spec analysis. All attempts at replication were successful.

Randomization

The group of flies (genotype and treatment) were randomly assigned into numbers. We use multiple apparatuses for behavioral experiments and optogenetic manipulation, and in this case, allocation was not relevant. The apparatuses were evenly used systematically in a group of flies, otherwise the data could be biased due to allocation.
For experiments other than those involving flies, the test samples were randomly numbered and were allocated into experimental groups.

Blinding

All samples were numbered and the investigators were blinded.

Reporting for specific materials, systems and methods

We require information from authors about some types of materials, experimental systems and methods used in many studies. Here, indicate whether each material, system or method listed is relevant to your study. If you are not sure if a list item applies to your research, read the appropriate section before selecting a response.

Materials & experimental systems

n/a	Involved in the study
[ ]	[x] Antibodies
[ ]	[x] Eukaryotic cell lines
[x]	[ ] Palaeontology
[ ]	[x] Animals and other organisms
[x]	[ ] Human research participants
[x]	[ ] Clinical data

Methods

n/a	Involved in the study
[ ]	[x] ChIP-seq
[x]	[ ] Flow cytometry
[x]	[ ] MRI-based neuroimaging

Antibodies

Antibodies used

anti-HA antibody-conjugated magnetic beads (88837, lot TA265667, Thermo Fisher Scientific, San Jose, CA, USA)
rabbit monoclonal anti-pERK antibodies (4376, lot 17, Cell Signaling Technology, Beverly, MA, USA)
anti-myc antibody-conjugated magnetic beads (88843, Thermo Fisher Scientific, San Jose, CA, USA)
anti-acetyl lysine antibodies (ab21623, lot GR193038-23, GR3203593-9, Abcam, Cambridge, MA, USA)
The anti-Rpd3 and anti-CBP antibodies were raised against the C-terminal 124 amino acids of Rpd3, and the N-terminal 1,000 amino acids of CBP.
The anti-CoRest antibody raised against the amino acids 634–820 was provided by G. Mandel
mouse monoclonal anti-myc antibody (626802, BioLegend, San Diego, CA, USA)
rabbit monoclonal anti-myc antibody (2278, lot 5, Cell Signaling Technology, Beverly, MA, USA)
rabbit monoclonal anti-HA antibody (3724, lot 9, Cell Signaling Technology, Beverly, MA, USA)
mouse monoclonal anti-HA antibody (901515, BioLegend, San Diego, CA, USA)
rabbit anti-histone H3 antibody (ab1791, lot 2767-3, Abcam, Cambridge, MA, USA)
mouse nc82 antibody (AB_2314866, Developmental Studies Hybridoma Bank, Univ. Iowa, USA)
rabbit anti-DsRed antibody (632496, Takara, Shiga, Japan)
mouse anti-alpha-tubulin antibody (T6199, Sigma, St. Louis, MO, USA)
chicken anti-GFP antibody (ab13970, GR261775-1, Abcam, Cambridge, MA, USA)
donkey anti-mouse IgG Alexa Fluor 488 antibody (715-545-150, lot 101662, Jackson ImmunoResearch Labs, Inc., West Grove, PA, USA),
donkey anti-chicken immunoglobulin Y (IgY) Alexa 488 antibody (703-545-155, lot 102758, Jackson ImmunoResearch Labs, Inc., West Grove, PA, USA),

	donkey anti-rabbit IgG Cy3 antibody (711-166-152, lot 93219, Jackson ImmunoResearch Labs, Inc., West Grove, PA, USA)<br>ANTI-FLAG M2 Affinity Gel (Sigma, St. Louis, MO, USA)
Validation	Anti-Rpd3 (data is available upon request), CBP (data is available upon request), CoRest (Fig. 2a), and alpha-tubulin (Supplementary Fig. 1c) antibodies were validated by western blot in fly, showing the specific single band corresponding to the expected molecular weight. Anti-pERK antibody in fly was validated in our previous report (Awata et al, PNAS, 2019). Anti-nc82 antibodies have been validated in the past studies (https://dshb.biology.uiowa.edu/nc82), and are in common use in the Drosophila community. Other commercially available primary antibodies for HA or myc epitope tags were validated using flies expressing proteins tagged by the epitope tags (Fig. 2a, b). The secondary antibodies were validated by showing loss of the immunofluorescence in the absence of the primary antibodies.

Eukaryotic cell lines

Policy information about cell lines

Cell line source(s)	The insect cell line Sf9 was purchased from Invitrogen.
Authentication	The insect cell line Sf9 was obtained from original source and was not further authenticated.
Mycoplasma contamination	The insect cell line Sf9 was tested negative for mycoplasma contamination.
Commonly misidentified lines<br>(See ICLAC register)	No commonly misidentified cell line was used.

Animals and other organisms

Policy information about studies involving animals; ARRIVE guidelines recommended for reporting animal research

Laboratory animals	Male and female adult (2-6 days of age) flies were used. Those include the CoRest-C::myc knock-in, MBsw2, UAS-Rpd3::FLAG-HA, UAS-CoRest-C::myc (WT or K/R mutant), UAS-CoRest-C-miRNA, UAS-CoRest-C::FLAG-HA, CoRest-K318R, and w(CS10) flies.
Wild animals	No wild animals were used in the study.
Field-collected samples	No field collected samples were used in the study.
Ethics oversight	The study design was approved by the appropriate ethics review board by Kyoto university.

Note that full information on the approval of the study protocol must also be provided in the manuscript.

ChIP-seq

Data deposition

- [x] Confirm that both raw and final processed data have been deposited in a public database such as GEO.
- [x] Confirm that you have deposited or provided access to graph files (e.g. BED files) for the called peaks.

Data access links<br>*May remain private before publication.*	The ChIP-seq and RNA-seq data ate available through NCBI Gene Expression Omnibus (GEO) repository under accession number GSE150642.
Files in database submission	Flies in the GEO are fastq, and the processed data (gene count file for RNA-seq and bed file for ChIP-seq).
Genome browser session<br>(e.g. UCSC)	no longer applicable

Methodology

Replicates	Three biological replicates were performed, each of which is derived from the MB nuclei collected from 500 flies.
Sequencing depth	Total number of reads and mapping efficiencies were shown in Supplementary Table 9. Total reads for ChIP-seq were 9-18 million, and those for RNA-seq were 30-40 million. Length of the reads was 150 bp (pair-end).
Antibodies	The anti-Rpd3 and anti-CBP antibodies were raised against the C-terminal 124 amino acids of Rpd3, and the N-terminal 1,000 amino acids of CBP. Rabbit monoclonal anti-myc antibody (2278, lot 5, Cell Signaling Technology, Beverly, MA, USA).
Peak calling parameters	Peaks were detected using MACS, using a default setting except for the following parameters; 10-4 as a P-value cutoff, and 3 as a enrichment factor.
Data quality	Read quality was assessed using FastQC (version 0.11.4): average read phred score was greater than 30. The reads were enriched near TSS, and especially, consistent to our finding, CBP, Rpd3, and CoRest-C colocalized at the specific gene loci.

Software | R3.3.2, StrandNGS, Treeview, trimommatic, STAR, HTseq-count, DESeq2, MACS2
